# An Immune-Related Signature Predicts Survival in Patients With Lung Adenocarcinoma

**DOI:** 10.3389/fonc.2019.01314

**Published:** 2019-12-10

**Authors:** Minghui Zhang, Kaibin Zhu, Haihong Pu, Zhuozhong Wang, Hongli Zhao, Jinfeng Zhang, Yan Wang

**Affiliations:** ^1^Department of Medical Oncology, Harbin Medical University Cancer Hospital, Harbin, China; ^2^Department of Thoracic Surgery, Harbin Medical University Cancer Hospital, Harbin, China

**Keywords:** hierarchal clustering, immunophenotypes, lung adenocarcinoma, patient prognosis, riskscore

## Abstract

We investigated the local immune status and its prognostic value in lung adenocarcinoma. In total, 513 lung adenocarcinoma samples from TCGA and ImmPort databases were collected and analyzed. The R package coxph was employed to mine immune-related genes that were significant prognostic indicators using both univariate and multivariate analyses. The R software package glmnet was then used for Lasso Cox regression analysis, and a prognosis prediction model was constructed for lung adenocarcinoma; clusterProfiler was selected for functional gene annotations and KEGG enrichment analysis. Finally, correlations between the RiskScore and clinical features or signaling pathways were established. Sixty-four immune-related genes remarkably correlated with patient prognosis and were further applied. Samples were hierarchically clustered into two subgroups. Accordingly, the LASSO regression algorithm was employed to screen the 14 most representative immune-related genes (*PSMD11, PPIA, MIF, BMP5, DKK1, PDGFB, ANGPTL4, IL1R2, THRB, LTBR, TNFRSF1, TNFRSF17, IL20RB*, and *MC1R*) with respect to patient prognosis. Then, the prognosis prediction model for lung adenocarcinoma patients (namely, the RiskScore equation) was constructed, and the training set samples were incorporated to evaluate the efficiency of this model to predict and classify patient prognosis. Subsequently, based on functional annotations and KEGG pathway analysis, the 14 immune-related genes were mainly enriched in pathways closely associated with lung adenocarcinoma and its immune microenvironment, such as cytokine–cytokine receptor interaction and human T-cell leukemia virus 1 infection. Furthermore, correlations between the RiskScore and clinical features of the training set samples and signaling pathways (such as p53, cell cycle, and DNA repair) were also demonstrated. Finally, the test set sample data were employed for independent testing and verifying the model. We established a prognostic prediction RiskScore model based on the expression profiles of 14 immune-related genes, which shows high prediction accuracy and stability in identifying immune features. This could provide clinical guidance for the diagnosis and prognosis of different immunophenotypes, and suggest multiple targets for precise advanced lung adenocarcinoma therapy based on subtype-specific immune molecules.

## Introduction

Lung adenocarcinoma is one of the most commonly encountered malignant tumors in the clinic, and is characterized by its high rate of metastasis and marked invasiveness. Accordingly, its 5-year survival rate is low, and thus, it has become one of the most important malignant tumors that threatens human life (1). Biomarkers can reliably estimate disease prognosis and patient survival, which is of great value to guide the clinical treatment of lung adenocarcinoma (2, 3). According to several large-sample clinical research studies, most early lung adenocarcinoma patients do not receive adjuvant systemic chemotherapy after surgery, because chemotherapy-related toxicity far outweighs the survival benefits to the patients (4). Therefore, it is necessary to recognize disease-associated risks in patients during early diagnosis and to administer additional adjuvant systemic chemotherapy for high-risk patients.

In recent years, increasing studies have reported methods to predict and stratify survival and prognosis for lung adenocarcinoma patients based on gene expression. Unfortunately, such studies have not been translated to routine clinical practice, which can be ascribed to small sample sizes, excessive data fitting, or inadequate evidence (5–8). The currently open and available large-scale databases containing gene expression data, such as TCGA and ImmPort, have made it possible to potentially mine more reliable biomarkers for lung adenocarcinoma to predict and classify patient prognosis (9, 10). Each part of the immune system has been verified to participate in, accelerate, and even determine different stages of cancer initiation and progression (11). In addition, immune escape has also been confirmed to be a novel marker for cancer. Recently, surprising effects have been achieved for the treatment of lung adenocarcinoma patients based on the immunotherapeutic PD-1/PD-L1, which targets specific immune checkpoints (12, 13). PD-1/PD-L1 pathway inhibitors were approved for the treatment of metastatic NSCLC patients. Additionally, histopathologically observed immunological phenomena such as cytotoxic lymphocyte endosmosis in lung adenocarcinoma have been shown to markedly correlate with patient prognosis (14). However, the molecular events underlying tumor cell–immunocyte interactions in lung adenocarcinoma microenvironments need to be further explored and summarized, which will ultimately determine their potential to predict the prognosis of patients with lung adenocarcinoma.

In this study, TCGA and ImmPort databases were analyzed and the clinical features of patients were considered to develop and verify a prognostic prediction model for lung adenocarcinoma based on immune-related genes. This could be ultimately used to assist clinicians in prognostic evaluations and therapeutic selection for advanced lung adenocarcinoma patients.

## Materials and Methods

### Pre-processing of Preliminary Sample Data and Primary Screening of Lung Adenocarcinoma Immune-Related Genes

The latest clinical follow-up information was downloaded using TCGA GDC API, as shown in Table S1. Moreover, RNA-Seq samples were downloaded and the immune-related gene set was downloaded from the ImmPort database, which covered 1811 genes.

First, the tumor tissues were pre-processed, by the following steps: (1) removing samples with no clinical data, (2) removing the normal tissue sample data, (3) removing the genes of FPKM < 1 from all samples, (4) preserving only the expression profiles of immune-related genes, and (5) filtering out genes with exceeding low abundance. From this, 513 samples concerning 897 immune-related genes were utilized to further analyze the model. The clinical characteristics of the patients enrolled in the study are presented in Table S2. Next, the 513 samples were divided into a training set (*n* = 256) and a test set (*n* = 257) such that the two populations satisfied the following criteria: (1) samples were randomly divided into training and testing sets; (2) age distribution, clinical stage, follow-up time, and ratio of death between the two groups were similar. The final training set data are presented in Table S3, clinical follow-up information is shown in Table S4, testing set data are displayed in Table S5, and clinical follow-up information is illustrated in Table S6.

### Univariate and Multifactor Survival Analysis of the Training Set Immune-Related Genes

All immune-related genes, as well as the survival data, were analyzed by the univariate and multivariate Cox proportional hazards regression model using the survival coxph function of the R package, and *p* < 0.05 was used as the significance level. The immune-related genes from the training set samples that were significant based on both univariate and multivariate analyses were selected as characteristic biomarkers for further analysis.

### Molecular Subtyping of Lung Adenocarcinoma, Screening of Prognosis-Specific Immune-Related Genes, and Construction of the Prognostic Prediction Model

First, the pheatmap from R software package (15) was used for the hierarchical clustering analysis of immune-related genes that were significant based on both univariate and multivariate analyses, and the similarity distances between the samples were calculated based on the Euclidean distance.

Moreover, glmnet from the R software package (16) was used for the LASSO Cox regression analysis, and the prognostic prediction model was constructed, which included 14 immune-related genes. The formula is as follows:

$$\begin{array}{l} \text{Risk Score}=0.06\;\times \text{ ENSG00000108671 }+\;0.611\\ \;\times \text{ ENSG00000196262 }+\;0.233\\ \;\times \text{ ENSG00000240972}+\;-0.139\\ \;\times \text{ ENSG00000112175 }+\;0.132\\ \;\times \text{ ENSG00000107984}+\;0.169\\ \;\times \text{ ENSG00000100311 }+\;0.055\\ \;\times \text{ ENSG00000167772}+\;0.295\\ \;\times \text{ ENSG00000115590 }+\;0.11\\ \;\times \text{ ENSG00000174564 }+\;0.488\\ \;\times \text{ ENSG00000111321 }+\;0.098\\ \;\times \text{ ENSG00000258839}+\;0.121\\ \;\times \text{ ENSG00000151090 }+\;-0.008\\ \;\times \text{ ENSG00000048462 }+\;0.524\\ \;\times \text{ ENSG00000067182}. \end{array}$$

Next, the expression profile data of the corresponding genes were extracted from the training set and substituted into the model to calculate the RiskScore of each sample. Based on this, survivalROC from the R software package (17) was further utilized for the receiver operating characteristic (ROC) curve analysis of the high- and low-risk prognostic classifications of the RiskScore. For this, the 1-, 3-, 5-, and 10-years prognostic prediction efficacies were analyzed.

### Functional Annotations and Signaling Pathway Enrichment Analysis of the Prognosis-Specific Immune-Related Genes

clusterProfiler from the R software package (18) was selected for functional gene annotations and KEGG enrichment analysis based on the aforementioned 14 prognosis-specific immune-related genes.

### Correlations Between the RiskScore and Clinical Features of Training Set Samples and Signaling Pathways

First, the distributions of the RiskScore among different clinical stages, invasion degrees, and lymph node metastasis degrees were analyzed. Next, stage classification was incorporated into the model to construct a multivariate regression model; 5-year survival predicted by the ROC curves for three models, namely, stage, RiskScore, and stage+RiskScore, were then compared. The median risk score of each model was used as the threshold to divide the samples into high- and low-risk groups to draw the Kaplan–Meier (KM) curve to evaluate differences in prognostic classifications among the three models. Then, the ssGSEA function of the R software package GSVA (19) was utilized to analyze the KEGG functional enrichment score of each sample in the training set. The correlation with the RiskScore was calculated based on the enrichment score of each sample for each pathway, and the top 20 most related KEGG pathways were selected for clustering analysis based on their enrichment scores.

### Test Set Sample Verification

The expression profile data of prognosis-specific immune-related genes were extracted from the test set and substituted into the model for calculation. The predicted results were calculated, and the prediction accuracy of this prognosis classification model, as well as the stability of immune feature recognition, was verified and analyzed.

### Statistical Analysis

The TCGA dataset was randomly divided in half into a training and testing set, where the training set was analyzed to identify potential prognostic genes and the testing set and entire set were used for validation.

First, Univariate Cox proportional hazards regression analysis was used to evaluate the association between the expression of immune-associated genes and patient overall survival (OS). Genes with a *p*-value of < 0.05 based on the log-rank test were selected as candidate variables. Second, the least absolute shrinkage and selection operator (LASSO)-Cox method was applied to reduce the number of candidate genes and to select the most significant immune-associated genes to build a prognostic risk score model. The formula of the risk score model is described as follows:

$$\begin{array}{l} \text{Riskscore}=\overset{\text{n}}{\underset{\text{i}=0}{\sum }}\beta i^{*}\chi i \end{array}$$

where βi refers to the coefficients of each gene and χi represents the expression value of the gene (FPKM). The risk score model was calculated for each patient and used to classify each patient into a low- or high-risk group based on the median risk score of the training dataset as the cutoff. Patients in the low-risk group had a higher OS, and those included in the high-risk group had a lower OS. KM survival curves and log-rank tests were used to assess differences in OS between the predicted high- and low-risk groups. The sensitivity and specificity of the diagnostic and prognostic prediction models were analyzed by the ROC curve and quantified based on the area under the ROC curve (AUC). All statistical tests were two-sided and *p*-values < 0.05 were considered statistically significant. All statistical analyses were performed using R software (version 3.5.0) and Bio-conductor.

## Results

### Mining of Characteristic Immune-Related Genes Based on the Survival and Prognosis Results of Lung Adenocarcinoma Patients

First, a series of data downloaded from TCGA and ImmPort databases were pre-processed (see Materials and Methods). Survival data for each immune-related gene were subjected to univariate Cox proportional hazards regression model analysis using the survival coxph function of the R package, with the significance threshold set as *p* < 0.05, as shown in Table S7. Eventually, 134 immune-related genes that were significantly differentially expressed with respect to prognosis were discovered, and the top 20 are shown in Table 1. Similarly, our results indicated that, T, N, and stage were also significantly correlated with prognosis, with log-rank *p*-values of 0.0002, 9.371E−06, and 2.15E−08, respectively (Table 2). Moreover, the univariate Cox model was used to select significant immune-related genes for multivariate Cox proportional hazards regression model analysis, with T, N, and stage used as the co-variants for this model. Finally, 64 significant immune-related genes were obtained (see Table S8), and immune-related genes (*n* = 64) from the training set samples that were significant based on both univariate and multivariate analyses were selected as characteristic biomarkers for further analysis.

**Table 1:** Top 20 immune-related genes regarding prognosis.

**Genes**	**Symbol**	***p* value**	**HR**	**Low 95%CI**	**High 95%CI**
ENSG00000111321.9	AC005840.1	3.92E−08	1.0105	1.0068	1.0144
ENSG00000067182.6	TNFRSF1A	1.35E−07	1.0075	1.0047	1.0103
ENSG00000172819.15	RARG	1.52E−07	1.0207	1.0129	1.0286
ENSG00000197747.7	S100A10	4.96E−07	1.0006	1.0004	1.0009
ENSG00000107984.8	DKK1	1.88E−06	1.0054	1.0031	1.0076
ENSG00000188643.9	S100A16	7.47E−06	1.0011	1.0006	1.0016
ENSG00000087191.11	PSMC5	1.13E−05	1.0136	1.0075	1.0198
ENSG00000185033.13	SEMA4B	4.28E−05	1.0026	1.0013	1.0038
ENSG00000108671.8	PSMD11	4.46E−05	1.0110	1.0057	1.0163
ENSG00000006831.9	ADIPOR2	6.08E−05	1.0159	1.0081	1.0238
ENSG00000163191.5	S100A11	7.34E−05	1.0002	1.0001	1.0003
ENSG00000174564.11	IL20RB	7.38E−05	1.0092	1.0046	1.0139
ENSG00000150630.3	VEGFC	0.000148	1.0105	1.0050	1.0159
ENSG00000011422.10	PLAUR	0.000281	1.0072	1.0033	1.0111
ENSG00000101000.4	PROCR	0.000312	1.0051	1.0023	1.0080
ENSG00000160691.17	SHC1	0.000379	1.0027	1.0012	1.0042
ENSG00000184009.8	ACTG1	0.00058	1.0001	1.0000	1.0002
ENSG00000213281.4	NRAS	0.000666	1.0111	1.0047	1.0176
ENSG00000167772.10	ANGPTL4	0.000838	1.0027	1.0011	1.0043
ENSG00000095539.14	SEMA4G	0.000883	1.0199	1.0081	1.0319

**Table 2:** Prognostic differences among clinical features.

**Clinical features**	**Sample size/death**	**Log rank *p-*value**
T	249/95	0.0002
N	245/94	9.371E−06
M	183/77	0.1468
Stage	248/94	2.15E−08
Age	347/93	0.9433
Gender	250/95	0.1767

### Employing Immune-Related Genes for Hierarchical Clustering of Lung Adenocarcinoma Subtypes and Clinical Feature Analysis

**Figure 1:**
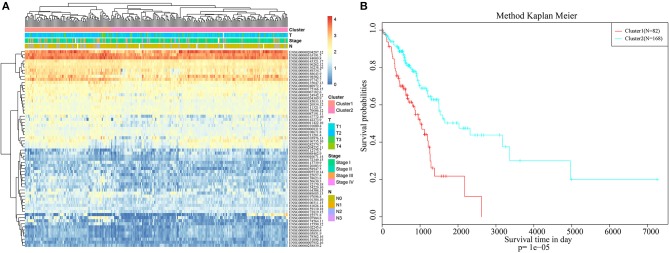
Hierarchical clustering analysis of the immune-related genes. **(A)** The heat map corresponding to the hierarchical clustering analysis that was generated using the pheatmap function with gene expression, TNM stage, clinico-pathological stage, and histological type as the annotations. **(B)** The survival curves of each immune sub-types in training set. The horizontal axis represents the survival time (days), and the vertical axis represents the probability of survival.

Pheatmap from the R software package was used for hierarchical cluster analysis of the immune-related genes that were significant based on both single-factor and multi-factor analyses, and similarity distances between samples were calculated according to the Euclidean distance, as shown in Figure 1A. These samples could be mainly clustered into two clusters, namely, Cluster1 and Cluster2. Among them, the proportion of samples associated with lymph node metastasis from Cluster1 was 47%, whereas that in Cluster2 was 26%; the difference between these two clusters was significant (chi-square test, *p* < 0.001). The proportion of samples exhibiting T1 invasion in Cluster1 was 22.6%, whereas that in Cluster2 was 39.8%, and this difference was also statistically significant (chi-square test, *p* < 0.001). The proportion of early-stage samples (Stage I and Stage II) in Cluster1 was 72.3%, whereas that in Cluster2 was 84.8%, and this difference was statistically significant (chi-square test, *p* < 0.001). Furthermore, the difference in prognosis between Cluster1 and Cluster2 was also analyzed, as presented in Figure 1B. There was also a significant difference in their prognoses (*p* < 0.001). These results suggested that immune-related genes could be used to predict prognosis in lung adenocarcinoma patients.

### Screening of Prognosis-Specific Immune-Related Genes and Construction of the Prognosis Prediction Model

**Figure 2:**
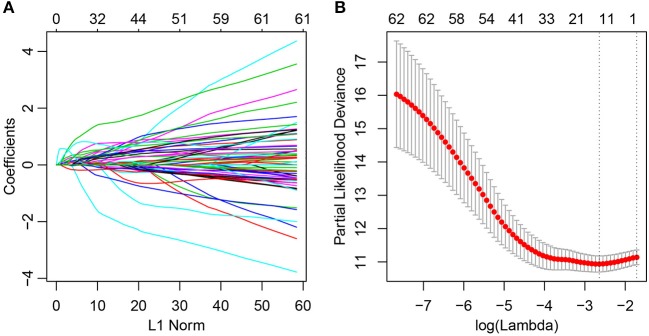
Construction of the prognosis prediction model for lung adenocarcinoma patients by LASSO. **(A)** The changing trajectory of each independent variable. The horizontal axis represents the log value of the independent variable lambda, and the vertical axis represents the coefficient of the independent variable. **(B)** Confidence intervals for each lambda.

At present, 64 immune-related genes have been recognized, which can be used to predict and distinguish prognostic differences between Cluster1 and Cluster2; however, this large number represents a disadvantage for clinical detection. Therefore, the number of immune-related genes was further narrowed such that high accuracy was maintained. The LASSO algorithm is a shrinkage estimate that can be used to construct a penalty function and obtain a relatively refined model; here, some coefficients can be shrunk and some are set to zero. Consequently, it preserves the advantages of subset shrinkage and is a biased estimate that can be utilized to process the multi-collinear data, which can estimate parameters while realizing variable selection, thus solving the multi-collinearity issue in regression analysis. In this study, glmnet from the R software package was used for LASSO Cox regression analysis. First, the change in trajectory of each independent variable was analyzed, as presented in Figure 2A, and this suggested that more independent variables had coefficients approaching zero with a gradual increase in lambda. Moreover, 3-fold cross-validation was also employed for model construction, and the confidence interval under each lambda is presented in Figure 2B. This revealed that the optimal model could be attained at lambda = 0.0711. As a result, this value was selected as the final model, which included 14 genes, and the model formula is shown in section Materials and Methods.

**Figure 3:**
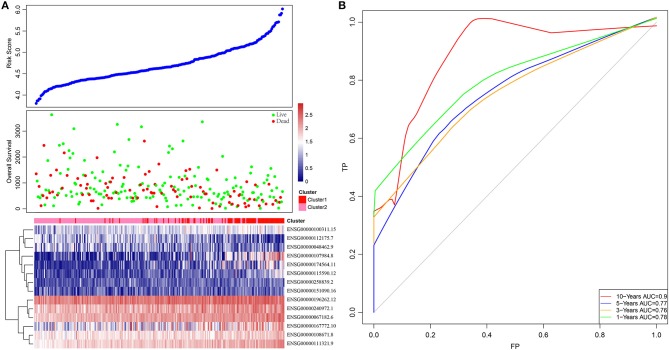
The relationship between RiskScore and patient outcome. **(A)** Comparison of RiskScore between each immune-related gene clusters in training set. The horizontal axis represents the samples, and the vertical axis represents RiskScores, overall survival, and immune-related gene expression, respectively. **(B)** 1-, 3-, 5-, and 10-years ROC analysis of prognosis classification for RiskScore.

Based on this, samples in each training set were substituted into the formula to calculate the sample RiskScore. The RiskScore distribution of Cluster1 and Cluster2 was then plotted, as shown in Figure 3A. It can be observed that the RiskScore in Cluster1 samples was generally greater than that in Cluster2 samples, whereas the OS in Cluster1 samples was remarkably lower than that in Cluster2, suggesting that samples with a high RiskScore had a poorer prognosis. Further, survivalROC of the R software package was adopted to perform ROC analysis of the prognostic classification of RiskScore. The 1-, 3-, 5-, and 10-years prognosis prediction classification efficiencies were analyzed, as shown in Figure 3B. It could be seen that the model demonstrated a high area under the curve (AUC), with an average value > 0.8.

### Functional Annotations of Prognosis-Specific Immune-Related Genes and Signaling Pathway Enrichment 1

**Figure 4:**
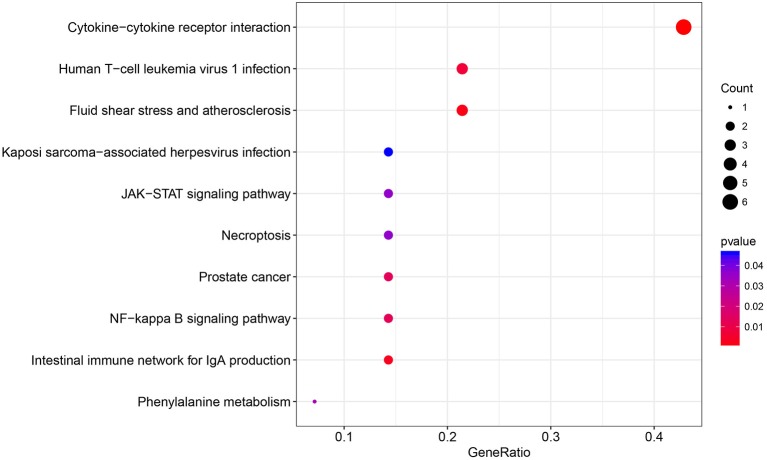
The KEGG pathway enrichment analysis of the 14 specific immune-related genes.

The immune relationships among these 14 genes were then analyzed, as shown in Table 3. It could be observed that 8 (57%) were related to Cytokine_Receptors. Subsequently, clusterProfiler of the R software package was used to perform KEGG enrichment analysis of these 14 genes with a threshold of *p* < 0.05. Finally, 10 significantly enriched pathways were obtained, as presented in Figure 4. Six genes were enriched in the cytokine–cytokine receptor interactions, whereas three were enriched in human T-cell leukemia virus 1 infection.

**Table 3:** The immune relationships of the 14 genes.

**ENSG**	**Symbol**	**Name**	**Category**
ENSG00000108671	PSMD11	Proteasome (prosome, macropain) 26s subunit, non-atpase, 11	Antigen_Processing_and_Presentation
ENSG00000196262	PPIA	Peptidylprolyl isomerase a (cyclophilin a)	Antimicrobials
ENSG00000240972	MIF	Macrophage migration inhibitory factor (glycosylation-inhibiting factor)	Antimicrobials
ENSG00000112175	BMP5	Bone morphogenetic protein 5	Cytokines
ENSG00000107984	DKK1	Dickkopf homolog 1 (xenopus laevis)	Cytokines
ENSG00000100311	PDGFB	Platelet-derived growth factor beta polypeptide (simian sarcoma viral (v-sis) oncogene homolog)	Cytokines
ENSG00000167772	ANGPTL4	Angiopoietin-like 4	Cytokine_Receptors
ENSG00000115590	IL1R2	Interleukin 1 receptor, type ii	Cytokine_Receptors
ENSG00000174564	IL20RB	Interleukin 20 receptor beta	Cytokine_Receptors
ENSG00000111321	LTBR	Lymphotoxin beta receptor (tnfr superfamily, member 3)	Cytokine_Receptors
ENSG00000258839	MC1R	Melanocortin 1 receptor (alpha melanocyte stimulating hormone receptor)	Cytokine_Receptors
ENSG00000151090	THRB	Thyroid hormone receptor, beta (erythroblastic leukemia viral (v-erb-a) oncogene homolog 2, avian)	Cytokine_Receptors
ENSG00000048462	TNFRSF17	Tumor necrosis factor receptor superfamily, member 17	Cytokine_Receptors
ENSG00000067182	TNFRSF1A	Tumor necrosis factor receptor superfamily, member 1a	Cytokine_Receptors

### Correlation Between the RiskScore and the Clinical Features of Training Set Samples and Signaling Pathways

First, the difference in the prediction accuracy between the RiskScore-based prognosis prediction model and the clinical feature-based model was compared. Specifically, the differences in prognosis prediction were analyzed from single-factor and multi-factor points of view based on age, sex, stage, T and N values, and RiskScore. The results are presented in Table 4 and suggest that the RiskScore had the smallest significant predictive *p* value.

**Table 4:** Multivariable Cox regression analysis.

	***p* value**	**HR**	**Low 95% CI**	**High 95% CI**
14-immune-related genes risk score	3.77E−15	5.7654	3.7256	8.9221
Age	0.9432	1.0008	0.9778	1.0244
Gender	0.1780	1.3193	0.8814	1.9748
AJCC stage				
Stage I–II vs III–IV	6.34E−06	2.8262	1.800	4.4371
AJCC stage T				
T1-vs-T2	0.4913	1.1817	0.7345	1.9011
T1-vs-T3	0.0014	1.7572	1.2421	2.4860
T1-vs-T4	0.0464	1.5087	1.0065	2.2617
AJCC stage N				
N0 vs N1–N3	1.86E−06	2.7910	1.8304	4.2558

**Figure 5:**
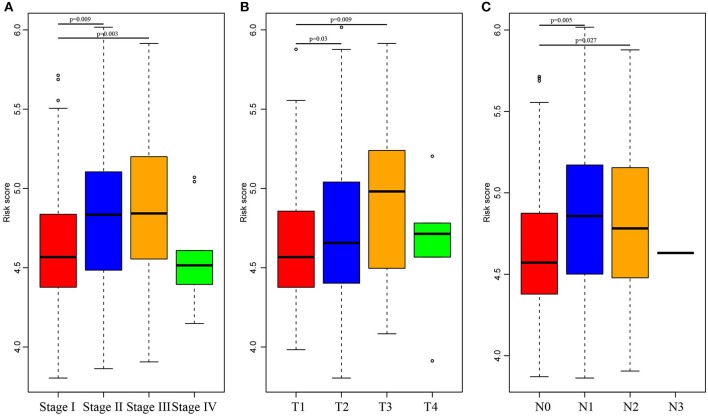
Comparison of RiskScore among different stages, invasion degree, and lymph node metastasis degree. The horizontal axis represents the different stages **(A)**, invasion degree **(B)**, and lymph node metastasis degree **(C)**, and the vertical axis represents RiskScores.

Subsequently, the RiskScore distribution among different clinical stages, invasion degrees, and lymph node metastasis degrees was analyzed, as shown in Figure 5. The RiskScores among different stages were statistically significant, and a higher stage was associated with a higher RiskScore. Similar phenomena could also be observed for invasion degree and lymph node metastasis degree, which revealed that the RiskScore was potentially associated with the clinical stage.

**Figure 6:**
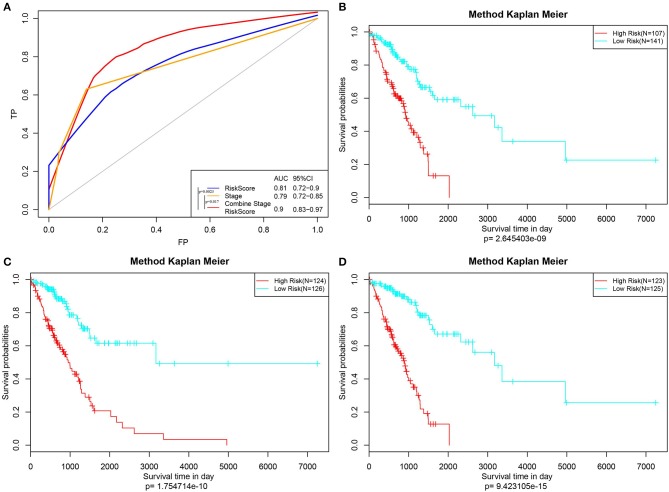
Construction of the prognosis prediction model by Stage, RiskScore, and Stage+RiskScore, and comparison of the reliability of prediction. **(A)** The 5-year survival predicted ROC curves of three distinct models, namely, Stage, RiskScore, and Stage+RiskScore, were compared. **(B–D)** Three models (Stage, RiskScore, and Stage+RiskScore) were used as the threshold to divide the samples into high and low risk groups, and the K–M curve was plotted.

**Figure 7:**
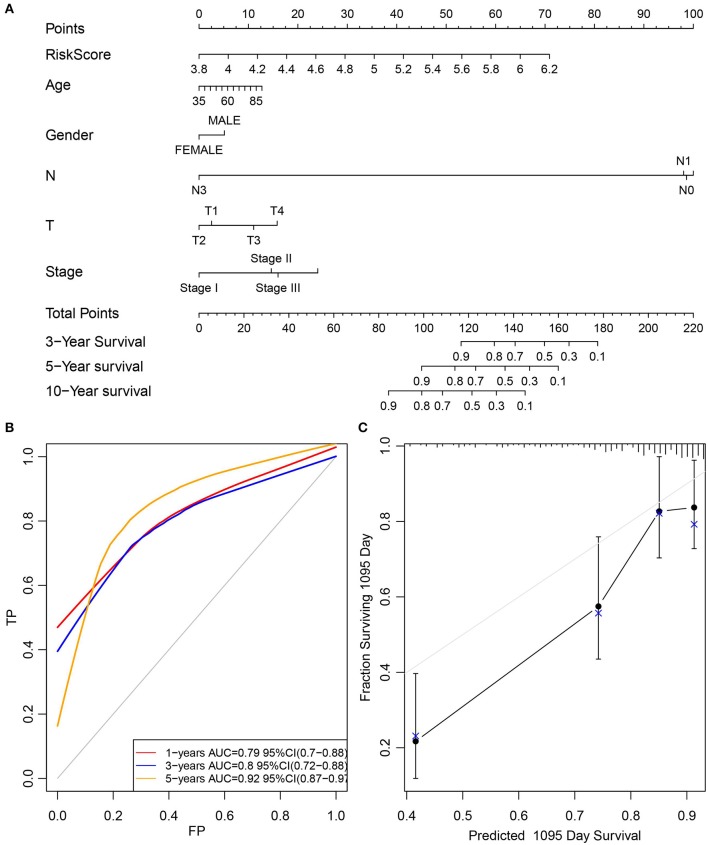
Construction of the nomogram model by combined age, gender, T, N, and stage with RiskScore. **(A)** The clinical features, including age, gender, T, N, and stage, were combined with RiskScore to construct the nomogram model. **(B)** The 1-, 3-, and 5-years survival predicted ROC curves were plotted. **(C)** The calibration plots for predicting patient 3-year OS. Nomogram-predicted probability of survival is plotted on the *x*-axis; actual survival is plotted on the *y*-axis.

Furthermore, the stage was incorporated into the model to construct the multivariate regression model. The 5-year survival predicted ROC curves of the three single models, namely, stage, RiskScore, and stage+RiskScore, were compared as presented in Figure 6A. Clearly, the AUC values followed the order stage+RiskScore > RiskScore > stage, and the stage+RiskScore value was significantly higher than those in the other two models. The respective median RiskScores of the three models were then used as thresholds to divide the samples into high- and low-risk groups, and a K–M curve was plotted, as shown in Figures 6B–D. Obviously, the stage+RiskScore model was associated with the most significant difference in prognosis. Based on this, the clinical features including age, sex, T, N, and stage were combined with the RiskScore to construct the nomogram model and to plot the 1-, 3-, and 5-years survival predicted by the ROC curves (Figures 7A–C).

**Figure 8:**
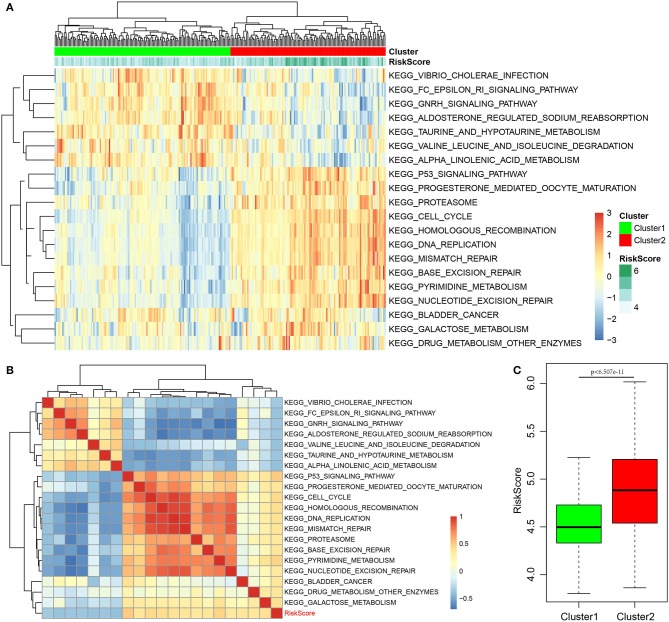
Correlation of RiskScore with signaling pathways. **(A)** KEGG functional enrichment score of each sample in the training set was analyzed, the correlation with RiskScore was calculated, respectively, based on the enrichment score of each pathway in each sample, and the top 20 most related KEGG pathways were shown. Clustering analysis had to be carried out according to the enrichment score. **(B)** The correlations of these 20 pathways with RiskScore. **(C)** The RiskScore distribution of two clusters was analyzed.

Finally, the ssGSEA function of GSVA in the R software package was used to analyze the KEGG functional enrichment score of each sample in the training set. The respective correlations with RiskScores were also calculated based on the enrichment score of each pathway in each sample, and the top 20 most related KEGG pathways were selected. Clustering analysis was performed according to enrichment scores, as presented in Figure 8A. Clearly, most samples were enriched in pathways closely correlated with tumorigenesis, such as P53, cell cycle, and DNA repair. These pathways could group the samples into two clusters, namely, Cluster1 and Cluster2. The RiskScore of Cluster2 was higher than that of Cluster1 and the RiskScore distribution of the two clusters was analyzed, as displayed in Figure 8C. The RiskScore of Cluster2 samples was remarkably higher than that of Cluster1. Meanwhile, the correlations between these 20 pathways and RiskScore are presented in Figure 8B. Based on this, seven pathways had a negative correlation, whereas 13 had a positive correlation, with an average correlation coefficient of 0.388.

### Test Set Sample Verification

**Figure 9:**
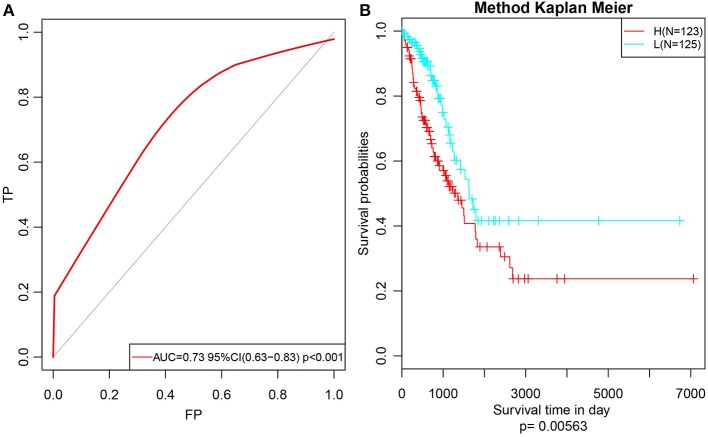
Verify the stability and reliability of the prognosis prediction model for lung adenocarcinoma patients in test set. **(A)** The RiskScore of each sample was calculated, and the ROC curves were plotted based on the RiskScore. **(B)** The prognostic difference after predicted classification by RiskScore in test set.

Subsequently, to further verify the stability and reliability of the prognostic prediction model, expression profile data of these 14 genes were extracted from the test set and substituted into the model for verification. The RiskScore of each sample was calculated, and the ROC curves were plotted based on these values; as presented in Figure 9A, the AUC was > 0.7. Furthermore, the median was used to divide the samples into high- and low-risk groups and to analyze the difference in prognosis between the two groups, as presented in Figure 9B. Clearly, there was a significant difference in prognosis between these two clusters with a *p*-value of 0.00563. Moreover, the prognosis for low-risk samples was remarkably superior to that for the other samples, which was consistent with the training set data. In summary, the prognosis model constructed based on the expression profiles of these 14 prognosis-specific immune-related genes had high predictive accuracy and stability to identify immune features.

## Discussion

Early lung adenocarcinoma is associated with high risks of recurrence and death after surgery (20). However, patients cannot achieve consistent therapeutic benefits from certain drugs due to their potential toxicities and side effects, and as a result, the application of systemic adjuvant chemotherapy after surgery remains a source of controversy in clinics (21). Therefore, it is of importance to mine potential lung adenocarcinoma biomarkers that could be used to predict patient prognosis and recurrence, and to identify high-risk patients who would benefit from early adjuvant chemotherapy.

In the era of immunotherapy, it is of crucial significance to mine molecular events that are related to the tumor immune microenvironment to uncover predictive biomarkers associated with survival. The literature suggests that the FoxP3:CD3 ratio in the tumor matrix, as well as expression levels of IL-12 and IL-17, is markedly correlated with post-operative recurrence in early lung adenocarcinoma (22–24). Moreover, the infiltration of numerous immune factors and immunocytes (including neutrophils, macrophages, and lymphocytes) has also been reported to correlate with angiogenesis, cell proliferation, and invasion of this disease (25).

In this study, 14 prognosis-specific immune-related genes were discovered through the mining, statistical analysis, and sorting of large datasets such as TCGA and ImmPort. From this, a prognostic prediction model was constructed, the RiskScore of patients was calculated, and prediction and verification were also carried out. Among all 14 prognosis-specific immune-related genes, 9 (e.g., *PSMD11, PPIA, MIF, BMP5, DKK1, PDGFB, ANGPTL4, IL1R2*, and *THRB*) (26–34) have been reported to be involved in the immune microenvironment-associated pathogenesis of lung adenocarcinoma or suggested to be significant predictors of recurrence-free or overall survival. This implies that our bioinformatics analysis using TCGA and ImmPort cohorts has prognostic value. The remaining five genes have not been previously associated with lung adenocarcinoma prognosis and could serve as new potential biomarkers for this disease. These include *IL20RB* (interleukin 20 receptor beta), *LTBR, TNFRSF1, TNFRSF17* (TNFR superfamily genes), and *MC1R* (melanocortin 1 receptor).

We are particularly interested in *TNFRSF1A* and *LTBR*. From a protein–protein interaction network generated in the previous studies, both were found to be highly interconnected nodes. These two genes encode tumor necrosis factor receptors, which have been repeatedly shown to take part in multiple tumor processes such as proliferation, metastasis, and angiogenesis. These receptors are not only expressed on some tumor cells but also on suppressive immune cells including regulatory T cells and myeloid-derived suppressor cells. They convert the tumor-inhibitory TNF into a tumor-promoting factor, and not only directly enhance the proliferation of some types of tumor cells, but also activate immunosuppressive cells and support immune escape and tumor development (35, 36). In addition, these two markers can induce tumor-infiltrating T-cell apoptosis and contribute to failed patient responses to immunotherapy. These can also shape the tumor microenvironment via FasL/Fas-mediated cell apoptosis induced by other cells in the tumor microenvironment, such as cancer cells, endothelial cells, and myeloid-derived suppressor cells (37).

Previous reports have provided elegant analyses regarding how the activation of tumor-intrinsic genes shapes the tumor microenvironment. In the current work, the proposed model, constructed based on the expression profiles of specific immune-related genes, could further classify patients with defined clinical stages into different subgroups based on predicted survival. Moreover, the RiskScore, calculated based on the expression profiles of specific immune-related genes, could be used in combination with clinical features to predict the survival of lung adenocarcinoma patients more precisely. Although we used bioinformatics to identify prognosis-specific immune-related genes involved in lung adenocarcinoma, the limitations of this study should be noted. The proposed validation cohort is based on retrospective data from TCGA, so the models need further validation in large sample clinical studies.

In conclusion, this model contributes new clinical lung adenocarcinoma markers. These data not only provide multiple targets for precise lung adenocarcinoma treatment, but also could be used to more accurately classify lung adenocarcinoma patients at the molecular subtype level. Furthermore, this model could be used to guide clinicians in decisions related to prognosis, clinical diagnosis, and medication for lung adenocarcinoma patients with different immunophenotypes.

## Data Availability Statement

All datasets generated for this study are included in the article/Supplementary Material.

## Author Contributions

KZ, YW, and MZ conceived, designed, planned the study, and helped interpret the results. ZW, HP, and HZ analyzed the data. JZ and HZ acquired data. HP and HZ provided study materials or patients. MZ, KZ, and YW drafted the manuscript. All authors revised and reviewed this work, and gave their final approval of the submitted manuscript.

### Conflict of Interest

The authors declare that the research was conducted in the absence of any commercial or financial relationships that could be construed as a potential conflict of interest.
