# Supplementary material for: An Immune-Related Signature Predicts Survival in Patients With Lung Adenocarcinoma
Source: Front Oncol. 2019 Dec 10;9:1314. doi: 10.3389/fonc.2019.01314 (PMC6914845; doi:10.3389/fonc.2019.01314)
Supplement: Supplementary file 1 [file Table_1.doc]

**Table S1. Clinical follow-up information of samples.**

A0_Samples A1_OS A2_Event A3_T A4_N A5_M A6_Stage additional_studies additional_studies.additional_study.disease_code additional_studies.additional_study.project_code age_at_initial_pathologic_diagnosis anatomic_neoplasm_subdivision anatomic_neoplasm_subdivision_other bcr_patient_barcode bcr_patient_uuid day_of_form_completion days_to_birth days_to_death days_to_initial_pathologic_diagnosis days_to_last_followup days_to_last_known_alive diagnosis dlco_predictive_percent drugs drugs.drug.bcr_drug_barcode drugs.drug.bcr_drug_uuid drugs.drug.clinical_trail_drug_classification drugs.drug.day_of_form_completion drugs.drug.days_to_drug_therapy_end drugs.drug.days_to_drug_therapy_start drugs.drug.drug_name drugs.drug.measure_of_response drugs.drug.month_of_form_completion drugs.drug.number_cycles drugs.drug.prescribed_dose drugs.drug.prescribed_dose_units drugs.drug.regimen_indication drugs.drug.regimen_indication_notes drugs.drug.regimen_number drugs.drug.route_of_administrations.route_of_administration drugs.drug.therapy_ongoing drugs.drug.therapy_types.therapy_type drugs.drug.therapy_types.therapy_type_notes drugs.drug.total_dose drugs.drug.total_dose_units drugs.drug.tx_on_clinical_trial drugs.drug.year_of_form_completion eastern_cancer_oncology_group egfr_mutation_identified egfr_mutation_performed egfr_mutation_result eml4_alk_translocation_identified eml4_alk_translocation_method eml4_alk_translocation_performed eml4_alk_translocation_result ethnicity follow_ups follow_ups.follow_up.additional_pharmaceutical_therapy follow_ups.follow_up.additional_radiation_therapy follow_ups.follow_up.additional_surgery_locoregional_procedure follow_ups.follow_up.additional_surgery_metastatic_procedure follow_ups.follow_up.bcr_followup_barcode follow_ups.follow_up.bcr_followup_uuid follow_ups.follow_up.day_of_form_completion follow_ups.follow_up.days_to_additional_surgery_locoregional_procedure follow_ups.follow_up.days_to_additional_surgery_metastatic_procedure follow_ups.follow_up.days_to_death follow_ups.follow_up.days_to_last_followup follow_ups.follow_up.days_to_new_tumor_event_after_initial_treatment follow_ups.follow_up.eastern_cancer_oncology_group follow_ups.follow_up.followup_case_report_form_submission_reason follow_ups.follow_up.followup_treatment_success follow_ups.follow_up.karnofsky_performance_score follow_ups.follow_up.lost_follow_up follow_ups.follow_up.month_of_form_completion follow_ups.follow_up.new_neoplasm_event_types.new_neoplasm_event_type follow_ups.follow_up.new_tumor_event_after_initial_treatment follow_ups.follow_up.performance_status_scale_timing follow_ups.follow_up.person_neoplasm_cancer_status follow_ups.follow_up.postoperative_rx_tx follow_ups.follow_up.primary_therapy_outcome_success follow_ups.follow_up.progression_determined_by_list.progression_determined_by follow_ups.follow_up.radiation_therapy follow_ups.follow_up.vital_status follow_ups.follow_up.year_of_form_completion gender histological_type history_of_neoadjuvant_treatment icd_10 icd_o_3_histology icd_o_3_site informed_consent_verified karnofsky_performance_score kras_gene_analysis_performed kras_mutation_found kras_mutation_result location_in_lung_parenchyma month_of_form_completion new_tumor_events.new_tumor_event.additional_pharmaceutical_therapy new_tumor_events.new_tumor_event.additional_radiation_therapy new_tumor_events.new_tumor_event.days_to_new_tumor_event_after_initial_treatment new_tumor_events.new_tumor_event.locoregional_procedure.additional_surgery_locoregional_procedure new_tumor_events.new_tumor_event.locoregional_procedure.days_to_additional_surgery_locoregional_procedure new_tumor_events.new_tumor_event.metastatic_procedure.additional_surgery_metastatic_procedure new_tumor_events.new_tumor_event.metastatic_procedure.days_to_additional_surgery_metastatic_procedure new_tumor_events.new_tumor_event.new_neoplasm_event_types.new_neoplasm_event_type new_tumor_events.new_tumor_event.progression_determined_by_list.progression_determined_by new_tumor_events.new_tumor_event_after_initial_treatment number_pack_years_smoked other_dx patient_id performance_status_scale_timing person_neoplasm_cancer_status post_bronchodilator_fev1_fvc_percent post_bronchodilator_fev1_percent postoperative_rx_tx pre_bronchodilator_fev1_fvc_percent pre_bronchodilator_fev1_percent primary_therapy_outcome_success pulmonary_function_test_performed race_list.race radiation_therapy radiations radiations.radiation.anatomic_treatment_site radiations.radiation.bcr_radiation_barcode radiations.radiation.bcr_radiation_uuid radiations.radiation.course_number radiations.radiation.day_of_form_completion radiations.radiation.days_to_radiation_therapy_end radiations.radiation.days_to_radiation_therapy_start radiations.radiation.measure_of_response radiations.radiation.month_of_form_completion radiations.radiation.numfractions radiations.radiation.radiation_dosage radiations.radiation.radiation_treatment_ongoing radiations.radiation.radiation_type radiations.radiation.radiation_type_notes radiations.radiation.regimen_indication radiations.radiation.regimen_indication_notes radiations.radiation.units radiations.radiation.year_of_form_completion residual_tumor stage_event.ann_arbor.b_symptoms stage_event.ann_arbor.extranodal_involvement stage_event.clinical_stage stage_event.gleason_grading.gleason_score stage_event.gleason_grading.primary_pattern stage_event.gleason_grading.secondary_pattern stage_event.gleason_grading.tertiary_pattern stage_event.igcccg_stage stage_event.masaoka_stage stage_event.pathologic_stage stage_event.psa.days_to_psa stage_event.psa.psa_value stage_event.serum_markers stage_event.system_version stage_event.tnm_categories.clinical_categories.clinical_M stage_event.tnm_categories.clinical_categories.clinical_N stage_event.tnm_categories.clinical_categories.clinical_T stage_event.tnm_categories.pathologic_categories.pathologic_M stage_event.tnm_categories.pathologic_categories.pathologic_N stage_event.tnm_categories.pathologic_categories.pathologic_T stopped_smoking_year tissue_prospective_collection_indicator tissue_retrospective_collection_indicator tissue_source_site tobacco_smoking_history tumor_tissue_site vital_status year_of_form_completion year_of_initial_pathologic_diagnosis year_of_tobacco_smoking_onset

TCGA-55-8205 599 Alive T2b N0 M0 Stage IIA 76 R-Lower Not Applicable TCGA-55-8205 95fa64b0-91e1-4427-b81a-53c47a053563 17 -28063 Not Applicable 0 33 Not Available Lung Adenocarcinoma Not Available TCGA-55-8205-D59042;TCGA-55-8205-D59043;TCGA-55-8205-D59044 0EC2801F-BAED-4EC4-973E-BE336BDA8894;E6B41481-C1F8-465D-8757-E2F2F2B0A4DB;DA27B801-8C82-4033-87DD-101B032FF22E Not Available;Not Available;Not Available 30;30;30 154;154;Not Available 93;93;488 Carboplatin;Alimta;Tarceva Complete Response;Complete Response;Not Applicable 4;4;4 Not Available;Not Available;Not Available Not Available;Not Available;Not Available Not Available;Not Available;Not Available Not Available;Not Available;Not Available Not Applicable;Not Applicable;Not Applicable Not Available;Not Available;Not Available Not Available;Not Available;Not Available NO;NO;YES Chemotherapy;Chemotherapy;Targeted Molecular therapy Not Available;Not Available;Not Available Not Available;Not Available;Not Available Not Available;Not Available;Not Available NO;NO;NO 2014;2014;2014 0 Not Available YES Not Available Not Available Not Available NO Not Available NOT HISPANIC OR LATINO YES YES Not Available NO TCGA-55-8205-F59040 324D6FE4-9E52-45A9-82EB-03C088BC06E3 30 Not Available Not Available Not Applicable 599 495 Unknown Scheduled Follow-up Submission Progressive Disease Unknown NO 4 Distant Metastasis YES Not Available WITH TUMOR YES Progressive Disease Convincing Imaging NO Alive 2014 FEMALE Lung Adenocarcinoma- Not Otherwise Specified (NOS) No C34.3 8140/3 C34.3 YES 100 YES NO Not Available Central Lung 6 Not Available Not Available Not Available Not Available Not Available Not Available Not Available Not Available Not Available NO 30 Yes, History of Prior Malignancy 8205 Preoperative TUMOR FREE Not Available Not Available Unknown 73 42 Unknown YES WHITE Unknown Distant Recurrence TCGA-55-8205-R59041 78705F45-C5A7-452B-9548-2FA5A83DD7C9 Not Available 30 525 508 Radiographic Progressive Disease 4 14 3500 NO External Not Applicable Not Available Not Available cGy 2014 R0 Not Applicable Not Applicable Not Applicable Not Applicable Not Applicable Not Applicable Not Applicable Not Applicable Not Applicable Stage IIA Not Applicable Not Applicable Not Applicable 7th Not Applicable Not Applicable Not Applicable M0 N0 T2b 2012 YES NO 55 4 Lung Alive 2012 2012 1951

TCGA-97-A4M0 652 Alive T2a N0 M0 Stage IB 60 R-Upper Not Applicable TCGA-97-A4M0 DD63D0FD-E24B-40A5-88C5-7D3D4106631E 7 -22206 Not Applicable 0 216 Not Available Lung Adenocarcinoma Not Available 0 Not Available NO Not Available Not Available Not Available NO Not Available NOT HISPANIC OR LATINO Not Available Not Available Not Available Not Available TCGA-97-A4M0-F57385 551C91CF-3AEC-44DA-BF0F-DAB40F977E32 10 Not Applicable Not Applicable Not Applicable 652 Not Applicable 0 Scheduled Follow-up Submission Complete Remission/Response Unknown NO 3 Not Available NO Other TUMOR FREE NO Complete Remission/Response Not Available NO Alive 2014 FEMALE Lung Adenocarcinoma Mixed Subtype No C34.1 8255/3 C34.1 YES Not Evaluated YES YES G12C Unknown 3 Not Available Not Available Not Available Not Available Not Available Not Available Not Available Not Available Not Available NO 34 No A4M0 Preoperative Unknown Not Available Not Available NO 90 97 Complete Remission/Response YES WHITE NO Not Evaluated Not Applicable Not Applicable Not Applicable Not Applicable Not Applicable Not Applicable Not Applicable Not Applicable Not Applicable Stage IB Not Applicable Not Applicable Not Applicable 7th Not Applicable Not Applicable Not Applicable M0 N0 T2a 2004 YES NO 97 4 Lung Alive 2013 2012 1970

TCGA-05-5420 457 Alive T2 N2 M0 Stage IIIA 67 L-Upper Not Applicable TCGA-05-5420 8b119d1c-6d21-4bbd-8a00-12da7b97d6c4 22 -24472 Not Applicable 0 31 Not Available Lung Adenocarcinoma Not Available Not Available Not Available Not Available Not Available Not Available Not Available Not Available Not Available Not Available Unknown Unknown Not Available Not Available TCGA-05-5420-F36422 5E6ACDEB-BC35-4B1C-BF79-F54891E7274D 30 Not Available Not Available Not Applicable 457 245 Not Available Scheduled Follow-up Submission Progressive Disease Not Available NO 10 Not Available YES Not Available WITH TUMOR Unknown Progressive Disease Not Available Unknown Alive 2012 MALE Lung Adenocarcinoma- Not Otherwise Specified (NOS) No C34.1 8140/3 C34.9 YES Not Available Not Available Not Available Not Available Not Available 3 Not Available Not Available Not Available Not Available Not Available Not Available Not Available Not Available Not Available Not Available 40 Yes 5420 Not Available TUMOR FREE Not Available Not Available Not Available Not Available Not Available Not Available Not Available Not Available Not Available R0 Not Applicable Not Applicable Not Applicable Not Applicable Not Applicable Not Applicable Not Applicable Not Applicable Not Applicable Stage IIIA Not Applicable Not Applicable Not Applicable 6th Not Applicable Not Applicable Not Applicable M0 N2 T2 2001 NO YES 05 4 Lung Alive 2011 2008 1961

TCGA-62-8398 444 Dead T2 N2 M0 Stage IIIA 55 R-Lower Not Applicable TCGA-62-8398 2fabeb98-05e3-4f55-97f5-fbc675e25a3d 4 -20197 444 0 Not Available Not Available Lung Adenocarcinoma Not Available TCGA-62-8398-D33507;TCGA-62-8398-D40458 316E364F-9824-4D22-AB44-6C9181EF3AAA;BB70829B-5995-4D0D-ABEF-22D1AD108527 Not Available;Not Available 5;21 113;113 41;41 Carboplatin;Vinorelbin Complete Response;Complete Response 7;2 Not Available;Not Available Not Available;Not Available Not Available;Not Available Not Available;Not Available Not Applicable;Not Applicable Not Available;Not Available Not Available;Not Available NO;NO Chemotherapy;Chemotherapy Not Available;Not Available Not Available;Not Available Not Available;Not Available NO;NO 2012;2013 Unknown Not Available NO Not Available Not Available Not Available NO Not Available NOT HISPANIC OR LATINO MALE Lung Adenocarcinoma Mixed Subtype No C34.3 8255/3 C34.3 YES 100 NO Not Available Not Available Central Lung 7 Not Available Not Available Not Available Not Available Not Available Not Available Not Available Not Available Not Available NO 20 No 8398 Preoperative TUMOR FREE Not Available Not Available YES 72 64 Complete Remission/Response YES WHITE NO R0 Not Applicable Not Applicable Not Applicable Not Applicable Not Applicable Not Applicable Not Applicable Not Applicable Not Applicable Stage IIIA Not Applicable Not Applicable Not Applicable 6th Not Applicable Not Applicable Not Applicable M0 N2 T2 1999 NO YES 62 4 Lung Dead 2012 2007 1970

TCGA-44-2657 1351 Alive T2 NX M0 Stage IB 74 L-Upper Not Applicable TCGA-44-2657 f40301ba-831e-4afd-9ce8-5f3c1a05ff7e 8 -27298 Not Applicable 0 400 Not Available Lung Adenocarcinoma 96 0 Not Available Not Available Not Available Not Available Not Available Not Available Not Available NOT HISPANIC OR LATINO Not Available;Not Available Not Available;Not Available Not Available;Not Available Not Available;Not Available TCGA-44-2657-F5295;TCGA-44-2657-F39296 c8b44a22-7cab-4d8d-a222-c557b242790d;AF57BA2D-C882-45E5-B422-31170E8D4F3C 8;16 Not Applicable;Not Applicable Not Applicable;Not Applicable Not Applicable;Not Applicable 400;1351 Not Applicable;Not Applicable 0;Not Available Not Available;Scheduled Follow-up Submission Complete Remission/Response;Complete Remission/Response Not Available;Not Available Not Available;NO 10;1 Not Available;Not Available NO;NO Pre-Adjuvant Therapy;Not Available TUMOR FREE;TUMOR FREE NO;NO Complete Remission/Response;Complete Remission/Response Not Available;Not Available NO;NO Alive;Alive 2010;2013 FEMALE Lung Adenocarcinoma- Not Otherwise Specified (NOS) No C34.1 8140/3 C34.1 YES Not Available NO Not Available Not Available Not Available 10 Not Available Not Available Not Available Not Available Not Available Not Available Not Available Not Available Not Available Not Available 25 No 2657 Pre-Adjuvant Therapy TUMOR FREE 87 147 Not Available 78 120 Not Available YES WHITE Not Available R0 Not Applicable Not Applicable Not Applicable Not Applicable Not Applicable Not Applicable Not Applicable Not Applicable Not Applicable Stage IB Not Applicable Not Applicable Not Applicable 6th Not Applicable Not Applicable Not Applicable M0 NX T2 2006 YES NO 44 4 Lung Alive 2010 2009 1959

TCGA-78-7633 1528 Dead T2 N0 M0 Stage IB 67 L-Upper Not Applicable TCGA-78-7633 44218b35-219c-4ad9-a01e-fde14067c4c0 31 -24713 Not Applicable 0 994 Not Available Lung Adenocarcinoma 57 0 Not Available NO Not Available Not Available Not Available NO Not Available Not Available Not Available;Unknown Not Available;Unknown Not Available;Not Available Not Available;Not Available TCGA-78-7633-F21095;TCGA-78-7633-F46157 f9269731-0d0b-4785-9c9b-6b9766317d29;8A95CDC5-FB75-4B7D-AE3F-923D2FF527B2 31;29 Not Available;Not Available Not Available;Not Available Not Applicable;1528 994;Not Available Not Available;1450 Not Available;Unknown Scheduled Follow-up Submission;Scheduled Follow-up Submission Not Available;Progressive Disease Not Available;Not Available Not Available;NO 1;8 Not Available;Locoregional Recurrence Not Available;YES Not Available;Not Available Not Available;WITH TUMOR Not Available;Unknown Not Available;Complete Remission/Response Not Available;Biopsy with Histologic Confirmation Not Available;Unknown Alive;Dead 2012;2013 MALE Lung Adenocarcinoma Mixed Subtype No C34.1 8255/3 C34.1 YES Not Available NO Not Available Not Available Not Available 1 Not Available Not Available Not Available Not Available Not Available Not Available Not Available Not Available Not Available Not Available 94 Yes 7633 Preoperative Not Available Not Available Not Available Not Available Not Available 100 Not Available YES WHITE Not Available R0 Not Applicable Not Applicable Not Applicable Not Applicable Not Applicable Not Applicable Not Applicable Not Applicable Not Applicable Stage IB Not Applicable Not Applicable Not Applicable 6th Not Applicable Not Applicable Not Applicable M0 N0 T2 2002 NO YES 78 4 Lung Alive 2012 2009 1955

TCGA-75-5126 T3 N2 M0 Stage IIIA Not Available L-Upper Not Applicable TCGA-75-5126 93766482-2f20-4c8f-bbd8-bdd203c37d5a 31 Not Available Not Applicable Not Available Not Available Not Available Lung Adenocarcinoma Not Available 1 Not Available NO Not Available Not Available Not Available NO Not Available Not Available NO NO NO Not Available TCGA-75-5126-F11746 8ee06ba1-ac92-4ac5-9748-27ba9607d9e7 4 Not Available Not Available Not Applicable Not Available Not Available 1 Not Available Progressive Disease Not Available Not Available 4 Not Available YES Adjuvant therapy WITH TUMOR NO Partial Remission/Response Not Available NO Alive 2011 FEMALE Lung Papillary Adenocarcinoma No C34.1 8260/3 C34.1 YES Not Available NO Not Available Not Available Peripheral Lung 3 Not Available Not Available Not Available Not Available Not Available Not Available Not Available Not Available Not Available Not Available 40 No 5126 Other WITH TUMOR Not Available Not Available Not Available Not Available Not Available Not Available Not Available Not Available Not Available R2 Not Applicable Not Applicable Not Applicable Not Applicable Not Applicable Not Applicable Not Applicable Not Applicable Not Applicable Stage IIIA Not Applicable Not Applicable Not Applicable Not Available Not Applicable Not Applicable Not Applicable M0 N2 T3 2007 NO YES 75 4 Lung Alive 2011 2007 Not Available

TCGA-55-8204 515 Alive T2a N0 MX Stage IB 87 L-Upper Not Applicable TCGA-55-8204 75589307-f141-4d0d-b35d-adbbc77b3dec 8 -31818 Not Applicable 0 0 Not Available Lung Adenocarcinoma 101 Unknown Not Available Unknown Not Available Not Available Not Available Unknown Not Available NOT HISPANIC OR LATINO Not Available Not Available Not Available Not Available TCGA-55-8204-F47802 A13C7C7D-9375-4938-BE6F-E877CD27ADBD 28 Not Applicable Not Applicable Not Applicable 515 Not Applicable Not Evaluated Scheduled Follow-up Submission Complete Remission/Response Not Evaluated NO 8 Not Available NO Not Available TUMOR FREE NO Complete Remission/Response Not Available NO Alive 2013 FEMALE Lung Adenocarcinoma- Not Otherwise Specified (NOS) No C34.1 8140/3 C34.1 YES Unknown Unknown Not Available Not Available Unknown 7 Not Available Not Available Not Available Not Available Not Available Not Available Not Available Not Available Not Available NO Not Available Yes, History of Prior Malignancy 8204 Unknown TUMOR FREE Not Available Not Available Unknown 88 85 Unknown YES WHITE Unknown R0 Not Applicable Not Applicable Not Applicable Not Applicable Not Applicable Not Applicable Not Applicable Not Applicable Not Applicable Stage IB Not Applicable Not Applicable Not Applicable 7th Not Applicable Not Applicable Not Applicable MX N0 T2a Not Available YES NO 55 3 Lung Alive 2012 2011 Not Available

TCGA-55-8085 904 Alive T1b N0 M0 Stage IA 64 L-Lower Not Applicable TCGA-55-8085 5fd0f07b-8bb9-4378-bd26-28a26057e8fe 15 -23596 Not Applicable 0 33 Not Available Lung Adenocarcinoma 51 1 Not Available Unknown Not Available Not Available Not Available Unknown Not Available NOT HISPANIC OR LATINO Not Available Not Available Not Available Not Available TCGA-55-8085-F65697 CA62DF16-FCE4-47BE-8B87-6AC56FCECE60 26 Not Applicable Not Applicable Not Applicable 904 Not Applicable Not Available Scheduled Follow-up Submission Complete Remission/Response Not Available NO 9 Not Available NO Not Available TUMOR FREE NO Complete Remission/Response Not Available NO Alive 2014 MALE Lung Papillary Adenocarcinoma No C34.3 8260/3 C34.3 YES Not Available Unknown Not Available Not Available Not Available 6 Not Available Not Available Not Available Not Available Not Available Not Available Not Available Not Available Not Available NO Not Available No 8085 Not Available TUMOR FREE 46 45 Unknown 39 35 Complete Remission/Response YES WHITE Unknown R0 Not Applicable Not Applicable Not Applicable Not Applicable Not Applicable Not Applicable Not Applicable Not Applicable Not Applicable Stage IA Not Applicable Not Applicable Not Applicable 7th Not Applicable Not Applicable Not Applicable M0 N0 T1b Not Available YES NO 55 2 Lung Alive 2012 2011 Not Available

TCGA-44-7670 882 Alive T1b N1 M0 Stage IIA 47 L-Upper Not Applicable TCGA-44-7670 9b38eded-3f46-4aaa-9991-68008d97bdbe 18 -17392 Not Applicable 0 531 Not Available Lung Adenocarcinoma 50 TCGA-44-7670-D20715;TCGA-44-7670-D20716 cd9d0292-629c-4b64-976d-92921bc73acf;98b04e0c-0fb1-4f87-97fc-97aed384b749 Not Available;Not Available 19;19 165;165 88;88 Docetaxel;Cisplatin Not Available;Not Available 1;1 4;4 100-120;98-125 mg;mg ADJUVANT;ADJUVANT Not Applicable;Not Applicable 1;1 IV;IV NO;NO Chemotherapy;Chemotherapy Not Available;Not Available 420;419 mg;mg Not Available;Not Available 2012;2012 Not Available Not Available Not Available Not Available Not Available Not Available Not Available Not Available HISPANIC OR LATINO Not Available;Not Available Not Available;Not Available Not Available;NO Not Available;Not Available TCGA-44-7670-F20714;TCGA-44-7670-F39052 58360ec4-71c7-4e83-bec3-c8948c4dcff4;AC8BF10A-8F00-4860-B41E-5D9CCAA18829 19;9 Not Applicable;Not Applicable Not Applicable;Not Applicable Not Applicable;Not Applicable 531;882 Not Applicable;Not Applicable Not Available;Not Available Scheduled Follow-up Submission;Scheduled Follow-up Submission Complete Remission/Response;Complete Remission/Response Not Available;Not Available Not Available;NO 1;1 Not Available;Not Available NO;NO Not Available;Not Available TUMOR FREE;TUMOR FREE YES;YES Complete Remission/Response;Complete Remission/Response Not Available;Not Available NO;NO Alive;Alive 2012;2013 FEMALE Lung Adenocarcinoma- Not Otherwise Specified (NOS) No C34.1 8140/3 C34.1 YES Not Available Not Available Not Available Not Available Not Available 1 Not Available Not Available Not Available Not Available Not Available Not Available Not Available Not Available Not Available Not Available 30 Yes 7670 Not Available TUMOR FREE 90 82 Not Available 86 80 Not Available YES WHITE Not Available Not Available Not Applicable Not Applicable Not Applicable Not Applicable Not Applicable Not Applicable Not Applicable Not Applicable Not Applicable Stage IIA Not Applicable Not Applicable Not Applicable 7th Not Applicable Not Applicable Not Applicable M0 N1 T1b Not Available YES NO 44 2 Lung Alive 2012 2010 1980

TCGA-91-6849 35 Alive T2 N2 MX Stage IIIA 75 L-Upper Not Applicable TCGA-91-6849 953a908d-0993-42f6-853b-512611d19a2c 20 -27676 Not Applicable 0 35 Not Available Lung Adenocarcinoma 66 Not Available Not Available YES Not Available Not Available Not Available Not Available Not Available NOT HISPANIC OR LATINO Not Available Not Available Not Available Not Available TCGA-91-6849-F32642 7A852ADD-9BFF-47D8-8ACB-A7FAAB892A7C 5 Not Available Not Available Not Applicable 35 Not Available Unknown Scheduled Follow-up Submission Unknown Unknown YES 6 Not Available Unknown Not Available Unknown Unknown Unknown Not Available Unknown Alive 2012 FEMALE Lung Adenocarcinoma- Not Otherwise Specified (NOS) No C34.1 8140/3 C34.1 YES 90 NO Not Available Not Available Not Available 7 Not Available Not Available Not Available Not Available Not Available Not Available Not Available Not Available Not Available Not Available 30 No 6849 Preoperative Not Available 75 86 Not Available 76 88 Not Available YES BLACK OR AFRICAN AMERICAN Not Available Not Available Not Applicable Not Applicable Not Applicable Not Applicable Not Applicable Not Applicable Not Applicable Not Applicable Not Applicable Stage IIIA Not Applicable Not Applicable Not Applicable 6th Not Applicable Not Applicable Not Applicable MX N2 T2 1976 NO YES 91 3 Lung Alive 2011 2009 1946

TCGA-55-8301 534 Alive T2a N0 MX Stage IB 58 R-Lower Not Applicable TCGA-55-8301 b36b7567-30d3-4d0c-84c4-b203986e0fdb 3 -21535 Not Applicable 0 44 Not Available Lung Adenocarcinoma Not Available TCGA-55-8301-D56263;TCGA-55-8301-D56264 81EAC4EA-9876-4E67-B2D2-230762F91928;D6B06699-89FF-4995-95D5-D7B9B1095651 Not Available;Not Available 30;30 Not Available;Not Available 261;261 Carboplatin;Taxol Partial Response;Partial Response 1;1 Not Available;Not Available Not Available;Not Available Not Available;Not Available Not Available;Not Available Not Applicable;Not Applicable Not Available;Not Available Not Available;Not Available NO;NO Chemotherapy;Chemotherapy Not Available;Not Available Not Available;Not Available Not Available;Not Available NO;NO 2014;2014 Unknown Not Available Not Available Not Available Not Available Not Available Not Available Not Available NOT HISPANIC OR LATINO YES YES NO NO TCGA-55-8301-F56261 F961BE96-B9C1-48C0-9CC2-B368B6495583 30 Not Available Not Available Not Applicable 534 238 Unknown Scheduled Follow-up Submission Partial Remission/Response Unknown NO 1 Locoregional Recurrence YES Not Available WITH TUMOR NO Complete Remission/Response Convincing Imaging NO Alive 2014 MALE Lung Adenocarcinoma- Not Otherwise Specified (NOS) No C34.3 8140/3 C34.3 YES Unknown Not Available Not Available Not Available Unknown 10 Not Available Not Available Not Available Not Available Not Available Not Available Not Available Not Available Not Available NO 100 No 8301 Unknown TUMOR FREE Not Available Not Available NO Not Available Not Available Complete Remission/Response Not Available WHITE NO Regional site TCGA-55-8301-R56262 F51AFBFF-1D27-42D6-A936-C1FFA21A4A43 Not Available 30 309 257 Partial Response 1 30 6660 NO External Not Applicable Not Available Not Available cGy 2014 R0 Not Applicable Not Applicable Not Applicable Not Applicable Not Applicable Not Applicable Not Applicable Not Applicable Not Applicable Stage IB Not Applicable Not Applicable Not Applicable 7th Not Applicable Not Applicable Not Applicable MX N0 T2a Not Available YES NO 55 2 Lung Alive 2012 2012 1962

TCGA-MP-A4TA 950 Dead T1 N0 M0 Stage IA 75 R-Upper Not Applicable TCGA-MP-A4TA 98EE294A-46ED-4572-BDD4-A932C3498A25 2 -27513 950 0 Not Available Not Available Lung Adenocarcinoma 46 1 Not Available NO Not Available Not Available Not Available NO Not Available NOT HISPANIC OR LATINO FEMALE Lung Adenocarcinoma- Not Otherwise Specified (NOS) No C34.1 8140/3 C34.1 YES Not Evaluated NO Not Available Not Available Peripheral Lung 4 YES YES 725 Not Available Not Available NO Not Available Distant Metastasis Biopsy with Histologic Confirmation YES 55 Yes, History of Prior Malignancy A4TA Pre-Adjuvant Therapy WITH TUMOR Not Available 74 NO Not Available 65 Complete Remission/Response YES WHITE NO R0 Not Applicable Not Applicable Not Applicable Not Applicable Not Applicable Not Applicable Not Applicable Not Applicable Not Applicable Stage IA Not Applicable Not Applicable Not Applicable 6th Not Applicable Not Applicable Not Applicable M0 N0 T1 2006 NO YES MP 4 Lung Dead 2013 2006 1951

TCGA-91-8496 505 Alive T2a NX MX Stage IB 63 L-Lower Not Applicable TCGA-91-8496 656a5eb4-e4a5-4d21-a800-3586f4d6588b 23 -23358 Not Applicable 0 197 Not Available Lung Adenocarcinoma Not Available 0 Not Available NO Not Available Not Available Not Available NO Not Available NOT HISPANIC OR LATINO Not Available Not Available Not Available Not Available TCGA-91-8496-F45467 7004F428-D84D-4816-B359-2D998F95C44B 15 Not Applicable Not Applicable Not Applicable 505 Not Applicable Not Evaluated Scheduled Follow-up Submission Complete Remission/Response Not Evaluated NO 7 Not Available NO Not Available TUMOR FREE NO Complete Remission/Response Not Available NO Alive 2013 FEMALE Lung Bronchioloalveolar Carcinoma Nonmucinous No C34.3 8252/3 C34.3 YES Not Evaluated NO Not Available Not Available Unknown 8 Not Available Not Available Not Available Not Available Not Available Not Available Not Available Not Available Not Available NO Not Available No 8496 Preoperative Unknown Not Available Not Available NO Not Available Not Available Unknown NO WHITE NO Not Evaluated Not Applicable Not Applicable Not Applicable Not Applicable Not Applicable Not Applicable Not Applicable Not Applicable Not Applicable Stage IB Not Applicable Not Applicable Not Applicable 7th Not Applicable Not Applicable Not Applicable MX NX T2a Not Available YES NO 91 1 Lung Alive 2012 2011 Not Available

TCGA-44-3396 1130 Alive T2 N2 M0 Stage IIIA 74 Discrepancy Not Available TCGA-44-3396 3bd6badb-27ff-4d8d-b206-4d28dc264862 18 -27073 Not Applicable 0 311 Not Available Lung Adenocarcinoma 48 TCGA-44-3396-D5017;TCGA-44-3396-D5019 cb7c2370-e79f-4706-9c22-02b0152ffc04;d9d59d53-accc-4e89-bc4c-c13fde7550f1 Not Available;Not Available 22;22 192;192 129;129 Alimta;Carboplatin Not Available;Not Available 10;10 4;4 850;600 mg;mg ADJUVANT;ADJUVANT Not Applicable;Not Applicable 1;1 IV;IV NO;NO Chemotherapy;Chemotherapy Not Available;Not Available 3400;2100 mg;mg Not Available;Not Available 2010;2010 1 Not Available Not Available Not Available Not Available Not Available Not Available Not Available NOT HISPANIC OR LATINO Not Available;Not Available Not Available;Not Available Not Available;Not Available Not Available;Not Available TCGA-44-3396-F5016;TCGA-44-3396-F39080 5e3024c8-c8ce-42c6-bfec-8fbe87e3146a;65BCFB65-301A-471C-9683-5088DE18ED24 22;11 Not Applicable;Not Applicable Not Applicable;Not Applicable Not Applicable;Not Applicable 411;1130 Not Applicable;Not Applicable 1;Not Available Not Available;Scheduled Follow-up Submission Complete Remission/Response;Complete Remission/Response Not Available;80 Not Available;NO 10;1 Not Available;Not Available NO;NO Adjuvant therapy;Other TUMOR FREE;TUMOR FREE YES;YES Complete Remission/Response;Complete Remission/Response Not Available;Not Available NO;NO Alive;Alive 2010;2013 FEMALE Lung Adenocarcinoma- Not Otherwise Specified (NOS) No C34.1 8140/3 C34.1 YES Not Available NO Not Available Not Available Not Available 10 Not Available Not Available Not Available Not Available Not Available Not Available Not Available Not Available Not Available Not Available 50 No 3396 Other TUMOR FREE Not Available Not Available Not Available 98 89 Not Available YES WHITE Not Available R0 Not Applicable Not Applicable Not Applicable Not Applicable Not Applicable Not Applicable Not Applicable Not Applicable Not Applicable Stage IIIA Not Applicable Not Applicable Not Applicable 7th Not Applicable Not Applicable Not Applicable M0 N2 T2 Not Available YES NO 44 2 Lung Alive 2010 2009 1959

TCGA-75-6203 T2 N2 M0 Stage IIIA Not Available L-Lower Not Applicable TCGA-75-6203 a8d6694c-a213-4544-ac0b-63bce16d8f4e 15 Not Available Not Applicable Not Available Not Available Not Available Lung Adenocarcinoma Not Available TCGA-75-6203-D15865;TCGA-75-6203-D15864;TCGA-75-6203-D15863 a50cc582-dab3-4a3c-b8e7-b5cf188baf9a;51cbf34c-bb80-4826-8d78-451bc17e2701;ebfe876c-5580-4fbe-9edb-8749e6f6a5bd Not Available;Not Available;Not Available 17;15;15 Not Available;Not Available;Not Available Not Available;Not Available;Not Available Not Available;Cisplatin;Navelbine Not Available;Not Available;Not Available 8;8;8 Not Available;04;8 Not Available;Not Available;Not Available Not Available;Not Available;Not Available Not Available;ADJUVANT;ADJUVANT Not Applicable;Not Applicable;Not Applicable 2;1;1 Not Available;IV;IV YES;NO;NO Not Available;Chemotherapy;Chemotherapy Not Available;Not Available;Not Available Not Available;Not Available;Not Available Not Available;Not Available;Not Available Not Available;Not Available;Not Available 2010;2011;2011 Not Available Not Available Not Available Not Available Not Available Not Available Not Available Not Available Not Available Not Available Not Available Not Available Not Available TCGA-75-6203-F15862 54091978-b5ea-4fa4-b94e-a707f5389de7 15 Not Available Not Available Not Applicable Not Available Not Available Not Available Not Available Complete Remission/Response Not Available Not Available 8 Not Available Not Available Not Available Not Available YES Complete Remission/Response Not Available NO Alive 2011 FEMALE Lung Adenocarcinoma- Not Otherwise Specified (NOS) No C34.3 8140/3 C34.3 YES Not Available Not Available Not Available Not Available Not Available 8 Not Available Not Available Not Available Not Available Not Available Not Available Not Available Not Available Not Available Not Available Not Available No 6203 Not Available TUMOR FREE Not Available Not Available Not Available Not Available Not Available Not Available Not Available Not Available Not Available R0 Not Applicable Not Applicable Not Applicable Not Applicable Not Applicable Not Applicable Not Applicable Not Applicable Not Applicable Stage IIIA Not Applicable Not Applicable Not Applicable 6th Not Applicable Not Applicable Not Applicable M0 N2 T2 Not Available NO YES 75 1 Lung Alive 2011 2008 Not Available

TCGA-55-7903 567 Alive T1b N0 MX Stage IA 64 R-Lower Not Applicable TCGA-55-7903 77c4dbb2-eceb-4e0d-bcde-63dc817d5f35 10 -23509 Not Applicable 0 19 Not Available Lung Adenocarcinoma 83 Not Available Not Available NO Not Available Not Available Not Available NO Not Available NOT HISPANIC OR LATINO Not Available Not Available Not Available Not Available TCGA-55-7903-F47840 A3E1AF4A-8377-4442-A98B-0F3218B1E228 29 Not Applicable Not Applicable Not Applicable 567 Not Applicable Not Evaluated Scheduled Follow-up Submission Complete Remission/Response Not Evaluated NO 8 Not Available NO Not Evaluated TUMOR FREE NO Complete Remission/Response Not Available NO Alive 2013 MALE Lung Adenocarcinoma- Not Otherwise Specified (NOS) No C34.3 8140/3 C34.3 YES Not Available NO Not Available Not Available Not Available 3 Not Available Not Available Not Available Not Available Not Available Not Available Not Available Not Available Not Available Not Available 40 No 7903 Not Available TUMOR FREE Not Available Not Available Not Available 63 66 Not Available YES WHITE Not Available R0 Not Applicable Not Applicable Not Applicable Not Applicable Not Applicable Not Applicable Not Applicable Not Applicable Not Applicable Stage IA Not Applicable Not Applicable Not Applicable 7th Not Applicable Not Applicable Not Applicable MX N0 T1b Not Available YES NO 55 2 Lung Alive 2012 2011 Not Available

TCGA-55-7281 872 Alive T1b N0 M0 Stage IA 70 R-Middle Not Applicable TCGA-55-7281 659668e8-f0d9-4ff2-bbc8-9246f2ef49ab 6 -25870 Not Applicable 0 18 Not Available Lung Adenocarcinoma Not Available TCGA-55-7281-D47811;TCGA-55-7281-D47814;TCGA-55-7281-D47815;TCGA-55-7281-D47816;TCGA-55-7281-D47817;TCGA-55-7281-D47818 F65BFFAD-A23B-4066-829C-5D92F15865DE;B212E1D8-57BE-4365-BFBA-1EA76A701912;DC5037B4-AFDE-459F-8830-340492F0AA06;638427FC-0F5A-4F6A-8E41-E90771270BA7;5046F93F-1D01-4A67-9B87-ED115A6C742A;F582DDC9-3FBE-4AC6-870A-530C86640E54 Not Available;Not Available;Not Available;Not Available;Not Available;Not Available 28;28;28;28;28;28 474;579;579;Not Available;Not Available;Not Available 424;508;508;712;712;712 Carboplatin;Carboplatin;Alimta;Carboplatin;Taxol;Avastin Clinical Progressive Disease;Clinical Progressive Disease;Clinical Progressive Disease;Partial Response;Partial Response;Partial Response 8;8;8;8;8;8 Not Available;Not Available;Not Available;Not Available;Not Available;Not Available Not Available;Not Available;Not Available;Not Available;Not Available;Not Available Not Available;Not Available;Not Available;Not Available;Not Available;Not Available Not Available;Not Available;Not Available;Not Available;Not Available;Not Available Not Applicable;Not Applicable;Not Applicable;Not Applicable;Not Applicable;Not Applicable Not Available;Not Available;Not Available;Not Available;Not Available;Not Available Not Available;Not Available;Not Available;Not Available;Not Available;Not Available NO;NO;NO;NO;NO;NO Chemotherapy;Chemotherapy;Chemotherapy;Chemotherapy;Chemotherapy;Chemotherapy Not Available;Not Available;Not Available;Not Available;Not Available;Not Available Not Available;Not Available;Not Available;Not Available;Not Available;Not Available Not Available;Not Available;Not Available;Not Available;Not Available;Not Available NO;NO;NO;NO;NO;NO 2013;2013;2013;2013;2013;2013 Not Available Not Available Not Available Not Available Not Available Not Available Not Available Not Available Not Available YES YES NO NO TCGA-55-7281-F47807 0804D88A-04B2-4C56-9A68-68D39DDB1617 28 Not Available Not Available Not Applicable 872 339 Not Evaluated Scheduled Follow-up Submission Partial Remission/Response Not Evaluated NO 8 Distant Metastasis YES Not Evaluated WITH TUMOR NO Complete Remission/Response Convincing Imaging NO Alive 2013 FEMALE Lung Adenocarcinoma- Not Otherwise Specified (NOS) No C34.2 8140/3 C34.2 YES Not Available NO Not Available Not Available Not Available 10 Not Available Not Available Not Available Not Available Not Available Not Available Not Available Not Available Not Available Not Available 20 No 7281 Not Available TUMOR FREE Not Available Not Available Not Available Not Available Not Available Not Available Not Available WHITE Not Available Regional site;Distant site TCGA-55-7281-R47808;TCGA-55-7281-R47810 F3791E9B-5039-47A4-8E77-A47AF6CD8BE4;722364F3-700A-4E78-8A04-C6D4C2353780 Not Available;Not Available 28;28 370;647 339;634 Radiographic Progressive Disease;Radiographic Progressive Disease 8;8 Not Available;10 Not Available;03000 NO;NO External;External Not Applicable;Not Applicable Not Available;Not Available Not Available;Not Available Not Available;cGy 2013;2013 R0 Not Applicable Not Applicable Not Applicable Not Applicable Not Applicable Not Applicable Not Applicable Not Applicable Not Applicable Stage IA Not Applicable Not Applicable Not Applicable 7th Not Applicable Not Applicable Not Applicable M0 N0 T1b Not Available YES NO 55 4 Lung Alive 2011 2011 Not Available

TCGA-55-6979 237 Dead T2 N1 M0 Stage IIB 59 L-Upper Not Applicable TCGA-55-6979 5af499be-d2b9-4eaf-9a9f-435dccb51917 26 -21780 237 0 Not Available Not Available Lung Adenocarcinoma Not Available TCGA-55-6979-D40547;TCGA-55-6979-D40548 F521F7CC-4443-4315-B2C2-B486B17D0DC5;7F10EFEE-013A-4927-8BAD-1683AED5FA99 Not Available;Not Available 22;22 149;149 57;57 Carboplatin;Taxol Clinical Progressive Disease;Clinical Progressive Disease 2;2 Not Available;Not Available Not Available;Not Available Not Available;Not Available Not Available;Not Available Not Applicable;Not Applicable Not Available;Not Available Not Available;Not Available NO;NO Chemotherapy;Chemotherapy Not Available;Not Available Not Available;Not Available Not Available;Not Available NO;NO 2013;2013 Not Available Not Available NO Not Available Not Available Not Available NO Not Available Not Available YES YES Not Available NO TCGA-55-6979-F37057 7DAF16E2-34A6-40ED-99FB-8C3F3205DA98 22 Not Available Not Available 237 Not Available 195 Unknown Scheduled Follow-up Submission Progressive Disease Unknown NO 2 Distant Metastasis YES Not Available WITH TUMOR YES Progressive Disease Convincing Imaging NO Dead 2013 FEMALE Lung Adenocarcinoma- Not Otherwise Specified (NOS) No C34.1 8140/3 C34.1 YES Not Available NO Not Available Not Available Not Available 7 Not Available Not Available Not Available Not Available Not Available Not Available Not Available Not Available Not Available Not Available Not Available Yes 6979 Not Available WITH TUMOR Not Available Not Available Not Available Not Available Not Available Not Available Not Available WHITE Not Available Not Available Not Applicable Not Applicable Not Applicable Not Applicable Not Applicable Not Applicable Not Applicable Not Applicable Not Applicable Stage IIB Not Applicable Not Applicable Not Applicable 6th Not Applicable Not Applicable Not Applicable M0 N1 T2 Not Available NO YES 55 3 Lung Dead 2011 2006 Not Available

TCGA-97-8176 468 Dead T3 N1 M0 Stage IIIA 63 R-Lower Not Applicable TCGA-97-8176 12c27db5-db2f-48dc-a2ca-9557b951f43e 5 -23170 Not Applicable 0 252 Not Available Lung Adenocarcinoma 61 TCGA-97-8176-D35660;TCGA-97-8176-D35661;TCGA-97-8176-D35662;TCGA-97-8176-D49551;TCGA-97-8176-D49550 3CDB750D-9A1C-4C74-B647-D988440014AE;C2AF0586-F52E-443A-9120-934B54628579;49597D40-3617-493C-8061-8DCCB9871B6B;51434296-C639-41F5-82B1-E61B82E05160;6B643397-9B35-4517-A6EC-013C81A73723 Not Available;Not Available;Not Available;Not Available;Not Available 5;5;5;3;3 161;417;417;417;417 77;77;77;77;77 CARBOplatin;ALIMTA;B12;Neulasta;Xgeva Partial Response;Partial Response;Partial Response;Unknown;Unknown 10;10;10;10;10 Not Available;Not Available;Not Available;Not Available;Not Available Not Available;Not Available;Not Available;Not Available;Not Available Not Available;Not Available;Not Available;Not Available;Not Available Not Available;Not Available;Not Available;Not Available;Not Available Not Applicable;Not Applicable;Not Applicable;Not Applicable;Not Applicable Not Available;Not Available;Not Available;Not Available;Not Available Not Available;Not Available;Not Available;Not Available;Not Available NO;NO;NO;NO;NO Chemotherapy;Chemotherapy;Ancillary;Ancillary;Ancillary Not Available;Not Available;Not Available;Not Available;Not Available Not Available;Not Available;Not Available;Not Available;Not Available Not Available;Not Available;Not Available;Not Available;Not Available NO;NO;NO;NO;NO 2012;2012;2012;2013;2013 0 Not Available YES Not Available Not Available Not Available NO Not Available NOT HISPANIC OR LATINO Not Available Not Available Not Available Not Available TCGA-97-8176-F49542 F462990E-8C16-40DE-BCD8-D98F16F60DA6 11 Not Applicable Not Applicable 468 Not Available Not Applicable Unknown Scheduled Follow-up Submission Progressive Disease Not Available NO 10 Not Available NO Unknown Unknown YES Stable Disease Not Available YES Dead 2013 MALE Lung Acinar Adenocarcinoma No C34.3 8550/3 C34.3 YES Not Available YES YES G12S Not Available 10 YES YES 39 Not Available Not Available YES 171 Distant Metastasis Biopsy with Histologic Confirmation YES 40 No 8176 Preoperative TUMOR FREE 68 104 YES 67 100 Unknown YES WHITE YES Distant Recurrence;Distant Recurrence;Distant Recurrence TCGA-97-8176-R35601;TCGA-97-8176-R35659;TCGA-97-8176-R49544 B10D270F-44DE-43D1-A82E-FB7D967C274B;411DC29A-8C31-4E66-8FF5-0A8675FA8495;B9986975-6FF6-4AC9-B988-2EA72265F180 Not Available;Not Available;Not Available 5;5;11 105;105;64 91;91;51 Partial Response;Partial Response;Partial Response 10;10;3 5;10;5 1500;3000;3000 NO;NO;NO External;External;External Not Applicable;Not Applicable;Not Applicable Not Available;Not Available;Not Available Not Available;Not Available;Not Available cGy;cGy;cGy 2012;2012;2014 R0 Not Applicable Not Applicable Not Applicable Not Applicable Not Applicable Not Applicable Not Applicable Not Applicable Not Applicable Stage IIIA Not Applicable Not Applicable Not Applicable 7th Not Applicable Not Applicable Not Applicable M0 N1 T3 2011 YES NO 97 4 Lung Alive 2012 2012 1971

TCGA-86-8074 24 Alive T1b N1 M0 Stage IIA 62 L-Lower Not Applicable TCGA-86-8074 482eb2a7-6fed-4e4c-b1b6-14d6d869f855 9 -22862 Not Applicable 0 24 Not Available Lung Adenocarcinoma Not Available 2 Not Available NO Not Available Not Available Not Available NO Not Available NOT HISPANIC OR LATINO Not Available Not Available Not Available Not Available TCGA-86-8074-F41667 69D69C1B-6691-4BAA-A3F5-3E34CBD24E19 26 Not Available Not Available Not Applicable Not Available Not Available Not Available Scheduled Follow-up Submission Not Available Not Available YES 3 Not Available Not Available Not Available Not Available Not Available Not Available Not Available Not Available Not Available 2013 FEMALE Lung Adenocarcinoma- Not Otherwise Specified (NOS) No C34.3 8260/3 C34.3 YES 80 NO Not Available Not Available Peripheral Lung 5 Not Available Not Available Not Available Not Available Not Available Not Available Not Available Not Available Not Available Unknown 40 No 8074 Not Available TUMOR FREE Not Available Not Available Unknown Not Available Not Available Unknown NO WHITE Unknown R0 Not Applicable Not Applicable Not Applicable Not Applicable Not Applicable Not Applicable Not Applicable Not Applicable Not Applicable Stage IIA Not Applicable Not Applicable Not Applicable 7th Not Applicable Not Applicable Not Applicable M0 N1 T1b Not Available YES NO 86 2 Lung Alive 2012 2011 1970

TCGA-86-8673 862 Alive T2 N0 M0 Stage IB 61 L-Upper Not Applicable TCGA-86-8673 bc4c4079-b449-485d-84e4-a40496e563e8 30 -22571 Not Applicable 0 0 Not Available Lung Adenocarcinoma Not Available Unknown Not Available Unknown Not Available Not Available Not Available Unknown Not Available NOT HISPANIC OR LATINO Not Available;NO Not Available;NO Not Available;YES Not Available;Not Available TCGA-86-8673-F41842;TCGA-86-8673-F59092 4D099A3E-8646-424C-AEF4-394D2DDC653A;7D5C34E4-E4BC-41BC-9704-35161E33F1E6 1;30 Not Applicable;639 Not Applicable;Not Available Not Applicable;Not Applicable 455;862 Not Applicable;636 Not Evaluated;Not Evaluated Scheduled Follow-up Submission;Scheduled Follow-up Submission Complete Remission/Response;Complete Remission/Response Not Evaluated;Not Evaluated NO;NO 4;4 Not Available;Locoregional Recurrence NO;YES Not Evaluated;Not Evaluated TUMOR FREE;TUMOR FREE NO;NO Complete Remission/Response;Complete Remission/Response Not Available;Not Available NO;NO Alive;Alive 2013;2014 MALE Lung Bronchioloalveolar Carcinoma Nonmucinous No C34.1 8252/3 C34.1 YES Unknown Unknown Not Available Not Available Unknown 8 Not Available Not Available Not Available Not Available Not Available Not Available Not Available Not Available Not Available Unknown 54 No 8673 Not Available TUMOR FREE Not Available Not Available Unknown Not Available Not Available Not Available NO WHITE Unknown R0 Not Applicable Not Applicable Not Applicable Not Applicable Not Applicable Not Applicable Not Applicable Not Applicable Not Applicable Stage IB Not Applicable Not Applicable Not Applicable 7th Not Applicable Not Applicable Not Applicable M0 N0 T2 Not Available YES NO 86 2 Lung Alive 2012 2011 1968

TCGA-44-2656 1429 Alive T2 N0 M0 Stage IB FPPP TCGA 59 R-Upper Not Applicable TCGA-44-2656 42ca54fc-c1ae-41cd-bca1-7fe9810db460 8 -21766 Not Applicable 0 582 Not Available Lung Adenocarcinoma 72 0 Not Available Not Available Not Available Not Available Not Available Not Available Not Available NOT HISPANIC OR LATINO Not Available;Not Available;YES Not Available;Not Available;YES Not Available;Not Available;Not Available Not Available;Not Available;Not Available TCGA-44-2656-F5294;TCGA-44-2656-F9736;TCGA-44-2656-F39934 ca338d6c-2526-45f1-9b0d-c0a045d0ac8c;f823f92c-9e8e-4393-9381-62cfc40ff094;01F40F24-6888-4940-8620-3E5F531523DD 8;14;6 Not Applicable;Not Available;Not Available Not Applicable;Not Available;Not Available Not Applicable;Not Applicable;Not Applicable 582;749;1429 Not Applicable;Not Available;568 Not Available;Not Available;Not Available Not Available;Not Available;Scheduled Follow-up Submission Complete Remission/Response;Not Available;Complete Remission/Response Not Available;Not Available;Not Available Not Available;Not Available;NO 10;4;2 Not Available;Not Available;New Primary Tumor NO;Not Available;YES Not Available;Not Available;Not Available TUMOR FREE;TUMOR FREE;TUMOR FREE NO;NO;NO Complete Remission/Response;Complete Remission/Response;Complete Remission/Response Not Available;Not Available;Biopsy with Histologic Confirmation NO;NO;NO Alive;Alive;Alive 2010;2011;2013 MALE Lung Adenocarcinoma- Not Otherwise Specified (NOS) No C34.1 8140/3 C34.1 YES Not Available NO Not Available Not Available Not Available 10 Not Available Not Available Not Available Not Available Not Available Not Available Not Available Not Available Not Available Not Available 23 No 2656 Pre-Adjuvant Therapy TUMOR FREE 86 84 Not Available 86 79 Not Available YES WHITE Not Available Not Available Not Applicable Not Applicable Not Applicable Not Applicable Not Applicable Not Applicable Not Applicable Not Applicable Not Applicable Stage IB Not Applicable Not Applicable Not Applicable 6th Not Applicable Not Applicable Not Applicable M0 N0 T2 2009 YES NO 44 4 Lung Alive 2010 2009 1994

TCGA-75-7030 T3 N0 M0 Stage IIB Not Available R-Lower Not Applicable TCGA-75-7030 df576520-a6b6-4c9b-8d06-3f59cc5342fd 16 Not Available Not Applicable Not Available Not Available Not Available Lung Adenocarcinoma Not Available TCGA-75-7030-D18798;TCGA-75-7030-D18797 0c13d354-be1b-4d41-a8d4-1579f902bf38;e83f6d9d-f80b-46df-948c-10ad8c4706a2 Not Available;Not Available 17;17 Not Available;Not Available Not Available;Not Available Cisplatin;Vinorelbine Tartrate Not Available;Not Available 11;11 4;4 92;46 mg/day;mg/day ADJUVANT;ADJUVANT Not Applicable;Not Applicable Not Available;Not Available IV;IV YES;NO Chemotherapy;Chemotherapy Not Available;Not Available 736;552 mg;mg Not Available;Not Available 2011;2011 0 Not Available Not Available Not Available Not Available Not Available Not Available Not Available Not Available NO NO NO NO TCGA-75-7030-F15878 63eba997-c2ac-44fa-8c2b-d620644e2410 16 Not Applicable Not Applicable Not Applicable Not Available Not Applicable 0 Not Available Complete Remission/Response Not Available Not Available 8 Not Available NO Post-Adjuvant Therapy Not Available YES Complete Remission/Response Not Available NO Alive 2011 MALE Lung Adenocarcinoma- Not Otherwise Specified (NOS) No C34.3 8140/3 C34.3 YES Not Available Not Available Not Available Not Available Peripheral Lung 8 Not Available Not Available Not Available Not Available Not Available Not Available Not Available Not Available Not Available Not Available Not Available No 7030 Preoperative TUMOR FREE Not Available Not Available Not Available Not Available Not Available Not Available Not Available Not Available Not Available R0 Not Applicable Not Applicable Not Applicable Not Applicable Not Applicable Not Applicable Not Applicable Not Applicable Not Applicable Stage IIB Not Applicable Not Applicable Not Applicable 6th Not Applicable Not Applicable Not Applicable M0 N0 T3 Not Available NO YES 75 1 Lung Alive 2011 2007 Not Available

TCGA-05-4390 1126 Alive T2 N0 M0 Stage IB 58 R-Upper Not Applicable TCGA-05-4390 b8475929-2d9d-4909-bd62-59684a140bd7 22 -21430 Not Applicable 0 1126 Not Available Lung Adenocarcinoma Not Available TCGA-05-4390-D36457;TCGA-05-4390-D36458 1C563458-F78C-43FA-9FD0-9F31F386B0D7;E9C5A69B-2C5F-40D1-9CE9-46F808524536 Not Available;Not Available 5;5 92;92 61;61 Cisplatin;Vinorelbine Complete Response;Complete Response 12;12 Not Available;Not Available Not Available;Not Available Not Available;Not Available Not Available;Not Available Not Applicable;Not Applicable Not Available;Not Available Not Available;Not Available NO;NO Chemotherapy;Chemotherapy Not Available;Not Available Not Available;Not Available Not Available;Not Available NO;NO 2012;2012 Not Available Not Available Not Available Not Available Not Available Not Available Not Available Not Available Not Available Unknown Unknown Not Available Not Available TCGA-05-4390-F36456 732D9E3F-5FEB-41B6-BE2A-142253ED6110 31 Not Available Not Available Not Applicable 1126 395 Not Available Scheduled Follow-up Submission Complete Remission/Response Not Available NO 10 Not Available YES Not Available TUMOR FREE YES Progressive Disease Not Available NO Alive 2012 FEMALE Lung Adenocarcinoma Mixed Subtype No C34.1 8255/3 C34.1 YES Not Available Not Available Not Available Not Available Not Available 7 Not Available Not Available Not Available Not Available Not Available Not Available Not Available Not Available Not Available Not Available 15 No 4390 Not Available TUMOR FREE Not Available Not Available Not Available Not Available Not Available Not Available Not Available Not Available Not Available R0 Not Applicable Not Applicable Not Applicable Not Applicable Not Applicable Not Applicable Not Applicable Not Applicable Not Applicable Stage IB Not Applicable Not Applicable Not Applicable 5th Not Applicable Not Applicable Not Applicable M0 N0 T2 1996 NO YES 05 4 Lung Alive 2010 2005 1966

TCGA-78-7540 1197 Dead T2 N0 M0 Stage IB 66 L-Lower Not Applicable TCGA-78-7540 05534064-8b43-4c82-95da-70fd83bd6560 18 -24440 Not Applicable 0 881 Not Available Lung Adenocarcinoma Not Available 0 Not Available NO Not Available Not Available Not Available NO Not Available Not Available Not Available;Not Available Not Available;Not Available Not Available;Not Available Not Available;Not Available TCGA-78-7540-F20484;TCGA-78-7540-F46104 ff5b85d0-1d1f-4e04-865e-c2f897aa5866;AEF11AFE-F0B6-41CA-A7A9-527C1A1BC8F0 19;2 Not Available;Not Available Not Available;Not Available Not Applicable;1197 881;Not Available Not Available;Not Available Not Available;Unknown Scheduled Follow-up Submission;Scheduled Follow-up Submission Not Available;Unknown Not Available;Not Available Not Available;NO 1;8 Not Available;Not Available Not Available;Unknown Not Available;Unknown Not Available;Unknown NO;Unknown Not Available;Complete Remission/Response Not Available;Not Available NO;Unknown Alive;Dead 2012;2013 FEMALE Lung Bronchioloalveolar Carcinoma Mucinous No C34.3 8253/3 C34.3 YES Not Available NO Not Available Not Available Not Available 1 Not Available Not Available Not Available Not Available Not Available Not Available Not Available Not Available Not Available Not Available Not Available No 7540 Preoperative Not Available Not Available Not Available Not Available Not Available Not Available Not Available Not Available WHITE Not Available R0 Not Applicable Not Applicable Not Applicable Not Applicable Not Applicable Not Applicable Not Applicable Not Applicable Not Applicable Stage IB Not Applicable Not Applicable Not Applicable 6th Not Applicable Not Applicable Not Applicable M0 N0 T2 Not Available NO YES 78 1 Lung Alive 2012 1999 Not Available

TCGA-35-5375 264 Alive T2 N2 M0 Stage IIIA 61 L-Upper Not Applicable TCGA-35-5375 5d2a9a4f-678d-4089-a8c5-81a5cb696629 20 -22628 Not Applicable 0 264 Not Available Lung Adenocarcinoma Not Available 1 Not Available Not Available Not Available Not Available Not Available Not Available Not Available NOT HISPANIC OR LATINO Not Available Not Available Not Available Not Available TCGA-35-5375-F68920 8B27426D-4A36-4ED5-82E4-1622898D6E17 24 Not Available Not Available Not Applicable Not Available Not Available Not Available Scheduled Follow-up Submission Not Applicable Not Available YES 8 Not Available Not Available Not Available Not Available Not Available Not Available Not Available Not Available Not Available 2015 MALE Lung Adenocarcinoma- Not Otherwise Specified (NOS) No C34.1 8140/3 C34.1 YES 90 Not Available Not Available Not Available Peripheral Lung 5 Not Available Not Available Not Available Not Available Not Available Not Available Not Available Not Available Not Available Not Available 35 No 5375 Post-Adjuvant Therapy TUMOR FREE Not Available Not Available Not Available Not Available Not Available Not Available Not Available WHITE Not Available R0 Not Applicable Not Applicable Not Applicable Not Applicable Not Applicable Not Applicable Not Applicable Not Applicable Not Applicable Stage IIIA Not Applicable Not Applicable Not Applicable Not Available Not Applicable Not Applicable Not Applicable M0 N2 T2 2006 NO YES 35 4 Lung Alive 2011 2010 1971

TCGA-50-5935 653 Dead T1 N0 M0 Stage IA 86 L-Upper Not Applicable TCGA-50-5935 100430c8-1446-45c8-af36-b6dbb3ddd0c1 2 -31610 653 0 653 Not Available Lung Adenocarcinoma Not Available Not Available Not Available YES Other Not Available Not Available Not Available Not Available NOT HISPANIC OR LATINO Not Available Not Available NO Not Available TCGA-50-5935-F32108 1e2da076-3f69-4de4-af22-9f3dfc1ce1f3 17 Not Applicable Not Applicable 653 Not Available Not Applicable Not Available Scheduled Follow-up Submission Complete Remission/Response Not Available NO 5 Not Available NO Not Available TUMOR FREE NO Complete Remission/Response Not Available YES Dead 2012 FEMALE Lung Adenocarcinoma- Not Otherwise Specified (NOS) No C34.1 8140/3 C34.1 YES Not Available YES YES Not Available Not Available 7 Not Available Not Available Not Available Not Available Not Available Not Available Not Available Not Available Not Available Not Available Not Available Yes 5935 Not Available Not Available Not Available Not Available Not Available Not Available Not Available Not Available Not Available WHITE Not Available Primary Tumor Field TCGA-50-5935-R32109 5aa7172f-ba04-4f8f-8dcc-82cb5f544459 1 17 0 0 Not Available 5 Not Available 100 NO IMPLANTS Not Applicable ADJUVANT Not Available cGy 2012 Not Available Not Applicable Not Applicable Not Applicable Not Applicable Not Applicable Not Applicable Not Applicable Not Applicable Not Applicable Stage IA Not Applicable Not Applicable Not Applicable 6th Not Applicable Not Applicable Not Applicable M0 N0 T1 Not Available NO YES 50 Not Available Lung Dead 2011 2006 Not Available

TCGA-05-4417 455 Alive T2 N0 M0 Stage IB 51 R-Upper Not Applicable TCGA-05-4417 a244a99a-d7cc-4fb1-bc76-b66886481621 22 -18780 Not Applicable 0 455 Not Available Lung Adenocarcinoma Not Available Not Available Not Available Not Available Not Available Not Available Not Available Not Available Not Available Not Available Not Available Not Available Not Available Not Available TCGA-05-4417-F36502 9B56A013-ECA5-4BE4-BC40-1E4BCAAE0501 31 Not Applicable Not Applicable Not Applicable 455 Not Applicable Not Available Scheduled Follow-up Submission Partial Remission/Response Not Available NO 10 Not Available NO Not Available TUMOR FREE NO Partial Remission/Response Not Available NO Alive 2012 FEMALE Lung Adenocarcinoma Mixed Subtype No C34.1 8255/3 C34.1 YES Not Available Not Available Not Available Not Available Not Available 7 Not Available Not Available Not Available Not Available Not Available Not Available Not Available Not Available Not Available Not Available 56 No 4417 Not Available TUMOR FREE Not Available Not Available Not Available Not Available Not Available Not Available Not Available Not Available Not Available R0 Not Applicable Not Applicable Not Applicable Not Applicable Not Applicable Not Applicable Not Applicable Not Applicable Not Applicable Stage IB Not Applicable Not Applicable Not Applicable 6th Not Applicable Not Applicable Not Applicable M0 N0 T2 2008 NO YES 05 4 Lung Alive 2010 2008 1971

TCGA-78-8648 1209 Dead T3 N0 M0 Stage IIB 58 R-Upper Not Applicable TCGA-78-8648 5ace9608-d38a-42f2-a877-ec7c9d211808 23 -21443 1209 0 Not Available Not Available Lung Adenocarcinoma 69 2 Not Available NO Not Available Not Available Not Available NO Not Available Not Evaluated FEMALE Lung Adenocarcinoma- Not Otherwise Specified (NOS) No C34.1 8140/3 C34.1 YES Not Available NO Not Available Not Available Peripheral Lung 10 Unknown Unknown 720 Unknown Not Available Not Available Not Available Locoregional Recurrence Biopsy with Histologic Confirmation YES 45 No 8648 Preoperative Unknown Not Available Not Available NO 83 70 Unknown YES Not Evaluated NO R0 Not Applicable Not Applicable Not Applicable Not Applicable Not Applicable Not Applicable Not Applicable Not Applicable Not Applicable Stage IIB Not Applicable Not Applicable Not Applicable 6th Not Applicable Not Applicable Not Applicable M0 N0 T3 Not Available NO YES 78 2 Lung Dead 2012 2002 1971

TCGA-05-4244 0 Alive T2 N2 M1 Stage IV 70 R-Lower Not Applicable TCGA-05-4244 34040b83-7e8a-4264-a551-b16621843e28 22 -25752 Not Applicable 0 0 Not Available Lung Adenocarcinoma Not Available Not Available Not Available Not Available Not Available Not Available Not Available Not Available Not Available Not Available MALE Lung Adenocarcinoma- Not Otherwise Specified (NOS) No C34.3 8140/3 C34.3 YES Not Available Not Available Not Available Not Available Peripheral Lung 7 Not Available Not Available Not Available Not Available Not Available Not Available Not Available Not Available Not Available Not Available 38 No 4244 Not Available TUMOR FREE Not Available Not Available Not Available Not Available Not Available Not Available Not Available Not Available Not Available RX Not Applicable Not Applicable Not Applicable Not Applicable Not Applicable Not Applicable Not Applicable Not Applicable Not Applicable Stage IV Not Applicable Not Applicable Not Applicable 6th Not Applicable Not Applicable Not Applicable M1 N2 T2 Not Available NO YES 05 4 Lung Alive 2010 2009 Not Available

TCGA-91-6831 310 Alive T2 N0 MX Stage IB 66 R-Middle Not Applicable TCGA-91-6831 db0b9e63-4272-4f29-bf0e-1ec0fe79a9d7 19 -24436 Not Applicable 0 39 Not Available Lung Adenocarcinoma Not Available Not Available Not Available Not Available Not Available Not Available Not Available Not Available Not Available NOT HISPANIC OR LATINO Not Available Not Available Not Available Not Available TCGA-91-6831-F32636 05ae61a2-c054-4273-b50c-4760cbb08a73 5 Not Applicable Not Applicable Not Applicable 310 Not Applicable Unknown Scheduled Follow-up Submission Complete Remission/Response Unknown YES 6 Not Available NO Not Available TUMOR FREE NO Complete Remission/Response Not Available NO Alive 2012 MALE Lung Adenocarcinoma- Not Otherwise Specified (NOS) No C34.2 8140/3 C34.2 YES Not Available NO Not Available Not Available Not Available 7 Not Available Not Available Not Available Not Available Not Available Not Available Not Available Not Available Not Available Not Available Not Available No 6831 Not Available TUMOR FREE Not Available Not Available Not Available Not Available Not Available Not Available NO WHITE Not Available Not Available Not Applicable Not Applicable Not Applicable Not Applicable Not Applicable Not Applicable Not Applicable Not Applicable Not Applicable Stage IB Not Applicable Not Applicable Not Applicable 5th Not Applicable Not Applicable Not Applicable MX N0 T2 Not Available NO YES 91 2 Lung Alive 2011 2001 Not Available

TCGA-S2-AA1A 513 Alive T1b N0 M0 Stage IA 68 R-Upper Not Applicable TCGA-S2-AA1A 31458638-E19C-43E5-AB13-9C64B3B3681D 22 -24903 Not Applicable 0 121 Not Available Lung Adenocarcinoma 65 Not Evaluated Not Available NO Not Available Not Available Not Available NO Not Available NOT HISPANIC OR LATINO Not Available Not Available Not Available Not Available TCGA-S2-AA1A-F67520 90F720D7-868B-4102-9B73-0824B7500158 7 Not Applicable Not Applicable Not Applicable 513 Not Applicable Not Evaluated Scheduled Follow-up Submission Complete Remission/Response Not Evaluated NO 11 Not Available NO Not Available TUMOR FREE NO Complete Remission/Response Not Available NO Alive 2014 FEMALE Lung Bronchioloalveolar Carcinoma Mucinous No C34.1 8253/3 C34.1 YES Not Evaluated NO Not Available Not Available Not Available 4 Not Available Not Available Not Available Not Available Not Available Not Available Not Available Not Available Not Available NO 95 No AA1A Not Available TUMOR FREE 83 2.16 NO 79 2.01 Complete Remission/Response YES BLACK OR AFRICAN AMERICAN NO R0 Not Applicable Not Applicable Not Applicable Not Applicable Not Applicable Not Applicable Not Applicable Not Applicable Not Applicable Stage IA Not Applicable Not Applicable Not Applicable 7th Not Applicable Not Applicable Not Applicable M0 N0 T1b 1995 YES NO S2 3 Lung Alive 2014 2013 1957

TCGA-55-7726 652 Alive T1b N0 MX Stage IA 72 L-Lower Not Applicable TCGA-55-7726 923b37e4-fcf7-46a0-8cb2-9139711fec7b 8 -26451 Not Applicable 0 39 Not Available Lung Adenocarcinoma 72 Not Available Not Available Not Available Not Available Not Available Not Available Not Available Not Available NOT HISPANIC OR LATINO Not Available Not Available Not Available Not Available TCGA-55-7726-F47844 3F1E5E80-6F83-41FA-8999-94F152C1EDCB 29 Not Applicable Not Applicable Not Applicable 652 Not Applicable Not Evaluated Scheduled Follow-up Submission Complete Remission/Response Not Evaluated NO 8 Not Available NO Not Evaluated TUMOR FREE NO Complete Remission/Response Not Available NO Alive 2013 FEMALE Lung Adenocarcinoma- Not Otherwise Specified (NOS) No C34.3 8140/3 C34.3 YES Not Available Not Available Not Available Not Available Not Available 3 Not Available Not Available Not Available Not Available Not Available Not Available Not Available Not Available Not Available Not Available 30 Yes 7726 Not Available TUMOR FREE 59 61 Not Available 55 49 Not Available YES WHITE Not Available R0 Not Applicable Not Applicable Not Applicable Not Applicable Not Applicable Not Applicable Not Applicable Not Applicable Not Applicable Stage IA Not Applicable Not Applicable Not Applicable 7th Not Applicable Not Applicable Not Applicable MX N0 T1b 1979 YES NO 55 3 Lung Alive 2012 2011 1955

TCGA-62-8402 1498 Dead T2 N2 M0 Stage IIIA 73 R-Upper Not Applicable TCGA-62-8402 433a32d0-93d1-4897-8e36-beb1ec1d998e 4 -26934 Not Applicable 0 1445 Not Available Lung Adenocarcinoma Not Available 0 Not Available YES Exon 19 Deletion Not Available Not Available NO Not Available NOT HISPANIC OR LATINO YES YES Not Available NO TCGA-62-8402-F52547 08295B1A-EE42-42DD-9431-741E62382F2E 4 Not Available Not Available 1498 Not Available 772 0 Scheduled Follow-up Submission Progressive Disease 100 NO 12 Distant Metastasis YES Preoperative WITH TUMOR NO Complete Remission/Response Convincing Imaging NO Dead 2013 FEMALE Lung Adenocarcinoma Mixed Subtype No C34.1 8255/3 C34.1 YES 100 NO Not Available Not Available Peripheral Lung 7 YES YES 772 NO Not Available NO Not Available Locoregional Recurrence;Distant Metastasis Convincing Imaging YES Not Available No 8402 Preoperative WITH TUMOR Not Available Not Available Unknown 80 134 Complete Remission/Response YES WHITE YES Regional site TCGA-62-8402-R33812 181E46B6-E825-45DA-A720-020A04CA720F Not Available 19 73 28 Complete Response 7 25 50 NO External Not Applicable Not Available Not Available Gy 2012 R0 Not Applicable Not Applicable Not Applicable Not Applicable Not Applicable Not Applicable Not Applicable Not Applicable Not Applicable Stage IIIA Not Applicable Not Applicable Not Applicable 6th Not Applicable Not Applicable Not Applicable M0 N2 T2 Not Available NO YES 62 1 Lung Alive 2012 2008 Not Available

TCGA-55-6986 3261 Alive T2 N0 M0 Stage IB 74 R-Lower Not Applicable TCGA-55-6986 028e99e9-5b9a-4954-bb6e-6d4709a3cea8 26 Not Available Not Applicable 0 2151 Not Available Lung Adenocarcinoma Not Available Not Available Not Available NO Not Available Not Available Not Available NO Not Available Not Available Not Available Not Available Not Available Not Available TCGA-55-6986-F45606 D147F3D1-27F0-467E-9212-AE14BCF08700 17 Not Applicable Not Applicable Not Applicable 3261 Not Applicable Not Evaluated Scheduled Follow-up Submission Complete Remission/Response Not Evaluated NO 7 Not Available NO Not Evaluated TUMOR FREE NO Complete Remission/Response Not Available NO Alive 2013 FEMALE Lung Bronchioloalveolar Carcinoma Nonmucinous No C34.3 8252/3 C34.3 YES Not Available NO Not Available Not Available Not Available 7 Not Available Not Available Not Available Not Available Not Available Not Available Not Available Not Available Not Available Not Available Not Available No 6986 Not Available TUMOR FREE Not Available Not Available Not Available Not Available Not Available Not Available Not Available WHITE Not Available Not Available Not Applicable Not Applicable Not Applicable Not Applicable Not Applicable Not Applicable Not Applicable Not Applicable Not Applicable Stage IB Not Applicable Not Applicable Not Applicable 6th Not Applicable Not Applicable Not Applicable M0 N0 T2 Not Available NO YES 55 1 Lung Alive 2011 2004 Not Available

TCGA-78-7152 1215 Dead T2 N0 M0 Stage IB 65 R-Upper Not Applicable TCGA-78-7152 b56dad1e-2fd5-47d8-9cc7-a54be9a1bae2 29 -23782 Not Applicable 0 1202 Not Available Lung Adenocarcinoma Not Available 1 Not Available NO Not Available Not Available Not Available NO Not Available Not Available Not Available;NO YES;YES NO;Not Available NO;NO TCGA-78-7152-F16986;TCGA-78-7152-F46144 37c9fd67-631d-4341-998d-1c995eaa7999;D9E98591-BA37-4BE6-9F12-3E9E4DBED53B 29;4 Not Available;Not Available Not Available;Not Available Not Applicable;1215 1202;Not Available 1202;1202 3;Unknown Scheduled Follow-up Submission;Scheduled Follow-up Submission Not Available;Progressive Disease Not Available;Not Available Not Available;NO 9;8 Not Available;Distant Metastasis YES;YES Other;Not Available WITH TUMOR;WITH TUMOR NO;NO Not Available;Complete Remission/Response Not Available;Biopsy with Histologic Confirmation;Convincing Imaging NO;NO Alive;Dead 2011;2013 MALE Lung Adenocarcinoma Mixed Subtype No C34.1 8255/3 C34.1 YES Not Available NO Not Available Not Available Peripheral Lung 9 Not Available Not Available Not Available Not Available Not Available Not Available Not Available Not Available Not Available Not Available 40 No 7152 Preoperative WITH TUMOR Not Available Not Available Not Available Not Available Not Available Not Available NO WHITE Not Available Distant site TCGA-78-7152-R16987 b75b20df-f44d-4dad-b13a-6212725b3643 1 29 Not Available 1154 Not Available 9 Not Available Not Available NO EXTERNAL BEAM Not Applicable PALLIATIVE Not Available Not Available 2011 R0 Not Applicable Not Applicable Not Applicable Not Applicable Not Applicable Not Applicable Not Applicable Not Applicable Not Applicable Stage IB Not Applicable Not Applicable Not Applicable 6th Not Applicable Not Applicable Not Applicable M0 N0 T2 1999 NO YES 78 4 Lung Alive 2011 2003 1959

TCGA-55-8089 702 Dead T1a N0 M0 Stage IA 56 R-Upper Not Applicable TCGA-55-8089 1070ff27-17c9-43dd-a4f6-daa643eb9123 17 -20576 Not Applicable 0 100 Not Available Lung Adenocarcinoma 90 0 Not Available YES Not Available Not Available Not Available YES Not Available NOT HISPANIC OR LATINO Not Available Not Available Not Available Not Available TCGA-55-8089-F59075 C8B09023-1E19-4E25-83B5-FB60003D08F0 30 Not Applicable Not Applicable 702 Not Available Not Applicable Not Evaluated Scheduled Follow-up Submission Complete Remission/Response Not Evaluated NO 4 Not Available NO Not Evaluated TUMOR FREE NO Complete Remission/Response Not Available NO Dead 2014 MALE Lung Adenocarcinoma- Not Otherwise Specified (NOS) No C34.1 8140/3 C34.1 YES 100 YES NO Not Available Peripheral Lung 6 Not Available Not Available Not Available Not Available Not Available Not Available Not Available Not Available Not Available NO 97 No 8089 Preoperative TUMOR FREE Not Available Not Available Unknown 85 70 Unknown YES WHITE Unknown R0 Not Applicable Not Applicable Not Applicable Not Applicable Not Applicable Not Applicable Not Applicable Not Applicable Not Applicable Stage IA Not Applicable Not Applicable Not Applicable 7th Not Applicable Not Applicable Not Applicable M0 N0 T1a Not Available YES NO 55 2 Lung Alive 2012 2011 1973

TCGA-97-7554 775 Alive T2a N2 M0 Stage IIIA 83 L-Lower Not Applicable TCGA-97-7554 a9644274-13bb-4228-9b4f-14260ccc26eb 17 -30327 Not Applicable 0 264 Not Available Lung Adenocarcinoma 53 TCGA-97-7554-D18639;TCGA-97-7554-D18627 779af18b-5c23-4e62-ae9c-b6288f15b3da;c7851da9-c4f2-4a19-b48c-a90e5b4d9cba Not Available;Not Available 21;21 111;111 49;49 Alimta;Carboplatin Not Available;Not Available 11;11 4;4 817;350 mg;mg ADJUVANT;ADJUVANT Not Applicable;Not Applicable 1;1 IV;IV NO;NO Chemotherapy;Chemotherapy Not Available;Not Available 3268;1400 mg;mg Not Available;Not Available 2011;2011 0 Not Available Not Available Not Available Not Available Not Available Not Available Not Available NOT HISPANIC OR LATINO Not Available Not Available Not Available Not Available TCGA-97-7554-F20552 C53D0705-AD5B-47B9-8D49-C786D4466CC7 17 Not Applicable Not Applicable Not Applicable 775 Not Applicable 1 Scheduled Follow-up Submission Not Available Not Available Not Available 1 Not Available NO Post-Adjuvant Therapy TUMOR FREE YES Complete Remission/Response Not Available NO Alive 2012 FEMALE Lung Papillary Adenocarcinoma No C34.3 8260/3 C34.3 YES Not Available YES YES G12V Not Available 11 Not Available Not Available Not Available Not Available Not Available Not Available Not Available Not Available Not Available Not Available 30 No 7554 Preoperative TUMOR FREE 75 75 Not Available 76 68 Not Available YES WHITE Not Available R0 Not Applicable Not Applicable Not Applicable Not Applicable Not Applicable Not Applicable Not Applicable Not Applicable Not Applicable Stage IIIA Not Applicable Not Applicable Not Applicable 7th Not Applicable Not Applicable Not Applicable M0 N2 T2a 1983 NO YES 97 3 Lung Alive 2011 2010 1945

TCGA-64-1681 1167 Dead T1 N0 M0 Stage IA 61 R-Upper Not Applicable TCGA-64-1681 c583fdd1-8cd2-4c15-a23e-0644261f65da 21 -22525 1167 0 Not Available Not Available Lung Adenocarcinoma Not Available TCGA-64-1681-D20366 27af4417-3bc7-4992-9965-9f4b6c1f6434 Not Available 21 Not Available 481 Tarceva Not Available 12 Not Available 150 mg RECURRENCE Not Applicable 1 PO NO Chemotherapy Not Available Not Available Not Available Not Available 2011 Not Available Not Available YES Exon 19 Deletion Not Available Not Available Not Available Not Available NOT HISPANIC OR LATINO YES YES NO Not Available TCGA-64-1681-F20365 2dd45a93-1ce5-4d03-902f-6cec7a998472 21 Not Available Not Available 1167 Not Available 439 Not Available Additional New Tumor Event Not Available Not Available Not Available 12 Not Available YES Not Available Not Available NO Not Available Not Available NO Dead 2011 FEMALE Lung Adenocarcinoma- Not Otherwise Specified (NOS) No C34.1 8140/3 C34.9 YES 0 YES NO Not Available Not Available 12 Not Available Not Available Not Available Not Available Not Available Not Available Not Available Not Available Not Available Not Available Not Available No 1681 Not Available Not Available Not Available Not Available Not Available Not Available Not Available Not Available Not Available WHITE Not Available Distant site TCGA-64-1681-R20367 99c88968-d1fb-474c-bf43-99a06c4386ac 1 21 Not Available 711 Not Available 12 Not Available 3000 NO EXTERNAL BEAM Not Applicable RECURRENCE Not Available cGy 2011 R0 Not Applicable Not Applicable Not Applicable Not Applicable Not Applicable Not Applicable Not Applicable Not Applicable Not Applicable Stage IA Not Applicable Not Applicable Not Applicable 6th Not Applicable Not Applicable Not Applicable M0 N0 T1 1988 NO YES 64 3 Lung Dead 2011 2008 1968

TCGA-78-7147 586 Dead T2 N1 M0 Stage IIB 67 R-Upper Not Applicable TCGA-78-7147 e7d30bc2-42e8-49c5-a5b3-9f58411b007a 28 -24809 586 0 Not Available Not Available Lung Adenocarcinoma Not Available 1 Not Available NO Not Available Not Available Not Available NO Not Available Not Available NO NO NO NO TCGA-78-7147-F16971 a52cbdf4-8abe-4493-9575-72d42251aea8 28 Not Available Not Available 586 Not Available 578 Not Available Scheduled Follow-up Submission Progressive Disease Not Available Not Available 9 Not Available YES Not Available WITH TUMOR NO Not Available Not Available NO Dead 2011 FEMALE Lung Adenocarcinoma- Not Otherwise Specified (NOS) No C34.1 8140/3 C34.1 YES Not Available NO Not Available Not Available Peripheral Lung 9 Not Available Not Available Not Available Not Available Not Available Not Available Not Available Not Available Not Available Not Available 50 No 7147 Preoperative WITH TUMOR Not Available Not Available Not Available Not Available Not Available Not Available Not Available WHITE Not Available R0 Not Applicable Not Applicable Not Applicable Not Applicable Not Applicable Not Applicable Not Applicable Not Applicable Not Applicable Stage IIB Not Applicable Not Applicable Not Applicable 6th Not Applicable Not Applicable Not Applicable M0 N1 T2 Not Available NO YES 78 2 Lung Dead 2011 2001 1950

TCGA-05-4396 303 Dead T4 N1 M0 Stage IIIB 76 R-Lower Not Applicable TCGA-05-4396 9f81c602-8afa-4588-b0b6-6e5a1a128d5a 22 -28094 303 0 Not Available Not Available Lung Adenocarcinoma Not Available Not Available Not Available Not Available Not Available Not Available Not Available Not Available Not Available Not Available MALE Lung Adenocarcinoma Mixed Subtype No C34.3 8255/3 C34.3 YES Not Available Not Available Not Available Not Available Not Available 7 Not Available Not Available Not Available Not Available Not Available Not Available Not Available Not Available Not Available Not Available 19 Yes 4396 Not Available Not Available Not Available Not Available Not Available Not Available Not Available Not Available Not Available Not Available Not Available R0 Not Applicable Not Applicable Not Applicable Not Applicable Not Applicable Not Applicable Not Applicable Not Applicable Not Applicable Stage IIIB Not Applicable Not Applicable Not Applicable 5th Not Applicable Not Applicable Not Applicable M0 N1 T4 1984 NO YES 05 3 Lung Dead 2010 2006 1947

TCGA-95-7039 1272 Alive T3 N0 MX Stage IIB 54 R-Upper Not Applicable TCGA-95-7039 ae702159-5b6a-41dd-868c-7540f9e1131c Not Available -19981 Not Applicable 0 34 Not Available Lung Adenocarcinoma Not Available 1 Not Available Not Available Not Available Not Available Not Available Not Available Not Available NOT HISPANIC OR LATINO Not Available;Not Available;NO Not Available;Not Available;NO Not Available;Not Available;NO Not Available;Not Available;Not Available TCGA-95-7039-F15635;TCGA-95-7039-F40734;TCGA-95-7039-F67192 1a1c33a9-1df7-4697-9cac-7d942e1bf067;4C4658AB-3BB7-4D82-B7DF-5CA813F3F854;7EAB8F50-92B9-438E-B11F-A8EF2BCB7807 Not Available;15;31 Not Available;Not Available;Not Available Not Available;Not Available;Not Available Not Applicable;Not Applicable;Not Applicable 34;34;1272 Not Available;Not Available;1258 1;Not Available;Unknown Scheduled Follow-up Submission;Scheduled Follow-up Submission;Additional New Tumor Event Complete Remission/Response;Complete Remission/Response;Progressive Disease Not Available;Not Available;Not Evaluated Not Available;YES;NO 8;3;10 Not Available;Not Available;Locoregional Recurrence Not Available;Unknown;YES Preoperative;Not Available;Not Available Not Available;Not Available;WITH TUMOR NO;NO;NO Complete Remission/Response;Not Available;Complete Remission/Response Not Available;Not Available;Biopsy with Histologic Confirmation;Convincing Imaging;Positive Biomarker(s) NO;NO;NO Alive;Alive;Alive 2011;2013;2014 FEMALE Lung Adenocarcinoma- Not Otherwise Specified (NOS) No C34.1 8140/3 C34.1 YES Not Available Not Available Not Available Not Available Not Available 8 Not Available Not Available Not Available Not Available Not Available Not Available Not Available Not Available Not Available Not Available 34 No 7039 Preoperative TUMOR FREE Not Available Not Available Not Available Not Available Not Available Not Available Not Available WHITE Not Available Not Available Not Applicable Not Applicable Not Applicable Not Applicable Not Applicable Not Applicable Not Applicable Not Applicable Not Applicable Stage IIB Not Applicable Not Applicable Not Applicable 7th Not Applicable Not Applicable Not Applicable MX N0 T3 Not Available YES NO 95 2 Lung Alive 2011 2011 Not Available

TCGA-62-A46S 1653 Dead T2 N0 M0 Stage IB 73 L-Upper Not Applicable TCGA-62-A46S E00A60D7-4253-443D-A187-680AD0931349 29 -26825 1653 0 Not Available Not Available Lung Adenocarcinoma Not Available TCGA-62-A46S-D36326;TCGA-62-A46S-D40574 A78743CC-2953-4ECE-A4AE-0C60106B1A3C;C9C16A96-C581-4DB9-B7F3-5B6ACCC39DFC Not Available;Not Available 31;25 198;198 78;78 Tarceva;Placebo Unknown;Unknown 10;2 Not Available;Not Available Not Available;Not Available Not Available;Not Available Not Available;Not Available Not Applicable;Not Applicable Not Available;Not Available Not Available;Not Available NO;NO Targeted Molecular therapy;Targeted Molecular therapy Not Available;Not Available Not Available;Not Available Not Available;Not Available YES;YES 2012;2013 0 Not Available NO Not Available Not Available Not Available NO Not Available NOT HISPANIC OR LATINO MALE Lung Papillary Adenocarcinoma No C34.1 8260/3 C34.1 YES 100 NO Not Available Not Available Peripheral Lung 10 YES NO 527 NO Not Available NO Not Available Locoregional Recurrence;Distant Metastasis Convincing Imaging YES 40 No A46S Preoperative WITH TUMOR Not Available Not Available YES 66 72 Complete Remission/Response YES WHITE NO R0 Not Applicable Not Applicable Not Applicable Not Applicable Not Applicable Not Applicable Not Applicable Not Applicable Not Applicable Stage IB Not Applicable Not Applicable Not Applicable 6th Not Applicable Not Applicable Not Applicable M0 N0 T2 Not Available NO YES 62 4 Lung Dead 2012 2007 Not Available

TCGA-55-A4DF 440 Dead T1b N0 MX Stage IA 88 R-Lower Not Applicable TCGA-55-A4DF 362000E4-CF7C-48FC-93CC-8F5994B442EA 25 -32432 Not Applicable 0 47 Not Available Lung Adenocarcinoma 82 Not Evaluated Not Available Unknown Not Available Not Available Not Available Unknown Not Available NOT HISPANIC OR LATINO NO YES NO NO TCGA-55-A4DF-F59072 B4A1C81E-113A-41E4-8C10-FEFA43BCB273 30 Not Available Not Available 440 Not Available 440 Not Evaluated Scheduled Follow-up Submission Progressive Disease Not Evaluated NO 4 Distant Metastasis YES Not Available WITH TUMOR NO Complete Remission/Response Convincing Imaging NO Dead 2014 MALE Lung Adenocarcinoma- Not Otherwise Specified (NOS) No C34.3 8140/3 C34.3 YES Not Evaluated Unknown Not Available Not Available Unknown 1 Not Available Not Available Not Available Not Available Not Available Not Available Not Available Not Available Not Available NO 60 Yes, History of Synchronous/Bilateral Malignancy A4DF Not Available TUMOR FREE Not Available 58 NO Not Available 47 Complete Remission/Response YES WHITE NO Distant Recurrence TCGA-55-A4DF-R59073 64AB51F5-F53A-403A-8BA3-694141F508E7 Not Available 30 440 440 Radiographic Progressive Disease 4 Not Available 3500 NO External Not Applicable Not Available Not Available cGy 2014 R0 Not Applicable Not Applicable Not Applicable Not Applicable Not Applicable Not Applicable Not Applicable Not Applicable Not Applicable Stage IA Not Applicable Not Applicable Not Applicable 7th Not Applicable Not Applicable Not Applicable MX N0 T1b 1984 YES NO 55 3 Lung Alive 2013 2012 Not Available

TCGA-MP-A4TE 896 Dead T2b N0 MX Stage IIA 56 L-Lower Not Applicable TCGA-MP-A4TE BEBD0025-74E2-451B-93B3-86F82DF43573 2 -20627 896 0 Not Available Not Available Lung Adenocarcinoma Not Available Not Evaluated Not Available NO Not Available Not Available Not Available NO Not Available NOT HISPANIC OR LATINO YES;Unknown NO;Unknown NO;Not Available Not Available;NO TCGA-MP-A4TE-F41404;TCGA-MP-A4TE-F41405 1239E6E6-49B6-494D-B3DC-33A65B8F4058;DCD8612E-0ACE-4A8A-AD8A-FDBC2CC58B58 18;18 Not Available;Not Available Not Available;Not Available 896;896 Not Available;Not Available 411;845 Not Evaluated;Not Evaluated Additional New Tumor Event;Additional New Tumor Event Progressive Disease;Unknown Not Evaluated;Not Evaluated NO;NO 3;3 Locoregional Recurrence;Distant Metastasis YES;YES Not Evaluated;Not Evaluated WITH TUMOR;WITH TUMOR NO;NO Complete Remission/Response;Complete Remission/Response Convincing Imaging;Convincing Imaging NO;NO Dead;Dead 2013;2013 MALE Lung Adenocarcinoma- Not Otherwise Specified (NOS) No C34.3 8140/3 C34.3 YES Not Evaluated YES YES G12C Peripheral Lung 4 NO NO 226 YES 226 Not Available Not Available Locoregional Recurrence Biopsy with Histologic Confirmation YES 40 No A4TE Not Evaluated WITH TUMOR Not Available Not Available NO Not Available Not Available Complete Remission/Response NO WHITE NO RX Not Applicable Not Applicable Not Applicable Not Applicable Not Applicable Not Applicable Not Applicable Not Applicable Not Applicable Stage IIA Not Applicable Not Applicable Not Applicable 7th Not Applicable Not Applicable Not Applicable MX N0 T2b 2010 NO YES MP 4 Lung Dead 2013 2010 1970

TCGA-97-A4LX 614 Alive T2a N0 M0 Stage IB 81 Other (please specify) Lingula TCGA-97-A4LX E2B0D873-4942-4483-9A88-D5C6338D0382 7 -29915 Not Applicable 0 272 Not Available Lung Adenocarcinoma 61 1 Not Available NO Not Available Not Available Not Available NO Not Available NOT HISPANIC OR LATINO Not Available Not Available Not Available Not Available TCGA-97-A4LX-F57384 81B37883-9051-4643-B671-9899F7A40B07 10 Not Applicable Not Applicable Not Applicable 614 Not Applicable 0 Scheduled Follow-up Submission Complete Remission/Response Unknown NO 3 Not Available NO Other TUMOR FREE NO Complete Remission/Response Not Available NO Alive 2014 MALE Lung Adenocarcinoma Mixed Subtype No C34.1 8255/3 C34.1 YES Not Evaluated YES YES G12A Unknown 3 Not Available Not Available Not Available Not Available Not Available Not Available Not Available Not Available Not Available NO 120 Yes, History of Synchronous/Bilateral Malignancy A4LX Preoperative Unknown 96 90 NO 91 91 Complete Remission/Response YES WHITE NO Not Evaluated Not Applicable Not Applicable Not Applicable Not Applicable Not Applicable Not Applicable Not Applicable Not Applicable Not Applicable Stage IB Not Applicable Not Applicable Not Applicable 7th Not Applicable Not Applicable Not Applicable M0 N0 T2a 1985 YES NO 97 3 Lung Alive 2013 2012 1945

TCGA-49-6745 522 Alive T2a N2 M0 Stage IIIA 82 L-Upper Not Applicable TCGA-49-6745 9c7875ad-ab71-4d48-b2e3-e4c7a46393e9 11 -30133 Not Applicable 0 156 Not Available Lung Adenocarcinoma Not Available TCGA-49-6745-D16687 8a687c99-eae5-4635-80db-1d8acc5bcff1 Not Available 23 156 64 Not Available Not Available 9 Not Available Not Available Not Available ADJUVANT Not Applicable 1 IV NO Chemotherapy Not Available Not Available Not Available Not Available 2011 Not Available Not Available YES Not Available Not Available Not Available Not Available Not Available Not Available Not Available Not Available Not Available Not Available TCGA-49-6745-F15015 47cc0678-9e26-4402-8fcf-1e549c6cf16d 11 Not Applicable Not Applicable Not Applicable 522 Not Applicable Not Available Scheduled Follow-up Submission Not Available Not Available Not Available 8 Not Available NO Not Available TUMOR FREE YES Not Available Not Available NO Alive 2011 MALE Lung Adenocarcinoma- Not Otherwise Specified (NOS) No C34.1 8260/3 C34.1 YES Not Available YES NO Not Available Not Available 8 Not Available Not Available Not Available Not Available Not Available Not Available Not Available Not Available Not Available Not Available 20 Yes 6745 Not Available TUMOR FREE Not Available Not Available Not Available Not Available Not Available Not Available Not Available WHITE Not Available Not Available Not Applicable Not Applicable Not Applicable Not Applicable Not Applicable Not Applicable Not Applicable Not Applicable Not Applicable Stage IIIA Not Applicable Not Applicable Not Applicable 7th Not Applicable Not Applicable Not Applicable M0 N2 T2a Not Available NO YES 49 4 Lung Alive 2011 2011 Not Available

TCGA-73-4662 2515 Alive T1 N0 M0 Stage IA 65 L-Upper Not Applicable TCGA-73-4662 294ff941-aea1-4588-9a0e-e9f5393e2bb6 6 -23762 Not Applicable 0 912 Not Available Lung Adenocarcinoma 116 0 Not Available NO Not Available Not Available Not Available NO Not Available NOT HISPANIC OR LATINO YES;Not Available YES;Not Available YES;Not Available NO;Not Available TCGA-73-4662-F8554;TCGA-73-4662-F71029 7772d756-649c-41b9-85f1-b38aa3c4cdea;FFBDD995-8D42-4D31-AB72-91A28CBECA08 6;18 36;Not Applicable Not Available;Not Applicable Not Applicable;Not Applicable 912;2515 21;Not Applicable 0;Not Evaluated Not Available;Scheduled Follow-up Submission Complete Remission/Response;Complete Remission/Response 100;Not Evaluated Not Available;NO 1;3 Not Available;Not Available YES;NO Adjuvant therapy;Not Evaluated TUMOR FREE;TUMOR FREE NO;NO Complete Remission/Response;Complete Remission/Response Not Available;Not Available NO;NO Alive;Alive 2011;2015 FEMALE Lung Adenocarcinoma- Not Otherwise Specified (NOS) No C34.1 8140/3 C34.1 YES 100 NO Not Available Not Available Peripheral Lung 1 Not Available Not Available Not Available Not Available Not Available Not Available Not Available Not Available Not Available Not Available 10 No 4662 Preoperative TUMOR FREE Not Available Not Available Not Available 115 111 Not Available YES WHITE Not Available R0 Not Applicable Not Applicable Not Applicable Not Applicable Not Applicable Not Applicable Not Applicable Not Applicable Not Applicable Stage IA Not Applicable Not Applicable Not Applicable 6th Not Applicable Not Applicable Not Applicable M0 N0 T1 1972 NO YES 73 3 Lung Alive 2011 2007 1955

TCGA-62-A472 910 Alive T3 N0 M0 Stage IIB 70 R-Lower Not Applicable TCGA-62-A472 E499069B-A16A-49E9-941A-E3E9EA62AF25 30 -25893 Not Applicable 0 540 Not Available Lung Adenocarcinoma Not Available 1 Not Available NO Not Available Not Available Not Available NO Not Available NOT HISPANIC OR LATINO NO YES NO Not Available TCGA-62-A472-F51442 1082EB06-8D5B-4D85-8A83-B2125E571F3E 19 Not Available Not Available Not Applicable 910 290 1 Scheduled Follow-up Submission Partial Remission/Response 80 NO 11 Locoregional Recurrence YES Preoperative WITH TUMOR NO Complete Remission/Response Convincing Imaging NO Alive 2013 MALE Lung Adenocarcinoma- Not Otherwise Specified (NOS) No C34.3 8140/3 C34.3 YES 80 NO Not Available Not Available Central Lung 10 NO YES 290 NO Not Available Not Available Not Available Locoregional Recurrence Biopsy with Histologic Confirmation;Convincing Imaging YES 60 No A472 Preoperative WITH TUMOR Not Available Not Available NO 70 87 Complete Remission/Response YES WHITE NO R0 Not Applicable Not Applicable Not Applicable Not Applicable Not Applicable Not Applicable Not Applicable Not Applicable Not Applicable Stage IIB Not Applicable Not Applicable Not Applicable 7th Not Applicable Not Applicable Not Applicable M0 N0 T3 Not Available NO YES 62 2 Lung Alive 2012 2011 Not Available

TCGA-97-A4M2 624 Alive T1a N0 M0 Stage IA 66 R-Middle Not Applicable TCGA-97-A4M2 345AEF28-D49B-491B-A337-B1AC55C37D35 7 -24313 Not Applicable 0 197 Not Available Lung Adenocarcinoma 91 1 Not Available NO Not Available Not Available Not Available NO Not Available NOT HISPANIC OR LATINO Not Available Not Available Not Available Not Available TCGA-97-A4M2-F57453 C19E7A8D-1092-4915-BC25-8935E50F3DA1 12 Not Applicable Not Applicable Not Applicable 624 Not Applicable Not Evaluated Scheduled Follow-up Submission Complete Remission/Response Not Evaluated NO 3 Not Available NO Not Available TUMOR FREE NO Complete Remission/Response Not Available NO Alive 2014 MALE Lung Adenocarcinoma Mixed Subtype No C34.2 8255/3 C34.2 YES Not Evaluated YES NO Not Available Unknown 3 Not Available Not Available Not Available Not Available Not Available Not Available Not Available Not Available Not Available NO 100 Yes, History of Prior Malignancy A4M2 Preoperative TUMOR FREE 89 103 NO 96 102 Complete Remission/Response YES WHITE NO R0 Not Applicable Not Applicable Not Applicable Not Applicable Not Applicable Not Applicable Not Applicable Not Applicable Not Applicable Stage IA Not Applicable Not Applicable Not Applicable 7th Not Applicable Not Applicable Not Applicable M0 N0 T1a Not Available YES NO 97 2 Lung Alive 2013 2012 1972

TCGA-NJ-A55R 603 Alive T1b N0 MX Stage IA 67 L-Upper Not Applicable TCGA-NJ-A55R 7C7D777B-BF00-4C24-97FC-922580D5605F 27 -24738 Not Applicable 0 603 Not Available Lung Adenocarcinoma Not Available Not Available Not Available NO Not Available Not Available Not Available NO Not Available NOT HISPANIC OR LATINO Not Available;Not Available Not Available;Not Available Not Available;Not Available Not Available;Not Available TCGA-NJ-A55R-F50899;TCGA-NJ-A55R-F70652 02EB453B-5913-4F97-A3A3-6E67ECC7568E;2000A52F-31B2-4A1B-ACB6-36A88942F0E0 1;27 Not Applicable;Not Applicable Not Applicable;Not Applicable Not Applicable;Not Applicable 603;603 Not Applicable;Not Applicable Not Available;Not Available Scheduled Follow-up Submission;Scheduled Follow-up Submission Stable Disease;Stable Disease Not Available;Not Available NO;YES 11;2 Not Available;Not Available NO;NO Not Available;Not Available TUMOR FREE;TUMOR FREE NO;NO Stable Disease;Stable Disease Not Available;Not Available NO;NO Alive;Alive 2013;2015 MALE Lung Adenocarcinoma- Not Otherwise Specified (NOS) No C34.1 8230/3 C34.1 YES Not Available NO Not Available Not Available Unknown 9 Not Available Not Available Not Available Not Available Not Available Not Available Not Available Not Available Not Available NO 5 No A55R Not Available TUMOR FREE Not Available Not Available NO Not Available Not Available Stable Disease NO WHITE NO Not Available Not Applicable Not Applicable Not Applicable Not Applicable Not Applicable Not Applicable Not Applicable Not Applicable Not Applicable Stage IA Not Applicable Not Applicable Not Applicable 7th Not Applicable Not Applicable Not Applicable MX N0 T1b 1986 Not Available Not Available NJ 3 Lung Alive 2013 2011 Not Available

TCGA-50-6595 189 Dead T2 N2 M0 Stage IIIA 74 R-Upper Not Applicable TCGA-50-6595 82476d2d-e403-4f6b-8dd6-cc84e3329478 25 -27197 189 0 Not Available Not Available Lung Adenocarcinoma Not Available TCGA-50-6595-D44132 5CE76459-2545-4AE1-BFB7-D99A28C8576B Not Available 13 162 123 Carboplatin Clinical Progressive Disease 6 Not Available Not Available Not Available Not Available Not Applicable Not Available Not Available NO Chemotherapy Not Available Not Available Not Available NO 2013 Not Available Not Available YES Not Available Not Available Not Available YES Not Available NOT HISPANIC OR LATINO NO NO NO YES TCGA-50-6595-F44127 AEA27241-AF39-4C5C-ADA3-91E25F21073D 13 Not Available 187 189 Not Available 182 Not Available Scheduled Follow-up Submission Progressive Disease Not Available NO 6 Distant Metastasis YES Not Available WITH TUMOR YES Progressive Disease Biopsy with Histologic Confirmation YES Dead 2013 FEMALE Lung Adenocarcinoma- Not Otherwise Specified (NOS) No C34.1 8140/3 C34.1 YES Not Available YES Not Available Not Available Not Available 8 Not Available Not Available Not Available Not Available Not Available Not Available Not Available Not Available Not Available Not Available Not Available No 6595 Not Available WITH TUMOR Not Available Not Available Not Available Not Available Not Available Not Available NO WHITE Not Available Primary Tumor Field TCGA-50-6595-R44128 DF7FD391-A020-46D8-8EA6-A937605E3002 Not Available 13 161 123 Radiographic Progressive Disease 6 27 54 NO External Not Applicable Not Available Not Available Gy 2013 Not Available Not Applicable Not Applicable Not Applicable Not Applicable Not Applicable Not Applicable Not Applicable Not Applicable Not Applicable Stage IIIA Not Applicable Not Applicable Not Applicable 6th Not Applicable Not Applicable Not Applicable M0 N2 T2 1979 NO YES 50 3 Lung Dead 2011 2009 Not Available

TCGA-55-6971 1400 Alive T2 N0 MX Stage IB 59 L-Lower Not Applicable TCGA-55-6971 8d0736fe-261c-445c-bfd2-a3ea3ceaf367 28 -21734 Not Applicable 0 25 Not Available Lung Adenocarcinoma Not Available Not Available Not Available NO Not Available Not Available Not Available NO Not Available Not Available Not Available Not Available Not Available Not Available TCGA-55-6971-F56190 12A8DE46-0514-47CB-A544-77296F337007 29 Not Applicable Not Applicable Not Applicable 1400 Not Applicable Unknown Scheduled Follow-up Submission Complete Remission/Response Unknown NO 1 Not Available NO Not Available TUMOR FREE NO Complete Remission/Response Not Available NO Alive 2014 FEMALE Lung Adenocarcinoma- Not Otherwise Specified (NOS) No C34.3 8140/3 C34.3 YES Not Available NO Not Available Not Available Not Available 7 Not Available Not Available Not Available Not Available Not Available Not Available Not Available Not Available Not Available Not Available 60 No 6971 Not Available TUMOR FREE Not Available Not Available Not Available Not Available Not Available Not Available Not Available WHITE Not Available R0 Not Applicable Not Applicable Not Applicable Not Applicable Not Applicable Not Applicable Not Applicable Not Applicable Not Applicable Stage IB Not Applicable Not Applicable Not Applicable 7th Not Applicable Not Applicable Not Applicable MX N0 T2 2010 NO YES 55 4 Lung Alive 2011 2010 1970

TCGA-55-6543 435 Alive T1b N0 MX Stage IA 60 R-Upper Not Applicable TCGA-55-6543 e68219b0-a9c2-49df-8f0d-db5ea97fd2dc 3 Not Available Not Applicable 0 2 Not Available Lung Adenocarcinoma Not Available Not Available Not Available Not Available Not Available Not Available Not Available Not Available Not Available Not Available Not Available Not Available Not Available Not Available TCGA-55-6543-F46659 328EC06C-F892-4324-A3ED-FB2A283BCC0B 13 Not Applicable Not Applicable Not Applicable 435 Not Applicable Unknown Scheduled Follow-up Submission Complete Remission/Response Unknown NO 8 Not Available NO Unknown TUMOR FREE NO Complete Remission/Response Not Available NO Alive 2013 FEMALE Lung Bronchioloalveolar Carcinoma Mucinous No C34.1 8253/3 C34.1 YES Not Available Not Available Not Available Not Available Peripheral Lung 12 Not Available Not Available Not Available Not Available Not Available Not Available Not Available Not Available Not Available Not Available 60 No 6543 Not Available TUMOR FREE Not Available Not Available Not Available Not Available Not Available Not Available Not Available WHITE Not Available Not Available Not Applicable Not Applicable Not Applicable Not Applicable Not Applicable Not Applicable Not Applicable Not Applicable Not Applicable Stage IA Not Applicable Not Applicable Not Applicable 7th Not Applicable Not Applicable Not Applicable MX N0 T1b 2004 NO YES 55 4 Lung Alive 2010 2010 Not Available

TCGA-55-6712 171 Dead T2a N1 MX Stage IIA 71 L-Upper Not Applicable TCGA-55-6712 f17d3463-af6c-42c2-a8e9-fae45f69286f 19 Not Available Not Applicable 0 24 Not Available Lung Adenocarcinoma Not Available TCGA-55-6712-D46640;TCGA-55-6712-D46641 54BFE572-6DCC-4E84-884A-ED9F30AA3A5E;E61C6649-A6C8-4399-BC96-CE8A7ADA6686 Not Available;Not Available 13;13 164;164 55;55 Carboplatin;Taxol Clinical Progressive Disease;Clinical Progressive Disease 8;8 Not Available;Not Available Not Available;Not Available Not Available;Not Available Not Available;Not Available Not Applicable;Not Applicable Not Available;Not Available Not Available;Not Available NO;NO Chemotherapy;Chemotherapy Not Available;Not Available Not Available;Not Available Not Available;Not Available NO;NO 2013;2013 Not Available Not Available Not Available Not Available Not Available Not Available Not Available Not Available Not Available Not Available Not Available Not Available Not Available TCGA-55-6712-F46639 635BF546-710A-4C39-9D56-E3D0FCFBC9F6 13 Not Applicable Not Applicable 171 Not Available Not Applicable Not Evaluated Scheduled Follow-up Submission Progressive Disease Not Evaluated NO 8 Not Available NO Not Evaluated WITH TUMOR YES Progressive Disease Not Available NO Dead 2013 MALE Lung Adenocarcinoma- Not Otherwise Specified (NOS) No C34.1 8140/3 C34.1 YES Not Available Not Available Not Available Not Available Not Available 7 Not Available Not Available Not Available Not Available Not Available Not Available Not Available Not Available Not Available Not Available Not Available No 6712 Not Available WITH TUMOR Not Available Not Available Not Available Not Available Not Available Not Available Not Available WHITE Not Available R1 Not Applicable Not Applicable Not Applicable Not Applicable Not Applicable Not Applicable Not Applicable Not Applicable Not Applicable Stage IIA Not Applicable Not Applicable Not Applicable 7th Not Applicable Not Applicable Not Applicable MX N1 T2a 2003 NO YES 55 4 Lung Alive 2011 2010 1970

TCGA-44-A47B 287 Alive T2a N0 M0 Stage IB 79 R-Upper Not Applicable TCGA-44-A47B 967D6548-5A84-4B7E-BC3F-2E522859FCE6 20 -28869 Not Applicable 0 105 Not Available Lung Adenocarcinoma 73 0 Not Available NO Not Available Not Available Not Available NO Not Available NOT HISPANIC OR LATINO Not Available Not Available Not Available Not Available TCGA-44-A47B-F41746 F1FE3CC6-00FC-40B7-AD09-0F7D71224725 27 Not Applicable Not Applicable Not Applicable 287 Not Applicable Not Available Scheduled Follow-up Submission Complete Remission/Response Not Available NO 3 Not Available NO Not Available TUMOR FREE NO Complete Remission/Response Not Available NO Alive 2013 MALE Lung Adenocarcinoma- Not Otherwise Specified (NOS) No C34.1 8140/3 C34.1 YES Not Evaluated NO Not Available Not Available Not Available 11 Not Available Not Available Not Available Not Available Not Available Not Available Not Available Not Available Not Available NO 4.5 No A47B Preoperative TUMOR FREE 102 96 NO 98 96 Complete Remission/Response YES WHITE NO R0 Not Applicable Not Applicable Not Applicable Not Applicable Not Applicable Not Applicable Not Applicable Not Applicable Not Applicable Stage IB Not Applicable Not Applicable Not Applicable 7th Not Applicable Not Applicable Not Applicable M0 N0 T2a 1965 YES NO 44 3 Lung Alive 2012 2012 1956

TCGA-67-3772 573 Alive T2 N0 M0 Stage IB 82 R-Upper Not Applicable TCGA-67-3772 673493f6-975c-49e8-934c-001e9a0fff90 3 -29989 Not Applicable 0 573 Not Available Lung Adenocarcinoma Not Available Not Available Not Available NO Not Available Not Available Not Available NO Not Available NOT HISPANIC OR LATINO FEMALE Lung Adenocarcinoma- Not Otherwise Specified (NOS) No C34.1 8140/3 C34.1 YES Not Available NO Not Available Not Available Not Available 3 Not Available Not Available Not Available Not Available Not Available Not Available Not Available Not Available Not Available Not Available Not Available No 3772 Not Available TUMOR FREE Not Available Not Available Not Available Not Available Not Available Not Available Not Available WHITE Not Available Not Available Not Applicable Not Applicable Not Applicable Not Applicable Not Applicable Not Applicable Not Applicable Not Applicable Not Applicable Stage IB Not Applicable Not Applicable Not Applicable 6th Not Applicable Not Applicable Not Applicable M0 N0 T2 Not Available YES NO 67 1 Lung Alive 2011 2009 Not Available

TCGA-78-7153 3635 Alive T2 N0 M0 Stage IB 65 R-Upper Not Applicable TCGA-78-7153 c353f949-5f66-4a2c-b8f0-17be8bf19e0a 29 -23922 Not Applicable 0 760 Not Available Lung Adenocarcinoma Not Available 0 Not Available NO Not Available Not Available Not Available NO Not Available Not Available Not Available;Not Available Not Available;Not Available Not Available;Not Available Not Available;Not Available TCGA-78-7153-F16980;TCGA-78-7153-F46077 932b04a8-46cd-464a-97f5-682b9dbe77de;C9C20E98-3841-44E5-B29C-946EDFFABBAF 29;23 Not Available;Not Available Not Available;Not Available Not Applicable;Not Applicable 760;3635 Not Available;Not Available Not Available;Unknown Scheduled Follow-up Submission;Scheduled Follow-up Submission Not Available;Unknown Not Available;Not Available Not Available;NO 9;8 Not Available;Not Available Not Available;Unknown Not Available;Unknown Not Available;Unknown NO;NO Not Available;Complete Remission/Response Not Available;Not Available NO;NO Alive;Alive 2011;2013 FEMALE Lung Adenocarcinoma Mixed Subtype No C34.8 8255/3 C34.8 YES Not Available NO Not Available Not Available Peripheral Lung 9 Not Available Not Available Not Available Not Available Not Available Not Available Not Available Not Available Not Available Not Available 20 No 7153 Preoperative Not Available Not Available Not Available Not Available Not Available Not Available Not Available NO WHITE Not Available R0 Not Applicable Not Applicable Not Applicable Not Applicable Not Applicable Not Applicable Not Applicable Not Applicable Not Applicable Stage IB Not Applicable Not Applicable Not Applicable 6th Not Applicable Not Applicable Not Applicable M0 N0 T2 1988 NO YES 78 4 Lung Alive 2011 2003 1948

TCGA-75-7025 3305 Alive T2 N0 M0 Stage IB Not Available L-Lower Not Applicable TCGA-75-7025 681b1176-9676-44d3-892c-1d5dba1ce25b 11 Not Available Not Applicable Not Available Not Available Not Available Lung Adenocarcinoma Not Available 0 Not Available Not Available Not Available Not Available Not Available Not Available Not Available Not Available Not Available;YES Not Available;NO Not Available;YES Not Available;YES TCGA-75-7025-F15874;TCGA-75-7025-F72041 68cda392-87e8-466a-9dc5-e7f00f2cb0ba;3D210D11-BA20-4227-95D6-9E77D7C10C7D 11;2 Not Applicable;1481 Not Applicable;2092 Not Applicable;Not Applicable Not Available;3305 Not Applicable;1481 Not Available;Unknown Not Available;Scheduled Follow-up Submission Not Available;Complete Remission/Response Not Available;Unknown Not Available;NO 8;4 Not Available;Locoregional Recurrence;Distant Metastasis NO;YES Not Available;Unknown TUMOR FREE;TUMOR FREE NO;NO Complete Remission/Response;Complete Remission/Response Not Available;Not Available NO;NO Alive;Alive 2011;2015 MALE Lung Adenocarcinoma- Not Otherwise Specified (NOS) No C34.1 8140/3 C34.1 YES Not Available Not Available Not Available Not Available Peripheral Lung 8 Not Available Not Available Not Available Not Available Not Available Not Available Not Available Not Available Not Available Not Available Not Available No 7025 Preoperative TUMOR FREE Not Available Not Available Not Available Not Available Not Available Not Available Not Available Not Available Not Available R0 Not Applicable Not Applicable Not Applicable Not Applicable Not Applicable Not Applicable Not Applicable Not Applicable Not Applicable Stage IB Not Applicable Not Applicable Not Applicable 6th Not Applicable Not Applicable Not Applicable M0 N0 T2 1986 NO YES 75 3 Lung Alive 2011 Not Available 1967

TCGA-MP-A4SY 1501 Dead T2 N1 M0 Stage IIB 61 R-Lower Not Applicable TCGA-MP-A4SY 8CC6BCEC-65DF-46A9-A88C-09762A132857 2 -22448 1501 0 Not Available Not Available Lung Adenocarcinoma 73 0 Not Available NO Not Available Not Available Not Available NO Not Available NOT HISPANIC OR LATINO MALE Lung Adenocarcinoma- Not Otherwise Specified (NOS) No C34.3 8140/3 C34.3 YES Not Evaluated NO Not Available Not Available Central Lung 4 YES NO 489 YES 489 Not Available Not Available Locoregional Recurrence Biopsy with Histologic Confirmation YES 40 No A4SY Pre-Adjuvant Therapy TUMOR FREE Not Available 74 NO Not Available 54 Complete Remission/Response YES WHITE NO R0 Not Applicable Not Applicable Not Applicable Not Applicable Not Applicable Not Applicable Not Applicable Not Applicable Not Applicable Stage IIB Not Applicable Not Applicable Not Applicable 5th Not Applicable Not Applicable Not Applicable M0 N1 T2 2002 NO YES MP 4 Lung Dead 2013 2002 Not Available

TCGA-55-6969 1239 Alive T2 N0 M0 Stage IB 52 L-Upper Not Applicable TCGA-55-6969 d6e274a0-6425-437b-bc07-1225be7808d5 25 -19270 Not Applicable 0 1239 Not Available Lung Adenocarcinoma Not Available Not Available Not Available NO Not Available Not Available Not Available NO Not Available Not Available MALE Lung Adenocarcinoma- Not Otherwise Specified (NOS) No C34.1 8140/3 C34.1 YES Not Available NO Not Available Not Available Not Available 7 Not Available Not Available Not Available Not Available Not Available Not Available Not Available Not Available Not Available Not Available Not Available No 6969 Not Available TUMOR FREE Not Available Not Available Not Available Not Available Not Available Not Available Not Available WHITE Not Available Not Available Not Applicable Not Applicable Not Applicable Not Applicable Not Applicable Not Applicable Not Applicable Not Applicable Not Applicable Stage IB Not Applicable Not Applicable Not Applicable 6th Not Applicable Not Applicable Not Applicable M0 N0 T2 2004 NO YES 55 4 Lung Alive 2011 2004 Not Available

TCGA-78-7220 807 Dead T2 N2 M0 Stage IIIA 53 L-Upper Not Applicable TCGA-78-7220 fd5c44ef-ea50-4fba-9e8d-e371cf34ebdb 8 -19480 807 0 Not Available Not Available Lung Adenocarcinoma Not Available 0 Not Available NO Not Available Not Available Not Available NO Not Available Not Available NO YES NO NO TCGA-78-7220-F19298 6125a3be-8022-43a7-8d60-00e74d6061c3 8 Not Available Not Available 807 Not Available 531 Not Available Scheduled Follow-up Submission Not Available Not Available Not Available 12 Not Available YES Not Available WITH TUMOR NO Not Available Not Available YES Dead 2011 FEMALE Lung Adenocarcinoma Mixed Subtype No C34.1 8255/3 C34.1 YES Not Available NO Not Available Not Available Peripheral Lung 12 Not Available Not Available Not Available Not Available Not Available Not Available Not Available Not Available Not Available Not Available 35 No 7220 Preoperative WITH TUMOR Not Available Not Available Not Available Not Available Not Available Not Available Not Available WHITE Not Available Primary Tumor Field;Distant site TCGA-78-7220-R19299;TCGA-78-7220-R19300 cea1849b-13c0-4d25-a6c7-aeae07d41d0b;d5d23e28-c8be-4193-8bf5-c8587e92133e 1;2 8;8 96;740 60;734 Not Available;Not Available 12;12 25;5 5000;2000 NO;NO EXTERNAL BEAM;EXTERNAL BEAM Not Applicable;Not Applicable ADJUVANT;RECURRENCE Not Available;Not Available cGy;cGy 2011;2011 R0 Not Applicable Not Applicable Not Applicable Not Applicable Not Applicable Not Applicable Not Applicable Not Applicable Not Applicable Stage IIIA Not Applicable Not Applicable Not Applicable 6th Not Applicable Not Applicable Not Applicable M0 N2 T2 Not Available NO YES 78 2 Lung Dead 2011 2000 1965

TCGA-73-4668 467 Alive T2 N1 M0 Stage IIB 66 L-Upper Not Applicable TCGA-73-4668 b2285924-9813-4f46-bb8f-91a0efd8a9b6 6 -24255 Not Applicable 0 467 Not Available Lung Adenocarcinoma Not Available TCGA-73-4668-D7959;TCGA-73-4668-D7956;TCGA-73-4668-D7960;TCGA-73-4668-D7957 b2535da4-0fa6-43d0-92c1-05717101d861;0a6f15d0-842e-4da6-8de0-8374e694987a;cca6912e-8a06-4270-8717-86cfd00d2ec7;9eb9db2a-643c-49ca-ab5b-7dd90adcbbc5 Not Available;Not Available;Not Available;Not Available 6;6;6;6 Not Available;110;Not Available;110 369;43;369;43 Gemcitabine;Pemetrexed;Carboplatin;Carboplatin Not Available;Not Available;Not Available;Not Available 1;1;1;1 5;4;5;4 1000;500;5;6 mg/m2;mg/m2;AUC;AUC PALLIATIVE;ADJUVANT;PALLIATIVE;ADJUVANT Not Applicable;Not Applicable;Not Applicable;Not Applicable 2;1;2;1 IV;IV;IV;IV YES;NO;YES;NO Chemotherapy;Chemotherapy;Chemotherapy;Chemotherapy Not Available;Not Available;Not Available;Not Available Not Available;4320;Not Available;2184 Not Available;mg;Not Available;mg Not Available;Not Available;Not Available;Not Available 2011;2011;2011;2011 0 Not Available NO Not Available Not Available Not Available NO Not Available NOT HISPANIC OR LATINO YES;YES NO;YES NO;NO NO;NO TCGA-73-4668-F7955;TCGA-73-4668-F7958 c82cb3f9-fb87-4f7f-80c0-9ec28a8368c0;32244464-38fc-4145-a984-d23cc7196130 6;6 Not Available;Not Available Not Available;Not Available Not Applicable;Not Applicable 467;467 236;282 0;Not Available Not Available;Not Available Not Available;Stable Disease 90;80 Not Available;Not Available 1;1 Not Available;Not Available YES;YES Post-Adjuvant Therapy;Post-Adjuvant Therapy WITH TUMOR;WITH TUMOR YES;Not Available Complete Remission/Response;Not Available Not Available;Not Available NO;NO Alive;Alive 2011;2011 FEMALE Lung Micropapillary Adenocarcinoma No C34.1 8507/3 C34.1 YES 100 NO Not Available Not Available Peripheral Lung 1 Not Available Not Available Not Available Not Available Not Available Not Available Not Available Not Available Not Available Not Available 55 No 4668 Preoperative WITH TUMOR Not Available Not Available Not Available Not Available Not Available Not Available Not Available AMERICAN INDIAN OR ALASKA NATIVE Not Available Distant site TCGA-73-4668-R7962 2be59a25-2f78-497d-a99d-68364ee85d2f 1 6 299 299 Not Available 1 1 4000 NO OTHER Gamma Knife PROGRESSION Not Available cGy 2011 R0 Not Applicable Not Applicable Not Applicable Not Applicable Not Applicable Not Applicable Not Applicable Not Applicable Not Applicable Stage IIB Not Applicable Not Applicable Not Applicable Not Available Not Applicable Not Applicable Not Applicable M0 N1 T2 2005 NO YES 73 4 Lung Alive 2011 2009 1968

TCGA-MN-A4N5 84 Alive T1a N0 M0 Stage IA 63 L-Upper Not Applicable TCGA-MN-A4N5 17E09B0D-EAB2-40EC-AE3C-BE9AB63FCEF0 4 -23257 Not Applicable 0 84 Not Available Lung Adenocarcinoma 82 0 Not Available YES Not Available Not Available IHC YES Not Available NOT HISPANIC OR LATINO Not Available Not Available Not Available Not Available TCGA-MN-A4N5-F56665 7484D5CB-5D52-4177-9FF8-CC427340FC45 17 Not Applicable Not Applicable Not Applicable 84 Not Applicable Not Available Scheduled Follow-up Submission Unknown Not Available YES 2 Not Available NO Not Available TUMOR FREE NO Complete Remission/Response Not Available NO Alive 2014 MALE Lung Adenocarcinoma- Not Otherwise Specified (NOS) No C34.1 8140/3 C34.1 YES 100 NO Not Available Not Available Unknown 12 Not Available Not Available Not Available Not Available Not Available Not Available Not Available Not Available Not Available Unknown Not Available No A4N5 Preoperative Unknown Not Available Not Available NO 77 105 Complete Remission/Response YES WHITE NO R0 Not Applicable Not Applicable Not Applicable Not Applicable Not Applicable Not Applicable Not Applicable Not Applicable Not Applicable Stage IA Not Applicable Not Applicable Not Applicable 7th Not Applicable Not Applicable Not Applicable M0 N0 T1a 2011 NO YES MN 4 Lung Alive 2012 2011 1971

TCGA-64-5815 866 Alive T2 N1 M0 Stage IIB 74 R-Upper Not Applicable TCGA-64-5815 a65700c2-e58c-4fd4-aeb1-5686b8f4d212 2 -27280 Not Applicable 0 224 Not Available Lung Adenocarcinoma 87 TCGA-64-5815-D11426;TCGA-64-5815-D11429;TCGA-64-5815-D11431;TCGA-64-5815-D11434;TCGA-64-5815-D31481;TCGA-64-5815-D31482;TCGA-64-5815-D31483;TCGA-64-5815-D31484 c5c5b4f0-2f37-4d6a-b486-29c7638c00e5;96ec4fe4-fb5e-4fd3-903d-aef8816562c7;0750d485-4281-41ea-a62c-7860f4e50db9;35b6dcb0-3140-4e22-8857-a5adcc1bd27f;65568c73-1054-4a71-bb8c-ecdd22eaf03e;f1173777-7ce0-422c-8f6f-e4216233f93e;20c7f73f-0afe-4e4a-b887-93338bfd9df6;4878b403-b86b-448f-a98c-f33fb9f7ea01 Not Available;Not Available;Not Available;Not Available;Not Available;Not Available;Not Available;Not Available 2;2;2;2;3;3;3;3 103;103;188;188;132;132;195;195 93;93;146;146;83;83;146;146 Carboplatin;Gemzar;Alimta;Carboplatin;Gemcitabine;Carboplatin;Carboplatin;Almita Not Available;Not Available;Not Available;Not Available;Complete Response;Complete Response;Complete Response;Complete Response 5;5;5;5;5;5;5;5 3;3;3;3;Not Available;Not Available;Not Available;Not Available Not Available;Not Available;Not Available;Not Available;Not Available;Not Available;Not Available;Not Available Not Available;Not Available;Not Available;Not Available;Not Available;Not Available;Not Available;Not Available ADJUVANT;ADJUVANT;ADJUVANT;ADJUVANT;Not Available;Not Available;Not Available;Not Available Not Applicable;Not Applicable;Not Applicable;Not Applicable;Not Applicable;Not Applicable;Not Applicable;Not Applicable 1;1;1;1;Not Available;Not Available;Not Available;Not Available IV;IV;IV;IV;Not Available;Not Available;Not Available;Not Available NO;NO;NO;NO;NO;NO;NO;NO Chemotherapy;Chemotherapy;Chemotherapy;Chemotherapy;Chemotherapy;Chemotherapy;Chemotherapy;Chemotherapy Not Available;Not Available;Not Available;Not Available;Not Available;Not Available;Not Available;Not Available Not Available;Not Available;Not Available;Not Available;Not Available;Not Available;Not Available;Not Available Not Available;Not Available;Not Available;Not Available;Not Available;Not Available;Not Available;Not Available Not Available;Not Available;Not Available;Not Available;NO;NO;NO;NO 2011;2011;2011;2011;2012;2012;2012;2012 Not Available Not Available YES Not Available Not Available Not Available Not Available Not Available NOT HISPANIC OR LATINO Not Available;Not Available Not Available;Not Available Not Available;Not Available Not Available;Not Available TCGA-64-5815-F11425;TCGA-64-5815-F31480 a63ab9cf-8d93-4b05-b5e0-1134350e3251;986ba4af-3586-4aca-af09-a3aded054b3c 2;3 Not Applicable;Not Available Not Applicable;Not Available Not Applicable;Not Applicable 224;866 Not Applicable;Not Available Not Available;1 Not Available;Scheduled Follow-up Submission Complete Remission/Response;Unknown Not Available;Unknown Not Available;Not Available 5;5 Not Available;Not Available NO;Unknown Not Available;Post-Adjuvant Therapy TUMOR FREE;Unknown YES;YES Complete Remission/Response;Complete Remission/Response Not Available;Not Available NO;NO Alive;Alive 2011;2012 MALE Lung Adenocarcinoma Mixed Subtype No C34.1 8255/3 C34.1 YES Not Available YES Not Available Not Available Central Lung 5 Not Available Not Available Not Available Not Available Not Available Not Available Not Available Not Available Not Available Not Available 30 No 5815 Not Available TUMOR FREE Not Available Not Available Not Available 76 89 Not Available YES WHITE Not Available R0 Not Applicable Not Applicable Not Applicable Not Applicable Not Applicable Not Applicable Not Applicable Not Applicable Not Applicable Stage IIB Not Applicable Not Applicable Not Applicable 6th Not Applicable Not Applicable Not Applicable M0 N1 T2 1985 NO YES 64 3 Lung Alive 2011 2009 Not Available

TCGA-80-5607 T2 N1 M0 Stage IIB Not Available L-Upper Not Applicable TCGA-80-5607 645903b5-ba37-48a1-985b-a019b8db3236 12 Not Available Not Applicable Not Available Not Available Not Available Lung Adenocarcinoma Not Available 1 Not Available Not Available Not Available Not Available Not Available Not Available Not Available Not Available NO NO NO NO TCGA-80-5607-F15867 f401b266-27b0-450f-a213-254caa5e63c9 15 Not Available Not Available Not Applicable Not Available Not Available 1 Not Available Not Available Not Available Not Available 8 Not Available YES Not Available WITH TUMOR NO Not Available Not Available NO Alive 2011 FEMALE Lung Adenocarcinoma- Not Otherwise Specified (NOS) No C34.1 8140/3 C34.1 YES Not Available Not Available Not Available Not Available Peripheral Lung 8 Not Available Not Available Not Available Not Available Not Available Not Available Not Available Not Available Not Available Not Available 15 Yes 5607 Preoperative WITH TUMOR Not Available Not Available Not Available Not Available Not Available Not Available Not Available Not Available Not Available R0 Not Applicable Not Applicable Not Applicable Not Applicable Not Applicable Not Applicable Not Applicable Not Applicable Not Applicable Stage IIB Not Applicable Not Applicable Not Applicable 6th Not Applicable Not Applicable Not Applicable M0 N1 T2 1985 NO YES 80 3 Lung Alive 2011 2007 1965

TCGA-49-AARR 4992 Alive T1 N0 MX Stage IA 68 L-Upper Not Applicable TCGA-49-AARR 9B93A7C5-F9B4-4461-8078-0201A725C9CF 19 -24942 Not Applicable 0 4630 Not Available Lung Adenocarcinoma 103.5 1 Not Available Not Available Not Available Not Available Not Available Not Available Not Available NOT HISPANIC OR LATINO NO NO NO Not Available TCGA-49-AARR-F70574 A41DDC21-E254-43A4-BBC7-FE6D974EB739 25 Not Available Not Available Not Applicable 4992 4812 1 Scheduled Follow-up Submission Stable Disease 80 NO 2 Locoregional Recurrence YES Preoperative WITH TUMOR NO Complete Remission/Response Convincing Imaging NO Alive 2015 MALE Lung Adenocarcinoma- Not Otherwise Specified (NOS) No C34.1 8140/3 C34.1 YES 90 Not Available Not Available Not Available Not Available 6 NO NO 1099 Not Available Not Available Not Available 1099 New Primary Tumor Biopsy with Histologic Confirmation;Convincing Imaging YES Not Available No AARR Preoperative TUMOR FREE 76.3 78.8 NO 77.8 80.3 Complete Remission/Response YES BLACK OR AFRICAN AMERICAN NO R0 Not Applicable Not Applicable Not Applicable Not Applicable Not Applicable Not Applicable Not Applicable Not Applicable Not Applicable Stage IA Not Applicable Not Applicable Not Applicable 5th Not Applicable Not Applicable Not Applicable MX N0 T1 1987 NO YES 49 3 Lung Alive 2014 2001 Not Available

TCGA-62-A46O 1454 Dead T2 N0 M0 Stage IB 65 R-Lower Not Applicable TCGA-62-A46O D7AE8EFB-AA4D-4807-9772-55B0A28CCD5C 29 -24050 1454 0 Not Available Not Available Lung Adenocarcinoma Not Available Unknown Not Available NO Not Available Not Available Not Available NO Not Available NOT HISPANIC OR LATINO FEMALE Lung Adenocarcinoma- Not Otherwise Specified (NOS) No C34.3 8140/3 C34.3 YES 100 NO Not Available Not Available Central Lung 10 NO NO 943 NO Not Available NO Not Available Locoregional Recurrence;Distant Metastasis Convincing Imaging YES 48 No A46O Preoperative WITH TUMOR Not Available Not Available NO 65 106 Complete Remission/Response YES WHITE NO R0 Not Applicable Not Applicable Not Applicable Not Applicable Not Applicable Not Applicable Not Applicable Not Applicable Not Applicable Stage IB Not Applicable Not Applicable Not Applicable 6th Not Applicable Not Applicable Not Applicable M0 N0 T2 Not Available NO YES 62 2 Lung Dead 2012 2006 Not Available

TCGA-78-7154 593 Dead T3 N2 M0 Stage IIIA 72 L-Upper Not Applicable TCGA-78-7154 d288b69f-1e79-4429-8646-9994b7c54f8f 27 -26481 593 0 Not Available Not Available Lung Adenocarcinoma Not Available 1 Not Available NO Not Available Not Available Not Available NO Not Available Not Available Not Available Not Available Not Available Not Available TCGA-78-7154-F16951 9a2d2bfa-8f97-4a0f-9f2c-95bdea60e9c7 28 Not Available Not Available 593 Not Available Not Available Not Available Scheduled Follow-up Submission Not Available Not Available Not Available 9 Not Available Not Available Not Available Not Available Not Available Not Available Not Available Not Available Dead 2011 MALE Lung Adenocarcinoma- Not Otherwise Specified (NOS) No C34.1 8140/3 C34.1 YES Not Available NO Not Available Not Available Peripheral Lung 9 Not Available Not Available Not Available Not Available Not Available Not Available Not Available Not Available Not Available Not Available 112.5 No 7154 Preoperative Not Available Not Available Not Available Not Available Not Available Not Available Not Available Not Available WHITE Not Available R0 Not Applicable Not Applicable Not Applicable Not Applicable Not Applicable Not Applicable Not Applicable Not Applicable Not Applicable Stage IIIA Not Applicable Not Applicable Not Applicable 6th Not Applicable Not Applicable Not Applicable M0 N2 T3 1993 NO YES 78 4 Lung Dead 2011 2003 1948

TCGA-44-A47F 337 Alive T2b N1 M0 Stage IIB 74 L-Upper Not Applicable TCGA-44-A47F F61D599A-43BD-4743-9568-678317E75955 23 -27122 Not Applicable 0 246 Not Available Lung Adenocarcinoma Not Available TCGA-44-A47F-D36953;TCGA-44-A47F-D36954 73D0B0CD-088C-4220-9ED5-280121CCD432;2F7AD0AB-78F2-4B48-8180-C42A2ECE1D3A Not Available;Not Available 20;20 185;185 122;122 Alimta;Cisplatin Complete Response;Complete Response 11;11 Not Available;Not Available Not Available;Not Available Not Available;Not Available Not Available;Not Available Not Applicable;Not Applicable Not Available;Not Available Not Available;Not Available NO;NO Chemotherapy;Chemotherapy Not Available;Not Available Not Available;Not Available Not Available;Not Available NO;NO 2012;2012 0 Not Available Not Available Not Available Not Available Not Available Not Available Not Available NOT HISPANIC OR LATINO Not Available Not Available Not Available Not Available TCGA-44-A47F-F41750 298CEB49-6D86-40DA-9DF6-5C65A0FC938A 27 Not Applicable Not Applicable Not Applicable 337 Not Applicable Not Available Scheduled Follow-up Submission Complete Remission/Response 90 NO 3 Not Available NO Post-Adjuvant Therapy TUMOR FREE YES Complete Remission/Response Not Available NO Alive 2013 MALE Lung Adenocarcinoma- Not Otherwise Specified (NOS) No C34.1 8140/3 C34.1 YES Not Available Not Available Not Available Not Available Not Available 11 Not Available Not Available Not Available Not Available Not Available Not Available Not Available Not Available Not Available NO 20 Yes, History of Prior Malignancy A47F Preoperative TUMOR FREE 127 84 YES 122 81 Complete Remission/Response YES WHITE NO Not Available Not Applicable Not Applicable Not Applicable Not Applicable Not Applicable Not Applicable Not Applicable Not Applicable Not Applicable Stage IIB Not Applicable Not Applicable Not Applicable 7th Not Applicable Not Applicable Not Applicable M0 N1 T2b 1987 YES NO 44 3 Lung Alive 2012 2012 1955

TCGA-55-6968 1293 Dead T1 N0 M1 Stage IV 61 R-Lower Not Applicable TCGA-55-6968 83a6ed20-b3cf-48b9-8ed3-a080cd4951fa 28 -22344 1293 0 Not Available Not Available Lung Adenocarcinoma Not Available TCGA-55-6968-D56252 FA5390E8-6281-46F2-891D-82EAF29D6654 Not Available 30 Not Available 13 Not Available Stable Disease 1 Not Available Not Available Not Available Not Available Not Applicable Not Available Not Available NO Chemotherapy Not Available Not Available Not Available NO 2014 Not Available Not Available Not Available Not Available Not Available Not Available Not Available Not Available NOT HISPANIC OR LATINO Not Available Not Available Not Available Not Available TCGA-55-6968-F56247 78B86F68-8C53-4B9F-8321-02FDEB8CB702 30 Not Applicable Not Applicable 1293 Not Available Not Applicable Unknown Scheduled Follow-up Submission Stable Disease Unknown NO 1 Not Available NO Not Available WITH TUMOR YES Stable Disease Not Available YES Dead 2014 MALE Lung Adenocarcinoma- Not Otherwise Specified (NOS) No C34.3 8140/3 C34.3 YES Not Available Not Available Not Available Not Available Not Available 7 Not Available Not Available Not Available Not Available Not Available Not Available Not Available Not Available Not Available Not Available Not Available No 6968 Not Available WITH TUMOR Not Available Not Available Not Available Not Available Not Available Not Available Not Available WHITE Not Available Distant site TCGA-55-6968-R56251 2FDE65C3-7B12-4A5E-9DEF-E8CAFBDAD8D6 Not Available 30 57 13 Stable Disease 1 Not Available Not Available NO External Not Applicable Not Available Not Available Not Available 2014 Not Available Not Applicable Not Applicable Not Applicable Not Applicable Not Applicable Not Applicable Not Applicable Not Applicable Not Applicable Stage IV Not Applicable Not Applicable Not Applicable 6th Not Applicable Not Applicable Not Applicable M1 N0 T1 Not Available NO YES 55 2 Lung Dead 2011 2004 Not Available

TCGA-97-8171 568 Alive T2a N2 M1a Stage IV 81 Other (please specify) RUL and RML TCGA-97-8171 397d3f69-1453-4057-b177-8723eec923d1 13 -29627 Not Applicable 0 107 Not Available Lung Adenocarcinoma 110 TCGA-97-8171-D44343 26978718-D8A3-4747-A8BE-D3C96E87A6A3 Not Available 19 Not Available 16 Tarceva Not Available 6 Not Available Not Available Not Available Not Available Not Applicable Not Available Not Available YES Targeted Molecular therapy Not Available Not Available Not Available NO 2013 1 Not Available YES Exon 19 Deletion Not Available Not Available NO Not Available NOT HISPANIC OR LATINO YES NO Not Available YES TCGA-97-8171-F44232 98AD8302-28ED-452F-8867-B5A52BF7A2EE 19 Not Available 441 Not Applicable 568 441 Not Evaluated Scheduled Follow-up Submission Progressive Disease Not Evaluated NO 6 Distant Metastasis YES Not Evaluated WITH TUMOR YES Progressive Disease Biopsy with Histologic Confirmation NO Alive 2013 MALE Lung Papillary Adenocarcinoma No C34.8 8260/3 C34.8 YES Not Available YES NO Not Available Not Available 6 Not Available Not Available Not Available Not Available Not Available Not Available Not Available Not Available Not Available NO 65 No 8171 Preoperative WITH TUMOR 55 104 Unknown 55 99 Progressive Disease YES ASIAN Unknown Not Available Not Applicable Not Applicable Not Applicable Not Applicable Not Applicable Not Applicable Not Applicable Not Applicable Not Applicable Stage IV Not Applicable Not Applicable Not Applicable 7th Not Applicable Not Applicable Not Applicable M1a N2 T2a Not Available YES NO 97 2 Lung Alive 2012 2011 1946

TCGA-05-4397 731 Dead T2 N1 M0 Stage IIB 65 R-Middle Not Applicable TCGA-05-4397 6dfd47d2-831a-4386-9051-f78199a16bb5 22 -23833 731 0 Not Available Not Available Lung Adenocarcinoma Not Available Not Available Not Available Not Available Not Available Not Available Not Available Not Available Not Available Not Available MALE Lung Adenocarcinoma Mixed Subtype No C34.2 8255/3 C34.2 YES Not Available Not Available Not Available Not Available Not Available 7 Not Available Not Available Not Available Not Available Not Available Not Available Not Available Not Available Not Available Not Available 45 No 4397 Not Available Not Available Not Available Not Available Not Available Not Available Not Available Not Available Not Available Not Available Not Available R0 Not Applicable Not Applicable Not Applicable Not Applicable Not Applicable Not Applicable Not Applicable Not Applicable Not Applicable Stage IIB Not Applicable Not Applicable Not Applicable 5th Not Applicable Not Applicable Not Applicable M0 N1 T2 Not Available NO YES 05 2 Lung Dead 2010 2006 Not Available

TCGA-50-5072 250 Dead T2 N2 M0 Stage IIIA 74 L-Upper Not Applicable TCGA-50-5072 1575148b-0f63-44ee-86fd-93ae1a02be0b 26 -27036 Not Applicable 0 246 Not Available Lung Adenocarcinoma Not Available TCGA-50-5072-D16665;TCGA-50-5072-D16677;TCGA-50-5072-D32061;TCGA-50-5072-D32062 4adceb9e-78a8-4ce3-b347-89e14fa7ad72;74b1d95f-22cc-40ec-9b91-d894a1520042;84dcbd76-6ce7-47f8-beba-5421a69c3189;26e785c9-d595-4ed7-a15b-7a0d73b47e07 Not Available;Not Available;Not Available;Not Available 22;23;16;16 68;68;182;182 22;22;121;121 Cisplatin;TAXOTERE;Carboplatin;Taxol Not Available;Not Available;Clinical Progressive Disease;Clinical Progressive Disease 9;9;5;5 3;3;Not Available;Not Available 4;4;Not Available;Not Available mg/m2;mg/m2;Not Available;Not Available OTHER, SPECIFY IN NOTES;OTHER, SPECIFY IN NOTES;Not Available;Not Available neoadjuvant;neoadjuvant;Not Applicable;Not Applicable 1;Not Available;Not Available;Not Available IV;IV;Not Available;Not Available NO;NO;NO;NO Chemotherapy;Chemotherapy;Chemotherapy;Chemotherapy Not Available;Not Available;Not Available;Not Available 225;225;Not Available;Not Available mg/m2;mg/m2;Not Available;Not Available Not Available;Not Available;NO;NO 2011;2011;2012;2012 Not Available Not Available YES Not Available Not Available Not Available YES Not Available NOT HISPANIC OR LATINO NO YES Not Available NO TCGA-50-5072-F32059 cf66baf1-832b-4912-8b40-7be0f47acbbc 16 Not Available Not Available 250 Not Available 213 Not Available Scheduled Follow-up Submission Progressive Disease Not Available NO 5 Distant Metastasis YES Not Available WITH TUMOR YES Progressive Disease Convincing Imaging YES Dead 2012 MALE Lung Adenocarcinoma- Not Otherwise Specified (NOS) Yes C34.1 8140/3 C34.1 YES Not Available YES Not Available Not Available Not Available 8 Not Available Not Available Not Available Not Available Not Available Not Available Not Available Not Available Not Available Not Available 15 No 5072 Not Available WITH TUMOR Not Available Not Available Not Available Not Available Not Available Not Available NO WHITE Not Available Primary Tumor Field TCGA-50-5072-R32060 1637644b-0d29-4cc1-8ddd-bd095e729cdb Not Available 16 182 152 Not Available 5 29 5800 NO EXTERNAL BEAM Not Applicable ADJUVANT Not Available cGy 2012 Not Available Not Applicable Not Applicable Not Applicable Not Applicable Not Applicable Not Applicable Not Applicable Not Applicable Not Applicable Stage IIIA Not Applicable Not Applicable Not Applicable 6th Not Applicable Not Applicable Not Applicable M0 N2 T2 1974 NO YES 50 3 Lung Alive 2011 2009 Not Available

TCGA-05-4403 578 Alive T2 N0 M0 Stage IB 76 R-Upper Not Applicable TCGA-05-4403 ce15f31f-2bad-4485-96fa-495bfa262e66 22 -27881 Not Applicable 0 578 Not Available Lung Adenocarcinoma Not Available Not Available Not Available Not Available Not Available Not Available Not Available Not Available Not Available Not Available MALE Lung Adenocarcinoma Mixed Subtype No C34.1 8255/3 C34.1 YES Not Available Not Available Not Available Not Available Not Available 7 Not Available Not Available Not Available Not Available Not Available Not Available Not Available Not Available Not Available Not Available Not Available No 4403 Not Available Not Available Not Available Not Available Not Available Not Available Not Available Not Available Not Available Not Available Not Available R0 Not Applicable Not Applicable Not Applicable Not Applicable Not Applicable Not Applicable Not Applicable Not Applicable Not Applicable Stage IB Not Applicable Not Applicable Not Applicable 5th Not Applicable Not Applicable Not Applicable M0 N0 T2 1975 NO YES 05 3 Lung Alive 2010 2006 Not Available

TCGA-86-A4P7 415 Alive T2a N0 M0 Stage IB 63 L-Lower Not Applicable TCGA-86-A4P7 9E640FF7-996E-427B-B56C-519A1E23BAAB 7 -23330 Not Applicable 0 14 Not Available Lung Adenocarcinoma Not Available 0 Not Available NO Not Available Not Available Not Available NO Not Available NOT HISPANIC OR LATINO Not Available Not Available Not Available Not Available TCGA-86-A4P7-F66092 575B0BCF-4A4C-489E-9FD7-5CF28FBD3A68 2 Not Applicable Not Applicable Not Applicable 415 Not Applicable 0 Scheduled Follow-up Submission Complete Remission/Response 100 NO 10 Not Available NO Other TUMOR FREE NO Complete Remission/Response Not Available NO Alive 2014 FEMALE Lung Adenocarcinoma- Not Otherwise Specified (NOS) No C34.3 8140/3 C34.3 YES 100 NO Not Available Not Available Peripheral Lung 2 Not Available Not Available Not Available Not Available Not Available Not Available Not Available Not Available Not Available Unknown Not Available No A4P7 Preoperative TUMOR FREE Not Available Not Available Unknown Not Available Not Available Complete Remission/Response NO WHITE Unknown R0 Not Applicable Not Applicable Not Applicable Not Applicable Not Applicable Not Applicable Not Applicable Not Applicable Not Applicable Stage IB Not Applicable Not Applicable Not Applicable 7th Not Applicable Not Applicable Not Applicable M0 N0 T2a Not Available YES NO 86 1 Lung Alive 2013 2012 Not Available

TCGA-95-A4VP 605 Alive T2b N2 M0 Stage IIIA 66 L-Lower Not Applicable TCGA-95-A4VP 9E22A3AF-7F28-4757-8684-ED64A2A27AD1 22 -24130 Not Applicable 0 168 Not Available Lung Adenocarcinoma Not Available TCGA-95-A4VP-D40540;TCGA-95-A4VP-D40543 EEC4E284-83E6-48D8-9E22-87A68963B598;B3A1D470-D0D9-4ABF-A3AF-1447CD0096B4 Not Available;Not Available 22;22 126;126 63;63 Carboplatin;Taxol Stable Disease;Stable Disease 2;2 Not Available;Not Available Not Available;Not Available Not Available;Not Available Not Available;Not Available Not Applicable;Not Applicable Not Available;Not Available Not Available;Not Available NO;NO Chemotherapy;Chemotherapy Not Available;Not Available Not Available;Not Available Not Available;Not Available NO;NO 2013;2013 Not Available Not Available NO Not Available Not Available Not Available NO Not Available Not Evaluated NO YES Not Available NO TCGA-95-A4VP-F58502 29D06D6D-2BAB-4F87-85B2-F4B369A1C6C4 11 Not Available Not Available Not Applicable 605 216 1 Scheduled Follow-up Submission Stable Disease Not Evaluated NO 4 Distant Metastasis YES Post-Adjuvant Therapy WITH TUMOR YES Stable Disease Biopsy with Histologic Confirmation;Convincing Imaging NO Alive 2014 FEMALE Lung Acinar Adenocarcinoma No C34.3 8550/3 C34.3 YES Not Available NO Not Available Not Available Not Available 2 Not Available Not Available Not Available Not Available Not Available Not Available Not Available Not Available Not Available NO 20 No A4VP Not Available TUMOR FREE Not Available Not Available YES Not Available Not Available Stable Disease NO WHITE NO Not Evaluated Not Applicable Not Applicable Not Applicable Not Applicable Not Applicable Not Applicable Not Applicable Not Applicable Not Applicable Stage IIIA Not Applicable Not Applicable Not Applicable 7th Not Applicable Not Applicable Not Applicable M0 N2 T2b 2012 YES NO 95 4 Lung Alive 2013 2012 1972

TCGA-NJ-A4YG 2261 Alive T2 N0 M0 Stage IB 65 L-Upper Not Applicable TCGA-NJ-A4YG 7FD03220-531E-4EF8-BECA-E9703E8C0AE1 26 -23812 Not Applicable 0 1726 Not Available Lung Adenocarcinoma Not Available Not Evaluated Not Available Not Available Not Available Not Available Not Available Not Available Not Available Not Evaluated Not Available;Not Available Not Available;Not Available Not Available;Not Available Not Available;Not Available TCGA-NJ-A4YG-F50865;TCGA-NJ-A4YG-F70643 C3CF3C86-3DA7-477A-A2DE-B89D114EE5BC;BF940C94-BC39-49DE-A9DF-843E7ED62019 1;27 Not Applicable;Not Applicable Not Applicable;Not Applicable Not Applicable;Not Applicable 1904;2261 Not Applicable;Not Applicable Not Available;Not Available Scheduled Follow-up Submission;Scheduled Follow-up Submission Stable Disease;Stable Disease Not Available;Not Available NO;NO 11;2 Not Available;Not Available NO;NO Not Available;Not Available TUMOR FREE;TUMOR FREE NO;NO Stable Disease;Stable Disease Not Available;Not Available NO;NO Alive;Alive 2013;2015 MALE Lung Adenocarcinoma- Not Otherwise Specified (NOS) No C34.1 8255/3 C34.1 YES Not Evaluated Not Available Not Available Not Available Unknown 4 Not Available Not Available Not Available Not Available Not Available Not Available Not Available Not Available Not Available NO 104 No A4YG Not Evaluated TUMOR FREE Not Available Not Available NO Not Available Not Available Not Applicable NO WHITE NO Not Evaluated Not Applicable Not Applicable Not Applicable Not Applicable Not Applicable Not Applicable Not Applicable Not Applicable Not Applicable Stage IB Not Applicable Not Applicable Not Applicable 6th Not Applicable Not Applicable Not Applicable M0 N0 T2 2007 NO YES NJ 4 Lung Alive 2013 2008 1955

TCGA-78-7155 1171 Dead T2 N0 M0 Stage IB 68 L-Upper Not Applicable TCGA-78-7155 fc79086c-af9a-4c27-b074-f7050b6f3381 28 -24863 1171 0 Not Available Not Available Lung Adenocarcinoma Not Available TCGA-78-7155-D16959;TCGA-78-7155-D17202 b699c307-543d-4e31-9adf-19275a9cb2ff;6db2eeea-88ce-495f-b14b-214400fff219 Not Available;Not Available 21;5 Not Available;253 253;253 Carboplatin;Gemcitabine Not Available;Not Available 9;10 2;2 Not Available;Not Available Not Available;Not Available OTHER, SPECIFY IN NOTES;OTHER, SPECIFY IN NOTES new primary;New Primary 1;1 IV;IV NO;NO Chemotherapy;Chemotherapy Not Available;Not Available Not Available;Not Available Not Available;Not Available Not Available;Not Available 2011;2011 0 Not Available NO Not Available Not Available Not Available NO Not Available Not Available YES YES NO NO TCGA-78-7155-F16958 8ede53b4-10e1-4371-b89d-c4f2423cda1b 28 Not Available Not Available 1171 Not Available 209 Not Available Scheduled Follow-up Submission Progressive Disease Not Available Not Available 9 Not Available YES Not Available Not Available Not Available Not Available Not Available Not Available Dead 2011 MALE Lung Adenocarcinoma- Not Otherwise Specified (NOS) No C34.1 8140/3 C34.1 YES Not Available NO Not Available Not Available Peripheral Lung 9 Not Available Not Available Not Available Not Available Not Available Not Available Not Available Not Available Not Available Not Available 48 No 7155 Preoperative Not Available Not Available Not Available Not Available Not Available Not Available Not Available Not Available WHITE Not Available Primary Tumor Field TCGA-78-7155-R16960 d46f3ed7-d9f7-4800-9aff-2d7226581603 1 28 314 Not Available Not Available 9 Not Available Not Available NO EXTERNAL BEAM Not Applicable OTHER, SPECIFY IN NOTES New Primary Not Available 2011 R0 Not Applicable Not Applicable Not Applicable Not Applicable Not Applicable Not Applicable Not Applicable Not Applicable Not Applicable Stage IB Not Applicable Not Applicable Not Applicable 6th Not Applicable Not Applicable Not Applicable M0 N0 T2 2005 NO YES 78 4 Lung Dead 2011 2005 1957

TCGA-75-6206 2590 Alive T2 N0 M0 Stage IB Not Available R-Upper Not Applicable TCGA-75-6206 c4c1469d-752f-42b6-8686-714ad4a8ac97 21 Not Available Not Applicable Not Available Not Available Not Available Lung Adenocarcinoma Not Available Not Available Not Available Not Available Not Available Not Available Not Available Not Available Not Available Not Available Not Available;Not Available Not Available;Not Available Not Available;Not Available Not Available;Not Available TCGA-75-6206-F15085;TCGA-75-6206-F72036 9fa30583-7256-4ed8-93ac-9cf0f6a928c0;DBEE9981-7853-4690-9B0B-62CBF6625199 21;2 Not Applicable;Not Applicable Not Applicable;Not Applicable Not Applicable;Not Applicable Not Available;2590 Not Applicable;Not Applicable 0;Not Evaluated Not Available;Scheduled Follow-up Submission Complete Remission/Response;Complete Remission/Response Not Available;Not Evaluated Not Available;NO 7;4 Not Available;Not Available NO;NO Adjuvant therapy;Not Available TUMOR FREE;TUMOR FREE NO;NO Complete Remission/Response;Complete Remission/Response Not Available;Not Available NO;NO Alive;Alive 2011;2015 MALE Lung Adenocarcinoma- Not Otherwise Specified (NOS) No C34.1 8140/3 C34.1 YES Not Available Not Available Not Available Not Available Peripheral Lung 7 Not Available Not Available Not Available Not Available Not Available Not Available Not Available Not Available Not Available Not Available 20 No 6206 Not Available TUMOR FREE Not Available Not Available Not Available Not Available Not Available Not Available Not Available Not Available Not Available R0 Not Applicable Not Applicable Not Applicable Not Applicable Not Applicable Not Applicable Not Applicable Not Applicable Not Applicable Stage IB Not Applicable Not Applicable Not Applicable 6th Not Applicable Not Applicable Not Applicable M0 N0 T2 1962 NO YES 75 3 Lung Alive 2011 Not Available Not Available

TCGA-55-7284 243 Dead T3 N0 MX Stage IIB 74 R-Lower Not Applicable TCGA-55-7284 0b15fcb8-1fb7-497e-80b9-d3db66a3c0a2 13 -27087 Not Applicable 0 121 Not Available Lung Adenocarcinoma 89 Not Available Not Available Not Available Not Available Not Available Not Available Not Available Not Available NOT HISPANIC OR LATINO NO NO NO NO TCGA-55-7284-F61486 411282A6-43D3-468C-8DD9-47C991653F13 30 Not Available Not Available 243 Not Available 231 Not Evaluated Scheduled Follow-up Submission Progressive Disease Not Evaluated NO 6 Distant Metastasis YES Not Available WITH TUMOR NO Complete Remission/Response Convincing Imaging NO Dead 2014 MALE Lung Bronchioloalveolar Carcinoma Nonmucinous No C34.8 8252/3 C34.8 YES Not Available Not Available Not Available Not Available Not Available 6 Not Available Not Available Not Available Not Available Not Available Not Available Not Available Not Available Not Available NO Not Available Yes, History of Synchronous/Bilateral Malignancy 7284 Not Available TUMOR FREE 102 70 Unknown 108 74 Unknown YES WHITE Unknown R0 Not Applicable Not Applicable Not Applicable Not Applicable Not Applicable Not Applicable Not Applicable Not Applicable Not Applicable Stage IIB Not Applicable Not Applicable Not Applicable 7th Not Applicable Not Applicable Not Applicable MX N0 T3 Not Available YES NO 55 3 Lung Alive 2012 2011 Not Available

TCGA-86-7701 947 Alive T2 N0 M1 Stage IV 66 R-Upper Not Applicable TCGA-86-7701 a3f6bc1c-19ab-4eeb-a9ac-3d2fac850bde 18 -24209 Not Applicable 0 11 Not Available Lung Adenocarcinoma Not Available TCGA-86-7701-D36483;TCGA-86-7701-D36484;TCGA-86-7701-D58402;TCGA-86-7701-D58403;TCGA-86-7701-D58404;TCGA-86-7701-D58405;TCGA-86-7701-D58407;TCGA-86-7701-D58408 EC1417C7-2349-4F98-939F-E7E643F30F87;24A17B71-0E0B-4803-A21C-02BCB336024D;08D438DF-4B04-42CE-92EB-6AAC1EF6952E;7741F434-FD67-473C-B872-5A2B21734ED8;444CAEFF-EDEE-460D-9C88-C49C232A90DB;D7831CC1-B080-4F51-A2C1-3A32B60AA83F;8C75D7CE-9FC9-4BFF-B261-DD159CFED86F;DC37E270-9E05-421E-A7F4-8B20DF86DC92 Not Available;Not Available;Tyrosine kinase inhibitor;Not Available;Not Available;Not Available;Not Available;Not Available 31;31;9;9;9;9;9;9 207;207;601;601;601;Not Available;Not Available;Not Available 27;27;454;454;454;706;936;936 Etoposide;Cisplatin;Not Available;Docetaxel;Zoledronic acid;Zoledronic acid;Gemcitabine;Docetaxel Complete Response;Complete Response;Stable Disease;Stable Disease;Stable Disease;Not Applicable;Not Applicable;Not Applicable 10;10;4;4;4;4;4;4 Not Available;Not Available;Not Available;Not Available;Not Available;Not Available;Not Available;Not Available Not Available;Not Available;Not Available;Not Available;Not Available;Not Available;Not Available;Not Available Not Available;Not Available;Not Available;Not Available;Not Available;Not Available;Not Available;Not Available Not Available;Not Available;Not Available;Not Available;Not Available;Not Available;Not Available;Not Available Not Applicable;Not Applicable;Not Applicable;Not Applicable;Not Applicable;Not Applicable;Not Applicable;Not Applicable Not Available;Not Available;Not Available;Not Available;Not Available;Not Available;Not Available;Not Available Not Available;Not Available;Not Available;Not Available;Not Available;Not Available;Not Available;Not Available NO;NO;NO;NO;NO;YES;YES;YES Chemotherapy;Chemotherapy;Chemotherapy;Chemotherapy;Ancillary;Ancillary;Chemotherapy;Chemotherapy Not Available;Not Available;Not Available;Not Available;Not Available;Not Available;Not Available;Not Available Not Available;Not Available;Not Available;Not Available;Not Available;Not Available;Not Available;Not Available Not Available;Not Available;Not Available;Not Available;Not Available;Not Available;Not Available;Not Available NO;NO;YES;NO;NO;NO;NO;NO 2012;2012;2014;2014;2014;2014;2014;2014 2 Not Available Not Available Not Available Not Available Not Available Not Available Not Available NOT HISPANIC OR LATINO YES;YES NO;YES Not Available;Not Available NO;NO TCGA-86-7701-F36476;TCGA-86-7701-F58406 277C6CF8-C341-45A1-AC77-78CA122BDD86;212F60F8-9A3F-4256-BC3A-C5150373B1E7 31;9 Not Available;Not Available Not Available;Not Available Not Applicable;Not Applicable 474;947 424;900 0;2 Scheduled Follow-up Submission;Scheduled Follow-up Submission Stable Disease;Progressive Disease 100;80 NO;NO 10;4 Distant Metastasis;Distant Metastasis YES;YES Post-Adjuvant Therapy;Post-Adjuvant Therapy WITH TUMOR;WITH TUMOR YES;YES Complete Remission/Response;Complete Remission/Response Convincing Imaging;Not Available NO;NO Alive;Alive 2012;2014 MALE Lung Adenocarcinoma- Not Otherwise Specified (NOS) No C34.1 8140/3 C34.1 YES 80 Not Available Not Available Not Available Peripheral Lung 1 Not Available Not Available Not Available Not Available Not Available Not Available Not Available Not Available Not Available Not Available Not Available No 7701 Not Available TUMOR FREE Not Available Not Available Not Available Not Available Not Available Not Available NO WHITE Not Available Distant Recurrence TCGA-86-7701-R58409 248F5702-58CA-4E1B-BC44-BCAC81B476C1 Not Available 9 929 917 Radiographic Progressive Disease 4 13 29 NO External Not Applicable Not Available Not Available Gy 2014 R0 Not Applicable Not Applicable Not Applicable Not Applicable Not Applicable Not Applicable Not Applicable Not Applicable Not Applicable Stage IV Not Applicable Not Applicable Not Applicable 7th Not Applicable Not Applicable Not Applicable M1 N0 T2 Not Available YES NO 86 1 Lung Alive 2012 2011 Not Available

TCGA-MP-A4TC 74 Dead T1 N2 M0 Stage IIIA 77 R-Middle Not Applicable TCGA-MP-A4TC 99C35245-E1C8-4805-B7A5-F703BB506030 2 -28471 74 0 Not Available Not Available Lung Adenocarcinoma 68 TCGA-MP-A4TC-D41397;TCGA-MP-A4TC-D41398 2D936FD6-139F-4EBD-9EAB-D843254E987A;ED1C314A-2C4D-4A19-A6A2-0FB17145C3E5 Not Available;Not Available 18;18 25;25 25;25 Cisplatin;Navelbine Complete Response;Complete Response 3;3 Not Available;Not Available Not Available;Not Available Not Available;Not Available Not Available;Not Available Not Applicable;Not Applicable Not Available;Not Available Not Available;Not Available NO;NO Chemotherapy;Chemotherapy Not Available;Not Available Not Available;Not Available Not Available;Not Available NO;NO 2013;2013 Not Evaluated Not Available NO Not Available Not Available Not Available NO Not Available NOT HISPANIC OR LATINO MALE Lung Adenocarcinoma- Not Otherwise Specified (NOS) No C34.2 8140/3 C34.2 YES Not Evaluated NO Not Available Not Available Unknown 4 Not Available Not Available Not Available Not Available Not Available Not Available Not Available Not Available Not Available NO 125 Yes, History of Prior Malignancy A4TC Not Evaluated TUMOR FREE Not Available 76 YES Not Available 83 Complete Remission/Response YES WHITE NO R0 Not Applicable Not Applicable Not Applicable Not Applicable Not Applicable Not Applicable Not Applicable Not Applicable Not Applicable Stage IIIA Not Applicable Not Applicable Not Applicable 6th Not Applicable Not Applicable Not Applicable M0 N2 T1 1976 NO YES MP 3 Lung Dead 2013 2008 1944

TCGA-86-8672 19 Dead T3 N0 M0 Stage IIB 59 R-Upper Not Applicable TCGA-86-8672 c0e263eb-1a83-4dc8-8abe-3dd2a59bae1b 31 -21682 Not Applicable 0 15 Not Available Lung Adenocarcinoma Not Available Unknown Not Available Not Available Not Available Not Available Not Available Not Available Not Available NOT HISPANIC OR LATINO Not Available Not Available Not Available Not Available TCGA-86-8672-F46401 892123F6-665F-4DAC-96FE-4E45BD22A9D6 7 Not Applicable Not Applicable 19 Not Available Not Applicable Not Evaluated Scheduled Follow-up Submission Unknown Not Evaluated NO 8 Not Available NO Not Evaluated Unknown NO Complete Remission/Response Not Available NO Dead 2013 MALE Lung Adenocarcinoma- Not Otherwise Specified (NOS) No C34.1 8140/3 C34.1 YES Unknown NO Not Available Not Available Central Lung 8 Not Available Not Available Not Available Not Available Not Available Not Available Not Available Not Available Not Available Unknown Not Available No 8672 Unknown TUMOR FREE Not Available Not Available Unknown Not Available Not Available Unknown NO WHITE Unknown R0 Not Applicable Not Applicable Not Applicable Not Applicable Not Applicable Not Applicable Not Applicable Not Applicable Not Applicable Stage IIB Not Applicable Not Applicable Not Applicable 7th Not Applicable Not Applicable Not Applicable M0 N0 T3 Not Available YES NO 86 1 Lung Alive 2012 2012 Not Available

TCGA-86-8280 701 Alive T2b N0 M0 Stage IIA 54 L-Lower Not Applicable TCGA-86-8280 d8faa3a7-6b3f-4e69-8c88-184e41055bd7 30 -19977 Not Applicable 0 16 Not Available Lung Adenocarcinoma Not Available TCGA-86-8280-D66080;TCGA-86-8280-D66081 7B11F24A-04FA-472B-A420-D8E1764C3600;2895FB6B-3FC1-4DFD-B743-1CB915268336 Not Available;Not Available 2;2 129;129 16;16 Cisplatin;Etoposide Complete Response;Complete Response 10;10 Not Available;Not Available Not Available;Not Available Not Available;Not Available Not Available;Not Available Not Applicable;Not Applicable Not Available;Not Available Not Available;Not Available NO;NO Chemotherapy;Chemotherapy Not Available;Not Available Not Available;Not Available Not Available;Not Available NO;NO 2014;2014 Not Available Not Available Not Available Not Available Not Available Not Available Not Available Not Available NOT HISPANIC OR LATINO Not Available Not Available Not Available Not Available TCGA-86-8280-F66079 F949DB0E-42F2-40F9-B663-70562177DC57 2 Not Applicable Not Applicable Not Applicable 701 Not Applicable 0 Scheduled Follow-up Submission Complete Remission/Response 100 NO 10 Not Available NO Other TUMOR FREE YES Complete Remission/Response Not Available NO Alive 2014 FEMALE Lung Bronchioloalveolar Carcinoma Nonmucinous No C34.3 8252/3 C34.3 YES Not Available NO Not Available Not Available Peripheral Lung 5 Not Available Not Available Not Available Not Available Not Available Not Available Not Available Not Available Not Available NO Not Available No 8280 Not Available TUMOR FREE Not Available Not Available Unknown Not Available Not Available Complete Remission/Response NO WHITE Unknown R0 Not Applicable Not Applicable Not Applicable Not Applicable Not Applicable Not Applicable Not Applicable Not Applicable Not Applicable Stage IIA Not Applicable Not Applicable Not Applicable 7th Not Applicable Not Applicable Not Applicable M0 N0 T2b Not Available YES NO 86 1 Lung Alive 2012 2011 Not Available

TCGA-50-6597 1268 Dead T2 N0 M0 Stage IB 79 R-Lower Not Applicable TCGA-50-6597 0d66bf6c-eed0-4726-bd5b-3bf6d610b4e0 26 -29195 Not Applicable 0 1015 Not Available Lung Adenocarcinoma Not Available Not Available Not Available YES Not Available Not Available Not Available Not Available Not Available NOT HISPANIC OR LATINO Not Available Not Available Not Available Not Available TCGA-50-6597-F44134 FC850BDD-7D70-423E-97AE-7FE982CCC259 13 Not Applicable Not Applicable 1268 Not Available Not Applicable Not Available Scheduled Follow-up Submission Complete Remission/Response Not Available NO 6 Not Available NO Not Available TUMOR FREE NO Partial Remission/Response Not Available NO Dead 2013 FEMALE Lung Adenocarcinoma- Not Otherwise Specified (NOS) No C34.3 8140/3 C34.3 YES Not Available YES NO Not Available Not Available 8 Not Available Not Available Not Available Not Available Not Available Not Available Not Available Not Available Not Available Not Available Not Available No 6597 Not Available TUMOR FREE Not Available Not Available Not Available Not Available Not Available Not Available NO WHITE Not Available R0 Not Applicable Not Applicable Not Applicable Not Applicable Not Applicable Not Applicable Not Applicable Not Applicable Not Applicable Stage IB Not Applicable Not Applicable Not Applicable 6th Not Applicable Not Applicable Not Applicable M0 N0 T2 Not Available NO YES 50 1 Lung Alive 2011 2007 Not Available

TCGA-05-4245 730 Alive T2 N2 M0 Stage IIIA 81 L-Upper Not Applicable TCGA-05-4245 03d09c05-49ab-4ba6-a8d7-e7ccf71fafd2 22 -29647 Not Applicable 0 395 Not Available Lung Adenocarcinoma Not Available Not Available Not Available Not Available Not Available Not Available Not Available Not Available Not Available Not Available NO NO Not Available Not Available TCGA-05-4245-F36308 8B6363A9-C846-48D4-8051-2A0B97BAD585 29 Not Available Not Available Not Applicable 730 334 Not Available Scheduled Follow-up Submission Complete Remission/Response Not Available NO 10 Not Available YES Not Available TUMOR FREE NO Not Applicable Not Available NO Alive 2012 MALE Lung Adenocarcinoma- Not Otherwise Specified (NOS) No C34.1 8140/3 C34.1 YES Not Available Not Available Not Available Not Available Not Available 7 Not Available Not Available Not Available Not Available Not Available Not Available Not Available Not Available Not Available Not Available 32 Yes 4245 Not Available TUMOR FREE Not Available Not Available Not Available Not Available Not Available Not Available Not Available Not Available Not Available R2 Not Applicable Not Applicable Not Applicable Not Applicable Not Applicable Not Applicable Not Applicable Not Applicable Not Applicable Stage IIIA Not Applicable Not Applicable Not Applicable 6th Not Applicable Not Applicable Not Applicable M0 N2 T2 Not Available NO YES 05 4 Lung Alive 2010 2009 Not Available

TCGA-J2-A4AE 1079 Alive T1a N0 MX Stage IA 77 L-Upper Not Applicable TCGA-J2-A4AE E5F956DD-F49F-435C-83C3-5B1B0A2050ED 27 -28163 Not Applicable 0 282 Not Available Lung Adenocarcinoma 89 Not Available Not Available NO Not Available Not Available Not Available NO Not Available NOT HISPANIC OR LATINO Not Available;Not Available Not Available;Not Available Not Available;Not Available Not Available;Not Available TCGA-J2-A4AE-F55522;TCGA-J2-A4AE-F70639 3A67D1E9-45FD-41D0-8E79-F27D9B40AE3A;F091573D-78D1-4263-B744-6928E374086D 8;27 Not Applicable;Not Applicable Not Applicable;Not Applicable Not Applicable;Not Applicable 671;1079 Not Applicable;Not Applicable Not Available;Not Available Scheduled Follow-up Submission;Scheduled Follow-up Submission Complete Remission/Response;Complete Remission/Response Not Available;Not Available NO;NO 1;2 Not Available;Not Available NO;NO Not Available;Not Available TUMOR FREE;TUMOR FREE NO;NO Complete Remission/Response;Complete Remission/Response Not Available;Not Available NO;NO Alive;Alive 2014;2015 FEMALE Lung Adenocarcinoma- Not Otherwise Specified (NOS) No C34.1 8140/3 C34.1 YES Not Available NO Not Available Not Available Not Available 12 Not Available Not Available Not Available Not Available Not Available Not Available Not Available Not Available Not Available NO Not Available No A4AE Not Available TUMOR FREE Not Available Not Available NO 0.72 92 Not Applicable YES WHITE NO R0 Not Applicable Not Applicable Not Applicable Not Applicable Not Applicable Not Applicable Not Applicable Not Applicable Not Applicable Stage IA Not Applicable Not Applicable Not Applicable 7th Not Applicable Not Applicable Not Applicable MX N0 T1a Not Available YES NO J2 1 Lung Alive 2012 2012 Not Available

TCGA-44-7660 592 Alive T2 N0 MX Stage IB 72 R-Upper Not Applicable TCGA-44-7660 a534b96b-1665-444a-9308-e610f2f0e510 22 -26594 Not Applicable 0 162 Not Available Lung Adenocarcinoma 80 TCGA-44-7660-D32371 452ec58f-55a9-4b79-9095-1800bc34f9a8 ASCI 24 141 78 recPRAME+AS15 ASCI Clinical Progressive Disease 5 Not Available Not Available Not Available Not Available Not Applicable Not Available Not Available NO Vaccine Not Available Not Available Not Available YES 2012 1 Not Available Not Available Not Available Not Available Not Available Not Available Not Available NOT HISPANIC OR LATINO NO;Not Available NO;Not Available NO;NO Not Available;Not Available TCGA-44-7660-F32361;TCGA-44-7660-F40409 57f9b0eb-17e0-4b6b-92a0-407511f014d9;4B16E925-6686-4721-B792-96761A62E44C 24;19 Not Available;Not Applicable Not Available;Not Applicable Not Applicable;Not Applicable 325;592 253;Not Applicable Not Available;Not Available Scheduled Follow-up Submission;Scheduled Follow-up Submission Progressive Disease;Stable Disease Not Available;70 NO;NO 5;2 Locoregional Recurrence;Not Available YES;NO Not Available;Other WITH TUMOR;WITH TUMOR YES;YES Progressive Disease;Progressive Disease Convincing Imaging;Not Available NO;NO Alive;Alive 2012;2013 MALE Lung Adenocarcinoma- Not Otherwise Specified (NOS) No C34.1 8140/3 C34.1 YES Not Available NO Not Available Not Available Not Available 12 Not Available Not Available Not Available Not Available Not Available Not Available Not Available Not Available Not Available Not Available 124 No 7660 Pre-Adjuvant Therapy TUMOR FREE 95 94 Not Available 92 83 Not Available YES WHITE Not Available Not Available Not Applicable Not Applicable Not Applicable Not Applicable Not Applicable Not Applicable Not Applicable Not Applicable Not Applicable Stage IB Not Applicable Not Applicable Not Applicable 7th Not Applicable Not Applicable Not Applicable MX N0 T2 Not Available YES NO 44 2 Lung Alive 2011 2011 1949

TCGA-86-A4P8 805 Alive T1b N2 MX Stage IIIA 59 R-Lower Not Applicable TCGA-86-A4P8 646910EE-5F5D-40AC-A21A-75790F89430A 7 -21738 Not Applicable 0 39 Not Available Lung Adenocarcinoma 85 TCGA-86-A4P8-D48293;TCGA-86-A4P8-D48295 C22B8623-E5C2-4F22-8CCD-7859A4802584;054E5CD2-940A-4364-A458-39387314D2AE Not Available;Not Available 11;11 106;106 43;43 Cisplatin;Alimta Complete Response;Complete Response 9;9 Not Available;Not Available Not Available;Not Available Not Available;Not Available Not Available;Not Available Not Applicable;Not Applicable Not Available;Not Available Not Available;Not Available NO;NO Chemotherapy;Chemotherapy Not Available;Not Available Not Available;Not Available Not Available;Not Available NO;NO 2013;2013 Unknown Not Available YES Not Available Not Available FISH YES Not Available NOT HISPANIC OR LATINO Not Available;Not Available Not Available;Not Available Not Available;Not Available Not Available;Not Available TCGA-86-A4P8-F48289;TCGA-86-A4P8-F65331 34D83F16-C390-4C4F-882D-B34483F55C29;F8D137C4-C479-4576-A4AB-6F01C2973E15 11;22 Not Applicable;Not Applicable Not Applicable;Not Applicable Not Applicable;Not Applicable 431;805 Not Applicable;Not Applicable 0;0 Scheduled Follow-up Submission;Scheduled Follow-up Submission Complete Remission/Response;Complete Remission/Response Unknown;Unknown NO;NO 9;9 Not Available;Not Available NO;NO Post-Adjuvant Therapy;Post-Adjuvant Therapy TUMOR FREE;TUMOR FREE YES;YES Unknown;Complete Remission/Response Not Available;Not Available YES;YES Alive;Alive 2013;2014 FEMALE Lung Adenocarcinoma- Not Otherwise Specified (NOS) No C34.3 8140/3 C34.3 YES Unknown NO Not Available Not Available Unknown 2 Not Available Not Available Not Available Not Available Not Available Not Available Not Available Not Available Not Available Unknown Not Available No A4P8 Unknown Unknown 78 89 Unknown 74 84 Complete Remission/Response YES WHITE Unknown Primary Tumor Field TCGA-86-A4P8-R48298 09736DC5-00BA-4D11-9400-13848477B168 Not Available 11 186 145 Complete Response 9 28 50.4 NO External Not Applicable Not Available Not Available Gy 2013 Not Evaluated Not Applicable Not Applicable Not Applicable Not Applicable Not Applicable Not Applicable Not Applicable Not Applicable Not Applicable Stage IIIA Not Applicable Not Applicable Not Applicable 7th Not Applicable Not Applicable Not Applicable MX N2 T1b Not Available YES NO 86 1 Lung Alive 2013 2012 Not Available

TCGA-93-8067 186 Alive T2a N0 MX Stage IB 77 L-Lower Not Applicable TCGA-93-8067 bbe88801-34f3-46d2-bbfd-b46c3901ed71 18 -28453 Not Applicable 0 186 Not Available Lung Adenocarcinoma 47 2 Not Available NO Not Available Not Available Not Available NO Not Available NOT HISPANIC OR LATINO Not Available Not Available Not Available Not Available TCGA-93-8067-F32193 88DF8B42-F107-47B4-84A7-2E62FE30E870 18 Not Applicable Not Applicable Not Applicable 186 Not Applicable 2 Scheduled Follow-up Submission Complete Remission/Response 80 NO 5 Not Available NO Preoperative TUMOR FREE NO Complete Remission/Response Not Available NO Alive 2012 MALE Lung Adenocarcinoma- Not Otherwise Specified (NOS) No C34.3 8140/3 C34.3 YES 80 NO Not Available Not Available Peripheral Lung 5 Not Available Not Available Not Available Not Available Not Available Not Available Not Available Not Available Not Available NO 20 Yes, History of Prior Malignancy 8067 Preoperative TUMOR FREE 94 103 NO 89 94 Complete Remission/Response YES ASIAN NO RX Not Applicable Not Applicable Not Applicable Not Applicable Not Applicable Not Applicable Not Applicable Not Applicable Not Applicable Stage IB Not Applicable Not Applicable Not Applicable 7th Not Applicable Not Applicable Not Applicable MX N0 T2a 2001 YES NO 93 4 Lung Alive 2012 2011 1961

TCGA-49-6743 1621 Alive T1 N2 MX Stage IIIA 81 R-Upper Not Applicable TCGA-49-6743 a391d49f-a822-460b-981c-6fbe1868ee38 10 -29807 Not Applicable 0 369 Not Available Lung Adenocarcinoma Not Available TCGA-49-6743-D14859;TCGA-49-6743-D16685;TCGA-49-6743-D16684 77afb595-62de-41ce-bbd0-3e811685cfcf;faeee125-10d2-46c8-81cc-75ade012acdb;586c3a98-5004-43da-832f-73b1ec351173 Not Available;Not Available;Not Available 10;23;23 140;140;140 18;48;48 Cisplatin;Bevacizumab;Pemetrexed Not Available;Not Available;Not Available 8;9;9 4;4;4 Not Available;Not Available;Not Available Not Available;Not Available;Not Available ADJUVANT;ADJUVANT;ADJUVANT Not Applicable;Not Applicable;Not Applicable 1;1;1 IV;IV;IV NO;NO;NO Chemotherapy;Chemotherapy;Chemotherapy Not Available;Not Available;Not Available Not Available;Not Available;Not Available Not Available;Not Available;Not Available Not Available;Not Available;Not Available 2011;2011;2011 Not Available Not Available Not Available Not Available Not Available Not Available Not Available Not Available Not Available Not Available;Not Available Not Available;Not Available Not Available;Not Available Not Available;Not Available TCGA-49-6743-F14858;TCGA-49-6743-F71136 cd1e65ca-466c-495d-b733-aac17946d06a;74E45F53-1B39-45A5-A07E-4B3BB42BC37E 10;18 Not Applicable;Not Applicable Not Applicable;Not Applicable Not Applicable;Not Applicable 369;1621 Not Applicable;Not Applicable Not Available;Not Available Scheduled Follow-up Submission;Scheduled Follow-up Submission Complete Remission/Response;Complete Remission/Response Not Available;100 Not Available;NO 8;3 Not Available;Not Available NO;NO Not Available;Not Available TUMOR FREE;Not Available YES;YES Complete Remission/Response;Complete Remission/Response Not Available;Not Available NO;NO Alive;Alive 2011;2015 FEMALE Lung Clear Cell Adenocarcinoma No C34.1 8310/3 C34.1 YES Not Available Not Available Not Available Not Available Not Available 8 Not Available Not Available Not Available Not Available Not Available Not Available Not Available Not Available Not Available Not Available 50 No 6743 Not Available TUMOR FREE Not Available Not Available Not Available Not Available Not Available Not Available Not Available WHITE Not Available Not Available Not Applicable Not Applicable Not Applicable Not Applicable Not Applicable Not Applicable Not Applicable Not Applicable Not Applicable Stage IIIA Not Applicable Not Applicable Not Applicable 7th Not Applicable Not Applicable Not Applicable MX N2 T1 Not Available NO YES 49 4 Lung Alive 2011 2010 Not Available

TCGA-62-8395 1216 Alive T3 N0 M0 Stage IIB 80 R-Upper Not Applicable TCGA-62-8395 5c266025-e590-457c-af86-4c2dc9797267 4 -29311 Not Applicable 0 705 Not Available Lung Adenocarcinoma Not Available 0 Not Available YES Not Available Not Available Not Available NO Not Available NOT HISPANIC OR LATINO YES NO NO Not Available TCGA-62-8395-F42099 2705FAEA-A87F-4437-8A72-3CD9216FC451 15 Not Available Not Available Not Applicable 1216 395 0 Scheduled Follow-up Submission Progressive Disease 100 NO 5 Locoregional Recurrence YES Preoperative WITH TUMOR NO Complete Remission/Response Convincing Imaging NO Alive 2013 FEMALE Lung Adenocarcinoma Mixed Subtype No C34.1 8255/3 C34.1 YES 100 NO Not Available Not Available Central Lung 7 YES NO 500 NO Not Available NO Not Available Locoregional Recurrence;Distant Metastasis Convincing Imaging YES Not Available No 8395 Preoperative WITH TUMOR Not Available Not Available NO 86 130 Complete Remission/Response YES WHITE NO R0 Not Applicable Not Applicable Not Applicable Not Applicable Not Applicable Not Applicable Not Applicable Not Applicable Not Applicable Stage IIB Not Applicable Not Applicable Not Applicable 6th Not Applicable Not Applicable Not Applicable M0 N0 T3 Not Available NO YES 62 1 Lung Alive 2012 2009 Not Available

TCGA-78-7149 3940 Alive T4 N0 M0 Stage IIIB 71 R-Lower Not Applicable TCGA-78-7149 2de1ba51-eae1-4a54-b7cd-d6d739fdc39d 3 -26285 Not Applicable 0 1093 Not Available Lung Adenocarcinoma Not Available 1 Not Available NO Not Available Not Available Not Available NO Not Available Not Available Not Available;Not Available Not Available;Not Available Not Available;Not Available Not Available;Not Available TCGA-78-7149-F17123;TCGA-78-7149-F46148 95813dc5-e402-4f0b-a425-2578fff0d0c2;20D89F59-36DA-4A8A-A462-4A3DA7D3CF50 3;23 Not Available;Not Available Not Available;Not Available Not Applicable;Not Applicable 1093;3940 Not Available;Not Available Not Available;Not Available Scheduled Follow-up Submission;Scheduled Follow-up Submission Not Available;Not Available Not Available;Not Available Not Available;NO 10;8 Not Available;Not Available Not Available;Unknown Not Available;Not Available Not Available;Not Available NO;NO Complete Remission/Response;Complete Remission/Response Not Available;Not Available NO;NO Alive;Alive 2011;2013 MALE Lung Adenocarcinoma Mixed Subtype No C34.3 8255/3 C34.3 YES Not Available NO Not Available Not Available Central Lung 10 Not Available Not Available Not Available Not Available Not Available Not Available Not Available Not Available Not Available Not Available 115 No 7149 Preoperative Not Available Not Available Not Available Not Available Not Available Not Available Not Available NO WHITE Not Available R0 Not Applicable Not Applicable Not Applicable Not Applicable Not Applicable Not Applicable Not Applicable Not Applicable Not Applicable Stage IIIB Not Applicable Not Applicable Not Applicable 6th Not Applicable Not Applicable Not Applicable M0 N0 T4 Not Available NO YES 78 2 Lung Alive 2011 2002 1950

TCGA-38-4631 354 Dead T2 N0 M0 Stage IB 72 L-Upper Not Applicable TCGA-38-4631 2483621a-4db3-41ab-aa33-b9427ea8a0af 14 -26538 354 0 Not Available Not Available Lung Adenocarcinoma Not Available Not Available Not Available NO Not Available Not Available Not Available NO Not Available NOT HISPANIC OR LATINO NO YES NO NO TCGA-38-4631-F4892 48e7462e-19a5-4897-9104-e131fa220329 14 Not Available Not Available 354 Not Available Not Available Not Available Not Available Not Available Not Available Not Available 12 Not Available YES Not Available WITH TUMOR NO Progressive Disease Not Available NO Dead 2010 FEMALE Lung Adenocarcinoma- Not Otherwise Specified (NOS) No C34.1 8140/3 C34.1 YES Not Available NO Not Available Not Available Peripheral Lung 12 Not Available Not Available Not Available Not Available Not Available Not Available Not Available Not Available Not Available Not Available 40 Yes 4631 Not Available WITH TUMOR Not Available Not Available Not Available Not Available Not Available Not Available Not Available WHITE Not Available Distant site TCGA-38-4631-R4893 adf6e9ab-7efe-43bf-83e4-ae864d19e186 1 14 115 87 Not Available 12 Not Available Not Available NO EXTERNAL BEAM Not Applicable PALLIATIVE Not Available Not Available 2010 R0 Not Applicable Not Applicable Not Applicable Not Applicable Not Applicable Not Applicable Not Applicable Not Applicable Not Applicable Stage IB Not Applicable Not Applicable Not Applicable Not Available Not Applicable Not Applicable Not Applicable M0 N0 T2 Not Available NO YES 38 4 Lung Dead 2010 1997 Not Available

TCGA-55-8614 536 Alive T2a N0 MX Stage IB 76 L-Upper Not Applicable TCGA-55-8614 a8de1beb-6296-44eb-bd63-c603f70faaf3 11 -27924 Not Applicable 0 41 Not Available Lung Adenocarcinoma 113 Not Evaluated Not Available Unknown Not Available Not Available Not Available Unknown Not Available NOT HISPANIC OR LATINO Not Available Not Available Not Available Not Available TCGA-55-8614-F47843 FC34AC51-73E6-4D1F-9BBE-658B99CDD4D5 29 Not Applicable Not Applicable Not Applicable 536 Not Applicable Not Evaluated Scheduled Follow-up Submission Complete Remission/Response Not Evaluated NO 8 Not Available NO Not Evaluated TUMOR FREE NO Complete Remission/Response Not Available NO Alive 2013 MALE Lung Adenocarcinoma- Not Otherwise Specified (NOS) No C34.1 8140/3 C34.1 YES Not Evaluated Unknown Not Available Not Available Unknown 1 Not Available Not Available Not Available Not Available Not Available Not Available Not Available Not Available Not Available NO 40 No 8614 Not Evaluated TUMOR FREE Not Available Not Available NO Not Available 94 Complete Remission/Response YES WHITE NO R0 Not Applicable Not Applicable Not Applicable Not Applicable Not Applicable Not Applicable Not Applicable Not Applicable Not Applicable Stage IB Not Applicable Not Applicable Not Applicable 7th Not Applicable Not Applicable Not Applicable MX N0 T2a 1995 YES NO 55 3 Lung Alive 2013 2011 Not Available

TCGA-49-AARO 3759 Alive T1a N0 MX Stage IA 39 L-Upper Not Applicable TCGA-49-AARO 5202458B-C38A-47C0-A0BA-5F1A59A762A0 19 -14527 Not Applicable 0 3755 Not Available Lung Adenocarcinoma 103.3 1 Not Available Not Available Not Available Not Available Not Available Not Available Not Available NOT HISPANIC OR LATINO YES NO NO Not Available TCGA-49-AARO-F70571 FC43C360-D46F-4775-B16A-C5739011E368 25 Not Available Not Available Not Applicable 3759 3521 3 Scheduled Follow-up Submission Stable Disease 40 NO 2 Locoregional Recurrence YES Post-Adjuvant Therapy WITH TUMOR YES Complete Remission/Response Convincing Imaging YES Alive 2015 FEMALE Lung Papillary Adenocarcinoma No C34.1 8260/3 C34.1 YES 90 Not Available Not Available Not Available Not Available 6 NO NO 1144 YES 3664 Not Available Not Available Locoregional Recurrence Convincing Imaging YES Not Available No AARO Preoperative WITH TUMOR 84.4 83.9 NO 83.6 83.0 Complete Remission/Response YES BLACK OR AFRICAN AMERICAN NO R0 Not Applicable Not Applicable Not Applicable Not Applicable Not Applicable Not Applicable Not Applicable Not Applicable Not Applicable Stage IA Not Applicable Not Applicable Not Applicable 6th Not Applicable Not Applicable Not Applicable MX N0 T1a Not Available NO YES 49 2 Lung Alive 2014 2003 Not Available

TCGA-05-4389 1369 Alive T1 N0 M0 Stage IA 70 R-Upper Not Applicable TCGA-05-4389 a3de401d-91fe-49a2-bb07-81c1a06506e6 22 -25660 Not Applicable 0 1369 Not Available Lung Adenocarcinoma Not Available Not Available Not Available Not Available Not Available Not Available Not Available Not Available Not Available Not Available Not Available Not Available Not Available Not Available TCGA-05-4389-F36455 A2734A4F-5C1D-4D7B-B01D-01305FF9E917 31 Not Applicable Not Applicable Not Applicable 1369 Not Applicable Not Available Scheduled Follow-up Submission Complete Remission/Response Not Available NO 10 Not Available NO Not Available TUMOR FREE NO Not Applicable Not Available NO Alive 2012 MALE Lung Adenocarcinoma Mixed Subtype No C34.1 8255/3 C34.1 YES Not Available Not Available Not Available Not Available Not Available 7 Not Available Not Available Not Available Not Available Not Available Not Available Not Available Not Available Not Available Not Available 43 No 4389 Not Available TUMOR FREE Not Available Not Available Not Available Not Available Not Available Not Available Not Available Not Available Not Available R0 Not Applicable Not Applicable Not Applicable Not Applicable Not Applicable Not Applicable Not Applicable Not Applicable Not Applicable Stage IA Not Applicable Not Applicable Not Applicable 5th Not Applicable Not Applicable Not Applicable M0 N0 T1 Not Available NO YES 05 3 Lung Alive 2010 2005 Not Available

TCGA-69-7761 186 Alive T2a N0 MX Stage IB 84 L-Lower Not Applicable TCGA-69-7761 77828a90-8e2a-4664-b674-d837e6e34fe4 13 -31041 Not Applicable 0 186 Not Available Lung Adenocarcinoma 80 TCGA-69-7761-D29256;TCGA-69-7761-D29253 15c9dc00-5f02-401f-96c4-e5098a9d7f91;827ec9b8-ee79-4f14-8cb1-001249e90c74 Not Available;Not Available 13;13 147;147 84;84 Alimta;Carboplatin Not Available;Not Available 3;3 4;4 1000/100;625/500 mg/mL;mg/mL ADJUVANT;ADJUVANT Not Applicable;Not Applicable 1;1 IV;IV NO;NO Chemotherapy;Chemotherapy Not Available;Not Available Not Available;Not Available Not Available;Not Available Not Available;Not Available 2012;2012 1 Not Available YES Other Not Available Not Available Not Available Not Available NOT HISPANIC OR LATINO Not Available Not Available Not Available Not Available TCGA-69-7761-F29252 86590873-3f3a-4004-8f1e-1c36818eb6fd 13 Not Applicable Not Applicable Not Applicable 186 Not Applicable 1 Scheduled Follow-up Submission Not Available Not Available Not Available 3 Not Available NO Adjuvant therapy TUMOR FREE YES Not Available Not Available NO Alive 2012 MALE Lung Acinar Adenocarcinoma No C34.3 8550/3 C34.3 YES Not Available Not Available Not Available Not Available Peripheral Lung 3 Not Available Not Available Not Available Not Available Not Available Not Available Not Available Not Available Not Available Not Available 20 No 7761 Other TUMOR FREE Not Available Not Available Not Available 91 98 Not Available YES WHITE Not Available Not Available Not Applicable Not Applicable Not Applicable Not Applicable Not Applicable Not Applicable Not Applicable Not Applicable Not Applicable Stage IB Not Applicable Not Applicable Not Applicable 7th Not Applicable Not Applicable Not Applicable MX N0 T2a 1975 NO YES 69 3 Lung Alive 2012 2011 1955

TCGA-91-6828 323 Alive T1a N0 M0 Stage IA 70 L-Lower Not Applicable TCGA-91-6828 9536e32d-2707-48d2-a36d-08c521665bb9 15 -25870 Not Applicable 0 38 Not Available Lung Adenocarcinoma 71 2 Not Available Not Available Not Available Not Available Not Available Not Available Not Available NOT HISPANIC OR LATINO Not Available Not Available Not Available Not Available TCGA-91-6828-F21014 182ca854-9147-4d4e-b2aa-7b899e591214 8 Not Applicable Not Applicable Not Applicable 323 Not Applicable 2 Scheduled Follow-up Submission Not Available Not Available Not Available 5 Not Available NO Preoperative TUMOR FREE NO Complete Remission/Response Not Available NO Alive 2012 MALE Lung Adenocarcinoma- Not Otherwise Specified (NOS) No C34.3 8140/3 C34.3 YES Not Available NO Not Available Not Available Not Available 7 Not Available Not Available Not Available Not Available Not Available Not Available Not Available Not Available Not Available Not Available Not Available No 6828 Preoperative Not Available Not Available Not Available Not Available Not Available 81 Not Available YES WHITE Not Available R0 Not Applicable Not Applicable Not Applicable Not Applicable Not Applicable Not Applicable Not Applicable Not Applicable Not Applicable Stage IA Not Applicable Not Applicable Not Applicable 7th Not Applicable Not Applicable Not Applicable M0 N0 T1a 1993 YES NO 91 3 Lung Alive 2011 2011 Not Available

TCGA-67-3771 610 Alive T1 N0 M0 Stage IA 77 L-Upper Not Applicable TCGA-67-3771 0df573ee-28f0-4244-b434-09e6ca59fbf0 21 -28406 Not Applicable 0 610 Not Available Lung Adenocarcinoma Not Available Not Available Not Available NO Not Available Not Available Not Available NO Not Available NOT HISPANIC OR LATINO FEMALE Lung Adenocarcinoma- Not Otherwise Specified (NOS) No C34.1 8140/3 C34.1 YES Not Available NO Not Available Not Available Central Lung 1 Not Available Not Available Not Available Not Available Not Available Not Available Not Available Not Available Not Available Not Available Not Available No 3771 Not Available TUMOR FREE Not Available Not Available Not Available Not Available Not Available Not Available Not Available BLACK OR AFRICAN AMERICAN Not Available R0 Not Applicable Not Applicable Not Applicable Not Applicable Not Applicable Not Applicable Not Applicable Not Applicable Not Applicable Stage IA Not Applicable Not Applicable Not Applicable 6th Not Applicable Not Applicable Not Applicable M0 N0 T1 Not Available YES NO 67 4 Lung Alive 2011 2009 Not Available

TCGA-49-4507 268 Dead T3 N1 M0 Stage IIIA 73 Discrepancy Not Available TCGA-49-4507 50b2c647-aecd-4c81-af22-0d8116593552 2 -26851 268 0 Not Available Not Available Lung Adenocarcinoma Not Available TCGA-49-4507-D7010;TCGA-49-4507-D13868;TCGA-49-4507-D17259 5d1cdc10-8244-4d21-b7fd-b69dfe9548dc;96cdd9a1-d9ad-4861-88e3-d65b2d13f956;7c8c9fcb-3b5f-42fe-9f31-c8319cde0fc7 Not Available;Not Available;Not Available 2;18;6 110;110;Not Available 47;47;174 Taxol;Carboplatin;Gemcitabine Not Available;Not Available;Not Available 2;7;10 4;4;Not Available Not Available;Not Available;Not Available Not Available;Not Available;Not Available ADJUVANT;ADJUVANT;RECURRENCE Not Applicable;Not Applicable;Not Applicable 1;1;2 IV;IV;IV NO;NO;NO Chemotherapy;Chemotherapy;Chemotherapy Not Available;Not Available;Not Available Not Available;4 cycles;Not Available g/m2;g/m2;Not Available Not Available;Not Available;Not Available 2011;2011;2011 1 Not Available Not Available Not Available Not Available Not Available Not Available Not Available NOT HISPANIC OR LATINO YES NO NO NO TCGA-49-4507-F7007 33c2605a-97c1-495f-ac44-17fff4c8ebd6 2 Not Available Not Available 268 Not Available 158 1 Not Available Progressive Disease Not Available Not Available 2 Not Available YES Preoperative WITH TUMOR YES Progressive Disease Not Available YES Dead 2011 FEMALE Lung Adenocarcinoma- Not Otherwise Specified (NOS) No C34.0 8140/3 C34.0 YES Not Available Not Available Not Available Not Available Not Available 2 Not Available Not Available Not Available Not Available Not Available Not Available Not Available Not Available Not Available Not Available 78 No 4507 Preoperative WITH TUMOR Not Available Not Available Not Available Not Available Not Available Not Available Not Available WHITE Not Available Primary Tumor Field TCGA-49-4507-R7009 becb6cbe-b49e-49ae-ae68-ac7e19de8c3b 1 2 95 46 Not Available 2 34 1620 NO EXTERNAL BEAM Not Applicable ADJUVANT Not Available cGy 2011 R0 Not Applicable Not Applicable Not Applicable Not Applicable Not Applicable Not Applicable Not Applicable Not Applicable Not Applicable Stage IIIA Not Applicable Not Applicable Not Applicable Not Available Not Applicable Not Applicable Not Applicable M0 N1 T3 1991 NO YES 49 4 Lung Dead 2011 1997 Not Available

TCGA-78-7158 179 Dead T4 N2 M0 Stage IIIB 59 L-Lower Not Applicable TCGA-78-7158 501c987e-d1eb-48a9-89eb-72a5062c90b4 15 -21742 179 0 Not Available Not Available Lung Adenocarcinoma Not Available TCGA-78-7158-D16167;TCGA-78-7158-D16978 b9fc15d3-ee12-436e-8bd1-d57382720f61;b2454d2f-cb59-470a-b300-029134f6bb34 Not Available;Not Available 15;29 103;103 54;54 Carboplatin;Paclitaxel Not Available;Not Available 9;9 3;3 Not Available;Not Available Not Available;Not Available ADJUVANT;ADJUVANT Not Applicable;Not Applicable 1;1 IV;IV NO;NO Chemotherapy;Chemotherapy Not Available;Not Available 1220;620 mg;mg Not Available;Not Available 2011;2011 1 Not Available NO Not Available Not Available Not Available NO Not Available Not Available Not Available Not Available NO NO TCGA-78-7158-F16166 cd749669-0955-4e60-80d4-d02a208e1f64 15 Not Available Not Available 179 Not Available 132 Not Available Scheduled Follow-up Submission Not Available Not Available Not Available 9 Not Available YES Not Available WITH TUMOR YES Progressive Disease Not Available NO Dead 2011 FEMALE Lung Adenocarcinoma Mixed Subtype No C34.3 8255/3 C34.3 YES Not Available NO Not Available Not Available Peripheral Lung 9 Not Available Not Available Not Available Not Available Not Available Not Available Not Available Not Available Not Available Not Available 14 No 7158 Preoperative WITH TUMOR Not Available Not Available Not Available Not Available Not Available Not Available Not Available WHITE Not Available R1 Not Applicable Not Applicable Not Applicable Not Applicable Not Applicable Not Applicable Not Applicable Not Applicable Not Applicable Stage IIIB Not Applicable Not Applicable Not Applicable 6th Not Applicable Not Applicable Not Applicable M0 N2 T4 2005 NO YES 78 4 Lung Dead 2011 2007 1963

TCGA-86-8671 839 Alive T2b N1 M0 Stage IIB 72 R-Upper Not Applicable TCGA-86-8671 c4cfa3cc-e305-42dd-be57-97efbb891659 30 -26448 Not Applicable 0 18 Not Available Lung Adenocarcinoma Not Available TCGA-86-8671-D66090;TCGA-86-8671-D66091 151A219B-0DB6-4158-A30B-35B97CC9A1A9;36EB3446-D9DC-4620-830A-8CE6A214F2DB Not Available;Not Available 2;2 104;104 18;18 Cisplatin;Etoposide Complete Response;Complete Response 10;10 Not Available;Not Available Not Available;Not Available Not Available;Not Available Not Available;Not Available Not Applicable;Not Applicable Not Available;Not Available Not Available;Not Available NO;NO Chemotherapy;Chemotherapy Not Available;Not Available Not Available;Not Available Not Available;Not Available NO;NO 2014;2014 1 Not Available Not Available Not Available Not Available Not Available Not Available Not Available NOT HISPANIC OR LATINO Not Available Not Available Not Available Not Available TCGA-86-8671-F66089 97BC43F2-A652-4731-B5BD-37770F5ADB10 2 Not Applicable Not Applicable Not Applicable 839 Not Applicable 0 Scheduled Follow-up Submission Complete Remission/Response 100 NO 10 Not Available NO Other TUMOR FREE YES Complete Remission/Response Not Available NO Alive 2014 FEMALE Lung Papillary Adenocarcinoma No C34.1 8260/3 C34.1 YES 90 Not Available Not Available Not Available Peripheral Lung 8 Not Available Not Available Not Available Not Available Not Available Not Available Not Available Not Available Not Available Unknown Not Available No 8671 Preoperative WITH TUMOR Not Available Not Available Unknown Not Available Not Available Complete Remission/Response NO WHITE Unknown R0 Not Applicable Not Applicable Not Applicable Not Applicable Not Applicable Not Applicable Not Applicable Not Applicable Not Applicable Stage IIB Not Applicable Not Applicable Not Applicable 7th Not Applicable Not Applicable Not Applicable M0 N1 T2b Not Available YES NO 86 1 Lung Alive 2012 2012 Not Available

TCGA-97-A4M6 568 Alive T1a N0 M0 Stage IA 45 R-Lower Not Applicable TCGA-97-A4M6 31FF69B5-9E58-44DA-8326-BDFC7EE495C4 7 -16764 Not Applicable 0 185 Not Available Lung Adenocarcinoma 92 0 Not Available YES Exon 19 Deletion Not Available Not Available NO Not Available NOT HISPANIC OR LATINO Not Available Not Available Not Available Not Available TCGA-97-A4M6-F57434 5B7F6A58-58A0-4D88-A6E1-339DD38CBEC4 11 Not Applicable Not Applicable Not Applicable 568 Not Applicable 0 Scheduled Follow-up Submission Complete Remission/Response Not Evaluated NO 3 Not Available NO Other TUMOR FREE NO Complete Remission/Response Not Available NO Alive 2014 FEMALE Lung Adenocarcinoma Mixed Subtype No C34.3 8255/3 C34.3 YES Not Evaluated YES NO Not Available Unknown 3 Not Available Not Available Not Available Not Available Not Available Not Available Not Available Not Available Not Available NO Not Available No A4M6 Preoperative Unknown 95 116 NO 95 110 Complete Remission/Response YES WHITE NO Not Evaluated Not Applicable Not Applicable Not Applicable Not Applicable Not Applicable Not Applicable Not Applicable Not Applicable Not Applicable Stage IA Not Applicable Not Applicable Not Applicable 7th Not Applicable Not Applicable Not Applicable M0 N0 T1a Not Available YES NO 97 1 Lung Alive 2013 2012 Not Available

TCGA-L4-A4E5 578 Alive T1 N0 M0 Stage I 48 L-Upper Not Applicable TCGA-L4-A4E5 32A7B827-C3F9-4607-AE63-DAE8CAA2BC97 5 -17680 Not Applicable 0 213 Not Available Lung Adenocarcinoma 93 1 Not Available NO Not Available Not Available Not Available NO Not Available NOT HISPANIC OR LATINO Not Available Not Available Not Available Not Available TCGA-L4-A4E5-F49031 DC4F62D9-03A2-4A06-A9B0-89FCCD37B4FB 25 Not Applicable Not Applicable Not Applicable 578 Not Applicable 1 Scheduled Follow-up Submission Complete Remission/Response Not Evaluated NO 9 Not Available NO Other TUMOR FREE NO Complete Remission/Response Not Available NO Alive 2013 FEMALE Lung Adenocarcinoma- Not Otherwise Specified (NOS) No C34.1 8140/3 C34.1 YES Not Evaluated NO Not Available Not Available Central Lung 12 Not Available Not Available Not Available Not Available Not Available Not Available Not Available Not Available Not Available NO 33 No A4E5 Preoperative TUMOR FREE 94 82 NO 94 82 Complete Remission/Response YES WHITE NO R0 Not Applicable Not Applicable Not Applicable Not Applicable Not Applicable Not Applicable Not Applicable Not Applicable Not Applicable Stage I Not Applicable Not Applicable Not Applicable 7th Not Applicable Not Applicable Not Applicable M0 N0 T1 2012 NO YES L4 4 Lung Alive 2012 2012 1979

TCGA-91-A4BC 44 Alive T2b N0 MX Stage IIA 59 R-Upper Not Applicable TCGA-91-A4BC EA0B9461-0D4D-4ADE-A255-1B03DD85E30E 5 -21565 Not Applicable 0 44 Not Available Lung Adenocarcinoma 44 Not Evaluated Not Available NO Not Available Not Available Not Available Not Available Not Available NOT HISPANIC OR LATINO Not Available Not Available Not Available Not Available TCGA-91-A4BC-F45192 5A4ACA69-D8C5-4169-9815-150180962775 10 Not Available Not Available Not Applicable 44 Not Available Not Evaluated Scheduled Follow-up Submission Unknown Not Evaluated YES 7 Not Available Unknown Not Evaluated Unknown Unknown Unknown Not Available Unknown Alive 2013 MALE Lung Adenocarcinoma- Not Otherwise Specified (NOS) No C34.1 8140/3 C34.1 YES Not Evaluated NO Not Available Not Available Not Available 2 Not Available Not Available Not Available Not Available Not Available Not Available Not Available Not Available Not Available Unknown 30 Yes, History of Synchronous/Bilateral Malignancy A4BC Not Evaluated Unknown Not Available 69 Unknown Not Available Not Available Unknown YES WHITE Unknown Not Evaluated Not Applicable Not Applicable Not Applicable Not Applicable Not Applicable Not Applicable Not Applicable Not Applicable Not Applicable Stage IIA Not Applicable Not Applicable Not Applicable 7th Not Applicable Not Applicable Not Applicable MX N0 T2b 2001 YES NO 91 4 Lung Alive 2013 2012 1976

TCGA-69-7980 411 Alive T1b N0 M0 Stage I 70 R-Upper Not Applicable TCGA-69-7980 aee86a89-0377-4080-b16c-408bfbe78687 9 -25583 Not Applicable 0 43 Not Available Lung Adenocarcinoma 71 Not Available Not Available YES Other Not Available Not Available NO Not Available NOT HISPANIC OR LATINO Not Available;Not Available Not Available;Not Available NO;Not Available Not Available;Not Available TCGA-69-7980-F39916;TCGA-69-7980-F57659 B9570D2D-FF75-4067-8D99-C36BCD332AC0;B3F0B9EB-890D-418B-9A53-A3F0240BB33D 11;19 Not Applicable;Not Applicable Not Applicable;Not Applicable Not Applicable;Not Applicable 411;362 Not Applicable;Not Applicable Not Evaluated;Not Available Scheduled Follow-up Submission;Scheduled Follow-up Submission Unknown;Not Applicable Not Evaluated;Not Available NO;NO 2;3 Not Available;Not Available NO;NO Not Evaluated;Not Available TUMOR FREE;TUMOR FREE NO;NO Complete Remission/Response;Complete Remission/Response Not Available;Not Available NO;NO Alive;Alive 2013;2014 FEMALE Lung Adenocarcinoma- Not Otherwise Specified (NOS) No C34.1 8550/3 C34.1 YES Not Available NO Not Available Not Available Not Available 4 Not Available Not Available Not Available Not Available Not Available Not Available Not Available Not Available Not Available Not Available 80 No 7980 Not Available TUMOR FREE Not Available Not Available Not Available 86 92 Not Available YES WHITE Not Available Not Available Not Applicable Not Applicable Not Applicable Not Applicable Not Applicable Not Applicable Not Applicable Not Applicable Not Applicable Stage I Not Applicable Not Applicable Not Applicable 7th Not Applicable Not Applicable Not Applicable M0 N0 T1b 2001 YES NO 69 4 Lung Alive 2012 2011 Not Available

TCGA-62-A46R 1725 Dead T2 N0 M0 Stage IB 54 L-Lower Not Applicable TCGA-62-A46R C497F8C7-2FB5-44D9-BC7C-69CFD0B51B54 29 -20063 1725 0 Not Available Not Available Lung Adenocarcinoma Not Available Unknown Not Available NO Not Available Not Available Not Available NO Not Available NOT HISPANIC OR LATINO FEMALE Lung Adenocarcinoma Mixed Subtype No C34.3 8255/3 C34.3 YES 100 NO Not Available Not Available Central Lung 10 Not Available Not Available Not Available Not Available Not Available Not Available Not Available Not Available Not Available NO 25 No A46R Preoperative TUMOR FREE Not Available Not Available NO 73 74 Complete Remission/Response YES WHITE NO R0 Not Applicable Not Applicable Not Applicable Not Applicable Not Applicable Not Applicable Not Applicable Not Applicable Not Applicable Stage IB Not Applicable Not Applicable Not Applicable 6th Not Applicable Not Applicable Not Applicable M0 N0 T2 Not Available NO YES 62 4 Lung Dead 2012 2006 Not Available

TCGA-91-8499 36 Alive T1b N0 MX Stage IA 76 L-Lower Not Applicable TCGA-91-8499 54480f58-7e63-4aed-a116-c2c2252e8364 29 -27853 Not Applicable 0 36 Not Available Lung Adenocarcinoma Not Available Unknown Not Available YES Not Available Not Available Not Available Not Available Not Available NOT HISPANIC OR LATINO Not Available Not Available Not Available Not Available TCGA-91-8499-F37976 A3296482-B65D-46DC-8306-36D9A0CE0CAB 7 Not Applicable Not Applicable Not Applicable 36 Not Applicable Not Available Scheduled Follow-up Submission Unknown Unknown YES 12 Not Available NO Not Available Unknown NO Unknown Not Available NO Alive 2012 FEMALE Lung Adenocarcinoma- Not Otherwise Specified (NOS) No C34.3 8140/3 C34.3 YES Unknown NO Not Available Not Available Unknown 8 Not Available Not Available Not Available Not Available Not Available Not Available Not Available Not Available Not Available NO 90 No 8499 Not Available Unknown 49 Not Available NO Not Available Not Available Unknown YES WHITE NO Not Evaluated Not Applicable Not Applicable Not Applicable Not Applicable Not Applicable Not Applicable Not Applicable Not Applicable Not Applicable Stage IA Not Applicable Not Applicable Not Applicable 7th Not Applicable Not Applicable Not Applicable MX N0 T1b Not Available YES NO 91 2 Lung Alive 2012 2011 1945

TCGA-95-8494 84 Alive T2a N1 M0 Stage IIA 67 R-Middle Not Applicable TCGA-95-8494 6ee9caec-65ac-46db-9426-ffaa31967ae2 30 -24773 Not Applicable 0 71 Not Available Lung Adenocarcinoma Not Available Not Available Not Available NO Not Available Not Available Not Available NO Not Available NOT HISPANIC OR LATINO Not Available Not Available Not Available Not Available TCGA-95-8494-F58476 5A2D5D46-74F5-48C7-8E7E-751B9C1F0BF3 10 Not Available Not Available Not Applicable 84 Not Available Not Available Scheduled Follow-up Submission Unknown Not Available YES 4 Not Available Unknown Not Available Not Available NO Complete Remission/Response Not Available NO Alive 2014 MALE Lung Adenocarcinoma- Not Otherwise Specified (NOS) No C34.2 8140/3 C34.2 YES Not Available NO Not Available Not Available Not Available 8 Not Available Not Available Not Available Not Available Not Available Not Available Not Available Not Available Not Available NO Not Available No 8494 Not Available Unknown Not Available Not Available NO Not Available Not Available Unknown NO WHITE NO Not Evaluated Not Applicable Not Applicable Not Applicable Not Applicable Not Applicable Not Applicable Not Applicable Not Applicable Not Applicable Stage IIA Not Applicable Not Applicable Not Applicable 7th Not Applicable Not Applicable Not Applicable M0 N1 T2a Not Available YES NO 95 5 Lung Alive 2012 2012 Not Available

TCGA-38-4626 3674 Alive T2b N0 M0 57 L-Upper Not Applicable TCGA-38-4626 2079155a-d91d-4246-a038-01934a580f32 7 -20903 Not Applicable 0 2595 Not Available Lung Adenocarcinoma 57 Not Available Not Available Not Available Not Available Not Available Not Available Not Available Not Available NOT HISPANIC OR LATINO Not Available;NO Not Available;NO Not Available;NO Not Available;Not Available TCGA-38-4626-F4507;TCGA-38-4626-F33409 f7d53f3d-25b9-43f5-9d12-f4c38dd72816;11F9E5D4-D903-4AC9-A6CC-3CB66E89CD21 8;26 Not Applicable;Not Available Not Applicable;Not Available Not Applicable;Not Applicable 2595;3674 Not Applicable;2518 Not Available;Not Available Not Available;Scheduled Follow-up Submission Not Available;Complete Remission/Response Not Available;Not Available Not Available;NO 12;7 Not Available;New Primary Tumor NO;YES Not Available;Not Available TUMOR FREE;TUMOR FREE NO;NO Complete Remission/Response;Complete Remission/Response Not Available;Not Available NO;NO Alive;Alive 2010;2012 FEMALE Lung Adenocarcinoma- Not Otherwise Specified (NOS) No C34.1 8140/3 C34.1 YES Not Available Not Available Not Available Not Available Central Lung 12 Not Available Not Available Not Available Not Available Not Available Not Available Not Available Not Available Not Available Not Available 40 No 4626 Not Available TUMOR FREE 68 85 Not Available 71 85 Not Available YES WHITE Not Available R0 Not Applicable Not Applicable Not Applicable Not Applicable Not Applicable Not Applicable Not Applicable Not Applicable Not Applicable Discrepancy Not Applicable Not Applicable Not Applicable 6th Not Applicable Not Applicable Not Applicable M0 N0 T2b Not Available NO YES 38 2 Lung Alive 2010 2003 1962

TCGA-78-7143 4961 Dead T2 N0 M0 Stage IB 62 L-Upper Not Applicable TCGA-78-7143 54254f5a-50e8-4150-a9b7-56a0470d2a56 23 -22673 4961 0 Not Available Not Available Lung Adenocarcinoma Not Available 0 Not Available NO Not Available Not Available Not Available NO Not Available Not Available NO;NO NO;NO NO;YES NO;NO TCGA-78-7143-F16669;TCGA-78-7143-F16667 b280d071-c071-4b1f-8df7-1eb99db3a117;6bba4fc0-b389-42ae-b498-67aeae1e3b46 23;23 Not Available;1577 Not Available;Not Available 4961;4961 Not Available;Not Available 4608;1500 Not Available;Not Available Additional New Tumor Event;Scheduled Follow-up Submission Not Available;Complete Remission/Response Not Available;Not Available Not Available;Not Available 9;9 Not Available;Not Available YES;YES Not Available;Not Available Discrepancy;Discrepancy Not Available;NO Not Available;Not Available Not Available;Not Available Not Available;NO Dead;Dead 2011;2011 FEMALE Lung Bronchioloalveolar Carcinoma Nonmucinous No C34.1 8252/3 C34.1 YES Not Available NO Not Available Not Available Central Lung 9 Not Available Not Available Not Available Not Available Not Available Not Available Not Available Not Available Not Available Not Available Not Available No 7143 Preoperative Discrepancy Not Available Not Available Not Available Not Available Not Available Not Available Not Available WHITE Not Available R0 Not Applicable Not Applicable Not Applicable Not Applicable Not Applicable Not Applicable Not Applicable Not Applicable Not Applicable Stage IB Not Applicable Not Applicable Not Applicable 6th Not Applicable Not Applicable Not Applicable M0 N0 T2 Not Available NO YES 78 1 Lung Dead 2011 1992 Not Available

TCGA-49-AAQV 677 Dead T1 N1 MX Stage II 63 L-Lower Not Applicable TCGA-49-AAQV 96DFA373-4597-49A5-942E-D9B4DC5D28FC 13 -23370 677 0 Not Available Not Available Lung Adenocarcinoma 95.5 1 Not Available YES Exon 19 Deletion Not Available FISH YES Not Available NOT HISPANIC OR LATINO FEMALE Lung Adenocarcinoma Mixed Subtype No C34.3 8255/3 C34.3 YES 90 Not Available Not Available Not Available Not Available 6 NO YES 509 Not Available Not Available YES 536 Distant Metastasis Biopsy with Histologic Confirmation;Convincing Imaging YES Not Available No AAQV Preoperative TUMOR FREE 77.29 81.7 NO 78.80 83.3 Complete Remission/Response YES BLACK OR AFRICAN AMERICAN NO R0 Not Applicable Not Applicable Not Applicable Not Applicable Not Applicable Not Applicable Not Applicable Not Applicable Not Applicable Stage II Not Applicable Not Applicable Not Applicable 7th Not Applicable Not Applicable Not Applicable MX N1 T1 Not Available NO YES 49 1 Lung Dead 2014 2012 Not Available

TCGA-55-6985 1233 Alive T2 N0 MX Stage IB 58 L-Upper Not Applicable TCGA-55-6985 e5cb0c86-8fe2-4cfc-b32b-e8ec3839ffc4 26 -21381 Not Applicable 0 1233 Not Available Lung Adenocarcinoma Not Available Not Available Not Available NO Not Available Not Available Not Available NO Not Available Not Available Not Available Not Available Not Available Not Available TCGA-55-6985-F37056 F432F92C-4BB0-437C-A742-B9E6B4BD8E55 22 Not Applicable Not Applicable Not Applicable 1233 Not Applicable Not Available Scheduled Follow-up Submission Not Available Not Available YES 2 Not Available NO Not Available TUMOR FREE NO Complete Remission/Response Not Available NO Alive 2013 FEMALE Lung Adenocarcinoma- Not Otherwise Specified (NOS) No C34.1 8140/3 C34.1 YES Not Available NO Not Available Not Available Not Available 7 Not Available Not Available Not Available Not Available Not Available Not Available Not Available Not Available Not Available Not Available 50 No 6985 Not Available TUMOR FREE Not Available Not Available Not Available Not Available Not Available Not Available Not Available WHITE Not Available R0 Not Applicable Not Applicable Not Applicable Not Applicable Not Applicable Not Applicable Not Applicable Not Applicable Not Applicable Stage IB Not Applicable Not Applicable Not Applicable 6th Not Applicable Not Applicable Not Applicable MX N0 T2 2004 NO YES 55 4 Lung Alive 2011 2004 Not Available

TCGA-05-4405 610 Alive T2 N0 M0 Stage IB 74 R-Lower Not Applicable TCGA-05-4405 f587c9ab-2949-4410-80d6-a050865d48aa 22 -27241 Not Applicable 0 610 Not Available Lung Adenocarcinoma Not Available Not Available Not Available Not Available Not Available Not Available Not Available Not Available Not Available Not Available Not Available Not Available Not Available Not Available TCGA-05-4405-F36494 83F914FE-F0D0-4B75-9F4B-DD17F59C62A0 31 Not Applicable Not Applicable Not Applicable 610 Not Applicable Not Available Scheduled Follow-up Submission Complete Remission/Response Not Available NO 10 Not Available NO Not Available TUMOR FREE NO Not Applicable Not Available NO Alive 2012 FEMALE Lung Adenocarcinoma Mixed Subtype No C34.3 8255/3 C34.3 YES Not Available Not Available Not Available Not Available Not Available 7 Not Available Not Available Not Available Not Available Not Available Not Available Not Available Not Available Not Available Not Available 65 Yes 4405 Not Available TUMOR FREE Not Available Not Available Not Available Not Available Not Available Not Available Not Available Not Available Not Available R0 Not Applicable Not Applicable Not Applicable Not Applicable Not Applicable Not Applicable Not Applicable Not Applicable Not Applicable Stage IB Not Applicable Not Applicable Not Applicable 5th Not Applicable Not Applicable Not Applicable M0 N0 T2 1994 NO YES 05 4 Lung Alive 2010 2006 1951

TCGA-50-5944 1750 Alive T1 N0 M0 Stage IA 69 R-Upper Not Applicable TCGA-50-5944 1c339545-c08a-4a77-b8e9-a7e49e53853f 2 -25440 Not Applicable 0 373 Not Available Lung Adenocarcinoma Not Available Not Available Not Available Not Available Not Available Not Available Not Available Not Available Not Available Not Available Not Available;Not Available Not Available;Not Available Not Available;Not Available Not Available;Not Available TCGA-50-5944-F32136;TCGA-50-5944-F70454 974a8262-421f-448c-805e-b4553844dc24;6CEEF46B-0ED2-4D96-BB16-9FB2A5D44BF9 17;16 Not Applicable;Not Applicable Not Applicable;Not Applicable Not Applicable;Not Applicable 764;1750 Not Applicable;Not Applicable Not Available;Not Available Scheduled Follow-up Submission;Scheduled Follow-up Submission Complete Remission/Response;Complete Remission/Response Not Available;Not Available NO;NO 5;2 Not Available;Not Available NO;NO Not Available;Not Available TUMOR FREE;TUMOR FREE NO;NO Complete Remission/Response;Complete Remission/Response Not Available;Not Available NO;NO Alive;Alive 2012;2015 FEMALE Lung Acinar Adenocarcinoma No C34.1 8550/3 C34.1 YES Not Available Not Available Not Available Not Available Not Available 7 Not Available Not Available Not Available Not Available Not Available Not Available Not Available Not Available Not Available Not Available Not Available No 5944 Not Available TUMOR FREE Not Available Not Available Not Available Not Available Not Available Not Available NO WHITE Not Available R0 Not Applicable Not Applicable Not Applicable Not Applicable Not Applicable Not Applicable Not Applicable Not Applicable Not Applicable Stage IA Not Applicable Not Applicable Not Applicable 7th Not Applicable Not Applicable Not Applicable M0 N0 T1 Not Available NO YES 50 Not Available Lung Alive 2011 2010 Not Available

TCGA-91-6835 79 Alive T1 N0 M0 Stage IA 81 R-Lower Not Applicable TCGA-91-6835 2f09479f-87fc-4c34-8e2c-333e970a3681 20 -29887 Not Applicable 0 35 Not Available Lung Adenocarcinoma 78 Not Available Not Available NO Not Available Not Available Not Available Not Available Not Available NOT HISPANIC OR LATINO Not Available Not Available Not Available Not Available TCGA-91-6835-F32643 169e8cda-2406-4085-97e2-7287e1bd2e41 5 Not Applicable Not Applicable Not Applicable 79 Not Applicable Unknown Scheduled Follow-up Submission Complete Remission/Response Unknown YES 6 Not Available NO Not Available TUMOR FREE NO Complete Remission/Response Not Available NO Alive 2012 FEMALE Lung Adenocarcinoma- Not Otherwise Specified (NOS) No C34.3 8140/3 C34.3 YES Not Available NO Not Available Not Available Not Available 7 Not Available Not Available Not Available Not Available Not Available Not Available Not Available Not Available Not Available Not Available 25 No 6835 Not Available Not Available Not Available 91 Not Available Not Available 75 Not Available YES WHITE Not Available R0 Not Applicable Not Applicable Not Applicable Not Applicable Not Applicable Not Applicable Not Applicable Not Applicable Not Applicable Stage IA Not Applicable Not Applicable Not Applicable 7th Not Applicable Not Applicable Not Applicable M0 N0 T1 1976 NO YES 91 3 Lung Alive 2011 2010 Not Available

TCGA-80-5608 2832 Alive T1 N0 M0 Stage IA Not Available R-Upper Not Applicable TCGA-80-5608 5c06d97f-2aaa-43de-b085-09d7829356ba 15 Not Available Not Applicable Not Available Not Available Not Available Lung Adenocarcinoma Not Available 1 Not Available Not Available Not Available Not Available Not Available Not Available Not Available Not Available Not Available;Not Available Not Available;Not Available Not Available;Not Available Not Available;Not Available TCGA-80-5608-F15870;TCGA-80-5608-F72033 242c20b9-5323-4ed7-91f3-3620555f7814;D9A05844-EA6C-4BE0-A6B9-C557069ACD65 15;2 Not Applicable;Not Applicable Not Applicable;Not Applicable Not Applicable;Not Applicable Not Available;2832 Not Applicable;Not Applicable Not Available;Unknown Not Available;Scheduled Follow-up Submission Not Available;Not Available Not Available;Unknown Not Available;NO 8;4 Not Available;Not Available NO;NO Not Available;Unknown TUMOR FREE;TUMOR FREE NO;NO Complete Remission/Response;Complete Remission/Response Not Available;Not Available NO;NO Alive;Alive 2011;2015 FEMALE Lung Adenocarcinoma- Not Otherwise Specified (NOS) No C34.1 8140/3 C34.1 YES Not Available Not Available Not Available Not Available Peripheral Lung 8 Not Available Not Available Not Available Not Available Not Available Not Available Not Available Not Available Not Available Not Available 26 No 5608 Preoperative TUMOR FREE Not Available Not Available Not Available Not Available Not Available Not Available Not Available Not Available Not Available R0 Not Applicable Not Applicable Not Applicable Not Applicable Not Applicable Not Applicable Not Applicable Not Applicable Not Applicable Stage IA Not Applicable Not Applicable Not Applicable 6th Not Applicable Not Applicable Not Applicable M0 N0 T1 Not Available NO YES 80 2 Lung Alive 2011 Not Available 1972

TCGA-MP-A5C7 2248 Alive T2 N0 M0 Stage IB 76 L-Upper Not Applicable TCGA-MP-A5C7 8F04660A-2EFB-4947-9EAB-375066B9EB33 10 -28015 Not Applicable 0 1490 Not Available Lung Adenocarcinoma 105 1 Not Available NO Not Available Not Available Not Available NO Not Available NOT HISPANIC OR LATINO Not Available;Not Available Not Available;Not Available Not Available;Not Available Not Available;Not Available TCGA-MP-A5C7-F54544;TCGA-MP-A5C7-F67079 2777977A-E3E5-4AFD-AE85-CCC79DF0E736;A86ED60C-DDCE-4E9A-9B2D-9888E9F12C9F 19;30 Not Applicable;Not Applicable Not Applicable;Not Applicable Not Applicable;Not Applicable 1862;2248 Not Applicable;Not Applicable Not Evaluated;1 Scheduled Follow-up Submission;Scheduled Follow-up Submission Complete Remission/Response;Complete Remission/Response Not Evaluated;Not Evaluated NO;NO 12;10 Not Available;Not Available NO;NO Not Available;Other TUMOR FREE;TUMOR FREE NO;NO Complete Remission/Response;Complete Remission/Response Not Available;Not Available NO;NO Alive;Alive 2013;2014 FEMALE Lung Adenocarcinoma- Not Otherwise Specified (NOS) No C34.1 8140/3 C34.1 YES Not Evaluated NO Not Available Not Available Central Lung 5 Not Available Not Available Not Available Not Available Not Available Not Available Not Available Not Available Not Available NO 15 No A5C7 Pre-Adjuvant Therapy TUMOR FREE Not Available 93 NO Not Available 89 Complete Remission/Response YES WHITE NO R0 Not Applicable Not Applicable Not Applicable Not Applicable Not Applicable Not Applicable Not Applicable Not Applicable Not Applicable Stage IB Not Applicable Not Applicable Not Applicable 6th Not Applicable Not Applicable Not Applicable M0 N0 T2 1968 NO YES MP 3 Lung Alive 2013 2008 1953

TCGA-44-8117 385 Alive T2a N0 M0 Stage IB 54 R-Upper Not Applicable TCGA-44-8117 6d96a298-be5a-4e14-afe8-ca4b6ca298d6 11 -19855 Not Applicable 0 259 Not Available Lung Adenocarcinoma 86 TCGA-44-8117-D32726;TCGA-44-8117-D32727 19D36EEB-480F-4671-BCA8-86F6494EA205;EEB0D889-2452-4652-AD4C-81CCD822D2CE Not Available;Not Available 13;13 176;133 113;113 Cisplatin;Docetaxel Complete Response;Complete Response 6;6 Not Available;Not Available Not Available;Not Available Not Available;Not Available Not Available;Not Available Not Applicable;Not Applicable Not Available;Not Available Not Available;Not Available NO;NO Chemotherapy;Chemotherapy Not Available;Not Available Not Available;Not Available Not Available;Not Available NO;NO 2012;2012 1 Not Available Unknown Not Available Not Available Not Available Unknown Not Available NOT HISPANIC OR LATINO Not Available Not Available Not Available Not Available TCGA-44-8117-F36070 E5A2A932-540F-4F64-8A4B-0357E1C9C18B 18 Not Applicable Not Applicable Not Applicable 385 Not Applicable 1 Scheduled Follow-up Submission Complete Remission/Response Not Available NO 10 Not Available NO Post-Adjuvant Therapy TUMOR FREE YES Complete Remission/Response Not Available NO Alive 2012 FEMALE Lung Adenocarcinoma- Not Otherwise Specified (NOS) No C34.1 8140/3 C34.1 YES Not Available Unknown Not Available Not Available Unknown 6 Not Available Not Available Not Available Not Available Not Available Not Available Not Available Not Available Not Available NO 54 No 8117 Pre-Adjuvant Therapy TUMOR FREE 88 65 YES 83 60 Complete Remission/Response YES WHITE NO Not Available Not Applicable Not Applicable Not Applicable Not Applicable Not Applicable Not Applicable Not Applicable Not Applicable Not Applicable Stage IB Not Applicable Not Applicable Not Applicable 7th Not Applicable Not Applicable Not Applicable M0 N0 T2a 2011 YES NO 44 4 Lung Alive 2012 2011 1975

TCGA-L9-A7SV 565 Alive T2a N1 M0 Stage IIA 69 L-Lower Not Applicable TCGA-L9-A7SV 54E90433-5218-4D83-848F-F995ECBA2A63 7 -25298 Not Applicable 0 111 Not Available Lung Adenocarcinoma 55 TCGA-L9-A7SV-D55465;TCGA-L9-A7SV-D55466 01FFB728-9015-4A08-9273-307A5FFCA9C7;AF22DDC1-B76C-49AC-AAC4-32359ACDB718 Not Available;Not Available 7;7 104;104 41;41 cisplatin;pemetrexed disodium Complete Response;Complete Response 1;1 Not Available;Not Available Not Available;Not Available Not Available;Not Available Not Available;Not Available Not Applicable;Not Applicable Not Available;Not Available Not Available;Not Available NO;NO Chemotherapy;Chemotherapy Not Available;Not Available Not Available;Not Available Not Available;Not Available NO;NO 2014;2014 0 Not Available NO Not Available Not Available Not Available NO Not Available NOT HISPANIC OR LATINO Not Available Not Available Not Available Not Available TCGA-L9-A7SV-F65116 4AC0A1A6-41A9-4E59-B676-A750306905C7 18 Not Applicable Not Applicable Not Applicable 565 Not Applicable 0 Scheduled Follow-up Submission Complete Remission/Response Not Available NO 9 Not Available NO Preoperative TUMOR FREE YES Complete Remission/Response Not Available NO Alive 2014 MALE Lung Adenocarcinoma- Not Otherwise Specified (NOS) No C34.3 8140/3 C34.3 YES Not Evaluated NO Not Available Not Available Unknown 1 Not Available Not Available Not Available Not Available Not Available Not Available Not Available Not Available Not Available NO 93 No A7SV Preoperative TUMOR FREE 40 45 YES 38 40 Complete Remission/Response YES BLACK OR AFRICAN AMERICAN NO R0 Not Applicable Not Applicable Not Applicable Not Applicable Not Applicable Not Applicable Not Applicable Not Applicable Not Applicable Stage IIA Not Applicable Not Applicable Not Applicable 7th Not Applicable Not Applicable Not Applicable M0 N1 T2a 1990 YES NO L9 3 Lung Alive 2014 2013 1959

TCGA-69-7760 202 Alive T3 N0 M0 Stage IIB 73 R-Lower Not Applicable TCGA-69-7760 8981f0c9-7155-4c96-bf77-7d40fe404323 24 -26777 Not Applicable 0 154 Not Available Lung Adenocarcinoma 125 TCGA-69-7760-D20981;TCGA-69-7760-D21152 94bb8b11-881e-449b-8206-95458a4e6762;5fa9b68b-32b6-4df9-a9a8-01cc626473d6 Not Available;Not Available 25;2 110;110 47;47 Carboplatin;pemetrexed disodium Not Available;Not Available 1;2 4;4 588.6;905 mg;mg ADJUVANT;ADJUVANT Not Applicable;Not Applicable 1;1 IV;IV NO;NO Chemotherapy;Chemotherapy Not Available;Not Available Not Available;Not Available Not Available;Not Available Not Available;Not Available 2012;2012 Not Available Not Available YES Not Available Not Available Not Available NO Not Available NOT HISPANIC OR LATINO Not Available Not Available Not Available Not Available TCGA-69-7760-F29247 58e0fa1d-b060-47b2-808b-631e2c8d3b82 13 Not Applicable Not Applicable Not Applicable 202 Not Applicable Not Available Scheduled Follow-up Submission Not Available Not Available Not Available 3 Not Available NO Post-Adjuvant Therapy TUMOR FREE YES Not Available Not Available NO Alive 2012 MALE Lung Adenocarcinoma Mixed Subtype No C34.3 8255/3 C34.3 YES Not Available NO Not Available Not Available Peripheral Lung 1 Not Available Not Available Not Available Not Available Not Available Not Available Not Available Not Available Not Available Not Available Not Available No 7760 Not Available Not Available Not Available Not Available Not Available 91 83 Not Available YES WHITE Not Available Not Available Not Applicable Not Applicable Not Applicable Not Applicable Not Applicable Not Applicable Not Applicable Not Applicable Not Applicable Stage IIB Not Applicable Not Applicable Not Applicable 7th Not Applicable Not Applicable Not Applicable M0 N0 T3 Not Available YES NO 69 1 Lung Alive 2012 2011 Not Available

TCGA-91-6830 60 Alive T1 N1 MX Stage IIA 65 L-Upper Not Applicable TCGA-91-6830 5a2f8140-8f90-4e94-b703-5fa5aa96be7b 19 -23922 Not Applicable 0 1 Not Available Lung Adenocarcinoma Not Available Not Available Not Available Not Available Not Available Not Available Not Available Not Available Not Available NOT HISPANIC OR LATINO NO YES Not Available YES TCGA-91-6830-F32595 557AFAED-4E1C-41AA-BFA7-0BEFFDF7227D 4 Not Available 18 Not Applicable 60 18 Unknown Scheduled Follow-up Submission Unknown Unknown YES 6 Distant Metastasis YES Not Available Unknown NO Unknown Biopsy with Histologic Confirmation NO Alive 2012 FEMALE Lung Adenocarcinoma- Not Otherwise Specified (NOS) No C34.1 8140/3 C34.1 YES Not Available NO Not Available Not Available Not Available 7 Not Available Not Available Not Available Not Available Not Available Not Available Not Available Not Available Not Available Not Available 12 No 6830 Not Available WITH TUMOR Not Available Not Available Not Available Not Available Not Available Not Available Not Available WHITE Not Available Distant site TCGA-91-6830-R32598 C7C5B07C-450D-4B29-8A87-B339547AAF1D Not Available 4 60 60 Unknown 6 1 2000 NO External Not Applicable Not Available Not Available cGy 2012 Not Available Not Applicable Not Applicable Not Applicable Not Applicable Not Applicable Not Applicable Not Applicable Not Applicable Not Applicable Stage IIA Not Applicable Not Applicable Not Applicable 5th Not Applicable Not Applicable Not Applicable MX N1 T1 2002 NO YES 91 4 Lung Alive 2011 2002 1990

TCGA-55-A493 28 Alive T2a N0 M0 Stage IB 54 R-Upper Not Applicable TCGA-55-A493 E43A2B72-35C6-4466-9007-CE96CAF77EA8 21 -20027 Not Applicable 0 28 Not Available Lung Adenocarcinoma 102 Not Evaluated Not Available NO Not Available Not Available Not Available NO Not Available NOT HISPANIC OR LATINO FEMALE Lung Adenocarcinoma- Not Otherwise Specified (NOS) No C34.1 8140/3 C34.1 YES Not Evaluated NO Not Available Not Available Unknown 2 Not Available Not Available Not Available Not Available Not Available Not Available Not Available Not Available Not Available NO 53 No A493 Not Evaluated TUMOR FREE 71 2 Unknown 65 2 Unknown NO WHITE Unknown R0 Not Applicable Not Applicable Not Applicable Not Applicable Not Applicable Not Applicable Not Applicable Not Applicable Not Applicable Stage IB Not Applicable Not Applicable Not Applicable 7th Not Applicable Not Applicable Not Applicable M0 N0 T2a Not Available YES NO 55 2 Lung Alive 2013 2012 1977

TCGA-95-7562 87 Dead T2a N1 M0 Stage IIA 71 R-Lower Not Applicable TCGA-95-7562 193201a3-1447-47b1-bdf1-11ae0eb3b2f3 13 -26000 87 0 Not Available Not Available Lung Adenocarcinoma 57 TCGA-95-7562-D33261;TCGA-95-7562-D33262 1CB62B54-E04A-4A35-A0C0-5362A3F24D93;4F33410C-01F5-4F0E-A0A8-A416520BE0F7 Not Available;Not Available 26;26 81;81 60;60 CISPLATIN;ALIMTA Unknown;Unknown 6;6 Not Available;Not Available Not Available;Not Available Not Available;Not Available Not Available;Not Available Not Applicable;Not Applicable Not Available;Not Available Not Available;Not Available NO;NO Chemotherapy;Chemotherapy Not Available;Not Available Not Available;Not Available Not Available;Not Available YES;YES 2012;2012 1 Not Available Not Available Not Available Not Available Not Available Not Available Not Available NOT HISPANIC OR LATINO Not Available Not Available Not Available Not Available TCGA-95-7562-F32832 CCBA82F1-C522-4EF5-B5BB-A1F58E29ED89 13 Not Applicable Not Applicable 87 Not Available Not Applicable 1 Scheduled Follow-up Submission Not Evaluated Not Available NO 6 Not Available NO Post-Adjuvant Therapy TUMOR FREE YES Not Evaluated Not Available NO Dead 2012 MALE Lung Adenocarcinoma- Not Otherwise Specified (NOS) No C34.3 8140/3 C34.3 YES Not Available Not Available Not Available Not Available Not Available 6 Not Available Not Available Not Available Not Available Not Available Not Available Not Available Not Available Not Available NO 10 Yes, History of Prior Malignancy 7562 Post-Adjuvant Therapy TUMOR FREE 72 90 YES 76 80 Not Evaluated YES BLACK OR AFRICAN AMERICAN NO RX Not Applicable Not Applicable Not Applicable Not Applicable Not Applicable Not Applicable Not Applicable Not Applicable Not Applicable Stage IIA Not Applicable Not Applicable Not Applicable 7th Not Applicable Not Applicable Not Applicable M0 N1 T2a 1983 YES NO 95 3 Lung Dead 2012 2011 1973

TCGA-50-5932 1235 Dead T2 N1 M0 Stage IIB 75 R-Upper Not Applicable TCGA-50-5932 ebcba7f2-ce13-4bae-97cd-91a6b1dcd465 2 -27454 1235 0 1091 Not Available Lung Adenocarcinoma Not Available Not Available Not Available Not Available Not Available Not Available Not Available Not Available Not Available NOT HISPANIC OR LATINO YES NO NO NO TCGA-50-5932-F32080 aa578f39-0f94-41df-ad90-1a68f7a7fb43 16 Not Available Not Available 1235 Not Available 1090 Not Available Scheduled Follow-up Submission Progressive Disease Not Available NO 5 Locoregional Recurrence YES Not Available WITH TUMOR NO Progressive Disease Biopsy with Histologic Confirmation;Convincing Imaging NO Dead 2012 MALE Lung Adenocarcinoma- Not Otherwise Specified (NOS) No C34.1 8140/3 C34.1 YES Not Available Not Available Not Available Not Available Central Lung 7 Not Available Not Available Not Available Not Available Not Available Not Available Not Available Not Available Not Available Not Available Not Available Yes 5932 Not Available WITH TUMOR Not Available Not Available Not Available Not Available Not Available Not Available Not Available WHITE Not Available Not Available Not Applicable Not Applicable Not Applicable Not Applicable Not Applicable Not Applicable Not Applicable Not Applicable Not Applicable Stage IIB Not Applicable Not Applicable Not Applicable 5th Not Applicable Not Applicable Not Applicable M0 N1 T2 Not Available NO YES 50 Not Available Lung Dead 2011 2001 Not Available

TCGA-55-1596 2065 Alive T2 N1 M0 Stage IIB 55 R-Upper Not Applicable TCGA-55-1596 199386c2-bb53-4fad-a1b6-59ab216a4a50 13 -20381 Not Applicable 0 1375 Not Available Lung Adenocarcinoma Not Available TCGA-55-1596-D40545;TCGA-55-1596-D40546 D3AC7328-D4A7-4F72-86F7-07379246F800;5871BBDC-FC66-413D-8DE6-E44CB7DACA4D Not Available;Not Available 22;22 Not Available;Not Available 34;34 Carboplatin;Taxol Complete Response;Complete Response 2;2 Not Available;Not Available Not Available;Not Available Not Available;Not Available Not Available;Not Available Not Applicable;Not Applicable Not Available;Not Available Not Available;Not Available NO;NO Chemotherapy;Chemotherapy Not Available;Not Available Not Available;Not Available Not Available;Not Available NO;NO 2013;2013 Not Available Not Available Not Available Not Available Not Available Not Available Not Available Not Available NOT HISPANIC OR LATINO Not Available Not Available Not Available Not Available TCGA-55-1596-F40544 123C8E70-9662-45C2-80BF-E22343435AD4 22 Not Applicable Not Applicable Not Applicable 2065 Not Applicable Not Available Scheduled Follow-up Submission Complete Remission/Response Not Available NO 2 Not Available NO Not Available TUMOR FREE YES Complete Remission/Response Not Available NO Alive 2013 MALE Lung Adenocarcinoma- Not Otherwise Specified (NOS) No C34.1 8140/3 C34.1 YES Not Available Not Available Not Available Not Available Not Available 9 Not Available Not Available Not Available Not Available Not Available Not Available Not Available Not Available Not Available Not Available 50 No 1596 Not Available TUMOR FREE Not Available Not Available Not Available Not Available Not Available Not Available Not Available ASIAN Not Available R0 Not Applicable Not Applicable Not Applicable Not Applicable Not Applicable Not Applicable Not Applicable Not Applicable Not Applicable Stage IIB Not Applicable Not Applicable Not Applicable 6th Not Applicable Not Applicable Not Applicable M0 N1 T2 Not Available NO YES 55 2 Lung Alive 2010 2004 1979

TCGA-05-4425 669 Alive T2 N0 M1 Stage IV 70 R-Middle Not Applicable TCGA-05-4425 69b23036-add9-42c9-941f-02fb74d9b08c 22 -25902 Not Applicable 0 669 Not Available Lung Adenocarcinoma Not Available TCGA-05-4425-D36541;TCGA-05-4425-D36542 9F72525F-2709-42EF-831A-25435428F054;E3F50215-27BD-43FC-BD2F-C7C06D44035E Not Available;Not Available 5;5 153;153 31;31 Cisplatin;Vinorelbine Unknown;Unknown 12;12 Not Available;Not Available Not Available;Not Available Not Available;Not Available Not Available;Not Available Not Applicable;Not Applicable Not Available;Not Available Not Available;Not Available NO;NO Chemotherapy;Chemotherapy Not Available;Not Available Not Available;Not Available Not Available;Not Available NO;NO 2012;2012 Not Available Not Available Not Available Not Available Not Available Not Available Not Available Not Available Not Available FEMALE Lung Adenocarcinoma Mixed Subtype No C34.2 8255/3 C34.2 YES Not Available Not Available Not Available Not Available Not Available 7 Not Available Not Available Not Available Not Available Not Available Not Available Not Available Not Available Not Available Not Available 28 No 4425 Not Available Not Available Not Available Not Available Not Available Not Available Not Available Not Available Not Available Not Available Not Available R1 Not Applicable Not Applicable Not Applicable Not Applicable Not Applicable Not Applicable Not Applicable Not Applicable Not Applicable Stage IV Not Applicable Not Applicable Not Applicable 6th Not Applicable Not Applicable Not Applicable M1 N0 T2 Not Available NO YES 05 3 Lung Alive 2010 2008 Not Available

TCGA-69-8254 409 Alive T2b 85 R-Lower Not Applicable TCGA-69-8254 f0c94f68-bfd9-45cb-b4fd-8bb1ae4e9a2b 4 -31134 Not Applicable 0 77 Not Available Lung Adenocarcinoma Not Available Not Available Not Available YES Other Not Available Not Available NO Not Available NOT HISPANIC OR LATINO Not Available Not Available Not Available Not Available TCGA-69-8254-F41950 F1B78CC3-B120-4814-91CD-93C4CD359BEA 5 Not Applicable Not Applicable Not Applicable 409 Not Applicable 0 Scheduled Follow-up Submission Complete Remission/Response Not Available NO 4 Not Available NO Preoperative TUMOR FREE NO Complete Remission/Response Not Available NO Alive 2013 MALE Lung Adenocarcinoma Mixed Subtype No C34.3 8255/3 C34.3 YES 80 NO Not Available Not Available Not Available 6 Not Available Not Available Not Available Not Available Not Available Not Available Not Available Not Available Not Available NO 40 No 8254 Other TUMOR FREE Not Available Not Available NO Not Available 62 Complete Remission/Response YES WHITE NO Not Available Not Applicable Not Applicable Not Applicable Not Applicable Not Applicable Not Applicable Not Applicable Not Applicable Not Applicable Not Available Not Applicable Not Applicable Not Applicable 7th Not Applicable Not Applicable Not Applicable Not Available Not Available T2b 1989 YES NO 69 3 Lung Alive 2012 2012 Not Available

TCGA-55-7573 487 Alive T1b N0 MX Stage IA 72 R-Upper Not Applicable TCGA-55-7573 8e41153b-9faf-45c3-8397-8a428ef5c0d5 11 -26302 Not Applicable 0 4 Not Available Lung Adenocarcinoma 51 1 Not Available NO Not Available Not Available Not Available NO Not Available NOT HISPANIC OR LATINO Not Available Not Available Not Available Not Available TCGA-55-7573-F46657 483D103F-ADB6-45F2-991D-ED029568C19C 13 Not Applicable Not Applicable Not Applicable 487 Not Applicable Unknown Scheduled Follow-up Submission Complete Remission/Response Unknown NO 8 Not Available NO Unknown TUMOR FREE NO Complete Remission/Response Not Available NO Alive 2013 FEMALE Lung Adenocarcinoma- Not Otherwise Specified (NOS) No C34.1 8140/3 C34.1 YES 90 NO Not Available Not Available Central Lung 1 Not Available Not Available Not Available Not Available Not Available Not Available Not Available Not Available Not Available Not Available Not Available Yes 7573 Preoperative TUMOR FREE 119 134 Not Available 115 130 Not Available YES WHITE Not Available Not Available Not Applicable Not Applicable Not Applicable Not Applicable Not Applicable Not Applicable Not Applicable Not Applicable Not Applicable Stage IA Not Applicable Not Applicable Not Applicable 7th Not Applicable Not Applicable Not Applicable MX N0 T1b 1983 YES NO 55 3 Lung Alive 2012 2011 Not Available

TCGA-95-A4VN 553 Alive T2a N1 M0 Stage IIA 62 R-Lower Not Applicable TCGA-95-A4VN 389F78A3-FA0B-406C-9434-AA0298341A8C 22 -22927 Not Applicable 0 142 Not Available Lung Adenocarcinoma 50 Not Available Not Available YES Not Available Not Available Not Available NO Not Available NOT HISPANIC OR LATINO Not Available Not Available Not Available Not Available TCGA-95-A4VN-F58501 878D540E-C5BA-45D0-BC56-66F85D659549 11 Not Applicable Not Applicable Not Applicable 553 Not Applicable 1 Scheduled Follow-up Submission Stable Disease Not Evaluated NO 4 Not Available NO Pre-Adjuvant Therapy TUMOR FREE NO Stable Disease Not Available NO Alive 2014 FEMALE Lung Solid Pattern Predominant Adenocarcinoma No C34.3 8140/3 C34.3 YES Not Available NO Not Available Not Available Not Available 2 Not Available Not Available Not Available Not Available Not Available Not Available Not Available Not Available Not Available NO 42 Yes, History of Prior Malignancy A4VN Not Available TUMOR FREE 65 67 NO 60 58 Stable Disease YES WHITE NO R0 Not Applicable Not Applicable Not Applicable Not Applicable Not Applicable Not Applicable Not Applicable Not Applicable Not Applicable Stage IIA Not Applicable Not Applicable Not Applicable 7th Not Applicable Not Applicable Not Applicable M0 N1 T2a 2007 YES NO 95 4 Lung Alive 2013 2012 1965

TCGA-44-8119 285 Alive T3 N0 M0 Stage IIB 73 R-Upper Not Applicable TCGA-44-8119 230dc42d-681c-4e31-9d2e-c724e61829fb 31 -27003 Not Applicable 0 99 Not Available Lung Adenocarcinoma 40 0 Not Available Unknown Not Available Not Available Not Available Unknown Not Available NOT HISPANIC OR LATINO Not Available Not Available NO Not Available TCGA-44-8119-F38976 B83A8367-B557-44BB-9319-F428D59570DE 8 Not Applicable Not Applicable Not Applicable 285 Not Applicable 0 Scheduled Follow-up Submission Complete Remission/Response Not Available NO 1 Not Available NO Preoperative TUMOR FREE NO Complete Remission/Response Not Available NO Alive 2013 MALE Lung Adenocarcinoma- Not Otherwise Specified (NOS) No C34.1 8140/3 C34.1 YES Not Available Unknown Not Available Not Available Not Available 5 Not Available Not Available Not Available Not Available Not Available Not Available Not Available Not Available Not Available NO 50 Yes, History of Prior Malignancy 8119 Preoperative TUMOR FREE 74 59 NO 73 55 Complete Remission/Response YES WHITE NO Not Available Not Applicable Not Applicable Not Applicable Not Applicable Not Applicable Not Applicable Not Applicable Not Applicable Not Applicable Stage IIB Not Applicable Not Applicable Not Applicable 7th Not Applicable Not Applicable Not Applicable M0 N0 T3 Not Available YES NO 44 2 Lung Alive 2012 2012 1961

TCGA-71-8520 210 Dead T2 N0 M0 Stage IB 60 L-Upper Not Applicable TCGA-71-8520 37884d39-64e8-4242-b6bd-4c0a1daa21cd 15 -21921 Not Applicable 0 3 Not Available Lung Adenocarcinoma Not Available Not Available Not Available Not Available Not Available Not Available Not Available Not Available Not Available NOT HISPANIC OR LATINO NO NO NO Not Available TCGA-71-8520-F70925 31390530-007C-4160-9E08-4A980480B334 10 Not Available Not Available 210 Not Available 179 Not Available Scheduled Follow-up Submission Progressive Disease Not Available NO 3 Locoregional Recurrence YES Not Available WITH TUMOR NO Complete Remission/Response Not Available YES Dead 2015 FEMALE Lung Adenocarcinoma- Not Otherwise Specified (NOS) No C34.1 8140/3 C34.1 YES 40 YES NO Not Available Not Available 4 Not Available Not Available Not Available Not Available Not Available Not Available Not Available Not Available Not Available NO Not Available No 8520 Preoperative TUMOR FREE Not Available Not Available NO Not Available Not Available Complete Remission/Response NO ASIAN NO Regional site TCGA-71-8520-R70926 E036E361-397E-4FDB-A92D-039E27A04A17 Not Available 10 80 51 Radiographic Progressive Disease 3 22 44 NO External Not Applicable Not Available Not Available Gy 2015 R0 Not Applicable Not Applicable Not Applicable Not Applicable Not Applicable Not Applicable Not Applicable Not Applicable Not Applicable Stage IB Not Applicable Not Applicable Not Applicable 7th Not Applicable Not Applicable Not Applicable M0 N0 T2 Not Available YES NO 71 1 Lung Alive 2013 2011 Not Available

TCGA-69-A59K 591 Alive T3 N0 M0 Stage IIB 60 R-Upper Not Applicable TCGA-69-A59K 67FA3E41-C7C6-44C5-9E67-6BCB2715AACC 18 -22093 Not Applicable 0 214 Not Available Lung Adenocarcinoma 75 TCGA-69-A59K-D40359;TCGA-69-A59K-D40360 095F50E9-408C-4353-B7D6-339A2BBC1895;EE6D5CA4-FDD0-45B6-A767-D5B34DE311ED Not Available;Not Available 18;18 135;135 72;72 cisplatin;pemetrexed Stable Disease;Stable Disease 2;2 Not Available;Not Available Not Available;Not Available Not Available;Not Available Not Available;Not Available Not Applicable;Not Applicable Not Available;Not Available Not Available;Not Available NO;NO Chemotherapy;Chemotherapy Not Available;Not Available Not Available;Not Available Not Available;Not Available NO;NO 2013;2013 2 Not Available YES Not Available Not Available Not Available NO Not Available NOT HISPANIC OR LATINO Not Available;Not Available Not Available;Not Available Not Available;Not Available Not Available;Not Available TCGA-69-A59K-F56712;TCGA-69-A59K-F57660 5E0B884C-C335-4788-8FFF-1F4CF6565627;6D298071-0FB0-40B6-A0D9-43F6B40F136C 19;19 Not Applicable;Not Applicable Not Applicable;Not Applicable Not Applicable;Not Applicable 591;522 Not Applicable;Not Applicable Not Available;Not Available Scheduled Follow-up Submission;Scheduled Follow-up Submission Not Applicable;Not Applicable Not Available;Not Available NO;NO 2;3 Not Available;Not Available NO;NO Not Available;Not Available TUMOR FREE;TUMOR FREE YES;YES Stable Disease;Stable Disease Not Available;Not Available NO;NO Alive;Alive 2014;2014 FEMALE Lung Adenocarcinoma Mixed Subtype No C34.1 8255/3 C34.1 YES Not Available Unknown Not Available Not Available Unknown 2 Not Available Not Available Not Available Not Available Not Available Not Available Not Available Not Available Not Available NO 25 No A59K Pre-Adjuvant Therapy TUMOR FREE Not Available 120 YES Not Available 118 Stable Disease YES BLACK OR AFRICAN AMERICAN NO Not Evaluated Not Applicable Not Applicable Not Applicable Not Applicable Not Applicable Not Applicable Not Applicable Not Applicable Not Applicable Stage IIB Not Applicable Not Applicable Not Applicable 7th Not Applicable Not Applicable Not Applicable M0 N0 T3 2012 YES NO 69 4 Lung Alive 2013 2012 1999

TCGA-44-6144 723 Alive T1a N0 M0 Stage IA 58 R-Upper Not Applicable TCGA-44-6144 22302e92-fdfa-462a-9249-efdaa26ee5ed 7 -21283 Not Applicable 0 9 9 Lung Adenocarcinoma 82 0 Not Available Not Available Not Available Not Available Not Available Not Available Not Available NOT HISPANIC OR LATINO Not Available;YES Not Available;NO Not Available;NO Not Available;NO TCGA-44-6144-F28683;TCGA-44-6144-F39034 4731cc65-66df-442b-ba6c-c440bd44e8b0;7EED8AFF-FF01-4647-B239-80D021B2BD52 28;9 Not Available;Not Available Not Available;Not Available Not Applicable;Not Applicable 407;723 399;399 Not Available;Not Available Scheduled Follow-up Submission;Scheduled Follow-up Submission Progressive Disease;Progressive Disease Not Available;70 Not Available;NO 2;1 Not Available;Locoregional Recurrence;Distant Metastasis YES;YES Not Available;Other WITH TUMOR;WITH TUMOR NO;NO Complete Remission/Response;Complete Remission/Response Not Available;Biopsy with Histologic Confirmation;Convincing Imaging NO;NO Alive;Alive 2012;2013 MALE Lung Adenocarcinoma- Not Otherwise Specified (NOS) No C34.1 8140/3 C34.1 YES Not Available Not Available Not Available Not Available Not Available 6 Not Available Not Available Not Available Not Available Not Available Not Available Not Available Not Available Not Available Not Available 67.5 No 6144 Preoperative TUMOR FREE 97 77 Not Available 87 63 Not Available YES WHITE Not Available RX Not Applicable Not Applicable Not Applicable Not Applicable Not Applicable Not Applicable Not Applicable Not Applicable Not Applicable Stage IA Not Applicable Not Applicable Not Applicable 7th Not Applicable Not Applicable Not Applicable M0 N0 T1a Not Available YES NO 44 2 Lung Alive 2011 2011 1968

TCGA-MP-A4TD 307 Dead T2 N2 M0 Stage IIIA 71 L-Lower Not Applicable TCGA-MP-A4TD 383D1253-349F-43B3-BEBD-9F1D1CF14B3D 8 -25983 307 0 Not Available Not Available Lung Adenocarcinoma 61 TCGA-MP-A4TD-D41400;TCGA-MP-A4TD-D41401 151B0D62-C2EE-442B-8FC7-DDCE9131ABBD;429B9434-707A-41B3-A4B6-07DA60A412A3 Not Available;Not Available 18;18 165;165 75;75 Cisplatin;Gemcitabine Complete Response;Complete Response 3;3 Not Available;Not Available Not Available;Not Available Not Available;Not Available Not Available;Not Available Not Applicable;Not Applicable Not Available;Not Available Not Available;Not Available NO;NO Chemotherapy;Chemotherapy Not Available;Not Available Not Available;Not Available Not Available;Not Available NO;NO 2013;2013 0 Not Available NO Not Available Not Available Not Available NO Not Available Unknown MALE Lung Adenocarcinoma- Not Otherwise Specified (NOS) No C34.3 8140/3 C34.3 YES Not Evaluated NO Not Available Not Available Peripheral Lung 5 YES Unknown 226 NO Not Available Not Available Not Available Locoregional Recurrence Convincing Imaging YES 20 Yes, History of Prior Malignancy A4TD Post-Adjuvant Therapy Unknown Not Available 92 YES Not Available 93 Complete Remission/Response YES WHITE NO RX Not Applicable Not Applicable Not Applicable Not Applicable Not Applicable Not Applicable Not Applicable Not Applicable Not Applicable Stage IIIA Not Applicable Not Applicable Not Applicable 6th Not Applicable Not Applicable Not Applicable M0 N2 T2 1990 NO YES MP 3 Lung Dead 2013 2008 Not Available

TCGA-64-5781 1559 Alive T2 N0 M0 Stage IB 55 R-Upper Not Applicable TCGA-64-5781 41c4c704-825e-49f5-9d54-dbd7d30725e0 3 -20219 Not Applicable 0 1202 Not Available Lung Adenocarcinoma 73 TCGA-64-5781-D11407;TCGA-64-5781-D11405;TCGA-64-5781-D11406;TCGA-64-5781-D31491;TCGA-64-5781-D31492;TCGA-64-5781-D31493 92010938-ac4a-4ab1-b25e-dba6632b92ba;4ffd6d8f-3ded-49b0-a532-d0fb97ef1033;83cf163f-43dc-4a75-9ed6-a433a2226488;04bd06de-39e1-47d6-bdb4-de3aeccf9f63;86ff15d3-47d0-47e5-ad61-cded2ed6345d;acadc104-cf2b-4ac0-8598-f4e35edbb3d1 Not Available;Not Available;Not Available;Not Available;Not Available;Not Available 3;3;3;3;3;3 187;215;215;215;215;187 159;101;101;131;131;159 Bevacizumab;Cisplatin;Gemcitabine;Cisplatin;Gemcitabine;Bevacizumab Not Available;Not Available;Not Available;Complete Response;Complete Response;Complete Response 5;5;5;5;5;5 3;4;4;Not Available;Not Available;Not Available 15;75;1250;Not Available;Not Available;Not Available mg/kg;mg/m2;mg/m2;Not Available;Not Available;Not Available PROGRESSION;ADJUVANT;PROGRESSION;Not Available;Not Available;Not Available Not Applicable;Not Applicable;Not Applicable;Not Applicable;Not Applicable;Not Applicable 1;1;1;Not Available;Not Available;Not Available IV;IV;IV;Not Available;Not Available;Not Available NO;NO;NO;NO;NO;NO Chemotherapy;Chemotherapy;Chemotherapy;Chemotherapy;Chemotherapy;Chemotherapy Not Available;Not Available;Not Available;Not Available;Not Available;Not Available Not Available;Not Available;Not Available;Not Available;Not Available;Not Available Not Available;Not Available;Not Available;Not Available;Not Available;Not Available Not Available;Not Available;Not Available;NO;NO;NO 2011;2011;2011;2012;2012;2012 0 Not Available Not Available Not Available Not Available Not Available Not Available Not Available NOT HISPANIC OR LATINO YES;NO NO;NO NO;NO YES;YES TCGA-64-5781-F11404;TCGA-64-5781-F31490 2d57acf6-f8b7-4de5-bc21-d804fc3b145d;295e781b-ad5c-445b-8dbc-9d12b2b6495d 3;3 Not Available;Not Available 112;112 Not Applicable;Not Applicable 1202;1559 96;96 0;0 Not Available;Additional New Tumor Event Complete Remission/Response;Complete Remission/Response Not Available;Unknown Not Available;Not Available 5;5 Not Available;Distant Metastasis YES;YES Post-Adjuvant Therapy;Unknown TUMOR FREE;TUMOR FREE NO;NO Progressive Disease;Progressive Disease Not Available;Not Available NO;NO Alive;Alive 2011;2012 FEMALE Lung Adenocarcinoma Mixed Subtype No C34.1 8255/3 C34.1 YES Not Available Not Available Not Available Not Available Central Lung 5 Not Available Not Available Not Available Not Available Not Available Not Available Not Available Not Available Not Available Not Available 25 No 5781 Post-Adjuvant Therapy TUMOR FREE Not Available Not Available Not Available 61 77 Not Available YES WHITE Not Available R0 Not Applicable Not Applicable Not Applicable Not Applicable Not Applicable Not Applicable Not Applicable Not Applicable Not Applicable Stage IB Not Applicable Not Applicable Not Applicable 6th Not Applicable Not Applicable Not Applicable M0 N0 T2 2007 NO YES 64 4 Lung Alive 2011 2007 1982

TCGA-75-6214 1115 Dead T2 N2 M0 Stage IIIA Not Available R-Upper Not Applicable TCGA-75-6214 32592562-11fa-435a-b40b-2b242bae53aa 12 Not Available Not Applicable Not Available Not Available Not Available Lung Adenocarcinoma Not Available 1 Not Available Not Available Not Available Not Available Not Available Not Available Not Available Not Available Not Available;NO Not Available;YES Not Available;Not Available Not Available;YES TCGA-75-6214-F15860;TCGA-75-6214-F72038 17e757bc-f78c-4be4-8ece-1109e163916d;8103EC55-5AAD-42CC-988D-A617888D510B 12;2 Not Available;Not Available Not Available;524 Not Applicable;1115 Not Available;Not Available Not Available;419 0;0 Not Available;Scheduled Follow-up Submission Progressive Disease;Progressive Disease Not Available;Not Evaluated Not Available;NO 8;4 Not Available;Distant Metastasis YES;YES Adjuvant therapy;Post-Adjuvant Therapy Not Available;WITH TUMOR NO;NO Complete Remission/Response;Complete Remission/Response Not Available;Not Available NO;NO Alive;Dead 2011;2015 FEMALE Lung Adenocarcinoma- Not Otherwise Specified (NOS) No C34.1 8140/3 C34.1 YES Not Available Not Available Not Available Not Available Peripheral Lung 8 Not Available Not Available Not Available Not Available Not Available Not Available Not Available Not Available Not Available Not Available 40 No 6214 Preoperative WITH TUMOR Not Available Not Available Not Available Not Available Not Available Not Available Not Available Not Available Not Available R0 Not Applicable Not Applicable Not Applicable Not Applicable Not Applicable Not Applicable Not Applicable Not Applicable Not Applicable Stage IIIA Not Applicable Not Applicable Not Applicable 6th Not Applicable Not Applicable Not Applicable M0 N2 T2 Not Available NO YES 75 2 Lung Alive 2011 Not Available 1970

TCGA-67-4679 448 Alive T3 N0 M0 69 R-Lower Not Applicable TCGA-67-4679 3e635902-5310-4406-8b4d-a238ce382639 21 -25202 Not Applicable 0 448 Not Available Lung Adenocarcinoma Not Available Not Available Not Available NO Not Available Not Available Not Available NO Not Available NOT HISPANIC OR LATINO MALE Lung Adenocarcinoma- Not Otherwise Specified (NOS) No C34.3 8140/3 C34.3 YES Not Available NO Not Available Not Available Central Lung 1 Not Available Not Available Not Available Not Available Not Available Not Available Not Available Not Available Not Available Not Available Not Available No 4679 Not Available TUMOR FREE Not Available Not Available Not Available Not Available Not Available Not Available Not Available WHITE Not Available R0 Not Applicable Not Applicable Not Applicable Not Applicable Not Applicable Not Applicable Not Applicable Not Applicable Not Applicable Discrepancy Not Applicable Not Applicable Not Applicable Not Available Not Applicable Not Applicable Not Applicable M0 N0 T3 Not Available YES NO 67 4 Lung Alive 2011 2009 Not Available

TCGA-05-4249 1523 Alive T2 N0 M0 Stage IB 67 R-Lower Not Applicable TCGA-05-4249 4addf05f-3668-4b3f-a17f-c0227329ca52 22 -24532 Not Applicable 0 1158 Not Available Lung Adenocarcinoma Not Available Not Available Not Available Not Available Not Available Not Available Not Available Not Available Not Available Not Available Not Available Not Available Not Available Not Available TCGA-05-4249-F36327 485963A2-753D-4E86-B3DF-E3F66189ABEE 29 Not Applicable Not Applicable Not Applicable 1523 Not Applicable Not Available Scheduled Follow-up Submission Complete Remission/Response Not Available NO 10 Not Available NO Not Available TUMOR FREE NO Not Applicable Not Available NO Alive 2012 MALE Lung Adenocarcinoma- Not Otherwise Specified (NOS) No C34.3 8140/3 C34.3 YES Not Available Not Available Not Available Not Available Peripheral Lung 7 Not Available Not Available Not Available Not Available Not Available Not Available Not Available Not Available Not Available Not Available 52 No 4249 Not Available TUMOR FREE Not Available Not Available Not Available Not Available Not Available Not Available Not Available Not Available Not Available R0 Not Applicable Not Applicable Not Applicable Not Applicable Not Applicable Not Applicable Not Applicable Not Applicable Not Applicable Stage IB Not Applicable Not Applicable Not Applicable 6th Not Applicable Not Applicable Not Applicable M0 N0 T2 Not Available NO YES 05 3 Lung Alive 2010 2007 Not Available

TCGA-86-8075 694 Dead T2 N0 M0 Stage IB 66 L-Upper Not Applicable TCGA-86-8075 8869000d-09d7-465a-ac32-54d1935c43e7 16 -24402 Not Applicable 0 43 Not Available Lung Adenocarcinoma Not Available TCGA-86-8075-D41680;TCGA-86-8075-D41681;TCGA-86-8075-D41682 3C971D88-EA87-4F2F-893C-B7BB558C402E;7E6A245F-8FC9-4F05-BA87-C6FC12A202EB;9C62E82D-EABF-452D-9CE1-6F3146D45E6C Not Available;Not Available;Not Available 25;25;25 364;364;Not Available 201;201;388 Etoposide;Cisplatin;Gefitinib Complete Response;Complete Response;Not Applicable 3;3;3 Not Available;Not Available;Not Available Not Available;Not Available;Not Available Not Available;Not Available;Not Available Not Available;Not Available;Not Available Not Applicable;Not Applicable;Not Applicable Not Available;Not Available;Not Available Not Available;Not Available;Not Available NO;NO;YES Chemotherapy;Chemotherapy;Targeted Molecular therapy Not Available;Not Available;Not Available Not Available;Not Available;Not Available Not Available;Not Available;Not Available NO;NO;NO 2013;2013;2013 2 Not Available NO Not Available Not Available Not Available NO Not Available NOT HISPANIC OR LATINO YES;Not Available NO;Not Available NO;Not Available NO;Not Available TCGA-86-8075-F41679;TCGA-86-8075-F63521 686D13D9-B93A-4157-9E8D-B441EA26F6D0;B67D4CCB-D243-4337-B31E-BE40BFD0ED38 25;12 Not Available;Not Applicable Not Available;Not Applicable Not Applicable;694 479;Not Available 199;Not Applicable 1;1 Scheduled Follow-up Submission;Scheduled Follow-up Submission Not Applicable;Complete Remission/Response 90;90 NO;NO 3;8 Locoregional Recurrence;Not Available YES;NO Pre-Adjuvant Therapy;Preoperative TUMOR FREE;TUMOR FREE NO;NO Complete Remission/Response;Complete Remission/Response Biopsy with Histologic Confirmation;Not Available NO;NO Alive;Dead 2013;2014 FEMALE Lung Adenocarcinoma- Not Otherwise Specified (NOS) No C34.1 8140/3 C34.1 YES 80 NO Not Available Not Available Peripheral Lung 5 Not Available Not Available Not Available Not Available Not Available Not Available Not Available Not Available Not Available Unknown Not Available No 8075 Preoperative TUMOR FREE Not Available Not Available Unknown Not Available Not Available Unknown NO WHITE Unknown R0 Not Applicable Not Applicable Not Applicable Not Applicable Not Applicable Not Applicable Not Applicable Not Applicable Not Applicable Stage IB Not Applicable Not Applicable Not Applicable 7th Not Applicable Not Applicable Not Applicable M0 N0 T2 Not Available YES NO 86 1 Lung Alive 2012 2011 Not Available

TCGA-97-7937 564 Alive T2a N0 MX Stage IB 65 L-Upper Not Applicable TCGA-97-7937 fcfe0b53-dfc1-42fa-9efc-2b2ff259297e 13 -23919 Not Applicable 0 181 Not Available Lung Adenocarcinoma 89 0 Not Available YES Not Available Not Available FISH YES Not Available NOT HISPANIC OR LATINO Not Available Not Available NO Not Available TCGA-97-7937-F33767 483E201C-601E-4BEC-9BD0-EF8AFFBA9552 12 Not Applicable Not Applicable Not Applicable 564 Not Applicable Not Available Scheduled Follow-up Submission Not Available Not Available NO 9 Not Available NO Not Available TUMOR FREE NO Stable Disease Not Available NO Alive 2012 MALE Lung Micropapillary Adenocarcinoma No C34.1 8507/3 C34.1 YES Not Available YES NO Not Available Not Available 2 Not Available Not Available Not Available Not Available Not Available Not Available Not Available Not Available Not Available Not Available 35 No 7937 Preoperative Not Available Not Available Not Available Not Available 77 120 Not Available YES WHITE Not Available Not Available Not Applicable Not Applicable Not Applicable Not Applicable Not Applicable Not Applicable Not Applicable Not Applicable Not Applicable Stage IB Not Applicable Not Applicable Not Applicable 7th Not Applicable Not Applicable Not Applicable MX N0 T2a 1994 YES NO 97 3 Lung Alive 2012 2011 Not Available

TCGA-86-8279 949 Alive T2a N1 M0 Stage IIA 46 R-Upper Not Applicable TCGA-86-8279 2923e404-38f2-437a-b57e-23401fbe0273 6 -17032 Not Applicable 0 21 Not Available Lung Adenocarcinoma Not Available TCGA-86-8279-D41676;TCGA-86-8279-D41677 9F2065E1-C62F-4D2A-92CB-B1DB2F7963E2;233E4E3A-65B3-467B-B214-C52773DD2094 Not Available;Not Available 25;25 119;119 28;28 Etoposide;Cisplatin Complete Response;Complete Response 3;3 Not Available;Not Available Not Available;Not Available Not Available;Not Available Not Available;Not Available Not Applicable;Not Applicable Not Available;Not Available Not Available;Not Available NO;NO Chemotherapy;Chemotherapy Not Available;Not Available Not Available;Not Available Not Available;Not Available NO;NO 2013;2013 2 Not Available Not Available Not Available Not Available Not Available Not Available Not Available NOT HISPANIC OR LATINO Not Available;Not Available Not Available;Not Available Not Available;Not Available Not Available;Not Available TCGA-86-8279-F41673;TCGA-86-8279-F59731 56E63750-3C49-4C3E-8586-0497C56C4A45;2203355D-451D-42E6-A83A-A8D2CC685DC5 25;20 Not Applicable;Not Applicable Not Applicable;Not Applicable Not Applicable;Not Applicable 482;949 Not Applicable;Not Applicable 1;1 Scheduled Follow-up Submission;Scheduled Follow-up Submission Complete Remission/Response;Complete Remission/Response 90;90 NO;NO 3;5 Not Available;Not Available NO;NO Pre-Adjuvant Therapy;Preoperative TUMOR FREE;TUMOR FREE YES;YES Complete Remission/Response;Complete Remission/Response Not Available;Not Available YES;YES Alive;Alive 2013;2014 MALE Lung Adenocarcinoma- Not Otherwise Specified (NOS) No C34.1 8140/3 C34.1 YES 80 NO Not Available Not Available Peripheral Lung 6 Not Available Not Available Not Available Not Available Not Available Not Available Not Available Not Available Not Available Unknown Not Available No 8279 Preoperative TUMOR FREE Not Available Not Available YES Not Available Not Available Complete Remission/Response NO WHITE YES Primary Tumor Field TCGA-86-8279-R41678 8C483E9F-5DD2-4828-91A6-F81F0A420544 Not Available 25 208 139 Complete Response 3 33 66 NO External Not Applicable Not Available Not Available Gy 2013 R0 Not Applicable Not Applicable Not Applicable Not Applicable Not Applicable Not Applicable Not Applicable Not Applicable Not Applicable Stage IIA Not Applicable Not Applicable Not Applicable 7th Not Applicable Not Applicable Not Applicable M0 N1 T2a Not Available YES NO 86 1 Lung Alive 2012 2011 Not Available

TCGA-38-4632 1357 Dead T2 N1 M1 Stage IV 42 L-Upper Not Applicable TCGA-38-4632 875333ab-9048-462d-aaa2-693ad127e3cc 13 -15418 1357 0 Not Available Not Available Lung Adenocarcinoma Not Available TCGA-38-4632-D4840;TCGA-38-4632-D4844;TCGA-38-4632-D13822;TCGA-38-4632-D13824 77d7b5e2-ec9e-44d8-a8bb-fc29c602ae6a;54475225-34f4-4948-996f-e97089cd5a04;d1a79d21-2ed8-415a-aea3-af762a661683;e8d4b552-6c8f-4cfb-93cd-267605c6a506 Not Available;Not Available;Not Available;Not Available 13;13;14;14 802;1169;802;1169 679;1136;679;1127 gemcitabine;Carboplatin;Paclitaxel;CPT-11 Not Available;Not Available;Not Available;Not Available 12;12;7;7 6;2;6;2 Not Available;Not Available;Not Available;Not Available mg;mg;Not Available;Not Available RECURRENCE;PALLIATIVE;RECURRENCE;PALLIATIVE Not Applicable;Not Applicable;Not Applicable;Not Applicable LCCC 9831;3;LCCC 9831;3 IV;IV;IV;IV NO;NO;NO;NO Chemotherapy;Chemotherapy;Chemotherapy;Chemotherapy Not Available;Not Available;Not Available;Not Available Not Available;Not Available;Not Available;Not Available Not Available;Not Available;mg/m2;mg/m2 Not Available;Not Available;Not Available;Not Available 2010;2010;2011;2011 Not Available Not Available NO Not Available Not Available Not Available NO Not Available NOT HISPANIC OR LATINO YES;YES NO;YES NO;NO NO;NO TCGA-38-4632-F4839;TCGA-38-4632-F4842 ecb636f6-dc7d-48c5-9a00-02aec73f03b1;1fb434a3-8648-499c-8e36-97c1ea94dad2 13;13 Not Available;Not Available Not Available;Not Available 1357;1357 Not Available;Not Available 680;925 Not Available;Not Available Not Available;Not Available Stable Disease;Progressive Disease Not Available;Not Available Not Available;Not Available 12;12 Not Available;Not Available YES;YES Not Available;Not Available WITH TUMOR;WITH TUMOR NO;NO Complete Remission/Response;Complete Remission/Response Not Available;Not Available NO;NO Dead;Dead 2010;2010 MALE Lung Adenocarcinoma- Not Otherwise Specified (NOS) No C34.1 8140/3 C34.1 YES Not Available NO Not Available Not Available Peripheral Lung 12 Not Available Not Available Not Available Not Available Not Available Not Available Not Available Not Available Not Available Not Available 10 No 4632 Not Available WITH TUMOR Not Available Not Available Not Available Not Available Not Available Not Available NO BLACK OR AFRICAN AMERICAN Not Available Distant Recurrence;Distant site TCGA-38-4632-R4843;TCGA-38-4632-R40104 5dd66aad-2ab7-4f32-9a98-b640c47d392f;E3818E10-E37C-4DCF-881F-A1B668E6A6B7 3;Not Available 13;12 955;70 955;35 Not Available;Complete Response 12;2 10;22 2500;5800 NO;NO EXTERNAL BEAM;External Not Applicable;Not Applicable PALLIATIVE;Not Available Not Available;Not Available cGy;cGy 2010;2013 R0 Not Applicable Not Applicable Not Applicable Not Applicable Not Applicable Not Applicable Not Applicable Not Applicable Not Applicable Stage IV Not Applicable Not Applicable Not Applicable 5th Not Applicable Not Applicable Not Applicable M1 N1 T2 1991 NO YES 38 4 Lung Dead 2010 1998 Not Available

TCGA-64-5779 864 Alive T2 N2 M0 Stage IIIA 61 L-Lower Not Applicable TCGA-64-5779 1c58d9b8-17a2-4fc6-9898-fe6f47bd2c2a 2 -22305 Not Applicable 0 507 Not Available Lung Adenocarcinoma 100 TCGA-64-5779-D11375;TCGA-64-5779-D11376;TCGA-64-5779-D31475;TCGA-64-5779-D31476 c0d60743-bdf0-436e-9702-4e8ce35c248d;621a2148-00ec-4c00-a0bc-980442ab9522;d2824bc2-e94d-4926-9b6b-0600e519bb80;b55016e7-e7a7-430d-93e0-377e6fb2fb50 Not Available;Not Available;Not Available;Not Available 2;2;3;3 157;157;157;157 94;94;94;94 Cisplatin;Pemetrexed;Cisplatin;Pemetrexed Not Available;Not Available;Complete Response;Complete Response 5;5;5;5 04;04;Not Available;Not Available 75;500;Not Available;Not Available mg/m2;mg/m2;Not Available;Not Available ADJUVANT;ADJUVANT;Not Available;Not Available Not Applicable;Not Applicable;Not Applicable;Not Applicable 01;01;Not Available;Not Available IV;IV;Not Available;Not Available NO;NO;NO;NO Chemotherapy;Chemotherapy;Chemotherapy;Chemotherapy Not Available;Not Available;Not Available;Not Available Not Available;Not Available;Not Available;Not Available Not Available;Not Available;Not Available;Not Available Not Available;Not Available;YES;YES 2011;2011;2012;2012 0 Not Available Not Available Not Available Not Available Not Available Not Available Not Available NOT HISPANIC OR LATINO Not Available;NO Not Available;YES Not Available;Not Available Not Available;YES TCGA-64-5779-F11373;TCGA-64-5779-F31473 04f76938-5fba-43c0-98dc-09975c964e2b;e630e4b1-4907-4640-a3cf-9909d6695755 2;3 Not Applicable;Not Available Not Applicable;797 Not Applicable;Not Applicable 507;864 Not Applicable;795 0;0 Not Available;Additional New Tumor Event Stable Disease;Unknown Not Available;Unknown Not Available;Not Available 5;5 Not Available;Distant Metastasis NO;YES Post-Adjuvant Therapy;Post-Adjuvant Therapy Not Available;Unknown YES;YES Stable Disease;Discrepancy Not Available;Not Available YES;YES Alive;Alive 2011;2012 MALE Lung Acinar Adenocarcinoma No C34.3 8550/3 C34.3 YES Not Available Not Available Not Available Not Available Central Lung 5 Not Available Not Available Not Available Not Available Not Available Not Available Not Available Not Available Not Available Not Available 40 No 5779 Post-Adjuvant Therapy TUMOR FREE Not Available Not Available Not Available 94 100 Not Available YES WHITE Not Available Primary Tumor Field;Primary Tumor Field;Distant site TCGA-64-5779-R11374;TCGA-64-5779-R31474;TCGA-64-5779-R31477 0eb7ccdf-6dff-4dd1-802d-f035caf16d3a;92e10658-9cdc-4b28-a801-c37c70128b36;2e1ad728-abff-4e0d-9a8b-01a72cad7afc 01;01;02 2;17;17 241;241;864 Not Available;201;852 Not Available;Not Available;Not Available 5;4;4 28;28;09 Not Available;5040;2700 NO;NO;NO EXTERNAL BEAM;EXTERNAL BEAM;EXTERNAL BEAM Not Applicable;Not Applicable;Not Applicable ADJUVANT;ADJUVANT;RECURRENCE Not Available;Not Available;Not Available cGy;cGy;cGy 2011;2012;2012 R0 Not Applicable Not Applicable Not Applicable Not Applicable Not Applicable Not Applicable Not Applicable Not Applicable Not Applicable Stage IIIA Not Applicable Not Applicable Not Applicable 6th Not Applicable Not Applicable Not Applicable M0 N2 T2 2009 NO YES 64 4 Lung Alive 2011 2009 1969

TCGA-75-5146 2368 Alive T2 N0 M0 Stage IB Not Available R-Lower Not Applicable TCGA-75-5146 1d276b62-5e64-48fb-b2ce-5192b511fe37 6 Not Available Not Applicable Not Available Not Available Not Available Lung Adenocarcinoma Not Available Not Available Not Available NO Not Available Not Available Not Available NO Not Available Not Available Not Available;YES Not Available;NO Not Available;Not Available Not Available;Not Available TCGA-75-5146-F11744;TCGA-75-5146-F72030 d54f5d79-f71c-4fc5-8a86-be0e098fae39;A034F00F-B0BD-4792-8085-A036CD719983 6;2 Not Applicable;Not Available Not Applicable;Not Available Not Applicable;Not Applicable Not Available;2368 Not Applicable;1773 Not Available;Not Evaluated Not Available;Scheduled Follow-up Submission Complete Remission/Response;Unknown Not Available;Not Evaluated Not Available;NO 4;4 Not Available;New Primary Tumor NO;YES Adjuvant therapy;Not Available TUMOR FREE;TUMOR FREE NO;NO Complete Remission/Response;Complete Remission/Response Not Available;Not Available NO;NO Alive;Alive 2011;2015 MALE Lung Adenocarcinoma- Not Otherwise Specified (NOS) No C34.3 8252/3 C34.3 YES Not Available NO Not Available Not Available Peripheral Lung 4 Not Available Not Available Not Available Not Available Not Available Not Available Not Available Not Available Not Available Not Available Not Available No 5146 Other TUMOR FREE Not Available Not Available Not Available Not Available Not Available Not Available Not Available Not Available Not Available R0 Not Applicable Not Applicable Not Applicable Not Applicable Not Applicable Not Applicable Not Applicable Not Applicable Not Applicable Stage IB Not Applicable Not Applicable Not Applicable 6th Not Applicable Not Applicable Not Applicable M0 N0 T2 1987 NO YES 75 3 Lung Alive 2011 Not Available Not Available

TCGA-78-7150 666 Dead T2 N1 M0 Stage IIB 59 L-Lower Not Applicable TCGA-78-7150 a0e57b39-960e-4e46-8aa0-d0ee2fd9a7cc 28 -21891 666 0 Not Available Not Available Lung Adenocarcinoma Not Available TCGA-78-7150-D16967;TCGA-78-7150-D16965;TCGA-78-7150-D16966 c90077f8-6d30-4eaa-b791-2d8a389fdfbd;071743ff-ba4b-4208-8816-a453c02c436e;a4b1e4fb-299c-4c6f-9057-96dc3d724a7c Not Available;Not Available;Not Available 28;28;28 642;397;397 607;285;285 Paclitaxel;Carboplatin;Gemcitabine Not Available;Not Available;Not Available 9;9;9 2;5;5 140;615;1800 mg/m2;AUC;mg RECURRENCE;PALLIATIVE;PALLIATIVE Not Applicable;Not Applicable;Not Applicable 3;1;2 IV;IV;IV NO;NO;NO Chemotherapy;Chemotherapy;Chemotherapy Not Available;Not Available;Not Available 700;2475;14180 mg/m2;mg;mg/m2 Not Available;Not Available;Not Available 2011;2011;2011 0 Not Available NO Not Available Not Available Not Available NO Not Available Not Available YES YES NO YES TCGA-78-7150-F16962 09ad8f79-3e06-43f3-9c00-cd8c7e87bdcc 28 Not Available 164 666 Not Available 139 Not Available Scheduled Follow-up Submission Not Available Not Available Not Available 9 Not Available YES Not Available WITH TUMOR NO Not Available Not Available NO Dead 2011 MALE Lung Adenocarcinoma- Not Otherwise Specified (NOS) No C34.3 8140/3 C34.3 YES Not Available NO Not Available Not Available Central Lung 9 Not Available Not Available Not Available Not Available Not Available Not Available Not Available Not Available Not Available Not Available 37 No 7150 Preoperative WITH TUMOR Not Available Not Available Not Available Not Available Not Available Not Available Not Available WHITE Not Available Distant Recurrence;Distant Recurrence TCGA-78-7150-R16964;TCGA-78-7150-R16963 969f1a71-c4c4-48e8-8589-faa544fa5515;65e0a687-005d-4fb1-aa3b-fb9f84bb7dc5 2;1 28;28 546;189 531;185 Not Available;Not Available 9;9 10;5 2500;2000 NO;NO EXTERNAL BEAM;EXTERNAL BEAM Not Applicable;Not Applicable RECURRENCE;PALLIATIVE Not Available;Not Available cGy;cGy 2011;2011 R0 Not Applicable Not Applicable Not Applicable Not Applicable Not Applicable Not Applicable Not Applicable Not Applicable Not Applicable Stage IIB Not Applicable Not Applicable Not Applicable 6th Not Applicable Not Applicable Not Applicable M0 N1 T2 2000 NO YES 78 4 Lung Dead 2011 2002 1963

TCGA-75-6207 T2 N2 M0 Stage IIIA Not Available R-Upper Not Applicable TCGA-75-6207 58babefe-7e81-4594-ba29-50d1de92a5ab 21 Not Available Not Available Not Available Not Available Not Available Lung Adenocarcinoma Not Available Not Available Not Available Not Available Not Available Not Available Not Available Not Available Not Available Not Available NO NO Not Available Not Available TCGA-75-6207-F15092 53c4832a-9167-41ab-8172-0713906b893e 21 Not Available Not Available Not Available Not Available Not Available Not Available Not Available Not Available Not Available Not Available 7 Not Available Not Available Not Available Not Available NO Complete Remission/Response Not Available NO Dead 2011 MALE Lung Adenocarcinoma- Not Otherwise Specified (NOS) No C34.1 8140/3 C34.9 YES Not Available Not Available Not Available Not Available Peripheral Lung 7 Not Available Not Available Not Available Not Available Not Available Not Available Not Available Not Available Not Available Not Available 10 No 6207 Not Available Not Available Not Available Not Available Not Available Not Available Not Available Not Available Not Available Not Available Not Available R0 Not Applicable Not Applicable Not Applicable Not Applicable Not Applicable Not Applicable Not Applicable Not Applicable Not Applicable Stage IIIA Not Applicable Not Applicable Not Applicable 6th Not Applicable Not Applicable Not Applicable M0 N2 T2 1972 NO YES 75 3 Lung Dead 2011 2007 Not Available

TCGA-78-8640 7062 Alive T1 N1 M0 Stage IIA 59 L-Upper Not Applicable TCGA-78-8640 18478121-f1d1-4bdd-9511-8ab22f4a0660 23 -21832 Not Applicable 0 6346 Not Available Lung Adenocarcinoma Not Available 0 Not Available NO Not Available Not Available Not Available NO Not Available Not Evaluated Not Available;Not Available Not Available;Not Available Not Available;Not Available Not Available;Not Available TCGA-78-8640-F46106;TCGA-78-8640-F70500 E3777749-8064-4049-A825-BB96779AEAD3;D76CC938-737A-46E4-BA56-1A16F671F442 4;25 Not Applicable;Not Available Not Applicable;Not Available Not Applicable;Not Applicable 6528;7062 Not Applicable;Not Available Unknown;Unknown Scheduled Follow-up Submission;Scheduled Follow-up Submission Complete Remission/Response;Unknown Not Available;Not Available NO;NO 8;3 Not Available;Not Available NO;Unknown Unknown;Not Available Unknown;Unknown NO;NO Complete Remission/Response;Complete Remission/Response Not Available;Not Available NO;NO Alive;Alive 2013;2015 MALE Lung Adenocarcinoma- Not Otherwise Specified (NOS) No C34.1 8140/3 C34.1 YES Not Available NO Not Available Not Available Peripheral Lung 10 Not Available Not Available Not Available Not Available Not Available Not Available Not Available Not Available Not Available NO 45 No 8640 Preoperative TUMOR FREE Not Available Not Available NO 73 66 Complete Remission/Response YES Not Evaluated NO R0 Not Applicable Not Applicable Not Applicable Not Applicable Not Applicable Not Applicable Not Applicable Not Applicable Not Applicable Stage IIA Not Applicable Not Applicable Not Applicable 6th Not Applicable Not Applicable Not Applicable M0 N1 T1 Not Available NO YES 78 2 Lung Alive 2012 1994 1949

TCGA-MP-A4T8 161 Dead T2 N2 M0 Stage IIIA 68 L-Upper Not Applicable TCGA-MP-A4T8 7C4ED533-EE66-48BE-A84B-FF8607742818 2 -25069 161 0 Not Available Not Available Lung Adenocarcinoma 102 TCGA-MP-A4T8-D41347;TCGA-MP-A4T8-D41348 5847897E-0C0F-4333-98FC-46101B12E294;254AEB4C-E848-471D-90B6-75DC48A1F902 Not Available;Not Available 15;15 72;72 42;42 Navelbine;Cisplatin Unknown;Unknown 3;3 Not Available;Not Available Not Available;Not Available Not Available;Not Available Not Available;Not Available Not Applicable;Not Applicable Not Available;Not Available Not Available;Not Available NO;NO Chemotherapy;Chemotherapy Not Available;Not Available Not Available;Not Available Not Available;Not Available NO;NO 2013;2013 1 Not Available NO Not Available Not Available Not Available NO Not Available Unknown MALE Lung Adenocarcinoma- Not Otherwise Specified (NOS) No C34.1 8140/3 C34.1 YES Not Evaluated NO Not Available Not Available Unknown 4 Not Available Not Available Not Available Not Available Not Available Not Available Not Available Not Available Not Available Unknown 25 Yes, History of Prior Malignancy A4T8 Pre-Adjuvant Therapy Unknown Not Available 72 YES Not Available 60 Unknown YES Unknown NO R0 Not Applicable Not Applicable Not Applicable Not Applicable Not Applicable Not Applicable Not Applicable Not Applicable Not Applicable Stage IIIA Not Applicable Not Applicable Not Applicable 6th Not Applicable Not Applicable Not Applicable M0 N2 T2 1972 NO YES MP 3 Lung Dead 2013 2006 1957

TCGA-97-8547 657 Alive T2a N2 MX Stage IIIA 78 R-Lower Not Applicable TCGA-97-8547 ac0d7a82-82cb-4aec-b859-e37375f3de8b 27 -28801 Not Applicable 0 148 Not Available Lung Adenocarcinoma 87 TCGA-97-8547-D35445;TCGA-97-8547-D35446;TCGA-97-8547-D35447 46926057-7C1B-4651-913D-B4C08C699594;8EDFE56A-3EB6-47A8-A4BA-2255FF3A3418;393D56D3-0031-4F0A-9E38-F16B66B602D5 Not Available;Not Available;Not Available 27;27;27 111;111;Not Available 47;47;124 Taxol;Carboplatin;TARCEVA Complete Response;Complete Response;Not Applicable 9;9;9 Not Available;Not Available;Not Available Not Available;Not Available;Not Available Not Available;Not Available;Not Available Not Available;Not Available;Not Available Not Applicable;Not Applicable;Not Applicable Not Available;Not Available;Not Available Not Available;Not Available;Not Available NO;NO;YES Chemotherapy;Chemotherapy;Not Available Not Available;Not Available;Not Available Not Available;Not Available;Not Available Not Available;Not Available;Not Available NO;NO;NO 2012;2012;2012 Not Evaluated Not Available YES Exon 19 Deletion Not Available Not Available NO Not Available Not Evaluated Not Available Not Available Not Available Not Available TCGA-97-8547-F49559 C213E7BE-D60E-412A-BD5D-8BCFC8749CA7 3 Not Applicable Not Applicable Not Applicable 657 Not Applicable Not Evaluated Scheduled Follow-up Submission Complete Remission/Response Not Evaluated NO 10 Not Available NO Not Evaluated TUMOR FREE YES Complete Remission/Response Not Available NO Alive 2013 FEMALE Lung Adenocarcinoma Mixed Subtype No C34.3 8550/3 C34.3 YES Not Available YES NO Not Available Unknown 9 Not Available Not Available Not Available Not Available Not Available Not Available Not Available Not Available Not Available NO Not Available No 8547 Not Available TUMOR FREE Not Available Not Available YES Not Available 136 Complete Remission/Response YES Not Evaluated NO Not Evaluated Not Applicable Not Applicable Not Applicable Not Applicable Not Applicable Not Applicable Not Applicable Not Applicable Not Applicable Stage IIIA Not Applicable Not Applicable Not Applicable 7th Not Applicable Not Applicable Not Applicable MX N2 T2a Not Available YES NO 97 1 Lung Alive 2012 2012 Not Available

TCGA-49-AARN 1135 Dead T1 N0 MX Stage IA 56 R-Lower Not Applicable TCGA-49-AARN 98EF41FA-3015-445A-BBDC-984361C911F7 19 -20605 1135 0 Not Available Not Available Lung Adenocarcinoma 110.6 1 Not Available Not Available Not Available Not Available Not Available Not Available Not Available NOT HISPANIC OR LATINO FEMALE Lung Adenocarcinoma- Not Otherwise Specified (NOS) No C34.3 8140/3 C34.3 YES 90 Not Available Not Available Not Available Not Available 6 Not Available Not Available Not Available Not Available Not Available Not Available Not Available Not Available Not Available Unknown Not Available Yes, History of Synchronous/Bilateral Malignancy AARN Preoperative Unknown 85.9 101.1 NO 80.2 94.4 Complete Remission/Response YES BLACK OR AFRICAN AMERICAN NO R0 Not Applicable Not Applicable Not Applicable Not Applicable Not Applicable Not Applicable Not Applicable Not Applicable Not Applicable Stage IA Not Applicable Not Applicable Not Applicable 5th Not Applicable Not Applicable Not Applicable MX N0 T1 Not Available NO YES 49 5 Lung Dead 2014 2000 Not Available

TCGA-73-4676 281 Dead T2a N1 M0 Stage IIA 45 R-Upper Not Applicable TCGA-73-4676 195a5afb-b79f-44d2-9d12-884487630c2b 18 -16746 Not Applicable 0 122 Not Available Lung Adenocarcinoma 86 TCGA-73-4676-D12586;TCGA-73-4676-D12585;TCGA-73-4676-D12587;TCGA-73-4676-D12556 48957710-70e7-4615-9717-fb2a9174b210;0989c0d2-81e6-4f78-93b4-3a7f35ba221b;1eba36d1-01f8-4cd4-b90d-98cc949417f0;da213998-af4a-4a37-967e-419359cab836 Not Available;Not Available;Not Available;Not Available 2;2;2;2 66;-40;66;-40 45;-74;45;-74 Docetaxel;Cisplatin;Cisplatin;Docetaxel Not Available;Not Available;Not Available;Not Available 6;6;6;6 2;3;2;3 Not Available;75;Not Available;75 mg/m2;mg/m2;mg/m2;mg/m2 ADJUVANT;OTHER, SPECIFY IN NOTES;ADJUVANT;OTHER, SPECIFY IN NOTES Not Applicable;neo-adjuvant;Not Applicable;neo-adjuvant 2;1;2;1 IV;IV;IV;IV NO;NO;NO;NO Chemotherapy;Chemotherapy;Chemotherapy;Chemotherapy Not Available;Not Available;Not Available;Not Available Not Available;225;Not Available;225 mg/m2;mg/m2;mg/m2;mg/m2 Not Available;Not Available;Not Available;Not Available 2011;2011;2011;2011 1 Not Available NO Not Available Not Available Not Available NO Not Available NOT HISPANIC OR LATINO Not Available;Not Available Not Available;Not Available Not Available;Not Available Not Available;Not Available TCGA-73-4676-F12553;TCGA-73-4676-F71022 f8c9076a-23b2-4792-bcbc-c88d250d1ff3;B89F1AE8-D8B3-4B9D-9B4F-D3F9F887FFF9 18;18 Not Applicable;Not Available Not Applicable;Not Available Not Applicable;281 164;Not Available Not Applicable;Not Available 0;Unknown Not Available;Scheduled Follow-up Submission Not Available;Unknown 90;Unknown Not Available;NO 5;3 Not Available;Not Available NO;Unknown Adjuvant therapy;Unknown Not Available;WITH TUMOR YES;YES Not Available;Unknown Not Available;Not Available YES;YES Alive;Dead 2011;2015 MALE Lung Adenocarcinoma- Not Otherwise Specified (NOS) Yes C34.1 8140/3 C34.1 YES 70 NO Not Available Not Available Peripheral Lung 5 Not Available Not Available Not Available Not Available Not Available Not Available Not Available Not Available Not Available Not Available 48 No 4676 Preoperative TUMOR FREE Not Available Not Available Not Available 67 78 Not Available YES WHITE Not Available Primary Tumor Field TCGA-73-4676-R12555 04a28cdd-15da-414f-8481-8b42047187f6 1 2 125 Not Available Not Available 6 Not Available Not Available NO Not Available Not Applicable ADJUVANT Not Available Not Available 2011 R0 Not Applicable Not Applicable Not Applicable Not Applicable Not Applicable Not Applicable Not Applicable Not Applicable Not Applicable Stage IIA Not Applicable Not Applicable Not Applicable 7th Not Applicable Not Applicable Not Applicable M0 N1 T2a 2009 NO YES 73 4 Lung Alive 2011 2010 1977

TCGA-53-7626 929 Dead T1 N1 M0 Stage IIA 76 R-Upper Not Applicable TCGA-53-7626 d3ff328c-2488-47bd-8663-366b6812c7b5 13 -28024 929 0 Not Available Not Available Lung Adenocarcinoma Not Available TCGA-53-7626-D39213;TCGA-53-7626-D39214 0BEFAE89-775A-44A5-8352-ECB58FBCA0B3;61FB2C2D-04A9-420C-881B-821008C23CCA Not Available;Not Available 15;15 Not Available;Not Available Not Available;Not Available Cisplatin;Taxotere Clinical Progressive Disease;Clinical Progressive Disease 1;1 Not Available;Not Available Not Available;Not Available Not Available;Not Available Not Available;Not Available Not Applicable;Not Applicable Not Available;Not Available Not Available;Not Available NO;NO Chemotherapy;Chemotherapy Not Available;Not Available Not Available;Not Available Not Available;Not Available NO;NO 2013;2013 0 Not Available NO Not Available Not Available Not Available NO Not Available NOT HISPANIC OR LATINO NO NO NO Not Available TCGA-53-7626-F38969 8B9047BD-EFD5-41E5-863C-04D4F982B422 15 Not Available Not Available 929 Not Available 865 Not Available Scheduled Follow-up Submission Progressive Disease Not Available NO 1 Locoregional Recurrence YES Not Available WITH TUMOR YES Progressive Disease Biopsy with Histologic Confirmation NO Dead 2013 FEMALE Lung Adenocarcinoma- Not Otherwise Specified (NOS) No C34.1 8140/3 C34.1 YES Not Available NO Not Available Not Available Peripheral Lung 12 Not Available Not Available Not Available Not Available Not Available Not Available Not Available Not Available Not Available Not Available 35 No 7626 Post-Adjuvant Therapy WITH TUMOR Not Available Not Available Not Available Not Available Not Available Not Available Not Available WHITE Not Available R0 Not Applicable Not Applicable Not Applicable Not Applicable Not Applicable Not Applicable Not Applicable Not Applicable Not Applicable Stage IIA Not Applicable Not Applicable Not Applicable 6th Not Applicable Not Applicable Not Applicable M0 N1 T1 2006 NO YES 53 4 Lung Dead 2011 2008 1971

TCGA-78-7146 173 Dead T2 N2 M0 Stage IIIA 71 L-Lower Not Applicable TCGA-78-7146 ff9def3d-17e5-4ef6-b74e-933f11ed6f00 28 -26272 173 0 Not Available Not Available Lung Adenocarcinoma Not Available 1 Not Available NO Not Available Not Available Not Available NO Not Available Not Available Not Available Not Available Not Available Not Available TCGA-78-7146-F16969 89a4cee2-d516-4b0e-8664-c1a31a242e71 28 Not Available Not Available 173 Not Available Not Available Not Available Scheduled Follow-up Submission Not Available Not Available Not Available 9 Not Available Not Available Not Available Not Available NO Not Available Not Available NO Dead 2011 FEMALE Lung Adenocarcinoma Mixed Subtype No C34.3 8255/3 C34.3 YES Not Available NO Not Available Not Available Peripheral Lung 9 Not Available Not Available Not Available Not Available Not Available Not Available Not Available Not Available Not Available Not Available 6 No 7146 Preoperative Not Available Not Available Not Available Not Available Not Available Not Available Not Available Not Available WHITE Not Available R0 Not Applicable Not Applicable Not Applicable Not Applicable Not Applicable Not Applicable Not Applicable Not Applicable Not Applicable Stage IIIA Not Applicable Not Applicable Not Applicable 6th Not Applicable Not Applicable Not Applicable M0 N2 T2 1998 NO YES 78 4 Lung Dead 2011 1999 1988

TCGA-38-4629 864 Dead T3 N0 M0 Stage IIB 68 R-Upper Not Applicable TCGA-38-4629 127bf818-f7e5-46b5-a9de-39f6d96b8b83 13 -25104 864 0 Not Available Not Available Lung Adenocarcinoma Not Available Not Available Not Available NO Not Available Not Available Not Available NO Not Available NOT HISPANIC OR LATINO NO NO NO NO TCGA-38-4629-F4887 5ece2db2-89aa-48dc-92be-f4df4bbbef10 14 Not Available Not Available 864 Not Available 379 Not Available Not Available Not Available Not Available Not Available 12 Not Available YES Not Available WITH TUMOR NO Complete Remission/Response Not Available NO Dead 2010 MALE Lung Adenocarcinoma- Not Otherwise Specified (NOS) No C34.1 8140/3 C34.1 YES Not Available NO Not Available Not Available Peripheral Lung 12 Not Available Not Available Not Available Not Available Not Available Not Available Not Available Not Available Not Available Not Available 100 Yes 4629 Not Available WITH TUMOR Not Available Not Available Not Available Not Available Not Available Not Available Not Available WHITE Not Available R0 Not Applicable Not Applicable Not Applicable Not Applicable Not Applicable Not Applicable Not Applicable Not Applicable Not Applicable Stage IIB Not Applicable Not Applicable Not Applicable Not Available Not Applicable Not Applicable Not Applicable M0 N0 T3 2003 NO YES 38 4 Lung Dead 2010 2003 Not Available

TCGA-93-7347 683 Alive T1a N0 MX Stage IA 76 R-Upper Not Applicable TCGA-93-7347 ef03654a-dcf1-41ff-a67e-4f3fbf9ba807 30 -27949 Not Applicable 0 297 Not Available Lung Adenocarcinoma 70 Not Available Not Available NO Not Available Not Available Not Available NO Not Available NOT HISPANIC OR LATINO Not Available;Not Available Not Available;Not Available Not Available;Not Available Not Available;Not Available TCGA-93-7347-F29886;TCGA-93-7347-F43155 3cb5e775-0ae6-4d83-8d2a-049f1eb2b119;1AA9F067-47F9-4CB2-837D-E9907C167259 30;14 Not Applicable;Not Applicable Not Applicable;Not Applicable Not Applicable;Not Applicable 297;683 Not Applicable;Not Applicable 0;Not Evaluated Scheduled Follow-up Submission;Scheduled Follow-up Submission Complete Remission/Response;Not Applicable 100;Not Evaluated Not Available;NO 3;5 Not Available;Not Available NO;NO Other;Not Available TUMOR FREE;TUMOR FREE NO;NO Complete Remission/Response;Complete Remission/Response Not Available;Not Available NO;NO Alive;Alive 2012;2013 FEMALE Lung Adenocarcinoma Mixed Subtype No C34.1 8255/3 C34.1 YES Not Available NO Not Available Not Available Central Lung 3 Not Available Not Available Not Available Not Available Not Available Not Available Not Available Not Available Not Available Not Available 11 No 7347 Not Available TUMOR FREE 61 84 Not Available 62 84 Not Available YES WHITE Not Available R0 Not Applicable Not Applicable Not Applicable Not Applicable Not Applicable Not Applicable Not Applicable Not Applicable Not Applicable Stage IA Not Applicable Not Applicable Not Applicable 7th Not Applicable Not Applicable Not Applicable MX N0 T1a 1967 YES NO 93 3 Lung Alive 2012 2011 1952

TCGA-L9-A5IP 58 Dead T3 N2 M1b Stage IV 40 R-Upper Not Applicable TCGA-L9-A5IP 2A43E61E-2151-4C5A-B062-74CE9DFD8D4A 14 -14681 58 0 Not Available Not Available Lung Adenocarcinoma Not Available Not Evaluated Not Available YES Not Available Not Available FISH YES Not Available NOT HISPANIC OR LATINO FEMALE Lung Adenocarcinoma- Not Otherwise Specified (NOS) No C34.1 8140/3 C34.1 YES Not Available NO Not Available Not Available Unknown 5 NO NO 49 NO Not Available NO Not Available Locoregional Recurrence;Distant Metastasis Convincing Imaging YES Not Available No A5IP Not Evaluated WITH TUMOR Not Available Not Available NO Not Available Not Available Progressive Disease NO BLACK OR AFRICAN AMERICAN NO R1 Not Applicable Not Applicable Not Applicable Not Applicable Not Applicable Not Applicable Not Applicable Not Applicable Not Applicable Stage IV Not Applicable Not Applicable Not Applicable 7th Not Applicable Not Applicable Not Applicable M1b N2 T3 2012 YES NO L9 4 Lung Dead 2013 2012 Not Available

TCGA-55-7995 889 Alive T1b N0 M0 Stage IA 73 L-Upper Not Applicable TCGA-55-7995 abd6647b-d2eb-404d-8283-443e92addf55 7 -26837 Not Applicable 0 5 Not Available Lung Adenocarcinoma 51 TCGA-55-7995-D65702;TCGA-55-7995-D65703 39A1C7F0-0574-41C9-98D5-D0D7AF66D866;386B9E74-6618-451B-8F90-9F3BFDE51CD1 Not Available;Not Available 26;26 525;525 483;483 Carboplatin;Paclitaxel Complete Response;Complete Response 9;9 Not Available;Not Available Not Available;Not Available Not Available;Not Available Not Available;Not Available Not Applicable;Not Applicable Not Available;Not Available Not Available;Not Available NO;NO Chemotherapy;Chemotherapy Not Available;Not Available Not Available;Not Available Not Available;Not Available NO;NO 2014;2014 Not Available Not Available NO Not Available Not Available Not Available NO Not Available NOT HISPANIC OR LATINO YES YES NO Not Available TCGA-55-7995-F65700 FD821C68-D4E0-4555-BAD0-65DC0C3F3FD0 26 Not Available Not Available Not Applicable 889 468 1 Scheduled Follow-up Submission Complete Remission/Response 80 NO 9 Locoregional Recurrence YES Post-Adjuvant Therapy TUMOR FREE NO Complete Remission/Response Convincing Imaging NO Alive 2014 FEMALE Lung Adenocarcinoma- Not Otherwise Specified (NOS) No C34.1 8140/3 C34.1 YES Not Available Unknown Not Available Not Available Peripheral Lung 5 Not Available Not Available Not Available Not Available Not Available Not Available Not Available Not Available Not Available NO 60 No 7995 Not Available TUMOR FREE Not Available Not Available Unknown 71 77 Unknown YES WHITE Unknown Regional site TCGA-55-7995-R65701 FB0F9AE8-CC60-4A32-80BE-77F43A903343 Not Available 26 525 483 Complete Response 9 Not Available 6500 NO External Not Applicable Not Available Not Available cGy 2014 R0 Not Applicable Not Applicable Not Applicable Not Applicable Not Applicable Not Applicable Not Applicable Not Applicable Not Applicable Stage IA Not Applicable Not Applicable Not Applicable 7th Not Applicable Not Applicable Not Applicable M0 N0 T1b 2002 YES NO 55 4 Lung Alive 2012 2012 Not Available

TCGA-69-8253 426 Alive T1a N1 MX Stage IIA 59 R-Lower Not Applicable TCGA-69-8253 44d8c859-4b7a-4e9d-bffc-0de0e6afdcc4 1 -21771 Not Applicable 0 100 Not Available Lung Adenocarcinoma Not Available TCGA-69-8253-D32564;TCGA-69-8253-D32565 E2D64977-0DCC-4BF6-A66E-7D40AF9263AF;3AF5EEC9-47EC-4D16-8427-FE85149B06A5 Not Available;Not Available 1;1 Not Available;Not Available 55;55 Cisplatin;Pemetrexed Not Applicable;Not Applicable 6;6 Not Available;Not Available Not Available;Not Available Not Available;Not Available Not Available;Not Available Not Applicable;Not Applicable Not Available;Not Available Not Available;Not Available YES;YES Chemotherapy;Chemotherapy Not Available;Not Available Not Available;Not Available Not Available;Not Available NO;NO 2012;2012 2 Not Available NO Not Available Not Available Not Available NO Not Available NOT HISPANIC OR LATINO Not Available Not Available Not Available Not Available TCGA-69-8253-F41949 909F53FB-3A49-456C-9593-B6B043711B7B 5 Not Applicable Not Applicable Not Applicable 426 Not Applicable 1 Scheduled Follow-up Submission Complete Remission/Response Not Evaluated NO 4 Not Available NO Preoperative TUMOR FREE YES Complete Remission/Response Not Available NO Alive 2013 FEMALE Lung Adenocarcinoma Mixed Subtype No C34.3 8255/3 C34.3 YES Not Available NO Not Available Not Available Not Available 6 Not Available Not Available Not Available Not Available Not Available Not Available Not Available Not Available Not Available NO 7.8 No 8253 Pre-Adjuvant Therapy Unknown Not Available Not Available YES Not Available 87 Unknown YES BLACK OR AFRICAN AMERICAN NO Not Available Not Applicable Not Applicable Not Applicable Not Applicable Not Applicable Not Applicable Not Applicable Not Applicable Not Applicable Stage IIA Not Applicable Not Applicable Not Applicable 7th Not Applicable Not Applicable Not Applicable MX N1 T1a 2011 YES NO 69 4 Lung Alive 2012 2012 1972

TCGA-05-4402 244 Dead T2 NX M1 Stage IV 57 R-Lower Not Applicable TCGA-05-4402 722d6818-f1cb-49f4-b68a-fd0a31304681 22 -20819 244 0 Not Available Not Available Lung Adenocarcinoma Not Available TCGA-05-4402-D36467;TCGA-05-4402-D36468;TCGA-05-4402-D36469 C22C6B31-7A7B-4961-B95D-137666A2C3FF;BA9F9930-88F2-4DA0-982D-BEDBEA793B76;8CEA61DA-614E-4CE9-8202-877E85ECC83F Not Available;Not Available;Not Available 5;5;5 91;91;122 30;30;122 Carboplatin;Vinorelbine;Erlotinib Complete Response;Complete Response;Complete Response 12;12;12 Not Available;Not Available;Not Available Not Available;Not Available;Not Available Not Available;Not Available;Not Available Not Available;Not Available;Not Available Not Applicable;Not Applicable;Not Applicable Not Available;Not Available;Not Available Not Available;Not Available;Not Available NO;NO;NO Chemotherapy;Chemotherapy;Immunotherapy Not Available;Not Available;Not Available Not Available;Not Available;Not Available Not Available;Not Available;Not Available NO;NO;NO 2012;2012;2012 Not Available Not Available Not Available Not Available Not Available Not Available Not Available Not Available Not Available Not Available Not Available Not Available Not Available TCGA-05-4402-F36466 DFAF4F5D-D945-4D17-8F16-6BFB6E538D7A 31 Not Applicable Not Applicable 244 Not Available Not Applicable Not Available Scheduled Follow-up Submission Complete Remission/Response Not Available NO 10 Not Available NO Not Available TUMOR FREE YES Complete Remission/Response Not Available NO Dead 2012 FEMALE Lung Adenocarcinoma Mixed Subtype No C34.3 8255/3 C34.3 YES Not Available Not Available Not Available Not Available Not Available 7 Not Available Not Available Not Available Not Available Not Available Not Available Not Available Not Available Not Available Not Available Not Available Yes 4402 Not Available TUMOR FREE Not Available Not Available Not Available Not Available Not Available Not Available Not Available Not Available Not Available R2 Not Applicable Not Applicable Not Applicable Not Applicable Not Applicable Not Applicable Not Applicable Not Applicable Not Applicable Stage IV Not Applicable Not Applicable Not Applicable 6th Not Applicable Not Applicable Not Applicable M1 NX T2 Not Available NO YES 05 1 Lung Dead 2010 2007 Not Available

TCGA-38-4628 1492 Dead T2 N1 M0 Stage IIB 65 R-Upper Not Applicable TCGA-38-4628 cc4bd56a-25c5-4c48-b583-ac3aeb778ca6 9 -24057 1492 0 Not Available Not Available Lung Adenocarcinoma 72 TCGA-38-4628-D4678;TCGA-38-4628-D13831 0c5ddee7-c7b0-44a1-97d7-c6eb11c9b514;357b5e2f-bb1b-41f7-8c7c-4ea6dd8a2fd3 Not Available;Not Available 9;14 1238;1238 1147;1147 Carboplatin;Taxol Not Available;Not Available 12;7 4;4 Not Available;Not Available Not Available;Not Available RECURRENCE;RECURRENCE Not Applicable;Not Applicable 1;1 IV;IV NO;NO Chemotherapy;Chemotherapy Not Available;Not Available Not Available;Not Available Not Available;Not Available Not Available;Not Available 2010;2011 Not Available Not Available NO Not Available Not Available Not Available NO Not Available NOT HISPANIC OR LATINO YES NO NO YES TCGA-38-4628-F4676 cca64327-9837-4503-abfd-9e6ab40ff8dc 9 Not Available 1118 1492 Not Available 1083 Not Available Not Available Stable Disease Not Available Not Available 12 Not Available YES Not Available WITH TUMOR NO Complete Remission/Response Not Available NO Dead 2010 FEMALE Lung Adenocarcinoma- Not Otherwise Specified (NOS) No C34.1 8140/3 C34.1 YES Not Available NO Not Available Not Available Central Lung 12 Not Available Not Available Not Available Not Available Not Available Not Available Not Available Not Available Not Available Not Available Not Available No 4628 Not Available WITH TUMOR 78 85 Not Available 74 86 Not Available YES WHITE Not Available R0 Not Applicable Not Applicable Not Applicable Not Applicable Not Applicable Not Applicable Not Applicable Not Applicable Not Applicable Stage IIB Not Applicable Not Applicable Not Applicable 5th Not Applicable Not Applicable Not Applicable M0 N1 T2 Not Available NO YES 38 1 Lung Dead 2010 2002 Not Available

TCGA-55-8505 440 Alive T1a N2 MX Stage IIIA 62 R-Lower Not Applicable TCGA-55-8505 f59e5a76-f803-470b-910c-a897a149d939 28 -22676 Not Applicable 0 29 Not Available Lung Adenocarcinoma Not Available TCGA-55-8505-D47799;TCGA-55-8505-D47801 E1832AA8-C7E7-4BF8-BF54-E7BC3EEF3619;57354507-5DEF-495C-8B74-0E7773AC1C4B Not Available;Not Available 28;28 159;159 96;96 Cisplatin;Alimta Complete Response;Complete Response 8;8 Not Available;Not Available Not Available;Not Available Not Available;Not Available Not Available;Not Available Not Applicable;Not Applicable Not Available;Not Available Not Available;Not Available NO;NO Chemotherapy;Chemotherapy Not Available;Not Available Not Available;Not Available Not Available;Not Available NO;NO 2013;2013 Not Evaluated Not Available Unknown Not Available Not Available Not Available Unknown Not Available NOT HISPANIC OR LATINO Not Available Not Available Not Available Not Available TCGA-55-8505-F47798 20147D1C-C4BA-40AF-9FF7-4122FAA97854 28 Not Applicable Not Applicable Not Applicable 440 Not Applicable Not Evaluated Scheduled Follow-up Submission Complete Remission/Response Not Evaluated NO 8 Not Available NO Not Available TUMOR FREE YES Complete Remission/Response Not Available NO Alive 2013 MALE Lung Adenocarcinoma- Not Otherwise Specified (NOS) No C34.3 8140/3 C34.3 YES Not Evaluated Unknown Not Available Not Available Unknown 12 Not Available Not Available Not Available Not Available Not Available Not Available Not Available Not Available Not Available NO Not Available No 8505 Not Available TUMOR FREE Not Available Not Available Unknown Not Available Not Available Complete Remission/Response Not Available WHITE Unknown R0 Not Applicable Not Applicable Not Applicable Not Applicable Not Applicable Not Applicable Not Applicable Not Applicable Not Applicable Stage IIIA Not Applicable Not Applicable Not Applicable 7th Not Applicable Not Applicable Not Applicable MX N2 T1a Not Available YES NO 55 1 Lung Alive 2012 2012 Not Available

TCGA-49-AAR9 260 Dead T3 N0 MX Stage IIB 61 Other (please specify) ALL 3 LOBES OF RT LUNG TCGA-49-AAR9 47B749E6-E060-4AA1-8F29-976E489BA789 18 -22510 260 0 Not Available Not Available Lung Adenocarcinoma Not Available 1 Not Available Not Available Not Available Not Available Not Available Not Available Not Available NOT HISPANIC OR LATINO MALE Lung Adenocarcinoma- Not Otherwise Specified (NOS) No C34.1 8140/3 C34.9 YES 80 Not Available Not Available Not Available Not Available 6 Not Available Not Available Not Available Not Available Not Available Not Available Not Available Not Available Not Available NO Not Available No AAR9 Preoperative WITH TUMOR Not Available Not Available NO Not Available Not Available Progressive Disease NO BLACK OR AFRICAN AMERICAN NO R0 Not Applicable Not Applicable Not Applicable Not Applicable Not Applicable Not Applicable Not Applicable Not Applicable Not Applicable Stage IIB Not Applicable Not Applicable Not Applicable 5th Not Applicable Not Applicable Not Applicable MX N0 T3 Not Available NO YES 49 2 Lung Dead 2014 1999 Not Available

TCGA-05-4427 791 Alive T2 N1 M0 Stage IIB 65 L-Upper Not Applicable TCGA-05-4427 78a24c56-576c-4245-bb42-6603b3d19897 22 -23893 Not Applicable 0 791 Not Available Lung Adenocarcinoma Not Available TCGA-05-4427-D36565;TCGA-05-4427-D36566 8809622A-D398-4D9D-95B7-92480AD0CA12;2978B988-DC8D-449A-83CC-6C4831AA0FE2 Not Available;Not Available 5;5 122;122 61;61 Cisplatin;Vinorelbine Complete Response;Complete Response 12;12 Not Available;Not Available Not Available;Not Available Not Available;Not Available Not Available;Not Available Not Applicable;Not Applicable Not Available;Not Available Not Available;Not Available NO;NO Chemotherapy;Chemotherapy Not Available;Not Available Not Available;Not Available Not Available;Not Available NO;NO 2012;2012 Not Available Not Available Not Available Not Available Not Available Not Available Not Available Not Available Not Available Not Available Not Available Not Available Not Available TCGA-05-4427-F36564 C0280C41-DC59-4741-8314-055BD0B90DC2 2 Not Applicable Not Applicable Not Applicable 791 Not Applicable Not Available Scheduled Follow-up Submission Complete Remission/Response Not Available NO 11 Not Available NO Not Available TUMOR FREE YES Complete Remission/Response Not Available NO Alive 2012 FEMALE Lung Adenocarcinoma Mixed Subtype No C34.1 8255/3 C34.1 YES Not Available Not Available Not Available Not Available Not Available 7 Not Available Not Available Not Available Not Available Not Available Not Available Not Available Not Available Not Available Not Available 8 Yes 4427 Not Available TUMOR FREE Not Available Not Available Not Available Not Available Not Available Not Available Not Available Not Available Not Available R0 Not Applicable Not Applicable Not Applicable Not Applicable Not Applicable Not Applicable Not Applicable Not Applicable Not Applicable Stage IIB Not Applicable Not Applicable Not Applicable 6th Not Applicable Not Applicable Not Applicable M0 N1 T2 2008 NO YES 05 4 Lung Alive 2010 2008 1993

TCGA-53-7624 1043 Dead T2 N0 M1 Stage IV 40 R-Upper Not Applicable TCGA-53-7624 54a9cc9e-3eee-4caa-9f33-4ea64a2999ba 13 -14794 1043 0 Not Available Not Available Lung Adenocarcinoma Not Available TCGA-53-7624-D39727;TCGA-53-7624-D39729;TCGA-53-7624-D39730;TCGA-53-7624-D39731;TCGA-53-7624-D39732;TCGA-53-7624-D39735;TCGA-53-7624-D39736;TCGA-53-7624-D39737;TCGA-53-7624-D39738;TCGA-53-7624-D39739 CABE6493-EEA3-4417-991E-E2D8BAA5D0F4;28681B5B-2CCC-41DB-B342-A1BB56EE7432;03D35A9D-2F9E-4DD6-BEF9-4EAE9D35C1DB;F6AB7F08-1341-440F-90F1-54F3E9CF0C7F;588CEFB2-2FCB-4A28-893B-35DDF6E44FB9;9628B2FD-EDBE-415B-A139-7EC10C28CF7E;5CDA082A-CFF9-4E2A-9C47-299425450EF8;87E2D964-3168-438C-9CB9-1F81D70122AE;0F25327C-C54D-4542-A956-47AFFD9D63B5;EAC0A9FD-301B-48F7-AFD2-499DFE2F0418 Not Available;Not Available;Not Available;Not Available;Not Available;Not Available;Not Available;Not Available;Not Available;Not Available 31;31;31;31;31;31;31;31;31;31 966;922;980;861;782;186;186;490;490;490 924;880;980;803;726;66;66;339;339;339 Irinotecan;Erlotinib;Abraxane;Docetaxel;Gemcitabine;Cisplatin;Vinorelbine;Carboplatinum;Bevacizumab;Pemetrexed Clinical Progressive Disease;Clinical Progressive Disease;Clinical Progressive Disease;Clinical Progressive Disease;Clinical Progressive Disease;Clinical Progressive Disease;Clinical Progressive Disease;Clinical Progressive Disease;Clinical Progressive Disease;Clinical Progressive Disease 1;1;1;1;1;1;1;1;1;1 Not Available;Not Available;Not Available;Not Available;Not Available;Not Available;Not Available;Not Available;Not Available;Not Available Not Available;Not Available;Not Available;Not Available;Not Available;Not Available;Not Available;Not Available;Not Available;Not Available Not Available;Not Available;Not Available;Not Available;Not Available;Not Available;Not Available;Not Available;Not Available;Not Available Not Available;Not Available;Not Available;Not Available;Not Available;Not Available;Not Available;Not Available;Not Available;Not Available Not Applicable;Not Applicable;Not Applicable;Not Applicable;Not Applicable;Not Applicable;Not Applicable;Not Applicable;Not Applicable;Not Applicable Not Available;Not Available;Not Available;Not Available;Not Available;Not Available;Not Available;Not Available;Not Available;Not Available Not Available;Not Available;Not Available;Not Available;Not Available;Not Available;Not Available;Not Available;Not Available;Not Available NO;NO;NO;NO;NO;NO;NO;NO;NO;NO Chemotherapy;Chemotherapy;Chemotherapy;Chemotherapy;Chemotherapy;Chemotherapy;Chemotherapy;Chemotherapy;Chemotherapy;Chemotherapy Not Available;Not Available;Not Available;Not Available;Not Available;Not Available;Not Available;Not Available;Not Available;Not Available Not Available;Not Available;Not Available;Not Available;Not Available;Not Available;Not Available;Not Available;Not Available;Not Available Not Available;Not Available;Not Available;Not Available;Not Available;Not Available;Not Available;Not Available;Not Available;Not Available NO;NO;NO;NO;NO;NO;NO;NO;NO;NO 2013;2013;2013;2013;2013;2013;2013;2013;2013;2013 0 Not Available NO Not Available Not Available Not Available NO Not Available HISPANIC OR LATINO YES YES Not Available YES TCGA-53-7624-F39722 9661057B-3EF1-4814-AE67-408068F09148 31 Not Available 593 1043 Not Available 400 Not Available Scheduled Follow-up Submission Progressive Disease 0 NO 1 Distant Metastasis YES Not Available WITH TUMOR YES Progressive Disease Biopsy with Histologic Confirmation NO Dead 2013 FEMALE Lung Adenocarcinoma- Not Otherwise Specified (NOS) No C34.1 8140/3 C34.1 YES Not Available NO Not Available Not Available Peripheral Lung 12 Not Available Not Available Not Available Not Available Not Available Not Available Not Available Not Available Not Available Not Available 50 No 7624 Post-Adjuvant Therapy WITH TUMOR Not Available Not Available Not Available Not Available Not Available Not Available Not Available WHITE Not Available Distant site TCGA-53-7624-R39726 78FACC85-69B0-4EEE-9713-CBF425E45FE0 Not Available 31 624 613 Radiographic Progressive Disease 1 10 30 NO External Not Applicable Not Available Not Available Gy 2013 R0 Not Applicable Not Applicable Not Applicable Not Applicable Not Applicable Not Applicable Not Applicable Not Applicable Not Applicable Stage IV Not Applicable Not Applicable Not Applicable 6th Not Applicable Not Applicable Not Applicable M1 N0 T2 Not Available NO YES 53 2 Lung Dead 2011 2008 1986

TCGA-44-5643 1013 Alive T2b N2 M0 Stage IIIA 53 R-Lower Not Applicable TCGA-44-5643 27fceec1-3298-4cdd-a4e6-8f5cf34604f0 13 -19426 Not Applicable 0 106 Not Available Lung Adenocarcinoma 75 TCGA-44-5643-D12952;TCGA-44-5643-D12950;TCGA-44-5643-D12954 2b6a8199-4f87-4880-b81d-ac2b704b0451;9fedb69f-cc66-4f2f-b9f0-71e1dd2d695b;21d67e97-9cd3-4de6-8df9-83f9a1330403 Not Available;Not Available;Not Available 14;14;14 208;Not Available;208 131;223;131 Carboplatin;Tarceva;Taxol Not Available;Not Available;Not Available 6;6;6 8;132;8 205-300;150;185-190 mg;mg;mg ADJUVANT;ADJUVANT;ADJUVANT Not Applicable;Not Applicable;Not Applicable 1;1;1 IV;PO;IV NO;YES;NO Chemotherapy;Chemotherapy;Chemotherapy Not Available;Not Available;Not Available 2035;19800;1505 mg;mg;mg Not Available;Not Available;Not Available 2011;2011;2011 Not Available Not Available Not Available Not Available Not Available Not Available Not Available Not Available NOT HISPANIC OR LATINO Not Available;Not Available Not Available;Not Available Not Available;NO Not Available;Not Available TCGA-44-5643-F12927;TCGA-44-5643-F39742 0dd73d87-f8f5-42fe-85e8-a7552e3c8915;AC910C02-04DD-4779-8BE1-E41E05FE1AD3 14;25 Not Available;Not Applicable Not Available;Not Applicable Not Applicable;Not Applicable 417;1013 Not Available;Not Applicable Not Available;1 Not Available;Scheduled Follow-up Submission Not Available;Complete Remission/Response Not Available;Not Available Not Available;NO 6;2 Not Available;Not Available Not Available;NO Not Available;Other WITH TUMOR;TUMOR FREE YES;Not Available Not Available;Not Available Not Available;Not Available YES;Not Available Alive;Alive 2011;2013 MALE Lung Adenocarcinoma- Not Otherwise Specified (NOS) No C34.3 8140/3 C34.3 YES Not Available Not Available Not Available Not Available Not Available 4 Not Available Not Available Not Available Not Available Not Available Not Available Not Available Not Available Not Available Not Available 20 No 5643 Not Available TUMOR FREE Not Available Not Available Not Available 92 66 Not Available YES BLACK OR AFRICAN AMERICAN Not Available Primary Tumor Field TCGA-44-5643-R12947 973a5961-8dc0-4467-863b-01a26eba6b3a 1 14 168 129 Not Available 6 25 60 NO OTHER IMRT ADJUVANT Not Available cGy 2011 Not Available Not Applicable Not Applicable Not Applicable Not Applicable Not Applicable Not Applicable Not Applicable Not Applicable Not Applicable Stage IIIA Not Applicable Not Applicable Not Applicable 7th Not Applicable Not Applicable Not Applicable M0 N2 T2b Not Available YES NO 44 2 Lung Alive 2011 2010 Not Available

TCGA-73-4670 131 Alive T2 N0 M1 Stage IV 69 L-Upper Not Applicable TCGA-73-4670 1b354837-4925-4480-ac32-6b44d0957314 6 -25526 Not Applicable 0 14 Not Available Lung Adenocarcinoma Not Available TCGA-73-4670-D12984;TCGA-73-4670-D12987;TCGA-73-4670-D12986;TCGA-73-4670-D12988 fce5249c-69e4-43b1-ba19-7261731a8a90;06e09d14-17d7-4d81-a98c-780ac3ff2162;2aaeb964-d8d0-4811-85a8-5b26948bf157;634e1395-f22a-4b7b-80d6-9f2d2929ccd1 Not Available;Not Available;Not Available;Not Available 15;15;15;15 110;110;110;54 33;33;54;33 carboplatin;Avastin;alimta;taxol Not Available;Not Available;Not Available;Not Available 6;6;6;6 4;4;4;1 538;930;680;308 mg;mg;mg;mg ADJUVANT;ADJUVANT;ADJUVANT;ADJUVANT Not Applicable;Not Applicable;Not Applicable;Not Applicable 1;1;2;1 IV;IV;IV;IV NO;NO;NO;NO Chemotherapy;Chemotherapy;Chemotherapy;Chemotherapy Not Available;Not Available;Not Available;Not Available 2152;3720;2720;308 mg;mg;mg;mg Not Available;Not Available;Not Available;Not Available 2011;2011;2011;2011 Not Available Not Available NO Not Available Not Available Not Available NO Not Available NOT HISPANIC OR LATINO Not Available Not Available Not Available Not Available TCGA-73-4670-F12983 9f66a7c0-c40c-412d-ab8c-b6bac0bfe58d 15 Not Applicable Not Applicable Not Applicable 131 Not Applicable 2 Not Available Not Available 50 Not Available 6 Not Available NO Adjuvant therapy Not Available YES Stable Disease Not Available NO Alive 2011 FEMALE Lung Adenocarcinoma- Not Otherwise Specified (NOS) Discrepancy C34.1 8140/3 C34.1 YES Not Available NO Not Available Not Available Peripheral Lung 1 Not Available Not Available Not Available Not Available Not Available Not Available Not Available Not Available Not Available Not Available 30 No 4670 Not Available WITH TUMOR Not Available Not Available Not Available Not Available Not Available Not Available Not Available WHITE Not Available R0 Not Applicable Not Applicable Not Applicable Not Applicable Not Applicable Not Applicable Not Applicable Not Applicable Not Applicable Stage IV Not Applicable Not Applicable Not Applicable Not Available Not Applicable Not Applicable Not Applicable M1 N0 T2 2009 NO YES 73 4 Lung Alive 2011 2010 1979

TCGA-O1-A52J 1798 Dead T1 N0 MX Stage IA 74 L-Lower Not Applicable TCGA-O1-A52J 42432463-8E92-4F25-B72A-F03953527AA5 11 -27223 1798 0 Not Available Not Available Lung Adenocarcinoma Not Available 1 Not Available NO Not Available Not Available Not Available NO Not Available NOT HISPANIC OR LATINO FEMALE Lung Adenocarcinoma- Not Otherwise Specified (NOS) No C34.3 8140/3 C34.3 YES Not Evaluated NO Not Available Not Available Unknown 4 NO NO 898 NO Not Available Not Available Not Available Locoregional Recurrence Not Available YES 45 No A52J Unknown WITH TUMOR Not Available Not Available NO Not Available Not Available Unknown NO WHITE NO Not Evaluated Not Applicable Not Applicable Not Applicable Not Applicable Not Applicable Not Applicable Not Applicable Not Applicable Not Applicable Stage IA Not Applicable Not Applicable Not Applicable 6th Not Applicable Not Applicable Not Applicable MX N0 T1 1980 NO YES O1 3 Lung Dead 2013 2007 1950

TCGA-44-3398 1163 Alive T1b N0 M0 Stage IA 77 R-Lower Not Applicable TCGA-44-3398 ffb0c0b7-165e-4439-b3e6-62431f40b7fe 11 -28392 Not Applicable 0 253 Not Available Lung Adenocarcinoma 60 Not Available Not Available Not Available Not Available Not Available Not Available Not Available Not Available NOT HISPANIC OR LATINO Not Available;Not Available Not Available;Not Available Not Available;Not Available Not Available;Not Available TCGA-44-3398-F5292;TCGA-44-3398-F38762 b338b0a9-78f2-44d5-a19c-5beca343abd0;9EF69CF2-2F1A-43EA-AACA-49F8E51CC52C 11;21 Not Applicable;Not Applicable Not Applicable;Not Applicable Not Applicable;Not Applicable 253;1163 Not Applicable;Not Applicable Not Available;Not Available Not Available;Scheduled Follow-up Submission Complete Remission/Response;Complete Remission/Response Not Available;Not Available Not Available;NO 10;12 Not Available;Not Available NO;NO Not Available;Not Available TUMOR FREE;TUMOR FREE NO;NO Complete Remission/Response;Complete Remission/Response Not Available;Not Available NO;NO Alive;Alive 2010;2012 FEMALE Lung Adenocarcinoma- Not Otherwise Specified (NOS) No C34.3 8140/3 C34.3 YES Not Available NO Not Available Not Available Not Available 10 Not Available Not Available Not Available Not Available Not Available Not Available Not Available Not Available Not Available Not Available 60 No 3398 Not Available TUMOR FREE 89 96 Not Available 84 87 Not Available YES WHITE Not Available R0 Not Applicable Not Applicable Not Applicable Not Applicable Not Applicable Not Applicable Not Applicable Not Applicable Not Applicable Stage IA Not Applicable Not Applicable Not Applicable 6th Not Applicable Not Applicable Not Applicable M0 N0 T1b 2009 YES NO 44 4 Lung Alive 2010 2009 1959

TCGA-49-AAR4 879 Dead T2 N2 MX Stage IIIA 51 L-Upper Not Applicable TCGA-49-AAR4 765AD5A5-134B-4680-A6D4-1DD113DC47CC 18 -18939 879 0 Not Available Not Available Lung Adenocarcinoma 109.4 TCGA-49-AAR4-D65009;TCGA-49-AAR4-D65010 4477C3EF-3A88-4C2F-955E-149887AC8C8F;AB97458F-B092-494A-BCEE-2FD308ED618A Not Available;Not Available 17;17 61;61 57;57 TAXOL;CARBOPLATIN Partial Response;Partial Response 9;9 Not Available;Not Available Not Available;Not Available Not Available;Not Available Not Available;Not Available Not Applicable;Not Applicable Not Available;Not Available Not Available;Not Available NO;NO Chemotherapy;Chemotherapy Not Available;Not Available Not Available;Not Available Not Available;Not Available NO;NO 2014;2014 1 Not Available Not Available Not Available Not Available Not Available Not Available Not Available NOT HISPANIC OR LATINO MALE Lung Adenocarcinoma- Not Otherwise Specified (NOS) No C34.1 8140/3 C34.1 YES 80 Not Available Not Available Not Available Not Available 6 Not Available Not Available Not Available Not Available Not Available Not Available Not Available Not Available Not Available NO 35 No AAR4 Preoperative WITH TUMOR 79.0 97.5 YES 81.2 100.2 Stable Disease YES BLACK OR AFRICAN AMERICAN YES Primary Tumor Field TCGA-49-AAR4-R65008 3013A2ED-A16A-4023-9E1D-FFE753568235 Not Available 17 153 118 Complete Response 9 Not Available 4,500 cGy NO External Not Applicable Not Available Not Available Not Available 2014 R0 Not Applicable Not Applicable Not Applicable Not Applicable Not Applicable Not Applicable Not Applicable Not Applicable Not Applicable Stage IIIA Not Applicable Not Applicable Not Applicable 6th Not Applicable Not Applicable Not Applicable MX N2 T2 Not Available NO YES 49 2 Lung Dead 2014 2006 Not Available

TCGA-44-7667 1097 Alive T3 N0 MX Stage IIB 49 R-Lower Not Applicable TCGA-44-7667 7f6455e8-fa3d-4452-acb2-8c9995073072 21 -18062 Not Applicable 0 557 Not Available Lung Adenocarcinoma 50 Not Available Not Available Not Available Not Available Not Available Not Available Not Available Not Available NOT HISPANIC OR LATINO Not Available;Not Available Not Available;Not Available Not Available;Not Available Not Available;Not Available TCGA-44-7667-F19718;TCGA-44-7667-F39968 c40ab53b-c1c0-402e-aedc-d93158731d75;3218051E-D4B6-489A-B881-073C5B57FBEA 21;7 Not Applicable;Not Applicable Not Applicable;Not Applicable Not Applicable;Not Applicable 557;1097 Not Applicable;Not Applicable Not Available;Not Available Scheduled Follow-up Submission;Scheduled Follow-up Submission Complete Remission/Response;Complete Remission/Response Not Available;Not Available Not Available;NO 12;2 Not Available;Not Available NO;NO Not Available;Not Available TUMOR FREE;TUMOR FREE NO;NO Complete Remission/Response;Complete Remission/Response Not Available;Not Available NO;NO Alive;Alive 2011;2013 FEMALE Lung Adenocarcinoma- Not Otherwise Specified (NOS) No C34.3 8140/3 C34.3 YES Not Available NO Not Available Not Available Not Available 12 Not Available Not Available Not Available Not Available Not Available Not Available Not Available Not Available Not Available Not Available 50 No 7667 Not Available TUMOR FREE Not Available Not Available Not Available 104 74 Not Available YES WHITE Not Available Not Available Not Applicable Not Applicable Not Applicable Not Applicable Not Applicable Not Applicable Not Applicable Not Applicable Not Applicable Stage IIB Not Applicable Not Applicable Not Applicable 6th Not Applicable Not Applicable Not Applicable MX N0 T3 Not Available YES NO 44 2 Lung Alive 2011 2009 1974

TCGA-38-7271 800 Dead T1 N0 M0 Stage IA 72 R-Upper Not Applicable TCGA-38-7271 8214a0d1-5e2d-4a7a-acb1-e5580755db83 10 -26440 800 0 Not Available Not Available Lung Adenocarcinoma 76 TCGA-38-7271-D18306;TCGA-38-7271-D18311;TCGA-38-7271-D18310 39b80d4f-cb2c-42f3-9e88-32dadd933c17;be281462-f8ee-4c19-95d2-3a8f7295265f;fac1fe79-b59c-4d7b-b36a-89b3c8e29166 Not Available;Not Available;Not Available 10;10;10 371;623;574 329;574;574 Cisplatin;Alimta;Carboplatin Not Available;Not Available;Not Available 11;11;11 3;2;1 30;500;Not Available mg/m2;mg/m2;AUC RECURRENCE;PALLIATIVE;PALLIATIVE Not Applicable;Not Applicable;Not Applicable 1;2;2 IV;IV;IV NO;NO;NO Chemotherapy;Chemotherapy;Chemotherapy Not Available;Not Available;Not Available 324;1760;798 mg;mg;mg Not Available;Not Available;Not Available 2011;2011;2011 Not Available Not Available NO Not Available Not Available Not Available NO Not Available NOT HISPANIC OR LATINO YES;YES;NO YES;YES;YES NO;NO;NO Not Available;NO;NO TCGA-38-7271-F18305;TCGA-38-7271-F18308;TCGA-38-7271-F18312 f7734211-9e7c-43ca-90e7-5c8de6e7bfee;24d3b53b-221b-4620-9d62-d627741e2a70;620bde19-e077-43c4-b7e0-f3b00df96316 10;10;10 Not Available;Not Available;Not Available Not Available;Not Available;Not Available 800;800;800 Not Available;Not Available;Not Available 304;561;633 Not Available;Not Available;Not Available Additional New Tumor Event;Additional New Tumor Event;Additional New Tumor Event Stable Disease;Progressive Disease;Progressive Disease Not Available;Not Available;Not Available Not Available;Not Available;Not Available 11;11;11 Not Available;Not Available;Not Available YES;YES;YES Not Available;Not Available;Not Available WITH TUMOR;WITH TUMOR;WITH TUMOR NO;Not Available;Not Available Complete Remission/Response;Not Available;Not Available Not Available;Not Available;Not Available NO;Not Available;Not Available Dead;Dead;Dead 2011;2011;2011 FEMALE Lung Adenocarcinoma- Not Otherwise Specified (NOS) No C34.1 8140/3 C34.1 YES Not Available NO Not Available Not Available Not Available 11 Not Available Not Available Not Available Not Available Not Available Not Available Not Available Not Available Not Available Not Available 25 Yes 7271 Not Available WITH TUMOR 46 45 Not Available 43 40 Not Available YES WHITE Not Available Distant site;Distant site;Local Recurrence TCGA-38-7271-R18309;TCGA-38-7271-R18313;TCGA-38-7271-R18307 06943636-0118-4a9a-80ad-eda14819da97;a027a42b-2d51-47e7-9dcf-60f81b2d234d;a8e5e91e-4df7-465e-88b0-eb30765f0f30 2;3;1 10;10;10 564;665;379 564;633;329 Not Available;Not Available;Not Available 11;11;11 1;14;23 2000;2500;7000 NO;NO;NO OTHER;EXTERNAL BEAM;EXTERNAL BEAM Cyber Knife Stereotactic Radiosurgery;Not Applicable;Not Applicable PALLIATIVE;PALLIATIVE;RECURRENCE Not Available;Not Available;Not Available cGy;cGy;cGy 2011;2011;2011 R0 Not Applicable Not Applicable Not Applicable Not Applicable Not Applicable Not Applicable Not Applicable Not Applicable Not Applicable Stage IA Not Applicable Not Applicable Not Applicable 6th Not Applicable Not Applicable Not Applicable M0 N0 T1 1999 NO YES 38 4 Lung Dead 2011 2006 1971

TCGA-44-3918 1036 Alive T1 N0 M0 Stage IA FPPP TCGA 60 L-Upper Not Applicable TCGA-44-3918 6e3b6b72-142d-4b8d-a462-28a205796e41 7 -22236 Not Applicable 0 197 197 Lung Adenocarcinoma 76 TCGA-44-3918-D20986;TCGA-44-3918-D20985 16f14090-dd3a-4b0c-91c7-998cee0e8d40;4cc469d8-02e3-4ef9-88d3-cd70b4fbc386 Not Available;Not Available 25;25 648;648 543;543 Carboplatin;Alimta Not Available;Not Available 1;1 6;6 500;930 mg;mg RECURRENCE;RECURRENCE Not Applicable;Not Applicable 1;1 IV;IV NO;NO Chemotherapy;Chemotherapy Not Available;Not Available 3380;5660 mg;mg Not Available;Not Available 2012;2012 Not Available Not Available Not Available Not Available Not Available Not Available Not Available Not Available NOT HISPANIC OR LATINO NO;YES;YES NO;YES;YES NO;NO;NO NO;Not Available;Not Available TCGA-44-3918-F5231;TCGA-44-3918-F21270;TCGA-44-3918-F38999 439f02a5-f262-430e-8cda-1308ad6cbb3d;02d8cb32-463a-4a11-bdf6-defceba7c69b;2C352F95-6915-4088-A9C0-D97582F74B5C 8;8;10 Not Applicable;Not Available;Not Available Not Applicable;Not Available;Not Available Not Applicable;Not Applicable;Not Applicable 197;715;1036 Not Applicable;511;511 Not Available;0;Not Available Not Available;Additional New Tumor Event;Scheduled Follow-up Submission Complete Remission/Response;Stable Disease;Stable Disease Not Available;Not Available;90 Not Available;Not Available;NO 10;2;1 Not Available;Not Available;Locoregional Recurrence NO;YES;YES Not Available;Other;Other TUMOR FREE;WITH TUMOR;WITH TUMOR NO;Not Available;NO Complete Remission/Response;Not Available;Complete Remission/Response Not Available;Not Available;Biopsy with Histologic Confirmation;Convincing Imaging NO;Not Available;NO Alive;Alive;Alive 2010;2012;2013 FEMALE Lung Adenocarcinoma- Not Otherwise Specified (NOS) No C34.1 8140/3 C34.1 YES Not Available NO Not Available Not Available Not Available 10 Not Available Not Available Not Available Not Available Not Available Not Available Not Available Not Available Not Available Not Available 20 No 3918 Not Available TUMOR FREE Not Available Not Available Not Available 103 104 Not Available YES WHITE Not Available Local Recurrence TCGA-44-3918-R21268 2b4b3df7-bfff-4e7e-b5be-3cad8f87f82d 1 8 697 686 Not Available 2 10 3000 NO EXTERNAL BEAM Not Applicable PALLIATIVE Not Available cGy 2012 R0 Not Applicable Not Applicable Not Applicable Not Applicable Not Applicable Not Applicable Not Applicable Not Applicable Not Applicable Stage IA Not Applicable Not Applicable Not Applicable 6th Not Applicable Not Applicable Not Applicable M0 N0 T1 1994 YES NO 44 3 Lung Alive 2010 2010 1974

TCGA-97-A4M5 634 Alive T1b N0 M0 Stage IA 83 R-Upper Not Applicable TCGA-97-A4M5 5FE77D4A-A8A5-4C90-8FF2-9C3BBBB309EF 7 -30665 Not Applicable 0 132 Not Available Lung Adenocarcinoma 51 Not Evaluated Not Available NO Not Available Not Available Not Available NO Not Available NOT HISPANIC OR LATINO Not Available Not Available Not Available Not Available TCGA-97-A4M5-F57443 45F6F53F-E17E-47FD-A616-202C86D48D1F 12 Not Applicable Not Applicable Not Applicable 634 Not Applicable Not Evaluated Scheduled Follow-up Submission Complete Remission/Response Not Evaluated NO 3 Not Available NO Not Evaluated TUMOR FREE NO Complete Remission/Response Not Available NO Alive 2014 MALE Lung Adenocarcinoma Mixed Subtype No C34.1 8255/3 C34.1 YES Not Evaluated YES YES G12C Unknown 3 Not Available Not Available Not Available Not Available Not Available Not Available Not Available Not Available Not Available NO 6 No A4M5 Not Evaluated Unknown 84 94 NO 85 98 Complete Remission/Response YES WHITE NO Not Evaluated Not Applicable Not Applicable Not Applicable Not Applicable Not Applicable Not Applicable Not Applicable Not Applicable Not Applicable Stage IA Not Applicable Not Applicable Not Applicable 7th Not Applicable Not Applicable Not Applicable M0 N0 T1b 1982 YES NO 97 3 Lung Alive 2013 2012 1952

TCGA-NJ-A4YP 50 Alive T2a N0 M0 Stage IB 52 R-Lower Not Applicable TCGA-NJ-A4YP 42259AA6-C9F5-4309-A956-DBF930312F44 27 -19106 Not Applicable 0 50 Not Available Lung Adenocarcinoma Not Available Unknown Not Available NO Not Available Not Available Not Available NO Not Available NOT HISPANIC OR LATINO Not Available Not Available Not Available Not Available TCGA-NJ-A4YP-F50888 5B07D5AE-C22E-4DED-8460-EA2FE846A2B4 1 Not Applicable Not Applicable Not Applicable 50 Not Applicable Not Available Scheduled Follow-up Submission Stable Disease Not Available YES 11 Not Available NO Not Available Not Available Not Available Not Available Not Available Not Available Alive 2013 MALE Lung Papillary Adenocarcinoma No C34.3 8255/3 C34.3 YES Unknown NO Not Available Not Available Unknown 9 Not Available Not Available Not Available Not Available Not Available Not Available Not Available Not Available Not Available NO 60 No A4YP Unknown TUMOR FREE Not Available Not Available NO Not Available Not Available Stable Disease NO WHITE NO Not Evaluated Not Applicable Not Applicable Not Applicable Not Applicable Not Applicable Not Applicable Not Applicable Not Applicable Not Applicable Stage IB Not Applicable Not Applicable Not Applicable 7th Not Applicable Not Applicable Not Applicable M0 N0 T2a Not Available Not Available Not Available NJ 5 Lung Alive 2013 2011 Not Available

TCGA-97-7552 1932 Alive T2 N0 MX Stage IB 70 L-Lower Not Applicable TCGA-97-7552 1b110eb4-a01d-4c50-ba18-77da4dedd549 17 -25578 Not Applicable 0 1476 Not Available Lung Adenocarcinoma Not Available Not Available Not Available Not Available Not Available Not Available Not Available Not Available Not Available NOT HISPANIC OR LATINO NO;NO NO;NO Not Available;Not Available Not Available;Not Available TCGA-97-7552-F18844;TCGA-97-7552-F37174 3e54d908-6857-428c-9f5c-f1832e592ace;85ED4CFE-DA99-429B-91F9-D17B739AD495 28;29 Not Available;Not Available Not Available;Not Available Not Applicable;Not Applicable 1932;1932 798;798 Not Available;Not Available Additional New Tumor Event;Scheduled Follow-up Submission Complete Remission/Response;Complete Remission/Response Not Available;Not Available Not Available;NO 11;11 Not Available;New Primary Tumor YES;YES Not Available;Not Available TUMOR FREE;TUMOR FREE NO;NO Not Available;Stable Disease Not Available;Not Available NO;NO Alive;Alive 2011;2012 MALE Lung Bronchioloalveolar Carcinoma Mucinous No C34.3 8253/3 C34.3 YES Not Available Not Available Not Available Not Available Not Available 11 Not Available Not Available Not Available Not Available Not Available Not Available Not Available Not Available Not Available Not Available 40 No 7552 Not Available TUMOR FREE Not Available Not Available Not Available 82 81 Not Available YES WHITE Not Available Not Available Not Applicable Not Applicable Not Applicable Not Applicable Not Applicable Not Applicable Not Applicable Not Applicable Not Applicable Stage IB Not Applicable Not Applicable Not Applicable 6th Not Applicable Not Applicable Not Applicable MX N0 T2 1985 NO YES 97 3 Lung Alive 2011 2007 Not Available

TCGA-86-8073 740 Alive T2a N0 M0 Stage IB 58 L-Upper Not Applicable TCGA-86-8073 cd902d08-215e-4bd0-88e4-4fd01ab43cbf 13 -21214 Not Applicable 0 13 Not Available Lung Adenocarcinoma Not Available Not Available Not Available NO Not Available Not Available Not Available NO Not Available NOT HISPANIC OR LATINO Not Available Not Available Not Available Not Available TCGA-86-8073-F66082 FD02835A-886E-4E52-B3D1-53DCF8D4BB81 2 Not Applicable Not Applicable Not Applicable 740 Not Applicable 0 Scheduled Follow-up Submission Complete Remission/Response 100 NO 10 Not Available NO Other TUMOR FREE NO Complete Remission/Response Not Available NO Alive 2014 MALE Lung Bronchioloalveolar Carcinoma Nonmucinous No C34.1 8250/3 C34.1 YES Not Available NO Not Available Not Available Peripheral Lung 6 Not Available Not Available Not Available Not Available Not Available Not Available Not Available Not Available Not Available Unknown 40 No 8073 Not Available TUMOR FREE Not Available Not Available Unknown Not Available Not Available Complete Remission/Response NO WHITE Unknown R0 Not Applicable Not Applicable Not Applicable Not Applicable Not Applicable Not Applicable Not Applicable Not Applicable Not Applicable Stage IB Not Applicable Not Applicable Not Applicable 7th Not Applicable Not Applicable Not Applicable M0 N0 T2a Not Available YES NO 86 2 Lung Alive 2012 2011 1970

TCGA-97-7938 18 Dead T1a N0 MX Stage IA 76 R-Upper Not Applicable TCGA-97-7938 e6e75590-9520-4b1c-8a28-4a1d2b2218fc 13 -27980 18 0 Not Available Not Available Lung Adenocarcinoma 53 Not Available Not Available YES Not Available Not Available Not Available NO Not Available NOT HISPANIC OR LATINO Not Available Not Available Not Available Not Available TCGA-97-7938-F57460 0EB1C458-4194-49CB-8BC9-D806490FF5E5 11 Not Applicable Not Applicable 18 Not Available Not Applicable Not Evaluated Scheduled Follow-up Submission Complete Remission/Response Not Evaluated NO 3 Not Available NO Not Evaluated TUMOR FREE NO Complete Remission/Response Not Available NO Dead 2014 FEMALE Lung Bronchioloalveolar Carcinoma Nonmucinous No C34.1 8252/3 C34.1 YES Not Available YES YES G12C Not Available 2 Not Available Not Available Not Available Not Available Not Available Not Available Not Available Not Available Not Available Not Available 40 No 7938 Not Available TUMOR FREE 59 54 Not Available 58 50 Not Available YES WHITE Not Available R0 Not Applicable Not Applicable Not Applicable Not Applicable Not Applicable Not Applicable Not Applicable Not Applicable Not Applicable Stage IA Not Applicable Not Applicable Not Applicable 7th Not Applicable Not Applicable Not Applicable MX N0 T1a 1996 YES NO 97 3 Lung Dead 2012 2011 Not Available

TCGA-49-6761 354 Alive T1 N2 MX Stage IIIA 68 R-Upper Not Applicable TCGA-49-6761 4cd3d483-2283-4c6a-a57a-444216119d34 2 -24849 Not Applicable 0 1 Not Available Lung Adenocarcinoma 1 Not Available Not Available Not Available Not Available Not Available Not Available Not Available Not Available NOT HISPANIC OR LATINO NO NO Not Available Not Available TCGA-49-6761-F22056 8ad6b3d6-48a1-4f9e-8ea6-cf0d7394cdf6 23 Not Applicable Not Applicable Not Applicable 354 Not Applicable Not Available Scheduled Follow-up Submission Not Available Not Available Not Available 2 Not Available NO Not Available Not Available NO Complete Remission/Response Not Available NO Alive 2012 FEMALE Lung Adenocarcinoma- Not Otherwise Specified (NOS) No C34.1 8140/3 C34.1 YES Not Available Not Available Not Available Not Available Not Available 3 Not Available Not Available Not Available Not Available Not Available Not Available Not Available Not Available Not Available Not Available 50 Yes 6761 Not Available TUMOR FREE 64 56 Not Available 64 52 Not Available YES WHITE Not Available R0 Not Applicable Not Applicable Not Applicable Not Applicable Not Applicable Not Applicable Not Applicable Not Applicable Not Applicable Stage IIIA Not Applicable Not Applicable Not Applicable 6th Not Applicable Not Applicable Not Applicable MX N2 T1 Not Available NO YES 49 4 Lung Alive 2012 2009 Not Available

TCGA-75-5125 2027 Dead T2 N1 M0 Stage IIB Not Available R-Upper Not Applicable TCGA-75-5125 205759a6-6391-491b-9857-0080c3a5871e 31 Not Available Not Applicable Not Available Not Available Not Available Lung Adenocarcinoma Not Available Not Available Not Available NO Not Available Not Available Not Available NO Not Available Not Available Not Available;NO Not Available;YES Not Available;NO Not Available;Not Available TCGA-75-5125-F11748;TCGA-75-5125-F72028 406f25c2-f2f7-4aa9-9016-034a5798f561;6219D889-CA6E-4935-8D96-082E561B630B 31;2 Not Applicable;Not Available Not Applicable;Not Available Not Applicable;2027 Not Available;Not Available Not Applicable;1752 Not Available;Unknown Not Available;Scheduled Follow-up Submission Complete Remission/Response;Progressive Disease Not Available;Not Evaluated Not Available;NO 3;4 Not Available;Not Available NO;YES Adjuvant therapy;Not Available TUMOR FREE;WITH TUMOR NO;NO Complete Remission/Response;Complete Remission/Response Not Available;Not Available NO;NO Alive;Dead 2011;2015 MALE Lung Adenocarcinoma- Not Otherwise Specified (NOS) No C34.1 8140/3 C34.1 YES Not Available NO Not Available Not Available Peripheral Lung 3 Not Available Not Available Not Available Not Available Not Available Not Available Not Available Not Available Not Available Not Available 25 No 5125 Other TUMOR FREE Not Available Not Available Not Available Not Available Not Available Not Available Not Available Not Available Not Available R0 Not Applicable Not Applicable Not Applicable Not Applicable Not Applicable Not Applicable Not Applicable Not Applicable Not Applicable Stage IIB Not Applicable Not Applicable Not Applicable 6th Not Applicable Not Applicable Not Applicable M0 N1 T2 1987 NO YES 75 3 Lung Alive 2011 Not Available Not Available

TCGA-91-A4BD 603 Alive T1b N1 MX Stage IIA 78 L-Lower Not Applicable TCGA-91-A4BD 0D980584-4484-4063-8243-C3CD67F6F588 5 -28544 Not Applicable 0 218 Not Available Lung Adenocarcinoma 94 1 Not Available NO Not Available Not Available Not Available Not Available Not Available NOT HISPANIC OR LATINO Not Available Not Available Not Available Not Available TCGA-91-A4BD-F58335 13A3B033-52C9-4C1F-B2FB-196BBE0A75D9 7 Not Applicable Not Applicable Not Applicable 603 Not Applicable Not Evaluated Scheduled Follow-up Submission Complete Remission/Response Not Evaluated NO 4 Not Available NO Not Available TUMOR FREE NO Complete Remission/Response Not Available NO Alive 2014 MALE Lung Adenocarcinoma- Not Otherwise Specified (NOS) No C34.3 8140/3 C34.3 YES Unknown NO Not Available Not Available Not Available 2 Not Available Not Available Not Available Not Available Not Available Not Available Not Available Not Available Not Available NO Not Available No A4BD Other TUMOR FREE 70 92 NO Not Available Not Available Complete Remission/Response YES WHITE NO RX Not Applicable Not Applicable Not Applicable Not Applicable Not Applicable Not Applicable Not Applicable Not Applicable Not Applicable Stage IIA Not Applicable Not Applicable Not Applicable 7th Not Applicable Not Applicable Not Applicable MX N1 T1b 1981 YES NO 91 3 Lung Alive 2013 2012 Not Available

TCGA-55-7910 1040 Alive T2b N0 M0 Stage IIA 50 L-Lower Not Applicable TCGA-55-7910 b2bc53d6-ef78-4ae9-a6b5-1c7cad9a43eb 10 -18289 Not Applicable 0 203 Not Available Lung Adenocarcinoma 44 TCGA-55-7910-D66141 76D8449C-257D-474B-A830-EDE15DAE2DDD Not Available 3 Not Available 253 Cisplatin Complete Response 10 Not Available Not Available Not Available Not Available Not Applicable Not Available Not Available NO Chemotherapy Not Available Not Available Not Available NO 2014 1 Not Available Not Available Not Available Not Available Not Available Not Available Not Available NOT HISPANIC OR LATINO NO YES Unknown Unknown TCGA-55-7910-F66140 50A3A722-AEC7-4FBC-AFDA-7B45FEA1263A 3 Not Available Not Available Not Applicable 1040 1018 Not Available Scheduled Follow-up Submission Not Applicable Not Available NO 10 Not Available YES Not Available WITH TUMOR YES Complete Remission/Response Not Available NO Alive 2014 FEMALE Lung Adenocarcinoma- Not Otherwise Specified (NOS) No C34.3 8140/3 C34.3 YES 80 Not Available Not Available Not Available Peripheral Lung 3 Not Available Not Available Not Available Not Available Not Available Not Available Not Available Not Available Not Available Not Available 15 No 7910 Preoperative WITH TUMOR 91 91 Not Available 97 86 Not Available YES BLACK OR AFRICAN AMERICAN Not Available Unknown TCGA-55-7910-R66142 ACCCBE41-BDFD-4BDF-9192-6EF3399B09D5 Not Available 3 Not Available 1040 Not Applicable 10 Not Available Not Available YES External Not Applicable Not Available Not Available Not Available 2014 R0 Not Applicable Not Applicable Not Applicable Not Applicable Not Applicable Not Applicable Not Applicable Not Applicable Not Applicable Stage IIA Not Applicable Not Applicable Not Applicable 7th Not Applicable Not Applicable Not Applicable M0 N0 T2b 2011 YES NO 55 4 Lung Alive 2012 2011 1996

TCGA-05-4410 0 Alive T2 N0 M0 Stage IB 62 L-Upper Not Applicable TCGA-05-4410 d28e465c-e553-4c53-8bb7-3d8199b9a8a5 22 -22888 Not Applicable 0 0 Not Available Lung Adenocarcinoma Not Available Not Available Not Available Not Available Not Available Not Available Not Available Not Available Not Available Not Available MALE Lung Adenocarcinoma Mixed Subtype No C34.1 8255/3 C34.1 YES Not Available Not Available Not Available Not Available Not Available 7 Not Available Not Available Not Available Not Available Not Available Not Available Not Available Not Available Not Available Not Available 98 Yes 4410 Not Available Not Available Not Available Not Available Not Available Not Available Not Available Not Available Not Available Not Available Not Available RX Not Applicable Not Applicable Not Applicable Not Applicable Not Applicable Not Applicable Not Applicable Not Applicable Not Applicable Stage IB Not Applicable Not Applicable Not Applicable 6th Not Applicable Not Applicable Not Applicable M0 N0 T2 2007 NO YES 05 4 Lung Alive 2010 2007 1958

TCGA-50-5939 460 Dead T2 N0 M0 Stage IB 85 R-Upper Not Applicable TCGA-50-5939 12b79b4f-c28c-418e-a1e7-e622f33fdcc2 2 -31236 460 0 Not Available Not Available Lung Adenocarcinoma Not Available Not Available Not Available Not Available Not Available Not Available Not Available Not Available Not Available NOT HISPANIC OR LATINO Not Available Not Available Not Available Not Available TCGA-50-5939-F32124 2105f694-4ccf-4c6e-8eba-917a55c789e3 17 Not Applicable Not Applicable 460 Not Available Not Applicable Not Available Scheduled Follow-up Submission Complete Remission/Response Not Available Not Available 5 Not Available NO Not Available TUMOR FREE NO Complete Remission/Response Not Available NO Dead 2012 MALE Lung Adenocarcinoma Mixed Subtype No C34.1 8255/3 C34.1 YES Not Available Not Available Not Available Not Available Not Available 7 Not Available Not Available Not Available Not Available Not Available Not Available Not Available Not Available Not Available Not Available 15 No 5939 Not Available TUMOR FREE Not Available Not Available Not Available Not Available Not Available Not Available Not Available WHITE Not Available Not Available Not Applicable Not Applicable Not Applicable Not Applicable Not Applicable Not Applicable Not Applicable Not Applicable Not Applicable Stage IB Not Applicable Not Applicable Not Applicable 6th Not Applicable Not Applicable Not Applicable M0 N0 T2 1968 NO YES 50 3 Lung Dead 2011 2008 Not Available

TCGA-62-A46P 594 Dead T2 N0 M0 Stage IB 65 L-Upper Not Applicable TCGA-62-A46P 0C59FF96-5E4A-4664-A0B1-D057B4725483 29 -23993 594 0 Not Available Not Available Lung Adenocarcinoma Not Available Unknown Not Available NO Not Available Not Available Not Available NO Not Available NOT HISPANIC OR LATINO MALE Lung Adenocarcinoma Mixed Subtype No C34.1 8255/3 C34.1 YES 100 NO Not Available Not Available Central Lung 10 YES NO 267 Not Available Not Available YES 267 Distant Metastasis Convincing Imaging YES 40 No A46P Preoperative TUMOR FREE Not Available Not Available NO 66 78 Complete Remission/Response YES WHITE NO R0 Not Applicable Not Applicable Not Applicable Not Applicable Not Applicable Not Applicable Not Applicable Not Applicable Not Applicable Stage IB Not Applicable Not Applicable Not Applicable 6th Not Applicable Not Applicable Not Applicable M0 N0 T2 Not Available NO YES 62 4 Lung Dead 2012 2006 Not Available

TCGA-50-5936 257 Dead T2 N2 M0 Stage IIIA 58 R-Lower Not Applicable TCGA-50-5936 ef42ae4c-a108-468e-beed-437cf3cf2962 2 -21238 257 0 Not Available Not Available Lung Adenocarcinoma Not Available TCGA-50-5936-D41910 26C486FA-3DCE-4BA5-A30E-544DBBDDEE25 Not Available 3 257 188 TAXOL Clinical Progressive Disease 4 Not Available Not Available Not Available Not Available Not Applicable Not Available Not Available NO Chemotherapy Not Available Not Available Not Available NO 2013 Not Available Not Available YES Not Available Not Available Not Available Not Available Not Available NOT HISPANIC OR LATINO YES NO YES NO TCGA-50-5936-F32110 a515c2f5-9d6e-4d08-a136-8480b38b7e6b 17 177 Not Available 257 Not Available 150 Not Available Scheduled Follow-up Submission Progressive Disease Not Available NO 5 Not Available YES Not Available WITH TUMOR NO Progressive Disease Biopsy with Histologic Confirmation;Convincing Imaging NO Dead 2012 MALE Mucinous (Colloid) Carcinoma No C34.30 8480/3 C34.3 YES Not Available YES YES G12C Not Available 7 Not Available Not Available Not Available Not Available Not Available Not Available Not Available Not Available Not Available Not Available Not Available No 5936 Not Available Not Available Not Available Not Available Not Available Not Available Not Available Not Available Not Available WHITE Not Available Not Available Not Applicable Not Applicable Not Applicable Not Applicable Not Applicable Not Applicable Not Applicable Not Applicable Not Applicable Stage IIIA Not Applicable Not Applicable Not Applicable 6th Not Applicable Not Applicable Not Applicable M0 N2 T2 Not Available NO YES 50 Not Available Lung Dead 2011 2007 Not Available

TCGA-78-8660 321 Dead T2 N1 M0 Stage IIB 69 R-Lower Not Applicable TCGA-78-8660 781f40c9-c099-4c96-8269-ebe2a449c93d 23 -25422 321 0 Not Available Not Available Lung Adenocarcinoma 87 1 Not Available NO Not Available Not Available Not Available NO Not Available Not Evaluated MALE Lung Adenocarcinoma- Not Otherwise Specified (NOS) No C34.3 8140/3 C34.3 YES Not Available NO Not Available Not Available Peripheral Lung 10 NO YES 242 Not Available Not Available YES 260 Distant Metastasis Biopsy with Histologic Confirmation YES 11 Yes, History of Prior Malignancy 8660 Preoperative WITH TUMOR Not Available Not Available NO 72 108 Unknown YES Not Evaluated YES Primary Tumor Field TCGA-78-8660-R36074 4B964C80-FF7E-42DD-93F3-6AA45D3B94EA Not Available 23 97 Not Available Radiographic Progressive Disease 10 Not Available Not Available NO External Not Applicable Not Available Not Available Not Available 2012 R0 Not Applicable Not Applicable Not Applicable Not Applicable Not Applicable Not Applicable Not Applicable Not Applicable Not Applicable Stage IIB Not Applicable Not Applicable Not Applicable 6th Not Applicable Not Applicable Not Applicable M0 N1 T2 2005 NO YES 78 4 Lung Dead 2012 2005 1954

TCGA-99-8028 1118 Alive T1a N0 M0 Stage IA 50 L-Upper Not Applicable TCGA-99-8028 9759bf3a-74ff-433f-99bb-f9406f222ed2 15 -18542 Not Applicable 0 459 Not Available Lung Adenocarcinoma 71 0 Not Available YES Not Available Not Available RT-PCR YES Not Available NOT HISPANIC OR LATINO Not Available Not Available Not Available Not Available TCGA-99-8028-F57699 ADD85632-529E-47C6-BEDA-7857D1A5C780 17 Not Applicable Not Applicable Not Applicable 1118 Not Applicable Not Available Scheduled Follow-up Submission Complete Remission/Response Not Available NO 4 Not Available NO Not Available TUMOR FREE NO Complete Remission/Response Not Available NO Alive 2014 FEMALE Lung Adenocarcinoma- Not Otherwise Specified (NOS) No C34.1 8140/3 C34.1 YES Not Evaluated YES YES G12C Unknown 6 Not Available Not Available Not Available Not Available Not Available Not Available Not Available Not Available Not Available NO 30 No 8028 Preoperative TUMOR FREE 91 98 NO 91 94 Not Available YES BLACK OR AFRICAN AMERICAN NO R0 Not Applicable Not Applicable Not Applicable Not Applicable Not Applicable Not Applicable Not Applicable Not Applicable Not Applicable Stage IA Not Applicable Not Applicable Not Applicable 7th Not Applicable Not Applicable Not Applicable M0 N0 T1a 2011 NO YES 99 4 Lung Alive 2012 2011 1981

TCGA-64-1677 628 Dead T2 N2 M0 Stage IIIA 77 R-Lower Not Applicable TCGA-64-1677 8a16ddb3-4861-4128-bed9-0b1f94fe8cf7 18 -28482 628 0 Not Available Not Available Lung Adenocarcinoma Not Available TCGA-64-1677-D8693;TCGA-64-1677-D8696;TCGA-64-1677-D8692 654eaa9e-2e91-4ef6-9e36-02a237406432;8a35805b-e7b8-4f10-8aae-d78f856a372e;a4d38c0d-dd9a-4143-ae98-9be774d485c5 Not Available;Not Available;Not Available 18;18;18 121;Not Available;121 51;Not Available;51 Gemcitabine;Not Available;Carboplatin Not Available;Not Available;Not Available 2;2;2 4;2;4 1600;Not Available;425 mg;Not Available;mg ADJUVANT;RECURRENCE;ADJUVANT Not Applicable;Not Applicable;Not Applicable 1;2;1 IV;Not Available;IV NO;NO;NO Chemotherapy;Chemotherapy;Chemotherapy Not Available;Not Available;Not Available Not Available;Not Available;Not Available Not Available;Not Available;Not Available Not Available;Not Available;Not Available 2011;2011;2011 Not Available Not Available Not Available Not Available Not Available Not Available Not Available Not Available NOT HISPANIC OR LATINO YES YES NO NO TCGA-64-1677-F8691 52f57fe8-b728-4758-942d-efc01c5cbf0e 18 Not Available Not Available 628 Not Available 360 Not Available Not Available Not Available 0 Not Available 2 Not Available YES Not Available Not Available YES Not Available Not Available NO Dead 2011 FEMALE Lung Adenocarcinoma- Not Otherwise Specified (NOS) No C34.1 8140/3 C34.1 YES 0 Not Available Not Available Not Available Not Available 2 Not Available Not Available Not Available Not Available Not Available Not Available Not Available Not Available Not Available Not Available 50 Yes 1677 Not Available Not Available Not Available Not Available Not Available Not Available Not Available Not Available Not Available WHITE Not Available Regional site TCGA-64-1677-R8694 45477fba-0eb8-4966-8c18-d8e9b539d9fc 2 18 448 417 Not Available 2 31 Not Available NO EXTERNAL BEAM Not Applicable RECURRENCE Not Available cGy 2011 R0 Not Applicable Not Applicable Not Applicable Not Applicable Not Applicable Not Applicable Not Applicable Not Applicable Not Applicable Stage IIIA Not Applicable Not Applicable Not Applicable Not Available Not Applicable Not Applicable Not Applicable M0 N2 T2 Not Available NO YES 64 2 Lung Dead 2011 2005 1955

TCGA-78-7162 3169 Dead T1 N0 M0 Stage IA 75 L-Upper Not Applicable TCGA-78-7162 16cd58db-d52c-403e-9e9c-6da2e99f6bad 3 -27593 3169 0 Not Available Not Available Lung Adenocarcinoma Not Available 0 Not Available NO Not Available Not Available Not Available NO Not Available Not Available NO;YES YES;NO NO;NO NO;NO TCGA-78-7162-F17125;TCGA-78-7162-F17127 19df3a15-5642-4b23-ab3b-d6fbc2d17193;125d989e-fe36-43e7-b9f2-515970b19506 3;3 Not Available;Not Available Not Available;Not Available 3169;3169 Not Available;Not Available 2218;2740 Not Available;Not Available Scheduled Follow-up Submission;Additional New Tumor Event Not Available;Not Available Not Available;Not Available Not Available;Not Available 10;10 Not Available;Not Available YES;YES Not Available;Not Available Not Available;Not Available NO;NO Not Available;Not Available Not Available;Not Available NO;NO Dead;Dead 2011;2011 MALE Lung Adenocarcinoma Mixed Subtype No C34.1 8255/3 C34.1 YES Not Available NO Not Available Not Available Peripheral Lung 10 Not Available Not Available Not Available Not Available Not Available Not Available Not Available Not Available Not Available Not Available 12 No 7162 Preoperative Not Available Not Available Not Available Not Available Not Available Not Available Not Available Not Available WHITE Not Available Regional site TCGA-78-7162-R17154 25072ff0-dbfb-45ab-9bfc-f640a5b12e6a 1 4 Not Available Not Available Not Available 10 Not Available Not Available NO EXTERNAL BEAM Not Applicable RECURRENCE Not Available Not Available 2011 R0 Not Applicable Not Applicable Not Applicable Not Applicable Not Applicable Not Applicable Not Applicable Not Applicable Not Applicable Stage IA Not Applicable Not Applicable Not Applicable 6th Not Applicable Not Applicable Not Applicable M0 N0 T1 Not Available NO YES 78 2 Lung Dead 2011 1991 1940

TCGA-55-8092 154 Dead T3 N0 MX Stage IIB 75 R-Upper Not Applicable TCGA-55-8092 4b92fce2-3772-40eb-ab34-a27937b58590 8 -27597 Not Applicable 0 26 Not Available Lung Adenocarcinoma 19 Not Available Not Available Not Available Not Available Not Available Not Available Not Available Not Available NOT HISPANIC OR LATINO NO NO NO Not Available TCGA-55-8092-F46643 08F57135-8BDA-492F-9F56-7D5C9BEF9DDE 13 Not Available Not Available 154 Not Available 127 Not Evaluated Scheduled Follow-up Submission Progressive Disease Not Evaluated NO 8 Locoregional Recurrence YES Not Evaluated WITH TUMOR NO Complete Remission/Response Convincing Imaging NO Dead 2013 MALE Lung Adenocarcinoma- Not Otherwise Specified (NOS) No C34.1 8140/3 C34.1 YES Not Available Not Available Not Available Not Available Not Available 7 Not Available Not Available Not Available Not Available Not Available Not Available Not Available Not Available Not Available NO 40 No 8092 Not Available TUMOR FREE 2 19 Unknown 66 68 Complete Remission/Response YES WHITE Unknown R0 Not Applicable Not Applicable Not Applicable Not Applicable Not Applicable Not Applicable Not Applicable Not Applicable Not Applicable Stage IIB Not Applicable Not Applicable Not Applicable 7th Not Applicable Not Applicable Not Applicable MX N0 T3 2012 YES NO 55 4 Lung Alive 2012 2012 1972

TCGA-44-5645 852 Alive T1 NX Stage IA FPPP TCGA 61 L-Upper Not Applicable TCGA-44-5645 ddeacccf-0953-4952-8a4e-c2617f2e7bcc 13 -22471 Not Applicable 0 208 Not Available Lung Adenocarcinoma 82 Not Available Not Available Not Available Not Available Not Available Not Available Not Available Not Available NOT HISPANIC OR LATINO Not Available;Not Available Not Available;Not Available Not Available;NO Not Available;Not Available TCGA-44-5645-F14601;TCGA-44-5645-F38800 9d996467-3fd5-408f-9225-258a86d0bd01;241EC925-44CC-4331-B9AF-BF5E912AF547 4;24 Not Applicable;Not Applicable Not Applicable;Not Applicable Not Applicable;Not Applicable 383;852 Not Applicable;Not Applicable Not Available;Not Available Scheduled Follow-up Submission;Scheduled Follow-up Submission Complete Remission/Response;Complete Remission/Response Not Available;Not Available Not Available;NO 8;12 Not Available;Not Available NO;NO Not Available;Not Available TUMOR FREE;TUMOR FREE NO;NO Complete Remission/Response;Complete Remission/Response Not Available;Not Available NO;NO Alive;Alive 2011;2012 FEMALE Lung Adenocarcinoma- Not Otherwise Specified (NOS) No C34.1 8140/3 C34.1 YES Not Available Not Available Not Available Not Available Not Available 4 Not Available Not Available Not Available Not Available Not Available Not Available Not Available Not Available Not Available Not Available 5 No 5645 Not Available TUMOR FREE Not Available Not Available Not Available 90 100 Not Available YES BLACK OR AFRICAN AMERICAN Not Available Not Available Not Applicable Not Applicable Not Applicable Not Applicable Not Applicable Not Applicable Not Applicable Not Applicable Not Applicable Stage IA Not Applicable Not Applicable Not Applicable 7th Not Applicable Not Applicable Not Applicable Not Available NX T1 1973 YES NO 44 3 Lung Alive 2011 2010 1963

TCGA-05-4384 426 Alive T2 N2 M0 Stage IIIA 66 R-Lower Not Applicable TCGA-05-4384 9a50e7e4-831d-489f-87d2-979e987561cc 22 -24411 Not Applicable 0 426 Not Available Lung Adenocarcinoma Not Available TCGA-05-4384-D36331;TCGA-05-4384-D36332;TCGA-05-4384-D36333;TCGA-05-4384-D36334 281E8624-371D-4473-BA5E-E41E0C21D551;19860FED-A03B-407A-9081-F0CD43C3BD54;8009725B-0627-43CB-B0EB-37B12CCB3848;5B01A1B0-5946-4ECC-A816-E6DC557CF563 Not Available;Not Available;Not Available;Not Available 5;5;5;5 153;153;426;426 61;61;395;245 Cisplatin;Vinorelbine;Cisplatin;Zoledronic acid Clinical Progressive Disease;Clinical Progressive Disease;Clinical Progressive Disease;Clinical Progressive Disease 12;12;12;12 Not Available;Not Available;Not Available;Not Available Not Available;Not Available;Not Available;Not Available Not Available;Not Available;Not Available;Not Available Not Available;Not Available;Not Available;Not Available Not Applicable;Not Applicable;Not Applicable;Not Applicable Not Available;Not Available;Not Available;Not Available Not Available;Not Available;Not Available;Not Available NO;NO;NO;NO Chemotherapy;Chemotherapy;Chemotherapy;Ancillary Not Available;Not Available;Not Available;Not Available Not Available;Not Available;Not Available;Not Available Not Available;Not Available;Not Available;Not Available NO;NO;NO;NO 2012;2012;2012;2012 Not Available Not Available Not Available Not Available Not Available Not Available Not Available Not Available Not Available Unknown YES Not Available Not Available TCGA-05-4384-F36330 F4555A19-ABBE-4673-9871-083B5C01710A 29 Not Available Not Available Not Applicable 426 183 Not Available Scheduled Follow-up Submission Progressive Disease Not Available NO 10 Distant Metastasis YES Not Available WITH TUMOR YES Progressive Disease Not Available YES Alive 2012 MALE Lung Adenocarcinoma Mixed Subtype No C34.3 8255/3 C34.3 YES Not Available Not Available Not Available Not Available Not Available 7 Not Available Not Available Not Available Not Available Not Available Not Available Not Available Not Available Not Available Not Available 20 Yes 4384 Not Available Not Available Not Available Not Available Not Available Not Available Not Available Not Available Not Available Not Available Not Available Distant Recurrence TCGA-05-4384-R36335 E131951D-3ADE-4C06-9406-088B151B75A3 Not Available 29 214 183 Radiographic Progressive Disease 10 Not Available Not Available NO External Not Applicable Not Available Not Available Gy 2012 RX Not Applicable Not Applicable Not Applicable Not Applicable Not Applicable Not Applicable Not Applicable Not Applicable Not Applicable Stage IIIA Not Applicable Not Applicable Not Applicable 6th Not Applicable Not Applicable Not Applicable M0 N2 T2 1987 NO YES 05 3 Lung Alive 2010 2009 1963

TCGA-83-5908 824 Alive T1 N0 M0 Stage IA 59 L-Upper Not Applicable TCGA-83-5908 25d4ea9e-f773-4f11-bac9-64efdad73211 25 -21760 Not Applicable 0 420 Not Available Lung Adenocarcinoma Not Available 1 Not Available NO Not Available Not Available Not Available NO Not Available NOT HISPANIC OR LATINO NO NO NO NO TCGA-83-5908-F12196 144eec6d-ffea-424d-be87-9284d029b02c 25 Not Applicable Not Applicable Not Applicable 824 Not Applicable 1 Not Available Stable Disease 90 Not Available 5 Not Available NO Other TUMOR FREE NO Stable Disease Not Available NO Alive 2011 FEMALE Lung Adenocarcinoma- Not Otherwise Specified (NOS) No C34.1 8140/3 C34.1 YES 90 NO Not Available Not Available Not Available 5 Not Available Not Available Not Available Not Available Not Available Not Available Not Available Not Available Not Available Not Available 69 No 5908 Preoperative TUMOR FREE Not Available Not Available Not Available Not Available Not Available Not Available Not Available WHITE Not Available R0 Not Applicable Not Applicable Not Applicable Not Applicable Not Applicable Not Applicable Not Applicable Not Applicable Not Applicable Stage IA Not Applicable Not Applicable Not Applicable 6th Not Applicable Not Applicable Not Applicable M0 N0 T1 2007 YES NO 83 4 Lung Alive 2011 2009 1962

TCGA-44-6778 1864 Alive T1 N0 MX Stage IA 59 Discrepancy Not Available TCGA-44-6778 5d5cc436-6e57-4d23-a164-7f5153c2e666 31 -21725 Not Applicable 0 1110 Not Available Lung Adenocarcinoma 53 Not Available Not Available Not Available Not Available Not Available Not Available Not Available Not Available Not Available Not Available;Not Available Not Available;Not Available Not Available;Not Available Not Available;Not Available TCGA-44-6778-F15765;TCGA-44-6778-F39376 cdcf76b6-e93b-4d22-8d67-6a137b04617f;E7E66030-97D0-480D-853E-39F85ED1F874 31;21 Not Applicable;Not Applicable Not Applicable;Not Applicable Not Applicable;Not Applicable 1110;1864 Not Applicable;Not Applicable Not Available;Not Available Scheduled Follow-up Submission;Scheduled Follow-up Submission Complete Remission/Response;Complete Remission/Response Not Available;Not Available Not Available;NO 8;1 Not Available;Not Available NO;NO Not Available;Not Available TUMOR FREE;TUMOR FREE NO;NO Complete Remission/Response;Complete Remission/Response Not Available;Not Available NO;NO Alive;Alive 2011;2013 MALE Lung Adenocarcinoma- Not Otherwise Specified (NOS) No C34.1 8140/3 C34.9 YES Not Available Not Available Not Available Not Available Not Available 8 Not Available Not Available Not Available Not Available Not Available Not Available Not Available Not Available Not Available Not Available 15 No 6778 Not Available TUMOR FREE Not Available Not Available Not Available 90 74 Not Available YES BLACK OR AFRICAN AMERICAN Not Available Not Available Not Applicable Not Applicable Not Applicable Not Applicable Not Applicable Not Applicable Not Applicable Not Applicable Not Applicable Stage IA Not Applicable Not Applicable Not Applicable 6th Not Applicable Not Applicable Not Applicable MX N0 T1 2004 NO YES 44 4 Lung Alive 2011 2007 1974

TCGA-86-7955 1072 Alive T2a N0 M0 Stage IB 62 R-Lower Not Applicable TCGA-86-7955 0d0d83d9-d558-4d38-977b-6f1b2471beda 14 -22772 Not Applicable 0 14 Not Available Lung Adenocarcinoma Not Available TCGA-86-7955-D41098;TCGA-86-7955-D41099;TCGA-86-7955-D63518;TCGA-86-7955-D63519 7C0A1FDD-293F-4DF9-AC3D-67AB0630FF20;6E123B02-3E4D-4660-9400-A5BDA061292D;B33DF174-A60F-4916-97D6-315FF1B3CBF3;4F1FAE32-57AF-472B-8BCD-8E1EAB496CDC Not Available;Not Available;Not Available;Not Available 12;12;12;12 274;274;922;922 27;27;823;823 Vepesid;Cisplatin;Gemcitabine;Cisplatin Complete Response;Complete Response;Complete Response;Complete Response 3;3;8;8 Not Available;Not Available;Not Available;Not Available Not Available;Not Available;Not Available;Not Available Not Available;Not Available;Not Available;Not Available Not Available;Not Available;Not Available;Not Available Not Applicable;Not Applicable;Not Applicable;Not Applicable Not Available;Not Available;Not Available;Not Available Not Available;Not Available;Not Available;Not Available NO;NO;NO;NO Chemotherapy;Chemotherapy;Chemotherapy;Chemotherapy Not Available;Not Available;Not Available;Not Available Not Available;Not Available;Not Available;Not Available Not Available;Not Available;Not Available;Not Available NO;NO;NO;NO 2013;2013;2014;2014 1 Not Available NO Not Available Not Available Not Available Not Available Not Available NOT HISPANIC OR LATINO Not Available;YES Not Available;YES Not Available;NO Not Available;Not Available TCGA-86-7955-F41096;TCGA-86-7955-F63517 BE3BF32A-D5A9-43DA-BE40-6F8B888E0949;F2DA8AAE-377B-4F5E-8640-51294564B4D4 12;12 Not Applicable;Not Available Not Applicable;Not Available Not Applicable;Not Applicable 508;1072 Not Applicable;820 1;0 Scheduled Follow-up Submission;Scheduled Follow-up Submission Complete Remission/Response;Complete Remission/Response 90;100 NO;NO 3;8 Not Available;Locoregional Recurrence NO;YES Preoperative;Post-Adjuvant Therapy TUMOR FREE;TUMOR FREE YES;YES Complete Remission/Response;Complete Remission/Response Not Available;Not Available YES;YES Alive;Alive 2013;2014 MALE Lung Adenocarcinoma- Not Otherwise Specified (NOS) No C34.3 8140/3 C34.3 YES 90 NO Not Available Not Available Peripheral Lung 3 Not Available Not Available Not Available Not Available Not Available Not Available Not Available Not Available Not Available Not Available Not Available No 7955 Not Available TUMOR FREE Not Available Not Available Not Available Not Available Not Available Not Available NO WHITE Not Available Primary Tumor Field;Regional site TCGA-86-7955-R41101;TCGA-86-7955-R63520 AA293A6B-ED04-439A-8F4C-E01F9D5B679E;29EF3999-EA2A-4E8E-A0AF-09FF97FDCE95 Not Available;Not Available 12;12 190;1057 114;1027 Complete Response;Complete Response 3;8 31;18 62;36 NO;NO External;External Not Applicable;Not Applicable Not Available;Not Available Not Available;Not Available Gy;Gy 2013;2014 RX Not Applicable Not Applicable Not Applicable Not Applicable Not Applicable Not Applicable Not Applicable Not Applicable Not Applicable Stage IB Not Applicable Not Applicable Not Applicable 7th Not Applicable Not Applicable Not Applicable M0 N0 T2a Not Available YES NO 86 1 Lung Alive 2012 2011 Not Available

TCGA-97-A4M1 601 Alive T1a N0 M0 Stage IA 52 R-Upper Not Applicable TCGA-97-A4M1 C0C879F7-C332-4D31-9F7E-A3BF361B36EE 7 -19272 Not Applicable 0 209 Not Available Lung Adenocarcinoma 111 0 Not Available NO Not Available Not Available Not Available NO Not Available NOT HISPANIC OR LATINO Not Available Not Available Not Available Not Available TCGA-97-A4M1-F57386 4503E4C1-A9BE-4125-9311-A8140E6486AF 10 Not Applicable Not Applicable Not Applicable 601 Not Applicable 0 Scheduled Follow-up Submission Complete Remission/Response Unknown NO 3 Not Available NO Other TUMOR FREE NO Complete Remission/Response Not Available NO Alive 2014 FEMALE Lung Adenocarcinoma Mixed Subtype No C34.1 8255/3 C34.1 YES Not Evaluated NO Not Available Not Available Unknown 3 Not Available Not Available Not Available Not Available Not Available Not Available Not Available Not Available Not Available NO 3 No A4M1 Preoperative Unknown 92 105 NO 87 100 Complete Remission/Response YES WHITE NO Not Evaluated Not Applicable Not Applicable Not Applicable Not Applicable Not Applicable Not Applicable Not Applicable Not Applicable Not Applicable Stage IA Not Applicable Not Applicable Not Applicable 7th Not Applicable Not Applicable Not Applicable M0 N0 T1a 1975 YES NO 97 3 Lung Alive 2013 2012 1972

TCGA-55-A48X 689 Alive T1b N1 M0 Stage IIA 63 L-Lower Not Applicable TCGA-55-A48X 32D9935A-F5CD-43B3-88BD-09E6E2B9090D 21 -23341 Not Applicable 0 33 Not Available Lung Adenocarcinoma 79 TCGA-55-A48X-D61502;TCGA-55-A48X-D61503 D62B9942-18BB-46BB-9CD2-27E149A14B9D;119EF573-924B-4764-935E-8156AEC3B7B8 Not Available;Not Available 30;30 160;150 66;66 Carboplatin;Alimta Complete Response;Complete Response 6;6 Not Available;Not Available Not Available;Not Available Not Available;Not Available Not Available;Not Available Not Applicable;Not Applicable Not Available;Not Available Not Available;Not Available NO;NO Chemotherapy;Chemotherapy Not Available;Not Available Not Available;Not Available Not Available;Not Available NO;NO 2014;2014 Not Evaluated Not Available Unknown Not Available Not Available Not Available Unknown Not Available NOT HISPANIC OR LATINO YES NO YES Not Available TCGA-55-A48X-F61500 9BD03D83-FD93-44FB-AFAF-230675A93AAA 30 615 Not Available Not Applicable 689 615 Unknown Scheduled Follow-up Submission Complete Remission/Response Unknown NO 6 New Primary Tumor YES Not Available TUMOR FREE YES Complete Remission/Response Biopsy with Histologic Confirmation NO Alive 2014 FEMALE Lung Adenocarcinoma- Not Otherwise Specified (NOS) No C34.3 8140/3 C34.3 YES Not Evaluated Unknown Not Available Not Available Unknown 2 Not Available Not Available Not Available Not Available Not Available Not Available Not Available Not Available Not Available NO 36 Yes, History of Prior Malignancy A48X Not Available TUMOR FREE Not Available Not Available Unknown Not Available 57 Unknown YES WHITE Unknown R0 Not Applicable Not Applicable Not Applicable Not Applicable Not Applicable Not Applicable Not Applicable Not Applicable Not Applicable Stage IIA Not Applicable Not Applicable Not Applicable 7th Not Applicable Not Applicable Not Applicable M0 N1 T1b 1999 YES NO 55 4 Lung Alive 2013 2012 1963

TCGA-50-6594 370 Dead T3 N2 M0 Stage IIIA 79 L-Upper Not Applicable TCGA-50-6594 8504fd86-a70a-4cba-9ec8-25c9e60ca549 25 -28924 370 0 Not Available Not Available Lung Adenocarcinoma Not Available Not Available Not Available Not Available Not Available Not Available Not Available YES Not Available NOT HISPANIC OR LATINO NO YES Not Available NO TCGA-50-6594-F44032 08A5B385-FE6B-4B2C-8BAF-CB57CE32AA51 13 Not Available Not Available 370 Not Available 285 Not Available Scheduled Follow-up Submission Progressive Disease Not Available NO 6 Distant Metastasis YES Not Available WITH TUMOR NO Progressive Disease Convincing Imaging NO Dead 2013 FEMALE Lung Adenocarcinoma- Not Otherwise Specified (NOS) No C34.1 8140/3 C34.1 YES Not Available Not Available NO Not Available Not Available 8 Not Available Not Available Not Available Not Available Not Available Not Available Not Available Not Available Not Available Not Available Not Available No 6594 Not Available WITH TUMOR Not Available Not Available Not Available Not Available Not Available Not Available Not Available BLACK OR AFRICAN AMERICAN Not Available Distant Recurrence TCGA-50-6594-R44126 FCD116A9-2F06-48CA-ACE7-2D26AD148F79 Not Available 13 318 317 Radiographic Progressive Disease 6 1 24 NO External Not Applicable Not Available Not Available Gy 2013 Not Available Not Applicable Not Applicable Not Applicable Not Applicable Not Applicable Not Applicable Not Applicable Not Applicable Not Applicable Stage IIIA Not Applicable Not Applicable Not Applicable 6th Not Applicable Not Applicable Not Applicable M0 N2 T3 1998 NO YES 50 4 Lung Dead 2011 2009 Not Available

TCGA-73-A9RS 340 Dead T3 N0 M0 Stage IIB 41 R-Upper Not Applicable TCGA-73-A9RS 2A394996-809F-459F-A647-D209921CCBD2 17 -15162 Not Applicable 0 305 Not Available Lung Adenocarcinoma Not Available TCGA-73-A9RS-D60824;TCGA-73-A9RS-D60826;TCGA-73-A9RS-D60827;TCGA-73-A9RS-D60828;TCGA-73-A9RS-D60829;TCGA-73-A9RS-D60830 F9D3AD0B-B57B-4BC4-BE99-581990D65A30;0462262C-D185-4D68-B111-FE7D35239FFC;F252506A-D42C-4B51-8A33-83867E2282B5;91902CAB-804C-475D-855B-44440709C0BE;F29AAA5F-2DEF-42E3-AAB4-39BC2259681E;BC782E72-1B7C-451F-A7DD-FF17F0EA035A Not Available;Not Available;Not Available;Not Available;Not Available;Not Available 17;17;17;17;17;17 186;186;186;242;284;Not Available 130;130;130;200;242;284 carboplatin;paclitaxel;bevacizumab;pemetrexed;docetaxel;gemcitabine Clinical Progressive Disease;Clinical Progressive Disease;Clinical Progressive Disease;Clinical Progressive Disease;Clinical Progressive Disease;Not Applicable 6;6;6;6;6;6 Not Available;Not Available;Not Available;Not Available;Not Available;Not Available Not Available;Not Available;Not Available;Not Available;Not Available;Not Available Not Available;Not Available;Not Available;Not Available;Not Available;Not Available Not Available;Not Available;Not Available;Not Available;Not Available;Not Available Not Applicable;Not Applicable;Not Applicable;Not Applicable;Not Applicable;Not Applicable Not Available;Not Available;Not Available;Not Available;Not Available;Not Available Not Available;Not Available;Not Available;Not Available;Not Available;Not Available NO;NO;NO;NO;NO;YES Chemotherapy;Chemotherapy;Immunotherapy;Chemotherapy;Chemotherapy;Chemotherapy Not Available;Not Available;Not Available;Not Available;Not Available;Not Available Not Available;Not Available;Not Available;Not Available;Not Available;Not Available Not Available;Not Available;Not Available;Not Available;Not Available;Not Available NO;NO;YES;NO;NO;NO 2014;2014;2014;2014;2014;2014 1 Not Available YES Not Available Not Available FISH YES Not Available NOT HISPANIC OR LATINO Not Available Not Available Not Available Not Available TCGA-73-A9RS-F64487 3D9025D6-1190-49DC-986A-FC52C19BBA9B 3 Not Applicable Not Applicable 340 Not Available Not Applicable 1 Scheduled Follow-up Submission Progressive Disease 80 NO 9 Not Available NO Preoperative WITH TUMOR NO Progressive Disease Not Available YES Dead 2014 MALE Lung Adenocarcinoma- Not Otherwise Specified (NOS) No C34.1 8480/3 C34.1 YES 80 YES NO Not Available Peripheral Lung 6 YES NO 107 NO Not Available Not Available Not Available Locoregional Recurrence Convincing Imaging YES 11 No A9RS Preoperative WITH TUMOR 81.9 60.2 NO 81.5 59.6 Progressive Disease YES BLACK OR AFRICAN AMERICAN YES Primary Tumor Field TCGA-73-A9RS-R60823 F991F8D3-049C-4842-AAFE-A155907BCBBB Not Available 17 53 53 Radiographic Progressive Disease 6 1 12 NO Internal Not Applicable Not Available Not Available Gy 2014 R0 Not Applicable Not Applicable Not Applicable Not Applicable Not Applicable Not Applicable Not Applicable Not Applicable Not Applicable Stage IIB Not Applicable Not Applicable Not Applicable 7th Not Applicable Not Applicable Not Applicable M0 N0 T3 Not Available NO YES 73 2 Lung Alive 2014 2013 1990

TCGA-62-8397 1289 Alive T3 N0 M0 Stage IIB 70 R-Lower Not Applicable TCGA-62-8397 9240d5fc-de23-4436-8099-da9bd3054860 4 -25728 Not Applicable 0 940 Not Available Lung Adenocarcinoma Not Available 0 Not Available NO Not Available Not Available Not Available NO Not Available NOT HISPANIC OR LATINO Not Available Not Available Not Available Not Available TCGA-62-8397-F42100 FF6E4644-94D8-4449-857F-11AF95093868 15 Not Applicable Not Applicable Not Applicable 1289 Not Applicable 0 Scheduled Follow-up Submission Complete Remission/Response 90 NO 5 Not Available NO Preoperative TUMOR FREE NO Complete Remission/Response Not Available NO Alive 2013 FEMALE Lung Adenocarcinoma Mixed Subtype No C34.3 8255/3 C34.3 YES 90 NO Not Available Not Available Peripheral Lung 7 Not Available Not Available Not Available Not Available Not Available Not Available Not Available Not Available Not Available NO Not Available No 8397 Preoperative TUMOR FREE Not Available Not Available NO 64 86 Complete Remission/Response YES WHITE NO R0 Not Applicable Not Applicable Not Applicable Not Applicable Not Applicable Not Applicable Not Applicable Not Applicable Not Applicable Stage IIB Not Applicable Not Applicable Not Applicable 6th Not Applicable Not Applicable Not Applicable M0 N0 T3 Not Available NO YES 62 1 Lung Alive 2012 2008 Not Available

TCGA-50-6590 1288 Dead T2 N0 M0 Stage IB 72 R-Upper Not Applicable TCGA-50-6590 114a34c0-983b-4d90-9181-1823c9637a63 25 -26444 1288 0 Not Available Not Available Lung Adenocarcinoma Not Available TCGA-50-6590-D43830;TCGA-50-6590-D43831 A075B030-D2CD-4488-9FD9-D0BFD2A087A7;A3CB9C93-4673-49EE-AB53-9823D496F039 Not Available;Not Available 4;4 189;189 92;92 Paclitaxel;Carboplatin Complete Response;Complete Response 6;6 Not Available;Not Available Not Available;Not Available Not Available;Not Available Not Available;Not Available Not Applicable;Not Applicable Not Available;Not Available Not Available;Not Available NO;NO Chemotherapy;Chemotherapy Not Available;Not Available Not Available;Not Available Not Available;Not Available NO;NO 2013;2013 Not Available Not Available YES Not Available Not Available Not Available Not Available Not Available NOT HISPANIC OR LATINO Not Available Not Available Not Available Not Available TCGA-50-6590-F43827 47ED4445-67EF-44BD-AC55-2C3E733F62CF 4 Not Applicable Not Applicable 1288 Not Available Not Applicable Not Available Scheduled Follow-up Submission Complete Remission/Response Not Available NO 6 Not Available NO Not Available TUMOR FREE YES Complete Remission/Response Not Available NO Dead 2013 FEMALE Lung Adenocarcinoma- Not Otherwise Specified (NOS) No C34.1 8140/3 C34.1 YES Not Available Not Available Not Available Not Available Not Available 8 Not Available Not Available Not Available Not Available Not Available Not Available Not Available Not Available Not Available Not Available 50 No 6590 Not Available TUMOR FREE Not Available Not Available Not Available Not Available Not Available Not Available Not Available WHITE Not Available Not Available Not Applicable Not Applicable Not Applicable Not Applicable Not Applicable Not Applicable Not Applicable Not Applicable Not Applicable Stage IB Not Applicable Not Applicable Not Applicable 6th Not Applicable Not Applicable Not Applicable M0 N0 T2 2002 NO YES 50 4 Lung Dead 2011 2005 Not Available

TCGA-73-4675 922 Dead T3 N1 M0 Stage IIIA 59 L-Lower Not Applicable TCGA-73-4675 ae39e358-08d7-4367-ae68-82b469e791e4 7 -21614 Not Applicable 0 40 Not Available Lung Adenocarcinoma 69 TCGA-73-4675-D71056;TCGA-73-4675-D71057;TCGA-73-4675-D71058;TCGA-73-4675-D71059;TCGA-73-4675-D71060;TCGA-73-4675-D71061;TCGA-73-4675-D71062 13B8CCE4-8461-4A03-A567-E5922A9A11A4;3C9951FA-7567-4A17-8ED5-031F790060D7;25DC92F4-A154-4196-9D5F-4BE559E42ABA;1C265224-D738-4F25-8103-9B037F9C9447;DC1FC3CB-3680-4D2A-86C2-B9DBC184D6BC;08130C1E-5BB6-4080-939E-11D5E4215EEF;3BF6C8DC-1CB9-4686-987C-7DA51220B8A3 Not Available;Not Available;Not Available;Not Available;Not Available;Not Available;Not Available 17;17;17;17;17;17;17 482;117;104;483;483;483;676 110;27;27;459;459;459;592 Carboplatin;Alimta;Cisplatin;Carboplatin;Taxotere;Avastin;Tarceva Complete Response;Complete Response;Complete Response;Stable Disease;Stable Disease;Stable Disease;Clinical Progressive Disease 3;3;3;3;3;3;3 Not Available;Not Available;Not Available;Not Available;Not Available;Not Available;Not Available Not Available;Not Available;Not Available;Not Available;Not Available;Not Available;Not Available Not Available;Not Available;Not Available;Not Available;Not Available;Not Available;Not Available Not Available;Not Available;Not Available;Not Available;Not Available;Not Available;Not Available Not Applicable;Not Applicable;Not Applicable;Not Applicable;Not Applicable;Not Applicable;Not Applicable Not Available;Not Available;Not Available;Not Available;Not Available;Not Available;Not Available Not Available;Not Available;Not Available;Not Available;Not Available;Not Available;Not Available NO;NO;NO;NO;NO;NO;NO Chemotherapy;Chemotherapy;Chemotherapy;Chemotherapy;Chemotherapy;Chemotherapy;Chemotherapy Not Available;Not Available;Not Available;Not Available;Not Available;Not Available;Not Available Not Available;Not Available;Not Available;Not Available;Not Available;Not Available;Not Available Not Available;Not Available;Not Available;Not Available;Not Available;Not Available;Not Available NO;NO;NO;NO;NO;NO;NO 2015;2015;2015;2015;2015;2015;2015 0 Not Available NO Not Available Not Available Not Available NO Not Available NOT HISPANIC OR LATINO Not Available;YES Not Available;NO Not Available;NO Not Available;Not Available TCGA-73-4675-F8551;TCGA-73-4675-F71055 05da2e27-6b81-4a1f-887c-1a42d8bc1786;AC8B9FDB-A4DE-4944-94BF-6BB1CAF80003 7;17 Not Available;Not Available Not Available;Not Available Not Applicable;922 40;Not Available Not Available;361 0;Unknown Not Available;Scheduled Follow-up Submission Not Available;Progressive Disease Not Available;Unknown Not Available;NO 1;3 Not Available;Locoregional Recurrence Not Available;YES Pre-Adjuvant Therapy;Unknown TUMOR FREE;Unknown Not Available;YES Not Available;Progressive Disease Not Available;Convincing Imaging Not Available;NO Alive;Dead 2011;2015 MALE Lung Adenocarcinoma- Not Otherwise Specified (NOS) No C34.3 8140/3 C34.3 YES Not Available NO Not Available Not Available Peripheral Lung 1 Not Available Not Available Not Available Not Available Not Available Not Available Not Available Not Available Not Available Not Available 41 No 4675 Pre-Adjuvant Therapy TUMOR FREE Not Available Not Available Not Available 55 66 Not Available YES WHITE Not Available RX Not Applicable Not Applicable Not Applicable Not Applicable Not Applicable Not Applicable Not Applicable Not Applicable Not Applicable Stage IIIA Not Applicable Not Applicable Not Applicable 7th Not Applicable Not Applicable Not Applicable M0 N1 T3 2010 NO YES 73 4 Lung Alive 2011 2010 1969

TCGA-MP-A4TF 336 Dead T2b N0 M0 Stage IIA 58 R-Upper Not Applicable TCGA-MP-A4TF 86DAC334-F30E-438E-810A-8E45F69DBB7E 2 -21387 336 0 Not Available Not Available Lung Adenocarcinoma 77 TCGA-MP-A4TF-D41409;TCGA-MP-A4TF-D41410 DB475CAC-B2D9-461C-A714-D5BBD7B9CF48;51556339-4419-45EF-B7B5-597FECF9A3D5 Not Available;Not Available 18;18 151;133 48;48 Navelbine;Cisplatin Partial Response;Partial Response 3;3 Not Available;Not Available Not Available;Not Available Not Available;Not Available Not Available;Not Available Not Applicable;Not Applicable Not Available;Not Available Not Available;Not Available NO;NO Chemotherapy;Chemotherapy Not Available;Not Available Not Available;Not Available Not Available;Not Available NO;NO 2013;2013 Not Evaluated Not Available NO Not Available Not Available Not Available NO Not Available NOT HISPANIC OR LATINO FEMALE Lung Adenocarcinoma- Not Otherwise Specified (NOS) No C34.1 8140/3 C34.1 YES Not Evaluated NO Not Available Not Available Peripheral Lung 4 NO YES 195 Not Available Not Available YES 202 Distant Metastasis Biopsy with Histologic Confirmation;Convincing Imaging YES 40 No A4TF Not Evaluated WITH TUMOR Not Available 108 YES Not Available 109 Partial Remission/Response YES WHITE NO R0 Not Applicable Not Applicable Not Applicable Not Applicable Not Applicable Not Applicable Not Applicable Not Applicable Not Applicable Stage IIA Not Applicable Not Applicable Not Applicable 7th Not Applicable Not Applicable Not Applicable M0 N0 T2b 2010 NO YES MP 4 Lung Dead 2013 2010 1970

TCGA-55-A490 99 Dead T2b N0 MX Stage IIA 78 R-Upper Not Applicable TCGA-55-A490 B89DE053-D253-447F-952E-9A2EDCF6BCA5 21 -28728 Not Applicable 0 48 Not Available Lung Adenocarcinoma 40 TCGA-55-A490-D59079;TCGA-55-A490-D59080 A6D28342-BD3F-4814-B710-BFBA6B19D0B3;2A35CBA5-76D7-4EAC-AF33-941BD871E623 Not Available;Not Available 30;30 89;89 89;89 Carboplatin;Alimta Unknown;Unknown 4;4 Not Available;Not Available Not Available;Not Available Not Available;Not Available Not Available;Not Available Not Applicable;Not Applicable Not Available;Not Available Not Available;Not Available NO;NO Chemotherapy;Chemotherapy Not Available;Not Available Not Available;Not Available Not Available;Not Available NO;NO 2014;2014 1 Not Available YES Not Available Not Available FISH YES Not Available NOT HISPANIC OR LATINO Not Available Not Available Not Available Not Available TCGA-55-A490-F59076 813389EA-D9E4-47C0-B21A-5FA647E9EEB8 30 Not Applicable Not Applicable 99 Not Available Not Applicable Not Evaluated Scheduled Follow-up Submission Not Applicable Not Evaluated NO 4 Not Available NO Not Available TUMOR FREE YES Not Applicable Not Available NO Dead 2014 MALE Lung Adenocarcinoma- Not Otherwise Specified (NOS) No C34.1 8140/3 C34.1 YES 80 YES YES G12C Peripheral Lung 2 Not Available Not Available Not Available Not Available Not Available Not Available Not Available Not Available Not Available NO 34 No A490 Preoperative TUMOR FREE Not Available Not Available Unknown 60 56 Unknown YES WHITE Unknown R0 Not Applicable Not Applicable Not Applicable Not Applicable Not Applicable Not Applicable Not Applicable Not Applicable Not Applicable Stage IIA Not Applicable Not Applicable Not Applicable 7th Not Applicable Not Applicable Not Applicable MX N0 T2b 1985 YES NO 55 3 Lung Alive 2013 2012 1951

TCGA-44-8120 260 Alive T2a N0 M0 Stage IB 58 R-Upper Not Applicable TCGA-44-8120 83e38dbd-edab-47f2-b19f-6ea38fc6bece 31 -21188 Not Applicable 0 169 Not Available Lung Adenocarcinoma Not Available 0 Not Available Unknown Not Available Not Available Not Available Unknown Not Available NOT HISPANIC OR LATINO Not Available Not Available Not Available Not Available TCGA-44-8120-F36757 9CE462FD-8E7F-483D-BB93-B5B2CDFCA189 13 Not Applicable Not Applicable Not Applicable 260 Not Applicable 0 Scheduled Follow-up Submission Complete Remission/Response Not Available NO 11 Not Available NO Preoperative TUMOR FREE NO Complete Remission/Response Not Available NO Alive 2012 MALE Lung Adenocarcinoma- Not Otherwise Specified (NOS) No C34.1 8140/3 C34.1 YES Not Available Unknown Not Available Not Available Not Available 5 Not Available Not Available Not Available Not Available Not Available Not Available Not Available Not Available Not Available NO 30 No 8120 Preoperative TUMOR FREE 100 101 NO 98 97 Complete Remission/Response YES BLACK OR AFRICAN AMERICAN NO Not Available Not Applicable Not Applicable Not Applicable Not Applicable Not Applicable Not Applicable Not Applicable Not Applicable Not Applicable Stage IB Not Applicable Not Applicable Not Applicable 7th Not Applicable Not Applicable Not Applicable M0 N0 T2a Not Available YES NO 44 5 Lung Alive 2012 2011 1981

TCGA-44-2666 97 Dead T2 N0 M0 Stage IB FPPP TCGA 43 R-Upper Not Applicable TCGA-44-2666 e16ca88f-488b-40f0-9169-e5a62482a2ff 8 -15970 97 0 97 97 Lung Adenocarcinoma 66 Not Available Not Available Not Available Not Available Not Available Not Available Not Available Not Available HISPANIC OR LATINO NO NO NO NO TCGA-44-2666-F5304 3f3411e8-5a55-4b73-b4e9-42c1e7bfdf7d 12 Not Applicable Not Applicable 97 97 Not Applicable Not Available Not Available Not Available Not Available Not Available 10 Not Available NO Not Available WITH TUMOR NO Not Available Not Available NO Dead 2010 MALE Lung Adenocarcinoma- Not Otherwise Specified (NOS) No C34.1 8140/3 C34.1 YES Not Available NO Not Available Not Available Not Available 10 Not Available Not Available Not Available Not Available Not Available Not Available Not Available Not Available Not Available Not Available 30 No 2666 Not Available WITH TUMOR Not Available Not Available Not Available 86 87 Not Available YES WHITE Not Available R0 Not Applicable Not Applicable Not Applicable Not Applicable Not Applicable Not Applicable Not Applicable Not Applicable Not Applicable Stage IB Not Applicable Not Applicable Not Applicable 6th Not Applicable Not Applicable Not Applicable M0 N0 T2 Not Available YES NO 44 2 Lung Dead 2010 2009 1979

TCGA-05-5423 151 Alive T2 N1 M0 Stage IIB 65 L-Upper Not Applicable TCGA-05-5423 33e3f736-7990-4f7a-ac35-863247566541 22 -23863 Not Applicable 0 151 Not Available Lung Adenocarcinoma Not Available Not Available Not Available Not Available Not Available Not Available Not Available Not Available Not Available Not Available MALE Lung Adenocarcinoma Mixed Subtype No C34.9 8255/3 C34.9 YES Not Available Not Available Not Available Not Available Not Available 3 Not Available Not Available Not Available Not Available Not Available Not Available Not Available Not Available Not Available Not Available 72 No 5423 Not Available TUMOR FREE Not Available Not Available Not Available Not Available Not Available Not Available Not Available Not Available Not Available R0 Not Applicable Not Applicable Not Applicable Not Applicable Not Applicable Not Applicable Not Applicable Not Applicable Not Applicable Stage IIB Not Applicable Not Applicable Not Applicable 6th Not Applicable Not Applicable Not Applicable M0 N1 T2 1980 NO YES 05 3 Lung Alive 2011 2006 1956

TCGA-50-8460 829 Alive T1a N0 M0 Stage IA 74 R-Upper Not Applicable TCGA-50-8460 497cc506-03fe-4ffa-860b-5a12adb1bc7d 18 -27270 Not Applicable 0 105 Not Available Lung Adenocarcinoma Not Available Not Available Not Available YES Not Available Not Available Not Available Not Available Not Available NOT HISPANIC OR LATINO Not Available Not Available Not Available Not Available TCGA-50-8460-F69335 D2F418F0-13A0-45DB-B9B4-F1D07DFA9D55 5 Not Applicable Not Applicable Not Applicable 829 Not Applicable Not Available Scheduled Follow-up Submission Complete Remission/Response Not Available NO 1 Not Available NO Not Available TUMOR FREE NO Complete Remission/Response Not Available YES Alive 2015 MALE Lung Papillary Adenocarcinoma No C34.1 8260/3 C34.1 YES Not Available YES NO Not Available Not Available 10 Not Available Not Available Not Available Not Available Not Available Not Available Not Available Not Available Not Available NO Not Available No 8460 Not Available Unknown Not Available Not Available NO Not Available Not Available Unknown NO WHITE YES Primary Tumor Field TCGA-50-8460-R42675 1EB8F1A2-25A0-4301-B2A6-731A17BE04FB Not Available 26 49 49 Complete Response 4 Not Available Not Available NO Internal Not Applicable Not Available Not Available Not Available 2013 R0 Not Applicable Not Applicable Not Applicable Not Applicable Not Applicable Not Applicable Not Applicable Not Applicable Not Applicable Stage IA Not Applicable Not Applicable Not Applicable 7th Not Applicable Not Applicable Not Applicable M0 N0 T1a Not Available YES NO 50 Unknown Lung Alive 2012 2012 Not Available

TCGA-86-7954 605 Alive T2 N0 M0 Stage IB 68 R-Lower Not Applicable TCGA-86-7954 079ae0b3-b64b-4b8e-ab7d-225b8046568c 30 -25062 Not Applicable 0 0 Not Available Lung Adenocarcinoma Not Available TCGA-86-7954-D57949;TCGA-86-7954-D57950 D1519FCC-3805-4E15-B46C-49EA3BCEEC7D;3D5B9CB0-F3CF-411C-8AFB-E3271BA29B8C Not Available;Not Available 28;28 81;81 55;55 Carboplatin;Taxol Complete Response;Complete Response 3;3 Not Available;Not Available Not Available;Not Available Not Available;Not Available Not Available;Not Available Not Applicable;Not Applicable Not Available;Not Available Not Available;Not Available NO;NO Chemotherapy;Chemotherapy Not Available;Not Available Not Available;Not Available Not Available;Not Available NO;NO 2014;2014 0 Not Available NO Not Available Not Available Not Available Not Available Not Available NOT HISPANIC OR LATINO Not Available Not Available Not Available Not Available TCGA-86-7954-F57948 F5479210-0898-47AC-989D-AD4BEE57AA48 28 Not Applicable Not Applicable Not Applicable 605 Not Applicable 0 Scheduled Follow-up Submission Complete Remission/Response 100 NO 3 Not Available NO Post-Adjuvant Therapy TUMOR FREE YES Complete Remission/Response Not Available NO Alive 2014 FEMALE Lung Bronchioloalveolar Carcinoma Nonmucinous No C34.3 8250/3 C34.3 YES 100 NO Not Available Not Available Peripheral Lung 3 Not Available Not Available Not Available Not Available Not Available Not Available Not Available Not Available Not Available Not Available 8 No 7954 Preoperative TUMOR FREE Not Available Not Available Not Available Not Available Not Available Not Available NO WHITE Not Available R0 Not Applicable Not Applicable Not Applicable Not Applicable Not Applicable Not Applicable Not Applicable Not Applicable Not Applicable Stage IB Not Applicable Not Applicable Not Applicable 7th Not Applicable Not Applicable Not Applicable M0 N0 T2 1971 YES NO 86 3 Lung Alive 2012 2011 1963

TCGA-55-8208 674 Alive T1b N0 M0 Stage IA 73 R-Upper Not Applicable TCGA-55-8208 77f8c850-b4e6-46a1-ab12-a50e0044a0c8 13 -26716 Not Applicable 0 0 Not Available Lung Adenocarcinoma 50 0 Not Available Not Available Not Available Not Available Not Available Not Available Not Available NOT HISPANIC OR LATINO Unknown YES Not Available YES TCGA-55-8208-F59045 6F292E9C-8A64-4D70-B169-8AE2D8E32EB1 30 Not Available 536 Not Applicable 674 511 Unknown Scheduled Follow-up Submission Stable Disease Unknown YES 4 Distant Metastasis YES Not Available WITH TUMOR NO Complete Remission/Response Biopsy with Histologic Confirmation;Convincing Imaging NO Alive 2014 FEMALE Lung Adenocarcinoma- Not Otherwise Specified (NOS) No C34.1 8140/3 C34.1 YES 100 Not Available Not Available Not Available Peripheral Lung 6 Not Available Not Available Not Available Not Available Not Available Not Available Not Available Not Available Not Available NO 50 No 8208 Preoperative TUMOR FREE 68 151 Unknown 72 156 Complete Remission/Response YES WHITE Unknown R0 Not Applicable Not Applicable Not Applicable Not Applicable Not Applicable Not Applicable Not Applicable Not Applicable Not Applicable Stage IA Not Applicable Not Applicable Not Applicable 7th Not Applicable Not Applicable Not Applicable M0 N0 T1b Not Available YES NO 55 2 Lung Alive 2012 2012 Not Available

TCGA-67-3774 385 Alive T2 N0 M0 Stage IB 73 L-Lower Not Applicable TCGA-67-3774 36ad7fc7-0cc1-4f01-aef1-f89b3cb4feac 3 -26704 Not Applicable 0 385 Not Available Lung Adenocarcinoma Not Available Not Available Not Available NO Not Available Not Available Not Available NO Not Available NOT HISPANIC OR LATINO FEMALE Lung Adenocarcinoma- Not Otherwise Specified (NOS) No C34.3 8140/3 C34.3 YES Not Available NO Not Available Not Available Not Available 3 Not Available Not Available Not Available Not Available Not Available Not Available Not Available Not Available Not Available Not Available Not Available No 3774 Not Available TUMOR FREE Not Available Not Available Not Available Not Available Not Available Not Available Not Available WHITE Not Available RX Not Applicable Not Applicable Not Applicable Not Applicable Not Applicable Not Applicable Not Applicable Not Applicable Not Applicable Stage IB Not Applicable Not Applicable Not Applicable 6th Not Applicable Not Applicable Not Applicable M0 N0 T2 Not Available YES NO 67 3 Lung Alive 2011 2009 Not Available

TCGA-78-7539 791 Alive T2b N0 M0 Stage IIA 75 R-Upper Not Applicable TCGA-78-7539 7be86a90-6da8-4c01-8a7a-bc5cfef9894f 19 -27445 Not Applicable 0 327 Not Available Lung Adenocarcinoma Not Available TCGA-78-7539-D20644;TCGA-78-7539-D20647;TCGA-78-7539-D20645;TCGA-78-7539-D20646 ee4766b1-b93f-47b2-8296-193336607673;ef32890a-c5fc-4abf-915c-3c0394c365a4;55910d2b-0159-402a-a7c3-dc40c6dd4fcd;0a178b02-54e3-4690-8a6b-a18c85384aa2 Not Available;Not Available;Not Available;Not Available 19;19;19;19 42;133;49;133 36;70;36;70 Cisplatin;Paclitaxel;Vinorelbine;Carboplatin Not Available;Not Available;Not Available;Not Available 1;1;1;1 1;4;1;4 75;262;37.5;284 mg;mg;mg;mg ADJUVANT;ADJUVANT;ADJUVANT;ADJUVANT Not Applicable;Not Applicable;Not Applicable;Not Applicable 1;2;1;2 IV;IV;IV;IV NO;NO;NO;NO Chemotherapy;Chemotherapy;Chemotherapy;Chemotherapy Not Available;Not Available;Not Available;Not Available 150;1048;112.5;1167 mg;mg;mg;mg Not Available;Not Available;Not Available;Not Available 2012;2012;2012;2012 1 Not Available NO Not Available Not Available Not Available NO Not Available Not Available Not Available;Unknown Not Available;YES Not Available;Not Available Not Available;YES TCGA-78-7539-F20643;TCGA-78-7539-F46143 e53bcc72-3116-4b4d-a4d9-712dab94cf72;73006682-67D7-4FC9-9CFE-3C60E6B3B825 19;4 Not Available;Not Available Not Available;688 Not Applicable;Not Applicable 327;791 Not Available;688 Not Available;Unknown Scheduled Follow-up Submission;Scheduled Follow-up Submission Not Available;Unknown Not Available;Not Available Not Available;NO 1;8 Not Available;Distant Metastasis Not Available;YES Not Available;Not Available Not Available;Unknown YES;YES Not Available;Complete Remission/Response Not Available;Biopsy with Histologic Confirmation NO;NO Alive;Alive 2012;2013 FEMALE Lung Adenocarcinoma Mixed Subtype No C34.1 8255/3 C34.1 YES Not Available NO Not Available Not Available Not Available 1 Not Available Not Available Not Available Not Available Not Available Not Available Not Available Not Available Not Available Not Available 2.2 No 7539 Preoperative Not Available Not Available Not Available Not Available Not Available Not Available Not Available NO WHITE Not Available R0 Not Applicable Not Applicable Not Applicable Not Applicable Not Applicable Not Applicable Not Applicable Not Applicable Not Applicable Stage IIA Not Applicable Not Applicable Not Applicable 7th Not Applicable Not Applicable Not Applicable M0 N0 T2b 1981 NO YES 78 3 Lung Alive 2012 2011 1953

TCGA-44-A4SU 409 Dead T1a N0 MX Stage IA 67 R-Lower Not Applicable TCGA-44-A4SU BF34664D-423D-4257-99D4-797F7D366455 29 -24820 Not Applicable 0 76 Not Available Lung Adenocarcinoma 74 1 Not Available Not Available Not Available Not Available Not Available Not Available Not Available NOT HISPANIC OR LATINO NO NO NO Not Available TCGA-44-A4SU-F49438 D0EC82D0-E3BC-4820-AD56-81831A4CF3EC 3 Not Available Not Available 409 Not Available 260 3 Scheduled Follow-up Submission Progressive Disease Not Evaluated NO 10 Locoregional Recurrence YES Other WITH TUMOR NO Complete Remission/Response Biopsy with Histologic Confirmation;Convincing Imaging NO Dead 2013 FEMALE Lung Adenocarcinoma- Not Otherwise Specified (NOS) No C34.3 8140/3 C34.3 YES Not Available Not Available Not Available Not Available Not Available 1 Not Available Not Available Not Available Not Available Not Available Not Available Not Available Not Available Not Available NO 50 No A4SU Preoperative TUMOR FREE 100 95 NO 100 88 Complete Remission/Response YES WHITE NO R0 Not Applicable Not Applicable Not Applicable Not Applicable Not Applicable Not Applicable Not Applicable Not Applicable Not Applicable Stage IA Not Applicable Not Applicable Not Applicable 7th Not Applicable Not Applicable Not Applicable MX N0 T1a 2011 YES NO 44 4 Lung Alive 2013 2012 1961

TCGA-55-6980 2109 Alive T1 N0 M0 Stage IA 56 R-Lower Not Applicable TCGA-55-6980 2f0710f4-827b-45b2-9b7e-c27385b481a7 27 Not Available Not Applicable 0 67 Not Available Lung Adenocarcinoma Not Available Not Available Not Available NO Not Available Not Available Not Available NO Not Available Not Available Not Available Not Available Not Available Not Available TCGA-55-6980-F46642 280D1471-7D87-47DB-AAD2-6281B990E361 13 Not Applicable Not Applicable Not Applicable 2109 Not Applicable Not Evaluated Scheduled Follow-up Submission Complete Remission/Response Not Evaluated NO 8 Not Available NO Not Evaluated TUMOR FREE NO Complete Remission/Response Not Available NO Alive 2013 MALE Lung Bronchioloalveolar Carcinoma Nonmucinous No C34.3 8252/3 C34.3 YES Not Available NO Not Available Not Available Not Available 7 Not Available Not Available Not Available Not Available Not Available Not Available Not Available Not Available Not Available Not Available Not Available No 6980 Not Available TUMOR FREE Not Available Not Available Not Available Not Available Not Available Not Available Not Available WHITE Not Available R0 Not Applicable Not Applicable Not Applicable Not Applicable Not Applicable Not Applicable Not Applicable Not Applicable Not Applicable Stage IA Not Applicable Not Applicable Not Applicable 6th Not Applicable Not Applicable Not Applicable M0 N0 T1 Not Available NO YES 55 1 Lung Alive 2011 2006 Not Available

TCGA-NJ-A55A 15 Alive T2 N0 M0 Stage IB 76 R-Lower Not Applicable TCGA-NJ-A55A B8B5ED35-B299-4C1E-9226-5CA055DC7A43 27 -28022 Not Applicable 0 3 Not Available Lung Adenocarcinoma Not Available Not Available Not Available NO Not Available Not Available Not Available NO Not Available NOT HISPANIC OR LATINO Not Available;Not Available Not Available;Not Available Not Available;Not Available Not Available;Not Available TCGA-NJ-A55A-F50896;TCGA-NJ-A55A-F70649 C6E65920-A007-4160-A4CD-196B5F996C14;24A27E33-8F11-4A44-BDB0-D081C9BBFBE0 1;27 Not Applicable;Not Applicable Not Applicable;Not Applicable Not Applicable;Not Applicable 8;15 Not Applicable;Not Applicable Not Available;Not Available Scheduled Follow-up Submission;Scheduled Follow-up Submission Stable Disease;Stable Disease Not Available;Not Available NO;YES 11;2 Not Available;Not Available NO;NO Not Available;Not Available TUMOR FREE;TUMOR FREE NO;NO Stable Disease;Stable Disease Not Available;Not Available NO;NO Alive;Alive 2013;2015 FEMALE Lung Adenocarcinoma- Not Otherwise Specified (NOS) No C34.3 8550/3 C34.3 YES Not Available NO Not Available Not Available Not Available 9 Not Available Not Available Not Available Not Available Not Available Not Available Not Available Not Available Not Available NO 25 No A55A Not Available TUMOR FREE Not Available Not Available NO Not Available Not Available Stable Disease NO WHITE NO Not Available Not Applicable Not Applicable Not Applicable Not Applicable Not Applicable Not Applicable Not Applicable Not Applicable Not Applicable Stage IB Not Applicable Not Applicable Not Applicable 6th Not Applicable Not Applicable Not Applicable M0 N0 T2 1983 Not Available Not Available NJ 3 Lung Alive 2013 2009 Not Available

TCGA-86-6562 376 Dead T2a N1 M0 Stage IIA 52 R-Upper Not Applicable TCGA-86-6562 dcd066ba-5894-453e-b1da-82092858c3db 21 -19301 Not Applicable 0 0 Not Available Lung Adenocarcinoma Not Available TCGA-86-6562-D29769;TCGA-86-6562-D29767 f359ca59-3a44-488c-bfc2-7aea795f3d57;02372dd0-f7cc-47c8-a3d0-0c6031d75cda Not Available;Not Available 28;28 253;253 27;27 Vinblastine;Cisplatin Not Available;Not Available 3;3 4;4 6;150 mg/day;mg/day ADJUVANT;ADJUVANT Not Applicable;Not Applicable Not Available;Not Available IV;IV NO;NO Chemotherapy;Chemotherapy Not Available;Not Available 120;600 mg;mg Not Available;Not Available 2012;2012 Not Available Not Available Not Available Not Available Not Available Not Available Not Available Not Available NOT HISPANIC OR LATINO NO NO NO Not Available TCGA-86-6562-F29765 a47bbc7f-a0b3-4a1f-bf61-b6fd5f425572 28 Not Available Not Available 376 Not Available 274 Not Available Scheduled Follow-up Submission Progressive Disease 0 Not Available 3 Not Available YES Not Available WITH TUMOR YES Progressive Disease Not Available NO Dead 2012 MALE Lung Adenocarcinoma- Not Otherwise Specified (NOS) No C34.1 8140/3 C34.1 YES Not Available NO Not Available Not Available Central Lung 6 Not Available Not Available Not Available Not Available Not Available Not Available Not Available Not Available Not Available Not Available Not Available No 6562 Not Available TUMOR FREE Not Available Not Available Not Available Not Available Not Available Not Available NO WHITE Not Available R0 Not Applicable Not Applicable Not Applicable Not Applicable Not Applicable Not Applicable Not Applicable Not Applicable Not Applicable Stage IIA Not Applicable Not Applicable Not Applicable 7th Not Applicable Not Applicable Not Applicable M0 N1 T2a Not Available YES NO 86 1 Lung Alive 2011 2011 Not Available

TCGA-86-7711 1046 Dead T2a N1 M0 Stage IIA 70 R-Lower Not Applicable TCGA-86-7711 9c894668-88f5-4308-a1c0-6b8acc6aaaec 2 -25719 Not Applicable 0 27 Not Available Lung Adenocarcinoma Not Available TCGA-86-7711-D36470;TCGA-86-7711-D36471 5969CF61-8D1A-4E11-8018-EA0CC6004B7B;88D02F6F-8FB9-47F9-8722-B3078EA28D7B Not Available;Not Available 31;31 181;181 37;37 Cisplatin;Etoposide Complete Response;Complete Response 10;10 Not Available;Not Available Not Available;Not Available Not Available;Not Available Not Available;Not Available Not Applicable;Not Applicable Not Available;Not Available Not Available;Not Available NO;NO Chemotherapy;Chemotherapy Not Available;Not Available Not Available;Not Available Not Available;Not Available NO;NO 2012;2012 2 Not Available Not Available Not Available Not Available Not Available Not Available Not Available HISPANIC OR LATINO Not Available;Not Available Not Available;Not Available Not Available;Not Available Not Available;Not Available TCGA-86-7711-F36464;TCGA-86-7711-F41203 A9C3D370-F629-4A00-A8EF-8DA24B519C43;1D299B3D-1130-4A71-9E9C-B523833689E9 31;13 Not Applicable;Not Applicable Not Applicable;Not Applicable Not Applicable;1046 630;Not Available Not Applicable;Not Applicable 0;Not Evaluated Scheduled Follow-up Submission;Scheduled Follow-up Submission Progressive Disease;Progressive Disease 100;Not Evaluated NO;NO 10;3 Not Available;Not Available NO;NO Post-Adjuvant Therapy;Not Evaluated WITH TUMOR;WITH TUMOR YES;YES Progressive Disease;Progressive Disease Not Available;Not Available NO;NO Alive;Dead 2012;2013 MALE Lung Adenocarcinoma- Not Otherwise Specified (NOS) No C34.3 8140/3 C34.3 YES 80 NO Not Available Not Available Peripheral Lung 5 Not Available Not Available Not Available Not Available Not Available Not Available Not Available Not Available Not Available Not Available 50 No 7711 Not Available TUMOR FREE Not Available Not Available Not Available Not Available Not Available Not Available NO WHITE Not Available R0 Not Applicable Not Applicable Not Applicable Not Applicable Not Applicable Not Applicable Not Applicable Not Applicable Not Applicable Stage IIA Not Applicable Not Applicable Not Applicable 7th Not Applicable Not Applicable Not Applicable M0 N1 T2a Not Available YES NO 86 2 Lung Alive 2011 2011 1960

TCGA-99-AA5R 658 Alive T1a N0 M0 Stage IA 70 L-Lower Not Applicable TCGA-99-AA5R D3E23815-7F9D-48AA-9AEF-3A1DD9D464D7 14 -25778 Not Applicable 0 189 Not Available Lung Adenocarcinoma 47 1 Not Available YES Not Available Not Available IHC YES Not Available NOT HISPANIC OR LATINO Not Available Not Available Not Available Not Available TCGA-99-AA5R-F69298 BF89B2AB-9B58-4E72-9EAC-7E5FA90BA49B 31 Not Applicable Not Applicable Not Applicable 658 Not Applicable Not Evaluated Scheduled Follow-up Submission Unknown Not Evaluated NO 12 Not Available NO Not Evaluated Unknown NO Complete Remission/Response Not Available NO Alive 2014 FEMALE Lung Adenocarcinoma- Not Otherwise Specified (NOS) No C34.3 8140/3 C34.3 YES Unknown YES YES G12C Unknown 5 Not Available Not Available Not Available Not Available Not Available Not Available Not Available Not Available Not Available NO 46 No AA5R Preoperative TUMOR FREE Not Available Not Available NO 65 64 Complete Remission/Response YES BLACK OR AFRICAN AMERICAN NO R0 Not Applicable Not Applicable Not Applicable Not Applicable Not Applicable Not Applicable Not Applicable Not Applicable Not Applicable Stage IA Not Applicable Not Applicable Not Applicable 7th Not Applicable Not Applicable Not Applicable M0 N0 T1a Not Available YES NO 99 2 Lung Alive 2014 2013 1967

TCGA-49-6742 488 Dead T2a N1 M0 Stage IIA 70 L-Upper Not Applicable TCGA-49-6742 21fb46f9-4bbb-441c-af19-a687e9138344 9 -25839 Not Applicable 0 Discrepancy Not Available Lung Adenocarcinoma Not Available TCGA-49-6742-D14744;TCGA-49-6742-D16680;TCGA-49-6742-D16679;TCGA-49-6742-D17830 3b0101a9-80a8-4824-a268-205c46c9ac31;f59fb2c2-a314-4463-af34-81a5299331fc;e4f03b6f-8150-4976-8b9a-1783d3aaaf2e;23b5df36-e5bf-4b57-a0db-04bc5b0c0c9c Not Available;Not Available;Not Available;Not Available 9;23;23;26 184;184;184;396 31;31;31;335 Alimta;Pemetrexed;Cisplatin;MDX-1106 clinical trial Not Available;Not Available;Not Available;Not Available 8;9;9;10 3;3;3;1 Not Available;Not Available;Not Available;Not Available Not Available;Not Available;Not Available;Not Available ADJUVANT;ADJUVANT;ADJUVANT;PROGRESSION Not Applicable;Not Applicable;Not Applicable;Not Applicable 1;1;1;2 IV;IV;IV;IV NO;NO;NO;NO Chemotherapy;Chemotherapy;Chemotherapy;Immunotherapy Not Available;Not Available;Not Available;Not Available Not Available;Not Available;Not Available;Not Available Not Available;Not Available;mg/m2;Not Available Not Available;Not Available;Not Available;Not Available 2011;2011;2011;2011 Not Available Not Available Not Available Not Available Not Available Not Available Not Available Not Available NOT HISPANIC OR LATINO YES;YES NO;NO NO;NO YES;YES TCGA-49-6742-F14741;TCGA-49-6742-F70579 e6565afe-c87f-4fa9-94e1-29b1e25d0ab8;E22C1C68-2566-426D-AEB5-065CFD61A1C2 9;25 Not Available;Not Available 214;214 Not Applicable;488 445;Not Available 214;214 Not Available;3 Scheduled Follow-up Submission;Scheduled Follow-up Submission Not Available;Progressive Disease Not Available;40 Not Available;NO 8;2 Not Available;Distant Metastasis YES;YES Not Available;Post-Adjuvant Therapy WITH TUMOR;WITH TUMOR YES;YES Progressive Disease;Progressive Disease Not Available;Not Available NO;NO Alive;Dead 2011;2015 MALE Mucinous (Colloid) Carcinoma No C34.1 8480/3 C34.1 YES Not Available YES NO Not Available Not Available 8 Not Available Not Available Not Available Not Available Not Available Not Available Not Available Not Available Not Available Not Available 10 Yes 6742 Not Available TUMOR FREE Not Available Not Available Not Available Not Available Not Available Not Available Not Available WHITE Not Available R0 Not Applicable Not Applicable Not Applicable Not Applicable Not Applicable Not Applicable Not Applicable Not Applicable Not Applicable Stage IIA Not Applicable Not Applicable Not Applicable 7th Not Applicable Not Applicable Not Applicable M0 N1 T2a Not Available NO YES 49 3 Lung Alive 2011 2010 Not Available

TCGA-67-6215 174 Alive T2a N0 M0 Stage IB 52 R-Lower Not Applicable TCGA-67-6215 dbd5b0de-94c9-45dd-afb3-6820a7ecaca2 2 -19310 Not Applicable 0 162 Not Available Lung Adenocarcinoma Not Available TCGA-67-6215-D13739;TCGA-67-6215-D13760 f689f65f-4ee1-458b-a0a3-0830819796fc;7199c0c9-6baf-4418-a719-d2c648677db4 Not Available;Not Available 11;12 148;148 85;85 CISPLATIN;ALIMTA Not Available;Not Available 7;7 4;4 135;900 mg;mg ADJUVANT;ADJUVANT Not Applicable;Not Applicable 1;1 IV;IV NO;NO Chemotherapy;Chemotherapy Not Available;Not Available Not Available;Not Available Not Available;Not Available Not Available;Not Available 2011;2011 2 Not Available NO Not Available Not Available Not Available NO Not Available NOT HISPANIC OR LATINO Not Available Not Available Not Available Not Available TCGA-67-6215-F13585 98f5011f-0421-4bb4-a740-a06c2e8d8794 6 Not Applicable Not Applicable Not Applicable 174 Not Applicable 2 Scheduled Follow-up Submission Not Available Not Available Not Available 7 Not Available NO Adjuvant therapy TUMOR FREE YES Complete Remission/Response Not Available NO Alive 2011 FEMALE Lung Adenocarcinoma- Not Otherwise Specified (NOS) No C34.3 8140/3 C34.3 YES Not Available NO Not Available Not Available Central Lung 6 Not Available Not Available Not Available Not Available Not Available Not Available Not Available Not Available Not Available Not Available Not Available No 6215 Post-Adjuvant Therapy TUMOR FREE Not Available Not Available Not Available Not Available Not Available Not Available Not Available WHITE Not Available R0 Not Applicable Not Applicable Not Applicable Not Applicable Not Applicable Not Applicable Not Applicable Not Applicable Not Applicable Stage IB Not Applicable Not Applicable Not Applicable 7th Not Applicable Not Applicable Not Applicable M0 N0 T2a Not Available YES NO 67 1 Lung Alive 2011 2010 Not Available

TCGA-NJ-A4YF 2161 Alive T1 N0 M0 Stage IA 50 R-Upper Not Applicable TCGA-NJ-A4YF 595FC3AD-F603-421B-B130-52F1F617050B 25 -18584 Not Applicable 0 1720 Not Available Lung Adenocarcinoma 77.5 Not Evaluated Not Available NO Not Available Not Available Not Available NO Not Available NOT HISPANIC OR LATINO Not Available Not Available Not Available Not Available TCGA-NJ-A4YF-F50858 635C2793-F06E-4540-80A4-D9F2C1D72DFB 1 Not Applicable Not Applicable Not Applicable 2161 Not Applicable Not Available Scheduled Follow-up Submission Stable Disease Not Available NO 11 Not Available NO Not Available TUMOR FREE NO Stable Disease Not Available NO Alive 2013 FEMALE Lung Adenocarcinoma Mixed Subtype No C34.1 8255/3 C34.1 YES Not Evaluated NO Not Available Not Available Unknown 4 Not Available Not Available Not Available Not Available Not Available Not Available Not Available Not Available Not Available NO 30 No A4YF Not Evaluated Unknown 95.1 83.9 Unknown 96 80.7 Not Applicable YES BLACK OR AFRICAN AMERICAN Unknown Not Evaluated Not Applicable Not Applicable Not Applicable Not Applicable Not Applicable Not Applicable Not Applicable Not Applicable Not Applicable Stage IA Not Applicable Not Applicable Not Applicable 6th Not Applicable Not Applicable Not Applicable M0 N0 T1 Not Available NO YES NJ 3 Lung Alive 2013 2007 Not Available

TCGA-50-6593 336 Dead T1 N2 M0 Stage IIIA 49 R-Upper Not Applicable TCGA-50-6593 e10568fe-0436-43f2-9f0f-48f9903868c4 25 -18152 336 0 Not Available Not Available Lung Adenocarcinoma Not Available Not Available Not Available YES Not Available Not Available Not Available YES Not Available NOT HISPANIC OR LATINO NO NO Not Available NO TCGA-50-6593-F43840 BB824133-F8BB-4916-968D-86355FA69BA7 13 Not Available Not Available 336 Not Available 266 Not Available Scheduled Follow-up Submission Progressive Disease Not Available NO 6 Distant Metastasis YES Not Available WITH TUMOR NO Progressive Disease Convincing Imaging YES Dead 2013 FEMALE Lung Adenocarcinoma Mixed Subtype No C34.1 8255/3 C34.1 YES Not Available YES NO Not Available Not Available 8 Not Available Not Available Not Available Not Available Not Available Not Available Not Available Not Available Not Available Not Available Not Available No 6593 Not Available WITH TUMOR Not Available Not Available Not Available Not Available Not Available Not Available NO WHITE Not Available Primary Tumor Field TCGA-50-6593-R44122 CAA51D9B-D706-4474-8194-EC7E82AFBF1E Not Available 13 210 173 Radiographic Progressive Disease 6 27 5400 NO External Not Applicable Not Available Not Available cGy 2013 R0 Not Applicable Not Applicable Not Applicable Not Applicable Not Applicable Not Applicable Not Applicable Not Applicable Not Applicable Stage IIIA Not Applicable Not Applicable Not Applicable 6th Not Applicable Not Applicable Not Applicable M0 N2 T1 Not Available NO YES 50 4 Lung Dead 2011 2008 Not Available

TCGA-50-5946 1617 Alive T1 N0 MX Stage IA 62 R-Upper Not Applicable TCGA-50-5946 c95957a7-1a1a-4c8d-bb61-7c99b500f224 2 -22852 Not Applicable 0 349 Not Available Lung Adenocarcinoma Not Available Not Available Not Available Not Available Not Available Not Available Not Available Not Available Not Available Not Available YES;NO YES;NO NO;Not Available YES;NO TCGA-50-5946-F32143;TCGA-50-5946-F70455 28fbb9d8-ddc3-44a6-9f34-2ee25dbe35c3;27EC65A2-6B63-4A0F-A8E3-7C0C7905CF5F 17;16 Not Available;Not Available 221;Not Available Not Applicable;Not Applicable 686;1617 221;811 Not Available;Not Available Scheduled Follow-up Submission;Not Available Progressive Disease;Progressive Disease Not Available;Not Available NO;NO 5;2 Distant Metastasis;Distant Metastasis YES;YES Not Available;Not Available WITH TUMOR;WITH TUMOR NO;NO Progressive Disease;Progressive Disease Biopsy with Histologic Confirmation;Convincing Imaging;Convincing Imaging NO;NO Alive;Alive 2012;2015 MALE Lung Adenocarcinoma Mixed Subtype No C34.1 8255/3 C34.1 YES Not Available Not Available Not Available Not Available Not Available 7 Not Available Not Available Not Available Not Available Not Available Not Available Not Available Not Available Not Available Not Available Not Available No 5946 Not Available WITH TUMOR Not Available Not Available Not Available Not Available Not Available Not Available NO WHITE Not Available Not Available Not Applicable Not Applicable Not Applicable Not Applicable Not Applicable Not Applicable Not Applicable Not Applicable Not Applicable Stage IA Not Applicable Not Applicable Not Applicable Not Available Not Applicable Not Applicable Not Applicable MX N0 T1 2009 NO YES 50 4 Lung Alive 2011 2010 Not Available

TCGA-38-A44F 133 Alive T2a N0 M0 Stage IB 80 R-Upper Not Applicable TCGA-38-A44F 258A3A41-211B-4C66-BD3B-EF03014A6967 31 -29534 Not Applicable 0 133 Not Available Lung Adenocarcinoma 35 Not Evaluated Not Available NO Not Available Not Available FISH YES Not Available NOT HISPANIC OR LATINO Not Available Not Available Not Available Not Available TCGA-38-A44F-F47557 25815E78-A0C8-4308-9087-3C032BCFED78 26 Not Available Not Available Not Applicable Not Available Not Available Not Available Scheduled Follow-up Submission Unknown Not Available YES 8 Not Available Unknown Not Available Not Available Not Available Not Available Not Available Not Available Alive 2013 MALE Lung Adenocarcinoma Mixed Subtype No C34.1 8550/3 C34.1 YES Not Evaluated NO Not Available Not Available Peripheral Lung 10 Not Available Not Available Not Available Not Available Not Available Not Available Not Available Not Available Not Available NO 12 No A44F Not Available TUMOR FREE 71 Not Available NO 72 52 Complete Remission/Response YES WHITE NO R0 Not Applicable Not Applicable Not Applicable Not Applicable Not Applicable Not Applicable Not Applicable Not Applicable Not Applicable Stage IB Not Applicable Not Applicable Not Applicable 7th Not Applicable Not Applicable Not Applicable M0 N0 T2a 1977 YES NO 38 3 Lung Alive 2012 2012 1965
[truncated: 501,642 more chars]
