# Supplementary material for: An Immune-Related Signature Predicts Survival in Patients With Lung Adenocarcinoma
Source: Front Oncol. 2019 Dec 10;9:1314. doi: 10.3389/fonc.2019.01314 (PMC6914845; doi:10.3389/fonc.2019.01314)
Supplement: Supplementary file 2 [file Table_2.doc]

**Table S2** Patient characteristics in training set and testing set

| Clinical Features | Overall | Training set | Testing set | P value |
| --- | --- | --- | --- | --- |
| OS | 513 | 256 | 257 | 0.99 |
| Event | 513 | 256 | 257 | 0.558 |
| Alive | 330 | 161 | 169 |  |
| Dead | 183 | 95 | 88 |  |
| T | 513 | 256 | 257 | 0.233 |
| T1 | 168 | 87 | 81 |  |
| T2 | 276 | 142 | 134 |  |
| T3 | 47 | 21 | 26 |  |
| T4 | 19 | 5 | 14 |  |
| TX | 3 | 1 | 2 |  |
| N | 512 | 256 | 256 | 0.886 |
| N0 | 330 | 168 | 162 |  |
| N1 | 95 | 49 | 46 |  |
| N2 | 74 | 33 | 41 |  |
| N3 | 2 | 1 | 1 |  |
| NX | 11 | 5 | 6 |  |
| M | 508 | 255 | 253 | 0.206 |
| M0 | 344 | 180 | 164 |  |
| M1 | 25 | 9 | 16 |  |
| MX | 140 | 66 | 74 |  |
| Stage | 505 | 254 | 251 | 0.357 |
| I | 274 | 143 | 131 |  |
| II | 121 | 62 | 59 |  |
| III | 84 | 40 | 44 |  |
| IV | 26 | 9 | 17 |  |
| Age | 494 | 247 | 247 | 0.64 |
| 0~50 | 33 | 13 | 20 |  |
| 50~60 | 103 | 52 | 51 |  |
| 60~70 | 169 | 90 | 79 |  |
| 70~80 | 158 | 78 | 80 |  |
| 80~100 | 31 | 14 | 17 |  |
| Gender | 510 | 256 | 257 | 0.754 |
| FEMALE | 276 | 140 | 136 |  |
| MALE | 237 | 116 | 121 |  |
