# Supplementary material for: An Immune-Related Signature Predicts Survival in Patients With Lung Adenocarcinoma
Source: Front Oncol. 2019 Dec 10;9:1314. doi: 10.3389/fonc.2019.01314 (PMC6914845; doi:10.3389/fonc.2019.01314)
Supplement: Supplementary file 3 [file Table_3.doc]

**Table_S3_ Data from the training set samples.**

Genes TCGA-50-6594-01 TCGA-75-7030-01 TCGA-78-7163-01 TCGA-38-4631-01 TCGA-75-6207-01 TCGA-38-7271-01 TCGA-55-8087-01 TCGA-38-4628-01 TCGA-93-A4JO-01 TCGA-62-8397-01 TCGA-55-8508-01 TCGA-91-8497-01 TCGA-55-7570-01 TCGA-86-8674-01 TCGA-78-7152-01 TCGA-49-AAR9-01 TCGA-50-5946-01 TCGA-55-8090-01 TCGA-73-4668-01 TCGA-53-A4EZ-01 TCGA-MN-A4N1-01 TCGA-62-8394-01 TCGA-78-7155-01 TCGA-05-4382-01 TCGA-44-7659-01 TCGA-95-8494-01 TCGA-69-7980-01 TCGA-49-4510-01 TCGA-44-6774-01 TCGA-44-7661-01 TCGA-75-5125-01 TCGA-75-6203-01 TCGA-MP-A4T9-01 TCGA-69-7764-01 TCGA-50-5066-01 TCGA-86-8668-01 TCGA-91-8496-01 TCGA-55-6987-01 TCGA-67-4679-01 TCGA-S2-AA1A-01 TCGA-73-4662-01 TCGA-67-3772-01 TCGA-69-A59K-01 TCGA-73-4675-01 TCGA-44-7660-01 TCGA-35-4123-01 TCGA-L9-A50W-01 TCGA-55-8085-01 TCGA-78-8648-01 TCGA-78-7539-01 TCGA-78-7159-01 TCGA-MP-A4TI-01 TCGA-86-8359-01 TCGA-50-5044-01 TCGA-67-6217-01 TCGA-78-7150-01 TCGA-91-6847-01 TCGA-78-7220-01 TCGA-44-8117-01 TCGA-78-7146-01 TCGA-97-A4M0-01 TCGA-55-A48Z-01 TCGA-44-3918-01 TCGA-78-7147-01 TCGA-62-A46S-01 TCGA-55-8203-01 TCGA-49-6744-01 TCGA-55-A57B-01 TCGA-55-8511-01 TCGA-97-8172-01 TCGA-05-4249-01 TCGA-55-6543-01 TCGA-78-7162-01 TCGA-62-8398-01 TCGA-78-7143-01 TCGA-05-4250-01 TCGA-05-5420-01 TCGA-50-6673-01 TCGA-73-4676-01 TCGA-78-8662-01 TCGA-93-7347-01 TCGA-49-6743-01 TCGA-49-6767-01 TCGA-55-6981-01 TCGA-05-4390-01 TCGA-86-7701-01 TCGA-93-8067-01 TCGA-55-A492-01 TCGA-69-7979-01 TCGA-55-7576-01 TCGA-44-5644-01 TCGA-44-7669-01 TCGA-44-7670-01 TCGA-05-4424-01 TCGA-55-A493-01 TCGA-86-8279-01 TCGA-44-3396-01 TCGA-62-8402-01 TCGA-44-6778-01 TCGA-MP-A5C7-01 TCGA-50-5045-01 TCGA-44-A47A-01 TCGA-86-7713-01 TCGA-97-A4M3-01 TCGA-71-6725-01 TCGA-62-A46Y-01 TCGA-55-8301-01 TCGA-62-A46V-01 TCGA-49-4507-01 TCGA-NJ-A4YF-01 TCGA-97-7554-01 TCGA-MP-A4SV-01 TCGA-49-4506-01 TCGA-05-4402-01 TCGA-55-A494-01 TCGA-49-4512-01 TCGA-44-A47B-01 TCGA-62-A470-01 TCGA-69-8255-01 TCGA-75-6214-01 TCGA-78-7633-01 TCGA-L9-A444-01 TCGA-78-7542-01 TCGA-73-4658-01 TCGA-91-6835-01 TCGA-86-A4JF-01 TCGA-86-6562-01 TCGA-86-8672-01 TCGA-67-3774-01 TCGA-55-8514-01 TCGA-86-8055-01 TCGA-05-4403-01 TCGA-05-4405-01 TCGA-97-8179-01 TCGA-44-3398-01 TCGA-44-7672-01 TCGA-95-7947-01 TCGA-73-7498-01 TCGA-44-2659-01 TCGA-97-A4M1-01 TCGA-97-7938-01 TCGA-55-8207-01 TCGA-MP-A4TF-01 TCGA-NJ-A55R-01 TCGA-91-6829-01 TCGA-44-2662-01 TCGA-86-A4D0-01 TCGA-97-8171-01 TCGA-86-6851-01 TCGA-64-5779-01 TCGA-86-7954-01 TCGA-50-5935-01 TCGA-95-7944-01 TCGA-MN-A4N5-01 TCGA-53-7813-01 TCGA-49-AAR4-01 TCGA-MP-A4TA-01 TCGA-NJ-A55O-01 TCGA-05-4433-01 TCGA-L9-A443-01 TCGA-50-7109-01 TCGA-MP-A4T7-01 TCGA-75-6205-01 TCGA-55-7281-01 TCGA-95-A4VK-01 TCGA-64-5781-01 TCGA-80-5607-01 TCGA-86-A4P8-01 TCGA-05-4432-01 TCGA-55-8089-01 TCGA-71-8520-01 TCGA-64-1676-01 TCGA-67-3773-01 TCGA-97-8176-01 TCGA-64-5778-01 TCGA-MP-A4TD-01 TCGA-50-6593-01 TCGA-97-7553-01 TCGA-55-6971-01 TCGA-86-8278-01 TCGA-55-7910-01 TCGA-50-6590-01 TCGA-55-A490-01 TCGA-78-7156-01 TCGA-49-AARR-01 TCGA-78-8660-01 TCGA-55-A48Y-01 TCGA-50-5068-01 TCGA-62-A471-01 TCGA-J2-A4AE-01 TCGA-55-7574-01 TCGA-75-7031-01 TCGA-05-4430-01 TCGA-55-8097-01 TCGA-44-6776-01 TCGA-86-8358-01 TCGA-50-5049-01 TCGA-55-7726-01 TCGA-86-8076-01 TCGA-L9-A8F4-01 TCGA-44-2668-01 TCGA-73-A9RS-01 TCGA-93-A4JP-01 TCGA-78-7535-01 TCGA-NJ-A4YQ-01 TCGA-78-7148-01 TCGA-55-6986-01 TCGA-J2-8192-01 TCGA-78-7153-01 TCGA-55-8092-01 TCGA-55-6968-01 TCGA-55-8512-01 TCGA-55-6985-01 TCGA-50-5941-01 TCGA-L9-A743-01 TCGA-44-6146-01 TCGA-4B-A93V-01 TCGA-MP-A4TC-01 TCGA-J2-A4AD-01 TCGA-50-8459-01 TCGA-62-A46P-01 TCGA-49-4486-01 TCGA-64-5815-01 TCGA-35-4122-01 TCGA-97-7547-01 TCGA-83-5908-01 TCGA-55-6712-01 TCGA-86-7714-01 TCGA-64-5774-01 TCGA-55-8505-01 TCGA-86-8056-01 TCGA-55-7913-01 TCGA-75-5147-01 TCGA-93-7348-01 TCGA-50-5072-01 TCGA-55-A48X-01 TCGA-55-8507-01 TCGA-44-2657-01 TCGA-78-7540-01 TCGA-55-7727-01 TCGA-86-A456-01 TCGA-55-6970-01 TCGA-55-8204-01 TCGA-44-6148-01 TCGA-99-AA5R-01 TCGA-86-7711-01 TCGA-05-5423-01 TCGA-93-A4JN-01 TCGA-38-4629-01 TCGA-55-6642-01 TCGA-55-8302-01 TCGA-95-8039-01 TCGA-53-7626-01 TCGA-55-6975-01 TCGA-55-8614-01 TCGA-86-A4P7-01

ENSG00000160862.11 152.123153686523 114.466270446777 2126.95263671875 0.263730496168137 2255.39794921875 13.2542610168457 6.86953544616699 3.18348860740662 5.36407089233398 813.623046875 42.8067741394043 22.048038482666 18.5435047149658 30.2660751342773 1.49950051307678 0.0554228536784649 0.287173390388489 4.42689371109009 5.28771209716797 0.497816860675812 10.8303413391113 42.8882713317871 0 0.468396604061127 4.60388135910034 606.6669921875 25.9523620605469 32.4032516479492 0.100663363933563 20.2149105072021 18.7550621032715 1244.46923828125 74.9180603027344 1.91656172275543 6.1907434463501 60.1469497680664 22.8138637542725 32.0410919189453 130.383346557617 7.29395866394043 1.78172886371613 15.7243175506592 19.8240661621094 0.363730758428574 1277.8076171875 0.389912545681 456.729217529297 0.849679410457611 21.3064346313477 0.120556265115738 0.706847965717316 9.51125717163086 19.2969131469727 8.72170352935791 9.74090385437012 0.308051288127899 11.4660291671753 0.751194834709167 3.50678896903992 180.812545776367 51.4335594177246 48.6686668395996 203.651473999023 393.482116699219 0.414209753274918 6.22844505310059 71.4446792602539 40.9753036499023 7.34246015548706 7.23348951339722 6.64370107650757 2186.0595703125 11.9249229431152 138.517288208008 97.3953857421875 134.660446166992 12.3456602096558 153.113250732422 13.9330358505249 0.536415100097656 5.09238386154175 437.571380615234 18.0557022094727 48.1669273376465 10.3155431747437 1.98906898498535 38.7843704223633 158.636352539062 0.437323272228241 2.02095627784729 115.417015075684 7.57736015319824 11.5986824035645 9.67436599731445 71.4246139526367 2.53997159004211 3.11900353431702 0.200266718864441 2.41104435920715 2.12196373939514 362.313934326172 5.76303100585938 0.707872569561005 3.10638189315796 16.809757232666 162.830276489258 6.40486001968384 494.058288574219 1086.45190429688 1.83094048500061 4.84497547149658 162.703338623047 2.05300736427307 131.284332275391 68.6447143554688 40.1328430175781 0.448574632406235 3.81933903694153 152.524551391602 1.37889921665192 3.93397951126099 0.882511556148529 16.5198154449463 86.4546127319336 1.97030544281006 731.689880371094 6.33602142333984 5.34100580215454 5.06354236602783 7.99876689910889 478.478973388672 110.95491027832 14.817346572876 45.3038902282715 1.27515208721161 7.11807012557983 530.773254394531 2.66384887695312 1189.63745117188 61.244026184082 18.2140941619873 4.91581773757935 128.533111572266 238.091690063477 2.39340996742249 72.7067031860352 14.7129802703857 101.02889251709 9.5566577911377 0.521482527256012 53.9283103942871 209.997009277344 159.453140258789 12.1036319732666 6.39238500595093 11.622597694397 153.337097167969 9.90520477294922 196.553649902344 18.4249820709229 10.0283117294312 42.7317695617676 465.360198974609 0.346223711967468 5.41633224487305 43.8702354431152 4.59892129898071 23.1293182373047 4.08719635009766 1.59938251972198 14.4555749893188 1790.10681152344 4.00206279754639 54.2767181396484 9.90514659881592 28.3084964752197 8.28778171539307 38.6573371887207 2.13345003128052 2.2871196269989 0.678522348403931 3.63252663612366 4.14465999603271 1.78972446918488 57.980770111084 3.01702094078064 0.871033251285553 5.70732831954956 0.78501558303833 380.140197753906 34.5625610351562 27.9115924835205 8.41555404663086 11.0209121704102 17.049955368042 26.9366550445557 0.865204811096191 25.235559463501 248.884170532227 11.9901103973389 10.9124402999878 99.7346267700195 268.315032958984 0.631070017814636 1642.81591796875 105.460220336914 10.5394954681396 220.17951965332 100.591049194336 2.96535491943359 0.983073115348816 77.1327590942383 71.4362716674805 43.2212677001953 51.2589111328125 153.536437988281 0.120932407677174 3.47352170944214 1276.27075195312 50.3809661865234 0.358568400144577 32.6952133178711 100.612426757812 408.696960449219 43.0126876831055 2.34543061256409 11.6639356613159 280.526763916016 2.32216191291809 2.80789971351624 1.6819806098938 85.6884765625 208.515274047852 84.1873779296875 7.16651964187622 48.2321166992188 31.1370964050293 60.88818359375 209.531753540039 15.5679969787598 48.4678497314453 12.5501880645752 9.96642780303955 124.649894714355 23.829761505127 255.357894897461 172.186874389648 11.3548717498779 45.3922271728516 0.164085954427719 13.9082803726196 80.7995834350586 6.73829555511475 9.69321537017822 16.934362411499 5.69837856292725

ENSG00000166710.16 4533.78515625 3655.923828125 2387.72875976562 2166.71533203125 1422.36157226562 3703.3662109375 1019.57598876953 1487.2919921875 2444.73657226562 1052.2744140625 1274.79675292969 2653.55541992188 1016.27789306641 840.302856445312 2502.6552734375 2474.373046875 1088.12536621094 2052.20849609375 1258.88464355469 1208.47888183594 516.020874023438 2674.55444335938 683.564880371094 3058.64282226562 2310.4326171875 2613.62670898438 2589.57373046875 2342.98071289062 1881.86437988281 3561.71069335938 3270.20727539062 4213.74658203125 2047.70385742188 1561.39428710938 6248.197265625 1612.2607421875 3111.58959960938 2624.35180664062 1635.10229492188 3884.59936523438 2529.84423828125 3540.27026367188 2579.74682617188 1002.47064208984 1030.04846191406 5772.67919921875 2699.96826171875 3983.5078125 3068.24096679688 3358.57543945312 1465.953125 3810.65625 1294.76489257812 3088.86010742188 4259.826171875 1984.07482910156 703.095153808594 1588.34826660156 1452.31433105469 1272.90625 3010.3896484375 2561.087890625 5098.87939453125 2497.00952148438 1963.26843261719 1700.58862304688 3837.67333984375 2138.04638671875 1873.32922363281 2081.67016601562 1675.80187988281 1987.44812011719 3096.19458007812 2832.28564453125 2259.90795898438 4760.0654296875 9651.833984375 1523.57946777344 1657.32495117188 902.690490722656 3288.4873046875 1959.83874511719 2531.14916992188 866.094604492188 985.708923339844 2815.25756835938 836.447875976562 1855.14721679688 896.113830566406 1906.30676269531 873.057312011719 2494.90551757812 1375.48205566406 2487.56591796875 2079.8994140625 1110.85925292969 3137.96875 7501.78515625 4784.3974609375 1150.27893066406 4397.4462890625 2820.79614257812 739.107421875 932.29150390625 1633.93688964844 2711.46606445312 4818.88232421875 1370.01623535156 1801.43444824219 541.872253417969 2111.46484375 2641.2861328125 3609.35717773438 2786.45678710938 508.219177246094 891.836120605469 2180.47290039062 1304.3896484375 2503.72583007812 2586.091796875 1631.25524902344 2842.58081054688 3700.0029296875 2677.22094726562 3452.52368164062 2137.08203125 1691.66784667969 4515.94873046875 5752.12255859375 1087.84045410156 2744.0703125 10910.0576171875 1507.63562011719 1361.08874511719 6369.99267578125 2874.27563476562 3245.14306640625 1375.09680175781 2192.935546875 2204.7587890625 2061.85961914062 1726.95422363281 2187.98168945312 1087.60131835938 2004.435546875 1035.57922363281 811.570678710938 842.016235351562 2248.77563476562 1418.283203125 4822.462890625 3128.37353515625 3892.6201171875 3222.76000976562 1002.44720458984 2577.75244140625 2900.96899414062 1721.966796875 6879.23828125 2094.67602539062 1973.00134277344 1263.50158691406 4480.1298828125 1862.25549316406 1876.84436035156 4764.455078125 2877.67456054688 4348.85546875 2161.9990234375 4655.73876953125 2329.02514648438 4492.2451171875 7403.1708984375 861.568359375 6398.3505859375 967.7197265625 1606.83666992188 4420.271484375 3000.20190429688 2697.30493164062 925.463317871094 3232.8564453125 593.495239257812 1565.41577148438 2526.25927734375 1880.07385253906 1305.13293457031 8335.6201171875 1592.06945800781 2942.6884765625 2725.91748046875 1883.08203125 3774.9296875 1191.74243164062 1153.92224121094 1620.70556640625 6353.287109375 1818.28308105469 2976.2939453125 1828.60827636719 1843.94506835938 2982.71899414062 3473.32006835938 622.52392578125 2767.61328125 3496.55810546875 1300.39587402344 3122.63208007812 1611.9912109375 5747.8056640625 2237.7998046875 1266.18725585938 2217.48974609375 4353.75634765625 3146.17236328125 1283.69262695312 1291.953125 3631.16455078125 1157.50366210938 2637.5068359375 1591.751953125 1281.40832519531 2721.076171875 6420.6865234375 2693.67407226562 7065.20458984375 4626.8671875 1815.60083007812 907.103271484375 1375.99853515625 2443.72631835938 1746.47448730469 6356.1669921875 1379.13635253906 1985.69104003906 1587.01586914062 1062.58374023438 3400.47583007812 2972.2587890625 3047.28588867188 3829.79345703125 2825.78833007812 2787.19580078125 1468.61877441406 3238.3125 2200.353515625 4254.76025390625 2324.9482421875 4988.08447265625 2025.43603515625 3111.52734375 3389.42749023438 3170.73071289062 1788.20202636719 1302.974609375 2240.3310546875

ENSG00000179218.12 1206.45776367188 901.832763671875 1383.08923339844 1135.87109375 1283.859375 1049.96606445312 1235.88781738281 1084.16369628906 1118.4423828125 647.801574707031 1450.28540039062 1094.4287109375 1142.05749511719 1959.31774902344 1502.84436035156 1710.15612792969 1316.19543457031 1046.51342773438 2039.47766113281 698.452026367188 1001.35034179688 1892.65148925781 3513.79467773438 1449.62976074219 996.90478515625 1829.74426269531 1446.10534667969 1090.02551269531 1530.73620605469 1761.90197753906 983.845764160156 1049.00842285156 884.365844726562 1446.22216796875 626.827941894531 942.612060546875 778.433837890625 988.562377929688 811.818115234375 1325.10217285156 1364.08630371094 1282.0126953125 668.814392089844 1458.05163574219 1083.71411132812 770.1884765625 1821.56518554688 2412.78466796875 1186.34521484375 1265.35290527344 2033.34240722656 1297.23645019531 1152.73803710938 1940.17065429688 2172.39916992188 1592.51208496094 1573.5458984375 1339 1079.92309570312 1515.97265625 1073.15539550781 859.585632324219 1067.00366210938 1481.99304199219 892.507995605469 1632.56701660156 1449.96264648438 1298.85339355469 1170.39099121094 854.000732421875 1679.7119140625 1357.45446777344 1266.55432128906 1319.45849609375 1178.49011230469 1188.07592773438 876.1669921875 1400.20678710938 1310.10864257812 2709.216796875 1421.69287109375 3844.72924804688 1346.17907714844 1522.220703125 2114.36254882812 1705.43884277344 1786.53454589844 2047.34802246094 1055.85192871094 1202.80383300781 1701.67028808594 1334.26501464844 1358.2353515625 2136.5068359375 1124.28234863281 2578.0283203125 1290.64343261719 2475.81323242188 1119.02954101562 1454.43493652344 1140.74206542969 2640.8134765625 1712.29528808594 1253.19897460938 2694.93383789062 1737.26110839844 1470.04467773438 2412.36376953125 1128.80773925781 1002.94732666016 1519.70141601562 1603.44384765625 812.246398925781 2046.10327148438 2388.42358398438 792.13427734375 1287.85119628906 1319.66772460938 1130.56066894531 1045.88354492188 1895.57934570312 1202.44079589844 2108.69921875 1332.20202636719 1431.00549316406 1828.29089355469 1188.64855957031 1278.44543457031 1612.52124023438 867.485717773438 1415.90161132812 1047.08825683594 1375.81567382812 1281.40393066406 1163.208984375 1352.62854003906 1812.44970703125 973.138427734375 1060.080078125 849.279663085938 1450.61108398438 934.604187011719 1219.33422851562 1116.93786621094 2303.33056640625 857.061462402344 1154.478515625 1083.54650878906 1186.11083984375 994.877197265625 3176.78637695312 1523.3193359375 1343.30883789062 1121.27978515625 1797.62231445312 921.034729003906 1514.76110839844 857.993469238281 1032.08935546875 896.587646484375 2080.05908203125 794.082763671875 1189.79479980469 1392.56262207031 583.084167480469 1587.19213867188 1118.66125488281 589.063598632812 1679.14709472656 1282.95495605469 1697.21374511719 1447.98071289062 1081.34313964844 1070.04736328125 1334.15844726562 945.175170898438 1789.44763183594 1279.21411132812 1142.24792480469 3196.51416015625 1427.9677734375 1711.02648925781 1548.78039550781 949.2646484375 757.918395996094 2165.24975585938 1675.49572753906 789.245483398438 1216.75805664062 764.009155273438 1069.93688964844 1000.91979980469 1662.46960449219 1193.87536621094 1169.72497558594 905.750854492188 1155.876953125 1168.75427246094 1333.41821289062 1136.4462890625 985.202026367188 1847.49206542969 1060.94506835938 1230.93713378906 645.775146484375 1461.05859375 1734.46936035156 1172.43408203125 1395.62915039062 1513.75842285156 995.0234375 808.181091308594 1205.28430175781 2228.1611328125 986.3056640625 540.280090332031 1295.87927246094 1062.67565917969 992.626098632812 888.902160644531 1717.35375976562 1192.05737304688 1504.41284179688 1064.70739746094 1391.53295898438 1697.72399902344 1181.5859375 1034.837890625 1978.30651855469 1229.58874511719 1330.23156738281 1554.4833984375 2245.40014648438 1134.99035644531 1376.83630371094 1250.68371582031 2129.22119140625 920.03173828125 1049.31457519531 1351.10205078125 1567.72619628906 1244.01806640625 1553.95324707031 867.611633300781 698.760192871094 1326.00207519531 1020.83294677734 1447.61096191406 1273.85461425781 1260.36779785156 753.782958984375 1525.40661621094 1416.49462890625 1220.72094726562 1537.35119628906 1261.83422851562

ENSG00000127022.13 751.661437988281 320.534759521484 906.056823730469 222.176193237305 492.593139648438 304.259094238281 426.606414794922 251.470489501953 254.186050415039 303.901031494141 424.134521484375 407.761871337891 313.687377929688 389.392211914062 374.769958496094 519.473510742188 328.531463623047 318.780578613281 291.177429199219 204.32585144043 115.399932861328 303.170440673828 557.703369140625 375.681243896484 479.601287841797 382.787170410156 322.863372802734 375.80078125 540.7880859375 531.311645507812 458.870025634766 342.339569091797 398.901031494141 848.543151855469 186.509399414062 539.576354980469 249.115386962891 749.352844238281 330.440185546875 241.989028930664 606.643310546875 337.782684326172 383.2216796875 563.342102050781 278.179504394531 207.374481201172 324.901885986328 479.0703125 289.086730957031 906.550048828125 546.711608886719 312.95849609375 207.61247253418 263.245697021484 605.216003417969 549.179260253906 376.523651123047 563.010375976562 337.719818115234 927.054321289062 472.402465820312 498.759887695312 380.236236572266 614.987182617188 692.619262695312 453.762908935547 442.514099121094 298.304046630859 231.910720825195 311.762542724609 400.696960449219 901.404907226562 586.957153320312 565.555236816406 443.217956542969 373.267517089844 327.01806640625 648.333557128906 447.072906494141 243.348541259766 453.309509277344 689.011047363281 207.256439208984 385.371856689453 501.641204833984 429.18994140625 544.860900878906 213.660018920898 270.426483154297 434.207000732422 500.269012451172 727.45654296875 470.028411865234 930.120788574219 515.100219726562 352.003784179688 315.150329589844 503.700927734375 418.013275146484 276.860748291016 219.288879394531 453.302978515625 583.100158691406 414.746032714844 313.05322265625 489.964813232422 420.683349609375 472.231597900391 216.983581542969 422.942810058594 418.035858154297 416.892303466797 478.644714355469 418.947021484375 497.655487060547 176.985443115234 352.765258789062 294.69091796875 434.458221435547 331.862335205078 900.146789550781 202.100860595703 445.776947021484 367.91650390625 498.260803222656 357.182495117188 297.458984375 383.135192871094 471.473846435547 187.786773681641 947.858703613281 322.766571044922 277.402069091797 408.446838378906 509.677795410156 415.740203857422 577.440246582031 486.140686035156 573.353820800781 314.072692871094 658.140563964844 658.281311035156 962.945739746094 571.432067871094 707.689453125 454.646331787109 530.119384765625 326.898010253906 302.713104248047 184.002197265625 1286.17321777344 681.463562011719 265.936126708984 278.322509765625 531.621337890625 106.071014404297 717.413635253906 368.022644042969 414.914611816406 303.824432373047 343.573547363281 437.9853515625 493.679595947266 148.9951171875 782.420349121094 385.378356933594 363.546325683594 252.493026733398 509.155395507812 470.090881347656 251.454162597656 196.268966674805 340.401916503906 466.296447753906 268.366271972656 260.493133544922 341.534027099609 455.494079589844 372.081817626953 736.028381347656 443.399932861328 409.0205078125 370.821960449219 551.036254882812 261.256530761719 408.779449462891 307.267547607422 316.791870117188 312.472045898438 323.19921875 273.686828613281 599.002563476562 548.486450195312 438.625213623047 489.651885986328 444.177764892578 277.415771484375 412.966705322266 449.335540771484 181.110702514648 373.221282958984 285.428466796875 408.638427734375 142.0107421875 308.536468505859 588.152099609375 659.213256835938 555.928405761719 605.847961425781 515.378112792969 312.634155273438 295.504943847656 318.088134765625 420.724090576172 292.759490966797 226.697036743164 208.573745727539 285.982849121094 226.709732055664 254.329788208008 422.401031494141 264.354675292969 653.116516113281 289.3056640625 826.312805175781 403.330810546875 417.732330322266 518.905639648438 485.450286865234 327.305725097656 1164.94177246094 393.648254394531 1009.23974609375 412.705657958984 557.297485351562 367.749755859375 191.017517089844 336.232788085938 262.077056884766 579.602722167969 293.031066894531 1128.35229492188 286.617034912109 382.128509521484 199.630905151367 464.342742919922 374.161926269531 263.253479003906 362.505401611328 623.022033691406 298.422821044922 416.071807861328 275.35498046875 397.716949462891 228.074249267578 312.127807617188

ENSG00000158477.6 5.75985908508301 44.9488410949707 2.13329553604126 0 2.84620976448059 16.9393844604492 225.914596557617 11.4956216812134 28.5544357299805 11.5418710708618 0 12.668683052063 0.332904070615768 1.96949863433838 12.164626121521 0.071367509663105 0.392902433872223 13.1727094650269 14.7369394302368 7.58188724517822 1.5523077249527 1.24892449378967 0 0.94338846206665 15.9808397293091 71.8415985107422 1.11240887641907 3.30400800704956 2.85171246528625 9.14030361175537 3.27467393875122 19.4374904632568 5.08596181869507 0.793266117572784 0.377808690071106 18.4511623382568 24.1156158447266 2.67766165733337 71.4470367431641 12.4898443222046 10.2819356918335 13.1345634460449 1.0332465171814 20.88942527771 0.446474015712738 9.56607437133789 61.0166168212891 1.13620603084564 1.38813614845276 29.9611663818359 0.251090109348297 15.4938983917236 4.73872852325439 24.4542846679688 41.708309173584 0.0360613465309143 7.3018159866333 0.696460843086243 0.832379639148712 1.33151769638062 69.268310546875 13.5815010070801 5.04105186462402 5.18269777297974 6.98720073699951 10.2748689651489 68.9668197631836 101.688201904297 3.59653854370117 20.4642658233643 360.715972900391 3.03248023986816 7.54224395751953 10.8861417770386 35.5814552307129 0.672193467617035 1.76637673377991 3.68941879272461 7.64776754379272 0.138147354125977 24.5714664459229 0.544240713119507 1.86356246471405 21.6119575500488 0.5814208984375 3.56083941459656 2.70217251777649 1.17506635189056 1.40784275531769 16.4643077850342 0.132461175322533 0.444702535867691 0.877085745334625 0.903863191604614 6.68634605407715 111.783645629883 7.64566993713379 0.368402302265167 2.32850980758667 2.99266505241394 51.6507034301758 207.282974243164 0.273456335067749 5.13393449783325 1.36772704124451 50.0608253479004 7.87597131729126 48.4228630065918 1.72821497917175 0.364879876375198 3.08942103385925 5.93366146087646 5.06324005126953 7.26297473907471 5.65147733688354 13.9483652114868 19.4014186859131 0.421553790569305 0.314338594675064 2.29571986198425 1.52172708511353 0.639226138591766 5.80350732803345 34.9437065124512 7.61143207550049 6.1712474822998 1.64017856121063 2.34028220176697 4.12017488479614 2.52170896530151 6.61711931228638 4.50433301925659 15.1429843902588 11.3658294677734 185.014266967773 8.01762199401855 1.1750385761261 7.20547771453857 3.05149602890015 7.29267740249634 9.33426761627197 8.9891471862793 2.1480975151062 0.578611254692078 4.62295818328857 0.546229600906372 1.09989953041077 0.630477130413055 0.99546605348587 0.410366147756577 1.79000449180603 31.001974105835 0.0200142655521631 1.58075976371765 0.944588661193848 1.87521171569824 0.171175360679626 3.93245768547058 8.0413932800293 9.23499584197998 0.22655026614666 2.03596353530884 32.699390411377 121.361053466797 0.618434071540833 1.01413905620575 19.1325778961182 4.69930171966553 1.05260896682739 3.4110631942749 9.04717922210693 18.8948421478271 43.6095962524414 1.74927866458893 0.523746490478516 5.12321186065674 54.1710395812988 55.737476348877 3.09062719345093 33.9069366455078 0.242701947689056 2.85469436645508 10.5365285873413 0.238408133387566 1.66753017902374 4.79910516738892 0.678876161575317 54.6266174316406 0.379071533679962 5.65448617935181 2.06634497642517 1.58265125751495 41.0743217468262 25.3687305450439 9.95802307128906 0.283383041620255 12.1569757461548 53.4259223937988 25.9835681915283 0.958496034145355 1.23891007900238 0.245324477553368 1.13903343677521 112.08065032959 0.838858187198639 7.34054851531982 9.65770149230957 64.5306930541992 1.28137373924255 0.0949865728616714 0.197795987129211 0.587710916996002 4.78573942184448 9.48626041412354 15.3691053390503 0.251376301050186 0.222462236881256 14.1978216171265 0.517472863197327 27.5719242095947 0.671600341796875 4.05759143829346 22.6195011138916 7.01132917404175 2.03933095932007 1.1831670999527 10.2029294967651 408.038818359375 0.645617127418518 0.731266617774963 18.2839813232422 5.7066011428833 5.28203010559082 6.49964952468872 1.07452356815338 27.0608577728271 0.61272132396698 2.81913614273071 5.40682935714722 7.2246470451355 1.49066078662872 1.46704316139221 6.59344911575317 2.94275498390198 11.1611089706421 2.52503705024719 72.0855484008789 16.6926956176758 0.634572267532349 0.316937953233719 2.43540143966675 61.2028770446777 20.6419734954834 0.4082852602005 5.59708023071289 4.30897665023804

ENSG00000158485.9 0.472049444913864 6.13963890075684 0.294739842414856 0.0637821331620216 0.712742567062378 4.68001317977905 7.95307540893555 3.09275960922241 2.97939562797546 4.10051584243774 0 8.55636405944824 0.181887745857239 0.0569074898958206 0.870355308055878 0.0536151602864265 0 1.93870115280151 0.592042326927185 2.65699219703674 0.814473688602448 0.457687854766846 0.150778740644455 0.255605667829514 3.33060693740845 4.17656660079956 1.18391144275665 0.958454370498657 0.973800837993622 3.36564707756042 3.99768471717834 2.84502029418945 2.48233675956726 0.794592916965485 1.30562019348145 3.23712062835693 6.40831470489502 0.237931802868843 7.89650726318359 17.2147731781006 11.5163040161133 4.65695476531982 1.64378333091736 2.22380018234253 0.0353069044649601 5.32043409347534 7.17247867584229 1.67554569244385 1.77896821498871 2.36580419540405 0.141474425792694 2.71596765518188 0.239327132701874 0.340210944414139 9.36537933349609 0.0541824959218502 0.161338865756989 0.174406439065933 0.375197410583496 0.11114539951086 6.11918258666992 0.93362283706665 0.570443749427795 2.40542364120483 0.20034983754158 0.860756456851959 5.70314645767212 17.0991020202637 0.557096064090729 4.56620311737061 10.5901727676392 0.295865505933762 5.33284187316895 1.15608954429626 7.67438888549805 0.374065428972244 3.83982563018799 1.49671387672424 0.65350741147995 0.691892147064209 6.2663893699646 0 3.06668782234192 6.2127857208252 0.26879695057869 1.68953490257263 1.69888126850128 0.802521646022797 0.16271510720253 5.29164123535156 0.132682740688324 1.0219064950943 0.451827138662338 0.944739162921906 7.32097053527832 4.60057735443115 1.1026576757431 0.774938881397247 3.43000674247742 1.17300152778625 8.00447845458984 42.0902557373047 0.0342392139136791 0.757181167602539 0.804139077663422 3.17550587654114 2.90261030197144 3.58247184753418 0.0970713421702385 0.126515835523605 0.811202824115753 1.05181443691254 2.22167801856995 0.854036152362823 0.254233360290527 2.41011738777161 0.408417612314224 0.633388340473175 0.257616311311722 0.134739831089973 0.722023785114288 0.533579468727112 0.288523495197296 7.11409997940063 9.96059989929199 0.461519926786423 0.189567908644676 0.0717611238360405 1.98339605331421 0.693740487098694 0.575807809829712 2.46933269500732 0.682574152946472 6.79853057861328 10.5982007980347 2.05820441246033 0.321001052856445 3.41562032699585 0.592430949211121 3.30530500411987 2.27814340591431 1.59105312824249 0.242065161466599 0.302389085292816 0.140323951840401 0.0656571984291077 0.82630443572998 0.0498577654361725 1.0057270526886 0.168157860636711 1.75851786136627 12.3056592941284 0.150358065962791 0.166674092411995 0 0.691090404987335 0.171461671590805 1.43860423564911 1.60587084293365 3.6663339138031 0.243138447403908 0.489448577165604 4.48499965667725 12.1053676605225 1.80678284168243 0.338611781597137 4.27780818939209 4.6753568649292 0.451872736215591 1.74061822891235 1.4843442440033 1.9492906332016 4.74810218811035 0.238936975598335 0.786933779716492 0.644831120967865 4.05947494506836 3.48941898345947 1.10564160346985 2.95960712432861 0.218797102570534 1.18857455253601 1.90140092372894 0.298508614301682 0.28363910317421 1.20178318023682 0.31044015288353 21.2093944549561 0 3.61320614814758 1.51254713535309 0.690371930599213 8.89608478546143 3.96705031394958 1.49620199203491 1.10704243183136 3.74181461334229 2.79726147651672 3.08906650543213 0.505998253822327 0.636819899082184 0.460752755403519 1.14093863964081 0.852377653121948 0.824102401733398 1.31765258312225 2.90215659141541 7.51037216186523 0.669660985469818 0.0285436380654573 2.02089357376099 0.415548652410507 1.59084439277649 2.41911959648132 10.8882761001587 0.629491865634918 0.100275456905365 0.407300293445587 0.194376915693283 5.7816309928894 0.567610621452332 0.366710811853409 5.1058783531189 1.88478195667267 1.33222305774689 1.59059083461761 2.56796860694885 33.7075462341309 0.204220116138458 0.244163244962692 2.57312083244324 1.82308983802795 1.29195535182953 2.56994271278381 0.522332191467285 1.92849862575531 0.0575387068092823 5.03310012817383 0.62490838766098 1.5646984577179 0.117880590260029 0.80472457408905 1.13219606876373 1.45518243312836 7.29919338226318 0.109967842698097 9.36005401611328 0.836030781269073 0.900480985641479 0.476202100515366 0.394069015979767 12.1273288726807 10.5507526397705 0.175272077322006 0.941882193088531 0.750640749931335

ENSG00000158481.11 8.41300678253174 29.8425006866455 2.11311531066895 0.434029668569565 4.91311502456665 44.9346923828125 56.5575332641602 9.75854778289795 17.8125286102295 29.1492042541504 1.32744669914246 30.4988174438477 0.486248731613159 0.608533442020416 11.657301902771 0.104241319000721 0.607641875743866 10.6047325134277 7.38992547988892 11.9137620925903 4.03083181381226 2.58059406280518 0.146575838327408 1.46829545497894 13.9299325942993 50.0518074035645 4.87444400787354 5.61431789398193 3.31329774856567 15.2685451507568 7.9518666267395 16.6507434844971 16.7500476837158 3.08977627754211 7.17388963699341 14.362717628479 37.2616729736328 7.06515026092529 37.7595710754395 25.8807468414307 20.9260101318359 29.8717288970947 3.55103039741516 19.7573280334473 1.30426418781281 16.7129249572754 22.1786403656006 3.50354385375977 5.90374708175659 16.325756072998 2.20049405097961 15.5182695388794 2.73370790481567 7.93746471405029 30.8535404205322 0.210688725113869 3.13683223724365 1.6954493522644 1.82369458675385 1.62070918083191 39.6411666870117 6.65572214126587 4.0974555015564 9.23459339141846 7.32317066192627 8.77251815795898 21.9887561798096 40.9596824645996 4.33253765106201 30.6689167022705 116.676399230957 4.19922876358032 12.4204587936401 13.0284986495972 46.224666595459 1.63637328147888 7.52047824859619 5.49664258956909 7.7293758392334 1.81603610515594 28.1833629608154 1.52871704101562 3.24044060707092 23.0310325622559 2.02510857582092 11.6795711517334 4.00285053253174 3.04259157180786 1.99306154251099 27.7732830047607 0.2579685151577 3.85905933380127 2.37917709350586 7.74904203414917 11.1169118881226 25.9531135559082 4.50207090377808 0.914767324924469 14.0044660568237 6.76580715179443 47.9906005859375 79.2802429199219 1.69752526283264 6.11862373352051 6.6880841255188 28.9347743988037 6.62014532089233 20.2810916900635 0.87288099527359 0.286974936723709 3.37341666221619 4.82033634185791 5.03941631317139 11.2542333602905 3.75663042068481 20.4752292633057 5.21106052398682 3.18128681182861 2.90087628364563 0.864494562149048 4.1821403503418 3.94216680526733 3.39693713188171 55.6121559143066 33.8305625915527 3.71160268783569 2.94854068756104 1.67425966262817 8.2382869720459 3.16450548171997 6.26928281784058 7.37931871414185 12.3862237930298 23.2102642059326 96.0691986083984 10.1218595504761 3.47159290313721 13.4595232009888 4.53221797943115 31.7795810699463 16.7482242584229 17.2543334960938 0.705953121185303 4.35428428649902 1.77336239814758 1.24462699890137 2.28932404518127 1.67214608192444 3.08349275588989 2.56103849411011 5.29609775543213 27.7100791931152 1.02316832542419 3.8076605796814 1.05119383335114 3.30745387077332 0.208352819085121 10.089204788208 8.92061614990234 7.0186071395874 1.0872608423233 3.8064432144165 26.7544345855713 88.3758087158203 10.8898019790649 0.548621892929077 18.963062286377 16.8815517425537 2.55329728126526 5.79700660705566 10.9361839294434 15.2082281112671 26.132251739502 2.78732061386108 5.30998849868774 10.5782079696655 33.4121704101562 15.8449239730835 6.87886238098145 12.0112333297729 1.27618944644928 2.6625440120697 7.19268751144409 1.91523957252502 9.28300476074219 4.50623846054077 1.59515857696533 113.23641204834 0.922803640365601 11.6291885375977 20.8949527740479 3.13193106651306 40.2408409118652 32.0087051391602 8.72697639465332 1.49010109901428 11.3911600112915 21.3422966003418 12.2993574142456 2.27027869224548 1.52386176586151 0.806237101554871 12.2004899978638 47.7911033630371 5.70216655731201 4.45951175689697 15.1457118988037 43.8374786376953 5.33001756668091 0.332975924015045 1.32896840572357 2.52478384971619 5.93856048583984 12.7753839492798 16.1417751312256 2.01941871643066 0.747349143028259 4.4544038772583 1.58725345134735 27.2198009490967 2.88156342506409 3.69857263565063 16.9846630096436 8.57228755950928 8.20222282409668 4.09302520751953 11.1959199905396 132.690017700195 0.992637813091278 1.30546510219574 23.0275554656982 3.4614691734314 15.7889947891235 11.7920074462891 2.16957306861877 25.7777118682861 1.56617546081543 17.052152633667 6.90328788757324 12.3588018417358 4.92757272720337 4.82981014251709 4.19617748260498 13.7653398513794 20.8380966186523 2.11132526397705 64.4987030029297 11.2878723144531 2.31718373298645 0.720110535621643 4.98009824752808 51.5668678283691 27.9811458587646 1.27789831161499 5.75537347793579 10.444073677063

ENSG00000158473.6 3.55608534812927 3.74720048904419 0.296554982662201 2.64598202705383 3.00894522666931 13.3375644683838 1.19781267642975 1.49301779270172 11.5912771224976 3.12077379226685 9.96206283569336 8.40750885009766 1.99900937080383 0.669477641582489 3.17353224754333 1.36108255386353 0.860054433345795 3.56534957885742 2.63935780525208 5.86081647872925 2.01720499992371 1.84202611446381 1.6337708234787 5.3809928894043 2.97344326972961 5.39497947692871 4.39828634262085 1.74991774559021 5.66775417327881 10.3477544784546 5.52174234390259 4.31403398513794 6.75074243545532 2.00896596908569 8.64643669128418 6.16720724105835 6.00742101669312 7.25892210006714 2.90716052055359 6.53790044784546 4.36373805999756 4.79790019989014 5.1454873085022 2.57954668998718 1.79261291027069 4.03180170059204 2.38586473464966 4.91323041915894 8.50807285308838 1.98063600063324 5.6938271522522 9.09467887878418 2.33391737937927 2.5699291229248 4.90392589569092 0.838710427284241 2.24768018722534 1.11587607860565 2.1295325756073 0.688183963298798 5.74537420272827 1.29378771781921 6.00447082519531 1.89327561855316 4.39142274856567 5.00293207168579 4.81805086135864 4.33117961883545 3.75984168052673 11.6395654678345 3.61119198799133 2.82116222381592 2.75163292884827 8.88148403167725 6.35711908340454 3.24256443977356 7.20249271392822 3.02044320106506 2.83244562149048 1.28520584106445 5.60962152481079 2.95999765396118 1.19707882404327 4.7187385559082 4.70170974731445 15.9823398590088 1.99683439731598 2.06213879585266 2.72022390365601 6.11711406707764 2.09492063522339 6.71676063537598 3.17061138153076 6.10550260543823 5.95571041107178 1.46744000911713 5.2632851600647 2.77611517906189 11.1710433959961 1.71889245510101 11.1460609436035 4.69896936416626 1.75960373878479 1.93391227722168 3.52219843864441 8.56907653808594 5.71424436569214 1.53315031528473 1.71296656131744 1.09669530391693 3.99030423164368 4.03158903121948 2.98742771148682 5.00888633728027 1.10190379619598 3.21165299415588 3.44551372528076 1.43798565864563 3.57566976547241 2.4360818862915 2.60784220695496 4.88960552215576 1.80631339550018 21.4782848358154 14.9996023178101 3.97468042373657 2.1127610206604 2.84368991851807 3.90748977661133 4.82413625717163 7.70095062255859 3.19946455955505 8.49960899353027 3.25733232498169 10.9297199249268 3.97309041023254 3.00617933273315 4.51296997070312 3.1578254699707 3.98098039627075 2.77705597877502 4.99137353897095 3.19745206832886 4.31802892684937 4.21392250061035 5.67112922668457 3.0185968875885 1.20395541191101 4.88596057891846 8.85011959075928 9.20167541503906 3.70205664634705 2.56950116157532 3.79261207580566 3.0966010093689 5.33236122131348 4.88357543945312 2.00418043136597 4.16664981842041 5.72763776779175 6.62398481369019 2.39917778968811 13.8218717575073 4.65125513076782 4.85841464996338 1.7821079492569 3.97306394577026 12.5247058868408 4.37168788909912 5.34881448745728 3.62789988517761 7.9844446182251 3.03036189079285 2.92188763618469 4.21327304840088 5.34014177322388 3.43514823913574 6.08367919921875 5.11156797409058 3.73619174957275 4.60610151290894 4.06364107131958 1.51001369953156 2.14401531219482 5.59063673019409 7.12222766876221 2.60865378379822 19.8443851470947 1.616339802742 2.50929713249207 11.7312774658203 3.75215458869934 10.0001792907715 4.05288887023926 1.23521339893341 5.00907468795776 13.5961685180664 3.93634700775146 3.94742727279663 4.41031646728516 1.97151291370392 0.741744518280029 5.43415355682373 5.34902477264404 4.11212062835693 1.84323215484619 24.6842956542969 5.40863752365112 2.17684412002563 4.45371961593628 6.40363836288452 2.18702554702759 3.46723532676697 7.89411115646362 4.57876634597778 1.28622555732727 3.99432754516602 2.3958044052124 0.746190011501312 12.4258861541748 3.826899766922 1.06433415412903 2.66745615005493 4.02201271057129 8.66122341156006 6.77810716629028 3.18004083633423 6.37720441818237 2.87668919563293 2.60784864425659 7.708327293396 0.705506682395935 5.35201787948608 4.96467733383179 1.17605376243591 11.2773942947388 3.31326413154602 10.7684364318848 1.49494636058807 4.66245222091675 3.28448891639709 3.96445536613464 9.31051349639893 10.0844011306763 7.20827484130859 3.47255325317383 4.49447393417358 5.69413805007935 3.47651815414429 5.04524850845337 3.34625101089478 5.25434160232544 9.49321937561035 2.22474193572998 4.54054594039917 4.82206726074219

ENSG00000158488.14 2.74005103111267 17.2173461914062 0.629901051521301 0.121165782213211 2.05149149894714 12.8283557891846 44.6897621154785 3.45213007926941 22.6710624694824 9.73709678649902 0.112784117460251 14.6446409225464 0.259146720170975 0.612601518630981 4.83772134780884 0.0339505858719349 0.26387232542038 4.17763614654541 1.9944543838501 2.41856551170349 0.914278924465179 0.84047943353653 0.143215849995613 0.353141516447067 5.4932918548584 13.1779842376709 2.49160075187683 2.11643743515015 1.66492080688477 5.16882085800171 3.97241878509521 6.94881582260132 5.30125427246094 2.89315867424011 1.29405057430267 9.61845016479492 14.5630054473877 1.31489408016205 20.541711807251 23.2277164459229 9.7825403213501 5.58359432220459 1.59024679660797 5.52573537826538 0.380074113607407 3.99759793281555 20.8005237579346 1.88177609443665 1.4372478723526 12.7654323577881 0.298618286848068 4.14162015914917 1.74280762672424 3.10220503807068 16.3269443511963 0.0686196833848953 1.09826505184174 0.809885561466217 0.989938676357269 0.738993287086487 20.2634773254395 3.63162970542908 2.40813732147217 5.55057191848755 2.30897951126099 2.54944896697998 18.7914619445801 45.305046081543 2.11661076545715 13.5935478210449 66.4524536132812 1.53627121448517 5.8626766204834 5.77519989013672 23.0504264831543 0.450050115585327 3.03935360908508 1.93062269687653 1.37939596176147 0.876250147819519 19.0514183044434 0.438144326210022 2.19520354270935 11.9596824645996 0.255314409732819 5.2304253578186 3.55553817749023 1.19422006607056 0.473964095115662 11.5380191802979 0.042009174823761 1.34397518634796 0.691430926322937 3.92591547966003 3.41766238212585 28.1389770507812 2.50094389915466 0.946374237537384 6.51592350006104 2.52544093132019 21.1882820129395 47.8713607788086 0.715479850769043 2.03773856163025 1.43331205844879 15.4562835693359 4.19443798065186 11.2425146102905 0.507112920284271 0.186931028962135 1.31272733211517 2.31527423858643 1.2931444644928 3.42506122589111 0.804937779903412 4.81069803237915 3.68535256385803 0.668464779853821 0.462200820446014 0.494861543178558 2.00027298927307 1.01363146305084 0.771404206752777 18.1310615539551 16.3912601470947 0.717333734035492 1.2003960609436 0.54529333114624 2.81635212898254 2.12889361381531 2.74678349494934 1.75186610221863 6.57942008972168 8.71253204345703 40.5744743347168 3.18161296844482 1.42286682128906 5.5616512298584 3.01745843887329 8.25735378265381 8.24977016448975 4.3882155418396 0.357658714056015 0.682151257991791 0.710855782032013 0.332607477903366 1.56971597671509 0.536712169647217 1.66561818122864 0.390434622764587 4.54150342941284 10.5217800140381 0.171379521489143 1.00265467166901 0.385161370038986 0.60593181848526 0.407153338193893 3.74145793914795 3.82541108131409 5.50046062469482 1.07773351669312 1.44635057449341 16.6528701782227 32.9947662353516 3.0400493144989 0.536045610904694 7.42218208312988 11.5401573181152 0.661694586277008 2.081946849823 4.50175762176514 3.51313376426697 12.396559715271 0.643031179904938 2.49154019355774 2.39890789985657 15.5947961807251 11.8329668045044 1.44692003726959 9.061203956604 0.277096658945084 1.79978668689728 2.90535020828247 0.907314419746399 3.02340269088745 4.16739225387573 0.842482209205627 40.2484703063965 0.120219998061657 5.78179025650024 5.29302930831909 1.74864971637726 15.6525211334229 9.5457706451416 4.73718023300171 0.701009571552277 7.85649633407593 14.4924755096436 13.6301431655884 0.911941707134247 0.806503653526306 0.58352255821228 5.22404003143311 12.268346786499 1.68831956386566 2.5958297252655 8.07631874084473 20.6968021392822 1.56367599964142 0.0903730615973473 0.790393829345703 0.888086676597595 3.06239342689514 8.11468029022217 9.91121006011963 2.31194686889648 0.148160040378571 1.4185254573822 0.541573047637939 13.5853576660156 0.998407244682312 1.42229449748993 13.943097114563 1.79024350643158 2.13712024688721 2.16252994537354 5.42036008834839 96.3798217773438 0.161647215485573 0.425179839134216 9.03342151641846 1.94810152053833 2.43482613563538 7.25801706314087 0.96219664812088 7.66628551483154 0.546526253223419 6.12175130844116 2.44620203971863 5.69715738296509 1.34361386299133 2.76942205429077 2.55409049987793 6.37934827804565 17.230297088623 1.42751049995422 21.6115303039551 3.67637228965759 0.670834422111511 0.30154424905777 0.819901704788208 26.0472831726074 19.5182838439941 0.443948328495026 2.51350808143616 2.64400243759155

ENSG00000010610.8 39.3697319030762 86.0190582275391 9.87982177734375 16.7351894378662 44.1392021179199 192.545639038086 99.1875 63.4242897033691 146.310882568359 30.9694442749023 29.0949211120605 117.010871887207 23.5158786773682 8.1759033203125 54.0444488525391 12.3050470352173 17.7894020080566 64.8683929443359 32.3079147338867 19.1572799682617 16.8919715881348 46.6979446411133 29.6710529327393 172.566452026367 43.1214141845703 71.8496627807617 98.1572875976562 29.6078548431396 94.8811950683594 160.333114624023 133.608840942383 152.024337768555 90.8483352661133 41.2779159545898 41.6418876647949 114.930305480957 92.49853515625 89.2317962646484 102.087379455566 83.930305480957 107.726890563965 50.5888671875 59.5409736633301 62.0428276062012 34.0462417602539 88.3816680908203 50.8523139953613 63.3762359619141 190.885498046875 86.3430633544922 29.899938583374 206.152450561523 43.5811767578125 52.6061325073242 81.8605041503906 27.2619075775146 7.41549682617188 25.1631622314453 52.2106018066406 51.4570732116699 96.76318359375 78.352897644043 139.563919067383 49.4281349182129 44.8351745605469 48.5379867553711 139.376724243164 108.552116394043 92.2472229003906 145.504943847656 141.845596313477 92.7049331665039 87.2717590332031 90.7243118286133 88.4829483032227 85.5887145996094 87.2294235229492 81.7510147094727 49.3980560302734 18.9331321716309 167.667343139648 75.1308212280273 43.7912673950195 52.0426139831543 44.3453407287598 80.7690124511719 33.8666687011719 25.9299163818359 35.1451835632324 73.8383102416992 13.0045289993286 100.841247558594 31.5516204833984 148.581314086914 92.7794799804688 55.4199256896973 105.175270080566 69.6015625 226.348999023438 19.6969814300537 123.746520996094 148.284912109375 31.0156631469727 33.7358055114746 28.2934265136719 76.5472793579102 105.149398803711 58.0506057739258 42.3410415649414 18.6900024414062 84.8576965332031 77.8134841918945 22.4732646942139 98.1624526977539 7.41348552703857 72.036865234375 69.7881851196289 34.510196685791 40.7691307067871 15.5438842773438 34.403751373291 89.5063552856445 17.8181991577148 180.375015258789 274.271514892578 43.6893119812012 72.2618637084961 68.6964416503906 104.27848815918 33.0391616821289 94.4342346191406 103.195983886719 93.471549987793 47.9405403137207 97.032096862793 124.818542480469 68.6155471801758 91.3049697875977 75.7140502929688 89.913932800293 61.1739196777344 115.477203369141 20.8763370513916 21.9752502441406 90.6575241088867 53.0604782104492 21.7183475494385 14.0377950668335 128.071807861328 34.0549964904785 189.411331176758 79.9922256469727 60.0186538696289 46.598461151123 15.305157661438 55.2109107971191 47.5846824645996 64.307373046875 110.260261535645 40.724681854248 50.3697624206543 48.1209259033203 319.915161132812 125.60026550293 43.9652976989746 32.810173034668 71.4073791503906 161.407043457031 67.2330474853516 161.83039855957 44.9435272216797 43.8308181762695 73.7524566650391 52.2012557983398 61.2495994567871 58.1349182128906 124.326622009277 234.31135559082 122.299201965332 63.9874610900879 19.6688232421875 123.636154174805 64.103889465332 28.2034950256348 88.4941101074219 107.721061706543 49.6091117858887 41.6657676696777 9.23171138763428 47.5444221496582 109.23543548584 58.3253593444824 120.633850097656 89.0541381835938 32.6386184692383 36.755428314209 218.139877319336 68.6317596435547 104.599792480469 77.0448760986328 34.3840408325195 26.5627040863037 109.141143798828 92.4419250488281 89.1680374145508 42.5584831237793 65.2302856445312 171.993408203125 24.4220142364502 44.5534858703613 89.3749542236328 38.7524833679199 85.2826461791992 181.041519165039 110.460731506348 11.9563627243042 20.9180316925049 91.6960601806641 22.7195777893066 202.964096069336 20.886287689209 11.0784406661987 170.957229614258 86.9464111328125 70.8695831298828 109.005676269531 103.770378112793 121.06282043457 20.3424167633057 35.4319725036621 91.4180526733398 17.5841102600098 73.6600036621094 40.5051116943359 52.3945960998535 91.8014602661133 40.3171424865723 116.781181335449 28.8173046112061 56.2893943786621 67.4566116333008 89.2245025634766 45.8350448608398 76.4066009521484 114.660232543945 73.7988204956055 82.2353134155273 70.6594696044922 123.027725219727 53.1641273498535 49.0420036315918 107.068992614746 161.71012878418 72.4230728149414 52.4448051452637 88.6376495361328

ENSG00000153563.14 3.62415528297424 18.854808807373 9.43330764770508 6.831130027771 7.83501529693604 51.4843444824219 2.70917868614197 1.4588862657547 25.0092124938965 1.63967049121857 4.71765995025635 7.87820816040039 13.440749168396 2.18453335762024 19.3410949707031 7.67689657211304 5.58542442321777 4.22093725204468 6.70901012420654 6.38744640350342 1.30746924877167 4.88431262969971 12.299524307251 7.06467580795288 7.01707696914673 2.62605810165405 21.6409568786621 4.07548999786377 5.12129735946655 14.241548538208 25.2148094177246 9.60629272460938 11.3414468765259 16.9288311004639 34.45166015625 8.31618404388428 7.01211786270142 52.1445274353027 10.3841409683228 14.196346282959 16.8304653167725 5.62681531906128 20.4200744628906 2.96079635620117 4.09313344955444 131.260955810547 3.10482120513916 24.1988582611084 39.3256530761719 32.8604736328125 7.92903089523315 36.8736686706543 62.3348655700684 15.6717967987061 24.1672649383545 6.73896646499634 9.39846611022949 14.2603893280029 8.85776615142822 12.5011053085327 27.9954376220703 12.2707271575928 40.219108581543 13.8507137298584 6.99873733520508 16.6490287780762 29.0730628967285 4.75804948806763 20.5942344665527 19.9301223754883 6.68711948394775 4.86101961135864 17.201681137085 8.41563606262207 5.79247808456421 48.1614875793457 23.909330368042 2.87275171279907 10.9753360748291 4.38239622116089 43.7628402709961 12.2422714233398 32.3480491638184 1.62175250053406 3.76622676849365 25.6905536651611 3.53712391853333 2.37212061882019 3.62280654907227 15.9103879928589 5.01695203781128 17.8187389373779 9.7562780380249 16.6824188232422 22.4211311340332 2.04700589179993 26.0510959625244 26.921875 42.8451614379883 1.18575155735016 24.7399749755859 7.30555438995361 3.08873701095581 3.52428364753723 0.262956917285919 17.9756393432617 27.3559703826904 0.910071730613708 47.2963333129883 2.46068787574768 11.5564031600952 31.3590621948242 31.2020893096924 5.42761850357056 1.81524324417114 1.30732333660126 25.9668731689453 2.43141651153564 49.8499755859375 3.78613710403442 1.61680471897125 65.9954376220703 10.4480609893799 15.2570486068726 75.7171401977539 12.127799987793 3.63851451873779 31.2110538482666 11.3052682876587 1.98707723617554 7.07321357727051 5.4241738319397 12.2568445205688 5.06422090530396 9.87082195281982 15.4416551589966 15.002537727356 6.48560619354248 24.2151927947998 7.26531171798706 14.51158618927 10.3558559417725 9.32323837280273 2.42315888404846 12.416296005249 1.83990085124969 6.05846643447876 0.468908578157425 35.6771049499512 12.888786315918 20.1853256225586 12.0897989273071 62.2899436950684 29.3837032318115 16.785493850708 114.19800567627 21.0129623413086 4.67434644699097 20.4016551971436 17.4519100189209 18.8722667694092 6.05935049057007 44.2940559387207 12.5265398025513 15.991925239563 51.8421249389648 30.6423625946045 35.8766937255859 15.3947896957397 63.1924018859863 3.89864468574524 13.6417751312256 14.6935682296753 5.66382265090942 63.757453918457 4.98162651062012 3.71401739120483 31.9012260437012 48.5828170776367 5.31658983230591 1.87579071521759 24.5588779449463 4.80781507492065 4.17107009887695 14.6264591217041 25.395336151123 5.00514268875122 27.3717727661133 9.18283557891846 11.5834722518921 34.1042709350586 20.9362812042236 13.2818803787231 5.97718477249146 4.26662826538086 14.1873064041138 71.8760681152344 4.36026763916016 39.0813941955566 28.2394828796387 13.7273101806641 11.1075134277344 6.65388488769531 2.2550687789917 37.0876083374023 19.2958393096924 7.3880033493042 10.1215763092041 8.14577293395996 38.1200103759766 19.2801551818848 6.10158395767212 16.6594734191895 46.3566246032715 25.0261421203613 2.44062399864197 4.32406854629517 13.7003622055054 4.1038990020752 9.20784568786621 3.58310055732727 3.66004467010498 6.30880308151245 26.162992477417 5.65956497192383 89.9359130859375 54.4567451477051 4.51283931732178 5.70322895050049 2.39006519317627 9.06410884857178 4.72390508651733 10.9462976455688 8.52366638183594 4.35653591156006 18.5692291259766 5.94157600402832 45.0882186889648 3.29298639297485 14.7909545898438 10.7924470901489 16.9633655548096 9.60241317749023 7.00407838821411 28.7291049957275 9.67752552032471 9.89931869506836 5.0814061164856 25.6203727722168 16.5943470001221 12.7069358825684 7.17851400375366 37.5037956237793 9.52047157287598 9.73641777038574 22.2057018280029

ENSG00000172116.20 0.412156969308853 4.79177045822144 3.28113532066345 5.34620189666748 2.90950798988342 17.17653465271 1.34054207801819 28.7754783630371 10.5169944763184 0.895063042640686 2.35489296913147 3.20175123214722 28.3136024475098 1.23508179187775 5.42804718017578 2.52787971496582 7.84852695465088 1.32097375392914 2.02043986320496 2.36130547523499 0.554131269454956 12.6393308639526 4.23155164718628 2.67810034751892 1.91292178630829 0.808371484279633 7.76577615737915 4.74520301818848 1.31181001663208 4.11117506027222 5.38529300689697 3.43276953697205 4.22548770904541 4.75732707977295 32.2022743225098 3.29229283332825 3.80984449386597 13.9215173721313 4.19267225265503 5.13087797164917 6.47835254669189 3.3995189666748 4.78408002853394 1.68533790111542 12.1811656951904 61.0338859558105 1.22774505615234 7.78403186798096 14.4154481887817 13.0421876907349 13.128888130188 11.4904375076294 17.7766819000244 4.63391399383545 8.30691719055176 2.75737738609314 0.694280803203583 10.1373739242554 1.70816469192505 2.70335555076599 9.87228393554688 5.62298774719238 19.3771839141846 7.53641510009766 2.99879860877991 8.2496862411499 9.91086101531982 2.1736204624176 5.58680009841919 8.18821430206299 3.47462248802185 1.90423738956451 7.01205062866211 2.30721735954285 2.87172055244446 13.6614151000977 12.2131795883179 0.871209442615509 3.17901182174683 1.76053893566132 15.9337015151978 3.95432472229004 6.88524055480957 0.557950794696808 6.88990449905396 5.47920513153076 1.14212906360626 1.26125919818878 1.24209940433502 4.72104358673096 2.83000731468201 4.94168853759766 14.9346570968628 7.60061359405518 7.31859016418457 0.730339407920837 8.74731731414795 38.6085624694824 11.3460721969604 0.263358980417252 11.9038209915161 3.27669358253479 1.99869561195374 0.920834302902222 0.133735597133636 4.57568550109863 11.4741249084473 0.433705449104309 14.6747484207153 0.946832537651062 3.81121754646301 9.97079467773438 19.465145111084 2.02398347854614 10.6548891067505 0.548954963684082 6.1895318031311 1.58007299900055 14.347354888916 2.2386040687561 0.517840981483459 21.9229888916016 3.91070079803467 4.04809522628784 30.1857967376709 4.01916742324829 1.22954726219177 10.9021854400635 3.28357338905334 0.639001667499542 3.04523229598999 2.20736360549927 3.51906538009644 2.15026688575745 5.43509197235107 4.75693464279175 4.80468320846558 2.39645433425903 9.65774917602539 3.69354557991028 6.07385206222534 3.88971519470215 2.89854860305786 0.608195066452026 4.09566783905029 1.08101880550385 1.23679685592651 0.205221936106682 10.2093830108643 5.1597580909729 12.3606243133545 3.64215064048767 17.5316371917725 8.29503059387207 8.90187549591064 37.2785873413086 7.89169883728027 1.53805387020111 5.89463424682617 4.39015960693359 5.31330490112305 2.04516768455505 10.1458787918091 3.15114521980286 5.48598480224609 23.3140754699707 9.09217834472656 11.1396503448486 8.34875202178955 16.4280185699463 1.63707041740417 10.4736566543579 7.59888362884521 2.32463645935059 26.6059513092041 1.90018045902252 1.67094016075134 11.8202390670776 11.8049783706665 2.15042924880981 1.14621984958649 7.77361392974854 1.51579427719116 1.83188831806183 7.85409450531006 7.88048458099365 1.89183378219604 16.2760314941406 2.37990736961365 3.72725820541382 11.2601823806763 4.87964057922363 4.86433124542236 3.41423869132996 2.29280805587769 10.9616937637329 34.5620765686035 0.761252343654633 16.4779243469238 10.9527149200439 4.28930997848511 2.82754874229431 1.44500684738159 0.862042427062988 15.7089967727661 6.13585042953491 2.05763339996338 3.73908686637878 2.2865788936615 15.0173358917236 6.58346509933472 12.012092590332 4.90515995025635 12.9667100906372 9.47284412384033 0.376884669065475 2.2013258934021 4.41988563537598 1.48379242420197 3.79052376747131 1.840775847435 1.44082522392273 1.79118311405182 14.1307191848755 2.65872836112976 35.6764831542969 22.3050308227539 1.47932648658752 33.4711723327637 0.913647353649139 3.62484526634216 1.66284215450287 4.25890827178955 2.11565279960632 1.8479311466217 6.6951699256897 1.93776416778564 20.1016311645508 1.08415699005127 4.83039808273315 1.98496603965759 4.2419228553772 3.09803175926208 21.655237197876 11.8933601379395 3.23023200035095 6.38548803329468 1.70323348045349 9.0581636428833 6.65252304077148 3.11769962310791 2.0322802066803 10.0669107437134 2.492267370224 2.7188835144043 7.79459238052368

ENSG00000019582.13 5893.2490234375 3679.2353515625 14225.9140625 457.930603027344 3247.20922851562 6630.94384765625 3155.65502929688 2069.49047851562 5372.19677734375 5343.8427734375 2622.85986328125 7792.58154296875 561.93505859375 1194.50427246094 5294.17626953125 711.591918945312 602.686950683594 3038.94873046875 1032.17663574219 632.617248535156 714.758239746094 4175.4111328125 661.958312988281 4464.1689453125 2935.63696289062 6083.43896484375 4402.84375 2457.77197265625 2162.77172851562 5007.5537109375 4716.19140625 11034.1513671875 3050.11157226562 1229.69470214844 2173.13793945312 11072.4990234375 5442.74658203125 4712.91796875 5262.41064453125 4090.23779296875 2766.26220703125 7057.49658203125 2640.78857421875 2486.93115234375 1534.28759765625 3847.61157226562 5678.91845703125 4897.57958984375 5772.5166015625 8073.8759765625 659.6123046875 6736.69775390625 1590.58618164062 3301.53076171875 8999.30859375 505.994140625 169.790405273438 723.090270996094 1247.07580566406 2218.78149414062 11236.4296875 6573.2587890625 4969.501953125 7931.921875 2658.36645507812 1807.30065917969 7911.9287109375 4390.36328125 3336.93237304688 4056.81665039062 4846.3095703125 4739.21533203125 3936.9326171875 1944.92529296875 8165.16552734375 3598.25610351562 3339.78295898438 1989.57727050781 2008.98803710938 697.792053222656 10175.896484375 1839.24291992188 2891.28588867188 1746.75927734375 895.150817871094 1800.78076171875 1489.02124023438 897.706176757812 4566.71337890625 2644.982421875 266.913269042969 2614.2373046875 1442.99975585938 3877.28833007812 5204.6162109375 2349.26171875 3975.46557617188 2732.7197265625 6615.248046875 740.9599609375 6478.4287109375 8442.310546875 708.054870605469 1861.44799804688 3548.93090820312 6782.3994140625 6318.7939453125 2245.24047851562 6544.095703125 493.272888183594 2818.7236328125 3594.6015625 768.340637207031 3352.09838867188 368.066558837891 6957.0859375 2573.03466796875 1386.74011230469 1712.61706542969 937.271850585938 663.665893554688 4983.43017578125 2736.03784179688 4813.736328125 6913.84619140625 8000.52978515625 1797.421875 5406.48876953125 4419.53515625 2561.9013671875 4567.8056640625 3174.84912109375 2526.68188476562 2156.13623046875 7120.11328125 4417.2314453125 6002.076171875 2496.58178710938 3992.22387695312 9079.2080078125 3419.92895507812 3796.83715820312 1194.92797851562 900.948852539062 2140.97509765625 623.595092773438 571.94873046875 4022.30517578125 8030.0048828125 686.427062988281 9290.1455078125 6782.72509765625 5222.3671875 4065.40747070312 1360.67858886719 2510.5166015625 4172.5302734375 2378.2529296875 3841.56494140625 2727.81005859375 1646.50512695312 2669.435546875 10702.173828125 6919.7802734375 1570.51672363281 3163.974609375 4745.7333984375 6721.5283203125 1972.39453125 6223.4228515625 2869.54296875 3101.05981445312 6673.11279296875 2097.33813476562 11252.0478515625 1520.00354003906 4108.3193359375 6894.86474609375 4131.62060546875 3138.15942382812 485.568756103516 3714.88037109375 3011.68286132812 825.593566894531 4371.1865234375 4249.78955078125 747.408386230469 3427.60913085938 507.334716796875 7867.63818359375 3309.61059570312 2881.3759765625 3478.177734375 4808.57666015625 1572.79931640625 947.741638183594 6782.41162109375 1231.68664550781 6399.45458984375 2975.56811523438 1243.71350097656 976.883239746094 6494.271484375 6755.16748046875 2975.72802734375 2292.119140625 5410.43603515625 7511.5458984375 4462.20654296875 5988.0380859375 2406.64965820312 1698.90344238281 3079.34545898438 8997.6455078125 5077.458984375 393.701812744141 951.526306152344 3236.83276367188 659.426391601562 5573.1865234375 1675.86840820312 2384.880859375 4709.400390625 3919.958984375 9353.50390625 3786.01977539062 9075.9375 5183.509765625 579.392639160156 710.33935546875 2758.326171875 592.957336425781 6727.24755859375 2130.99975585938 1747.63244628906 4975.6494140625 1924.40417480469 9526.23828125 1711.74719238281 2574.11108398438 4840.95751953125 2479.10571289062 1326.49169921875 4013.83447265625 4875.7890625 1466.44287109375 7545.41015625 2463.291015625 4096.42236328125 1382.154296875 2769.744140625 6004.04296875 7034.25634765625 962.237548828125 2313.10571289062 3248.2548828125

ENSG00000118260.13 15.5543384552002 14.7719583511353 11.5297145843506 8.61418533325195 18.719669342041 16.3361396789551 26.4308567047119 11.4404039382935 11.7778701782227 5.50116872787476 9.22060966491699 14.7242517471313 14.2170209884644 11.0103960037231 15.3573398590088 16.794282913208 25.099063873291 15.8806066513062 15.7089014053345 11.3227415084839 9.29340934753418 12.2960538864136 30.6662673950195 14.2729539871216 15.0650882720947 15.5899839401245 17.6392631530762 9.84634208679199 17.0675487518311 24.9062728881836 15.0267009735107 14.1436338424683 21.6639251708984 23.6740989685059 12.1657009124756 22.7829837799072 15.0691175460815 12.0255126953125 15.1658248901367 10.258228302002 17.6488609313965 18.3634662628174 17.9614448547363 12.8119468688965 14.3515644073486 15.6764230728149 12.4785232543945 16.0516109466553 13.2182455062866 13.5104265213013 17.7603950500488 15.4240112304688 11.8987131118774 5.67034816741943 15.1185531616211 17.2246017456055 28.1364040374756 25.8415718078613 13.2512760162354 19.9254684448242 22.0759658813477 12.7082748413086 13.3574695587158 12.1588582992554 15.2543535232544 15.9669198989868 15.9913463592529 19.9951648712158 11.5334587097168 22.5237636566162 27.8952293395996 16.0954837799072 19.671989440918 13.6992073059082 21.7953243255615 22.7584915161133 11.7727422714233 15.4014320373535 13.4643850326538 10.3927984237671 14.1124153137207 14.2321882247925 15.5600023269653 13.8790063858032 14.0981540679932 20.6767311096191 19.9143295288086 7.3017110824585 14.4655675888062 24.3399715423584 12.3330507278442 23.403341293335 17.817325592041 23.166675567627 15.6020336151123 17.1019611358643 11.4936552047729 18.9514617919922 27.0950469970703 19.7328872680664 10.6158113479614 14.1780767440796 26.6479072570801 17.3677444458008 12.0888214111328 16.7588176727295 11.9058895111084 14.4837551116943 5.01705503463745 4.50977754592896 16.8086318969727 21.9718189239502 6.48995733261108 22.2238960266113 20.5274276733398 7.19825983047485 21.2500610351562 12.8114786148071 9.32685375213623 19.285005569458 19.294153213501 7.15191507339478 22.0920124053955 13.9194917678833 30.1522731781006 12.0909233093262 20.8703880310059 11.0091304779053 14.4745082855225 9.65629959106445 24.7812614440918 12.9214448928833 21.4028873443604 15.700457572937 14.3927764892578 10.5922441482544 20.583438873291 17.7990951538086 15.0371189117432 13.5957174301147 29.736795425415 16.9062061309814 22.6766395568848 8.87445831298828 25.4630832672119 13.9563875198364 11.8158731460571 11.8110570907593 15.6820697784424 15.1241970062256 17.4518642425537 18.0600261688232 6.44911241531372 15.8689832687378 13.3319787979126 10.649510383606 11.8782644271851 10.7566108703613 17.4580497741699 13.84694480896 15.798791885376 7.97468900680542 18.1623306274414 11.3487300872803 12.6051321029663 9.6446008682251 12.7889270782471 20.8935890197754 14.3395099639893 13.0178232192993 20.8858680725098 8.49074840545654 9.87620449066162 9.99951267242432 11.9977722167969 8.87208938598633 16.0376815795898 21.9539909362793 16.1405200958252 17.0257587432861 13.207688331604 15.2611675262451 14.928900718689 18.0571670532227 11.4260663986206 12.1896047592163 19.6227569580078 17.7946872711182 8.65067005157471 13.6654453277588 16.341869354248 7.17482089996338 18.7287063598633 19.4148788452148 12.4458780288696 24.1930675506592 16.2828311920166 20.5374641418457 15.1054677963257 11.6704187393188 17.8993072509766 7.04843425750732 20.0237827301025 6.03805351257324 13.4049100875854 13.9783563613892 14.0754795074463 24.1381587982178 9.14664268493652 12.9955530166626 21.3893165588379 12.7365388870239 16.2333908081055 17.0984001159668 12.8559131622314 17.7095642089844 7.82572650909424 15.0205278396606 15.4679174423218 10.0523509979248 8.92200946807861 6.97219753265381 14.9823122024536 11.7341394424438 22.8966369628906 25.8249568939209 19.4888896942139 26.3734397888184 16.7808151245117 11.587776184082 30.3599987030029 13.2555704116821 16.4161224365234 14.5349779129028 11.4218549728394 19.7457122802734 22.9775943756104 13.8960561752319 12.2206010818481 22.6681613922119 15.2679052352905 25.4312877655029 15.8869218826294 17.1726722717285 13.8313655853271 14.1789598464966 16.6981716156006 19.5155353546143 13.7636671066284 16.2963085174561 14.9436960220337 13.5728521347046 22.7369518280029 17.262149810791 18.1332530975342 16.0830879211426

ENSG00000164733.19 688.990661621094 451.086578369141 312.834381103516 619.442626953125 600.084716796875 430.5986328125 554.378540039062 472.219055175781 493.690673828125 180.810455322266 339.234405517578 483.458709716797 285.723358154297 677.847839355469 332.5224609375 558.219848632812 266.381927490234 770.552734375 504.043029785156 162.184494018555 355.070037841797 305.983001708984 236.854904174805 1596.03308105469 340.081665039062 304.620086669922 632.209899902344 266.091522216797 1037.18225097656 1700.7919921875 1667.86791992188 1009.37487792969 685.546142578125 449.409149169922 768.539855957031 435.082885742188 472.390686035156 859.376281738281 502.7626953125 275.076202392578 435.575469970703 394.705169677734 526.920471191406 583.770751953125 270.706604003906 830.351867675781 407.843841552734 1089.94775390625 1562.85217285156 474.692840576172 272.060852050781 1615.15197753906 471.026275634766 405.117492675781 359.966156005859 319.351989746094 104.33016204834 495.164611816406 381.628448486328 1964.85766601562 280.59326171875 447.819274902344 978.248840332031 503.994903564453 673.41259765625 483.862640380859 768.19140625 351.233612060547 462.08349609375 534.206787109375 580.943298339844 907.288940429688 384.167602539062 697.781799316406 510.259216308594 1235.91137695312 986.792724609375 444.492553710938 1724.16027832031 122.013069152832 827.705749511719 1025.17211914062 627.8544921875 596.768676757812 666.195739746094 387.810577392578 293.087036132812 220.188400268555 430.732482910156 434.494842529297 235.950469970703 686.693786621094 701.845336914062 1051.55212402344 1200.22705078125 334.349060058594 837.203674316406 832.568603515625 1458.94409179688 374.490936279297 906.016784667969 838.531127929688 291.192687988281 199.107818603516 412.272521972656 501.813629150391 626.404113769531 443.839263916016 311.797027587891 273.4384765625 580.489990234375 515.742004394531 281.319305419922 497.009979248047 795.263061523438 360.300994873047 665.572143554688 913.235473632812 365.660278320312 447.277893066406 349.176574707031 536.31201171875 271.198699951172 1315.24597167969 1041.09790039062 456.240295410156 809.172546386719 989.935729980469 813.027465820312 365.343353271484 812.284973144531 956.982360839844 757.414245605469 301.556549072266 1301.58642578125 841.746337890625 492.814270019531 249.712539672852 301.341125488281 300.823669433594 348.049438476562 824.726745605469 492.049774169922 321.205230712891 1175.42065429688 554.655578613281 200.030090332031 648.380249023438 550.5419921875 252.033721923828 732.802978515625 409.654449462891 615.694396972656 330.790435791016 247.412445068359 391.423004150391 509.017303466797 576.493530273438 685.809265136719 299.899810791016 312.694091796875 499.377655029297 668.629821777344 902.324462890625 373.512786865234 365.93115234375 767.971618652344 842.8251953125 743.686401367188 1182.44519042969 472.88671875 741.861389160156 809.505676269531 967.014404296875 495.299926757812 287.3125 712.00048828125 1608.12817382812 1126.04638671875 514.341125488281 379.746826171875 852.556030273438 451.693786621094 160.210510253906 501.032318115234 1308.01867675781 376.994567871094 451.364471435547 371.292358398438 256.967346191406 554.082946777344 417.975250244141 1012.734375 388.343566894531 227.723205566406 321.630249023438 1003.08258056641 604.484130859375 561.58984375 653.053894042969 474.403045654297 252.721588134766 434.715881347656 303.94677734375 459.545074462891 645.666320800781 465.974853515625 951.746276855469 220.912826538086 354.361022949219 839.318725585938 426.992919921875 671.408996582031 724.29296875 844.36962890625 152.06689453125 280.477844238281 909.750183105469 331.351806640625 1129.00622558594 429.498901367188 159.32926940918 1392.57568359375 905.853576660156 787.197998046875 1064.87548828125 911.274841308594 489.607604980469 343.279693603516 552.547485351562 743.243713378906 441.800659179688 459.766387939453 521.070678710938 498.316314697266 668.44921875 435.263092041016 570.7509765625 424.217681884766 437.164337158203 520.139770507812 798.825134277344 987.944213867188 301.436096191406 330.66015625 2786.0185546875 702.851135253906 373.219146728516 1196.96166992188 551.259704589844 576.358337402344 416.988250732422 582.106811523438 587.347473144531 865.109680175781 616.728271484375

ENSG00000196188.9 2.25995230674744 1075.35229492188 17.3370018005371 1.33247601985931 164.127777099609 417.365783691406 106.742683410645 29.2529029846191 182.241455078125 0.446167171001434 12.9301433563232 106.732757568359 11.423228263855 804.321411132812 3379.2431640625 25.6497631072998 3.35525012016296 276.437896728516 302.159698486328 0.624457836151123 131.945236206055 29.5213813781738 0.0787481591105461 8.60447406768799 929.498291015625 196.920700073242 30.8437004089355 411.539306640625 14.6983394622803 316.221740722656 216.530868530273 147.193756103516 227.74577331543 121.179138183594 12.6002244949341 572.747497558594 30.1534748077393 159.986999511719 760.43359375 1383.83569335938 50.3580474853516 736.822204589844 57.6154975891113 1359.82360839844 5.03410863876343 11.2393674850464 125.551307678223 1626.77368164062 192.678802490234 55.1496849060059 5.93572854995728 341.160247802734 273.83251953125 187.634414672852 126.238212585449 2.15066576004028 0.547712564468384 28.9053707122803 20.0039138793945 10.4197254180908 156.546905517578 4.87609052658081 76.4354019165039 23.4650497436523 140.256744384766 182.286376953125 613.418640136719 1359.71533203125 93.5430145263672 189.778991699219 251.039108276367 48.9839630126953 266.510437011719 34.9308395385742 217.063079833984 156.487777709961 57.7156677246094 105.500297546387 100.203338623047 11.9609785079956 1504.06237792969 95.4357757568359 1.67129576206207 64.4631729125977 0.491351962089539 8.57892799377441 72.0806655883789 45.3088226318359 9.87493705749512 14.6081218719482 0.970158576965332 87.6323089599609 78.1482620239258 131.310012817383 763.150939941406 181.518539428711 1098.11083984375 0.20236611366272 127.369338989258 128.999649047852 139.142318725586 180.130432128906 31.8841953277588 330.825164794922 43.118236541748 218.302047729492 1037.23071289062 2.77904462814331 19.3159599304199 0.947092533111572 49.1224136352539 418.203002929688 11.310284614563 547.57958984375 54.4663848876953 4.76134824752808 1240.00598144531 347.895233154297 13.2192316055298 35.8612747192383 130.035232543945 20.6777515411377 476.562286376953 215.070587158203 30.8277683258057 5866.5419921875 38.711669921875 366.995697021484 665.349426269531 191.279312133789 58.8028831481934 3464.36279296875 59.1777267456055 25.848331451416 1660.35705566406 126.148696899414 93.3607864379883 116.128532409668 6.99541664123535 57.1565017700195 766.802001953125 29.3609180450439 90.773078918457 378.895385742188 28.3257637023926 13.9908075332642 12.4936389923096 88.2740020751953 84.0697250366211 1.7857723236084 1045.48193359375 120.514465332031 115.295478820801 171.248962402344 54.498950958252 39.953296661377 1.16415476799011 279.421264648438 2919.34375 153.334442138672 178.008193969727 76.4112854003906 412.9560546875 1649.95751953125 41.8167304992676 162.642044067383 1033.76330566406 189.490829467773 51.3600234985352 217.456161499023 70.5466384887695 146.158065795898 849.014465332031 166.502548217773 63.1968154907227 117.620429992676 1097.5400390625 176.081344604492 219.508163452148 1312.55224609375 19.2358779907227 2.88790369033813 7.42633628845215 248.043228149414 46.465950012207 2070.18139648438 508.665466308594 519.184875488281 26.5489139556885 153.695983886719 929.999694824219 37.4586868286133 1295.17944335938 389.792785644531 1379.56030273438 7.07161092758179 99.0758972167969 28.421989440918 624.296264648438 6.70841503143311 286.774475097656 6.97856712341309 94.6540756225586 1567.69567871094 24.9384136199951 105.469772338867 118.705390930176 304.472717285156 558.088623046875 409.364501953125 7.72972774505615 60.7416114807129 77.2700042724609 121.325241088867 340.96435546875 2255.51782226562 0.925229609012604 469.081481933594 6.96416568756104 468.488586425781 443.653259277344 545.0185546875 291.751373291016 92.1271362304688 68.4423904418945 35.2326354980469 658.970947265625 192.510131835938 1.8132084608078 81.6769943237305 270.653472900391 0.357058733701706 298.034057617188 407.981262207031 127.547218322754 329.329406738281 173.064300537109 133.775833129883 3104.84814453125 924.718505859375 185.89924621582 93.4867935180664 18.8852577209473 91.990119934082 54.5771293640137 8.57196521759033 80.8437118530273 149.015213012695 8.1334228515625 31.696590423584 52.3646812438965 1181.50659179688 2391.13403320312 2.15120053291321 308.400451660156 94.1145477294922

ENSG00000135047.13 178.404174804688 76.2774810791016 97.0340805053711 565.445617675781 205.724105834961 131.768341064453 114.081642150879 223.533203125 121.560852050781 53.1888580322266 94.1626586914062 117.018707275391 100.40486907959 173.635787963867 167.405426025391 392.5849609375 103.969276428223 199.473098754883 122.453666687012 154.365859985352 96.9599533081055 171.681137084961 648.433288574219 651.281921386719 92.7978973388672 117.09684753418 131.046249389648 97.8794708251953 193.662673950195 391.108245849609 226.348892211914 207.214141845703 247.37580871582 102.248840332031 663.464782714844 130.275207519531 240.367233276367 304.010833740234 90.2533569335938 75.2094039916992 109.378425598145 133.484100341797 136.061996459961 217.818313598633 96.0914916992188 225.391647338867 164.792633056641 205.70817565918 406.026794433594 164.242752075195 106.435882568359 362.360778808594 124.351348876953 185.304397583008 92.2013092041016 304.22314453125 54.8876037597656 735.649475097656 134.149917602539 643.618896484375 167.94416809082 150.880249023438 399.136413574219 88.9407424926758 123.796897888184 91.8582916259766 193.063980102539 85.9113311767578 125.736526489258 96.0642242431641 163.294036865234 333.011871337891 145.421936035156 1603.83312988281 159.378356933594 259.432952880859 902.392395019531 159.26887512207 295.468322753906 62.0886917114258 194.705184936523 506.388854980469 118.864723205566 82.3947677612305 234.357345581055 110.683807373047 242.572692871094 113.773475646973 138.456665039062 179.252044677734 101.151512145996 309.022613525391 290.885009765625 286.239044189453 199.534057617188 108.562156677246 208.502578735352 134.852569580078 513.472412109375 119.878173828125 269.232147216797 102.398292541504 172.058334350586 177.737350463867 96.0361709594727 162.162414550781 199.464324951172 93.5285263061523 1118.9296875 93.6404418945312 151.059097290039 168.858703613281 2043.98474121094 234.70654296875 39.9025039672852 78.8970413208008 150.051071166992 185.541168212891 199.458557128906 125.376892089844 142.913436889648 112.846977233887 307.58544921875 283.214904785156 230.851684570312 432.017150878906 273.835906982422 325.3681640625 123.674575805664 111.602607727051 257.701477050781 182.300720214844 130.124710083008 166.373764038086 256.423187255859 187.703430175781 267.996551513672 87.1713180541992 82.1308059692383 93.7567749023438 195.435531616211 160.183959960938 265.748870849609 154.755218505859 392.662139892578 232.40544128418 67.500358581543 88.23291015625 133.800476074219 46.0369453430176 280.959777832031 116.333137512207 299.110229492188 205.362854003906 40.6443481445312 103.026916503906 454.949462890625 271.00146484375 175.117691040039 168.943634033203 90.9157867431641 189.885772705078 271.708801269531 114.338775634766 167.219177246094 361.992218017578 112.238227844238 228.122940063477 218.851791381836 652.1015625 102.303161621094 199.979431152344 131.576202392578 399.952331542969 243.151901245117 125.060119628906 153.38200378418 721.054321289062 377.835052490234 175.329391479492 135.883529663086 382.842895507812 168.648086547852 113.309753417969 196.611923217773 202.618606567383 137.904815673828 205.004501342773 961.656921386719 78.3913497924805 169.160751342773 126.138778686523 205.857864379883 69.8381576538086 124.553253173828 125.522148132324 328.953643798828 106.003265380859 167.968505859375 213.747650146484 204.7470703125 333.436798095703 251.43473815918 50.4601631164551 533.224670410156 459.459442138672 118.832733154297 197.802734375 64.5553512573242 113.312385559082 182.96907043457 118.55354309082 119.872062683105 255.812316894531 249.648300170898 40.5295944213867 106.814971923828 342.717468261719 112.381820678711 201.263931274414 105.382217407227 122.471557617188 347.000213623047 483.185302734375 93.0010986328125 206.154174804688 148.517608642578 91.9481964111328 94.0994262695312 318.977813720703 164.33967590332 484.325866699219 267.642913818359 90.9147033691406 820.260070800781 145.00520324707 176.477722167969 147.113067626953 62.8203239440918 381.301666259766 239.124084472656 439.212768554688 101.425254821777 114.837257385254 119.221290588379 256.172332763672 316.218505859375 104.500312805176 513.374267578125 115.458885192871 278.348266601562 145.953567504883 180.025939941406 389.388488769531 71.0433807373047 306.901428222656

ENSG00000163131.9 294.055114746094 147.010299682617 66.1647109985352 81.0633544921875 227.126235961914 284.565734863281 96.617546081543 324.688598632812 159.300247192383 103.668838500977 98.7467727661133 230.206558227539 28.4159832000732 48.6116905212402 245.273239135742 32.7264251708984 66.9235992431641 109.162498474121 87.3562088012695 216.79719543457 111.681770324707 615.868225097656 81.6371612548828 502.270782470703 218.376678466797 129.986236572266 190.187728881836 81.8563461303711 113.996879577637 195.647399902344 268.08349609375 387.585357666016 161.525604248047 177.096221923828 311.566741943359 206.84880065918 354.603271484375 260.190765380859 233.746566772461 333.908660888672 335.976715087891 226.461929321289 172.333374023438 247.897857666016 81.032356262207 226.203033447266 98.8018341064453 203.620590209961 427.326080322266 494.327697753906 60.9932823181152 294.053802490234 124.07275390625 65.4643783569336 187.977645874023 57.6895599365234 10.9393129348755 106.360916137695 84.4273986816406 165.865982055664 256.576324462891 150.940841674805 446.6005859375 263.640838623047 205.530792236328 113.618629455566 230.876815795898 172.437286376953 139.897857666016 192.256256103516 136.778472900391 170.579132080078 216.215087890625 167.086563110352 275.469940185547 217.77522277832 722.780456542969 190.87467956543 262.553680419922 85.0590438842773 245.515686035156 247.056869506836 418.612884521484 76.5283508300781 64.5646743774414 255.931747436523 125.683212280273 162.486282348633 60.3439025878906 174.516708374023 92.0208740234375 253.902801513672 113.634185791016 228.035552978516 160.780914306641 59.8662147521973 225.015930175781 293.490814208984 543.604431152344 48.8037185668945 390.508758544922 208.118286132812 54.2820663452148 56.4920883178711 60.4930191040039 199.445297241211 238.196319580078 81.8195877075195 159.083236694336 39.2245483398438 254.342697143555 150.135711669922 82.2101593017578 147.184906005859 44.2900047302246 77.2555999755859 99.5668258666992 118.876052856445 99.2148056030273 54.7792930603027 265.990203857422 216.208129882812 81.6679153442383 259.613494873047 646.899841308594 173.718536376953 244.348937988281 854.127685546875 277.369873046875 80.5006103515625 243.356689453125 298.918914794922 158.223052978516 170.330932617188 277.302337646484 258.264129638672 322.237640380859 119.759704589844 150.425827026367 183.259414672852 169.545867919922 230.782852172852 76.0607299804688 73.6561126708984 253.046859741211 152.508544921875 89.042121887207 59.3030891418457 252.694961547852 75.5857315063477 313.427764892578 436.479614257812 269.092529296875 153.760391235352 46.5151100158691 132.84831237793 129.523010253906 207.866180419922 492.277618408203 165.037857055664 145.37809753418 136.714950561523 333.424774169922 175.787063598633 167.207595825195 219.590896606445 145.942276000977 430.889739990234 204.014236450195 479.310852050781 314.57373046875 361.453277587891 279.867828369141 154.852905273438 375.765197753906 76.4996795654297 215.464218139648 583.593017578125 404.756591796875 102.513786315918 60.279167175293 197.752014160156 43.9829902648926 124.807662963867 208.101425170898 170.144943237305 140.852600097656 208.44841003418 104.390632629395 159.861267089844 156.834503173828 115.511573791504 195.196426391602 75.5236206054688 169.490524291992 89.7137603759766 499.422698974609 64.4098510742188 214.978790283203 159.001266479492 210.470001220703 99.7569961547852 267.232513427734 79.8026809692383 146.445922851562 142.018524169922 123.34716796875 287.373168945312 144.818984985352 479.195465087891 253.749084472656 126.824417114258 205.882171630859 262.167205810547 226.133743286133 274.844909667969 67.2291412353516 242.549896240234 63.2483100891113 213.494689941406 139.876815795898 108.901947021484 243.195022583008 365.01611328125 420.481170654297 476.044128417969 210.408843994141 217.282653808594 100.364067077637 81.4864501953125 327.532928466797 299.987426757812 246.212646484375 120.559432983398 109.388885498047 188.979644775391 97.2006759643555 246.25114440918 186.203155517578 237.96435546875 254.852310180664 287.008483886719 125.037689208984 147.842376708984 156.723678588867 117.49390411377 327.967620849609 142.41145324707 251.588973999023 140.238037109375 133.159469604492 203.355133056641 239.242263793945 59.506908416748 61.1990280151367 201.169006347656

ENSG00000158869.9 110.434600830078 64.3502044677734 18.1269588470459 72.9160842895508 117.861640930176 187.7724609375 101.298690795898 173.331497192383 247.262771606445 68.1155166625977 62.7172508239746 121.216758728027 52.2469749450684 18.6935787200928 123.709617614746 60.9827919006348 53.1662101745605 156.520965576172 84.0634994506836 52.4831771850586 56.6273880004883 113.653778076172 74.7304840087891 378.359588623047 66.2429504394531 212.474029541016 257.388061523438 64.6899261474609 194.467208862305 312.714080810547 238.877288818359 343.560760498047 188.061080932617 48.4854202270508 254.159591674805 105.940017700195 316.163726806641 362.339385986328 143.069122314453 64.74072265625 110.559310913086 128.373519897461 131.423812866211 150.230041503906 133.811157226562 466.834350585938 112.515609741211 220.55387878418 354.814636230469 200.397903442383 87.4866714477539 471.631774902344 130.566101074219 169.554214477539 118.459739685059 74.8789215087891 18.9696598052979 67.8068313598633 111.665985107422 157.862503051758 128.107772827148 184.256301879883 488.229400634766 131.506378173828 113.360809326172 87.3926696777344 279.203094482422 110.519409179688 153.172515869141 137.38932800293 204.111114501953 252.862808227539 104.020500183105 132.970275878906 175.588134765625 219.338165283203 431.371459960938 116.717277526855 147.763687133789 39.2483711242676 210.532043457031 163.936508178711 198.446395874023 83.0721588134766 139.739212036133 133.927169799805 99.2544403076172 39.6011962890625 130.682678222656 180.762496948242 39.2649383544922 208.680892944336 65.8195953369141 245.5703125 227.426071166992 132.594711303711 265.777435302734 235.496200561523 480.471313476562 26.4801464080811 415.753967285156 194.721710205078 63.9161567687988 43.2122001647949 191.61701965332 217.897644042969 242.381942749023 145.309463500977 171.831756591797 57.1211128234863 145.04035949707 169.820175170898 84.4338226318359 192.967666625977 17.806360244751 218.130996704102 193.263305664062 126.792655944824 132.280227661133 39.3862419128418 33.3912582397461 180.912490844727 79.7001342773438 452.204650878906 337.303985595703 243.830856323242 134.418197631836 340.196868896484 128.113662719727 98.9236221313477 248.585891723633 265.917572021484 188.769989013672 95.0432891845703 438.526397705078 263.414794921875 117.75560760498 83.3644027709961 96.8008193969727 145.130798339844 88.7015914916992 284.935516357422 75.6629409790039 25.1885356903076 154.733367919922 82.4231033325195 77.2448959350586 39.934440612793 222.636291503906 36.2577285766602 401.869842529297 109.158737182617 245.911697387695 131.872451782227 36.772705078125 144.395263671875 141.677322387695 200.521728515625 243.561431884766 98.2748336791992 100.90901184082 114.268974304199 472.753692626953 411.352569580078 52.3664512634277 148.22607421875 175.683456420898 229.757217407227 156.282806396484 494.345764160156 115.878288269043 357.013244628906 238.897857666016 122.141342163086 212.480758666992 70.8597259521484 254.461517333984 576.642150878906 290.767700195312 147.986480712891 85.3819351196289 304.660949707031 141.463424682617 17.4516048431396 152.893707275391 248.939010620117 70.2092437744141 277.299926757812 33.1742630004883 110.225677490234 132.595199584961 172.499877929688 270.86572265625 107.638137817383 33.9206352233887 105.854972839355 424.845123291016 124.871711730957 142.332229614258 175.340393066406 110.79736328125 74.2763061523438 228.924041748047 442.21630859375 147.876800537109 96.185905456543 149.641845703125 333.323364257812 56.5085105895996 91.7805252075195 247.286026000977 49.5352630615234 204.150543212891 323.294097900391 228.270462036133 7.46966600418091 79.374153137207 296.02734375 35.8915786743164 311.084381103516 47.3666343688965 21.690954208374 302.590057373047 565.850402832031 65.1614608764648 266.392211914062 204.497863769531 109.298934936523 51.7909278869629 57.2390518188477 182.67301940918 51.4446105957031 213.399505615234 67.5361785888672 109.94393157959 120.698707580566 118.401405334473 189.511993408203 90.7179412841797 141.658264160156 199.75439453125 168.5 69.9902267456055 111.120819091797 163.386489868164 249.880096435547 272.095642089844 71.9168548583984 462.057525634766 72.43310546875 171.239562988281 146.021759033203 272.793426513672 69.1156539916992 69.02587890625 203.807418823242

ENSG00000104870.11 76.5078125 122.031730651855 96.6940307617188 113.197708129883 85.2552108764648 117.015312194824 367.109069824219 239.501937866211 153.863494873047 293.915771484375 60.8704566955566 225.157165527344 38.2606620788574 242.685485839844 133.967559814453 66.4051284790039 62.9416694641113 111.268684387207 76.0468368530273 131.022216796875 83.1543197631836 167.291656494141 57.1582565307617 152.173156738281 171.100311279297 98.8407897949219 181.147155761719 318.259246826172 127.381332397461 120.61939239502 125.559303283691 247.238647460938 230.398452758789 75.9690246582031 106.799423217773 191.039901733398 403.563354492188 60.377082824707 206.256332397461 146.930374145508 166.019714355469 237.460311889648 82.5476303100586 198.252105712891 101.881866455078 91.0525665283203 521.908142089844 82.2110061645508 191.327621459961 227.235626220703 90.3468322753906 134.83625793457 101.006454467773 64.462028503418 167.302932739258 76.9973754882812 99.1042251586914 51.9087982177734 158.840850830078 52.4628677368164 314.753051757812 107.541618347168 115.444450378418 147.847869873047 221.313247680664 86.0447006225586 152.989791870117 90.2814178466797 69.8186340332031 216.081115722656 258.961151123047 158.011337280273 171.995895385742 72.9142608642578 113.574768066406 97.0364990234375 125.360832214355 182.4755859375 99.2000503540039 151.497528076172 190.841613769531 83.227409362793 64.8976440429688 100.548858642578 163.067321777344 69.8466567993164 89.1830444335938 181.623168945312 123.203170776367 116.411026000977 62.7176284790039 78.5714569091797 124.345458984375 126.386657714844 73.4916839599609 109.438095092773 121.359649658203 125.660552978516 147.29524230957 134.263565063477 197.41438293457 190.643600463867 149.952621459961 178.591644287109 135.78254699707 246.088500976562 110.15608215332 160.582885742188 55.643913269043 42.349609375 116.636894226074 140.511276245117 28.4847450256348 140.972244262695 241.179382324219 128.839172363281 278.386077880859 179.305587768555 70.2157211303711 91.3460540771484 286.411743164062 169.345687866211 25.7202835083008 165.942321777344 172.612487792969 165.082138061523 145.499847412109 54.5993270874023 251.490524291992 149.386901855469 117.417594909668 271.857391357422 181.174118041992 147.765640258789 219.845184326172 122.177864074707 80.7627182006836 186.380752563477 132.733108520508 231.582611083984 137.176864624023 163.806030273438 71.6685256958008 71.840705871582 136.529113769531 57.9444732666016 52.8283882141113 151.470108032227 107.008453369141 59.4554672241211 171.182022094727 133.218215942383 116.460479736328 130.254272460938 56.0522613525391 98.5058975219727 88.8862686157227 177.560211181641 169.408081054688 143.45475769043 91.6341705322266 100.611854553223 151.292877197266 150.112747192383 75.4818496704102 34.9207077026367 125.55721282959 290.609985351562 232.939926147461 98.6169509887695 182.289764404297 154.537048339844 250.810211181641 77.7779769897461 263.405975341797 93.7828598022461 167.753936767578 202.292953491211 113.370780944824 149.06867980957 76.0677795410156 62.1045379638672 58.8642044067383 111.723770141602 126.645584106445 134.929382324219 68.4783935546875 130.323303222656 35.0809669494629 97.7391662597656 113.47452545166 63.1327171325684 224.786529541016 313.513580322266 351.070404052734 69.1756134033203 171.598007202148 19.2404270172119 97.3437271118164 90.7950973510742 32.0308151245117 57.8213233947754 178.688949584961 95.1863479614258 67.5924606323242 97.0199279785156 179.353302001953 143.710845947266 155.83610534668 83.5393829345703 52.9327659606934 140.373153686523 97.1716384887695 170.087615966797 112.365592956543 67.5493240356445 42.3932266235352 193.258117675781 74.9723358154297 147.000991821289 284.874389648438 120.826240539551 130.574310302734 123.504447937012 86.9028930664062 130.928619384766 46.1222343444824 158.901901245117 93.4775009155273 66.0728378295898 139.259521484375 125.926399230957 139.572509765625 121.615753173828 65.1776809692383 127.414123535156 146.953277587891 132.086502075195 147.71257019043 27.4936561584473 127.834770202637 80.4933319091797 42.3665466308594 152.76579284668 164.240814208984 80.8749847412109 257.662719726562 135.220916748047 100.984527587891 58.7236022949219 101.303497314453 134.382537841797 176.916290283203 45.7603797912598 193.078842163086 181.016860961914

ENSG00000167004.11 444.881896972656 357.972747802734 716.260803222656 224.181945800781 259.931518554688 312.720672607422 478.538116455078 181.908096313477 321.815826416016 186.851531982422 530.524047851562 444.422515869141 882.337036132812 1915.59790039062 628.835815429688 1307.04541015625 676.379211425781 414.917266845703 444.177856445312 301.317352294922 158.698852539062 465.047424316406 980.023376464844 423.954284667969 409.54931640625 385.016967773438 434.805267333984 627.260803222656 639.593811035156 441.824523925781 528.599243164062 395.963409423828 327.325408935547 524.491088867188 129.540756225586 342.343109130859 158.760238647461 297.029632568359 275.706695556641 539.775451660156 318.290496826172 271.045501708984 235.902786254883 685.714721679688 357.224609375 174.498641967773 560.505859375 1606.45361328125 316.666046142578 477.909393310547 1192.16247558594 337.645172119141 371.952239990234 562.125549316406 842.90625 736.147521972656 401.339782714844 995.643127441406 331.46630859375 293.603546142578 289.217620849609 300.208190917969 221.370193481445 441.055206298828 758.849487304688 516.779724121094 417.591949462891 357.007354736328 288.534881591797 261.628753662109 424.90380859375 636.425598144531 913.380065917969 1023.48999023438 402.524261474609 377.471221923828 287.130920410156 482.547790527344 308.969512939453 726.60107421875 371.714813232422 1054.05651855469 388.518951416016 346.428131103516 895.952514648438 701.727783203125 496.023681640625 979.221496582031 268.808959960938 392.514007568359 963.550109863281 515.019592285156 389.018798828125 719.897216796875 341.802337646484 558.553649902344 331.314849853516 1583.40637207031 321.398071289062 1231.39514160156 226.360885620117 713.858520507812 1146.76818847656 1385.63513183594 977.564208984375 560.714782714844 446.039703369141 896.56689453125 450.277221679688 517.994201660156 318.566925048828 404.705291748047 449.329376220703 645.494995117188 1304.56384277344 304.907043457031 476.000457763672 941.75244140625 805.817321777344 318.019409179688 1547.23974609375 259.677490234375 482.408294677734 390.591033935547 319.560791015625 569.264709472656 293.345153808594 564.706115722656 700.627685546875 372.639862060547 401.171264648438 612.010864257812 360.664764404297 510.052551269531 282.65673828125 382.012756347656 651.113464355469 745.552001953125 173.330596923828 232.526321411133 607.705383300781 386.659301757812 590.171752929688 542.77392578125 497.020599365234 201.242691040039 451.780212402344 484.917236328125 251.063461303711 420.621490478516 1742.55883789062 539.755126953125 627.635803222656 453.477447509766 1222.64672851562 292.876831054688 569.921508789062 420.860961914062 663.049926757812 390.809173583984 1099.35314941406 519.171020507812 408.10595703125 322.290374755859 461.631866455078 655.478515625 418.713195800781 201.116577148438 702.913635253906 440.114990234375 270.990203857422 275.198516845703 278.595031738281 626.541809082031 648.474792480469 278.029815673828 413.592529296875 436.108612060547 428.250152587891 802.956237792969 699.778930664062 426.553009033203 355.151977539062 861.359802246094 268.605895996094 780.760375976562 884.906677246094 175.582046508789 686.316162109375 308.279296875 288.789672851562 417.001770019531 417.746887207031 515.008972167969 684.763427734375 369.550720214844 334.614135742188 458.545135498047 653.685241699219 329.306671142578 192.54866027832 771.845886230469 365.157684326172 341.947692871094 413.500579833984 894.592834472656 638.43408203125 425.496978759766 594.505126953125 829.236328125 239.361511230469 423.778961181641 278.99462890625 823.425964355469 331.008728027344 503.750640869141 1187.36657714844 395.467437744141 494.398864746094 297.483825683594 1520.01440429688 398.496246337891 434.643249511719 186.299087524414 537.621215820312 581.623291015625 272.616516113281 396.505310058594 983.666625976562 780.967712402344 501.126068115234 1305.10107421875 764.69287109375 322.064422607422 1017.69323730469 354.453155517578 438.436767578125 225.666549682617 489.937286376953 435.939544677734 459.375 534.40478515625 600.568359375 370.19873046875 216.424102783203 766.157348632812 262.285125732422 422.525817871094 280.285766601562 543.087768554688 277.567321777344 517.066162109375 288.295928955078 483.357849121094 551.003173828125 374.017395019531

ENSG00000010704.17 12.3385829925537 7.56763648986816 13.7204971313477 11.8059892654419 22.3622493743896 7.42255592346191 6.05869245529175 10.3148708343506 8.77635383605957 8.79872226715088 4.97656583786011 7.6205883026123 12.0942535400391 14.6601715087891 5.91918277740479 19.9931640625 19.0611877441406 11.0533876419067 10.9266080856323 14.1892156600952 6.44311571121216 16.2234439849854 1.44548308849335 21.6826496124268 11.4048643112183 14.9574127197266 14.9791326522827 6.31897783279419 7.32486915588379 14.378363609314 8.49649524688721 9.37697982788086 6.86769104003906 15.6909351348877 10.367790222168 21.0971202850342 11.1887607574463 5.27448225021362 11.7776327133179 10.0132627487183 15.7173471450806 10.8375844955444 10.3934907913208 12.0192356109619 4.5043830871582 5.69009017944336 18.165584564209 14.8119306564331 9.84672260284424 12.3599872589111 6.1206693649292 6.88063049316406 4.63287734985352 6.65686464309692 11.1270599365234 17.4743576049805 4.63024759292603 28.7240505218506 13.5961122512817 17.0347709655762 17.0268993377686 7.49695014953613 13.632851600647 21.3264980316162 3.96946883201599 6.38173198699951 12.403416633606 19.7893028259277 1.93088984489441 6.29733180999756 9.9601583480835 9.46920776367188 15.585054397583 21.3559112548828 20.3051528930664 14.7029457092285 6.07974052429199 20.5759696960449 13.5742416381836 38.8797035217285 8.4349308013916 37.5423812866211 17.2891044616699 13.2967834472656 4.01401472091675 9.43683338165283 8.9963207244873 12.9213056564331 7.32759952545166 12.1426057815552 3.79969453811646 9.66153526306152 12.8836431503296 15.6175374984741 4.72888708114624 8.23322772979736 10.137261390686 9.52457904815674 8.83618068695068 27.353982925415 11.5439386367798 10.3607654571533 5.13407182693481 11.2373857498169 15.1107311248779 12.8505582809448 4.60119247436523 26.7166137695312 15.5577554702759 1.21288013458252 20.139986038208 10.6252460479736 3.5525496006012 18.8416576385498 8.87420177459717 5.31910753250122 5.79782104492188 13.7012777328491 7.90518856048584 12.3740310668945 32.1172561645508 4.57754755020142 9.35400867462158 10.2179164886475 17.8798389434814 21.3386344909668 9.47505569458008 18.5219573974609 11.8932285308838 2.80721569061279 18.8062362670898 7.98653316497803 13.3483839035034 17.1693916320801 21.1405296325684 5.29746294021606 17.0932025909424 16.7772178649902 13.9423360824585 6.66689872741699 20.4750652313232 23.2576942443848 20.1914348602295 9.31934452056885 24.6630153656006 11.1766176223755 2.56944274902344 11.0669736862183 11.7716073989868 4.03712224960327 26.0294380187988 14.9024076461792 5.66970443725586 14.5754280090332 5.183265209198 5.27935934066772 16.0477924346924 9.09335422515869 14.9064035415649 6.08683490753174 6.76563167572021 12.3722677230835 13.8311128616333 9.13875007629395 14.4787797927856 16.71653175354 9.56909942626953 12.0087270736694 7.13670253753662 10.5145092010498 23.7360172271729 13.3306112289429 14.6343231201172 16.3134498596191 11.9363927841187 6.52859592437744 12.2572898864746 12.8662719726562 9.94726753234863 12.1699962615967 10.7208652496338 6.96125459671021 1.39201760292053 10.7865495681763 4.88059186935425 7.18846273422241 6.2792911529541 13.5222225189209 23.1943225860596 6.87739515304565 6.84907197952271 4.41229772567749 8.90048217773438 6.87488126754761 14.6590127944946 1.71649718284607 9.27055549621582 5.9384388923645 7.01176643371582 5.157066822052 4.74436235427856 16.1961708068848 16.2774543762207 6.35377073287964 22.0331954956055 17.9343948364258 6.55195665359497 19.4203567504883 1.76958668231964 17.4148368835449 2.99520635604858 10.1254291534424 9.2601432800293 23.2558650970459 7.13733673095703 12.8432989120483 2.67854714393616 5.36896276473999 20.9470844268799 8.63297939300537 6.88334083557129 7.7917366027832 10.8840818405151 8.28162479400635 9.67158699035645 8.96979427337646 8.01490020751953 15.2929630279541 8.39601135253906 24.8178462982178 17.3010520935059 7.27531766891479 10.3819065093994 12.609338760376 24.6755847930908 17.1712837219238 10.0845832824707 9.67719841003418 9.49600505828857 10.9858894348145 17.1687622070312 17.2269630432129 4.51385307312012 11.4053068161011 7.38141250610352 33.3098640441895 13.0224485397339 10.5608730316162 9.43348979949951 7.20554637908936 10.2126617431641 15.2391557693481 10.2644357681274 15.9412651062012 7.39238214492798 10.5636978149414

ENSG00000206503.10 888.258239746094 1800.27795410156 2110.73315429688 863.100769042969 1369.70593261719 2102.37158203125 537.558349609375 1649.71997070312 1554.25085449219 2829.72338867188 1115.45715332031 2335.32934570312 1038.27062988281 1243.04919433594 1513.92346191406 2368.32080078125 587.674438476562 1123.21813964844 2236.93676757812 3805.78295898438 1359.25537109375 5059.92333984375 226.984725952148 1601.64587402344 1690.86010742188 2900.66357421875 3148.69409179688 1674.15747070312 1059.54248046875 2694.337890625 1457.81164550781 1580.43798828125 1603.04760742188 1101.36743164062 1689.29089355469 1157.38049316406 1311.9755859375 1462.05090332031 981.815856933594 962.088806152344 2045.61389160156 2762.27905273438 1287.70568847656 537.918701171875 952.4755859375 1804.85510253906 3764.53466796875 2930.60986328125 1708.23767089844 7529.5341796875 811.274963378906 2920.34375 658.328918457031 2587.92236328125 3427.201171875 917.027893066406 617.150634765625 977.168090820312 427.507995605469 1073.92077636719 3719.16748046875 2589.17700195312 2063.28979492188 5186.40478515625 942.854370117188 983.044250488281 2756.02465820312 1761.73510742188 2676.57568359375 1100.90368652344 1139.12512207031 1036.67407226562 2071.87109375 2395.224609375 1831.09606933594 2711.0830078125 1434.80029296875 1514.01721191406 673.892395019531 216.686859130859 2147.80249023438 825.967834472656 2803.2314453125 1071.57482910156 626.417297363281 869.222045898438 1538.42248535156 1831.0517578125 857.5439453125 1213.32800292969 850.639709472656 1453.39904785156 1828.48706054688 1366.87683105469 1246.53527832031 1519.71667480469 1566.14392089844 7150.24853515625 2152.02124023438 1680.23461914062 2977.11206054688 2213.8779296875 257.078033447266 669.789672851562 1155.8857421875 2270.78540039062 4087.03100585938 850.489013671875 2081.02392578125 385.237884521484 3022.685546875 1760.60852050781 761.369689941406 1991.67919921875 499.92041015625 869.305725097656 1049.08312988281 909.334228515625 877.84912109375 3451.30249023438 3251.8564453125 2585.6728515625 4253.55859375 1463.39953613281 2457.0625 1734.3193359375 1317.87756347656 3377.3662109375 2704.31079101562 1136.35144042969 2119.833984375 4263.9287109375 693.666625976562 2231.73217773438 2822.2626953125 3155.68115234375 1597.69152832031 1435.04138183594 2879.470703125 1797.39575195312 1038.05712890625 933.981628417969 3241.36279296875 2398.67797851562 1885.90002441406 422.048858642578 818.179321289062 856.883178710938 3635.01538085938 545.997741699219 4896.0234375 3359.26928710938 2020.40625 5527.912109375 1036.33776855469 2462.08935546875 3073.33618164062 1190.2001953125 4675.4267578125 1069.76989746094 968.948059082031 992.782592773438 2340.28637695312 2266.2451171875 1895.08996582031 3171.60400390625 3394.01220703125 2190.60107421875 1472.03295898438 3063.841796875 2437.98217773438 2869.36108398438 2794.20874023438 691.817504882812 5439.30029296875 1224.58520507812 1271.76477050781 1787.11450195312 1394.08996582031 2883.87377929688 629.065063476562 3911.72705078125 383.037689208984 775.646667480469 1263.92626953125 1604.69689941406 546.843139648438 2101.75341796875 1566.44372558594 2159.66015625 1507.81848144531 2803.48193359375 1541.29772949219 793.586120605469 1236.4404296875 2745.986328125 2373.60913085938 1100.27233886719 1534.65979003906 881.763488769531 2496.69799804688 1573.7177734375 2787.30419921875 515.009338378906 1612.24194335938 2127.24633789062 1034.92883300781 1910.75305175781 1351.99291992188 5795.9541015625 1376.19274902344 1074.53955078125 1845.611328125 5646.60986328125 2258.66137695312 658.541076660156 587.754760742188 2812.35327148438 1049.22290039062 1685.85571289062 1900.84716796875 991.410217285156 1912.58312988281 1617.04675292969 747.792236328125 4956.77783203125 5277.61767578125 1027.58264160156 1312.79321289062 935.697143554688 765.447692871094 1092.52185058594 4010.40844726562 1035.013671875 650.8505859375 3865.2431640625 1034.22338867188 2024.75390625 1933.16748046875 1494.4013671875 3092.65869140625 1217.63195800781 844.381591796875 769.006713867188 1713.41552734375 830.765258789062 1105.78930664062 3411.83325195312 5237.7998046875 521.091613769531 2432.734375 2769.01147460938 1458.22766113281 759.362548828125 1473.33532714844 971.457641601562

ENSG00000234745.8 1804.19519042969 2837.09887695312 2713.25 1179.55334472656 1379.83825683594 3092.361328125 692.106323242188 1246.47021484375 2045.07983398438 2662.1240234375 1436.8037109375 2667.77856445312 437.799682617188 440.549835205078 1973.18811035156 1163.34130859375 723.64697265625 1348.50500488281 1305.38110351562 2920.36401367188 866.065979003906 4089.75634765625 390.679504394531 2457.59033203125 2205.18237304688 2183.43237304688 5283.2294921875 1197.30456542969 1307.84838867188 3084.34521484375 2452.91845703125 2612.47021484375 1832.625 1215.39575195312 2446.09497070312 1827.06469726562 1544.85473632812 3011.42724609375 1416.12072753906 2704.55444335938 2374.60522460938 2421.44262695312 1584.63293457031 653.92529296875 1000.63006591797 2889.57836914062 3564.55615234375 6081.6904296875 3051.7314453125 11015.9443359375 717.373291015625 4186.42333984375 1135.12292480469 3305.93041992188 6353.09326171875 1299.39221191406 798.515869140625 583.28271484375 682.995178222656 1271.912109375 4136.92578125 3192.11669921875 3349.31616210938 6827.76953125 1197.49304199219 1282.3642578125 3684.01049804688 2140.98657226562 2103.20434570312 1567.79943847656 1493.83483886719 1341.92834472656 2598.08642578125 1926.82983398438 2615.20239257812 5433.5244140625 2006.14587402344 1588.96643066406 932.804504394531 336.470489501953 2832.90478515625 1234.47827148438 3502.6845703125 1150.24523925781 641.136901855469 1237.13830566406 2233.44384765625 871.1201171875 874.836547851562 1837.93835449219 411.572540283203 1920.07824707031 1788.74169921875 1966.01000976562 2239.6611328125 1066.16369628906 2293.0859375 6574.24853515625 3128.39575195312 552.562561035156 3424.21337890625 3299.78393554688 417.266357421875 677.986633300781 1897.99743652344 2368.41967773438 5945.70654296875 1216.78833007812 1920.552734375 339.521759033203 3248.50463867188 1910.30505371094 2074.755859375 2355.63330078125 166.07112121582 1399.78894042969 1137.12072753906 519.917846679688 1170.36413574219 2930.59814453125 1091.63220214844 5739.42578125 5783.37060546875 1649.50793457031 3398.15380859375 2222.35766601562 1434.47119140625 4247.1591796875 3091.99609375 1116.28466796875 2904.64965820312 4523.77783203125 835.327270507812 1560.66003417969 2528.37548828125 4234.373046875 2594.64013671875 1257.53991699219 3228.37670898438 3189.85864257812 1586.43322753906 1421.07189941406 4603.05126953125 1744.90063476562 2730.453125 574.4404296875 603.366027832031 1071.26062011719 5896.1669921875 935.543029785156 7978.8603515625 4714.08984375 4315.11572265625 7142.26318359375 929.423950195312 2680.19946289062 3580.05322265625 1715.61450195312 6892.376953125 1612.314453125 1220.68090820312 1108.630859375 4172.65478515625 1979.27429199219 1676.4111328125 4798.048828125 5121.427734375 2983.09716796875 2097.67651367188 4895.94580078125 3920.13598632812 6840.74560546875 3844.5517578125 636.921142578125 6369.47802734375 909.096313476562 1445.04064941406 3501.80297851562 2388.59106445312 2536.57739257812 351.430877685547 6019.07763671875 383.092712402344 742.642456054688 2056.87451171875 2388.62084960938 782.037536621094 2553.06396484375 1134.62451171875 3837.44018554688 2241.70336914062 2521.74975585938 2191.90014648438 1030.20568847656 638.152038574219 1879.17236328125 3178.77685546875 1033.05920410156 2456.16528320312 1476.36206054688 2318.353515625 998.183288574219 5185.5751953125 724.3935546875 2179.77954101562 2014.13122558594 1062.00122070312 3109.53369140625 692.859680175781 6520.060546875 2192.18334960938 1254.81689453125 2846.7119140625 8756.5107421875 3381.375 573.182495117188 565.574523925781 4611.66064453125 1408.42578125 2319.2392578125 1458.96655273438 939.720642089844 2722.4833984375 2435.21508789062 2178.82641601562 5903.54248046875 6379.62646484375 1296.771484375 603.469665527344 780.672424316406 1609.27917480469 783.116943359375 4803.0537109375 1431.52844238281 1112.15759277344 4053.99145507812 1001.86547851562 3802.31689453125 2001.97717285156 1811.62451171875 3430.873046875 1783.04223632812 1725.48449707031 1111.31469726562 2672.64453125 962.181823730469 1724.53796386719 3991.74438476562 5172.60400390625 1311.91552734375 2446.39135742188 4174.9248046875 2938.900390625 1089.35217285156 2010.64794921875 2107.54223632812

ENSG00000204525.13 1247.89392089844 2173.39770507812 1034.75793457031 1670.81457519531 1030.49475097656 2332.60424804688 905.62451171875 1475.06774902344 1639.14501953125 1714.35168457031 739.113220214844 2068.35229492188 299.717041015625 1412.37353515625 1314.82739257812 898.357543945312 520.426025390625 1081.99353027344 1413.18566894531 1699.15576171875 911.978820800781 4063.18505859375 420.746459960938 2447.20703125 1382.2578125 1461.51220703125 2498.38403320312 1389.03393554688 1334.7626953125 2593.22827148438 1949.44592285156 1531.19360351562 2278.36401367188 1602.72521972656 1574.02136230469 1427.1337890625 1322.31018066406 1941.41088867188 1096.39184570312 2608.39208984375 2530.53076171875 2064.62719726562 1128.39099121094 604.195617675781 599.955017089844 2897.90551757812 1997.20007324219 2299.025390625 1790.84924316406 5609.689453125 992.277770996094 3109.34545898438 909.859985351562 2681.50756835938 4043.83251953125 1459.12719726562 973.635131835938 458.593505859375 458.618072509766 785.902099609375 3038.11767578125 2993.89697265625 2728.16943359375 5096.9677734375 1760.55895996094 1375.62927246094 2458.47143554688 1378.77502441406 1891.3896484375 1272.98376464844 985.355590820312 1538.6953125 1473.01684570312 1356.40344238281 1837.29162597656 3625.41259765625 2501.49536132812 1938.24780273438 966.927795410156 178.10285949707 1960.27587890625 1000.73150634766 2605.09497070312 1445.97277832031 1061.03845214844 883.217651367188 1776.20703125 1401.75402832031 680.277770996094 1164.97937011719 836.422912597656 1411.99633789062 2284.9443359375 1317.56420898438 1076.2822265625 1482.21960449219 2085.52465820312 5150.6533203125 2146.80688476562 1168.1533203125 2118.28271484375 2465.87646484375 495.5693359375 527.577941894531 1191.23803710938 1253.46008300781 3154.57788085938 623.197814941406 1010.20416259766 231.913513183594 2180.22583007812 1811.85339355469 1592.16284179688 2622.90673828125 396.293395996094 879.919860839844 879.852416992188 304.756469726562 679.396911621094 2988.0166015625 1159.49938964844 2380.64501953125 3906.0078125 1354.53332519531 1935.46411132812 1570.65454101562 1345.40246582031 2279.37841796875 2636.71850585938 694.3115234375 1603.57250976562 3723.2626953125 830.874694824219 1348.84826660156 2363.4775390625 3087.03833007812 2670.16333007812 1120.12866210938 2005.34606933594 2058.80395507812 1091.91833496094 1183.34558105469 3564.72534179688 1942.76184082031 2645.29516601562 294.218505859375 433.372772216797 557.71826171875 4318.1396484375 610.237854003906 4632.7666015625 3673.56396484375 2988.26049804688 4116.73828125 577.747985839844 2068.26440429688 4119.12890625 1115.63916015625 3614.21044921875 959.130798339844 1664.78137207031 1007.40112304688 2450.09252929688 1719.58984375 1076.30688476562 3186.51977539062 2476.13232421875 2231.69604492188 1518.21960449219 3274.81616210938 2257.306640625 4136.50537109375 2740.9736328125 800.136413574219 3993.33178710938 815.0498046875 1259.29833984375 2159.22241210938 1817.29077148438 1735.11682128906 270.308837890625 2913.91357421875 323.595336914062 491.667236328125 1440.12377929688 1209.81604003906 639.719055175781 2244.8955078125 1922.74877929688 2627.7353515625 1366.96264648438 1756.16455078125 1264.7041015625 1251.66296386719 1027.66357421875 2159.92822265625 2621.025390625 684.592895507812 1687.9736328125 660.5625 1037.93493652344 1218.06787109375 3029.72827148438 318.381256103516 1512.97009277344 1459.63171386719 1132.75988769531 2010.25915527344 836.129455566406 6232.9169921875 1346.20788574219 800.176086425781 1680.78857421875 6465.0009765625 2199.51684570312 612.880920410156 604.398132324219 2741.33813476562 1091.39733886719 1738.45861816406 982.809814453125 806.853759765625 1851.27722167969 3179.1279296875 1152.73962402344 5480.7109375 3571.61303710938 895.4248046875 903.391845703125 689.640991210938 1179.90563964844 588.187133789062 2205.04248046875 970.157653808594 663.946228027344 3414.63208007812 670.577270507812 2873.52270507812 1150.67846679688 941.073486328125 2456.75561523438 1474.83251953125 1109.46875 965.596313476562 2120.77392578125 1029.92712402344 1959.07421875 3475.05444335938 2671.46997070312 1336.03686523438 2410.77709960938 3077.7373046875 2153.92529296875 737.245544433594 1200.216796875 1511.90576171875

ENSG00000204257.13 424.106842041016 135.439483642578 391.630249023438 28.9681396484375 256.648315429688 231.453216552734 151.46452331543 173.564651489258 264.426940917969 371.199127197266 157.471282958984 336.895263671875 36.197566986084 119.172904968262 332.151824951172 63.6066398620605 54.4835395812988 156.141494750977 102.210716247559 58.1061058044434 59.3693733215332 351.488586425781 26.2712898254395 186.535919189453 175.977462768555 198.608001708984 180.115432739258 182.861618041992 91.8322143554688 219.507415771484 206.500625610352 511.834503173828 211.495040893555 79.1390609741211 145.689712524414 415.178283691406 461.589263916016 262.004821777344 362.721038818359 201.412872314453 154.771682739258 592.974487304688 108.917678833008 112.458793640137 84.7060623168945 189.311904907227 672.365417480469 624.210327148438 295.086853027344 434.600067138672 45.5099334716797 287.867065429688 180.040054321289 96.0051498413086 483.050476074219 50.2902946472168 10.9025497436523 43.845947265625 64.5299911499023 156.761077880859 546.887451171875 327.497772216797 333.506988525391 332.872253417969 311.980987548828 131.686569213867 259.819854736328 312.552368164062 178.186096191406 192.563369750977 309.667449951172 235.716751098633 231.234939575195 107.417320251465 452.373107910156 185.582214355469 260.939086914062 124.295951843262 110.597541809082 46.4692687988281 449.607696533203 105.651229858398 102.579635620117 109.219009399414 52.8275108337402 79.3694686889648 102.608703613281 79.347038269043 118.599662780762 141.809982299805 54.1252899169922 131.166015625 77.212028503418 231.278060913086 211.575485229492 136.457611083984 148.258178710938 227.933746337891 307.522552490234 85.7344284057617 377.017028808594 354.092407226562 68.0265197753906 149.488555908203 405.503173828125 329.827758789062 253.16862487793 240.260025024414 374.874816894531 33.6170654296875 168.533477783203 192.019882202148 57.3480529785156 194.684616088867 23.373119354248 306.721801757812 148.478210449219 132.327301025391 99.6529846191406 71.4463500976562 121.580253601074 554.321350097656 103.578689575195 251.588516235352 293.804107666016 537.410034179688 91.6277313232422 261.881530761719 262.449554443359 247.479751586914 184.572525024414 168.842498779297 138.490631103516 250.380798339844 313.778503417969 165.611267089844 398.029022216797 121.483612060547 371.942474365234 693.148132324219 246.831924438477 191.237182617188 66.4079208374023 105.250106811523 134.492538452148 47.8380889892578 45.7165832519531 648.580871582031 344.593109130859 44.5276565551758 402.002868652344 409.450866699219 222.960250854492 328.992065429688 135.539947509766 120.340614318848 276.473846435547 161.561950683594 205.94221496582 170.101547241211 101.057418823242 153.585266113281 274.232971191406 295.040679931641 98.6131973266602 122.813247680664 270.070159912109 425.209686279297 98.9761047363281 257.090362548828 163.903244018555 281.677581787109 441.667327880859 126.013328552246 538.565551757812 85.2192840576172 272.16064453125 332.454284667969 183.818374633789 218.769424438477 38.9858589172363 131.974670410156 79.3934020996094 37.1063652038574 218.423675537109 179.072326660156 58.5215034484863 249.904479980469 66.3418045043945 419.569305419922 163.686752319336 168.957229614258 174.770706176758 321.96337890625 83.4268798828125 61.5066871643066 296.004730224609 79.3763275146484 356.468688964844 152.778686523438 56.6857109069824 44.946849822998 536.4228515625 246.214141845703 141.227630615234 104.536033630371 314.363555908203 353.886199951172 209.69677734375 424.755523681641 100.422027587891 107.805488586426 126.742958068848 504.015991210938 252.528030395508 37.6346244812012 50.0138626098633 155.499420166016 116.594863891602 197.995147705078 138.705078125 188.895751953125 171.945114135742 216.876312255859 361.887023925781 174.922164916992 248.16862487793 228.060974121094 44.85791015625 44.4241714477539 178.316925048828 35.8799438476562 342.308807373047 171.84001159668 80.0698013305664 281.340881347656 120.209693908691 342.260803222656 115.090400695801 215.023712158203 403.450958251953 127.047950744629 61.1602020263672 154.18034362793 266.199737548828 100.827262878418 507.641540527344 169.984130859375 187.894943237305 59.7423210144043 137.354080200195 364.645690917969 410.478485107422 46.2185020446777 124.111083984375 296.084655761719

ENSG00000242574.7 108.313293457031 43.2905387878418 72.5495681762695 23.3574142456055 61.4953918457031 89.2602157592773 58.2742195129395 122.412414550781 80.7441787719727 83.4070739746094 25.4423694610596 90.9159164428711 11.4057397842407 10.3858308792114 61.439037322998 10.9739456176758 30.1790409088135 40.0273056030273 22.0641899108887 18.4230365753174 26.2052783966064 71.8163909912109 16.4925365447998 101.29914855957 35.746223449707 61.1785278320312 66.3982696533203 48.4015083312988 42.6650276184082 82.937858581543 115.286102294922 149.846923828125 58.1118011474609 24.9829502105713 186.344375610352 116.858909606934 170.756683349609 75.2439804077148 81.25732421875 54.1233024597168 60.0474700927734 163.64958190918 45.100757598877 32.4860343933105 21.5740356445312 119.37890625 68.1415786743164 106.042861938477 112.432586669922 113.536491394043 10.7405071258545 115.54271697998 24.9947738647461 18.4740028381348 108.192535400391 10.7166109085083 2.49000787734985 22.9272346496582 29.0278244018555 39.3032112121582 110.733436584473 71.918327331543 158.318054199219 60.3316459655762 44.0216178894043 41.2719306945801 87.5554733276367 81.558479309082 70.8365859985352 92.348518371582 102.464859008789 55.4683952331543 46.4901695251465 54.9194297790527 117.587287902832 88.4349670410156 300.064636230469 48.208869934082 43.8848457336426 12.7724342346191 112.820930480957 38.1342468261719 29.4884967803955 43.7655029296875 19.8964595794678 29.7601318359375 21.469202041626 14.5020236968994 34.3285179138184 51.2841987609863 10.6562337875366 63.6513557434082 24.485294342041 82.8648986816406 67.6115341186523 29.9656352996826 76.590446472168 193.366271972656 149.353408813477 11.541654586792 178.743957519531 97.3742218017578 34.1990165710449 20.960844039917 68.8228530883789 96.6684188842773 79.8364028930664 55.4949798583984 58.1947898864746 8.17678260803223 54.791919708252 55.2660179138184 30.8145294189453 73.3966598510742 5.27221584320068 48.3285217285156 45.4692497253418 38.247200012207 53.8097076416016 18.6468067169189 10.9269104003906 110.796020507812 24.6902980804443 109.979782104492 178.877136230469 84.480598449707 33.0630073547363 55.5694694519043 77.4674987792969 38.5891799926758 54.3668937683105 92.2332916259766 58.2064895629883 46.7139434814453 161.874176025391 66.7956008911133 107.127258300781 30.3602733612061 103.566635131836 109.025749206543 58.3929481506348 63.9869995117188 20.1665496826172 16.4927101135254 54.8147392272949 31.6488456726074 17.7670993804932 131.236587524414 149.460525512695 13.2366065979004 187.587310791016 89.6306457519531 51.817813873291 67.1183242797852 17.438060760498 38.8400459289551 63.6124649047852 61.880786895752 108.990264892578 44.9629554748535 28.3874969482422 32.5378150939941 175.093673706055 83.2509155273438 32.2396392822266 37.0550765991211 52.688720703125 153.577758789062 42.7915191650391 123.972770690918 38.4188270568848 133.682556152344 166.260467529297 34.2804641723633 150.512832641602 26.6794128417969 64.253288269043 138.073852539062 72.8718719482422 42.167854309082 7.0376992225647 59.1429214477539 17.7879047393799 9.01720428466797 54.2302169799805 60.6591644287109 17.8222732543945 227.120468139648 7.72344970703125 85.4678421020508 71.1883087158203 44.8489608764648 83.8940887451172 63.4343528747559 15.5651388168335 27.1019134521484 188.930816650391 29.5683193206787 87.7468643188477 59.441478729248 32.9126892089844 14.7742786407471 119.801383972168 32.1645584106445 60.7970085144043 19.6119346618652 59.4837036132812 132.57829284668 27.3304500579834 124.903953552246 58.584529876709 22.5244884490967 45.6336135864258 158.785034179688 78.6104888916016 8.77197933197021 14.4842548370361 47.0079116821289 20.119701385498 87.77685546875 18.3708305358887 25.6673698425293 56.8534507751465 194.008071899414 92.210578918457 81.5377044677734 90.5519256591797 70.9904098510742 6.04352283477783 12.7316312789917 77.0313262939453 9.44996738433838 110.426498413086 40.8027954101562 21.7009372711182 73.1721267700195 29.1063041687012 137.285919189453 39.9228057861328 55.272216796875 124.365852355957 60.3419380187988 19.8062877655029 48.338436126709 82.2077026367188 23.6945858001709 341.065093994141 46.2245979309082 107.917839050293 20.660831451416 39.3420791625977 101.011093139648 108.132530212402 16.9918842315674 30.0809745788574 118.773773193359

ENSG00000204252.11 104.471832275391 50.0761871337891 90.9665298461914 6.07663154602051 66.5073471069336 106.575218200684 28.3509540557861 13.3783779144287 68.3213195800781 50.1695518493652 21.3245849609375 119.306091308594 8.79086399078369 4.25423192977905 70.3106918334961 12.4530954360962 8.79017543792725 31.0689544677734 11.0092430114746 6.0166015625 4.20935344696045 28.5658493041992 13.4474744796753 50.3441352844238 18.3406772613525 22.9350776672363 62.8135795593262 26.472900390625 27.9342727661133 60.3826560974121 157.072525024414 169.845275878906 66.3760452270508 19.8465023040771 37.1665840148926 233.512329101562 40.4618835449219 78.518798828125 63.1133079528809 55.6808815002441 47.6964263916016 100.128356933594 42.2950553894043 20.7695617675781 18.9548435211182 30.1376419067383 147.353546142578 122.510620117188 95.2581634521484 138.638153076172 7.37874460220337 73.9684829711914 9.42417621612549 26.7816905975342 131.180923461914 4.46499156951904 1.4024658203125 11.4209699630737 18.2968349456787 26.02809715271 130.398986816406 114.516067504883 111.669479370117 31.9668159484863 27.8652076721191 29.5042915344238 89.118408203125 143.573287963867 59.5646324157715 66.6937255859375 60.2772598266602 42.7134819030762 74.01611328125 43.6074142456055 125.759155273438 68.5053024291992 58.1372261047363 42.481502532959 25.8472995758057 7.19321203231812 135.969161987305 43.0649337768555 21.1407241821289 12.2124900817871 9.25279235839844 26.7948341369629 12.3448934555054 6.80863189697266 18.760986328125 38.5481033325195 4.42893123626709 61.7443580627441 41.1349105834961 99.902717590332 53.9768447875977 12.5790843963623 60.0964736938477 36.7801399230957 179.229476928711 11.6648168563843 65.387565612793 83.6086807250977 8.04792213439941 10.5969038009644 6.25486087799072 97.3955001831055 79.0973205566406 18.1168823242188 17.011194229126 5.99736166000366 39.1437721252441 38.8537292480469 7.8731837272644 66.3940200805664 1.37901973724365 33.6481018066406 27.0350799560547 21.3259429931641 31.8438987731934 16.3693790435791 9.801513671875 102.467796325684 18.7266998291016 75.8032073974609 158.695007324219 33.0867500305176 32.7153472900391 32.0880584716797 79.3295516967773 17.9614887237549 49.9329528808594 42.3657455444336 38.342903137207 20.4026718139648 46.6734924316406 46.2016372680664 68.182373046875 28.5668563842773 56.1821632385254 102.38013458252 73.0488815307617 50.047981262207 18.2082843780518 9.8374195098877 38.8869094848633 26.5164127349854 11.3919105529785 127.852172851562 309.007690429688 9.56953144073486 128.823867797852 102.738639831543 33.4909133911133 57.3017463684082 7.40225982666016 21.2385482788086 93.362419128418 47.4308700561523 93.3811264038086 44.7948455810547 18.5482749938965 39.5821075439453 139.997451782227 75.947151184082 40.3324203491211 10.7533712387085 56.4030952453613 97.4562835693359 32.2880058288574 117.200691223145 15.9996175765991 19.2907123565674 74.7752304077148 37.3029327392578 114.100494384766 15.1343259811401 61.2123756408691 136.926712036133 85.2173538208008 37.4763450622559 5.32540798187256 45.1728134155273 14.2574138641357 7.57692527770996 78.8328704833984 72.5100860595703 12.1512031555176 36.4050254821777 6.70033168792725 112.187461853027 67.124397277832 32.8064346313477 38.9564361572266 58.0255279541016 24.5255870819092 16.2557315826416 104.324043273926 21.3977947235107 158.062576293945 51.9290924072266 30.3865509033203 12.9447174072266 113.612258911133 20.2177581787109 42.7459335327148 21.533374786377 77.5679016113281 167.33186340332 33.52978515625 41.0490531921387 35.5059356689453 22.9376487731934 42.9463691711426 176.549423217773 76.1129379272461 12.0383272171021 10.2391185760498 26.2530136108398 12.1519975662231 84.8318786621094 6.68838310241699 14.7590360641479 37.4487686157227 46.4597549438477 125.440811157227 51.1091918945312 157.529266357422 68.1516342163086 4.40254211425781 9.16890144348145 64.036247253418 6.04186820983887 57.8747367858887 33.2056846618652 26.7474517822266 79.7398376464844 31.4303760528564 138.253448486328 25.49147605896 76.5759429931641 44.9637184143066 63.2974967956543 21.3198184967041 64.7261276245117 77.8902740478516 18.047664642334 167.089279174805 45.1705627441406 47.0576057434082 16.6498641967773 32.7087516784668 61.5989685058594 160.098129272461 8.8672513961792 40.2121887207031 46.556396484375

ENSG00000241106.5 30.7162437438965 23.4435482025146 9.84753131866455 6.84799766540527 7.34590721130371 44.9090995788574 8.90304565429688 2.47949457168579 16.2551822662354 7.76088333129883 27.3693084716797 42.6416625976562 9.59352684020996 3.11903882026672 15.9736890792847 4.62928533554077 1.98150408267975 5.88753938674927 6.77432823181152 9.89963722229004 1.69555807113647 18.315767288208 0.622631728649139 20.4472045898438 13.2592296600342 12.1106643676758 13.7254972457886 17.4183597564697 3.50945639610291 14.0579891204834 33.7090492248535 12.3368482589722 6.10288906097412 9.39623260498047 22.119987487793 52.2235984802246 20.8194637298584 19.8291435241699 13.5800561904907 32.425163269043 21.8176174163818 46.5719108581543 11.4845876693726 3.69858193397522 7.26337909698486 9.44996166229248 22.2280654907227 46.071720123291 12.711820602417 52.5123443603516 13.1004705429077 14.8567972183228 3.99807024002075 8.78689098358154 65.4111938476562 7.32251024246216 0.545104801654816 2.57526421546936 3.73253774642944 7.84418630599976 24.8545551300049 30.7759571075439 24.9599857330322 26.4167156219482 8.09281826019287 17.2614555358887 30.5906391143799 19.595043182373 22.2102336883545 27.2263507843018 15.6430807113647 5.24245643615723 11.7059545516968 5.65807723999023 21.5295886993408 13.4527654647827 26.1163082122803 28.1768321990967 7.48253631591797 1.19479870796204 28.7392711639404 8.45368957519531 4.20455026626587 10.1999359130859 7.66895914077759 20.8600273132324 4.75622272491455 4.45878982543945 2.46778726577759 6.32157611846924 2.68971753120422 11.5383319854736 7.12393760681152 6.69418001174927 17.533073425293 7.53632116317749 26.326696395874 8.31185436248779 21.632303237915 2.73016428947449 37.9160385131836 15.4721069335938 2.57070517539978 2.55823731422424 1.47584199905396 22.5964584350586 38.2774620056152 11.1545343399048 19.9331912994385 1.17153108119965 13.3654184341431 13.4626760482788 6.79015111923218 11.4884643554688 0.26723238825798 3.9731068611145 4.36966180801392 3.40812706947327 9.96114063262939 4.41073513031006 12.7167797088623 37.657844543457 6.33028078079224 8.43133735656738 28.9906978607178 6.45773363113403 5.12384366989136 21.443754196167 13.6279640197754 3.76625800132751 14.6700820922852 7.98247098922729 10.7051725387573 6.47163200378418 20.036506652832 15.4531488418579 9.27888298034668 8.05977058410645 10.1143989562988 42.7999153137207 11.3317089080811 5.81359529495239 15.6300096511841 12.8133401870728 3.10800957679749 1.75000441074371 3.25707602500916 18.9226722717285 33.4957847595215 16.5813789367676 15.8436965942383 18.8959484100342 8.01518535614014 20.1475601196289 12.7362842559814 14.2092123031616 33.5032005310059 6.36496019363403 8.72696876525879 13.4671058654785 8.72589492797852 5.0528712272644 24.4899673461914 17.2076454162598 13.3329076766968 15.4234199523926 14.1961984634399 11.9994678497314 8.12125968933105 17.2313060760498 3.81261467933655 5.96674680709839 12.3400726318359 7.08612823486328 106.037628173828 15.5228643417358 12.1915349960327 14.4093456268311 20.9191207885742 11.4988012313843 7.33759260177612 17.6150131225586 3.56896424293518 1.70333063602448 14.7651119232178 8.89417457580566 7.22550201416016 34.0221061706543 3.84865713119507 48.390869140625 31.6183700561523 8.12060165405273 7.98551893234253 11.6409339904785 4.33295774459839 4.34768724441528 31.0510597229004 8.40081691741943 28.941822052002 15.0209465026855 3.23656582832336 2.69830513000488 17.363130569458 7.1261682510376 25.0044612884521 3.18775486946106 5.04601335525513 27.4439811706543 15.3349847793579 53.4697799682617 9.81783485412598 3.27596783638 8.36092185974121 32.4478225708008 28.4421768188477 1.32335603237152 5.24503231048584 6.38365936279297 20.1980419158936 18.2963180541992 2.50964546203613 7.21018314361572 10.4224109649658 32.4464111328125 26.6731414794922 12.7852554321289 23.4871482849121 20.4650077819824 5.05988883972168 9.07431793212891 12.3017702102661 1.6895991563797 22.8414154052734 16.9412879943848 5.63299989700317 41.0247077941895 4.36324882507324 43.5881690979004 5.50226974487305 23.6425895690918 12.3907842636108 9.48320484161377 12.6446590423584 13.4469127655029 22.5454864501953 8.75185489654541 32.7476425170898 20.0863380432129 9.44534397125244 36.7467575073242 10.439980506897 20.7359600067139 28.9580497741699 8.22472190856934 8.03145408630371 15.0054912567139

ENSG00000231389.6 372.25390625 337.453338623047 353.570434570312 63.8373908996582 339.665344238281 550.8974609375 237.870056152344 266.250030517578 153.244491577148 250.885757446289 129.202697753906 603.334045410156 21.0451316833496 22.5396423339844 322.80517578125 43.0698204040527 55.7241172790527 161.644561767578 118.620246887207 44.8965454101562 27.5745658874512 315.138549804688 85.883674621582 440.478973388672 141.361206054688 373.904907226562 396.731201171875 131.222015380859 162.338333129883 343.871551513672 612.87451171875 843.609619140625 187.703277587891 113.005546569824 225.483917236328 912.455261230469 696.622863769531 409.518096923828 553.938354492188 265.904327392578 303.760925292969 945.680725097656 208.886764526367 162.39533996582 72.4572906494141 404.020965576172 376.938049316406 579.405456542969 463.156372070312 691.56884765625 42.8270263671875 519.380615234375 89.3898086547852 108.046989440918 1125.83557128906 27.5355110168457 14.3897676467896 70.7852554321289 152.802612304688 237.467575073242 724.452941894531 441.126373291016 585.151428222656 440.852661132812 156.003723144531 199.344161987305 420.641723632812 279.091278076172 350.0009765625 459.407867431641 587.458984375 317.3759765625 330.265899658203 164.915740966797 601.279846191406 378.511077880859 415.421478271484 184.136856079102 207.332138061523 45.7239265441895 748.943786621094 202.639465332031 229.961395263672 108.983795166016 54.6996879577637 128.0458984375 43.6975326538086 63.7178535461426 130.908798217773 241.444351196289 12.4940605163574 151.180969238281 110.039131164551 481.964416503906 302.929229736328 197.540939331055 361.138641357422 158.504669189453 814.524963378906 62.3827476501465 280.080810546875 549.471435546875 57.5524368286133 86.9082489013672 423.849273681641 323.533325195312 244.03547668457 222.768737792969 202.362030029297 24.932071685791 271.925842285156 267.788421630859 94.3793258666992 278.776397705078 16.2272815704346 386.974731445312 265.332214355469 94.1784973144531 148.874588012695 75.2698440551758 34.0384674072266 604.345153808594 196.965972900391 558.172790527344 880.534240722656 487.630676269531 201.770614624023 356.643432617188 464.582061767578 69.5999298095703 351.140899658203 379.733489990234 199.044937133789 202.060745239258 436.390167236328 229.5625 567.195251464844 181.976181030273 548.147705078125 928.533752441406 338.748626708984 261.938629150391 48.5058975219727 26.3186798095703 138.504348754883 95.607292175293 41.9426078796387 533.041259765625 650.731567382812 47.7485847473145 798.212829589844 669.2119140625 218.375961303711 332.130187988281 85.5038604736328 133.76545715332 289.914733886719 192.350814819336 384.463104248047 236.712554931641 134.443878173828 138.316146850586 1044.79089355469 451.006683349609 87.1224594116211 74.0841369628906 371.902069091797 585.470092773438 189.105987548828 429.234130859375 234.630081176758 255.001937866211 548.013488769531 147.029495239258 898.576904296875 92.3913726806641 393.425842285156 443.742401123047 381.263397216797 368.517578125 19.7180042266846 330.084747314453 119.597579956055 73.3443145751953 312.521179199219 260.511932373047 69.1529006958008 298.809112548828 17.2208919525146 540.099426269531 284.239532470703 185.010803222656 381.716094970703 279.750549316406 109.627029418945 99.5531463623047 669.087829589844 107.982437133789 520.599426269531 247.361602783203 200.120803833008 56.6711502075195 404.941955566406 356.592590332031 198.086288452148 157.346176147461 375.279937744141 796.464172363281 211.227951049805 531.888427734375 223.129058837891 111.402305603027 211.143218994141 795.017700195312 409.412841796875 64.3786926269531 37.3965644836426 222.60791015625 58.4579429626465 419.093231201172 54.7347259521484 149.300033569336 278.443481445312 397.345886230469 440.277465820312 263.064971923828 774.295043945312 353.482238769531 31.5591239929199 52.4718399047852 479.6455078125 30.7918529510498 340.946868896484 228.189575195312 144.571350097656 397.685333251953 156.078399658203 888.827819824219 123.334838867188 317.463928222656 195.927398681641 316.668029785156 114.45288848877 381.442932128906 269.823120117188 133.29541015625 727.026672363281 83.3350982666016 364.936981201172 96.4503936767578 138.661193847656 212.450988769531 680.839721679688 85.5850982666016 163.979461669922 259.930053710938

ENSG00000223865.9 365.314056396484 452.723876953125 470.770263671875 84.321647644043 420.594146728516 853.79638671875 570.285461425781 478.053131103516 803.379211425781 954.181274414062 270.956573486328 1029.587890625 85.1156997680664 96.0823135375977 629.959777832031 122.949104309082 76.8554840087891 395.107452392578 167.322235107422 85.8564987182617 102.031784057617 395.735290527344 92.4696044921875 552.735046386719 277.696350097656 513.497680664062 493.235717773438 445.877807617188 298.070953369141 652.295532226562 638.232177734375 1363.06005859375 444.787261962891 183.551406860352 521.919128417969 1116.48937988281 1118.95788574219 546.582214355469 735.205505371094 481.415771484375 465.439147949219 1269.44311523438 271.172698974609 294.744934082031 193.394866943359 468.367828369141 1647.51550292969 898.228942871094 750.700866699219 1090.294921875 53.8588714599609 934.983154296875 139.26838684082 335.531860351562 1801.64038085938 53.1610641479492 20.4625358581543 91.5596313476562 229.147827148438 212.656097412109 1064.43151855469 533.374206542969 633.868530273438 579.458618164062 245.160354614258 257.829071044922 885.361999511719 916.2734375 542.138732910156 671.658264160156 593.259155273438 394.750213623047 569.761779785156 233.960037231445 774.835693359375 471.022705078125 615.817199707031 284.135223388672 257.471343994141 101.63712310791 1098.01770019531 267.363677978516 292.963684082031 191.575073242188 108.051246643066 167.485504150391 189.429336547852 135.637725830078 219.97705078125 381.669464111328 37.9005088806152 420.737091064453 188.570251464844 539.105224609375 402.909729003906 264.455841064453 452.759399414062 298.631927490234 1130.15942382812 91.5395126342773 1160.3193359375 1009.19140625 81.3232192993164 187.311538696289 498.581176757812 992.866638183594 714.381408691406 339.001586914062 588.162841796875 63.741138458252 318.102844238281 358.490173339844 199.699905395508 370.092071533203 24.5501461029053 533.4140625 296.711151123047 213.797515869141 251.955032348633 113.167846679688 115.617736816406 805.922973632812 245.237243652344 810.804931640625 933.871276855469 679.973754882812 261.320495605469 449.063690185547 612.589782714844 209.914672851562 422.203674316406 500.12353515625 348.897857666016 396.459045410156 796.85546875 523.791748046875 571.317565917969 276.459381103516 738.41943359375 1848.2255859375 437.567077636719 481.244842529297 98.3239059448242 114.989479064941 250.143035888672 173.272872924805 90.0555648803711 1297.38244628906 1090.73266601562 81.2751998901367 824.190856933594 802.639221191406 428.096405029297 500.967742919922 97.8933792114258 292.997039794922 366.989349365234 399.60888671875 505.209411621094 406.760864257812 193.225799560547 368.64697265625 1098.99157714844 956.531494140625 226.953857421875 275.644073486328 575.971557617188 1092.30615234375 291.720458984375 923.453735351562 274.973846435547 647.007568359375 1020.11108398438 260.736145019531 1128.42895507812 185.564254760742 508.842010498047 1068.04516601562 507.786590576172 467.378570556641 39.6245384216309 451.470672607422 352.273559570312 95.5183639526367 507.484893798828 520.671936035156 120.917434692383 958.388427734375 54.2547225952148 866.883117675781 505.24853515625 305.686859130859 523.975463867188 646.232299804688 150.991027832031 148.928985595703 1175.32092285156 224.548080444336 1157.47570800781 506.186584472656 208.189987182617 153.467819213867 900.601867675781 475.375762939453 394.530883789062 204.60205078125 619.044860839844 1057.13317871094 318.267883300781 600.328674316406 293.251373291016 168.26286315918 421.836151123047 1376.96630859375 768.731628417969 73.2780685424805 129.459335327148 433.441284179688 114.291534423828 739.831420898438 94.0635147094727 194.447479248047 530.661560058594 709.500732421875 699.144348144531 464.753784179688 854.710510253906 722.372619628906 46.1372108459473 86.6163864135742 512.964477539062 87.334587097168 481.834320068359 272.727355957031 234.063400268555 598.343322753906 239.390274047852 1116.05126953125 222.998168945312 391.278869628906 674.44775390625 360.929565429688 180.962692260742 494.05322265625 773.460388183594 183.127044677734 1190.01940917969 401.847412109375 639.575744628906 169.731689453125 403.747894287109 827.956604003906 1085.22277832031 127.639961242676 299.478485107422 467.598876953125

ENSG00000196735.10 82.9751129150391 121.347427368164 199.712173461914 33.7304992675781 43.791618347168 279.518859863281 116.277404785156 62.252140045166 189.012573242188 105.494171142578 40.2209205627441 260.679718017578 26.3641738891602 12.3198919296265 106.276443481445 38.492504119873 18.1825695037842 77.4269256591797 34.9531631469727 8.1934232711792 20.7564277648926 312.776031494141 35.1623306274414 264.981201171875 52.5046882629395 135.214340209961 56.1416130065918 45.9831047058105 70.1680679321289 298.182556152344 215.401504516602 395.029357910156 129.662307739258 46.9258079528809 69.0486145019531 178.406143188477 134.554306030273 174.153274536133 135.686599731445 83.8081741333008 170.552734375 109.943092346191 131.696685791016 100.583045959473 63.7439498901367 107.833679199219 189.620635986328 255.90022277832 171.062530517578 281.361053466797 17.2845115661621 232.535079956055 18.1703071594238 68.6370086669922 104.303504943848 15.0470809936523 7.31868076324463 22.9704399108887 32.6857604980469 53.2046661376953 307.483276367188 355.238464355469 248.82861328125 101.522598266602 47.4246025085449 90.9553756713867 503.310577392578 128.670593261719 167.916687011719 143.590072631836 235.146865844727 107.893142700195 100.459922790527 32.1119270324707 111.538047790527 65.4404678344727 154.964385986328 33.033073425293 66.2861404418945 14.1148691177368 425.463745117188 106.901741027832 120.040252685547 59.9225769042969 30.5709972381592 68.4277496337891 19.9247646331787 15.5394296646118 16.9347743988037 162.526550292969 12.0108327865601 144.881454467773 48.8169975280762 221.994400024414 137.931503295898 79.9309310913086 128.83903503418 156.619110107422 488.750579833984 28.4179801940918 136.929122924805 239.137435913086 20.2101058959961 32.7748756408691 59.4208145141602 188.233154296875 294.985229492188 72.071647644043 161.663665771484 15.975022315979 104.725914001465 140.457229614258 49.7624435424805 113.09831237793 5.11990165710449 64.3634338378906 48.4038581848145 54.0197448730469 59.7963562011719 51.3008651733398 19.9144802093506 326.202239990234 25.9533710479736 173.03450012207 481.062774658203 136.872131347656 91.7885513305664 112.824241638184 135.769195556641 59.8171768188477 185.890899658203 130.570526123047 50.387020111084 35.9722061157227 172.278762817383 223.753219604492 242.98469543457 66.4396820068359 209.576110839844 223.55534362793 54.1940841674805 53.1249122619629 14.5108051300049 17.0359058380127 79.733512878418 35.2972793579102 20.9754829406738 166.6044921875 229.487335205078 13.331371307373 236.802047729492 249.022705078125 106.173767089844 136.545700073242 20.6741333007812 51.6342391967773 82.0431823730469 123.087303161621 224.423980712891 26.1261558532715 86.119026184082 88.160888671875 539.712341308594 89.0345306396484 44.5053482055664 49.6889686584473 125.27791595459 170.468734741211 111.925277709961 251.619873046875 50.4555587768555 144.592788696289 269.722137451172 76.3520889282227 414.781860351562 53.9730186462402 186.612777709961 244.537216186523 140.172271728516 95.5604400634766 13.6006708145142 136.072174072266 27.936164855957 28.9876537322998 123.533493041992 99.8975219726562 43.8342323303223 146.945327758789 7.83790254592896 285.080963134766 146.258819580078 97.1518859863281 209.551376342773 96.5280609130859 34.7802391052246 61.7894287109375 278.178436279297 79.7025146484375 355.434265136719 118.886993408203 76.3066940307617 39.6209449768066 415.749206542969 87.2712554931641 148.292449951172 61.4742889404297 104.048072814941 288.319519042969 44.2341728210449 75.3945083618164 58.8085289001465 29.5096473693848 99.1363906860352 652.263549804688 201.851333618164 14.5659627914429 48.6407585144043 138.406143188477 29.3585758209229 203.980621337891 14.9236440658569 12.0606918334961 107.018516540527 208.959228515625 151.253479003906 165.474853515625 385.733154296875 228.976821899414 15.895604133606 28.3225116729736 114.657188415527 22.4703960418701 60.3001747131348 97.9825973510742 70.6879043579102 92.5620880126953 82.5144653320312 307.681396484375 89.6493453979492 216.77473449707 221.987915039062 96.5878601074219 37.8137550354004 122.293350219727 176.989959716797 57.0867805480957 193.063247680664 48.9099731445312 203.617492675781 54.0451965332031 131.590103149414 236.328720092773 405.998474121094 16.6250381469727 49.7914657592773 96.5871810913086

ENSG00000237541.3 439.798187255859 63.3448143005371 389.713684082031 0.175149753689766 117.730712890625 181.3896484375 116.707656860352 118.348960876465 122.269577026367 36.2831115722656 29.9982089996338 72.8726348876953 25.5981044769287 3.38588356971741 87.9892654418945 27.8265819549561 25.6516704559326 21.5865345001221 77.4524230957031 30.0667018890381 7.08255386352539 39.0458221435547 57.6216659545898 68.1490097045898 103.15901184082 58.3342933654785 212.149917602539 13.0698986053467 125.059608459473 264.201690673828 398.075286865234 121.3876953125 196.702743530273 87.4619216918945 274.926147460938 714.489990234375 396.138702392578 209.575225830078 420.792755126953 96.0115280151367 62.4776611328125 282.600280761719 45.8916511535645 40.2990417480469 15.9006175994873 251.79052734375 165.111801147461 290.769775390625 240.608108520508 552.886047363281 12.6045904159546 202.183685302734 33.6818771362305 22.484058380127 454.244110107422 21.8223190307617 2.6952006816864 44.0616569519043 160.528900146484 275.555816650391 434.140472412109 8.50526142120361 176.547470092773 96.3947067260742 63.8567771911621 25.9497718811035 93.9229507446289 442.629669189453 149.820449829102 114.534469604492 115.833419799805 23.9948081970215 208.00358581543 127.06616973877 216.72412109375 301.348358154297 333.0791015625 136.038269042969 176.815231323242 69.032585144043 27.2285652160645 63.1051139831543 4.4547553062439 17.3640022277832 6.21262216567993 74.0612716674805 111.016777038574 47.858642578125 85.2841796875 22.6915473937988 3.7650032043457 41.409725189209 12.8210401535034 20.2140235900879 142.781326293945 60.5513534545898 188.192764282227 41.0912666320801 10.6120901107788 2.25479555130005 643.135009765625 199.049819946289 13.1318836212158 14.0783720016479 70.3903198242188 554.314575195312 4.80288791656494 162.659484863281 31.8766117095947 30.4957942962646 155.72673034668 6.64778709411621 48.2317771911621 82.1918792724609 7.02795219421387 110.209648132324 70.8907089233398 76.9167861938477 84.5969085693359 6.36407041549683 28.7494792938232 231.166473388672 15.4352512359619 137.019454956055 196.870010375977 34.2187957763672 1.67737758159637 115.937309265137 208.618637084961 15.191593170166 4.98957824707031 86.1849517822266 98.5096893310547 160.280944824219 385.81787109375 183.134552001953 457.419403076172 21.410961151123 427.296447753906 1198.81262207031 87.9087905883789 251.467819213867 29.3956699371338 35.9485130310059 83.5542144775391 38.0129928588867 22.99338722229 17.8442707061768 757.08447265625 58.9529075622559 350.623321533203 22.4644393920898 93.9468536376953 83.8730697631836 10.392954826355 62.4320220947266 209.996673583984 4.65594387054443 65.8673477172852 148.851272583008 53.2803421020508 4.36818408966064 29.204065322876 398.292053222656 88.362663269043 88.387336730957 199.622909545898 375.732116699219 32.0299797058105 370.025054931641 29.7483978271484 4.62085819244385 72.0799865722656 7.60024166107178 72.1170654296875 19.7733402252197 34.4955520629883 268.174530029297 196.40592956543 34.6921234130859 0.0667589604854584 199.145248413086 130.458480834961 3.82537627220154 280.746887207031 211.944396972656 0.568325400352478 48.504451751709 4.60523796081543 5.3632926940918 79.4275360107422 10.251389503479 60.5171356201172 63.395694732666 13.6955490112305 5.69027185440063 378.742401123047 150.603088378906 11.1900386810303 238.38883972168 168.552368164062 0.253051221370697 38.1995887756348 50.1032638549805 4.96980714797974 9.02355766296387 201.195037841797 262.653015136719 1.34088754653931 39.8706283569336 262.785217285156 47.4041404724121 163.791809082031 672.564392089844 55.7300758361816 6.56877279281616 41.1208305358887 43.620418548584 47.4344711303711 71.7295532226562 3.98333024978638 76.4908828735352 246.244934082031 113.003448486328 24.6736621856689 342.834411621094 119.95189666748 68.2795257568359 4.72007751464844 8.54871654510498 225.798706054688 8.81320858001709 110.995178222656 18.4899291992188 35.3808135986328 115.379325866699 7.79490947723389 286.641845703125 6.55214405059814 10.5628700256348 252.815567016602 139.058792114258 1.78124487400055 41.9582366943359 21.3126564025879 11.5506830215454 1022.82794189453 20.7046527862549 303.739501953125 14.578221321106 95.537353515625 23.5009365081787 248.531997680664 100.352752685547 47.5417442321777 22.7173175811768

ENSG00000237541.3.1 439.798187255859 63.3448143005371 389.713684082031 0.175149753689766 117.730712890625 181.3896484375 116.707656860352 118.348960876465 122.269577026367 36.2831115722656 29.9982089996338 72.8726348876953 25.5981044769287 3.38588356971741 87.9892654418945 27.8265819549561 25.6516704559326 21.5865345001221 77.4524230957031 30.0667018890381 7.08255386352539 39.0458221435547 57.6216659545898 68.1490097045898 103.15901184082 58.3342933654785 212.149917602539 13.0698986053467 125.059608459473 264.201690673828 398.075286865234 121.3876953125 196.702743530273 87.4619216918945 274.926147460938 714.489990234375 396.138702392578 209.575225830078 420.792755126953 96.0115280151367 62.4776611328125 282.600280761719 45.8916511535645 40.2990417480469 15.9006175994873 251.79052734375 165.111801147461 290.769775390625 240.608108520508 552.886047363281 12.6045904159546 202.183685302734 33.6818771362305 22.484058380127 454.244110107422 21.8223190307617 2.6952006816864 44.0616569519043 160.528900146484 275.555816650391 434.140472412109 8.50526142120361 176.547470092773 96.3947067260742 63.8567771911621 25.9497718811035 93.9229507446289 442.629669189453 149.820449829102 114.534469604492 115.833419799805 23.9948081970215 208.00358581543 127.06616973877 216.72412109375 301.348358154297 333.0791015625 136.038269042969 176.815231323242 69.032585144043 27.2285652160645 63.1051139831543 4.4547553062439 17.3640022277832 6.21262216567993 74.0612716674805 111.016777038574 47.858642578125 85.2841796875 22.6915473937988 3.7650032043457 41.409725189209 12.8210401535034 20.2140235900879 142.781326293945 60.5513534545898 188.192764282227 41.0912666320801 10.6120901107788 2.25479555130005 643.135009765625 199.049819946289 13.1318836212158 14.0783720016479 70.3903198242188 554.314575195312 4.80288791656494 162.659484863281 31.8766117095947 30.4957942962646 155.72673034668 6.64778709411621 48.2317771911621 82.1918792724609 7.02795219421387 110.209648132324 70.8907089233398 76.9167861938477 84.5969085693359 6.36407041549683 28.7494792938232 231.166473388672 15.4352512359619 137.019454956055 196.870010375977 34.2187957763672 1.67737758159637 115.937309265137 208.618637084961 15.191593170166 4.98957824707031 86.1849517822266 98.5096893310547 160.280944824219 385.81787109375 183.134552001953 457.419403076172 21.410961151123 427.296447753906 1198.81262207031 87.9087905883789 251.467819213867 29.3956699371338 35.9485130310059 83.5542144775391 38.0129928588867 22.99338722229 17.8442707061768 757.08447265625 58.9529075622559 350.623321533203 22.4644393920898 93.9468536376953 83.8730697631836 10.392954826355 62.4320220947266 209.996673583984 4.65594387054443 65.8673477172852 148.851272583008 53.2803421020508 4.36818408966064 29.204065322876 398.292053222656 88.362663269043 88.387336730957 199.622909545898 375.732116699219 32.0299797058105 370.025054931641 29.7483978271484 4.62085819244385 72.0799865722656 7.60024166107178 72.1170654296875 19.7733402252197 34.4955520629883 268.174530029297 196.40592956543 34.6921234130859 0.0667589604854584 199.145248413086 130.458480834961 3.82537627220154 280.746887207031 211.944396972656 0.568325400352478 48.504451751709 4.60523796081543 5.3632926940918 79.4275360107422 10.251389503479 60.5171356201172 63.395694732666 13.6955490112305 5.69027185440063 378.742401123047 150.603088378906 11.1900386810303 238.38883972168 168.552368164062 0.253051221370697 38.1995887756348 50.1032638549805 4.96980714797974 9.02355766296387 201.195037841797 262.653015136719 1.34088754653931 39.8706283569336 262.785217285156 47.4041404724121 163.791809082031 672.564392089844 55.7300758361816 6.56877279281616 41.1208305358887 43.620418548584 47.4344711303711 71.7295532226562 3.98333024978638 76.4908828735352 246.244934082031 113.003448486328 24.6736621856689 342.834411621094 119.95189666748 68.2795257568359 4.72007751464844 8.54871654510498 225.798706054688 8.81320858001709 110.995178222656 18.4899291992188 35.3808135986328 115.379325866699 7.79490947723389 286.641845703125 6.55214405059814 10.5628700256348 252.815567016602 139.058792114258 1.78124487400055 41.9582366943359 21.3126564025879 11.5506830215454 1022.82794189453 20.7046527862549 303.739501953125 14.578221321106 95.537353515625 23.5009365081787 248.531997680664 100.352752685547 47.5417442321777 22.7173175811768

ENSG00000179344.15 78.2404708862305 184.579254150391 385.045257568359 116.481727600098 59.7000503540039 157.109786987305 117.071258544922 286.842803955078 107.790901184082 857.065795898438 160.153503417969 408.940124511719 15.573769569397 46.1196098327637 152.731018066406 49.7654991149902 17.11887550354 207.070678710938 80.501579284668 40.3981704711914 127.890968322754 740.820617675781 39.116382598877 379.810699462891 36.8616371154785 130.237396240234 93.851936340332 181.720397949219 50.748046875 387.206787109375 247.347137451172 531.181030273438 161.094467163086 60.3503379821777 53.8205490112305 200.95491027832 269.996551513672 314.957305908203 240.15185546875 224.234436035156 141.504928588867 149.563629150391 86.5567321777344 132.891082763672 106.109169006348 202.103317260742 831.116455078125 499.263854980469 381.909393310547 1304.00891113281 29.5525493621826 251.674728393555 20.7982540130615 158.752944946289 271.589324951172 38.0543098449707 15.1567325592041 17.8933753967285 276.153106689453 93.9803695678711 633.16259765625 785.395568847656 475.972137451172 334.842224121094 51.1444816589355 100.887008666992 626.42431640625 200.670715332031 241.464508056641 232.369903564453 421.051208496094 323.772735595703 95.781005859375 44.688850402832 127.031051635742 154.620407104492 146.398071289062 238.030090332031 84.6609573364258 37.9659576416016 784.900817871094 49.6000061035156 240.656219482422 393.919250488281 48.6492195129395 46.7257690429688 47.6877670288086 34.0717811584473 63.9490280151367 335.155944824219 14.5311126708984 89.9360122680664 183.719360351562 402.340454101562 247.320068359375 478.484039306641 306.546508789062 277.235321044922 550.258850097656 125.383567810059 222.018966674805 221.626220703125 11.4052400588989 126.554504394531 594.140075683594 193.726226806641 418.990905761719 209.465042114258 258.617156982422 9.14118194580078 368.658508300781 303.247253417969 39.240348815918 182.74528503418 34.6121864318848 187.746246337891 142.07258605957 120.146499633789 76.9704895019531 64.8515167236328 55.0097732543945 646.321960449219 52.9923667907715 248.211242675781 512.059936523438 704.688720703125 356.029541015625 162.469360351562 359.139068603516 494.009185791016 471.238922119141 53.0855331420898 163.531066894531 86.3703002929688 249.53498840332 221.785064697266 379.905639648438 183.695571899414 283.977416992188 265.763824462891 99.5760879516602 184.70849609375 21.346061706543 15.87034034729 56.641918182373 75.1747436523438 45.5765686035156 412.934173583984 410.004791259766 15.6897048950195 339.254638671875 683.366455078125 230.579025268555 301.708740234375 58.0976753234863 102.710052490234 77.8681259155273 172.109497070312 192.490478515625 89.7402420043945 37.1270027160645 134.205505371094 770.531494140625 310.147552490234 42.2916069030762 35.5910034179688 301.492340087891 315.254730224609 170.275955200195 236.355575561523 128.671783447266 525.920471191406 912.542724609375 196.054885864258 721.094665527344 84.5152435302734 455.958984375 272.561737060547 268.090576171875 253.769973754883 35.3104782104492 240.759368896484 100.849792480469 59.2701568603516 123.98511505127 136.402633666992 46.9926414489746 132.865768432617 7.8173451423645 469.504760742188 76.4960021972656 247.422470092773 530.229736328125 109.001159667969 106.524627685547 105.325660705566 266.380798339844 51.0462379455566 373.961212158203 185.999954223633 73.2406768798828 79.7932815551758 687.940612792969 113.56713104248 239.279525756836 186.841659545898 368.391845703125 433.856903076172 221.888488769531 226.83642578125 76.8461608886719 52.4103240966797 251.337493896484 1186.51379394531 279.440734863281 7.80729150772095 32.1471328735352 221.193756103516 90.1161804199219 256.402648925781 88.7151947021484 45.1880302429199 183.096527099609 75.8266754150391 268.017639160156 159.772354125977 205.355850219727 524.8818359375 22.046215057373 24.3960971832275 627.155517578125 63.674259185791 105.178375244141 182.58708190918 45.6691665649414 274.901519775391 233.270401000977 547.981750488281 123.900451660156 127.353309631348 209.896713256836 130.540298461914 76.3699951171875 186.895538330078 475.600311279297 56.2913055419922 262.3271484375 169.657241821289 410.310852050781 62.568042755127 184.272308349609 730.409912109375 697.331665039062 28.4440975189209 121.697357177734 289.651428222656

ENSG00000204287.12 3172.59033203125 3159.98974609375 3512.9462890625 1057.21655273438 2820.48120117188 5761.169921875 3268.27465820312 5013.31787109375 3710.81225585938 3756.87377929688 2032.22351074219 5633.64453125 545.13037109375 856.499877929688 3418.1572265625 589.463439941406 590.015258789062 2434.79614257812 1256.11499023438 526.258422851562 524.398803710938 4590.1337890625 831.650146484375 5291.2392578125 2045.79565429688 3401.37573242188 4095.26831054688 3385.9990234375 2333.03515625 5937.06396484375 5882.9560546875 8046.57177734375 3233.64672851562 1208.33374023438 10221.837890625 6479.6787109375 12418.0537109375 5130.58349609375 6096.333984375 2999.97192382812 3807.34155273438 12871.302734375 2524.82397460938 2370.95068359375 928.229248046875 6718.447265625 5413.1904296875 6559.255859375 4911.30810546875 7242.56787109375 475.198211669922 6965.80029296875 1150.90869140625 1527.74865722656 9487.03125 418.549621582031 149.898620605469 679.140686035156 1636.13732910156 2358.90747070312 5997.79345703125 4128.39697265625 9306.90625 5003.35693359375 1852.91809082031 1763.36730957031 6443.248046875 4624.01904296875 3537.97094726562 3864.37353515625 5165.67578125 3432.3857421875 3084.41748046875 1684.74914550781 5950.283203125 4332.42138671875 12883.4287109375 2384.36840820312 2877.01635742188 642.228088378906 7082.93701171875 2053.9970703125 2654.51440429688 1785.72924804688 886.822875976562 1463.24267578125 1431.84887695312 596.974060058594 1772.595703125 3068.84399414062 250.670074462891 2770.58862304688 1210.78125 4259.658203125 3166.09350585938 2654.6376953125 4467.85888671875 2464.4931640625 7824.36669921875 794.390625 10901.1318359375 5808.6044921875 591.784790039062 1572.79711914062 4645.52734375 6173.8701171875 5100.72509765625 2507.38647460938 4248.5244140625 401.927490234375 3261.1416015625 3184.18579101562 1665.86633300781 4162.54052734375 169.514297485352 3225.87060546875 2300.33569335938 1547.29479980469 1562.01672363281 946.153991699219 727.777709960938 6508.177734375 1454.86975097656 6267.19482421875 7116.38232421875 5523.86865234375 1827.50939941406 4238.5048828125 4706.76806640625 1873.65478515625 4037.1357421875 3901.04956054688 2304.43408203125 3356.47290039062 12414.95703125 4578.47412109375 6168.26513671875 2072.0986328125 5125.5634765625 7705.09912109375 3757.826171875 4497.30078125 805.861450195312 684.139404296875 2561.82666015625 1529.58276367188 585.082641601562 5796.63525390625 8817.33203125 573.866271972656 8531.2490234375 6731.61181640625 4008.14916992188 3702.22778320312 1040.08825683594 1626.1748046875 2974.35302734375 2194.603515625 3571.30249023438 2087.36987304688 1696.63244628906 2394.88916015625 10446.4345703125 6243.6591796875 1313.50964355469 2377.25512695312 4246.55908203125 6008.1103515625 2162.99267578125 7452.37158203125 1849.71594238281 6766.0126953125 9212.0615234375 1604.34899902344 10459.6650390625 1012.11364746094 4197.74853515625 9175.498046875 4119.49365234375 3521.5986328125 277.720184326172 3530.02661132812 2690.38500976562 565.419616699219 3863.41064453125 2903.791015625 997.545471191406 12505.908203125 236.39453125 5766.20751953125 2640.3671875 2030.5537109375 4310.2421875 3858.98095703125 1204.25634765625 953.230407714844 9879.904296875 2231.09716796875 7329.1953125 3084.31005859375 2197.84594726562 724.09814453125 7964.7900390625 2598.97485351562 2933.38720703125 1569.10815429688 4699.39892578125 8649.9990234375 1948.63500976562 4175.60986328125 2542.60864257812 1144.83190917969 3205.74365234375 11949.73046875 4428.634765625 528.8056640625 748.81787109375 2774.05834960938 745.127624511719 6023.4111328125 833.469665527344 1789.36657714844 4129.2880859375 9884.6357421875 4002.4453125 3843.31225585938 6770.18701171875 5496.90087890625 302.303009033203 637.442077636719 3962.5986328125 558.096862792969 5505.52978515625 2370.79541015625 1443.28247070312 4236.21875 1796.77819824219 9129.265625 1498.65612792969 4649.54443359375 4351.583984375 3061.42407226562 1302.59216308594 3116.45776367188 3750.27001953125 1643.86206054688 15373.9404296875 2326.70483398438 5974.92333984375 1333.27465820312 2340.93798828125 6790.79248046875 7477.08935546875 939.623352050781 1540.92980957031 3042.75024414062

ENSG00000196126.9 1534.75354003906 1791.40124511719 2672.47485351562 794.643737792969 1628.52185058594 3707.01684570312 2147.60107421875 1587.42138671875 2684.19604492188 5013.326171875 1126.02453613281 4010.87890625 330.106414794922 544.621704101562 1885.3603515625 388.935821533203 238.163543701172 1634.58569335938 666.571472167969 159.068435668945 486.706909179688 4284.73193359375 334.538024902344 3594.609375 1227.67248535156 3369.97290039062 929.782775878906 1591.06530761719 1040.50769042969 2947.66674804688 2375.58813476562 6082.8359375 1579.07299804688 589.930419921875 918.32177734375 3682.5263671875 5174.4443359375 2326.0419921875 2852.9375 1886.36657714844 2085.54443359375 6532.55029296875 1343.58813476562 1594.34411621094 759.986389160156 2318.9521484375 4509.93505859375 3455.484375 2787.865234375 3895.59399414062 184.340942382812 3142.44873046875 679.307922363281 1576.78857421875 3175.20141601562 379.5458984375 106.147735595703 329.247009277344 479.783660888672 775.893127441406 4324.73876953125 4081.18872070312 4397.41015625 3212.20336914062 803.528076171875 1062.78002929688 4692.34716796875 2450.30224609375 1664.25500488281 1746.7802734375 3540.37841796875 2988.828125 1845.56616210938 523.839782714844 3322.03369140625 1280.18286132812 2757.35327148438 763.398620605469 874.58984375 267.499633789062 5507.20361328125 1145.70825195312 1840.89929199219 1662.82727050781 592.65087890625 610.512878417969 506.356750488281 583.582946777344 358.549865722656 2029.78845214844 190.486221313477 1742.55139160156 970.059387207031 2728.92993164062 1878.73315429688 1677.38098144531 2275.26879882812 1537.2958984375 4816.9296875 783.827270507812 2272.04418945312 3763.76440429688 357.09228515625 1086.18786621094 5602.78515625 4061.87866210938 3338.13671875 1499.8505859375 4902.36376953125 210.370910644531 1397.22692871094 2214.42431640625 709.822570800781 1909.90405273438 86.4337005615234 1639.52368164062 992.844177246094 777.30810546875 688.158508300781 616.613586425781 373.235168457031 4262.27880859375 1857.40222167969 2461.35571289062 4262.150390625 4334.52099609375 1823.21240234375 1899.80236816406 2232.84936523438 2192.32104492188 3330.82592773438 2483.40112304688 1090.93041992188 1083.62878417969 3668.98608398438 2035.24182128906 3109.5068359375 1583.05432128906 2742.091796875 5214.77587890625 1450.4697265625 1158.62829589844 387.573425292969 307.235931396484 1116.49145507812 601.773498535156 246.759750366211 2988.74438476562 2299.49975585938 147.152328491211 3312.08276367188 5333.07421875 2564.12329101562 2245.8466796875 491.494079589844 894.063415527344 2161.96435546875 1486.28686523438 2341.46630859375 1494.41467285156 761.642150878906 1297.40551757812 7703.2763671875 1801.34985351562 746.916137695312 1276.11779785156 2092.75854492188 3122.59765625 1451.82165527344 2915.33959960938 1478.02807617188 4272.986328125 5287.94189453125 1083.47229003906 8121.873046875 803.523620605469 3266.77392578125 3296.22534179688 1853.68273925781 2318.29370117188 162.909866333008 1882.56774902344 644.3203125 436.537933349609 1640.45983886719 1604.30737304688 523.586120605469 3811.83544921875 224.122756958008 4178.4482421875 1782.38049316406 1284.255859375 2883.26977539062 3316.46020507812 814.331909179688 629.590881347656 3477.447265625 683.824523925781 2958.00244140625 1096.19323730469 831.55712890625 506.412170410156 5968.72314453125 2866.84204101562 1808.44677734375 1150.603515625 2578.84887695312 3897.48413085938 1791.83435058594 4305.14794921875 676.427307128906 793.295654296875 1667.416015625 6888.8125 3355.62817382812 385.533630371094 442.931030273438 2132.75952148438 517.691284179688 2881.79833984375 775.691772460938 766.680358886719 2113.61279296875 4976.54638671875 1733.84167480469 1408.16638183594 4772.5087890625 3280.66577148438 211.098251342773 429.808258056641 1626.95910644531 386.558502197266 2644.4462890625 1597.2255859375 1049.49670410156 1880.55432128906 1422.94519042969 4214.49609375 1103.1455078125 1926.1259765625 4225.1494140625 981.293823242188 666.163696289062 2384.85473632812 3153.08056640625 1112.54028320312 3183.46362304688 1514.57177734375 3371.79467773438 846.11328125 1519.63659667969 5441.3349609375 4611.3525390625 287.255340576172 810.839538574219 2314.91845703125

ENSG00000198502.5 1157.09448242188 414.644897460938 845.452026367188 269.752868652344 606.537048339844 546.819702148438 380.328704833984 528.708312988281 433.053344726562 756.264526367188 1137.27880859375 2297.98608398438 48.8336143493652 65.3989028930664 657.073303222656 49.4465789794922 80.1224594116211 329.619476318359 179.128326416016 75.6744537353516 71.4013061523438 2438.263671875 126.869522094727 1479.14770507812 370.8896484375 438.647521972656 516.323425292969 73.9537048339844 291.204681396484 707.431091308594 886.971923828125 819.280822753906 325.8623046875 183.863128662109 505.047943115234 1728.29333496094 1629.70971679688 1888.39453125 1181.01391601562 1343.06506347656 364.186218261719 1501.17395019531 181.990310668945 186.400100708008 93.8545837402344 474.403472900391 715.362121582031 746.78759765625 1859.54772949219 2896.3369140625 51.4276351928711 1036.72766113281 102.145660400391 167.840637207031 1910.74169921875 158.247711181641 82.2568893432617 72.7316360473633 252.838363647461 438.256225585938 927.760131835938 3300.85034179688 4208.27783203125 2432.22094726562 185.260635375977 141.871109008789 2618.1982421875 1273.59533691406 541.968933105469 536.040710449219 466.147125244141 1749.3955078125 515.593994140625 309.275360107422 825.437622070312 735.188171386719 834.617492675781 395.120544433594 450.455230712891 136.888397216797 4259.34228515625 228.215377807617 992.671142578125 535.050964355469 231.083770751953 117.94896697998 278.756378173828 134.075576782227 213.332290649414 854.836608886719 77.9297790527344 237.506225585938 483.270843505859 1361.18786621094 1065.58862304688 1013.8759765625 1602.31066894531 183.36003112793 864.008850097656 516.095520019531 1256.13623046875 578.200927734375 57.5059814453125 457.087280273438 717.579284667969 1107.35278320312 711.195251464844 355.217864990234 448.856140136719 57.1023216247559 357.600433349609 815.821655273438 155.061416625977 675.509643554688 21.5045127868652 973.307556152344 248.697174072266 189.922256469727 151.331954956055 104.154853820801 138.459884643555 2566.6845703125 312.515625 1746.72155761719 593.595642089844 685.301696777344 1122.36157226562 457.074768066406 1760.71484375 325.18896484375 2341.33032226562 317.968231201172 380.37255859375 509.009552001953 1379.57788085938 746.249389648438 1183.59533691406 1060.02392578125 1168.82397460938 2339.78881835938 481.351867675781 721.011047363281 103.26961517334 67.2714462280273 222.318237304688 426.955810546875 50.4075241088867 3785.5654296875 1234.45349121094 88.1847915649414 1133.95336914062 4974.65185546875 1488.22265625 525.77783203125 268.919464111328 222.24250793457 523.937927246094 252.506240844727 391.0595703125 321.734893798828 95.8312759399414 384.335113525391 3160.56298828125 994.953918457031 207.638076782227 192.944198608398 656.05078125 1068.26953125 684.112060546875 790.152038574219 278.077392578125 1104.13928222656 2057.19873046875 152.17512512207 4059.86254882812 91.3817901611328 1353.044921875 931.915893554688 1353.20520019531 1692.08557128906 19.6214122772217 1040.09313964844 403.359741210938 249.983917236328 823.122375488281 538.8076171875 85.5816879272461 595.842041015625 28.2711143493652 3692.580078125 172.1318359375 205.777420043945 365.221374511719 516.143981933594 472.341796875 77.7818984985352 1162.36096191406 185.536544799805 604.800903320312 456.099060058594 344.444305419922 248.734100341797 4334.56005859375 196.928039550781 649.520751953125 541.063720703125 2305.8232421875 1149.4404296875 1363.91711425781 663.133911132812 392.140380859375 589.811950683594 1041.48718261719 5924.8583984375 531.770751953125 54.0126571655273 103.617965698242 361.653564453125 181.24397277832 743.320129394531 499.389251708984 515.494689941406 665.368347167969 534.387390136719 648.835388183594 535.554565429688 766.691528320312 1912.3720703125 29.7887859344482 76.6367340087891 590.500061035156 178.346420288086 604.4033203125 1708.14343261719 133.954483032227 607.739196777344 722.525817871094 3778.11596679688 190.325180053711 282.588745117188 493.3955078125 545.413330078125 97.5773696899414 1258.57495117188 1486.08044433594 211.875610351562 2135.5361328125 262.152069091797 1870.24047851562 277.964111328125 228.304672241211 4335.24755859375 755.783569335938 148.352172851562 209.602416992188 1166.76538085938

ENSG00000204592.8 462.542663574219 1377.27685546875 1077.93566894531 532.091857910156 741.552062988281 1669.67895507812 726.818786621094 669.029907226562 1130.78125 1268.14221191406 460.847412109375 1239.92687988281 253.072418212891 367.123352050781 992.175720214844 656.26904296875 289.906341552734 649.511291503906 848.130310058594 719.638000488281 222.93293762207 1371.72241210938 214.931915283203 1047.61279296875 860.776794433594 546.670349121094 1185.49584960938 522.049194335938 737.810729980469 1297.951171875 971.244750976562 1853.18811035156 744.449157714844 694.788391113281 836.87158203125 1206.66088867188 909.9814453125 910.506591796875 1016.87536621094 1077.70080566406 1046.19104003906 1369.8125 563.377746582031 376.395599365234 319.673522949219 891.981201171875 1254.00158691406 1265.36828613281 1152.59509277344 3041.61669921875 444.555877685547 1382.67211914062 617.026245117188 878.581665039062 1706.34057617188 437.67919921875 249.503646850586 656.206726074219 526.200622558594 640.777526855469 1271.67810058594 1142.90502929688 1072.333984375 1868.48767089844 722.741943359375 601.451782226562 1195.81970214844 1177.5185546875 1028.65783691406 1224.91943359375 945.375549316406 833.011840820312 1811.44897460938 1005.17224121094 889.821655273438 1297.25646972656 1068.61059570312 770.10498046875 466.367889404297 378.419189453125 1167.9970703125 634.472229003906 1105.12890625 454.50146484375 299.504760742188 573.534606933594 506.932769775391 554.810424804688 475.613372802734 800.283447265625 298.978698730469 695.437133789062 1083.83166503906 1124.34997558594 730.146423339844 642.3525390625 754.292785644531 2529.63330078125 1956.53308105469 401.374725341797 1154.16638183594 1091.17041015625 258.903350830078 474.898864746094 876.107971191406 1059.40356445312 1580.98828125 626.188049316406 928.613586425781 186.456069946289 1088.13793945312 855.567626953125 534.623657226562 1010.77117919922 363.620300292969 569.729064941406 761.587890625 359.123199462891 744.268127441406 958.676025390625 798.273742675781 1651.31628417969 1927.45874023438 904.54052734375 1734.89758300781 1100.03271484375 653.014343261719 1321.84680175781 1260.61279296875 617.094421386719 897.3740234375 1780.3076171875 555.844787597656 713.535278320312 773.054260253906 1230.90954589844 1020.78784179688 893.798522949219 1626.89038085938 1735.29528808594 980.309265136719 723.708312988281 1311.71533203125 477.663604736328 1026.36511230469 317.846557617188 240.05110168457 957.649291992188 1861.77661132812 501.357696533203 1805.60900878906 1639.91516113281 813.729431152344 1430.62255859375 303.530700683594 832.603454589844 1160.810546875 789.0087890625 2052.08203125 675.369934082031 739.716125488281 545.344848632812 1367.65185546875 890.460876464844 857.259765625 915.35302734375 1893.84741210938 2234.970703125 794.905639648438 1868.34729003906 924.424743652344 1193.61535644531 1330.02954101562 427.290069580078 1604.10461425781 478.683502197266 608.491394042969 1738.80139160156 1024.61767578125 986.308959960938 245.135131835938 1136.64147949219 213.591827392578 682.228149414062 1169.85046386719 845.395141601562 317.067291259766 873.318786621094 648.490966796875 1280.1962890625 1128.07250976562 678.230712890625 1094.14916992188 922.587341308594 613.515380859375 502.010192871094 1352.79284667969 462.458465576172 1065.13073730469 653.048400878906 706.280822753906 506.811187744141 1780.19506835938 315.374145507812 852.050598144531 721.542907714844 1039.03942871094 1204.39624023438 353.711669921875 2861.85034179688 641.254821777344 849.319458007812 923.109252929688 2097.03344726562 1111.50927734375 595.925842285156 246.400039672852 967.774719238281 488.244445800781 956.843994140625 653.829223632812 375.944030761719 987.114929199219 808.366638183594 927.309753417969 1789.02905273438 1917.4287109375 817.019287109375 248.857238769531 444.450103759766 963.558898925781 300.243103027344 1378.89025878906 615.161804199219 579.761047363281 1548.31628417969 579.375732421875 1333.43322753906 1094.8984375 692.795227050781 1512.79650878906 920.745910644531 797.670166015625 724.835266113281 2018.58190917969 414.232543945312 822.474670410156 1006.36187744141 1238.759765625 631.080688476562 873.068298339844 1013.14025878906 1492.46374511719 588.619018554688 866.496948242188 1174.626953125

ENSG00000204642.12 45.2200469970703 82.7060623168945 86.4676132202148 86.1072006225586 36.7217178344727 88.6504364013672 30.5123901367188 34.5585784912109 107.240737915039 76.1082153320312 41.525016784668 84.2028579711914 15.5734596252441 24.2618961334229 59.7472190856934 51.6076164245605 22.9940395355225 57.3595275878906 42.5008926391602 82.0134429931641 37.7881813049316 230.906661987305 9.34935760498047 69.4595413208008 79.0101852416992 59.1570625305176 94.1186218261719 21.6737232208252 29.409143447876 128.291412353516 63.566837310791 71.3518295288086 57.5470008850098 43.7179412841797 109.84651184082 64.5081481933594 65.2487564086914 112.350151062012 41.8261184692383 97.2225952148438 88.8073348999023 66.6907730102539 53.8237838745117 22.0579357147217 24.7173137664795 91.2589797973633 161.012252807617 199.584747314453 92.0192031860352 509.236755371094 21.6356163024902 156.816436767578 47.4802780151367 158.292816162109 167.180450439453 47.7843589782715 15.0969543457031 22.7308807373047 23.7973175048828 28.5492286682129 209.811340332031 167.77473449707 114.67765045166 212.524337768555 29.0816879272461 63.6230278015137 80.6061019897461 80.4327392578125 88.8298110961914 76.6571884155273 39.3866691589355 36.3303337097168 100.30167388916 73.3916244506836 97.4091262817383 112.885009765625 106.493988037109 44.9163551330566 25.2125988006592 13.5820083618164 91.7128829956055 33.5068550109863 112.57746887207 19.9072399139404 15.8685064315796 33.0815353393555 67.542594909668 56.8911972045898 23.7443027496338 60.0981597900391 10.8276081085205 46.4192047119141 60.3758201599121 45.6617317199707 77.4986343383789 39.3705673217773 89.6026306152344 355.337493896484 103.367065429688 24.6970596313477 114.964164733887 153.533233642578 11.3304042816162 23.9922637939453 50.6852760314941 93.5107727050781 188.208129882812 32.2612457275391 114.72966003418 12.4109058380127 77.5210647583008 88.2795333862305 75.8988037109375 59.2290916442871 11.4352331161499 47.1832504272461 40.4311218261719 15.6352243423462 37.8797416687012 99.0978240966797 49.1974296569824 146.86296081543 220.484924316406 53.5704345703125 120.14379119873 66.4282608032227 31.0003833770752 220.387054443359 144.592498779297 30.9810581207275 64.8965377807617 119.97193145752 33.1627006530762 41.7734985351562 56.7685317993164 142.346145629883 73.6744842529297 63.4026336669922 111.653053283691 128.988143920898 37.991382598877 47.9481544494629 168.347900390625 76.9988098144531 60.7498168945312 26.7582149505615 15.2250204086304 44.8993759155273 208.976104736328 27.1389827728271 252.810806274414 160.153030395508 162.398864746094 427.255157470703 20.5841655731201 164.185516357422 164.187683105469 66.7212219238281 207.72526550293 61.6324882507324 36.6206130981445 48.8655281066895 112.78653717041 75.3401641845703 108.915412902832 115.425521850586 201.621032714844 149.888229370117 48.3986778259277 229.976806640625 135.888793945312 137.84342956543 107.228988647461 26.3690509796143 299.986053466797 40.5797538757324 40.648509979248 110.293846130371 73.81494140625 102.056335449219 7.49815940856934 216.019866943359 8.22036075592041 34.0654563903809 80.4961547851562 76.9093399047852 22.1704120635986 111.103332519531 79.3100509643555 148.636215209961 97.8553695678711 54.391040802002 67.2664566040039 36.0587501525879 23.6687793731689 45.6353454589844 89.6883697509766 15.2507619857788 92.3723907470703 77.2320098876953 97.656120300293 39.2873954772949 232.879257202148 18.0890579223633 80.9567184448242 61.948184967041 31.2356128692627 92.9032287597656 30.201545715332 221.357894897461 67.1234436035156 35.7466850280762 51.253833770752 167.878662109375 159.750717163086 26.3725490570068 26.5356960296631 151.533157348633 41.9161148071289 79.1981353759766 52.459529876709 82.1700286865234 51.3143615722656 63.9173049926758 60.7213821411133 308.163635253906 136.981353759766 49.5687141418457 19.5359573364258 30.1620616912842 55.2668914794922 13.5814590454102 164.006057739258 45.0321807861328 58.6742782592773 198.974151611328 30.7608127593994 92.0750503540039 59.3278007507324 48.8882713317871 145.05256652832 67.6481552124023 37.8189010620117 28.3030490875244 116.482627868652 29.4303283691406 70.1680679321289 166.651626586914 109.536743164062 34.6107482910156 91.1025543212891 116.908073425293 85.6449813842773 33.0266990661621 65.1040420532227 82.2678298950195

ENSG00000204632.10 8.52861881256104 28.8014812469482 17.0831661224365 2.3215537071228 9.89507865905762 1.72474551200867 4.50714063644409 4.3551721572876 14.3806953430176 14.6006746292114 11.4155054092407 3.67953848838806 5.64891481399536 1.75612699985504 9.41198062896729 9.96959114074707 2.33557462692261 9.9368257522583 5.15308523178101 15.1108884811401 73.4103546142578 53.634765625 2.20715951919556 3.80601119995117 7.29329586029053 6.49558401107788 19.0388317108154 4.43367195129395 6.54954195022583 18.3663349151611 11.241623878479 23.2926616668701 6.77836656570435 3.72000670433044 20.7067451477051 12.8071002960205 9.00400066375732 18.9978618621826 4.64395427703857 17.2643909454346 161.437805175781 11.5681228637695 20.9913368225098 2.31645512580872 5.50361156463623 11.1216039657593 50.2930870056152 28.5172176361084 10.7271499633789 31.0062808990479 4.02997446060181 18.1572780609131 5.01833534240723 27.188892364502 51.2787971496582 6.13079690933228 1.34044873714447 10.0281229019165 6.13553047180176 5.27672719955444 21.9869632720947 7.80956840515137 10.757700920105 29.3886623382568 1.99747538566589 11.9739379882812 23.5578212738037 19.2267284393311 16.4422340393066 8.37576770782471 8.440354347229 3.60526299476624 24.351131439209 12.7051811218262 5.25187015533447 17.1671199798584 2.72555088996887 2.67563414573669 4.48151111602783 1.69715690612793 12.4258041381836 4.92744588851929 250.568939208984 2.81848311424255 0.744413495063782 7.72413539886475 3.87330627441406 7.08033037185669 5.58778238296509 5.56903600692749 0.367455154657364 17.2915725708008 14.3899784088135 10.7458715438843 53.1925239562988 1.43404793739319 17.6561126708984 32.9366340637207 24.3721122741699 3.83637404441833 20.4145088195801 1.33957231044769 2.1944751739502 6.40321111679077 6.05650997161865 18.0011920928955 22.2900905609131 7.87875413894653 2.36188101768494 4.17114734649658 16.2608661651611 14.6240634918213 5.77100133895874 20.8549785614014 3.86238622665405 3.10930633544922 3.55483150482178 6.43178415298462 11.4321794509888 22.1332530975342 9.11718845367432 36.6049728393555 32.8496208190918 9.44392395019531 8.22203063964844 15.8025302886963 1.39998543262482 19.9872856140137 11.3186435699463 4.94039249420166 17.4956722259521 7.36329412460327 8.28148651123047 5.92378616333008 9.29331970214844 692.052673339844 5.23868560791016 7.57710075378418 11.1689500808716 46.0735359191895 6.64714241027832 8.78463554382324 39.0736846923828 6.68460130691528 4.16375303268433 17.2481422424316 3.13836312294006 2.4459433555603 25.2614364624023 3.54819774627686 16.8065757751465 1.7053564786911 21.3437671661377 2152.20434570312 3.10163307189941 6.56202697753906 16.6536884307861 6.54532289505005 48.3459358215332 14.8622579574585 5.57922029495239 6.03516721725464 14.5985660552979 3.10451102256775 6.41297435760498 5.76052951812744 44.0711326599121 49.1497764587402 20.1569366455078 50.8832130432129 24.6646976470947 5.65548658370972 11.2710189819336 1.89062440395355 23.1121597290039 4.33697414398193 102.252494812012 24.776834487915 13.5894193649292 10.8451881408691 1.09646761417389 16.2743453979492 2.87819623947144 4.67674779891968 4.37645673751831 25.2675361633301 4.21097803115845 5.59408283233643 4.09359979629517 6.52934503555298 6.23609066009521 19.3014373779297 13.8705224990845 4.39458322525024 4.51006460189819 8.35534763336182 12.9338750839233 1.94508683681488 13.2354612350464 12.3501405715942 7.59715938568115 7.07280302047729 23.680736541748 2.91656875610352 2.45490169525146 10.7350454330444 5.80136728286743 15.5071172714233 0.960406184196472 5.44312429428101 5.78484201431274 5.1787428855896 19.1837959289551 34.2480201721191 36.5441207885742 2.78933119773865 5.36897373199463 14.2207126617432 12.7041807174683 21.7775688171387 5.14004755020142 4.24366903305054 20.3898887634277 124.129241943359 17.1825046539307 380.127777099609 25.7995223999023 6.00934171676636 6.05970764160156 5.89253282546997 11.6472320556641 5.11653423309326 12.9001865386963 4.27036142349243 20.3269996643066 23.313175201416 6.60161209106445 25.6694831848145 10.2040224075317 217.904006958008 32.9726295471191 15.5588550567627 11.7022638320923 4.11859369277954 13.4520206451416 3.56756138801575 4.53445816040039 13.1569347381592 21.438440322876 2.51201367378235 32.9185409545898 6.25078392028809 12.358717918396 7.8357949256897 18.0463809967041 15.4799680709839

ENSG00000153029.13 17.4583320617676 16.2824859619141 18.3303165435791 13.4396896362305 21.0307502746582 21.1719627380371 37.2089042663574 33.1755294799805 13.4051284790039 15.6039371490479 6.37903451919556 18.7365970611572 4.87896680831909 15.2096948623657 24.0716361999512 9.32913970947266 14.5876979827881 23.1363201141357 21.8260688781738 10.8889455795288 15.0493240356445 17.1366367340088 2.84633564949036 23.7914409637451 32.8614959716797 27.3055934906006 24.7552642822266 8.79083919525146 13.9797916412354 19.968957901001 28.5712833404541 23.9054279327393 24.6014785766602 16.4130249023438 11.6478900909424 43.3033218383789 39.9773902893066 14.4672899246216 29.5633602142334 20.08864402771 38.3688163757324 32.5888938903809 27.0717391967773 21.7555503845215 9.38380718231201 15.4942111968994 36.8814430236816 24.4710235595703 16.1298980712891 16.6778106689453 11.2528018951416 23.1270999908447 16.3662776947021 12.7634782791138 37.5020942687988 19.9149360656738 7.7466344833374 11.4083414077759 31.7373504638672 14.0319290161133 47.9935188293457 12.3136157989502 28.8108253479004 23.4480533599854 11.4569759368896 16.3297882080078 20.8153800964355 40.1006889343262 8.65438652038574 22.6149635314941 31.4413318634033 17.3870372772217 25.8917427062988 11.750732421875 43.5039291381836 23.1436767578125 13.7776718139648 22.136890411377 22.7959442138672 5.49294853210449 28.0877590179443 25.9640522003174 19.3900566101074 16.9108982086182 12.8259754180908 12.9706325531006 22.5936870574951 28.2334518432617 12.1603956222534 24.9349155426025 3.78184103965759 11.2614545822144 14.3505153656006 22.7247314453125 19.5295543670654 17.0647583007812 12.5566120147705 10.4079570770264 22.7777309417725 38.2710609436035 16.4103679656982 43.0182609558105 7.89419364929199 8.24856090545654 18.1780052185059 21.5580081939697 23.273796081543 33.6412124633789 5.59881353378296 4.87731552124023 24.3523559570312 17.9609432220459 3.93495059013367 30.1626071929932 12.7703323364258 7.46688175201416 22.8250522613525 12.965048789978 6.17346954345703 10.8538646697998 58.7627639770508 10.8052396774292 21.0162582397461 20.7238273620605 42.060131072998 21.0687294006348 43.3673133850098 12.6579170227051 39.2310409545898 5.11262845993042 37.0577392578125 16.9372596740723 39.5671005249023 29.590841293335 26.7414493560791 13.2208023071289 27.2867012023926 25.6039390563965 23.3705539703369 25.0611515045166 36.7057952880859 34.6704216003418 9.78942394256592 19.3644618988037 23.788423538208 6.97128295898438 6.02982139587402 29.3406791687012 27.0690746307373 5.93895149230957 27.5287113189697 39.0929069519043 7.74107599258423 32.6268119812012 10.0047836303711 14.4383783340454 11.8468675613403 9.76805782318115 21.8646392822266 13.652904510498 6.67616367340088 9.6108865737915 25.0529479980469 22.6916904449463 15.6845188140869 24.3727684020996 20.4663467407227 21.2300891876221 10.9915571212769 17.956563949585 40.1046714782715 26.0907936096191 27.8626899719238 20.0122184753418 15.9226636886597 14.3259449005127 25.2010536193848 29.098949432373 22.3512783050537 40.4313278198242 10.2633113861084 10.5111436843872 11.8247900009155 50.7294120788574 13.7846260070801 14.8255920410156 17.0284481048584 19.1712989807129 6.79960584640503 21.0724391937256 20.7303314208984 9.50110721588135 20.5845432281494 16.4773902893066 22.0194530487061 10.1155490875244 17.3321380615234 13.2567148208618 27.8500995635986 6.96434593200684 14.5765237808228 12.0876626968384 19.9730453491211 13.0294198989868 21.5867156982422 15.3300924301147 15.7279376983643 43.6812171936035 10.9427042007446 17.3662281036377 8.0893726348877 14.1310386657715 17.9190540313721 22.3822917938232 19.5998153686523 11.4511575698853 4.87330293655396 18.6142845153809 13.2097082138062 15.886773109436 10.8139295578003 16.7729606628418 29.2278881072998 18.3023548126221 25.599760055542 18.367748260498 20.8098659515381 73.6671371459961 11.1084003448486 14.3468427658081 17.5843105316162 10.010931968689 25.1601696014404 39.1872520446777 22.200870513916 32.4562530517578 14.0389089584351 18.1227970123291 14.238335609436 31.4786682128906 23.5195598602295 20.3994426727295 12.7826824188232 26.9524707794189 13.6501836776733 18.658712387085 38.730281829834 26.0749855041504 16.623420715332 15.7050428390503 11.0052785873413 41.3205223083496 34.8479995727539 9.95228672027588 15.531533241272 15.2694473266602

ENSG00000204389.9 148.515930175781 32.2762908935547 73.8949127197266 66.1139221191406 82.671272277832 43.473934173584 68.0092239379883 130.745162963867 162.143676757812 47.9468727111816 48.7654075622559 62.1096382141113 97.0022048950195 231.795455932617 24.123176574707 271.5205078125 341.829284667969 52.2979431152344 119.091781616211 130.671813964844 93.9511184692383 69.8387832641602 116.434669494629 78.8127517700195 38.2073554992676 296.263366699219 64.0691757202148 50.7965354919434 47.8713493347168 87.7416076660156 67.3448638916016 53.6602478027344 80.1500549316406 45.8695030212402 93.6359405517578 88.4624786376953 22.3758316040039 80.1928100585938 62.619457244873 78.1601104736328 70.9128036499023 41.9477348327637 8.67074203491211 61.8678512573242 113.20484161377 65.4485244750977 128.862884521484 228.713928222656 72.5125961303711 94.8568878173828 62.5797843933105 54.4025039672852 198.730178833008 65.4836883544922 45.8608932495117 158.414077758789 114.86792755127 56.1454582214355 98.9324340820312 333.543304443359 88.8213348388672 127.884994506836 95.6831588745117 182.716018676758 104.478775024414 148.961471557617 36.7118873596191 35.0710792541504 84.0685501098633 67.5496139526367 67.9481506347656 97.2073669433594 133.284805297852 120.136161804199 80.2895126342773 100.728523254395 20.9701480865479 18.4371662139893 50.4224510192871 60.9511604309082 48.6943588256836 65.76708984375 176.225555419922 130.915878295898 71.9966888427734 36.4920463562012 227.5673828125 205.282485961914 100.485198974609 69.7105941772461 95.975227355957 91.617561340332 187.02082824707 63.6928482055664 112.463958740234 192.83415222168 72.4584503173828 110.212440490723 108.107368469238 100.03865814209 69.8108825683594 52.9683303833008 83.3893051147461 115.628974914551 176.512802124023 82.0403213500977 112.918663024902 286.396148681641 171.926803588867 178.219161987305 94.4415893554688 176.097381591797 20.7652416229248 127.830703735352 508.515838623047 87.9840240478516 24.7240295410156 112.033599853516 122.517402648926 277.272430419922 78.0871734619141 92.8105926513672 507.277893066406 69.5319137573242 26.5640525817871 272.963043212891 96.2788238525391 772.229187011719 43.188045501709 107.872901916504 113.785919189453 47.9653549194336 127.48713684082 259.138458251953 19.5027236938477 133.438354492188 84.7395782470703 138.765151977539 30.8383293151855 100.175590515137 61.9155158996582 107.842391967773 400.420928955078 55.3411293029785 53.9638328552246 124.606140136719 207.582534790039 160.741744995117 69.7314834594727 93.5612640380859 98.5646514892578 95.5400695800781 41.612003326416 21.9119701385498 31.4010047912598 40.5591430664062 242.247512817383 48.9560775756836 87.6863174438477 215.774200439453 74.6383056640625 119.731819152832 86.5309906005859 45.5642967224121 34.3099594116211 841.966064453125 63.4486961364746 64.247200012207 34.8597030639648 42.0865516662598 121.666702270508 101.455924987793 31.4364109039307 98.9010696411133 48.9707870483398 109.385704040527 82.9032974243164 68.7181091308594 36.8147926330566 89.6925964355469 159.761093139648 136.108016967773 193.886581420898 217.148620605469 74.3121948242188 149.955642700195 79.1081161499023 30.0355548858643 137.355484008789 49.542308807373 50.7631645202637 72.8755264282227 75.1729049682617 73.2708053588867 50.5558891296387 299.018768310547 51.4215927124023 176.824127197266 80.9961776733398 179.708770751953 171.651077270508 771.92138671875 140.995910644531 52.9489479064941 82.2773818969727 74.2751693725586 21.1105632781982 247.428161621094 69.2261734008789 187.353210449219 57.9711265563965 93.3563003540039 109.361541748047 143.514739990234 43.5599555969238 84.4897079467773 102.833419799805 160.123519897461 269.692443847656 37.4218521118164 41.5272407531738 70.8599319458008 209.36198425293 60.187629699707 81.7079849243164 665.567626953125 86.8265380859375 31.5626430511475 124.946441650391 107.957420349121 50.6396255493164 55.1494102478027 47.404712677002 63.7602424621582 80.1137313842773 71.4789199829102 53.5325508117676 37.8519592285156 131.577545166016 33.8995056152344 89.9372329711914 99.6019439697266 78.1199111938477 65.3726501464844 173.429016113281 252.920791625977 54.9119453430176 82.4321975708008 123.724403381348 37.5745162963867 268.616577148438 62.1525917053223 69.895866394043 65.6312103271484 136.449905395508 96.2851486206055

ENSG00000204388.6 167.086975097656 32.7604560852051 90.5380172729492 78.8114166259766 183.753341674805 71.7309799194336 93.5477752685547 81.3777694702148 168.272613525391 41.3152198791504 50.1000518798828 81.4502182006836 112.398574829102 256.98876953125 106.596946716309 267.546417236328 408.696868896484 114.56371307373 129.064331054688 125.008140563965 73.4010925292969 124.377174377441 189.644775390625 92.7845611572266 97.4217147827148 300.006134033203 104.742057800293 57.9047012329102 54.2626457214355 190.926773071289 71.2304611206055 38.682731628418 68.3840103149414 71.5961074829102 103.972480773926 95.9476013183594 21.1768951416016 76.6380920410156 103.806274414062 108.023979187012 97.5808334350586 56.5235824584961 60.3361740112305 62.477367401123 140.90185546875 60.1777153015137 95.9081497192383 239.611267089844 96.5386352539062 128.386169433594 78.6243743896484 62.6611328125 212.023788452148 83.2093124389648 62.3801307678223 251.159088134766 125.479042053223 240.98078918457 120.503883361816 294.966064453125 74.0492706298828 108.810401916504 90.7862014770508 190.542572021484 78.9794845581055 117.738578796387 64.4274139404297 87.8047866821289 85.7607498168945 76.490608215332 97.3497085571289 94.0067138671875 155.90788269043 123.395698547363 106.883285522461 130.962448120117 22.9350643157959 120.243728637695 48.7282295227051 75.767951965332 52.3052139282227 90.150032043457 230.380416870117 142.281402587891 64.4041137695312 48.5112953186035 183.474761962891 106.71166229248 134.013610839844 61.476505279541 82.3775100708008 97.3267517089844 232.205520629883 59.7595062255859 86.5510101318359 247.843002319336 60.0727958679199 123.86595916748 103.972473144531 126.138381958008 61.2116851806641 73.7808685302734 136.280960083008 151.778259277344 214.459945678711 62.0517807006836 89.6569290161133 244.180999755859 110.611801147461 65.8073043823242 103.242546081543 180.898452758789 25.9533596038818 131.417327880859 406.510406494141 61.7824592590332 78.4497756958008 139.902572631836 105.762031555176 209.032287597656 96.0710754394531 68.3520355224609 391.4208984375 102.319068908691 91.2835540771484 308.804351806641 121.751327514648 726.677429199219 46.8273468017578 106.01065826416 99.6444396972656 75.7345275878906 151.794906616211 243.274658203125 19.9970989227295 95.8695220947266 103.112197875977 138.934783935547 94.3986968994141 107.459808349609 140.991897583008 108.114616394043 298.746917724609 66.5859069824219 73.6107940673828 117.278167724609 165.771301269531 240.988891601562 72.7330017089844 123.912200927734 102.287788391113 127.069564819336 57.3254280090332 215.65544128418 45.8118629455566 43.0071105957031 232.713577270508 53.9895401000977 146.917129516602 196.353973388672 93.8827819824219 135.102661132812 87.144416809082 99.8424835205078 27.8512001037598 1025.79626464844 111.207916259766 47.9144668579102 42.1325149536133 42.2369766235352 131.331878662109 108.500480651855 49.0307846069336 94.1048965454102 186.578033447266 107.603073120117 89.5157775878906 68.1904144287109 54.7364921569824 109.058372497559 226.565673828125 97.5111694335938 116.124626159668 126.13166809082 56.5812759399414 236.268188476562 75.535270690918 31.7810363769531 167.799713134766 171.027542114258 57.1783218383789 63.8819999694824 80.3611450195312 87.2399063110352 149.736358642578 278.348724365234 45.3066864013672 144.233322143555 105.124435424805 146.178955078125 197.579818725586 773.23779296875 142.877212524414 46.2741394042969 67.9199447631836 84.3085174560547 36.851490020752 288.396545410156 96.9935989379883 168.560760498047 61.0770149230957 99.4797897338867 134.544540405273 139.542541503906 46.317554473877 88.7894515991211 102.633567810059 115.28205871582 223.378173828125 22.3949356079102 38.2489852905273 84.6937789916992 429.585205078125 82.411491394043 62.0220947265625 704.591064453125 87.6277236938477 44.8457107543945 114.954917907715 96.8050079345703 97.2609024047852 57.4864158630371 49.5327682495117 83.3055877685547 168.453979492188 65.0361557006836 87.0482330322266 41.9691505432129 101.88355255127 92.0484237670898 156.159271240234 108.419845581055 123.538528442383 95.4195022583008 218.158340454102 219.884765625 61.4129180908203 97.8953552246094 107.599784851074 58.527904510498 169.743209838867 65.0235824584961 88.7735824584961 111.001770019531 120.469856262207 132.11311340332

ENSG00000204390.9 2.15706562995911 2.21238017082214 4.74759674072266 1.32904517650604 5.22293329238892 2.56628084182739 3.08417391777039 1.62405097484589 2.91351842880249 1.12425708770752 1.62777388095856 3.26391267776489 4.3635368347168 2.05954098701477 1.21966052055359 3.23398041725159 4.62717819213867 2.99885153770447 2.05176186561584 0.910599410533905 1.54285478591919 2.45953249931335 7.44114398956299 2.42096495628357 2.1236457824707 1.42151379585266 1.92849636077881 2.10227012634277 2.13593077659607 2.71529102325439 3.64230227470398 2.96093821525574 2.80244374275208 2.7232141494751 1.68089234828949 7.50992012023926 1.37932443618774 2.13495850563049 1.84319376945496 2.35992622375488 3.0944676399231 1.54892730712891 3.22989869117737 1.20398306846619 1.21971201896667 1.589359998703 4.37234926223755 3.86582636833191 1.74916458129883 4.09284782409668 3.25824952125549 3.28253507614136 1.47639143466949 3.39528918266296 1.90203583240509 4.04068470001221 2.96374535560608 1.75331950187683 3.36040592193604 6.61269235610962 3.35544800758362 3.09608554840088 4.8484354019165 2.66037964820862 3.12007069587708 3.10603141784668 2.67676949501038 5.6526837348938 1.86344683170319 3.12171196937561 3.15603876113892 2.92027616500854 3.7162458896637 3.24014091491699 4.7484917640686 1.68197107315063 1.76495862007141 2.91812133789062 1.0750504732132 1.13819515705109 2.60817646980286 4.20805788040161 2.63209366798401 1.49898099899292 1.36339962482452 2.57348585128784 3.07895064353943 2.86040306091309 5.99589824676514 2.47284889221191 3.67420125007629 2.52162623405457 9.16708278656006 2.78450155258179 1.40652167797089 4.0134220123291 2.04478693008423 1.85151147842407 3.15981268882751 6.15341663360596 2.09101510047913 1.8246443271637 3.00400638580322 2.41334772109985 6.63056564331055 3.4046037197113 2.16300249099731 4.448890209198 1.75655627250671 3.19124245643616 3.06433153152466 1.78521418571472 0.947531700134277 3.90258288383484 3.03910827636719 1.35031592845917 1.51169908046722 1.96813488006592 8.25215148925781 4.28529834747314 3.59628319740295 2.10663199424744 2.72475528717041 1.83766567707062 5.66417837142944 1.74851143360138 3.01453232765198 2.87256050109863 1.81265580654144 2.37025904655457 3.93627071380615 1.95585906505585 3.74288892745972 3.90548968315125 1.8482518196106 2.73854970932007 12.1894292831421 4.63221168518066 2.01977515220642 2.65662097930908 1.67863643169403 1.86123216152191 7.83143663406372 2.78776097297668 3.57801246643066 1.90816211700439 2.7412748336792 3.48578095436096 2.2908022403717 1.59829294681549 6.44745111465454 3.58357572555542 1.84685099124908 8.27129936218262 0.852364778518677 2.24460196495056 6.44042825698853 2.47926568984985 2.47400403022766 3.06204080581665 3.27978825569153 3.66797828674316 2.01219129562378 2.57122850418091 4.89712476730347 3.52787137031555 3.42476892471313 3.37470269203186 2.55527234077454 2.68664598464966 3.46994948387146 0.822224318981171 3.76523113250732 3.0134801864624 2.84291815757751 1.83426141738892 2.74541163444519 3.80166220664978 2.74845838546753 4.55356788635254 1.87964248657227 2.06860971450806 1.69994223117828 5.72904205322266 5.70288562774658 2.98117518424988 1.19160687923431 1.7722133398056 2.08211183547974 2.78452706336975 2.44455862045288 2.52376532554626 2.89809513092041 3.80682492256165 2.8129403591156 3.40879487991333 2.02482295036316 2.69590282440186 3.44183325767517 4.43941879272461 1.34307539463043 5.00253629684448 5.3419394493103 1.11165523529053 3.72149443626404 3.47886562347412 1.50763714313507 3.30524969100952 1.03277242183685 3.8660135269165 1.46667587757111 3.87371730804443 2.65190744400024 2.93985962867737 1.66678583621979 1.03554391860962 1.35632002353668 1.53548145294189 4.39135980606079 2.54152178764343 1.34874451160431 2.22450876235962 1.92486500740051 3.63577055931091 1.07143414020538 3.36053133010864 5.16319131851196 4.10744476318359 3.13554954528809 3.17980861663818 4.04615879058838 2.65542578697205 2.68196105957031 3.04392719268799 5.8585991859436 3.73059129714966 2.05083417892456 2.97850155830383 2.39867544174194 3.27112865447998 2.39166927337646 3.26156044006348 3.72503209114075 6.23013210296631 3.97717332839966 4.10045194625854 2.30442190170288 3.41280674934387 0.929464340209961 2.23406934738159 3.11783194541931 3.0627269744873 2.51695251464844 2.2585916519165 4.1687331199646 3.26717901229858

ENSG00000126803.9 11.3038158416748 5.16780948638916 12.0459032058716 8.28615665435791 7.91703176498413 9.33497905731201 1.27166080474854 13.6263637542725 2.53006196022034 0.866399943828583 2.68089890480042 4.13229274749756 12.2101249694824 3.35298109054565 2.91901206970215 16.6494693756104 3.98307371139526 2.46650838851929 3.61411237716675 3.22402334213257 14.3304462432861 30.9898338317871 4.87884140014648 5.01634216308594 1.64592289924622 1.14764404296875 4.96094179153442 5.2697696685791 9.94677639007568 5.72639894485474 4.57422685623169 11.5107688903809 15.6437330245972 1.8707731962204 2.26175117492676 11.7853765487671 1.53298449516296 1.7601979970932 1.70066630840302 4.44695281982422 2.46026182174683 2.99569320678711 3.98034882545471 6.02926206588745 2.10585474967957 3.44198179244995 5.82237768173218 3.6872227191925 8.39895248413086 2.5265154838562 2.76716327667236 3.6673378944397 2.18162822723389 1.46231234073639 3.60165333747864 18.9844627380371 4.42187738418579 49.2883110046387 15.0095186233521 3.2340772151947 3.27016139030457 16.3717021942139 1.92081665992737 7.34429740905762 4.20901107788086 3.11106014251709 4.69170141220093 7.83325147628784 4.41240882873535 4.18853569030762 4.39181613922119 16.6036014556885 5.25830030441284 4.22885608673096 9.02795219421387 4.90477657318115 4.54065704345703 8.17877101898193 1.9462685585022 10.0077648162842 3.50035691261292 22.6319980621338 1.73863077163696 14.8622903823853 2.36913061141968 6.09703588485718 2.14852952957153 3.52695536613464 1.60309720039368 7.32058668136597 22.6839923858643 17.8334922790527 1.43654763698578 8.1128978729248 4.86475849151611 7.22941923141479 2.47867631912231 2.33910298347473 28.508716583252 1.56731855869293 9.41553115844727 1.80438029766083 6.58125448226929 4.67385196685791 1.97056078910828 2.08296203613281 4.34921932220459 1.19584131240845 3.29921984672546 2.41348052024841 17.0855312347412 2.46729922294617 1.12714838981628 5.78584766387939 5.86897563934326 3.25151181221008 2.58884215354919 2.21773624420166 13.5353908538818 1.52269756793976 10.9622917175293 4.72864818572998 4.54410791397095 3.62031698226929 10.1257400512695 5.06570148468018 44.1432075500488 1.99277794361115 6.12462949752808 2.29374718666077 14.81272315979 3.64946436882019 6.68454790115356 11.3451385498047 4.34644222259521 6.76072263717651 27.4979763031006 4.14602327346802 3.20945620536804 12.5188970565796 4.29776811599731 3.20166063308716 7.54039001464844 7.31562566757202 8.79308032989502 2.74283862113953 32.7730827331543 1.77581059932709 3.97290349006653 13.9684257507324 4.97075986862183 7.37000799179077 6.21587610244751 6.54023408889771 1.4198145866394 6.02055025100708 3.26053929328918 5.65744018554688 7.8294095993042 5.43454742431641 4.93179178237915 3.388099193573 2.3383264541626 3.13111186027527 7.09287118911743 6.70483016967773 5.02029705047607 10.5294742584229 6.61514949798584 2.13271164894104 9.16830730438232 2.9087131023407 2.69879531860352 2.0771017074585 4.15818738937378 4.84648895263672 5.7018518447876 5.75411701202393 6.37203311920166 2.85868263244629 2.23663902282715 13.1650619506836 3.07424426078796 2.46521496772766 13.6869249343872 9.64570713043213 1.61706912517548 2.29249620437622 4.69907855987549 2.45242762565613 5.84387254714966 2.03753542900085 4.46282577514648 1.6923656463623 7.65601491928101 42.6071510314941 4.82688045501709 6.32483816146851 3.34875726699829 16.7018642425537 7.10918140411377 6.60885524749756 19.3385391235352 2.430992603302 4.17899942398071 4.93768119812012 1.89953052997589 8.68643569946289 2.47612714767456 6.52726316452026 55.3874244689941 6.76071262359619 8.29761409759521 3.45757484436035 4.32729816436768 3.98256897926331 3.11553406715393 6.4175181388855 5.97979021072388 7.15366458892822 1.79705691337585 2.52925038337708 5.79867553710938 7.45271110534668 4.40726661682129 7.15080785751343 6.20099782943726 3.89390778541565 14.4243488311768 7.84158277511597 3.75547528266907 2.04982233047485 6.7594518661499 6.96286725997925 17.0734272003174 6.88177251815796 4.52949190139771 5.65566778182983 5.72732734680176 4.15618753433228 3.27349710464478 7.01244354248047 3.05008387565613 5.37896871566772 11.9252443313599 18.1698913574219 4.25904846191406 8.66964054107666 2.3726921081543 3.88413405418396 14.722261428833 3.68104720115662 8.92094230651855 87.2928237915039 25.1809501647949 8.10009384155273

ENSG00000170606.12 129.568939208984 53.1562271118164 114.75218963623 59.8983612060547 191.576400756836 55.9911079406738 84.2099151611328 71.2674942016602 52.6518325805664 45.4866180419922 48.671070098877 48.2962226867676 114.834548950195 74.7207183837891 83.5699691772461 67.7432098388672 85.3945770263672 66.5138168334961 66.0911483764648 43.4378356933594 40.3263969421387 73.8406524658203 251.640716552734 49.0946884155273 102.057571411133 105.382789611816 75.7927474975586 51.0391044616699 71.3477249145508 117.468002319336 133.453765869141 65.0198593139648 68.0967407226562 26.2280387878418 34.9714279174805 85.4720993041992 36.5885581970215 58.7856140136719 51.4051513671875 31.384672164917 91.8860321044922 42.2299537658691 135.114395141602 86.5183639526367 57.009838104248 59.3198471069336 31.7924118041992 75.2533187866211 44.5797386169434 96.2296600341797 65.964241027832 68.7439422607422 46.0386734008789 65.8872299194336 69.7130813598633 139.734725952148 109.761978149414 122.576507568359 66.3048248291016 136.433670043945 80.3371810913086 56.1011848449707 49.0453300476074 90.1424179077148 83.7271575927734 72.442268371582 64.7943954467773 58.4486503601074 57.6681900024414 59.4207572937012 86.5392227172852 135.698974609375 87.8533020019531 101.888427734375 108.518493652344 78.0809020996094 46.7962303161621 91.1434631347656 79.2117919921875 99.0461273193359 80.8901748657227 87.322883605957 99.3678359985352 86.5562896728516 110.706108093262 82.1542816162109 94.0484313964844 59.460807800293 45.3446884155273 79.6461868286133 49.6655082702637 154.480972290039 76.8925552368164 129.944351196289 112.671501159668 78.1913986206055 73.612434387207 73.8025588989258 80.5519332885742 39.4485473632812 44.1501350402832 81.9421844482422 129.812408447266 74.0063400268555 72.6269989013672 87.9677658081055 60.7520141601562 55.0344924926758 64.706657409668 22.9454727172852 78.7437896728516 75.2503967285156 82.5016708374023 90.61669921875 88.5263290405273 74.1809539794922 78.1241226196289 41.69970703125 142.943695068359 95.1257171630859 115.842384338379 34.0844535827637 120.701988220215 61.9111289978027 112.438301086426 104.049240112305 66.0092315673828 68.3622894287109 100.983032226562 38.1314582824707 84.386100769043 65.5974349975586 74.3014297485352 60.1856231689453 54.5940628051758 65.5544509887695 73.5616073608398 69.4001541137695 50.7971267700195 64.5718154907227 107.749397277832 60.3236274719238 161.330612182617 66.0238647460938 89.5424575805664 95.1890106201172 106.584014892578 93.9067687988281 67.2386093139648 45.3600616455078 157.399917602539 107.631843566895 86.9427185058594 68.8827743530273 79.2301559448242 39.0213775634766 153.413146972656 71.4786987304688 93.6205520629883 53.9167366027832 83.7814636230469 56.6666526794434 91.0918655395508 47.5488166809082 40.0569953918457 73.5968322753906 75.6012191772461 47.1132278442383 76.4418411254883 75.9956283569336 62.7556076049805 25.4097328186035 46.2075042724609 124.153511047363 45.1873741149902 46.2767333984375 74.8692092895508 70.980827331543 64.1158447265625 79.7715148925781 27.0926895141602 108.050559997559 37.3752059936523 100.648170471191 53.7186965942383 84.1681060791016 66.197883605957 26.7426872253418 66.5450439453125 61.7859420776367 65.1326065063477 67.5542907714844 67.8820343017578 77.8514404296875 109.530090332031 55.9871673583984 55.7874641418457 93.0204315185547 68.2844619750977 89.7867584228516 54.7242088317871 106.185913085938 76.8639831542969 32.6667404174805 67.152702331543 91.3966064453125 104.018775939941 80.9222640991211 90.2628707885742 71.6360015869141 67.6225967407227 55.681079864502 56.9699363708496 79.5676193237305 70.419677734375 51.9458999633789 39.8183555603027 61.6285629272461 48.7617645263672 49.6429443359375 61.3946380615234 46.9047203063965 56.7685165405273 48.1259574890137 90.0149688720703 76.7411727905273 90.0274124145508 74.1411285400391 90.505241394043 83.8472366333008 61.7347679138184 77.0707168579102 59.4900131225586 42.7899932861328 133.266143798828 73.1060333251953 51.3263473510742 36.5110359191895 67.7373199462891 68.2639923095703 67.1360397338867 112.279891967773 65.5632019042969 71.443603515625 34.9100151062012 77.0298538208008 76.6796035766602 56.6367874145508 69.807975769043 70.6762084960938 42.9064292907715 78.3469161987305 55.4188461303711 106.105308532715 55.3536491394043 62.945915222168

ENSG00000044574.7 861.919982910156 470.944854736328 774.506103515625 327.854522705078 1247.30883789062 757.710021972656 1258.69482421875 359.639282226562 498.970581054688 290.853454589844 730.449340820312 600.453857421875 697.896789550781 1697.3115234375 813.365661621094 1301.85620117188 1219.18322753906 619.591674804688 1046.44885253906 469.145904541016 501.503967285156 918.38720703125 1821.25537109375 740.926879882812 629.864868164062 636.240661621094 788.377380371094 518.309265136719 850.979125976562 1046.66943359375 430.672760009766 745.357849121094 662.938110351562 787.946228027344 261.152526855469 601.286743164062 398.305084228516 942.360900878906 563.5 596.474548339844 521.988220214844 462.813720703125 622.948913574219 581.509521484375 418.374359130859 423.872039794922 663.86376953125 1595.35485839844 631.210571289062 790.628234863281 1084.50305175781 520.439147949219 607.964172363281 794.156066894531 1010.80297851562 1221.90563964844 593.192810058594 1315.07800292969 460.129333496094 990.195495605469 1018.90612792969 499.629302978516 473.169403076172 999.326232910156 987.762756347656 1002.11938476562 707.49072265625 690.301879882812 709.655517578125 584.242614746094 858.42236328125 1101.99975585938 1139.84033203125 671.717163085938 775.819885253906 548.980590820312 434.460296630859 623.845886230469 538.906555175781 1295.20141601562 760.650573730469 1851.98876953125 936.955200195312 944.983764648438 859.830017089844 1126.31616210938 1122.87707519531 841.672241210938 594.535522460938 1082.70520019531 881.130859375 785.469177246094 847.992370605469 1278.6865234375 575.916625976562 839.188049316406 481.74365234375 1139.94055175781 752.231811523438 1305.99914550781 393.826293945312 1283.17077636719 1609.287109375 1238.96252441406 1067.10791015625 1097.15441894531 729.239624023438 729.2353515625 642.673217773438 511.584991455078 760.081298828125 839.0185546875 453.520690917969 954.811645507812 1077.56115722656 328.360046386719 1012.86755371094 612.753356933594 711.170532226562 730.618408203125 2248.38256835938 573.475769042969 1493.39782714844 656.773071289062 1060.60339355469 1079.77844238281 868.205261230469 572.642456054688 774.458801269531 395.648803710938 1000.58428955078 482.873107910156 935.054138183594 653.590270996094 434.551940917969 757.051025390625 1353.00024414062 973.214782714844 407.639526367188 959.486450195312 963.801879882812 646.886474609375 1281.13671875 1009.078125 911.488647460938 333.918609619141 683.859436035156 680.2724609375 699.938903808594 497.512573242188 867.780700683594 1069.61767578125 930.583618164062 899.356262207031 1039.86694335938 443.248046875 727.115112304688 678.622314453125 595.997253417969 561.386840820312 1021.14483642578 485.980438232422 609.858825683594 627.708618164062 469.025634765625 915.637329101562 510.356201171875 278.811126708984 768.506225585938 620.111511230469 953.816650390625 723.6337890625 396.941528320312 742.159362792969 819.842956542969 484.497955322266 775.542541503906 784.864929199219 674.56982421875 2378.1259765625 826.239318847656 1106.72705078125 842.119689941406 1089.4462890625 458.416473388672 806.805053710938 1137.56066894531 398.397918701172 916.299438476562 468.6572265625 568.9609375 966.546264648438 740.746276855469 672.590209960938 1362.11853027344 634.035034179688 708.647705078125 989.039855957031 818.242309570312 518.04931640625 610.981811523438 1409.54370117188 637.909362792969 356.047393798828 502.825897216797 661.119567871094 1161.5224609375 660.296142578125 889.281982421875 1125.32751464844 741.767822265625 402.8388671875 692.798828125 1171.41235351562 523.746337890625 259.262878417969 928.934509277344 895.747497558594 554.453918457031 322.977966308594 1145.19873046875 599.142639160156 858.271240234375 314.986785888672 761.942810058594 1071.12890625 514.303100585938 598.975708007812 1219.95520019531 648.7060546875 930.860595703125 1157.55773925781 1063.26440429688 906.780578613281 805.946228027344 1133.06335449219 809.894653320312 594.697631835938 625.754638671875 1078.25158691406 592.909423828125 951.374572753906 663.902893066406 489.074676513672 311.062866210938 644.287902832031 549.814880371094 711.804382324219 613.724914550781 724.986694335938 460.362548828125 943.881164550781 884.396423339844 671.980895996094 802.567626953125 542.345092773438

ENSG00000173110.7 32.9578552246094 1.18690323829651 2.4277811050415 5.74160289764404 16.0877418518066 14.5597581863403 10.8761320114136 9.08284854888916 52.9976768493652 3.11624503135681 3.73760485649109 9.41179656982422 5.21699905395508 3.28124141693115 10.6399183273315 12.7441720962524 19.3279876708984 20.6509246826172 30.5697612762451 6.13581943511963 14.0711479187012 19.8732280731201 2.83878660202026 12.82399559021 7.92951965332031 135.1103515625 8.52268218994141 4.74267435073853 4.92732524871826 32.7758903503418 8.68458461761475 7.27631568908691 33.0188903808594 3.8179669380188 64.6935958862305 8.20438861846924 7.15317249298096 12.1027202606201 14.7227468490601 4.88757658004761 10.3213367462158 6.64777803421021 7.14605522155762 12.1730298995972 5.85802745819092 22.4495868682861 5.45456838607788 12.6482934951782 9.20349979400635 8.07719039916992 8.73996925354004 23.610746383667 10.0679397583008 10.2084817886353 8.53742122650146 15.8756313323975 6.73969125747681 7.55920648574829 9.54747581481934 91.6489028930664 35.7666969299316 21.5013942718506 6.76845598220825 3.4781653881073 7.09623765945435 7.04106760025024 5.34604978561401 7.60548305511475 106.231117248535 9.7693395614624 27.406608581543 7.86819982528687 5.42548036575317 16.8537483215332 6.89498329162598 13.1390676498413 3.85392475128174 8.08852577209473 13.6816911697388 1.09911918640137 14.0812492370605 8.54907989501953 4.47156190872192 16.2784938812256 16.4870529174805 4.63891696929932 12.7061777114868 6.30348443984985 45.3783683776855 12.0394506454468 3.00550746917725 44.6773643493652 6.29146957397461 4.13410520553589 47.2552108764648 60.9438438415527 3.92794060707092 12.0173425674438 28.2127094268799 5.26811790466309 14.2700157165527 16.2461338043213 5.37870407104492 7.51772165298462 14.6492824554443 19.9382419586182 13.9468517303467 53.066478729248 4.38340950012207 2.95265865325928 4.18944692611694 24.8422012329102 2.02013325691223 7.20221567153931 40.1474342346191 29.5933589935303 8.11000061035156 3.9750394821167 3.94084501266479 19.4859027862549 3.64628100395203 16.1363334655762 14.6932811737061 12.5569410324097 17.6897640228271 100.790542602539 14.01611328125 120.077445983887 6.47126626968384 19.0269412994385 25.679666519165 4.0357084274292 49.8428802490234 2.42855858802795 7.42132759094238 9.31373500823975 9.67927932739258 6.1903223991394 2.94004702568054 11.6416034698486 15.9587430953979 13.3967885971069 195.164672851562 6.09353542327881 10.2375526428223 66.3470611572266 4.86163949966431 2.77208948135376 30.6485233306885 5.80431842803955 13.4097509384155 11.6578683853149 6.91792964935303 29.7134704589844 4.49326086044312 22.9265213012695 37.0233573913574 4.65528917312622 6.83876276016235 66.8375930786133 5.72211074829102 29.5650882720947 16.3724994659424 13.3156156539917 2.58166289329529 369.363891601562 4.22837257385254 10.9189510345459 23.7947845458984 5.65156412124634 24.9587898254395 25.0843982696533 19.0393905639648 6.50186252593994 7.13564443588257 7.18474388122559 10.1110200881958 17.6668815612793 8.36995410919189 34.0147666931152 7.68096828460693 16.3881530761719 10.3601989746094 5.02302742004395 7.25935554504395 45.6908569335938 2.00915384292603 11.3695230484009 18.6821041107178 11.491153717041 7.58774995803833 3.06896662712097 18.5800724029541 20.7925071716309 1.29949700832367 22.3960342407227 9.3565092086792 4.28904104232788 9.04873561859131 15.0915460586548 53.0891227722168 356.914733886719 13.967827796936 13.7168216705322 6.41737461090088 7.4654016494751 11.6829690933228 44.7379570007324 1.79763090610504 4.45038747787476 6.46962261199951 7.18195056915283 21.1533298492432 19.7724914550781 10.5702610015869 1.40739619731903 3.28422164916992 30.1046409606934 30.878137588501 5.93184947967529 8.01501655578613 49.5703430175781 67.2634811401367 35.7431602478027 28.1132202148438 541.492919921875 6.68453645706177 6.91676807403564 24.541618347168 3.05268406867981 41.2765731811523 7.97770118713379 28.4145336151123 7.71145391464233 8.77256202697754 30.730110168457 4.57021379470825 6.4796142578125 1.48739504814148 14.0392274856567 28.6440868377686 6.05214595794678 17.3195934295654 4.77479553222656 25.359582901001 8.97721004486084 15.9967412948608 28.2561473846436 70.1370239257812 6.03939151763916 32.0952415466309 15.8136968612671 36.3831100463867 2.47495126724243 9.49997520446777 24.1249408721924

ENSG00000109971.12 1178.38586425781 370.216857910156 748.607116699219 796.087219238281 1565.59448242188 567.842224121094 544.411376953125 845.081848144531 494.125122070312 443.162689208984 469.154663085938 527.095947265625 803.388916015625 408.733673095703 707.270874023438 684.737487792969 752.313293457031 692.698120117188 480.414672851562 249.294677734375 287.865936279297 635.077453613281 1785.71166992188 775.965209960938 546.205078125 782.912658691406 532.954711914062 353.719787597656 905.47607421875 1083.00085449219 1704.85290527344 756.658569335938 788.25537109375 339.501678466797 697.11865234375 806.967224121094 710.801147460938 489.207122802734 411.183502197266 414.166107177734 707.828979492188 719.720764160156 905.794921875 454.562347412109 407.737518310547 1127.75146484375 342.431823730469 472.666839599609 531.836120605469 540.263671875 571.869445800781 732.748474121094 528.280944824219 284.032287597656 593.779113769531 1040.32067871094 935.639404296875 825.858337402344 836.389953613281 1261.75805664062 629.146911621094 268.784118652344 901.574340820312 688.92724609375 541.834411621094 723.844848632812 485.571960449219 784.060852050781 448.659698486328 536.554748535156 1005.58801269531 1096.35815429688 755.974853515625 990.315002441406 632.454162597656 1263.20788574219 1089.30456542969 722.173950195312 1001.44708251953 452.279602050781 763.949340820312 723.195556640625 727.875244140625 1026.77014160156 1027.86645507812 738.485900878906 689.756042480469 564.30712890625 511.384429931641 912.580688476562 350.717498779297 752.8076171875 661.083740234375 999.916442871094 1056.10693359375 686.582092285156 567.480529785156 716.945678710938 938.460021972656 443.881958007812 350.232208251953 907.829223632812 1172.90478515625 377.965881347656 552.391662597656 709.832702636719 776.529113769531 477.229370117188 123.734352111816 194.748809814453 634.646179199219 703.748596191406 1054.59191894531 1361.333984375 840.611999511719 133.396881103516 485.570739746094 301.632110595703 834.05712890625 630.364807128906 978.783142089844 226.373107910156 1190.51025390625 852.062866210938 844.876525878906 529.960693359375 778.126037597656 640.588317871094 722.534729003906 400.036956787109 979.164367675781 945.789428710938 845.650085449219 600.052185058594 1187.53161621094 395.493316650391 933.952087402344 431.832336425781 634.286437988281 592.682495117188 413.103637695312 484.346557617188 700.849731445312 440.400512695312 1238.63854980469 844.357604980469 1290.89208984375 442.374725341797 629.320190429688 364.537017822266 1148.95935058594 571.4111328125 375.585266113281 395.528930664062 222.485885620117 266.829772949219 867.473449707031 684.599182128906 754.429809570312 290.675872802734 728.662902832031 642.004943847656 889.56103515625 308.352111816406 525.849365234375 667.371276855469 594.096008300781 385.643585205078 888.287475585938 653.995666503906 710.664978027344 427.176574707031 535.54541015625 667.716796875 279.114135742188 388.586090087891 536.068603515625 836.285217285156 493.639007568359 1123.69946289062 308.935729980469 880.031677246094 843.026428222656 693.889343261719 564.47900390625 744.902465820312 733.579162597656 827.912414550781 656.731689453125 391.419128417969 597.220886230469 512.815795898438 1051.47509765625 487.304748535156 745.3984375 585.347290039062 862.8095703125 686.418029785156 979.173278808594 896.261474609375 575.519165039062 713.056274414062 549.25 68.0250701904297 613.993103027344 622.943420410156 1908.59606933594 757.950744628906 866.868835449219 461.602416992188 780.666259765625 540.672729492188 663.08642578125 776.550964355469 437.978759765625 590.504272460938 140.134262084961 737.72216796875 439.508361816406 269.620697021484 384.704559326172 380.776611328125 817.144958496094 1345.90087890625 856.660766601562 1442.94250488281 813.636657714844 472.382354736328 507.629486083984 818.908996582031 943.29345703125 769.365051269531 1067.49230957031 516.726196289062 679.966369628906 469.073333740234 518.794067382812 523.704040527344 414.479949951172 748.33837890625 658.638488769531 1455.85400390625 787.966247558594 518.564636230469 314.585052490234 950.651611328125 1221.6962890625 352.570007324219 1569.41003417969 670.390808105469 485.381225585938 439.369262695312 639.546081542969 687.073486328125 599.033630371094 493.689727783203

ENSG00000080824.17 347.992553710938 486.106353759766 496.745758056641 718.0986328125 993.38037109375 392.599182128906 584.182800292969 966.083190917969 284.95068359375 321.271911621094 242.229721069336 258.937164306641 971.685974121094 518.323059082031 485.92041015625 738.08349609375 861.009216308594 461.034240722656 573.823547363281 650.015441894531 284.441345214844 864.495544433594 981.322448730469 421.641723632812 347.171783447266 724.671142578125 835.922790527344 293.445159912109 544.649230957031 940.576477050781 935.308959960938 476.82373046875 897.568542480469 571.812255859375 564.415710449219 669.561462402344 396.182678222656 459.085235595703 464.960113525391 242.073318481445 470.483245849609 413.607757568359 710.906555175781 378.130157470703 695.616027832031 703.623107910156 349.437316894531 807.115173339844 376.932220458984 589.674072265625 1127.48107910156 550.683044433594 315.825927734375 542.998107910156 328.409393310547 558.545288085938 1378.56115722656 876.75927734375 435.585205078125 1106.34069824219 555.8349609375 434.170043945312 698.429138183594 465.948455810547 400.390045166016 1453.33837890625 394.477264404297 591.192260742188 433.503387451172 471.462127685547 762.47607421875 862.506896972656 674.302062988281 682.402648925781 693.673217773438 1040.98767089844 333.433868408203 672.513549804688 683.066223144531 295.662841796875 359.962615966797 630.239868164062 1075.10729980469 704.3125 1759.78784179688 1049.58251953125 1390.59582519531 350.755889892578 1071.521484375 1107.23706054688 571.800842285156 928.868225097656 635.233154296875 2000.62951660156 1143.96606445312 431.453857421875 326.533355712891 894.191467285156 1511.62524414062 450.501983642578 360.688751220703 562.950256347656 877.585205078125 331.228393554688 512.03271484375 532.412231445312 634.023559570312 541.131225585938 393.838714599609 469.641235351562 465.629730224609 656.593994140625 401.757415771484 1193.70739746094 1251.40441894531 256.648651123047 709.749084472656 430.620208740234 807.331970214844 633.190063476562 715.528930664062 175.958908081055 1310.99072265625 534.132385253906 1305.69970703125 502.092437744141 732.4033203125 455.363708496094 670.093505859375 152.178344726562 1001.73834228516 707.718933105469 506.778778076172 671.756103515625 301.981781005859 497.718658447266 774.919189453125 423.417266845703 273.188995361328 405.853698730469 586.850158691406 599.117980957031 1638.47314453125 502.719879150391 684.367065429688 353.487030029297 713.932678222656 1437.20495605469 591.310241699219 295.192047119141 968.061950683594 518.1171875 641.32666015625 427.306427001953 574.081237792969 439.035034179688 566.000183105469 435.065734863281 1143.22705078125 573.781005859375 833.571594238281 261.886566162109 612.212829589844 323.636383056641 306.111022949219 488.744903564453 543.151000976562 329.134460449219 546.702453613281 513.064453125 506.285064697266 768.844177246094 264.773223876953 480.270660400391 439.694763183594 390.291778564453 528.079650878906 591.222473144531 996.878173828125 871.529174804688 335.116027832031 1620.67053222656 411.402435302734 1177.40075683594 637.420288085938 1109.91528320312 477.058837890625 417.428863525391 423.875091552734 367.721160888672 469.350433349609 1243.16870117188 615.376403808594 382.732147216797 473.828582763672 1081.66528320312 510.416625976562 1096.83459472656 592.622863769531 800.164001464844 574.498901367188 895.490051269531 647.419494628906 186.195861816406 487.509857177734 727.098388671875 732.88671875 695.080871582031 608.803833007812 474.216522216797 997.694580078125 715.472473144531 718.238159179688 762.523315429688 367.452087402344 281.078826904297 276.090148925781 410.967041015625 410.918823242188 316.00634765625 347.660430908203 546.220947265625 590.518737792969 370.5732421875 583.539916992188 1638.99877929688 380.087432861328 384.477569580078 1042.4326171875 495.124084472656 380.038879394531 681.325256347656 707.236450195312 415.712341308594 897.790771484375 498.940734863281 790.215087890625 316.745513916016 716.705871582031 491.436889648438 501.163879394531 1027.77355957031 1056.45666503906 877.300170898438 249.983840942383 492.567077636719 667.64306640625 478.448822021484 1298.38317871094 559.998474121094 954.493896484375 331.492767333984 472.512268066406 859.726135253906 690.386779785156 339.790496826172

ENSG00000096384.18 1930.9267578125 952.720703125 1126.79797363281 967.062438964844 3013.5537109375 1106.78002929688 1500 1192.03002929688 1234.55700683594 1574.97998046875 849.222351074219 990.439758300781 2234.38671875 1817.77844238281 1604.14807128906 1648.05944824219 2398.00415039062 1153.193359375 2463.19677734375 1264.78234863281 1014.80755615234 1606.7705078125 2844.05786132812 1046.79052734375 1251.1083984375 1322.61706542969 1853.9423828125 660.503295898438 1562.58459472656 2281.00463867188 2231.11108398438 809.117248535156 1246.45166015625 2231.6689453125 589.610412597656 1715.15844726562 716.743530273438 837.513000488281 1435.62329101562 591.697509765625 2146.54711914062 1021.37390136719 1004.7177734375 838.063537597656 1156.63879394531 851.657165527344 1412.56164550781 3648.95092773438 890.003601074219 1992.78149414062 1518.83068847656 1511.07312011719 1367.17895507812 1419.22790527344 1495.71862792969 3065.5634765625 3218.4306640625 2636.61547851562 1525.04748535156 3685.69018554688 1348.85864257812 802.628723144531 1455.48303222656 1837.43090820312 1173.17443847656 2242.21997070312 1173.3828125 1167.74914550781 1093.06787109375 1233.46850585938 2071.72387695312 1257.42102050781 2588.08813476562 1856.62097167969 1423.326171875 2044.26110839844 567.534851074219 2051.45629882812 1630.04577636719 4813.8017578125 992.558715820312 1257.71667480469 2068.296875 1335.77844238281 2442.03491210938 940.7392578125 2283.18627929688 855.6796875 1613.71398925781 2079.90844726562 808.195556640625 2096.95971679688 6358.8984375 2406.56982421875 2727.97021484375 2990.32592773438 1160.17822265625 1489.43542480469 1299.908203125 2005.77624511719 706.827941894531 1071.35791015625 2496.27856445312 1142.66540527344 1745.51171875 1213.73291015625 1691.20092773438 1886.31286621094 762.251953125 993.198181152344 1580.77709960938 1913.6318359375 732.621337890625 2043.75939941406 2771.45483398438 840.9208984375 1222.07861328125 1129.13012695312 3418.39135742188 2782.99926757812 2167.37451171875 1204.28759765625 2860.75756835938 1247.71838378906 2141.21630859375 1463.01184082031 1084.94360351562 1317.78137207031 1192.013671875 1007.81274414062 1660.79064941406 995.151794433594 1574.45239257812 2891.48315429688 711.311401367188 943.920959472656 1152.62512207031 1826.81213378906 1271.42199707031 996.004577636719 2372.70971679688 2042.48718261719 2983.79248046875 1162.75622558594 1859.80737304688 741.401672363281 2201.2021484375 3733.998046875 1123.501953125 907.699523925781 2856.935546875 1774.79797363281 1593.57531738281 2235.7470703125 1330.18725585938 603.950500488281 1429.75646972656 1029.78686523438 1588.42993164062 942.400085449219 1753.27770996094 992.762451171875 1275.65014648438 994.4853515625 706.792724609375 1040.15063476562 1724.65979003906 711.484008789062 1156.29467773438 1115.2314453125 1041.14489746094 605.761901855469 827.078308105469 2108.67822265625 1164.99108886719 1200.30419921875 1392.30456542969 1625.08728027344 817.352661132812 1460.12023925781 750.149475097656 1772.70349121094 2058.99951171875 2068.87841796875 802.90478515625 2077.87451171875 1074.68786621094 389.420104980469 1459.95617675781 891.765258789062 968.395446777344 1790.07995605469 2946.96557617188 1702.28857421875 1075.98608398438 3223.82666015625 1032.07373046875 1637.41552734375 1298.71228027344 1573.29760742188 958.488159179688 1401.06579589844 1408.94812011719 407.901916503906 879.872192382812 1476.37158203125 2253.63623046875 1382.20739746094 1238.94067382812 888.270385742188 1017.33575439453 1391.5244140625 1491.64855957031 2999.7421875 1262.44909667969 829.0478515625 869.003540039062 911.525329589844 1370.0283203125 652.893249511719 868.725158691406 975.732971191406 1598.12585449219 1060.64184570312 1011.23443603516 2192.46313476562 1275.01171875 1398.74853515625 1608.20178222656 1302.453125 1573.11779785156 1073.67700195312 1611.17810058594 853.463134765625 2053.65576171875 1303.82604980469 1795.73388671875 703.540466308594 950.890563964844 1762.63830566406 1844.99438476562 2713.20068359375 1512.26867675781 1241.51538085938 667.377624511719 1521.19470214844 878.532836914062 1160.21850585938 1057.40539550781 1214.32019042969 1388.99438476562 1510.45788574219 878.699035644531 1610.65319824219 1640.07641601562 1231.29443359375

ENSG00000090339.7 395.561492919922 126.910339355469 114.536827087402 38.6059188842773 229.093704223633 321.582824707031 503.435424804688 441.416198730469 643.288940429688 170.460556030273 35.1755180358887 116.204002380371 21.3099098205566 185.955047607422 511.226867675781 50.2522735595703 548.030944824219 487.217803955078 728.333312988281 30.3791332244873 465.78076171875 214.972640991211 76.5065765380859 423.088958740234 1108.09228515625 456.649291992188 619.956298828125 237.845291137695 204.757507324219 290.349761962891 228.497314453125 178.405349731445 500.76123046875 132.5751953125 116.28303527832 1271.18298339844 199.513412475586 127.755386352539 588.57080078125 135.682891845703 1324.30627441406 216.576171875 373.5859375 362.813903808594 26.1549282073975 275.2666015625 244.23503112793 131.03157043457 152.330017089844 339.178314208984 79.1521911621094 260.737243652344 274.29150390625 33.6032218933105 1039.43664550781 74.0141525268555 16.2606258392334 54.5328407287598 1181.52282714844 386.385406494141 320.054046630859 106.282814025879 241.398086547852 202.251052856445 61.4933967590332 101.621978759766 389.436614990234 530.139587402344 337.386657714844 254.850402832031 508.948699951172 292.298278808594 203.427474975586 108.09977722168 263.209686279297 175.921081542969 62.299877166748 144.707244873047 305.807861328125 405.166076660156 508.344024658203 278.919494628906 202.814682006836 690.415832519531 70.8510437011719 71.9351577758789 197.894180297852 21.5837936401367 286.913269042969 676.202697753906 33.6872215270996 175.997924804688 250.279907226562 163.249740600586 390.871795654297 690.345092773438 557.40869140625 230.08869934082 228.862426757812 65.4505462646484 265.960327148438 387.231140136719 77.367919921875 240.838150024414 735.371826171875 393.079681396484 373.105712890625 626.65673828125 42.8879623413086 30.3515110015869 576.619995117188 383.288696289062 27.2254447937012 358.389282226562 158.781585693359 231.712829589844 636.790344238281 66.9954299926758 125.965400695801 73.2473907470703 168.357467651367 426.300231933594 127.888473510742 502.951812744141 667.983459472656 542.807861328125 496.236999511719 162.99089050293 446.207946777344 516.124206542969 456.707702636719 80.6683731079102 1152.71228027344 146.537780761719 176.341873168945 341.855072021484 87.3148803710938 215.434448242188 275.086090087891 173.621612548828 882.158264160156 1274.64306640625 92.7878341674805 35.9898147583008 945.7998046875 222.924407958984 25.0227451324463 74.4967575073242 402.053588867188 42.641357421875 221.242156982422 422.877777099609 243.028686523438 598.607482910156 49.3781547546387 207.189849853516 216.115631103516 96.8924179077148 109.640525817871 229.685958862305 59.8915710449219 116.176513671875 334.154113769531 1008.3369140625 156.15168762207 143.922561645508 114.359115600586 321.070709228516 327.842468261719 168.118850708008 1314.13732910156 119.468811035156 492.900054931641 97.4948043823242 202.419357299805 68.1754302978516 451.947418212891 447.981475830078 260.613098144531 373.886596679688 84.911994934082 166.977172851562 1289.896484375 64.6090698242188 127.319030761719 466.265380859375 308.463348388672 196.598526000977 48.3094940185547 160.341323852539 359.947723388672 44.4950866699219 636.957214355469 702.854370117188 70.5335006713867 41.0298271179199 206.898483276367 94.0941619873047 229.116333007812 88.1125717163086 549.303771972656 41.9018974304199 204.971984863281 117.318054199219 69.139404296875 84.9973678588867 196.261871337891 346.159362792969 43.4350814819336 74.4794769287109 75.0406112670898 255.726196289062 628.258178710938 571.731811523438 589.112182617188 35.9928894042969 66.18017578125 538.843322753906 136.660766601562 58.6882133483887 94.6505279541016 46.5957984924316 221.893661499023 129.527725219727 64.255126953125 272.349334716797 525.698120117188 288.614654541016 75.353630065918 134.873672485352 826.4853515625 139.714447021484 198.415893554688 332.794342041016 108.012870788574 490.217102050781 335.035736083984 280.973785400391 63.2330017089844 356.442443847656 562.506530761719 129.303787231445 78.3767013549805 106.580459594727 211.090896606445 252.843231201172 368.985626220703 537.011047363281 260.128814697266 116.711921691895 259.033752441406 753.642944335938 596.897644042969 47.9310722351074 145.158843994141 409.490875244141

ENSG00000197919.4 0 0 0.222876220941544 0 0 0 0 0 0 0 0 0 0 0 0 0 0 0 0 0 0 0.0553750731050968 0 0.0281140059232712 0 0 0 0 0 0 0 0 0 0 0.068680576980114 0 0 0 0 0 0.0308945216238499 0 0 0 0.256304234266281 0 0 0 0 0 0 0.201184570789337 0 1.07020390033722 0 0 0.0976009145379066 0 0 0 0 0 0.0383429229259491 0 0 0 0 0 0 0 0 0 0 0 0.188212424516678 0 0 0 0 0 0 0.0761043801903725 0 0 0 0 0 0 0 0 0 0.0951070412993431 0 0 0.25861456990242 0 0 0 0 0 0 0 0 0 0 0 0 0 0 0 0 0 0 0 0 0 0 0 0 0 0 0 0.81452214717865 0 0 0 0 0 0 0 0 0.0829892829060555 0 0 0.042041402310133 0 0.0485468879342079 0 0 0 0 0 0 0 0 0 0 0 0 0 0 0 0.218299567699432 0 0.0817683860659599 0 0 0 0 0 0 0.197392523288727 0.123325988650322 0 0 0 0 0 0 0.272993743419647 0 0 0 0.0722717046737671 0 0 0 0 0 0 0 0.125047072768211 0 0 0 0 0 0 0 0 0 0 0 0.0799947306513786 0 0 0 0 0 0 0 0 0.106185153126717 0 0 0 0.184804320335388 0 0 0 0 0.062845878303051 0 0 0 0 0 0.0615984313189983 0 0 0 0 0 0 0 0.962217748165131 0 0.251742750406265 0 0 0 0 0 0 0 0.0648129433393478 0 0 0.0687335208058357 0 0 0 0.0428071804344654 0 0.055893674492836 0 0 0 0 0 0.0681112930178642 0 0 0 0.0813979431986809 0

ENSG00000188379.6 0 0 0 0 0 0 0 0 0 0 0 0 0 0 0 0 0 0 0 0 0 0 0 0 0 0 0 0 0 0 0 0 0 0 0 0 0 0 0 0 0 0 0 0 0 0 0 0 0 0 0 0 0 0 0 0 0 0 0 0 0 0 0 0 0 0 0 0 0 0 0 0 0 0 0 0 0 0 0 0 0 0 0 0 0 0 0 0 0 0 0 0.0483076758682728 0 0 0 0 0 0 0 0 0 0 0 0 0 0 0 0 0 0 0 0.0615599416196346 0 0 0 0 0 0 0 0 0 0 0 0 0 0 0 0 0 0 0 0 0 0 0 0 0 0 0 0 0 0 0 0 0 0 0 0 0 0 0 0 0 0 0 0 0 0 0 0 0 0 0 0 0 0 0 0 0 0 0 0 0 0 0 0 0 0 0 0 0 0 0 0 0 0 0 0 0 0 0 0 0 0 0 0 0 0 0 0 0 0 0 0 0 0 0 0 0 0 0 0 0 0 0 0 0 0 0 0 0 0 0 0 0 0 0 0 0 0 0 0 0 0 0 0 0 0 0 0 0 0 0 0 0 0 0 0 0 0 0 0 0 0 0 0

ENSG00000236637.2 0 0 0 0 0 0 0 0 0 0 0 0 0 0 0 0 0 0 0 0 0 0 0 0 0 0 0 0 0 0 0 0 0 0 0 0 0 0 0 0 0 0 0 0 0 0 0 0 0 0 0 0 0 0 0 0 0 0 0 0 0 0 0 0 0 0 0 0 0 0 0 0 0 0 0 0 0 0 0 0 0 0 0 0 0 0 0 0 0 0 0 0 0 0 0 0 0 0 0 0 0 0 0 0 0 0 0 0 0 0 0 0 0 0 0 0 0 0 0 0 0 0 0 0 0 0 0 0 0 0 0 0 0 0 0 0 0 0 0 0 0 0 0 0 0 0 0 0 0 0 0 0 0 0 0 0 0 0 0 0 0 0 0 0 0 0 0 0 0 0 0 0 0 0 0 0 0 0 0 0 0 0 0 0 0.0674295499920845 0 0 0 0 0 0 0 0 0.0471543557941914 0 0 0 0 0 0 0 0 0 0 0 0 0 0 0 0 0 0 0 0 0 0 0 0 0 0 0 0 0 0.0480074286460876 0 0 0 0 0 0 0 0 0 0 0 0 0 0 0 0 0 0 0 0 0 0 0 0 0 0 0 0 0 0 0.0959630236029625 0

ENSG00000147873.5 0 0 0 0 0 0 0 0 0 0 0 0.0876329094171524 0 0 0.13668192923069 0 0 0.0408971533179283 0 0 0 0.0646884068846703 0 0 0.328422755002975 0 0 0 0 0 0.0869265049695969 0.082062803208828 0 0 0 0 0 0.0305715091526508 0 0 0 0 0.064535565674305 0.0397855825722218 0.0499018542468548 0 0 0 0 0 0 0.0783403590321541 0 0 0 0 0 0 0 0 0 0.439852684736252 0 0.0576230995357037 0 0 0 0.0578168109059334 0 0.0419074818491936 0 0 0 0 0.293156266212463 0 0 0 0 0 0 0 0 0 0 0 0 0 0.0459954552352428 0 0 0 0 0 0 0 0.0283357743173838 0 0.0969576388597488 0.110525980591774 0 0.165018752217293 0 0.133772507309914 0 0.200963169336319 0.157787412405014 0.0744613483548164 0 0.119209453463554 0 0 0 0 0 0.0740521103143692 0 0 0.0303423460572958 0 0 0.678732514381409 0 0 0 0 0 0 0 0.0754242092370987 0.0542554445564747 0 0 0 0 0 0.34027087688446 0.0431031100451946 0 0.127989903092384 0 0 0 0.0801354050636292 0 0.0463990792632103 0 0 0 0 0.0974683612585068 0 0 0.058893196284771 0.28656217455864 0 0 0 0 0 0 0 0 0.135252252221107 0 0.0797641724348068 0 0 0 0 0 0 0 0 0 0 0 0 0 0.040611956268549 0 0 0 0 0 0 0 0 0 0.0690109580755234 0 0 0 0.140173137187958 0.100699655711651 0 0 0 0 0 0.0692357420921326 0 0 0 0.0685155764222145 0 0 0.0455577857792377 0 0 0 0.0734156966209412 0 0 0 0 0.0472422353923321 0.0719584450125694 0 0.0675339326262474 0 0 0 0 0 0.0661205723881721 0.109984837472439 0 0 0 0 0.0402610562741756 0 0 0 0.0757135972380638 0 0 0 0.276437997817993 0.0833046659827232 0 0.1000135242939 0 0 0 0 0 0 0 0 0 0 0 0 0

ENSG00000120235.4 0 0 0 0 0 0 0 0 0 0 0 0 0 0 0 0 0 0 0 0 0 0 0 0 0 0 0 0 0 0 0 0 0 0 0 0 0 0 0 0 0 0 0 0 0.0637993961572647 0 0 0 0 0 0 0 0 0 0 0 0 0 0 0 0 0 0 0 0 0 0 0 0 0 0 0 0 0 0 0 0 0 0 0 0 0 0 0 0 0 0 0 0 0 0 0.0355111509561539 0 0 0 0 0 0 0 0 0 0 0 0 0 0 0 0 0 0 0 0 0 0 0 0 0 0 0 0 0 0 0 0 0 0 0 0 0 0 0 0 0 0 0 0 0 0 0 0 0 0 0 0 0 0 0 0 0 0 0 0 0 0 0 0 0 0 0 0 0 0 0 0 0 0 0 0 0 0 0 0 0 0 0 0 0 0 0 0 0 0 0 0 0 0 0 0.0608337484300137 0 0 0 0 0 0 0 0 0 0 0 0 0 0 0 0 0 0 0 0 0 0 0 0 0 0 0 0 0 0 0 0 0 0 0 0 0 0 0 0 0 0 0 0 0 0 0 0 0 0 0 0 0 0 0 0 0 0 0 0 0 0 0 0 0 0 0 0

ENSG00000214042.1 0 0 0 0 0 0.121347524225712 0 0 0 0 0 0 0 0 0 0 0 0 0 0 0 0 0 0 0 0 0 0 0 0 0 0 0 0 0 0 0 0 0 0 0 0 0 0 0 0 0 0 0 0 0 0 0 0 0 0 0 0 0 0 0 0 0 0 0 0 0 0 0 0 0 0 0 0 0 0 0 0 0 0 0 0 0 0 0 0 0 0 0 0 0 0 0 0 0 0 0 0 0 0 0 0 0 0 0 0 0 0 0 0 0 0 0 0 0 0 0 0 0 0 0 0 0 0 0 0 0 0 0 0 0 0 0 0 0 0 0 0 0 0 0 0.0669235438108444 0 0 0 0 0 0 0 0 0 0 0 0 0 0 0 0 0 0 0 0 0 0 0 0 0 0 0 0 0 0 0 0 0 0 0 0 0 0 0 0 0 0 0 0 0 0 0 0 0 0 0 0.187721684575081 0 0 0 0 0 0 0 0 0 0 0 0 0 0 0 0 0 0 0 0 0 0 0 0 0 0 0 0 0 0 0 0 0 0 0 0 0 0 0 0 0 0 0 0 0 0 0 0 0 0 0 0 0 0 0 0 0 0 0 0 0 0

ENSG00000120242.3 0.105631873011589 0 0 0 0 0 0 0 0 0 0 0 0 0 0 0 0 0 0 0 0 0 0 0 0 0 0 0 0 0.0519407205283642 0 0 0 0 0 0 0 0 0 0 0 0 0 0 0 0 0 0 0 0 0 0 0 0 0 0 0.0541548915207386 0 0 0 0 0 0 0 0 0 0 0 0 0 0 0 0 0 0.208863273262978 0 0 0 0 0 0 0 0 0 0 0 0 0 0 0 0 0 0 0 0 0 0 0 0 0 0 0 0 0 0 0 0 0 0 0 0 0 0 0.0318516865372658 0 0 0 0 0 0 0 0 0 0 0 0 0 0 0 0 0 0 0 0 0 0 0 0 0 0 0 0 0 0.0253749266266823 0 0 0 0 0 0 0 0 0 0 0 0 0 0 0 0 0 0 0 0 0 0 0 0 0 0 0 0 0 0 0 0 0 0 0 0 0 0 0 0 0 0 0 0 0 0 0 0 0 0 0 0 0 0 0 0 0 0 0 0 0 0 0 0 0 0 0 0.0697413831949234 0 0 0 0 0 0 0 0 0 0 0 0 0 0 0 0 0 0 0 0 0 0 0 0 0 0 0 0 0 0 0 0 0 0 0 0 0 0 0 0 0 0 0 0

ENSG00000186803.3 0 0 0 0 0 0 0 0 0 0 0 0 0 0 0 0 0 0 0 0 0 0 0 0 0 0 0 0 0 0 0 0 0 0 0 0 0 0 0 0 0 0 0 0 0 0 0 0 0 0 0 0 0 0 0 0 0 0 0 0 0 0.128804206848145 0 0 0 0 0 0 0 0 0 0 0 0 0 0 0 0 0 0 0 0 0 0 0 0 0 0 0 0 0 0 0 0 0 0 0 0 0 0 0 0 0 0 0 0 0 0 0 0 0.0652837157249451 0 0 0 0 0 0 0 0 0 0 0 0 0 0 0 0 0 0 0 0 0 0 0 0 0 0 0 0 0 0 0 0 0 0 0 0 0 0 0 0 0 0 0 0 0 0 0.0744271129369736 0 0 0 0 0 0 0 0 0 0 0 0 0 0 0 0 0 0 0 0 0 0 0.105649687349796 0 0 0 0 0 0 0 0 0 0 0 0 0 0 0 0 0 0 0 0 0 0 0 0 0 0 0 0 0 0 0 0 0 0 0 0 0 0 0 0 0 0 0 0 0 0 0 0 0 0 0 0 0 0 0 0.0833504647016525 0 0 0 0 0 0 0 0 0 0 0 0 0 0 0 0 0 0 0

ENSG00000233816.3 0 0 0 0 0 0 0 0 0 0 0 0 0 0 0 0 0 0 0 0 0 0 0.0683353915810585 0 0 0 0 0 0 0 0 0 0 0 0 0 0 0 0 0 0 0 0 0 0 0 0 0 0 0 0 0 0 0.0616756677627563 0 0 0 0 0 0 0 0 0 0 0 0 0 0 0 0 0 0 0 0 0 0 0 0 0 0 0 0 0 0 0 0 0 0 0 0 0 0 0 0 0 0 0 0 0 0 0 0 0 0 0 0 0 0 0 0 0 0 0 0 0 0 0 0 0 0 0 0 0 0 0 0 0 0 0 0 0 0 0 0 0 0 0 0 0 0 0 0 0 0 0 0 0 0 0 0 0 0 0 0 0 0 0 0 0 0 0 0 0 0 0 0 0 0.0864876806735992 0 0 0 0 0 0 0 0 0 0 0 0 0 0 0 0 0 0 0 0 0 0 0 0 0 0 0 0 0 0 0 0 0 0 0 0 0 0 0 0 0 0 0 0 0 0 0 0 0.0302976500242949 0 0 0 0 0 0 0 0 0 0 0 0 0 0 0 0 0 0 0 0 0 0 0 0 0 0 0 0 0 0 0 0 0 0 0 0 0 0 0

ENSG00000228083.2 0 0 0 0 0 0 0 0 0 0 0 0 0.128148198127747 0 0 0 0 0 0 0 0 0.0644923821091652 0 0 0 0 0 0 0 0 0 0 0 0 0 0 0 0 0 0 0 0 0 0.0396650247275829 0 0 0 0 0 0 0 0 0 0 0 0 0 0 0 0 0 0 0 0 0 0 0 0 0 0 0 0 0 0 0 0 0 0 0 0 0 0 0 0 0 0 0 0 0 0 0 0.0553830415010452 0 0 0 0 0 0 0 0 0 0 0 0 0 0 0 0 0 0 0 0 0 0 0 0 0 0 0.0302503984421492 0 0 0 0 0 0 0 0.0890394747257233 0 0 0 0 0 0 0.0570223033428192 0 0 0 0 0 0 0 0 0 0 0 0 0 0 0 0.0789832398295403 0.0485865026712418 0 0 0 0 0 0 0 0 0 0 0 0 0 0 0 0 0 0 0 0 0 0 0 0 0 0 0 0 0 0 0 0 0 0 0 0 0 0 0 0 0 0 0 0 0 0 0 0 0 0 0 0.0618340894579887 0 0 0 0 0 0 0 0.167507231235504 0 0 0 0.0639273747801781 0 0 0 0 0 0 0 0 0 0 0 0 0 0 0.0860120505094528 0 0 0 0 0 0 0 0 0 0 0 0 0.0498552285134792 0 0 0 0 0 0 0 0 0 0 0 0 0

ENSG00000147885.4 0 0 0 0 0 0 0 0 0 0 0 0 0 0 0 0 0 0 0 0 0 0 0 0 0 0 0 0 0 0 0 0 0 0 0 0 0 0 0 0 0 0 0 0 0 0 0 0 0 0 0 0 0 0 0 0 0 0 0 0 0 0 0 0 0 0 0 0 0 0 0 0 0 0 0 0 0 0 0 0 0 0 0 0 0 0 0 0 0 0 0 0 0 0 0 0 0 0 0 0 0 0 0 0 0 0 0 0 0 0 0 0 0 0 0 0 0 0 0 0 0 0 0 0 0 0 0 0 0 0 0 0 0 0 0 0 0 0 0 0 0 0 0 0 0 0 0 0 0.0383110567927361 0 0.102450773119926 0 0 0 0 0 0 0 0 0 0 0 0 0 0 0 0 0 0 0 0 0 0 0 0 0 0 0 0 0 0 0 0 0 0 0 0 0 0 0 0 0 0 0 0 0 0 0 0 0 0 0 0 0 0 0 0 0 0 0 0 0 0 0 0 0 0 0 0 0 0 0 0 0.0500013493001461 0 0 0 0 0 0 0 0 0 0 0 0 0 0 0 0 0 0 0 0 0 0 0 0 0 0 0 0 0 0 0 0

ENSG00000234829.3 0 0 0 0 0 0 0 0 0 0 0 0 0 0 0 0 0 0 0 0 0 0 0 0 0 0 0 0 0 0 0 0 0 0 0 0 0 0 0 0 0 0 0 0 0 0 0 0 0 0 0 0 0 0 0 0 0 0 0 0 0 0.047451589256525 0 0 0 0 0 0 0 0 0 0 0 0 0 0 0 0 0 0 0 0 0 0 0 0 0 0 0 0 0 0 0 0 0 0 0 0 0 0 0 0.0623081550002098 0 0 0 0 0 0 0 0 0 0 0 0 0 0 0 0 0 0 0 0 0 0 0 0 0 0 0 0 0 0 0 0 0 0 0 0 0 0 0 0 0 0 0 0 0 0 0 0 0 0 0 0 0 0 0 0 0 0 0 0 0 0 0 0 0 0 0 0 0 0 0 0 0 0 0 0 0 0 0 0 0 0 0 0 0 0 0 0 0 0 0 0 0 0 0 0 0 0 0 0 0 0 0 0 0 0 0 0 0 0 0 0 0 0 0 0 0 0 0 0 0 0.0718458592891693 0 0 0 0 0 0 0 0 0 0 0 0 0 0 0 0 0 0 0 0 0 0 0 0 0 0 0 0 0 0 0 0

ENSG00000137080.4 0 0 0 0 0 0.0873370468616486 0.0976082161068916 0 0.135294824838638 0 0 0 0 0 0 0 0 0 0 0 0.0504346005618572 0 0.102703280746937 0 0 0 0 0 0 0 0 0 0.066307820379734 0 0 0 0 0 0.0726854652166367 0 0 0 0 0 0 0 0 0 0 0.0907872244715691 0 0 0 0 0 0 0 0 0 0 0 0 0 0 0.109175011515617 0.0378262102603912 0 0 0 0 0.104543782770634 0.0806117951869965 0 0 0 0 0 0 0 0 0 0 0 0.225698843598366 0 0 0 0.109327897429466 0.0443335101008415 0.0355114676058292 0 0.160632446408272 0 0 0 0 0.0546238459646702 0 0 0 0 0.0795280784368515 0 0 0.040573425590992 0 0.0506953708827496 0 0 0 0 0 0 0.0646365284919739 0.207805991172791 0 0 0 0 0 0 0 0 0.0800956338644028 0 0 0.0860830843448639 0.0977605208754539 0 0.145397841930389 0 0 0 0.220515921711922 0.0946752727031708 0.12370153516531 0.0546626597642899 0 0 0 0 0 0 0 0 0 0.168851763010025 0.0339607298374176 0.0702618807554245 0 0.140919834375381 0 0 0.0567652173340321 0.184138566255569 0 0 0 0 0 0 0 0 0 0 0 0 0.0649924501776695 0 0 0 0 0 0 0 0 0 0 0 0 0 0 0 0 0.0644004866480827 0 0 0 0 0 0.108450010418892 0 0.0447202175855637 0 0 0 0 0 0 0.0530249029397964 0 0 0.0597810000181198 0 0 0 0 0 0 0 0 0.0707629844546318 0 0.0890696793794632 0 0 0 0 0.105920232832432 0 0 0 0 0 0 0.0637314543128014 0 0 0 0 0 0 0 0.0700208991765976 0 0.145955711603165 0.0783852487802505 0.0678872838616371 0 0 0 0 0.0481998771429062 0 0.062934972345829 0 0 0 0 0 0 0 0 0.119386985898018 0 0

ENSG00000111537.4 0.360431879758835 0.100455045700073 0.126589313149452 0.949662566184998 0.371053844690323 0.587409734725952 0.246184289455414 0 2.16116237640381 0 0.271990597248077 0.49709016084671 0.937438786029816 0.651772975921631 0.664556980133057 1.16672396659851 1.15339422225952 0.629675209522247 0.108492478728294 0.190194174647331 0.0424015074968338 0.52419912815094 3.54014348983765 1.11777400970459 0.0887118950486183 0.577319741249084 3.90834879875183 0.168882623314857 0.446125477552414 2.87998080253601 5.91699743270874 0.930988430976868 0.390225321054459 0.682547509670258 7.02165842056274 0.950651228427887 0.137188270688057 13.7988195419312 0.244433224201202 1.49019515514374 1.11134040355682 0.306032210588455 0.575256764888763 0.0322400443255901 1.05138051509857 14.4609327316284 0.524348020553589 4.2725715637207 2.31852126121521 3.12940096855164 1.9444020986557 10.2841987609863 7.53791999816895 0.389650464057922 0.860757052898407 1.92374551296234 0.323373138904572 2.33043074607849 1.82631480693817 3.05512976646423 1.78082883358002 1.52756726741791 11.0341997146606 1.54092121124268 0.917858839035034 2.63951182365417 0.719619274139404 0.0937030985951424 1.4037172794342 0.815028250217438 0.35156923532486 0.271088600158691 1.27246069908142 1.76545703411102 0.178168252110481 5.1410961151123 11.9646844863892 0.0634895041584969 0.187118798494339 0.39621901512146 2.29145884513855 1.72903168201447 11.2242155075073 0.284625113010406 0.230893924832344 7.41772413253784 0.824475824832916 0.275743275880814 0.298177450895309 0.29855290055275 0.607856869697571 0.675236582756042 0.603734612464905 0.405760794878006 7.83402442932129 0.399176836013794 0.734775960445404 13.3766946792603 7.14979028701782 0.6269491314888 2.51816391944885 0.869193613529205 0.509793162345886 0.271004647016525 0.136443972587585 1.79134404659271 5.02924728393555 0.301696836948395 8.67186546325684 0.193201512098312 0.464543461799622 6.82643175125122 5.26898193359375 0.21736553311348 0.116471461951733 0 0.935537934303284 0.483621656894684 15.9082832336426 0.246912404894829 0.172280922532082 14.8501996994019 1.87256014347076 1.48144042491913 3.45059132575989 1.15328311920166 0.0723719894886017 6.82172536849976 1.23906743526459 0.122239232063293 0.747415542602539 0.576110064983368 0.434314370155334 0.648874461650848 0.596967220306396 2.25330424308777 2.06802558898926 0.558854103088379 1.35704624652863 0.674153745174408 1.14152669906616 0.445442646741867 4.06621837615967 0.0649373084306717 0.32143172621727 0.5639888048172 0.662468194961548 0 13.5862712860107 0.577783763408661 5.01541900634766 0.955519914627075 19.4939594268799 5.05872297286987 4.87649726867676 4.26202964782715 2.01287984848022 0.35307052731514 0.788244128227234 1.22743690013885 4.67831897735596 0.840863049030304 2.91861414909363 0.767206788063049 1.06423461437225 17.9043064117432 0.587935209274292 1.6938591003418 1.77904009819031 14.9517374038696 0.178952574729919 2.28980731964111 1.00395631790161 0.0684148371219635 8.42972278594971 0.415427714586258 0.23246993124485 1.73532319068909 5.1496753692627 1.02020037174225 0.668247282505035 5.74112844467163 0.189366310834885 0.683775901794434 0.758001029491425 9.831618309021 0.863486707210541 5.01254081726074 2.01134085655212 1.50991213321686 1.36764538288116 0.878552138805389 0.789543151855469 0.530080020427704 0.0816014483571053 1.17038595676422 6.76669883728027 1.06791996955872 6.6242847442627 6.86520195007324 2.07587838172913 2.11083769798279 0.753888547420502 0.211080089211464 4.66378402709961 1.62092185020447 0.0874710083007812 1.25519549846649 0.335552155971527 7.51906871795654 4.96951866149902 0.594920337200165 2.84236073493958 6.21527910232544 3.481365442276 0.216290935873985 1.33988749980927 5.83111572265625 0.0445247627794743 0.273628890514374 0.505630135536194 0.0787502527236938 0.182745799422264 3.70059251785278 0.305164366960526 22.6109561920166 2.49551796913147 0 0.526269197463989 0 0.693422794342041 1.0766361951828 0.458003252744675 0.235472574830055 0.326311439275742 1.28843665122986 0.527201950550079 4.22350358963013 0.0650654733181 3.24814653396606 0.540044069290161 2.4043493270874 0.810454487800598 0.0641019120812416 4.28577852249146 1.73179042339325 0.656342327594757 0.372370034456253 3.54902005195618 1.45441043376923 3.15934634208679 0.989595174789429 2.11743640899658 0.100371330976486 0.385270237922668 1.77318060398102

ENSG00000125498.18 0.0655624270439148 0.0730909407138824 0.0307020656764507 0 0 0 0.0597077757120132 0.0218415204435587 0.165521964430809 0 2.22637462615967 0.103337727487087 0 0 0.0805884525179863 0.0446792989969254 0 0.0482263937592506 0.197347447276115 0 0 0.0762813091278076 0 0.116184405982494 0.129093304276466 0 0.174104630947113 0.880631148815155 0.162300139665604 0.0322379991412163 1.0762996673584 0.629001080989838 0.0811221152544022 0.0551800690591335 0.236525386571884 0 0 1.60423707962036 0.0444623194634914 0.250214725732803 0.0425584018230438 0 0 0.0469156168401241 0.20595695078373 0.132349103689194 0 0.18441541492939 0.255598872900009 0.0555353090167046 75.9246063232422 0.0923798531293869 0.349018722772598 0.170105472207069 0.0481758192181587 0.0451520830392838 0.0336122624576092 0.0968924760818481 0 0 0.125393077731133 0.0370485223829746 0.290503740310669 0.0339749120175838 0.267133116722107 0.0462772287428379 0.161105826497078 0.272712975740433 0.0928493440151215 0.22238002717495 0 0.147932752966881 0.246890842914581 0 0 0 0 0.0461948700249195 0 0 0.0943733379244804 0.524183750152588 0.111111879348755 0.0690309479832649 3.02396559715271 0.234657615423203 0.26395046710968 1.47128975391388 0.135595932602882 0.0217226650565863 0 0.22927388548851 0 0.196820661425591 0 0.0290440488606691 0.0835346728563309 0.0461273156106472 0.571667790412903 0.749417662620544 0.143141597509384 0.0486480034887791 0.627718865871429 0.0788730382919312 0 0.0394963435828686 0.372129499912262 0 0.242678344249725 0.0351432897150517 0 0.250432044267654 0 0.0593080706894398 0 0 1.10613095760345 0.087970606982708 0.053670059889555 0 0.0501405373215675 0.84483414888382 0.0801454186439514 0 0.0512376613914967 0 0.157973259687424 0.239203751087189 0.100171521306038 0 0 0 0.0632013082504272 0 0.1737409979105 0.227007821202278 0.30093851685524 0 0.0858595594763756 0.11319537460804 0.237306609749794 0 0.336201637983322 0.157494321465492 0.0584683157503605 0 0.0344293490052223 0 3.69626188278198 0 0 0.173808738589287 0.902148365974426 0.0694475322961807 0 0.08860132843256 0.285769462585449 0.17126239836216 0.191175103187561 0.141012847423553 0.040523074567318 0.169947415590286 0.339772701263428 0.0398727543652058 0 0.987617671489716 0.0712968111038208 0.636102974414825 0.211815342307091 9.64323997497559 0 0 0.304365515708923 0 0.655778169631958 0.0335849560797215 0 0.650439918041229 0.429971724748611 0.0239450428634882 0 0.043064296245575 0.0688913315534592 0.149254307150841 0.15757729113102 0.333828657865524 0.0739143118262291 0 0.158210664987564 0 0.265359133481979 0.191770002245903 0.136778682470322 0.192842736840248 0 0.0709642544388771 0.205143332481384 0 0.0547706820070744 0 0 0 0.0365685448050499 0 0.161588698625565 0 0.0636437833309174 0 0.0348781794309616 0 0.396253645420074 0.259717911481857 1.53781616687775 0.81727010011673 0.302452117204666 0 0.640648722648621 0 0 0.0398183986544609 0 0 0 0.25242617726326 0 1.71534311771393 0.0648476928472519 0 580.197875976562 0.0508673377335072 0.0630666837096214 0.0712144523859024 0 0 0.276994377374649 0.0446411743760109 0 0.913599014282227 0.0473415479063988 0 0 0.0583133697509766 0.058968547731638 0.233202308416367 1.30892705917358 0.0687298998236656 0 0.232230767607689 0.176564902067184 0.220463931560516 0.0469129756093025 0.0514305680990219 0.584382057189941 0.0730300396680832 0.224257677793503 0

ENSG00000243772.5 0.085709884762764 0.047775961458683 0.160547465085983 0.034742709249258 0.0352943055331707 0.0349211767315865 0 0 0.189338773488998 0 4.43049383163452 0.135093614459038 0 0.0309980288147926 0 0 0 0 0.0386989004909992 0 0 0 0.0821305513381958 0.177202761173248 0.0421909801661968 0.0784487500786781 0.151738196611404 0.0535465404391289 0.0530438162386417 0.105361983180046 0.100503467023373 0.316267043352127 0.0530255436897278 0.0360684990882874 0.123684041202068 0.0452125407755375 0.0326230563223362 0.777621686458588 0.0871885046362877 0.0545176975429058 0 0.083170011639595 0.0248717721551657 0.0153332185000181 0.076927974820137 0.237902820110321 0.020781459286809 0.464952737092972 0.40097388625145 0.0363007076084614 3.62193298339844 0.362305045127869 0.0977727472782135 0.0741264000535011 0.0314901657402515 0 0.0219706874340773 0 0.0510933585464954 0 0.163926601409912 0.048433605581522 0.569664120674133 0.0888309255242348 0.545661926269531 0.0604983009397984 0.157960459589958 0.111411929130554 0.121382109820843 0.016150988638401 0.0418011844158173 0 0.181552976369858 0 0 0.0611270554363728 0.0615172944962978 0.0301953181624413 0 0 0.16449923813343 0.651003062725067 0.145256772637367 0 0.0366040356457233 0.0511280559003353 0.156846791505814 1.26770842075348 0.0354529619216919 0.0283980816602707 0.0361367464065552 0.128455772995949 0 0 0.0582160465419292 0.0759386718273163 0.0546025149524212 0.0603023245930672 0.336304038763046 0.212981551885605 0.0935646370053291 0 0.410309046506882 0 0.0162230338901281 0.025816822424531 0.202702313661575 0 0.846010267734528 0.0689143240451813 0.0736449286341667 0.0545650385320187 0 0.0516890361905098 0 0 0.333703190088272 0 0.058469146490097 0.0880728214979172 0 0.348774701356888 0.192086145281792 0 0.167457714676857 0 0.0688395798206329 0.117266811430454 0.261908829212189 0.0290682129561901 0.0209098495543003 0.0124543327838182 0 0 0.0567829795181751 0.329741954803467 0.0874261036515236 0.0332235582172871 0.0280610881745815 0.0739902555942535 0.0387789197266102 0.0385182723402977 0.0439517013728619 0.0411785244941711 0.0764357373118401 0 0.0225047767162323 0 0.61806309223175 0 0.0187819730490446 0.0757401660084724 0.606070399284363 0.113486111164093 0 0.115828692913055 0.0233491826802492 0.0839593708515167 0.291577488183975 0 0.132439717650414 0.0444345138967037 0.166569530963898 0.0260628629475832 0.0281191784888506 0.153704106807709 0 0.545723438262939 0.0923021957278252 6.23308038711548 0 0.0816766396164894 0.0397897586226463 0.0650755763053894 0.100858815014362 0.0219528377056122 0 0.500189006328583 0.0803004056215286 0.0156516991555691 0.119180761277676 0 0.0450308844447136 0.130080282688141 0.128750681877136 0.21820741891861 0.120785407721996 0.0366760268807411 0.0258536357432604 0.0265965703874826 0.0867261290550232 0.250701248645782 0.0894055142998695 0.0540222078561783 0 0.0927717015147209 0.214547410607338 0.0461725406348705 0.0537013597786427 0.0424033626914024 0.16009920835495 0.0501952432096004 0.215127572417259 0.0752915516495705 0.607329964637756 0.0265828669071198 0.0832016319036484 0 0.0227981526404619 0.0310959573835135 0.550399541854858 0.169764846563339 1.45579957962036 0.213683754205704 0.0494245253503323 0 0.436967581510544 0.0831975713372231 0 0 0.0343536287546158 0 0.0434565357863903 0.256664484739304 0 1.52895748615265 0.0423877537250519 0 0.444962233304977 0.0664989724755287 0.123670779168606 0.0155164636671543 0.0335114225745201 0 0.232788369059563 0.0583594888448715 0.0313418544828892 0.0542886219918728 0 0.0532690919935703 0.0963159427046776 0.152466371655464 0.173451811075211 0.121946334838867 0.754924774169922 0.164726331830025 0.104051142930984 0.0252996422350407 0.144264757633209 0.0864638835191727 0.0919941291213036 0.0672352761030197 0.590335607528687 0.0954722985625267 0.07329311221838 0.0766651555895805

ENSG00000189013.13 2.9920654296875 0 0.476986199617386 0.9805948138237 0.0524296499788761 1.45251023769379 0.028988054022193 0 1.12505006790161 0 7.92661714553833 0.25085175037384 0.515120089054108 0.598617970943451 0.391255974769592 0.911053419113159 0.477681845426559 0.39803558588028 0.459897369146347 1.28996658325195 0.0599130354821682 0.481448382139206 0 0.620481312274933 0.313373237848282 0.349606603384018 1.6060243844986 2.76413035392761 0.551575660705566 0.313030213117599 1.74180746078491 0.563777208328247 0.236308157444 0.107159428298473 1.37799370288849 0.335815876722336 0 8.9962158203125 0.215864032506943 0.485915809869766 0.309930831193924 0.185323432087898 1.51482653617859 0.0455549545586109 0.714227557182312 4.27297973632812 0.123483337461948 2.71158480644226 2.03512477874756 1.34811651706696 3.66323566436768 3.00497078895569 5.37392807006836 2.25735092163086 0.421007513999939 0.394583106040955 2.83945631980896 0.235205918550491 0.68309211730957 0.674512088298798 1.09580767154694 0.575584530830383 4.71840381622314 0.230926916003227 0.745733737945557 0.83129894733429 1.83809220790863 0.165502294898033 0.450782477855682 0.191938206553459 0.0620956122875214 0.143642351031303 0.239730417728424 0.658291518688202 0.377625733613968 1.72527921199799 0.913838684558868 0 0 0.111970953643322 1.00799918174744 1.22155487537384 0.539446890354156 0 0.16312575340271 3.56968140602112 1.32807564735413 0.324686110019684 2.89659309387207 0.274204552173615 0.322086542844772 1.39935350418091 0.548403918743134 0.382224798202515 2.76735591888428 0.282017052173615 0.616451025009155 0.71663224697113 2.33137011528015 0.664406061172485 0.750546872615814 0.141711309552193 1.96706664562225 0.0765855386853218 0.0481985956430435 0 6.50406408309937 0 13.8242206573486 10.2030906677246 0.364664852619171 3.12066769599915 0.962406933307648 0.211155995726585 0 0.0847905203700066 6.27906560897827 0.17083851993084 3.00521206855774 0.675965666770935 0.0730295181274414 2.50417113304138 2.49027252197266 1.18935513496399 1.19403958320618 2.27462363243103 0.0511305592954159 3.13559484481812 0.389065265655518 0.0863615944981575 0.248492449522018 1.05455112457275 0.429578304290771 0.32744824886322 0.281170189380646 2.13076615333557 1.9805383682251 0.14806067943573 0.333477705717087 0.183187425136566 0.115212082862854 0.0572188496589661 3.52567100524902 0.0917559713125229 1.41931486129761 0.0796912238001823 1.13664793968201 0 5.30012607574463 0.408202230930328 1.33922874927521 0.168767884373665 18.8579883575439 3.67512011528015 0.218744650483131 0.387142598628998 3.81537008285522 0.207869246602058 0.185630574822426 0.319487333297729 3.81673741340637 0.528059363365173 1.64959251880646 0.696894288063049 0.334168016910553 9.0417594909668 2.21532917022705 0.347430646419525 1.16547584533691 30.8032550811768 0.189644053578377 1.37507975101471 0.945722222328186 0 0.936410367488861 0.228276401758194 0 1.41175734996796 6.26252269744873 0.441761046648026 1.65240025520325 1.29627525806427 0.0334466621279716 0.241542622447014 0.765035927295685 1.20397388935089 0.287082523107529 0.926197350025177 2.88041591644287 0.316073387861252 2.51221537590027 3.35174751281738 0.743745982646942 0.106999911367893 0.0576511360704899 1.44702875614166 1.19516229629517 0.411535531282425 0.505230903625488 0.661396563053131 1.66479027271271 2.38607931137085 0.319571733474731 0.466022938489914 1.92205262184143 0.789776384830475 0.123595923185349 0.260820984840393 0.101599894464016 1.87081682682037 1.77952134609222 0.294216394424438 4.7885890007019 2.75103378295898 1.68866169452667 0 2.92101693153381 2.05983138084412 0.0943697690963745 0.541289985179901 0 0.0741823688149452 0.0645546391606331 1.47063136100769 0.215597584843636 6.92735767364502 1.88900887966156 0 20.5320281982422 0.0493920668959618 0.214331597089767 0.437944084405899 0.199124723672867 0.374311238527298 0.537921786308289 1.64716601371765 0.838049232959747 1.69355952739716 0.597590863704681 0.395655900239944 0.572308897972107 1.13244307041168 0.801616609096527 0.0905755609273911 1.00929582118988 3.1811101436615 0.412180960178375 0.187912955880165 5.37905883789062 18.0675048828125 1.77654325962067 0.674176037311554 2.83716821670532 0.21273598074913 0.925453126430511 0.493505954742432

ENSG00000221957.7 0.273013710975647 0.304363757371902 0.0958866775035858 0 0 0.0556176155805588 0 0 0 0 9.11650562286377 0.430316835641861 0 0 0.167792364954948 0 0 0.0753087624907494 0 0 0 0 0.130806177854538 0.443494915962219 0 0 0.302084535360336 0.0426407605409622 0.591365933418274 0.167806014418602 0 0.201482549309731 0.211129382252693 0.287224590778351 0.393973678350449 0.0720082819461823 0 0.168884754180908 0.046287264674902 0.173656493425369 0 0 0 0 0.306300580501556 0.241117358207703 0.0330978333950043 0.164558306336403 1.01114165782928 0.115629494190216 13.1325750350952 0.528943598270416 0.31143781542778 0.413203984498978 0.100306369364262 0 0.314926832914352 0 0 0.048211395740509 0.0435132719576359 0 0.10997361689806 0 1.11239016056061 0 0.503155112266541 0.177441507577896 0.24165078997612 0.257230639457703 0 0.102669738233089 1.09235382080078 0 0 0.0649031475186348 0.0489881187677383 0 0 0.3601453602314 0.130995810031891 0 0 0.0718643069267273 0.466382741928101 0 0.2747842669487 0 0.564645707607269 0.113071322441101 0 0.238684386014938 0 0 0.695388495922089 0.060472309589386 0.243497341871262 0.240103006362915 1.54734265804291 0.237445294857025 0.0298033617436886 0 0.0297037940472364 0.246331080794334 0.103351220488548 0.164469853043556 0.258268982172012 0 2.9264018535614 0 0.195485770702362 0.347614586353302 0.0778742879629135 0.0823231413960457 0 0 0.930083453655243 0 0.670475244522095 0.187026932835579 0.0782978236675262 0.462900072336197 0.611856460571289 0 0 0 0.219276338815689 0.435788184404373 0.312849134206772 0 0.0333023183047771 0.0198355410248041 0.0657953917980194 0 0.0301453601568937 0.315100401639938 0.765821099281311 0 0.178767278790474 0.157121941447258 0 0 0.280000776052475 0.0327917262911797 0.426076918840408 0 0 0 0.201347470283508 0.243138700723648 0.837573230266571 0.241256892681122 0.965265154838562 0.253042995929718 0 0.507308721542358 0.0371873490512371 0 0.0663406103849411 0.0489335581660271 0.253118008375168 0.672306895256042 0.442148238420486 0.041509322822094 0 0.0979194492101669 0 0.662211656570435 0 35.122673034668 0 0 0 0 0.28110945224762 0.104890339076519 0 2.03141140937805 0.0639456957578659 0.124639309942722 0 0 0.107578448951244 0.103586949408054 0.0820225104689598 0 0.0384740568697453 0 0.576465308666229 0 0.138125389814377 0.26618817448616 0.0284785479307175 0.0286797024309635 0.24724018573761 0.221630915999413 0.512552201747894 0.0735372304916382 0 0.371438145637512 0.0424973107874393 0.399720221757889 0.114208482205868 0.119913965463638 0.168221071362495 0.12701253592968 0.0662560239434242 0.335563719272614 0.0363097451627254 0 1.65007102489471 0.811133980751038 3.42268943786621 2.15539765357971 0.118074826896191 0.109221547842026 1.3628853559494 0.0441685281693935 0.0674516931176186 0.1243581995368 0 0.0795338302850723 0 0.145992740988731 0.0770502164959908 1.2987265586853 0.27003738284111 0.0902547985315323 0.398629277944565 0.0529551766812801 0.36110383272171 0.0494249537587166 0.0266861822456121 0.0445904247462749 0.453142672777176 0 0 0.475548774003983 0.19713868200779 0 0 0.394594430923462 0 0 0.60116982460022 0.262353330850601 0 0.0402937717735767 0.0689294934272766 0.321317970752716 0.195354044437408 0.294478386640549 0.774286389350891 0.0760275349020958 0 1.58732163906097

ENSG00000167633.15 0 0.0861043259501457 0 0 0 0 0.017584590241313 0.0128651326522231 0.170618087053299 0 7.22717809677124 0.121736355125904 0 0.0279331263154745 0.0949367135763168 0.0263170804828405 0.0340904705226421 0.028406398370862 0.0116241946816444 0.0652094334363937 0 0.0449313558638096 0 0.136870294809341 0.0380193889141083 0 0.153827130794525 0.0965043529868126 0 0.0759555399417877 0.120755054056644 0.341995716094971 0.167239427566528 0.0650045275688171 0.0557274483144283 0 0.0293974876403809 0.74320387840271 0.0523785501718521 0 0.0752034857869148 0 0.0224125999957323 0 0.0173304472118616 0.0584673844277859 0.0187267120927572 0.0931068882346153 0.692545235157013 0 7.06003189086914 0.571344375610352 0.176211103796959 0.0333986133337021 0.0283766072243452 0.0265955608338118 0 0.0570717789232731 0.030694367364049 0.0545558929443359 0.0738592594861984 0 0.357781827449799 0.0400239266455173 0.786736190319061 0.0272582974284887 0.284684538841248 0.0401584729552269 0.191416010260582 0.0727703794836998 0.0565022006630898 0.029045207425952 0.290848165750504 0 0 0.0367221161723137 0 0 0.0267312563955784 0.135846525430679 0.0185293182730675 0.61751127243042 0.0981709808111191 0 0.131939396262169 0.092145636677742 0.183740332722664 0 0.175711706280708 0.0511804930865765 0 0.385849505662918 0.0369633436203003 0 0.0262299962341785 0.017107579857111 0.059044498950243 0.108679994940758 0.505087196826935 0.307077139616013 0.0843135342001915 0 0.0336127355694771 0.0929158851504326 0.058475986123085 0.0930568352341652 0.273990482091904 0 0.50029993057251 0.0414003282785416 0.0442422367632389 0.442529767751694 0.0550764612853527 0.0465783290565014 0.0499163381755352 0.0514352135360241 0.751771509647369 0 0.158064186573029 0.0132274506613612 0.103368550539017 0.261908292770386 0.0944148004055023 0.0577184520661831 0.0603601932525635 0.0205943416804075 0.155082836747169 0 0.11800642311573 0 0 0.0112229231745005 0 0.0993175208568573 0.13644964993 0.415994614362717 0.196954816579819 0.0299386139959097 0.0379298627376556 0.155573949217796 0.0698893889784813 0 0.158424064517021 0.00927675887942314 0.0344391129910946 0 0.0202796403318644 0 1.36706578731537 0.0275135152041912 0.135399386286736 0.238879978656769 0.63470995426178 0.163624450564384 0 0.130470290780067 0.10520277172327 0.0504386499524117 0.112606301903725 0.166119292378426 0.143213838338852 0.220226019620895 0 0.0234859213232994 0 0.498624294996262 0.0419953763484955 0.702522218227386 0.138626500964165 12.4993362426758 0 0.0490672960877419 0.0358555838465691 0.205244526267052 0.249937877058983 0.0989113599061966 0 0.540879964828491 0.397984266281128 0.0423124544322491 0 0.0507316254079342 0.0405784994363785 0.0879140421748161 0.0232041161507368 0.477535724639893 0.108842857182026 0.0660994499921799 0.0232973825186491 0.0239668600261211 0 0.112956702709198 0.145018145442009 0.129815503954887 0.0699440911412239 0.0417994931340218 0.0725003480911255 0 0 0.0764215365052223 0.0240449253469706 0.180928945541382 0 0.0226157251745462 0.166563719511032 0.0239545106887817 0 0.0474653802812099 0.102720029652119 0.0280213728547096 1.28371119499207 0.178476095199585 1.62420606613159 0.609760582447052 0.0445377230644226 0 0.0164067856967449 0.124952480196953 0 0 0 0.0112500358372927 0.0391598120331764 0.115643516182899 0.0435949116945267 0.275556653738022 0.152786821126938 0 1.10265922546387 0 0.185738250613213 0 0 0 0.0466159209609032 0.0525892488658428 0.0847288817167282 0.0489208921790123 0.0418278053402901 0.144006505608559 0 0.171739250421524 0.0347337648272514 0.0274722501635551 0.498873710632324 0 0.0937631875276566 0.0227981638163328 0.130000725388527 0.207772925496101 0.110531069338322 0.0605874583125114 0.359859853982925 0.0430162847042084 0.0660463273525238 0.253311514854431

ENSG00000240403.4 0.233887076377869 0.0651860684156418 0.109526395797729 0 0.0481559485197067 0 0 0 0.221430838108063 0.0634902790188789 3.17694306373596 0.36864647269249 0.0337950736284256 0 0.07187270373106 0.358624577522278 0.0516170039772987 0.0215053223073483 0 0 0 0 0 0.241777434945107 0.1726975440979 0.053518183529377 0.0776374936103821 0.292238026857376 0 0.0287514179944992 0.36567485332489 0.258910983800888 0.108522973954678 0.0984245389699936 0.253133833408356 0.123377002775669 0 7.21798706054688 0 0.446307331323624 0.0569334886968136 0.0567390583455563 0.101805977523327 0.0418416298925877 0.13120174407959 0.0295088458806276 0.0567089170217514 0.140974834561348 1.13977766036987 0.0990581884980202 40.8312377929688 0.288360983133316 0.133402228355408 0.0505694486200809 0.0859310775995255 0 0 0.0432067103683949 0.0464749000966549 0 0.149108871817589 0.0330416783690453 0.329745054244995 0 0.0893408730626106 0 0.0718410164117813 0.121609352529049 0.207018911838531 0.154256150126457 0.0285170041024685 0.0439778789877892 0.165141955018044 0.0318227484822273 0 0.111203260719776 0.0839348882436752 0 0 0.154265776276588 0.75750058889389 0.467492550611496 0.0990949869155884 0 0.249714821577072 0.418558180332184 0.085601419210434 0 0.120931044220924 0.135613307356834 0.0493053793907166 0.408955216407776 0.0279834344983101 0.131650686264038 0.397153168916702 0.0777086839079857 0.163900643587112 0.329108685255051 1.47853934764862 0.0581188835203648 0.102128520607948 0.0867733210325241 0.788851797580719 0.105514220893383 0 0.070449523627758 0.138284668326378 0 0.793585240840912 0.0313424952328205 0.133975967764854 0.372245758771896 0.150106117129326 0.0881563723087311 0.0377896204590797 0 0.189711838960648 0 0.446745365858078 0.0200279224663973 0.0223588868975639 0.356904059648514 0.214432761073112 0.0873925685882568 0.182784959673882 0.218275874853134 0 0.0533333830535412 0.178675681352615 0.158644005656242 0.0570592656731606 0.0849641710519791 0 0.0601513870060444 0.0516502298414707 0.607369840145111 0.357855468988419 0.203987702727318 0.17229089140892 0.0673021003603935 0.21164159476757 0.0262773837894201 0 0.0280922204256058 0.260724455118179 0.048796895891428 0.0307057723402977 0 1.99323630332947 0.0833174288272858 0.307516068220139 0 0.40228995680809 0 0 0.434604436159134 0.0318578891456127 0.0381850376725197 0.0568330809473991 0.0838414132595062 0.18070225417614 0.121253915131092 0.530295193195343 0.177802309393883 0.0383661203086376 0.293601840734482 0 0.177283629775047 0.167917549610138 8.79196071624756 0 0.0371468439698219 0.0542895942926407 0.044394925236702 0.137612909078598 0.209668889641762 0 1.16018795967102 0.164344176650047 0.170842826366425 0 0.0384068340063095 0 0.177483007311821 0.140535101294518 0.127596274018288 0.164800956845284 0 0.211649969220161 0 0.0591650567948818 0.114019878208637 0.0975887179374695 0 0.0529518127441406 0 1.53683733940125 0 0.390777289867401 0.0578556209802628 0.400475442409515 0.958817422389984 0.0978408306837082 0 0.144112676382065 0.108809888362885 0.0567606240510941 0 0 0.0636415109038353 0.618447005748749 0.231629058718681 0.851275444030762 0.923249661922455 0.236023843288422 0 0.273259937763214 0.113515697419643 0 0.106535956263542 0 0.0170338843017817 0.0592925883829594 0.375209927558899 0.132015720009804 0.869219541549683 0.115668669342995 0 61.507984161377 0 0.0562459379434586 0 0.091446727514267 0 0.176455035805702 0.0398131757974625 0.128289550542831 0.370360046625137 0.0422214977443218 0.218042865395546 0.087609700858593 0.130016773939133 0.0262955129146576 0.0831924676895142 0.137337043881416 0 0.283937096595764 0 0.275571078062057 0.825806021690369 0.0418392792344093 0.0917365774512291 1.16081082820892 0 0.050000973045826 0.627616882324219

ENSG00000134545.12 1.38308048248291 1.04304957389832 1.35250675678253 0.428720921278 0.167510598897934 1.5911009311676 0.111138790845871 0.0135517800226808 1.23239636421204 0 0.153486162424088 0.48087665438652 0.282135009765625 0.11769600212574 0.100003756582737 0.47126892209053 0.57455962896347 0.448837965726852 0.244892254471779 0.0686898455023766 0.191419690847397 0.331306308507919 0.116940140724182 0.552672564983368 0.720874547958374 0.595722079277039 1.36831510066986 0.698878645896912 0.100700676441193 0.54006415605545 1.46280109882355 0.630435764789581 0.553662896156311 0.0684740021824837 5.60601997375488 0.171666890382767 0.80512934923172 6.57611513137817 0.24828365445137 1.65597999095917 0.211246177554131 0.473680675029755 2.8802764415741 0.130991607904434 0.346853047609329 6.48726463317871 0.23671455681324 0.686533868312836 1.17355704307556 1.24046695232391 0.146298602223396 3.43907785415649 1.36118400096893 0.914710998535156 0.418476074934006 1.06457149982452 1.96037495136261 0.420825034379959 0.452656537294388 0.890749275684357 1.65976202487946 0.459742218255997 6.71825456619263 0.126480355858803 0.828726530075073 0.358914375305176 1.42442512512207 0.27496200799942 0.662506520748138 0.567242205142975 0.198392942547798 0.397740602493286 1.03400409221649 0.332086533308029 0.321733057498932 2.12751436233521 3.64959669113159 0 0.816581428050995 0.321968346834183 0.878322541713715 0.813087046146393 0.585993647575378 0.128492742776871 0.0694906860589981 0.727977871894836 1.10172951221466 0.51867949962616 0.185089945793152 0.377385020256042 0.240112468600273 1.44287407398224 0.778723657131195 0.457947254180908 1.71305775642395 0.126144602894783 0.238417491316795 0.77274364233017 2.94398307800293 0.0808666795492172 1.86508524417877 0 0.300957322120667 0.244687676429749 0.0153992539271712 0.539129495620728 3.0015869140625 0.0272399373352528 5.78447866439819 0.109024949371815 0.372828602790833 1.68331527709961 1.0558922290802 0.147193044424057 0.0262902602553368 0 0.369551330804825 0.163746610283852 0.499501585960388 0.125400930643082 0.13999579846859 1.40702414512634 0.44754296541214 0.668789565563202 0.82656329870224 0.802660346031189 0.294048100709915 1.85520327091217 0.155380949378014 0.275921761989594 0.436657786369324 0.555630385875702 0.274497032165527 0.376626193523407 1.14985883235931 0.860745012760162 0.580907225608826 0.946095645427704 0.279680013656616 0.397987902164459 0.809815347194672 0.127968326210976 1.79395592212677 0.117262624204159 0.943207919597626 0.74685662984848 0.704946756362915 0.0128894690424204 3.30673575401306 0.202873930335045 1.17666482925415 0.323523908853531 4.66455507278442 2.82235503196716 0.314496129751205 0.412301629781723 2.81477093696594 0.478176295757294 0.395388096570969 0 1.15657484531403 0.611583948135376 1.15948379039764 0.519528090953827 0.453752607107162 8.92903423309326 1.72523462772369 0.715350568294525 0.64251172542572 5.83324289321899 0.16157466173172 3.1270124912262 0.604308843612671 0.339741289615631 1.07704544067383 0.125028640031815 0.13993002474308 1.89916098117828 0.800340235233307 0.356566339731216 0.301677256822586 0.694711089134216 0.0427442900836468 0.27781879901886 1.00214600563049 1.30194234848022 0.55033016204834 4.38652372360229 1.76693987846375 0.504920840263367 0.823223114013672 0.991546094417572 0.86562967300415 0.427325427532196 0.699933469295502 1.45300507545471 1.14554846286774 0.175311878323555 4.72363615036011 0.744628489017487 3.39398813247681 1.04822111129761 0.294960975646973 0.202493712306023 0.551425933837891 1.94294333457947 0.197441980242729 0.666649878025055 0.432810008525848 1.04785192012787 0.645380854606628 0.510290801525116 1.25026917457581 2.23116564750671 1.12595582008362 0.0976435765624046 0.639451086521149 0.658107757568359 0.160804033279419 0.889405429363251 0.0652184188365936 0.260710597038269 0.164999544620514 8.00503444671631 0.0688825473189354 6.45837259292603 0.603530585765839 0.37654110789299 0.290377855300903 0.126244515180588 0.293477445840836 0.500771105289459 0.349907457828522 0.186030313372612 0.22096773982048 1.2464120388031 0.446255534887314 1.59748995304108 0.264361649751663 0.556205987930298 0.304750561714172 0.615078568458557 0.658576905727386 0.578770458698273 0.955454528331757 1.05188846588135 2.46918988227844 0.216134712100029 1.47894382476807 1.69618332386017 0.494829297065735 0.654167115688324 1.08775627613068 0.135936558246613 0.38264274597168 0.921781539916992

ENSG00000205809.8 0.07321647554636 0.65299129486084 0.342863649129868 0.178070873022079 0.120598688721657 0.83526611328125 0.166695818305016 0.097565583884716 0.646960139274597 0 0.165752783417702 1.15401840209961 0.380854070186615 0 0.17999342083931 0.399162858724594 1.00181400775909 0 0.0440773367881775 0.309081375598907 0.585700809955597 0.0425933673977852 0.070158876478672 0.259496569633484 0.0720821171998978 1.13923394680023 0.324050635099411 0.0686120837926865 0.181247770786285 0.360016077756882 0.114471599459648 0.594366788864136 0.951222956180573 0.0616220347583294 1.63765847682953 0.077244371175766 0.167206779122353 4.22718954086304 0.0993061065673828 0.512280881404877 0.213870882987976 0.319710731506348 0.892347395420074 0.0785891339182854 0.131429299712181 3.10380291938782 0 2.91264891624451 0.513789594173431 0.372112512588501 0.0877726599574089 2.73386430740356 2.72835302352905 0.253285795450211 0.0538000799715519 0.60508006811142 0.938407719135284 0.0541020669043064 0.174583166837692 1.60322988033295 0.326741397380829 0.289616137742996 1.97600340843201 0.0758825838565826 0.298319429159164 0.180879458785057 0.404806613922119 0 0.414755940437317 0.27593520283699 0 0 0.0689284950494766 0.358626842498779 0 0.0348112918436527 4.57187509536743 0 0.405444890260696 0.321944504976273 0.105390906333923 0.585379302501678 0.806543231010437 0.462539553642273 0 0.349403470754623 0.37515577673912 0.149368613958359 0.121140792965889 0.266845345497131 2.22259068489075 7.42516946792603 0.315359711647034 0.109899193048477 1.79028880596161 0.0973043441772461 0.223888546228409 0.154537290334702 0 0.90968269109726 0.191823109984398 0.0543273910880089 0.095591127872467 0.220202565193176 0.0277166049927473 0.308751255273819 0.831147074699402 0 9.77893352508545 0.0784921497106552 0.0419401079416275 0.978840053081512 0.0835369229316711 0.220773205161095 0 0 4.94106292724609 0.0982406884431839 0.259721577167511 0.426331579685211 0 1.19174385070801 1.13369154930115 0.711296200752258 0.457754999399185 0.702817976474762 0.0588052570819855 1.40242969989777 0.279664993286133 0 0.500134527683258 0.361724019050598 0.0705797150731087 0.301278620958328 0.355711221694946 0.619690477848053 0.112023800611496 0.227046132087708 0.0719123929738998 0.969145715236664 0.530021727085114 0.164518505334854 7.65920734405518 0.193469017744064 0.391765028238297 0.183305770158768 0.961219727993011 0 4.12777996063232 0 0.12835393846035 0 6.54855060577393 0.853106737136841 0.0628945603966713 1.33575820922852 0.359022885560989 0.143442258238792 0 0.419934183359146 0.588300943374634 0.113872706890106 1.89719653129578 0.22263839840889 0.288244992494583 0.0525198392570019 1.27392494678497 0.355182230472565 0.551935076713562 14.9986562728882 0 1.39542281627655 1.15565490722656 0 2.84318923950195 0.0375058129429817 0.0629638358950615 1.1963826417923 1.92067420482635 0.133702471852303 0.135744690895081 0.0480918176472187 0 0.222238555550575 0.263960361480713 0.106514669954777 0 1.25319957733154 2.4735324382782 0.0908790081739426 0.963099777698517 0.642474234104156 0.152746841311455 0 0.132609158754349 0.633991539478302 0.824733734130859 0.0788845047354698 0.703395843505859 2.02845907211304 0.820575177669525 2.40120124816895 0 0.214389204978943 0.766926467418671 0.363328725099564 0.142147690057755 0.239976182579994 0.0389500148594379 1.08909499645233 0.719085395336151 0.386718064546585 1.42125332355499 0.425918251276016 0 0.117163605988026 0.497698038816452 0.852844476699829 0.21706934273243 0.177867904305458 0.117384441196918 0.127975717186928 0 1.34683430194855 0.0826529264450073 7.92361783981323 1.15869283676147 0.193635374307632 0.0950257033109665 0 0.105644047260284 0.344622820615768 0.687040090560913 0.143498465418816 0.530283331871033 0.14955835044384 0 0.463753044605255 0.185039445757866 0.910087764263153 0.109702073037624 0.423287391662598 0.395116835832596 0.208341926336288 0.816853106021881 0.844291150569916 0.237024679780006 0.172894954681396 1.4048924446106 1.62493252754211 0.523898065090179 0.0287174060940742 0.830588757991791 0.652447104454041 0.375657767057419 0.654901444911957

ENSG00000205810.7 0.0956023558974266 0.1065803617239 0.0895387828350067 0.620042383670807 0 0.155806839466095 0.0435325838625431 0.0318490490317345 0 0 0.0721438527107239 0.22602878510952 0.0552555359899998 0 0.117513127624989 0 0.421973496675491 0.0351615771651268 0.0863309130072594 0.121074832975864 0.179947853088379 0 0.458049476146698 0.112945817410946 0.0941211581230164 0.35001266002655 0.380815953016281 0.029863391071558 0.118332058191299 0.188036262989044 1.12103390693665 0.211661979556084 0.0591456517577171 0.0804629251360893 0.896736204624176 0 0.0727767050266266 0.893657386302948 0 0.121620044112206 0 0.0927693471312523 0.665818154811859 0 0.0429034270346165 0.964949548244476 0 0.537824749946594 0.37271124124527 0.242942780256271 0 1.14501118659973 0.363524973392487 0 0.210748225450516 0.263360917568207 0.196052014827728 0.211931169033051 0.07598727196455 0.877884149551392 0 0 0.654668688774109 0 0.0973825827240944 0.0337404534220695 0.293653249740601 0.149125054478645 0.203087702393532 0.252211481332779 0 0.14380918443203 0.405014932155609 0.15609222650528 0.126021444797516 0.40909332036972 0.960644960403442 0 0.264704644680023 0 0.0458713583648205 0.382179260253906 0.324044048786163 0 0 0.684349894523621 0.0699799060821533 0 0 0 0.322460651397705 2.5790741443634 0 0 0.259740948677063 0.0423516891896725 0.0730855688452721 0.13452459871769 0.750239253044128 0.142538085579872 0.459200263023376 0 0.0832120180130005 0 0 0.0575931072235107 0.135658696293831 0 0.737230539321899 0 0.0547632612287998 0.426039844751358 0.245426088571548 0.20179209113121 0 0 0.868510007858276 0.128277689218521 0.0782610476016998 0 0.0365571714937687 0.38902959227562 0.272689908742905 0.142888367176056 0.373570740222931 0.0509835556149483 0.0767849087715149 0.610407114028931 0.146068930625916 0 0.0466464459896088 0.0555671602487564 0 0 0.0422245115041733 0.183900073170662 0.097516655921936 0.0370581597089767 0.156499236822128 0 0.346037685871124 0.042963981628418 1.17658853530884 0.0229656342417002 0.255773425102234 0.039891891181469 0.15061342716217 0 1.37879765033722 0.136225447058678 0.0837990120053291 0 1.38858544826508 0.607605457305908 0.164249047636986 0.258394837379456 1.40638160705566 0 0.0929230824112892 0.411246508359909 0.118180528283119 0.0495630651712418 0.495452553033829 0.0581419765949249 0.125458553433418 0.617199599742889 0.519820809364319 0.173917084932327 0.102955542504787 3.56437563896179 0 0.485885888338089 0.0887644365429878 0 0.224999502301216 0.0489731840789318 0 0.167375966906548 0.895685315132141 0.0698327571153641 0.0886242538690567 0.125591695308685 0.0502282679080963 0.0725469514727592 0.0574443340301514 0.208622142672539 0.215561956167221 0.409091055393219 0.3460513651371 0.237330362200737 0.0967358946800232 0 0.15955924987793 0.040171567350626 0 0.103479243814945 0.119655035436153 0.206006720662117 0.039932981133461 0.662164688110352 1.13099110126495 0.111977361142635 0.31994292140007 0.0279938522726297 0.117813371121883 0.0593020208179951 0.185609221458435 0.039168581366539 0.0508589446544647 0.554959893226624 0.0722265467047691 0.0631195977330208 0.309300005435944 0.238346725702286 0.165387034416199 0.0764931440353394 0.0406167954206467 0.061866719275713 0.330677807331085 0 0.153274640440941 0.0278506968170404 0.0969444140791893 0.736169755458832 0 1.59173178672791 0 0 0.0620398633182049 0 0.183926180005074 0.0692293792963028 0.074758492410183 0.062457662075758 0 0.390571355819702 0 0.302772581577301 0 0.237669259309769 0.0716216936707497 0.0425159335136414 0 0 0.168411359190941 0.100221134722233 0 0.112878724932671 0.257464855909348 0.321477919816971 0 0.149990901350975 0.309868723154068 1.81035590171814 0 0.342054814100266

ENSG00000134539.15 0.416203111410141 0.674902737140656 0.537459075450897 0.480564892292023 0.103871203958988 1.8653312921524 0.109116606414318 0.0756295919418335 1.05872619152069 0.0479314364492893 0.261730760335922 0.6212198138237 0.513910830020905 0.14140222966671 0.348812222480774 0.713379800319672 0.192055642604828 0.171629875898361 0.334080040454865 0.287507504224777 0.0474787726998329 0.150410577654839 0.40486478805542 0.59973418712616 0.478047221899033 0.109665535390377 1.54344296455383 0.413667351007462 1.13958871364594 0.679076254367828 1.28172147274017 1.31704568862915 0.963635087013245 1.01903676986694 2.43881583213806 0.319345414638519 0.638464629650116 3.85931396484375 0.461872667074203 0.497381567955017 0.513730525970459 0.409988909959793 1.8006626367569 0.155683621764183 0.571657836437225 2.67011141777039 0.159015670418739 1.52293026447296 2.00611352920532 0.8172727227211 0.412010550498962 2.97221183776855 1.65453553199768 0.68718546628952 0.463378071784973 0.577611446380615 0.29743492603302 0.72226756811142 0.624026477336884 0.677064836025238 1.14980638027191 0.24588181078434 2.52240419387817 0.209143623709679 0.568482935428619 0.712186455726624 1.34039795398712 0.452481925487518 1.16545557975769 0.727245569229126 0.215286657214165 0.232405036687851 1.12502086162567 0.566287219524384 0.124689102172852 2.26970076560974 2.13633680343628 0.155513525009155 0.445240378379822 0.116461619734764 1.74888908863068 0.962993323802948 1.23438000679016 0.451501458883286 0.204678907990456 1.8583037853241 0.496220409870148 0.228354841470718 0.3677918612957 0.231922283768654 0.388179332017899 0.54816609621048 0.510038495063782 0.468546003103256 1.957444190979 0.164822146296501 0.817939102649689 0.949463605880737 3.24965739250183 0.620536983013153 0.638837277889252 0.290110260248184 0.266207724809647 0.117588996887207 0.040582749992609 0.55464631319046 1.48541748523712 0.0971240550279617 3.54019808769226 0.121688984334469 0.317881137132645 1.50949895381927 1.33467316627502 0.241491943597794 0.0652090236544609 0.0755923837423325 2.21379232406616 0.203074410557747 3.61357069015503 0.157679051160812 0.27730855345726 4.19986867904663 0.493361294269562 0.923667788505554 1.75446879863739 0.628875255584717 0.612849831581116 1.94415581226349 0.871965289115906 0.124044306576252 0.627684772014618 0.302388280630112 0.468083322048187 0.379504323005676 1.18371284008026 0.921909511089325 0.668967366218567 0.601328432559967 0.54711788892746 0.896418392658234 0.593457400798798 0.323074132204056 0.795502007007599 0.195416390895844 0.674851536750793 0.31049844622612 0.433816969394684 0.011988895945251 2.74911117553711 0.512183845043182 0.842949509620667 0.323209941387177 5.17504215240479 1.04538810253143 0.628381848335266 1.51693391799927 1.05136513710022 0.354166001081467 0.44744461774826 0.759544253349304 0.865289986133575 0.640777170658112 1.53600513935089 0.464053452014923 0.99719512462616 3.61881160736084 1.93386244773865 4.43962240219116 0.563664436340332 7.08438777923584 0.206642717123032 0.649011135101318 1.07147705554962 0.378247112035751 1.50640046596527 0.520088255405426 0.119307138025761 1.91367423534393 2.09737730026245 0.412263631820679 0.268907725811005 1.44974553585052 0.0993944630026817 0.507245123386383 0.719935595989227 1.4311546087265 0.746490478515625 0.771754026412964 0.87500387430191 0.598794221878052 2.34816312789917 1.09442436695099 0.736737608909607 0.198734328150749 0.536814153194427 0.948769748210907 2.00473761558533 0.706606388092041 1.16688549518585 1.16993582248688 2.20665645599365 0.443174064159393 0.587395429611206 0.0849402025341988 0.97916966676712 0.927066028118134 0.324442863464355 0.310035854578018 0.26502525806427 2.17120409011841 0.967131853103638 0.553743541240692 1.31592917442322 1.06908178329468 0.592735052108765 0.312828958034515 0.308103322982788 1.10182762145996 0.320949733257294 0.436611354351044 0.272977530956268 0.163500428199768 0.498781353235245 1.69417345523834 0.156615048646927 7.65703153610229 1.42835569381714 0.358571439981461 0.204613223671913 0.357164472341537 0.715794503688812 0.168960422277451 0.22930121421814 0.234829917550087 0.293069064617157 0.807234644889832 0.37818044424057 1.50584650039673 0.173034384846687 0.501667261123657 0.609434604644775 0.91985297203064 0.578531503677368 0.408235371112823 2.41059303283691 0.678705811500549 0.47464519739151 0.308996081352234 1.08690285682678 1.00937116146088 0.509891510009766 0.50705099105835 1.35667264461517 0.330145746469498 0.447581142187119 1.74483847618103

ENSG00000226979.7 1.45324385166168 1.19747960567474 0.562181890010834 0.768359959125519 0.884633362293243 8.64983558654785 1.2371574640274 0.420986831188202 6.18134546279907 0.4802525639534 1.04897236824036 4.92969179153442 0.474745869636536 0.0457029081881046 3.65028119087219 0.473646849393845 0.418329536914825 1.20840847492218 0.285284638404846 0.800195157527924 0.356787800788879 1.87462317943573 0.605458676815033 2.46335005760193 2.23940110206604 2.19760394096375 1.64993453025818 0.197370037436485 2.34620404243469 5.12634325027466 3.70451021194458 2.09834170341492 0.781798601150513 1.32946789264679 2.59859299659729 4.59957551956177 0.865778744220734 5.54146862030029 0.856993913650513 5.14431095123291 3.28118658065796 1.34886729717255 0.770079910755157 0.655603229999542 0.623816311359406 5.22950410842896 1.77710807323456 3.57992458343506 2.06915903091431 4.73661518096924 1.06044733524323 9.65967655181885 3.60385727882385 0.710388720035553 2.13571119308472 1.17488992214203 0.518290638923645 0.747025907039642 0.703090131282806 1.33892774581909 3.74619960784912 2.60645008087158 5.24302530288696 1.34244906902313 0.772333025932312 4.08078813552856 2.56183314323425 2.46395468711853 2.41600728034973 6.88187456130981 2.98909759521484 0.712836623191833 2.8552348613739 2.5102961063385 4.7058162689209 1.65228378772736 5.21524620056152 0.845868766307831 0.306155204772949 0.277832329273224 9.42853164672852 0.959826648235321 2.78412985801697 0.598745167255402 0.971429407596588 5.80443239212036 0.763131439685822 0.676737844944 0.470440596342087 1.65384888648987 0.319676011800766 1.16792225837708 0.786210477352142 1.37519502639771 4.20579957962036 0.72775661945343 1.32028067111969 6.09024000167847 4.0769100189209 0.408220738172531 6.53882694244385 2.95366477966309 0.907427847385406 0.494080305099487 0.119594663381577 2.01738333702087 5.61857652664185 0.973141670227051 1.34479010105133 0.372555255889893 1.91825890541077 3.25821161270142 0.576726973056793 1.562291264534 0.245012491941452 0.883637368679047 0.697008013725281 0.339119851589203 1.89652752876282 0.432842969894409 0.314092814922333 4.02811050415039 1.4160407781601 1.41654455661774 13.2830305099487 1.95434010028839 1.11645352840424 2.4781756401062 2.31692051887512 0.557149112224579 1.75725793838501 1.08338356018066 0.852726399898529 0.519996106624603 1.70230174064636 4.88596153259277 3.06136059761047 1.76343083381653 2.64785528182983 1.16362500190735 1.48654758930206 1.33457946777344 1.68484032154083 1.79102873802185 1.01425874233246 0.079094797372818 1.26086354255676 0.500515103340149 5.21903800964355 1.12540876865387 6.3414192199707 3.51760077476501 4.25053644180298 2.10825443267822 1.24836838245392 3.11665534973145 1.61800301074982 1.48545718193054 2.64079165458679 0.498294085264206 2.46035933494568 1.2775114774704 5.07546424865723 1.07594454288483 4.10437965393066 1.72229993343353 0.687109112739563 2.29886937141418 2.08669209480286 3.65008687973022 0.815640509128571 0.963379979133606 1.75995802879333 0.959461987018585 5.09312391281128 2.78354907035828 1.35841679573059 4.42481565475464 3.78858876228333 1.49997699260712 0.820016741752625 3.61070680618286 1.29465734958649 0.431522846221924 2.050137758255 5.1015739440918 0.961652100086212 4.5422534942627 0.571771681308746 1.41168534755707 3.58028602600098 1.04728281497955 1.84544932842255 2.97357487678528 0.171658992767334 1.16263723373413 5.93108749389648 1.22536647319794 5.6742992401123 5.68920278549194 0.590118110179901 0.148013845086098 1.40968894958496 0.148011237382889 5.37261486053467 1.41095793247223 1.47205090522766 2.56280255317688 1.34452676773071 2.59037208557129 2.4344961643219 0.625745236873627 2.45303225517273 7.71875667572021 4.08075284957886 0.151665151119232 0.536880612373352 2.8621814250946 0.499538570642471 2.14895558357239 0.962356507778168 0.349729090929031 1.72993052005768 1.94616639614105 2.28249621391296 2.70511937141418 2.74981093406677 0.417759656906128 0.615041136741638 0.637291371822357 1.51948213577271 1.14385902881622 3.4833071231842 2.7656843662262 0.762708365917206 7.70094871520996 0.73935741186142 7.56398010253906 0.912489891052246 4.86941480636597 1.51473498344421 1.54545545578003 0.369393676519394 1.30351638793945 4.30378103256226 0.816924273967743 2.60798764228821 2.2753803730011 2.31844353675842 1.01984560489655 1.67282438278198 4.75826025009155 3.30232286453247 1.12610042095184 0.75643390417099 2.44906258583069

ENSG00000179583.16 9.4846019744873 11.1443338394165 14.0145778656006 0.767035186290741 14.7475652694702 12.2218532562256 20.2875003814697 1.70257365703583 25.3109149932861 8.99342155456543 22.3029499053955 28.0102119445801 1.88256144523621 4.26884603500366 14.8231601715088 2.08228516578674 2.43034315109253 12.2191762924194 3.48054099082947 3.79764747619629 1.4962170124054 8.57645797729492 2.15508532524109 7.32050371170044 9.19258880615234 14.9062051773071 12.5591173171997 4.16911697387695 5.28905534744263 22.7313289642334 28.14817237854 17.616283416748 15.4059038162231 5.12376832962036 2.36133646965027 29.102783203125 8.96165370941162 24.0399055480957 20.4482421875 21.5516891479492 10.156322479248 5.45590209960938 17.3914432525635 5.02508640289307 3.7970016002655 6.81001234054565 28.1638259887695 18.5699253082275 17.8199596405029 14.8790445327759 2.08252930641174 29.7770328521729 13.6061697006226 3.42062020301819 25.877254486084 1.12693238258362 0.978071272373199 2.20053815841675 6.23491907119751 8.27170181274414 37.5506706237793 16.1095733642578 10.0214614868164 7.66384315490723 8.44862747192383 13.2416105270386 12.072452545166 33.1052284240723 17.1223182678223 28.2512493133545 15.7795515060425 12.359806060791 7.02356672286987 6.97258996963501 21.7489395141602 6.93573904037476 4.53645467758179 4.3495512008667 4.14958953857422 1.59591841697693 23.3607616424561 7.09948253631592 7.34699773788452 7.82252502441406 2.26541018486023 6.67092895507812 6.05422115325928 1.72452569007874 7.86885976791382 8.04514312744141 1.02669274806976 13.7615432739258 3.57415819168091 12.5719327926636 34.0491142272949 10.1898069381714 12.8434286117554 11.0162076950073 28.6915740966797 3.93513369560242 11.8014736175537 36.9722442626953 5.48445558547974 6.67537832260132 7.91486835479736 21.3926792144775 21.1800632476807 8.92181873321533 13.3960094451904 2.88911485671997 6.70351505279541 24.0439167022705 1.73427653312683 8.42313766479492 3.44831895828247 14.0218925476074 13.5521202087402 6.08738946914673 6.12628269195557 2.56336069107056 1.76743268966675 31.0370979309082 8.58587837219238 8.09631443023682 37.9645843505859 18.7680282592773 3.92410445213318 15.455997467041 9.65693950653076 9.78940963745117 8.63870334625244 3.36940693855286 10.0999460220337 13.9773950576782 6.06264209747314 9.46984481811523 32.2826690673828 10.9813976287842 14.9599123001099 20.5842189788818 13.2702474594116 11.2084255218506 7.15034770965576 6.20550537109375 6.52875232696533 10.7823572158813 2.50054597854614 26.9959602355957 39.3167877197266 4.29864835739136 31.8540954589844 12.2739286422729 29.1977481842041 15.3287267684937 6.02900314331055 9.80965518951416 20.5986175537109 10.4683971405029 13.3646144866943 10.6696462631226 5.70409727096558 10.3447971343994 39.1357421875 12.1589689254761 12.2227058410645 2.74254727363586 16.2513484954834 38.9663734436035 5.45642995834351 19.9061489105225 8.97916507720947 5.4244499206543 8.75581550598145 9.63303375244141 10.7366199493408 8.43794918060303 12.8982706069946 28.2365036010742 20.8453922271729 14.4140396118164 3.77429628372192 10.2184715270996 3.89520311355591 2.58349585533142 20.0047054290771 12.7375860214233 2.53550338745117 4.38707971572876 2.4375364780426 17.432746887207 22.8430404663086 7.15299892425537 7.18354749679565 16.5867252349854 3.22359156608582 5.8759126663208 10.0314922332764 3.64302372932434 28.1822090148926 14.7369070053101 10.4734363555908 1.98020815849304 27.5886096954346 9.50574111938477 13.6234102249146 6.78766822814941 16.5997753143311 25.7585926055908 3.94411253929138 19.1748313903809 7.11864280700684 9.48776054382324 11.0334129333496 25.1413116455078 25.8080577850342 3.99606347084045 2.59960126876831 12.350733757019 3.54847741127014 16.7864589691162 3.4254367351532 1.41879534721375 5.82726907730103 3.78542947769165 24.6926765441895 21.9319763183594 16.139045715332 21.7920169830322 2.74781560897827 2.53915452957153 18.0867195129395 2.01608562469482 7.47128486633301 8.30909442901611 2.88330936431885 25.1509170532227 7.0669903755188 17.2121334075928 3.22834086418152 10.5941171646118 16.593074798584 10.308648109436 4.90706920623779 10.5649948120117 25.123327255249 5.00528717041016 11.216100692749 18.1576175689697 6.22122764587402 5.951012134552 8.2572135925293 18.5092811584473 21.0076847076416 1.5376558303833 8.55489444732666 17.1246719360352

ENSG00000204516.8 10.4730710983276 5.81598091125488 2.92060279846191 5.05618190765381 19.4797706604004 11.5706911087036 3.67939019203186 9.2778844833374 10.1504964828491 6.38873767852783 4.46961832046509 11.221302986145 2.06305766105652 3.51817655563354 5.25541543960571 14.8891286849976 30.3674964904785 6.59293460845947 12.6807088851929 19.671760559082 5.16819524765015 25.3518676757812 4.2473201751709 27.5033149719238 7.26004505157471 18.9562244415283 6.2498459815979 5.32994270324707 13.2543745040894 27.0603561401367 36.7961921691895 12.1001873016357 17.5450782775879 10.6963338851929 28.1603851318359 12.1665687561035 6.53929042816162 16.4997653961182 15.508451461792 6.41213226318359 17.2123336791992 9.26824760437012 18.0299186706543 5.10853719711304 4.78801012039185 34.4641494750977 10.0622158050537 8.51139354705811 13.6099557876587 13.1408491134644 8.44068431854248 25.20285987854 5.90641021728516 10.6520795822144 16.5731353759766 11.8860673904419 0.744058728218079 24.9847507476807 3.25800204277039 35.4371299743652 8.40232276916504 14.2081212997437 20.7142639160156 16.0783882141113 5.27411270141602 11.8500137329102 10.0001640319824 12.1554155349731 8.16589641571045 11.1019926071167 8.07296180725098 8.26054859161377 8.9734411239624 14.4524965286255 30.1961421966553 15.7777280807495 11.8243799209595 27.2783851623535 5.10450458526611 3.00112557411194 16.0728778839111 6.49174737930298 32.2743453979492 9.3751335144043 18.2594356536865 8.70430469512939 5.85730266571045 3.0408627986908 5.48404598236084 11.5537405014038 10.2865877151489 8.89651966094971 21.3628826141357 7.06528472900391 35.9406967163086 4.81331920623779 12.6243162155151 6.54055023193359 15.664514541626 4.15224981307983 15.0554065704346 15.7459917068481 3.00443577766418 2.47739696502686 2.33127546310425 11.0588636398315 23.3214664459229 7.64351940155029 15.7773876190186 5.21436929702759 27.1874732971191 20.3268795013428 3.54675912857056 20.6511631011963 5.14618158340454 4.10115814208984 11.6066207885742 1.99999499320984 7.23544549942017 10.1572065353394 11.0393772125244 28.0658397674561 70.6619644165039 19.7863121032715 25.7805995941162 18.9309692382812 4.12706565856934 24.0070209503174 7.85097074508667 1.54315006732941 18.334888458252 8.59515476226807 21.7042961120605 17.7345886230469 7.79599046707153 21.5493545532227 5.30137920379639 6.81170845031738 11.0056886672974 6.09507322311401 4.86270761489868 7.98540306091309 16.7351989746094 5.19187498092651 6.47142696380615 14.5342206954956 15.0575380325317 0.894873797893524 13.7956352233887 7.54546785354614 13.0824098587036 9.046875 33.5239372253418 26.5085048675537 5.15535354614258 16.5652503967285 6.83885765075684 11.5783910751343 10.751446723938 6.74926376342773 9.16435527801514 6.18195533752441 21.0903759002686 7.37127208709717 4.09224462509155 23.0723152160645 10.578631401062 13.6291828155518 17.6852931976318 17.6617584228516 9.7375020980835 3.38904547691345 24.328182220459 8.04106712341309 7.08523273468018 9.50415802001953 19.76708984375 15.3370151519775 15.6919717788696 5.75903081893921 12.9448432922363 36.5858764648438 4.7193021774292 2.44077181816101 3.51178288459778 12.012001991272 3.11763620376587 11.4471817016602 15.8073825836182 9.32365703582764 13.9709024429321 4.74229764938354 8.96884918212891 6.65877532958984 3.23250222206116 42.8813858032227 16.6918354034424 28.0194187164307 8.5034122467041 7.12193870544434 19.2942047119141 5.28350687026978 8.62008666992188 6.98329591751099 7.32320785522461 6.05847072601318 2.66539621353149 8.96739387512207 2.83792209625244 13.0782995223999 19.2028656005859 4.48027324676514 15.418794631958 10.4637451171875 15.539288520813 3.29538345336914 2.06643414497375 13.4786310195923 1.78315472602844 12.268705368042 3.5217010974884 1.57691884040833 13.3248338699341 28.3586978912354 10.7601537704468 42.7065391540527 17.6140041351318 2.80093097686768 1.11999917030334 11.1080961227417 11.4516067504883 6.90225315093994 20.136754989624 4.43328905105591 13.6838541030884 9.50809097290039 17.8433742523193 17.3667221069336 7.81734466552734 10.2877321243286 16.6030254364014 14.2342987060547 13.1065082550049 4.49258661270142 9.65068912506104 6.00352478027344 13.7777166366577 28.2743034362793 19.3049869537354 14.6936779022217 11.7040672302246 9.96951675415039 9.71008777618408 6.11698341369629 4.19280862808228 9.30940055847168

ENSG00000001167.13 46.1929740905762 20.1301784515381 17.9499073028564 25.4250736236572 64.0381851196289 26.8228988647461 54.0143547058105 38.5966339111328 46.422981262207 15.3537340164185 16.8423976898193 20.0630836486816 50.6834335327148 50.1187591552734 28.6376800537109 54.519905090332 61.5986938476562 43.8925743103027 82.3428497314453 27.0134220123291 25.0297508239746 31.194953918457 116.592887878418 34.6439552307129 43.6343841552734 25.4099445343018 67.3501205444336 19.2601089477539 45.8045349121094 80.319465637207 37.5964546203613 14.0483722686768 38.3414039611816 54.3418350219727 23.2309036254883 40.1662254333496 20.5635528564453 15.8194198608398 32.3812141418457 19.6002635955811 57.6055068969727 33.8276786804199 40.6815910339355 15.1777257919312 19.5668430328369 33.2096061706543 34.4381446838379 69.9307632446289 22.7240619659424 51.5570373535156 50.2325172424316 43.0738563537598 43.2766952514648 23.0634632110596 28.906551361084 38.1592063903809 82.1488342285156 115.36018371582 45.0702056884766 96.2183532714844 42.1541862487793 31.5385112762451 50.9376640319824 55.6950302124023 34.4391441345215 49.843017578125 27.2097206115723 49.2638359069824 46.3719940185547 32.5930557250977 98.1341094970703 18.6059894561768 33.6188735961914 34.8576202392578 63.3203659057617 50.6211853027344 17.176664352417 72.2573852539062 39.4901695251465 199.622772216797 25.3145389556885 41.7673873901367 67.1576461791992 44.2703742980957 32.3955497741699 27.3147354125977 54.9246559143066 9.54718589782715 41.8000183105469 43.6250381469727 14.983208656311 45.6790199279785 329.262664794922 40.1580772399902 45.3024673461914 100.403396606445 21.7662906646729 44.4135246276855 35.7075309753418 42.2645301818848 23.0627822875977 32.5234184265137 45.9965324401855 30.5918502807617 33.1418151855469 42.713134765625 34.5853385925293 45.9357528686523 25.3879985809326 14.4334621429443 87.7119750976562 57.1834678649902 17.9568996429443 42.9673690795898 57.3255958557129 18.4689388275146 32.7217025756836 24.0350208282471 37.1067848205566 54.4503059387207 35.9439392089844 60.3912239074707 37.5864143371582 43.4085960388184 129.344192504883 52.1709327697754 54.6103286743164 44.9442863464355 31.0645809173584 38.1301727294922 34.9178848266602 19.826961517334 62.1301879882812 60.4663887023926 22.0809268951416 16.2977428436279 54.8583030700684 33.9024887084961 41.7496452331543 23.2365665435791 80.9408569335938 43.7316246032715 51.1557197570801 23.1476726531982 62.8193626403809 19.4070739746094 26.5110702514648 40.2234802246094 32.5852890014648 37.2398414611816 94.5730895996094 43.110221862793 25.4165096282959 56.6480865478516 31.6654567718506 20.5100803375244 39.6817779541016 25.0696716308594 35.5212478637695 47.6322937011719 43.5742874145508 33.5179481506348 38.6549186706543 29.3189353942871 33.9752655029297 29.5821075439453 36.4848403930664 22.3878288269043 25.1520557403564 25.7454071044922 41.4938468933105 19.2104072570801 23.7173156738281 32.3171539306641 36.2608795166016 25.6395874023438 45.7673606872559 45.0243530273438 24.1210346221924 39.3792953491211 25.1748714447021 27.4852352142334 46.0267524719238 29.3056201934814 17.7886600494385 49.9818687438965 38.4087181091309 18.0904350280762 37.6487998962402 37.3187217712402 28.2950077056885 22.6594524383545 90.9621658325195 44.2776069641113 17.7082633972168 62.6233215332031 26.1860237121582 38.6609420776367 25.9829444885254 52.2743492126465 29.8195285797119 25.0286312103271 46.7270011901855 11.5359058380127 18.4012050628662 34.4234313964844 83.5320281982422 39.5734481811523 23.6547069549561 21.8891353607178 34.3978805541992 19.7362442016602 40.0870208740234 59.3529853820801 33.6285514831543 23.3879127502441 16.4195442199707 26.5381107330322 51.8831825256348 15.9337739944458 17.3142833709717 14.0441188812256 37.6717224121094 28.3851280212402 23.3261394500732 57.829029083252 34.4379501342773 35.5303115844727 51.206413269043 34.1635551452637 58.1610679626465 35.8483543395996 52.009349822998 35.2204170227051 36.3970375061035 61.9639663696289 47.3890342712402 27.4345169067383 19.6096858978271 66.8686904907227 42.3945579528809 80.0066680908203 27.0419158935547 22.7611103057861 18.2293758392334 36.902172088623 25.568359375 50.9872398376465 18.8366508483887 22.5636825561523 32.0847091674805 41.7145118713379 33.661735534668 36.120719909668 43.047233581543 32.7833480834961

ENSG00000120837.6 16.6531753540039 17.1335430145264 17.3391590118408 20.0537281036377 34.9079933166504 20.4454669952393 26.7422866821289 18.2739753723145 16.6328544616699 8.56032371520996 11.0962934494019 11.7977657318115 26.3281707763672 15.7138061523438 21.0414867401123 22.5164184570312 20.164852142334 12.7872934341431 14.5448894500732 23.7639541625977 12.181809425354 13.462589263916 17.4643669128418 21.3944568634033 14.0405378341675 19.7218284606934 20.6513156890869 10.4108505249023 26.1219692230225 23.8253650665283 31.6976013183594 17.2255382537842 16.6596927642822 25.869966506958 16.7453117370605 19.6720352172852 14.767502784729 14.1798696517944 15.5437803268433 13.5788440704346 17.1350650787354 19.3400497436523 25.4863567352295 15.9260358810425 17.8984851837158 16.0822200775146 16.924295425415 20.3422756195068 20.6338863372803 17.7734851837158 33.2113800048828 21.4835166931152 13.9445695877075 9.15480518341064 11.7487173080444 25.8201580047607 24.7842426300049 18.8020057678223 15.8854198455811 25.3876762390137 18.0711765289307 18.2954216003418 17.7693920135498 18.2005977630615 20.5724830627441 26.9403209686279 17.2230129241943 17.7896919250488 13.4856357574463 23.6608581542969 16.5244998931885 15.1235275268555 19.6077575683594 26.5633087158203 16.4055709838867 17.2571640014648 18.0401992797852 18.0686817169189 9.99446964263916 8.39379978179932 15.2158155441284 15.9526376724243 19.478343963623 10.096152305603 24.3247394561768 34.7162590026855 18.5766983032227 13.4066543579102 15.3065462112427 19.304479598999 29.7469959259033 41.5865592956543 21.6734142303467 18.6810436248779 15.2972002029419 25.8598918914795 18.379711151123 23.6668300628662 33.115550994873 29.1762828826904 19.0882968902588 15.215989112854 25.2600193023682 17.9028415679932 16.7664184570312 13.8343830108643 19.4932098388672 22.5699920654297 18.5935382843018 7.73952198028564 17.0236377716064 22.2892074584961 10.7067708969116 18.1295280456543 19.8366680145264 18.1691246032715 30.895544052124 21.8398208618164 22.5938625335693 14.4726238250732 16.4280071258545 11.2792940139771 15.7191743850708 21.1499004364014 19.5911464691162 17.2658538818359 17.3594627380371 18.6841735839844 11.2840309143066 7.14995956420898 28.1148948669434 10.7541284561157 21.9026966094971 16.5506191253662 15.5221605300903 17.3289966583252 14.3098802566528 20.6962509155273 11.9411573410034 18.0975551605225 25.0887088775635 16.3413619995117 30.6383895874023 17.4646511077881 32.961051940918 11.8278331756592 39.1790618896484 12.651481628418 13.9584665298462 24.5656051635742 22.3421440124512 23.0721645355225 16.5226402282715 11.2361240386963 26.3661460876465 12.4047765731812 17.2538509368896 11.9455528259277 10.9344749450684 16.1149005889893 37.7913932800293 19.9879150390625 20.5430850982666 12.8887948989868 14.0219430923462 23.0177116394043 16.6166439056396 27.7032566070557 18.9197769165039 21.4849510192871 19.6590023040771 27.3074073791504 12.1928243637085 16.527982711792 12.7038927078247 20.8844337463379 15.2162733078003 20.8715629577637 17.1996879577637 13.7495565414429 28.7402973175049 21.6443462371826 14.4155111312866 33.7773666381836 16.0607223510742 12.1939830780029 25.9274120330811 20.3926124572754 24.862585067749 14.2109937667847 24.1779613494873 17.7698345184326 23.9408721923828 25.691873550415 10.3281145095825 27.109806060791 20.427209854126 23.2630481719971 18.5277729034424 15.9846754074097 8.86598873138428 22.0475215911865 29.100944519043 9.8369140625 15.6781635284424 19.533483505249 9.05028438568115 28.3166751861572 12.6173286437988 15.0805263519287 27.9733009338379 15.8052177429199 17.2845420837402 15.845344543457 13.2935667037964 10.347749710083 8.68396377563477 16.0933856964111 20.8345699310303 16.2470626831055 13.3496150970459 13.8186655044556 19.8062362670898 14.0683269500732 12.4121742248535 25.3353862762451 21.2028388977051 20.7035312652588 36.4587593078613 16.0991439819336 20.2583770751953 28.4722442626953 20.2954540252686 16.7968769073486 31.1558322906494 14.611120223999 19.6941184997559 12.7923974990845 13.328221321106 19.8742523193359 21.7499313354492 17.4315319061279 13.5939998626709 21.8033428192139 19.8173542022705 18.1541805267334 19.7866821289062 22.4603137969971 12.926700592041 21.7293643951416 14.9426918029785 14.5141620635986 20.7347259521484 29.8477115631104 14.0891370773315 17.0218963623047

ENSG00000066136.18 20.8361473083496 21.8576145172119 50.9632987976074 17.859733581543 24.8315753936768 23.4636688232422 36.3779449462891 27.1508808135986 23.5279197692871 22.9386978149414 38.4078216552734 34.9509735107422 37.6334991455078 38.6725196838379 24.5221309661865 34.6972160339355 45.1215133666992 17.8810882568359 16.6214218139648 22.3257617950439 28.4183864593506 50.2846069335938 57.4889221191406 30.4869728088379 20.1928043365479 27.6793804168701 51.8572998046875 25.6502304077148 21.4916610717773 18.4720230102539 26.5957717895508 21.9041538238525 27.0455474853516 39.639949798584 11.5885915756226 42.5527648925781 34.8757743835449 29.2591094970703 24.188497543335 17.9586791992188 25.9998531341553 33.6100845336914 33.4123802185059 26.2673568725586 110.770851135254 15.7912683486938 31.6100254058838 34.4062461853027 19.3910598754883 36.539909362793 19.6988220214844 28.4159393310547 22.8195419311523 26.0135231018066 31.3388290405273 46.6736068725586 23.2745532989502 32.9180641174316 26.9836978912354 24.2485828399658 46.1004295349121 25.0509433746338 16.3781661987305 55.2337608337402 20.3121662139893 18.0456714630127 35.8558578491211 34.1281394958496 24.3467407226562 29.2633495330811 30.8914813995361 21.357608795166 24.9199733734131 18.3387260437012 37.5390625 16.2531681060791 12.3975124359131 22.0215244293213 24.5263156890869 60.3163909912109 26.9897155761719 33.0719337463379 28.5377712249756 23.3286609649658 18.631175994873 21.9022808074951 24.512825012207 19.6672039031982 22.9456462860107 33.1696319580078 91.9666519165039 28.9416561126709 24.9294204711914 21.6104412078857 18.2064342498779 39.5415954589844 16.1499347686768 22.5874099731445 28.8920974731445 43.6859970092773 22.0190467834473 44.2077598571777 44.8906402587891 42.1036186218262 28.4147701263428 23.6007194519043 22.9959831237793 34.5302505493164 27.4879417419434 19.8361740112305 19.1049766540527 29.2700862884521 7.92438840866089 41.4001007080078 21.5084476470947 33.075740814209 23.9544448852539 20.1673336029053 27.4653987884521 20.0910015106201 22.4223899841309 19.6144523620605 38.5156059265137 21.0045909881592 31.0831050872803 26.0429515838623 23.2865810394287 29.5635032653809 28.7124137878418 29.4683170318604 30.5874633789062 17.3512210845947 21.5677909851074 21.6858596801758 16.7756824493408 16.4565715789795 35.4868392944336 33.9893455505371 25.0957107543945 41.7617073059082 34.4845237731934 23.3364086151123 27.3795356750488 19.0738906860352 36.7115478515625 6.66410207748413 18.9298000335693 26.0016078948975 22.7357635498047 20.2570934295654 39.3096656799316 26.7396793365479 17.0827617645264 28.853048324585 17.4015197753906 20.9596614837646 32.6480407714844 24.391134262085 30.6068553924561 25.9085311889648 22.0231018066406 20.263256072998 27.2532348632812 29.1605587005615 42.4151649475098 27.1938533782959 21.4587631225586 33.2103271484375 37.670597076416 23.8761291503906 52.2460441589355 15.2594442367554 21.3106632232666 28.6461524963379 21.7945461273193 15.3524312973022 26.9242935180664 25.311466217041 18.5891017913818 34.9446868896484 23.8590888977051 18.058048248291 22.2551937103271 36.2068099975586 17.9428157806396 24.5234355926514 15.5583305358887 14.3646249771118 26.2967872619629 29.0056209564209 23.8467044830322 22.9075374603271 18.9045352935791 32.8777160644531 32.1528778076172 46.3195419311523 20.3397388458252 19.0194320678711 19.1138668060303 35.6584625244141 10.1580057144165 18.0707263946533 27.0366172790527 29.3511753082275 15.8809242248535 23.3698043823242 25.6516952514648 30.7600421905518 13.6535663604736 24.5222911834717 23.3662624359131 25.8534450531006 19.9832897186279 28.4233856201172 14.0072393417358 12.5035266876221 13.8162975311279 26.4756126403809 26.4899749755859 23.2109622955322 21.8499202728271 20.512414932251 22.0273418426514 13.6335077285767 26.8089027404785 34.469596862793 25.6539096832275 27.9353160858154 20.9745044708252 34.8859062194824 34.9012336730957 20.9755668640137 34.1636238098145 24.3770580291748 35.0419158935547 31.1823768615723 43.5063438415527 29.0530738830566 20.1454734802246 22.4147796630859 32.4794692993164 22.3771705627441 19.4807586669922 31.2793483734131 24.6927433013916 24.3582515716553 17.5512809753418 37.2972640991211 15.4104900360107 19.8273677825928 25.2628707885742 28.5185394287109 22.2917060852051 26.7351322174072 29.7888317108154 25.1626014709473

ENSG00000100600.13 92.6089935302734 149.749801635742 84.7843780517578 59.0815773010254 117.512283325195 130.766418457031 197.299682617188 243.326248168945 104.252868652344 246.105895996094 77.2345886230469 132.746398925781 163.163162231445 122.591125488281 166.148788452148 166.083358764648 160.274185180664 270.808044433594 115.494529724121 379.895263671875 78.6000595092773 415.295013427734 174.300247192383 310.511535644531 123.597808837891 129.257736206055 128.969055175781 97.8288192749023 177.968032836914 158.688095092773 190.428970336914 380.300964355469 429.111145019531 224.449066162109 94.238410949707 178.301895141602 119.439491271973 151.179946899414 425.294738769531 106.747032165527 174.772277832031 139.973114013672 78.4649353027344 161.464614868164 78.8883895874023 118.433090209961 475.401947021484 152.811737060547 297.6123046875 259.585052490234 265.437103271484 206.383544921875 79.6549072265625 63.3198089599609 277.6806640625 149.017364501953 126.71964263916 368.117492675781 255.234298706055 120.013938903809 140.871841430664 143.778350830078 190.999526977539 191.481155395508 137.127624511719 205.909317016602 158.017303466797 165.604675292969 284.788513183594 188.365173339844 204.646911621094 313.354797363281 180.558303833008 141.722030639648 149.236541748047 138.344863891602 93.3193054199219 430.066284179688 143.133453369141 111.49560546875 199.890426635742 220.484893798828 126.561019897461 243.118927001953 560.798645019531 413.369445800781 152.442352294922 85.5215072631836 136.052825927734 230.595916748047 69.6648178100586 300.611450195312 361.194610595703 258.938781738281 146.376388549805 118.001678466797 165.444549560547 203.964920043945 411.627990722656 209.47688293457 219.301498413086 198.526901245117 184.32942199707 116.253112792969 248.835586547852 175.644683837891 146.402938842773 241.327377319336 48.5707130432129 121.763786315918 172.706207275391 233.122787475586 87.5412902832031 314.778259277344 159.890640258789 122.371841430664 319.72216796875 438.993408203125 97.0560684204102 40.2631225585938 329.939422607422 145.241455078125 147.652038574219 217.760848999023 415.572143554688 120.375747680664 215.726745605469 141.186752319336 125.238502502441 172.830032348633 197.952789306641 441.073150634766 123.463813781738 140.152893066406 132.951568603516 173.929458618164 124.180557250977 178.496307373047 151.23974609375 188.128280639648 237.902465820312 255.721282958984 149.801345825195 90.5820617675781 191.358917236328 81.9017028808594 94.5304183959961 309.432281494141 214.70964050293 58.8205337524414 352.298004150391 246.654510498047 63.3498382568359 178.512359619141 138.891571044922 136.925598144531 167.091842651367 255.038619995117 264.049041748047 158.581192016602 163.282211303711 141.771713256836 276.949951171875 85.646369934082 134.403717041016 50.1964378356934 199.091552734375 338.018615722656 219.915481567383 174.662628173828 356.351104736328 78.6480941772461 282.365875244141 183.798858642578 113.366218566895 161.107879638672 213.650299072266 263.753265380859 334.793334960938 291.845245361328 113.665626525879 154.329864501953 243.706802368164 56.0494918823242 126.598243713379 162.306762695312 123.62149810791 63.9833106994629 109.717956542969 156.300155639648 160.84001159668 156.134017944336 172.944183349609 142.620758056641 142.649291992188 173.392578125 145.819320678711 55.6817321777344 125.336502075195 109.221672058105 81.9362106323242 88.6563262939453 151.618469238281 51.3581123352051 188.704437255859 229.222229003906 309.864074707031 194.38166809082 91.6447219848633 56.7710571289062 146.118591308594 104.742721557617 136.648422241211 293.903350830078 181.082870483398 87.5213317871094 202.465621948242 180.918273925781 64.1899948120117 149.022903442383 163.638977050781 116.196907043457 216.361831665039 101.705665588379 281.762603759766 310.253387451172 68.9362106323242 237.515426635742 126.569793701172 110.376457214355 197.879470825195 232.259948730469 160.529708862305 238.256820678711 200.626480102539 116.032653808594 360.983093261719 179.933670043945 198.866241455078 174.805099487305 290.865386962891 297.356323242188 114.549522399902 96.5789108276367 163.551513671875 83.4305191040039 152.271423339844 160.610000610352 153.034423828125 137.002456665039 143.811431884766 240.199783325195 194.481018066406 184.397445678711 174.12776184082 309.590942382812

ENSG00000204264.7 259.661346435547 147.971786499023 213.395385742188 512.787841796875 206.956817626953 148.906600952148 84.75390625 210.335815429688 165.6494140625 85.7842864990234 121.300354003906 137.932830810547 73.5207138061523 81.6021041870117 183.930877685547 115.135215759277 77.5660552978516 162.890655517578 148.612426757812 250.595520019531 72.8076553344727 279.706634521484 14.6795043945312 160.509780883789 151.842483520508 197.652297973633 274.670196533203 154.804077148438 101.105133056641 301.765319824219 223.087905883789 130.336502075195 135.222991943359 176.868728637695 467.947631835938 105.861221313477 175.133544921875 173.312942504883 111.793296813965 111.292175292969 211.046936035156 228.076065063477 104.562774658203 57.434398651123 87.4574508666992 399.838073730469 168.047775268555 376.864440917969 126.399436950684 494.537078857422 116.125045776367 287.201263427734 133.935638427734 246.429428100586 213.756973266602 181.237213134766 83.2784194946289 155.203903198242 38.789722442627 235.856674194336 230.696502685547 213.171783447266 408.617279052734 398.996887207031 88.6392211914062 206.703826904297 185.765579223633 148.864471435547 130.093032836914 88.8287353515625 225.365966796875 116.536201477051 147.002075195312 289.425811767578 194.687805175781 350.993713378906 518.93505859375 145.986801147461 116.189765930176 17.2733325958252 165.226348876953 121.911598205566 297.221893310547 102.020378112793 104.164016723633 135.508102416992 147.658157348633 94.400016784668 93.8954620361328 136.300582885742 65.6035461425781 231.999176025391 219.682952880859 151.68132019043 174.323837280273 160.062194824219 202.32453918457 833.49072265625 159.527114868164 97.8321914672852 331.023071289062 289.047729492188 82.0983276367188 55.5185585021973 135.349395751953 184.112106323242 300.292907714844 125.37100982666 514.892456054688 42.612060546875 217.938980102539 170.392135620117 442.646148681641 199.965698242188 96.0363464355469 130.402328491211 89.6435623168945 56.5265960693359 282.86279296875 238.822387695312 101.048683166504 331.281005859375 479.693359375 177.650985717773 182.190322875977 281.995971679688 112.976707458496 441.098602294922 189.839218139648 119.866966247559 153.04231262207 305.853820800781 67.0868988037109 156.298538208008 263.125885009766 258.666015625 206.012054443359 134.011840820312 158.578262329102 164.897674560547 118.076293945312 109.414649963379 268.214508056641 112.791900634766 174.625137329102 76.0220947265625 134.835403442383 158.883422851562 207.90007019043 73.905632019043 419.741577148438 290.037231445312 435.789611816406 321.788055419922 137.893539428711 153.235473632812 349.691741943359 91.7175445556641 263.392211914062 122.577842712402 135.941787719727 101.912231445312 202.446929931641 167.166931152344 229.824813842773 374.170227050781 360.729919433594 143.289764404297 158.611877441406 362.327972412109 194.82048034668 382.477447509766 507.933135986328 158.101303100586 648.581359863281 107.117385864258 144.444259643555 176.406707763672 141.627395629883 151.224182128906 59.5431327819824 222.953186035156 16.8157005310059 88.6396484375 123.081848144531 156.521514892578 38.1190071105957 523.436401367188 218.074325561523 165.089416503906 135.632797241211 296.804870605469 205.248947143555 96.4410095214844 69.117561340332 184.053985595703 206.182922363281 100.033226013184 179.395812988281 82.6913604736328 181.637985229492 288.949157714844 257.222106933594 122.123580932617 135.755416870117 201.622970581055 104.043502807617 157.603530883789 114.007095336914 381.450469970703 154.589294433594 119.853736877441 172.891784667969 393.110046386719 199.991668701172 63.6499176025391 75.6834716796875 204.607116699219 97.891845703125 133.179092407227 133.504501342773 162.445037841797 152.069732666016 529.266662597656 114.07640838623 453.931610107422 266.787811279297 134.205184936523 142.266006469727 86.4678344726562 146.282089233398 143.340850830078 388.512664794922 91.6462554931641 143.610702514648 277.496887207031 118.199012756348 167.969528198242 159.882217407227 201.708908081055 238.959411621094 219.515670776367 130.822250366211 80.5281753540039 140.584808349609 89.0720977783203 333.248687744141 283.071014404297 299.474243164062 113.108688354492 244.640319824219 216.739105224609 161.993377685547 97.8749542236328 124.496376037598 137.254928588867

ENSG00000100764.12 12.5231790542603 6.59334659576416 10.9280624389648 15.351658821106 13.4534091949463 6.08220767974854 8.8253927230835 9.62582492828369 4.14883613586426 4.39082527160645 4.40924072265625 4.71707105636597 20.6948623657227 6.64878225326538 7.22588348388672 12.2004728317261 8.79058265686035 10.6793947219849 9.70541954040527 7.78329753875732 6.18633890151978 17.4515705108643 15.2605638504028 12.5273771286011 5.26137399673462 9.99481868743896 12.6858434677124 5.20285129547119 7.783371925354 13.3317451477051 11.4887313842773 6.78362798690796 11.3293924331665 9.04073619842529 13.5087366104126 7.74309349060059 9.51963138580322 5.88690853118896 10.5465545654297 5.38219547271729 9.64974594116211 11.3137044906616 5.41746854782104 8.64273834228516 8.89770126342773 13.0176887512207 9.36404991149902 11.0808820724487 4.38914823532104 7.80124664306641 10.7097816467285 10.3162851333618 5.20218896865845 7.02532052993774 9.5817403793335 15.8137521743774 15.1238498687744 30.1044311523438 13.9395027160645 12.3565149307251 6.82544612884521 9.16044425964355 9.37144374847412 8.62202835083008 6.55057144165039 8.9275016784668 6.94911050796509 13.874963760376 9.90199565887451 9.78176116943359 12.0675525665283 13.4116249084473 7.39584493637085 8.62860679626465 9.04057121276855 12.204909324646 10.8678722381592 9.08733558654785 13.7242116928101 6.76776313781738 6.87208271026611 15.7095336914062 12.574182510376 9.5844898223877 23.2341289520264 13.8143672943115 14.9368534088135 5.25143051147461 16.0633964538574 10.70667552948 8.11150074005127 12.3078718185425 11.210883140564 16.8321914672852 6.54588460922241 8.30189323425293 6.68203639984131 115.50609588623 12.0378704071045 8.23347663879395 6.37843227386475 8.57855129241943 10.2954378128052 5.36834669113159 6.53452825546265 6.68573474884033 9.07471084594727 8.66031074523926 2.77488732337952 4.11551237106323 8.52052593231201 9.66234302520752 6.56495046615601 15.6418514251709 5.74494886398315 4.16399765014648 10.8312120437622 5.44975090026855 7.64616823196411 8.04810810089111 9.7068452835083 4.20437431335449 12.2818002700806 9.265456199646 14.5899829864502 9.08194065093994 11.2743835449219 5.62196779251099 7.37594747543335 4.14448738098145 13.3853578567505 8.30909824371338 6.64570808410645 10.207371711731 8.14321041107178 8.55297470092773 12.8102798461914 8.99743938446045 6.47374868392944 7.0329213142395 8.91413593292236 10.503378868103 16.7899551391602 4.94255065917969 12.1848497390747 6.64527273178101 9.37347888946533 13.4677867889404 9.26649379730225 6.61236238479614 16.0595684051514 9.82288837432861 5.66504096984863 7.07607316970825 6.74842023849487 7.69157648086548 7.86167430877686 5.23502540588379 7.947434425354 4.02302026748657 10.5150175094604 5.47650527954102 11.5168380737305 5.53606748580933 7.39885711669922 7.44976139068604 8.40744495391846 5.96280717849731 10.9604892730713 9.61210536956787 10.2596378326416 7.40138101577759 8.96456050872803 7.10077428817749 5.84852695465088 5.38395023345947 9.16099834442139 8.89889907836914 11.9998273849487 10.8261280059814 3.81465554237366 16.8961906433105 20.683837890625 9.27329254150391 6.60424327850342 6.67324447631836 7.21990871429443 8.8728609085083 8.16758251190186 5.69365549087524 8.03920650482178 12.4706211090088 9.95995712280273 6.81911277770996 12.4863319396973 19.3973274230957 8.52811622619629 13.9532871246338 6.5330638885498 10.9634952545166 13.0992431640625 6.84376001358032 10.273832321167 1.46053302288055 6.89310455322266 9.59135341644287 10.4447288513184 12.6846446990967 4.77626657485962 13.5980930328369 10.9684467315674 6.24524402618408 8.29914093017578 13.9009418487549 7.87891149520874 3.81986427307129 4.40473079681396 6.45558786392212 7.81646966934204 6.577965259552 4.59818935394287 7.22380495071411 9.97131824493408 8.72572612762451 7.33003759384155 20.5284595489502 7.10074329376221 8.19755554199219 12.8895101547241 8.55527400970459 7.53974866867065 12.280556678772 12.7459545135498 6.4939751625061 16.4070949554443 10.0310258865356 11.92076587677 5.96888017654419 6.62451219558716 10.5621433258057 7.11315822601318 13.9984178543091 14.7244882583618 7.31211280822754 5.78450107574463 6.17504835128784 10.6111335754395 9.45436763763428 13.1809387207031 7.22418928146362 11.3063087463379 10.4107465744019 11.7209949493408 14.1480073928833 16.0710411071777 6.91538095474243

ENSG00000161057.9 124.92066192627 56.5430145263672 140.080413818359 245.577011108398 125.95149230957 55.5354919433594 69.2706069946289 108.551345825195 69.2513732910156 37.7252082824707 40.4742088317871 47.353946685791 137.713851928711 78.3089141845703 53.1536636352539 49.742301940918 57.958553314209 77.8901214599609 64.1390686035156 97.0364379882812 82.6349716186523 131.213928222656 153.828506469727 70.7221145629883 64.8062286376953 183.758041381836 67.9476013183594 48.4983825683594 71.3782501220703 99.7112579345703 157.940170288086 59.041446685791 89.911750793457 64.6182250976562 142.32926940918 42.054630279541 77.4551467895508 76.2368774414062 56.9900093078613 59.8257904052734 104.780029296875 95.1057815551758 117.113182067871 81.4779891967773 81.7730102539062 155.471801757812 109.28572845459 77.6078872680664 51.2527961730957 78.2775726318359 52.844181060791 86.8505935668945 82.6868286132812 67.4197463989258 53.5894546508789 105.42960357666 124.079864501953 113.579856872559 103.518768310547 78.7509841918945 77.2866516113281 69.8198318481445 106.899757385254 108.764701843262 65.4087295532227 70.8335876464844 71.0816879272461 64.9201278686523 69.2141418457031 62.4532089233398 73.8295364379883 100.44165802002 67.9285430908203 66.4844055175781 104.476692199707 105.898162841797 109.231155395508 44.1075096130371 174.551177978516 65.7463760375977 76.5345840454102 99.553955078125 370.083679199219 121.567604064941 61.1793212890625 79.0889053344727 58.436149597168 82.2709426879883 108.617851257324 90.3954544067383 73.37255859375 69.2106475830078 87.5910034179688 89.7961959838867 116.05354309082 88.5406875610352 109.664176940918 68.7489929199219 83.4442672729492 60.9887924194336 94.5201644897461 78.6976470947266 80.9201889038086 50.5294876098633 91.314567565918 108.444396972656 92.1886596679688 85.5622100830078 81.7539291381836 44.8789749145508 81.1884994506836 92.6209106445312 43.0206756591797 106.951858520508 76.0234069824219 64.2269744873047 47.0424118041992 56.6491394042969 66.9045104980469 69.1071319580078 72.7494277954102 57.7507743835449 116.580871582031 99.9608001708984 99.1055068969727 98.6458892822266 116.103645324707 104.633926391602 49.3866577148438 72.9497680664062 118.906097412109 68.3437423706055 87.6773986816406 96.7088928222656 127.28125 62.0104751586914 117.201683044434 64.8602828979492 76.231559753418 36.6695442199707 84.0706253051758 36.287971496582 89.7491836547852 49.4428443908691 123.493019104004 95.6432571411133 72.0189895629883 111.148857116699 72.0600204467773 34.6543998718262 165.225280761719 93.0927047729492 76.1453552246094 72.536262512207 56.0130996704102 67.9130020141602 82.4620819091797 59.8628158569336 68.2024993896484 77.3730087280273 60.1662521362305 47.2973976135254 98.4271469116211 83.4243011474609 55.5873527526855 121.984939575195 70.6727066040039 55.0213088989258 71.5237808227539 102.082153320312 139.384979248047 121.515556335449 85.2827987670898 88.3224334716797 109.675048828125 52.9795150756836 69.306526184082 96.8703384399414 64.4325332641602 49.6574859619141 58.5195655822754 115.469741821289 72.1020050048828 84.5785903930664 48.4996490478516 48.535961151123 56.0608520507812 106.564224243164 71.6008758544922 51.3067626953125 59.6383018493652 58.1918411254883 79.8474044799805 66.7509841918945 90.1481857299805 127.893432617188 94.7726058959961 120.119514465332 54.343074798584 71.4400482177734 49.7162322998047 88.5461883544922 67.2196655273438 43.3458099365234 76.0540924072266 63.3836364746094 70.4342422485352 104.991600036621 50.1512870788574 63.4302864074707 72.7214736938477 47.925220489502 68.3456878662109 87.0053176879883 75.3267974853516 52.8175392150879 36.401008605957 94.8110809326172 94.3757095336914 55.9925422668457 31.009220123291 61.6046409606934 71.7831420898438 199.589324951172 69.9151611328125 139.560424804688 125.553199768066 58.5769424438477 66.048225402832 85.6092147827148 104.350189208984 73.9718017578125 156.715057373047 75.4100952148438 77.5310668945312 77.1794662475586 67.5617904663086 59.1617965698242 106.084457397461 87.6448745727539 95.770393371582 93.4365692138672 84.9442672729492 52.9849624633789 50.1464691162109 76.7350921630859 141.321624755859 65.5066070556641 131.431594848633 43.1643943786621 79.4867858886719 58.1468276977539 77.7215194702148 109.19083404541 80.0048370361328 69.6640701293945

ENSG00000165916.7 155.320571899414 113.806648254395 328.739990234375 304.8486328125 160.285079956055 105.190010070801 127.441673278809 281.32080078125 149.194274902344 85.5037994384766 96.3941955566406 98.5784759521484 233.499404907227 165.433898925781 118.782844543457 204.498123168945 168.97428894043 168.290054321289 124.291282653809 102.331916809082 215.8154296875 300.217681884766 247.163665771484 126.28636932373 137.295150756836 150.062545776367 146.024368286133 78.4626693725586 133.696517944336 205.575042724609 141.624465942383 106.938911437988 122.37370300293 177.315811157227 200.824783325195 111.108917236328 131.304901123047 92.9218444824219 99.6105194091797 107.168640136719 207.796615600586 157.59635925293 81.9436187744141 149.430313110352 185.889450073242 179.23258972168 187.513946533203 156.525894165039 104.674613952637 164.202011108398 125.501365661621 215.69287109375 245.738662719727 215.068786621094 140.422378540039 256.210479736328 201.136962890625 164.549728393555 143.019073486328 202.291107177734 145.748962402344 158.045486450195 152.654479980469 272.600402832031 93.0808029174805 135.63720703125 135.408416748047 212.540252685547 111.587120056152 117.745529174805 140.165832519531 207.925735473633 149.923934936523 110.539627075195 188.945236206055 151.4794921875 134.564590454102 108.010887145996 185.686462402344 205.123992919922 154.279006958008 261.201721191406 144.772094726562 176.636917114258 143.085647583008 96.3057174682617 159.879653930664 112.787956237793 233.948791503906 170.379150390625 160.858001708984 240.371643066406 229.069534301758 153.519836425781 154.823043823242 257.624786376953 131.604110717773 207.800918579102 141.317337036133 117.836410522461 192.305862426758 233.397171020508 139.324783325195 109.699157714844 252.842758178711 133.599090576172 168.486129760742 183.797958374023 158.499237060547 125.259010314941 115.571464538574 155.458862304688 92.7610015869141 216.40446472168 135.785095214844 135.067733764648 106.826736450195 117.240852355957 196.319259643555 198.929275512695 130.180786132812 113.515625 316.52587890625 133.981842041016 145.503112792969 170.084182739258 149.109817504883 156.229904174805 127.128364562988 149.443420410156 162.509094238281 127.38599395752 125.787376403809 120.072288513184 133.92041015625 193.381942749023 174.367156982422 128.918045043945 158.663818359375 158.29606628418 127.849975585938 136.114044189453 125.863777160645 83.7155456542969 215.189498901367 68.0700759887695 156.419052124023 112.59349822998 111.778869628906 111.277877807617 224.251251220703 143.56037902832 113.27946472168 149.124328613281 89.3081741333008 152.098281860352 252.561965942383 95.4489517211914 128.517044067383 189.314575195312 134.419448852539 120.496597290039 167.435073852539 171.501052856445 151.145477294922 116.292167663574 153.0166015625 103.775924682617 219.023941040039 113.837394714355 186.026153564453 145.484298706055 167.851058959961 182.987579345703 201.016204833984 102.166496276855 156.254119873047 125.734550476074 107.206916809082 149.099807739258 85.2457962036133 123.496246337891 119.909698486328 126.096801757812 102.598915100098 230.48371887207 101.062767028809 186.534042358398 148.160568237305 108.156120300293 123.787773132324 160.793563842773 134.302124023438 143.963531494141 146.684036254883 249.958374023438 141.860305786133 149.888000488281 91.9882507324219 113.342460632324 116.014526367188 168.368911743164 165.815048217773 300.034210205078 96.8616333007812 154.224029541016 152.226303100586 136.990783691406 112.744621276855 122.201812744141 186.378997802734 90.0672302246094 123.247100830078 270.576446533203 133.527893066406 63.314136505127 88.1139602661133 161.299591064453 143.798355102539 141.541488647461 120.798057556152 137.161895751953 166.644882202148 125.072723388672 127.244567871094 201.718444824219 187.348052978516 120.32502746582 107.333335876465 151.859268188477 137.602890014648 132.394866943359 242.743392944336 119.680847167969 178.030578613281 161.278747558594 150.587844848633 115.514808654785 158.986968994141 134.02458190918 117.371849060059 99.8897323608398 179.102279663086 106.171188354492 104.030059814453 88.7842330932617 215.779159545898 154.380096435547 152.330047607422 102.609420776367 147.989944458008 196.417907714844 130.897750854492 247.888519287109 191.084976196289 150.446411132812

ENSG00000013275.6 119.682929992676 71.6082458496094 153.001556396484 164.868530273438 139.880996704102 75.0458984375 86.4886474609375 182.017028808594 146.116134643555 35.2970542907715 85.4233932495117 74.0287322998047 124.607887268066 159.170989990234 97.491096496582 261.791656494141 103.156074523926 114.382987976074 127.161460876465 151.765563964844 168.154678344727 361.606109619141 181.081344604492 104.226455688477 98.3640670776367 105.756164550781 183.820571899414 81.3632888793945 99.308708190918 136.668182373047 165.159088134766 61.7564735412598 135.570861816406 203.743423461914 139.898941040039 100.315170288086 62.8619956970215 70.776985168457 72.2784118652344 75.3373794555664 136.680465698242 102.572975158691 78.4266128540039 118.051879882812 137.999572753906 138.481857299805 126.888359069824 74.3833923339844 67.0399398803711 116.00316619873 100.786743164062 124.712799072266 129.026840209961 135.836959838867 100.10684967041 322.255676269531 137.520431518555 255.765914916992 64.7946395874023 146.343856811523 114.143531799316 173.508346557617 96.1975631713867 155.158554077148 116.361633300781 113.151435852051 106.656234741211 116.807922363281 99.65869140625 90.5057754516602 140.375106811523 105.474136352539 97.2204818725586 139.281265258789 90.8219223022461 214.696548461914 173.320159912109 139.790481567383 148.47135925293 159.880996704102 102.033508300781 228.285400390625 116.767036437988 116.816497802734 153.004821777344 81.5145721435547 115.086761474609 119.526214599609 203.613662719727 165.274505615234 105.301849365234 151.142105102539 195.598617553711 195.968200683594 276.758911132812 107.118225097656 120.364135742188 130.593032836914 88.3605346679688 58.9319038391113 132.588531494141 178.908401489258 154.996917724609 111.180252075195 109.202796936035 97.3333511352539 111.936065673828 121.616561889648 179.13916015625 103.734771728516 100.478538513184 120.334205627441 104.552642822266 130.836685180664 128.014770507812 92.7167129516602 122.535224914551 60.3364105224609 145.67448425293 94.0361557006836 114.000045776367 98.7572402954102 175.025115966797 104.214645385742 192.205093383789 160.188827514648 100.852592468262 142.758041381836 104.883491516113 111.431640625 200.762496948242 107.779426574707 114.530616760254 122.647872924805 102.36954498291 99.6240081787109 135.985122680664 79.2218704223633 70.1032180786133 86.3430862426758 96.6084442138672 92.5703659057617 186.924621582031 89.5201797485352 141.749984741211 319.304992675781 113.337844848633 53.4530258178711 96.7095413208008 72.6245422363281 200.704788208008 143.791748046875 170.277587890625 100.645713806152 110.196174621582 124.706954956055 124.454452514648 99.8081665039062 95.1009216308594 138.811569213867 112.225761413574 112.414260864258 182.464553833008 121.21834564209 153.801147460938 179.717086791992 113.450813293457 62.3422241210938 162.575149536133 132.750396728516 152.218826293945 96.4519195556641 70.0119705200195 158.768966674805 191.4521484375 101.98486328125 125.975532531738 85.1395492553711 94.8554306030273 109.538307189941 79.065315246582 138.833709716797 122.498634338379 87.994140625 85.3403854370117 179.89958190918 74.514274597168 101.482421875 245.843948364258 64.6610412597656 89.1399993896484 103.152328491211 124.450126647949 87.9255523681641 135.699127197266 191.80256652832 119.150451660156 123.300765991211 72.4039916992188 165.21272277832 78.5559768676758 153.4306640625 169.285766601562 75.138786315918 84.3115234375 149.562133789062 114.216621398926 88.4994049072266 79.201545715332 141.94157409668 114.248420715332 81.0522308349609 100.781021118164 140.803955078125 152.533081054688 43.3368377685547 70.3621139526367 162.844085693359 71.3842926025391 93.212287902832 103.165061950684 75.7993087768555 107.935241699219 664.595458984375 119.484840393066 225.064361572266 122.880485534668 73.8720550537109 166.641632080078 143.727310180664 137.639343261719 180.276153564453 395.963958740234 93.0586242675781 184.765014648438 102.8115234375 120.483818054199 93.7469100952148 133.01806640625 87.8428344726562 98.0666961669922 149.676040649414 155.431137084961 76.6369400024414 64.0944290161133 183.626663208008 84.1735000610352 147.635437011719 108.934814453125 65.6478652954102 174.600341796875 143.749786376953 96.4023361206055 104.326721191406 218.332321166992 122.85954284668

ENSG00000087191.11 170.59147644043 65.1556777954102 118.369354248047 75.5999450683594 116.39811706543 73.8371047973633 86.8280944824219 69.1841354370117 133.188430786133 53.295295715332 56.0379981994629 56.7891693115234 167.653366088867 106.313522338867 70.8176498413086 168.959228515625 94.1231384277344 88.6422653198242 70.3982391357422 82.0961761474609 113.481056213379 130.553115844727 113.038208007812 74.2739715576172 93.0105743408203 146.116409301758 85.7909317016602 48.240779876709 62.4464378356934 124.158790588379 95.2359848022461 53.7207221984863 121.931243896484 135.738693237305 47.8835945129395 96.1644515991211 37.8290367126465 59.1116256713867 54.5543098449707 56.7652473449707 89.1645202636719 66.6628112792969 41.3389549255371 91.1306076049805 115.410293579102 73.0407409667969 93.1787948608398 85.7182388305664 61.5983963012695 132.730346679688 83.3528518676758 116.361755371094 59.1693000793457 104.812400817871 89.1420211791992 125.430015563965 76.1295318603516 144.6416015625 115.326812744141 293.67578125 91.8795623779297 95.7223358154297 91.2446365356445 141.019653320312 75.8663711547852 81.5312728881836 81.4179000854492 128.758880615234 84.8286743164062 75.6319885253906 74.4243698120117 91.0952758789062 91.3921051025391 82.9586486816406 106.313423156738 139.857391357422 43.5093040466309 60.7619132995605 138.539916992188 116.123710632324 75.8941955566406 133.909057617188 100.125862121582 97.6193237304688 55.5776557922363 45.5807495117188 89.2205047607422 151.272033691406 92.5441970825195 135.380020141602 115.404502868652 94.2354202270508 134.681365966797 100.751220703125 117.297317504883 101.895706176758 76.844970703125 121.793235778809 57.652759552002 79.022819519043 82.2496337890625 116.024711608887 116.143455505371 85.4541702270508 149.264526367188 93.9625396728516 82.7818222045898 122.892677307129 106.41650390625 62.2102890014648 82.4007797241211 80.8879852294922 56.084888458252 87.4814071655273 97.4394454956055 75.140495300293 67.3317718505859 93.5725479125977 221.767837524414 110.072929382324 111.041305541992 84.8783493041992 167.549911499023 75.1678771972656 109.936950683594 80.5459747314453 73.1892623901367 196.401000976562 62.2571144104004 108.465621948242 112.300216674805 64.2386245727539 77.6644744873047 92.3601303100586 44.3496932983398 76.1808929443359 78.7134552001953 89.2355880737305 53.538215637207 100.047973632812 78.3443222045898 94.1853866577148 101.028511047363 59.3407859802246 113.505599975586 40.9919128417969 98.4925003051758 78.7063674926758 73.6787719726562 41.9875946044922 146.508758544922 86.3579406738281 93.3642196655273 104.953300476074 80.6725463867188 103.459686279297 104.918228149414 56.1893272399902 64.8844833374023 161.365921020508 70.1471710205078 75.2478179931641 108.657150268555 99.719108581543 119.824203491211 96.7170181274414 63.6626510620117 67.0562362670898 86.6923828125 106.446166992188 111.433601379395 65.1436157226562 46.6840858459473 144.647735595703 96.301399230957 94.0609741210938 126.859725952148 72.5367965698242 67.3513031005859 93.2644805908203 47.403434753418 70.1950759887695 131.41552734375 85.0831298828125 73.260383605957 137.440322875977 48.3907356262207 54.3899803161621 134.752243041992 71.7394790649414 67.522087097168 100.835052490234 63.9987144470215 95.6336364746094 92.5284042358398 182.054046630859 67.305290222168 123.594039916992 60.6884498596191 58.6388893127441 50.6962432861328 77.9602890014648 85.5247955322266 68.4180450439453 121.649459838867 85.37890625 89.0045394897461 81.7591552734375 72.8330459594727 47.4450531005859 86.8125762939453 61.7938270568848 69.176643371582 122.3681640625 95.5100631713867 31.6834907531738 60.5957412719727 100.064910888672 104.693923950195 70.7830276489258 89.9587020874023 69.9836807250977 120.413688659668 43.9919776916504 73.1101150512695 118.283760070801 132.562866210938 68.3952026367188 101.724983215332 98.8634796142578 104.151084899902 72.0499572753906 99.3938293457031 70.5196838378906 128.705673217773 94.8679580688477 119.273506164551 58.10595703125 83.2740097045898 99.5061340332031 86.2870101928711 71.1470565795898 90.7107543945312 80.6496887207031 73.0429153442383 118.923149108887 40.2185325622559 71.5746688842773 80.1131973266602 53.2625160217285 147.200012207031 141.193511962891 69.8881301879883 76.0741348266602 67.2430648803711 86.8295593261719

ENSG00000100519.10 50.9738693237305 18.1846981048584 36.7501525878906 80.5089569091797 31.6517562866211 17.2124710083008 30.2644424438477 36.2939338684082 20.2942943572998 13.3633632659912 13.7481756210327 19.5483589172363 61.0764389038086 33.963436126709 22.7005443572998 55.8899307250977 33.5899467468262 31.8521614074707 26.8146743774414 36.590705871582 24.1313858032227 44.8092765808105 55.6061592102051 27.8490371704102 64.1056976318359 19.4677581787109 24.9238395690918 26.296594619751 21.2609920501709 34.2465286254883 30.5367527008057 18.1650981903076 36.3817100524902 24.9879417419434 49.8924827575684 19.5613059997559 31.6346569061279 17.6196956634521 19.5376052856445 17.1958904266357 22.6335277557373 64.0746002197266 19.529541015625 22.0589370727539 29.4518203735352 65.0340118408203 30.4446983337402 65.9225616455078 13.9879207611084 24.0926609039307 44.5253028869629 32.6050148010254 30.2648277282715 23.9770965576172 33.3493309020996 38.4590339660645 51.912670135498 49.4051361083984 38.5683059692383 54.3040428161621 27.8399791717529 43.4681663513184 55.6133079528809 35.0046463012695 22.4950122833252 33.2826957702637 15.5625696182251 31.8808460235596 44.736442565918 25.9829750061035 34.9116439819336 33.1642227172852 22.4683036804199 20.102029800415 37.6763687133789 36.3991584777832 50.9044303894043 28.6190032958984 39.5589447021484 19.2986316680908 15.4489984512329 59.4254150390625 47.159107208252 31.2293357849121 62.1068878173828 31.728458404541 39.2718925476074 20.8806076049805 29.7856693267822 24.7187900543213 21.4728965759277 28.0301704406738 41.5945053100586 39.8416213989258 50.145450592041 27.4940013885498 26.745096206665 36.1523094177246 29.4944839477539 29.7800846099854 55.5652275085449 29.3442249298096 27.124828338623 25.6849403381348 33.767505645752 29.4176807403564 27.988941192627 43.612735748291 28.0756168365479 13.5948724746704 27.1823978424072 32.563289642334 28.5062980651855 43.3113479614258 31.5312614440918 16.1672039031982 48.8492202758789 33.1646347045898 33.6503562927246 23.52414894104 24.9102191925049 24.7418041229248 35.6597633361816 24.3357124328613 32.6946411132812 30.9542541503906 32.6700592041016 27.9635314941406 24.0369472503662 12.1386404037476 43.7590827941895 23.7063808441162 18.5522956848145 31.7797584533691 68.7715454101562 40.7674713134766 27.2990074157715 26.2807636260986 15.0702114105225 24.9461002349854 36.170467376709 22.0771923065186 45.2227058410645 18.1225433349609 38.0803146362305 16.9791240692139 49.4694290161133 25.5543041229248 21.8142776489258 26.2212333679199 35.6543846130371 61.195873260498 13.9216260910034 39.046947479248 30.1158351898193 30.4388217926025 39.0656242370605 20.3179779052734 20.8589363098145 36.0929565429688 37.9161415100098 19.7798366546631 28.9445037841797 18.7876434326172 26.8148612976074 26.095100402832 36.0987663269043 23.6428680419922 26.0439434051514 31.5639247894287 29.1026821136475 63.546142578125 36.7864418029785 20.8712997436523 26.5998020172119 19.9577045440674 24.8003959655762 26.3233489990234 29.2862014770508 35.3855667114258 20.9682006835938 35.9099235534668 18.9355316162109 30.9559631347656 18.7120513916016 62.8354721069336 22.8381004333496 53.8054580688477 33.8223838806152 25.0169410705566 30.3829326629639 35.3831748962402 31.7837009429932 23.1694297790527 28.2948246002197 28.8967380523682 27.3920154571533 31.1028881072998 21.8921756744385 36.5103912353516 29.6194324493408 52.7120399475098 34.6332855224609 13.5815305709839 17.0553512573242 31.8556003570557 33.9896087646484 42.8681030273438 18.0261726379395 17.7910747528076 31.5284996032715 19.4179229736328 26.0422611236572 30.9875812530518 22.2125854492188 11.138144493103 13.6169958114624 25.5443553924561 39.7709655761719 17.1495304107666 24.5289421081543 40.7028923034668 33.6464767456055 56.6966972351074 12.5188369750977 65.9326324462891 53.1022987365723 30.5699596405029 36.8293266296387 27.4507446289062 21.2149085998535 36.4872703552246 87.227897644043 22.6354885101318 42.7088584899902 29.7938480377197 32.1087417602539 20.5427913665771 25.0660953521729 45.8934173583984 31.0776519775391 42.5784187316895 35.3320426940918 18.1676750183105 18.4671154022217 18.46315574646 49.7785606384277 37.8864860534668 33.099494934082 21.762601852417 33.1993751525879 34.0259666442871 25.0382175445557 44.2964363098145 44.8812942504883 28.6645469665527

ENSG00000173692.11 66.9469375610352 57.5326805114746 52.5842742919922 49.7456359863281 63.0995826721191 55.9668273925781 52.1335868835449 69.1921157836914 43.563102722168 27.0415573120117 36.6124687194824 40.1702880859375 185.701858520508 60.3008766174316 57.6344032287598 130.869979858398 90.4877548217773 65.1279754638672 68.180061340332 63.9723892211914 146.591445922852 97.0821151733398 160.215698242188 83.3165130615234 50.5154991149902 64.800048828125 64.5702667236328 35.5654067993164 83.2042083740234 103.011650085449 67.9656143188477 44.2222175598145 75.1773986816406 61.7056121826172 26.8084373474121 57.3030967712402 24.8371849060059 55.3802261352539 36.6625137329102 29.7031955718994 82.8418655395508 60.086742401123 90.2829055786133 87.8560791015625 71.8429183959961 38.6552810668945 56.7611961364746 65.7570877075195 41.8551254272461 68.91357421875 63.4900245666504 76.651123046875 57.6227836608887 60.8088264465332 66.6999588012695 198.571640014648 90.4696731567383 183.10205078125 57.7351531982422 132.748687744141 51.9340591430664 48.4005813598633 31.1775512695312 81.3143615722656 50.2183990478516 54.6996078491211 62.4833869934082 90.972053527832 62.0580444335938 46.3147201538086 69.0260696411133 57.0202445983887 84.0912780761719 76.3482666015625 78.2492828369141 83.3586044311523 36.2048530578613 74.2143402099609 99.3603363037109 48.0082702636719 74.7518692016602 122.648078918457 94.8832473754883 67.7484664916992 58.6819534301758 185.212844848633 130.851455688477 58.4795341491699 65.5712661743164 62.7234230041504 77.8622817993164 183.736511230469 128.714889526367 132.756622314453 71.3407745361328 61.9955520629883 59.6767463684082 497.809997558594 91.7490081787109 67.8783950805664 32.7160606384277 55.955394744873 109.072891235352 66.7421875 41.8035049438477 52.0828170776367 57.2463836669922 58.0494003295898 54.8628463745117 38.1310958862305 59.1351585388184 63.8088874816895 33.0738258361816 94.1137771606445 61.0579223632812 39.4403953552246 53.560962677002 42.7280693054199 68.3772735595703 125.692153930664 97.4632873535156 30.9389667510986 98.9176177978516 56.3547096252441 89.6247100830078 67.8721160888672 76.8139343261719 64.1324081420898 61.4238586425781 27.6849613189697 104.756721496582 48.5842933654785 91.6842956542969 70.4807510375977 33.026065826416 53.5058364868164 91.3916625976562 60.1192398071289 41.5503158569336 39.405345916748 81.7171936035156 75.9951095581055 83.8939895629883 69.4363021850586 90.8753890991211 59.3307075500488 91.4799957275391 39.3616065979004 62.4674339294434 53.1238822937012 89.4224853515625 72.921257019043 42.7910003662109 60.2792129516602 32.1533584594727 53.3712615966797 94.866325378418 47.9894790649414 100.238563537598 51.0308265686035 92.2261123657227 45.9289741516113 97.5653686523438 73.9016723632812 53.4649887084961 53.9722480773926 62.0339126586914 39.7106781005859 74.8608551025391 97.647331237793 115.390350341797 21.1311187744141 31.2561016082764 69.9471130371094 46.5855255126953 37.5675201416016 102.006927490234 69.2353744506836 66.1907806396484 63.3214416503906 46.0549545288086 73.8685989379883 34.0502891540527 78.1940612792969 41.001766204834 75.5078887939453 60.446949005127 31.7618083953857 88.5496063232422 38.5413627624512 56.5433731079102 58.836669921875 58.7449378967285 47.2283248901367 93.5271453857422 98.0675964355469 53.6797370910645 110.109344482422 49.8025093078613 44.7402191162109 71.8400726318359 68.9218444824219 43.6783256530762 32.4033584594727 41.8760528564453 74.6280364990234 54.6973571777344 84.7384033203125 42.4022216796875 54.3347663879395 95.7363433837891 46.5752792358398 67.7362899780273 83.7166366577148 67.2169189453125 65.3992614746094 49.1810760498047 71.6117706298828 81.1533126831055 49.1277961730957 29.6657810211182 38.6790199279785 59.4545249938965 41.3392028808594 90.838493347168 112.316368103027 89.2591552734375 45.1029663085938 265.746307373047 101.420593261719 73.6593322753906 84.0259704589844 70.5758590698242 60.324836730957 154.320220947266 63.9528617858887 58.5402221679688 44.2670669555664 69.0436859130859 94.7880096435547 56.8751640319824 101.888610839844 95.7540435791016 42.8839225769043 36.0680618286133 103.371757507324 35.516300201416 59.5335464477539 57.1252326965332 51.9966430664062 78.2446594238281 86.1777725219727 75.5504684448242 140.494659423828 79.5397491455078 53.4221000671387

ENSG00000175166.15 278.072021484375 106.086715698242 156.108505249023 206.718856811523 264.336883544922 93.9761352539062 102.406967163086 161.390853881836 84.0067367553711 58.0011940002441 65.4002990722656 59.7667694091797 262.004302978516 103.02848815918 119.838645935059 339.881561279297 248.902191162109 120.378440856934 190.697402954102 119.817352294922 175.61962890625 258.047729492188 388.271697998047 170.328231811523 126.340324401855 202.270477294922 190.265441894531 81.1781311035156 104.381340026855 209.660446166992 246.989318847656 80.1344909667969 147.428314208984 110.547569274902 89.3396148681641 113.347412109375 73.4661560058594 102.225555419922 94.3716278076172 80.8069305419922 138.526870727539 72.7702331542969 220.374160766602 122.500328063965 181.422790527344 110.623840332031 116.963920593262 147.26692199707 109.836044311523 87.5190734863281 105.054298400879 145.68229675293 98.7104187011719 214.537841796875 113.478675842285 304.223449707031 121.30460357666 309.092315673828 120.811767578125 280.524841308594 119.626655578613 69.5484313964844 104.309196472168 255.030822753906 104.95792388916 76.4594192504883 133.930618286133 164.142196655273 118.474960327148 73.0010070800781 137.276168823242 130.154876708984 102.035346984863 137.306396484375 166.281982421875 131.273498535156 67.3607177734375 112.655647277832 275.125640869141 216.826919555664 130.236038208008 251.964736938477 171.538696289062 172.881088256836 127.351493835449 81.73779296875 228.186050415039 85.1083984375 253.661636352539 166.507659912109 96.5684814453125 324.500854492188 426.286895751953 240.446884155273 214.628036499023 123.71150970459 123.050437927246 110.281860351562 130.72590637207 110.439506530762 88.2367553710938 91.9206390380859 117.539176940918 56.8884315490723 118.531021118164 108.153785705566 132.155364990234 120.853630065918 90.4356994628906 140.704040527344 139.801956176758 171.351303100586 48.9761390686035 210.786254882812 87.1387405395508 61.3747520446777 136.15364074707 55.9560775756836 104.501853942871 250.04850769043 134.999862670898 106.334762573242 274.691009521484 137.954971313477 137.930191040039 162.578506469727 116.519088745117 96.7839508056641 141.102584838867 68.3686065673828 263.174377441406 100.319198608398 141.576248168945 112.251953125 73.595100402832 78.3530502319336 229.215454101562 96.8271179199219 97.4117126464844 71.6998138427734 130.565490722656 109.680252075195 254.370208740234 116.554222106934 372.298370361328 132.096740722656 161.563278198242 79.7744979858398 149.945892333984 90.7404861450195 227.315856933594 93.3392105102539 94.100715637207 125.454528808594 66.0454025268555 100.365997314453 188.405776977539 72.4882354736328 112.309776306152 61.1819381713867 81.5321197509766 107.254470825195 193.304718017578 99.4166641235352 71.4057312011719 155.332168579102 156.427749633789 74.8148574829102 158.862197875977 154.803985595703 148.493881225586 76.7060394287109 80.5233612060547 191.154571533203 107.810302734375 71.6554946899414 152.608306884766 156.887069702148 106.067993164062 175.978546142578 54.1074447631836 201.977905273438 122.377197265625 124.841850280762 78.9466552734375 149.124572753906 117.106872558594 53.5521697998047 187.705535888672 70.7869033813477 94.943733215332 94.4877624511719 172.281997680664 102.086532592773 87.5249328613281 181.513473510742 93.2931442260742 177.838027954102 84.9437484741211 228.113235473633 250.885803222656 100.261009216309 110.262428283691 39.7925262451172 142.578979492188 101.787231445312 156.107116699219 108.509353637695 74.3718414306641 86.4861221313477 181.737182617188 92.5399703979492 118.201446533203 171.37548828125 116.629776000977 73.7554931640625 83.1917495727539 126.859329223633 130.132904052734 92.2035903930664 74.0235900878906 81.2126312255859 156.070297241211 97.9406967163086 150.491485595703 278.588958740234 115.462898254395 121.602348327637 98.5584945678711 183.155502319336 137.160522460938 203.534378051758 229.862701416016 96.6055679321289 264.260314941406 170.39875793457 101.227348327637 74.9346084594727 115.095970153809 151.332763671875 109.437957763672 132.694473266602 150.912139892578 85.321044921875 64.6817779541016 196.876495361328 88.4177703857422 108.541809082031 187.366653442383 64.7558059692383 124.17121887207 177.636596679688 126.825996398926 220.740127563477 270.643707275391 79.5127410888672

ENSG00000108344.13 222.929992675781 54.177001953125 103.970840454102 130.739624023438 137.96842956543 60.4628486633301 88.8160858154297 157.446487426758 48.5622253417969 33.8500137329102 55.7424278259277 70.6202392578125 234.629302978516 88.9813537597656 69.4636077880859 138.888641357422 154.315521240234 85.3822860717773 99.8732223510742 87.9025344848633 140.286392211914 248.547210693359 138.981292724609 133.560256958008 80.428840637207 179.679260253906 133.356719970703 71.7294616699219 90.8827438354492 125.631706237793 147.603103637695 47.8241806030273 113.762634277344 60.4768600463867 76.7479629516602 104.919494628906 65.2526702880859 62.6337089538574 62.5997009277344 43.3288345336914 140.239456176758 79.4667739868164 347.084930419922 111.508262634277 124.234184265137 78.7640228271484 136.001693725586 143.125946044922 62.1294403076172 129.307922363281 95.1664962768555 91.2940902709961 90.9476165771484 65.4527130126953 74.4889984130859 118.965026855469 142.458251953125 128.820617675781 102.161827087402 163.949661254883 87.3911972045898 84.7579498291016 66.8573532104492 160.413558959961 66.4487838745117 80.7765502929688 89.6706771850586 138.018676757812 188.525955200195 81.0255355834961 127.432563781738 69.9250411987305 87.2861404418945 104.216903686523 110.704856872559 164.672256469727 63.3801231384277 80.6327896118164 181.339920043945 127.978996276855 91.2114105224609 162.875366210938 115.014297485352 99.4719009399414 127.093063354492 52.1487350463867 127.488746643066 79.6111526489258 90.6944351196289 112.3583984375 65.4572372436523 120.339286804199 198.862442016602 182.908615112305 117.239700317383 142.313613891602 104.922859191895 179.385162353516 107.379806518555 50.6108589172363 76.6259613037109 77.893180847168 125.942611694336 72.4269027709961 88.9113235473633 71.4579086303711 71.5516357421875 76.484016418457 107.446395874023 68.3464660644531 75.5330505371094 125.32780456543 42.8463592529297 140.429077148438 132.20280456543 58.1005058288574 81.6336441040039 89.0577926635742 90.8391342163086 69.4520568847656 130.704986572266 91.3335037231445 147.163558959961 84.6119689941406 171.699188232422 82.4289016723633 91.5407943725586 102.647758483887 82.7119140625 77.5330200195312 127.197723388672 57.1151313781738 78.5180282592773 96.5053100585938 57.2720565795898 79.6410598754883 107.822074890137 81.1517868041992 63.7355461120605 73.6223297119141 68.8427429199219 85.8675155639648 104.669296264648 70.2293395996094 138.355285644531 59.8280334472656 143.332153320312 76.6342468261719 96.7038955688477 67.1196823120117 278.458465576172 115.721961975098 91.9872360229492 100.997268676758 61.4028701782227 94.5815124511719 98.9353103637695 54.2170219421387 73.4093856811523 161.666000366211 93.4721298217773 62.3085594177246 99.1124572753906 111.03881072998 77.8450546264648 95.1578216552734 97.5435943603516 53.6145324707031 146.653869628906 104.543167114258 185.756973266602 90.8498916625977 71.335205078125 72.7368545532227 96.0037231445312 84.6715927124023 144.540298461914 94.1512985229492 56.5701370239258 85.3036651611328 52.9403915405273 93.5248260498047 190.294036865234 135.596389770508 52.2228317260742 128.803466796875 62.3647994995117 52.4887886047363 170.489730834961 49.3290023803711 67.1372833251953 128.327270507812 100.267845153809 94.1477203369141 101.192565917969 114.989120483398 87.6511764526367 79.7529907226562 62.6601829528809 143.652114868164 47.9065589904785 74.3228302001953 91.6386413574219 36.8564910888672 89.3127899169922 83.6583786010742 97.8473968505859 88.0922317504883 62.7967796325684 68.0297393798828 100.27099609375 65.688117980957 78.6118087768555 111.253120422363 110.486442565918 40.174430847168 63.676830291748 78.0480270385742 131.470138549805 53.5772438049316 57.9027976989746 77.2263259887695 71.2727355957031 105.545761108398 75.3389053344727 117.598655700684 61.0567359924316 60.6656227111816 141.998748779297 103.197830200195 108.061965942383 111.359916687012 127.938835144043 73.6513824462891 120.39249420166 96.7235946655273 178.416702270508 52.1094284057617 59.6448516845703 73.7350158691406 97.2945861816406 66.0150833129883 94.8157272338867 70.1489791870117 53.6179542541504 97.786506652832 75.0288543701172 69.217170715332 153.724212646484 64.9938201904297 131.815322875977 111.754989624023 95.8835144042969 75.9520568847656 94.0976181030273 90.8656768798828

ENSG00000159352.14 178.858337402344 89.0877914428711 224.699340820312 111.021690368652 215.535430908203 120.603248596191 142.265640258789 236.045043945312 156.986679077148 86.49462890625 141.619491577148 117.89656829834 234.579788208008 341.015991210938 160.352966308594 226.949127197266 272.379364013672 111.601844787598 159.515563964844 231.166717529297 249.575012207031 577.094970703125 189.907241821289 295.337188720703 168.662521362305 211.128509521484 176.905487060547 114.809555053711 106.180953979492 131.216644287109 156.967559814453 78.4807510375977 170.364883422852 161.398254394531 281.908660888672 102.439315795898 123.541229248047 168.793411254883 110.845741271973 86.8991546630859 276.251068115234 136.914978027344 114.472923278809 177.842788696289 168.14729309082 158.753601074219 134.275268554688 288.212188720703 98.1548385620117 233.797317504883 143.719924926758 167.058303833008 219.23779296875 178.141403198242 181.993728637695 256.493194580078 210.754684448242 218.675262451172 220.850051879883 315.525177001953 185.845230102539 149.649307250977 180.109100341797 214.362564086914 142.006149291992 183.202911376953 142.663681030273 152.588577270508 213.940612792969 108.98868560791 164.823165893555 94.818000793457 174.066665649414 155.336883544922 187.591690063477 169.372116088867 119.55158996582 116.059188842773 344.388366699219 387.702026367188 116.628608703613 217.964935302734 421.037628173828 124.605445861816 155.835632324219 136.21630859375 178.62825012207 407.253723144531 288.657409667969 225.810760498047 302.647827148438 252.808486938477 278.588500976562 202.943939208984 118.430786132812 267.545074462891 86.9974060058594 150.429275512695 166.450500488281 233.440673828125 180.074447631836 213.425094604492 201.011489868164 163.940460205078 190.0986328125 127.537643432617 188.835052490234 217.44514465332 353.757568359375 172.505416870117 144.553283691406 179.627517700195 223.409515380859 163.599990844727 134.691787719727 204.684219360352 112.296882629395 136.754440307617 141.161727905273 136.522430419922 249.767715454102 319.0078125 203.705017089844 155.256927490234 224.53125 215.824935913086 188.674575805664 159.469711303711 168.878173828125 176.92366027832 164.736938476562 125.148139953613 177.834579467773 299.604461669922 88.8605117797852 166.257461547852 163.319229125977 207.134231567383 107.499908447266 161.757431030273 122.77124786377 183.815277099609 248.36149597168 181.564392089844 162.447570800781 91.6976852416992 263.859313964844 193.202667236328 121.43482208252 98.6006774902344 281.214721679688 241.101501464844 107.112915039062 206.375030517578 148.628707885742 131.02214050293 214.238952636719 131.128784179688 113.583015441895 280.012115478516 216.078979492188 173.552322387695 143.46321105957 158.168533325195 185.278778076172 209.575164794922 101.568481445312 82.931999206543 403.855224609375 137.244995117188 196.434234619141 245.130325317383 94.3271255493164 253.539016723633 231.249252319336 163.165954589844 199.273361206055 124.09928894043 229.801406860352 122.807746887207 133.805603027344 111.432907104492 257.926696777344 209.681747436523 87.3181228637695 174.830261230469 124.243522644043 164.675338745117 242.249282836914 83.8427734375 140.659545898438 190.754287719727 176.729339599609 114.081199645996 196.088684082031 275.224151611328 114.515365600586 122.037101745605 156.656951904297 139.681091308594 101.927841186523 193.695617675781 158.711990356445 196.441970825195 175.038299560547 159.142730712891 99.9931488037109 111.054618835449 145.531173706055 174.822006225586 167.4208984375 113.515518188477 113.842636108398 205.676040649414 135.671127319336 58.4267082214355 199.205276489258 165.602996826172 186.969390869141 108.825225830078 174.389785766602 203.612762451172 158.390075683594 146.794448852539 145.24201965332 339.564208984375 122.505447387695 85.3422164916992 158.278427124023 150.612289428711 217.547225952148 256.397857666016 251.769409179688 124.974098205566 283.412628173828 171.47998046875 218.604873657227 84.2145080566406 133.994110107422 150.798980712891 158.141647338867 220.709579467773 143.292526245117 100.36149597168 106.621284484863 249.834503173828 175.507873535156 178.023864746094 161.613723754883 115.996192932129 153.484375 146.264419555664 118.840843200684 233.923080444336 137.723770141602 155.584045410156

ENSG00000095261.12 20.0583763122559 22.7757778167725 39.6365089416504 1.92727971076965 72.7474136352539 26.656400680542 57.0116539001465 32.9245834350586 25.9140872955322 14.2779636383057 17.3229236602783 17.5836658477783 27.0364627838135 40.1049499511719 36.8919067382812 50.0364799499512 38.7584190368652 26.9042568206787 27.0802440643311 29.9919910430908 17.0829467773438 34.8913688659668 76.432014465332 25.5403308868408 31.498649597168 29.555850982666 31.5651073455811 19.6091690063477 26.513111114502 45.2358360290527 61.598445892334 20.9982833862305 26.7490997314453 34.1390838623047 28.8381252288818 18.2618980407715 24.7890701293945 49.4822196960449 36.6943550109863 14.0659399032593 27.6242523193359 18.5508556365967 26.0707683563232 18.51780128479 19.7034759521484 45.0153312683105 31.125825881958 42.8279342651367 17.9567451477051 36.0789070129395 23.8532180786133 29.6935749053955 17.9096412658691 23.3442115783691 32.8808441162109 42.0729179382324 32.8435325622559 57.2011413574219 28.5988540649414 51.3910789489746 40.3365669250488 32.1990623474121 28.6882228851318 30.3105564117432 30.9505252838135 33.096981048584 30.2124691009521 27.0647640228271 26.1446094512939 43.3692245483398 61.3162460327148 33.3948211669922 37.5691184997559 27.7227592468262 40.4443550109863 37.1232490539551 23.6567535400391 23.5899028778076 44.3788185119629 31.5341701507568 33.7325477600098 55.3772888183594 56.2998008728027 21.1977653503418 19.3746681213379 33.9755859375 50.8087577819824 14.600209236145 41.628044128418 28.8235168457031 30.9044208526611 52.7879600524902 24.2477359771729 47.2248954772949 29.9225425720215 27.6777076721191 25.9039306640625 28.189826965332 39.2980346679688 30.1398143768311 25.9407062530518 38.7155342102051 58.4868278503418 38.5494842529297 36.3912887573242 31.4920406341553 20.8960113525391 27.4107646942139 21.1050472259521 64.8429336547852 39.7607460021973 45.0721664428711 17.6658306121826 7.80305194854736 30.3921928405762 19.6576271057129 32.5397300720215 25.5516910552979 24.0961627960205 35.8027038574219 32.375301361084 17.8864097595215 51.3203392028809 33.162483215332 44.1245040893555 33.7810211181641 40.4546966552734 32.2546195983887 36.9085731506348 17.3847522735596 35.4141235351562 25.8647556304932 44.9263763427734 23.6787948608398 38.4114990234375 19.7303199768066 85.5279159545898 35.1419410705566 35.5025291442871 31.6812725067139 50.4406929016113 20.4546546936035 45.5371475219727 37.8871421813965 41.9594573974609 18.1964263916016 34.7733039855957 43.3128051757812 31.0552444458008 22.5300045013428 29.0752716064453 38.7547225952148 22.1297073364258 51.6223297119141 30.6108779907227 18.7238883972168 25.500186920166 30.1927070617676 34.8043937683105 22.5296669006348 35.6932907104492 16.5611324310303 30.9286670684814 25.5120506286621 34.9342079162598 31.8497180938721 33.796329498291 27.059383392334 24.4546337127686 23.1120204925537 40.7699356079102 25.9736843109131 30.1198253631592 33.1286506652832 44.9780197143555 18.9137706756592 33.6259994506836 34.0911712646484 28.9774398803711 27.5125293731689 27.7537097930908 34.59716796875 24.4725456237793 47.974681854248 19.3136367797852 24.1911926269531 34.0320358276367 26.6607761383057 41.7703971862793 23.6830768585205 30.7700481414795 36.506217956543 46.7647552490234 29.2705345153809 45.793212890625 36.560302734375 34.1395378112793 53.8945579528809 29.1277198791504 21.5254306793213 22.2183475494385 23.6100463867188 32.8730735778809 25.1033592224121 24.5811538696289 36.1130599975586 41.0341300964355 33.2675971984863 24.990608215332 40.1418075561523 42.3560447692871 14.861759185791 24.5957698822021 39.0800476074219 26.317684173584 27.8778114318848 39.0426025390625 48.7001075744629 24.9008140563965 15.7616167068481 25.0916652679443 13.4108896255493 31.4641513824463 23.9947261810303 40.4649200439453 24.0458526611328 24.2730236053467 59.3682708740234 32.7825813293457 29.4150714874268 50.2856330871582 46.1035804748535 46.8520202636719 21.888988494873 24.8554039001465 44.6824684143066 33.6134071350098 19.026086807251 27.2868041992188 58.2379722595215 23.2268886566162 47.5638580322266 26.1260738372803 26.4246845245361 19.7756671905518 25.457914352417 33.3294143676758 28.5659599304199 27.759672164917 26.7466125488281 36.9803619384766 33.897876739502 41.4261703491211 34.8110046386719 31.9756946563721 29.8141822814941

ENSG00000103035.9 133.544357299805 62.0702209472656 187.986618041992 115.896202087402 129.315231323242 53.300838470459 61.3931465148926 66.279914855957 47.2508735656738 33.5332260131836 52.654899597168 55.5895500183105 174.187072753906 72.4614105224609 45.9858856201172 133.23551940918 81.5921249389648 71.9212875366211 107.499496459961 41.7996253967285 48.4407844543457 90.7379531860352 81.5245208740234 82.3991241455078 67.433349609375 78.2365112304688 88.6763153076172 36.5228424072266 122.111946105957 89.7930145263672 59.3373336791992 51.0806770324707 75.4325485229492 104.979804992676 68.2063293457031 39.807861328125 66.6551666259766 56.6149406433105 49.3030319213867 49.9624938964844 69.3056945800781 104.380889892578 78.6676330566406 67.6447830200195 51.1944923400879 56.9041976928711 65.9439315795898 90.0864181518555 45.2857780456543 72.4558868408203 59.9753456115723 83.8124618530273 55.0140113830566 74.4454803466797 88.3577499389648 121.183601379395 68.0953063964844 141.360870361328 62.3209419250488 104.430946350098 38.9427871704102 37.1889038085938 56.487979888916 86.8470153808594 61.2425575256348 74.5983657836914 89.7904205322266 92.5122528076172 67.1070938110352 65.0782928466797 91.7763214111328 65.675666809082 68.3860321044922 167.618225097656 60.2714576721191 94.156364440918 97.2011489868164 93.784049987793 81.6541366577148 68.8314056396484 68.9965515136719 96.0107879638672 98.6689300537109 76.4199829101562 90.2532348632812 94.715690612793 123.202842712402 71.0117492675781 61.9493789672852 95.3818283081055 68.7679138183594 103.275024414062 112.736854553223 123.283599853516 70.4918823242188 177.355575561523 80.6869964599609 128.394607543945 87.3847961425781 89.1226501464844 65.0294570922852 84.3197402954102 87.1077423095703 56.2346878051758 24.6857013702393 59.5775833129883 57.8421630859375 68.209342956543 66.7806091308594 71.7981948852539 61.7439346313477 69.1820297241211 72.402458190918 82.8313827514648 53.7678604125977 60.4721717834473 44.5501289367676 43.8563652038574 68.8473663330078 82.579833984375 81.0251007080078 38.5211219787598 160.800262451172 53.2517852783203 69.525276184082 48.3826484680176 85.6075592041016 57.1046943664551 79.5901870727539 21.898401260376 127.933975219727 64.5128173828125 52.706428527832 103.179565429688 72.7990646362305 85.7875900268555 61.8580169677734 58.876708984375 65.189453125 58.5986824035645 73.5784683227539 50.0966873168945 74.8074111938477 91.9583892822266 111.950088500977 43.9573669433594 128.647445678711 29.2801990509033 56.4950637817383 68.4049606323242 97.9567642211914 96.7729034423828 54.1980476379395 91.5403442382812 53.2297592163086 44.197868347168 116.277297973633 51.7669715881348 75.6463165283203 48.3023529052734 100.416534423828 47.1384544372559 76.2870254516602 45.4074440002441 74.9830932617188 74.3886184692383 71.0442504882812 48.3596954345703 90.5427093505859 155.29670715332 101.385185241699 109.625434875488 55.9342956542969 96.7410278320312 35.7047119140625 66.1932983398438 65.0001220703125 88.126091003418 56.1407661437988 72.7948837280273 65.8343734741211 105.371894836426 70.6886138916016 75.6850051879883 43.0815734863281 93.9928512573242 80.0649032592773 58.961669921875 110.625801086426 46.8593215942383 64.2809295654297 103.961456298828 81.1246948242188 75.4870681762695 90.6122741699219 95.1220474243164 52.6144180297852 98.9139022827148 52.4016265869141 77.2451324462891 61.7265663146973 68.6382064819336 61.2827682495117 20.3964042663574 65.5270233154297 98.1923751831055 69.5618591308594 90.2264633178711 57.427173614502 46.7938537597656 78.6189651489258 56.2500686645508 72.2463912963867 83.2183837890625 63.1726150512695 51.2142219543457 79.8293228149414 66.8756713867188 93.8951110839844 63.274543762207 49.448413848877 55.1819114685059 80.0978775024414 60.7796096801758 98.5295639038086 116.731452941895 64.5387420654297 58.2637481689453 69.4282302856445 88.4772033691406 85.3258285522461 90.961540222168 162.18620300293 43.4133644104004 162.218490600586 60.5471229553223 75.9309387207031 55.2327003479004 65.7136535644531 64.327522277832 80.0011215209961 69.1866455078125 97.8847351074219 43.6257553100586 44.8765869140625 48.2050476074219 99.3608093261719 87.3987808227539 116.731132507324 58.9143714904785 91.9745330810547 50.635669708252 76.8258438110352 150.495239257812 90.906120300293 61.6009101867676

ENSG00000099341.10 162.877593994141 100.484153747559 183.963577270508 332.979736328125 102.498764038086 85.8327560424805 130.193588256836 213.759353637695 106.550094604492 56.4192390441895 94.1573638916016 105.54736328125 95.4258346557617 144.582153320312 143.203567504883 290.524993896484 173.909576416016 164.205001831055 136.591201782227 138.024002075195 137.553756713867 244.183120727539 164.746520996094 145.123062133789 138.221893310547 117.373840332031 188.887878417969 183.760025024414 131.163909912109 162.837783813477 182.535079956055 93.3605880737305 134.114608764648 241.415191650391 295.306274414062 105.534225463867 146.591812133789 78.5011978149414 103.986145019531 83.9742431640625 168.366973876953 189.366226196289 81.6055068969727 205.182006835938 117.43025970459 270.322479248047 144.220336914062 74.5111541748047 90.6731491088867 176.605667114258 105.701904296875 128.446548461914 113.760154724121 169.41716003418 135.215698242188 273.954559326172 124.403366088867 265.602478027344 143.703323364258 136.69677734375 126.508178710938 270.088256835938 139.690277099609 170.383880615234 116.817886352539 112.309997558594 141.359848022461 118.081764221191 116.835144042969 131.481384277344 208.640991210938 126.38516998291 130.643890380859 158.917938232422 101.224235534668 305.143615722656 270.035003662109 149.380554199219 184.701873779297 143.022125244141 161.101577758789 204.830932617188 108.425872802734 117.679161071777 202.400207519531 100.744972229004 151.605209350586 88.0110397338867 141.105117797852 133.790466308594 193.288955688477 151.473419189453 182.99983215332 148.928192138672 305.096954345703 130.388580322266 142.593246459961 128.421661376953 102.631774902344 58.7957153320312 184.363876342773 154.742965698242 151.613220214844 103.606941223145 91.4974822998047 120.09635925293 108.33821105957 159.271102905273 159.226531982422 123.89323425293 138.877014160156 112.197898864746 201.222030639648 156.36735534668 121.508087158203 88.0604095458984 160.968322753906 58.5256958007812 85.7541809082031 331.648620605469 162.612747192383 91.6704940795898 173.368255615234 140.183288574219 192.454864501953 136.81298828125 117.47533416748 119.225379943848 205.40998840332 120.188804626465 161.443710327148 162.014999389648 116.600364685059 140.902938842773 237.372985839844 104.015205383301 136.285888671875 109.802810668945 93.7709579467773 114.690216064453 135.551605224609 135.355316162109 155.953109741211 90.2168502807617 212.868392944336 255.173797607422 116.494552612305 54.0844764709473 100.576805114746 116.849403381348 141.544448852539 194.251663208008 154.85661315918 79.9184112548828 84.319694519043 125.031898498535 108.135780334473 129.542892456055 144.948394775391 151.744522094727 132.306777954102 149.108627319336 191.699737548828 121.36988067627 201.915298461914 219.244583129883 184.349975585938 86.7001342773438 192.953338623047 130.596817016602 159.173568725586 135.294967651367 171.870712280273 149.160736083984 336.891510009766 105.326507568359 127.515571594238 98.1675186157227 115.40869140625 126.603485107422 83.0963897705078 139.860855102539 200.563629150391 131.993591308594 95.0006103515625 185.299377441406 96.6349334716797 224.48779296875 199.742324829102 78.3583068847656 111.26741027832 93.7738494873047 170.151260375977 131.667266845703 234.952239990234 267.812957763672 173.153823852539 124.112655639648 104.457214355469 135.367782592773 104.099113464355 186.304382324219 164.444625854492 67.523811340332 76.2712707519531 153.939041137695 183.502182006836 103.442451477051 88.9047775268555 117.223754882812 148.527938842773 108.190200805664 116.586296081543 128.512771606445 163.244583129883 88.6908569335938 101.890998840332 185.597595214844 73.2863006591797 108.560188293457 119.176300048828 134.974838256836 193.515777587891 908.314208984375 150.770950317383 218.440444946289 113.624435424805 110.588371276855 160.610244750977 142.303802490234 170.107833862305 181.219100952148 431.573760986328 96.9873962402344 197.422317504883 92.7798843383789 133.389739990234 120.450630187988 122.192649841309 99.164192199707 98.8778991699219 282.144958496094 134.653915405273 100.732048034668 122.145324707031 134.396240234375 207.687438964844 119.339019775391 199.23583984375 107.995124816895 156.356460571289 179.135360717773 110.88614654541 102.250938415527 150.767440795898 103.365409851074

ENSG00000101843.17 77.4375152587891 40.4530296325684 111.547981262207 113.979118347168 96.6376342773438 46.4077568054199 58.5315856933594 86.3736267089844 50.2841148376465 30.3864879608154 32.8724822998047 33.9080543518066 104.011100769043 46.6178550720215 46.4679069519043 76.496452331543 57.0121040344238 48.5896148681641 75.6643142700195 60.8957939147949 46.6674537658691 110.038841247559 65.4360656738281 33.8127784729004 39.1514930725098 58.7443389892578 68.4280014038086 60.0338935852051 47.841381072998 61.2745933532715 113.674201965332 46.7398567199707 69.9977569580078 78.0217361450195 112.036399841309 50.4312133789062 146.879425048828 44.6046257019043 59.3260078430176 43.5495719909668 109.268852233887 110.028968811035 69.7839202880859 47.9716110229492 70.4245681762695 68.8353118896484 40.7330284118652 51.5804290771484 38.3153839111328 53.9687347412109 56.1545295715332 57.1345901489258 58.1440010070801 42.3834495544434 75.9564361572266 67.1316452026367 58.8754806518555 52.9666442871094 40.3529815673828 107.852516174316 51.6447143554688 99.9520874023438 87.6636810302734 94.0190353393555 51.1921119689941 50.1857109069824 64.324462890625 78.3456954956055 54.7385940551758 63.9363784790039 67.9583129882812 79.6259613037109 50.1072158813477 77.925163269043 80.2788925170898 72.9785766601562 92.9001235961914 38.6808319091797 105.505630493164 36.4252090454102 43.5802192687988 91.3168106079102 106.691665649414 56.3455924987793 63.3212776184082 66.0818405151367 67.4483871459961 35.3975219726562 76.7271575927734 49.3032608032227 59.7278709411621 67.2265472412109 67.1994094848633 70.9804229736328 63.4501991271973 64.5165557861328 51.0669631958008 79.4466552734375 55.2233848571777 59.1361618041992 87.9350204467773 51.7631759643555 61.5936126708984 40.4584045410156 60.5889472961426 67.6225891113281 69.8775634765625 60.2531852722168 42.8573989868164 41.0301818847656 122.003715515137 62.0566253662109 49.2561340332031 97.46484375 42.7202033996582 36.9459800720215 66.9024658203125 30.2430686950684 25.7218170166016 47.9268836975098 59.5465850830078 43.8614692687988 156.714965820312 63.3729438781738 63.936466217041 65.4793090820312 69.0065002441406 36.5171890258789 65.7041702270508 31.6060276031494 107.688232421875 55.0227394104004 69.3136138916016 80.3736190795898 82.2220306396484 43.9405555725098 69.0861282348633 46.2451477050781 47.4030647277832 50.3454704284668 57.6329345703125 55.5205764770508 84.7292098999023 38.973217010498 75.3871841430664 43.9707565307617 75.7056503295898 38.3537788391113 48.3214302062988 36.3778991699219 88.9441223144531 83.062629699707 32.3793869018555 53.2343788146973 41.9443588256836 48.2834777832031 38.0815391540527 46.6357498168945 51.3206024169922 44.4783973693848 62.5113945007324 33.7364196777344 48.3427848815918 78.4731750488281 46.79833984375 53.2745590209961 49.7497444152832 39.1085929870605 55.5156059265137 60.0155754089355 106.89909362793 212.689880371094 53.3335990905762 53.2372055053711 111.660140991211 31.1400146484375 54.464900970459 60.2203140258789 50.3460388183594 74.6908874511719 51.5530128479004 37.4959907531738 24.3404655456543 72.513557434082 47.3610420227051 51.9448509216309 54.0417938232422 136.555557250977 65.053337097168 36.6745376586914 45.2069396972656 50.1747932434082 82.6294326782227 43.7164039611816 36.0942535400391 62.851245880127 59.7161865234375 72.7087326049805 40.9772453308105 52.1556358337402 37.0996856689453 43.2856521606445 57.1828269958496 29.131046295166 48.6492042541504 67.8849182128906 65.162956237793 107.558876037598 40.6256866455078 68.9818115234375 48.1850624084473 51.4802322387695 69.1308822631836 90.3171539306641 47.7170181274414 32.1401824951172 36.331600189209 55.8883666992188 73.5285415649414 37.0823135375977 35.8144035339355 63.2430801391602 76.8952560424805 103.754852294922 39.4010772705078 89.7040328979492 66.1748657226562 59.2558822631836 76.290412902832 61.812198638916 57.938835144043 85.2121658325195 139.169662475586 38.4312515258789 72.3831558227539 52.8484344482422 52.2923622131348 44.6952857971191 41.7358360290527 84.8269500732422 67.8772125244141 53.5521659851074 66.0564498901367 55.7868118286133 36.5863304138184 58.2677574157715 176.132858276367 40.8147239685059 87.2194900512695 43.7645072937012 91.0195617675781 114.069450378418 54.7373199462891 74.0772247314453 61.2956695556641 40.1355438232422

ENSG00000108671.8 102.404251098633 28.8382167816162 75.4225463867188 80.8570098876953 103.118461608887 33.3386840820312 38.9391708374023 56.7382164001465 24.2159671783447 12.2316045761108 23.7880706787109 30.0085010528564 83.5117721557617 52.5160102844238 38.6165771484375 115.368423461914 82.668571472168 36.2224235534668 48.039794921875 57.521598815918 49.6796569824219 138.548980712891 202.390289306641 58.599925994873 45.8016586303711 83.9206390380859 60.4491310119629 25.0362415313721 51.1306266784668 82.0514297485352 51.3944625854492 25.8512191772461 61.5525131225586 57.5397033691406 23.2726802825928 46.283561706543 17.9279327392578 45.3407440185547 31.9531784057617 20.9817562103271 74.4289016723633 52.0662803649902 38.4125938415527 62.4694366455078 40.9211463928223 37.1023368835449 53.7091789245605 69.9990844726562 29.7014865875244 65.9865646362305 51.5503349304199 53.6743087768555 40.8980102539062 49.4764518737793 44.4674873352051 192.971649169922 99.6549835205078 169.643341064453 37.898063659668 92.3072967529297 45.4995079040527 45.2481956481934 33.5377349853516 93.6288146972656 39.1050338745117 43.2261161804199 54.6592788696289 67.6143264770508 63.8989143371582 38.4615516662598 61.124195098877 39.4847297668457 48.9927062988281 60.3617858886719 72.9592056274414 88.1153182983398 27.1657066345215 45.3133583068848 75.7908554077148 62.7507286071777 48.396297454834 108.22297668457 82.7447357177734 62.1444206237793 40.6089172363281 37.7904357910156 97.3025207519531 38.7788467407227 53.3988265991211 74.3992004394531 72.1474761962891 86.4016952514648 123.572143554688 115.282905578613 70.145378112793 72.0517501831055 55.1734809875488 106.934997558594 54.2682838439941 27.825309753418 32.355224609375 34.418342590332 70.212646484375 42.2690467834473 57.7909889221191 40.6067810058594 39.907398223877 43.3202476501465 63.5272789001465 30.0522556304932 40.6722450256348 67.0466003417969 34.9708099365234 71.9194869995117 58.3605461120605 38.3414993286133 63.502555847168 60.0914039611816 68.5931549072266 45.2414283752441 54.464656829834 57.1800384521484 74.945182800293 43.6061973571777 96.6243591308594 53.7817535400391 57.7621307373047 49.6223678588867 43.8199081420898 38.4522361755371 73.1479797363281 26.0480327606201 50.4749374389648 58.8120918273926 33.1910438537598 42.4521636962891 53.9765815734863 43.5295219421387 43.1885414123535 27.3267784118652 49.0494956970215 46.3290367126465 68.9900817871094 41.3044586181641 107.418869018555 32.4451141357422 104.22282409668 34.1865348815918 45.6171340942383 43.4625015258789 160.689529418945 69.0750732421875 28.1006088256836 53.5338897705078 53.1366653442383 43.7745971679688 67.1805572509766 28.1616401672363 34.0473289489746 106.137542724609 70.8788070678711 33.0168762207031 66.4958572387695 44.674129486084 33.1360816955566 59.4718704223633 46.9115142822266 28.8023433685303 69.1866607666016 72.7074737548828 91.1968383789062 36.0340766906738 29.8911304473877 68.3647232055664 41.4983139038086 51.6220054626465 80.6972885131836 48.8108329772949 32.0613021850586 51.0625610351562 40.7951736450195 57.5715827941895 112.152679443359 72.4222869873047 27.2485599517822 125.958320617676 46.9066734313965 17.3113250732422 123.13436126709 33.6974182128906 39.7168045043945 49.223518371582 43.4147834777832 41.1337699890137 64.5210342407227 90.4320602416992 31.6032562255859 63.985237121582 27.4931030273438 39.3656921386719 28.5111637115479 45.2067565917969 62.1849899291992 15.5373191833496 50.9816398620605 46.6150131225586 47.9132652282715 51.2794456481934 37.7238883972168 36.4891166687012 68.9239883422852 34.8015518188477 41.4009284973145 59.1074295043945 61.1784973144531 18.9166259765625 38.8308334350586 51.323974609375 118.397163391113 27.7362995147705 25.746265411377 27.1767082214355 38.7822341918945 25.7319049835205 43.742431640625 94.4653091430664 80.9639587402344 31.4447708129883 75.1587524414062 68.5827789306641 78.4470672607422 60.7379112243652 69.2899475097656 37.6133613586426 131.912033081055 54.8637771606445 74.6701354980469 23.1046085357666 35.904541015625 63.985782623291 70.2196273803711 41.8395538330078 58.9469528198242 34.9716033935547 29.1167945861816 76.1432876586914 27.5234107971191 41.188159942627 71.8655014038086 36.4648475646973 91.631950378418 62.5442695617676 46.7944602966309 87.9197616577148 51.0710411071777 49.1575813293457

ENSG00000185627.16 83.6892623901367 55.4662284851074 210.745376586914 154.452926635742 136.635437011719 58.4776725769043 79.9061431884766 155.370407104492 86.824821472168 34.7489051818848 55.804801940918 53.9247360229492 96.0349349975586 81.717399597168 74.2869338989258 92.255859375 37.6809349060059 83.3475036621094 93.030158996582 61.9500122070312 128.901321411133 152.084732055664 123.347023010254 74.2964477539062 72.1927261352539 66.6108474731445 108.369445800781 65.8262786865234 72.2968902587891 111.227180480957 78.0033264160156 54.659969329834 68.5829010009766 76.2495498657227 102.030006408691 59.0970001220703 75.0298919677734 60.9621429443359 59.2691345214844 57.4500198364258 109.666328430176 126.907646179199 51.6841812133789 70.3321533203125 70.7129058837891 177.655578613281 92.3306732177734 124.182106018066 52.4541015625 83.965934753418 70.712287902832 131.367813110352 75.4111938476562 87.4718933105469 84.2836074829102 52.4668312072754 116.499801635742 89.8434600830078 66.4726867675781 169.147537231445 89.769775390625 76.7466888427734 114.024421691895 84.756217956543 57.762035369873 99.2036819458008 81.4674377441406 67.5157699584961 56.1301383972168 68.6514739990234 98.9795227050781 90.8236312866211 71.5377044677734 74.5920715332031 80.2573394775391 117.806594848633 147.878890991211 61.8219947814941 89.1498794555664 81.8599014282227 79.0733337402344 74.6054382324219 81.0999908447266 116.091117858887 90.3212280273438 60.5115394592285 90.7434844970703 113.736618041992 82.6621475219727 69.2947845458984 79.849494934082 125.427429199219 175.99755859375 102.637825012207 99.4411087036133 99.850471496582 118.428802490234 134.947738647461 95.1150131225586 66.3299560546875 118.71875 105.370658874512 77.5621566772461 64.2994918823242 79.5732879638672 83.3430557250977 99.5444030761719 76.3609313964844 90.6527252197266 45.5138549804688 82.6241607666016 79.0312347412109 67.4837265014648 120.268257141113 84.2781372070312 64.8559494018555 90.7291488647461 98.9474945068359 82.3551406860352 110.490303039551 59.8569145202637 58.569995880127 143.798126220703 68.236442565918 69.5063247680664 70.3773727416992 67.3091430664062 117.284736633301 83.966682434082 87.764533996582 84.2625274658203 73.6200408935547 63.4305648803711 66.05615234375 118.12866973877 62.2058563232422 70.546501159668 77.0535430908203 72.7991180419922 64.6602783203125 76.4585800170898 66.4360656738281 85.0754776000977 60.1512107849121 125.470947265625 38.5619239807129 150.657943725586 57.208251953125 75.5478363037109 71.5665664672852 132.021957397461 83.435417175293 73.3485260009766 95.7330932617188 92.6176452636719 121.68537902832 108.317451477051 56.7131843566895 63.9114379882812 123.037391662598 79.8317260742188 74.9693374633789 104.312034606934 104.958137512207 88.4492034912109 70.700927734375 68.2755966186523 62.4337615966797 109.159683227539 69.443977355957 97.5969467163086 122.072952270508 93.2441024780273 88.8530960083008 123.040374755859 65.5383453369141 74.4845962524414 51.258171081543 64.5377349853516 68.9592819213867 57.6679763793945 67.3864898681641 51.1906394958496 56.6931533813477 56.7327308654785 96.2112579345703 68.4201889038086 161.668472290039 94.4901428222656 55.7112998962402 64.2508163452148 120.274169921875 80.5589065551758 101.501823425293 80.2088241577148 65.564811706543 81.5481567382812 116.01537322998 60.7778778076172 64.7082290649414 47.4063529968262 74.6167221069336 93.9745788574219 55.5705490112305 55.4390602111816 72.0774765014648 79.5192413330078 81.5642852783203 58.0063858032227 52.8238716125488 51.7638473510742 55.6798248291016 77.5216674804688 81.8512802124023 89.5751724243164 40.5636024475098 67.5896911621094 83.59765625 96.9721832275391 66.2924423217773 75.0420074462891 86.4272994995117 51.6655349731445 182.860748291016 67.0425109863281 97.2549209594727 110.080764770508 61.6808929443359 71.2068252563477 79.953857421875 79.2964859008789 102.507675170898 239.023651123047 84.5616226196289 83.36083984375 92.9296035766602 86.2776412963867 71.4269332885742 86.0242919921875 96.9865036010742 66.5431823730469 51.8799324035645 102.026573181152 54.3601188659668 58.6727638244629 46.6458435058594 137.234329223633 73.4232177734375 102.057403564453 40.1340370178223 84.1335296630859 115.434181213379 67.077018737793 109.583381652832 70.1083221435547 79.2991180419922

ENSG00000092010.13 639.036010742188 272.257080078125 367.154357910156 306.695190429688 448.13720703125 327.954956054688 345.731048583984 310.095275878906 266.037750244141 172.768203735352 183.220260620117 268.611175537109 141.754501342773 263.119171142578 446.45166015625 196.032791137695 255.350234985352 408.641143798828 206.69270324707 337.872497558594 143.133010864258 431.122741699219 74.4621353149414 241.013687133789 368.623504638672 318.958618164062 341.199127197266 301.426300048828 228.137573242188 486.091644287109 373.872161865234 317.071319580078 311.169738769531 302.424377441406 674.637939453125 255.308639526367 427.636352539062 366.951354980469 245.146484375 223.305465698242 264.270263671875 378.667144775391 273.062103271484 280.655303955078 280.944732666016 976.3046875 280.151519775391 594.047180175781 240.146774291992 489.363983154297 351.829254150391 572.034729003906 300.775115966797 336.452941894531 254.572494506836 197.979827880859 542.386657714844 231.548797607422 241.251022338867 516.115173339844 454.741790771484 323.677001953125 820.393188476562 400.002075195312 264.423004150391 432.20751953125 373.115661621094 298.366668701172 341.36083984375 320.241455078125 312.623138427734 419.267517089844 361.227264404297 287.454742431641 386.497650146484 653.990600585938 705.771545410156 174.633422851562 254.229400634766 204.472793579102 423.825408935547 202.052780151367 470.381713867188 221.12353515625 677.412414550781 390.170135498047 376.266387939453 387.871063232422 246.487365722656 274.535003662109 179.636306762695 280.98046875 481.188293457031 610.856567382812 609.800964355469 272.581756591797 433.769683837891 597.836608886719 310.303894042969 256.338073730469 538.893981933594 376.410675048828 334.211822509766 222.811096191406 260.480590820312 371.494445800781 670.047424316406 248.953063964844 391.797332763672 150.064041137695 322.424682617188 403.177703857422 509.148834228516 453.782257080078 295.911376953125 302.699432373047 376.402770996094 196.129913330078 301.950775146484 260.550689697266 261.773406982422 391.4384765625 476.113189697266 363.577484130859 383.396728515625 398.136047363281 329.928009033203 342.5888671875 338.481323242188 304.044586181641 313.874084472656 467.867065429688 196.687515258789 315.383605957031 643.631042480469 241.747665405273 365.170379638672 276.587707519531 346.914428710938 328.547027587891 206.655410766602 333.654846191406 415.038452148438 217.118927001953 291.154876708984 108.048477172852 457.712341308594 475.457916259766 298.33642578125 162.665115356445 394.60888671875 457.698608398438 439.096008300781 329.541961669922 489.022430419922 331.4892578125 451.417266845703 183.267974853516 437.723785400391 329.231811523438 399.624572753906 186.956436157227 494.767883300781 251.997055053711 314.173309326172 574.6513671875 434.849914550781 293.113983154297 348.163757324219 503.411804199219 273.709716796875 705.428405761719 981.36767578125 145.650787353516 754.412719726562 220.402313232422 254.531478881836 296.438842773438 418.515441894531 288.172668457031 295.624877929688 312.644775390625 131.738830566406 436.200012207031 209.539001464844 299.787384033203 142.004135131836 716.347351074219 185.473037719727 208.357147216797 314.078033447266 599.567321777344 387.792022705078 254.666091918945 310.281585693359 419.681579589844 465.579833984375 235.300643920898 322.780426025391 203.470825195312 275.529724121094 336.424285888672 345.468505859375 198.144729614258 254.21028137207 321.701599121094 318.661956787109 271.005065917969 403.194976806641 319.642486572266 296.235260009766 248.386428833008 304.935089111328 517.029418945312 329.48193359375 84.0668563842773 568.569396972656 336.691223144531 155.446426391602 235.223434448242 342.977355957031 262.494873046875 355.060211181641 655.762390136719 210.019241333008 754.110900878906 524.79443359375 240.225952148438 516.947692871094 153.665512084961 158.093688964844 339.387756347656 812.039428710938 199.201873779297 246.405456542969 256.664398193359 313.6748046875 330.330596923828 283.470245361328 418.841278076172 368.958923339844 310.142303466797 305.146728515625 240.979522705078 307.282653808594 228.686492919922 625.814697265625 293.002746582031 542.845764160156 236.933944702148 465.593963623047 416.3583984375 295.084533691406 256.516998291016 363.814178466797 253.481109619141
[truncated: 4,976,140 more chars]
