# Supplementary material for: An Immune-Related Signature Predicts Survival in Patients With Lung Adenocarcinoma
Source: Front Oncol. 2019 Dec 10;9:1314. doi: 10.3389/fonc.2019.01314 (PMC6914845; doi:10.3389/fonc.2019.01314)
Supplement: Supplementary file 4 [file Table_4.doc]

**Table S4. Clinical follow-up information of training set samples.**

A1_OS A2_Event A3_T A4_N A5_M A6_Stage additional_studies additional_studies.additional_study.disease_code additional_studies.additional_study.project_code age_at_initial_pathologic_diagnosis anatomic_neoplasm_subdivision anatomic_neoplasm_subdivision_other bcr_patient_barcode bcr_patient_uuid day_of_form_completion days_to_birth days_to_death days_to_initial_pathologic_diagnosis days_to_last_followup days_to_last_known_alive diagnosis dlco_predictive_percent drugs drugs.drug.bcr_drug_barcode drugs.drug.bcr_drug_uuid drugs.drug.clinical_trail_drug_classification drugs.drug.day_of_form_completion drugs.drug.days_to_drug_therapy_end drugs.drug.days_to_drug_therapy_start drugs.drug.drug_name drugs.drug.measure_of_response drugs.drug.month_of_form_completion drugs.drug.number_cycles drugs.drug.prescribed_dose drugs.drug.prescribed_dose_units drugs.drug.regimen_indication drugs.drug.regimen_indication_notes drugs.drug.regimen_number drugs.drug.route_of_administrations.route_of_administration drugs.drug.therapy_ongoing drugs.drug.therapy_types.therapy_type drugs.drug.therapy_types.therapy_type_notes drugs.drug.total_dose drugs.drug.total_dose_units drugs.drug.tx_on_clinical_trial drugs.drug.year_of_form_completion eastern_cancer_oncology_group egfr_mutation_identified egfr_mutation_performed egfr_mutation_result eml4_alk_translocation_identified eml4_alk_translocation_method eml4_alk_translocation_performed eml4_alk_translocation_result ethnicity follow_ups follow_ups.follow_up.additional_pharmaceutical_therapy follow_ups.follow_up.additional_radiation_therapy follow_ups.follow_up.additional_surgery_locoregional_procedure follow_ups.follow_up.additional_surgery_metastatic_procedure follow_ups.follow_up.bcr_followup_barcode follow_ups.follow_up.bcr_followup_uuid follow_ups.follow_up.day_of_form_completion follow_ups.follow_up.days_to_additional_surgery_locoregional_procedure follow_ups.follow_up.days_to_additional_surgery_metastatic_procedure follow_ups.follow_up.days_to_death follow_ups.follow_up.days_to_last_followup follow_ups.follow_up.days_to_new_tumor_event_after_initial_treatment follow_ups.follow_up.eastern_cancer_oncology_group follow_ups.follow_up.followup_case_report_form_submission_reason follow_ups.follow_up.followup_treatment_success follow_ups.follow_up.karnofsky_performance_score follow_ups.follow_up.lost_follow_up follow_ups.follow_up.month_of_form_completion follow_ups.follow_up.new_neoplasm_event_types.new_neoplasm_event_type follow_ups.follow_up.new_tumor_event_after_initial_treatment follow_ups.follow_up.performance_status_scale_timing follow_ups.follow_up.person_neoplasm_cancer_status follow_ups.follow_up.postoperative_rx_tx follow_ups.follow_up.primary_therapy_outcome_success follow_ups.follow_up.progression_determined_by_list.progression_determined_by follow_ups.follow_up.radiation_therapy follow_ups.follow_up.vital_status follow_ups.follow_up.year_of_form_completion gender histological_type history_of_neoadjuvant_treatment icd_10 icd_o_3_histology icd_o_3_site informed_consent_verified karnofsky_performance_score kras_gene_analysis_performed kras_mutation_found kras_mutation_result location_in_lung_parenchyma month_of_form_completion new_tumor_events.new_tumor_event.additional_pharmaceutical_therapy new_tumor_events.new_tumor_event.additional_radiation_therapy new_tumor_events.new_tumor_event.days_to_new_tumor_event_after_initial_treatment new_tumor_events.new_tumor_event.locoregional_procedure.additional_surgery_locoregional_procedure new_tumor_events.new_tumor_event.locoregional_procedure.days_to_additional_surgery_locoregional_procedure new_tumor_events.new_tumor_event.metastatic_procedure.additional_surgery_metastatic_procedure new_tumor_events.new_tumor_event.metastatic_procedure.days_to_additional_surgery_metastatic_procedure new_tumor_events.new_tumor_event.new_neoplasm_event_types.new_neoplasm_event_type new_tumor_events.new_tumor_event.progression_determined_by_list.progression_determined_by new_tumor_events.new_tumor_event_after_initial_treatment number_pack_years_smoked other_dx patient_id performance_status_scale_timing person_neoplasm_cancer_status post_bronchodilator_fev1_fvc_percent post_bronchodilator_fev1_percent postoperative_rx_tx pre_bronchodilator_fev1_fvc_percent pre_bronchodilator_fev1_percent primary_therapy_outcome_success pulmonary_function_test_performed race_list.race radiation_therapy radiations radiations.radiation.anatomic_treatment_site radiations.radiation.bcr_radiation_barcode radiations.radiation.bcr_radiation_uuid radiations.radiation.course_number radiations.radiation.day_of_form_completion radiations.radiation.days_to_radiation_therapy_end radiations.radiation.days_to_radiation_therapy_start radiations.radiation.measure_of_response radiations.radiation.month_of_form_completion radiations.radiation.numfractions radiations.radiation.radiation_dosage radiations.radiation.radiation_treatment_ongoing radiations.radiation.radiation_type radiations.radiation.radiation_type_notes radiations.radiation.regimen_indication radiations.radiation.regimen_indication_notes radiations.radiation.units radiations.radiation.year_of_form_completion residual_tumor stage_event.ann_arbor.b_symptoms stage_event.ann_arbor.extranodal_involvement stage_event.clinical_stage stage_event.gleason_grading.gleason_score stage_event.gleason_grading.primary_pattern stage_event.gleason_grading.secondary_pattern stage_event.gleason_grading.tertiary_pattern stage_event.igcccg_stage stage_event.masaoka_stage stage_event.pathologic_stage stage_event.psa.days_to_psa stage_event.psa.psa_value stage_event.serum_markers stage_event.system_version stage_event.tnm_categories.clinical_categories.clinical_M stage_event.tnm_categories.clinical_categories.clinical_N stage_event.tnm_categories.clinical_categories.clinical_T stage_event.tnm_categories.pathologic_categories.pathologic_M stage_event.tnm_categories.pathologic_categories.pathologic_N stage_event.tnm_categories.pathologic_categories.pathologic_T stopped_smoking_year tissue_prospective_collection_indicator tissue_retrospective_collection_indicator tissue_source_site tobacco_smoking_history tumor_tissue_site vital_status year_of_form_completion year_of_initial_pathologic_diagnosis year_of_tobacco_smoking_onset

370 Dead T3 N2 M0 Stage IIIA NA 79 L-Upper Not Applicable TCGA-50-6594 8504fd86-a70a-4cba-9ec8-25c9e60ca549 25 -28924 370 0 Not Available Not Available Lung Adenocarcinoma Not Available NA Not Available Not Available Not Available Not Available Not Available Not Available YES Not Available NOT HISPANIC OR LATINO NA NO YES Not Available NO TCGA-50-6594-F44032 08A5B385-FE6B-4B2C-8BAF-CB57CE32AA51 13 Not Available Not Available 370 Not Available 285 Not Available Scheduled Follow-up Submission Progressive Disease Not Available NO 6 Distant Metastasis YES Not Available WITH TUMOR NO Progressive Disease Convincing Imaging NO Dead 2013 FEMALE Lung Adenocarcinoma- Not Otherwise Specified (NOS) No C34.1 8140/3 C34.1 YES Not Available Not Available NO Not Available Not Available 8 Not Available Not Available Not Available Not Available Not Available Not Available Not Available Not Available Not Available Not Available Not Available No 6594 Not Available WITH TUMOR Not Available Not Available Not Available Not Available Not Available Not Available Not Available BLACK OR AFRICAN AMERICAN Not Available NA Distant Recurrence TCGA-50-6594-R44126 FCD116A9-2F06-48CA-ACE7-2D26AD148F79 Not Available 13 318 317 Radiographic Progressive Disease 6 1 24 NO External Not Applicable Not Available Not Available Gy 2013 Not Available Not Applicable Not Applicable Not Applicable Not Applicable Not Applicable Not Applicable Not Applicable Not Applicable Not Applicable Stage IIIA Not Applicable Not Applicable Not Applicable 6th Not Applicable Not Applicable Not Applicable M0 N2 T3 1998 NO YES 50 4 Lung Dead 2011 2009 Not Available

NA T3 N0 M0 Stage IIB NA Not Available R-Lower Not Applicable TCGA-75-7030 df576520-a6b6-4c9b-8d06-3f59cc5342fd 16 Not Available Not Applicable Not Available Not Available Not Available Lung Adenocarcinoma Not Available NA TCGA-75-7030-D18798;TCGA-75-7030-D18797 0c13d354-be1b-4d41-a8d4-1579f902bf38;e83f6d9d-f80b-46df-948c-10ad8c4706a2 Not Available;Not Available 17;17 Not Available;Not Available Not Available;Not Available Cisplatin;Vinorelbine Tartrate Not Available;Not Available 11;11 4;4 92;46 mg/day;mg/day ADJUVANT;ADJUVANT Not Applicable;Not Applicable Not Available;Not Available IV;IV YES;NO Chemotherapy;Chemotherapy Not Available;Not Available 736;552 mg;mg Not Available;Not Available 2011;2011 0 Not Available Not Available Not Available Not Available Not Available Not Available Not Available Not Available NA NO NO NO NO TCGA-75-7030-F15878 63eba997-c2ac-44fa-8c2b-d620644e2410 16 Not Applicable Not Applicable Not Applicable Not Available Not Applicable 0 Not Available Complete Remission/Response Not Available Not Available 8 Not Available NO Post-Adjuvant Therapy Not Available YES Complete Remission/Response Not Available NO Alive 2011 MALE Lung Adenocarcinoma- Not Otherwise Specified (NOS) No C34.3 8140/3 C34.3 YES Not Available Not Available Not Available Not Available Peripheral Lung 8 Not Available Not Available Not Available Not Available Not Available Not Available Not Available Not Available Not Available Not Available Not Available No 7030 Preoperative TUMOR FREE Not Available Not Available Not Available Not Available Not Available Not Available Not Available Not Available Not Available NA R0 Not Applicable Not Applicable Not Applicable Not Applicable Not Applicable Not Applicable Not Applicable Not Applicable Not Applicable Stage IIB Not Applicable Not Applicable Not Applicable 6th Not Applicable Not Applicable Not Applicable M0 N0 T3 Not Available NO YES 75 1 Lung Alive 2011 2007 Not Available

7248 Alive T2 N0 M0 Stage IB NA 60 R-Lower Not Applicable TCGA-78-7163 5abd6651-69be-42f8-bdc0-308e68136d31 26 -22002 Not Applicable 0 6812 Not Available Lung Adenocarcinoma Not Available NA 1 Not Available NO Not Available Not Available Not Available NO Not Available Not Available NA Not Available;Not Available Not Available;Not Available Not Available;Not Available Not Available;Not Available TCGA-78-7163-F16824;TCGA-78-7163-F46140 18ffcd96-26be-4690-8195-11c72cba0f56;6E88BDD8-0163-4A50-AA1F-6E3E77AB3A5D 27;4 Not Applicable;Not Available Not Applicable;Not Available Not Applicable;Not Applicable 6812;7248 Not Applicable;Not Available Not Available;Unknown Scheduled Follow-up Submission;Scheduled Follow-up Submission Complete Remission/Response;Unknown Not Available;Not Available Not Available;NO 9;8 Not Available;Not Available NO;Unknown Not Available;Not Available TUMOR FREE;Unknown NO;NO Not Available;Complete Remission/Response Not Available;Not Available NO;NO Alive;Alive 2011;2013 MALE Lung Adenocarcinoma- Not Otherwise Specified (NOS) No C34.3 8140/3 C34.3 YES Not Available NO Not Available Not Available Peripheral Lung 9 Not Available Not Available Not Available Not Available Not Available Not Available Not Available Not Available Not Available Not Available 41 No 7163 Preoperative TUMOR FREE Not Available Not Available Not Available Not Available Not Available Not Available NO WHITE Not Available NA R0 Not Applicable Not Applicable Not Applicable Not Applicable Not Applicable Not Applicable Not Applicable Not Applicable Not Applicable Stage IB Not Applicable Not Applicable Not Applicable 6th Not Applicable Not Applicable Not Applicable M0 N0 T2 1991 NO YES 78 4 Lung Alive 2011 1992 1950

354 Dead T2 N0 M0 Stage IB NA 72 L-Upper Not Applicable TCGA-38-4631 2483621a-4db3-41ab-aa33-b9427ea8a0af 14 -26538 354 0 Not Available Not Available Lung Adenocarcinoma Not Available NA Not Available Not Available NO Not Available Not Available Not Available NO Not Available NOT HISPANIC OR LATINO NA NO YES NO NO TCGA-38-4631-F4892 48e7462e-19a5-4897-9104-e131fa220329 14 Not Available Not Available 354 Not Available Not Available Not Available Not Available Not Available Not Available Not Available 12 Not Available YES Not Available WITH TUMOR NO Progressive Disease Not Available NO Dead 2010 FEMALE Lung Adenocarcinoma- Not Otherwise Specified (NOS) No C34.1 8140/3 C34.1 YES Not Available NO Not Available Not Available Peripheral Lung 12 Not Available Not Available Not Available Not Available Not Available Not Available Not Available Not Available Not Available Not Available 40 Yes 4631 Not Available WITH TUMOR Not Available Not Available Not Available Not Available Not Available Not Available Not Available WHITE Not Available NA Distant site TCGA-38-4631-R4893 adf6e9ab-7efe-43bf-83e4-ae864d19e186 1 14 115 87 Not Available 12 Not Available Not Available NO EXTERNAL BEAM Not Applicable PALLIATIVE Not Available Not Available 2010 R0 Not Applicable Not Applicable Not Applicable Not Applicable Not Applicable Not Applicable Not Applicable Not Applicable Not Applicable Stage IB Not Applicable Not Applicable Not Applicable Not Available Not Applicable Not Applicable Not Applicable M0 N0 T2 Not Available NO YES 38 4 Lung Dead 2010 1997 Not Available

NA T2 N2 M0 Stage IIIA NA Not Available R-Upper Not Applicable TCGA-75-6207 58babefe-7e81-4594-ba29-50d1de92a5ab 21 Not Available Not Available Not Available Not Available Not Available Lung Adenocarcinoma Not Available NA Not Available Not Available Not Available Not Available Not Available Not Available Not Available Not Available Not Available NA NO NO Not Available Not Available TCGA-75-6207-F15092 53c4832a-9167-41ab-8172-0713906b893e 21 Not Available Not Available Not Available Not Available Not Available Not Available Not Available Not Available Not Available Not Available 7 Not Available Not Available Not Available Not Available NO Complete Remission/Response Not Available NO Dead 2011 MALE Lung Adenocarcinoma- Not Otherwise Specified (NOS) No C34.1 8140/3 C34.9 YES Not Available Not Available Not Available Not Available Peripheral Lung 7 Not Available Not Available Not Available Not Available Not Available Not Available Not Available Not Available Not Available Not Available 10 No 6207 Not Available Not Available Not Available Not Available Not Available Not Available Not Available Not Available Not Available Not Available Not Available NA R0 Not Applicable Not Applicable Not Applicable Not Applicable Not Applicable Not Applicable Not Applicable Not Applicable Not Applicable Stage IIIA Not Applicable Not Applicable Not Applicable 6th Not Applicable Not Applicable Not Applicable M0 N2 T2 1972 NO YES 75 3 Lung Dead 2011 2007 Not Available

800 Dead T1 N0 M0 Stage IA NA 72 R-Upper Not Applicable TCGA-38-7271 8214a0d1-5e2d-4a7a-acb1-e5580755db83 10 -26440 800 0 Not Available Not Available Lung Adenocarcinoma 76 NA TCGA-38-7271-D18306;TCGA-38-7271-D18311;TCGA-38-7271-D18310 39b80d4f-cb2c-42f3-9e88-32dadd933c17;be281462-f8ee-4c19-95d2-3a8f7295265f;fac1fe79-b59c-4d7b-b36a-89b3c8e29166 Not Available;Not Available;Not Available 10;10;10 371;623;574 329;574;574 Cisplatin;Alimta;Carboplatin Not Available;Not Available;Not Available 11;11;11 3;2;1 30;500;Not Available mg/m2;mg/m2;AUC RECURRENCE;PALLIATIVE;PALLIATIVE Not Applicable;Not Applicable;Not Applicable 1;2;2 IV;IV;IV NO;NO;NO Chemotherapy;Chemotherapy;Chemotherapy Not Available;Not Available;Not Available 324;1760;798 mg;mg;mg Not Available;Not Available;Not Available 2011;2011;2011 Not Available Not Available NO Not Available Not Available Not Available NO Not Available NOT HISPANIC OR LATINO NA YES;YES;NO YES;YES;YES NO;NO;NO Not Available;NO;NO TCGA-38-7271-F18305;TCGA-38-7271-F18308;TCGA-38-7271-F18312 f7734211-9e7c-43ca-90e7-5c8de6e7bfee;24d3b53b-221b-4620-9d62-d627741e2a70;620bde19-e077-43c4-b7e0-f3b00df96316 10;10;10 Not Available;Not Available;Not Available Not Available;Not Available;Not Available 800;800;800 Not Available;Not Available;Not Available 304;561;633 Not Available;Not Available;Not Available Additional New Tumor Event;Additional New Tumor Event;Additional New Tumor Event Stable Disease;Progressive Disease;Progressive Disease Not Available;Not Available;Not Available Not Available;Not Available;Not Available 11;11;11 Not Available;Not Available;Not Available YES;YES;YES Not Available;Not Available;Not Available WITH TUMOR;WITH TUMOR;WITH TUMOR NO;Not Available;Not Available Complete Remission/Response;Not Available;Not Available Not Available;Not Available;Not Available NO;Not Available;Not Available Dead;Dead;Dead 2011;2011;2011 FEMALE Lung Adenocarcinoma- Not Otherwise Specified (NOS) No C34.1 8140/3 C34.1 YES Not Available NO Not Available Not Available Not Available 11 Not Available Not Available Not Available Not Available Not Available Not Available Not Available Not Available Not Available Not Available 25 Yes 7271 Not Available WITH TUMOR 46 45 Not Available 43 40 Not Available YES WHITE Not Available NA Distant site;Distant site;Local Recurrence TCGA-38-7271-R18309;TCGA-38-7271-R18313;TCGA-38-7271-R18307 06943636-0118-4a9a-80ad-eda14819da97;a027a42b-2d51-47e7-9dcf-60f81b2d234d;a8e5e91e-4df7-465e-88b0-eb30765f0f30 2;3;1 10;10;10 564;665;379 564;633;329 Not Available;Not Available;Not Available 11;11;11 1;14;23 2000;2500;7000 NO;NO;NO OTHER;EXTERNAL BEAM;EXTERNAL BEAM Cyber Knife Stereotactic Radiosurgery;Not Applicable;Not Applicable PALLIATIVE;PALLIATIVE;RECURRENCE Not Available;Not Available;Not Available cGy;cGy;cGy 2011;2011;2011 R0 Not Applicable Not Applicable Not Applicable Not Applicable Not Applicable Not Applicable Not Applicable Not Applicable Not Applicable Stage IA Not Applicable Not Applicable Not Applicable 6th Not Applicable Not Applicable Not Applicable M0 N0 T1 1999 NO YES 38 4 Lung Dead 2011 2006 1971

462 Alive T2a N0 MX Stage IB NA 59 R-Upper Not Applicable TCGA-55-8087 e254aaeb-012c-4d92-8a03-cce1f6a2bcd6 15 -21655 Not Applicable 0 21 Not Available Lung Adenocarcinoma 142 NA Not Available Not Available NO Not Available Not Available Not Available NO Not Available NOT HISPANIC OR LATINO NA Not Available Not Available Not Available Not Available TCGA-55-8087-F47822 7E1BBF1B-2639-4442-A633-A2B9D0B088C9 28 Not Applicable Not Applicable Not Applicable 462 Not Applicable Not Evaluated Scheduled Follow-up Submission Complete Remission/Response Not Evaluated NO 8 Not Available NO Not Evaluated TUMOR FREE NO Complete Remission/Response Not Available NO Alive 2013 FEMALE Lung Adenocarcinoma- Not Otherwise Specified (NOS) No C34.1 8140/3 C34.1 YES Not Available NO Not Available Not Available Not Available 6 Not Available Not Available Not Available Not Available Not Available Not Available Not Available Not Available Not Available NO Not Available No 8087 Not Available TUMOR FREE Not Available Not Available Unknown Not Available 126 Complete Remission/Response YES WHITE Unknown NA R0 Not Applicable Not Applicable Not Applicable Not Applicable Not Applicable Not Applicable Not Applicable Not Applicable Not Applicable Stage IB Not Applicable Not Applicable Not Applicable 7th Not Applicable Not Applicable Not Applicable MX N0 T2a Not Available YES NO 55 1 Lung Alive 2012 2011 Not Available

1492 Dead T2 N1 M0 Stage IIB NA 65 R-Upper Not Applicable TCGA-38-4628 cc4bd56a-25c5-4c48-b583-ac3aeb778ca6 9 -24057 1492 0 Not Available Not Available Lung Adenocarcinoma 72 NA TCGA-38-4628-D4678;TCGA-38-4628-D13831 0c5ddee7-c7b0-44a1-97d7-c6eb11c9b514;357b5e2f-bb1b-41f7-8c7c-4ea6dd8a2fd3 Not Available;Not Available 9;14 1238;1238 1147;1147 Carboplatin;Taxol Not Available;Not Available 12;7 4;4 Not Available;Not Available Not Available;Not Available RECURRENCE;RECURRENCE Not Applicable;Not Applicable 1;1 IV;IV NO;NO Chemotherapy;Chemotherapy Not Available;Not Available Not Available;Not Available Not Available;Not Available Not Available;Not Available 2010;2011 Not Available Not Available NO Not Available Not Available Not Available NO Not Available NOT HISPANIC OR LATINO NA YES NO NO YES TCGA-38-4628-F4676 cca64327-9837-4503-abfd-9e6ab40ff8dc 9 Not Available 1118 1492 Not Available 1083 Not Available Not Available Stable Disease Not Available Not Available 12 Not Available YES Not Available WITH TUMOR NO Complete Remission/Response Not Available NO Dead 2010 FEMALE Lung Adenocarcinoma- Not Otherwise Specified (NOS) No C34.1 8140/3 C34.1 YES Not Available NO Not Available Not Available Central Lung 12 Not Available Not Available Not Available Not Available Not Available Not Available Not Available Not Available Not Available Not Available Not Available No 4628 Not Available WITH TUMOR 78 85 Not Available 74 86 Not Available YES WHITE Not Available NA R0 Not Applicable Not Applicable Not Applicable Not Applicable Not Applicable Not Applicable Not Applicable Not Applicable Not Applicable Stage IIB Not Applicable Not Applicable Not Applicable 5th Not Applicable Not Applicable Not Applicable M0 N1 T2 Not Available NO YES 38 1 Lung Dead 2010 2002 Not Available

33 Dead T1a N0 MX Stage IA NA 70 R-Lower Not Applicable TCGA-93-A4JO 3DAEBE75-C6DF-478E-8A4C-11186FD5EAD9 27 -25898 33 0 Not Available Not Available Lung Adenocarcinoma 81 NA 1 Not Available NO Not Available Not Available Not Available NO Not Available NOT HISPANIC OR LATINO NA MALE Lung Adenocarcinoma Mixed Subtype No C34.3 8255/3 C34.3 YES 90 NO Not Available Not Available Unknown 2 Not Available Not Available Not Available Not Available Not Available Not Available Not Available Not Available Not Available NO 50 No A4JO Preoperative TUMOR FREE 126 97 NO 125 98 Not Applicable YES WHITE NO NA RX Not Applicable Not Applicable Not Applicable Not Applicable Not Applicable Not Applicable Not Applicable Not Applicable Not Applicable Stage IA Not Applicable Not Applicable Not Applicable 7th Not Applicable Not Applicable Not Applicable MX N0 T1a 2006 YES NO 93 4 Lung Dead 2014 2012 1956

1289 Alive T3 N0 M0 Stage IIB NA 70 R-Lower Not Applicable TCGA-62-8397 9240d5fc-de23-4436-8099-da9bd3054860 4 -25728 Not Applicable 0 940 Not Available Lung Adenocarcinoma Not Available NA 0 Not Available NO Not Available Not Available Not Available NO Not Available NOT HISPANIC OR LATINO NA Not Available Not Available Not Available Not Available TCGA-62-8397-F42100 FF6E4644-94D8-4449-857F-11AF95093868 15 Not Applicable Not Applicable Not Applicable 1289 Not Applicable 0 Scheduled Follow-up Submission Complete Remission/Response 90 NO 5 Not Available NO Preoperative TUMOR FREE NO Complete Remission/Response Not Available NO Alive 2013 FEMALE Lung Adenocarcinoma Mixed Subtype No C34.3 8255/3 C34.3 YES 90 NO Not Available Not Available Peripheral Lung 7 Not Available Not Available Not Available Not Available Not Available Not Available Not Available Not Available Not Available NO Not Available No 8397 Preoperative TUMOR FREE Not Available Not Available NO 64 86 Complete Remission/Response YES WHITE NO NA R0 Not Applicable Not Applicable Not Applicable Not Applicable Not Applicable Not Applicable Not Applicable Not Applicable Not Applicable Stage IIB Not Applicable Not Applicable Not Applicable 6th Not Applicable Not Applicable Not Applicable M0 N0 T3 Not Available NO YES 62 1 Lung Alive 2012 2008 Not Available

617 Alive T2a N1 MX Stage IIA NA 60 R-Upper Not Applicable TCGA-55-8508 bab335f9-b241-41a7-b107-79b9676b37b8 28 -22159 Not Applicable 0 15 Not Available Lung Adenocarcinoma Not Available NA TCGA-55-8508-D66138;TCGA-55-8508-D66139 09E9C95E-CD77-45FB-AF89-25C96E794AB8;E05A3BB7-4A18-439D-BDD9-4DBBA8128428 Not Available;Not Available 3;3 120;120 57;57 Cisplatin;Pemetrexed Complete Response;Complete Response 10;10 Not Available;Not Available Not Available;Not Available Not Available;Not Available Not Available;Not Available Not Applicable;Not Applicable Not Available;Not Available Not Available;Not Available NO;NO Chemotherapy;Chemotherapy Not Available;Not Available Not Available;Not Available Not Available;Not Available NO;NO 2014;2014 2 Not Available Unknown Not Available Not Available Not Available Unknown Not Available NOT HISPANIC OR LATINO NA Not Available Not Available Not Available Not Available TCGA-55-8508-F66137 58A4C50E-35F0-439A-B28A-732F1048D6C4 3 Not Applicable Not Applicable Not Applicable 617 Not Applicable Not Available Scheduled Follow-up Submission Complete Remission/Response Not Available NO 10 Not Available NO Not Available TUMOR FREE YES Complete Remission/Response Not Available NO Alive 2014 FEMALE Mucinous (Colloid) Carcinoma No C34.1 8480/3 C34.1 YES 60 Unknown Not Available Not Available Central Lung 12 Not Available Not Available Not Available Not Available Not Available Not Available Not Available Not Available Not Available NO Not Available No 8508 Preoperative Unknown Not Available Not Available YES Not Available Not Available Complete Remission/Response Not Available BLACK OR AFRICAN AMERICAN NO NA R0 Not Applicable Not Applicable Not Applicable Not Applicable Not Applicable Not Applicable Not Applicable Not Applicable Not Applicable Stage IIA Not Applicable Not Applicable Not Applicable 7th Not Applicable Not Applicable Not Applicable MX N1 T2a Not Available YES NO 55 2 Lung Alive 2012 2012 Not Available

434 Dead T1a N0 MX Stage IA NA 75 R-Upper Not Applicable TCGA-91-8497 4ad63dac-76a7-4527-abce-d7a0184e8739 20 -27632 Not Applicable 0 174 Not Available Lung Adenocarcinoma Not Available NA Not Evaluated Not Available Not Available Not Available Not Available Not Available Not Available Not Available NOT HISPANIC OR LATINO NA Not Available Not Available Not Available Not Available TCGA-91-8497-F58334 4D8EEDE1-07CE-42E8-89C1-747366728090 7 Not Applicable Not Applicable 434 Not Available Not Applicable Not Evaluated Scheduled Follow-up Submission Complete Remission/Response Not Evaluated NO 4 Not Available NO Not Available TUMOR FREE NO Complete Remission/Response Not Available NO Dead 2014 FEMALE Lung Adenocarcinoma- Not Otherwise Specified (NOS) No C34.1 8140/3 C34.1 YES Not Evaluated NO Not Available Not Available Unknown 8 Not Available Not Available Not Available Not Available Not Available Not Available Not Available Not Available Not Available NO Not Available No 8497 Not Evaluated Unknown Not Available Not Available NO Not Available Not Available Unknown NO WHITE NO NA Not Evaluated Not Applicable Not Applicable Not Applicable Not Applicable Not Applicable Not Applicable Not Applicable Not Applicable Not Applicable Stage IA Not Applicable Not Applicable Not Applicable 7th Not Applicable Not Applicable Not Applicable MX N0 T1a Not Available YES NO 91 1 Lung Alive 2012 2012 Not Available

824 Alive T1a N0 MX Stage IA NA 60 R-Upper Not Applicable TCGA-55-7570 ff07ea4b-4e50-410d-99d6-96a351dad7b1 11 -22218 Not Applicable 0 6 Not Available Lung Adenocarcinoma Not Available NA Not Available Not Available Not Available Not Available Not Available Not Available Not Available Not Available NOT HISPANIC OR LATINO NA Not Available Not Available Not Available Not Available TCGA-55-7570-F56185 A3150ABF-460E-41B1-8D92-F9298F52C36B 29 Not Applicable Not Applicable Not Applicable 824 Not Applicable Not Evaluated Scheduled Follow-up Submission Complete Remission/Response Not Evaluated NO 1 Not Available NO Not Evaluated TUMOR FREE NO Complete Remission/Response Not Available NO Alive 2014 MALE Lung Adenocarcinoma- Not Otherwise Specified (NOS) No C34.1 8140/3 C34.1 YES Not Available Not Available Not Available Not Available Not Available 1 Not Available Not Available Not Available Not Available Not Available Not Available Not Available Not Available Not Available Not Available Not Available No 7570 Not Available TUMOR FREE Not Available Not Available Not Available Not Available Not Available Not Available Not Available BLACK OR AFRICAN AMERICAN Not Available NA R0 Not Applicable Not Applicable Not Applicable Not Applicable Not Applicable Not Applicable Not Applicable Not Applicable Not Applicable Stage IA Not Applicable Not Applicable Not Applicable 7th Not Applicable Not Applicable Not Applicable MX N0 T1a Not Available YES NO 55 2 Lung Alive 2012 2011 1971

806 Alive T2a N1 M0 Stage IIA NA 50 R-Middle Not Applicable TCGA-86-8674 0a1eea76-e19a-4547-9b24-abf7051cc4db 30 -18479 Not Applicable 0 0 Not Available Lung Adenocarcinoma Not Available NA TCGA-86-8674-D42028;TCGA-86-8674-D42029 55CE92CA-ED7F-460F-BCDD-25F915DE1A54;34F415AA-90DB-4E2C-9F08-160D35EAA160 Not Available;Not Available 9;9 81;81 57;57 Vinblastine;Cisplatin Clinical Progressive Disease;Clinical Progressive Disease 4;4 Not Available;Not Available Not Available;Not Available Not Available;Not Available Not Available;Not Available Not Applicable;Not Applicable Not Available;Not Available Not Available;Not Available NO;NO Chemotherapy;Chemotherapy Not Available;Not Available Not Available;Not Available Not Available;Not Available NO;NO 2013;2013 Unknown Not Available Unknown Not Available Not Available Not Available Unknown Not Available NOT HISPANIC OR LATINO NA NO;Not Available NO;Not Available NO;Not Available Not Available;Not Available TCGA-86-8674-F42027;TCGA-86-8674-F59096 5B567A02-6171-4C9E-ACB9-E6DBF6EEA647;6757DE70-2A11-4D3A-8663-03DCF4FC2E57 9;30 Not Available;Not Applicable Not Available;Not Applicable Not Applicable;Not Applicable 405;806 334;Not Applicable Not Evaluated;Not Evaluated Scheduled Follow-up Submission;Scheduled Follow-up Submission Progressive Disease;Complete Remission/Response Not Evaluated;Not Evaluated NO;NO 4;4 Locoregional Recurrence;Not Available YES;NO Not Evaluated;Not Evaluated WITH TUMOR;TUMOR FREE YES;YES Complete Remission/Response;Complete Remission/Response Not Available;Not Available NO;NO Alive;Alive 2013;2014 MALE Lung Papillary Adenocarcinoma No C34.2 8260/3 C34.2 YES Unknown Unknown Not Available Not Available Peripheral Lung 8 Not Available Not Available Not Available Not Available Not Available Not Available Not Available Not Available Not Available NO 37.5 No 8674 Not Available TUMOR FREE Not Available Not Available Unknown Not Available Not Available Complete Remission/Response NO WHITE Unknown NA R0 Not Applicable Not Applicable Not Applicable Not Applicable Not Applicable Not Applicable Not Applicable Not Applicable Not Applicable Stage IIA Not Applicable Not Applicable Not Applicable 7th Not Applicable Not Applicable Not Applicable M0 N1 T2a 2012 YES NO 86 4 Lung Alive 2012 2012 1982

1215 Dead T2 N0 M0 Stage IB NA 65 R-Upper Not Applicable TCGA-78-7152 b56dad1e-2fd5-47d8-9cc7-a54be9a1bae2 29 -23782 Not Applicable 0 1202 Not Available Lung Adenocarcinoma Not Available NA 1 Not Available NO Not Available Not Available Not Available NO Not Available Not Available NA Not Available;NO YES;YES NO;Not Available NO;NO TCGA-78-7152-F16986;TCGA-78-7152-F46144 37c9fd67-631d-4341-998d-1c995eaa7999;D9E98591-BA37-4BE6-9F12-3E9E4DBED53B 29;4 Not Available;Not Available Not Available;Not Available Not Applicable;1215 1202;Not Available 1202;1202 3;Unknown Scheduled Follow-up Submission;Scheduled Follow-up Submission Not Available;Progressive Disease Not Available;Not Available Not Available;NO 9;8 Not Available;Distant Metastasis YES;YES Other;Not Available WITH TUMOR;WITH TUMOR NO;NO Not Available;Complete Remission/Response Not Available;Biopsy with Histologic Confirmation;Convincing Imaging NO;NO Alive;Dead 2011;2013 MALE Lung Adenocarcinoma Mixed Subtype No C34.1 8255/3 C34.1 YES Not Available NO Not Available Not Available Peripheral Lung 9 Not Available Not Available Not Available Not Available Not Available Not Available Not Available Not Available Not Available Not Available 40 No 7152 Preoperative WITH TUMOR Not Available Not Available Not Available Not Available Not Available Not Available NO WHITE Not Available NA Distant site TCGA-78-7152-R16987 b75b20df-f44d-4dad-b13a-6212725b3643 1 29 Not Available 1154 Not Available 9 Not Available Not Available NO EXTERNAL BEAM Not Applicable PALLIATIVE Not Available Not Available 2011 R0 Not Applicable Not Applicable Not Applicable Not Applicable Not Applicable Not Applicable Not Applicable Not Applicable Not Applicable Stage IB Not Applicable Not Applicable Not Applicable 6th Not Applicable Not Applicable Not Applicable M0 N0 T2 1999 NO YES 78 4 Lung Alive 2011 2003 1959

260 Dead T3 N0 MX Stage IIB NA 61 Other (please specify) ALL 3 LOBES OF RT LUNG TCGA-49-AAR9 47B749E6-E060-4AA1-8F29-976E489BA789 18 -22510 260 0 Not Available Not Available Lung Adenocarcinoma Not Available NA 1 Not Available Not Available Not Available Not Available Not Available Not Available Not Available NOT HISPANIC OR LATINO NA MALE Lung Adenocarcinoma- Not Otherwise Specified (NOS) No C34.1 8140/3 C34.9 YES 80 Not Available Not Available Not Available Not Available 6 Not Available Not Available Not Available Not Available Not Available Not Available Not Available Not Available Not Available NO Not Available No AAR9 Preoperative WITH TUMOR Not Available Not Available NO Not Available Not Available Progressive Disease NO BLACK OR AFRICAN AMERICAN NO NA R0 Not Applicable Not Applicable Not Applicable Not Applicable Not Applicable Not Applicable Not Applicable Not Applicable Not Applicable Stage IIB Not Applicable Not Applicable Not Applicable 5th Not Applicable Not Applicable Not Applicable MX N0 T3 Not Available NO YES 49 2 Lung Dead 2014 1999 Not Available

1617 Alive T1 N0 MX Stage IA NA 62 R-Upper Not Applicable TCGA-50-5946 c95957a7-1a1a-4c8d-bb61-7c99b500f224 2 -22852 Not Applicable 0 349 Not Available Lung Adenocarcinoma Not Available NA Not Available Not Available Not Available Not Available Not Available Not Available Not Available Not Available Not Available NA YES;NO YES;NO NO;Not Available YES;NO TCGA-50-5946-F32143;TCGA-50-5946-F70455 28fbb9d8-ddc3-44a6-9f34-2ee25dbe35c3;27EC65A2-6B63-4A0F-A8E3-7C0C7905CF5F 17;16 Not Available;Not Available 221;Not Available Not Applicable;Not Applicable 686;1617 221;811 Not Available;Not Available Scheduled Follow-up Submission;Not Available Progressive Disease;Progressive Disease Not Available;Not Available NO;NO 5;2 Distant Metastasis;Distant Metastasis YES;YES Not Available;Not Available WITH TUMOR;WITH TUMOR NO;NO Progressive Disease;Progressive Disease Biopsy with Histologic Confirmation;Convincing Imaging;Convincing Imaging NO;NO Alive;Alive 2012;2015 MALE Lung Adenocarcinoma Mixed Subtype No C34.1 8255/3 C34.1 YES Not Available Not Available Not Available Not Available Not Available 7 Not Available Not Available Not Available Not Available Not Available Not Available Not Available Not Available Not Available Not Available Not Available No 5946 Not Available WITH TUMOR Not Available Not Available Not Available Not Available Not Available Not Available NO WHITE Not Available NA Not Available Not Applicable Not Applicable Not Applicable Not Applicable Not Applicable Not Applicable Not Applicable Not Applicable Not Applicable Stage IA Not Applicable Not Applicable Not Applicable Not Available Not Applicable Not Applicable Not Applicable MX N0 T1 2009 NO YES 50 4 Lung Alive 2011 2010 Not Available

598 Dead T1a N0 M0 Stage IA NA 80 R-Lower Not Applicable TCGA-55-8090 4d51ee44-e6f4-4bcb-be28-e9df54b39a8d 8 -29235 Not Applicable 0 6 Not Available Lung Adenocarcinoma 58 NA Not Available Not Available Not Available Not Available Not Available Not Available Not Available Not Available NOT HISPANIC OR LATINO NA NO NO Not Available NO TCGA-55-8090-F56189 05C48DBC-1101-4B07-947B-180BC0D657EE 29 Not Available Not Available 598 Not Available 548 Unknown Scheduled Follow-up Submission Progressive Disease Unknown NO 1 Distant Metastasis YES Not Available WITH TUMOR NO Complete Remission/Response Convincing Imaging NO Dead 2014 MALE Lung Adenocarcinoma- Not Otherwise Specified (NOS) No C34.3 8140/3 C34.3 YES Not Available Not Available Not Available Not Available Not Available 7 Not Available Not Available Not Available Not Available Not Available Not Available Not Available Not Available Not Available NO Not Available Yes, History of Synchronous/Bilateral Malignancy 8090 Not Available WITH TUMOR 4 6 Unknown 103 83 Complete Remission/Response YES WHITE Unknown NA R0 Not Applicable Not Applicable Not Applicable Not Applicable Not Applicable Not Applicable Not Applicable Not Applicable Not Applicable Stage IA Not Applicable Not Applicable Not Applicable 7th Not Applicable Not Applicable Not Applicable M0 N0 T1a Not Available YES NO 55 2 Lung Alive 2012 2011 Not Available

467 Alive T2 N1 M0 Stage IIB NA 66 L-Upper Not Applicable TCGA-73-4668 b2285924-9813-4f46-bb8f-91a0efd8a9b6 6 -24255 Not Applicable 0 467 Not Available Lung Adenocarcinoma Not Available NA TCGA-73-4668-D7959;TCGA-73-4668-D7956;TCGA-73-4668-D7960;TCGA-73-4668-D7957 b2535da4-0fa6-43d0-92c1-05717101d861;0a6f15d0-842e-4da6-8de0-8374e694987a;cca6912e-8a06-4270-8717-86cfd00d2ec7;9eb9db2a-643c-49ca-ab5b-7dd90adcbbc5 Not Available;Not Available;Not Available;Not Available 6;6;6;6 Not Available;110;Not Available;110 369;43;369;43 Gemcitabine;Pemetrexed;Carboplatin;Carboplatin Not Available;Not Available;Not Available;Not Available 1;1;1;1 5;4;5;4 1000;500;5;6 mg/m2;mg/m2;AUC;AUC PALLIATIVE;ADJUVANT;PALLIATIVE;ADJUVANT Not Applicable;Not Applicable;Not Applicable;Not Applicable 2;1;2;1 IV;IV;IV;IV YES;NO;YES;NO Chemotherapy;Chemotherapy;Chemotherapy;Chemotherapy Not Available;Not Available;Not Available;Not Available Not Available;4320;Not Available;2184 Not Available;mg;Not Available;mg Not Available;Not Available;Not Available;Not Available 2011;2011;2011;2011 0 Not Available NO Not Available Not Available Not Available NO Not Available NOT HISPANIC OR LATINO NA YES;YES NO;YES NO;NO NO;NO TCGA-73-4668-F7955;TCGA-73-4668-F7958 c82cb3f9-fb87-4f7f-80c0-9ec28a8368c0;32244464-38fc-4145-a984-d23cc7196130 6;6 Not Available;Not Available Not Available;Not Available Not Applicable;Not Applicable 467;467 236;282 0;Not Available Not Available;Not Available Not Available;Stable Disease 90;80 Not Available;Not Available 1;1 Not Available;Not Available YES;YES Post-Adjuvant Therapy;Post-Adjuvant Therapy WITH TUMOR;WITH TUMOR YES;Not Available Complete Remission/Response;Not Available Not Available;Not Available NO;NO Alive;Alive 2011;2011 FEMALE Lung Micropapillary Adenocarcinoma No C34.1 8507/3 C34.1 YES 100 NO Not Available Not Available Peripheral Lung 1 Not Available Not Available Not Available Not Available Not Available Not Available Not Available Not Available Not Available Not Available 55 No 4668 Preoperative WITH TUMOR Not Available Not Available Not Available Not Available Not Available Not Available Not Available AMERICAN INDIAN OR ALASKA NATIVE Not Available NA Distant site TCGA-73-4668-R7962 2be59a25-2f78-497d-a99d-68364ee85d2f 1 6 299 299 Not Available 1 1 4000 NO OTHER Gamma Knife PROGRESSION Not Available cGy 2011 R0 Not Applicable Not Applicable Not Applicable Not Applicable Not Applicable Not Applicable Not Applicable Not Applicable Not Applicable Stage IIB Not Applicable Not Applicable Not Applicable Not Available Not Applicable Not Applicable Not Applicable M0 N1 T2 2005 NO YES 73 4 Lung Alive 2011 2009 1968

1071 Alive T2a N1 MX Stage IIA NA 63 L-Upper Not Applicable TCGA-53-A4EZ 7CA711EC-9AC0-4FA7-8A60-32AE84B8789A 7 -23201 Not Applicable 0 280 Not Available Lung Adenocarcinoma 86 NA TCGA-53-A4EZ-D40957;TCGA-53-A4EZ-D40958 EAB7D354-078B-4978-AD4F-A6B8C3C68D35;58DBAB3C-5977-4D7B-8AF2-07D3D6AC5F27 Not Available;Not Available 7;7 226;226 132;132 Cisplatin;Navelbine Complete Response;Complete Response 3;3 Not Available;Not Available Not Available;Not Available Not Available;Not Available Not Available;Not Available Not Applicable;Not Applicable Not Available;Not Available Not Available;Not Available NO;NO Chemotherapy;Chemotherapy Not Available;Not Available Not Available;Not Available Not Available;Not Available NO;NO 2013;2013 0 Not Available YES Not Available Not Available FISH YES Not Available HISPANIC OR LATINO NA Not Available Not Available Not Available Not Available TCGA-53-A4EZ-F61081 F04EA78A-0FDE-41CC-8D26-94D7E06CF8A2 19 Not Applicable Not Applicable Not Applicable 1071 Not Applicable 1 Scheduled Follow-up Submission Complete Remission/Response 80 NO 3 Not Available NO Post-Adjuvant Therapy TUMOR FREE YES Stable Disease Not Available YES Alive 2015 MALE Lung Acinar Adenocarcinoma No C34.1 8550/3 C34.1 YES 100 YES NO Not Available Peripheral Lung 3 Not Available Not Available Not Available Not Available Not Available Not Available Not Available Not Available Not Available NO 40 No A4EZ Preoperative TUMOR FREE 73 75 YES 73 76 Not Applicable YES WHITE NO NA Primary Tumor Field TCGA-53-A4EZ-R71153 8A1064A2-9B4F-4B7B-A6E4-5A95F83F36B1 Not Available 20 428 Not Available Not Available 3 30 2 NO Systemic Not Applicable Not Available Not Available Gy 2015 RX Not Applicable Not Applicable Not Applicable Not Applicable Not Applicable Not Applicable Not Applicable Not Applicable Not Applicable Stage IIA Not Applicable Not Applicable Not Applicable 7th Not Applicable Not Applicable Not Applicable MX N1 T2a 1996 YES NO 53 3 Lung Alive 2013 2012 1956

827 Alive T2a N1 M0 Stage IIA NA 60 R-Upper Not Applicable TCGA-MN-A4N1 75A0BB0B-6528-4FB8-A9E6-254905D21DF4 4 -21939 Not Applicable 0 285 Not Available Lung Adenocarcinoma 55 NA 0 Not Available YES Not Available Not Available IHC YES Not Available NOT HISPANIC OR LATINO NA Not Available Not Available Not Available Not Available TCGA-MN-A4N1-F56662 0BAC11C6-A02B-49D5-A830-26313F1AC0F6 17 Not Applicable Not Applicable Not Applicable 827 Not Applicable Not Available Scheduled Follow-up Submission Complete Remission/Response Not Available NO 2 Not Available NO Not Available TUMOR FREE NO Complete Remission/Response Not Available NO Alive 2014 MALE Lung Adenocarcinoma- Not Otherwise Specified (NOS) No C34.1 8140/3 C34.1 YES 100 NO Not Available Not Available Unknown 12 Not Available Not Available Not Available Not Available Not Available Not Available Not Available Not Available Not Available NO Not Available Yes, History of Prior Malignancy A4N1 Preoperative Unknown Not Available Not Available NO 74 117 Complete Remission/Response YES BLACK OR AFRICAN AMERICAN NO NA R0 Not Applicable Not Applicable Not Applicable Not Applicable Not Applicable Not Applicable Not Applicable Not Applicable Not Applicable Stage IIA Not Applicable Not Applicable Not Applicable 7th Not Applicable Not Applicable Not Applicable M0 N1 T2a Not Available NO YES MN 2 Lung Alive 2012 2011 1967

139 Dead T4 N2 M0 Stage IIIB NA 65 L-Lower Not Applicable TCGA-62-8394 742e0e32-25b5-4d41-8bf5-364b571cfd93 5 -23758 139 0 Not Available Not Available Lung Adenocarcinoma Not Available NA TCGA-62-8394-D33506;TCGA-62-8394-D40459 66473842-A8EB-488E-BDE4-81A6B15B53B9;16DB8141-F66A-4A03-ABB6-CF51C96CC392 Not Available;Not Available 5;21 124;124 54;54 Carboplatin;Gemcitabine Unknown;Unknown 7;2 Not Available;Not Available Not Available;Not Available Not Available;Not Available Not Available;Not Available Not Applicable;Not Applicable Not Available;Not Available Not Available;Not Available NO;NO Chemotherapy;Chemotherapy Not Available;Not Available Not Available;Not Available Not Available;Not Available NO;NO 2012;2013 Unknown Not Available NO Not Available Not Available Not Available NO Not Available NOT HISPANIC OR LATINO NA FEMALE Lung Adenocarcinoma Mixed Subtype No C34.3 8255/3 C34.3 YES 100 NO Not Available Not Available Central Lung 7 Not Available Not Available Not Available Not Available Not Available Not Available Not Available Not Available Not Available NO Not Available No 8394 Preoperative TUMOR FREE Not Available Not Available YES 74 115 Complete Remission/Response YES WHITE NO NA R0 Not Applicable Not Applicable Not Applicable Not Applicable Not Applicable Not Applicable Not Applicable Not Applicable Not Applicable Stage IIIB Not Applicable Not Applicable Not Applicable 6th Not Applicable Not Applicable Not Applicable M0 N2 T4 Not Available NO YES 62 1 Lung Dead 2012 2008 Not Available

1171 Dead T2 N0 M0 Stage IB NA 68 L-Upper Not Applicable TCGA-78-7155 fc79086c-af9a-4c27-b074-f7050b6f3381 28 -24863 1171 0 Not Available Not Available Lung Adenocarcinoma Not Available NA TCGA-78-7155-D16959;TCGA-78-7155-D17202 b699c307-543d-4e31-9adf-19275a9cb2ff;6db2eeea-88ce-495f-b14b-214400fff219 Not Available;Not Available 21;5 Not Available;253 253;253 Carboplatin;Gemcitabine Not Available;Not Available 9;10 2;2 Not Available;Not Available Not Available;Not Available OTHER, SPECIFY IN NOTES;OTHER, SPECIFY IN NOTES new primary;New Primary 1;1 IV;IV NO;NO Chemotherapy;Chemotherapy Not Available;Not Available Not Available;Not Available Not Available;Not Available Not Available;Not Available 2011;2011 0 Not Available NO Not Available Not Available Not Available NO Not Available Not Available NA YES YES NO NO TCGA-78-7155-F16958 8ede53b4-10e1-4371-b89d-c4f2423cda1b 28 Not Available Not Available 1171 Not Available 209 Not Available Scheduled Follow-up Submission Progressive Disease Not Available Not Available 9 Not Available YES Not Available Not Available Not Available Not Available Not Available Not Available Dead 2011 MALE Lung Adenocarcinoma- Not Otherwise Specified (NOS) No C34.1 8140/3 C34.1 YES Not Available NO Not Available Not Available Peripheral Lung 9 Not Available Not Available Not Available Not Available Not Available Not Available Not Available Not Available Not Available Not Available 48 No 7155 Preoperative Not Available Not Available Not Available Not Available Not Available Not Available Not Available Not Available WHITE Not Available NA Primary Tumor Field TCGA-78-7155-R16960 d46f3ed7-d9f7-4800-9aff-2d7226581603 1 28 314 Not Available Not Available 9 Not Available Not Available NO EXTERNAL BEAM Not Applicable OTHER, SPECIFY IN NOTES New Primary Not Available 2011 R0 Not Applicable Not Applicable Not Applicable Not Applicable Not Applicable Not Applicable Not Applicable Not Applicable Not Applicable Stage IB Not Applicable Not Applicable Not Applicable 6th Not Applicable Not Applicable Not Applicable M0 N0 T2 2005 NO YES 78 4 Lung Dead 2011 2005 1957

607 Alive T2 N0 M0 Stage IB NA 68 L-Upper Not Applicable TCGA-05-4382 3434b91a-c05f-460f-a078-7b1bb6e7085d 22 -24868 Not Applicable 0 607 Not Available Lung Adenocarcinoma Not Available NA Not Available Not Available Not Available Not Available Not Available Not Available Not Available Not Available Not Available NA Unknown YES Not Available Not Available TCGA-05-4382-F36329 A8CCFEBD-82AA-41E1-85E0-75E97DEA2051 29 Not Available Not Available Not Applicable 607 334 Not Available Scheduled Follow-up Submission Complete Remission/Response Not Available NO 10 Distant Metastasis YES Not Available TUMOR FREE NO Complete Remission/Response Not Available YES Alive 2012 MALE Lung Adenocarcinoma Mixed Subtype No C34.1 8255/3 C34.1 YES Not Available Not Available Not Available Not Available Not Available 7 Not Available Not Available Not Available Not Available Not Available Not Available Not Available Not Available Not Available Not Available 62 Yes 4382 Not Available TUMOR FREE Not Available Not Available Not Available Not Available Not Available Not Available Not Available Not Available Not Available NA Distant Recurrence TCGA-05-4382-R36336 6AE3BFBC-DEA0-46D9-A3B6-1EF2CE28AA24 Not Available 29 393 365 Complete Response 10 Not Available Not Available NO External Not Applicable Not Available Not Available Gy 2012 R0 Not Applicable Not Applicable Not Applicable Not Applicable Not Applicable Not Applicable Not Applicable Not Applicable Not Applicable Stage IB Not Applicable Not Applicable Not Applicable 6th Not Applicable Not Applicable Not Applicable M0 N0 T2 Not Available NO YES 05 4 Lung Alive 2010 2009 Not Available

691 Alive T1b N0 MX Stage IA NA 70 L-Upper Not Applicable TCGA-44-7659 b86b1616-20a2-4300-8daa-03dcc9938215 30 -25730 Not Applicable 0 299 Not Available Lung Adenocarcinoma 43 NA Not Available Not Available Not Available Not Available Not Available Not Available Not Available Not Available NOT HISPANIC OR LATINO NA Not Available;Not Available Not Available;Not Available Not Available;NO Not Available;Not Available TCGA-44-7659-F30936;TCGA-44-7659-F39654 2f3b8b99-565e-4ad3-a8f0-edc89b22bd70;AD1BC6F4-A6B6-45BF-B2A2-C2EE5E33B07B 19;29 Not Applicable;Not Applicable Not Applicable;Not Applicable Not Applicable;Not Applicable 444;691 Not Applicable;Not Applicable Not Available;Not Available Scheduled Follow-up Submission;Scheduled Follow-up Submission Not Available;Complete Remission/Response Not Available;Not Available Not Available;NO 4;1 Not Available;Not Available NO;NO Not Available;Not Available TUMOR FREE;TUMOR FREE Not Available;NO Not Available;Complete Remission/Response Not Available;Not Available Not Available;NO Alive;Alive 2012;2013 MALE Lung Adenocarcinoma- Not Otherwise Specified (NOS) No C34.1 8140/3 C34.1 YES Not Available NO Not Available Not Available Not Available 12 Not Available Not Available Not Available Not Available Not Available Not Available Not Available Not Available Not Available Not Available 40 No 7659 Not Available TUMOR FREE 60 47 Not Available 55 42 Not Available YES WHITE Not Available NA Not Available Not Applicable Not Applicable Not Applicable Not Applicable Not Applicable Not Applicable Not Applicable Not Applicable Not Applicable Stage IA Not Applicable Not Applicable Not Applicable 7th Not Applicable Not Applicable Not Applicable MX N0 T1b Not Available YES NO 44 2 Lung Alive 2011 2010 1989

84 Alive T2a N1 M0 Stage IIA NA 67 R-Middle Not Applicable TCGA-95-8494 6ee9caec-65ac-46db-9426-ffaa31967ae2 30 -24773 Not Applicable 0 71 Not Available Lung Adenocarcinoma Not Available NA Not Available Not Available NO Not Available Not Available Not Available NO Not Available NOT HISPANIC OR LATINO NA Not Available Not Available Not Available Not Available TCGA-95-8494-F58476 5A2D5D46-74F5-48C7-8E7E-751B9C1F0BF3 10 Not Available Not Available Not Applicable 84 Not Available Not Available Scheduled Follow-up Submission Unknown Not Available YES 4 Not Available Unknown Not Available Not Available NO Complete Remission/Response Not Available NO Alive 2014 MALE Lung Adenocarcinoma- Not Otherwise Specified (NOS) No C34.2 8140/3 C34.2 YES Not Available NO Not Available Not Available Not Available 8 Not Available Not Available Not Available Not Available Not Available Not Available Not Available Not Available Not Available NO Not Available No 8494 Not Available Unknown Not Available Not Available NO Not Available Not Available Unknown NO WHITE NO NA Not Evaluated Not Applicable Not Applicable Not Applicable Not Applicable Not Applicable Not Applicable Not Applicable Not Applicable Not Applicable Stage IIA Not Applicable Not Applicable Not Applicable 7th Not Applicable Not Applicable Not Applicable M0 N1 T2a Not Available YES NO 95 5 Lung Alive 2012 2012 Not Available

411 Alive T1b N0 M0 Stage I NA 70 R-Upper Not Applicable TCGA-69-7980 aee86a89-0377-4080-b16c-408bfbe78687 9 -25583 Not Applicable 0 43 Not Available Lung Adenocarcinoma 71 NA Not Available Not Available YES Other Not Available Not Available NO Not Available NOT HISPANIC OR LATINO NA Not Available;Not Available Not Available;Not Available NO;Not Available Not Available;Not Available TCGA-69-7980-F39916;TCGA-69-7980-F57659 B9570D2D-FF75-4067-8D99-C36BCD332AC0;B3F0B9EB-890D-418B-9A53-A3F0240BB33D 11;19 Not Applicable;Not Applicable Not Applicable;Not Applicable Not Applicable;Not Applicable 411;362 Not Applicable;Not Applicable Not Evaluated;Not Available Scheduled Follow-up Submission;Scheduled Follow-up Submission Unknown;Not Applicable Not Evaluated;Not Available NO;NO 2;3 Not Available;Not Available NO;NO Not Evaluated;Not Available TUMOR FREE;TUMOR FREE NO;NO Complete Remission/Response;Complete Remission/Response Not Available;Not Available NO;NO Alive;Alive 2013;2014 FEMALE Lung Adenocarcinoma- Not Otherwise Specified (NOS) No C34.1 8550/3 C34.1 YES Not Available NO Not Available Not Available Not Available 4 Not Available Not Available Not Available Not Available Not Available Not Available Not Available Not Available Not Available Not Available 80 No 7980 Not Available TUMOR FREE Not Available Not Available Not Available 86 92 Not Available YES WHITE Not Available NA Not Available Not Applicable Not Applicable Not Applicable Not Applicable Not Applicable Not Applicable Not Applicable Not Applicable Not Applicable Stage I Not Applicable Not Applicable Not Applicable 7th Not Applicable Not Applicable Not Applicable M0 N0 T1b 2001 YES NO 69 4 Lung Alive 2012 2011 Not Available

896 Dead T2 N1 M0 Stage IIB NA 51 L-Upper Not Applicable TCGA-49-4510 f77b6930-a1ce-446e-a15d-c018cbbecfee 3 -18762 896 0 Not Available Not Available Lung Adenocarcinoma Not Available NA 1 Not Available Not Available Not Available Not Available Not Available Not Available Not Available NOT HISPANIC OR LATINO NA YES NO NO NO TCGA-49-4510-F58842 7F6D5AAD-0EEC-452F-9AEC-D99D92CD3A3E 25 Not Available Not Available 896 Not Available 502 1 Scheduled Follow-up Submission Stable Disease 80 NO 4 Distant Metastasis YES Post-Adjuvant Therapy WITH TUMOR YES Stable Disease Convincing Imaging YES Dead 2014 FEMALE Lung Adenocarcinoma- Not Otherwise Specified (NOS) No C34.1 8140/3 C34.1 YES Not Available Not Available Not Available Not Available Not Available 2 Not Available Not Available Not Available Not Available Not Available Not Available Not Available Not Available Not Available Not Available 55.5 No 4510 Pre-Adjuvant Therapy WITH TUMOR Not Available Not Available Not Available Not Available Not Available Not Available NO BLACK OR AFRICAN AMERICAN Not Available NA R0 Not Applicable Not Applicable Not Applicable Not Applicable Not Applicable Not Applicable Not Applicable Not Applicable Not Applicable Stage IIB Not Applicable Not Applicable Not Applicable 5th Not Applicable Not Applicable Not Applicable M0 N1 T2 Not Available NO YES 49 2 Lung Dead 2011 2001 Not Available

658 Alive T1 N2 M0 Stage IIIA NA 56 L-Lower Not Applicable TCGA-44-6774 33c71ead-4c2a-476d-85ef-885dbbfc2cbd 24 -20462 Not Applicable 0 166 Not Available Lung Adenocarcinoma 101 NA TCGA-44-6774-D30170;TCGA-44-6774-D30176;TCGA-44-6774-D30173 0AF8AD7E-12CE-4B54-A0AD-E385FCB7BFDF;43806307-3701-4257-911D-EA7D9340D5DE;DB768637-FE0C-46B2-8620-1FA1BE5C429A Not Available;Not Available;Not Available 3;3;3 154;154;112 91;133;91 Alimta;Carboplatin;Cisplatin Not Available;Not Available;Not Available 4;4;4 4;2;2 1000;525-560;150 mg;mg;mg ADJUVANT;ADJUVANT;ADJUVANT Not Applicable;Not Applicable;Not Applicable 1;1;1 IV;IV;IV NO;NO;NO Chemotherapy;Chemotherapy;Chemotherapy Not Available;Not Available;Not Available 4000;1085;300 mg;mg;mg Not Available;Not Available;Not Available 2012;2012;2012 1 Not Available Not Available Not Available Not Available Not Available Not Available Not Available NOT HISPANIC OR LATINO NA Not Available;Not Available Not Available;Not Available Not Available;NO Not Available;Not Available TCGA-44-6774-F30164;TCGA-44-6774-F39646 E1541BC7-F0F2-4081-85B4-DE781B1C27EC;F8E892DC-9234-421F-94F8-256BFAAE6DF6 3;28 Not Applicable;Not Applicable Not Applicable;Not Applicable Not Applicable;Not Applicable 361;658 Not Applicable;Not Applicable 1;Not Available Scheduled Follow-up Submission;Scheduled Follow-up Submission Not Available;Complete Remission/Response Not Available;Not Available Not Available;NO 4;1 Not Available;Not Available NO;NO Pre-Adjuvant Therapy;Not Available TUMOR FREE;TUMOR FREE YES;YES Complete Remission/Response;Complete Remission/Response Not Available;Not Available YES;YES Alive;Alive 2012;2013 FEMALE Lung Adenocarcinoma- Not Otherwise Specified (NOS) No C34.3 8140/3 C34.3 YES Not Available Not Available Not Available Not Available Not Available 8 Not Available Not Available Not Available Not Available Not Available Not Available Not Available Not Available Not Available Not Available 20 No 6774 Other Not Available Not Available Not Available Not Available 84 96 Not Available YES WHITE Not Available NA Primary Tumor Field TCGA-44-6774-R30167 F2186547-7A4A-4F49-9913-F70E94CF7A3E 1 3 228 193 Not Available 4 25 5000 NO EXTERNAL BEAM Not Applicable ADJUVANT Not Available cGy 2012 Not Available Not Applicable Not Applicable Not Applicable Not Applicable Not Applicable Not Applicable Not Applicable Not Applicable Not Applicable Stage IIIA Not Applicable Not Applicable Not Applicable 7th Not Applicable Not Applicable Not Applicable M0 N2 T1 Not Available YES NO 44 2 Lung Alive 2011 2011 1971

557 Dead T2a N0 M0 Stage IB NA 69 R-Lower Not Applicable TCGA-44-7661 cbfef004-b437-4d51-9d88-a2db50aa6481 17 -25483 Not Applicable 0 153 Not Available Lung Adenocarcinoma Not Available NA 0 Not Available YES Not Available Not Available Not Available YES Not Available NOT HISPANIC OR LATINO NA YES;YES YES;YES NO;NO Not Available;NO TCGA-44-7661-F33227;TCGA-44-7661-F39321 11a48e0b-2787-45f8-8a4d-920b7298072f;6C7C6AA4-9911-4736-9167-EAB29F024AEB 25;17 Not Available;Not Available Not Available;Not Available Not Applicable;557 366;Not Available 335;335 Not Available;Not Available Scheduled Follow-up Submission;Scheduled Follow-up Submission Progressive Disease;Progressive Disease Not Available;Not Available NO;NO 6;1 Locoregional Recurrence;Locoregional Recurrence YES;YES Not Available;Not Available WITH TUMOR;WITH TUMOR NO;NO Complete Remission/Response;Complete Remission/Response Convincing Imaging;Convincing Imaging NO;NO Alive;Dead 2012;2013 FEMALE Lung Adenocarcinoma- Not Otherwise Specified (NOS) No C34.3 8140/3 C34.3 YES Not Available Not Available Not Available Not Available Peripheral Lung 1 Not Available Not Available Not Available Not Available Not Available Not Available Not Available Not Available Not Available Not Available 22 Yes 7661 Preoperative TUMOR FREE 95 81 Not Available 92 68 Not Available YES WHITE Not Available NA Not Available Not Applicable Not Applicable Not Applicable Not Applicable Not Applicable Not Applicable Not Applicable Not Applicable Not Applicable Stage IB Not Applicable Not Applicable Not Applicable 7th Not Applicable Not Applicable Not Applicable M0 N0 T2a 1976 YES NO 44 3 Lung Alive 2012 2011 1961

2027 Dead T2 N1 M0 Stage IIB NA Not Available R-Upper Not Applicable TCGA-75-5125 205759a6-6391-491b-9857-0080c3a5871e 31 Not Available Not Applicable Not Available Not Available Not Available Lung Adenocarcinoma Not Available NA Not Available Not Available NO Not Available Not Available Not Available NO Not Available Not Available NA Not Available;NO Not Available;YES Not Available;NO Not Available;Not Available TCGA-75-5125-F11748;TCGA-75-5125-F72028 406f25c2-f2f7-4aa9-9016-034a5798f561;6219D889-CA6E-4935-8D96-082E561B630B 31;2 Not Applicable;Not Available Not Applicable;Not Available Not Applicable;2027 Not Available;Not Available Not Applicable;1752 Not Available;Unknown Not Available;Scheduled Follow-up Submission Complete Remission/Response;Progressive Disease Not Available;Not Evaluated Not Available;NO 3;4 Not Available;Not Available NO;YES Adjuvant therapy;Not Available TUMOR FREE;WITH TUMOR NO;NO Complete Remission/Response;Complete Remission/Response Not Available;Not Available NO;NO Alive;Dead 2011;2015 MALE Lung Adenocarcinoma- Not Otherwise Specified (NOS) No C34.1 8140/3 C34.1 YES Not Available NO Not Available Not Available Peripheral Lung 3 Not Available Not Available Not Available Not Available Not Available Not Available Not Available Not Available Not Available Not Available 25 No 5125 Other TUMOR FREE Not Available Not Available Not Available Not Available Not Available Not Available Not Available Not Available Not Available NA R0 Not Applicable Not Applicable Not Applicable Not Applicable Not Applicable Not Applicable Not Applicable Not Applicable Not Applicable Stage IIB Not Applicable Not Applicable Not Applicable 6th Not Applicable Not Applicable Not Applicable M0 N1 T2 1987 NO YES 75 3 Lung Alive 2011 Not Available Not Available

NA T2 N2 M0 Stage IIIA NA Not Available L-Lower Not Applicable TCGA-75-6203 a8d6694c-a213-4544-ac0b-63bce16d8f4e 15 Not Available Not Applicable Not Available Not Available Not Available Lung Adenocarcinoma Not Available NA TCGA-75-6203-D15865;TCGA-75-6203-D15864;TCGA-75-6203-D15863 a50cc582-dab3-4a3c-b8e7-b5cf188baf9a;51cbf34c-bb80-4826-8d78-451bc17e2701;ebfe876c-5580-4fbe-9edb-8749e6f6a5bd Not Available;Not Available;Not Available 17;15;15 Not Available;Not Available;Not Available Not Available;Not Available;Not Available Not Available;Cisplatin;Navelbine Not Available;Not Available;Not Available 8;8;8 Not Available;04;8 Not Available;Not Available;Not Available Not Available;Not Available;Not Available Not Available;ADJUVANT;ADJUVANT Not Applicable;Not Applicable;Not Applicable 2;1;1 Not Available;IV;IV YES;NO;NO Not Available;Chemotherapy;Chemotherapy Not Available;Not Available;Not Available Not Available;Not Available;Not Available Not Available;Not Available;Not Available Not Available;Not Available;Not Available 2010;2011;2011 Not Available Not Available Not Available Not Available Not Available Not Available Not Available Not Available Not Available NA Not Available Not Available Not Available Not Available TCGA-75-6203-F15862 54091978-b5ea-4fa4-b94e-a707f5389de7 15 Not Available Not Available Not Applicable Not Available Not Available Not Available Not Available Complete Remission/Response Not Available Not Available 8 Not Available Not Available Not Available Not Available YES Complete Remission/Response Not Available NO Alive 2011 FEMALE Lung Adenocarcinoma- Not Otherwise Specified (NOS) No C34.3 8140/3 C34.3 YES Not Available Not Available Not Available Not Available Not Available 8 Not Available Not Available Not Available Not Available Not Available Not Available Not Available Not Available Not Available Not Available Not Available No 6203 Not Available TUMOR FREE Not Available Not Available Not Available Not Available Not Available Not Available Not Available Not Available Not Available NA R0 Not Applicable Not Applicable Not Applicable Not Applicable Not Applicable Not Applicable Not Applicable Not Applicable Not Applicable Stage IIIA Not Applicable Not Applicable Not Applicable 6th Not Applicable Not Applicable Not Applicable M0 N2 T2 Not Available NO YES 75 1 Lung Alive 2011 2008 Not Available

1265 Dead T2 N2 MX Stage IIIA NA 54 L-Upper Not Applicable TCGA-MP-A4T9 70081320-540F-41D2-8687-EE6D011F8EB0 2 -19767 1265 0 Not Available Not Available Lung Adenocarcinoma 106 NA TCGA-MP-A4T9-D41350;TCGA-MP-A4T9-D41351;TCGA-MP-A4T9-D41352 C9F75A26-D387-48A4-BA06-A9D5C58CA315;758F08E1-B373-4964-9966-691B1D562B6C;ECBC2354-5C73-46FD-94A7-D519D192334F Not Available;Not Available;Not Available 15;15;15 55;55;62 34;34;62 Vinorelbine;Cisplatin;Gemcitabine Complete Response;Complete Response;Complete Response 3;3;3 Not Available;Not Available;Not Available Not Available;Not Available;Not Available Not Available;Not Available;Not Available Not Available;Not Available;Not Available Not Applicable;Not Applicable;Not Applicable Not Available;Not Available;Not Available Not Available;Not Available;Not Available NO;NO;NO Chemotherapy;Chemotherapy;Chemotherapy Not Available;Not Available;Not Available Not Available;Not Available;Not Available Not Available;Not Available;Not Available NO;NO;NO 2013;2013;2013 Unknown Not Available NO Not Available Not Available Not Available NO Not Available NOT HISPANIC OR LATINO NA YES;YES;NO YES;YES;NO Not Available;Not Available;YES YES;NO;Not Available TCGA-MP-A4T9-F41355;TCGA-MP-A4T9-F41356;TCGA-MP-A4T9-F41357 714B05C6-BB16-44AD-8A30-BA165B380950;CF943926-D77C-4F79-B844-7E46E5029D58;5FB23630-A7A5-4A7D-9CF1-AFE2A8EBD806 15;15;15 Not Available;Not Available;1255 938;Not Available;Not Available 1265;1265;1265 Not Available;Not Available;Not Available 418;889;1248 Not Evaluated;Not Evaluated;Not Evaluated Additional New Tumor Event;Additional New Tumor Event;Additional New Tumor Event Progressive Disease;Partial Remission/Response;Progressive Disease Not Evaluated;Not Evaluated;Not Evaluated NO;NO;NO 3;3;3 Distant Metastasis;Distant Metastasis;Locoregional Recurrence YES;YES;YES Not Evaluated;Not Evaluated;Not Evaluated WITH TUMOR;WITH TUMOR;WITH TUMOR YES;YES;YES Complete Remission/Response;Complete Remission/Response;Complete Remission/Response Biopsy with Histologic Confirmation;Convincing Imaging;Biopsy with Histologic Confirmation YES;YES;YES Dead;Dead;Dead 2013;2013;2013 FEMALE Lung Adenocarcinoma- Not Otherwise Specified (NOS) No C34.1 8140/3 C34.1 YES Not Evaluated NO Not Available Not Available Unknown 4 NO YES 341 NO Not Available NO Not Available Locoregional Recurrence;Distant Metastasis Convincing Imaging YES 24 No A4T9 Unknown WITH TUMOR Not Available 105 YES Not Available 99 Complete Remission/Response YES WHITE YES NA Primary Tumor Field TCGA-MP-A4T9-R41353 8CE9F14D-8BF0-438D-94D0-89396D078E74 Not Available 15 166 132 Complete Response 3 25 5000 NO External Not Applicable Not Available Not Available cGy 2013 RX Not Applicable Not Applicable Not Applicable Not Applicable Not Applicable Not Applicable Not Applicable Not Applicable Not Applicable Stage IIIA Not Applicable Not Applicable Not Applicable 6th Not Applicable Not Applicable Not Applicable MX N2 T2 2004 NO YES MP 4 Lung Dead 2013 2006 1980

414 Alive T1b N0 M0 Stage IA NA 75 R-Upper Not Applicable TCGA-69-7764 243c6fd6-5516-46e5-bcef-292d93d7b7d3 26 -27693 Not Applicable 0 414 Not Available Lung Adenocarcinoma Not Available NA 0 Not Available YES Not Available Not Available Not Available NO Not Available NOT HISPANIC OR LATINO NA Not Available Not Available Not Available Not Available TCGA-69-7764-F20992 7523c29c-28ca-4dcc-8407-7545d04f3af4 26 Not Applicable Not Applicable Not Applicable 414 Not Applicable 0 Scheduled Follow-up Submission Not Available Not Available Not Available 1 Not Available NO Other TUMOR FREE NO Not Available Not Available NO Alive 2012 MALE Lung Adenocarcinoma Mixed Subtype No C34.1 8255/3 C34.1 YES Not Available NO Not Available Not Available Peripheral Lung 1 Not Available Not Available Not Available Not Available Not Available Not Available Not Available Not Available Not Available Not Available 30 No 7764 Other Not Available Not Available Not Available Not Available 94 106 Not Available YES WHITE Not Available NA Not Available Not Applicable Not Applicable Not Applicable Not Applicable Not Applicable Not Applicable Not Applicable Not Applicable Not Applicable Stage IA Not Applicable Not Applicable Not Applicable 7th Not Applicable Not Applicable Not Applicable M0 N0 T1b 1991 NO YES 69 3 Lung Alive 2012 2010 1977

1442 Alive T2 N0 M0 Stage IB NA 72 R-Upper Not Applicable TCGA-50-5066 3199cfe5-3be3-43cd-a36b-5cf2c7a9929f 2 -26385 Not Applicable 0 297 Not Available Lung Adenocarcinoma Not Available NA TCGA-50-5066-D32029;TCGA-50-5066-D32030;TCGA-50-5066-D32031 5be0e05b-35a4-4283-90e3-38874324ed7a;ad7d0412-3694-4490-8268-f0191cbe615c;1ac70461-ad8a-48f2-b045-ce88d310315b Not Available;Not Available;Not Available 15;15;15 567;567;567 508;508;508 Carboplatin;Taxol;Avastin Stable Disease;Stable Disease;Stable Disease 5;5;5 Not Available;Not Available;Not Available Not Available;Not Available;Not Available Not Available;Not Available;Not Available Not Available;Not Available;Not Available Not Applicable;Not Applicable;Not Applicable Not Available;Not Available;Not Available Not Available;Not Available;Not Available NO;NO;NO Chemotherapy;Chemotherapy;Chemotherapy Not Available;Not Available;Not Available Not Available;Not Available;Not Available Not Available;Not Available;Not Available NO;NO;NO 2012;2012;2012 Not Available Not Available YES Not Available Not Available Not Available YES Not Available NOT HISPANIC OR LATINO NA YES;Not Available NO;Not Available NO;Not Available YES;Not Available TCGA-50-5066-F32028;TCGA-50-5066-F70450 e8fd3201-f957-4c6e-9942-c2fc7699fcd8;AB6C6622-1AE5-480A-A3DB-CB949ED02E15 15;16 Not Available;Not Applicable 598;Not Applicable Not Applicable;Not Applicable 944;1442 477;Not Applicable Not Available;Not Available Scheduled Follow-up Submission;Scheduled Follow-up Submission Progressive Disease;Complete Remission/Response Not Available;Not Available NO;NO 5;2 Distant Metastasis;Not Available YES;NO Not Available;Not Available WITH TUMOR;TUMOR FREE YES;YES Complete Remission/Response;Complete Remission/Response Not Available;Not Available NO;NO Alive;Alive 2012;2015 MALE Lung Adenocarcinoma Mixed Subtype No C34.1 8255/3 C34.1 YES Not Available YES NO Not Available Not Available 7 Not Available Not Available Not Available Not Available Not Available Not Available Not Available Not Available Not Available Not Available Not Available No 5066 Not Available Not Available Not Available Not Available Not Available Not Available Not Available Not Available NO BLACK OR AFRICAN AMERICAN Not Available NA R0 Not Applicable Not Applicable Not Applicable Not Applicable Not Applicable Not Applicable Not Applicable Not Applicable Not Applicable Stage IB Not Applicable Not Applicable Not Applicable 6th Not Applicable Not Applicable Not Applicable M0 N0 T2 Not Available NO YES 50 1 Lung Alive 2011 2009 Not Available

423 Alive T1b N0 M0 Stage IA NA 61 L-Lower Not Applicable TCGA-86-8668 f2d8b332-2f03-46e5-a9d5-f163972e5fa7 30 -22383 Not Applicable 0 45 Not Available Lung Adenocarcinoma Not Available NA 0 Not Available Not Available Not Available Not Available Not Available Not Available Not Available NOT HISPANIC OR LATINO NA Not Available Not Available Not Available Not Available TCGA-86-8668-F66084 1631B6D9-406C-4FFC-BC60-4B3233895638 2 Not Applicable Not Applicable Not Applicable 423 Not Applicable 0 Scheduled Follow-up Submission Complete Remission/Response 100 NO 10 Not Available NO Other TUMOR FREE NO Complete Remission/Response Not Available NO Alive 2014 FEMALE Lung Bronchioloalveolar Carcinoma Mucinous No C34.3 8253/3 C34.3 YES 100 NO Not Available Not Available Peripheral Lung 8 Not Available Not Available Not Available Not Available Not Available Not Available Not Available Not Available Not Available Unknown Not Available No 8668 Preoperative TUMOR FREE Not Available Not Available Unknown Not Available Not Available Complete Remission/Response NO WHITE Unknown NA R0 Not Applicable Not Applicable Not Applicable Not Applicable Not Applicable Not Applicable Not Applicable Not Applicable Not Applicable Stage IA Not Applicable Not Applicable Not Applicable 7th Not Applicable Not Applicable Not Applicable M0 N0 T1b Not Available YES NO 86 1 Lung Alive 2012 2012 Not Available

505 Alive T2a NX MX Stage IB NA 63 L-Lower Not Applicable TCGA-91-8496 656a5eb4-e4a5-4d21-a800-3586f4d6588b 23 -23358 Not Applicable 0 197 Not Available Lung Adenocarcinoma Not Available NA 0 Not Available NO Not Available Not Available Not Available NO Not Available NOT HISPANIC OR LATINO NA Not Available Not Available Not Available Not Available TCGA-91-8496-F45467 7004F428-D84D-4816-B359-2D998F95C44B 15 Not Applicable Not Applicable Not Applicable 505 Not Applicable Not Evaluated Scheduled Follow-up Submission Complete Remission/Response Not Evaluated NO 7 Not Available NO Not Available TUMOR FREE NO Complete Remission/Response Not Available NO Alive 2013 FEMALE Lung Bronchioloalveolar Carcinoma Nonmucinous No C34.3 8252/3 C34.3 YES Not Evaluated NO Not Available Not Available Unknown 8 Not Available Not Available Not Available Not Available Not Available Not Available Not Available Not Available Not Available NO Not Available No 8496 Preoperative Unknown Not Available Not Available NO Not Available Not Available Unknown NO WHITE NO NA Not Evaluated Not Applicable Not Applicable Not Applicable Not Applicable Not Applicable Not Applicable Not Applicable Not Applicable Not Applicable Stage IB Not Applicable Not Applicable Not Applicable 7th Not Applicable Not Applicable Not Applicable MX NX T2a Not Available YES NO 91 1 Lung Alive 2012 2011 Not Available

2137 Alive T1 N0 M0 Stage IA NA 77 L-Lower Not Applicable TCGA-55-6987 2efc156c-464a-4c2f-a854-39fefe32081c 27 -28382 Not Applicable 0 1170 Not Available Lung Adenocarcinoma Not Available NA Not Available Not Available NO Not Available Not Available Not Available NO Not Available NOT HISPANIC OR LATINO NA Not Available Not Available Not Available Not Available TCGA-55-6987-F47803 18F46E93-2092-4031-B41F-BDDD78B2223D 28 Not Applicable Not Applicable Not Applicable 2137 Not Applicable Not Evaluated Scheduled Follow-up Submission Complete Remission/Response Not Evaluated NO 8 Not Available NO Not Evaluated TUMOR FREE NO Complete Remission/Response Not Available NO Alive 2013 MALE Lung Adenocarcinoma- Not Otherwise Specified (NOS) No C34.3 8140/3 C34.3 YES Not Available NO Not Available Not Available Not Available 7 Not Available Not Available Not Available Not Available Not Available Not Available Not Available Not Available Not Available Not Available Not Available No 6987 Not Available TUMOR FREE Not Available Not Available Not Available Not Available Not Available Not Available Not Available WHITE Not Available NA Not Available Not Applicable Not Applicable Not Applicable Not Applicable Not Applicable Not Applicable Not Applicable Not Applicable Not Applicable Stage IA Not Applicable Not Applicable Not Applicable 6th Not Applicable Not Applicable Not Applicable M0 N0 T1 2006 NO YES 55 4 Lung Alive 2011 2006 Not Available

448 Alive T3 N0 M0 NA 69 R-Lower Not Applicable TCGA-67-4679 3e635902-5310-4406-8b4d-a238ce382639 21 -25202 Not Applicable 0 448 Not Available Lung Adenocarcinoma Not Available NA Not Available Not Available NO Not Available Not Available Not Available NO Not Available NOT HISPANIC OR LATINO NA MALE Lung Adenocarcinoma- Not Otherwise Specified (NOS) No C34.3 8140/3 C34.3 YES Not Available NO Not Available Not Available Central Lung 1 Not Available Not Available Not Available Not Available Not Available Not Available Not Available Not Available Not Available Not Available Not Available No 4679 Not Available TUMOR FREE Not Available Not Available Not Available Not Available Not Available Not Available Not Available WHITE Not Available NA R0 Not Applicable Not Applicable Not Applicable Not Applicable Not Applicable Not Applicable Not Applicable Not Applicable Not Applicable Discrepancy Not Applicable Not Applicable Not Applicable Not Available Not Applicable Not Applicable Not Applicable M0 N0 T3 Not Available YES NO 67 4 Lung Alive 2011 2009 Not Available

513 Alive T1b N0 M0 Stage IA NA 68 R-Upper Not Applicable TCGA-S2-AA1A 31458638-E19C-43E5-AB13-9C64B3B3681D 22 -24903 Not Applicable 0 121 Not Available Lung Adenocarcinoma 65 NA Not Evaluated Not Available NO Not Available Not Available Not Available NO Not Available NOT HISPANIC OR LATINO NA Not Available Not Available Not Available Not Available TCGA-S2-AA1A-F67520 90F720D7-868B-4102-9B73-0824B7500158 7 Not Applicable Not Applicable Not Applicable 513 Not Applicable Not Evaluated Scheduled Follow-up Submission Complete Remission/Response Not Evaluated NO 11 Not Available NO Not Available TUMOR FREE NO Complete Remission/Response Not Available NO Alive 2014 FEMALE Lung Bronchioloalveolar Carcinoma Mucinous No C34.1 8253/3 C34.1 YES Not Evaluated NO Not Available Not Available Not Available 4 Not Available Not Available Not Available Not Available Not Available Not Available Not Available Not Available Not Available NO 95 No AA1A Not Available TUMOR FREE 83 2.16 NO 79 2.01 Complete Remission/Response YES BLACK OR AFRICAN AMERICAN NO NA R0 Not Applicable Not Applicable Not Applicable Not Applicable Not Applicable Not Applicable Not Applicable Not Applicable Not Applicable Stage IA Not Applicable Not Applicable Not Applicable 7th Not Applicable Not Applicable Not Applicable M0 N0 T1b 1995 YES NO S2 3 Lung Alive 2014 2013 1957

2515 Alive T1 N0 M0 Stage IA NA 65 L-Upper Not Applicable TCGA-73-4662 294ff941-aea1-4588-9a0e-e9f5393e2bb6 6 -23762 Not Applicable 0 912 Not Available Lung Adenocarcinoma 116 NA 0 Not Available NO Not Available Not Available Not Available NO Not Available NOT HISPANIC OR LATINO NA YES;Not Available YES;Not Available YES;Not Available NO;Not Available TCGA-73-4662-F8554;TCGA-73-4662-F71029 7772d756-649c-41b9-85f1-b38aa3c4cdea;FFBDD995-8D42-4D31-AB72-91A28CBECA08 6;18 36;Not Applicable Not Available;Not Applicable Not Applicable;Not Applicable 912;2515 21;Not Applicable 0;Not Evaluated Not Available;Scheduled Follow-up Submission Complete Remission/Response;Complete Remission/Response 100;Not Evaluated Not Available;NO 1;3 Not Available;Not Available YES;NO Adjuvant therapy;Not Evaluated TUMOR FREE;TUMOR FREE NO;NO Complete Remission/Response;Complete Remission/Response Not Available;Not Available NO;NO Alive;Alive 2011;2015 FEMALE Lung Adenocarcinoma- Not Otherwise Specified (NOS) No C34.1 8140/3 C34.1 YES 100 NO Not Available Not Available Peripheral Lung 1 Not Available Not Available Not Available Not Available Not Available Not Available Not Available Not Available Not Available Not Available 10 No 4662 Preoperative TUMOR FREE Not Available Not Available Not Available 115 111 Not Available YES WHITE Not Available NA R0 Not Applicable Not Applicable Not Applicable Not Applicable Not Applicable Not Applicable Not Applicable Not Applicable Not Applicable Stage IA Not Applicable Not Applicable Not Applicable 6th Not Applicable Not Applicable Not Applicable M0 N0 T1 1972 NO YES 73 3 Lung Alive 2011 2007 1955

573 Alive T2 N0 M0 Stage IB NA 82 R-Upper Not Applicable TCGA-67-3772 673493f6-975c-49e8-934c-001e9a0fff90 3 -29989 Not Applicable 0 573 Not Available Lung Adenocarcinoma Not Available NA Not Available Not Available NO Not Available Not Available Not Available NO Not Available NOT HISPANIC OR LATINO NA FEMALE Lung Adenocarcinoma- Not Otherwise Specified (NOS) No C34.1 8140/3 C34.1 YES Not Available NO Not Available Not Available Not Available 3 Not Available Not Available Not Available Not Available Not Available Not Available Not Available Not Available Not Available Not Available Not Available No 3772 Not Available TUMOR FREE Not Available Not Available Not Available Not Available Not Available Not Available Not Available WHITE Not Available NA Not Available Not Applicable Not Applicable Not Applicable Not Applicable Not Applicable Not Applicable Not Applicable Not Applicable Not Applicable Stage IB Not Applicable Not Applicable Not Applicable 6th Not Applicable Not Applicable Not Applicable M0 N0 T2 Not Available YES NO 67 1 Lung Alive 2011 2009 Not Available

591 Alive T3 N0 M0 Stage IIB NA 60 R-Upper Not Applicable TCGA-69-A59K 67FA3E41-C7C6-44C5-9E67-6BCB2715AACC 18 -22093 Not Applicable 0 214 Not Available Lung Adenocarcinoma 75 NA TCGA-69-A59K-D40359;TCGA-69-A59K-D40360 095F50E9-408C-4353-B7D6-339A2BBC1895;EE6D5CA4-FDD0-45B6-A767-D5B34DE311ED Not Available;Not Available 18;18 135;135 72;72 cisplatin;pemetrexed Stable Disease;Stable Disease 2;2 Not Available;Not Available Not Available;Not Available Not Available;Not Available Not Available;Not Available Not Applicable;Not Applicable Not Available;Not Available Not Available;Not Available NO;NO Chemotherapy;Chemotherapy Not Available;Not Available Not Available;Not Available Not Available;Not Available NO;NO 2013;2013 2 Not Available YES Not Available Not Available Not Available NO Not Available NOT HISPANIC OR LATINO NA Not Available;Not Available Not Available;Not Available Not Available;Not Available Not Available;Not Available TCGA-69-A59K-F56712;TCGA-69-A59K-F57660 5E0B884C-C335-4788-8FFF-1F4CF6565627;6D298071-0FB0-40B6-A0D9-43F6B40F136C 19;19 Not Applicable;Not Applicable Not Applicable;Not Applicable Not Applicable;Not Applicable 591;522 Not Applicable;Not Applicable Not Available;Not Available Scheduled Follow-up Submission;Scheduled Follow-up Submission Not Applicable;Not Applicable Not Available;Not Available NO;NO 2;3 Not Available;Not Available NO;NO Not Available;Not Available TUMOR FREE;TUMOR FREE YES;YES Stable Disease;Stable Disease Not Available;Not Available NO;NO Alive;Alive 2014;2014 FEMALE Lung Adenocarcinoma Mixed Subtype No C34.1 8255/3 C34.1 YES Not Available Unknown Not Available Not Available Unknown 2 Not Available Not Available Not Available Not Available Not Available Not Available Not Available Not Available Not Available NO 25 No A59K Pre-Adjuvant Therapy TUMOR FREE Not Available 120 YES Not Available 118 Stable Disease YES BLACK OR AFRICAN AMERICAN NO NA Not Evaluated Not Applicable Not Applicable Not Applicable Not Applicable Not Applicable Not Applicable Not Applicable Not Applicable Not Applicable Stage IIB Not Applicable Not Applicable Not Applicable 7th Not Applicable Not Applicable Not Applicable M0 N0 T3 2012 YES NO 69 4 Lung Alive 2013 2012 1999

922 Dead T3 N1 M0 Stage IIIA NA 59 L-Lower Not Applicable TCGA-73-4675 ae39e358-08d7-4367-ae68-82b469e791e4 7 -21614 Not Applicable 0 40 Not Available Lung Adenocarcinoma 69 NA TCGA-73-4675-D71056;TCGA-73-4675-D71057;TCGA-73-4675-D71058;TCGA-73-4675-D71059;TCGA-73-4675-D71060;TCGA-73-4675-D71061;TCGA-73-4675-D71062 13B8CCE4-8461-4A03-A567-E5922A9A11A4;3C9951FA-7567-4A17-8ED5-031F790060D7;25DC92F4-A154-4196-9D5F-4BE559E42ABA;1C265224-D738-4F25-8103-9B037F9C9447;DC1FC3CB-3680-4D2A-86C2-B9DBC184D6BC;08130C1E-5BB6-4080-939E-11D5E4215EEF;3BF6C8DC-1CB9-4686-987C-7DA51220B8A3 Not Available;Not Available;Not Available;Not Available;Not Available;Not Available;Not Available 17;17;17;17;17;17;17 482;117;104;483;483;483;676 110;27;27;459;459;459;592 Carboplatin;Alimta;Cisplatin;Carboplatin;Taxotere;Avastin;Tarceva Complete Response;Complete Response;Complete Response;Stable Disease;Stable Disease;Stable Disease;Clinical Progressive Disease 3;3;3;3;3;3;3 Not Available;Not Available;Not Available;Not Available;Not Available;Not Available;Not Available Not Available;Not Available;Not Available;Not Available;Not Available;Not Available;Not Available Not Available;Not Available;Not Available;Not Available;Not Available;Not Available;Not Available Not Available;Not Available;Not Available;Not Available;Not Available;Not Available;Not Available Not Applicable;Not Applicable;Not Applicable;Not Applicable;Not Applicable;Not Applicable;Not Applicable Not Available;Not Available;Not Available;Not Available;Not Available;Not Available;Not Available Not Available;Not Available;Not Available;Not Available;Not Available;Not Available;Not Available NO;NO;NO;NO;NO;NO;NO Chemotherapy;Chemotherapy;Chemotherapy;Chemotherapy;Chemotherapy;Chemotherapy;Chemotherapy Not Available;Not Available;Not Available;Not Available;Not Available;Not Available;Not Available Not Available;Not Available;Not Available;Not Available;Not Available;Not Available;Not Available Not Available;Not Available;Not Available;Not Available;Not Available;Not Available;Not Available NO;NO;NO;NO;NO;NO;NO 2015;2015;2015;2015;2015;2015;2015 0 Not Available NO Not Available Not Available Not Available NO Not Available NOT HISPANIC OR LATINO NA Not Available;YES Not Available;NO Not Available;NO Not Available;Not Available TCGA-73-4675-F8551;TCGA-73-4675-F71055 05da2e27-6b81-4a1f-887c-1a42d8bc1786;AC8B9FDB-A4DE-4944-94BF-6BB1CAF80003 7;17 Not Available;Not Available Not Available;Not Available Not Applicable;922 40;Not Available Not Available;361 0;Unknown Not Available;Scheduled Follow-up Submission Not Available;Progressive Disease Not Available;Unknown Not Available;NO 1;3 Not Available;Locoregional Recurrence Not Available;YES Pre-Adjuvant Therapy;Unknown TUMOR FREE;Unknown Not Available;YES Not Available;Progressive Disease Not Available;Convincing Imaging Not Available;NO Alive;Dead 2011;2015 MALE Lung Adenocarcinoma- Not Otherwise Specified (NOS) No C34.3 8140/3 C34.3 YES Not Available NO Not Available Not Available Peripheral Lung 1 Not Available Not Available Not Available Not Available Not Available Not Available Not Available Not Available Not Available Not Available 41 No 4675 Pre-Adjuvant Therapy TUMOR FREE Not Available Not Available Not Available 55 66 Not Available YES WHITE Not Available NA RX Not Applicable Not Applicable Not Applicable Not Applicable Not Applicable Not Applicable Not Applicable Not Applicable Not Applicable Stage IIIA Not Applicable Not Applicable Not Applicable 7th Not Applicable Not Applicable Not Applicable M0 N1 T3 2010 NO YES 73 4 Lung Alive 2011 2010 1969

592 Alive T2 N0 MX Stage IB NA 72 R-Upper Not Applicable TCGA-44-7660 a534b96b-1665-444a-9308-e610f2f0e510 22 -26594 Not Applicable 0 162 Not Available Lung Adenocarcinoma 80 NA TCGA-44-7660-D32371 452ec58f-55a9-4b79-9095-1800bc34f9a8 ASCI 24 141 78 recPRAME+AS15 ASCI Clinical Progressive Disease 5 Not Available Not Available Not Available Not Available Not Applicable Not Available Not Available NO Vaccine Not Available Not Available Not Available YES 2012 1 Not Available Not Available Not Available Not Available Not Available Not Available Not Available NOT HISPANIC OR LATINO NA NO;Not Available NO;Not Available NO;NO Not Available;Not Available TCGA-44-7660-F32361;TCGA-44-7660-F40409 57f9b0eb-17e0-4b6b-92a0-407511f014d9;4B16E925-6686-4721-B792-96761A62E44C 24;19 Not Available;Not Applicable Not Available;Not Applicable Not Applicable;Not Applicable 325;592 253;Not Applicable Not Available;Not Available Scheduled Follow-up Submission;Scheduled Follow-up Submission Progressive Disease;Stable Disease Not Available;70 NO;NO 5;2 Locoregional Recurrence;Not Available YES;NO Not Available;Other WITH TUMOR;WITH TUMOR YES;YES Progressive Disease;Progressive Disease Convincing Imaging;Not Available NO;NO Alive;Alive 2012;2013 MALE Lung Adenocarcinoma- Not Otherwise Specified (NOS) No C34.1 8140/3 C34.1 YES Not Available NO Not Available Not Available Not Available 12 Not Available Not Available Not Available Not Available Not Available Not Available Not Available Not Available Not Available Not Available 124 No 7660 Pre-Adjuvant Therapy TUMOR FREE 95 94 Not Available 92 83 Not Available YES WHITE Not Available NA Not Available Not Applicable Not Applicable Not Applicable Not Applicable Not Applicable Not Applicable Not Applicable Not Applicable Not Applicable Stage IB Not Applicable Not Applicable Not Applicable 7th Not Applicable Not Applicable Not Applicable MX N0 T2 Not Available YES NO 44 2 Lung Alive 2011 2011 1949

182 Alive T1 N0 M0 Stage IA NA 38 R-Upper Not Applicable TCGA-35-4123 6cf49cf0-de4c-4c90-8358-eae19c6206b0 20 -14064 Not Applicable 0 182 Not Available Lung Adenocarcinoma Not Available NA 1 Not Available NO Not Available Not Available Not Available NO Not Available NOT HISPANIC OR LATINO NA Not Available Not Available Not Available Not Available TCGA-35-4123-F68918 EC7AA234-A6E0-4907-8754-531418756764 23 Not Available Not Available Not Applicable Not Available Not Available Not Available Scheduled Follow-up Submission Not Applicable Not Available YES 12 Not Available Unknown Not Available Not Available Not Available Not Available Not Available Not Available Not Available 2014 MALE Lung Adenocarcinoma- Not Otherwise Specified (NOS) No C34.1 8140/3 C34.1 YES 90 NO Not Available Not Available Peripheral Lung 12 Not Available Not Available Not Available Not Available Not Available Not Available Not Available Not Available Not Available Not Available 20 No 4123 Preoperative TUMOR FREE Not Available Not Available Not Available Not Available Not Available Not Available YES WHITE Not Available NA R0 Not Applicable Not Applicable Not Applicable Not Applicable Not Applicable Not Applicable Not Applicable Not Applicable Not Applicable Stage IA Not Applicable Not Applicable Not Applicable 7th Not Applicable Not Applicable Not Applicable M0 N0 T1 2010 NO YES 35 4 Lung Alive 2010 2010 1990

442 Dead T1b N1 MX Stage IIA NA 75 L-Lower Not Applicable TCGA-L9-A50W A815619F-DBBA-469F-A1F7-DFBD51CD86C5 8 -27414 Not Applicable 0 266 Not Available Lung Adenocarcinoma Not Available NA 0 Not Available YES T790M Not Available FISH YES Not Available NOT HISPANIC OR LATINO NA NO NO NO NO TCGA-L9-A50W-F50396 F09EA740-220C-4B79-BDA9-418554DFD3D0 23 Not Available Not Available 442 Not Available 389 0 Scheduled Follow-up Submission Progressive Disease Not Available NO 10 Distant Metastasis YES Preoperative WITH TUMOR NO Stable Disease Biopsy with Histologic Confirmation;Convincing Imaging NO Dead 2013 MALE Lung Adenocarcinoma Mixed Subtype No C34.3 8255/3 C34.3 YES Not Evaluated NO Not Available Not Available Unknown 3 Not Available Not Available Not Available Not Available Not Available Not Available Not Available Not Available Not Available NO 19 No A50W Preoperative TUMOR FREE Not Available Not Available NO 103 81 Complete Remission/Response YES BLACK OR AFRICAN AMERICAN NO NA R0 Not Applicable Not Applicable Not Applicable Not Applicable Not Applicable Not Applicable Not Applicable Not Applicable Not Applicable Stage IIA Not Applicable Not Applicable Not Applicable 7th Not Applicable Not Applicable Not Applicable MX N1 T1b 1986 YES NO L9 3 Lung Alive 2013 2012 1967

904 Alive T1b N0 M0 Stage IA NA 64 L-Lower Not Applicable TCGA-55-8085 5fd0f07b-8bb9-4378-bd26-28a26057e8fe 15 -23596 Not Applicable 0 33 Not Available Lung Adenocarcinoma 51 NA 1 Not Available Unknown Not Available Not Available Not Available Unknown Not Available NOT HISPANIC OR LATINO NA Not Available Not Available Not Available Not Available TCGA-55-8085-F65697 CA62DF16-FCE4-47BE-8B87-6AC56FCECE60 26 Not Applicable Not Applicable Not Applicable 904 Not Applicable Not Available Scheduled Follow-up Submission Complete Remission/Response Not Available NO 9 Not Available NO Not Available TUMOR FREE NO Complete Remission/Response Not Available NO Alive 2014 MALE Lung Papillary Adenocarcinoma No C34.3 8260/3 C34.3 YES Not Available Unknown Not Available Not Available Not Available 6 Not Available Not Available Not Available Not Available Not Available Not Available Not Available Not Available Not Available NO Not Available No 8085 Not Available TUMOR FREE 46 45 Unknown 39 35 Complete Remission/Response YES WHITE Unknown NA R0 Not Applicable Not Applicable Not Applicable Not Applicable Not Applicable Not Applicable Not Applicable Not Applicable Not Applicable Stage IA Not Applicable Not Applicable Not Applicable 7th Not Applicable Not Applicable Not Applicable M0 N0 T1b Not Available YES NO 55 2 Lung Alive 2012 2011 Not Available

1209 Dead T3 N0 M0 Stage IIB NA 58 R-Upper Not Applicable TCGA-78-8648 5ace9608-d38a-42f2-a877-ec7c9d211808 23 -21443 1209 0 Not Available Not Available Lung Adenocarcinoma 69 NA 2 Not Available NO Not Available Not Available Not Available NO Not Available Not Evaluated NA FEMALE Lung Adenocarcinoma- Not Otherwise Specified (NOS) No C34.1 8140/3 C34.1 YES Not Available NO Not Available Not Available Peripheral Lung 10 Unknown Unknown 720 Unknown Not Available Not Available Not Available Locoregional Recurrence Biopsy with Histologic Confirmation YES 45 No 8648 Preoperative Unknown Not Available Not Available NO 83 70 Unknown YES Not Evaluated NO NA R0 Not Applicable Not Applicable Not Applicable Not Applicable Not Applicable Not Applicable Not Applicable Not Applicable Not Applicable Stage IIB Not Applicable Not Applicable Not Applicable 6th Not Applicable Not Applicable Not Applicable M0 N0 T3 Not Available NO YES 78 2 Lung Dead 2012 2002 1971

791 Alive T2b N0 M0 Stage IIA NA 75 R-Upper Not Applicable TCGA-78-7539 7be86a90-6da8-4c01-8a7a-bc5cfef9894f 19 -27445 Not Applicable 0 327 Not Available Lung Adenocarcinoma Not Available NA TCGA-78-7539-D20644;TCGA-78-7539-D20647;TCGA-78-7539-D20645;TCGA-78-7539-D20646 ee4766b1-b93f-47b2-8296-193336607673;ef32890a-c5fc-4abf-915c-3c0394c365a4;55910d2b-0159-402a-a7c3-dc40c6dd4fcd;0a178b02-54e3-4690-8a6b-a18c85384aa2 Not Available;Not Available;Not Available;Not Available 19;19;19;19 42;133;49;133 36;70;36;70 Cisplatin;Paclitaxel;Vinorelbine;Carboplatin Not Available;Not Available;Not Available;Not Available 1;1;1;1 1;4;1;4 75;262;37.5;284 mg;mg;mg;mg ADJUVANT;ADJUVANT;ADJUVANT;ADJUVANT Not Applicable;Not Applicable;Not Applicable;Not Applicable 1;2;1;2 IV;IV;IV;IV NO;NO;NO;NO Chemotherapy;Chemotherapy;Chemotherapy;Chemotherapy Not Available;Not Available;Not Available;Not Available 150;1048;112.5;1167 mg;mg;mg;mg Not Available;Not Available;Not Available;Not Available 2012;2012;2012;2012 1 Not Available NO Not Available Not Available Not Available NO Not Available Not Available NA Not Available;Unknown Not Available;YES Not Available;Not Available Not Available;YES TCGA-78-7539-F20643;TCGA-78-7539-F46143 e53bcc72-3116-4b4d-a4d9-712dab94cf72;73006682-67D7-4FC9-9CFE-3C60E6B3B825 19;4 Not Available;Not Available Not Available;688 Not Applicable;Not Applicable 327;791 Not Available;688 Not Available;Unknown Scheduled Follow-up Submission;Scheduled Follow-up Submission Not Available;Unknown Not Available;Not Available Not Available;NO 1;8 Not Available;Distant Metastasis Not Available;YES Not Available;Not Available Not Available;Unknown YES;YES Not Available;Complete Remission/Response Not Available;Biopsy with Histologic Confirmation NO;NO Alive;Alive 2012;2013 FEMALE Lung Adenocarcinoma Mixed Subtype No C34.1 8255/3 C34.1 YES Not Available NO Not Available Not Available Not Available 1 Not Available Not Available Not Available Not Available Not Available Not Available Not Available Not Available Not Available Not Available 2.2 No 7539 Preoperative Not Available Not Available Not Available Not Available Not Available Not Available Not Available NO WHITE Not Available NA R0 Not Applicable Not Applicable Not Applicable Not Applicable Not Applicable Not Applicable Not Applicable Not Applicable Not Applicable Stage IIA Not Applicable Not Applicable Not Applicable 7th Not Applicable Not Applicable Not Applicable M0 N0 T2b 1981 NO YES 78 3 Lung Alive 2012 2011 1953

1974 Alive T1 NX M0 Stage IA NA 60 L-Upper Not Applicable TCGA-78-7159 4d153600-81f9-44fb-b485-6355ec306d6a 27 -22261 Not Applicable 0 1221 Not Available Lung Adenocarcinoma Not Available NA 1 Not Available NO Not Available Not Available Not Available NO Not Available Not Available NA Not Available;Not Available Not Available;Not Available Not Available;Not Available Not Available;Not Available TCGA-78-7159-F16828;TCGA-78-7159-F46103 9d4d1342-5a7d-40a6-a7e8-c2c343ef1bcd;437BB295-ED73-4457-A339-512A3547FDFB 27;4 Not Available;Not Available Not Available;Not Available Not Applicable;Not Applicable 1221;1974 Not Available;Not Available Not Available;Unknown Scheduled Follow-up Submission;Scheduled Follow-up Submission Not Available;Unknown Not Available;Not Available Not Available;NO 9;8 Not Available;Not Available Not Available;Unknown Not Available;Unknown Not Available;Unknown NO;NO Not Available;Complete Remission/Response Not Available;Not Available NO;NO Alive;Alive 2011;2013 FEMALE Lung Adenocarcinoma Mixed Subtype No C34.1 8255/3 C34.1 YES Not Available NO Not Available Not Available Peripheral Lung 9 Not Available Not Available Not Available Not Available Not Available Not Available Not Available Not Available Not Available Not Available 21 No 7159 Preoperative Not Available Not Available Not Available Not Available Not Available Not Available Not Available Not Available WHITE Not Available NA R0 Not Applicable Not Applicable Not Applicable Not Applicable Not Applicable Not Applicable Not Applicable Not Applicable Not Applicable Stage IA Not Applicable Not Applicable Not Applicable 6th Not Applicable Not Applicable Not Applicable M0 NX T1 2007 NO YES 78 4 Lung Alive 2011 2008 1965

429 Dead T2a N1 M0 Stage IIA NA 72 R-Lower Not Applicable TCGA-MP-A4TI F327DEA0-2D30-482F-809C-76A148A749B7 2 -26565 429 0 Not Available Not Available Lung Adenocarcinoma Not Available NA Not Evaluated Not Available YES Not Available Not Available Not Available NO Not Available NOT HISPANIC OR LATINO NA MALE Lung Adenocarcinoma- Not Otherwise Specified (NOS) No C34.3 8140/3 C34.3 YES Not Evaluated YES YES G12C Unknown 4 YES YES 84 Unknown Not Available Unknown Not Available Locoregional Recurrence;Distant Metastasis Convincing Imaging YES 35 No A4TI Not Evaluated WITH TUMOR Not Available Not Available Unknown Not Available Not Available Unknown NO WHITE Unknown NA R0 Not Applicable Not Applicable Not Applicable Not Applicable Not Applicable Not Applicable Not Applicable Not Applicable Not Applicable Stage IIA Not Applicable Not Applicable Not Applicable 7th Not Applicable Not Applicable Not Applicable M0 N1 T2a 1990 NO YES MP 3 Lung Dead 2013 2010 1955

444 Dead T3 N2 M0 Stage IIIA NA 52 L-Lower Not Applicable TCGA-86-8359 7e7f089c-25f7-4e14-aed5-d741047b9e42 17 -19088 Not Applicable 0 26 Not Available Lung Adenocarcinoma Not Available NA 1 Not Available Unknown Not Available Not Available Not Available Unknown Not Available NOT HISPANIC OR LATINO NA Not Available Not Available Not Available Not Available TCGA-86-8359-F43296 5A4B847F-5627-4BA7-86C0-99956A979DE4 16 Not Available Not Available 444 Not Available Not Available 4 Scheduled Follow-up Submission Unknown 20 NO 5 Not Available Unknown Post-Adjuvant Therapy Unknown Unknown Unknown Not Available Unknown Dead 2013 MALE Lung Adenocarcinoma- Not Otherwise Specified (NOS) No C34.3 8140/3 C34.3 YES 80 Unknown Not Available Not Available Peripheral Lung 7 Not Available Not Available Not Available Not Available Not Available Not Available Not Available Not Available Not Available Unknown 36 No 8359 Preoperative TUMOR FREE Not Available Not Available Unknown Not Available Not Available Unknown NO WHITE Unknown NA R0 Not Applicable Not Applicable Not Applicable Not Applicable Not Applicable Not Applicable Not Applicable Not Applicable Not Applicable Stage IIIA Not Applicable Not Applicable Not Applicable 7th Not Applicable Not Applicable Not Applicable M0 N2 T3 Not Available YES NO 86 2 Lung Alive 2012 2012 1982

624 Dead T4 N1 M0 Stage IIIB NA 72 R-Lower Not Applicable TCGA-50-5044 fa0c7392-d485-41ac-bf7e-244a528209ae 25 -26567 624 0 Not Available Not Available Lung Adenocarcinoma Not Available NA Not Available Not Available Not Available Not Available Not Available Not Available Not Available Not Available NOT HISPANIC OR LATINO NA YES YES NO NO TCGA-50-5044-F31976 699a5471-d724-47d8-9a63-e846f461acd2 14 Not Available Not Available 624 Not Available 483 Not Available Scheduled Follow-up Submission Progressive Disease Not Available NO 5 Distant Metastasis YES Not Available WITH TUMOR NO Progressive Disease Convincing Imaging YES Dead 2012 FEMALE Lung Adenocarcinoma- Not Otherwise Specified (NOS) No C34.3 8140/3 C34.3 YES Not Available Not Available Not Available Not Available Not Available 8 Not Available Not Available Not Available Not Available Not Available Not Available Not Available Not Available Not Available Not Available Not Available No 5044 Not Available WITH TUMOR Not Available Not Available Not Available Not Available Not Available Not Available Not Available WHITE Not Available NA Primary Tumor Field TCGA-50-5044-R40427 B8DB558B-F9D7-4576-AA04-5B5F98E27BFE Not Available 19 500 491 Unknown 2 7 14.7 NO External Not Applicable Not Available Not Available Not Available 2013 Not Available Not Applicable Not Applicable Not Applicable Not Applicable Not Applicable Not Applicable Not Applicable Not Applicable Not Applicable Stage IIIB Not Applicable Not Applicable Not Applicable 5th Not Applicable Not Applicable Not Applicable M0 N1 T4 Not Available NO YES 50 Not Available Lung Dead 2011 2002 Not Available

422 Alive T2a N1 M0 Stage IIA NA 73 L-Upper Not Applicable TCGA-67-6217 108a71cf-b9db-47cd-aa74-c03ec989b41b 6 -26920 Not Applicable 0 422 Not Available Lung Adenocarcinoma Not Available NA TCGA-67-6217-D13740 912f0718-4f53-4ab2-9972-8bbadf753d8d Not Available 11 443 399 Tarceva Not Available 7 1 100 mg PROGRESSION Not Applicable 1 PO NO Chemotherapy Not Available Not Available Not Available Not Available 2011 Not Available Not Available YES L858R Not Available RT-PCR YES Not Available NOT HISPANIC OR LATINO NA YES Not Available NO NO TCGA-67-6217-F13566 b1095ed9-280b-4211-9fa7-7013f6da1d5d 5 Not Available Not Available Not Applicable 422 296 Not Available Scheduled Follow-up Submission Progressive Disease Not Available Not Available 7 Not Available YES Not Available WITH TUMOR NO Progressive Disease Not Available NO Alive 2011 FEMALE Lung Adenocarcinoma- Not Otherwise Specified (NOS) No C34.1 8140/3 C34.1 YES Not Available YES NO Not Available Central Lung 6 Not Available Not Available Not Available Not Available Not Available Not Available Not Available Not Available Not Available Not Available Not Available Yes 6217 Not Available WITH TUMOR Not Available Not Available Not Available Not Available Not Available Not Available Not Available WHITE Not Available NA R0 Not Applicable Not Applicable Not Applicable Not Applicable Not Applicable Not Applicable Not Applicable Not Applicable Not Applicable Stage IIA Not Applicable Not Applicable Not Applicable 7th Not Applicable Not Applicable Not Applicable M0 N1 T2a 1965 YES NO 67 3 Lung Alive 2011 2010 Not Available

666 Dead T2 N1 M0 Stage IIB NA 59 L-Lower Not Applicable TCGA-78-7150 a0e57b39-960e-4e46-8aa0-d0ee2fd9a7cc 28 -21891 666 0 Not Available Not Available Lung Adenocarcinoma Not Available NA TCGA-78-7150-D16967;TCGA-78-7150-D16965;TCGA-78-7150-D16966 c90077f8-6d30-4eaa-b791-2d8a389fdfbd;071743ff-ba4b-4208-8816-a453c02c436e;a4b1e4fb-299c-4c6f-9057-96dc3d724a7c Not Available;Not Available;Not Available 28;28;28 642;397;397 607;285;285 Paclitaxel;Carboplatin;Gemcitabine Not Available;Not Available;Not Available 9;9;9 2;5;5 140;615;1800 mg/m2;AUC;mg RECURRENCE;PALLIATIVE;PALLIATIVE Not Applicable;Not Applicable;Not Applicable 3;1;2 IV;IV;IV NO;NO;NO Chemotherapy;Chemotherapy;Chemotherapy Not Available;Not Available;Not Available 700;2475;14180 mg/m2;mg;mg/m2 Not Available;Not Available;Not Available 2011;2011;2011 0 Not Available NO Not Available Not Available Not Available NO Not Available Not Available NA YES YES NO YES TCGA-78-7150-F16962 09ad8f79-3e06-43f3-9c00-cd8c7e87bdcc 28 Not Available 164 666 Not Available 139 Not Available Scheduled Follow-up Submission Not Available Not Available Not Available 9 Not Available YES Not Available WITH TUMOR NO Not Available Not Available NO Dead 2011 MALE Lung Adenocarcinoma- Not Otherwise Specified (NOS) No C34.3 8140/3 C34.3 YES Not Available NO Not Available Not Available Central Lung 9 Not Available Not Available Not Available Not Available Not Available Not Available Not Available Not Available Not Available Not Available 37 No 7150 Preoperative WITH TUMOR Not Available Not Available Not Available Not Available Not Available Not Available Not Available WHITE Not Available NA Distant Recurrence;Distant Recurrence TCGA-78-7150-R16964;TCGA-78-7150-R16963 969f1a71-c4c4-48e8-8589-faa544fa5515;65e0a687-005d-4fb1-aa3b-fb9f84bb7dc5 2;1 28;28 546;189 531;185 Not Available;Not Available 9;9 10;5 2500;2000 NO;NO EXTERNAL BEAM;EXTERNAL BEAM Not Applicable;Not Applicable RECURRENCE;PALLIATIVE Not Available;Not Available cGy;cGy 2011;2011 R0 Not Applicable Not Applicable Not Applicable Not Applicable Not Applicable Not Applicable Not Applicable Not Applicable Not Applicable Stage IIB Not Applicable Not Applicable Not Applicable 6th Not Applicable Not Applicable Not Applicable M0 N1 T2 2000 NO YES 78 4 Lung Dead 2011 2002 1963

842 Alive T2 N0 MX Stage IB NA 62 R-Lower Not Applicable TCGA-91-6847 97831d28-ab41-4c18-bfc2-c4c6bc757d13 19 -22862 Not Applicable 0 842 Not Available Lung Adenocarcinoma Not Available NA Not Available Not Available Not Available Not Available Not Available Not Available Not Available Not Available NOT HISPANIC OR LATINO NA NO YES Not Available NO TCGA-91-6847-F32637 FB5F10C7-9572-4BC0-B5F4-245D88F78D07 5 Not Available Not Available Not Applicable 842 772 Unknown Scheduled Follow-up Submission Stable Disease Unknown YES 6 Distant Metastasis YES Not Available WITH TUMOR NO Progressive Disease Convincing Imaging YES Alive 2012 FEMALE Lung Adenocarcinoma- Not Otherwise Specified (NOS) No C34.3 8140/3 C34.3 YES Not Available NO Not Available Not Available Not Available 7 Not Available Not Available Not Available Not Available Not Available Not Available Not Available Not Available Not Available Not Available Not Available No 6847 Not Available Not Available Not Available Not Available Not Available Not Available Not Available Not Available Not Available WHITE Not Available NA Distant site;Distant site TCGA-91-6847-R32638;TCGA-91-6847-R32640 8EF10C9E-D1AB-4797-A3E8-9204A1446CC0;C84D7330-6ED5-4C67-A689-182C0A994A88 Not Available;Not Available 5;5 781;842 772;842 Stable Disease;Unknown 6;6 Not Available;1 40;14.5 NO;NO External;External Not Applicable;Not Applicable Not Available;Not Available Not Available;Not Available Gy;Gy 2012;2012 Not Available Not Applicable Not Applicable Not Applicable Not Applicable Not Applicable Not Applicable Not Applicable Not Applicable Not Applicable Stage IB Not Applicable Not Applicable Not Applicable 5th Not Applicable Not Applicable Not Applicable MX N0 T2 1967 NO YES 91 3 Lung Alive 2011 2002 Not Available

807 Dead T2 N2 M0 Stage IIIA NA 53 L-Upper Not Applicable TCGA-78-7220 fd5c44ef-ea50-4fba-9e8d-e371cf34ebdb 8 -19480 807 0 Not Available Not Available Lung Adenocarcinoma Not Available NA 0 Not Available NO Not Available Not Available Not Available NO Not Available Not Available NA NO YES NO NO TCGA-78-7220-F19298 6125a3be-8022-43a7-8d60-00e74d6061c3 8 Not Available Not Available 807 Not Available 531 Not Available Scheduled Follow-up Submission Not Available Not Available Not Available 12 Not Available YES Not Available WITH TUMOR NO Not Available Not Available YES Dead 2011 FEMALE Lung Adenocarcinoma Mixed Subtype No C34.1 8255/3 C34.1 YES Not Available NO Not Available Not Available Peripheral Lung 12 Not Available Not Available Not Available Not Available Not Available Not Available Not Available Not Available Not Available Not Available 35 No 7220 Preoperative WITH TUMOR Not Available Not Available Not Available Not Available Not Available Not Available Not Available WHITE Not Available NA Primary Tumor Field;Distant site TCGA-78-7220-R19299;TCGA-78-7220-R19300 cea1849b-13c0-4d25-a6c7-aeae07d41d0b;d5d23e28-c8be-4193-8bf5-c8587e92133e 1;2 8;8 96;740 60;734 Not Available;Not Available 12;12 25;5 5000;2000 NO;NO EXTERNAL BEAM;EXTERNAL BEAM Not Applicable;Not Applicable ADJUVANT;RECURRENCE Not Available;Not Available cGy;cGy 2011;2011 R0 Not Applicable Not Applicable Not Applicable Not Applicable Not Applicable Not Applicable Not Applicable Not Applicable Not Applicable Stage IIIA Not Applicable Not Applicable Not Applicable 6th Not Applicable Not Applicable Not Applicable M0 N2 T2 Not Available NO YES 78 2 Lung Dead 2011 2000 1965

385 Alive T2a N0 M0 Stage IB NA 54 R-Upper Not Applicable TCGA-44-8117 6d96a298-be5a-4e14-afe8-ca4b6ca298d6 11 -19855 Not Applicable 0 259 Not Available Lung Adenocarcinoma 86 NA TCGA-44-8117-D32726;TCGA-44-8117-D32727 19D36EEB-480F-4671-BCA8-86F6494EA205;EEB0D889-2452-4652-AD4C-81CCD822D2CE Not Available;Not Available 13;13 176;133 113;113 Cisplatin;Docetaxel Complete Response;Complete Response 6;6 Not Available;Not Available Not Available;Not Available Not Available;Not Available Not Available;Not Available Not Applicable;Not Applicable Not Available;Not Available Not Available;Not Available NO;NO Chemotherapy;Chemotherapy Not Available;Not Available Not Available;Not Available Not Available;Not Available NO;NO 2012;2012 1 Not Available Unknown Not Available Not Available Not Available Unknown Not Available NOT HISPANIC OR LATINO NA Not Available Not Available Not Available Not Available TCGA-44-8117-F36070 E5A2A932-540F-4F64-8A4B-0357E1C9C18B 18 Not Applicable Not Applicable Not Applicable 385 Not Applicable 1 Scheduled Follow-up Submission Complete Remission/Response Not Available NO 10 Not Available NO Post-Adjuvant Therapy TUMOR FREE YES Complete Remission/Response Not Available NO Alive 2012 FEMALE Lung Adenocarcinoma- Not Otherwise Specified (NOS) No C34.1 8140/3 C34.1 YES Not Available Unknown Not Available Not Available Unknown 6 Not Available Not Available Not Available Not Available Not Available Not Available Not Available Not Available Not Available NO 54 No 8117 Pre-Adjuvant Therapy TUMOR FREE 88 65 YES 83 60 Complete Remission/Response YES WHITE NO NA Not Available Not Applicable Not Applicable Not Applicable Not Applicable Not Applicable Not Applicable Not Applicable Not Applicable Not Applicable Stage IB Not Applicable Not Applicable Not Applicable 7th Not Applicable Not Applicable Not Applicable M0 N0 T2a 2011 YES NO 44 4 Lung Alive 2012 2011 1975

173 Dead T2 N2 M0 Stage IIIA NA 71 L-Lower Not Applicable TCGA-78-7146 ff9def3d-17e5-4ef6-b74e-933f11ed6f00 28 -26272 173 0 Not Available Not Available Lung Adenocarcinoma Not Available NA 1 Not Available NO Not Available Not Available Not Available NO Not Available Not Available NA Not Available Not Available Not Available Not Available TCGA-78-7146-F16969 89a4cee2-d516-4b0e-8664-c1a31a242e71 28 Not Available Not Available 173 Not Available Not Available Not Available Scheduled Follow-up Submission Not Available Not Available Not Available 9 Not Available Not Available Not Available Not Available NO Not Available Not Available NO Dead 2011 FEMALE Lung Adenocarcinoma Mixed Subtype No C34.3 8255/3 C34.3 YES Not Available NO Not Available Not Available Peripheral Lung 9 Not Available Not Available Not Available Not Available Not Available Not Available Not Available Not Available Not Available Not Available 6 No 7146 Preoperative Not Available Not Available Not Available Not Available Not Available Not Available Not Available Not Available WHITE Not Available NA R0 Not Applicable Not Applicable Not Applicable Not Applicable Not Applicable Not Applicable Not Applicable Not Applicable Not Applicable Stage IIIA Not Applicable Not Applicable Not Applicable 6th Not Applicable Not Applicable Not Applicable M0 N2 T2 1998 NO YES 78 4 Lung Dead 2011 1999 1988

652 Alive T2a N0 M0 Stage IB NA 60 R-Upper Not Applicable TCGA-97-A4M0 DD63D0FD-E24B-40A5-88C5-7D3D4106631E 7 -22206 Not Applicable 0 216 Not Available Lung Adenocarcinoma Not Available NA 0 Not Available NO Not Available Not Available Not Available NO Not Available NOT HISPANIC OR LATINO NA Not Available Not Available Not Available Not Available TCGA-97-A4M0-F57385 551C91CF-3AEC-44DA-BF0F-DAB40F977E32 10 Not Applicable Not Applicable Not Applicable 652 Not Applicable 0 Scheduled Follow-up Submission Complete Remission/Response Unknown NO 3 Not Available NO Other TUMOR FREE NO Complete Remission/Response Not Available NO Alive 2014 FEMALE Lung Adenocarcinoma Mixed Subtype No C34.1 8255/3 C34.1 YES Not Evaluated YES YES G12C Unknown 3 Not Available Not Available Not Available Not Available Not Available Not Available Not Available Not Available Not Available NO 34 No A4M0 Preoperative Unknown Not Available Not Available NO 90 97 Complete Remission/Response YES WHITE NO NA Not Evaluated Not Applicable Not Applicable Not Applicable Not Applicable Not Applicable Not Applicable Not Applicable Not Applicable Not Applicable Stage IB Not Applicable Not Applicable Not Applicable 7th Not Applicable Not Applicable Not Applicable M0 N0 T2a 2004 YES NO 97 4 Lung Alive 2013 2012 1970

651 Alive T1a N3 MX Stage IIIB NA 60 R-Upper Not Applicable TCGA-55-A48Z 16D3C1C1-CF3A-44B6-B564-8AE5464B243D 21 -22056 Not Applicable 0 29 Not Available Lung Adenocarcinoma 110 NA TCGA-55-A48Z-D59069;TCGA-55-A48Z-D59070 AFA50190-D879-4A5D-A4A9-F1A7F1E161AA;1B807520-A86F-4B84-8830-F8DDCC3231BE Not Available;Not Available 30;30 202;202 58;58 Cisplatin;Alimta Stable Disease;Stable Disease 4;4 Not Available;Not Available Not Available;Not Available Not Available;Not Available Not Available;Not Available Not Applicable;Not Applicable Not Available;Not Available Not Available;Not Available NO;NO Chemotherapy;Chemotherapy Not Available;Not Available Not Available;Not Available Not Available;Not Available NO;NO 2014;2014 2 Not Available YES Other Not Available FISH YES Not Available NOT HISPANIC OR LATINO NA NO YES NO NO TCGA-55-A48Z-F59061 D7D0B518-0721-4E90-96F0-BBDE43C09282 30 Not Available Not Available Not Applicable 651 536 Unknown Scheduled Follow-up Submission Not Applicable Unknown NO 4 Distant Metastasis YES Not Available WITH TUMOR YES Stable Disease Convincing Imaging YES Alive 2014 FEMALE Lung Adenocarcinoma Mixed Subtype No C34.1 8255/3 C34.1 YES 70 YES NO Not Available Central Lung 2 Not Available Not Available Not Available Not Available Not Available Not Available Not Available Not Available Not Available NO 40 No A48Z Preoperative WITH TUMOR Not Available Not Available Unknown 85 87 Unknown YES WHITE Unknown NA Primary Tumor Field;Distant Recurrence TCGA-55-A48Z-R59063;TCGA-55-A48Z-R59068 5402A6C5-9B30-41B2-84A2-CA7417D81721;E95BD641-FE8E-4767-B875-0517421D30B7 Not Available;Not Available 30;30 195;567 154;539 Stable Disease;Radiographic Progressive Disease 4;4 Not Available;Not Available 5040;Not Available NO;NO External;External Not Applicable;Not Applicable Not Available;Not Available Not Available;Not Available cGy;Not Available 2014;2014 R0 Not Applicable Not Applicable Not Applicable Not Applicable Not Applicable Not Applicable Not Applicable Not Applicable Not Applicable Stage IIIB Not Applicable Not Applicable Not Applicable 7th Not Applicable Not Applicable Not Applicable MX N3 T1a 2012 YES NO 55 4 Lung Alive 2013 2012 1972

1036 Alive T1 N0 M0 Stage IA NA FPPP TCGA 60 L-Upper Not Applicable TCGA-44-3918 6e3b6b72-142d-4b8d-a462-28a205796e41 7 -22236 Not Applicable 0 197 197 Lung Adenocarcinoma 76 NA TCGA-44-3918-D20986;TCGA-44-3918-D20985 16f14090-dd3a-4b0c-91c7-998cee0e8d40;4cc469d8-02e3-4ef9-88d3-cd70b4fbc386 Not Available;Not Available 25;25 648;648 543;543 Carboplatin;Alimta Not Available;Not Available 1;1 6;6 500;930 mg;mg RECURRENCE;RECURRENCE Not Applicable;Not Applicable 1;1 IV;IV NO;NO Chemotherapy;Chemotherapy Not Available;Not Available 3380;5660 mg;mg Not Available;Not Available 2012;2012 Not Available Not Available Not Available Not Available Not Available Not Available Not Available Not Available NOT HISPANIC OR LATINO NA NO;YES;YES NO;YES;YES NO;NO;NO NO;Not Available;Not Available TCGA-44-3918-F5231;TCGA-44-3918-F21270;TCGA-44-3918-F38999 439f02a5-f262-430e-8cda-1308ad6cbb3d;02d8cb32-463a-4a11-bdf6-defceba7c69b;2C352F95-6915-4088-A9C0-D97582F74B5C 8;8;10 Not Applicable;Not Available;Not Available Not Applicable;Not Available;Not Available Not Applicable;Not Applicable;Not Applicable 197;715;1036 Not Applicable;511;511 Not Available;0;Not Available Not Available;Additional New Tumor Event;Scheduled Follow-up Submission Complete Remission/Response;Stable Disease;Stable Disease Not Available;Not Available;90 Not Available;Not Available;NO 10;2;1 Not Available;Not Available;Locoregional Recurrence NO;YES;YES Not Available;Other;Other TUMOR FREE;WITH TUMOR;WITH TUMOR NO;Not Available;NO Complete Remission/Response;Not Available;Complete Remission/Response Not Available;Not Available;Biopsy with Histologic Confirmation;Convincing Imaging NO;Not Available;NO Alive;Alive;Alive 2010;2012;2013 FEMALE Lung Adenocarcinoma- Not Otherwise Specified (NOS) No C34.1 8140/3 C34.1 YES Not Available NO Not Available Not Available Not Available 10 Not Available Not Available Not Available Not Available Not Available Not Available Not Available Not Available Not Available Not Available 20 No 3918 Not Available TUMOR FREE Not Available Not Available Not Available 103 104 Not Available YES WHITE Not Available NA Local Recurrence TCGA-44-3918-R21268 2b4b3df7-bfff-4e7e-b5be-3cad8f87f82d 1 8 697 686 Not Available 2 10 3000 NO EXTERNAL BEAM Not Applicable PALLIATIVE Not Available cGy 2012 R0 Not Applicable Not Applicable Not Applicable Not Applicable Not Applicable Not Applicable Not Applicable Not Applicable Not Applicable Stage IA Not Applicable Not Applicable Not Applicable 6th Not Applicable Not Applicable Not Applicable M0 N0 T1 1994 YES NO 44 3 Lung Alive 2010 2010 1974

586 Dead T2 N1 M0 Stage IIB NA 67 R-Upper Not Applicable TCGA-78-7147 e7d30bc2-42e8-49c5-a5b3-9f58411b007a 28 -24809 586 0 Not Available Not Available Lung Adenocarcinoma Not Available NA 1 Not Available NO Not Available Not Available Not Available NO Not Available Not Available NA NO NO NO NO TCGA-78-7147-F16971 a52cbdf4-8abe-4493-9575-72d42251aea8 28 Not Available Not Available 586 Not Available 578 Not Available Scheduled Follow-up Submission Progressive Disease Not Available Not Available 9 Not Available YES Not Available WITH TUMOR NO Not Available Not Available NO Dead 2011 FEMALE Lung Adenocarcinoma- Not Otherwise Specified (NOS) No C34.1 8140/3 C34.1 YES Not Available NO Not Available Not Available Peripheral Lung 9 Not Available Not Available Not Available Not Available Not Available Not Available Not Available Not Available Not Available Not Available 50 No 7147 Preoperative WITH TUMOR Not Available Not Available Not Available Not Available Not Available Not Available Not Available WHITE Not Available NA R0 Not Applicable Not Applicable Not Applicable Not Applicable Not Applicable Not Applicable Not Applicable Not Applicable Not Applicable Stage IIB Not Applicable Not Applicable Not Applicable 6th Not Applicable Not Applicable Not Applicable M0 N1 T2 Not Available NO YES 78 2 Lung Dead 2011 2001 1950

1653 Dead T2 N0 M0 Stage IB NA 73 L-Upper Not Applicable TCGA-62-A46S E00A60D7-4253-443D-A187-680AD0931349 29 -26825 1653 0 Not Available Not Available Lung Adenocarcinoma Not Available NA TCGA-62-A46S-D36326;TCGA-62-A46S-D40574 A78743CC-2953-4ECE-A4AE-0C60106B1A3C;C9C16A96-C581-4DB9-B7F3-5B6ACCC39DFC Not Available;Not Available 31;25 198;198 78;78 Tarceva;Placebo Unknown;Unknown 10;2 Not Available;Not Available Not Available;Not Available Not Available;Not Available Not Available;Not Available Not Applicable;Not Applicable Not Available;Not Available Not Available;Not Available NO;NO Targeted Molecular therapy;Targeted Molecular therapy Not Available;Not Available Not Available;Not Available Not Available;Not Available YES;YES 2012;2013 0 Not Available NO Not Available Not Available Not Available NO Not Available NOT HISPANIC OR LATINO NA MALE Lung Papillary Adenocarcinoma No C34.1 8260/3 C34.1 YES 100 NO Not Available Not Available Peripheral Lung 10 YES NO 527 NO Not Available NO Not Available Locoregional Recurrence;Distant Metastasis Convincing Imaging YES 40 No A46S Preoperative WITH TUMOR Not Available Not Available YES 66 72 Complete Remission/Response YES WHITE NO NA R0 Not Applicable Not Applicable Not Applicable Not Applicable Not Applicable Not Applicable Not Applicable Not Applicable Not Applicable Stage IB Not Applicable Not Applicable Not Applicable 6th Not Applicable Not Applicable Not Applicable M0 N0 T2 Not Available NO YES 62 4 Lung Dead 2012 2007 Not Available

547 Alive T1b N0 M0 Stage IA NA 69 R-Lower Not Applicable TCGA-55-8203 9a9bd705-1ef1-4a6c-bb1e-bdd346dc01c0 16 -25477 Not Applicable 0 4 Not Available Lung Adenocarcinoma 39 NA Not Available Not Available NO Not Available Not Available Not Available NO Not Available NOT HISPANIC OR LATINO NA Not Available Not Available Not Available Not Available TCGA-55-8203-F47819 C9045FA4-F6A7-401C-8BB1-2A97C88D9330 28 Not Applicable Not Applicable Not Applicable 547 Not Applicable Not Evaluated Scheduled Follow-up Submission Complete Remission/Response Not Evaluated NO 8 Not Available NO Not Evaluated TUMOR FREE NO Complete Remission/Response Not Available NO Alive 2013 FEMALE Lung Adenocarcinoma- Not Otherwise Specified (NOS) No C34.3 8140/3 C34.3 YES Not Available NO Not Available Not Available Not Available 6 Not Available Not Available Not Available Not Available Not Available Not Available Not Available Not Available Not Available NO 50 Yes, History of Prior Malignancy 8203 Not Available TUMOR FREE 50 48 Unknown 52 49 Complete Remission/Response YES WHITE Unknown NA R0 Not Applicable Not Applicable Not Applicable Not Applicable Not Applicable Not Applicable Not Applicable Not Applicable Not Applicable Stage IA Not Applicable Not Applicable Not Applicable 7th Not Applicable Not Applicable Not Applicable M0 N0 T1b Not Available YES NO 55 2 Lung Alive 2012 2011 Not Available

1683 Alive T2a N1 MX Stage IIA NA 64 L-Upper Not Applicable TCGA-49-6744 15340dd3-84ae-49fb-989a-eb212f3e73da 10 -23484 Not Applicable 0 890 Not Available Lung Adenocarcinoma Not Available NA TCGA-49-6744-D16686 9954442a-123d-4dc2-a41d-966d898e3090 Not Available 23 108 16 Not Available Not Available 9 Not Available Not Available Not Available ADJUVANT Not Applicable 1 IV NO Chemotherapy Not Available Not Available Not Available Not Available 2011 Not Available Not Available Not Available Not Available Not Available Not Available Not Available Not Available Not Available NA Not Available;NO Not Available;NO Not Available;Not Available Not Available;Not Available TCGA-49-6744-F14923;TCGA-49-6744-F70580 1841d2d6-26a6-417f-b575-0af2e3d8ca1a;64733B43-3503-4D09-8FA4-D6BACF138AF4 10;25 Not Applicable;Not Applicable Not Applicable;Not Applicable Not Applicable;Not Applicable 890;1683 Not Applicable;Not Applicable Not Available;1 Not Available;Scheduled Follow-up Submission Complete Remission/Response;Complete Remission/Response Not Available;90 Not Available;NO 8;2 Not Available;Not Available NO;NO Not Available;Post-Adjuvant Therapy TUMOR FREE;TUMOR FREE YES;YES Complete Remission/Response;Complete Remission/Response Not Available;Not Available NO;NO Alive;Alive 2011;2015 FEMALE Lung Adenocarcinoma Mixed Subtype No C34.1 8255/3 C34.1 YES Not Available YES YES Not Available Not Available 8 Not Available Not Available Not Available Not Available Not Available Not Available Not Available Not Available Not Available Not Available 20 No 6744 Not Available TUMOR FREE Not Available Not Available Not Available Not Available Not Available Not Available Not Available WHITE Not Available NA R0 Not Applicable Not Applicable Not Applicable Not Applicable Not Applicable Not Applicable Not Applicable Not Applicable Not Applicable Stage IIA Not Applicable Not Applicable Not Applicable 7th Not Applicable Not Applicable Not Applicable MX N1 T2a Not Available NO YES 49 4 Lung Alive 2011 2010 Not Available

546 Alive T1b N0 M0 Stage IA NA 80 L-Upper Not Applicable TCGA-55-A57B 3B21B982-DBA2-45F4-AD8D-21DC86FCAAA7 31 -29452 Not Applicable 0 21 Not Available Lung Adenocarcinoma 77 NA 0 Not Available NO Not Available Not Available Not Available NO Not Available NOT HISPANIC OR LATINO NA Not Available Not Available Not Available Not Available TCGA-55-A57B-F60133 828E9149-B556-4073-8F6B-55F1F065F37C 29 Not Applicable Not Applicable Not Applicable 546 Not Applicable Unknown Scheduled Follow-up Submission Complete Remission/Response Unknown NO 5 Not Available NO Not Available TUMOR FREE NO Complete Remission/Response Not Available NO Alive 2014 FEMALE Lung Adenocarcinoma Mixed Subtype No C34.1 8255/3 C34.1 YES 90 NO Not Available Not Available Peripheral Lung 5 Not Available Not Available Not Available Not Available Not Available Not Available Not Available Not Available Not Available NO Not Available No A57B Preoperative TUMOR FREE Not Available Not Available Unknown 99 114 Not Applicable YES BLACK OR AFRICAN AMERICAN Unknown NA R0 Not Applicable Not Applicable Not Applicable Not Applicable Not Applicable Not Applicable Not Applicable Not Applicable Not Applicable Stage IA Not Applicable Not Applicable Not Applicable 7th Not Applicable Not Applicable Not Applicable M0 N0 T1b Not Available YES NO 55 1 Lung Alive 2013 2012 Not Available

552 Alive T2a N0 MX Stage IB NA 73 R-Upper Not Applicable TCGA-55-8511 1f1df7ea-00b9-4432-86de-da773d1451e5 11 -26986 Not Applicable 0 9 Not Available Lung Adenocarcinoma 31 NA 2 Not Available Unknown Not Available Not Available Not Available Unknown Not Available NOT HISPANIC OR LATINO NA Unknown Unknown NO NO TCGA-55-8511-F66028 E2BDF53C-410E-4BF9-910E-C89C1F94487E 1 Not Available Not Available Not Applicable 552 460 Not Available Scheduled Follow-up Submission Progressive Disease Not Available NO 10 Not Available YES Not Available WITH TUMOR NO Complete Remission/Response Not Available NO Alive 2014 FEMALE Lung Adenocarcinoma- Not Otherwise Specified (NOS) No C34.1 8140/3 C34.1 YES 70 Unknown Not Available Not Available Unknown 1 Not Available Not Available Not Available Not Available Not Available Not Available Not Available Not Available Not Available NO 58 Yes, History of Prior Malignancy 8511 Preoperative TUMOR FREE Not Available Not Available NO 82 53 Complete Remission/Response YES WHITE NO NA R0 Not Applicable Not Applicable Not Applicable Not Applicable Not Applicable Not Applicable Not Applicable Not Applicable Not Applicable Stage IB Not Applicable Not Applicable Not Applicable 7th Not Applicable Not Applicable Not Applicable MX N0 T2a Not Available YES NO 55 2 Lung Alive 2013 2012 1953

545 Alive T2a N0 M0 Stage IB NA 75 R-Upper Not Applicable TCGA-97-8172 cbf1f718-6bb7-4daf-b9d6-fb294281decb 13 -27416 Not Applicable 0 182 Not Available Lung Adenocarcinoma 64.98 NA 1 Not Available YES Not Available Not Available Not Available NO Not Available NOT HISPANIC OR LATINO NA Not Available Not Available Not Available Not Available TCGA-97-8172-F44230 52560AC4-46A3-4DEC-8DD6-A80E5C3A6658 19 Not Applicable Not Applicable Not Applicable 545 Not Applicable Not Evaluated Scheduled Follow-up Submission Complete Remission/Response Not Evaluated NO 6 Not Available NO Not Evaluated TUMOR FREE NO Complete Remission/Response Not Available NO Alive 2013 FEMALE Lung Acinar Adenocarcinoma No C34.1 8550/3 C34.1 YES Not Available YES YES G12V Unknown 6 Not Available Not Available Not Available Not Available Not Available Not Available Not Available Not Available Not Available NO 20 Yes, History of Prior Malignancy 8172 Preoperative TUMOR FREE 105.10 107.25 NO 93.27 104.64 Complete Remission/Response YES WHITE NO NA Not Available Not Applicable Not Applicable Not Applicable Not Applicable Not Applicable Not Applicable Not Applicable Not Applicable Not Applicable Stage IB Not Applicable Not Applicable Not Applicable 7th Not Applicable Not Applicable Not Applicable M0 N0 T2a 1981 YES NO 97 3 Lung Alive 2012 2011 1961

1523 Alive T2 N0 M0 Stage IB NA 67 R-Lower Not Applicable TCGA-05-4249 4addf05f-3668-4b3f-a17f-c0227329ca52 22 -24532 Not Applicable 0 1158 Not Available Lung Adenocarcinoma Not Available NA Not Available Not Available Not Available Not Available Not Available Not Available Not Available Not Available Not Available NA Not Available Not Available Not Available Not Available TCGA-05-4249-F36327 485963A2-753D-4E86-B3DF-E3F66189ABEE 29 Not Applicable Not Applicable Not Applicable 1523 Not Applicable Not Available Scheduled Follow-up Submission Complete Remission/Response Not Available NO 10 Not Available NO Not Available TUMOR FREE NO Not Applicable Not Available NO Alive 2012 MALE Lung Adenocarcinoma- Not Otherwise Specified (NOS) No C34.3 8140/3 C34.3 YES Not Available Not Available Not Available Not Available Peripheral Lung 7 Not Available Not Available Not Available Not Available Not Available Not Available Not Available Not Available Not Available Not Available 52 No 4249 Not Available TUMOR FREE Not Available Not Available Not Available Not Available Not Available Not Available Not Available Not Available Not Available NA R0 Not Applicable Not Applicable Not Applicable Not Applicable Not Applicable Not Applicable Not Applicable Not Applicable Not Applicable Stage IB Not Applicable Not Applicable Not Applicable 6th Not Applicable Not Applicable Not Applicable M0 N0 T2 Not Available NO YES 05 3 Lung Alive 2010 2007 Not Available

435 Alive T1b N0 MX Stage IA NA 60 R-Upper Not Applicable TCGA-55-6543 e68219b0-a9c2-49df-8f0d-db5ea97fd2dc 3 Not Available Not Applicable 0 2 Not Available Lung Adenocarcinoma Not Available NA Not Available Not Available Not Available Not Available Not Available Not Available Not Available Not Available Not Available NA Not Available Not Available Not Available Not Available TCGA-55-6543-F46659 328EC06C-F892-4324-A3ED-FB2A283BCC0B 13 Not Applicable Not Applicable Not Applicable 435 Not Applicable Unknown Scheduled Follow-up Submission Complete Remission/Response Unknown NO 8 Not Available NO Unknown TUMOR FREE NO Complete Remission/Response Not Available NO Alive 2013 FEMALE Lung Bronchioloalveolar Carcinoma Mucinous No C34.1 8253/3 C34.1 YES Not Available Not Available Not Available Not Available Peripheral Lung 12 Not Available Not Available Not Available Not Available Not Available Not Available Not Available Not Available Not Available Not Available 60 No 6543 Not Available TUMOR FREE Not Available Not Available Not Available Not Available Not Available Not Available Not Available WHITE Not Available NA Not Available Not Applicable Not Applicable Not Applicable Not Applicable Not Applicable Not Applicable Not Applicable Not Applicable Not Applicable Stage IA Not Applicable Not Applicable Not Applicable 7th Not Applicable Not Applicable Not Applicable MX N0 T1b 2004 NO YES 55 4 Lung Alive 2010 2010 Not Available

3169 Dead T1 N0 M0 Stage IA NA 75 L-Upper Not Applicable TCGA-78-7162 16cd58db-d52c-403e-9e9c-6da2e99f6bad 3 -27593 3169 0 Not Available Not Available Lung Adenocarcinoma Not Available NA 0 Not Available NO Not Available Not Available Not Available NO Not Available Not Available NA NO;YES YES;NO NO;NO NO;NO TCGA-78-7162-F17125;TCGA-78-7162-F17127 19df3a15-5642-4b23-ab3b-d6fbc2d17193;125d989e-fe36-43e7-b9f2-515970b19506 3;3 Not Available;Not Available Not Available;Not Available 3169;3169 Not Available;Not Available 2218;2740 Not Available;Not Available Scheduled Follow-up Submission;Additional New Tumor Event Not Available;Not Available Not Available;Not Available Not Available;Not Available 10;10 Not Available;Not Available YES;YES Not Available;Not Available Not Available;Not Available NO;NO Not Available;Not Available Not Available;Not Available NO;NO Dead;Dead 2011;2011 MALE Lung Adenocarcinoma Mixed Subtype No C34.1 8255/3 C34.1 YES Not Available NO Not Available Not Available Peripheral Lung 10 Not Available Not Available Not Available Not Available Not Available Not Available Not Available Not Available Not Available Not Available 12 No 7162 Preoperative Not Available Not Available Not Available Not Available Not Available Not Available Not Available Not Available WHITE Not Available NA Regional site TCGA-78-7162-R17154 25072ff0-dbfb-45ab-9bfc-f640a5b12e6a 1 4 Not Available Not Available Not Available 10 Not Available Not Available NO EXTERNAL BEAM Not Applicable RECURRENCE Not Available Not Available 2011 R0 Not Applicable Not Applicable Not Applicable Not Applicable Not Applicable Not Applicable Not Applicable Not Applicable Not Applicable Stage IA Not Applicable Not Applicable Not Applicable 6th Not Applicable Not Applicable Not Applicable M0 N0 T1 Not Available NO YES 78 2 Lung Dead 2011 1991 1940

444 Dead T2 N2 M0 Stage IIIA NA 55 R-Lower Not Applicable TCGA-62-8398 2fabeb98-05e3-4f55-97f5-fbc675e25a3d 4 -20197 444 0 Not Available Not Available Lung Adenocarcinoma Not Available NA TCGA-62-8398-D33507;TCGA-62-8398-D40458 316E364F-9824-4D22-AB44-6C9181EF3AAA;BB70829B-5995-4D0D-ABEF-22D1AD108527 Not Available;Not Available 5;21 113;113 41;41 Carboplatin;Vinorelbin Complete Response;Complete Response 7;2 Not Available;Not Available Not Available;Not Available Not Available;Not Available Not Available;Not Available Not Applicable;Not Applicable Not Available;Not Available Not Available;Not Available NO;NO Chemotherapy;Chemotherapy Not Available;Not Available Not Available;Not Available Not Available;Not Available NO;NO 2012;2013 Unknown Not Available NO Not Available Not Available Not Available NO Not Available NOT HISPANIC OR LATINO NA MALE Lung Adenocarcinoma Mixed Subtype No C34.3 8255/3 C34.3 YES 100 NO Not Available Not Available Central Lung 7 Not Available Not Available Not Available Not Available Not Available Not Available Not Available Not Available Not Available NO 20 No 8398 Preoperative TUMOR FREE Not Available Not Available YES 72 64 Complete Remission/Response YES WHITE NO NA R0 Not Applicable Not Applicable Not Applicable Not Applicable Not Applicable Not Applicable Not Applicable Not Applicable Not Applicable Stage IIIA Not Applicable Not Applicable Not Applicable 6th Not Applicable Not Applicable Not Applicable M0 N2 T2 1999 NO YES 62 4 Lung Dead 2012 2007 1970

4961 Dead T2 N0 M0 Stage IB NA 62 L-Upper Not Applicable TCGA-78-7143 54254f5a-50e8-4150-a9b7-56a0470d2a56 23 -22673 4961 0 Not Available Not Available Lung Adenocarcinoma Not Available NA 0 Not Available NO Not Available Not Available Not Available NO Not Available Not Available NA NO;NO NO;NO NO;YES NO;NO TCGA-78-7143-F16669;TCGA-78-7143-F16667 b280d071-c071-4b1f-8df7-1eb99db3a117;6bba4fc0-b389-42ae-b498-67aeae1e3b46 23;23 Not Available;1577 Not Available;Not Available 4961;4961 Not Available;Not Available 4608;1500 Not Available;Not Available Additional New Tumor Event;Scheduled Follow-up Submission Not Available;Complete Remission/Response Not Available;Not Available Not Available;Not Available 9;9 Not Available;Not Available YES;YES Not Available;Not Available Discrepancy;Discrepancy Not Available;NO Not Available;Not Available Not Available;Not Available Not Available;NO Dead;Dead 2011;2011 FEMALE Lung Bronchioloalveolar Carcinoma Nonmucinous No C34.1 8252/3 C34.1 YES Not Available NO Not Available Not Available Central Lung 9 Not Available Not Available Not Available Not Available Not Available Not Available Not Available Not Available Not Available Not Available Not Available No 7143 Preoperative Discrepancy Not Available Not Available Not Available Not Available Not Available Not Available Not Available WHITE Not Available NA R0 Not Applicable Not Applicable Not Applicable Not Applicable Not Applicable Not Applicable Not Applicable Not Applicable Not Applicable Stage IB Not Applicable Not Applicable Not Applicable 6th Not Applicable Not Applicable Not Applicable M0 N0 T2 Not Available NO YES 78 1 Lung Dead 2011 1992 Not Available

121 Dead T3 N1 M0 Stage IIIA NA 79 R-Lower Not Applicable TCGA-05-4250 f98ecd8a-b878-4f53-b911-20cd8e17281c 22 -29068 121 0 Not Available Not Available Lung Adenocarcinoma Not Available NA Not Available Not Available Not Available Not Available Not Available Not Available Not Available Not Available Not Available NA FEMALE Lung Adenocarcinoma- Not Otherwise Specified (NOS) No C34.3 8140/3 C34.3 YES Not Available Not Available Not Available Not Available Not Available 7 Not Available Not Available Not Available Not Available Not Available Not Available Not Available Not Available Not Available Not Available 47 No 4250 Not Available Not Available Not Available Not Available Not Available Not Available Not Available Not Available Not Available Not Available Not Available NA R2 Not Applicable Not Applicable Not Applicable Not Applicable Not Applicable Not Applicable Not Applicable Not Applicable Not Applicable Stage IIIA Not Applicable Not Applicable Not Applicable 6th Not Applicable Not Applicable Not Applicable M0 N1 T3 Not Available NO YES 05 4 Lung Dead 2010 2007 Not Available

457 Alive T2 N2 M0 Stage IIIA NA 67 L-Upper Not Applicable TCGA-05-5420 8b119d1c-6d21-4bbd-8a00-12da7b97d6c4 22 -24472 Not Applicable 0 31 Not Available Lung Adenocarcinoma Not Available NA Not Available Not Available Not Available Not Available Not Available Not Available Not Available Not Available Not Available NA Unknown Unknown Not Available Not Available TCGA-05-5420-F36422 5E6ACDEB-BC35-4B1C-BF79-F54891E7274D 30 Not Available Not Available Not Applicable 457 245 Not Available Scheduled Follow-up Submission Progressive Disease Not Available NO 10 Not Available YES Not Available WITH TUMOR Unknown Progressive Disease Not Available Unknown Alive 2012 MALE Lung Adenocarcinoma- Not Otherwise Specified (NOS) No C34.1 8140/3 C34.9 YES Not Available Not Available Not Available Not Available Not Available 3 Not Available Not Available Not Available Not Available Not Available Not Available Not Available Not Available Not Available Not Available 40 Yes 5420 Not Available TUMOR FREE Not Available Not Available Not Available Not Available Not Available Not Available Not Available Not Available Not Available NA R0 Not Applicable Not Applicable Not Applicable Not Applicable Not Applicable Not Applicable Not Applicable Not Applicable Not Applicable Stage IIIA Not Applicable Not Applicable Not Applicable 6th Not Applicable Not Applicable Not Applicable M0 N2 T2 2001 NO YES 05 4 Lung Alive 2011 2008 1961

22 Dead T1 N0 M0 Stage I NA 84 R-Upper Not Applicable TCGA-50-6673 ab8d7f84-dd71-42c7-9471-4db967a9c89c 9 -30689 22 0 Not Available Not Available Lung Adenocarcinoma Not Available NA Not Available Not Available Not Available Not Available Not Available Not Available Not Available Not Available Not Available NA Not Available Not Available Not Available Not Available TCGA-50-6673-F44135 48C0BF4B-CE63-4AF9-AFE7-8EC001B9BBED 13 Not Applicable Not Applicable 22 Not Available Not Applicable Not Available Scheduled Follow-up Submission Unknown Not Available NO 6 Not Available NO Not Available Unknown NO Unknown Not Available YES Dead 2013 FEMALE Lung Adenocarcinoma- Not Otherwise Specified (NOS) No C34.1 8140/3 C34.1 YES Not Available Not Available Not Available Not Available Not Available 1 Not Available Not Available Not Available Not Available Not Available Not Available Not Available Not Available Not Available Not Available Not Available No 6673 Not Available Not Available Not Available Not Available Not Available Not Available Not Available Not Available Not Available WHITE Not Available NA Primary Tumor Field TCGA-50-6673-R67043 14FB9A30-F935-4AFB-A869-099A1866D8E7 Not Available 29 0 0 Radiographic Progressive Disease 10 50 .508 NO Internal Not Applicable Not Available Not Available mCi 2014 R0 Not Applicable Not Applicable Not Applicable Not Applicable Not Applicable Not Applicable Not Applicable Not Applicable Not Applicable Stage I Not Applicable Not Applicable Not Applicable 6th Not Applicable Not Applicable Not Applicable M0 N0 T1 Not Available NO YES 50 1 Lung Dead 2012 2004 Not Available

281 Dead T2a N1 M0 Stage IIA NA 45 R-Upper Not Applicable TCGA-73-4676 195a5afb-b79f-44d2-9d12-884487630c2b 18 -16746 Not Applicable 0 122 Not Available Lung Adenocarcinoma 86 NA TCGA-73-4676-D12586;TCGA-73-4676-D12585;TCGA-73-4676-D12587;TCGA-73-4676-D12556 48957710-70e7-4615-9717-fb2a9174b210;0989c0d2-81e6-4f78-93b4-3a7f35ba221b;1eba36d1-01f8-4cd4-b90d-98cc949417f0;da213998-af4a-4a37-967e-419359cab836 Not Available;Not Available;Not Available;Not Available 2;2;2;2 66;-40;66;-40 45;-74;45;-74 Docetaxel;Cisplatin;Cisplatin;Docetaxel Not Available;Not Available;Not Available;Not Available 6;6;6;6 2;3;2;3 Not Available;75;Not Available;75 mg/m2;mg/m2;mg/m2;mg/m2 ADJUVANT;OTHER, SPECIFY IN NOTES;ADJUVANT;OTHER, SPECIFY IN NOTES Not Applicable;neo-adjuvant;Not Applicable;neo-adjuvant 2;1;2;1 IV;IV;IV;IV NO;NO;NO;NO Chemotherapy;Chemotherapy;Chemotherapy;Chemotherapy Not Available;Not Available;Not Available;Not Available Not Available;225;Not Available;225 mg/m2;mg/m2;mg/m2;mg/m2 Not Available;Not Available;Not Available;Not Available 2011;2011;2011;2011 1 Not Available NO Not Available Not Available Not Available NO Not Available NOT HISPANIC OR LATINO NA Not Available;Not Available Not Available;Not Available Not Available;Not Available Not Available;Not Available TCGA-73-4676-F12553;TCGA-73-4676-F71022 f8c9076a-23b2-4792-bcbc-c88d250d1ff3;B89F1AE8-D8B3-4B9D-9B4F-D3F9F887FFF9 18;18 Not Applicable;Not Available Not Applicable;Not Available Not Applicable;281 164;Not Available Not Applicable;Not Available 0;Unknown Not Available;Scheduled Follow-up Submission Not Available;Unknown 90;Unknown Not Available;NO 5;3 Not Available;Not Available NO;Unknown Adjuvant therapy;Unknown Not Available;WITH TUMOR YES;YES Not Available;Unknown Not Available;Not Available YES;YES Alive;Dead 2011;2015 MALE Lung Adenocarcinoma- Not Otherwise Specified (NOS) Yes C34.1 8140/3 C34.1 YES 70 NO Not Available Not Available Peripheral Lung 5 Not Available Not Available Not Available Not Available Not Available Not Available Not Available Not Available Not Available Not Available 48 No 4676 Preoperative TUMOR FREE Not Available Not Available Not Available 67 78 Not Available YES WHITE Not Available NA Primary Tumor Field TCGA-73-4676-R12555 04a28cdd-15da-414f-8481-8b42047187f6 1 2 125 Not Available Not Available 6 Not Available Not Available NO Not Available Not Applicable ADJUVANT Not Available Not Available 2011 R0 Not Applicable Not Applicable Not Applicable Not Applicable Not Applicable Not Applicable Not Applicable Not Applicable Not Applicable Stage IIA Not Applicable Not Applicable Not Applicable 7th Not Applicable Not Applicable Not Applicable M0 N1 T2a 2009 NO YES 73 4 Lung Alive 2011 2010 1977

3361 Dead T2 N0 M0 Stage IB NA 53 R-Upper Not Applicable TCGA-78-8662 16e64a1e-08b0-4c27-8c69-fa64551f8ca4 23 -19563 3361 0 Not Available Not Available Lung Adenocarcinoma 76 NA 0 Not Available NO Not Available Not Available Not Available NO Not Available Not Evaluated NA FEMALE Lung Adenocarcinoma- Not Otherwise Specified (NOS) No C34.1 8140/3 C34.1 YES Not Available NO Not Available Not Available Peripheral Lung 10 Unknown YES 3044 NO Not Available Not Available Not Available Locoregional Recurrence Biopsy with Histologic Confirmation YES 45 No 8662 Preoperative Unknown Not Available Not Available NO 64 96 Complete Remission/Response YES Not Evaluated NO NA R0 Not Applicable Not Applicable Not Applicable Not Applicable Not Applicable Not Applicable Not Applicable Not Applicable Not Applicable Stage IB Not Applicable Not Applicable Not Applicable 6th Not Applicable Not Applicable Not Applicable M0 N0 T2 Not Available NO YES 78 2 Lung Dead 2012 2000 1964

683 Alive T1a N0 MX Stage IA NA 76 R-Upper Not Applicable TCGA-93-7347 ef03654a-dcf1-41ff-a67e-4f3fbf9ba807 30 -27949 Not Applicable 0 297 Not Available Lung Adenocarcinoma 70 NA Not Available Not Available NO Not Available Not Available Not Available NO Not Available NOT HISPANIC OR LATINO NA Not Available;Not Available Not Available;Not Available Not Available;Not Available Not Available;Not Available TCGA-93-7347-F29886;TCGA-93-7347-F43155 3cb5e775-0ae6-4d83-8d2a-049f1eb2b119;1AA9F067-47F9-4CB2-837D-E9907C167259 30;14 Not Applicable;Not Applicable Not Applicable;Not Applicable Not Applicable;Not Applicable 297;683 Not Applicable;Not Applicable 0;Not Evaluated Scheduled Follow-up Submission;Scheduled Follow-up Submission Complete Remission/Response;Not Applicable 100;Not Evaluated Not Available;NO 3;5 Not Available;Not Available NO;NO Other;Not Available TUMOR FREE;TUMOR FREE NO;NO Complete Remission/Response;Complete Remission/Response Not Available;Not Available NO;NO Alive;Alive 2012;2013 FEMALE Lung Adenocarcinoma Mixed Subtype No C34.1 8255/3 C34.1 YES Not Available NO Not Available Not Available Central Lung 3 Not Available Not Available Not Available Not Available Not Available Not Available Not Available Not Available Not Available Not Available 11 No 7347 Not Available TUMOR FREE 61 84 Not Available 62 84 Not Available YES WHITE Not Available NA R0 Not Applicable Not Applicable Not Applicable Not Applicable Not Applicable Not Applicable Not Applicable Not Applicable Not Applicable Stage IA Not Applicable Not Applicable Not Applicable 7th Not Applicable Not Applicable Not Applicable MX N0 T1a 1967 YES NO 93 3 Lung Alive 2012 2011 1952

1621 Alive T1 N2 MX Stage IIIA NA 81 R-Upper Not Applicable TCGA-49-6743 a391d49f-a822-460b-981c-6fbe1868ee38 10 -29807 Not Applicable 0 369 Not Available Lung Adenocarcinoma Not Available NA TCGA-49-6743-D14859;TCGA-49-6743-D16685;TCGA-49-6743-D16684 77afb595-62de-41ce-bbd0-3e811685cfcf;faeee125-10d2-46c8-81cc-75ade012acdb;586c3a98-5004-43da-832f-73b1ec351173 Not Available;Not Available;Not Available 10;23;23 140;140;140 18;48;48 Cisplatin;Bevacizumab;Pemetrexed Not Available;Not Available;Not Available 8;9;9 4;4;4 Not Available;Not Available;Not Available Not Available;Not Available;Not Available ADJUVANT;ADJUVANT;ADJUVANT Not Applicable;Not Applicable;Not Applicable 1;1;1 IV;IV;IV NO;NO;NO Chemotherapy;Chemotherapy;Chemotherapy Not Available;Not Available;Not Available Not Available;Not Available;Not Available Not Available;Not Available;Not Available Not Available;Not Available;Not Available 2011;2011;2011 Not Available Not Available Not Available Not Available Not Available Not Available Not Available Not Available Not Available NA Not Available;Not Available Not Available;Not Available Not Available;Not Available Not Available;Not Available TCGA-49-6743-F14858;TCGA-49-6743-F71136 cd1e65ca-466c-495d-b733-aac17946d06a;74E45F53-1B39-45A5-A07E-4B3BB42BC37E 10;18 Not Applicable;Not Applicable Not Applicable;Not Applicable Not Applicable;Not Applicable 369;1621 Not Applicable;Not Applicable Not Available;Not Available Scheduled Follow-up Submission;Scheduled Follow-up Submission Complete Remission/Response;Complete Remission/Response Not Available;100 Not Available;NO 8;3 Not Available;Not Available NO;NO Not Available;Not Available TUMOR FREE;Not Available YES;YES Complete Remission/Response;Complete Remission/Response Not Available;Not Available NO;NO Alive;Alive 2011;2015 FEMALE Lung Clear Cell Adenocarcinoma No C34.1 8310/3 C34.1 YES Not Available Not Available Not Available Not Available Not Available 8 Not Available Not Available Not Available Not Available Not Available Not Available Not Available Not Available Not Available Not Available 50 No 6743 Not Available TUMOR FREE Not Available Not Available Not Available Not Available Not Available Not Available Not Available WHITE Not Available NA Not Available Not Applicable Not Applicable Not Applicable Not Applicable Not Applicable Not Applicable Not Applicable Not Applicable Not Applicable Stage IIIA Not Applicable Not Applicable Not Applicable 7th Not Applicable Not Applicable Not Applicable MX N2 T1 Not Available NO YES 49 4 Lung Alive 2011 2010 Not Available

677 Alive T3 N0 MX Stage IIB NA 46 R-Middle Not Applicable TCGA-49-6767 6bffe800-ec2b-4638-9333-97fe85dcd91c 10 -17108 Not Applicable 0 0 Not Available Lung Adenocarcinoma Not Available NA Not Available Not Available Not Available Not Available Not Available Not Available Not Available Not Available Not Available NA Not Available Not Available Not Available Not Available TCGA-49-6767-F58822 DC7C7640-FF2D-4F4E-8220-4EFFAD635D0E 24 Not Available Not Available Not Applicable 677 Not Available Unknown Scheduled Follow-up Submission Unknown Unknown NO 4 Not Available Unknown Unknown Unknown Unknown Unknown Not Available Unknown Alive 2014 FEMALE Lung Adenocarcinoma- Not Otherwise Specified (NOS) No C34.2 8140/3 C34.2 YES Not Available Not Available Not Available Not Available Not Available 8 Not Available Not Available Not Available Not Available Not Available Not Available Not Available Not Available Not Available Not Available 30 No 6767 Not Available TUMOR FREE Not Available Not Available Not Available Not Available Not Available Not Available NO WHITE Not Available NA R0 Not Applicable Not Applicable Not Applicable Not Applicable Not Applicable Not Applicable Not Applicable Not Applicable Not Applicable Stage IIB Not Applicable Not Applicable Not Applicable 7th Not Applicable Not Applicable Not Applicable MX N0 T3 Not Available NO YES 49 2 Lung Alive 2011 2010 Not Available

1379 Dead T1 N2 M0 Stage IIIA NA 53 L-Upper Not Applicable TCGA-55-6981 0a45f302-5748-48f3-9dc9-66c01843a68e 27 -19497 1379 0 Not Available Not Available Lung Adenocarcinoma Not Available NA TCGA-55-6981-D56245 C7F9FCD5-F2FB-4097-9417-19F1B5F1855B Not Available 30 Not Available 25 Chemo, NOS Complete Response 1 Not Available Not Available Not Available Not Available Not Applicable Not Available Not Available NO Chemotherapy Not Available Not Available Not Available NO 2014 Not Available Not Available NO Not Available Not Available Not Available NO Not Available NOT HISPANIC OR LATINO NA Not Available Not Available Not Available Not Available TCGA-55-6981-F56243 1E30CFED-13C2-4A05-B32F-1A96D4DE789B 30 Not Applicable Not Applicable 1379 Not Available Not Applicable Unknown Scheduled Follow-up Submission Complete Remission/Response Unknown NO 1 Not Available NO Not Available TUMOR FREE YES Complete Remission/Response Not Available YES Dead 2014 FEMALE Lung Adenocarcinoma- Not Otherwise Specified (NOS) No C34.1 8140/3 C34.1 YES Not Available NO Not Available Not Available Not Available 7 Not Available Not Available Not Available Not Available Not Available Not Available Not Available Not Available Not Available Not Available Not Available No 6981 Not Available TUMOR FREE Not Available Not Available Not Available Not Available Not Available Not Available Not Available WHITE Not Available NA Primary Tumor Field TCGA-55-6981-R56244 524B09AA-4C5A-48CA-B8B5-92CC0C233997 Not Available 30 114 65 Complete Response 1 34 6120 NO External Not Applicable Not Available Not Available cGy 2014 R0 Not Applicable Not Applicable Not Applicable Not Applicable Not Applicable Not Applicable Not Applicable Not Applicable Not Applicable Stage IIIA Not Applicable Not Applicable Not Applicable 6th Not Applicable Not Applicable Not Applicable M0 N2 T1 Not Available NO YES 55 3 Lung Dead 2011 2005 Not Available

1126 Alive T2 N0 M0 Stage IB NA 58 R-Upper Not Applicable TCGA-05-4390 b8475929-2d9d-4909-bd62-59684a140bd7 22 -21430 Not Applicable 0 1126 Not Available Lung Adenocarcinoma Not Available NA TCGA-05-4390-D36457;TCGA-05-4390-D36458 1C563458-F78C-43FA-9FD0-9F31F386B0D7;E9C5A69B-2C5F-40D1-9CE9-46F808524536 Not Available;Not Available 5;5 92;92 61;61 Cisplatin;Vinorelbine Complete Response;Complete Response 12;12 Not Available;Not Available Not Available;Not Available Not Available;Not Available Not Available;Not Available Not Applicable;Not Applicable Not Available;Not Available Not Available;Not Available NO;NO Chemotherapy;Chemotherapy Not Available;Not Available Not Available;Not Available Not Available;Not Available NO;NO 2012;2012 Not Available Not Available Not Available Not Available Not Available Not Available Not Available Not Available Not Available NA Unknown Unknown Not Available Not Available TCGA-05-4390-F36456 732D9E3F-5FEB-41B6-BE2A-142253ED6110 31 Not Available Not Available Not Applicable 1126 395 Not Available Scheduled Follow-up Submission Complete Remission/Response Not Available NO 10 Not Available YES Not Available TUMOR FREE YES Progressive Disease Not Available NO Alive 2012 FEMALE Lung Adenocarcinoma Mixed Subtype No C34.1 8255/3 C34.1 YES Not Available Not Available Not Available Not Available Not Available 7 Not Available Not Available Not Available Not Available Not Available Not Available Not Available Not Available Not Available Not Available 15 No 4390 Not Available TUMOR FREE Not Available Not Available Not Available Not Available Not Available Not Available Not Available Not Available Not Available NA R0 Not Applicable Not Applicable Not Applicable Not Applicable Not Applicable Not Applicable Not Applicable Not Applicable Not Applicable Stage IB Not Applicable Not Applicable Not Applicable 5th Not Applicable Not Applicable Not Applicable M0 N0 T2 1996 NO YES 05 4 Lung Alive 2010 2005 1966

947 Alive T2 N0 M1 Stage IV NA 66 R-Upper Not Applicable TCGA-86-7701 a3f6bc1c-19ab-4eeb-a9ac-3d2fac850bde 18 -24209 Not Applicable 0 11 Not Available Lung Adenocarcinoma Not Available NA TCGA-86-7701-D36483;TCGA-86-7701-D36484;TCGA-86-7701-D58402;TCGA-86-7701-D58403;TCGA-86-7701-D58404;TCGA-86-7701-D58405;TCGA-86-7701-D58407;TCGA-86-7701-D58408 EC1417C7-2349-4F98-939F-E7E643F30F87;24A17B71-0E0B-4803-A21C-02BCB336024D;08D438DF-4B04-42CE-92EB-6AAC1EF6952E;7741F434-FD67-473C-B872-5A2B21734ED8;444CAEFF-EDEE-460D-9C88-C49C232A90DB;D7831CC1-B080-4F51-A2C1-3A32B60AA83F;8C75D7CE-9FC9-4BFF-B261-DD159CFED86F;DC37E270-9E05-421E-A7F4-8B20DF86DC92 Not Available;Not Available;Tyrosine kinase inhibitor;Not Available;Not Available;Not Available;Not Available;Not Available 31;31;9;9;9;9;9;9 207;207;601;601;601;Not Available;Not Available;Not Available 27;27;454;454;454;706;936;936 Etoposide;Cisplatin;Not Available;Docetaxel;Zoledronic acid;Zoledronic acid;Gemcitabine;Docetaxel Complete Response;Complete Response;Stable Disease;Stable Disease;Stable Disease;Not Applicable;Not Applicable;Not Applicable 10;10;4;4;4;4;4;4 Not Available;Not Available;Not Available;Not Available;Not Available;Not Available;Not Available;Not Available Not Available;Not Available;Not Available;Not Available;Not Available;Not Available;Not Available;Not Available Not Available;Not Available;Not Available;Not Available;Not Available;Not Available;Not Available;Not Available Not Available;Not Available;Not Available;Not Available;Not Available;Not Available;Not Available;Not Available Not Applicable;Not Applicable;Not Applicable;Not Applicable;Not Applicable;Not Applicable;Not Applicable;Not Applicable Not Available;Not Available;Not Available;Not Available;Not Available;Not Available;Not Available;Not Available Not Available;Not Available;Not Available;Not Available;Not Available;Not Available;Not Available;Not Available NO;NO;NO;NO;NO;YES;YES;YES Chemotherapy;Chemotherapy;Chemotherapy;Chemotherapy;Ancillary;Ancillary;Chemotherapy;Chemotherapy Not Available;Not Available;Not Available;Not Available;Not Available;Not Available;Not Available;Not Available Not Available;Not Available;Not Available;Not Available;Not Available;Not Available;Not Available;Not Available Not Available;Not Available;Not Available;Not Available;Not Available;Not Available;Not Available;Not Available NO;NO;YES;NO;NO;NO;NO;NO 2012;2012;2014;2014;2014;2014;2014;2014 2 Not Available Not Available Not Available Not Available Not Available Not Available Not Available NOT HISPANIC OR LATINO NA YES;YES NO;YES Not Available;Not Available NO;NO TCGA-86-7701-F36476;TCGA-86-7701-F58406 277C6CF8-C341-45A1-AC77-78CA122BDD86;212F60F8-9A3F-4256-BC3A-C5150373B1E7 31;9 Not Available;Not Available Not Available;Not Available Not Applicable;Not Applicable 474;947 424;900 0;2 Scheduled Follow-up Submission;Scheduled Follow-up Submission Stable Disease;Progressive Disease 100;80 NO;NO 10;4 Distant Metastasis;Distant Metastasis YES;YES Post-Adjuvant Therapy;Post-Adjuvant Therapy WITH TUMOR;WITH TUMOR YES;YES Complete Remission/Response;Complete Remission/Response Convincing Imaging;Not Available NO;NO Alive;Alive 2012;2014 MALE Lung Adenocarcinoma- Not Otherwise Specified (NOS) No C34.1 8140/3 C34.1 YES 80 Not Available Not Available Not Available Peripheral Lung 1 Not Available Not Available Not Available Not Available Not Available Not Available Not Available Not Available Not Available Not Available Not Available No 7701 Not Available TUMOR FREE Not Available Not Available Not Available Not Available Not Available Not Available NO WHITE Not Available NA Distant Recurrence TCGA-86-7701-R58409 248F5702-58CA-4E1B-BC44-BCAC81B476C1 Not Available 9 929 917 Radiographic Progressive Disease 4 13 29 NO External Not Applicable Not Available Not Available Gy 2014 R0 Not Applicable Not Applicable Not Applicable Not Applicable Not Applicable Not Applicable Not Applicable Not Applicable Not Applicable Stage IV Not Applicable Not Applicable Not Applicable 7th Not Applicable Not Applicable Not Applicable M1 N0 T2 Not Available YES NO 86 1 Lung Alive 2012 2011 Not Available

186 Alive T2a N0 MX Stage IB NA 77 L-Lower Not Applicable TCGA-93-8067 bbe88801-34f3-46d2-bbfd-b46c3901ed71 18 -28453 Not Applicable 0 186 Not Available Lung Adenocarcinoma 47 NA 2 Not Available NO Not Available Not Available Not Available NO Not Available NOT HISPANIC OR LATINO NA Not Available Not Available Not Available Not Available TCGA-93-8067-F32193 88DF8B42-F107-47B4-84A7-2E62FE30E870 18 Not Applicable Not Applicable Not Applicable 186 Not Applicable 2 Scheduled Follow-up Submission Complete Remission/Response 80 NO 5 Not Available NO Preoperative TUMOR FREE NO Complete Remission/Response Not Available NO Alive 2012 MALE Lung Adenocarcinoma- Not Otherwise Specified (NOS) No C34.3 8140/3 C34.3 YES 80 NO Not Available Not Available Peripheral Lung 5 Not Available Not Available Not Available Not Available Not Available Not Available Not Available Not Available Not Available NO 20 Yes, History of Prior Malignancy 8067 Preoperative TUMOR FREE 94 103 NO 89 94 Complete Remission/Response YES ASIAN NO NA RX Not Applicable Not Applicable Not Applicable Not Applicable Not Applicable Not Applicable Not Applicable Not Applicable Not Applicable Stage IB Not Applicable Not Applicable Not Applicable 7th Not Applicable Not Applicable Not Applicable MX N0 T2a 2001 YES NO 93 4 Lung Alive 2012 2011 1961

596 Alive T1a N0 MX Stage IA NA 70 R-Upper Not Applicable TCGA-55-A492 39AB7174-5707-498D-9959-523680E04198 21 -25719 Not Applicable 0 1 Not Available Lung Adenocarcinoma 52 NA Not Evaluated Not Available NO Not Available Not Available Not Available NO Not Available NOT HISPANIC OR LATINO NA Not Available Not Available Not Available Not Available TCGA-55-A492-F60135 98B54E54-A0F9-4C7A-A526-F632A36A972D 29 Not Applicable Not Applicable Not Applicable 596 Not Applicable 2 Scheduled Follow-up Submission Complete Remission/Response 80 NO 5 Not Available NO Other TUMOR FREE NO Complete Remission/Response Not Available NO Alive 2014 FEMALE Lung Adenocarcinoma- Not Otherwise Specified (NOS) No C34.1 8140/3 C34.1 YES Not Evaluated NO Not Available Not Available Unknown 2 Not Available Not Available Not Available Not Available Not Available Not Available Not Available Not Available Not Available NO 35 Yes, History of Prior Malignancy A492 Not Available TUMOR FREE Not Available 92 Unknown Not Available 95 Unknown YES WHITE Unknown NA R0 Not Applicable Not Applicable Not Applicable Not Applicable Not Applicable Not Applicable Not Applicable Not Applicable Not Applicable Stage IA Not Applicable Not Applicable Not Applicable 7th Not Applicable Not Applicable Not Applicable MX N0 T1a 1995 YES NO 55 3 Lung Alive 2013 2012 1962

408 Alive T2a N0 MX Stage IB NA 71 R-Upper Not Applicable TCGA-69-7979 a5bd7d50-9c14-49e6-89cf-6bf440c42309 9 -26180 Not Applicable 0 89 Not Available Lung Adenocarcinoma Not Available NA Not Available Not Available NO Not Available Not Available Not Available NO Not Available NOT HISPANIC OR LATINO NA Not Available;Not Available Not Available;Not Available Not Available;Not Available Not Available;Not Available TCGA-69-7979-F39913;TCGA-69-7979-F40026 F557D080-A35A-4FC9-A673-5055389DCCF4;E05A8EC6-B473-4218-A7AF-9E5D2027CA4C 11;19 Not Applicable;Not Available Not Applicable;Not Available Not Applicable;Not Applicable 89;408 Not Applicable;Not Available Not Evaluated;Not Available Scheduled Follow-up Submission;Not Available Stable Disease;Unknown Unknown;Not Available NO;Not Available 2;3 Not Available;Not Available NO;Unknown Not Evaluated;Not Available TUMOR FREE;Not Available NO;Not Available Stable Disease;Not Available Not Available;Not Available NO;Not Available Alive;Alive 2013;2014 FEMALE Lung Acinar Adenocarcinoma No C34.1 8550/3 C34.1 YES Not Available NO Not Available Not Available Not Available 4 Not Available Not Available Not Available Not Available Not Available Not Available Not Available Not Available Not Available Not Available 28 No 7979 Not Available Not Available 71 64 Not Available 70 59 Not Available YES WHITE Not Available NA Not Available Not Applicable Not Applicable Not Applicable Not Applicable Not Applicable Not Applicable Not Applicable Not Applicable Not Applicable Stage IB Not Applicable Not Applicable Not Applicable 7th Not Applicable Not Applicable Not Applicable MX N0 T2a Not Available NO YES 69 2 Lung Alive 2012 2011 1955

670 Alive T2a N0 M0 Stage IB NA 54 R-Upper Not Applicable TCGA-55-7576 055e9a43-3c00-4110-a303-e4407676bec0 11 -19917 Not Applicable 0 40 Not Available Lung Adenocarcinoma Not Available NA TCGA-55-7576-D45604;TCGA-55-7576-D45605 081CF755-293A-4A75-8F90-BC270CBB0C58;12803CED-7A32-4FB2-B0BB-7351A5C61BA7 Not Available;Not Available 17;17 168;168 104;104 Alimta;Cisplatin Complete Response;Complete Response 7;7 Not Available;Not Available Not Available;Not Available Not Available;Not Available Not Available;Not Available Not Applicable;Not Applicable Not Available;Not Available Not Available;Not Available NO;NO Chemotherapy;Chemotherapy Not Available;Not Available Not Available;Not Available Not Available;Not Available NO;NO 2013;2013 Not Available Not Available Not Available Not Available Not Available Not Available Not Available Not Available NOT HISPANIC OR LATINO NA Not Available Not Available Not Available Not Available TCGA-55-7576-F45603 062163B2-5EE5-4AC5-A2C1-AF02D4B0CD90 17 Not Applicable Not Applicable Not Applicable 670 Not Applicable Unknown Scheduled Follow-up Submission Complete Remission/Response Unknown NO 7 Not Available NO Unknown TUMOR FREE YES Complete Remission/Response Not Available NO Alive 2013 MALE Lung Adenocarcinoma- Not Otherwise Specified (NOS) No C34.1 8140/3 C34.1 YES Not Available Not Available Not Available Not Available Not Available 1 Not Available Not Available Not Available Not Available Not Available Not Available Not Available Not Available Not Available Not Available Not Available No 7576 Not Available TUMOR FREE Not Available Not Available Not Available Not Available Not Available Not Available Not Available BLACK OR AFRICAN AMERICAN Not Available NA R0 Not Applicable Not Applicable Not Applicable Not Applicable Not Applicable Not Applicable Not Applicable Not Applicable Not Applicable Stage IB Not Applicable Not Applicable Not Applicable 7th Not Applicable Not Applicable Not Applicable M0 N0 T2a Not Available YES NO 55 2 Lung Alive 2012 2011 1972

863 Alive T2a N0 Stage IB NA 51 R-Lower Not Applicable TCGA-44-5644 1fc81cd4-fa89-4135-8c3c-027ffae82b05 12 -18644 Not Applicable 0 498 Not Available Lung Adenocarcinoma 61 NA Not Available Not Available Not Available Not Available Not Available Not Available Not Available Not Available NOT HISPANIC OR LATINO NA Not Available;Not Available Not Available;Not Available Not Available;NO Not Available;Not Available TCGA-44-5644-F17419;TCGA-44-5644-F38817 002c588c-531f-435b-98a5-bcd8255fb0c1;A4C3F323-E35C-4654-9855-2092454AAB32 12;26 Not Applicable;Not Applicable Not Applicable;Not Applicable Not Applicable;Not Applicable 498;863 Not Applicable;Not Applicable Not Available;Not Available Scheduled Follow-up Submission;Scheduled Follow-up Submission Complete Remission/Response;Complete Remission/Response Not Available;Not Available Not Available;NO 10;12 Not Available;Not Available NO;NO Not Available;Not Available TUMOR FREE;TUMOR FREE NO;NO Complete Remission/Response;Complete Remission/Response Not Available;Not Available NO;NO Alive;Alive 2011;2012 FEMALE Lung Adenocarcinoma- Not Otherwise Specified (NOS) No C34.3 8140/3 C34.3 YES Not Available Not Available Not Available Not Available Not Available 10 Not Available Not Available Not Available Not Available Not Available Not Available Not Available Not Available Not Available Not Available 17.5 No 5644 Not Available TUMOR FREE Not Available Not Available Not Available 103 59 Not Available YES WHITE Not Available NA Not Available Not Applicable Not Applicable Not Applicable Not Applicable Not Applicable Not Applicable Not Applicable Not Applicable Not Applicable Stage IB Not Applicable Not Applicable Not Applicable 7th Not Applicable Not Applicable Not Applicable Not Available N0 T2a Not Available YES NO 44 2 Lung Alive 2011 2010 1975

574 Dead T1b N1 MX Stage IIA NA 59 R-Upper Not Applicable TCGA-44-7669 4b22ad71-c585-4077-ad3c-c6e026d0c7fa 21 -21893 574 0 Not Available Not Available Lung Adenocarcinoma 52 NA TCGA-44-7669-D20477;TCGA-44-7669-D20479;TCGA-44-7669-D20476;TCGA-44-7669-D20478 8187bd18-8f5a-4de2-9aa1-fc17056a991c;600924bb-9e59-41f0-9493-647a4c8d40e8;c6e5598b-8c2a-45db-9833-9ca073f0c347;96593f12-a433-4978-8449-36f7fb287d10 Not Available;Not Available;Not Available;Not Available 13;13;13;13 180;539;180;539 117;539;117;539 Alimta;Carboplatin;Cisplatin;Gemzar Not Available;Not Available;Not Available;Not Available 1;1;1;1 4;1;4;1 975;460;145;1900 mg;mg;mg;mg ADJUVANT;PALLIATIVE;ADJUVANT;PALLIATIVE Not Applicable;Not Applicable;Not Applicable;Not Applicable 1;2;1;2 IV;IV;IV;IV NO;NO;NO;NO Chemotherapy;Chemotherapy;Chemotherapy;Chemotherapy Not Available;Not Available;Not Available;Not Available 3900;460;580;1900 mg;mg;mg;mg Not Available;Not Available;Not Available;Not Available 2012;2012;2012;2012 Not Available Not Available Not Available Not Available Not Available Not Available Not Available Not Available NOT HISPANIC OR LATINO NA NO;NO;YES NO;YES;YES NO;Not Available;NO YES;NO;NO TCGA-44-7669-F20203;TCGA-44-7669-F20504;TCGA-44-7669-F20505 acda688e-dc06-46a1-b242-5151d5db16d9;669a7ac9-cbe5-43c2-950e-2fb09904248f;2f13835e-7692-473a-8e9c-204fc08242a0 6;16;16 Not Available;Not Available;Not Available 390;Not Available;Not Available 574;574;574 Not Available;Not Available;Not Available 390;418;525 4;Not Available;Not Available Scheduled Follow-up Submission;Additional New Tumor Event;Additional New Tumor Event Progressive Disease;Progressive Disease;Progressive Disease Not Available;Not Available;Not Available Not Available;Not Available;Not Available 1;1;1 Not Available;Not Available;Not Available YES;YES;YES Post-Adjuvant Therapy;Not Available;Not Available WITH TUMOR;WITH TUMOR;WITH TUMOR YES;Not Available;Not Available Complete Remission/Response;Not Available;Not Available Not Available;Not Available;Not Available NO;Not Available;Not Available Dead;Dead;Dead 2012;2012;2012 MALE Lung Adenocarcinoma- Not Otherwise Specified (NOS) No C34.1 8140/3 C34.1 YES Not Available NO Not Available Not Available Not Available 12 Not Available Not Available Not Available Not Available Not Available Not Available Not Available Not Available Not Available Not Available 20 No 7669 Not Available WITH TUMOR 84 80 Not Available 80 73 Not Available YES BLACK OR AFRICAN AMERICAN Not Available NA Distant site;Distant Recurrence TCGA-44-7669-R20489;TCGA-44-7669-R20506 1c8ca0de-06fc-4cc4-8e81-0b2c8f09778a;8fad2a3d-2f24-4766-9878-9a7767a87e16 1;2 16;16 474;532 474;526 Not Available;Not Available 1;1 1;5 2000;2000 NO;NO OTHER;EXTERNAL BEAM Cyberknife;Not Applicable PROGRESSION;PALLIATIVE Not Available;Not Available cGy;cGy 2012;2012 Not Available Not Applicable Not Applicable Not Applicable Not Applicable Not Applicable Not Applicable Not Applicable Not Applicable Not Applicable Stage IIA Not Applicable Not Applicable Not Applicable 7th Not Applicable Not Applicable Not Applicable MX N1 T1b Not Available YES NO 44 2 Lung Dead 2011 2010 1970

882 Alive T1b N1 M0 Stage IIA NA 47 L-Upper Not Applicable TCGA-44-7670 9b38eded-3f46-4aaa-9991-68008d97bdbe 18 -17392 Not Applicable 0 531 Not Available Lung Adenocarcinoma 50 NA TCGA-44-7670-D20715;TCGA-44-7670-D20716 cd9d0292-629c-4b64-976d-92921bc73acf;98b04e0c-0fb1-4f87-97fc-97aed384b749 Not Available;Not Available 19;19 165;165 88;88 Docetaxel;Cisplatin Not Available;Not Available 1;1 4;4 100-120;98-125 mg;mg ADJUVANT;ADJUVANT Not Applicable;Not Applicable 1;1 IV;IV NO;NO Chemotherapy;Chemotherapy Not Available;Not Available 420;419 mg;mg Not Available;Not Available 2012;2012 Not Available Not Available Not Available Not Available Not Available Not Available Not Available Not Available HISPANIC OR LATINO NA Not Available;Not Available Not Available;Not Available Not Available;NO Not Available;Not Available TCGA-44-7670-F20714;TCGA-44-7670-F39052 58360ec4-71c7-4e83-bec3-c8948c4dcff4;AC8BF10A-8F00-4860-B41E-5D9CCAA18829 19;9 Not Applicable;Not Applicable Not Applicable;Not Applicable Not Applicable;Not Applicable 531;882 Not Applicable;Not Applicable Not Available;Not Available Scheduled Follow-up Submission;Scheduled Follow-up Submission Complete Remission/Response;Complete Remission/Response Not Available;Not Available Not Available;NO 1;1 Not Available;Not Available NO;NO Not Available;Not Available TUMOR FREE;TUMOR FREE YES;YES Complete Remission/Response;Complete Remission/Response Not Available;Not Available NO;NO Alive;Alive 2012;2013 FEMALE Lung Adenocarcinoma- Not Otherwise Specified (NOS) No C34.1 8140/3 C34.1 YES Not Available Not Available Not Available Not Available Not Available 1 Not Available Not Available Not Available Not Available Not Available Not Available Not Available Not Available Not Available Not Available 30 Yes 7670 Not Available TUMOR FREE 90 82 Not Available 86 80 Not Available YES WHITE Not Available NA Not Available Not Applicable Not Applicable Not Applicable Not Applicable Not Applicable Not Applicable Not Applicable Not Applicable Not Applicable Stage IIA Not Applicable Not Applicable Not Applicable 7th Not Applicable Not Applicable Not Applicable M0 N1 T1b Not Available YES NO 44 2 Lung Alive 2012 2010 1980

913 Alive T3 N0 M0 Stage IIB NA 70 R-Upper Not Applicable TCGA-05-4424 66763a0c-6cda-4832-a0cc-e7b496d78eaa 22 -25689 Not Applicable 0 913 Not Available Lung Adenocarcinoma Not Available NA TCGA-05-4424-D36537 65585949-E6ED-42E6-B1D5-002FBB849BB1 Not Available 5 Not Available 699 Erlotinib Not Applicable 12 Not Available Not Available Not Available Not Available Not Applicable Not Available Not Available YES Immunotherapy Not Available Not Available Not Available NO 2012 Not Available Not Available Not Available Not Available Not Available Not Available Not Available Not Available Not Available NA Unknown YES Not Available Not Available TCGA-05-4424-F36536 97188E69-9925-4A74-B986-E22FD997CD52 1 Not Available Not Available Not Applicable 913 153 Not Available Scheduled Follow-up Submission Partial Remission/Response Not Available NO 11 Distant Metastasis YES Not Available WITH TUMOR YES Progressive Disease Not Available YES Alive 2012 MALE Lung Adenocarcinoma Mixed Subtype No C34.1 8255/3 C34.1 YES Not Available Not Available Not Available Not Available Not Available 7 Not Available Not Available Not Available Not Available Not Available Not Available Not Available Not Available Not Available Not Available 50 No 4424 Not Available WITH TUMOR Not Available Not Available Not Available Not Available Not Available Not Available Not Available Not Available Not Available NA Primary Tumor Field;Distant Recurrence;Distant Recurrence TCGA-05-4424-R36538;TCGA-05-4424-R36539;TCGA-05-4424-R36540 2FB387B1-1AE0-442B-BB82-D85638F7A0E0;D458480E-E0FB-4E6B-85DC-CBB28101BE78;A5534445-62B5-4F9F-983B-F42DCE652083 Not Available;Not Available;Not Available 1;1;1 30;Not Available;Not Available 30;153;699 Partial Response;Partial Response;Partial Response 11;11;11 Not Available;Not Available;Not Available 60;3;30 NO;YES;YES External;Unknown;Unknown Not Applicable;Not Applicable;Not Applicable Not Available;Not Available;Not Available Not Available;Not Available;Not Available Gy;Gy;Gy 2012;2012;2012 R1 Not Applicable Not Applicable Not Applicable Not Applicable Not Applicable Not Applicable Not Applicable Not Applicable Not Applicable Stage IIB Not Applicable Not Applicable Not Applicable 6th Not Applicable Not Applicable Not Applicable M0 N0 T3 2007 NO YES 05 4 Lung Alive 2010 2008 1957

28 Alive T2a N0 M0 Stage IB NA 54 R-Upper Not Applicable TCGA-55-A493 E43A2B72-35C6-4466-9007-CE96CAF77EA8 21 -20027 Not Applicable 0 28 Not Available Lung Adenocarcinoma 102 NA Not Evaluated Not Available NO Not Available Not Available Not Available NO Not Available NOT HISPANIC OR LATINO NA FEMALE Lung Adenocarcinoma- Not Otherwise Specified (NOS) No C34.1 8140/3 C34.1 YES Not Evaluated NO Not Available Not Available Unknown 2 Not Available Not Available Not Available Not Available Not Available Not Available Not Available Not Available Not Available NO 53 No A493 Not Evaluated TUMOR FREE 71 2 Unknown 65 2 Unknown NO WHITE Unknown NA R0 Not Applicable Not Applicable Not Applicable Not Applicable Not Applicable Not Applicable Not Applicable Not Applicable Not Applicable Stage IB Not Applicable Not Applicable Not Applicable 7th Not Applicable Not Applicable Not Applicable M0 N0 T2a Not Available YES NO 55 2 Lung Alive 2013 2012 1977

949 Alive T2a N1 M0 Stage IIA NA 46 R-Upper Not Applicable TCGA-86-8279 2923e404-38f2-437a-b57e-23401fbe0273 6 -17032 Not Applicable 0 21 Not Available Lung Adenocarcinoma Not Available NA TCGA-86-8279-D41676;TCGA-86-8279-D41677 9F2065E1-C62F-4D2A-92CB-B1DB2F7963E2;233E4E3A-65B3-467B-B214-C52773DD2094 Not Available;Not Available 25;25 119;119 28;28 Etoposide;Cisplatin Complete Response;Complete Response 3;3 Not Available;Not Available Not Available;Not Available Not Available;Not Available Not Available;Not Available Not Applicable;Not Applicable Not Available;Not Available Not Available;Not Available NO;NO Chemotherapy;Chemotherapy Not Available;Not Available Not Available;Not Available Not Available;Not Available NO;NO 2013;2013 2 Not Available Not Available Not Available Not Available Not Available Not Available Not Available NOT HISPANIC OR LATINO NA Not Available;Not Available Not Available;Not Available Not Available;Not Available Not Available;Not Available TCGA-86-8279-F41673;TCGA-86-8279-F59731 56E63750-3C49-4C3E-8586-0497C56C4A45;2203355D-451D-42E6-A83A-A8D2CC685DC5 25;20 Not Applicable;Not Applicable Not Applicable;Not Applicable Not Applicable;Not Applicable 482;949 Not Applicable;Not Applicable 1;1 Scheduled Follow-up Submission;Scheduled Follow-up Submission Complete Remission/Response;Complete Remission/Response 90;90 NO;NO 3;5 Not Available;Not Available NO;NO Pre-Adjuvant Therapy;Preoperative TUMOR FREE;TUMOR FREE YES;YES Complete Remission/Response;Complete Remission/Response Not Available;Not Available YES;YES Alive;Alive 2013;2014 MALE Lung Adenocarcinoma- Not Otherwise Specified (NOS) No C34.1 8140/3 C34.1 YES 80 NO Not Available Not Available Peripheral Lung 6 Not Available Not Available Not Available Not Available Not Available Not Available Not Available Not Available Not Available Unknown Not Available No 8279 Preoperative TUMOR FREE Not Available Not Available YES Not Available Not Available Complete Remission/Response NO WHITE YES NA Primary Tumor Field TCGA-86-8279-R41678 8C483E9F-5DD2-4828-91A6-F81F0A420544 Not Available 25 208 139 Complete Response 3 33 66 NO External Not Applicable Not Available Not Available Gy 2013 R0 Not Applicable Not Applicable Not Applicable Not Applicable Not Applicable Not Applicable Not Applicable Not Applicable Not Applicable Stage IIA Not Applicable Not Applicable Not Applicable 7th Not Applicable Not Applicable Not Applicable M0 N1 T2a Not Available YES NO 86 1 Lung Alive 2012 2011 Not Available

1130 Alive T2 N2 M0 Stage IIIA NA 74 Discrepancy Not Available TCGA-44-3396 3bd6badb-27ff-4d8d-b206-4d28dc264862 18 -27073 Not Applicable 0 311 Not Available Lung Adenocarcinoma 48 NA TCGA-44-3396-D5017;TCGA-44-3396-D5019 cb7c2370-e79f-4706-9c22-02b0152ffc04;d9d59d53-accc-4e89-bc4c-c13fde7550f1 Not Available;Not Available 22;22 192;192 129;129 Alimta;Carboplatin Not Available;Not Available 10;10 4;4 850;600 mg;mg ADJUVANT;ADJUVANT Not Applicable;Not Applicable 1;1 IV;IV NO;NO Chemotherapy;Chemotherapy Not Available;Not Available 3400;2100 mg;mg Not Available;Not Available 2010;2010 1 Not Available Not Available Not Available Not Available Not Available Not Available Not Available NOT HISPANIC OR LATINO NA Not Available;Not Available Not Available;Not Available Not Available;Not Available Not Available;Not Available TCGA-44-3396-F5016;TCGA-44-3396-F39080 5e3024c8-c8ce-42c6-bfec-8fbe87e3146a;65BCFB65-301A-471C-9683-5088DE18ED24 22;11 Not Applicable;Not Applicable Not Applicable;Not Applicable Not Applicable;Not Applicable 411;1130 Not Applicable;Not Applicable 1;Not Available Not Available;Scheduled Follow-up Submission Complete Remission/Response;Complete Remission/Response Not Available;80 Not Available;NO 10;1 Not Available;Not Available NO;NO Adjuvant therapy;Other TUMOR FREE;TUMOR FREE YES;YES Complete Remission/Response;Complete Remission/Response Not Available;Not Available NO;NO Alive;Alive 2010;2013 FEMALE Lung Adenocarcinoma- Not Otherwise Specified (NOS) No C34.1 8140/3 C34.1 YES Not Available NO Not Available Not Available Not Available 10 Not Available Not Available Not Available Not Available Not Available Not Available Not Available Not Available Not Available Not Available 50 No 3396 Other TUMOR FREE Not Available Not Available Not Available 98 89 Not Available YES WHITE Not Available NA R0 Not Applicable Not Applicable Not Applicable Not Applicable Not Applicable Not Applicable Not Applicable Not Applicable Not Applicable Stage IIIA Not Applicable Not Applicable Not Applicable 7th Not Applicable Not Applicable Not Applicable M0 N2 T2 Not Available YES NO 44 2 Lung Alive 2010 2009 1959

1498 Dead T2 N2 M0 Stage IIIA NA 73 R-Upper Not Applicable TCGA-62-8402 433a32d0-93d1-4897-8e36-beb1ec1d998e 4 -26934 Not Applicable 0 1445 Not Available Lung Adenocarcinoma Not Available NA 0 Not Available YES Exon 19 Deletion Not Available Not Available NO Not Available NOT HISPANIC OR LATINO NA YES YES Not Available NO TCGA-62-8402-F52547 08295B1A-EE42-42DD-9431-741E62382F2E 4 Not Available Not Available 1498 Not Available 772 0 Scheduled Follow-up Submission Progressive Disease 100 NO 12 Distant Metastasis YES Preoperative WITH TUMOR NO Complete Remission/Response Convincing Imaging NO Dead 2013 FEMALE Lung Adenocarcinoma Mixed Subtype No C34.1 8255/3 C34.1 YES 100 NO Not Available Not Available Peripheral Lung 7 YES YES 772 NO Not Available NO Not Available Locoregional Recurrence;Distant Metastasis Convincing Imaging YES Not Available No 8402 Preoperative WITH TUMOR Not Available Not Available Unknown 80 134 Complete Remission/Response YES WHITE YES NA Regional site TCGA-62-8402-R33812 181E46B6-E825-45DA-A720-020A04CA720F Not Available 19 73 28 Complete Response 7 25 50 NO External Not Applicable Not Available Not Available Gy 2012 R0 Not Applicable Not Applicable Not Applicable Not Applicable Not Applicable Not Applicable Not Applicable Not Applicable Not Applicable Stage IIIA Not Applicable Not Applicable Not Applicable 6th Not Applicable Not Applicable Not Applicable M0 N2 T2 Not Available NO YES 62 1 Lung Alive 2012 2008 Not Available

1864 Alive T1 N0 MX Stage IA NA 59 Discrepancy Not Available TCGA-44-6778 5d5cc436-6e57-4d23-a164-7f5153c2e666 31 -21725 Not Applicable 0 1110 Not Available Lung Adenocarcinoma 53 NA Not Available Not Available Not Available Not Available Not Available Not Available Not Available Not Available Not Available NA Not Available;Not Available Not Available;Not Available Not Available;Not Available Not Available;Not Available TCGA-44-6778-F15765;TCGA-44-6778-F39376 cdcf76b6-e93b-4d22-8d67-6a137b04617f;E7E66030-97D0-480D-853E-39F85ED1F874 31;21 Not Applicable;Not Applicable Not Applicable;Not Applicable Not Applicable;Not Applicable 1110;1864 Not Applicable;Not Applicable Not Available;Not Available Scheduled Follow-up Submission;Scheduled Follow-up Submission Complete Remission/Response;Complete Remission/Response Not Available;Not Available Not Available;NO 8;1 Not Available;Not Available NO;NO Not Available;Not Available TUMOR FREE;TUMOR FREE NO;NO Complete Remission/Response;Complete Remission/Response Not Available;Not Available NO;NO Alive;Alive 2011;2013 MALE Lung Adenocarcinoma- Not Otherwise Specified (NOS) No C34.1 8140/3 C34.9 YES Not Available Not Available Not Available Not Available Not Available 8 Not Available Not Available Not Available Not Available Not Available Not Available Not Available Not Available Not Available Not Available 15 No 6778 Not Available TUMOR FREE Not Available Not Available Not Available 90 74 Not Available YES BLACK OR AFRICAN AMERICAN Not Available NA Not Available Not Applicable Not Applicable Not Applicable Not Applicable Not Applicable Not Applicable Not Applicable Not Applicable Not Applicable Stage IA Not Applicable Not Applicable Not Applicable 6th Not Applicable Not Applicable Not Applicable MX N0 T1 2004 NO YES 44 4 Lung Alive 2011 2007 1974

2248 Alive T2 N0 M0 Stage IB NA 76 L-Upper Not Applicable TCGA-MP-A5C7 8F04660A-2EFB-4947-9EAB-375066B9EB33 10 -28015 Not Applicable 0 1490 Not Available Lung Adenocarcinoma 105 NA 1 Not Available NO Not Available Not Available Not Available NO Not Available NOT HISPANIC OR LATINO NA Not Available;Not Available Not Available;Not Available Not Available;Not Available Not Available;Not Available TCGA-MP-A5C7-F54544;TCGA-MP-A5C7-F67079 2777977A-E3E5-4AFD-AE85-CCC79DF0E736;A86ED60C-DDCE-4E9A-9B2D-9888E9F12C9F 19;30 Not Applicable;Not Applicable Not Applicable;Not Applicable Not Applicable;Not Applicable 1862;2248 Not Applicable;Not Applicable Not Evaluated;1 Scheduled Follow-up Submission;Scheduled Follow-up Submission Complete Remission/Response;Complete Remission/Response Not Evaluated;Not Evaluated NO;NO 12;10 Not Available;Not Available NO;NO Not Available;Other TUMOR FREE;TUMOR FREE NO;NO Complete Remission/Response;Complete Remission/Response Not Available;Not Available NO;NO Alive;Alive 2013;2014 FEMALE Lung Adenocarcinoma- Not Otherwise Specified (NOS) No C34.1 8140/3 C34.1 YES Not Evaluated NO Not Available Not Available Central Lung 5 Not Available Not Available Not Available Not Available Not Available Not Available Not Available Not Available Not Available NO 15 No A5C7 Pre-Adjuvant Therapy TUMOR FREE Not Available 93 NO Not Available 89 Complete Remission/Response YES WHITE NO NA R0 Not Applicable Not Applicable Not Applicable Not Applicable Not Applicable Not Applicable Not Applicable Not Applicable Not Applicable Stage IB Not Applicable Not Applicable Not Applicable 6th Not Applicable Not Applicable Not Applicable M0 N0 T2 1968 NO YES MP 3 Lung Alive 2013 2008 1953

2174 Dead T2 N1 M0 NA 57 R-Lower Not Applicable TCGA-50-5045 9ee20c0d-a1c3-47f3-abda-131b3f190f52 30 -20961 2174 0 Not Available Not Available Lung Adenocarcinoma Not Available NA Not Available Not Available Not Available Not Available Not Available Not Available Not Available Not Available NOT HISPANIC OR LATINO NA YES YES NO Not Available TCGA-50-5045-F32015 403e16e6-4db0-4a0c-866e-00b26e1fad62 15 Not Available Not Available 2174 Not Available 1433 Not Available Scheduled Follow-up Submission Progressive Disease Not Available NO 5 Locoregional Recurrence YES Not Available WITH TUMOR Not Available Progressive Disease Biopsy with Histologic Confirmation YES Dead 2012 FEMALE Lung Adenocarcinoma- Not Otherwise Specified (NOS) No C34.3 8140/3 C34.3 YES Not Available Not Available Not Available Not Available Not Available 6 Not Available Not Available Not Available Not Available Not Available Not Available Not Available Not Available Not Available Not Available Not Available No 5045 Not Available WITH TUMOR Not Available Not Available Not Available Not Available Not Available Not Available Not Available BLACK OR AFRICAN AMERICAN Not Available NA Primary Tumor Field TCGA-50-5045-R32016 86ec2fc8-2b21-48d4-9c2c-d70695750424 Not Available 15 Not Available 63 Not Available 5 Not Available Not Available Not Available OTHER Radioactive vicryl Mesh ADJUVANT Not Available Not Available 2012 Not Available Not Applicable Not Applicable Not Applicable Not Applicable Not Applicable Not Applicable Not Applicable Not Applicable Not Applicable Discrepancy Not Applicable Not Applicable Not Applicable 6th Not Applicable Not Applicable Not Applicable M0 N1 T2 Not Available NO YES 50 Not Available Lung Dead 2011 2004 Not Available

466 Alive T2a N0 MX Stage IB NA 78 R-Upper Not Applicable TCGA-44-A47A 7B9479FF-23D6-4689-91F0-02EFC94300F9 27 -28782 Not Applicable 0 202 Not Available Lung Adenocarcinoma Not Available NA 1 Not Available NO Not Available Not Available Not Available NO Not Available NOT HISPANIC OR LATINO NA NO;NO NO;YES NO;NO NO;NO TCGA-44-A47A-F46608;TCGA-44-A47A-F46609 F1D65C08-A2C8-432A-B4A0-AB16EC44BC2B;77A73903-5DE5-4FE9-96D7-AC3D0CCB31B6 12;12 Not Available;Not Available Not Available;Not Available Not Applicable;Not Applicable 466;466 400;397 Not Evaluated;Not Evaluated Additional New Tumor Event;Additional New Tumor Event Progressive Disease;Not Applicable Not Evaluated;Not Evaluated NO;NO 8;8 Locoregional Recurrence;Distant Metastasis YES;YES Not Available;Not Available WITH TUMOR;WITH TUMOR NO;NO Complete Remission/Response;Complete Remission/Response Biopsy with Histologic Confirmation;Convincing Imaging;Convincing Imaging NO;NO Alive;Alive 2013;2013 FEMALE Lung Adenocarcinoma- Not Otherwise Specified (NOS) No C34.1 8140/3 C34.1 YES Not Evaluated NO Not Available Not Available Not Available 11 Not Available Not Available Not Available Not Available Not Available Not Available Not Available Not Available Not Available NO 80 No A47A Preoperative TUMOR FREE Not Available Not Available NO Not Available Not Available Complete Remission/Response NO WHITE NO NA R0 Not Applicable Not Applicable Not Applicable Not Applicable Not Applicable Not Applicable Not Applicable Not Applicable Not Applicable Stage IB Not Applicable Not Applicable Not Applicable 7th Not Applicable Not Applicable Not Applicable MX N0 T2a 2010 YES NO 44 4 Lung Alive 2012 2012 1970

1157 Alive T2b N0 M0 Stage IIA NA 70 R-Middle Not Applicable TCGA-86-7713 cc68632c-b1e3-491b-b562-9468e2d1c101 2 -25791 Not Applicable 0 7 Not Available Lung Adenocarcinoma Not Available NA TCGA-86-7713-D35429;TCGA-86-7713-D35430 505DA070-940F-431E-B3D0-0DEC1E27D175;20185B1B-9AFC-48FB-A72A-4E830C7CB786 Not Available;Not Available 27;27 182;182 29;29 Etoposide;Cisplatin Complete Response;Complete Response 9;9 Not Available;Not Available Not Available;Not Available Not Available;Not Available Not Available;Not Available Not Applicable;Not Applicable Not Available;Not Available Not Available;Not Available NO;NO Chemotherapy;Chemotherapy Not Available;Not Available Not Available;Not Available Not Available;Not Available NO;NO 2012;2012 1 Not Available Not Available Not Available Not Available Not Available Not Available Not Available NOT HISPANIC OR LATINO NA Not Available;Not Available Not Available;Not Available Not Available;Not Available Not Available;Not Available TCGA-86-7713-F35420;TCGA-86-7713-F63513 B1E72965-1FBD-4BC1-8B24-56DDA7973B73;EB9FA841-4000-4DE3-B4DB-8714AB0669CD 27;12 Not Applicable;Not Applicable Not Applicable;Not Applicable Not Applicable;Not Applicable 422;1157 Not Applicable;Not Applicable 0;0 Scheduled Follow-up Submission;Scheduled Follow-up Submission Complete Remission/Response;Complete Remission/Response 100;100 NO;NO 9;8 Not Available;Not Available NO;NO Post-Adjuvant Therapy;Post-Adjuvant Therapy TUMOR FREE;TUMOR FREE YES;YES Complete Remission/Response;Complete Remission/Response Not Available;Not Available NO;NO Alive;Alive 2012;2014 MALE Lung Adenocarcinoma- Not Otherwise Specified (NOS) No C34.2 8140/3 C34.2 YES 80 NO Not Available Not Available Peripheral Lung 5 Not Available Not Available Not Available Not Available Not Available Not Available Not Available Not Available Not Available Not Available Not Available No 7713 Not Available TUMOR FREE Not Available Not Available Not Available Not Available Not Available Not Available NO WHITE Not Available NA R0 Not Applicable Not Applicable Not Applicable Not Applicable Not Applicable Not Applicable Not Applicable Not Applicable Not Applicable Stage IIA Not Applicable Not Applicable Not Applicable 7th Not Applicable Not Applicable Not Applicable M0 N0 T2b Not Available YES NO 86 1 Lung Alive 2011 2011 Not Available

540 Alive T1b N0 M0 Stage IA NA 69 R-Upper Not Applicable TCGA-97-A4M3 575752F8-2788-43CD-AFDE-F0F600F39D18 7 -25384 Not Applicable 0 237 Not Available Lung Adenocarcinoma 106 NA 1 Not Available NO Not Available Not Available Not Available NO Not Available NOT HISPANIC OR LATINO NA NO NO Not Available Not Available TCGA-97-A4M3-F57436 F59A23A0-77D8-4C10-B37D-67549803202E 11 Not Available Not Available Not Applicable 540 54 1 Scheduled Follow-up Submission Complete Remission/Response Unknown NO 3 New Primary Tumor YES Other TUMOR FREE NO Complete Remission/Response Biopsy with Histologic Confirmation NO Alive 2014 FEMALE Lung Adenocarcinoma Mixed Subtype No C34.1 8255/3 C34.1 YES Not Evaluated YES NO Not Available Unknown 3 NO NO 54 Not Available Not Available Not Available Not Available New Primary Tumor Biopsy with Histologic Confirmation YES 37.5 Yes, History of Prior Malignancy A4M3 Preoperative Unknown 69 73 NO 61 61 Complete Remission/Response YES WHITE NO NA Not Evaluated Not Applicable Not Applicable Not Applicable Not Applicable Not Applicable Not Applicable Not Applicable Not Applicable Not Applicable Stage IA Not Applicable Not Applicable Not Applicable 7th Not Applicable Not Applicable Not Applicable M0 N0 T1b 1984 YES NO 97 3 Lung Alive 2013 2012 1959

256 Alive T2 N0 M0 Stage IB NA 48 R-Upper Not Applicable TCGA-71-6725 4b7cd595-e7f9-45a8-b736-a8c7c42d9539 9 -17793 Not Applicable 0 61 Not Available Lung Adenocarcinoma Not Available NA TCGA-71-6725-D70932;TCGA-71-6725-D70933 C96AEDBF-65F7-4278-9841-02DE35F2FBD4;58FFFDD4-06DF-4156-903D-ECAA85C8D3B5 Not Available;Not Available 10;10 127;127 107;107 Cisplatin;Etoposide Clinical Progressive Disease;Clinical Progressive Disease 3;3 Not Available;Not Available Not Available;Not Available Not Available;Not Available Not Available;Not Available Not Applicable;Not Applicable Not Available;Not Available Not Available;Not Available NO;NO Chemotherapy;Chemotherapy Not Available;Not Available Not Available;Not Available Not Available;Not Available NO;NO 2015;2015 Not Available Not Available Not Available Not Available Not Available Not Available Not Available Not Available NOT HISPANIC OR LATINO NA NO YES NO Not Available TCGA-71-6725-F70928 91E9D55E-8826-486C-8511-B70EB38747D7 10 Not Available Not Available Not Applicable 256 164 2 Scheduled Follow-up Submission Progressive Disease Not Available NO 3 Locoregional Recurrence YES Post-Adjuvant Therapy WITH TUMOR YES Progressive Disease Not Available NO Alive 2015 FEMALE Lung Papillary Adenocarcinoma No C34.1 8260/3 C34.1 YES 60 Not Available Not Available Not Available Not Available 8 Not Available Not Available Not Available Not Available Not Available Not Available Not Available Not Available Not Available Not Available Not Available No 6725 Not Available TUMOR FREE Not Available Not Available Not Available Not Available Not Available Not Available Not Available ASIAN Not Available NA Primary Tumor Field TCGA-71-6725-R70931 F21BE49A-8969-4E71-BF16-E5FFB34417E3 Not Available 10 204 159 Radiographic Progressive Disease 3 35 70 NO External Not Applicable Not Available Not Available Gy 2015 RX Not Applicable Not Applicable Not Applicable Not Applicable Not Applicable Not Applicable Not Applicable Not Applicable Not Applicable Stage IB Not Applicable Not Applicable Not Applicable 6th Not Applicable Not Applicable Not Applicable M0 N0 T2 Not Available YES NO 71 2 Lung Alive 2011 2011 Not Available

414 Dead T2 N2 M0 Stage IIIA NA 70 R-Lower Not Applicable TCGA-62-A46Y 562A562B-CA03-44AC-B282-B51B457D8DAE 30 -25905 414 0 Not Available Not Available Lung Adenocarcinoma Not Available NA TCGA-62-A46Y-D36365;TCGA-62-A46Y-D40578 55FA6477-77DE-4CB4-9BDF-8AC7045569AA;06D3010F-39E9-4C9D-96D8-EAE50C4C705B Not Available;Not Available 30;25 256;256 74;74 Cisplatin;Navelbine Complete Response;Complete Response 10;2 Not Available;Not Available Not Available;Not Available Not Available;Not Available Not Available;Not Available Not Applicable;Not Applicable Not Available;Not Available Not Available;Not Available NO;NO Chemotherapy;Chemotherapy Not Available;Not Available Not Available;Not Available Not Available;Not Available NO;NO 2012;2013 Unknown Not Available NO Not Available Not Available Not Available NO Not Available NOT HISPANIC OR LATINO NA FEMALE Lung Adenocarcinoma Mixed Subtype No C34.3 8255/3 C34.3 YES 90 NO Not Available Not Available Peripheral Lung 10 YES NO 314 Not Available Not Available NO Not Available Distant Metastasis Biopsy with Histologic Confirmation;Convincing Imaging YES Not Available No A46Y Preoperative WITH TUMOR Not Available Not Available YES 84 88 Complete Remission/Response YES WHITE YES NA Regional site TCGA-62-A46Y-R36366 7364E4A0-C21C-485D-B4FF-5335F2218905 Not Available 31 256 227 Complete Response 10 Not Available 50 NO External Not Applicable Not Available Not Available Gy 2012 R0 Not Applicable Not Applicable Not Applicable Not Applicable Not Applicable Not Applicable Not Applicable Not Applicable Not Applicable Stage IIIA Not Applicable Not Applicable Not Applicable 6th Not Applicable Not Applicable Not Applicable M0 N2 T2 Not Available NO YES 62 1 Lung Dead 2012 2008 Not Available

534 Alive T2a N0 MX Stage IB NA 58 R-Lower Not Applicable TCGA-55-8301 b36b7567-30d3-4d0c-84c4-b203986e0fdb 3 -21535 Not Applicable 0 44 Not Available Lung Adenocarcinoma Not Available NA TCGA-55-8301-D56263;TCGA-55-8301-D56264 81EAC4EA-9876-4E67-B2D2-230762F91928;D6B06699-89FF-4995-95D5-D7B9B1095651 Not Available;Not Available 30;30 Not Available;Not Available 261;261 Carboplatin;Taxol Partial Response;Partial Response 1;1 Not Available;Not Available Not Available;Not Available Not Available;Not Available Not Available;Not Available Not Applicable;Not Applicable Not Available;Not Available Not Available;Not Available NO;NO Chemotherapy;Chemotherapy Not Available;Not Available Not Available;Not Available Not Available;Not Available NO;NO 2014;2014 Unknown Not Available Not Available Not Available Not Available Not Available Not Available Not Available NOT HISPANIC OR LATINO NA YES YES NO NO TCGA-55-8301-F56261 F961BE96-B9C1-48C0-9CC2-B368B6495583 30 Not Available Not Available Not Applicable 534 238 Unknown Scheduled Follow-up Submission Partial Remission/Response Unknown NO 1 Locoregional Recurrence YES Not Available WITH TUMOR NO Complete Remission/Response Convincing Imaging NO Alive 2014 MALE Lung Adenocarcinoma- Not Otherwise Specified (NOS) No C34.3 8140/3 C34.3 YES Unknown Not Available Not Available Not Available Unknown 10 Not Available Not Available Not Available Not Available Not Available Not Available Not Available Not Available Not Available NO 100 No 8301 Unknown TUMOR FREE Not Available Not Available NO Not Available Not Available Complete Remission/Response Not Available WHITE NO NA Regional site TCGA-55-8301-R56262 F51AFBFF-1D27-42D6-A936-C1FFA21A4A43 Not Available 30 309 257 Partial Response 1 30 6660 NO External Not Applicable Not Available Not Available cGy 2014 R0 Not Applicable Not Applicable Not Applicable Not Applicable Not Applicable Not Applicable Not Applicable Not Applicable Not Applicable Stage IB Not Applicable Not Applicable Not Applicable 7th Not Applicable Not Applicable Not Applicable MX N0 T2a Not Available YES NO 55 2 Lung Alive 2012 2012 1962

2199 Alive T2 N0 M0 Stage IB NA 78 L-Lower Not Applicable TCGA-62-A46V F809E21D-5F2E-4F3B-A1AA-D6B8D463552A 30 -28495 Not Applicable 0 1631 Not Available Lung Adenocarcinoma Not Available NA Unknown Not Available NO Not Available Not Available Not Available NO Not Available NOT HISPANIC OR LATINO NA Not Available Not Available Not Available Not Available TCGA-62-A46V-F51440 58E3C427-7F74-4CC2-9847-DF0700AF43D5 19 Not Applicable Not Applicable Not Applicable 2199 Not Applicable Unknown Scheduled Follow-up Submission Complete Remission/Response 90 NO 11 Not Available NO Preoperative TUMOR FREE NO Complete Remission/Response Not Available NO Alive 2013 FEMALE Lung Adenocarcinoma Mixed Subtype No C34.3 8255/3 C34.3 YES 90 NO Not Available Not Available Peripheral Lung 10 Not Available Not Available Not Available Not Available Not Available Not Available Not Available Not Available Not Available NO Not Available No A46V Preoperative TUMOR FREE Not Available Not Available NO 51 100 Complete Remission/Response YES WHITE NO NA R0 Not Applicable Not Applicable Not Applicable Not Applicable Not Applicable Not Applicable Not Applicable Not Applicable Not Applicable Stage IB Not Applicable Not Applicable Not Applicable 6th Not Applicable Not Applicable Not Applicable M0 N0 T2 Not Available NO YES 62 3 Lung Alive 2012 2007 Not Available

268 Dead T3 N1 M0 Stage IIIA NA 73 Discrepancy Not Available TCGA-49-4507 50b2c647-aecd-4c81-af22-0d8116593552 2 -26851 268 0 Not Available Not Available Lung Adenocarcinoma Not Available NA TCGA-49-4507-D7010;TCGA-49-4507-D13868;TCGA-49-4507-D17259 5d1cdc10-8244-4d21-b7fd-b69dfe9548dc;96cdd9a1-d9ad-4861-88e3-d65b2d13f956;7c8c9fcb-3b5f-42fe-9f31-c8319cde0fc7 Not Available;Not Available;Not Available 2;18;6 110;110;Not Available 47;47;174 Taxol;Carboplatin;Gemcitabine Not Available;Not Available;Not Available 2;7;10 4;4;Not Available Not Available;Not Available;Not Available Not Available;Not Available;Not Available ADJUVANT;ADJUVANT;RECURRENCE Not Applicable;Not Applicable;Not Applicable 1;1;2 IV;IV;IV NO;NO;NO Chemotherapy;Chemotherapy;Chemotherapy Not Available;Not Available;Not Available Not Available;4 cycles;Not Available g/m2;g/m2;Not Available Not Available;Not Available;Not Available 2011;2011;2011 1 Not Available Not Available Not Available Not Available Not Available Not Available Not Available NOT HISPANIC OR LATINO NA YES NO NO NO TCGA-49-4507-F7007 33c2605a-97c1-495f-ac44-17fff4c8ebd6 2 Not Available Not Available 268 Not Available 158 1 Not Available Progressive Disease Not Available Not Available 2 Not Available YES Preoperative WITH TUMOR YES Progressive Disease Not Available YES Dead 2011 FEMALE Lung Adenocarcinoma- Not Otherwise Specified (NOS) No C34.0 8140/3 C34.0 YES Not Available Not Available Not Available Not Available Not Available 2 Not Available Not Available Not Available Not Available Not Available Not Available Not Available Not Available Not Available Not Available 78 No 4507 Preoperative WITH TUMOR Not Available Not Available Not Available Not Available Not Available Not Available Not Available WHITE Not Available NA Primary Tumor Field TCGA-49-4507-R7009 becb6cbe-b49e-49ae-ae68-ac7e19de8c3b 1 2 95 46 Not Available 2 34 1620 NO EXTERNAL BEAM Not Applicable ADJUVANT Not Available cGy 2011 R0 Not Applicable Not Applicable Not Applicable Not Applicable Not Applicable Not Applicable Not Applicable Not Applicable Not Applicable Stage IIIA Not Applicable Not Applicable Not Applicable Not Available Not Applicable Not Applicable Not Applicable M0 N1 T3 1991 NO YES 49 4 Lung Dead 2011 1997 Not Available

2161 Alive T1 N0 M0 Stage IA NA 50 R-Upper Not Applicable TCGA-NJ-A4YF 595FC3AD-F603-421B-B130-52F1F617050B 25 -18584 Not Applicable 0 1720 Not Available Lung Adenocarcinoma 77.5 NA Not Evaluated Not Available NO Not Available Not Available Not Available NO Not Available NOT HISPANIC OR LATINO NA Not Available Not Available Not Available Not Available TCGA-NJ-A4YF-F50858 635C2793-F06E-4540-80A4-D9F2C1D72DFB 1 Not Applicable Not Applicable Not Applicable 2161 Not Applicable Not Available Scheduled Follow-up Submission Stable Disease Not Available NO 11 Not Available NO Not Available TUMOR FREE NO Stable Disease Not Available NO Alive 2013 FEMALE Lung Adenocarcinoma Mixed Subtype No C34.1 8255/3 C34.1 YES Not Evaluated NO Not Available Not Available Unknown 4 Not Available Not Available Not Available Not Available Not Available Not Available Not Available Not Available Not Available NO 30 No A4YF Not Evaluated Unknown 95.1 83.9 Unknown 96 80.7 Not Applicable YES BLACK OR AFRICAN AMERICAN Unknown NA Not Evaluated Not Applicable Not Applicable Not Applicable Not Applicable Not Applicable Not Applicable Not Applicable Not Applicable Not Applicable Stage IA Not Applicable Not Applicable Not Applicable 6th Not Applicable Not Applicable Not Applicable M0 N0 T1 Not Available NO YES NJ 3 Lung Alive 2013 2007 Not Available

775 Alive T2a N2 M0 Stage IIIA NA 83 L-Lower Not Applicable TCGA-97-7554 a9644274-13bb-4228-9b4f-14260ccc26eb 17 -30327 Not Applicable 0 264 Not Available Lung Adenocarcinoma 53 NA TCGA-97-7554-D18639;TCGA-97-7554-D18627 779af18b-5c23-4e62-ae9c-b6288f15b3da;c7851da9-c4f2-4a19-b48c-a90e5b4d9cba Not Available;Not Available 21;21 111;111 49;49 Alimta;Carboplatin Not Available;Not Available 11;11 4;4 817;350 mg;mg ADJUVANT;ADJUVANT Not Applicable;Not Applicable 1;1 IV;IV NO;NO Chemotherapy;Chemotherapy Not Available;Not Available 3268;1400 mg;mg Not Available;Not Available 2011;2011 0 Not Available Not Available Not Available Not Available Not Available Not Available Not Available NOT HISPANIC OR LATINO NA Not Available Not Available Not Available Not Available TCGA-97-7554-F20552 C53D0705-AD5B-47B9-8D49-C786D4466CC7 17 Not Applicable Not Applicable Not Applicable 775 Not Applicable 1 Scheduled Follow-up Submission Not Available Not Available Not Available 1 Not Available NO Post-Adjuvant Therapy TUMOR FREE YES Complete Remission/Response Not Available NO Alive 2012 FEMALE Lung Papillary Adenocarcinoma No C34.3 8260/3 C34.3 YES Not Available YES YES G12V Not Available 11 Not Available Not Available Not Available Not Available Not Available Not Available Not Available Not Available Not Available Not Available 30 No 7554 Preoperative TUMOR FREE 75 75 Not Available 76 68 Not Available YES WHITE Not Available NA R0 Not Applicable Not Applicable Not Applicable Not Applicable Not Applicable Not Applicable Not Applicable Not Applicable Not Applicable Stage IIIA Not Applicable Not Applicable Not Applicable 7th Not Applicable Not Applicable Not Applicable M0 N2 T2a 1983 NO YES 97 3 Lung Alive 2011 2010 1945

2620 Dead T2 N0 M0 Stage IB NA 67 L-Lower Not Applicable TCGA-MP-A4SV 706420C4-8820-4B41-80D9-F3EFD1D2A4F0 2 -24516 2620 0 Not Available Not Available Lung Adenocarcinoma 48 NA Not Evaluated Not Available NO Not Available Not Available Not Available NO Not Available Unknown NA MALE Lung Adenocarcinoma- Not Otherwise Specified (NOS) No C34.3 8140/3 C34.3 YES Not Evaluated NO Not Available Not Available Unknown 4 Not Available Not Available Not Available Not Available Not Available Not Available Not Available Not Available Not Available Unknown 110 No A4SV Not Evaluated Unknown Not Available 78 NO Not Available 62 Complete Remission/Response YES Unknown NO NA R0 Not Applicable Not Applicable Not Applicable Not Applicable Not Applicable Not Applicable Not Applicable Not Applicable Not Applicable Stage IB Not Applicable Not Applicable Not Applicable 5th Not Applicable Not Applicable Not Applicable M0 N0 T2 Not Available NO YES MP 2 Lung Dead 2013 2001 1946

999 Dead T2 N1 M0 Stage IIB NA 68 L-Upper Not Applicable TCGA-49-4506 b61abdfd-b7a0-4e1a-b570-d1eaecad3a9e 3 -25132 999 0 Not Available Not Available Lung Adenocarcinoma Not Available NA 1 Not Available Not Available Not Available Not Available Not Available Not Available Not Available NOT HISPANIC OR LATINO NA NO NO NO Not Available TCGA-49-4506-F58841 8288A530-E15A-453B-A072-A3824044F22E 25 Not Available Not Available 999 Not Available 692 2 Scheduled Follow-up Submission Progressive Disease 70 NO 4 Locoregional Recurrence YES Post-Adjuvant Therapy Discrepancy YES Complete Remission/Response Convincing Imaging YES Dead 2014 FEMALE Lung Adenocarcinoma- Not Otherwise Specified (NOS) No C34.1 8140/3 C34.1 YES Not Available Not Available Not Available Not Available Peripheral Lung 2 Not Available Not Available Not Available Not Available Not Available Not Available Not Available Not Available Not Available Not Available 100 No 4506 Pre-Adjuvant Therapy Discrepancy Not Available Not Available Not Available Not Available Not Available Not Available NO WHITE Not Available NA R1 Not Applicable Not Applicable Not Applicable Not Applicable Not Applicable Not Applicable Not Applicable Not Applicable Not Applicable Stage IIB Not Applicable Not Applicable Not Applicable 4th Not Applicable Not Applicable Not Applicable M0 N1 T2 Not Available NO YES 49 2 Lung Dead 2011 1997 Not Available

244 Dead T2 NX M1 Stage IV NA 57 R-Lower Not Applicable TCGA-05-4402 722d6818-f1cb-49f4-b68a-fd0a31304681 22 -20819 244 0 Not Available Not Available Lung Adenocarcinoma Not Available NA TCGA-05-4402-D36467;TCGA-05-4402-D36468;TCGA-05-4402-D36469 C22C6B31-7A7B-4961-B95D-137666A2C3FF;BA9F9930-88F2-4DA0-982D-BEDBEA793B76;8CEA61DA-614E-4CE9-8202-877E85ECC83F Not Available;Not Available;Not Available 5;5;5 91;91;122 30;30;122 Carboplatin;Vinorelbine;Erlotinib Complete Response;Complete Response;Complete Response 12;12;12 Not Available;Not Available;Not Available Not Available;Not Available;Not Available Not Available;Not Available;Not Available Not Available;Not Available;Not Available Not Applicable;Not Applicable;Not Applicable Not Available;Not Available;Not Available Not Available;Not Available;Not Available NO;NO;NO Chemotherapy;Chemotherapy;Immunotherapy Not Available;Not Available;Not Available Not Available;Not Available;Not Available Not Available;Not Available;Not Available NO;NO;NO 2012;2012;2012 Not Available Not Available Not Available Not Available Not Available Not Available Not Available Not Available Not Available NA Not Available Not Available Not Available Not Available TCGA-05-4402-F36466 DFAF4F5D-D945-4D17-8F16-6BFB6E538D7A 31 Not Applicable Not Applicable 244 Not Available Not Applicable Not Available Scheduled Follow-up Submission Complete Remission/Response Not Available NO 10 Not Available NO Not Available TUMOR FREE YES Complete Remission/Response Not Available NO Dead 2012 FEMALE Lung Adenocarcinoma Mixed Subtype No C34.3 8255/3 C34.3 YES Not Available Not Available Not Available Not Available Not Available 7 Not Available Not Available Not Available Not Available Not Available Not Available Not Available Not Available Not Available Not Available Not Available Yes 4402 Not Available TUMOR FREE Not Available Not Available Not Available Not Available Not Available Not Available Not Available Not Available Not Available NA R2 Not Applicable Not Applicable Not Applicable Not Applicable Not Applicable Not Applicable Not Applicable Not Applicable Not Applicable Stage IV Not Applicable Not Applicable Not Applicable 6th Not Applicable Not Applicable Not Applicable M1 NX T2 Not Available NO YES 05 1 Lung Dead 2010 2007 Not Available

481 Alive T2a N0 MX Stage IB NA 61 R-Lower Not Applicable TCGA-55-A494 ADBABA9C-5EFC-4130-82F6-8055EAB13795 21 -22540 Not Applicable 0 5 Not Available Lung Adenocarcinoma 76 NA 1 Not Available YES Not Available Not Available FISH YES Not Available NOT HISPANIC OR LATINO NA Not Available Not Available Not Available Not Available TCGA-55-A494-F59038 C756AD9D-B853-4622-A840-F6365F418A50 30 Not Applicable Not Applicable Not Applicable 481 Not Applicable Unknown Scheduled Follow-up Submission Complete Remission/Response Unknown NO 4 Not Available NO Not Available TUMOR FREE NO Complete Remission/Response Not Available NO Alive 2014 FEMALE Lung Adenocarcinoma- Not Otherwise Specified (NOS) No C34.3 8140/3 C34.3 YES 80 YES YES G12D Peripheral Lung 2 Not Available Not Available Not Available Not Available Not Available Not Available Not Available Not Available Not Available NO 7 No A494 Preoperative TUMOR FREE Not Available Not Available Unknown 95 77 Unknown YES WHITE Unknown NA R0 Not Applicable Not Applicable Not Applicable Not Applicable Not Applicable Not Applicable Not Applicable Not Applicable Not Applicable Stage IB Not Applicable Not Applicable Not Applicable 7th Not Applicable Not Applicable Not Applicable MX N0 T2a 1980 YES NO 55 3 Lung Alive 2013 2012 1967

905 Dead T2 N2 MX Stage IIIA NA 69 R-Middle Not Applicable TCGA-49-4512 a1e65587-24c1-4b41-92a7-4e1f15fffd78 9 -25502 905 0 157 Not Available Lung Adenocarcinoma Not Available NA TCGA-49-4512-D14718 11659d14-75e7-4990-bcf1-fd6d91969685 Not Available 9 Not Available Not Available Not Available Not Available 8 Not Available Not Available Not Available ADJUVANT Not Applicable 1 IV NO Chemotherapy Not Available Not Available Not Available Not Available 2011 Not Available Not Available Not Available Not Available Not Available Not Available Not Available Not Available NOT HISPANIC OR LATINO NA Not Available Not Available Not Available Not Available TCGA-49-4512-F14815 5d052835-b26a-42e0-aa2d-fb53c06891f4 10 Not Applicable Not Applicable 905 Not Available Not Applicable Not Available Scheduled Follow-up Submission Not Available Not Available Not Available 8 Not Available NO Not Available WITH TUMOR YES Progressive Disease Not Available NO Dead 2011 FEMALE Lung Adenocarcinoma- Not Otherwise Specified (NOS) No C34.2 8140/3 C34.2 YES Not Available Not Available Not Available Not Available Not Available 8 Not Available Not Available Not Available Not Available Not Available Not Available Not Available Not Available Not Available Not Available Not Available No 4512 Not Available WITH TUMOR Not Available Not Available Not Available Not Available Not Available Not Available Not Available WHITE Not Available NA R1 Not Applicable Not Applicable Not Applicable Not Applicable Not Applicable Not Applicable Not Applicable Not Applicable Not Applicable Stage IIIA Not Applicable Not Applicable Not Applicable 6th Not Applicable Not Applicable Not Applicable MX N2 T2 Not Available NO YES 49 1 Lung Dead 2011 2007 Not Available

287 Alive T2a N0 M0 Stage IB NA 79 R-Upper Not Applicable TCGA-44-A47B 967D6548-5A84-4B7E-BC3F-2E522859FCE6 20 -28869 Not Applicable 0 105 Not Available Lung Adenocarcinoma 73 NA 0 Not Available NO Not Available Not Available Not Available NO Not Available NOT HISPANIC OR LATINO NA Not Available Not Available Not Available Not Available TCGA-44-A47B-F41746 F1FE3CC6-00FC-40B7-AD09-0F7D71224725 27 Not Applicable Not Applicable Not Applicable 287 Not Applicable Not Available Scheduled Follow-up Submission Complete Remission/Response Not Available NO 3 Not Available NO Not Available TUMOR FREE NO Complete Remission/Response Not Available NO Alive 2013 MALE Lung Adenocarcinoma- Not Otherwise Specified (NOS) No C34.1 8140/3 C34.1 YES Not Evaluated NO Not Available Not Available Not Available 11 Not Available Not Available Not Available Not Available Not Available Not Available Not Available Not Available Not Available NO 4.5 No A47B Preoperative TUMOR FREE 102 96 NO 98 96 Complete Remission/Response YES WHITE NO NA R0 Not Applicable Not Applicable Not Applicable Not Applicable Not Applicable Not Applicable Not Applicable Not Applicable Not Applicable Stage IB Not Applicable Not Applicable Not Applicable 7th Not Applicable Not Applicable Not Applicable M0 N0 T2a 1965 YES NO 44 3 Lung Alive 2012 2012 1956

1194 Dead T2 N0 M0 Stage IB NA 84 R-Upper Not Applicable TCGA-62-A470 E60A8F8A-0FE6-40E5-9F30-BCFF48AC73F4 30 -30689 1194 0 Not Available Not Available Lung Adenocarcinoma Not Available NA 0 Not Available NO Not Available Not Available Not Available NO Not Available NOT HISPANIC OR LATINO NA MALE Lung Acinar Adenocarcinoma No C34.1 8550/3 C34.1 YES 100 NO Not Available Not Available Central Lung 10 NO YES 539 NO Not Available Not Available Not Available Locoregional Recurrence Convincing Imaging YES 30 No A470 Preoperative WITH TUMOR Not Available Not Available NO 74 111 Complete Remission/Response YES WHITE NO NA R0 Not Applicable Not Applicable Not Applicable Not Applicable Not Applicable Not Applicable Not Applicable Not Applicable Not Applicable Stage IB Not Applicable Not Applicable Not Applicable 6th Not Applicable Not Applicable Not Applicable M0 N0 T2 Not Available NO YES 62 3 Lung Dead 2012 2009 Not Available

129 Alive T1a N0 M0 Stage IA NA 71 R-Upper Not Applicable TCGA-69-8255 552712d3-da16-441e-ba93-11810793d598 4 -26289 Not Applicable 0 129 Not Available Lung Adenocarcinoma 60 NA Not Available Not Available YES Other Not Available Not Available NO Not Available HISPANIC OR LATINO NA Not Available Not Available Not Available Not Available TCGA-69-8255-F32611 C4D99C65-5914-401C-9071-C2ED9877F39C 4 Not Applicable Not Applicable Not Applicable 129 Not Applicable Not Available Scheduled Follow-up Submission Unknown Not Available NO 6 Not Available NO Not Available Not Available NO Unknown Not Available NO Alive 2012 MALE Lung Solid Pattern Predominant Adenocarcinoma No C34.1 8230/3 C34.1 YES Not Available NO Not Available Not Available Not Available 6 Not Available Not Available Not Available Not Available Not Available Not Available Not Available Not Available Not Available NO 40 No 8255 Not Available Unknown Not Available 60 NO Not Available 57 Unknown YES WHITE NO NA Not Available Not Applicable Not Applicable Not Applicable Not Applicable Not Applicable Not Applicable Not Applicable Not Applicable Not Applicable Stage IA Not Applicable Not Applicable Not Applicable 7th Not Applicable Not Applicable Not Applicable M0 N0 T1a Not Available YES NO 69 2 Lung Alive 2012 2011 1971

1115 Dead T2 N2 M0 Stage IIIA NA Not Available R-Upper Not Applicable TCGA-75-6214 32592562-11fa-435a-b40b-2b242bae53aa 12 Not Available Not Applicable Not Available Not Available Not Available Lung Adenocarcinoma Not Available NA 1 Not Available Not Available Not Available Not Available Not Available Not Available Not Available Not Available NA Not Available;NO Not Available;YES Not Available;Not Available Not Available;YES TCGA-75-6214-F15860;TCGA-75-6214-F72038 17e757bc-f78c-4be4-8ece-1109e163916d;8103EC55-5AAD-42CC-988D-A617888D510B 12;2 Not Available;Not Available Not Available;524 Not Applicable;1115 Not Available;Not Available Not Available;419 0;0 Not Available;Scheduled Follow-up Submission Progressive Disease;Progressive Disease Not Available;Not Evaluated Not Available;NO 8;4 Not Available;Distant Metastasis YES;YES Adjuvant therapy;Post-Adjuvant Therapy Not Available;WITH TUMOR NO;NO Complete Remission/Response;Complete Remission/Response Not Available;Not Available NO;NO Alive;Dead 2011;2015 FEMALE Lung Adenocarcinoma- Not Otherwise Specified (NOS) No C34.1 8140/3 C34.1 YES Not Available Not Available Not Available Not Available Peripheral Lung 8 Not Available Not Available Not Available Not Available Not Available Not Available Not Available Not Available Not Available Not Available 40 No 6214 Preoperative WITH TUMOR Not Available Not Available Not Available Not Available Not Available Not Available Not Available Not Available Not Available NA R0 Not Applicable Not Applicable Not Applicable Not Applicable Not Applicable Not Applicable Not Applicable Not Applicable Not Applicable Stage IIIA Not Applicable Not Applicable Not Applicable 6th Not Applicable Not Applicable Not Applicable M0 N2 T2 Not Available NO YES 75 2 Lung Alive 2011 Not Available 1970

1528 Dead T2 N0 M0 Stage IB NA 67 L-Upper Not Applicable TCGA-78-7633 44218b35-219c-4ad9-a01e-fde14067c4c0 31 -24713 Not Applicable 0 994 Not Available Lung Adenocarcinoma 57 NA 0 Not Available NO Not Available Not Available Not Available NO Not Available Not Available NA Not Available;Unknown Not Available;Unknown Not Available;Not Available Not Available;Not Available TCGA-78-7633-F21095;TCGA-78-7633-F46157 f9269731-0d0b-4785-9c9b-6b9766317d29;8A95CDC5-FB75-4B7D-AE3F-923D2FF527B2 31;29 Not Available;Not Available Not Available;Not Available Not Applicable;1528 994;Not Available Not Available;1450 Not Available;Unknown Scheduled Follow-up Submission;Scheduled Follow-up Submission Not Available;Progressive Disease Not Available;Not Available Not Available;NO 1;8 Not Available;Locoregional Recurrence Not Available;YES Not Available;Not Available Not Available;WITH TUMOR Not Available;Unknown Not Available;Complete Remission/Response Not Available;Biopsy with Histologic Confirmation Not Available;Unknown Alive;Dead 2012;2013 MALE Lung Adenocarcinoma Mixed Subtype No C34.1 8255/3 C34.1 YES Not Available NO Not Available Not Available Not Available 1 Not Available Not Available Not Available Not Available Not Available Not Available Not Available Not Available Not Available Not Available 94 Yes 7633 Preoperative Not Available Not Available Not Available Not Available Not Available 100 Not Available YES WHITE Not Available NA R0 Not Applicable Not Applicable Not Applicable Not Applicable Not Applicable Not Applicable Not Applicable Not Applicable Not Applicable Stage IB Not Applicable Not Applicable Not Applicable 6th Not Applicable Not Applicable Not Applicable M0 N0 T2 2002 NO YES 78 4 Lung Alive 2012 2009 1955

307 Alive T1a N0 MX Stage IA NA 60 R-Upper Not Applicable TCGA-L9-A444 4A1D30E8-E93A-41ED-8E54-08EAFF6556A7 26 -22202 Not Applicable 0 6 Not Available Lung Adenocarcinoma 57 NA 0 Not Available NO Not Available Not Available Not Available NO Not Available NOT HISPANIC OR LATINO NA Not Available Not Available Not Available Not Available TCGA-L9-A444-F46270 B1C53C2B-9FB6-4024-888D-0192BE820066 5 Not Applicable Not Applicable Not Applicable 307 Not Applicable Not Evaluated Scheduled Follow-up Submission Complete Remission/Response Not Evaluated NO 8 Not Available NO Not Evaluated TUMOR FREE NO Complete Remission/Response Not Available NO Alive 2013 FEMALE Lung Adenocarcinoma- Not Otherwise Specified (NOS) No C34.1 8140/3 C34.1 YES Not Available NO Not Available Not Available Central Lung 10 Not Available Not Available Not Available Not Available Not Available Not Available Not Available Not Available Not Available NO 10 Yes, History of Prior Malignancy A444 Preoperative Not Available 62 54 NO 58 49 Complete Remission/Response YES WHITE NO NA R0 Not Applicable Not Applicable Not Applicable Not Applicable Not Applicable Not Applicable Not Applicable Not Applicable Not Applicable Stage IA Not Applicable Not Applicable Not Applicable 7th Not Applicable Not Applicable Not Applicable MX N0 T1a Not Available YES NO L9 2 Lung Alive 2012 2012 1972

321 Dead T2 N0 M0 Stage IB NA 56 R-Upper Not Applicable TCGA-78-7542 e64f76e4-f748-4d9e-ba71-53e2818a7920 13 -20763 321 0 Not Available Not Available Lung Adenocarcinoma Not Available NA 0 Not Available NO Not Available Not Available Not Available NO Not Available Not Available NA Not Available Not Available Not Available Not Available TCGA-78-7542-F20456 d15aada7-429d-413c-855e-e49c5ab30779 13 Not Available Not Available 321 Not Available Not Available Not Available Scheduled Follow-up Submission Not Available Not Available Not Available 1 Not Available Not Available Not Available Not Available Not Available Not Available Not Available Not Available Dead 2012 MALE Lung Adenocarcinoma- Not Otherwise Specified (NOS) No C34.1 8140/3 C34.1 YES Not Available NO Not Available Not Available Not Available 1 Not Available Not Available Not Available Not Available Not Available Not Available Not Available Not Available Not Available Not Available 60 No 7542 Preoperative Not Available Not Available Not Available Not Available Not Available Not Available Not Available Not Available WHITE Not Available NA R0 Not Applicable Not Applicable Not Applicable Not Applicable Not Applicable Not Applicable Not Applicable Not Applicable Not Applicable Stage IB Not Applicable Not Applicable Not Applicable 6th Not Applicable Not Applicable Not Applicable M0 N0 T2 Not Available NO YES 78 2 Lung Dead 2012 1993 1952

1600 Dead T2 N0 M0 Stage IB NA 80 L-Lower Not Applicable TCGA-73-4658 afb91740-2668-46d7-ba01-bb26153ad1c7 15 -29508 1600 0 1600 Not Available Lung Adenocarcinoma Not Available NA Not Available Not Available NO Not Available Not Available Not Available Not Available Not Available NOT HISPANIC OR LATINO NA Not Available Not Available Not Available Not Available TCGA-73-4658-F12975 54126ff4-4956-4563-97f0-8103fd637634 15 Not Applicable Not Applicable 1600 1600 Not Applicable Not Available Not Available Complete Remission/Response Not Available Not Available 6 Not Available NO Not Available TUMOR FREE NO Complete Remission/Response Not Available NO Dead 2011 FEMALE Lung Adenocarcinoma- Not Otherwise Specified (NOS) No C34.3 8140/3 C34.3 YES Not Available NO Not Available Not Available Central Lung 6 Not Available Not Available Not Available Not Available Not Available Not Available Not Available Not Available Not Available Not Available 25 Yes 4658 Not Available TUMOR FREE Not Available Not Available Not Available Not Available Not Available Not Available Not Available WHITE Not Available NA R0 Not Applicable Not Applicable Not Applicable Not Applicable Not Applicable Not Applicable Not Applicable Not Applicable Not Applicable Stage IB Not Applicable Not Applicable Not Applicable 6th Not Applicable Not Applicable Not Applicable M0 N0 T2 Not Available NO YES 73 3 Lung Dead 2011 2004 Not Available

79 Alive T1 N0 M0 Stage IA NA 81 R-Lower Not Applicable TCGA-91-6835 2f09479f-87fc-4c34-8e2c-333e970a3681 20 -29887 Not Applicable 0 35 Not Available Lung Adenocarcinoma 78 NA Not Available Not Available NO Not Available Not Available Not Available Not Available Not Available NOT HISPANIC OR LATINO NA Not Available Not Available Not Available Not Available TCGA-91-6835-F32643 169e8cda-2406-4085-97e2-7287e1bd2e41 5 Not Applicable Not Applicable Not Applicable 79 Not Applicable Unknown Scheduled Follow-up Submission Complete Remission/Response Unknown YES 6 Not Available NO Not Available TUMOR FREE NO Complete Remission/Response Not Available NO Alive 2012 FEMALE Lung Adenocarcinoma- Not Otherwise Specified (NOS) No C34.3 8140/3 C34.3 YES Not Available NO Not Available Not Available Not Available 7 Not Available Not Available Not Available Not Available Not Available Not Available Not Available Not Available Not Available Not Available 25 No 6835 Not Available Not Available Not Available 91 Not Available Not Available 75 Not Available YES WHITE Not Available NA R0 Not Applicable Not Applicable Not Applicable Not Applicable Not Applicable Not Applicable Not Applicable Not Applicable Not Applicable Stage IA Not Applicable Not Applicable Not Applicable 7th Not Applicable Not Applicable Not Applicable M0 N0 T1 1976 NO YES 91 3 Lung Alive 2011 2010 Not Available

737 Dead T3 N0 M0 Stage IIB NA 56 R-Upper Not Applicable TCGA-86-A4JF E01E4FB3-236A-4D16-9073-555876B688E9 14 -20491 Not Applicable 0 0 Not Available Lung Adenocarcinoma Not Available NA TCGA-86-A4JF-D52793;TCGA-86-A4JF-D52796 85E463A3-A1DE-4C8E-8C0F-9BD9AB0377D8;F06AAA11-F3BF-4A51-BA60-963FDE4D8CB8 Not Available;Not Available 6;6 171;536 31;444 Not Available;Not Available Complete Response;Complete Response 12;12 Not Available;Not Available Not Available;Not Available Not Available;Not Available Not Available;Not Available Not Applicable;Not Applicable Not Available;Not Available Not Available;Not Available NO;NO Chemotherapy;Chemotherapy Not Available;Not Available Not Available;Not Available Not Available;Not Available NO;NO 2013;2013 1 Not Available NO Not Available Not Available Not Available NO Not Available NOT HISPANIC OR LATINO NA YES;Not Available Unknown;Not Available Not Available;Not Available NO;Not Available TCGA-86-A4JF-F51505;TCGA-86-A4JF-F70857 D96618EB-06F6-47F9-8C4C-D1E3264691D1;C9326E9D-3C7D-45BF-A0F0-3FD9F0CF2214 6;10 Not Available;Not Applicable Not Available;Not Applicable Not Applicable;737 536;Not Available 436;Not Applicable 2;4 Scheduled Follow-up Submission;Scheduled Follow-up Submission Progressive Disease;Progressive Disease 70;20 NO;NO 12;3 Distant Metastasis;Not Available YES;NO Post-Adjuvant Therapy;Post-Adjuvant Therapy WITH TUMOR;WITH TUMOR YES;YES Complete Remission/Response;Complete Remission/Response Not Available;Not Available NO;NO Alive;Dead 2013;2015 MALE Lung Adenocarcinoma- Not Otherwise Specified (NOS) No C34.1 8140/3 C34.1 YES 90 NO Not Available Not Available Central Lung 2 Not Available Not Available Not Available Not Available Not Available Not Available Not Available Not Available Not Available NO Not Available No A4JF Preoperative TUMOR FREE Not Available Not Available Unknown Not Available Not Available Complete Remission/Response NO WHITE Unknown NA R0 Not Applicable Not Applicable Not Applicable Not Applicable Not Applicable Not Applicable Not Applicable Not Applicable Not Applicable Stage IIB Not Applicable Not Applicable Not Applicable 7th Not Applicable Not Applicable Not Applicable M0 N0 T3 2012 YES NO 86 4 Lung Alive 2013 2012 Not Available

376 Dead T2a N1 M0 Stage IIA NA 52 R-Upper Not Applicable TCGA-86-6562 dcd066ba-5894-453e-b1da-82092858c3db 21 -19301 Not Applicable 0 0 Not Available Lung Adenocarcinoma Not Available NA TCGA-86-6562-D29769;TCGA-86-6562-D29767 f359ca59-3a44-488c-bfc2-7aea795f3d57;02372dd0-f7cc-47c8-a3d0-0c6031d75cda Not Available;Not Available 28;28 253;253 27;27 Vinblastine;Cisplatin Not Available;Not Available 3;3 4;4 6;150 mg/day;mg/day ADJUVANT;ADJUVANT Not Applicable;Not Applicable Not Available;Not Available IV;IV NO;NO Chemotherapy;Chemotherapy Not Available;Not Available 120;600 mg;mg Not Available;Not Available 2012;2012 Not Available Not Available Not Available Not Available Not Available Not Available Not Available Not Available NOT HISPANIC OR LATINO NA NO NO NO Not Available TCGA-86-6562-F29765 a47bbc7f-a0b3-4a1f-bf61-b6fd5f425572 28 Not Available Not Available 376 Not Available 274 Not Available Scheduled Follow-up Submission Progressive Disease 0 Not Available 3 Not Available YES Not Available WITH TUMOR YES Progressive Disease Not Available NO Dead 2012 MALE Lung Adenocarcinoma- Not Otherwise Specified (NOS) No C34.1 8140/3 C34.1 YES Not Available NO Not Available Not Available Central Lung 6 Not Available Not Available Not Available Not Available Not Available Not Available Not Available Not Available Not Available Not Available Not Available No 6562 Not Available TUMOR FREE Not Available Not Available Not Available Not Available Not Available Not Available NO WHITE Not Available NA R0 Not Applicable Not Applicable Not Applicable Not Applicable Not Applicable Not Applicable Not Applicable Not Applicable Not Applicable Stage IIA Not Applicable Not Applicable Not Applicable 7th Not Applicable Not Applicable Not Applicable M0 N1 T2a Not Available YES NO 86 1 Lung Alive 2011 2011 Not Available

19 Dead T3 N0 M0 Stage IIB NA 59 R-Upper Not Applicable TCGA-86-8672 c0e263eb-1a83-4dc8-8abe-3dd2a59bae1b 31 -21682 Not Applicable 0 15 Not Available Lung Adenocarcinoma Not Available NA Unknown Not Available Not Available Not Available Not Available Not Available Not Available Not Available NOT HISPANIC OR LATINO NA Not Available Not Available Not Available Not Available TCGA-86-8672-F46401 892123F6-665F-4DAC-96FE-4E45BD22A9D6 7 Not Applicable Not Applicable 19 Not Available Not Applicable Not Evaluated Scheduled Follow-up Submission Unknown Not Evaluated NO 8 Not Available NO Not Evaluated Unknown NO Complete Remission/Response Not Available NO Dead 2013 MALE Lung Adenocarcinoma- Not Otherwise Specified (NOS) No C34.1 8140/3 C34.1 YES Unknown NO Not Available Not Available Central Lung 8 Not Available Not Available Not Available Not Available Not Available Not Available Not Available Not Available Not Available Unknown Not Available No 8672 Unknown TUMOR FREE Not Available Not Available Unknown Not Available Not Available Unknown NO WHITE Unknown NA R0 Not Applicable Not Applicable Not Applicable Not Applicable Not Applicable Not Applicable Not Applicable Not Applicable Not Applicable Stage IIB Not Applicable Not Applicable Not Applicable 7th Not Applicable Not Applicable Not Applicable M0 N0 T3 Not Available YES NO 86 1 Lung Alive 2012 2012 Not Available

385 Alive T2 N0 M0 Stage IB NA 73 L-Lower Not Applicable TCGA-67-3774 36ad7fc7-0cc1-4f01-aef1-f89b3cb4feac 3 -26704 Not Applicable 0 385 Not Available Lung Adenocarcinoma Not Available NA Not Available Not Available NO Not Available Not Available Not Available NO Not Available NOT HISPANIC OR LATINO NA FEMALE Lung Adenocarcinoma- Not Otherwise Specified (NOS) No C34.3 8140/3 C34.3 YES Not Available NO Not Available Not Available Not Available 3 Not Available Not Available Not Available Not Available Not Available Not Available Not Available Not Available Not Available Not Available Not Available No 3774 Not Available TUMOR FREE Not Available Not Available Not Available Not Available Not Available Not Available Not Available WHITE Not Available NA RX Not Applicable Not Applicable Not Applicable Not Applicable Not Applicable Not Applicable Not Applicable Not Applicable Not Applicable Stage IB Not Applicable Not Applicable Not Applicable 6th Not Applicable Not Applicable Not Applicable M0 N0 T2 Not Available YES NO 67 3 Lung Alive 2011 2009 Not Available

520 Alive T2a N0 MX Stage IB NA 70 R-Upper Not Applicable TCGA-55-8514 6845099a-b2c9-48d8-b856-9903d39bb92e 11 -25608 Not Applicable 0 202 Not Available Lung Adenocarcinoma 90 NA Not Evaluated Not Available Unknown Not Available Not Available Not Available Unknown Not Available NOT HISPANIC OR LATINO NA Not Available Not Available Not Available Not Available TCGA-55-8514-F57917 6DD813FA-5CF4-4E9F-AA13-F9EA9CC8391B 28 Not Applicable Not Applicable Not Applicable 520 Not Applicable Not Evaluated Scheduled Follow-up Submission Complete Remission/Response Not Evaluated NO 3 Not Available NO Not Available TUMOR FREE NO Complete Remission/Response Not Available NO Alive 2014 FEMALE Lung Adenocarcinoma- Not Otherwise Specified (NOS) No C34.1 8140/3 C34.1 YES Not Evaluated Unknown Not Available Not Available Unknown 1 Not Available Not Available Not Available Not Available Not Available Not Available Not Available Not Available Not Available NO 20 No 8514 Not Available TUMOR FREE Not Available Not Available NO 114 85 Complete Remission/Response YES BLACK OR AFRICAN AMERICAN NO NA R0 Not Applicable Not Applicable Not Applicable Not Applicable Not Applicable Not Applicable Not Applicable Not Applicable Not Applicable Stage IB Not Applicable Not Applicable Not Applicable 7th Not Applicable Not Applicable Not Applicable MX N0 T2a 2011 YES NO 55 4 Lung Alive 2013 2012 1971

124 Dead T2a N1 M0 Stage IIA NA 79 L-Upper Not Applicable TCGA-86-8055 34d8e84e-c3e1-417d-8b9b-8563d9fa0f8e 9 -29124 Not Applicable 0 10 Not Available Lung Adenocarcinoma Not Available NA 2 Not Available NO Not Available Not Available Not Available Not Available Not Available NOT HISPANIC OR LATINO NA Not Available Not Available NO Not Available TCGA-86-8055-F40636 BA1E1408-283C-44C3-BA03-E0144E835EC2 26 Not Applicable Not Applicable 124 Not Available Not Applicable Not Evaluated Scheduled Follow-up Submission Complete Remission/Response Not Evaluated NO 2 Not Available NO Not Evaluated TUMOR FREE NO Complete Remission/Response Not Available NO Dead 2013 MALE Lung Adenocarcinoma- Not Otherwise Specified (NOS) No C34.1 8140/3 C34.1 YES 80 NO Not Available Not Available Peripheral Lung 5 Not Available Not Available Not Available Not Available Not Available Not Available Not Available Not Available Not Available Unknown 60 No 8055 Preoperative TUMOR FREE Not Available Not Available Unknown Not Available Not Available Unknown NO WHITE Unknown NA R0 Not Applicable Not Applicable Not Applicable Not Applicable Not Applicable Not Applicable Not Applicable Not Applicable Not Applicable Stage IIA Not Applicable Not Applicable Not Applicable 7th Not Applicable Not Applicable Not Applicable M0 N1 T2a Not Available YES NO 86 2 Lung Alive 2012 2011 1955

578 Alive T2 N0 M0 Stage IB NA 76 R-Upper Not Applicable TCGA-05-4403 ce15f31f-2bad-4485-96fa-495bfa262e66 22 -27881 Not Applicable 0 578 Not Available Lung Adenocarcinoma Not Available NA Not Available Not Available Not Available Not Available Not Available Not Available Not Available Not Available Not Available NA MALE Lung Adenocarcinoma Mixed Subtype No C34.1 8255/3 C34.1 YES Not Available Not Available Not Available Not Available Not Available 7 Not Available Not Available Not Available Not Available Not Available Not Available Not Available Not Available Not Available Not Available Not Available No 4403 Not Available Not Available Not Available Not Available Not Available Not Available Not Available Not Available Not Available Not Available Not Available NA R0 Not Applicable Not Applicable Not Applicable Not Applicable Not Applicable Not Applicable Not Applicable Not Applicable Not Applicable Stage IB Not Applicable Not Applicable Not Applicable 5th Not Applicable Not Applicable Not Applicable M0 N0 T2 1975 NO YES 05 3 Lung Alive 2010 2006 Not Available

610 Alive T2 N0 M0 Stage IB NA 74 R-Lower Not Applicable TCGA-05-4405 f587c9ab-2949-4410-80d6-a050865d48aa 22 -27241 Not Applicable 0 610 Not Available Lung Adenocarcinoma Not Available NA Not Available Not Available Not Available Not Available Not Available Not Available Not Available Not Available Not Available NA Not Available Not Available Not Available Not Available TCGA-05-4405-F36494 83F914FE-F0D0-4B75-9F4B-DD17F59C62A0 31 Not Applicable Not Applicable Not Applicable 610 Not Applicable Not Available Scheduled Follow-up Submission Complete Remission/Response Not Available NO 10 Not Available NO Not Available TUMOR FREE NO Not Applicable Not Available NO Alive 2012 FEMALE Lung Adenocarcinoma Mixed Subtype No C34.3 8255/3 C34.3 YES Not Available Not Available Not Available Not Available Not Available 7 Not Available Not Available Not Available Not Available Not Available Not Available Not Available Not Available Not Available Not Available 65 Yes 4405 Not Available TUMOR FREE Not Available Not Available Not Available Not Available Not Available Not Available Not Available Not Available Not Available NA R0 Not Applicable Not Applicable Not Applicable Not Applicable Not Applicable Not Applicable Not Applicable Not Applicable Not Applicable Stage IB Not Applicable Not Applicable Not Applicable 5th Not Applicable Not Applicable Not Applicable M0 N0 T2 1994 NO YES 05 4 Lung Alive 2010 2006 1951

435 Alive T1a N0 M0 Stage IA NA 72 R-Upper Not Applicable TCGA-97-8179 11c2e619-8aeb-4f86-b03b-78f9a8099263 13 -26305 Not Applicable 0 15 Not Available Lung Adenocarcinoma 73 NA 0 Not Available YES Not Available Not Available FISH YES Not Available NOT HISPANIC OR LATINO NA Not Available Not Available Not Available Not Available TCGA-97-8179-F44247 0FB47890-87D5-4A74-80DE-3F0F16E906DB 19 Not Applicable Not Applicable Not Applicable 435 Not Applicable Not Evaluated Scheduled Follow-up Submission Complete Remission/Response Not Evaluated NO 6 Not Available NO Not Evaluated TUMOR FREE NO Complete Remission/Response Not Available NO Alive 2013 MALE Lung Acinar Adenocarcinoma No C34.1 8550/3 C34.1 YES Not Available YES NO Not Available Not Available 6 Not Available Not Available Not Available Not Available Not Available Not Available Not Available Not Available Not Available NO 60 No 8179 Preoperative TUMOR FREE 91 95 NO 86 90 Complete Remission/Response YES WHITE NO NA Not Available Not Applicable Not Applicable Not Applicable Not Applicable Not Applicable Not Applicable Not Applicable Not Applicable Not Applicable Stage IA Not Applicable Not Applicable Not Applicable 7th Not Applicable Not Applicable Not Applicable M0 N0 T1a 1988 YES NO 97 3 Lung Alive 2012 2012 1958

1163 Alive T1b N0 M0 Stage IA NA 77 R-Lower Not Applicable TCGA-44-3398 ffb0c0b7-165e-4439-b3e6-62431f40b7fe 11 -28392 Not Applicable 0 253 Not Available Lung Adenocarcinoma 60 NA Not Available Not Available Not Available Not Available Not Available Not Available Not Available Not Available NOT HISPANIC OR LATINO NA Not Available;Not Available Not Available;Not Available Not Available;Not Available Not Available;Not Available TCGA-44-3398-F5292;TCGA-44-3398-F38762 b338b0a9-78f2-44d5-a19c-5beca343abd0;9EF69CF2-2F1A-43EA-AACA-49F8E51CC52C 11;21 Not Applicable;Not Applicable Not Applicable;Not Applicable Not Applicable;Not Applicable 253;1163 Not Applicable;Not Applicable Not Available;Not Available Not Available;Scheduled Follow-up Submission Complete Remission/Response;Complete Remission/Response Not Available;Not Available Not Available;NO 10;12 Not Available;Not Available NO;NO Not Available;Not Available TUMOR FREE;TUMOR FREE NO;NO Complete Remission/Response;Complete Remission/Response Not Available;Not Available NO;NO Alive;Alive 2010;2012 FEMALE Lung Adenocarcinoma- Not Otherwise Specified (NOS) No C34.3 8140/3 C34.3 YES Not Available NO Not Available Not Available Not Available 10 Not Available Not Available Not Available Not Available Not Available Not Available Not Available Not Available Not Available Not Available 60 No 3398 Not Available TUMOR FREE 89 96 Not Available 84 87 Not Available YES WHITE Not Available NA R0 Not Applicable Not Applicable Not Applicable Not Applicable Not Applicable Not Applicable Not Applicable Not Applicable Not Applicable Stage IA Not Applicable Not Applicable Not Applicable 6th Not Applicable Not Applicable Not Applicable M0 N0 T1b 2009 YES NO 44 4 Lung Alive 2010 2009 1959

719 Alive T1b N0 M0 Stage IA NA 52 L-Upper Not Applicable TCGA-44-7672 4036c6fc-c82e-47bb-a384-6f299dc30b52 10 -19200 Not Applicable 0 418 Not Available Lung Adenocarcinoma 82 NA Not Available Not Available Not Available Not Available Not Available Not Available Not Available Not Available NOT HISPANIC OR LATINO NA Not Available;Not Available Not Available;Not Available Not Available;Not Available Not Available;Not Available TCGA-44-7672-F20374;TCGA-44-7672-F39074 80e34e50-79ea-44cf-9fc7-6a93b7e88cf0;1624E3CC-EAF9-4542-BB4C-CE2D8256625D 10;10 Not Applicable;Not Applicable Not Applicable;Not Applicable Not Applicable;Not Applicable 418;719 Not Applicable;Not Applicable Not Available;Not Available Scheduled Follow-up Submission;Scheduled Follow-up Submission Complete Remission/Response;Complete Remission/Response Not Available;Not Available Not Available;NO 1;1 Not Available;Not Available NO;NO Not Available;Not Available TUMOR FREE;TUMOR FREE NO;NO Complete Remission/Response;Complete Remission/Response Not Available;Not Available NO;NO Alive;Alive 2012;2013 FEMALE Lung Adenocarcinoma- Not Otherwise Specified (NOS) No C34.1 8140/3 C34.1 YES Not Available Not Available Not Available Not Available Not Available 1 Not Available Not Available Not Available Not Available Not Available Not Available Not Available Not Available Not Available Not Available 20 No 7672 Not Available TUMOR FREE Not Available Not Available Not Available 90 105 Not Available YES WHITE Not Available NA Not Available Not Applicable Not Applicable Not Applicable Not Applicable Not Applicable Not Applicable Not Applicable Not Applicable Not Applicable Stage IA Not Applicable Not Applicable Not Applicable 7th Not Applicable Not Applicable Not Applicable M0 N0 T1b Not Available YES NO 44 2 Lung Alive 2012 2010 1980

477 Alive T1a N0 M0 Stage IA NA 67 L-Upper Not Applicable TCGA-95-7947 e0acafdd-06d0-48a6-bd68-577ef666a419 21 -24752 Not Applicable 0 40 Not Available Lung Adenocarcinoma Not Available NA Not Available Not Available Not Available Not Available Not Available Not Available Not Available Not Available NOT HISPANIC OR LATINO NA Not Available;Not Available Not Available;Not Available Not Available;Not Available Not Available;Not Available TCGA-95-7947-F29538;TCGA-95-7947-F40940 B3F9AD18-04AD-45A3-8BF2-D7B6E98FFF80;D77E598A-8BC0-454B-A226-D9BB32070504 22;7 Not Applicable;Not Applicable Not Applicable;Not Applicable Not Applicable;Not Applicable 40;477 Not Applicable;Not Applicable Not Available;Not Available Scheduled Follow-up Submission;Scheduled Follow-up Submission Not Available;Complete Remission/Response Not Available;Not Available Not Available;NO 3;3 Not Available;Not Available NO;NO Not Available;Not Available Not Available;TUMOR FREE NO;NO Complete Remission/Response;Complete Remission/Response Not Available;Not Available NO;NO Alive;Alive 2012;2013 MALE Lung Adenocarcinoma Mixed Subtype No C34.1 8255/3 C34.1 YES Not Available Not Available Not Available Not Available Not Available 3 Not Available Not Available Not Available Not Available Not Available Not Available Not Available Not Available Not Available Not Available 100 Yes 7947 Not Available TUMOR FREE Not Available 37 Not Available Not Available 37 Not Available YES WHITE Not Available NA R0 Not Applicable Not Applicable Not Applicable Not Applicable Not Applicable Not Applicable Not Applicable Not Applicable Not Applicable Stage IA Not Applicable Not Applicable Not Applicable 7th Not Applicable Not Applicable Not Applicable M0 N0 T1a 2006 YES NO 95 4 Lung Alive 2012 2011 1956

1189 Alive T1b N0 M0 Stage IA NA 58 R-Lower Not Applicable TCGA-73-7498 ad74c0e8-8878-4efc-997d-981cd547bc5b 31 -21527 Not Applicable 0 621 Not Available Lung Adenocarcinoma 74.0 NA TCGA-73-7498-D32539;TCGA-73-7498-D32540;TCGA-73-7498-D32543;TCGA-73-7498-D48756;TCGA-73-7498-D48757 0809DF70-4919-4CE8-926D-7348AC6DC9B7;DE51B406-96D0-467C-9B21-E48C69E073BA;554C1877-3CCE-449E-9CA4-5FF8E51826CE;5FD1CDCF-3998-4DC4-A725-FE3A445BE552;C2185383-54C4-42AD-B2BF-402648CCF841 Not Available;Not Available;Not Available;Not Available;Not Available 31;31;31;20;20 110;68;110;131;131 47;47;68;47;47 Pemetrexed;Cisplatin;Carboplatin;pemetrexed;cisplatin Complete Response;Unknown;Complete Response;Complete Response;Complete Response 5;5;5;9;9 Not Available;Not Available;Not Available;Not Available;Not Available Not Available;Not Available;Not Available;Not Available;Not Available Not Available;Not Available;Not Available;Not Available;Not Available Not Available;Not Available;Not Available;Not Available;Not Available Not Applicable;Not Applicable;Not Applicable;Not Applicable;Not Applicable Not Available;Not Available;Not Available;Not Available;Not Available Not Available;Not Available;Not Available;Not Available;Not Available NO;NO;NO;NO;NO Chemotherapy;Chemotherapy;Chemotherapy;Chemotherapy;Chemotherapy Not Available;Not Available;Not Available;Not Available;Not Available Not Available;Not Available;Not Available;Not Available;Not Available Not Available;Not Available;Not Available;Not Available;Not Available NO;NO;NO;NO;NO 2012;2012;2012;2013;2013 1 Not Available NO Not Available Not Available Not Available NO Not Available NOT HISPANIC OR LATINO NA Not Available Not Available Not Available Not Available TCGA-73-7498-F48755 E191A2F8-AD1C-44A4-97DF-59279AC7A789 20 Not Applicable Not Applicable Not Applicable 1189 Not Applicable 0 Scheduled Follow-up Submission Complete Remission/Response 100 NO 9 Not Available NO Pre-Adjuvant Therapy TUMOR FREE YES Complete Remission/Response Not Available NO Alive 2013 FEMALE Lung Bronchioloalveolar Carcinoma Nonmucinous No C34.3 8252/3 C34.3 YES 80 NO Not Available Not Available Peripheral Lung 5 Not Available Not Available Not Available Not Available Not Available Not Available Not Available Not Available Not Available NO 28 Yes, History of Synchronous/Bilateral Malignancy 7498 Preoperative TUMOR FREE 89.6 60.1 YES 91.9 61.2 Complete Remission/Response YES WHITE NO NA R0 Not Applicable Not Applicable Not Applicable Not Applicable Not Applicable Not Applicable Not Applicable Not Applicable Not Applicable Stage IA Not Applicable Not Applicable Not Applicable 7th Not Applicable Not Applicable Not Applicable M0 N0 T1b 1997 NO YES 73 4 Lung Alive 2012 2010 1969

1367 Alive T1 N1 M0 Stage IIB NA 65 Discrepancy Not Available TCGA-44-2659 bf15f7ad-9d92-473b-91d1-f24aa373ab97 11 -23808 Not Applicable 0 529 Not Available Lung Adenocarcinoma 67 NA TCGA-44-2659-D5025;TCGA-44-2659-D5026 110a7cbc-d353-458a-93f3-10c16adcb893;cc1ab570-e73a-425d-9314-fcd5bcd1998e Not Available;Not Available 20;20 101;101 39;39 Alimta;Cisplatin Not Available;Not Available 10;10 4;4 1100;160 mg;mg ADJUVANT;ADJUVANT Not Applicable;Not Applicable 1;1 IV;IV NO;NO Chemotherapy;Chemotherapy Not Available;Not Available 4400;640 mg;mg Not Available;Not Available 2010;2010 1 Not Available Not Available Not Available Not Available Not Available Not Available Not Available NOT HISPANIC OR LATINO NA Not Available;Not Available;YES;Not Available Not Available;Not Available;NO;Not Available Not Available;Not Available;NO;Not Available Not Available;Not Available;Not Available;Not Available TCGA-44-2659-F5024;TCGA-44-2659-F10479;TCGA-44-2659-F32677;TCGA-44-2659-F40300 075c8c08-1113-488d-9a27-0bfe68135696;cf63a898-7c03-4b02-8491-13b75918bc4b;280f4a45-10dc-4ed2-943f-73782c35f16f;2386C68E-B1CA-41B8-A7E5-129D0CE2E2CA 20;27;7;15 Not Applicable;Not Available;Not Available;Not Applicable Not Applicable;Not Available;Not Available;Not Applicable Not Applicable;Not Applicable;Not Applicable;Not Applicable 529;701;1147;1367 Not Applicable;Not Available;1146;Not Applicable 1;1;Not Available;Not Available Not Available;Not Available;Additional New Tumor Event;Scheduled Follow-up Submission Complete Remission/Response;Complete Remission/Response;Complete Remission/Response;Complete Remission/Response Not Available;Not Available;Not Available;Not Available Not Available;Not Available;NO;NO 10;4;6;2 Not Available;Not Available;Locoregional Recurrence;Not Available NO;Not Available;YES;NO Adjuvant therapy;Adjuvant therapy;Not Available;Not Available TUMOR FREE;TUMOR FREE;WITH TUMOR;TUMOR FREE YES;Not Available;YES;YES Complete Remission/Response;Not Available;Complete Remission/Response;Complete Remission/Response Not Available;Not Available;Biopsy with Histologic Confirmation;Convincing Imaging;Not Available NO;Not Available;NO;NO Alive;Alive;Alive;Alive 2010;2011;2012;2013 FEMALE Lung Adenocarcinoma- Not Otherwise Specified (NOS) No C34.3 8140/3 C34.3 YES Not Available NO Not Available Not Available Not Available 10 Not Available Not Available Not Available Not Available Not Available Not Available Not Available Not Available Not Available Not Available 57 No 2659 Other TUMOR FREE 87 73 Not Available 77 64 Not Available YES WHITE Not Available NA R0 Not Applicable Not Applicable Not Applicable Not Applicable Not Applicable Not Applicable Not Applicable Not Applicable Not Applicable Stage IIB Not Applicable Not Applicable Not Applicable 6th Not Applicable Not Applicable Not Applicable M0 N1 T1 2003 YES NO 44 4 Lung Alive 2010 2009 1965

601 Alive T1a N0 M0 Stage IA NA 52 R-Upper Not Applicable TCGA-97-A4M1 C0C879F7-C332-4D31-9F7E-A3BF361B36EE 7 -19272 Not Applicable 0 209 Not Available Lung Adenocarcinoma 111 NA 0 Not Available NO Not Available Not Available Not Available NO Not Available NOT HISPANIC OR LATINO NA Not Available Not Available Not Available Not Available TCGA-97-A4M1-F57386 4503E4C1-A9BE-4125-9311-A8140E6486AF 10 Not Applicable Not Applicable Not Applicable 601 Not Applicable 0 Scheduled Follow-up Submission Complete Remission/Response Unknown NO 3 Not Available NO Other TUMOR FREE NO Complete Remission/Response Not Available NO Alive 2014 FEMALE Lung Adenocarcinoma Mixed Subtype No C34.1 8255/3 C34.1 YES Not Evaluated NO Not Available Not Available Unknown 3 Not Available Not Available Not Available Not Available Not Available Not Available Not Available Not Available Not Available NO 3 No A4M1 Preoperative Unknown 92 105 NO 87 100 Complete Remission/Response YES WHITE NO NA Not Evaluated Not Applicable Not Applicable Not Applicable Not Applicable Not Applicable Not Applicable Not Applicable Not Applicable Not Applicable Stage IA Not Applicable Not Applicable Not Applicable 7th Not Applicable Not Applicable Not Applicable M0 N0 T1a 1975 YES NO 97 3 Lung Alive 2013 2012 1972

18 Dead T1a N0 MX Stage IA NA 76 R-Upper Not Applicable TCGA-97-7938 e6e75590-9520-4b1c-8a28-4a1d2b2218fc 13 -27980 18 0 Not Available Not Available Lung Adenocarcinoma 53 NA Not Available Not Available YES Not Available Not Available Not Available NO Not Available NOT HISPANIC OR LATINO NA Not Available Not Available Not Available Not Available TCGA-97-7938-F57460 0EB1C458-4194-49CB-8BC9-D806490FF5E5 11 Not Applicable Not Applicable 18 Not Available Not Applicable Not Evaluated Scheduled Follow-up Submission Complete Remission/Response Not Evaluated NO 3 Not Available NO Not Evaluated TUMOR FREE NO Complete Remission/Response Not Available NO Dead 2014 FEMALE Lung Bronchioloalveolar Carcinoma Nonmucinous No C34.1 8252/3 C34.1 YES Not Available YES YES G12C Not Available 2 Not Available Not Available Not Available Not Available Not Available Not Available Not Available Not Available Not Available Not Available 40 No 7938 Not Available TUMOR FREE 59 54 Not Available 58 50 Not Available YES WHITE Not Available NA R0 Not Applicable Not Applicable Not Applicable Not Applicable Not Applicable Not Applicable Not Applicable Not Applicable Not Applicable Stage IA Not Applicable Not Applicable Not Applicable 7th Not Applicable Not Applicable Not Applicable MX N0 T1a 1996 YES NO 97 3 Lung Dead 2012 2011 Not Available

977 Alive T2a N0 MX Stage IB NA 73 L-Lower Not Applicable TCGA-55-8207 b256e065-c681-4f82-8b68-a8ef7c413791 8 -26897 Not Applicable 0 66 Not Available Lung Adenocarcinoma 48 NA Unknown Not Available Unknown Not Available Not Available Not Available Unknown Not Available NOT HISPANIC OR LATINO NA Not Available Not Available Not Available Not Available TCGA-55-8207-F65849 29690F66-8E25-4910-9609-41662B16E775 30 Not Applicable Not Applicable Not Applicable 977 Not Applicable Not Available Scheduled Follow-up Submission Complete Remission/Response Not Available NO 9 Not Available NO Not Available TUMOR FREE NO Complete Remission/Response Not Available NO Alive 2014 MALE Lung Acinar Adenocarcinoma No C34.3 8550/3 C34.3 YES Unknown Unknown Not Available Not Available Unknown 7 Not Available Not Available Not Available Not Available Not Available Not Available Not Available Not Available Not Available NO Not Available Yes, History of Synchronous/Bilateral Malignancy 8207 Unknown TUMOR FREE 84 83 NO 86 84 Complete Remission/Response YES WHITE NO NA R0 Not Applicable Not Applicable Not Applicable Not Applicable Not Applicable Not Applicable Not Applicable Not Applicable Not Applicable Stage IB Not Applicable Not Applicable Not Applicable 7th Not Applicable Not Applicable Not Applicable MX N0 T2a Not Available YES NO 55 3 Lung Alive 2012 2011 Not Available

336 Dead T2b N0 M0 Stage IIA NA 58 R-Upper Not Applicable TCGA-MP-A4TF 86DAC334-F30E-438E-810A-8E45F69DBB7E 2 -21387 336 0 Not Available Not Available Lung Adenocarcinoma 77 NA TCGA-MP-A4TF-D41409;TCGA-MP-A4TF-D41410 DB475CAC-B2D9-461C-A714-D5BBD7B9CF48;51556339-4419-45EF-B7B5-597FECF9A3D5 Not Available;Not Available 18;18 151;133 48;48 Navelbine;Cisplatin Partial Response;Partial Response 3;3 Not Available;Not Available Not Available;Not Available Not Available;Not Available Not Available;Not Available Not Applicable;Not Applicable Not Available;Not Available Not Available;Not Available NO;NO Chemotherapy;Chemotherapy Not Available;Not Available Not Available;Not Available Not Available;Not Available NO;NO 2013;2013 Not Evaluated Not Available NO Not Available Not Available Not Available NO Not Available NOT HISPANIC OR LATINO NA FEMALE Lung Adenocarcinoma- Not Otherwise Specified (NOS) No C34.1 8140/3 C34.1 YES Not Evaluated NO Not Available Not Available Peripheral Lung 4 NO YES 195 Not Available Not Available YES 202 Distant Metastasis Biopsy with Histologic Confirmation;Convincing Imaging YES 40 No A4TF Not Evaluated WITH TUMOR Not Available 108 YES Not Available 109 Partial Remission/Response YES WHITE NO NA R0 Not Applicable Not Applicable Not Applicable Not Applicable Not Applicable Not Applicable Not Applicable Not Applicable Not Applicable Stage IIA Not Applicable Not Applicable Not Applicable 7th Not Applicable Not Applicable Not Applicable M0 N0 T2b 2010 NO YES MP 4 Lung Dead 2013 2010 1970

603 Alive T1b N0 MX Stage IA NA 67 L-Upper Not Applicable TCGA-NJ-A55R 7C7D777B-BF00-4C24-97FC-922580D5605F 27 -24738 Not Applicable 0 603 Not Available Lung Adenocarcinoma Not Available NA Not Available Not Available NO Not Available Not Available Not Available NO Not Available NOT HISPANIC OR LATINO NA Not Available;Not Available Not Available;Not Available Not Available;Not Available Not Available;Not Available TCGA-NJ-A55R-F50899;TCGA-NJ-A55R-F70652 02EB453B-5913-4F97-A3A3-6E67ECC7568E;2000A52F-31B2-4A1B-ACB6-36A88942F0E0 1;27 Not Applicable;Not Applicable Not Applicable;Not Applicable Not Applicable;Not Applicable 603;603 Not Applicable;Not Applicable Not Available;Not Available Scheduled Follow-up Submission;Scheduled Follow-up Submission Stable Disease;Stable Disease Not Available;Not Available NO;YES 11;2 Not Available;Not Available NO;NO Not Available;Not Available TUMOR FREE;TUMOR FREE NO;NO Stable Disease;Stable Disease Not Available;Not Available NO;NO Alive;Alive 2013;2015 MALE Lung Adenocarcinoma- Not Otherwise Specified (NOS) No C34.1 8230/3 C34.1 YES Not Available NO Not Available Not Available Unknown 9 Not Available Not Available Not Available Not Available Not Available Not Available Not Available Not Available Not Available NO 5 No A55R Not Available TUMOR FREE Not Available Not Available NO Not Available Not Available Stable Disease NO WHITE NO NA Not Available Not Applicable Not Applicable Not Applicable Not Applicable Not Applicable Not Applicable Not Applicable Not Applicable Not Applicable Stage IA Not Applicable Not Applicable Not Applicable 7th Not Applicable Not Applicable Not Applicable MX N0 T1b 1986 Not Available Not Available NJ 3 Lung Alive 2013 2011 Not Available

1258 Dead T2 N0 MX Stage IB NA 78 R-Lower Not Applicable TCGA-91-6829 1427cd18-5ad3-491a-9981-908e31ae49db 19 -28841 1258 0 Not Available Not Available Lung Adenocarcinoma Not Available NA 3 Not Available Not Available Not Available Not Available Not Available Not Available Not Available NOT HISPANIC OR LATINO NA Not Available Not Available Not Available Not Available TCGA-91-6829-F32591 3d7c1967-95c7-4bfe-ac6c-b8e1de1816c8 4 Not Applicable Not Applicable 1258 Not Available Not Applicable Not Available Scheduled Follow-up Submission Progressive Disease 0 NO 6 Not Available NO Not Available Unknown NO Progressive Disease Not Available NO Dead 2012 MALE Lung Bronchioloalveolar Carcinoma Nonmucinous No C34.3 8252/3 C34.3 YES Not Available NO Not Available Not Available Not Available 7 Not Available Not Available Not Available Not Available Not Available Not Available Not Available Not Available Not Available Not Available 94.5 No 6829 Other TUMOR FREE Not Available Not Available Not Available Not Available Not Available Not Available Not Available WHITE Not Available NA R0 Not Applicable Not Applicable Not Applicable Not Applicable Not Applicable Not Applicable Not Applicable Not Applicable Not Applicable Stage IB Not Applicable Not Applicable Not Applicable 5th Not Applicable Not Applicable Not Applicable MX N0 T2 2003 NO YES 91 4 Lung Dead 2011 2001 1940

1280 Alive T2 N0 M0 Stage IB NA FPPP TCGA 65 R-Upper Not Applicable TCGA-44-2662 ccda26c1-a6d6-4317-8cf8-8a87e15ce12e 12 -24014 Not Applicable 0 480 Not Available Lung Adenocarcinoma 78 NA TCGA-44-2662-D5033;TCGA-44-2662-D5035;TCGA-44-2662-D5034 49760024-30c8-4540-a1fc-44ee2672a837;ada4d1fb-41a4-4448-8509-1d01a0c093c1;11737323-43bd-4c4f-9c44-43683d677c92 Not Available;Not Available;Not Available 21;21;21 162;362;362 76;277;277 rec MAGE 3-AS + AS15 ACS1 / Placebo Vaccine;Taxol;Carboplatin Not Available;Not Available;Not Available 10;10;10 5;8;8 0.5;480;935 mL;mg;mg ADJUVANT;PROGRESSION;PROGRESSION Not Applicable;Not Applicable;Not Applicable 1;2;2 IM;IV;IV NO;NO;NO Other, specify in notes;Chemotherapy;Chemotherapy GSK Mage Vaccine Study;Not Available;Not Available 2.5;1608;3817 mL;mg;mg Not Available;Not Available;Not Available 2010;2010;2010 Not Available Not Available Not Available Not Available Not Available Not Available Not Available Not Available NOT HISPANIC OR LATINO NA YES;Not Available;Not Available YES;Not Available;Not Available NO;Not Available;NO NO;Not Available;Not Available TCGA-44-2662-F5032;TCGA-44-2662-F12836;TCGA-44-2662-F39415 b2f9a164-783a-4a61-ba4e-eab35f83bab7;8e0b739c-e99b-4cc0-863d-b56cc7271eb2;529CB643-2E54-48E0-8587-7ABEE3A9830E 21;9;22 Not Available;Not Applicable;Not Applicable Not Available;Not Applicable;Not Applicable Not Applicable;Not Applicable;Not Applicable 480;673;1280 245;Not Applicable;Not Applicable 1;0;Not Available Not Available;Not Available;Scheduled Follow-up Submission Partial Remission/Response;Complete Remission/Response;Complete Remission/Response Not Available;Not Available;Not Available Not Available;Not Available;NO 10;6;1 Not Available;Not Available;Not Available YES;NO;NO Post-Adjuvant Therapy;Post-Adjuvant Therapy;Not Available Discrepancy;TUMOR FREE;TUMOR FREE YES;YES;YES Progressive Disease;Progressive Disease;Progressive Disease Not Available;Not Available;Not Available NO;NO;NO Alive;Alive;Alive 2010;2011;2013 MALE Lung Adenocarcinoma- Not Otherwise Specified (NOS) No C34.1 8140/3 C34.1 YES Not Available NO Not Available Not Available Not Available 10 Not Available Not Available Not Available Not Available Not Available Not Available Not Available Not Available Not Available Not Available 56 Yes 2662 Not Available Discrepancy 94 73 Not Available 88 67 Not Available YES WHITE Not Available NA Local Recurrence TCGA-44-2662-R5036 1a2f8bdd-81c3-426c-ba0b-6b486d266c9a 1 21 316 277 Not Available 10 30 6000 NO EXTERNAL BEAM Not Applicable PROGRESSION Not Available cGy 2010 R0 Not Applicable Not Applicable Not Applicable Not Applicable Not Applicable Not Applicable Not Applicable Not Applicable Not Applicable Stage IB Not Applicable Not Applicable Not Applicable 6th Not Applicable Not Applicable Not Applicable M0 N0 T2 1984 YES NO 44 3 Lung Alive 2010 2009 1956

116 Dead T2b N0 M0 Stage IIA NA 48 R-Upper Not Applicable TCGA-86-A4D0 528A0FCE-0719-4B19-A69D-3F18681696C5 6 -17607 Not Applicable 0 30 Not Available Lung Adenocarcinoma Not Available NA TCGA-86-A4D0-D52966;TCGA-86-A4D0-D52968 6BF8E543-7F35-4B18-83AE-963B50ADA0CD;781B2224-221B-4BDA-9872-513C7BF06A99 Not Available;Not Available 9;9 80;80 76;76 Etoposide;Cisplatin Complete Response;Complete Response 12;12 Not Available;Not Available Not Available;Not Available Not Available;Not Available Not Available;Not Available Not Applicable;Not Applicable Not Available;Not Available Not Available;Not Available NO;NO Chemotherapy;Chemotherapy Not Available;Not Available Not Available;Not Available Not Available;Not Available NO;NO 2013;2013 1 Not Available NO Not Available Not Available Not Available NO Not Available NOT HISPANIC OR LATINO NA Not Available Not Available Not Available Not Available TCGA-86-A4D0-F52852 7EA60B7C-46CC-46A0-90F7-88199CDE946A 6 Not Applicable Not Applicable 116 Not Available Not Applicable Not Evaluated Scheduled Follow-up Submission Complete Remission/Response Not Evaluated NO 12 Not Available NO Not Evaluated TUMOR FREE YES Complete Remission/Response Not Available NO Dead 2013 MALE Lung Adenocarcinoma- Not Otherwise Specified (NOS) No C34.1 8140/3 C34.1 YES 90 NO Not Available Not Available Not Available 12 Not Available Not Available Not Available Not Available Not Available Not Available Not Available Not Available Not Available NO Not Available No A4D0 Preoperative TUMOR FREE Not Available Not Available Unknown Not Available Not Available Unknown NO WHITE Unknown NA R0 Not Applicable Not Applicable Not Applicable Not Applicable Not Applicable Not Applicable Not Applicable Not Applicable Not Applicable Stage IIA Not Applicable Not Applicable Not Applicable 7th Not Applicable Not Applicable Not Applicable M0 N0 T2b Not Available YES NO 86 2 Lung Alive 2012 2012 1983

568 Alive T2a N2 M1a Stage IV NA 81 Other (please specify) RUL and RML TCGA-97-8171 397d3f69-1453-4057-b177-8723eec923d1 13 -29627 Not Applicable 0 107 Not Available Lung Adenocarcinoma 110 NA TCGA-97-8171-D44343 26978718-D8A3-4747-A8BE-D3C96E87A6A3 Not Available 19 Not Available 16 Tarceva Not Available 6 Not Available Not Available Not Available Not Available Not Applicable Not Available Not Available YES Targeted Molecular therapy Not Available Not Available Not Available NO 2013 1 Not Available YES Exon 19 Deletion Not Available Not Available NO Not Available NOT HISPANIC OR LATINO NA YES NO Not Available YES TCGA-97-8171-F44232 98AD8302-28ED-452F-8867-B5A52BF7A2EE 19 Not Available 441 Not Applicable 568 441 Not Evaluated Scheduled Follow-up Submission Progressive Disease Not Evaluated NO 6 Distant Metastasis YES Not Evaluated WITH TUMOR YES Progressive Disease Biopsy with Histologic Confirmation NO Alive 2013 MALE Lung Papillary Adenocarcinoma No C34.8 8260/3 C34.8 YES Not Available YES NO Not Available Not Available 6 Not Available Not Available Not Available Not Available Not Available Not Available Not Available Not Available Not Available NO 65 No 8171 Preoperative WITH TUMOR 55 104 Unknown 55 99 Progressive Disease YES ASIAN Unknown NA Not Available Not Applicable Not Applicable Not Applicable Not Applicable Not Applicable Not Applicable Not Applicable Not Applicable Not Applicable Stage IV Not Applicable Not Applicable Not Applicable 7th Not Applicable Not Applicable Not Applicable M1a N2 T2a Not Available YES NO 97 2 Lung Alive 2012 2011 1946

179 Alive T1b N1 M0 Stage IIA NA 73 L-Upper Not Applicable TCGA-86-6851 035c0b2b-c722-443b-8962-db4ee92c7532 7 -26808 Not Applicable 0 0 Not Available Lung Adenocarcinoma Not Available NA Not Available Not Available Not Available Not Available Not Available Not Available Not Available Not Available NOT HISPANIC OR LATINO NA Not Available Not Available Not Available Not Available TCGA-86-6851-F53594 40FE93C5-B9C4-47D2-B194-C15151EBC3CF 12 Not Applicable Not Applicable Not Applicable 179 Not Applicable 0 Scheduled Follow-up Submission Complete Remission/Response 100 YES 12 Not Available NO Post-Adjuvant Therapy TUMOR FREE NO Complete Remission/Response Not Available NO Alive 2013 FEMALE Lung Adenocarcinoma Mixed Subtype No C34.1 8255/3 C34.1 YES Not Available Not Available Not Available Not Available Not Available 10 Not Available Not Available Not Available Not Available Not Available Not Available Not Available Not Available Not Available Not Available 52 No 6851 Not Available TUMOR FREE Not Available Not Available Not Available Not Available Not Available Not Available NO WHITE Not Available NA Not Available Not Applicable Not Applicable Not Applicable Not Applicable Not Applicable Not Applicable Not Applicable Not Applicable Not Applicable Stage IIA Not Applicable Not Applicable Not Applicable 7th Not Applicable Not Applicable Not Applicable M0 N1 T1b Not Available YES NO 86 2 Lung Alive 2011 2011 1959

864 Alive T2 N2 M0 Stage IIIA NA 61 L-Lower Not Applicable TCGA-64-5779 1c58d9b8-17a2-4fc6-9898-fe6f47bd2c2a 2 -22305 Not Applicable 0 507 Not Available Lung Adenocarcinoma 100 NA TCGA-64-5779-D11375;TCGA-64-5779-D11376;TCGA-64-5779-D31475;TCGA-64-5779-D31476 c0d60743-bdf0-436e-9702-4e8ce35c248d;621a2148-00ec-4c00-a0bc-980442ab9522;d2824bc2-e94d-4926-9b6b-0600e519bb80;b55016e7-e7a7-430d-93e0-377e6fb2fb50 Not Available;Not Available;Not Available;Not Available 2;2;3;3 157;157;157;157 94;94;94;94 Cisplatin;Pemetrexed;Cisplatin;Pemetrexed Not Available;Not Available;Complete Response;Complete Response 5;5;5;5 04;04;Not Available;Not Available 75;500;Not Available;Not Available mg/m2;mg/m2;Not Available;Not Available ADJUVANT;ADJUVANT;Not Available;Not Available Not Applicable;Not Applicable;Not Applicable;Not Applicable 01;01;Not Available;Not Available IV;IV;Not Available;Not Available NO;NO;NO;NO Chemotherapy;Chemotherapy;Chemotherapy;Chemotherapy Not Available;Not Available;Not Available;Not Available Not Available;Not Available;Not Available;Not Available Not Available;Not Available;Not Available;Not Available Not Available;Not Available;YES;YES 2011;2011;2012;2012 0 Not Available Not Available Not Available Not Available Not Available Not Available Not Available NOT HISPANIC OR LATINO NA Not Available;NO Not Available;YES Not Available;Not Available Not Available;YES TCGA-64-5779-F11373;TCGA-64-5779-F31473 04f76938-5fba-43c0-98dc-09975c964e2b;e630e4b1-4907-4640-a3cf-9909d6695755 2;3 Not Applicable;Not Available Not Applicable;797 Not Applicable;Not Applicable 507;864 Not Applicable;795 0;0 Not Available;Additional New Tumor Event Stable Disease;Unknown Not Available;Unknown Not Available;Not Available 5;5 Not Available;Distant Metastasis NO;YES Post-Adjuvant Therapy;Post-Adjuvant Therapy Not Available;Unknown YES;YES Stable Disease;Discrepancy Not Available;Not Available YES;YES Alive;Alive 2011;2012 MALE Lung Acinar Adenocarcinoma No C34.3 8550/3 C34.3 YES Not Available Not Available Not Available Not Available Central Lung 5 Not Available Not Available Not Available Not Available Not Available Not Available Not Available Not Available Not Available Not Available 40 No 5779 Post-Adjuvant Therapy TUMOR FREE Not Available Not Available Not Available 94 100 Not Available YES WHITE Not Available NA Primary Tumor Field;Primary Tumor Field;Distant site TCGA-64-5779-R11374;TCGA-64-5779-R31474;TCGA-64-5779-R31477 0eb7ccdf-6dff-4dd1-802d-f035caf16d3a;92e10658-9cdc-4b28-a801-c37c70128b36;2e1ad728-abff-4e0d-9a8b-01a72cad7afc 01;01;02 2;17;17 241;241;864 Not Available;201;852 Not Available;Not Available;Not Available 5;4;4 28;28;09 Not Available;5040;2700 NO;NO;NO EXTERNAL BEAM;EXTERNAL BEAM;EXTERNAL BEAM Not Applicable;Not Applicable;Not Applicable ADJUVANT;ADJUVANT;RECURRENCE Not Available;Not Available;Not Available cGy;cGy;cGy 2011;2012;2012 R0 Not Applicable Not Applicable Not Applicable Not Applicable Not Applicable Not Applicable Not Applicable Not Applicable Not Applicable Stage IIIA Not Applicable Not Applicable Not Applicable 6th Not Applicable Not Applicable Not Applicable M0 N2 T2 2009 NO YES 64 4 Lung Alive 2011 2009 1969

605 Alive T2 N0 M0 Stage IB NA 68 R-Lower Not Applicable TCGA-86-7954 079ae0b3-b64b-4b8e-ab7d-225b8046568c 30 -25062 Not Applicable 0 0 Not Available Lung Adenocarcinoma Not Available NA TCGA-86-7954-D57949;TCGA-86-7954-D57950 D1519FCC-3805-4E15-B46C-49EA3BCEEC7D;3D5B9CB0-F3CF-411C-8AFB-E3271BA29B8C Not Available;Not Available 28;28 81;81 55;55 Carboplatin;Taxol Complete Response;Complete Response 3;3 Not Available;Not Available Not Available;Not Available Not Available;Not Available Not Available;Not Available Not Applicable;Not Applicable Not Available;Not Available Not Available;Not Available NO;NO Chemotherapy;Chemotherapy Not Available;Not Available Not Available;Not Available Not Available;Not Available NO;NO 2014;2014 0 Not Available NO Not Available Not Available Not Available Not Available Not Available NOT HISPANIC OR LATINO NA Not Available Not Available Not Available Not Available TCGA-86-7954-F57948 F5479210-0898-47AC-989D-AD4BEE57AA48 28 Not Applicable Not Applicable Not Applicable 605 Not Applicable 0 Scheduled Follow-up Submission Complete Remission/Response 100 NO 3 Not Available NO Post-Adjuvant Therapy TUMOR FREE YES Complete Remission/Response Not Available NO Alive 2014 FEMALE Lung Bronchioloalveolar Carcinoma Nonmucinous No C34.3 8250/3 C34.3 YES 100 NO Not Available Not Available Peripheral Lung 3 Not Available Not Available Not Available Not Available Not Available Not Available Not Available Not Available Not Available Not Available 8 No 7954 Preoperative TUMOR FREE Not Available Not Available Not Available Not Available Not Available Not Available NO WHITE Not Available NA R0 Not Applicable Not Applicable Not Applicable Not Applicable Not Applicable Not Applicable Not Applicable Not Applicable Not Applicable Stage IB Not Applicable Not Applicable Not Applicable 7th Not Applicable Not Applicable Not Applicable M0 N0 T2 1971 YES NO 86 3 Lung Alive 2012 2011 1963

653 Dead T1 N0 M0 Stage IA NA 86 L-Upper Not Applicable TCGA-50-5935 100430c8-1446-45c8-af36-b6dbb3ddd0c1 2 -31610 653 0 653 Not Available Lung Adenocarcinoma Not Available NA Not Available Not Available YES Other Not Available Not Available Not Available Not Available NOT HISPANIC OR LATINO NA Not Available Not Available NO Not Available TCGA-50-5935-F32108 1e2da076-3f69-4de4-af22-9f3dfc1ce1f3 17 Not Applicable Not Applicable 653 Not Available Not Applicable Not Available Scheduled Follow-up Submission Complete Remission/Response Not Available NO 5 Not Available NO Not Available TUMOR FREE NO Complete Remission/Response Not Available YES Dead 2012 FEMALE Lung Adenocarcinoma- Not Otherwise Specified (NOS) No C34.1 8140/3 C34.1 YES Not Available YES YES Not Available Not Available 7 Not Available Not Available Not Available Not Available Not Available Not Available Not Available Not Available Not Available Not Available Not Available Yes 5935 Not Available Not Available Not Available Not Available Not Available Not Available Not Available Not Available Not Available WHITE Not Available NA Primary Tumor Field TCGA-50-5935-R32109 5aa7172f-ba04-4f8f-8dcc-82cb5f544459 1 17 0 0 Not Available 5 Not Available 100 NO IMPLANTS Not Applicable ADJUVANT Not Available cGy 2012 Not Available Not Applicable Not Applicable Not Applicable Not Applicable Not Applicable Not Applicable Not Applicable Not Applicable Not Applicable Stage IA Not Applicable Not Applicable Not Applicable 6th Not Applicable Not Applicable Not Applicable M0 N0 T1 Not Available NO YES 50 Not Available Lung Dead 2011 2006 Not Available

377 Alive T1a N0 M0 Stage IA NA 71 L-Lower Not Applicable TCGA-95-7944 abab925c-bef3-4072-b9c4-40f808744593 21 -26003 Not Applicable 0 21 Not Available Lung Adenocarcinoma 60 NA Not Available Not Available Not Available Not Available Not Available Not Available Not Available Not Available NOT HISPANIC OR LATINO NA Not Available;Not Available Not Available;Not Available Not Available;Not Available Not Available;Not Available TCGA-95-7944-F29525;TCGA-95-7944-F40938 747C761B-CB03-4D90-A93D-6C327834296D;9B9307F4-5CE2-402C-9634-04C7816FDE90 21;7 Not Applicable;Not Applicable Not Applicable;Not Applicable Not Applicable;Not Applicable 21;377 Not Applicable;Not Applicable Not Available;Not Available Scheduled Follow-up Submission;Scheduled Follow-up Submission Not Available;Complete Remission/Response Not Available;Not Available Not Available;NO 3;3 Not Available;Not Available NO;NO Not Available;Not Available Not Available;TUMOR FREE NO;NO Not Available;Stable Disease Not Available;Not Available NO;NO Alive;Alive 2012;2013 MALE Lung Adenocarcinoma- Not Otherwise Specified (NOS) No C34.3 8140/3 C34.3 YES Not Available Not Available Not Available Not Available Not Available 3 Not Available Not Available Not Available Not Available Not Available Not Available Not Available Not Available Not Available Not Available 50 No 7944 Not Available TUMOR FREE Not Available 87 Not Available Not Available 86 Not Available YES WHITE Not Available NA R0 Not Applicable Not Applicable Not Applicable Not Applicable Not Applicable Not Applicable Not Applicable Not Applicable Not Applicable Stage IA Not Applicable Not Applicable Not Applicable 7th Not Applicable Not Applicable Not Applicable M0 N0 T1a Not Available YES NO 95 2 Lung Alive 2012 2011 1961

84 Alive T1a N0 M0 Stage IA NA 63 L-Upper Not Applicable TCGA-MN-A4N5 17E09B0D-EAB2-40EC-AE3C-BE9AB63FCEF0 4 -23257 Not Applicable 0 84 Not Available Lung Adenocarcinoma 82 NA 0 Not Available YES Not Available Not Available IHC YES Not Available NOT HISPANIC OR LATINO NA Not Available Not Available Not Available Not Available TCGA-MN-A4N5-F56665 7484D5CB-5D52-4177-9FF8-CC427340FC45 17 Not Applicable Not Applicable Not Applicable 84 Not Applicable Not Available Scheduled Follow-up Submission Unknown Not Available YES 2 Not Available NO Not Available TUMOR FREE NO Complete Remission/Response Not Available NO Alive 2014 MALE Lung Adenocarcinoma- Not Otherwise Specified (NOS) No C34.1 8140/3 C34.1 YES 100 NO Not Available Not Available Unknown 12 Not Available Not Available Not Available Not Available Not Available Not Available Not Available Not Available Not Available Unknown Not Available No A4N5 Preoperative Unknown Not Available Not Available NO 77 105 Complete Remission/Response YES WHITE NO NA R0 Not Applicable Not Applicable Not Applicable Not Applicable Not Applicable Not Applicable Not Applicable Not Applicable Not Applicable Stage IA Not Applicable Not Applicable Not Applicable 7th Not Applicable Not Applicable Not Applicable M0 N0 T1a 2011 NO YES MN 4 Lung Alive 2012 2011 1971

424 Alive T4 N0 M0 Stage IIIB NA 51 R-Upper Not Applicable TCGA-53-7813 42d208bd-cd77-4bfb-ad53-9fc072a87393 14 -18875 Not Applicable 0 418 Not Available Lung Adenocarcinoma 88 NA TCGA-53-7813-D41063;TCGA-53-7813-D41065;TCGA-53-7813-D41069;TCGA-53-7813-D41072 C5ECD05C-F0BC-43C9-9F9B-747BF40673B6;CE53D275-2F09-480B-B9DD-7A65EC848C45;81C8185C-C45B-44F1-A49B-4F668D595E8F;B98BBB32-9B61-4BA9-8A39-D94A205614DC Not Available;Not Available;Not Available;Not Available 11;11;11;11 40;40;146;146 40;40;62;62 Cisplatin;docetaxel;carboplatin;Taxol Unknown;Unknown;Stable Disease;Stable Disease 3;3;3;3 Not Available;Not Available;Not Available;Not Available Not Available;Not Available;Not Available;Not Available Not Available;Not Available;Not Available;Not Available Not Available;Not Available;Not Available;Not Available Not Applicable;Not Applicable;Not Applicable;Not Applicable Not Available;Not Available;Not Available;Not Available Not Available;Not Available;Not Available;Not Available NO;NO;NO;NO Chemotherapy;Chemotherapy;Chemotherapy;Chemotherapy Not Available;Not Available;Not Available;Not Available Not Available;Not Available;Not Available;Not Available Not Available;Not Available;Not Available;Not Available NO;NO;NO;NO 2013;2013;2013;2013 1 Not Available NO Not Available Not Available Not Available NO Not Available NOT HISPANIC OR LATINO NA Not Available Not Available Not Available Not Available TCGA-53-7813-F39281 F1618090-79FF-4125-8030-11B958CA39B2 31 Not Available Not Available Not Applicable 424 Not Available Not Available Scheduled Follow-up Submission Not Applicable Not Available YES 1 Not Available Unknown Not Available Not Available YES Progressive Disease Not Available NO Alive 2013 FEMALE Lung Bronchioloalveolar Carcinoma Nonmucinous No C34.1 8252/3 C34.1 YES Not Available NO Not Available Not Available Not Available 3 Not Available Not Available Not Available Not Available Not Available Not Available Not Available Not Available Not Available Not Available 35 No 7813 Pre-Adjuvant Therapy TUMOR FREE 69 91 Not Available 67 90 Not Available YES WHITE Not Available NA Not Available Not Applicable Not Applicable Not Applicable Not Applicable Not Applicable Not Applicable Not Applicable Not Applicable Not Applicable Stage IIIB Not Applicable Not Applicable Not Applicable 6th Not Applicable Not Applicable Not Applicable M0 N0 T4 2008 NO YES 53 4 Lung Alive 2012 2008 1973

879 Dead T2 N2 MX Stage IIIA NA 51 L-Upper Not Applicable TCGA-49-AAR4 765AD5A5-134B-4680-A6D4-1DD113DC47CC 18 -18939 879 0 Not Available Not Available Lung Adenocarcinoma 109.4 NA TCGA-49-AAR4-D65009;TCGA-49-AAR4-D65010 4477C3EF-3A88-4C2F-955E-149887AC8C8F;AB97458F-B092-494A-BCEE-2FD308ED618A Not Available;Not Available 17;17 61;61 57;57 TAXOL;CARBOPLATIN Partial Response;Partial Response 9;9 Not Available;Not Available Not Available;Not Available Not Available;Not Available Not Available;Not Available Not Applicable;Not Applicable Not Available;Not Available Not Available;Not Available NO;NO Chemotherapy;Chemotherapy Not Available;Not Available Not Available;Not Available Not Available;Not Available NO;NO 2014;2014 1 Not Available Not Available Not Available Not Available Not Available Not Available Not Available NOT HISPANIC OR LATINO NA MALE Lung Adenocarcinoma- Not Otherwise Specified (NOS) No C34.1 8140/3 C34.1 YES 80 Not Available Not Available Not Available Not Available 6 Not Available Not Available Not Available Not Available Not Available Not Available Not Available Not Available Not Available NO 35 No AAR4 Preoperative WITH TUMOR 79.0 97.5 YES 81.2 100.2 Stable Disease YES BLACK OR AFRICAN AMERICAN YES NA Primary Tumor Field TCGA-49-AAR4-R65008 3013A2ED-A16A-4023-9E1D-FFE753568235 Not Available 17 153 118 Complete Response 9 Not Available 4,500 cGy NO External Not Applicable Not Available Not Available Not Available 2014 R0 Not Applicable Not Applicable Not Applicable Not Applicable Not Applicable Not Applicable Not Applicable Not Applicable Not Applicable Stage IIIA Not Applicable Not Applicable Not Applicable 6th Not Applicable Not Applicable Not Applicable MX N2 T2 Not Available NO YES 49 2 Lung Dead 2014 2006 Not Available

950 Dead T1 N0 M0 Stage IA NA 75 R-Upper Not Applicable TCGA-MP-A4TA 98EE294A-46ED-4572-BDD4-A932C3498A25 2 -27513 950 0 Not Available Not Available Lung Adenocarcinoma 46 NA 1 Not Available NO Not Available Not Available Not Available NO Not Available NOT HISPANIC OR LATINO NA FEMALE Lung Adenocarcinoma- Not Otherwise Specified (NOS) No C34.1 8140/3 C34.1 YES Not Evaluated NO Not Available Not Available Peripheral Lung 4 YES YES 725 Not Available Not Available NO Not Available Distant Metastasis Biopsy with Histologic Confirmation YES 55 Yes, History of Prior Malignancy A4TA Pre-Adjuvant Therapy WITH TUMOR Not Available 74 NO Not Available 65 Complete Remission/Response YES WHITE NO NA R0 Not Applicable Not Applicable Not Applicable Not Applicable Not Applicable Not Applicable Not Applicable Not Applicable Not Applicable Stage IA Not Applicable Not Applicable Not Applicable 6th Not Applicable Not Applicable Not Applicable M0 N0 T1 2006 NO YES MP 4 Lung Dead 2013 2006 1951

13 Alive T1b N1 M0 Stage IIA NA 56 R-Upper Not Applicable TCGA-NJ-A55O 8AB8C4BA-311E-41D0-85EA-A0245ACA1BDD 27 -20628 Not Applicable 0 13 Not Available Lung Adenocarcinoma Not Available NA Not Evaluated Not Available NO Not Available Not Available Not Available NO Not Available NOT HISPANIC OR LATINO NA Not Available Not Available Not Available Not Available TCGA-NJ-A55O-F50898 FFE4E344-2FBF-47D2-A1D5-8C91E9F635EC 1 Not Applicable Not Applicable Not Applicable 13 Not Applicable Not Available Scheduled Follow-up Submission Stable Disease Not Available YES 11 Not Available NO Not Available Not Available NO Stable Disease Not Available NO Alive 2013 FEMALE Mucinous (Colloid) Carcinoma No C34.1 8480/3 C34.1 YES Not Evaluated NO Not Available Not Available Unknown 9 Not Available Not Available Not Available Not Available Not Available Not Available Not Available Not Available Not Available NO 30 No A55O Not Evaluated TUMOR FREE Not Available Not Available NO Not Available Not Available Stable Disease NO WHITE NO NA Not Available Not Applicable Not Applicable Not Applicable Not Applicable Not Applicable Not Applicable Not Applicable Not Applicable Not Applicable Stage IIA Not Applicable Not Applicable Not Applicable 7th Not Applicable Not Applicable Not Applicable M0 N1 T1b 2000 Not Available Not Available NJ 4 Lung Alive 2013 2011 Not Available

730 Alive T2 N0 M0 Stage IB NA 82 L-Lower Not Applicable TCGA-05-4433 aea68827-dc0f-484d-a00d-06deeaa4b3ce 22 -30194 Not Applicable 0 730 Not Available Lung Adenocarcinoma Not Available NA Not Available Not Available Not Available Not Available Not Available Not Available Not Available Not Available Not Available NA Not Available Not Available Not Available Not Available TCGA-05-4433-F36530 B10301B6-0587-4CEE-84C4-26BEC5D40614 1 Not Applicable Not Applicable Not Applicable 730 Not Applicable Not Available Scheduled Follow-up Submission Complete Remission/Response Not Available NO 11 Not Available NO Not Available TUMOR FREE Unknown Not Applicable Not Available Unknown Alive 2012 MALE Lung Adenocarcinoma Mixed Subtype No C34.3 8255/3 C34.3 YES Not Available Not Available Not Available Not Available Not Available 7 Not Available Not Available Not Available Not Available Not Available Not Available Not Available Not Available Not Available Not Available 1 No 4433 Not Available TUMOR FREE Not Available Not Available Not Available Not Available Not Available Not Available Not Available Not Available Not Available NA R0 Not Applicable Not Applicable Not Applicable Not Applicable Not Applicable Not Applicable Not Applicable Not Applicable Not Applicable Stage IB Not Applicable Not Applicable Not Applicable 6th Not Applicable Not Applicable Not Applicable M0 N0 T2 1948 NO YES 05 3 Lung Alive 2010 2008 1946

193 Dead T1a N0 MX Stage IA NA 63 L-Lower Not Applicable TCGA-L9-A443 3B89AC82-63F6-41B9-A03D-34B9FFC1550D 26 -23062 Not Applicable 0 116 Not Available Lung Adenocarcinoma 61 NA 1 Not Available NO Not Available Not Available Not Available NO Not Available NOT HISPANIC OR LATINO NA Not Available Not Available Not Available Not Available TCGA-L9-A443-F46410 398C8700-07A7-4896-9EE8-F4E5D8693E79 7 Not Applicable Not Applicable 193 Not Available Not Applicable Unknown Scheduled Follow-up Submission Unknown Not Available NO 8 Not Available NO Unknown TUMOR FREE NO Complete Remission/Response Not Available NO Dead 2013 FEMALE Lung Adenocarcinoma- Not Otherwise Specified (NOS) No C34.3 8140/3 C34.3 YES Not Available NO Not Available Not Available Central Lung 10 Not Available Not Available Not Available Not Available Not Available Not Available Not Available Not Available Not Available NO 90 Yes, History of Prior Malignancy A443 Preoperative Not Available 74 69 NO Not Available Not Available Complete Remission/Response YES WHITE NO NA R0 Not Applicable Not Applicable Not Applicable Not Applicable Not Applicable Not Applicable Not Applicable Not Applicable Not Applicable Stage IA Not Applicable Not Applicable Not Applicable 7th Not Applicable Not Applicable Not Applicable MX N0 T1a 2005 YES NO L9 4 Lung Alive 2012 2012 1960

308 Dead T1 N0 M0 Stage IA NA 60 R-Lower Not Applicable TCGA-50-7109 7eb540b4-8dd3-4382-a488-e48b1c7213b3 9 -21979 Not Applicable 0 255 Not Available Lung Adenocarcinoma Not Available NA Not Available Not Available Not Available Not Available Not Available Not Available Not Available Not Available NOT HISPANIC OR LATINO NA NO NO NO NO TCGA-50-7109-F44136 DE5C9A3D-232A-4614-AA6C-B1156B85E208 13 Not Available Not Available 308 Not Available 15 Not Available Scheduled Follow-up Submission Progressive Disease Not Available NO 6 Distant Metastasis YES Not Available WITH TUMOR NO Progressive Disease Convincing Imaging NO Dead 2013 MALE Lung Adenocarcinoma- Not Otherwise Specified (NOS) No C34.3 8140/3 C34.3 YES Not Available Not Available Not Available Not Available Not Available 1 Not Available Not Available Not Available Not Available Not Available Not Available Not Available Not Available Not Available Not Available 120 No 7109 Not Available Not Available Not Available Not Available Not Available Not Available Not Available Not Available NO WHITE Not Available NA Not Available Not Applicable Not Applicable Not Applicable Not Applicable Not Applicable Not Applicable Not Applicable Not Applicable Not Applicable Stage IA Not Applicable Not Applicable Not Applicable 7th Not Applicable Not Applicable Not Applicable M0 N0 T1 Not Available NO YES 50 2 Lung Alive 2012 2011 Not Available

167 Dead T2 N0 M1 Stage IV NA 75 R-Lower Not Applicable TCGA-MP-A4T7 47062AD2-BA82-45C8-A871-BDEA84F0C405 2 -27603 167 0 Not Available Not Available Lung Adenocarcinoma 57 NA Not Evaluated Not Available NO Not Available Not Available Not Available NO Not Available Unknown NA FEMALE Lung Adenocarcinoma- Not Otherwise Specified (NOS) No C34.3 8140/3 C34.3 YES Not Evaluated NO Not Available Not Available Unknown 4 Not Available Not Available Not Available Not Available Not Available Not Available Not Available Not Available Not Available Unknown 50 No A4T7 Not Evaluated Unknown Not Available 91 NO Not Available 87 Unknown YES Unknown NO NA R0 Not Applicable Not Applicable Not Applicable Not Applicable Not Applicable Not Applicable Not Applicable Not Applicable Not Applicable Stage IV Not Applicable Not Applicable Not Applicable 6th Not Applicable Not Applicable Not Applicable M1 N0 T2 2004 NO YES MP 4 Lung Dead 2013 2005 1954

NA T2a N0 M0 Stage IB NA Not Available L-Upper Not Applicable TCGA-75-6205 9a63f744-4ed4-4b4d-aac6-f9bda66aa563 21 Not Available Not Available Not Available Not Available Not Available Lung Adenocarcinoma Not Available NA Not Available Not Available Not Available Not Available Not Available Not Available Not Available Not Available Not Available NA NO NO NO NO TCGA-75-6205-F15083 c118a061-f9b0-4470-ba1e-07a472245a8f 21 Not Available Not Available Not Available Not Available Not Available Not Available Not Available Not Available Not Available Not Available 7 Not Available YES Adjuvant therapy WITH TUMOR NO Complete Remission/Response Not Available NO Dead 2011 FEMALE Lung Adenocarcinoma- Not Otherwise Specified (NOS) No C34.1 8140/3 C34.1 YES Not Available Not Available Not Available Not Available Peripheral Lung 7 Not Available Not Available Not Available Not Available Not Available Not Available Not Available Not Available Not Available Not Available Not Available No 6205 Not Available TUMOR FREE Not Available Not Available Not Available Not Available Not Available Not Available Not Available Not Available Not Available NA R0 Not Applicable Not Applicable Not Applicable Not Applicable Not Applicable Not Applicable Not Applicable Not Applicable Not Applicable Stage IB Not Applicable Not Applicable Not Applicable 7th Not Applicable Not Applicable Not Applicable M0 N0 T2a Not Available NO YES 75 1 Lung Dead 2011 2006 Not Available

872 Alive T1b N0 M0 Stage IA NA 70 R-Middle Not Applicable TCGA-55-7281 659668e8-f0d9-4ff2-bbc8-9246f2ef49ab 6 -25870 Not Applicable 0 18 Not Available Lung Adenocarcinoma Not Available NA TCGA-55-7281-D47811;TCGA-55-7281-D47814;TCGA-55-7281-D47815;TCGA-55-7281-D47816;TCGA-55-7281-D47817;TCGA-55-7281-D47818 F65BFFAD-A23B-4066-829C-5D92F15865DE;B212E1D8-57BE-4365-BFBA-1EA76A701912;DC5037B4-AFDE-459F-8830-340492F0AA06;638427FC-0F5A-4F6A-8E41-E90771270BA7;5046F93F-1D01-4A67-9B87-ED115A6C742A;F582DDC9-3FBE-4AC6-870A-530C86640E54 Not Available;Not Available;Not Available;Not Available;Not Available;Not Available 28;28;28;28;28;28 474;579;579;Not Available;Not Available;Not Available 424;508;508;712;712;712 Carboplatin;Carboplatin;Alimta;Carboplatin;Taxol;Avastin Clinical Progressive Disease;Clinical Progressive Disease;Clinical Progressive Disease;Partial Response;Partial Response;Partial Response 8;8;8;8;8;8 Not Available;Not Available;Not Available;Not Available;Not Available;Not Available Not Available;Not Available;Not Available;Not Available;Not Available;Not Available Not Available;Not Available;Not Available;Not Available;Not Available;Not Available Not Available;Not Available;Not Available;Not Available;Not Available;Not Available Not Applicable;Not Applicable;Not Applicable;Not Applicable;Not Applicable;Not Applicable Not Available;Not Available;Not Available;Not Available;Not Available;Not Available Not Available;Not Available;Not Available;Not Available;Not Available;Not Available NO;NO;NO;NO;NO;NO Chemotherapy;Chemotherapy;Chemotherapy;Chemotherapy;Chemotherapy;Chemotherapy Not Available;Not Available;Not Available;Not Available;Not Available;Not Available Not Available;Not Available;Not Available;Not Available;Not Available;Not Available Not Available;Not Available;Not Available;Not Available;Not Available;Not Available NO;NO;NO;NO;NO;NO 2013;2013;2013;2013;2013;2013 Not Available Not Available Not Available Not Available Not Available Not Available Not Available Not Available Not Available NA YES YES NO NO TCGA-55-7281-F47807 0804D88A-04B2-4C56-9A68-68D39DDB1617 28 Not Available Not Available Not Applicable 872 339 Not Evaluated Scheduled Follow-up Submission Partial Remission/Response Not Evaluated NO 8 Distant Metastasis YES Not Evaluated WITH TUMOR NO Complete Remission/Response Convincing Imaging NO Alive 2013 FEMALE Lung Adenocarcinoma- Not Otherwise Specified (NOS) No C34.2 8140/3 C34.2 YES Not Available NO Not Available Not Available Not Available 10 Not Available Not Available Not Available Not Available Not Available Not Available Not Available Not Available Not Available Not Available 20 No 7281 Not Available TUMOR FREE Not Available Not Available Not Available Not Available Not Available Not Available Not Available WHITE Not Available NA Regional site;Distant site TCGA-55-7281-R47808;TCGA-55-7281-R47810 F3791E9B-5039-47A4-8E77-A47AF6CD8BE4;722364F3-700A-4E78-8A04-C6D4C2353780 Not Available;Not Available 28;28 370;647 339;634 Radiographic Progressive Disease;Radiographic Progressive Disease 8;8 Not Available;10 Not Available;03000 NO;NO External;External Not Applicable;Not Applicable Not Available;Not Available Not Available;Not Available Not Available;cGy 2013;2013 R0 Not Applicable Not Applicable Not Applicable Not Applicable Not Applicable Not Applicable Not Applicable Not Applicable Not Applicable Stage IA Not Applicable Not Applicable Not Applicable 7th Not Applicable Not Applicable Not Applicable M0 N0 T1b Not Available YES NO 55 4 Lung Alive 2011 2011 Not Available

651 Alive T2b N2 M0 Stage IIIA NA 74 R-Upper Not Applicable TCGA-95-A4VK D344DAE0-ADF4-4697-B703-7690C13D8331 21 -27040 Not Applicable 0 121 Not Available Lung Adenocarcinoma 81 NA TCGA-95-A4VK-D40500;TCGA-95-A4VK-D40512 676EB575-B085-4F0A-AAD2-CD3F2A17544E;F28DCD0F-6819-45FB-9F86-5FACA3E69090 Not Available;Not Available 21;21 121;121 76;76 Cisplatin;Alimta Stable Disease;Stable Disease 2;2 Not Available;Not Available Not Available;Not Available Not Available;Not Available Not Available;Not Available Not Applicable;Not Applicable Not Available;Not Available Not Available;Not Available NO;NO Chemotherapy;Chemotherapy Not Available;Not Available Not Available;Not Available Not Available;Not Available NO;NO 2013;2013 Not Available Not Available YES Not Available Not Available Not Available NO Not Available NOT HISPANIC OR LATINO NA NO YES Not Available NO TCGA-95-A4VK-F58477 7505B09D-6FAB-4A2B-9EA8-7146661CF8D2 10 Not Available Not Available Not Applicable 651 496 1 Scheduled Follow-up Submission Stable Disease Not Evaluated NO 4 Distant Metastasis YES Post-Adjuvant Therapy WITH TUMOR YES Stable Disease Convincing Imaging YES Alive 2014 FEMALE Lung Adenocarcinoma Mixed Subtype No C34.1 8255/3 C34.1 YES Not Available NO Not Available Not Available Not Available 2 Not Available Not Available Not Available Not Available Not Available Not Available Not Available Not Available Not Available NO 45 Yes, History of Prior Malignancy A4VK Not Available TUMOR FREE 72 99 YES 67 99 Stable Disease YES WHITE YES NA Regional site TCGA-95-A4VK-R40497 9E26A096-8F54-4594-B63A-E5754FBBD9DD Not Available 21 202 202 Stable Disease 2 28 50.4 NO External Not Applicable Not Available Not Available Gy 2013 RX Not Applicable Not Applicable Not Applicable Not Applicable Not Applicable Not Applicable Not Applicable Not Applicable Not Applicable Stage IIIA Not Applicable Not Applicable Not Applicable 7th Not Applicable Not Applicable Not Applicable M0 N2 T2b 1986 YES NO 95 3 Lung Alive 2013 2012 1956

1559 Alive T2 N0 M0 Stage IB NA 55 R-Upper Not Applicable TCGA-64-5781 41c4c704-825e-49f5-9d54-dbd7d30725e0 3 -20219 Not Applicable 0 1202 Not Available Lung Adenocarcinoma 73 NA TCGA-64-5781-D11407;TCGA-64-5781-D11405;TCGA-64-5781-D11406;TCGA-64-5781-D31491;TCGA-64-5781-D31492;TCGA-64-5781-D31493 92010938-ac4a-4ab1-b25e-dba6632b92ba;4ffd6d8f-3ded-49b0-a532-d0fb97ef1033;83cf163f-43dc-4a75-9ed6-a433a2226488;04bd06de-39e1-47d6-bdb4-de3aeccf9f63;86ff15d3-47d0-47e5-ad61-cded2ed6345d;acadc104-cf2b-4ac0-8598-f4e35edbb3d1 Not Available;Not Available;Not Available;Not Available;Not Available;Not Available 3;3;3;3;3;3 187;215;215;215;215;187 159;101;101;131;131;159 Bevacizumab;Cisplatin;Gemcitabine;Cisplatin;Gemcitabine;Bevacizumab Not Available;Not Available;Not Available;Complete Response;Complete Response;Complete Response 5;5;5;5;5;5 3;4;4;Not Available;Not Available;Not Available 15;75;1250;Not Available;Not Available;Not Available mg/kg;mg/m2;mg/m2;Not Available;Not Available;Not Available PROGRESSION;ADJUVANT;PROGRESSION;Not Available;Not Available;Not Available Not Applicable;Not Applicable;Not Applicable;Not Applicable;Not Applicable;Not Applicable 1;1;1;Not Available;Not Available;Not Available IV;IV;IV;Not Available;Not Available;Not Available NO;NO;NO;NO;NO;NO Chemotherapy;Chemotherapy;Chemotherapy;Chemotherapy;Chemotherapy;Chemotherapy Not Available;Not Available;Not Available;Not Available;Not Available;Not Available Not Available;Not Available;Not Available;Not Available;Not Available;Not Available Not Available;Not Available;Not Available;Not Available;Not Available;Not Available Not Available;Not Available;Not Available;NO;NO;NO 2011;2011;2011;2012;2012;2012 0 Not Available Not Available Not Available Not Available Not Available Not Available Not Available NOT HISPANIC OR LATINO NA YES;NO NO;NO NO;NO YES;YES TCGA-64-5781-F11404;TCGA-64-5781-F31490 2d57acf6-f8b7-4de5-bc21-d804fc3b145d;295e781b-ad5c-445b-8dbc-9d12b2b6495d 3;3 Not Available;Not Available 112;112 Not Applicable;Not Applicable 1202;1559 96;96 0;0 Not Available;Additional New Tumor Event Complete Remission/Response;Complete Remission/Response Not Available;Unknown Not Available;Not Available 5;5 Not Available;Distant Metastasis YES;YES Post-Adjuvant Therapy;Unknown TUMOR FREE;TUMOR FREE NO;NO Progressive Disease;Progressive Disease Not Available;Not Available NO;NO Alive;Alive 2011;2012 FEMALE Lung Adenocarcinoma Mixed Subtype No C34.1 8255/3 C34.1 YES Not Available Not Available Not Available Not Available Central Lung 5 Not Available Not Available Not Available Not Available Not Available Not Available Not Available Not Available Not Available Not Available 25 No 5781 Post-Adjuvant Therapy TUMOR FREE Not Available Not Available Not Available 61 77 Not Available YES WHITE Not Available NA R0 Not Applicable Not Applicable Not Applicable Not Applicable Not Applicable Not Applicable Not Applicable Not Applicable Not Applicable Stage IB Not Applicable Not Applicable Not Applicable 6th Not Applicable Not Applicable Not Applicable M0 N0 T2 2007 NO YES 64 4 Lung Alive 2011 2007 1982

NA T2 N1 M0 Stage IIB NA Not Available L-Upper Not Applicable TCGA-80-5607 645903b5-ba37-48a1-985b-a019b8db3236 12 Not Available Not Applicable Not Available Not Available Not Available Lung Adenocarcinoma Not Available NA 1 Not Available Not Available Not Available Not Available Not Available Not Available Not Available Not Available NA NO NO NO NO TCGA-80-5607-F15867 f401b266-27b0-450f-a213-254caa5e63c9 15 Not Available Not Available Not Applicable Not Available Not Available 1 Not Available Not Available Not Available Not Available 8 Not Available YES Not Available WITH TUMOR NO Not Available Not Available NO Alive 2011 FEMALE Lung Adenocarcinoma- Not Otherwise Specified (NOS) No C34.1 8140/3 C34.1 YES Not Available Not Available Not Available Not Available Peripheral Lung 8 Not Available Not Available Not Available Not Available Not Available Not Available Not Available Not Available Not Available Not Available 15 Yes 5607 Preoperative WITH TUMOR Not Available Not Available Not Available Not Available Not Available Not Available Not Available Not Available Not Available NA R0 Not Applicable Not Applicable Not Applicable Not Applicable Not Applicable Not Applicable Not Applicable Not Applicable Not Applicable Stage IIB Not Applicable Not Applicable Not Applicable 6th Not Applicable Not Applicable Not Applicable M0 N1 T2 1985 NO YES 80 3 Lung Alive 2011 2007 1965

805 Alive T1b N2 MX Stage IIIA NA 59 R-Lower Not Applicable TCGA-86-A4P8 646910EE-5F5D-40AC-A21A-75790F89430A 7 -21738 Not Applicable 0 39 Not Available Lung Adenocarcinoma 85 NA TCGA-86-A4P8-D48293;TCGA-86-A4P8-D48295 C22B8623-E5C2-4F22-8CCD-7859A4802584;054E5CD2-940A-4364-A458-39387314D2AE Not Available;Not Available 11;11 106;106 43;43 Cisplatin;Alimta Complete Response;Complete Response 9;9 Not Available;Not Available Not Available;Not Available Not Available;Not Available Not Available;Not Available Not Applicable;Not Applicable Not Available;Not Available Not Available;Not Available NO;NO Chemotherapy;Chemotherapy Not Available;Not Available Not Available;Not Available Not Available;Not Available NO;NO 2013;2013 Unknown Not Available YES Not Available Not Available FISH YES Not Available NOT HISPANIC OR LATINO NA Not Available;Not Available Not Available;Not Available Not Available;Not Available Not Available;Not Available TCGA-86-A4P8-F48289;TCGA-86-A4P8-F65331 34D83F16-C390-4C4F-882D-B34483F55C29;F8D137C4-C479-4576-A4AB-6F01C2973E15 11;22 Not Applicable;Not Applicable Not Applicable;Not Applicable Not Applicable;Not Applicable 431;805 Not Applicable;Not Applicable 0;0 Scheduled Follow-up Submission;Scheduled Follow-up Submission Complete Remission/Response;Complete Remission/Response Unknown;Unknown NO;NO 9;9 Not Available;Not Available NO;NO Post-Adjuvant Therapy;Post-Adjuvant Therapy TUMOR FREE;TUMOR FREE YES;YES Unknown;Complete Remission/Response Not Available;Not Available YES;YES Alive;Alive 2013;2014 FEMALE Lung Adenocarcinoma- Not Otherwise Specified (NOS) No C34.3 8140/3 C34.3 YES Unknown NO Not Available Not Available Unknown 2 Not Available Not Available Not Available Not Available Not Available Not Available Not Available Not Available Not Available Unknown Not Available No A4P8 Unknown Unknown 78 89 Unknown 74 84 Complete Remission/Response YES WHITE Unknown NA Primary Tumor Field TCGA-86-A4P8-R48298 09736DC5-00BA-4D11-9400-13848477B168 Not Available 11 186 145 Complete Response 9 28 50.4 NO External Not Applicable Not Available Not Available Gy 2013 Not Evaluated Not Applicable Not Applicable Not Applicable Not Applicable Not Applicable Not Applicable Not Applicable Not Applicable Not Applicable Stage IIIA Not Applicable Not Applicable Not Applicable 7th Not Applicable Not Applicable Not Applicable MX N2 T1b Not Available YES NO 86 1 Lung Alive 2013 2012 Not Available

761 Alive T2 N1 M0 Stage IIB NA 66 R-Upper Not Applicable TCGA-05-4432 e737f650-b72d-44e7-b750-558a56716803 22 -24350 Not Applicable 0 761 Not Available Lung Adenocarcinoma Not Available NA TCGA-05-4432-D36425;TCGA-05-4432-D36426 50774761-63F6-4356-AFF1-6CB33BC143F8;7CDBF312-289A-49AD-989C-52221C8B04E8 Not Available;Not Available 5;5 61;61 31;31 Cisplatin;Vinorelbine Complete Response;Complete Response 12;12 Not Available;Not Available Not Available;Not Available Not Available;Not Available Not Available;Not Available Not Applicable;Not Applicable Not Available;Not Available Not Available;Not Available NO;NO Chemotherapy;Chemotherapy Not Available;Not Available Not Available;Not Available Not Available;Not Available NO;NO 2012;2012 Not Available Not Available Not Available Not Available Not Available Not Available Not Available Not Available Not Available NA Not Available Not Available Not Available Not Available TCGA-05-4432-F36424 2D609D44-E554-4B5E-AB4C-FA32A56E80D9 30 Not Applicable Not Applicable Not Applicable 761 Not Applicable Not Available Scheduled Follow-up Submission Complete Remission/Response Not Available NO 10 Not Available NO Not Available TUMOR FREE YES Complete Remission/Response Not Available NO Alive 2012 MALE Lung Adenocarcinoma Mixed Subtype No C34.1 8255/3 C34.1 YES Not Available Not Available Not Available Not Available Not Available 7 Not Available Not Available Not Available Not Available Not Available Not Available Not Available Not Available Not Available Not Available 58 No 4432 Not Available TUMOR FREE Not Available Not Available Not Available Not Available Not Available Not Available Not Available Not Available Not Available NA R0 Not Applicable Not Applicable Not Applicable Not Applicable Not Applicable Not Applicable Not Applicable Not Applicable Not Applicable Stage IIB Not Applicable Not Applicable Not Applicable 6th Not Applicable Not Applicable Not Applicable M0 N1 T2 Not Available NO YES 05 2 Lung Alive 2010 2008 Not Available

702 Dead T1a N0 M0 Stage IA NA 56 R-Upper Not Applicable TCGA-55-8089 1070ff27-17c9-43dd-a4f6-daa643eb9123 17 -20576 Not Applicable 0 100 Not Available Lung Adenocarcinoma 90 NA 0 Not Available YES Not Available Not Available Not Available YES Not Available NOT HISPANIC OR LATINO NA Not Available Not Available Not Available Not Available TCGA-55-8089-F59075 C8B09023-1E19-4E25-83B5-FB60003D08F0 30 Not Applicable Not Applicable 702 Not Available Not Applicable Not Evaluated Scheduled Follow-up Submission Complete Remission/Response Not Evaluated NO 4 Not Available NO Not Evaluated TUMOR FREE NO Complete Remission/Response Not Available NO Dead 2014 MALE Lung Adenocarcinoma- Not Otherwise Specified (NOS) No C34.1 8140/3 C34.1 YES 100 YES NO Not Available Peripheral Lung 6 Not Available Not Available Not Available Not Available Not Available Not Available Not Available Not Available Not Available NO 97 No 8089 Preoperative TUMOR FREE Not Available Not Available Unknown 85 70 Unknown YES WHITE Unknown NA R0 Not Applicable Not Applicable Not Applicable Not Applicable Not Applicable Not Applicable Not Applicable Not Applicable Not Applicable Stage IA Not Applicable Not Applicable Not Applicable 7th Not Applicable Not Applicable Not Applicable M0 N0 T1a Not Available YES NO 55 2 Lung Alive 2012 2011 1973

210 Dead T2 N0 M0 Stage IB NA 60 L-Upper Not Applicable TCGA-71-8520 37884d39-64e8-4242-b6bd-4c0a1daa21cd 15 -21921 Not Applicable 0 3 Not Available Lung Adenocarcinoma Not Available NA Not Available Not Available Not Available Not Available Not Available Not Available Not Available Not Available NOT HISPANIC OR LATINO NA NO NO NO Not Available TCGA-71-8520-F70925 31390530-007C-4160-9E08-4A980480B334 10 Not Available Not Available 210 Not Available 179 Not Available Scheduled Follow-up Submission Progressive Disease Not Available NO 3 Locoregional Recurrence YES Not Available WITH TUMOR NO Complete Remission/Response Not Available YES Dead 2015 FEMALE Lung Adenocarcinoma- Not Otherwise Specified (NOS) No C34.1 8140/3 C34.1 YES 40 YES NO Not Available Not Available 4 Not Available Not Available Not Available Not Available Not Available Not Available Not Available Not Available Not Available NO Not Available No 8520 Preoperative TUMOR FREE Not Available Not Available NO Not Available Not Available Complete Remission/Response NO ASIAN NO NA Regional site TCGA-71-8520-R70926 E036E361-397E-4FDB-A92D-039E27A04A17 Not Available 10 80 51 Radiographic Progressive Disease 3 22 44 NO External Not Applicable Not Available Not Available Gy 2015 R0 Not Applicable Not Applicable Not Applicable Not Applicable Not Applicable Not Applicable Not Applicable Not Applicable Not Applicable Stage IB Not Applicable Not Applicable Not Applicable 7th Not Applicable Not Applicable Not Applicable M0 N0 T2 Not Available YES NO 71 1 Lung Alive 2013 2011 Not Available

1728 Alive T1a N0 M0 Stage IA NA 58 R-Lower Not Applicable TCGA-64-1676 fb6b289c-aaf9-4d8e-b6e0-5d35bce1b6e1 15 -21428 Not Applicable 0 1728 Not Available Lung Adenocarcinoma Not Available NA Not Available Not Available Not Available Not Available Not Available Not Available Not Available Not Available NOT HISPANIC OR LATINO NA Not Available Not Available Not Available Not Available TCGA-64-1676-F4366 d70bd353-950d-4da4-b04a-934497c1974a 15 Not Applicable Not Applicable Not Applicable 1728 Not Applicable Not Available Not Available Complete Remission/Response Not Available Not Available 11 Not Available NO Not Available TUMOR FREE NO Complete Remission/Response Not Available NO Alive 2010 MALE Lung Adenocarcinoma- Not Otherwise Specified (NOS) No C34.3 8140/3 C34.3 YES Not Available Not Available Not Available Not Available Central Lung 11 Not Available Not Available Not Available Not Available Not Available Not Available Not Available Not Available Not Available Not Available 60 No 1676 Not Available TUMOR FREE Not Available Not Available Not Available Not Available Not Available Not Available Not Available WHITE Not Available NA R0 Not Applicable Not Applicable Not Applicable Not Applicable Not Applicable Not Applicable Not Applicable Not Applicable Not Applicable Stage IA Not Applicable Not Applicable Not Applicable Not Available Not Applicable Not Applicable Not Applicable M0 N0 T1a Not Available NO YES 64 2 Lung Alive 2010 2005 Not Available

427 Alive T2 N0 M0 Stage IB NA 84 L-Lower Not Applicable TCGA-67-3773 009be09b-f9f6-43b7-8f45-4a648f8123ce 21 -30706 Not Applicable 0 427 Not Available Lung Adenocarcinoma Not Available NA Not Available Not Available NO Not Available Not Available Not Available NO Not Available NOT HISPANIC OR LATINO NA FEMALE Lung Adenocarcinoma- Not Otherwise Specified (NOS) No C34.3 8140/3 C34.3 YES Not Available NO Not Available Not Available Central Lung 1 Not Available Not Available Not Available Not Available Not Available Not Available Not Available Not Available Not Available Not Available Not Available No 3773 Not Available TUMOR FREE Not Available Not Available Not Available Not Available Not Available Not Available Not Available WHITE Not Available NA R0 Not Applicable Not Applicable Not Applicable Not Applicable Not Applicable Not Applicable Not Applicable Not Applicable Not Applicable Stage IB Not Applicable Not Applicable Not Applicable 6th Not Applicable Not Applicable Not Applicable M0 N0 T2 Not Available YES NO 67 3 Lung Alive 2011 2009 Not Available

468 Dead T3 N1 M0 Stage IIIA NA 63 R-Lower Not Applicable TCGA-97-8176 12c27db5-db2f-48dc-a2ca-9557b951f43e 5 -23170 Not Applicable 0 252 Not Available Lung Adenocarcinoma 61 NA TCGA-97-8176-D35660;TCGA-97-8176-D35661;TCGA-97-8176-D35662;TCGA-97-8176-D49551;TCGA-97-8176-D49550 3CDB750D-9A1C-4C74-B647-D988440014AE;C2AF0586-F52E-443A-9120-934B54628579;49597D40-3617-493C-8061-8DCCB9871B6B;51434296-C639-41F5-82B1-E61B82E05160;6B643397-9B35-4517-A6EC-013C81A73723 Not Available;Not Available;Not Available;Not Available;Not Available 5;5;5;3;3 161;417;417;417;417 77;77;77;77;77 CARBOplatin;ALIMTA;B12;Neulasta;Xgeva Partial Response;Partial Response;Partial Response;Unknown;Unknown 10;10;10;10;10 Not Available;Not Available;Not Available;Not Available;Not Available Not Available;Not Available;Not Available;Not Available;Not Available Not Available;Not Available;Not Available;Not Available;Not Available Not Available;Not Available;Not Available;Not Available;Not Available Not Applicable;Not Applicable;Not Applicable;Not Applicable;Not Applicable Not Available;Not Available;Not Available;Not Available;Not Available Not Available;Not Available;Not Available;Not Available;Not Available NO;NO;NO;NO;NO Chemotherapy;Chemotherapy;Ancillary;Ancillary;Ancillary Not Available;Not Available;Not Available;Not Available;Not Available Not Available;Not Available;Not Available;Not Available;Not Available Not Available;Not Available;Not Available;Not Available;Not Available NO;NO;NO;NO;NO 2012;2012;2012;2013;2013 0 Not Available YES Not Available Not Available Not Available NO Not Available NOT HISPANIC OR LATINO NA Not Available Not Available Not Available Not Available TCGA-97-8176-F49542 F462990E-8C16-40DE-BCD8-D98F16F60DA6 11 Not Applicable Not Applicable 468 Not Available Not Applicable Unknown Scheduled Follow-up Submission Progressive Disease Not Available NO 10 Not Available NO Unknown Unknown YES Stable Disease Not Available YES Dead 2013 MALE Lung Acinar Adenocarcinoma No C34.3 8550/3 C34.3 YES Not Available YES YES G12S Not Available 10 YES YES 39 Not Available Not Available YES 171 Distant Metastasis Biopsy with Histologic Confirmation YES 40 No 8176 Preoperative TUMOR FREE 68 104 YES 67 100 Unknown YES WHITE YES NA Distant Recurrence;Distant Recurrence;Distant Recurrence TCGA-97-8176-R35601;TCGA-97-8176-R35659;TCGA-97-8176-R49544 B10D270F-44DE-43D1-A82E-FB7D967C274B;411DC29A-8C31-4E66-8FF5-0A8675FA8495;B9986975-6FF6-4AC9-B988-2EA72265F180 Not Available;Not Available;Not Available 5;5;11 105;105;64 91;91;51 Partial Response;Partial Response;Partial Response 10;10;3 5;10;5 1500;3000;3000 NO;NO;NO External;External;External Not Applicable;Not Applicable;Not Applicable Not Available;Not Available;Not Available Not Available;Not Available;Not Available cGy;cGy;cGy 2012;2012;2014 R0 Not Applicable Not Applicable Not Applicable Not Applicable Not Applicable Not Applicable Not Applicable Not Applicable Not Applicable Stage IIIA Not Applicable Not Applicable Not Applicable 7th Not Applicable Not Applicable Not Applicable M0 N1 T3 2011 YES NO 97 4 Lung Alive 2012 2012 1971

1305 Alive T2 N0 M0 Stage IB NA 60 R-Upper Not Applicable TCGA-64-5778 8fdbd88f-19ae-4ab8-bcd7-6bfa1bbde742 2 -21927 Not Applicable 0 926 Not Available Lung Adenocarcinoma Not Available NA TCGA-64-5778-D31472 fc448027-ade7-47c9-8323-907f35fc2702 Not Available 3 Not Available 1174 Tarceva Clinical Progressive Disease 5 Not Available Not Available Not Available Not Available Not Applicable Not Available Not Available YES Chemotherapy Not Available Not Available Not Available NO 2012 1 Not Available Not Available Not Available Not Available Not Available Not Available Not Available NOT HISPANIC OR LATINO NA Not Available;NO;YES Not Available;YES;NO Not Available;Not Available;NO Not Available;YES;NO TCGA-64-5778-F11415;TCGA-64-5778-F31469;TCGA-64-5778-F31471 842094a7-5a39-4da3-827b-ee53549be95d;f7a90387-29d8-4956-93db-8347c2b623a8;cca86d2f-f557-4d7d-8128-137f7a6a6c3a 2;3;3 Not Applicable;Not Available;Not Available Not Applicable;975;Not Available Not Applicable;Not Applicable;Not Applicable 926;1305;1305 Not Applicable;962;1168 Not Available;1;1 Not Available;Additional New Tumor Event;Additional New Tumor Event Complete Remission/Response;Progressive Disease;Progressive Disease Not Available;Unknown;Unknown Not Available;NO;NO 5;5;5 Not Available;Distant Metastasis;Not Available NO;YES;YES Not Available;Unknown;Unknown TUMOR FREE;WITH TUMOR;WITH TUMOR NO;NO;NO Complete Remission/Response;Complete Remission/Response;Complete Remission/Response Not Available;Not Available;Not Available NO;NO;NO Alive;Alive;Alive 2011;2012;2012 MALE Lung Bronchioloalveolar Carcinoma Nonmucinous No C34.1 8252/3 C34.1 YES Discrepancy Not Available Not Available Not Available Central Lung 5 Not Available Not Available Not Available Not Available Not Available Not Available Not Available Not Available Not Available Not Available 68 No 5778 Other TUMOR FREE Not Available Not Available Not Available Not Available Not Available Not Available YES WHITE Not Available NA Distant site TCGA-64-5778-R31470 a16a4831-91fc-43b0-83ac-f24a068b3b7c 1 16 1022 994 Not Available 4 20 Not Available NO EXTERNAL BEAM Not Applicable RECURRENCE Not Available cGy 2012 R0 Not Applicable Not Applicable Not Applicable Not Applicable Not Applicable Not Applicable Not Applicable Not Applicable Not Applicable Stage IB Not Applicable Not Applicable Not Applicable 6th Not Applicable Not Applicable Not Applicable M0 N0 T2 2000 NO YES 64 4 Lung Alive 2011 2008 1966

307 Dead T2 N2 M0 Stage IIIA NA 71 L-Lower Not Applicable TCGA-MP-A4TD 383D1253-349F-43B3-BEBD-9F1D1CF14B3D 8 -25983 307 0 Not Available Not Available Lung Adenocarcinoma 61 NA TCGA-MP-A4TD-D41400;TCGA-MP-A4TD-D41401 151B0D62-C2EE-442B-8FC7-DDCE9131ABBD;429B9434-707A-41B3-A4B6-07DA60A412A3 Not Available;Not Available 18;18 165;165 75;75 Cisplatin;Gemcitabine Complete Response;Complete Response 3;3 Not Available;Not Available Not Available;Not Available Not Available;Not Available Not Available;Not Available Not Applicable;Not Applicable Not Available;Not Available Not Available;Not Available NO;NO Chemotherapy;Chemotherapy Not Available;Not Available Not Available;Not Available Not Available;Not Available NO;NO 2013;2013 0 Not Available NO Not Available Not Available Not Available NO Not Available Unknown NA MALE Lung Adenocarcinoma- Not Otherwise Specified (NOS) No C34.3 8140/3 C34.3 YES Not Evaluated NO Not Available Not Available Peripheral Lung 5 YES Unknown 226 NO Not Available Not Available Not Available Locoregional Recurrence Convincing Imaging YES 20 Yes, History of Prior Malignancy A4TD Post-Adjuvant Therapy Unknown Not Available 92 YES Not Available 93 Complete Remission/Response YES WHITE NO NA RX Not Applicable Not Applicable Not Applicable Not Applicable Not Applicable Not Applicable Not Applicable Not Applicable Not Applicable Stage IIIA Not Applicable Not Applicable Not Applicable 6th Not Applicable Not Applicable Not Applicable M0 N2 T2 1990 NO YES MP 3 Lung Dead 2013 2008 Not Available

336 Dead T1 N2 M0 Stage IIIA NA 49 R-Upper Not Applicable TCGA-50-6593 e10568fe-0436-43f2-9f0f-48f9903868c4 25 -18152 336 0 Not Available Not Available Lung Adenocarcinoma Not Available NA Not Available Not Available YES Not Available Not Available Not Available YES Not Available NOT HISPANIC OR LATINO NA NO NO Not Available NO TCGA-50-6593-F43840 BB824133-F8BB-4916-968D-86355FA69BA7 13 Not Available Not Available 336 Not Available 266 Not Available Scheduled Follow-up Submission Progressive Disease Not Available NO 6 Distant Metastasis YES Not Available WITH TUMOR NO Progressive Disease Convincing Imaging YES Dead 2013 FEMALE Lung Adenocarcinoma Mixed Subtype No C34.1 8255/3 C34.1 YES Not Available YES NO Not Available Not Available 8 Not Available Not Available Not Available Not Available Not Available Not Available Not Available Not Available Not Available Not Available Not Available No 6593 Not Available WITH TUMOR Not Available Not Available Not Available Not Available Not Available Not Available NO WHITE Not Available NA Primary Tumor Field TCGA-50-6593-R44122 CAA51D9B-D706-4474-8194-EC7E82AFBF1E Not Available 13 210 173 Radiographic Progressive Disease 6 27 5400 NO External Not Applicable Not Available Not Available cGy 2013 R0 Not Applicable Not Applicable Not Applicable Not Applicable Not Applicable Not Applicable Not Applicable Not Applicable Not Applicable Stage IIIA Not Applicable Not Applicable Not Applicable 6th Not Applicable Not Applicable Not Applicable M0 N2 T1 Not Available NO YES 50 4 Lung Dead 2011 2008 Not Available

1870 Alive T1 N0 MX Stage IA NA 58 L-Lower Not Applicable TCGA-97-7553 37c8d73a-45ae-40fc-ba9a-721b755c1160 17 -21518 Not Applicable 0 1332 Not Available Lung Adenocarcinoma 64 NA Not Available Not Available Not Available Not Available Not Available Not Available Not Available Not Available NOT HISPANIC OR LATINO NA Not Available Not Available Not Available Not Available TCGA-97-7553-F31865 BA07C466-8C62-4560-BB7C-07344BAF0FA8 14 Not Applicable Not Applicable Not Applicable 1870 Not Applicable Not Evaluated Scheduled Follow-up Submission Not Available Not Evaluated NO 5 Not Available NO Not Evaluated TUMOR FREE NO Complete Remission/Response Not Available NO Alive 2012 FEMALE Lung Bronchioloalveolar Carcinoma Nonmucinous No C34.3 8252/3 C34.3 YES Not Available Not Available Not Available Not Available Central Lung 11 Not Available Not Available Not Available Not Available Not Available Not Available Not Available Not Available Not Available Not Available 20 No 7553 Not Available TUMOR FREE 70 99 Not Available 70 99 Not Available YES WHITE Not Available NA Not Available Not Applicable Not Applicable Not Applicable Not Applicable Not Applicable Not Applicable Not Applicable Not Applicable Not Applicable Stage IA Not Applicable Not Applicable Not Applicable 6th Not Applicable Not Applicable Not Applicable MX N0 T1 Not Available NO YES 97 2 Lung Alive 2011 2007 1967

1400 Alive T2 N0 MX Stage IB NA 59 L-Lower Not Applicable TCGA-55-6971 8d0736fe-261c-445c-bfd2-a3ea3ceaf367 28 -21734 Not Applicable 0 25 Not Available Lung Adenocarcinoma Not Available NA Not Available Not Available NO Not Available Not Available Not Available NO Not Available Not Available NA Not Available Not Available Not Available Not Available TCGA-55-6971-F56190 12A8DE46-0514-47CB-A544-77296F337007 29 Not Applicable Not Applicable Not Applicable 1400 Not Applicable Unknown Scheduled Follow-up Submission Complete Remission/Response Unknown NO 1 Not Available NO Not Available TUMOR FREE NO Complete Remission/Response Not Available NO Alive 2014 FEMALE Lung Adenocarcinoma- Not Otherwise Specified (NOS) No C34.3 8140/3 C34.3 YES Not Available NO Not Available Not Available Not Available 7 Not Available Not Available Not Available Not Available Not Available Not Available Not Available Not Available Not Available Not Available 60 No 6971 Not Available TUMOR FREE Not Available Not Available Not Available Not Available Not Available Not Available Not Available WHITE Not Available NA R0 Not Applicable Not Applicable Not Applicable Not Applicable Not Applicable Not Applicable Not Applicable Not Applicable Not Applicable Stage IB Not Applicable Not Applicable Not Applicable 7th Not Applicable Not Applicable Not Applicable MX N0 T2 2010 NO YES 55 4 Lung Alive 2011 2010 1970

944 Alive T2 N1 M0 Stage IIB NA 63 L-Lower Not Applicable TCGA-86-8278 a816e355-be6f-4d16-b051-b6d86ed8cded 6 -23117 Not Applicable 0 23 Not Available Lung Adenocarcinoma Not Available NA TCGA-86-8278-D41670;TCGA-86-8278-D41671;TCGA-86-8278-D41672 964E5BFB-3A3B-47C8-A346-6F5BDACA4D99;9ACDE909-44BF-475C-AAFC-8C721F24CFD9;8C3C7ABD-D1E8-4672-965B-91AF4B8F03CE Not Available;Not Available;Not Available 25;25;25 156;156;Not Available 29;29;178 Etoposide;Cisplatin;Gefitinib Stable Disease;Stable Disease;Not Applicable 3;3;3 Not Available;Not Available;Not Available Not Available;Not Available;Not Available Not Available;Not Available;Not Available Not Available;Not Available;Not Available Not Applicable;Not Applicable;Not Applicable Not Available;Not Available;Not Available Not Available;Not Available;Not Available NO;NO;YES Chemotherapy;Chemotherapy;Targeted Molecular therapy Not Available;Not Available;Not Available Not Available;Not Available;Not Available Not Available;Not Available;Not Available NO;NO;NO 2013;2013;2013 2 Not Available Not Available Not Available Not Available Not Available Not Available Not Available NOT HISPANIC OR LATINO NA YES;Not Available NO;Not Available NO;Not Available NO;Not Available TCGA-86-8278-F41668;TCGA-86-8278-F59732 AC1B771F-E726-4EE8-99BB-16A15628DF26;7676E1AE-1FFF-4CF0-B2AF-D97D745A06F7 25;20 Not Available;Not Applicable Not Available;Not Applicable Not Applicable;Not Applicable 476;944 29;Not Applicable 1;1 Scheduled Follow-up Submission;Scheduled Follow-up Submission Stable Disease;Stable Disease 90;90 NO;NO 3;5 Not Available;Not Available YES;NO Preoperative;Preoperative WITH TUMOR;WITH TUMOR NO;NO Complete Remission/Response;Complete Remission/Response Not Available;Not Available NO;NO Alive;Alive 2013;2014 FEMALE Lung Adenocarcinoma- Not Otherwise Specified (NOS) No C34.30 8480/3 C34.3 YES 80 NO Not Available Not Available Peripheral Lung 6 Not Available Not Available Not Available Not Available Not Available Not Available Not Available Not Available Not Available NO Not Available No 8278 Not Available TUMOR FREE Not Available Not Available Not Available Not Available Not Available Complete Remission/Response Not Available WHITE Not Available NA R0 Not Applicable Not Applicable Not Applicable Not Applicable Not Applicable Not Applicable Not Applicable Not Applicable Not Applicable Stage IIB Not Applicable Not Applicable Not Applicable 7th Not Applicable Not Applicable Not Applicable M0 N1 T2 Not Available YES NO 86 1 Lung Alive 2012 2011 Not Available

1040 Alive T2b N0 M0 Stage IIA NA 50 L-Lower Not Applicable TCGA-55-7910 b2bc53d6-ef78-4ae9-a6b5-1c7cad9a43eb 10 -18289 Not Applicable 0 203 Not Available Lung Adenocarcinoma 44 NA TCGA-55-7910-D66141 76D8449C-257D-474B-A830-EDE15DAE2DDD Not Available 3 Not Available 253 Cisplatin Complete Response 10 Not Available Not Available Not Available Not Available Not Applicable Not Available Not Available NO Chemotherapy Not Available Not Available Not Available NO 2014 1 Not Available Not Available Not Available Not Available Not Available Not Available Not Available NOT HISPANIC OR LATINO NA NO YES Unknown Unknown TCGA-55-7910-F66140 50A3A722-AEC7-4FBC-AFDA-7B45FEA1263A 3 Not Available Not Available Not Applicable 1040 1018 Not Available Scheduled Follow-up Submission Not Applicable Not Available NO 10 Not Available YES Not Available WITH TUMOR YES Complete Remission/Response Not Available NO Alive 2014 FEMALE Lung Adenocarcinoma- Not Otherwise Specified (NOS) No C34.3 8140/3 C34.3 YES 80 Not Available Not Available Not Available Peripheral Lung 3 Not Available Not Available Not Available Not Available Not Available Not Available Not Available Not Available Not Available Not Available 15 No 7910 Preoperative WITH TUMOR 91 91 Not Available 97 86 Not Available YES BLACK OR AFRICAN AMERICAN Not Available NA Unknown TCGA-55-7910-R66142 ACCCBE41-BDFD-4BDF-9192-6EF3399B09D5 Not Available 3 Not Available 1040 Not Applicable 10 Not Available Not Available YES External Not Applicable Not Available Not Available Not Available 2014 R0 Not Applicable Not Applicable Not Applicable Not Applicable Not Applicable Not Applicable Not Applicable Not Applicable Not Applicable Stage IIA Not Applicable Not Applicable Not Applicable 7th Not Applicable Not Applicable Not Applicable M0 N0 T2b 2011 YES NO 55 4 Lung Alive 2012 2011 1996

1288 Dead T2 N0 M0 Stage IB NA 72 R-Upper Not Applicable TCGA-50-6590 114a34c0-983b-4d90-9181-1823c9637a63 25 -26444 1288 0 Not Available Not Available Lung Adenocarcinoma Not Available NA TCGA-50-6590-D43830;TCGA-50-6590-D43831 A075B030-D2CD-4488-9FD9-D0BFD2A087A7;A3CB9C93-4673-49EE-AB53-9823D496F039 Not Available;Not Available 4;4 189;189 92;92 Paclitaxel;Carboplatin Complete Response;Complete Response 6;6 Not Available;Not Available Not Available;Not Available Not Available;Not Available Not Available;Not Available Not Applicable;Not Applicable Not Available;Not Available Not Available;Not Available NO;NO Chemotherapy;Chemotherapy Not Available;Not Available Not Available;Not Available Not Available;Not Available NO;NO 2013;2013 Not Available Not Available YES Not Available Not Available Not Available Not Available Not Available NOT HISPANIC OR LATINO NA Not Available Not Available Not Available Not Available TCGA-50-6590-F43827 47ED4445-67EF-44BD-AC55-2C3E733F62CF 4 Not Applicable Not Applicable 1288 Not Available Not Applicable Not Available Scheduled Follow-up Submission Complete Remission/Response Not Available NO 6 Not Available NO Not Available TUMOR FREE YES Complete Remission/Response Not Available NO Dead 2013 FEMALE Lung Adenocarcinoma- Not Otherwise Specified (NOS) No C34.1 8140/3 C34.1 YES Not Available Not Available Not Available Not Available Not Available 8 Not Available Not Available Not Available Not Available Not Available Not Available Not Available Not Available Not Available Not Available 50 No 6590 Not Available TUMOR FREE Not Available Not Available Not Available Not Available Not Available Not Available Not Available WHITE Not Available NA Not Available Not Applicable Not Applicable Not Applicable Not Applicable Not Applicable Not Applicable Not Applicable Not Applicable Not Applicable Stage IB Not Applicable Not Applicable Not Applicable 6th Not Applicable Not Applicable Not Applicable M0 N0 T2 2002 NO YES 50 4 Lung Dead 2011 2005 Not Available

99 Dead T2b N0 MX Stage IIA NA 78 R-Upper Not Applicable TCGA-55-A490 B89DE053-D253-447F-952E-9A2EDCF6BCA5 21 -28728 Not Applicable 0 48 Not Available Lung Adenocarcinoma 40 NA TCGA-55-A490-D59079;TCGA-55-A490-D59080 A6D28342-BD3F-4814-B710-BFBA6B19D0B3;2A35CBA5-76D7-4EAC-AF33-941BD871E623 Not Available;Not Available 30;30 89;89 89;89 Carboplatin;Alimta Unknown;Unknown 4;4 Not Available;Not Available Not Available;Not Available Not Available;Not Available Not Available;Not Available Not Applicable;Not Applicable Not Available;Not Available Not Available;Not Available NO;NO Chemotherapy;Chemotherapy Not Available;Not Available Not Available;Not Available Not Available;Not Available NO;NO 2014;2014 1 Not Available YES Not Available Not Available FISH YES Not Available NOT HISPANIC OR LATINO NA Not Available Not Available Not Available Not Available TCGA-55-A490-F59076 813389EA-D9E4-47C0-B21A-5FA647E9EEB8 30 Not Applicable Not Applicable 99 Not Available Not Applicable Not Evaluated Scheduled Follow-up Submission Not Applicable Not Evaluated NO 4 Not Available NO Not Available TUMOR FREE YES Not Applicable Not Available NO Dead 2014 MALE Lung Adenocarcinoma- Not Otherwise Specified (NOS) No C34.1 8140/3 C34.1 YES 80 YES YES G12C Peripheral Lung 2 Not Available Not Available Not Available Not Available Not Available Not Available Not Available Not Available Not Available NO 34 No A490 Preoperative TUMOR FREE Not Available Not Available Unknown 60 56 Unknown YES WHITE Unknown NA R0 Not Applicable Not Applicable Not Applicable Not Applicable Not Applicable Not Applicable Not Applicable Not Applicable Not Applicable Stage IIA Not Applicable Not Applicable Not Applicable 7th Not Applicable Not Applicable Not Applicable MX N0 T2b 1985 YES NO 55 3 Lung Alive 2013 2012 1951

976 Dead T4 N1 M1 Stage IV NA 62 Not Available Not Applicable TCGA-78-7156 7b0622ab-63ea-483f-ae40-d3ea587bdbba 29 -22720 976 0 Not Available Not Available Lung Adenocarcinoma Not Available NA 1 Not Available NO Not Available Not Available Not Available NO Not Available Not Available NA Not Available Not Available Not Available Not Available TCGA-78-7156-F16982 03cedf57-e893-47ff-a0d8-d67a938ec858 29 Not Available Not Available 976 Not Available Not Available Not Available Scheduled Follow-up Submission Not Available Not Available Not Available 9 Not Available Not Available Not Available Not Available NO Not Available Not Available NO Dead 2011 MALE Lung Adenocarcinoma Mixed Subtype No C34.9 8255/3 C34.9 YES Not Available NO Not Available Not Available Peripheral Lung 9 Not Available Not Available Not Available Not Available Not Available Not Available Not Available Not Available Not Available Not Available 84 No 7156 Preoperative Not Available Not Available Not Available Not Available Not Available Not Available Not Available Not Available WHITE Not Available NA R0 Not Applicable Not Applicable Not Applicable Not Applicable Not Applicable Not Applicable Not Applicable Not Applicable Not Applicable Stage IV Not Applicable Not Applicable Not Applicable 6th Not Applicable Not Applicable Not Applicable M1 N1 T4 1989 NO YES 78 3 Lung Dead 2011 2006 1961

4992 Alive T1 N0 MX Stage IA NA 68 L-Upper Not Applicable TCGA-49-AARR 9B93A7C5-F9B4-4461-8078-0201A725C9CF 19 -24942 Not Applicable 0 4630 Not Available Lung Adenocarcinoma 103.5 NA 1 Not Available Not Available Not Available Not Available Not Available Not Available Not Available NOT HISPANIC OR LATINO NA NO NO NO Not Available TCGA-49-AARR-F70574 A41DDC21-E254-43A4-BBC7-FE6D974EB739 25 Not Available Not Available Not Applicable 4992 4812 1 Scheduled Follow-up Submission Stable Disease 80 NO 2 Locoregional Recurrence YES Preoperative WITH TUMOR NO Complete Remission/Response Convincing Imaging NO Alive 2015 MALE Lung Adenocarcinoma- Not Otherwise Specified (NOS) No C34.1 8140/3 C34.1 YES 90 Not Available Not Available Not Available Not Available 6 NO NO 1099 Not Available Not Available Not Available 1099 New Primary Tumor Biopsy with Histologic Confirmation;Convincing Imaging YES Not Available No AARR Preoperative TUMOR FREE 76.3 78.8 NO 77.8 80.3 Complete Remission/Response YES BLACK OR AFRICAN AMERICAN NO NA R0 Not Applicable Not Applicable Not Applicable Not Applicable Not Applicable Not Applicable Not Applicable Not Applicable Not Applicable Stage IA Not Applicable Not Applicable Not Applicable 5th Not Applicable Not Applicable Not Applicable MX N0 T1 1987 NO YES 49 3 Lung Alive 2014 2001 Not Available

321 Dead T2 N1 M0 Stage IIB NA 69 R-Lower Not Applicable TCGA-78-8660 781f40c9-c099-4c96-8269-ebe2a449c93d 23 -25422 321 0 Not Available Not Available Lung Adenocarcinoma 87 NA 1 Not Available NO Not Available Not Available Not Available NO Not Available Not Evaluated NA MALE Lung Adenocarcinoma- Not Otherwise Specified (NOS) No C34.3 8140/3 C34.3 YES Not Available NO Not Available Not Available Peripheral Lung 10 NO YES 242 Not Available Not Available YES 260 Distant Metastasis Biopsy with Histologic Confirmation YES 11 Yes, History of Prior Malignancy 8660 Preoperative WITH TUMOR Not Available Not Available NO 72 108 Unknown YES Not Evaluated YES NA Primary Tumor Field TCGA-78-8660-R36074 4B964C80-FF7E-42DD-93F3-6AA45D3B94EA Not Available 23 97 Not Available Radiographic Progressive Disease 10 Not Available Not Available NO External Not Applicable Not Available Not Available Not Available 2012 R0 Not Applicable Not Applicable Not Applicable Not Applicable Not Applicable Not Applicable Not Applicable Not Applicable Not Applicable Stage IIB Not Applicable Not Applicable Not Applicable 6th Not Applicable Not Applicable Not Applicable M0 N1 T2 2005 NO YES 78 4 Lung Dead 2012 2005 1954

630 Alive T2b N0 M0 Stage IIA NA 69 L-Lower Not Applicable TCGA-55-A48Y 12C07138-E3FA-418A-89E3-628DA12BC99E 21 -25384 Not Applicable 0 42 Not Available Lung Adenocarcinoma 42 NA TCGA-55-A48Y-D59059;TCGA-55-A48Y-D59060 47E0007B-5A41-4E22-8E2B-2483E168CA99;A497B509-E9BB-4278-851A-F25CB653F363 Not Available;Not Available 30;30 163;163 91;91 Carboplatin;Alimta Complete Response;Complete Response 4;4 Not Available;Not Available Not Available;Not Available Not Available;Not Available Not Available;Not Available Not Applicable;Not Applicable Not Available;Not Available Not Available;Not Available NO;NO Chemotherapy;Chemotherapy Not Available;Not Available Not Available;Not Available Not Available;Not Available NO;NO 2014;2014 Not Evaluated Not Available Unknown Not Available Not Available Not Available Unknown Not Available NOT HISPANIC OR LATINO NA Not Available Not Available Not Available Not Available TCGA-55-A48Y-F59057 D3AB8E13-5211-4F85-B576-F476B974C2D1 30 Not Applicable Not Applicable Not Applicable 630 Not Applicable Unknown Scheduled Follow-up Submission Complete Remission/Response Unknown NO 4 Not Available NO Not Available TUMOR FREE YES Complete Remission/Response Not Available NO Alive 2014 MALE Lung Adenocarcinoma- Not Otherwise Specified (NOS) No C34.3 8140/3 C34.3 YES Not Evaluated Unknown Not Available Not Available Unknown 2 Not Available Not Available Not Available Not Available Not Available Not Available Not Available Not Available Not Available NO 50 No A48Y Not Available TUMOR FREE Not Available Not Available Unknown Not Available 38 Unknown YES WHITE Unknown NA R0 Not Applicable Not Applicable Not Applicable Not Applicable Not Applicable Not Applicable Not Applicable Not Applicable Not Applicable Stage IIA Not Applicable Not Applicable Not Applicable 7th Not Applicable Not Applicable Not Applicable M0 N0 T2b Not Available YES NO 55 2 Lung Alive 2013 2012 1962

1499 Dead T2 N1 MX Stage IIB NA 59 R-Lower Not Applicable TCGA-50-5068 591c068f-bbb1-4df2-9abb-d1a2e4a58372 2 -21728 1499 0 1499 Not Available Lung Adenocarcinoma Not Available NA TCGA-50-5068-D32058 416e251e-06ba-4e59-b9b0-22db5fac92e1 Not Available 16 140 99 Taxol Clinical Progressive Disease 5 Not Available Not Available Not Available Not Available Not Applicable Not Available Not Available NO Chemotherapy Not Available Not Available Not Available NO 2012 Not Available Not Available Not Available Not Available Not Available Not Available Not Available Not Available NOT HISPANIC OR LATINO NA YES NO YES NO TCGA-50-5068-F32057 271667fa-b39c-433f-a7d7-f65d87b30992 16 686 Not Available 1499 Not Available 686 Not Available Scheduled Follow-up Submission Progressive Disease Not Available NO 5 Distant Metastasis YES Not Available WITH TUMOR YES Progressive Disease Biopsy with Histologic Confirmation;Convincing Imaging NO Dead 2012 FEMALE Lung Adenocarcinoma- Not Otherwise Specified (NOS) No C34.3 8140/3 C34.3 YES Not Available Not Available Not Available Not Available Not Available 7 Not Available Not Available Not Available Not Available Not Available Not Available Not Available Not Available Not Available Not Available Not Available No 5068 Not Available WITH TUMOR Not Available Not Available Not Available Not Available Not Available Not Available Not Available WHITE Not Available NA Not Available Not Applicable Not Applicable Not Applicable Not Applicable Not Applicable Not Applicable Not Applicable Not Applicable Not Applicable Stage IIB Not Applicable Not Applicable Not Applicable 6th Not Applicable Not Applicable Not Applicable MX N1 T2 Not Available NO YES 50 Not Available Lung Dead 2011 2003 Not Available

1246 Alive T2b N1 M0 Stage IIB NA 64 L-Upper Not Applicable TCGA-62-A471 0075437E-BA1A-46BE-86D6-9773209A2B5E 30 -23689 Not Applicable 0 883 Not Available Lung Adenocarcinoma Not Available NA TCGA-62-A471-D36369;TCGA-62-A471-D40576;TCGA-62-A471-D40577 0B09C8A2-1EDD-4523-9B04-7C6DCA69D4CE;AB370E23-6CB3-4C2D-AAEE-F18B9832C078;8E2B3458-E311-41FF-BA45-78E93803E232 Not Available;Not Available;Not Available 30;25;25 115;115;115 41;41;41 Cisplatin;Vinorelbine;Gemcitabine Complete Response;Complete Response;Complete Response 10;2;2 Not Available;Not Available;Not Available Not Available;Not Available;Not Available Not Available;Not Available;Not Available Not Available;Not Available;Not Available Not Applicable;Not Applicable;Not Applicable Not Available;Not Available;Not Available Not Available;Not Available;Not Available NO;NO;NO Chemotherapy;Chemotherapy;Chemotherapy Not Available;Not Available;Not Available Not Available;Not Available;Not Available Not Available;Not Available;Not Available NO;NO;NO 2012;2013;2013 0 Not Available NO Not Available Not Available Not Available NO Not Available NOT HISPANIC OR LATINO NA Not Available Not Available Not Available Not Available TCGA-62-A471-F51439 9BA3092F-235D-4C9D-943F-BD40B1D24721 19 Not Applicable Not Applicable Not Applicable 1246 Not Applicable 0 Scheduled Follow-up Submission Complete Remission/Response 100 NO 11 Not Available NO Preoperative TUMOR FREE YES Complete Remission/Response Not Available NO Alive 2013 MALE Lung Adenocarcinoma- Not Otherwise Specified (NOS) No C34.1 8140/3 C34.1 YES 100 NO Not Available Not Available Peripheral Lung 10 Not Available Not Available Not Available Not Available Not Available Not Available Not Available Not Available Not Available NO 30 No A471 Preoperative TUMOR FREE Not Available Not Available YES 75 104 Complete Remission/Response YES WHITE NO NA R0 Not Applicable Not Applicable Not Applicable Not Applicable Not Applicable Not Applicable Not Applicable Not Applicable Not Applicable Stage IIB Not Applicable Not Applicable Not Applicable 7th Not Applicable Not Applicable Not Applicable M0 N1 T2b 2006 NO YES 62 4 Lung Alive 2012 2010 Not Available

1079 Alive T1a N0 MX Stage IA NA 77 L-Upper Not Applicable TCGA-J2-A4AE E5F956DD-F49F-435C-83C3-5B1B0A2050ED 27 -28163 Not Applicable 0 282 Not Available Lung Adenocarcinoma 89 NA Not Available Not Available NO Not Available Not Available Not Available NO Not Available NOT HISPANIC OR LATINO NA Not Available;Not Available Not Available;Not Available Not Available;Not Available Not Available;Not Available TCGA-J2-A4AE-F55522;TCGA-J2-A4AE-F70639 3A67D1E9-45FD-41D0-8E79-F27D9B40AE3A;F091573D-78D1-4263-B744-6928E374086D 8;27 Not Applicable;Not Applicable Not Applicable;Not Applicable Not Applicable;Not Applicable 671;1079 Not Applicable;Not Applicable Not Available;Not Available Scheduled Follow-up Submission;Scheduled Follow-up Submission Complete Remission/Response;Complete Remission/Response Not Available;Not Available NO;NO 1;2 Not Available;Not Available NO;NO Not Available;Not Available TUMOR FREE;TUMOR FREE NO;NO Complete Remission/Response;Complete Remission/Response Not Available;Not Available NO;NO Alive;Alive 2014;2015 FEMALE Lung Adenocarcinoma- Not Otherwise Specified (NOS) No C34.1 8140/3 C34.1 YES Not Available NO Not Available Not Available Not Available 12 Not Available Not Available Not Available Not Available Not Available Not Available Not Available Not Available Not Available NO Not Available No A4AE Not Available TUMOR FREE Not Available Not Available NO 0.72 92 Not Applicable YES WHITE NO NA R0 Not Applicable Not Applicable Not Applicable Not Applicable Not Applicable Not Applicable Not Applicable Not Applicable Not Applicable Stage IA Not Applicable Not Applicable Not Applicable 7th Not Applicable Not Applicable Not Applicable MX N0 T1a Not Available YES NO J2 1 Lung Alive 2012 2012 Not Available

995 Dead T2a N0 M0 Stage IB NA 64 R-Upper Not Applicable TCGA-55-7574 a7911bdc-13f1-434a-bfa3-f8ba55d15846 11 -23703 Not Applicable 0 95 Not Available Lung Adenocarcinoma Not Available NA TCGA-55-7574-D20394;TCGA-55-7574-D20393;TCGA-55-7574-D64281;TCGA-55-7574-D64282 50bc6792-9c2d-4274-950b-9916578c4a38;fac718aa-4cd8-4b69-a353-5c3619f96f9a;4FE5B8E6-4EEE-41ED-986D-BD7841309AFC;855D0B62-EF5B-4164-B03B-74957166BF47 Not Available;Not Available;Not Available;Not Available 11;11;28;28 184;184;631;631 130;130;478;478 Carboplatin;Docetaxel;Topotecan;Avastin Not Available;Not Available;Partial Response;Partial Response 1;1;8;8 5;5;Not Available;Not Available 160;30;Not Available;Not Available mg;mg;Not Available;Not Available ADJUVANT;ADJUVANT;Not Available;Not Available Not Applicable;Not Applicable;Not Applicable;Not Applicable 1;1;Not Available;Not Available IV;IV;Not Available;Not Available NO;NO;NO;NO Chemotherapy;Chemotherapy;Chemotherapy;Chemotherapy Not Available;Not Available;Not Available;Not Available Not Available;Not Available;Not Available;Not Available Not Available;Not Available;Not Available;Not Available Not Available;Not Available;NO;NO 2012;2012;2014;2014 Not Available Not Available Not Available Not Available Not Available Not Available Not Available Not Available NOT HISPANIC OR LATINO NA YES YES NO NO TCGA-55-7574-F64279 5044B8DF-923E-4D53-9106-5A864756F25C 28 Not Available Not Available 995 Not Available 478 3 Scheduled Follow-up Submission Progressive Disease 50 NO 8 Distant Metastasis YES Post-Adjuvant Therapy WITH TUMOR YES Complete Remission/Response Convincing Imaging YES Dead 2014 FEMALE Lung Adenocarcinoma- Not Otherwise Specified (NOS) No C34.1 8140/3 C34.1 YES Not Available NO Not Available Not Available Not Available 1 Not Available Not Available Not Available Not Available Not Available Not Available Not Available Not Available Not Available Not Available 2 No 7574 Not Available WITH TUMOR Not Available Not Available Not Available Not Available Not Available Not Available Not Available WHITE Not Available NA Primary Tumor Field;Distant Recurrence TCGA-55-7574-R20395;TCGA-55-7574-R64280 17dcc733-d220-4557-aa17-6c2fe68d4dd6;9DB59113-7988-4908-A908-B0A73E2F7D33 1;Not Available 11;28 162;487 123;487 Not Available;Stable Disease 1;8 Not Available;Not Available 3800;Not Available NO;NO EXTERNAL BEAM;External Not Applicable;Not Applicable ADJUVANT;Not Available Not Available;Not Available cGy;Not Available 2012;2014 R1 Not Applicable Not Applicable Not Applicable Not Applicable Not Applicable Not Applicable Not Applicable Not Applicable Not Applicable Stage IB Not Applicable Not Applicable Not Applicable 7th Not Applicable Not Applicable Not Applicable M0 N0 T2a 2011 YES NO 55 4 Lung Alive 2012 2011 1971

NA T2 N0 M0 Stage IB NA Not Available R-Upper Not Applicable TCGA-75-7031 4efc2dac-6330-4b7a-a529-a27285f5fc23 16 Not Available Not Applicable Not Available Not Available Not Available Lung Adenocarcinoma Not Available NA 0 Not Available Not Available Not Available Not Available Not Available Not Available Not Available Not Available NA NO NO NO NO TCGA-75-7031-F15880 2281f87d-086d-493d-9bbe-8a41dc1a216f 16 Not Applicable Not Applicable Not Applicable Not Available Not Applicable 0 Not Available Not Available Not Available Not Available 8 Not Available NO Not Available Not Available NO Not Available Not Available NO Alive 2011 FEMALE Lung Adenocarcinoma- Not Otherwise Specified (NOS) No C34.1 8140/3 C34.1 YES Not Available Not Available Not Available Not Available Peripheral Lung 8 Not Available Not Available Not Available Not Available Not Available Not Available Not Available Not Available Not Available Not Available 5 No 7031 Preoperative TUMOR FREE Not Available Not Available Not Available Not Available Not Available Not Available Not Available Not Available Not Available NA R0 Not Applicable Not Applicable Not Applicable Not Applicable Not Applicable Not Applicable Not Applicable Not Applicable Not Applicable Stage IB Not Applicable Not Applicable Not Applicable 6th Not Applicable Not Applicable Not Applicable M0 N0 T2 1975 NO YES 75 3 Lung Alive 2011 2008 1965

761 Alive T2 N0 M0 Stage IB NA 59 L-Lower Not Applicable TCGA-05-4430 3a23cdb5-2327-45ac-b0b5-d4afe038c757 22 -21884 Not Applicable 0 761 Not Available Lung Adenocarcinoma Not Available NA Not Available Not Available Not Available Not Available Not Available Not Available Not Available Not Available Not Available NA Not Available Not Available Not Available Not Available TCGA-05-4430-F36427 A35C227E-F4EF-4535-9765-FD2DAD9B0C06 30 Not Applicable Not Applicable Not Applicable 761 Not Applicable Not Available Scheduled Follow-up Submission Complete Remission/Response Not Available NO 10 Not Available NO Not Available TUMOR FREE Unknown Not Applicable Not Available Unknown Alive 2012 FEMALE Lung Adenocarcinoma Mixed Subtype No C34.3 8255/3 C34.3 YES Not Available Not Available Not Available Not Available Not Available 7 Not Available Not Available Not Available Not Available Not Available Not Available Not Available Not Available Not Available Not Available 80 No 4430 Not Available TUMOR FREE Not Available Not Available Not Available Not Available Not Available Not Available Not Available Not Available Not Available NA R0 Not Applicable Not Applicable Not Applicable Not Applicable Not Applicable Not Applicable Not Applicable Not Applicable Not Applicable Stage IB Not Applicable Not Applicable Not Applicable 6th Not Applicable Not Applicable Not Applicable M0 N0 T2 Not Available NO YES 05 4 Lung Alive 2010 2008 Not Available

476 Alive T1a N0 MX Stage IA NA 60 R-Upper Not Applicable TCGA-55-8097 afe83af5-af22-49c0-88b4-f1d0dfcbe238 15 -22260 Not Applicable 0 15 Not Available Lung Adenocarcinoma 74 NA Not Available Not Available NO Not Available Not Available Not Available NO Not Available NOT HISPANIC OR LATINO NA Not Available Not Available Not Available Not Available TCGA-55-8097-F47821 CD4B0E92-54B8-474F-AAD7-FB4A9C9CE4E3 28 Not Applicable Not Applicable Not Applicable 476 Not Applicable Not Evaluated Scheduled Follow-up Submission Complete Remission/Response Not Evaluated NO 8 Not Available NO Not Available TUMOR FREE NO Complete Remission/Response Not Available NO Alive 2013 FEMALE Lung Adenocarcinoma- Not Otherwise Specified (NOS) No C34.1 8140/3 C34.1 YES Not Available NO Not Available Not Available Unknown 6 Not Available Not Available Not Available Not Available Not Available Not Available Not Available Not Available Not Available NO 35 No 8097 Not Available TUMOR FREE Not Available 87 Unknown Not Available 79 Complete Remission/Response YES WHITE Unknown NA R0 Not Applicable Not Applicable Not Applicable Not Applicable Not Applicable Not Applicable Not Applicable Not Applicable Not Applicable Stage IA Not Applicable Not Applicable Not Applicable 7th Not Applicable Not Applicable Not Applicable MX N0 T1a 2008 YES NO 55 4 Lung Alive 2012 2012 1973

2616 Alive T1 N0 MX Stage IA NA 60 R-Upper Not Applicable TCGA-44-6776 c2a1de2e-6451-4c95-8ce6-263f2b7e6eff 25 -22150 Not Applicable 0 1938 Not Available Lung Adenocarcinoma 80 NA Not Available Not Available Not Available Not Available Not Available Not Available Not Available Not Available Not Available NA Not Available;Not Available Not Available;Not Available Not Available;NO Not Available;Not Available TCGA-44-6776-F15756;TCGA-44-6776-F39378 452c565f-f54c-4dcb-b781-4c6940df5e67;323859A8-8025-4125-B8C2-A96C40DB24D1 31;21 Not Applicable;Not Applicable Not Applicable;Not Applicable Not Applicable;Not Applicable 1938;2616 Not Applicable;Not Applicable Not Available;Not Available Scheduled Follow-up Submission;Scheduled Follow-up Submission Complete Remission/Response;Complete Remission/Response Not Available;Not Available Not Available;NO 8;1 Not Available;Not Available NO;NO Not Available;Not Available TUMOR FREE;TUMOR FREE NO;NO Complete Remission/Response;Complete Remission/Response Not Available;Not Available NO;NO Alive;Alive 2011;2013 FEMALE Lung Adenocarcinoma- Not Otherwise Specified (NOS) No C34.1 8140/3 C34.1 YES Not Available Not Available Not Available Not Available Not Available 8 Not Available Not Available Not Available Not Available Not Available Not Available Not Available Not Available Not Available Not Available 45 Yes 6776 Not Available TUMOR FREE 85 82 Not Available 95 74 Not Available YES WHITE Not Available NA Not Available Not Applicable Not Applicable Not Applicable Not Applicable Not Applicable Not Applicable Not Applicable Not Applicable Not Applicable Stage IA Not Applicable Not Applicable Not Applicable 6th Not Applicable Not Applicable Not Applicable MX N0 T1 2005 NO YES 44 4 Lung Alive 2011 2005 1960

653 Alive T2a N0 M0 Stage IB NA 44 R-Upper Not Applicable TCGA-86-8358 467d96d7-4a42-4116-8c32-0e4b34f96d8a 6 -16409 Not Applicable 0 19 Not Available Lung Adenocarcinoma Not Available NA 2 Not Available Not Available Not Available Not Available Not Available Not Available Not Available NOT HISPANIC OR LATINO NA Not Available Not Available Not Available Not Available TCGA-86-8358-F58410 AE9316FF-560B-4692-AC3C-90AC5D7895B2 9 Not Applicable Not Applicable Not Applicable 653 Not Applicable 0 Scheduled Follow-up Submission Complete Remission/Response 100 NO 4 Not Available NO Other TUMOR FREE NO Complete Remission/Response Not Available NO Alive 2014 MALE Lung Adenocarcinoma- Not Otherwise Specified (NOS) No C34.1 8140/3 C34.1 YES 90 NO Not Available Not Available Peripheral Lung 7 Not Available Not Available Not Available Not Available Not Available Not Available Not Available Not Available Not Available NO 20 No 8358 Preoperative TUMOR FREE Not Available Not Available Unknown Not Available Not Available Unknown Not Available WHITE Unknown NA R0 Not Applicable Not Applicable Not Applicable Not Applicable Not Applicable Not Applicable Not Applicable Not Applicable Not Applicable Stage IB Not Applicable Not Applicable Not Applicable 7th Not Applicable Not Applicable Not Applicable M0 N0 T2a Not Available YES NO 86 2 Lung Alive 2012 2012 1992

3094 Alive T2 N0 M0 Stage IA NA 70 R-Upper Not Applicable TCGA-50-5049 9944f46f-9fa8-4e2c-a806-776bec7f8803 25 -25577 Not Applicable 0 1485 Not Available Lung Adenocarcinoma Not Available NA Not Available Not Available YES Not Available Not Available Not Available Not Available Not Available NOT HISPANIC OR LATINO NA NO;NO NO;YES Not Available;NO NO;Not Available TCGA-50-5049-F32017;TCGA-50-5049-F70448 79588753-1945-43ea-8245-803ff0d345a2;90D123CB-CB20-432C-B1EB-DE4AC04FBEB9 15;16 1520;Not Available Not Available;Not Available Not Applicable;Not Applicable 2146;3094 1568;2544 Not Available;Not Available Scheduled Follow-up Submission;Scheduled Follow-up Submission Stable Disease;Unknown Not Available;Not Available NO;NO 5;2 Locoregional Recurrence;Locoregional Recurrence YES;YES Not Available;Not Available WITH TUMOR;WITH TUMOR NO;NO Progressive Disease;Progressive Disease Biopsy with Histologic Confirmation;Convincing Imaging;Convincing Imaging YES;YES Alive;Alive 2012;2015 MALE Lung Adenocarcinoma- Not Otherwise Specified (NOS) No C34.1 8140/3 C34.1 YES Not Available YES NO Not Available Not Available 8 Not Available Not Available Not Available Not Available Not Available Not Available Not Available Not Available Not Available Not Available Not Available No 5049 Not Available TUMOR FREE Not Available Not Available Not Available Not Available Not Available Not Available NO WHITE Not Available NA Primary Tumor Field;Local Recurrence;Local Recurrence TCGA-50-5049-R32018;TCGA-50-5049-R40451;TCGA-50-5049-R70449 f905863f-864f-47e2-b71c-97e4751409f6;5A078CE3-58C7-4D83-90B5-53A805868ACD;6A997CE9-D4FE-4BB9-BB53-165C43494E55 01;Not Available;Not Available 15;20;16 0;1572;2544 0;1572;2544 Not Available;Partial Response;Unknown 5;2;2 Not Available;4;Not Available 10;12;Not Available NO;NO;NO IMPLANTS;External;External Not Applicable;Not Applicable;Not Applicable ADJUVANT;Not Available;Not Available Not Available;Not Available;Not Available Not Available;Gy;Not Available 2012;2013;2015 Not Available Not Applicable Not Applicable Not Applicable Not Applicable Not Applicable Not Applicable Not Applicable Not Applicable Not Applicable Stage IA Not Applicable Not Applicable Not Applicable 6th Not Applicable Not Applicable Not Applicable M0 N0 T2 Not Available NO YES 50 Not Available Lung Alive 2011 2006 Not Available

652 Alive T1b N0 MX Stage IA NA 72 L-Lower Not Applicable TCGA-55-7726 923b37e4-fcf7-46a0-8cb2-9139711fec7b 8 -26451 Not Applicable 0 39 Not Available Lung Adenocarcinoma 72 NA Not Available Not Available Not Available Not Available Not Available Not Available Not Available Not Available NOT HISPANIC OR LATINO NA Not Available Not Available Not Available Not Available TCGA-55-7726-F47844 3F1E5E80-6F83-41FA-8999-94F152C1EDCB 29 Not Applicable Not Applicable Not Applicable 652 Not Applicable Not Evaluated Scheduled Follow-up Submission Complete Remission/Response Not Evaluated NO 8 Not Available NO Not Evaluated TUMOR FREE NO Complete Remission/Response Not Available NO Alive 2013 FEMALE Lung Adenocarcinoma- Not Otherwise Specified (NOS) No C34.3 8140/3 C34.3 YES Not Available Not Available Not Available Not Available Not Available 3 Not Available Not Available Not Available Not Available Not Available Not Available Not Available Not Available Not Available Not Available 30 Yes 7726 Not Available TUMOR FREE 59 61 Not Available 55 49 Not Available YES WHITE Not Available NA R0 Not Applicable Not Applicable Not Applicable Not Applicable Not Applicable Not Applicable Not Applicable Not Applicable Not Applicable Stage IA Not Applicable Not Applicable Not Applicable 7th Not Applicable Not Applicable Not Applicable MX N0 T1b 1979 YES NO 55 3 Lung Alive 2012 2011 1955

993 Alive T1 N0 M0 Stage IA NA 42 R-Upper Not Applicable TCGA-86-8076 8609edfc-119d-4d63-9188-c86aabd5ca52 9 -15605 Not Applicable 0 26 Not Available Lung Adenocarcinoma Not Available NA TCGA-86-8076-D41684;TCGA-86-8076-D41685 50F44FAB-0ED8-4107-BFA7-F1D90752FECF;6DF79C11-EF79-4AF7-90DA-26FB54BB5161 Not Available;Not Available 25;25 193;193 40;40 Etoposide;Cisplatin Complete Response;Complete Response 3;3 Not Available;Not Available Not Available;Not Available Not Available;Not Available Not Available;Not Available Not Applicable;Not Applicable Not Available;Not Available Not Available;Not Available NO;NO Chemotherapy;Chemotherapy Not Available;Not Available Not Available;Not Available Not Available;Not Available NO;NO 2013;2013 2 Not Available NO Not Available Not Available Not Available NO Not Available NOT HISPANIC OR LATINO NA Not Available;Not Available Not Available;Not Available Not Available;Not Available Not Available;Not Available TCGA-86-8076-F41683;TCGA-86-8076-F63522 A93C981E-4BCD-444A-889C-3F75CF9D1642;F06BE3BF-4A95-4C31-A00E-C6DA89CC89B6 25;12 Not Applicable;Not Applicable Not Applicable;Not Applicable Not Applicable;Not Applicable 489;993 Not Applicable;Not Applicable 2;0 Scheduled Follow-up Submission;Scheduled Follow-up Submission Complete Remission/Response;Complete Remission/Response 80;100 NO;NO 3;8 Not Available;Not Available NO;NO Preoperative;Post-Adjuvant Therapy TUMOR FREE;TUMOR FREE YES;YES Complete Remission/Response;Complete Remission/Response Not Available;Not Available NO;NO Alive;Alive 2013;2014 MALE Lung Papillary Adenocarcinoma No C34.1 8260/3 C34.1 YES 80 NO Not Available Not Available Peripheral Lung 5 Not Available Not Available Not Available Not Available Not Available Not Available Not Available Not Available Not Available Unknown Not Available No 8076 Preoperative TUMOR FREE Not Available Not Available Unknown Not Available Not Available Unknown NO WHITE Unknown NA R0 Not Applicable Not Applicable Not Applicable Not Applicable Not Applicable Not Applicable Not Applicable Not Applicable Not Applicable Stage IA Not Applicable Not Applicable Not Applicable 7th Not Applicable Not Applicable Not Applicable M0 N0 T1 Not Available YES NO 86 1 Lung Alive 2012 2011 Not Available

476 Alive T2a N0 MX Stage IB NA 64 L-Upper Not Applicable TCGA-L9-A8F4 927FC5E0-2C14-4C20-A49B-F10D8A1175C3 14 -23617 Not Applicable 0 102 Not Available Lung Adenocarcinoma 35 NA 0 Not Available NO Not Available Not Available Not Available NO Not Available NOT HISPANIC OR LATINO NA Not Available Not Available Not Available Not Available TCGA-L9-A8F4-F64972 230AAB9B-F943-4A2F-BB3D-A975974BAD7D 16 Not Applicable Not Applicable Not Applicable 476 Not Applicable 0 Scheduled Follow-up Submission Not Applicable Not Available NO 9 Not Available NO Preoperative Unknown NO Not Applicable Not Available YES Alive 2014 FEMALE Lung Adenocarcinoma- Not Otherwise Specified (NOS) No C34.1 8140/3 C34.1 YES Not Available NO Not Available Not Available Unknown 2 Not Available Not Available Not Available Not Available Not Available Not Available Not Available Not Available Not Available NO 14 No A8F4 Preoperative TUMOR FREE 48 54 NO 45 53 Not Applicable YES BLACK OR AFRICAN AMERICAN YES NA Primary Tumor Field TCGA-L9-A8F4-R56626 2B7AD134-01C1-4492-A078-579A3A274609 Not Available 14 Not Available 64 Not Applicable 2 Not Available 82 YES Internal Not Applicable Not Available Not Available Gy 2014 R0 Not Applicable Not Applicable Not Applicable Not Applicable Not Applicable Not Applicable Not Applicable Not Applicable Not Applicable Stage IB Not Applicable Not Applicable Not Applicable 7th Not Applicable Not Applicable Not Applicable MX N0 T2a Not Available YES NO L9 2 Lung Alive 2014 2013 1973

761 Dead T2 N0 M0 Stage IB NA FPPP TCGA 51 R-Upper Not Applicable TCGA-44-2668 bab43415-d413-40be-a4c0-2c40a52afe6a 22 -18856 Not Applicable 0 246 Not Available Lung Adenocarcinoma 53 NA 0 Not Available Not Available Not Available Not Available Not Available Not Available Not Available NOT HISPANIC OR LATINO NA Not Available;Not Available Not Available;YES Not Available;YES Not Available;NO TCGA-44-2668-F5041;TCGA-44-2668-F18688 5510cceb-3418-48f7-870a-01d65ebd2f55;878f1377-c807-4832-887e-3dcc134fdbae 22;22 Not Applicable;453 Not Applicable;Not Available Not Applicable;761 246;Not Available Not Applicable;433 0;Not Available Not Available;Additional New Tumor Event Complete Remission/Response;Progressive Disease Not Available;Not Available Not Available;Not Available 10;11 Not Available;Not Available NO;YES Not Available;Not Available TUMOR FREE;WITH TUMOR NO;Not Available Complete Remission/Response;Not Available Not Available;Not Available NO;Not Available Alive;Dead 2010;2011 MALE Lung Adenocarcinoma- Not Otherwise Specified (NOS) No C34.1 8140/3 C34.1 YES Not Available NO Not Available Not Available Not Available 10 Not Available Not Available Not Available Not Available Not Available Not Available Not Available Not Available Not Available Not Available 60 No 2668 Other TUMOR FREE Not Available Not Available Not Available 104 82 Not Available YES WHITE Not Available NA Distant Recurrence;Distant Recurrence;Distant Recurrence;Distant Recurrence TCGA-44-2668-R18707;TCGA-44-2668-R18708;TCGA-44-2668-R18690;TCGA-44-2668-R18706 0b7f3a19-ed59-4ad2-bb47-860f1d7c29ed;ba5b2712-d4ea-49ef-afe9-e835ad30d748;023f6abf-6688-4f41-8699-acaf19e4dff2;b807d917-e8d2-44fb-b1a4-9fe135310bc3 2;2;1;2 22;22;22;22 587;587;488;589 586;587;468;586 Not Available;Not Available;Not Available;Not Available 11;11;11;11 2;1;14;3 1800;1800;3500;2100 NO;NO;NO;NO OTHER;OTHER;EXTERNAL BEAM;OTHER Cyberknife;Cyberknife;Not Applicable;Cyberknife PALLIATIVE;PALLIATIVE;PALLIATIVE;PALLIATIVE Not Available;Not Available;Not Available;Not Available cGy;cGy;cGy;cGy 2011;2011;2011;2011 R0 Not Applicable Not Applicable Not Applicable Not Applicable Not Applicable Not Applicable Not Applicable Not Applicable Not Applicable Stage IB Not Applicable Not Applicable Not Applicable 6th Not Applicable Not Applicable Not Applicable M0 N0 T2 2009 YES NO 44 4 Lung Alive 2010 2009 1979

340 Dead T3 N0 M0 Stage IIB NA 41 R-Upper Not Applicable TCGA-73-A9RS 2A394996-809F-459F-A647-D209921CCBD2 17 -15162 Not Applicable 0 305 Not Available Lung Adenocarcinoma Not Available NA TCGA-73-A9RS-D60824;TCGA-73-A9RS-D60826;TCGA-73-A9RS-D60827;TCGA-73-A9RS-D60828;TCGA-73-A9RS-D60829;TCGA-73-A9RS-D60830 F9D3AD0B-B57B-4BC4-BE99-581990D65A30;0462262C-D185-4D68-B111-FE7D35239FFC;F252506A-D42C-4B51-8A33-83867E2282B5;91902CAB-804C-475D-855B-44440709C0BE;F29AAA5F-2DEF-42E3-AAB4-39BC2259681E;BC782E72-1B7C-451F-A7DD-FF17F0EA035A Not Available;Not Available;Not Available;Not Available;Not Available;Not Available 17;17;17;17;17;17 186;186;186;242;284;Not Available 130;130;130;200;242;284 carboplatin;paclitaxel;bevacizumab;pemetrexed;docetaxel;gemcitabine Clinical Progressive Disease;Clinical Progressive Disease;Clinical Progressive Disease;Clinical Progressive Disease;Clinical Progressive Disease;Not Applicable 6;6;6;6;6;6 Not Available;Not Available;Not Available;Not Available;Not Available;Not Available Not Available;Not Available;Not Available;Not Available;Not Available;Not Available Not Available;Not Available;Not Available;Not Available;Not Available;Not Available Not Available;Not Available;Not Available;Not Available;Not Available;Not Available Not Applicable;Not Applicable;Not Applicable;Not Applicable;Not Applicable;Not Applicable Not Available;Not Available;Not Available;Not Available;Not Available;Not Available Not Available;Not Available;Not Available;Not Available;Not Available;Not Available NO;NO;NO;NO;NO;YES Chemotherapy;Chemotherapy;Immunotherapy;Chemotherapy;Chemotherapy;Chemotherapy Not Available;Not Available;Not Available;Not Available;Not Available;Not Available Not Available;Not Available;Not Available;Not Available;Not Available;Not Available Not Available;Not Available;Not Available;Not Available;Not Available;Not Available NO;NO;YES;NO;NO;NO 2014;2014;2014;2014;2014;2014 1 Not Available YES Not Available Not Available FISH YES Not Available NOT HISPANIC OR LATINO NA Not Available Not Available Not Available Not Available TCGA-73-A9RS-F64487 3D9025D6-1190-49DC-986A-FC52C19BBA9B 3 Not Applicable Not Applicable 340 Not Available Not Applicable 1 Scheduled Follow-up Submission Progressive Disease 80 NO 9 Not Available NO Preoperative WITH TUMOR NO Progressive Disease Not Available YES Dead 2014 MALE Lung Adenocarcinoma- Not Otherwise Specified (NOS) No C34.1 8480/3 C34.1 YES 80 YES NO Not Available Peripheral Lung 6 YES NO 107 NO Not Available Not Available Not Available Locoregional Recurrence Convincing Imaging YES 11 No A9RS Preoperative WITH TUMOR 81.9 60.2 NO 81.5 59.6 Progressive Disease YES BLACK OR AFRICAN AMERICAN YES NA Primary Tumor Field TCGA-73-A9RS-R60823 F991F8D3-049C-4842-AAFE-A155907BCBBB Not Available 17 53 53 Radiographic Progressive Disease 6 1 12 NO Internal Not Applicable Not Available Not Available Gy 2014 R0 Not Applicable Not Applicable Not Applicable Not Applicable Not Applicable Not Applicable Not Applicable Not Applicable Not Applicable Stage IIB Not Applicable Not Applicable Not Applicable 7th Not Applicable Not Applicable Not Applicable M0 N0 T3 Not Available NO YES 73 2 Lung Alive 2014 2013 1990

578 Alive TX NX M1b Stage IV NA 64 R-Lower Not Applicable TCGA-93-A4JP 30F276A8-3A4B-4DE8-AEA0-7F605D100401 27 -23620 Not Applicable 0 269 Not Available Lung Adenocarcinoma Not Available NA TCGA-93-A4JP-D65104 6D848A43-59A3-46FE-9574-9F37B0059EC8 Not Available 18 101 17 Carboplatin Partial Response 9 Not Available Not Available Not Available Not Available Not Applicable Not Available Not Available NO Chemotherapy Not Available Not Available Not Available NO 2014 0 Not Available YES L861Q Not Available Not Available NO Not Available NOT HISPANIC OR LATINO NA YES YES Not Available NO TCGA-93-A4JP-F56970 ED886C70-F6A0-43DD-8B8D-D9182B3D1DC2 28 Not Available Not Available Not Applicable 578 497 1 Scheduled Follow-up Submission Progressive Disease Unknown NO 2 Distant Metastasis YES Post-Adjuvant Therapy WITH TUMOR YES Partial Remission/Response Convincing Imaging NO Alive 2014 MALE Lung Papillary Adenocarcinoma No C34.3 8260/3 C34.3 YES Not Available NO Not Available Not Available Unknown 2 Not Available Not Available Not Available Not Available Not Available Not Available Not Available Not Available Not Available NO Not Available No A4JP Preoperative WITH TUMOR Not Available Not Available YES Not Available Not Available Partial Remission/Response NO ASIAN NO NA RX Not Applicable Not Applicable Not Applicable Not Applicable Not Applicable Not Applicable Not Applicable Not Applicable Not Applicable Stage IV Not Applicable Not Applicable Not Applicable 7th Not Applicable Not Applicable Not Applicable M1b NX TX Not Available YES NO 93 1 Lung Alive 2014 2012 Not Available

949 Dead T2 N0 M0 Stage IB NA 45 L-Upper Not Applicable TCGA-78-7535 46592b7b-6968-42a6-83af-0917c9f4a9a5 19 -16571 949 0 Not Available Not Available Lung Adenocarcinoma Not Available NA TCGA-78-7535-D20641;TCGA-78-7535-D20640 81a1889c-bbcb-408b-8c95-1a0acf920311;b445a5a2-5b5c-47d3-962d-04da28489e68 Not Available;Not Available 19;19 124;130 60;60 Vinblastine;Cisplatin Not Available;Not Available 1;1 3;4 8;160 mg;mg ADJUVANT;ADJUVANT Not Applicable;Not Applicable 1;1 IV;IV NO;NO Chemotherapy;Chemotherapy Not Available;Not Available 56;640 mg;mg Not Available;Not Available 2012;2012 0 Not Available NO Not Available Not Available Not Available NO Not Available Not Available NA NO NO NO YES TCGA-78-7535-F20633 63e1af54-b78a-4f59-9871-30d86f0546f6 19 Not Available 809 949 Not Available 809 Not Available Scheduled Follow-up Submission Progressive Disease Not Available Not Available 1 Not Available YES Not Available WITH TUMOR YES Stable Disease Not Available NO Dead 2012 MALE Lung Adenocarcinoma- Not Otherwise Specified (NOS) No C34.1 8140/3 C34.1 YES Not Available NO Not Available Not Available Not Available 1 Not Available Not Available Not Available Not Available Not Available Not Available Not Available Not Available Not Available Not Available 35 No 7535 Preoperative WITH TUMOR Not Available Not Available Not Available Not Available Not Available Not Available Not Available WHITE Not Available NA R0 Not Applicable Not Applicable Not Applicable Not Applicable Not Applicable Not Applicable Not Applicable Not Applicable Not Applicable Stage IB Not Applicable Not Applicable Not Applicable 6th Not Applicable Not Applicable Not Applicable M0 N0 T2 Not Available NO YES 78 2 Lung Dead 2012 2000 1972

1432 Alive T1b N0 M0 Stage IA NA 69 L-Upper Not Applicable TCGA-NJ-A4YQ 52DF074F-A402-4B78-9472-F8EB268EFDED 27 -25427 Not Applicable 0 886 Not Available Lung Adenocarcinoma Not Available NA Not Available Not Available NO Not Available Not Available Not Available NO Not Available NOT HISPANIC OR LATINO NA Not Available;Not Available Not Available;Not Available Not Available;Not Available Not Available;Not Available TCGA-NJ-A4YQ-F50895;TCGA-NJ-A4YQ-F70646 D75D2FE9-18DE-4D15-B47A-208D923F3550;C88AC622-0782-4420-B5C0-FEFFABE44A85 1;27 Not Applicable;Not Applicable Not Applicable;Not Applicable Not Applicable;Not Applicable 886;1432 Not Applicable;Not Applicable Not Available;Not Available Scheduled Follow-up Submission;Scheduled Follow-up Submission Stable Disease;Stable Disease Not Available;Not Available NO;NO 11;2 Not Available;Not Available NO;NO Not Available;Not Available TUMOR FREE;TUMOR FREE NO;NO Stable Disease;Stable Disease Not Available;Not Available NO;NO Alive;Alive 2013;2015 FEMALE Lung Adenocarcinoma- Not Otherwise Specified (NOS) No C34.1 8255/3 C34.1 YES Not Available NO Not Available Not Available Unknown 9 Not Available Not Available Not Available Not Available Not Available Not Available Not Available Not Available Not Available NO 40 No A4YQ Not Available TUMOR FREE Not Available Not Available NO Not Available Not Available Stable Disease NO WHITE NO NA RX Not Applicable Not Applicable Not Applicable Not Applicable Not Applicable Not Applicable Not Applicable Not Applicable Not Applicable Stage IA Not Applicable Not Applicable Not Applicable 7th Not Applicable Not Applicable Not Applicable M0 N0 T1b 2011 Not Available Not Available NJ 4 Lung Alive 2013 2011 Not Available

626 Dead T2 N1 M0 Stage IIB NA 71 L-Upper Not Applicable TCGA-78-7148 e8060c45-451d-4779-9584-f284db47e63e 4 -25990 626 0 Not Available Not Available Lung Adenocarcinoma Not Available NA 0 Not Available NO Not Available Not Available Not Available NO Not Available Not Available NA Not Available Not Available NO YES TCGA-78-7148-F17170 208bf3ac-10c9-4d63-ad87-332ddb82517d 4 Not Available 182 626 Not Available 182 Not Available Scheduled Follow-up Submission Not Available Not Available Not Available 10 Not Available YES Not Available Not Available NO Not Available Not Available NO Dead 2011 MALE Lung Adenocarcinoma Mixed Subtype No C34.1 8255/3 C34.1 YES Not Available NO Not Available Not Available Peripheral Lung 10 Not Available Not Available Not Available Not Available Not Available Not Available Not Available Not Available Not Available Not Available 57 No 7148 Preoperative Not Available Not Available Not Available Not Available Not Available Not Available Not Available Not Available WHITE Not Available NA R0 Not Applicable Not Applicable Not Applicable Not Applicable Not Applicable Not Applicable Not Applicable Not Applicable Not Applicable Stage IIB Not Applicable Not Applicable Not Applicable 6th Not Applicable Not Applicable Not Applicable M0 N1 T2 Not Available NO YES 78 2 Lung Dead 2011 2001 1944

3261 Alive T2 N0 M0 Stage IB NA 74 R-Lower Not Applicable TCGA-55-6986 028e99e9-5b9a-4954-bb6e-6d4709a3cea8 26 Not Available Not Applicable 0 2151 Not Available Lung Adenocarcinoma Not Available NA Not Available Not Available NO Not Available Not Available Not Available NO Not Available Not Available NA Not Available Not Available Not Available Not Available TCGA-55-6986-F45606 D147F3D1-27F0-467E-9212-AE14BCF08700 17 Not Applicable Not Applicable Not Applicable 3261 Not Applicable Not Evaluated Scheduled Follow-up Submission Complete Remission/Response Not Evaluated NO 7 Not Available NO Not Evaluated TUMOR FREE NO Complete Remission/Response Not Available NO Alive 2013 FEMALE Lung Bronchioloalveolar Carcinoma Nonmucinous No C34.3 8252/3 C34.3 YES Not Available NO Not Available Not Available Not Available 7 Not Available Not Available Not Available Not Available Not Available Not Available Not Available Not Available Not Available Not Available Not Available No 6986 Not Available TUMOR FREE Not Available Not Available Not Available Not Available Not Available Not Available Not Available WHITE Not Available NA Not Available Not Applicable Not Applicable Not Applicable Not Applicable Not Applicable Not Applicable Not Applicable Not Applicable Not Applicable Stage IB Not Applicable Not Applicable Not Applicable 6th Not Applicable Not Applicable Not Applicable M0 N0 T2 Not Available NO YES 55 1 Lung Alive 2011 2004 Not Available

739 Alive T2a N1 MX Stage IIA NA 65 L-Upper Not Applicable TCGA-J2-8192 369f14c4-2191-4962-a309-3e23ddc4e5fc 6 -23892 Not Applicable 0 154 Not Available Lung Adenocarcinoma 112 NA TCGA-J2-8192-D32572;TCGA-J2-8192-D32574;TCGA-J2-8192-D55392 5FA2C5DE-EEB3-450E-8D40-F0B739F6106D;5751CCC2-7B5B-4703-8405-CF1E474220FB;A136AC0B-F93F-4B43-AE17-770DADF20159 Not Available;Not Available;Not Available 6;6;16 Not Available;Not Available;Not Available 105;105;641 cisplatin;pemetrexed;Erlotinib Not Available;Not Available;Not Available 6;6;4 Not Available;Not Available;Not Available Not Available;Not Available;Not Available Not Available;Not Available;Not Available Not Available;Not Available;Not Available Not Applicable;Not Applicable;Not Applicable Not Available;Not Available;Not Available Not Available;Not Available;Not Available YES;YES;YES Chemotherapy;Chemotherapy;Chemotherapy Not Available;Not Available;Not Available Not Available;Not Available;Not Available Not Available;Not Available;Not Available NO;NO;NO 2012;2012;2014 Not Available Not Available YES Exon 19 Deletion Not Available Not Available NO Not Available NOT HISPANIC OR LATINO NA YES NO NO NO TCGA-J2-8192-F55390 EFDC3166-C726-484E-8EB1-5A436DF66A0A 18 Not Available Not Available Not Applicable 739 482 Not Evaluated Scheduled Follow-up Submission Unknown Not Evaluated NO 2 Locoregional Recurrence YES Not Available Unknown YES Progressive Disease Biopsy with Histologic Confirmation NO Alive 2014 FEMALE Lung Adenocarcinoma- Not Otherwise Specified (NOS) No C34.1 8140/3 C34.1 YES Not Available YES NO Not Available Not Available 6 Not Available Not Available Not Available Not Available Not Available Not Available Not Available Not Available Not Available NO Not Available Yes, History of Prior Malignancy 8192 Not Available TUMOR FREE 100 89 YES 97 77 Unknown YES WHITE NO NA Not Available Not Applicable Not Applicable Not Applicable Not Applicable Not Applicable Not Applicable Not Applicable Not Applicable Not Applicable Stage IIA Not Applicable Not Applicable Not Applicable 7th Not Applicable Not Applicable Not Applicable MX N1 T2a Not Available YES NO J2 1 Lung Alive 2012 2011 Not Available

3635 Alive T2 N0 M0 Stage IB NA 65 R-Upper Not Applicable TCGA-78-7153 c353f949-5f66-4a2c-b8f0-17be8bf19e0a 29 -23922 Not Applicable 0 760 Not Available Lung Adenocarcinoma Not Available NA 0 Not Available NO Not Available Not Available Not Available NO Not Available Not Available NA Not Available;Not Available Not Available;Not Available Not Available;Not Available Not Available;Not Available TCGA-78-7153-F16980;TCGA-78-7153-F46077 932b04a8-46cd-464a-97f5-682b9dbe77de;C9C20E98-3841-44E5-B29C-946EDFFABBAF 29;23 Not Available;Not Available Not Available;Not Available Not Applicable;Not Applicable 760;3635 Not Available;Not Available Not Available;Unknown Scheduled Follow-up Submission;Scheduled Follow-up Submission Not Available;Unknown Not Available;Not Available Not Available;NO 9;8 Not Available;Not Available Not Available;Unknown Not Available;Unknown Not Available;Unknown NO;NO Not Available;Complete Remission/Response Not Available;Not Available NO;NO Alive;Alive 2011;2013 FEMALE Lung Adenocarcinoma Mixed Subtype No C34.8 8255/3 C34.8 YES Not Available NO Not Available Not Available Peripheral Lung 9 Not Available Not Available Not Available Not Available Not Available Not Available Not Available Not Available Not Available Not Available 20 No 7153 Preoperative Not Available Not Available Not Available Not Available Not Available Not Available Not Available NO WHITE Not Available NA R0 Not Applicable Not Applicable Not Applicable Not Applicable Not Applicable Not Applicable Not Applicable Not Applicable Not Applicable Stage IB Not Applicable Not Applicable Not Applicable 6th Not Applicable Not Applicable Not Applicable M0 N0 T2 1988 NO YES 78 4 Lung Alive 2011 2003 1948

154 Dead T3 N0 MX Stage IIB NA 75 R-Upper Not Applicable TCGA-55-8092 4b92fce2-3772-40eb-ab34-a27937b58590 8 -27597 Not Applicable 0 26 Not Available Lung Adenocarcinoma 19 NA Not Available Not Available Not Available Not Available Not Available Not Available Not Available Not Available NOT HISPANIC OR LATINO NA NO NO NO Not Available TCGA-55-8092-F46643 08F57135-8BDA-492F-9F56-7D5C9BEF9DDE 13 Not Available Not Available 154 Not Available 127 Not Evaluated Scheduled Follow-up Submission Progressive Disease Not Evaluated NO 8 Locoregional Recurrence YES Not Evaluated WITH TUMOR NO Complete Remission/Response Convincing Imaging NO Dead 2013 MALE Lung Adenocarcinoma- Not Otherwise Specified (NOS) No C34.1 8140/3 C34.1 YES Not Available Not Available Not Available Not Available Not Available 7 Not Available Not Available Not Available Not Available Not Available Not Available Not Available Not Available Not Available NO 40 No 8092 Not Available TUMOR FREE 2 19 Unknown 66 68 Complete Remission/Response YES WHITE Unknown NA R0 Not Applicable Not Applicable Not Applicable Not Applicable Not Applicable Not Applicable Not Applicable Not Applicable Not Applicable Stage IIB Not Applicable Not Applicable Not Applicable 7th Not Applicable Not Applicable Not Applicable MX N0 T3 2012 YES NO 55 4 Lung Alive 2012 2012 1972

1293 Dead T1 N0 M1 Stage IV NA 61 R-Lower Not Applicable TCGA-55-6968 83a6ed20-b3cf-48b9-8ed3-a080cd4951fa 28 -22344 1293 0 Not Available Not Available Lung Adenocarcinoma Not Available NA TCGA-55-6968-D56252 FA5390E8-6281-46F2-891D-82EAF29D6654 Not Available 30 Not Available 13 Not Available Stable Disease 1 Not Available Not Available Not Available Not Available Not Applicable Not Available Not Available NO Chemotherapy Not Available Not Available Not Available NO 2014 Not Available Not Available Not Available Not Available Not Available Not Available Not Available Not Available NOT HISPANIC OR LATINO NA Not Available Not Available Not Available Not Available TCGA-55-6968-F56247 78B86F68-8C53-4B9F-8321-02FDEB8CB702 30 Not Applicable Not Applicable 1293 Not Available Not Applicable Unknown Scheduled Follow-up Submission Stable Disease Unknown NO 1 Not Available NO Not Available WITH TUMOR YES Stable Disease Not Available YES Dead 2014 MALE Lung Adenocarcinoma- Not Otherwise Specified (NOS) No C34.3 8140/3 C34.3 YES Not Available Not Available Not Available Not Available Not Available 7 Not Available Not Available Not Available Not Available Not Available Not Available Not Available Not Available Not Available Not Available Not Available No 6968 Not Available WITH TUMOR Not Available Not Available Not Available Not Available Not Available Not Available Not Available WHITE Not Available NA Distant site TCGA-55-6968-R56251 2FDE65C3-7B12-4A5E-9DEF-E8CAFBDAD8D6 Not Available 30 57 13 Stable Disease 1 Not Available Not Available NO External Not Applicable Not Available Not Available Not Available 2014 Not Available Not Applicable Not Applicable Not Applicable Not Applicable Not Applicable Not Applicable Not Applicable Not Applicable Not Applicable Stage IV Not Applicable Not Applicable Not Applicable 6th Not Applicable Not Applicable Not Applicable M1 N0 T1 Not Available NO YES 55 2 Lung Dead 2011 2004 Not Available

607 Dead T1a N1 M1b Stage IV NA 41 R-Lower Not Applicable TCGA-55-8512 202e5df9-69f2-432c-a59d-cc5aa176e973 11 -15266 Not Applicable 0 3 Not Available Lung Adenocarcinoma Not Available NA 1 Not Available Unknown Not Available Not Available Not Available Unknown Not Available NOT HISPANIC OR LATINO NA Not Available Not Available Not Available Not Available TCGA-55-8512-F64286 C86A17B3-ACBB-498F-8D5F-5A2A4EE2A07A 28 Not Applicable Not Applicable 607 Not Available Not Applicable Not Available Scheduled Follow-up Submission Progressive Disease Not Available NO 8 Not Available NO Not Available WITH TUMOR NO Progressive Disease Not Available NO Dead 2014 MALE Lung Bronchioloalveolar Carcinoma Nonmucinous No C34.3 8252/3 C34.3 YES 90 Unknown Not Available Not Available Unknown 1 Not Available Not Available Not Available Not Available Not Available Not Available Not Available Not Available Not Available NO 19 No 8512 Preoperative WITH TUMOR Not Available Not Available Unknown Not Available Not Available Not Applicable NO WHITE Unknown NA R0 Not Applicable Not Applicable Not Applicable Not Applicable Not Applicable Not Applicable Not Applicable Not Applicable Not Applicable Stage IV Not Applicable Not Applicable Not Applicable 7th Not Applicable Not Applicable Not Applicable M1b N1 T1a Not Available YES NO 55 2 Lung Alive 2013 2012 1989

1233 Alive T2 N0 MX Stage IB NA 58 L-Upper Not Applicable TCGA-55-6985 e5cb0c86-8fe2-4cfc-b32b-e8ec3839ffc4 26 -21381 Not Applicable 0 1233 Not Available Lung Adenocarcinoma Not Available NA Not Available Not Available NO Not Available Not Available Not Available NO Not Available Not Available NA Not Available Not Available Not Available Not Available TCGA-55-6985-F37056 F432F92C-4BB0-437C-A742-B9E6B4BD8E55 22 Not Applicable Not Applicable Not Applicable 1233 Not Applicable Not Available Scheduled Follow-up Submission Not Available Not Available YES 2 Not Available NO Not Available TUMOR FREE NO Complete Remission/Response Not Available NO Alive 2013 FEMALE Lung Adenocarcinoma- Not Otherwise Specified (NOS) No C34.1 8140/3 C34.1 YES Not Available NO Not Available Not Available Not Available 7 Not Available Not Available Not Available Not Available Not Available Not Available Not Available Not Available Not Available Not Available 50 No 6985 Not Available TUMOR FREE Not Available Not Available Not Available Not Available Not Available Not Available Not Available WHITE Not Available NA R0 Not Applicable Not Applicable Not Applicable Not Applicable Not Applicable Not Applicable Not Applicable Not Applicable Not Applicable Stage IB Not Applicable Not Applicable Not Applicable 6th Not Applicable Not Applicable Not Applicable MX N0 T2 2004 NO YES 55 4 Lung Alive 2011 2004 Not Available

1474 Alive T2a N2 M0 Stage IIIA NA 55 R-Upper Not Applicable TCGA-50-5941 f3f024c2-52f1-400e-946c-398f5b72e6dc 2 -20350 Not Applicable 0 285 Not Available Lung Adenocarcinoma Not Available NA TCGA-50-5941-D32131;TCGA-50-5941-D32132 c30f3b22-ea7d-4ef7-8735-85a743e37de2;1d4ba45d-7254-42be-a255-644f5af80b46 Not Available;Not Available 17;17 Not Available;Not Available Not Available;Not Available Cisplatin;Docetaxel Complete Response;Complete Response 5;5 Not Available;Not Available Not Available;Not Available Not Available;Not Available Not Available;Not Available Not Applicable;Not Applicable Not Available;Not Available Not Available;Not Available NO;NO Chemotherapy;Chemotherapy Not Available;Not Available Not Available;Not Available Not Available;Not Available NO;NO 2012;2012 Not Available Not Available Not Available Not Available Not Available Not Available Not Available Not Available Not Available NA Not Available;Not Available Not Available;Not Available Not Available;Not Available Not Available;Not Available TCGA-50-5941-F32129;TCGA-50-5941-F70451 f165ea21-c724-4532-8246-fa21fc059d3e;0895F08D-E71D-4197-896D-5BE29B632798 17;16 Not Applicable;Not Applicable Not Applicable;Not Applicable Not Applicable;Not Applicable 567;1474 Not Applicable;Not Applicable Not Available;Not Available Scheduled Follow-up Submission;Scheduled Follow-up Submission Complete Remission/Response;Complete Remission/Response Not Available;Not Available Not Available;NO 5;2 Not Available;Not Available NO;NO Not Available;Not Available TUMOR FREE;TUMOR FREE YES;YES Complete Remission/Response;Complete Remission/Response Not Available;Not Available NO;NO Alive;Alive 2012;2015 FEMALE Lung Adenocarcinoma- Not Otherwise Specified (NOS) No C34.1 8140/3 C34.1 YES Not Available Not Available Not Available Not Available Not Available 7 Not Available Not Available Not Available Not Available Not Available Not Available Not Available Not Available Not Available Not Available 25 No 5941 Not Available Not Available Not Available Not Available Not Available Not Available Not Available Not Available NO WHITE Not Available NA Not Available Not Applicable Not Applicable Not Applicable Not Applicable Not Applicable Not Applicable Not Applicable Not Applicable Not Applicable Stage IIIA Not Applicable Not Applicable Not Applicable 7th Not Applicable Not Applicable Not Applicable M0 N2 T2a Not Available NO YES 50 2 Lung Alive 2011 2010 Not Available

664 Alive T2a N1 M0 Stage IIA NA 56 L-Upper Not Applicable TCGA-L9-A743 A5F2576A-D628-47DE-A5DF-76A4F3105DCC 14 -20780 Not Applicable 0 312 Not Available Lung Adenocarcinoma 45 NA TCGA-L9-A743-D58553;TCGA-L9-A743-D58554 88D31EDF-D154-4FAF-B5A3-0BABE6D20D73;BD488F92-7CB9-4C9E-B60D-9F199C561475 Not Available;Not Available 14;14 193;193 120;120 TAXOTERE;CISPLATIN Complete Response;Complete Response 4;4 Not Available;Not Available Not Available;Not Available Not Available;Not Available Not Available;Not Available Not Applicable;Not Applicable Not Available;Not Available Not Available;Not Available NO;NO Chemotherapy;Chemotherapy Not Available;Not Available Not Available;Not Available Not Available;Not Available NO;NO 2014;2014 0 Not Available NO Not Available Not Available Not Available NO Not Available NOT HISPANIC OR LATINO NA Not Available Not Available Not Available Not Available TCGA-L9-A743-F66554 F0B1840B-09D9-4DEB-978E-CEAB73FADEC9 20 Not Applicable Not Applicable Not Applicable 664 Not Applicable 0 Scheduled Follow-up Submission Complete Remission/Response Not Available NO 10 Not Available NO Preoperative TUMOR FREE YES Complete Remission/Response Not Available NO Alive 2014 MALE Lung Adenocarcinoma- Not Otherwise Specified (NOS) No C34.1 8140/3 C34.1 YES Not Available NO Not Available Not Available Central Lung 4 Not Available Not Available Not Available Not Available Not Available Not Available Not Available Not Available Not Available NO 40.5 Yes, History of Prior Malignancy A743 Preoperative TUMOR FREE 81 78 YES 85 88 Complete Remission/Response YES BLACK OR AFRICAN AMERICAN NO NA R0 Not Applicable Not Applicable Not Applicable Not Applicable Not Applicable Not Applicable Not Applicable Not Applicable Not Applicable Stage IIA Not Applicable Not Applicable Not Applicable 7th Not Applicable Not Applicable Not Applicable M0 N1 T2a Not Available YES NO L9 2 Lung Alive 2014 2013 1986

728 Alive T3 N0 M0 Stage IIB NA FPPP TCGA 64 L-Lower Not Applicable TCGA-44-6146 0c0b610e-fe4c-406d-a5ed-5cc3b11dabf5 7 -23378 Not Applicable 0 302 Not Available Lung Adenocarcinoma 76 NA TCGA-44-6146-D12761;TCGA-44-6146-D12762;TCGA-44-6146-D12759 e3862c58-05db-4b2a-8c54-fc72bee3ce23;b7252803-5a38-42ce-8246-c0298c823279;e1605149-0bd0-4889-8db8-71f1eeacda90 Not Available;Not Available;Not Available 7;7;7 107;107;44 44;66;44 Alimta;Carboplatin;Cisplatin Not Available;Not Available;Not Available 6;6;6 4;3;1 1100;456-570;165 mg;mg;mg ADJUVANT;ADJUVANT;ADJUVANT Not Applicable;Not Applicable;Not Applicable 1;1;1 IV;IV;IV NO;NO;NO Chemotherapy;Chemotherapy;Chemotherapy Not Available;Not Available;Not Available 4400;1600;165 mg;mg;mg Not Available;Not Available;Not Available 2011;2011;2011 1 Not Available Not Available Not Available Not Available Not Available Not Available Not Available NOT HISPANIC OR LATINO NA Not Available;YES Not Available;NO Not Available;NO Not Available;Not Available TCGA-44-6146-F12758;TCGA-44-6146-F34551 37ef67dc-0389-44bd-9265-02d220a94c2f;0DE929CF-9DF4-4EB5-9E02-DDF823FD3413 7;22 Not Available;Not Available Not Available;Not Available Not Applicable;Not Applicable 302;728 Not Available;631 1;0 Not Available;Additional New Tumor Event Not Available;Unknown Not Available;Not Available Not Available;NO 6;8 Not Available;Locoregional Recurrence Not Available;YES Adjuvant therapy;Other TUMOR FREE;Unknown YES;Not Available Complete Remission/Response;Not Available Not Available;Convincing Imaging NO;Not Available Alive;Alive 2011;2012 MALE Lung Mucinous Adenocarcinoma No C34.30 8480/3 C34.3 YES Not Available Not Available Not Available Not Available Not Available 6 Not Available Not Available Not Available Not Available Not Available Not Available Not Available Not Available Not Available Not Available 15 Yes 6146 Other TUMOR FREE 95 94 Not Available 88 87 Not Available YES WHITE Not Available NA Not Available Not Applicable Not Applicable Not Applicable Not Applicable Not Applicable Not Applicable Not Applicable Not Applicable Not Applicable Stage IIB Not Applicable Not Applicable Not Applicable 7th Not Applicable Not Applicable Not Applicable M0 N0 T3 1980 YES NO 44 3 Lung Alive 2011 2010 Not Available

300 Dead T1b N0 M0 Stage IA NA 52 R-Lower Not Applicable TCGA-4B-A93V F1F80DD2-891D-46DD-A3E0-F007628CB515 11 -19035 Not Applicable 0 254 Not Available Lung Adenocarcinoma Not Available NA 0 Not Available Not Available Not Available Not Available Not Available Not Available Not Available NOT HISPANIC OR LATINO NA NO;NO YES;YES NO;NO NO;NO TCGA-4B-A93V-F67799;TCGA-4B-A93V-F67919 A3A0DE3B-6050-4CEB-9F60-77C7AC386672;36B3E382-04CE-480A-BA97-2A3D1877B59A 11;12 Not Available;Not Available Not Available;Not Available Not Applicable;300 254;Not Available 251;251 2;Not Evaluated Scheduled Follow-up Submission;Additional New Tumor Event Progressive Disease;Progressive Disease 60;0 NO;YES 11;11 Distant Metastasis;Distant Metastasis YES;YES Pre-Adjuvant Therapy;Post-Adjuvant Therapy WITH TUMOR;WITH TUMOR NO;NO Progressive Disease;Progressive Disease Convincing Imaging;Convincing Imaging YES;YES Alive;Dead 2014;2014 FEMALE Lung Adenocarcinoma Mixed Subtype No C34.3 8140/3 C34.3 YES 100 Not Available Not Available Not Available Peripheral Lung 11 NO YES 251 NO Not Available NO Not Available Distant Metastasis Convincing Imaging YES 30 No A93V Preoperative WITH TUMOR Not Available Not Available NO Not Available Not Available Progressive Disease NO BLACK OR AFRICAN AMERICAN YES NA Distant site TCGA-4B-A93V-R67898 AA1EAA14-5EC7-47BC-8073-6F27DB7718CB Not Available 12 267 252 Radiographic Progressive Disease 11 10 2500 NO External Not Applicable Not Available Not Available cGy 2014 R0 Not Applicable Not Applicable Not Applicable Not Applicable Not Applicable Not Applicable Not Applicable Not Applicable Not Applicable Stage IA Not Applicable Not Applicable Not Applicable 7th Not Applicable Not Applicable Not Applicable M0 N0 T1b 2013 YES NO 4B 4 Lung Alive 2014 2013 1984

74 Dead T1 N2 M0 Stage IIIA NA 77 R-Middle Not Applicable TCGA-MP-A4TC 99C35245-E1C8-4805-B7A5-F703BB506030 2 -28471 74 0 Not Available Not Available Lung Adenocarcinoma 68 NA TCGA-MP-A4TC-D41397;TCGA-MP-A4TC-D41398 2D936FD6-139F-4EBD-9EAB-D843254E987A;ED1C314A-2C4D-4A19-A6A2-0FB17145C3E5 Not Available;Not Available 18;18 25;25 25;25 Cisplatin;Navelbine Complete Response;Complete Response 3;3 Not Available;Not Available Not Available;Not Available Not Available;Not Available Not Available;Not Available Not Applicable;Not Applicable Not Available;Not Available Not Available;Not Available NO;NO Chemotherapy;Chemotherapy Not Available;Not Available Not Available;Not Available Not Available;Not Available NO;NO 2013;2013 Not Evaluated Not Available NO Not Available Not Available Not Available NO Not Available NOT HISPANIC OR LATINO NA MALE Lung Adenocarcinoma- Not Otherwise Specified (NOS) No C34.2 8140/3 C34.2 YES Not Evaluated NO Not Available Not Available Unknown 4 Not Available Not Available Not Available Not Available Not Available Not Available Not Available Not Available Not Available NO 125 Yes, History of Prior Malignancy A4TC Not Evaluated TUMOR FREE Not Available 76 YES Not Available 83 Complete Remission/Response YES WHITE NO NA R0 Not Applicable Not Applicable Not Applicable Not Applicable Not Applicable Not Applicable Not Applicable Not Applicable Not Applicable Stage IIIA Not Applicable Not Applicable Not Applicable 6th Not Applicable Not Applicable Not Applicable M0 N2 T1 1976 NO YES MP 3 Lung Dead 2013 2008 1944

550 Dead T1a N0 MX Stage IA NA 61 L-Upper Not Applicable TCGA-J2-A4AD 096BD95F-9900-4DB2-B1C4-103902C3B31F 27 -22414 Not Applicable 0 287 Not Available Lung Adenocarcinoma Not Available NA Unknown Not Available NO Not Available Not Available Not Available NO Not Available NOT HISPANIC OR LATINO NA NO NO Not Available Not Available TCGA-J2-A4AD-F55524 7AD20CAA-27DA-470D-838D-F1A687F16E39 8 Not Available Not Available 550 Not Available 524 Not Available Scheduled Follow-up Submission Not Applicable Not Available NO 1 Locoregional Recurrence;Distant Metastasis YES Not Available WITH TUMOR NO Unknown Convincing Imaging NO Dead 2014 FEMALE Lung Adenocarcinoma- Not Otherwise Specified (NOS) No C34.1 8140/3 C34.1 YES Unknown NO Not Available Not Available Not Available 12 Not Available Not Available Not Available Not Available Not Available Not Available Not Available Not Available Not Available NO 120 No A4AD Unknown TUMOR FREE Not Available Not Available NO Not Available 1.95 Unknown YES WHITE NO NA RX Not Applicable Not Applicable Not Applicable Not Applicable Not Applicable Not Applicable Not Applicable Not Applicable Not Applicable Stage IA Not Applicable Not Applicable Not Applicable 7th Not Applicable Not Applicable Not Applicable MX N0 T1a Not Available YES NO J2 2 Lung Alive 2012 2012 1970

1119 Alive T3 N0 M0 Stage IIB NA 68 R-Lower Not Applicable TCGA-50-8459 9cfc1519-33e7-4145-a4ad-78b5c449853a 16 -24859 Not Applicable 0 231 Not Available Lung Adenocarcinoma 70 NA TCGA-50-8459-D42678;TCGA-50-8459-D42680;TCGA-50-8459-D42681 F90C20E9-5A78-4BD2-8814-AEFA21BA329D;4E2BDC04-BC96-442A-A92E-F5E7E00A1E90;C21CFFFF-014E-482A-B414-AFF5106E530C Not Available;Not Available;Not Available 26;26;26 147;147;147 76;76;76 ALIMTA;Cisplatin;Avastin Complete Response;Complete Response;Complete Response 4;4;4 Not Available;Not Available;Not Available Not Available;Not Available;Not Available Not Available;Not Available;Not Available Not Available;Not Available;Not Available Not Applicable;Not Applicable;Not Applicable Not Available;Not Available;Not Available Not Available;Not Available;Not Available NO;NO;NO Chemotherapy;Chemotherapy;Chemotherapy Not Available;Not Available;Not Available Not Available;Not Available;Not Available Not Available;Not Available;Not Available YES;YES;YES 2013;2013;2013 Not Available Not Available YES Not Available Not Available Not Available Not Available Not Available NOT HISPANIC OR LATINO NA NO;Not Available NO;Not Available YES;Not Available Not Available;Not Available TCGA-50-8459-F69334;TCGA-50-8459-F70410 9A74D1CF-D426-4C2E-AF2F-25F0C20A6705;63A0899A-AF36-4E80-A590-B5EF20006A8C 5;11 432;Not Applicable Not Available;Not Applicable Not Applicable;Not Applicable 589;1119 432;Not Applicable Not Available;Not Available Scheduled Follow-up Submission;Scheduled Follow-up Submission Progressive Disease;Progressive Disease Not Available;Not Available NO;NO 1;2 Locoregional Recurrence;Not Available YES;NO Not Available;Not Available WITH TUMOR;WITH TUMOR YES;YES Progressive Disease;Progressive Disease Biopsy with Histologic Confirmation;Not Available NO;NO Alive;Alive 2015;2015 MALE Mucinous (Colloid) Carcinoma No C34.30 8480/3 C34.3 YES Not Available YES YES G12C Not Available 10 Not Available Not Available Not Available Not Available Not Available Not Available Not Available Not Available Not Available NO 10 No 8459 Not Available WITH TUMOR Not Available Not Available YES 70 72 Progressive Disease YES WHITE NO NA Not Available Not Applicable Not Applicable Not Applicable Not Applicable Not Applicable Not Applicable Not Applicable Not Applicable Not Applicable Stage IIB Not Applicable Not Applicable Not Applicable 7th Not Applicable Not Applicable Not Applicable M0 N0 T3 Not Available YES NO 50 2 Lung Alive 2012 2012 Not Available

594 Dead T2 N0 M0 Stage IB NA 65 L-Upper Not Applicable TCGA-62-A46P 0C59FF96-5E4A-4664-A0B1-D057B4725483 29 -23993 594 0 Not Available Not Available Lung Adenocarcinoma Not Available NA Unknown Not Available NO Not Available Not Available Not Available NO Not Available NOT HISPANIC OR LATINO NA MALE Lung Adenocarcinoma Mixed Subtype No C34.1 8255/3 C34.1 YES 100 NO Not Available Not Available Central Lung 10 YES NO 267 Not Available Not Available YES 267 Distant Metastasis Convincing Imaging YES 40 No A46P Preoperative TUMOR FREE Not Available Not Available NO 66 78 Complete Remission/Response YES WHITE NO NA R0 Not Applicable Not Applicable Not Applicable Not Applicable Not Applicable Not Applicable Not Applicable Not Applicable Not Applicable Stage IB Not Applicable Not Applicable Not Applicable 6th Not Applicable Not Applicable Not Applicable M0 N0 T2 Not Available NO YES 62 4 Lung Dead 2012 2006 Not Available

2318 Dead T1 N0 M0 Stage IA NA 72 R-Lower Not Applicable TCGA-49-4486 61c655ec-52b5-453f-a6cc-b2aba445b027 3 -26415 2318 0 Not Available Not Available Lung Adenocarcinoma Not Available NA 1 Not Available Not Available Not Available Not Available Not Available Not Available Not Available NOT HISPANIC OR LATINO NA NO NO NO NO TCGA-49-4486-F58843 67E6E0FC-CA40-4FBA-B179-4F85F0E039F6 25 Not Available Not Available 2318 Not Available 2045 2 Scheduled Follow-up Submission Progressive Disease 60 NO 4 Distant Metastasis YES Other WITH TUMOR NO Complete Remission/Response Convincing Imaging NO Dead 2014 MALE Lung Adenocarcinoma- Not Otherwise Specified (NOS) No C34.3 8140/3 C34.3 YES Not Available Not Available Not Available Not Available Not Available 2 Not Available Not Available Not Available Not Available Not Available Not Available Not Available Not Available Not Available Not Available 17.5 No 4486 Other WITH TUMOR 69 96 Not Available 77 108 Not Available YES WHITE Not Available NA R0 Not Applicable Not Applicable Not Applicable Not Applicable Not Applicable Not Applicable Not Applicable Not Applicable Not Applicable Stage IA Not Applicable Not Applicable Not Applicable 3rd Not Applicable Not Applicable Not Applicable M0 N0 T1 1967 NO YES 49 3 Lung Dead 2011 1992 1932

866 Alive T2 N1 M0 Stage IIB NA 74 R-Upper Not Applicable TCGA-64-5815 a65700c2-e58c-4fd4-aeb1-5686b8f4d212 2 -27280 Not Applicable 0 224 Not Available Lung Adenocarcinoma 87 NA TCGA-64-5815-D11426;TCGA-64-5815-D11429;TCGA-64-5815-D11431;TCGA-64-5815-D11434;TCGA-64-5815-D31481;TCGA-64-5815-D31482;TCGA-64-5815-D31483;TCGA-64-5815-D31484 c5c5b4f0-2f37-4d6a-b486-29c7638c00e5;96ec4fe4-fb5e-4fd3-903d-aef8816562c7;0750d485-4281-41ea-a62c-7860f4e50db9;35b6dcb0-3140-4e22-8857-a5adcc1bd27f;65568c73-1054-4a71-bb8c-ecdd22eaf03e;f1173777-7ce0-422c-8f6f-e4216233f93e;20c7f73f-0afe-4e4a-b887-93338bfd9df6;4878b403-b86b-448f-a98c-f33fb9f7ea01 Not Available;Not Available;Not Available;Not Available;Not Available;Not Available;Not Available;Not Available 2;2;2;2;3;3;3;3 103;103;188;188;132;132;195;195 93;93;146;146;83;83;146;146 Carboplatin;Gemzar;Alimta;Carboplatin;Gemcitabine;Carboplatin;Carboplatin;Almita Not Available;Not Available;Not Available;Not Available;Complete Response;Complete Response;Complete Response;Complete Response 5;5;5;5;5;5;5;5 3;3;3;3;Not Available;Not Available;Not Available;Not Available Not Available;Not Available;Not Available;Not Available;Not Available;Not Available;Not Available;Not Available Not Available;Not Available;Not Available;Not Available;Not Available;Not Available;Not Available;Not Available ADJUVANT;ADJUVANT;ADJUVANT;ADJUVANT;Not Available;Not Available;Not Available;Not Available Not Applicable;Not Applicable;Not Applicable;Not Applicable;Not Applicable;Not Applicable;Not Applicable;Not Applicable 1;1;1;1;Not Available;Not Available;Not Available;Not Available IV;IV;IV;IV;Not Available;Not Available;Not Available;Not Available NO;NO;NO;NO;NO;NO;NO;NO Chemotherapy;Chemotherapy;Chemotherapy;Chemotherapy;Chemotherapy;Chemotherapy;Chemotherapy;Chemotherapy Not Available;Not Available;Not Available;Not Available;Not Available;Not Available;Not Available;Not Available Not Available;Not Available;Not Available;Not Available;Not Available;Not Available;Not Available;Not Available Not Available;Not Available;Not Available;Not Available;Not Available;Not Available;Not Available;Not Available Not Available;Not Available;Not Available;Not Available;NO;NO;NO;NO 2011;2011;2011;2011;2012;2012;2012;2012 Not Available Not Available YES Not Available Not Available Not Available Not Available Not Available NOT HISPANIC OR LATINO NA Not Available;Not Available Not Available;Not Available Not Available;Not Available Not Available;Not Available TCGA-64-5815-F11425;TCGA-64-5815-F31480 a63ab9cf-8d93-4b05-b5e0-1134350e3251;986ba4af-3586-4aca-af09-a3aded054b3c 2;3 Not Applicable;Not Available Not Applicable;Not Available Not Applicable;Not Applicable 224;866 Not Applicable;Not Available Not Available;1 Not Available;Scheduled Follow-up Submission Complete Remission/Response;Unknown Not Available;Unknown Not Available;Not Available 5;5 Not Available;Not Available NO;Unknown Not Available;Post-Adjuvant Therapy TUMOR FREE;Unknown YES;YES Complete Remission/Response;Complete Remission/Response Not Available;Not Available NO;NO Alive;Alive 2011;2012 MALE Lung Adenocarcinoma Mixed Subtype No C34.1 8255/3 C34.1 YES Not Available YES Not Available Not Available Central Lung 5 Not Available Not Available Not Available Not Available Not Available Not Available Not Available Not Available Not Available Not Available 30 No 5815 Not Available TUMOR FREE Not Available Not Available Not Available 76 89 Not Available YES WHITE Not Available NA R0 Not Applicable Not Applicable Not Applicable Not Applicable Not Applicable Not Applicable Not Applicable Not Applicable Not Applicable Stage IIB Not Applicable Not Applicable Not Applicable 6th Not Applicable Not Applicable Not Applicable M0 N1 T2 1985 NO YES 64 3 Lung Alive 2011 2009 Not Available

225 Alive T1 N0 M0 Stage IA NA 69 L-Upper Not Applicable TCGA-35-4122 44adf4cb-2829-4153-80fd-a8b7ee0e654a 20 -25491 Not Applicable 0 225 Not Available Lung Adenocarcinoma Not Available NA 1 Not Available NO Not Available Not Available Not Available NO Not Available NOT HISPANIC OR LATINO NA Not Available Not Available Not Available Not Available TCGA-35-4122-F68915 38E92345-0056-4825-BE80-B4E43166B1D2 23 Not Available Not Available Not Applicable Not Available Not Available Not Available Scheduled Follow-up Submission Not Applicable Not Available YES 12 Not Available Not Available Not Available Not Available Unknown Unknown Not Available Unknown Not Available 2014 MALE Lung Adenocarcinoma- Not Otherwise Specified (NOS) No C34.1 8140/3 C34.1 YES 80 NO Not Available Not Available Peripheral Lung 12 Not Available Not Available Not Available Not Available Not Available Not Available Not Available Not Available Not Available Not Available 45 No 4122 Preoperative TUMOR FREE Not Available Not Available Not Available Not Available Not Available Not Available YES WHITE Not Available NA R0 Not Applicable Not Applicable Not Applicable Not Applicable Not Applicable Not Applicable Not Applicable Not Applicable Not Applicable Stage IA Not Applicable Not Applicable Not Applicable 7th Not Applicable Not Applicable Not Applicable M0 N0 T1 2010 NO YES 35 4 Lung Alive 2010 2010 1965
[truncated: 54,023 more chars]
