# Supplementary material for: An Immune-Related Signature Predicts Survival in Patients With Lung Adenocarcinoma
Source: Front Oncol. 2019 Dec 10;9:1314. doi: 10.3389/fonc.2019.01314 (PMC6914845; doi:10.3389/fonc.2019.01314)
Supplement: Supplementary file 5 [file Table_5.doc]

**Table S5. Data from the test set samples.**

Genes TCGA-55-8205-01 TCGA-75-5126-01 TCGA-91-6849-01 TCGA-55-7903-01 TCGA-55-6979-01 TCGA-86-8074-01 TCGA-86-8673-01 TCGA-44-2656-01 TCGA-35-5375-01 TCGA-05-4417-01 TCGA-05-4244-01 TCGA-91-6831-01 TCGA-64-1681-01 TCGA-05-4396-01 TCGA-95-7039-01 TCGA-55-A4DF-01 TCGA-MP-A4TE-01 TCGA-97-A4LX-01 TCGA-49-6745-01 TCGA-62-A472-01 TCGA-97-A4M2-01 TCGA-50-6595-01 TCGA-75-7025-01 TCGA-MP-A4SY-01 TCGA-62-A46O-01 TCGA-78-7154-01 TCGA-05-4397-01 TCGA-95-A4VP-01 TCGA-NJ-A4YG-01 TCGA-75-6206-01 TCGA-55-7284-01 TCGA-86-8280-01 TCGA-50-6597-01 TCGA-62-8395-01 TCGA-78-7149-01 TCGA-49-AARO-01 TCGA-05-4389-01 TCGA-69-7761-01 TCGA-91-6828-01 TCGA-67-3771-01 TCGA-78-7158-01 TCGA-86-8671-01 TCGA-97-A4M6-01 TCGA-L4-A4E5-01 TCGA-91-A4BC-01 TCGA-62-A46R-01 TCGA-91-8499-01 TCGA-38-4626-01 TCGA-49-AAQV-01 TCGA-50-5944-01 TCGA-80-5608-01 TCGA-L9-A7SV-01 TCGA-69-7760-01 TCGA-91-6830-01 TCGA-95-7562-01 TCGA-50-5932-01 TCGA-55-1596-01 TCGA-05-4425-01 TCGA-69-8254-01 TCGA-55-7573-01 TCGA-95-A4VN-01 TCGA-44-8119-01 TCGA-86-8075-01 TCGA-97-7937-01 TCGA-38-4632-01 TCGA-75-5146-01 TCGA-78-8640-01 TCGA-MP-A4T8-01 TCGA-97-8547-01 TCGA-49-AARN-01 TCGA-L9-A5IP-01 TCGA-55-7995-01 TCGA-69-8253-01 TCGA-05-4427-01 TCGA-53-7624-01 TCGA-44-5643-01 TCGA-73-4670-01 TCGA-O1-A52J-01 TCGA-44-7667-01 TCGA-97-A4M5-01 TCGA-NJ-A4YP-01 TCGA-97-7552-01 TCGA-86-8073-01 TCGA-49-6761-01 TCGA-91-A4BD-01 TCGA-05-4410-01 TCGA-50-5939-01 TCGA-50-5936-01 TCGA-99-8028-01 TCGA-64-1677-01 TCGA-44-5645-01 TCGA-05-4384-01 TCGA-86-7955-01 TCGA-44-8120-01 TCGA-44-2666-01 TCGA-50-8460-01 TCGA-55-8208-01 TCGA-44-A4SU-01 TCGA-55-6980-01 TCGA-NJ-A55A-01 TCGA-49-6742-01 TCGA-67-6215-01 TCGA-38-A44F-01 TCGA-78-8655-01 TCGA-44-7671-01 TCGA-49-4488-01 TCGA-35-3615-01 TCGA-55-6972-01 TCGA-44-2665-01 TCGA-86-8054-01 TCGA-69-7978-01 TCGA-05-5425-01 TCGA-55-7227-01 TCGA-49-4490-01 TCGA-MP-A4TH-01 TCGA-55-7815-01 TCGA-49-4494-01 TCGA-55-8094-01 TCGA-55-8615-01 TCGA-50-5051-01 TCGA-50-5055-01 TCGA-64-1678-01 TCGA-97-8174-01 TCGA-55-8506-01 TCGA-78-7536-01 TCGA-99-8032-01 TCGA-05-5429-01 TCGA-49-AARQ-01 TCGA-05-4426-01 TCGA-J2-8194-01 TCGA-55-6984-01 TCGA-91-7771-01 TCGA-49-4514-01 TCGA-05-4420-01 TCGA-38-4630-01 TCGA-78-7145-01 TCGA-38-4627-01 TCGA-73-4666-01 TCGA-44-6145-01 TCGA-05-4422-01 TCGA-50-5930-01 TCGA-73-4659-01 TCGA-99-8025-01 TCGA-50-8457-01 TCGA-55-7994-01 TCGA-64-1679-01 TCGA-64-1680-01 TCGA-44-3919-01 TCGA-55-8621-01 TCGA-75-6211-01 TCGA-38-4625-01 TCGA-69-7763-01 TCGA-44-A47G-01 TCGA-55-8206-01 TCGA-05-4418-01 TCGA-49-4487-01 TCGA-44-A479-01 TCGA-55-1592-01 TCGA-L4-A4E6-01 TCGA-44-6777-01 TCGA-J2-A4AG-01 TCGA-86-8281-01 TCGA-78-7166-01 TCGA-44-2655-01 TCGA-69-7765-01 TCGA-44-2661-01 TCGA-73-4677-01 TCGA-44-4112-01 TCGA-49-AARE-01 TCGA-95-7948-01 TCGA-86-7953-01 TCGA-55-8513-01 TCGA-50-5931-01 TCGA-MN-A4N4-01 TCGA-55-5899-01 TCGA-97-8177-01 TCGA-55-A491-01 TCGA-97-8175-01 TCGA-91-6840-01 TCGA-64-5775-01 TCGA-44-6147-01 TCGA-MP-A4T6-01 TCGA-44-A4SS-01 TCGA-55-7907-01 TCGA-86-8585-01 TCGA-69-7973-01 TCGA-NJ-A7XG-01 TCGA-44-6779-01 TCGA-50-6591-01 TCGA-MP-A4TJ-01 TCGA-97-7546-01 TCGA-50-6592-01 TCGA-55-7283-01 TCGA-55-8299-01 TCGA-99-7458-01 TCGA-99-8033-01 TCGA-97-A4M7-01 TCGA-44-6775-01 TCGA-78-7537-01 TCGA-44-3917-01 TCGA-49-4505-01 TCGA-05-4398-01 TCGA-55-8620-01 TCGA-75-5122-01 TCGA-67-6216-01 TCGA-55-7725-01 TCGA-55-1594-01 TCGA-97-8552-01 TCGA-50-5942-01 TCGA-73-7499-01 TCGA-78-7161-01 TCGA-97-7941-01 TCGA-MP-A4SW-01 TCGA-05-5715-01 TCGA-05-4434-01 TCGA-55-7911-01 TCGA-55-7728-01 TCGA-55-8510-01 TCGA-91-6836-01 TCGA-NJ-A4YI-01 TCGA-38-6178-01 TCGA-91-6848-01 TCGA-55-7724-01 TCGA-55-6978-01 TCGA-55-8619-01 TCGA-55-8091-01 TCGA-44-7662-01 TCGA-49-AAR3-01 TCGA-55-6982-01 TCGA-55-7816-01 TCGA-MP-A4TK-01 TCGA-78-7167-01 TCGA-69-7974-01 TCGA-62-8399-01 TCGA-78-7160-01 TCGA-49-AAR0-01 TCGA-67-3770-01 TCGA-95-7043-01 TCGA-55-6983-01 TCGA-49-AAR2-01 TCGA-95-7567-01 TCGA-93-A4JQ-01 TCGA-75-7027-01 TCGA-55-7914-01 TCGA-50-5933-01 TCGA-55-A4DG-01 TCGA-75-6212-01 TCGA-55-8616-01 TCGA-55-8096-01 TCGA-86-8669-01 TCGA-05-5428-01 TCGA-MP-A4T4-01 TCGA-05-4415-01 TCGA-80-5611-01 TCGA-05-4395-01 TCGA-69-8453-01 TCGA-49-4501-01

ENSG00000160862.11 67.7811660766602 194.906799316406 5.04723596572876 8.70651435852051 143.341369628906 275.221252441406 45.6800842285156 469.864776611328 0.20400270819664 13.4762649536133 544.487976074219 0.772799134254456 55.2527503967285 26.7456798553467 10.4294242858887 2.01709318161011 17.6093692779541 15.0626420974731 44.3909797668457 56.5276756286621 55.3298950195312 4.72140407562256 272.407531738281 7.47779273986816 0.841214239597321 663.217834472656 165.191482543945 2.47674465179443 45.2150688171387 0.36288794875145 428.044281005859 199.134628295898 0.796324729919434 2.22369050979614 3.63318061828613 54.9479827880859 85.2760696411133 110.43482208252 25.282564163208 1.22701704502106 37.12451171875 149.786026000977 63.4154891967773 59.3096122741699 0.933089792728424 8.97841548919678 2166.55151367188 31.7898120880127 456.007110595703 5.7092113494873 5.25834846496582 4.80988121032715 114.527473449707 87.8832397460938 7.28294944763184 6.80778503417969 1132.70300292969 0.484225422143936 248.705474853516 158.847198486328 59.2768440246582 1.09805512428284 22.9059600830078 11.7498836517334 4.13544797897339 23.1820392608643 5.05142068862915 30.4487323760986 346.594482421875 70.0409774780273 52.6715850830078 18.3930721282959 7.99661350250244 1.55937778949738 0.056735523045063 1.55444240570068 34.2652053833008 27.7519512176514 0.334257006645203 5.94019412994385 0.750577211380005 59.6507339477539 3.41583466529846 18.9479751586914 1403.19812011719 20.0441055297852 11.4436092376709 7.91568279266357 8.00849723815918 5.30308389663696 28.4770889282227 19.1968955993652 26.4017581939697 14.9125547409058 0.252729654312134 777.992248535156 20.7692394256592 422.531707763672 308.709014892578 6.28739452362061 10.2832078933716 1.57856369018555 112.772689819336 44.756591796875 16.4374599456787 1.49867737293243 1.04593431949615 2.74474239349365 311.096527099609 5.08386945724487 185.328109741211 52.9320602416992 10.4366493225098 297.239593505859 3.05427098274231 63.4931907653809 188.019882202148 21.3322238922119 10.2385282516479 16.8546581268311 3.34674525260925 15.5159502029419 20.6583290100098 0.54403954744339 0.888094127178192 18.3319797515869 265.203369140625 4.74810314178467 432.577117919922 25.9724082946777 22.9736270904541 9.77061367034912 257.177642822266 81.448112487793 0.506872534751892 220.000900268555 505.845092773438 41.6577568054199 20.8677082061768 11.5939331054688 25.0423622131348 28.6487998962402 1.66715562343597 25.2254657745361 10.999490737915 102.957313537598 62.7919998168945 18.0649070739746 17.2354145050049 104.536071777344 735.903503417969 5.74491500854492 12.3544464111328 1047.08740234375 1.85613191127777 8.23021793365479 19.2696304321289 151.945327758789 9.96030235290527 309.985961914062 7.9145884513855 10.8148250579834 4.21911239624023 66.4674606323242 5.93873643875122 11.7741584777832 9.1601505279541 1.30684518814087 3.37406158447266 5.39334058761597 7.52307271957397 49.1793174743652 190.230010986328 106.164619445801 12.288854598999 245.096694946289 8.22971534729004 7.75022125244141 0.532241940498352 1.26401770114899 7.35437202453613 6.03320121765137 8.34120655059814 0.603548526763916 1.76785457134247 2.72392344474792 204.430084228516 169.361328125 0.038826510310173 8.60693836212158 61.3310050964355 2.09571957588196 12.7689485549927 63.7518615722656 186.662475585938 3.21711993217468 266.139678955078 4.24353361129761 6.1845965385437 0.0776792615652084 75.2588348388672 382.263946533203 62.6606369018555 0.532760083675385 10.1598558425903 248.894470214844 1.45363521575928 973.232482910156 391.69775390625 1259.71044921875 6.32917451858521 90.2362976074219 435.580474853516 11.2362537384033 8.88637638092041 5.13445806503296 176.628982543945 59.139778137207 0.89805668592453 391.337066650391 64.9475250244141 3.60602450370789 10.7959136962891 7.584547996521 18.5445079803467 1673.21667480469 10.1350994110107 1.06261169910431 16.8311195373535 52.6469688415527 229.23518371582 1.51503443717957 1.99678540229797 11.403413772583 366.059753417969 59.1622886657715 761.618896484375 3.1111741065979 16.8639335632324 3.13352942466736 1.66754531860352 26.4748287200928 80.5628356933594 0.443223237991333 154.878555297852 129.438812255859 1587.58447265625 3.93438839912415 476.235412597656 15.86536693573 0.877534985542297 18.3933067321777 1.93153893947601 255.102020263672 324.133819580078 89.695686340332 26.9873790740967

ENSG00000166710.16 5276.41455078125 5026.9033203125 1853.55859375 2400.9619140625 3472.97924804688 3186.26196289062 1086.20361328125 4571.43798828125 2768.9677734375 2973.3681640625 2377.005859375 1918.76318359375 3314.35327148438 4298.1943359375 1065.81909179688 1978.67907714844 571.552673339844 3639.68530273438 3299.93994140625 2694.02758789062 2537.720703125 4391.20703125 2294.47119140625 1433.41027832031 1969.275390625 2379.7373046875 2754.03173828125 1242.91137695312 1703.974609375 1966.7216796875 4848.62548828125 4339.20458984375 2175.49975585938 1188.802734375 949.031311035156 1758.8642578125 3211.0966796875 7351.22412109375 2181.30053710938 3096.61450195312 1132.96240234375 4194.03271484375 4246.888671875 876.302978515625 1936.17919921875 2742.23950195312 2783.97143554688 3402.71337890625 2095.8759765625 1596.21301269531 2188.35546875 383.173583984375 1173.20556640625 2411.91723632812 2805.56958007812 2014.97033691406 4928.1162109375 4469.6904296875 3101.84716796875 3168.06689453125 2048.44750976562 2003.16882324219 2826.25390625 1056.01403808594 2817.2314453125 2587.26196289062 1230.73950195312 769.368469238281 1839.83996582031 2369.35034179688 2246.47143554688 2815.38720703125 1639.92797851562 3315.70361328125 4262.86865234375 1714.96081542969 1360.34436035156 2351.35278320312 738.32861328125 3744.37158203125 2150.65478515625 7187.1552734375 2118.48803710938 1522.98522949219 2809.14379882812 4019.72534179688 3063.0888671875 1662.48583984375 4916.41259765625 1848.24157714844 1852.91040039062 1768.91613769531 719.585815429688 1338.03588867188 925.164367675781 1463.62463378906 3969.70678710938 1118.17504882812 3334.50317382812 2504.08764648438 2550.43896484375 1644.72399902344 5387.26025390625 2331.56030273438 1155.17553710938 3524.58032226562 1870.1416015625 480.399353027344 4864.9970703125 994.094299316406 3420.25512695312 5113.0634765625 5237.84765625 1405.21984863281 2996.15502929688 3015.04858398438 3416.81298828125 1460.27319335938 665.084838867188 959.484313964844 5308.07861328125 1259.14489746094 2058.10766601562 1622.18225097656 1607.19714355469 1529.70947265625 5388.68408203125 216.434005737305 2859.03198242188 1498.95263671875 1916.16711425781 3890.63842773438 613.763366699219 2074.1396484375 1034.48718261719 1659.87194824219 3918.01245117188 3971.24609375 4822.1806640625 5420.576171875 1855.93041992188 1836.59521484375 1370.16625976562 2250.28955078125 2439.72485351562 2548.041015625 1915.10510253906 3443.04443359375 4015.28344726562 1400.69030761719 3923.59716796875 1615.4404296875 3645.4736328125 1500.25659179688 4116.4482421875 2379.48901367188 3365.38452148438 2208.62963867188 3768.99560546875 3476.77612304688 2116.71728515625 1277.34265136719 823.450561523438 3646.24340820312 3154.90478515625 5433.12060546875 2198.12622070312 1021.08868408203 891.994689941406 3025.71557617188 2565.79931640625 3392.3271484375 1075.38659667969 1732.68139648438 2583.51171875 3390.30883789062 1358.2529296875 3378.046875 2138.5654296875 2625.95971679688 1282.11376953125 2103.9619140625 2235.08569335938 2004.21459960938 1737.55505371094 1236.11999511719 1249.35021972656 6696.24658203125 702.797241210938 4280.009765625 2192.30151367188 3704.69848632812 2201.64819335938 4584.9736328125 4496.64208984375 3335.14575195312 1987.04577636719 3759.80297851562 1853.92895507812 873.830505371094 4028.41625976562 2845.67065429688 1539.8955078125 6939.65869140625 1956.40246582031 3667.14331054688 2393.29125976562 1959.12329101562 1521.60021972656 4961.8955078125 1215.48754882812 2123.74365234375 1746.12341308594 2681.43994140625 4906.1494140625 3951.048828125 1605.61169433594 2510.07397460938 1750.50378417969 1150.20703125 1865.71215820312 2513.50512695312 3044.62622070312 5599.4990234375 2895.3466796875 3509.15747070312 2670.32788085938 2480.9462890625 4215.29052734375 4980.013671875 1853.44580078125 999.950805664062 2398.22900390625 994.821472167969 3949.646484375 1502.15588378906 5113.06591796875 748.474975585938 2124.35766601562 1368.05834960938 2013.78271484375 2614.59008789062 1686.388671875 2632.84326171875 3629.59057617188 1008.05755615234 2809.51953125 897.730590820312 1845.05139160156 1573.81359863281 2929.55078125 3814.93872070312 1043.423828125 4474.27880859375 1973.72290039062 4753.93115234375 3881.8193359375

ENSG00000179218.12 2363.63647460938 1520.74438476562 1280.10192871094 1247.67712402344 933.497192382812 1953.54077148438 1269.88513183594 706.968078613281 1025.73706054688 1712.53833007812 1439.27490234375 1454.31103515625 1788.29516601562 2732.19140625 1396.55456542969 1910.55688476562 2626.09350585938 1208.23486328125 1293.33605957031 796.546142578125 781.160278320312 2165.85693359375 920.77099609375 827.539001464844 1078.06323242188 1137.73547363281 2904.92407226562 1441.78002929688 1159.59545898438 1617.83532714844 998.865539550781 1403.13525390625 1126.76940917969 2168.66552734375 1655.62890625 1234.95007324219 1624.81872558594 1219.40112304688 1816.64782714844 1362.34045410156 1469.31494140625 1143.82263183594 739.30126953125 984.9287109375 728.334350585938 813.704406738281 1324.31359863281 1784.27917480469 1112.4580078125 1461.86608886719 1891.560546875 718.028198242188 908.728454589844 2809.70361328125 1804.78283691406 1605.67248535156 2139.00805664062 1845.193359375 986.718139648438 1503.50354003906 1013.38958740234 1395.84155273438 1214.09069824219 1378.548828125 993.061279296875 1336.62194824219 1333.75402832031 1088.54211425781 1131.07067871094 1469.79235839844 1178.52758789062 1172.4169921875 1373.61389160156 1256.83874511719 1277.7314453125 1797.19250488281 1194.97326660156 1559.578125 1657.04223632812 1053.46545410156 1105.99755859375 938.421691894531 945.620910644531 1141.64794921875 934.186157226562 1554.76184082031 1489.29467773438 1176.45141601562 972.332153320312 1901.3212890625 628.309509277344 945.496826171875 1603.23913574219 1851.6083984375 187.033538818359 1599.17395019531 1171.86437988281 894.853393554688 1273.74450683594 851.519409179688 986.546142578125 1520.10083007812 934.135192871094 1446.30578613281 1470.22009277344 1431.955078125 1048.2529296875 883.710205078125 1870.79382324219 2176.49877929688 1417.51318359375 2030.13977050781 1232.85791015625 1266.84326171875 1260.97033691406 1786.83190917969 1847.87744140625 1978.86840820312 1876.67919921875 1389.12072753906 1506.51037597656 904.191040039062 872.07958984375 1703.29821777344 1394.96789550781 1558.06982421875 1088.18676757812 852.57421875 1442.10729980469 1164.44079589844 1077.67529296875 1612.61071777344 616.642028808594 3034.09521484375 2410.91650390625 1275.98876953125 1292.04064941406 1398.1748046875 1459.24780273438 1361.34631347656 1648.30749511719 1936.90075683594 1174.59521484375 689.186889648438 2102.80004882812 1672.60473632812 1536.20812988281 1538.10388183594 928.906188964844 1497.44519042969 2487.06689453125 1026.0234375 861.193359375 955.421081542969 1030.80639648438 1924.81274414062 1629.7080078125 1883.90893554688 768.921875 2023.50280761719 1056.96984863281 1488.41870117188 683.347412109375 1567.83361816406 1123.99340820312 961.532165527344 1447.90734863281 172.095153808594 876.981567382812 1812.71948242188 1688.4580078125 1143.29382324219 960.098693847656 2488.50341796875 1593.86206054688 1339.87524414062 1733.7255859375 1697.79431152344 4305.7255859375 2012.21508789062 156.076934814453 1591.17419433594 990.707153320312 740.8125 2061.89306640625 2623.73022460938 1227.99145507812 1165.51416015625 2231.49853515625 1278.75476074219 1103.59704589844 1356.05358886719 1770.58337402344 1304.54675292969 1445.04724121094 1135.85998535156 1159.16455078125 1329.23461914062 2290.9609375 384.163940429688 1065.96374511719 1326.82751464844 1548.708984375 1268.79064941406 1387.20324707031 849.853820800781 2905.26635742188 715.760070800781 731.682434082031 1835.88366699219 1974.65405273438 991.394653320312 1282.82763671875 1961.60827636719 1265.86743164062 1278.97546386719 699.853210449219 1109.42431640625 734.093872070312 969.507629394531 2013.42724609375 1712.33630371094 817.071350097656 1222.51281738281 786.820190429688 1031.68811035156 1424.04211425781 1184.49475097656 1577.5126953125 1141.84509277344 1052.49816894531 1547.81567382812 1220.1982421875 970.843811035156 927.678588867188 1128.01550292969 1071.67553710938 755.950866699219 1839.8740234375 1055.41137695312 1930.62951660156 1106.70434570312 1231.59289550781 1482.32153320312 1534.7529296875 816.151428222656 1013.31280517578 864.562927246094 1070.04272460938 1337.53894042969 893.09228515625 1068.20922851562 1316.12084960938 1327.51013183594 1066.11364746094 1100.810546875 2130.82739257812

ENSG00000127022.13 509.555084228516 464.202056884766 413.731323242188 470.407867431641 269.109741210938 529.2001953125 247.444519042969 412.383850097656 259.361968994141 425.644775390625 685.443603515625 459.928802490234 359.841552734375 859.077087402344 300.417663574219 541.736511230469 301.314147949219 468.790985107422 456.289733886719 436.873199462891 285.122589111328 615.689331054688 320.432922363281 1325.126953125 334.960357666016 931.488403320312 545.81005859375 312.496490478516 445.110870361328 609.391052246094 382.504974365234 454.429138183594 317.764678955078 404.737274169922 276.494720458984 199.548309326172 543.313293457031 603.032775878906 646.7802734375 455.423004150391 487.098571777344 272.247528076172 444.074676513672 353.27294921875 218.21501159668 234.657897949219 391.096832275391 425.680480957031 439.0419921875 1642.39978027344 363.625640869141 230.2001953125 502.180511474609 1088.177734375 564.478576660156 1121.73571777344 403.968933105469 432.397491455078 304.603790283203 567.197509765625 348.775970458984 374.409301757812 600.709838867188 247.973052978516 417.523834228516 383.592010498047 606.015380859375 378.024658203125 346.742492675781 311.006866455078 397.168914794922 343.325897216797 478.986846923828 287.079254150391 324.235961914062 388.499542236328 462.347991943359 530.013549804688 306.544464111328 425.905456542969 436.647277832031 374.605499267578 358.559356689453 389.516540527344 250.533538818359 267.303192138672 697.303771972656 717.683227539062 275.700714111328 315.844512939453 443.738708496094 409.616455078125 299.370910644531 329.019378662109 293.414398193359 403.109161376953 303.227905273438 217.891098022461 516.674194335938 315.692962646484 297.491149902344 879.950744628906 211.400634765625 291.822662353516 599.537414550781 240.257873535156 541.685607910156 468.166534423828 727.021484375 913.318298339844 386.058959960938 451.401916503906 402.176910400391 283.97998046875 562.98046875 833.206176757812 308.923126220703 470.376892089844 321.480987548828 420.090148925781 308.813751220703 442.189056396484 686.747436523438 188.264083862305 610.884765625 544.9814453125 294.211761474609 75.0946578979492 1072.3564453125 868.166137695312 341.796569824219 661.223266601562 280.828338623047 432.339080810547 273.810943603516 470.312225341797 488.23828125 541.382446289062 341.214874267578 628.028930664062 438.225738525391 656.571044921875 263.685180664062 344.59765625 586.150268554688 368.894134521484 719.05322265625 404.691680908203 346.345031738281 322.752410888672 372.390228271484 409.105285644531 272.144348144531 491.519470214844 288.862213134766 298.655944824219 456.089965820312 319.21728515625 244.454620361328 575.139282226562 435.944671630859 506.416809082031 226.493911743164 476.046142578125 309.265563964844 305.138793945312 524.659362792969 341.503753662109 98.9547653198242 564.106811523438 427.963684082031 307.368896484375 770.915161132812 266.298309326172 510.117279052734 418.380249023438 339.802642822266 250.289901733398 432.991455078125 589.807434082031 488.056304931641 153.1748046875 376.934204101562 222.335174560547 152.368667602539 897.73291015625 530.412353515625 548.127685546875 571.696166992188 413.995025634766 474.826171875 392.371704101562 504.876586914062 639.925842285156 685.766967773438 522.501281738281 344.979064941406 456.741088867188 489.210266113281 185.714813232422 511.173248291016 366.553039550781 299.929290771484 460.293121337891 404.320739746094 504.946319580078 622.784912109375 295.361602783203 439.661590576172 532.849792480469 289.962310791016 287.440155029297 298.949432373047 522.056762695312 453.982727050781 421.370727539062 94.9887466430664 185.865966796875 507.005035400391 312.795196533203 681.722412109375 589.735290527344 352.985473632812 413.682067871094 237.153717041016 509.676910400391 279.442413330078 149.65283203125 686.777282714844 546.935791015625 299.839385986328 467.219329833984 458.160858154297 310.310363769531 489.485412597656 267.427886962891 614.77001953125 214.327499389648 625.021545410156 88.1042785644531 306.532562255859 373.395660400391 419.853546142578 486.1669921875 718.526977539062 274.193023681641 456.379150390625 217.392761230469 321.834655761719 969.953796386719 239.520370483398 218.781784057617 593.933654785156 410.422729492188 512.780029296875 272.025634765625 649.821838378906

ENSG00000158477.6 6.77342510223389 5.40664863586426 7.655930519104 11.0459403991699 8.47092819213867 53.7826614379883 4.01888227462769 7.72466087341309 0.525384902954102 8.67663192749023 15.6281232833862 3.22231388092041 88.0330810546875 2.27209424972534 2.82473373413086 0.478466987609863 0.218733981251717 11.815616607666 9.90428161621094 26.0972499847412 9.41576671600342 0.502455234527588 53.9850692749023 27.9176597595215 0.116059683263302 0.913937211036682 0.183072999119759 0.627811372280121 48.466926574707 4.10174512863159 4.0123348236084 22.8446178436279 37.9851264953613 29.824254989624 0.495361417531967 20.2806053161621 17.8324565887451 5.5851263999939 18.1692905426025 0.180573537945747 12.7259101867676 27.5488891601562 17.161075592041 8.3856725692749 0.210267946124077 7.48272514343262 0.288053780794144 4.29080724716187 5.53664398193359 0.380260288715363 4.25553798675537 1.43119966983795 0.449022948741913 0.527172863483429 2.65948629379272 0.676811635494232 0.508168697357178 82.7516937255859 7.4412088394165 93.5038604736328 18.9258918762207 0.104737438261509 2.78663229942322 10.0529642105103 40.1547241210938 7.14749383926392 0.780560255050659 0.689886510372162 19.6303691864014 41.0147514343262 0.171346664428711 0.483807772397995 13.2416725158691 3.21279454231262 0.292231231927872 7.09672832489014 0.942525804042816 22.1589603424072 0.101275220513344 1.78016209602356 3.98685956001282 7.35370063781738 0.888823866844177 3.53485655784607 7.77340698242188 0.48334476351738 2.03423929214478 3.99609994888306 3.58042049407959 3.14338517189026 78.2482757568359 8.86631298065186 0.10365030169487 21.1302661895752 7.21386861801147 41.7720375061035 2.8306896686554 24.373119354248 85.8109436035156 49.3608283996582 0.429921805858612 41.1725883483887 28.1886825561523 11.1374502182007 0.9918212890625 34.2291412353516 8.69160747528076 0.405584514141083 97.0010604858398 2.15452837944031 35.6440010070801 1.33356761932373 62.8859481811523 3.37903642654419 20.6333484649658 19.0501232147217 34.4390029907227 0.11917270720005 1.55487656593323 21.2474746704102 93.9684982299805 1.06611287593842 1.80124473571777 3.00826454162598 0.187133029103279 1.46393322944641 3.02935743331909 4.23104286193848 9.12075138092041 45.6966209411621 3.78096413612366 1.5726912021637 0.186767399311066 5.77689456939697 6.52695178985596 21.9257946014404 2.06777143478394 0.382727712392807 0.826135873794556 3.38535141944885 0.405620336532593 5.44314384460449 0.792016267776489 6.24968385696411 1.57081317901611 80.1593399047852 2.73710155487061 41.1308860778809 4.7767219543457 0.898346602916718 2.50215697288513 1.21507704257965 23.2388763427734 114.395767211914 0.802165031433105 1.09798848628998 0.429524928331375 1.50252914428711 5.63232803344727 65.4981460571289 37.7760887145996 11.5053434371948 0.528607726097107 35.9155464172363 41.233699798584 56.4064407348633 8.41051197052002 3.90846800804138 0.281003624200821 7.44459295272827 8.77348709106445 46.128231048584 0.677397668361664 0 0.125589236617088 38.3622627258301 0.339114516973495 80.0446548461914 0.571135938167572 0.908463537693024 1.44682967662811 8.32762050628662 1.56231212615967 0.82575786113739 0.63900351524353 0.989314913749695 8.44008350372314 2.23956298828125 0.599958479404449 2.44691252708435 7.19209337234497 0.417646527290344 14.4807748794556 1.38441216945648 37.7141075134277 1.77542388439178 103.529144287109 34.5815887451172 24.4224662780762 0.416778653860092 27.4409713745117 16.6755046844482 0.0814203172922134 64.3343963623047 96.4472732543945 11.7647752761841 0.158629894256592 31.1172313690186 8.00093746185303 5.78641557693481 0.309214860200882 6.338791847229 2.47162008285522 4.7010612487793 3.21287679672241 2.74579882621765 24.1380805969238 19.7865962982178 4.17803144454956 42.7993316650391 3.30944108963013 1.98120331764221 4.05932521820068 2.44163846969604 7.6159815788269 49.0408897399902 0.501956701278687 0.235916450619698 3.71229958534241 2.14604520797729 18.5974292755127 1.14509105682373 14.5240449905396 1.20115089416504 9.06484317779541 3.27697801589966 7.65346670150757 0.35219606757164 18.582447052002 0.382264822721481 4.70826244354248 5.46167087554932 1.38710784912109 0.940033257007599 2.70166039466858 1.46999323368073 60.1621246337891 2.78815197944641 0.874423325061798 8.28835964202881 16.3849105834961 3.03881645202637 0.0355317853391171 2.90739774703979 2.78058648109436 12.7122993469238 14.0219049453735

ENSG00000158485.9 2.20370984077454 3.26459956169128 5.71017456054688 1.59009563922882 1.63174951076508 2.79088926315308 0 1.95613014698029 0.473637282848358 5.06640195846558 1.90087485313416 0.427196085453033 8.30767154693604 0.449189752340317 0.967972636222839 0.410800576210022 0.109549932181835 5.81009578704834 6.62064838409424 2.64941596984863 11.6386003494263 0.30197736620903 6.66985654830933 0.954472124576569 0 0.274639785289764 0.595982432365417 0.113195054233074 5.13052082061768 1.794264793396 1.80164110660553 9.04877090454102 5.0910177230835 2.06734991073608 0.124047502875328 15.9937715530396 30.2102661132812 7.15032625198364 1.52227818965912 0.203485026955605 2.30534386634827 6.09029579162598 7.91410779953003 1.27226710319519 0.0902655646204948 5.7135853767395 0.216401666402817 0.36778050661087 3.13421773910522 0.317413628101349 0.409466952085495 0.333681285381317 0.0843326300382614 0.352036416530609 0.630931794643402 0.532670021057129 0.381764024496078 10.707010269165 2.10818433761597 7.12410449981689 3.67224025726318 1.02289807796478 1.20640635490417 0.114429302513599 6.41531944274902 1.68457615375519 0.502627968788147 0.0431900322437286 5.42347764968872 3.49260234832764 0.257449865341187 0.528673112392426 1.81105518341064 2.74885225296021 0.109770014882088 3.28089165687561 0.0480052083730698 4.38211965560913 0.0760834589600563 0.543300330638885 0.499190986156464 3.53913307189941 1.19849503040314 0.194310501217842 2.83142137527466 0.798852801322937 0.670930862426758 0 0.901971161365509 0.407152056694031 10.298734664917 1.37561190128326 0.031147101894021 6.117995262146 0.162991002202034 8.64566612243652 1.19087827205658 2.61856508255005 4.74648189544678 5.10130214691162 0.193788424134254 2.4620213508606 3.40171504020691 2.84991526603699 0.203211858868599 11.4457559585571 1.02531063556671 0.0870563387870789 12.6999492645264 0.249015241861343 2.69032621383667 1.78106427192688 4.17978572845459 0.692322611808777 10.5397272109985 2.13908410072327 1.4068067073822 0.0895290374755859 0.535382866859436 1.71330153942108 15.4064445495605 0.237310245633125 2.56394505500793 1.05258989334106 0 0.458244323730469 0.650233805179596 0.454084247350693 0.291574656963348 2.5084822177887 0.436994761228561 1.91289079189301 0.240531176328659 0.162456825375557 3.78651571273804 3.31990814208984 1.40891814231873 1.3619647026062 0.195991024374962 2.06004118919373 0.406298816204071 0.548148274421692 0.0939482823014259 3.62982726097107 5.96040630340576 2.76988887786865 0.404510140419006 3.31940579414368 3.46647453308105 0.0710407197475433 0.263405382633209 0.107392005622387 7.59893369674683 10.3253345489502 0.12298571318388 2.58219790458679 1.04251277446747 1.15631294250488 1.47176051139832 4.69106864929199 8.28920650482178 1.48620247840881 0.044124323874712 4.99243307113647 6.75546884536743 15.3667736053467 3.55493092536926 0.734063506126404 0.487165927886963 1.0885283946991 1.84394466876984 2.36950707435608 0.0782920122146606 0.0445078685879707 1.32089281082153 6.35188293457031 0.382141977548599 8.47536277770996 0.51488208770752 0.113747887313366 0.395249933004379 3.31796407699585 2.46475720405579 0.510880053043365 0.900101661682129 0.439179748296738 1.55569863319397 0.454724818468094 0.0751202404499054 1.29759109020233 2.8437328338623 0.337894111871719 3.48334288597107 0.575344443321228 17.1993732452393 0.275957614183426 11.3979806900024 10.907901763916 2.54087591171265 0.300582528114319 3.37700009346008 4.85916996002197 0.244669511914253 28.6324100494385 3.88567686080933 2.09568881988525 1.57306277751923 8.29006671905518 1.84002220630646 5.5051703453064 0.132742315530777 1.39376938343048 1.27913975715637 0.863302767276764 3.99506974220276 0.162852123379707 5.98665142059326 4.8138575553894 0.72864180803299 2.83156681060791 0.6215580701828 6.79076910018921 0.250650942325592 3.16257190704346 1.45887887477875 1.26110172271729 0.471371531486511 0.56714653968811 0.65620744228363 1.94293904304504 3.8461902141571 0.637225747108459 1.87945020198822 0.180474013090134 2.1924889087677 1.68331241607666 3.3410427570343 0.132294446229935 2.14271998405457 0.287178158760071 0.325591087341309 2.95600008964539 0.146770566701889 0.403545290231705 0.998181045055389 0.752958357334137 28.9123210906982 1.2772022485733 0.547428667545319 3.0291895866394 0.65300989151001 1.38766002655029 0.0533868260681629 2.73839449882507 0.865236401557922 7.00345897674561 5.16572093963623

ENSG00000158481.11 9.1144380569458 7.97090339660645 11.2629108428955 8.16361045837402 8.56583213806152 21.3039569854736 1.49420440196991 8.74737739562988 3.29978346824646 15.5563554763794 12.6453561782837 1.31507921218872 37.9109420776367 1.65934145450592 3.61919522285461 2.24634194374084 0.638977646827698 15.644455909729 22.2606410980225 13.805025100708 20.8692646026611 0.733899772167206 43.2057609558105 12.2576055526733 0.0565066486597061 2.50965237617493 1.49299108982086 5.68538904190063 26.3116588592529 5.3844256401062 9.26232433319092 27.8371543884277 25.0711402893066 16.2950553894043 1.36668360233307 33.8904724121094 25.158618927002 7.33718204498291 8.08982086181641 2.57156848907471 10.9857034683228 24.8347969055176 38.9142456054688 3.90989398956299 1.09686803817749 17.8723754882812 0.490862339735031 4.90024757385254 11.3046770095825 4.38163471221924 2.60265588760376 3.27984285354614 0.532882273197174 3.85001540184021 2.65782785415649 4.04842758178711 2.78341865539551 24.3950538635254 9.30298328399658 50.1169548034668 11.8995943069458 0.968888223171234 7.58856582641602 3.59674835205078 44.2160491943359 16.5126628875732 1.14010739326477 1.25958383083344 14.8779993057251 16.1462879180908 0.291985869407654 5.62118148803711 13.0030860900879 8.34256744384766 0.586906254291534 6.71110963821411 0.676672697067261 23.2584571838379 0.517738699913025 5.93159961700439 1.72052490711212 14.6430253982544 10.286057472229 2.77044820785522 15.0240459442139 2.18855810165405 4.71054220199585 2.96070218086243 5.19834470748901 3.56222581863403 33.3722114562988 8.516282081604 0.908366739749908 20.3762054443359 5.07032632827759 41.6144790649414 8.86454486846924 20.6685371398926 46.1417579650879 45.4902610778809 0.879137635231018 24.7822265625 21.2852172851562 11.2239770889282 4.7740626335144 18.3219833374023 7.08203268051147 1.77722346782684 42.2383460998535 2.01728367805481 16.5637855529785 4.59907865524292 33.7652473449707 7.17892694473267 34.4246292114258 11.6845731735229 12.6813135147095 0.217583626508713 2.03452277183533 13.6019430160522 83.4949493408203 2.8260178565979 10.4130115509033 2.04649877548218 0.455552667379379 3.23708915710449 4.56523036956787 4.10866546630859 4.33674144744873 13.8447237014771 6.19014310836792 8.31336975097656 1.79266965389252 1.57928419113159 4.02523183822632 13.4059867858887 13.6964521408081 1.91244530677795 6.54145669937134 6.67539405822754 2.27109694480896 7.14044332504272 2.07013583183289 17.988431930542 8.28307342529297 23.585277557373 3.08033776283264 21.0696201324463 7.45163679122925 2.34805679321289 3.70127558708191 3.6539478302002 19.7769012451172 75.0896072387695 1.76945173740387 9.20414161682129 4.39163064956665 7.81504154205322 19.8961696624756 36.678596496582 49.6406517028809 9.31499767303467 1.45840883255005 39.3383255004883 15.0427331924438 49.1533889770508 9.03058528900146 3.09227442741394 1.5786212682724 7.07890033721924 21.5486831665039 26.1298542022705 0.228328973054886 0.865344643592834 0.978341698646545 40.3804969787598 2.88936638832092 36.9259185791016 2.08554148674011 1.71394681930542 4.61079025268555 8.39401435852051 3.76522660255432 3.19268202781677 2.80003786087036 4.17085409164429 7.42418527603149 3.09434676170349 1.67960500717163 15.5575294494629 26.488597869873 1.36082684993744 22.4013214111328 8.30355739593506 40.4189796447754 1.49036347866058 41.8045349121094 38.6527366638184 7.92713785171509 1.36361837387085 29.8462047576904 22.1635398864746 1.30817210674286 60.6787605285645 33.3852424621582 12.2236356735229 1.99261271953583 41.8967094421387 15.8004693984985 7.44019079208374 1.12911915779114 12.7426891326904 8.42360305786133 5.03543138504028 15.2805452346802 4.01058864593506 27.939754486084 13.9361696243286 3.75960445404053 22.89035987854 4.12892150878906 6.87277364730835 11.6146574020386 4.14021444320679 11.5673017501831 7.1805591583252 1.52744090557098 3.17019081115723 4.62489032745361 17.0392112731934 19.5057601928711 3.34510207176208 19.2856502532959 2.76323318481445 12.6590709686279 10.9638185501099 10.5745983123779 1.47897791862488 32.191707611084 1.76809680461884 2.35947823524475 26.2484302520752 1.31265044212341 2.5499279499054 4.33426141738892 6.19734573364258 57.6214790344238 7.35027599334717 5.58777856826782 11.6481313705444 9.64907455444336 4.65615510940552 0.467088252305984 4.94383096694946 3.72495293617249 14.5890865325928 29.3180313110352

ENSG00000158473.6 10.2212009429932 7.8033766746521 4.04799890518188 4.15355634689331 10.1842031478882 3.63282656669617 3.26297616958618 5.2888445854187 7.77149868011475 4.32292032241821 4.13094472885132 6.26004314422607 2.44441437721252 1.83563709259033 1.70582485198975 3.65638470649719 1.98969507217407 6.99506616592407 4.92672443389893 4.79011583328247 6.21322679519653 6.07674217224121 17.4195575714111 2.02178978919983 0.575851142406464 1.90455889701843 16.3928756713867 6.19106101989746 4.05189752578735 2.27022838592529 4.3548583984375 6.21741771697998 2.53007793426514 3.56338286399841 2.66264414787292 9.02914619445801 6.47253751754761 4.67326068878174 6.15803098678589 9.91142845153809 1.56735229492188 10.5119552612305 8.75715446472168 2.05832815170288 12.2119932174683 5.64891767501831 3.43908643722534 5.80016946792603 4.55231285095215 1.80811655521393 1.75521779060364 2.15789437294006 0.809357404708862 3.21508622169495 1.45411169528961 1.01943027973175 3.07292079925537 2.65180277824402 5.28000211715698 6.16600227355957 6.99617624282837 6.31728363037109 6.48111534118652 1.79490959644318 4.1060962677002 3.82450389862061 3.94204926490784 1.44407677650452 8.55620098114014 3.84449791908264 1.35495436191559 8.694993019104 1.90631020069122 6.72505044937134 0.441784113645554 3.76663613319397 1.56048882007599 5.6137170791626 1.64881265163422 3.41573023796082 2.00906085968018 6.14644384384155 5.4489688873291 2.04530572891235 7.37781381607056 4.22683191299438 5.53897666931152 4.41805601119995 6.44243717193604 3.12601709365845 4.735182762146 3.22766041755676 0.578564763069153 4.1019139289856 1.56425786018372 6.01870489120483 9.60676574707031 3.09750175476074 5.18315172195435 13.6670017242432 0.719933032989502 2.56642961502075 8.42502021789551 5.24853801727295 1.17435336112976 4.06316709518433 2.00477719306946 0.485127568244934 3.58165502548218 2.26136350631714 6.97407102584839 5.41056108474731 4.62926864624023 3.61986684799194 8.7649507522583 3.65804934501648 2.0113537311554 0.0277170427143574 2.68209910392761 4.13724660873413 18.6177196502686 5.43664836883545 7.97292470932007 3.37369608879089 5.37752962112427 5.61791706085205 2.86299109458923 1.90321910381317 6.51733446121216 3.88297009468079 1.81672441959381 4.31962394714355 1.61341643333435 2.94582390785217 0.927685379981995 2.88574910163879 8.54471397399902 2.71727991104126 5.33951568603516 4.5509467124939 3.77354764938354 6.74726009368896 3.2575409412384 7.57308483123779 5.32526350021362 1.96300268173218 2.21241736412048 6.28677272796631 2.93234086036682 8.5334005355835 3.21739745140076 1.56261801719666 6.55135726928711 16.2476444244385 3.53334188461304 6.30650091171265 13.709132194519 2.91497778892517 10.4512023925781 9.93182277679443 9.48134613037109 2.32476282119751 0.669356048107147 4.47367763519287 4.3615665435791 10.1163463592529 1.12466096878052 2.67657852172852 1.66907846927643 1.65011072158813 9.64391899108887 8.2068042755127 1.30886316299438 4.51953411102295 4.59561109542847 7.86584711074829 3.73322010040283 8.0309362411499 2.44414710998535 3.09891033172607 4.58866214752197 3.11872577667236 4.46933555603027 3.47956228256226 3.1209921836853 7.59309101104736 1.25514245033264 2.42136430740356 0.697688043117523 14.9081869125366 4.73806285858154 4.93150568008423 3.18542313575745 8.68674087524414 5.91898059844971 3.094571352005 8.4448823928833 5.22882080078125 2.14034557342529 3.59818530082703 4.27756881713867 11.2959585189819 4.27967977523804 20.1395244598389 18.3273868560791 11.6219549179077 5.90303182601929 7.01403427124023 5.20277976989746 5.23769807815552 1.56162238121033 5.81488513946533 6.08059740066528 5.68550872802734 6.92031669616699 5.61308622360229 7.2751350402832 6.97661066055298 1.24935615062714 3.92170715332031 4.29752397537231 16.3002986907959 3.31086182594299 3.60305786132812 5.50448751449585 2.85565066337585 4.3779182434082 3.81889343261719 2.53941774368286 11.1343183517456 4.16038656234741 1.55848801136017 12.0680780410767 2.8774311542511 6.17060518264771 6.12330150604248 2.06868815422058 2.33453011512756 9.82977104187012 4.18849229812622 2.9689838886261 9.15139770507812 2.41731691360474 4.59126758575439 2.80181813240051 4.63104486465454 2.56349921226501 4.01731967926025 4.16913223266602 6.5854287147522 5.37754964828491 6.12528991699219 1.22306299209595 4.44071006774902 1.10207796096802 6.22296237945557 5.283766746521

ENSG00000158488.14 2.30883455276489 2.59606122970581 3.19661116600037 3.14653825759888 4.21573543548584 13.1139287948608 0.764736950397491 3.83989334106445 0.499866873025894 5.98600292205811 3.39864277839661 1.03696358203888 23.2598781585693 1.36530864238739 1.46164238452911 0.975488126277924 0.184986710548401 7.3582239151001 9.03860855102539 6.97916030883789 14.557469367981 0.334635645151138 18.7757205963135 6.23484134674072 0 0.973892867565155 0.261271685361862 0.74067485332489 16.376537322998 1.38317286968231 4.73890972137451 12.6928834915161 9.0350456237793 7.78385305404663 0.759319365024567 15.0260562896729 15.5310478210449 9.90452480316162 4.7554783821106 1.11671996116638 5.75335693359375 19.6239986419678 20.4660320281982 2.65079689025879 0 9.044997215271 0.456771582365036 1.21924090385437 4.748375415802 1.36676609516144 1.03714275360107 1.50255024433136 0.0534017346799374 1.28178489208221 1.24296152591705 0.889248788356781 0.241743430495262 28.5182590484619 5.59483289718628 31.4327239990234 10.0765743255615 0.249125823378563 3.16806125640869 1.59411489963531 7.62262296676636 7.06701946258545 0.671919822692871 0.328189343214035 6.96267986297607 16.21848487854 0.271707236766815 0.899695158004761 5.45553779602051 1.23119044303894 0.139018759131432 6.27593374252319 0.0911946371197701 8.22894954681396 0.12044533342123 1.45552289485931 1.16382801532745 8.5816822052002 5.44254016876221 0.943327665328979 4.89321517944336 0.735789775848389 0.708086311817169 0.6612189412117 1.65226376056671 1.34066331386566 27.377140045166 4.67629814147949 0.216955199837685 14.9232406616211 3.30273222923279 16.4240169525146 2.69320273399353 5.71689510345459 24.9191761016846 16.8968734741211 0.327232360839844 11.3472929000854 9.61741924285889 7.31112384796143 1.07232749462128 17.9261417388916 2.39199137687683 0.49613818526268 27.2930774688721 0.367927670478821 4.17947006225586 2.57283663749695 12.3390960693359 1.94843411445618 21.3904514312744 3.16056227684021 2.57530450820923 0.0850383564829826 0.246559351682663 3.90567445755005 27.7120800018311 0.262974858283997 8.35231590270996 0.666528761386871 0.296739935874939 0.909208357334137 1.46398532390594 0.619313895702362 0.683142364025116 11.4777574539185 1.66030240058899 4.2751579284668 0.304621875286102 0.92584902048111 3.3809654712677 3.39595317840576 2.74512505531311 0.632451236248016 0.620534479618073 5.76545524597168 0.38591930270195 1.0551917552948 1.46743559837341 7.34522676467896 2.25444340705872 13.3132648468018 2.13455748558044 11.7667350769043 3.9263870716095 0.989668548107147 0.576202690601349 1.1560605764389 9.53234100341797 33.2536354064941 0.57629668712616 3.22481727600098 4.84108304977417 1.6561872959137 5.79562091827393 20.7297096252441 18.792049407959 5.15130710601807 0.307348042726517 15.3619136810303 11.99232006073 22.1047859191895 6.22743892669678 2.68567943572998 0.699236750602722 3.08990263938904 6.75738477706909 14.769926071167 0.148729935288429 0.39457044005394 1.07540428638458 12.1413908004761 0.618400394916534 19.2510681152344 1.08679151535034 0.144056558609009 5.00566148757935 3.56921291351318 2.74989604949951 1.80237519741058 0.873952567577362 0.727340221405029 6.11962938308716 2.59149789810181 0 3.19538426399231 9.16734981536865 0.213963821530342 6.27789878845215 1.09297168254852 24.8696632385254 0.485399842262268 22.7290534973145 18.307746887207 6.13646936416626 0.444119900465012 8.3439884185791 4.61543989181519 0.34859573841095 20.1977405548096 19.1413764953613 6.86602973937988 0.845181286334991 23.5412254333496 7.0685920715332 4.52758073806763 0.378252327442169 4.30779695510864 2.69124507904053 2.68363785743713 7.43878126144409 0.446863889694214 9.72109413146973 12.628532409668 0.993776023387909 13.2589540481567 1.63994836807251 4.9480619430542 3.06856822967529 1.22827804088593 3.26217603683472 8.78420639038086 1.0148503780365 1.30185604095459 2.70093536376953 5.91601800918579 5.92739295959473 1.71491253376007 4.14337873458862 0.399983823299408 7.11000442504883 4.42356634140015 4.03447580337524 0.753952205181122 11.1218872070312 0.848628997802734 1.03086602687836 7.09615421295166 0.334580898284912 1.69292557239532 1.72767317295074 2.06610679626465 41.3351745605469 2.68508124351501 0.987943589687347 4.56097269058228 2.64642405509949 1.81409919261932 0 3.17904853820801 1.33059358596802 8.81196975708008 9.36425590515137

ENSG00000010610.8 215.222137451172 190.581985473633 61.6394309997559 49.3985061645508 192.911026000977 78.3187484741211 32.1985092163086 69.1776657104492 27.6322860717773 112.124908447266 71.3945999145508 87.2734146118164 76.7503051757812 51.1632347106934 46.2806854248047 77.5884704589844 8.69204521179199 200.529434204102 132.820358276367 57.2252998352051 107.881050109863 86.9081115722656 66.49658203125 72.9071655273438 5.76791954040527 45.0250434875488 62.1673240661621 45.1474914550781 65.6214904785156 84.2968673706055 86.6567916870117 138.448013305664 74.1581192016602 75.3255462646484 24.3037548065186 103.283821105957 104.631843566895 112.42008972168 154.484161376953 102.238906860352 36.9982109069824 225.901031494141 115.660278320312 24.2599639892578 62.4931449890137 59.7616920471191 54.1667594909668 136.483337402344 43.0464096069336 95.650032043457 29.5291213989258 23.0671825408936 14.6702070236206 77.1247253417969 77.5548553466797 42.6125984191895 36.0230941772461 175.378265380859 65.1579208374023 120.198852539062 119.645210266113 54.0561256408691 92.6456680297852 28.061897277832 76.3320693969727 55.6559410095215 36.0722999572754 30.7232093811035 83.8304748535156 42.3349456787109 20.5313911437988 153.256744384766 31.6029109954834 86.181282043457 30.8730869293213 56.2576637268066 39.2627182006836 110.817283630371 27.0957069396973 113.687507629395 35.6314888000488 131.425933837891 99.935676574707 51.3184585571289 57.240837097168 81.5247268676758 98.0264739990234 71.3818054199219 160.963104248047 22.5554981231689 60.1093101501465 99.0375137329102 7.006263256073 91.6680297851562 16.4712314605713 60.0151062011719 161.793228149414 42.9457473754883 118.628051757812 130.282852172852 26.7208576202393 63.557071685791 90.0098190307617 97.8243408203125 49.9889831542969 61.3635330200195 38.682731628418 4.98175811767578 122.775177001953 32.8705024719238 196.524368286133 99.413215637207 132.548736572266 46.1271438598633 88.7944107055664 91.5580062866211 49.1509437561035 4.85074281692505 28.5036964416504 24.7540874481201 145.737045288086 3.81033658981323 154.360061645508 54.0710945129395 78.3567810058594 39.2691307067871 19.6162624359131 25.5488414764404 43.2218246459961 89.8275299072266 21.8524627685547 125.270820617676 18.6696395874023 28.1509189605713 50.6380805969238 77.9664840698242 140.367416381836 154.530563354492 97.5191116333008 87.2834243774414 61.031925201416 109.245742797852 39.0819129943848 92.4846496582031 132.453369140625 128.925476074219 29.304256439209 142.285247802734 174.70832824707 34.9224128723145 64.4986343383789 82.7511901855469 119.324821472168 211.724365234375 53.2027626037598 91.6763458251953 125.55534362793 55.8535308837891 191.470733642578 296.341857910156 91.9492874145508 42.6426620483398 10.3954524993896 56.3330535888672 81.2665557861328 125.436111450195 67.4832763671875 23.6844787597656 37.0221672058105 25.8439388275146 155.161437988281 156.667770385742 16.7531757354736 48.2970542907715 71.5477066040039 93.1729965209961 68.8864517211914 82.3022918701172 76.6896820068359 41.9287452697754 29.4154167175293 51.653018951416 108.570014953613 83.9724807739258 79.0518264770508 61.1020774841309 15.9422616958618 131.57421875 6.08223247528076 141.574172973633 117.689002990723 86.6585998535156 68.9834518432617 112.269676208496 130.927841186523 36.3896179199219 124.156883239746 159.619995117188 34.5155143737793 14.333911895752 74.8426513671875 122.636711120605 45.8573150634766 452.428558349609 81.3658828735352 66.396842956543 61.6369323730469 101.922554016113 68.1053161621094 84.6021881103516 30.8289165496826 82.7679138183594 63.1001281738281 92.0675277709961 154.849975585938 70.9179611206055 182.890533447266 125.755561828613 25.2838554382324 101.339965820312 62.3392333984375 140.38703918457 108.414596557617 186.726852416992 113.041511535645 121.760848999023 67.4445953369141 94.8609161376953 99.3957138061523 134.46240234375 118.08812713623 28.2991218566895 164.366470336914 55.3594589233398 130.540130615234 70.5798416137695 57.23388671875 11.6577434539795 84.2244720458984 20.8625545501709 67.7790069580078 95.0124282836914 55.6648864746094 45.5097885131836 213.776245117188 48.5271263122559 97.5616149902344 40.8822326660156 90.0965728759766 71.8578262329102 24.5079612731934 108.443664550781 23.5302753448486 166.050765991211 35.0502052307129 158.912506103516 65.9933700561523

ENSG00000153563.14 60.969783782959 32.4373931884766 13.8508377075195 19.2465877532959 36.5810050964355 9.21605587005615 14.3293952941895 35.5930633544922 20.9695930480957 18.9742698669434 6.88204765319824 6.28627061843872 2.54337596893311 3.75902843475342 12.4050559997559 22.5110855102539 2.13070702552795 36.8715438842773 35.7208709716797 24.1649475097656 21.138988494873 14.1713943481445 13.2023811340332 2.91189575195312 2.18671703338623 6.76842641830444 5.96594285964966 12.1233034133911 5.76775217056274 5.24066066741943 12.9808788299561 13.8650569915771 6.84009265899658 7.07808065414429 3.60314607620239 11.5167684555054 50.3097267150879 38.6944541931152 25.7509670257568 17.1066856384277 2.48136639595032 50.544361114502 11.2700891494751 7.45190000534058 93.5221099853516 14.415638923645 12.2945213317871 9.26815414428711 8.81557559967041 4.53270816802979 9.40685367584229 5.87798690795898 1.3272989988327 23.1085643768311 11.329514503479 2.91846537590027 9.79438972473145 10.0184869766235 10.0659675598145 13.4385614395142 34.4829292297363 10.4408483505249 3.06468987464905 1.69849109649658 15.5231695175171 10.9663763046265 10.5691566467285 1.95638716220856 1.73969912528992 9.71277904510498 10.1958017349243 50.6218757629395 3.416499376297 17.0121116638184 9.77599620819092 15.9530420303345 3.47367334365845 4.54383134841919 4.13271999359131 6.33699035644531 11.7589111328125 84.7623291015625 9.80609893798828 20.4876232147217 3.12486791610718 26.5678405761719 3.60574531555176 5.110755443573 51.3924674987793 11.6908884048462 4.00561237335205 5.86427164077759 4.81850147247314 7.86553192138672 7.60202312469482 8.23433780670166 27.0370292663574 5.11602020263672 8.61334323883057 18.3021984100342 4.14106750488281 2.76354122161865 11.5685653686523 20.1690559387207 2.78227996826172 23.9897880554199 6.29745006561279 2.07195997238159 4.95866584777832 4.65207624435425 39.4096527099609 38.3515892028809 11.0282030105591 4.577064037323 18.4521617889404 8.95433521270752 15.9654655456543 1.44345235824585 1.0089156627655 4.66961908340454 41.4847030639648 4.53209352493286 9.66735649108887 8.65168952941895 12.2084932327271 5.00752353668213 3.74412298202515 6.20816707611084 4.81290769577026 3.63433074951172 3.25916528701782 31.553731918335 9.84893608093262 3.9555983543396 10.9591159820557 2.05869150161743 6.6149845123291 56.8832054138184 60.4897117614746 41.0921669006348 18.2872200012207 3.65289425849915 3.45015645027161 17.5386447906494 44.9931602478027 1.81912231445312 4.21847105026245 24.1230220794678 14.5438957214355 11.6173276901245 18.4396286010742 6.01885843276978 33.8043975830078 4.0015025138855 15.9384622573853 37.667121887207 37.6373023986816 5.61189985275269 7.8389778137207 8.61664390563965 15.6428823471069 5.23973226547241 2.46450877189636 5.80718660354614 11.2152633666992 29.6050815582275 11.2060451507568 10.6453351974487 7.4056191444397 5.605064868927 13.0122699737549 5.00276374816895 3.42619109153748 8.90151214599609 13.5215225219727 12.0215148925781 7.46512317657471 3.3720235824585 24.2780227661133 3.03471159934998 11.9105215072632 10.174111366272 28.181978225708 16.8377704620361 21.5377960205078 10.6081972122192 1.80967593193054 47.5494003295898 0.25953072309494 45.992359161377 20.8204860687256 44.2678070068359 16.2311458587646 60.0229721069336 36.5490036010742 27.1659564971924 12.0731229782104 7.38399505615234 8.54022407531738 6.28853607177734 19.3689098358154 13.9981641769409 17.8217735290527 198.633895874023 12.4593505859375 15.739857673645 10.0277147293091 6.52282047271729 11.6310272216797 54.1467323303223 4.20390224456787 9.51593685150146 6.89024829864502 5.7844352722168 16.121114730835 43.6561584472656 8.46777248382568 21.6064186096191 11.8552751541138 1.74486720561981 4.81177282333374 30.53173828125 13.5347366333008 83.3310699462891 17.6233310699463 5.69860506057739 11.399694442749 51.8973579406738 7.33662557601929 24.5017967224121 9.4101734161377 4.64767980575562 14.3092164993286 11.638934135437 46.4687652587891 9.93502998352051 7.60576725006104 6.9828634262085 20.6880168914795 5.97747755050659 15.3278427124023 14.8192749023438 5.73556184768677 21.2808647155762 10.933295249939 17.0919876098633 3.09916067123413 8.49174308776855 7.18692207336426 9.89696407318115 6.59272241592407 45.3299713134766 22.0718669891357 6.83279895782471 7.62978172302246 15.5196857452393 10.177396774292

ENSG00000172116.20 20.8403415679932 10.2273302078247 3.65410137176514 4.79723834991455 12.1630115509033 2.18361496925354 5.34073877334595 17.2990016937256 8.56625366210938 7.6295337677002 3.68201494216919 18.1168727874756 0.976283371448517 1.21020984649658 4.51370334625244 13.0661687850952 0.710546433925629 9.76624202728271 8.9550609588623 7.61394357681274 6.61730432510376 4.18094491958618 4.0453724861145 0.676722228527069 1.13104236125946 2.34313106536865 2.76194024085999 6.5229868888855 2.4683358669281 1.86825859546661 5.83412647247314 5.62905883789062 4.56205749511719 2.508948802948 1.29970371723175 5.55451440811157 20.9633979797363 9.47614860534668 7.13935422897339 6.39602327346802 1.3870040178299 19.8854370117188 6.03747892379761 2.18073678016663 38.2355041503906 9.17269515991211 4.15679311752319 3.2705397605896 4.66931581497192 1.75786507129669 6.60027170181274 1.80356156826019 0.284011840820312 7.24517059326172 3.20035433769226 0.869771420955658 3.28564882278442 4.10667562484741 2.49957394599915 3.72639608383179 12.1910448074341 3.63134741783142 1.83228123188019 0.542372822761536 7.28266477584839 6.51372480392456 3.00929641723633 0.549491167068481 0.72281414270401 4.29829931259155 3.59656429290771 26.5088157653809 1.74262690544128 5.87058925628662 6.28453969955444 4.85962295532227 1.2574325799942 1.58322489261627 1.11033272743225 2.50215911865234 4.70383548736572 16.7963428497314 3.25461959838867 4.12023735046387 1.01535856723785 10.8157205581665 1.6737277507782 1.36757743358612 14.9470205307007 5.42381620407104 2.1876003742218 1.57133448123932 0.885787546634674 1.92357468605042 2.80556130409241 4.26692342758179 12.1463556289673 1.930459856987 3.42305064201355 6.38741683959961 1.98206865787506 0.629733502864838 4.84062767028809 8.91421794891357 1.08991980552673 10.850133895874 3.25106811523438 1.04243433475494 1.69258606433868 1.95678699016571 12.3220987319946 30.1575832366943 3.48057556152344 2.82091736793518 8.3168773651123 2.28695750236511 8.34615707397461 0.804032206535339 0.327825397253036 1.79510617256165 17.6014099121094 1.90920805931091 3.36861658096313 3.05831694602966 3.3901686668396 2.42348313331604 2.81163334846497 12.6260795593262 2.07301163673401 1.21117699146271 1.07456910610199 7.915846824646 3.09019303321838 4.42902755737305 4.6686224937439 0.382244259119034 3.46066403388977 23.0131149291992 15.6945276260376 16.6733646392822 5.70131587982178 1.44400608539581 1.124960064888 8.26769542694092 17.6033458709717 0.899114668369293 1.5641131401062 11.9339590072632 3.69045066833496 2.87097525596619 3.88883924484253 2.08965039253235 7.88257217407227 1.64883649349213 7.01355075836182 9.0182991027832 8.3593578338623 1.89558064937592 2.8568902015686 2.60475087165833 4.02521324157715 1.87327992916107 0.776022434234619 1.76488053798676 3.11121821403503 17.0215930938721 3.57319474220276 3.41827845573425 2.62505078315735 2.83720970153809 3.69954991340637 1.7733166217804 10.7811117172241 3.53078246116638 5.036865234375 4.20093488693237 9.88259315490723 1.00150763988495 8.41310119628906 1.36204552650452 4.88072204589844 3.70974135398865 7.9932713508606 7.75599431991577 8.08353233337402 22.1984062194824 0.635056436061859 15.4501543045044 1.51792073249817 15.7534799575806 6.19119310379028 10.2956962585449 4.29136037826538 21.8603363037109 12.6419925689697 9.45421409606934 4.45302295684814 2.85279059410095 3.20613145828247 3.26182055473328 10.1753349304199 4.00995349884033 6.31723833084106 69.6845855712891 5.38836002349854 5.31890058517456 46.5911712646484 1.88823735713959 3.48853993415833 24.5944976806641 1.19211709499359 3.00507497787476 2.47044157981873 2.17320394515991 6.88738536834717 9.54703140258789 3.45786619186401 7.71995449066162 3.18795847892761 0.836493968963623 1.70561683177948 7.51879405975342 5.31490278244019 28.2127208709717 8.63213157653809 1.42917776107788 3.97454237937927 16.4473247528076 3.6218581199646 9.12665557861328 5.16734218597412 1.78835165500641 4.74063777923584 3.07272982597351 12.4803133010864 3.84231448173523 4.88451147079468 1.58412659168243 6.94889688491821 2.36413550376892 4.29744005203247 4.65561485290527 1.9332138299942 7.02172040939331 2.88851284980774 5.82290458679199 1.08446741104126 3.51751327514648 3.54382252693176 3.07306742668152 3.86078071594238 12.5961408615112 5.19404554367065 3.12287783622742 2.55313730239868 5.71780681610107 5.62840700149536

ENSG00000019582.13 6799.22509765625 7882.32177734375 2906.75146484375 3342.0576171875 5454.9189453125 3126.86547851562 1820.91564941406 1118.06018066406 1249.78662109375 2715.51879882812 3957.494140625 1972.73620605469 6934.03173828125 1567.25793457031 1668.26330566406 3276.61791992188 250.303863525391 6873.33203125 5907.8583984375 2958.60546875 6349.02490234375 2530.65991210938 9999.8076171875 3287.27734375 118.523574829102 1159.71557617188 3885.1552734375 2210.302734375 7729.44677734375 4149.609375 3047.78540039062 7133.26123046875 7667.2890625 3585.087890625 930.884521484375 4119.0224609375 4021.80712890625 5562.47509765625 4597.12841796875 2709.40454101562 4595.26806640625 11059.779296875 11239.4990234375 956.759582519531 2042.56909179688 1732.755859375 1879.70446777344 4396.82373046875 3236.96557617188 7769.80322265625 1974.28588867188 814.615600585938 1321.943359375 2794.2744140625 4165.12451171875 1099.82434082031 1195.47412109375 6630.2607421875 4950.2197265625 10116.91015625 4276.67333984375 1646.08178710938 2673.21069335938 1389.83410644531 2581.86254882812 2877.38940429688 1990.47863769531 545.013549804688 6187.73095703125 3497.33984375 746.916564941406 4790.90625 2108.88427734375 2967.95751953125 1465.35583496094 1633.06408691406 584.754211425781 11510.5107421875 735.488098144531 3201.68286132812 2175.72338867188 6104.32861328125 2584.55981445312 2207.013671875 3880.51879882812 5753.90283203125 3228.49340820312 1894.83178710938 8023.24755859375 1632.470703125 3385.07763671875 2695.5341796875 278.646331787109 2410.53198242188 318.135772705078 7648.771484375 5005.72412109375 1859.63415527344 9283.71875 4105.9541015625 1532.94458007812 4747.42431640625 5292.0078125 5699.05908203125 1164.66809082031 3006.3505859375 2580.23413085938 514.4189453125 7801.4150390625 628.5830078125 7947.53857421875 3611.99731445312 9041.8623046875 4274.6728515625 7848.12890625 1531.28393554688 4099.15185546875 181.455718994141 600.660339355469 1604.580078125 6962.40966796875 106.571594238281 2833.57885742188 2316.29028320312 2329.9931640625 1039.5322265625 573.6015625 2736.74682617188 3439.96044921875 1850.83081054688 929.673706054688 5282.21826171875 1532.41625976562 577.081970214844 1014.17333984375 2715.5517578125 3379.2490234375 5977.10986328125 5646.55712890625 7032.0615234375 2169.28564453125 2678.47631835938 1651.65551757812 7656.572265625 6898.3603515625 9306.0947265625 6042.03271484375 6183.1767578125 6130.80712890625 803.521423339844 2536.60888671875 2221.72290039062 6915.0126953125 8933.8388671875 1594.73620605469 3591.82690429688 2697.00317382812 2678.36767578125 6719.28369140625 8927.755859375 5437.87060546875 1067.50427246094 661.390075683594 6440.48583984375 3780.39916992188 8669.7646484375 2145.23266601562 613.853698730469 1448.4541015625 4911.72998046875 3353.68115234375 6108.720703125 403.116333007812 1466.72326660156 1966.18859863281 8443.1904296875 3327.39135742188 2925.38500976562 1935.70141601562 1212.36401367188 579.81689453125 4083.82788085938 3225.29174804688 2564.23876953125 4625.736328125 1312.28405761719 5599.244140625 6543.4755859375 204.780288696289 5055.99072265625 7238.06640625 3153.2080078125 5474.77197265625 5192.4462890625 6171.59814453125 4814.83203125 8168.734375 5692.60400390625 4624.0048828125 245.389205932617 4958.591796875 3011.25146484375 1748.00122070312 19709.47265625 8653.4482421875 5289.2373046875 1485.00280761719 10865.158203125 4872.65283203125 9151.7060546875 624.124084472656 3704.47705078125 6900.412109375 2273.17138671875 5637.53076171875 3308.71826171875 6842.458984375 5431.5927734375 715.388916015625 4954.978515625 4848.0595703125 2689.1162109375 2708.751953125 4485.26220703125 5972.14013671875 10376.4990234375 2098.61279296875 4425.35498046875 5530.1904296875 5261.94140625 3775.63256835938 604.424865722656 4762.44140625 1322.9326171875 3943.54663085938 4242.85693359375 9590.2021484375 321.717620849609 3878.11059570312 1677.55505371094 2660.38208007812 5364.2744140625 1017.22985839844 3214.74853515625 5974.23486328125 2863.53076171875 12260.0126953125 1635.53540039062 4865.23388671875 2254.1298828125 1052.19738769531 5454.41845703125 566.629333496094 6581.1826171875 1993.669921875 7545.54541015625 6185.533203125

ENSG00000118260.13 29.0824394226074 13.7157192230225 10.3689346313477 18.8951015472412 14.739426612854 25.8982810974121 9.88515758514404 25.4023170471191 11.0063161849976 17.0760936737061 17.0400810241699 17.9766998291016 13.3039627075195 19.3712215423584 16.2396602630615 20.0872631072998 9.51248073577881 16.0630397796631 23.2559871673584 21.1372756958008 13.607141494751 17.8402404785156 15.5202741622925 14.8280649185181 14.7946796417236 21.56689453125 12.6404180526733 7.00343418121338 12.3028345108032 24.6975517272949 17.848949432373 16.615442276001 4.78214311599731 18.2997035980225 10.1026487350464 9.8613109588623 13.007022857666 23.0992412567139 28.4622325897217 13.3506374359131 21.0346775054932 14.8722038269043 15.5323133468628 9.2380542755127 7.84454870223999 17.5815830230713 28.7850379943848 15.1985597610474 9.94770812988281 20.7360763549805 11.8073024749756 17.4742679595947 7.71597290039062 20.2334537506104 26.6317462921143 20.8829917907715 14.6558504104614 17.9976329803467 11.9855728149414 24.2182350158691 17.6212215423584 15.9459924697876 21.2880802154541 16.9214935302734 10.9428739547729 15.5847492218018 11.5376081466675 12.7862339019775 11.8956489562988 14.3154745101929 10.8274154663086 18.7311267852783 8.76379013061523 30.7983131408691 14.1134719848633 20.6734714508057 9.77756977081299 11.9175357818604 21.9755058288574 13.7667875289917 15.4910821914673 23.2470550537109 26.1417655944824 15.9759759902954 6.87525320053101 17.4579582214355 16.5304756164551 13.9708042144775 9.46151256561279 10.5587396621704 23.1602916717529 20.1892261505127 11.3719301223755 21.1285057067871 35.0260429382324 9.10999774932861 13.5529317855835 15.8409452438354 13.6167364120483 19.3522624969482 10.6628150939941 15.909782409668 11.58034324646 18.4395656585693 15.5482521057129 11.1614942550659 12.8269786834717 14.5605478286743 20.0996150970459 22.3542232513428 20.5177364349365 14.9367008209229 20.4216327667236 13.4263591766357 17.0452785491943 27.1133079528809 9.76356601715088 10.8653650283813 12.0121469497681 9.67671012878418 12.5350294113159 7.33837032318115 20.652946472168 13.305700302124 52.6342582702637 10.9713020324707 14.8416290283203 5.66400575637817 17.1449565887451 22.6529674530029 11.6055116653442 19.4429149627686 4.2227349281311 10.9518194198608 25.9203033447266 20.5472583770752 19.2794017791748 16.0637874603271 16.6757144927979 13.7134065628052 9.29352855682373 12.8927230834961 15.5672550201416 14.6924571990967 22.0184631347656 22.885684967041 15.558536529541 18.4815082550049 14.0201206207275 19.5616111755371 22.3034515380859 15.4583930969238 13.7687196731567 21.8461437225342 7.91259956359863 10.0007925033569 16.6640281677246 22.2374401092529 13.1468458175659 16.8731479644775 19.3319416046143 13.7267141342163 3.93106269836426 13.3592462539673 20.3068408966064 13.9467144012451 16.7323665618896 46.3437538146973 5.27589273452759 17.9753303527832 18.3023452758789 15.5348100662231 18.5583953857422 13.7664575576782 13.8559226989746 13.3776693344116 8.31413173675537 15.087456703186 21.2665119171143 19.7622833251953 48.1984062194824 14.1700420379639 17.5871143341064 17.2999095916748 9.40693283081055 19.8057994842529 12.5963954925537 15.8336839675903 23.9573173522949 15.4809732437134 25.468692779541 16.7165699005127 20.2217178344727 16.1456069946289 24.6833400726318 17.5292892456055 14.5456743240356 16.8061447143555 11.6177845001221 7.7950553894043 13.3644361495972 27.4927940368652 12.7524881362915 14.0329608917236 13.3202743530273 20.8948440551758 20.8208847045898 11.5753345489502 19.1763954162598 11.4048833847046 12.4884700775146 11.8548908233643 15.3657073974609 16.6253299713135 15.8747024536133 19.9379482269287 7.57480716705322 13.1790285110474 24.4079933166504 23.8511180877686 19.9374084472656 22.8094959259033 18.0276279449463 29.924898147583 10.9328784942627 13.5226984024048 16.2256183624268 9.33703804016113 12.6077919006348 22.6396179199219 13.2798290252686 23.9709358215332 18.0569515228271 15.7695875167847 21.6533393859863 12.1580209732056 12.2709522247314 11.7669162750244 20.8070678710938 3.84458804130554 14.8388395309448 17.5994243621826 18.4965400695801 17.0427017211914 25.5272655487061 17.3179626464844 12.5665006637573 10.8818426132202 15.4168224334717 13.1417779922485 19.9600067138672 13.9432649612427 13.7904443740845 15.1795501708984 7.34017419815063 11.7008657455444 16.9630947113037

ENSG00000164733.19 1787.91870117188 1666.62316894531 496.950500488281 307.611328125 1070.31005859375 535.685974121094 457.0966796875 389.176239013672 418.245086669922 810.6767578125 668.977905273438 681.676208496094 358.602142333984 430.282257080078 509.300231933594 683.419555664062 243.600601196289 873.008911132812 730.546752929688 421.260955810547 606.174682617188 1588.38208007812 419.716247558594 1243.45666503906 250.029281616211 311.439178466797 1770.09045410156 328.016510009766 711.997131347656 1156.3720703125 596.237365722656 610.98291015625 264.389007568359 625.267822265625 235.954513549805 638.18310546875 1022.01232910156 620.024658203125 968.551330566406 1081.47399902344 271.570190429688 818.953247070312 677.672607421875 249.055511474609 515.141479492188 317.105712890625 592.652160644531 2053.38134765625 233.427154541016 526.509948730469 349.908538818359 180.799270629883 142.469940185547 639.879211425781 730.133972167969 283.586669921875 450.575958251953 819.454345703125 391.883880615234 418.152679443359 784.662170410156 742.720397949219 638.464782714844 338.441314697266 1699.06079101562 521.585876464844 557.675170898438 453.822387695312 503.761169433594 322.800537109375 587.224914550781 1312.78271484375 402.246978759766 639.183654785156 355.415100097656 322.200836181641 651.380126953125 547.240417480469 448.179229736328 550.493713378906 578.345092773438 543.731994628906 1059.68713378906 796.743713378906 268.400421142578 443.602233886719 864.673034667969 1406.77319335938 1097.50207519531 317.265838623047 183.863861083984 794.897033691406 311.662261962891 450.705017089844 84.7902221679688 694.259704589844 1476.57189941406 226.54638671875 518.310424804688 383.731628417969 471.072143554688 873.408386230469 415.631683349609 760.674499511719 616.981018066406 403.971405029297 333.563293457031 290.807373046875 1279.07653808594 299.472534179688 1827.60107421875 952.049987792969 880.002197265625 277.333068847656 466.034851074219 897.49462890625 533.270874023438 356.965667724609 449.195861816406 364.857788085938 506.927734375 111.329765319824 618.470336914062 636.074279785156 832.92431640625 314.886413574219 388.667449951172 156.295516967773 1575.10290527344 530.983154296875 347.608673095703 921.593933105469 339.237609863281 521.204345703125 1135.54919433594 604.256225585938 1001.64935302734 1776.12585449219 724.731079101562 620.706359863281 634.029846191406 1222.2275390625 328.809112548828 521.383361816406 1197.49792480469 699.352783203125 548.946044921875 724.805541992188 823.248474121094 442.115753173828 1039.28393554688 367.863952636719 782.114990234375 444.054138183594 853.654052734375 474.100433349609 496.991180419922 868.599548339844 2264.9326171875 1459.07202148438 565.273803710938 427.8583984375 242.658752441406 542.861877441406 720.890869140625 735.045776367188 407.588317871094 205.440933227539 378.187957763672 231.984359741211 1217.49597167969 819.787902832031 304.670532226562 761.403015136719 686.461853027344 932.272399902344 467.747863769531 925.936401367188 412.941772460938 2115.48657226562 129.845230102539 334.962615966797 811.48779296875 671.188293457031 775.169006347656 549.279968261719 351.775177001953 1144.41015625 180.057876586914 737.125854492188 419.568237304688 1759.09619140625 470.195770263672 1147.82141113281 512.625671386719 516.31005859375 300.659912109375 1233.05322265625 301.685638427734 123.800163269043 329.963531494141 997.15380859375 436.912200927734 2068.552734375 835.264221191406 312.551055908203 729.245056152344 581.767639160156 264.561920166016 603.014587402344 149.725936889648 526.000854492188 511.821228027344 1047.73327636719 1329.63134765625 835.894165039062 1041.89526367188 778.313110351562 370.891906738281 416.534942626953 838.182739257812 954.555419921875 619.088073730469 1196.81323242188 687.325866699219 708.627746582031 686.121459960938 895.178100585938 863.125732421875 1008.53942871094 958.143310546875 403.584747314453 1634.76306152344 338.062683105469 893.647399902344 452.810211181641 612.486083984375 235.173004150391 545.630065917969 317.790557861328 609.068115234375 443.934020996094 774.866333007812 459.382751464844 1292.95788574219 244.588302612305 631.025207519531 242.222534179688 876.238525390625 521.74853515625 363.386169433594 811.310302734375 478.881439208984 1312.85498046875 496.163604736328 892.632751464844 444.394226074219

ENSG00000196188.9 304.540130615234 179.027420043945 94.8713607788086 239.356811523438 133.253753662109 311.747467041016 46.1016044616699 330.705017089844 7.00880289077759 42.6274070739746 3191.0927734375 1.59898638725281 237.187210083008 312.864135742188 54.2104225158691 38.2437858581543 0.0953588932752609 78.9686126708984 240.777603149414 91.270637512207 206.993423461914 227.859680175781 106.601951599121 1069.59326171875 2.15543985366821 75.8786163330078 0.251408368349075 103.931365966797 1558.294921875 589.437133789062 3139.54907226562 56.2129859924316 3.63851952552795 352.283111572266 285.754150390625 47.825984954834 385.459289550781 2126.40405273438 55.3354682922363 6.9079008102417 159.049545288086 117.830978393555 7.46669769287109 12.0891561508179 3.46505188941956 1.68455564975739 16.7648525238037 645.037414550781 34.0848083496094 162.694152832031 95.0173721313477 1.97510302066803 9.97617721557617 53.1815872192383 1.61099028587341 151.998870849609 0.0996931418776512 1029.63037109375 1564.16577148438 919.985595703125 437.82763671875 0.986280798912048 13.0463104248047 15.080376625061 161.31672668457 2980.2607421875 53.3188362121582 135.275115966797 667.085876464844 255.53662109375 0.336149960756302 21.3297634124756 2652.31713867188 275.156555175781 1.20393586158752 6.24725198745728 23.0536861419678 17.6254463195801 0.615917086601257 273.559600830078 1741.21313476562 2636.9306640625 108.431648254395 133.789443969727 25.4782238006592 36.6775970458984 243.964126586914 118.298400878906 196.153579711914 175.432861328125 149.718399047852 141.761428833008 12.3306846618652 49.2527465820312 168.677841186523 152.182067871094 234.357345581055 26.9644584655762 169.156234741211 65.9923934936523 125.872871398926 2317.32299804688 975.397644042969 331.235900878906 270.496948242188 146.935470581055 1682.34338378906 105.257171630859 22.7153511047363 39.7967567443848 232.92707824707 130.694183349609 203.849426269531 70.1472396850586 755.657104492188 4.8411693572998 601.192443847656 63.5920906066895 10.2187585830688 52.1678161621094 126.293823242188 3.84218716621399 865.318481445312 1173.248046875 1.25636565685272 26.4858703613281 3682.75390625 5.54583597183228 284.981567382812 319.098907470703 3327.94897460938 1855.92553710938 93.0453109741211 3.39389395713806 2.93084263801575 490.196166992188 9.33956527709961 27.5518703460693 739.220397949219 1107.63000488281 27.2146797180176 108.6279296875 48.5599365234375 550.124450683594 379.073089599609 1338.61950683594 1747.38159179688 575.757690429688 296.558044433594 8.27394390106201 70.073356628418 308.12109375 1176.70715332031 8.81033039093018 19.2440643310547 98.9363327026367 3.0853853225708 72.4266510009766 324.569061279297 239.971588134766 2189.5126953125 751.778198242188 13.538990020752 517.319274902344 258.887023925781 419.573150634766 1072.87609863281 73.0133514404297 10.0247468948364 6.97898197174072 40.5300025939941 2002.14208984375 8.09623241424561 3.20786595344543 0.262807995080948 135.814376831055 0.975742518901825 1014.91870117188 29.6249961853027 0.0891117379069328 24.4619159698486 82.4064254760742 1108.84228515625 1.98209524154663 119.96997833252 93.2313766479492 179.021835327148 20.1393203735352 0.0392335243523121 104.02710723877 228.641952514648 25.0341091156006 1134.57922363281 681.739685058594 1113.36901855469 1002.50933837891 117.524276733398 67.9038314819336 2387.80639648438 1.6876118183136 362.082824707031 187.980651855469 12.4909925460815 33.018497467041 1360.50170898438 77.7591171264648 16.2820930480957 65.1338348388672 67.4622039794922 5.34720802307129 71.9626388549805 411.455352783203 124.777183532715 290.655303955078 46.9966125488281 1672.95166015625 439.448577880859 84.2106704711914 6.762122631073 893.893188476562 180.329223632812 43.4344139099121 193.701751708984 68.9765090942383 1578.99694824219 28.3216724395752 1.55917918682098 4.85039234161377 1092.85339355469 349.398223876953 122.268997192383 69.1242065429688 1279.37829589844 26.7455081939697 317.327362060547 59.1450996398926 85.7864837646484 4.73295593261719 387.985778808594 36.363353729248 1129.29211425781 469.445281982422 39.8144950866699 60.646800994873 87.2352600097656 41.1604080200195 122.171234130859 18.5173759460449 45.788330078125 30.0594062805176 258.414794921875 20.5966968536377 34.6860466003418 3.9500720500946 28.3013820648193 1961.35656738281 116.540138244629

ENSG00000135047.13 537.348693847656 502.198120117188 408.5458984375 118.860557556152 393.800201416016 278.372680664062 158.62580871582 170.194473266602 146.51725769043 178.391998291016 236.128921508789 230.50846862793 107.902656555176 274.430847167969 99.3051452636719 176.631011962891 85.1523818969727 203.744491577148 259.926422119141 287.551879882812 130.162628173828 424.819427490234 74.1223449707031 201.408599853516 807.271789550781 228.837158203125 973.614990234375 113.725143432617 120.381423950195 362.216949462891 160.37971496582 118.499519348145 183.181335449219 206.12841796875 76.2349472045898 134.361679077148 257.885803222656 99.0432357788086 218.640594482422 349.808044433594 64.6037292480469 210.080902099609 154.284957885742 64.6565475463867 202.505233764648 112.09228515625 191.046279907227 2390.28833007812 113.467155456543 149.905487060547 77.608154296875 79.2203903198242 50.1951065063477 249.177734375 264.450073242188 136.417846679688 317.488189697266 290.787139892578 148.32096862793 97.4219741821289 225.954177856445 253.287994384766 351.591644287109 127.941482543945 670.945678710938 125.271224975586 253.97265625 228.115112304688 114.284820556641 123.280303955078 2080.80590820312 297.563415527344 387.215850830078 141.754623413086 467.607025146484 110.624794006348 580.37353515625 99.5767669677734 157.432708740234 248.971389770508 173.009216308594 115.251029968262 376.677337646484 139.15950012207 124.197425842285 105.548110961914 516.982604980469 2366.1064453125 268.884246826172 55.8854942321777 60.721622467041 290.745269775391 183.368698120117 161.128112792969 28.0611820220947 46.6490821838379 463.355560302734 60.7426071166992 121.660110473633 101.684799194336 558.379638671875 152.805389404297 134.209747314453 119.087135314941 294.443542480469 226.477935791016 257.559844970703 77.0956344604492 372.575531005859 141.314300537109 425.467498779297 541.829284667969 160.013290405273 82.3193817138672 92.5117416381836 256.986480712891 161.864761352539 56.0065383911133 111.572593688965 33.7395858764648 127.347061157227 71.6733932495117 101.905975341797 306.836791992188 182.245315551758 113.998832702637 77.9112548828125 25.9079570770264 246.196914672852 293.43994140625 73.4680023193359 262.522644042969 94.757942199707 229.322937011719 107.579521179199 137.208084106445 283.162902832031 470.068969726562 521.508056640625 136.950546264648 257.940734863281 565.2587890625 108.457855224609 94.4692840576172 220.833068847656 191.785034179688 102.197364807129 263.253234863281 268.549438476562 110.578514099121 684.638854980469 136.641571044922 201.133758544922 100.972923278809 872.835571289062 181.977569580078 154.720809936523 413.620483398438 971.342102050781 299.219177246094 167.96028137207 244.386825561523 360.787414550781 251.030258178711 202.284118652344 218.401824951172 147.500030517578 53.2351150512695 428.687713623047 99.3018417358398 304.229461669922 172.799819946289 60.5173110961914 490.777130126953 187.913772583008 362.088989257812 151.659973144531 213.074600219727 114.093925476074 543.939270019531 19.8666973114014 72.1466979980469 290.368713378906 163.130111694336 442.251983642578 128.853302001953 48.3637809753418 426.397308349609 113.075180053711 221.910827636719 135.505615234375 746.461181640625 90.5878601074219 438.199981689453 153.900161743164 215.837707519531 99.4829406738281 279.970245361328 91.9768142700195 61.9989624023438 147.44465637207 331.992340087891 203.908386230469 527.183166503906 143.389999389648 117.384262084961 229.088088989258 122.256874084473 65.9969940185547 170.744293212891 71.9648742675781 153.240600585938 151.542388916016 206.656295776367 616.244750976562 282.677185058594 155.982543945312 202.889053344727 217.481399536133 160.623428344727 168.027572631836 437.791473388672 162.661437988281 324.326690673828 170.687774658203 258.500152587891 286.315185546875 258.149932861328 240.120864868164 508.142120361328 197.838455200195 123.477104187012 309.660491943359 124.497314453125 551.315856933594 153.711212158203 186.131484985352 78.0011596679688 140.595947265625 57.19287109375 157.921005249023 143.669357299805 368.021667480469 80.3018417358398 286.771148681641 84.0538330078125 92.7198486328125 66.1471939086914 182.427032470703 153.835723876953 337.890380859375 154.053131103516 2248.34033203125 365.506744384766 618.661071777344 278.61767578125 193.179458618164

ENSG00000163131.9 543.354553222656 479.749847412109 254.754089355469 128.930603027344 348.507171630859 335.382751464844 167.881088256836 467.439575195312 107.047294616699 284.053924560547 198.422760009766 135.030532836914 293.429351806641 140.944976806641 129.863204956055 297.529144287109 75.1821365356445 344.649536132812 313.456848144531 116.752388000488 318.984252929688 145.791488647461 118.711250305176 146.554565429688 17.1456508636475 72.492431640625 152.91813659668 72.4799118041992 172.698181152344 389.333740234375 437.575958251953 281.601989746094 246.370239257812 103.201110839844 129.170761108398 120.837242126465 308.752288818359 537.144409179688 268.678527832031 264.167663574219 184.207321166992 326.189178466797 213.007049560547 147.3955078125 124.446228027344 199.568389892578 209.226577758789 367.787536621094 173.547500610352 189.139617919922 153.744766235352 174.257308959961 70.842399597168 282.685028076172 140.80500793457 290.819763183594 134.672714233398 312.224945068359 403.609039306641 287.677001953125 214.800399780273 149.39924621582 275.857360839844 86.6858749389648 176.137252807617 218.3193359375 108.974464416504 46.7693481445312 153.328018188477 134.826171875 170.043426513672 311.306823730469 165.724945068359 241.119995117188 135.871353149414 76.0236358642578 57.0837326049805 210.94465637207 69.2095794677734 381.944244384766 248.861282348633 437.792724609375 230.324127197266 158.397994995117 145.444564819336 372.768463134766 164.723205566406 145.017669677734 355.291046142578 93.6866760253906 226.432281494141 197.196243286133 17.1827697753906 124.017936706543 53.2131118774414 126.978302001953 429.160858154297 80.478889465332 162.842758178711 191.554306030273 113.70516204834 221.127807617188 229.83349609375 179.864486694336 170.448150634766 192.850692749023 90.9744567871094 101.394485473633 240.983108520508 94.8684539794922 281.564178466797 504.699645996094 154.361175537109 98.2490692138672 178.735366821289 318.069854736328 218.529525756836 28.1954517364502 135.840744018555 56.8118743896484 280.438354492188 57.1582107543945 208.913619995117 130.072143554688 125.909271240234 70.0232696533203 320.070190429688 65.3454513549805 101.167175292969 120.597587585449 133.94645690918 377.406341552734 55.4340972900391 58.9310531616211 57.2357368469238 152.721420288086 193.156539916992 430.946868896484 376.377197265625 190.550018310547 202.759002685547 180.083740234375 152.322204589844 180.921279907227 399.258544921875 150.49723815918 149.99284362793 258.8037109375 317.202575683594 124.510131835938 179.387725830078 111.349044799805 257.053192138672 353.707794189453 147.339828491211 231.477890014648 218.781341552734 240.719467163086 465.278198242188 383.551452636719 184.913024902344 202.364837646484 103.278160095215 168.132263183594 259.945556640625 312.082214355469 231.09196472168 80.2756118774414 68.1418151855469 63.9415435791016 189.988159179688 486.696716308594 36.1646881103516 80.3273315429688 141.994171142578 241.031982421875 122.736892700195 203.660873413086 123.957130432129 85.212646484375 78.3112258911133 282.733093261719 390.737640380859 210.364624023438 214.144165039062 98.7021865844727 43.8787117004395 209.926696777344 13.6071949005127 315.699371337891 223.069519042969 254.299270629883 197.503723144531 260.813232421875 391.435668945312 174.240020751953 227.66813659668 324.818389892578 232.95263671875 74.8689956665039 138.869476318359 260.420806884766 108.35587310791 632.915893554688 143.457931518555 300.062896728516 213.537994384766 190.652145385742 181.463043212891 426.553466796875 55.151912689209 148.996200561523 198.006454467773 357.786071777344 369.073120117188 159.494369506836 288.280700683594 329.877655029297 175.499633789062 173.919204711914 150.220306396484 258.095062255859 283.028930664062 379.377563476562 244.058074951172 170.692825317383 139.340805053711 251.216873168945 261.762023925781 247.3671875 158.704513549805 83.2583389282227 180.946365356445 130.063751220703 426.782257080078 130.365692138672 262.514190673828 138.871368408203 220.854995727539 52.0822830200195 126.226226806641 146.063919067383 54.3823471069336 185.889892578125 359.620666503906 134.137542724609 143.706069946289 80.606201171875 164.454864501953 154.5166015625 204.554107666016 272.287231445312 60.5238418579102 500.246002197266 71.7367706298828 464.834625244141 267.581604003906

ENSG00000158869.9 558.651062011719 588.765563964844 150.054321289062 165.313919067383 474.526275634766 239.383758544922 107.002632141113 125.400321960449 136.508514404297 182.03742980957 200.426986694336 211.93733215332 144.214248657227 52.6516571044922 75.6404342651367 224.634429931641 30.9660377502441 289.867614746094 283.641754150391 187.863922119141 249.775863647461 237.941101074219 140.387405395508 183.337738037109 13.0378532409668 77.816780090332 135.138259887695 86.8707580566406 201.972732543945 214.829879760742 143.693893432617 193.432983398438 123.012672424316 75.6807327270508 33.6877746582031 258.598327636719 196.604721069336 156.717041015625 272.423034667969 288.082244873047 91.250846862793 296.920379638672 163.437805175781 43.295352935791 200.440826416016 121.821166992188 166.597854614258 743.314392089844 96.3902206420898 136.604080200195 80.3572311401367 47.8039932250977 66.8764724731445 173.715728759766 235.812408447266 70.9859237670898 86.6424102783203 335.833862304688 111.335021972656 122.270027160645 247.649856567383 178.956161499023 232.754241943359 82.4086837768555 337.338165283203 103.197776794434 109.910766601562 50.0625801086426 147.397338867188 78.9567184448242 92.2072525024414 319.021881103516 77.3126907348633 251.962921142578 149.237411499023 91.7900924682617 113.997184753418 201.683944702148 108.613677978516 170.304626464844 126.580146789551 180.125625610352 194.642135620117 177.053359985352 171.575988769531 142.505279541016 187.491760253906 156.395111083984 604.931396484375 94.8592529296875 33.2986869812012 160.089492797852 18.7052249908447 129.656646728516 14.9773349761963 92.8526763916016 394.491302490234 40.0422973632812 164.34716796875 90.0044860839844 68.611946105957 178.72526550293 183.523086547852 210.329071044922 53.8142509460449 180.9794921875 102.29874420166 10.7082014083862 288.258239746094 83.3006439208984 354.640808105469 308.720611572266 303.790496826172 84.4899368286133 102.488273620605 258.249694824219 176.18782043457 17.2310886383057 56.4158935546875 46.197826385498 197.312545776367 16.0544452667236 100.754531860352 218.354904174805 297.563751220703 81.9620361328125 30.3195114135742 120.856452941895 191.770095825195 155.97346496582 24.7528915405273 191.001342773438 58.7080688476562 104.783210754395 81.3639144897461 186.256072998047 241.94221496582 478.751861572266 223.585296630859 150.930770874023 139.638870239258 245.708724975586 70.2419357299805 132.802124023438 298.816650390625 280.532287597656 60.4876937866211 258.023101806641 362.305480957031 78.6440658569336 301.992736816406 112.747406005859 260.224517822266 244.397445678711 166.403884887695 164.933212280273 263.145660400391 177.648178100586 640.99658203125 500.810485839844 134.786560058594 68.8216552734375 24.3915061950684 98.6746978759766 203.286682128906 392.405670166016 65.8999099731445 26.3204402923584 96.8166427612305 75.0123291015625 273.649230957031 259.096466064453 28.4372997283936 95.5801849365234 173.578582763672 357.453399658203 186.78971862793 231.787155151367 97.7301177978516 207.977203369141 59.6272850036621 105.746742248535 179.865539550781 185.56233215332 312.260589599609 91.9081497192383 25.650541305542 351.124908447266 16.632043838501 235.343185424805 139.615905761719 272.004241943359 143.854675292969 333.479644775391 152.001495361328 159.424224853516 139.276168823242 352.950958251953 59.9846153259277 28.7806224822998 204.672424316406 300.855590820312 126.665023803711 1102.63708496094 176.295883178711 215.58268737793 144.446166992188 169.143463134766 56.4489822387695 354.898162841797 33.7119369506836 113.475624084473 151.546203613281 261.099822998047 325.229583740234 205.262115478516 602.078308105469 225.058990478516 94.3295211791992 277.339263916016 99.23974609375 316.883575439453 319.18994140625 379.792083740234 225.03401184082 240.578536987305 210.684036254883 331.261108398438 246.18244934082 386.866729736328 271.249145507812 29.0008792877197 446.592041015625 78.9365463256836 286.604248046875 225.687423706055 183.83512878418 22.0159072875977 116.74681854248 71.1093521118164 171.074417114258 119.709358215332 111.930061340332 87.5222854614258 441.813110351562 66.1025619506836 170.550872802734 49.8283729553223 291.324768066406 97.0220489501953 156.864624023438 218.685989379883 102.98201751709 548.742126464844 117.588165283203 387.815795898438 120.630676269531

ENSG00000104870.11 124.914520263672 170.628051757812 237.169555664062 112.424102783203 119.678443908691 85.907600402832 107.53581237793 33.9383659362793 160.021987915039 99.5126800537109 149.940567016602 77.5210342407227 259.230926513672 73.4585189819336 133.790100097656 84.3532867431641 183.620956420898 193.517700195312 94.2754211425781 107.592193603516 184.168487548828 106.349342346191 168.641937255859 110.2646484375 22.9095649719238 52.8090209960938 148.742080688477 143.439544677734 146.640319824219 182.393829345703 248.160079956055 200.564376831055 125.35733795166 115.394737243652 79.2869491577148 124.753707885742 157.004547119141 66.9614868164062 145.710800170898 115.934532165527 155.083541870117 179.162399291992 162.628936767578 75.5951614379883 81.8799819946289 135.302291870117 117.168838500977 278.97900390625 48.8620529174805 133.315093994141 197.077728271484 127.312240600586 30.4611434936523 87.0265121459961 70.0004730224609 138.42301940918 156.675567626953 232.160171508789 178.634048461914 151.06071472168 93.8012390136719 109.896362304688 191.456878662109 64.3539428710938 179.952713012695 236.049606323242 64.9507827758789 63.7007904052734 150.182998657227 159.331466674805 24.3559455871582 102.029090881348 154.858032226562 84.1371154785156 47.9790077209473 58.6317901611328 58.2372856140137 217.513442993164 75.8444366455078 192.825500488281 100.337684631348 114.28491973877 111.196754455566 64.8914642333984 216.437850952148 106.261627197266 87.7708740234375 108.822303771973 115.321258544922 71.1298446655273 72.1848373413086 118.182960510254 58.7353553771973 121.332168579102 29.6951808929443 326.230560302734 129.234939575195 134.714752197266 139.717056274414 176.205871582031 82.2730026245117 150.021331787109 136.956390380859 167.187255859375 149.484527587891 119.403678894043 143.360961914062 59.8050727844238 134.765213012695 61.1009330749512 93.1611022949219 249.60090637207 119.692756652832 117.287544250488 220.231170654297 18.1153202056885 200.024642944336 55.7274780273438 131.253128051758 42.077507019043 159.632843017578 32.0100135803223 162.088272094727 149.525009155273 69.7528991699219 73.6524810791016 289.558166503906 62.5285568237305 113.534278869629 119.644317626953 44.7642517089844 219.693435668945 45.8323364257812 265.147155761719 92.1394195556641 119.796348571777 208.805526733398 124.133316040039 83.2081298828125 171.228546142578 83.2251739501953 172.195724487305 178.156616210938 218.917129516602 84.568244934082 104.286674499512 194.542907714844 153.77360534668 152.506500244141 142.798751831055 95.0746078491211 130.730850219727 110.533538818359 272.726348876953 85.5728607177734 71.5263595581055 109.861602783203 140.168960571289 347.84228515625 175.957229614258 233.949752807617 191.630783081055 22.1182289123535 110.811897277832 98.2842330932617 228.902725219727 216.442123413086 25.1389923095703 48.8349685668945 233.45182800293 79.3077850341797 188.57096862793 118.927314758301 122.123710632324 87.8501586914062 243.349548339844 101.033638000488 185.892990112305 240.596328735352 131.148208618164 17.1437168121338 197.672546386719 116.29443359375 95.1017532348633 209.745056152344 128.112350463867 104.326667785645 57.0648574829102 56.3264122009277 126.252388000488 165.237945556641 96.5767440795898 133.446350097656 131.111907958984 121.268013000488 46.8016967773438 241.67333984375 118.940048217773 151.932907104492 23.6133422851562 158.858154296875 108.391174316406 36.5196952819824 486.064056396484 278.739440917969 67.315803527832 381.600616455078 181.99365234375 133.559692382812 136.55500793457 94.5624389648438 229.59049987793 152.117385864258 188.111526489258 99.1623153686523 93.6189041137695 192.140350341797 164.194854736328 59.2084426879883 260.555786132812 186.103240966797 72.9541397094727 39.130199432373 93.0355453491211 178.342620849609 100.66032409668 84.1244049072266 75.5999908447266 98.4125366210938 145.669616699219 117.22013092041 419.030456542969 318.144897460938 136.016220092773 101.766395568848 122.841270446777 333.350708007812 203.921264648438 129.627487182617 76.2037124633789 118.569007873535 110.01097869873 52.2828140258789 172.219161987305 92.9420471191406 112.463066101074 396.617309570312 240.565383911133 120.204170227051 308.290618896484 158.089889526367 101.590476989746 79.525260925293 88.8832168579102 34.543701171875 233.618270874023 246.475173950195

ENSG00000167004.11 472.792846679688 347.478698730469 1252.33142089844 319.288940429688 251.873718261719 435.449157714844 559.6875 175.119552612305 209.073455810547 651.877502441406 391.822784423828 537.633056640625 584.7685546875 2004.32592773438 347.232604980469 686.092041015625 819.436706542969 349.727447509766 320.18359375 334.133453369141 312.236175537109 605.732971191406 326.670837402344 183.873001098633 701.976623535156 710.475341796875 342.015472412109 715.609680175781 408.062957763672 857.954895019531 643.209777832031 736.532165527344 404.973602294922 388.583618164062 1608.962890625 410.271942138672 396.4208984375 741.600769042969 278.736328125 336.059143066406 435.040283203125 247.792984008789 486.527221679688 238.716613769531 251.73698425293 317.447601318359 347.233612060547 410.8701171875 446.552215576172 700.008178710938 983.548583984375 31.922830581665 360.298675537109 804.450805664062 596.46533203125 1714.24975585938 946.551513671875 567.662292480469 452.874450683594 639.153076171875 307.564788818359 747.807434082031 551.320678710938 377.236175537109 247.117965698242 548.306518554688 295.459899902344 349.635284423828 425.31689453125 381.6796875 767.864990234375 482.005157470703 1088.06372070312 413.751190185547 500.902130126953 591.535461425781 718.602966308594 499.809722900391 293.990386962891 602.913208007812 625.286010742188 402.658050537109 284.031585693359 282.208312988281 408.161041259766 422.630828857422 770.783996582031 746.798706054688 348.064178466797 306.037872314453 257.919525146484 486.771697998047 656.928283691406 527.659912109375 123.794654846191 334.267852783203 343.651123046875 280.036682128906 501.881866455078 327.703033447266 668.143615722656 597.083068847656 383.127716064453 450.023468017578 923.974853515625 362.341583251953 339.786987304688 232.690505981445 515.946899414062 1126.14636230469 411.638580322266 506.622375488281 574.4609375 413.776275634766 838.421508789062 848.053466796875 484.127105712891 913.663024902344 645.174865722656 636.145629882812 470.400512695312 280.941680908203 376.734832763672 289.618682861328 282.825561523438 786.419189453125 464.1396484375 165.199722290039 407.696380615234 800.608093261719 470.967163085938 1218.86499023438 325.200408935547 1761.56884765625 430.0517578125 413.344360351562 373.155639648438 355.342712402344 668.773620605469 863.951354980469 1089.15979003906 620.051086425781 476.887786865234 243.327911376953 559.577941894531 533.967407226562 526.250793457031 298.608001708984 286.539337158203 927.700439453125 408.103271484375 538.474731445312 329.636199951172 433.794342041016 418.853485107422 560.478515625 763.690002441406 529.632934570312 213.270004272461 425.648040771484 417.598815917969 1332.77758789062 525.657592773438 921.780151367188 243.461624145508 183.477478027344 937.05908203125 109.137420654297 289.234527587891 919.397216796875 565.447082519531 337.002227783203 598.557495117188 588.9326171875 614.525512695312 314.747741699219 447.243530273438 388.580108642578 1307.04211425781 560.76318359375 107.316299438477 531.169128417969 236.829711914062 385.129241943359 694.689758300781 1089.05346679688 429.72802734375 633.832458496094 547.640930175781 520.272827148438 398.912170410156 422.479095458984 493.349212646484 610.865539550781 442.886047363281 647.664428710938 207.854095458984 428.012969970703 956.643798828125 102.07559967041 284.309173583984 212.971374511719 210.161651611328 370.068359375 409.340209960938 591.83203125 1620.50402832031 323.9638671875 313.266387939453 474.338439941406 1122.28820800781 323.068756103516 272.167144775391 641.789489746094 523.035705566406 459.518981933594 227.83544921875 219.539657592773 261.305755615234 244.621765136719 459.610321044922 445.284545898438 391.578491210938 426.708679199219 244.042327880859 425.583618164062 323.798248291016 319.569915771484 491.007019042969 456.992401123047 346.8505859375 1085.82592773438 354.650573730469 386.489105224609 597.561584472656 267.927032470703 295.387329101562 505.251159667969 593.236572265625 844.637268066406 421.176605224609 303.828094482422 809.679260253906 488.444976806641 459.556396484375 223.5849609375 441.771575927734 288.046630859375 366.531219482422 517.960754394531 194.092254638672 295.625579833984 755.214782714844 428.319549560547 369.584259033203 357.750122070312 470.581176757812

ENSG00000010704.17 13.4441289901733 15.0792999267578 12.0428791046143 10.5168285369873 6.16102647781372 11.3352909088135 14.3845157623291 11.7807292938232 5.14219856262207 6.7889347076416 13.1475114822388 7.40328884124756 10.8183326721191 33.3240242004395 15.3564214706421 9.13877391815186 7.05540180206299 15.8803186416626 11.6609344482422 8.5185604095459 10.4732732772827 9.14891815185547 10.5754899978638 12.12633228302 24.5904483795166 10.923210144043 22.9218368530273 5.24965763092041 10.4753408432007 13.2892446517944 15.6026639938354 14.1155366897583 6.4877724647522 12.9243946075439 9.24946212768555 11.5761089324951 14.3449983596802 15.3184719085693 6.06202030181885 13.6053495407104 17.2673187255859 9.34022617340088 14.6595764160156 10.0884609222412 4.10489654541016 7.88323783874512 2.1455225944519 10.4924087524414 7.3947606086731 21.8782157897949 5.24944639205933 9.73349666595459 9.79503059387207 38.4651756286621 2.35223627090454 21.9381122589111 36.2704391479492 21.1871452331543 14.8537931442261 16.0998477935791 7.85804653167725 4.46796083450317 23.4363918304443 6.63831043243408 8.5759449005127 12.0432367324829 10.117657661438 4.52273845672607 20.2532577514648 10.5695066452026 11.8975877761841 12.7437877655029 4.9224681854248 11.734263420105 17.2017345428467 6.28391933441162 30.551233291626 17.1855239868164 1.40268409252167 11.9168910980225 9.89477729797363 12.6782493591309 21.8009929656982 3.15245270729065 6.98905897140503 12.1035318374634 19.9925556182861 26.9301280975342 6.0022234916687 6.84575080871582 7.34409856796265 13.5436382293701 5.48200035095215 14.9054670333862 10.3970308303833 17.1627006530762 9.2002649307251 11.3845338821411 8.63399696350098 16.3499240875244 24.8025283813477 15.1147193908691 7.38440418243408 29.8722515106201 13.3872737884521 12.5858945846558 20.9588432312012 17.3337993621826 20.313325881958 5.93753242492676 13.2529020309448 17.1294059753418 6.41073369979858 19.2496185302734 8.79145336151123 22.4599781036377 14.3921566009521 2.54187512397766 7.93219995498657 4.88540029525757 11.1601552963257 3.09172415733337 11.1415271759033 12.8001356124878 6.81940269470215 4.73852872848511 11.7746744155884 0.489413887262344 20.3265819549561 15.6244173049927 8.93119430541992 8.20114040374756 4.88776302337646 16.2703819274902 4.77444458007812 6.99948263168335 9.73285102844238 28.717658996582 19.0140647888184 11.565673828125 11.9100141525269 5.8855767250061 8.89884757995605 10.1543750762939 32.2039604187012 18.2114944458008 27.9908542633057 11.0954504013062 7.93303728103638 3.40526032447815 23.957124710083 7.12761259078979 8.50027179718018 9.30375289916992 21.0110530853271 6.62953805923462 5.24733686447144 14.0427627563477 8.88624095916748 12.0397205352783 11.6657114028931 20.3720016479492 9.99497509002686 21.8801918029785 12.4899501800537 11.2348394393921 15.4040899276733 14.9968852996826 4.39891529083252 17.6046924591064 8.36137104034424 12.686713218689 7.39021396636963 6.5425386428833 11.1787929534912 17.2478904724121 9.86330318450928 16.2705383300781 11.3909149169922 7.03217506408691 6.58247852325439 13.5115337371826 18.6089954376221 10.2259588241577 8.58482933044434 8.09677219390869 15.4587984085083 15.8277883529663 3.58234214782715 6.71603441238403 7.91812658309937 10.4892196655273 19.9310493469238 8.87734508514404 13.0799522399902 11.4791078567505 15.5285892486572 13.2768869400024 8.64978122711182 2.96166491508484 12.3378210067749 5.72699356079102 2.69141864776611 21.5089683532715 13.6531352996826 9.16074180603027 18.9122848510742 7.31590986251831 11.6694707870483 17.7655754089355 10.4497737884521 11.534517288208 11.2992553710938 18.0380840301514 19.2785396575928 11.5157260894775 7.37636137008667 12.4963607788086 8.66622066497803 14.7634086608887 24.8535633087158 13.5371170043945 7.20880937576294 7.66005611419678 8.09123420715332 10.1944961547852 10.4128675460815 9.81119728088379 15.5458288192749 15.0089464187622 7.09657955169678 18.3738307952881 20.7556457519531 11.2460746765137 21.9008312225342 4.34472608566284 22.4419059753418 6.58529090881348 7.8687686920166 7.28673410415649 12.1362409591675 12.1900157928467 12.9016370773315 8.40698623657227 9.60206127166748 15.1772518157959 12.212646484375 5.776780128479 7.47515058517456 9.01817798614502 13.4996995925903 8.13156509399414 20.9053859710693 19.1844444274902 12.585057258606 9.68095397949219 9.89083576202393

ENSG00000206503.10 3398.2783203125 3293.087890625 1539.25012207031 1017.0595703125 2484.56713867188 4336.640625 1121.34460449219 1015.91632080078 1619.5458984375 1455.34924316406 1507.66821289062 1002.54010009766 3648.91479492188 2692.33764648438 2156.75854492188 3078.11938476562 940.279235839844 3731.71850585938 2044.63977050781 1115.49035644531 1655.53039550781 4176.32666015625 1743.146484375 1804.91821289062 355.645935058594 1238.15002441406 3897.91870117188 1679.13708496094 1804.38757324219 1806.75366210938 2368.345703125 5278.22021484375 3065.85400390625 535.40234375 1303.76403808594 4246.05322265625 3128.40600585938 3228.1298828125 2284.27026367188 1351.32800292969 951.965393066406 3774.25708007812 3617.48901367188 694.249389648438 1416.37292480469 2138.55444335938 1638.32275390625 1482.23217773438 2058.49462890625 1065.54382324219 2365.212890625 637.709167480469 834.36083984375 1764.21215820312 1897.68115234375 2112.01733398438 5665.74609375 2106.95922851562 1901.71350097656 1983.48486328125 2015.98095703125 723.327270507812 3128.83081054688 639.4580078125 2349.01025390625 1441.24401855469 1520.08422851562 446.006286621094 1528.21838378906 2443.36157226562 834.862976074219 1721.11437988281 928.170349121094 2365.60302734375 1784.97192382812 521.404174804688 1212.14453125 1771.17138671875 570.884765625 1644.28369140625 1097.73706054688 2971.98559570312 1284.88684082031 2415.962890625 1733.12292480469 3878.90356445312 2219.91333007812 1079.01647949219 2538.603515625 4441.7490234375 472.575042724609 815.854736328125 442.518737792969 1550.70422363281 254.220855712891 1976.16564941406 2356.27856445312 1680.22863769531 5199.47509765625 1213.69250488281 2209.66284179688 970.068603515625 3015.78198242188 1865.60168457031 516.820251464844 4668.12744140625 1076.18957519531 359.222106933594 2828.22119140625 694.581665039062 3499.83203125 4152.65478515625 3292.443359375 3350.8046875 2207.60571289062 796.211181640625 2367.015625 1525.69934082031 783.237487792969 1253.85327148438 2693.80688476562 131.380783081055 1384.76635742188 1411.59814453125 904.607482910156 941.562255859375 1706.11389160156 1964.87414550781 3192.03051757812 865.106994628906 973.92626953125 2738.62646484375 1229.32629394531 1733.01171875 998.187927246094 651.3095703125 2904.4228515625 3238.51538085938 2681.24291992188 3662.98974609375 1015.66735839844 960.839233398438 995.63671875 1388.91796875 5416.69677734375 1601.90979003906 1707.79772949219 3798.87060546875 2069.02783203125 627.705688476562 3531.22290039062 840.356018066406 3586.64111328125 817.031372070312 1574.70141601562 1345.86474609375 1493.53161621094 1127.21215820312 1939.39428710938 1673.72473144531 1191.58728027344 716.618957519531 1194.52270507812 1964.85424804688 1413.35900878906 2298.701171875 2240.283203125 134.352584838867 682.605773925781 2769.677734375 1402.07360839844 1788.57836914062 1145.77478027344 2464.98120117188 5259.0673828125 2151.09521484375 1250.38537597656 3501.880859375 1860.32116699219 1310.79406738281 304.425384521484 2571.72875976562 5036.82177734375 933.017822265625 1770.96472167969 1275.82995605469 856.237487792969 5369.38134765625 789.271118164062 2156.87890625 1597.44885253906 2361.2841796875 1406.57946777344 3028.96459960938 2708.35668945312 2859.6328125 2912.3916015625 1851.99877929688 1043.31164550781 211.835418701172 4450.2431640625 1509.1591796875 2037.80810546875 4516.09326171875 2277.18872070312 1317.79663085938 1619.52465820312 1146.39233398438 671.354187011719 5663.8486328125 1648.76257324219 1952.74719238281 1751.052734375 1717.78430175781 2829.60083007812 2190.19311523438 1942.38500976562 3728.70947265625 1498.91809082031 853.220031738281 2333.63305664062 1348.3505859375 760.947082519531 2489.94140625 1976.27844238281 2109.3037109375 1951.6123046875 3755.68237304688 4050.85522460938 1402.99438476562 1246.67114257812 965.472961425781 1674.05688476562 410.900360107422 1963.05627441406 1922.74670410156 3367.19775390625 526.548461914062 1737.67248535156 2136.45751953125 1601.44580078125 1592.64794921875 1001.63336181641 2387.37768554688 2355.64233398438 1772.0234375 2164.5576171875 722.698425292969 577.086853027344 2278.8740234375 752.927795410156 3814.67993164062 312.930847167969 3006.30883789062 2242.52221679688 2833.79736328125 3009.15771484375

ENSG00000234745.8 5304.92822265625 4624.82861328125 1322.44128417969 2942.38891601562 3735.50463867188 5118.5361328125 955.394165039062 1622.10583496094 1765.2578125 1908.755859375 2544.7275390625 1294.26684570312 4270.65087890625 3328.98999023438 2091.8330078125 5448.3203125 350.310455322266 6310.53173828125 3202.77392578125 1587.73059082031 2442.1123046875 5308.56298828125 3092.552734375 2041.18298339844 240.542068481445 1060.65905761719 4708.81005859375 1378.71643066406 2400.16772460938 1658.36889648438 3020.50805664062 6493.6728515625 2587.04150390625 791.919982910156 672.804504394531 2143.56811523438 4045.55908203125 4316.42041015625 2831.95458984375 1817.70690917969 1866.23852539062 4960.82080078125 5434.9580078125 1023.92156982422 1990.83508300781 2936.59204101562 2686.15356445312 2476.83422851562 3099.3251953125 2098.91064453125 2052.48291015625 652.294250488281 795.034484863281 2178.52270507812 3682.43774414062 1759.85717773438 4627.34375 2736.0244140625 2878.81176757812 3002.37719726562 2309.93237304688 1180.15417480469 3762.79614257812 626.078735351562 1578.12817382812 1380.80407714844 1464.69421386719 571.295654296875 2261.6806640625 3802.0185546875 887.582275390625 2088.84594726562 669.195007324219 2526.4365234375 2259.11279296875 850.465576171875 994.348205566406 6341.60546875 607.606384277344 2012.40563964844 1767.02258300781 4434.3544921875 1602.68994140625 1316.49816894531 2095.0703125 6409.5810546875 3818.71704101562 1076.98291015625 4631.76953125 2812.61840820312 976.697998046875 1059.44873046875 343.072021484375 1125.28173828125 477.919982910156 2072.19360351562 3424.54028320312 2053.8544921875 5920.23779296875 1664.94470214844 1764.47631835938 1182.18029785156 3580.39135742188 2480.28564453125 523.965270996094 5248.9091796875 1049.875 328.325073242188 3612.60424804688 438.583709716797 5563.51904296875 6275.8037109375 3262.38208007812 3622.0693359375 2139.54443359375 1037.18029785156 3468.02294921875 1284.54858398438 425.456298828125 505.175689697266 3952.24877929688 183.494125366211 1549.23181152344 1815.40856933594 1124.25622558594 909.284118652344 1523.103515625 2048.91723632812 3075.16430664062 1079.7216796875 1076.61511230469 2909.3828125 758.179260253906 531.312133789062 1052.7841796875 1525.2763671875 3896.07983398438 5236.1357421875 5173.41064453125 4520.4677734375 1329.60632324219 1233.94689941406 1048.10021972656 2186.091796875 5185.1220703125 2885.02856445312 1906.982421875 4219.30810546875 3775.79321289062 609.548522949219 4776.73486328125 1072.78125 4438.865234375 1485.49780273438 3240.2900390625 1929.94543457031 1928.40551757812 1563.23828125 3148.44067382812 2644.82348632812 1906.49133300781 798.662963867188 913.461547851562 2703.61547851562 2473.15209960938 3252.90625 2116.53833007812 332.273406982422 752.775695800781 3168.17724609375 1992.98754882812 2955.55346679688 1256.28955078125 2676.99853515625 3234.47583007812 4045.34228515625 1730.54992675781 3426.96704101562 2141.82470703125 1287.07495117188 539.474792480469 3368.07202148438 5413.80908203125 1189.92553710938 1478.89501953125 769.950439453125 904.708251953125 7176.60498046875 983.667114257812 4084.80029296875 1856.13110351562 3056.56665039062 2396.29858398438 3925.78979492188 3949.9208984375 4053.93334960938 4170.40283203125 3201.61572265625 1127.11450195312 252.093414306641 5978.06005859375 2102.85229492188 1846.94262695312 7737.94140625 2537.2822265625 2080.4453125 1124.12390136719 1545.634765625 998.29736328125 6935.38525390625 968.344360351562 2913.15844726562 1953.43359375 2203.02612304688 4020.2529296875 2643.52954101562 2179.38989257812 4310.62841796875 1806.18383789062 1556.63623046875 3139.01538085938 2042.09240722656 1496.10766601562 3591.59375 3006.44091796875 2563.10913085938 3154.29565429688 4090.40063476562 6927.72216796875 2972.64453125 1595.23376464844 500.642913818359 2174.25439453125 707.723205566406 2098.3720703125 1994.21423339844 3028.54028320312 368.733856201172 1497.16345214844 1781.93786621094 1818.38854980469 2537.12036132812 1226.28076171875 3330.48168945312 3963.6484375 2178.14868164062 2817.28100585938 814.446044921875 1559.98205566406 1699.3603515625 548.716613769531 3907.66552734375 628.014953613281 4436.203125 1499.02392578125 4113.93896484375 3799.30932617188

ENSG00000204525.13 2800.09790039062 3529.28271484375 1323.03833007812 2389.314453125 1537.46252441406 4376.64501953125 940.726867675781 553.771179199219 1897.43713378906 1474.67614746094 1439.91967773438 1317.546875 3044.32885742188 2507.97412109375 1232.23706054688 3178.35888671875 695.260314941406 3725.56225585938 1779.10754394531 837.900939941406 1556.57202148438 2773.2109375 1548.26611328125 1561.02587890625 295.640502929688 899.652099609375 4474.82666015625 980.94482421875 1221.90502929688 1228.1689453125 2375.14331054688 5293.0224609375 2031.04541015625 960.661254882812 931.515075683594 1092.84741210938 2596.2919921875 3529.32958984375 2744.15966796875 979.571655273438 1029.99060058594 3352.74609375 2297.38208007812 1308.29895019531 1573.69641113281 1240.06604003906 2405.01513671875 1939.14208984375 2234.1044921875 1622.79382324219 1855.30297851562 691.967102050781 468.347045898438 1864.03430175781 1725.24816894531 1736.11645507812 3782.11596679688 1900.11755371094 2132.57934570312 2267.11499023438 2062.66015625 1195.94885253906 2985.84985351562 459.525756835938 1747.25305175781 1408.89099121094 1289.54406738281 494.156463623047 1184.5556640625 1933.828125 711.375427246094 2002.08337402344 894.416564941406 1428.4189453125 3198.27734375 551.027893066406 728.321838378906 4849.4208984375 514.910583496094 2409.66430664062 1315.85327148438 3928.10302734375 1235.16149902344 1160.150390625 1568.84594726562 4171.7294921875 2181.61767578125 1033.38061523438 2975.58740234375 3053.166015625 715.134216308594 977.881713867188 758.335754394531 1046.21313476562 276.796051025391 1992.06896972656 2621.53393554688 1643.60327148438 3203.80029296875 1271.18920898438 1375.89367675781 910.807434082031 2794.44653320312 2354.94018554688 593.800598144531 3362.40869140625 1619.65576171875 411.6298828125 2465.58959960938 526.65087890625 3277.43139648438 4639.4443359375 2292.18041992188 1639.77221679688 1807.5859375 813.538635253906 1796.39367675781 668.199462890625 367.354614257812 768.367980957031 3220.57446289062 441.019592285156 1755.01098632812 1378.9580078125 804.72314453125 991.145385742188 1539.30651855469 633.362060546875 2510.82592773438 645.307006835938 1154.61840820312 2222.35864257812 501.957550048828 1257.60522460938 1606.37756347656 819.097595214844 3119.38354492188 4459.26904296875 3007.03466796875 3004.89086914062 1282.18115234375 1449.05725097656 902.275817871094 1278.91857910156 3278.5224609375 2197.0458984375 1419.90283203125 2718.16259765625 1790.40380859375 389.730926513672 2499.13232421875 1257.13171386719 2555.11669921875 992.093017578125 2329.25170898438 1599.66381835938 1118.15063476562 2328.24560546875 1570.986328125 1952.05224609375 1323.4697265625 684.65185546875 501.174530029297 2966.87890625 1397.34558105469 2214.79467773438 2146.46606445312 195.661407470703 512.264709472656 1958.87048339844 1831.18383789062 1553.96594238281 1060.021484375 2174.97021484375 2058.4365234375 2221.05419921875 1439.91967773438 1751.24096679688 2730.39794921875 915.796508789062 207.925903320312 1950.00244140625 4520.392578125 879.397705078125 1195.48852539062 1357.13586425781 730.068542480469 2156.759765625 620.042114257812 2301.02954101562 1058.93151855469 2750.03857421875 1721.97619628906 2673.5322265625 2989.31225585938 3216.20166015625 2549.38134765625 2240.42553710938 987.664855957031 105.208236694336 3815.92236328125 1487.48864746094 1447.35083007812 5701.22509765625 1916.28503417969 833.784423828125 1973.72033691406 1259.294921875 550.592834472656 4196.38623046875 1469.77392578125 1821.8349609375 1103.904296875 1930.08032226562 2702.84790039062 1698.87182617188 1216.40625 2377.59252929688 1358.59338378906 996.7001953125 2786.65283203125 1699.19738769531 656.825500488281 2066.03759765625 1902.91906738281 1715.95715332031 2530.04223632812 3145.73095703125 4031.9365234375 1771.90759277344 1317.71276855469 618.180603027344 1576.71154785156 651.222412109375 1665.16284179688 2026.90588378906 2210.80615234375 324.647399902344 1183.59240722656 1062.47705078125 1086.181640625 1815.77478027344 752.671142578125 1977.6181640625 2193.99975585938 1349.05249023438 2517.13256835938 625.206848144531 1743.13464355469 1379.31433105469 418.652923583984 3408.384765625 446.638671875 4130.19677734375 1463.33764648438 2481.6337890625 3655.27124023438

ENSG00000204257.13 383.097290039062 339.170135498047 287.518676757812 207.489654541016 271.466064453125 207.469177246094 108.218528747559 181.184234619141 94.2587738037109 114.115829467773 221.35627746582 70.3963165283203 329.687530517578 199.707229614258 94.4839859008789 215.748092651367 14.0720224380493 378.2900390625 236.641418457031 156.64697265625 484.864379882812 89.6462631225586 457.466400146484 180.699249267578 9.91988182067871 61.7773666381836 298.769378662109 155.2275390625 464.962738037109 245.953750610352 223.752365112305 396.898864746094 247.335784912109 198.983474731445 124.970252990723 208.092041015625 247.381301879883 229.046249389648 194.814971923828 149.473571777344 378.596496582031 550.658203125 829.706970214844 107.387077331543 120.230682373047 118.282386779785 108.767456054688 320.947448730469 229.175476074219 551.830017089844 155.433609008789 53.3942680358887 75.9532775878906 157.802169799805 254.192184448242 92.9309921264648 287.367065429688 303.190551757812 159.091156005859 567.369934082031 233.678100585938 63.1270484924316 162.148422241211 129.496353149414 122.260894775391 229.294982910156 130.942123413086 38.8273086547852 252.884307861328 192.827743530273 39.8259963989258 241.036895751953 73.6932373046875 170.114700317383 58.3147811889648 65.2480163574219 36.9192047119141 840.601501464844 34.492244720459 191.420104980469 178.66471862793 224.249069213867 172.31755065918 89.2846832275391 270.830108642578 346.745635986328 147.044799804688 84.167366027832 351.306945800781 119.423141479492 127.275695800781 127.025810241699 53.0010261535645 146.101013183594 24.0472850799561 313.255676269531 270.142181396484 143.832672119141 349.286834716797 253.722122192383 110.452514648438 218.867279052734 266.396545410156 277.255554199219 84.4624328613281 139.546981811523 234.3642578125 58.3255157470703 341.840637207031 39.8235206604004 280.927398681641 234.063537597656 223.037521362305 197.765487670898 289.700134277344 146.918823242188 183.266677856445 17.595422744751 30.1433792114258 66.6209945678711 304.922393798828 27.0429744720459 127.802139282227 162.386383056641 102.193969726562 51.3957023620605 59.938777923584 196.964462280273 303.358551025391 110.743446350098 48.0111618041992 286.528289794922 106.307609558105 44.359561920166 48.7239265441895 138.655563354492 224.713302612305 325.254302978516 261.898620605469 377.074401855469 118.099227905273 153.899017333984 122.231010437012 314.596771240234 373.757751464844 202.365463256836 489.319976806641 296.446960449219 309.070404052734 48.3385314941406 178.017211914062 102.190132141113 293.520233154297 404.684692382812 81.2327423095703 148.826675415039 167.993682861328 210.597045898438 548.571838378906 359.883056640625 427.0009765625 81.5805435180664 26.0156784057617 521.974914550781 257.308471679688 481.226989746094 174.547576904297 68.2948455810547 60.0390663146973 499.525726318359 173.627944946289 380.408630371094 24.7829437255859 93.9902877807617 81.4082565307617 545.451110839844 224.282196044922 123.898643493652 84.5623626708984 55.6062889099121 59.9461097717285 427.310333251953 140.469497680664 121.297416687012 159.523681640625 77.7582931518555 297.701080322266 268.706298828125 21.669849395752 281.847137451172 341.480865478516 178.547927856445 387.979919433594 225.271408081055 304.999114990234 423.109252929688 596.376525878906 220.310348510742 256.481903076172 18.8365268707275 400.15234375 159.546813964844 80.5131912231445 857.922119140625 388.280975341797 378.164093017578 93.8157577514648 365.556762695312 203.840545654297 455.690460205078 27.3671035766602 175.818618774414 583.086303710938 199.176895141602 268.633422851562 183.039947509766 303.234497070312 342.618957519531 26.7212181091309 239.360061645508 381.240875244141 102.217262268066 204.726135253906 176.564361572266 275.097747802734 460.210327148438 80.2708206176758 183.547241210938 247.817993164062 262.874542236328 175.818878173828 37.2199974060059 203.11946105957 54.5201187133789 182.292617797852 244.691299438477 925.803161621094 55.4598999023438 173.239654541016 288.769836425781 106.893478393555 229.718795776367 47.7522735595703 189.499877929688 237.169204711914 324.785064697266 579.379699707031 120.289169311523 425.348846435547 106.615760803223 72.9046630859375 281.740417480469 24.0705261230469 220.995529174805 72.4148483276367 363.117553710938 318.602783203125

ENSG00000242574.7 167.658264160156 139.491363525391 58.999626159668 46.1327362060547 125.105522155762 61.301929473877 19.5269737243652 116.391731262207 48.7795562744141 45.5301246643066 87.5920486450195 32.3893508911133 73.4336318969727 39.0644569396973 44.0475120544434 91.4690933227539 3.14070606231689 129.647079467773 80.2907638549805 50.337760925293 132.623809814453 36.5150985717773 68.0163040161133 51.5312957763672 4.47677087783813 23.4893989562988 78.3516235351562 27.9404544830322 80.4992752075195 47.4813613891602 90.8401641845703 128.617691040039 37.6598320007324 64.113899230957 8.1039924621582 60.6797256469727 111.144256591797 106.559356689453 90.4174423217773 72.7693939208984 70.3864669799805 193.156509399414 180.569152832031 21.572509765625 32.163143157959 46.8655128479004 87.5917739868164 87.9196624755859 40.4933128356934 107.08812713623 27.8169918060303 27.9102363586426 15.0624771118164 52.9861145019531 89.8637390136719 17.9365367889404 65.6882171630859 118.23885345459 49.0300750732422 128.647079467773 73.1401748657227 30.0173244476318 71.817497253418 29.6510791778564 97.8113403320312 64.8917007446289 44.8029937744141 8.37417984008789 49.0705757141113 31.1205501556396 10.7627058029175 97.1977767944336 19.8330116271973 62.9279823303223 18.3722114562988 30.3156490325928 13.8681926727295 186.402313232422 10.2944355010986 61.3562202453613 36.4811325073242 98.1442565917969 66.1884841918945 28.8823490142822 62.4718475341797 114.650085449219 45.2615280151367 26.1031513214111 129.956726074219 35.5794067382812 70.9645690917969 48.7962341308594 5.01240873336792 51.2491722106934 8.96301746368408 46.0482368469238 117.709144592285 29.643892288208 88.0604248046875 80.4649963378906 13.415111541748 53.0571632385254 88.6463317871094 80.6097640991211 15.468111038208 53.691535949707 69.409049987793 7.69621086120605 116.301284790039 16.3800411224365 121.07438659668 138.923919677734 83.6058883666992 39.5122146606445 75.2230377197266 40.8431434631348 51.3648300170898 4.56107616424561 10.0641098022461 11.8261976242065 127.713844299316 10.0274419784546 48.4065132141113 35.5516929626465 49.9287986755371 18.3467044830322 23.3616218566895 45.031665802002 33.352352142334 36.8835754394531 12.0481014251709 81.4184722900391 10.4638805389404 15.3982744216919 33.5685539245605 43.201732635498 77.8619613647461 130.59912109375 86.7307662963867 109.461235046387 33.0879325866699 62.1918144226074 26.6244983673096 79.7743682861328 132.622436523438 75.1467132568359 122.071250915527 109.559265136719 85.988410949707 18.7133159637451 69.5791473388672 28.5463199615479 124.368545532227 122.233657836914 44.9128265380859 51.4165267944336 79.5624237060547 77.2082443237305 175.787551879883 130.241394042969 126.214546203613 23.1715774536133 5.13716650009155 103.38272857666 56.9445381164551 233.953186035156 33.709644317627 27.0578498840332 12.9154958724976 93.8803405761719 102.875648498535 169.822128295898 7.03871250152588 33.0115928649902 35.7011947631836 160.559555053711 70.4167404174805 60.2475471496582 32.0684127807617 17.1631641387939 36.8668212890625 86.3022003173828 62.1331024169922 56.1874732971191 54.4336280822754 29.8658924102783 38.7800521850586 89.1484298706055 7.54147624969482 95.7889862060547 97.6315307617188 62.4191513061523 86.0799865722656 86.6731796264648 96.6770629882812 97.3084106445312 130.445831298828 94.4453201293945 27.2349033355713 24.9590854644775 107.273254394531 76.6772537231445 36.7893180847168 456.173522949219 59.8493537902832 72.0051574707031 66.1405258178711 114.403495788574 36.797679901123 158.70051574707 5.60896873474121 40.8731422424316 110.164016723633 70.8167037963867 121.405181884766 58.1082992553711 108.380958557129 119.022926330566 9.75891208648682 54.0173950195312 86.8936157226562 59.0441131591797 63.4717636108398 98.6041717529297 76.1399917602539 94.4702453613281 33.0264892578125 57.6593360900879 84.0682067871094 105.239616394043 53.4370193481445 9.59586048126221 92.9745864868164 19.7271556854248 80.2982711791992 60.1936569213867 295.94677734375 5.80809545516968 52.3624229431152 17.1739311218262 27.9589328765869 77.9149169921875 21.6576595306396 34.0125617980957 104.437171936035 73.0487747192383 95.1841049194336 32.6561546325684 103.894264221191 33.829963684082 77.3572616577148 105.465133666992 10.431245803833 77.9408721923828 33.0115928649902 150.389923095703 109.130043029785

ENSG00000204252.11 223.492767333984 120.243919372559 31.7962474822998 23.2039852142334 104.169227600098 35.6515922546387 8.64651679992676 62.2006225585938 11.2536067962646 34.3731269836426 22.3685283660889 27.6530113220215 80.2968826293945 11.7452154159546 24.4661026000977 76.7933807373047 1.56174194812775 102.565757751465 88.9560852050781 42.7814598083496 125.065376281738 19.1099300384521 83.2917404174805 32.5064277648926 1.73815667629242 21.0851058959961 45.422607421875 34.5700569152832 96.313346862793 26.6911392211914 102.351165771484 72.4848403930664 20.8462219238281 71.7666168212891 10.8549289703369 46.8880805969238 69.8366470336914 85.5155258178711 109.35474395752 56.979866027832 63.6866302490234 200.369750976562 244.181350708008 14.6840295791626 38.918586730957 20.0748062133789 36.1423683166504 78.8266220092773 33.0399589538574 198.616271972656 20.677152633667 15.3021802902222 22.4467830657959 58.9688453674316 53.9551277160645 13.9751653671265 16.9688491821289 90.257080078125 54.0218505859375 94.7956924438477 54.086612701416 37.1992988586426 22.2322177886963 10.3315801620483 35.4899024963379 25.7481002807617 29.6521682739258 4.67043304443359 63.2640647888184 30.8188209533691 7.44483852386475 80.1009979248047 20.4567832946777 67.4884338378906 15.9923028945923 26.8322982788086 6.04240989685059 454.130615234375 6.26977396011353 56.5565338134766 19.2507286071777 153.22233581543 51.209545135498 24.1200275421143 31.2785339355469 185.497604370117 47.2795562744141 34.098991394043 106.463325500488 17.0166645050049 174.414291381836 40.656322479248 3.42229986190796 38.1649703979492 7.93423366546631 40.6887474060059 89.9492568969727 19.4193229675293 75.047477722168 66.1407012939453 23.5386238098145 35.5861587524414 93.9242935180664 64.0804748535156 15.0266342163086 22.5520172119141 18.8198471069336 3.17835927009583 79.7728042602539 13.4350004196167 61.6146430969238 72.68896484375 95.0402603149414 27.4694366455078 70.7953262329102 25.5362491607666 13.1805419921875 1.72770726680756 4.19697856903076 12.2918281555176 92.879280090332 1.52651643753052 57.7098503112793 19.1177387237549 54.4982299804688 16.0715599060059 6.33054113388062 24.3390789031982 54.097713470459 27.4044799804688 17.8428115844727 61.0154609680176 8.33555603027344 5.90696334838867 12.3229742050171 31.9667530059814 41.7286262512207 72.3185653686523 91.3402633666992 156.734954833984 29.2434883117676 56.1568870544434 17.0845603942871 113.521842956543 249.239379882812 63.1590919494629 164.233444213867 93.8054275512695 126.541320800781 15.7347574234009 33.7542915344238 36.9301910400391 145.187301635742 63.1827735900879 20.637414932251 55.6416397094727 54.6667785644531 90.1471481323242 131.277633666992 144.625686645508 119.490859985352 15.8700590133667 3.9583215713501 43.7988433837891 45.6935844421387 122.741905212402 23.5485610961914 22.5744667053223 9.32779502868652 156.394180297852 36.3819808959961 127.317359924316 8.87459087371826 18.3851222991943 27.1195964813232 136.922515869141 46.6533203125 22.5852680206299 29.5993022918701 9.78885364532471 19.4808616638184 40.1841697692871 45.4012870788574 52.0982437133789 36.1169166564941 26.3006401062012 85.4009170532227 62.0743598937988 18.3883571624756 110.238639831543 258.432556152344 88.4014053344727 64.4349060058594 113.552154541016 78.0514450073242 80.2588195800781 141.706359863281 93.3710403442383 12.7693681716919 7.82988691329956 121.51114654541 36.1597480773926 30.9028644561768 433.371002197266 48.4996757507324 110.63346862793 29.3703517913818 141.0283203125 91.2338256835938 35.4007606506348 3.50781178474426 55.1316604614258 144.477111816406 20.9850521087646 110.120300292969 40.7887344360352 45.890209197998 81.3729782104492 7.81499004364014 21.7120342254639 38.8295669555664 60.6148376464844 60.2808647155762 91.5933456420898 93.6374282836914 148.323654174805 18.4331893920898 44.863166809082 99.9945526123047 80.982551574707 33.1758689880371 7.36712884902954 73.5548095703125 19.9551544189453 88.4318466186523 48.9017601013184 190.51936340332 5.33596324920654 49.7595405578613 9.39735412597656 20.1205501556396 88.2392501831055 14.595477104187 25.0813884735107 111.272041320801 54.2642402648926 157.489196777344 18.3143539428711 111.465873718262 28.4851036071777 16.5523853302002 73.370964050293 10.785083770752 42.5297508239746 11.2345485687256 111.352806091309 42.8260078430176

ENSG00000241106.5 19.3433265686035 9.57633304595947 14.1665344238281 5.52158164978027 22.1013374328613 9.3817663192749 6.71808958053589 15.2742385864258 4.32659244537354 6.16972684860229 6.85611152648926 7.53743505477905 21.0836486816406 3.70980477333069 6.37313413619995 21.6673526763916 2.90619802474976 37.217716217041 16.8734607696533 8.51377964019775 17.63108253479 3.68431091308594 25.1338214874268 8.07145595550537 3.84050536155701 3.34046530723572 5.90313529968262 12.8614683151245 22.5226001739502 5.91573333740234 7.46578311920166 16.7123794555664 23.9401302337646 13.0467138290405 8.69267177581787 12.8323364257812 26.08567237854 33.7023582458496 13.3342723846436 13.7500247955322 5.73562526702881 47.4664154052734 70.4219207763672 4.49881267547607 16.5024929046631 7.12660551071167 6.87814903259277 8.72253227233887 17.7039318084717 24.8802375793457 4.56416463851929 3.98064851760864 7.56644773483276 29.4707336425781 4.73708248138428 2.07237792015076 9.55437469482422 7.43598318099976 11.0976667404175 62.0004081726074 12.4425001144409 7.67999410629272 8.20528984069824 2.43424010276794 9.41685771942139 17.7070922851562 8.80545806884766 1.81593370437622 13.6104764938354 14.9178113937378 4.60687065124512 13.2227849960327 6.46854114532471 13.8429508209229 24.7248401641846 7.23599529266357 4.34314060211182 32.6365547180176 1.51378655433655 8.75445938110352 5.6730523109436 37.3953285217285 4.73058128356934 5.64107036590576 5.49178409576416 48.2553977966309 14.6644248962402 7.31729984283447 23.5813007354736 13.7561740875244 18.0932273864746 4.34871339797974 1.42651391029358 7.12118434906006 7.34249019622803 11.8596506118774 21.8165302276611 12.58851146698 19.762300491333 22.9805698394775 4.4619312286377 8.86813735961914 35.2168731689453 9.49165534973145 2.46659588813782 8.13523483276367 10.8988990783691 1.83015108108521 20.7684803009033 1.27757692337036 20.2799644470215 16.1304111480713 13.3484573364258 6.81516742706299 31.6201038360596 4.43572950363159 4.58984613418579 0.941066741943359 1.09628009796143 7.33224868774414 67.9507293701172 2.22717833518982 12.6838006973267 1.90599882602692 2.65052533149719 8.83069896697998 5.31595897674561 21.9768600463867 24.6718788146973 6.40959024429321 5.43706703186035 15.5448846817017 6.01975202560425 3.25844216346741 3.6406044960022 4.45793962478638 10.3883943557739 14.9068431854248 49.2957496643066 35.6121482849121 15.4051322937012 5.53128242492676 6.5834584236145 24.320348739624 30.2746162414551 8.41883659362793 42.5699005126953 30.2733135223389 17.4121265411377 6.34720945358276 16.4685707092285 7.7808632850647 46.4370956420898 17.0107021331787 9.34466457366943 19.9258689880371 14.9463443756104 9.15710735321045 11.9824361801147 15.9440774917603 37.1040344238281 10.4831323623657 3.3957085609436 29.7984828948975 13.9156408309937 52.2023963928223 9.27355003356934 6.6749210357666 7.51043081283569 49.3465042114258 5.4494194984436 19.6806659698486 4.17353677749634 8.05345630645752 1.8890209197998 31.9636611938477 18.6175937652588 14.0148601531982 7.86040353775024 0.469715088605881 14.021746635437 11.3452463150024 32.4288215637207 7.17828416824341 11.1732749938965 5.072913646698 9.83976745605469 8.26213932037354 3.04564499855042 27.8741149902344 31.8711490631104 20.2229843139648 16.0942726135254 25.984827041626 15.4788017272949 37.1332321166992 33.9986801147461 19.197437286377 11.224422454834 1.84304988384247 27.7742404937744 8.68100070953369 7.80723571777344 47.5952949523926 16.7321166992188 17.9921569824219 5.44006681442261 15.3093194961548 9.90076637268066 34.477954864502 1.42021059989929 8.54602718353271 29.4931907653809 3.77119421958923 28.870454788208 12.9606809616089 12.178915977478 18.7465915679932 3.02992415428162 5.98625898361206 10.5778617858887 6.35573863983154 13.2360696792603 10.0678043365479 18.5180397033691 18.0982551574707 8.77694892883301 13.4132595062256 14.2878742218018 4.15905475616455 19.2052631378174 3.34903645515442 10.1651830673218 2.77777457237244 11.8721380233765 13.5547189712524 43.8108825683594 1.58924198150635 22.2059001922607 10.8286867141724 11.000527381897 36.238941192627 7.89006280899048 15.3764762878418 9.64281463623047 20.7663307189941 23.4690570831299 9.16739749908447 22.4824352264404 7.07571744918823 1.51988232135773 20.7700881958008 1.52316236495972 4.65049076080322 5.02996015548706 12.1895761489868 22.5102825164795

ENSG00000231389.6 918.382385253906 826.917297363281 139.626388549805 256.663177490234 382.153869628906 408.291198730469 91.4992294311523 377.7421875 129.259613037109 268.858337402344 279.873046875 199.510162353516 683.338439941406 118.892036437988 155.625640869141 323.533996582031 11.370379447937 638.806579589844 306.247467041016 247.604507446289 521.963684082031 121.291366577148 504.322113037109 205.785034179688 11.2150650024414 84.4895706176758 468.703063964844 114.859016418457 483.432800292969 268.164978027344 368.421722412109 677.085876464844 404.514190673828 261.469970703125 47.4361915588379 199.99006652832 496.388244628906 479.639404296875 526.017883300781 316.060241699219 405.08251953125 809.879333496094 653.087341308594 97.2328109741211 153.260192871094 179.760986328125 120.881690979004 416.560852050781 190.90966796875 790.231140136719 106.621528625488 96.4407348632812 94.6493988037109 310.947296142578 239.467056274414 119.838249206543 69.8283462524414 701.242492675781 165.128082275391 854.371948242188 339.009216308594 152.584838867188 237.46711730957 112.001258850098 260.711029052734 320.271179199219 150.284713745117 37.2003440856934 379.221588134766 101.828681945801 37.009765625 434.056884765625 112.262390136719 309.400695800781 97.0510711669922 141.400695800781 48.7050514221191 1253.3408203125 41.7030487060547 300.214111328125 135.264205932617 635.383178710938 315.457153320312 142.935348510742 293.935485839844 1093.84875488281 263.079467773438 140.522094726562 663.164245605469 131.895599365234 248.606872558594 200.697479248047 16.547061920166 205.9208984375 39.2601165771484 369.052703857422 438.886138916016 143.852386474609 590.483642578125 222.849151611328 92.1182708740234 365.460205078125 182.695785522461 351.413879394531 40.9833564758301 182.631500244141 140.762496948242 33.9737815856934 682.398986816406 39.7456169128418 599.035705566406 347.436096191406 378.387420654297 222.498397827148 317.749206542969 189.313385009766 256.209503173828 9.50603485107422 32.9322967529297 49.8967781066895 618.566955566406 13.6496753692627 354.261810302734 164.048034667969 141.676818847656 61.337532043457 65.8231658935547 166.508438110352 230.829437255859 117.585098266602 39.9526672363281 489.000579833984 69.729248046875 55.4458427429199 100.808074951172 197.649017333984 335.236297607422 586.839782714844 500.406768798828 635.261169433594 187.967544555664 185.536804199219 138.574447631836 552.307373046875 738.293640136719 321.7080078125 721.202941894531 607.262084960938 551.127624511719 71.3436508178711 284.235412597656 127.775329589844 439.878814697266 544.671447753906 152.265502929688 272.101715087891 145.300415039062 243.512664794922 662.636108398438 926.179992675781 628.443908691406 111.671852111816 29.550573348999 544.60595703125 469.571319580078 1034.91564941406 187.048263549805 97.4492263793945 58.2252311706543 282.771026611328 231.449737548828 514.4658203125 35.5317420959473 84.890983581543 208.783676147461 868.356140136719 243.667373657227 206.698852539062 181.710235595703 109.595138549805 155.781280517578 247.288497924805 293.649230957031 195.686981201172 107.942680358887 114.268295288086 243.066848754883 400.954772949219 24.3568382263184 399.506469726562 651.78173828125 246.447174072266 498.372955322266 439.121612548828 729.967102050781 337.811889648438 708.656494140625 531.731750488281 170.325317382812 68.2258453369141 334.322204589844 331.274383544922 119.204566955566 2453.31884765625 408.025634765625 244.663162231445 172.768951416016 286.645538330078 350.282257080078 815.286010742188 33.6089706420898 129.794525146484 537.629211425781 217.193572998047 604.478637695312 247.598983764648 435.658660888672 518.271423339844 70.8297653198242 291.409729003906 405.705352783203 250.371627807617 301.864959716797 379.080139160156 492.884490966797 547.883666992188 118.524604797363 336.226348876953 411.898345947266 493.23876953125 261.881622314453 45.8847427368164 299.499786376953 129.526947021484 403.897613525391 273.979522705078 912.298400878906 26.167163848877 196.970672607422 28.1495037078857 189.673645019531 295.269317626953 78.2647094726562 120.524528503418 531.387023925781 233.648559570312 853.289001464844 114.080696105957 523.736206054688 176.814987182617 102.885864257812 526.456176757812 55.4154434204102 492.872253417969 156.91423034668 496.740325927734 392.114501953125

ENSG00000223865.9 1113.01525878906 1081.21984863281 401.306243896484 416.232299804688 656.935363769531 467.607604980469 157.463638305664 511.99658203125 172.973495483398 386.174713134766 466.572082519531 248.894882202148 941.827880859375 269.417297363281 239.673461914062 410.653381347656 20.2959785461426 955.994445800781 695.495788574219 314.002227783203 1005.65887451172 247.532730102539 906.032897949219 365.733123779297 13.9454612731934 158.410369873047 412.569946289062 282.471374511719 888.675109863281 449.224304199219 541.68408203125 1059.43371582031 444.500885009766 469.958099365234 112.636108398438 495.759887695312 686.234069824219 562.737548828125 620.410461425781 395.365386962891 642.260070800781 1769.0009765625 1532.7197265625 152.166061401367 241.825042724609 203.634765625 361.730010986328 628.171081542969 425.588409423828 1042.20324707031 263.901336669922 144.080123901367 90.1614379882812 367.922210693359 572.085205078125 154.031707763672 187.092758178711 704.235412597656 464.877532958984 1264.11376953125 515.906066894531 248.514495849609 295.108978271484 160.067367553711 435.725158691406 445.866882324219 239.918853759766 61.5453300476074 512.669189453125 338.273101806641 114.910186767578 579.779663085938 188.915878295898 400.685943603516 137.93147277832 264.017150878906 70.578369140625 2108.22778320312 102.181503295898 499.030578613281 271.621368408203 682.290222167969 458.106048583984 262.937408447266 573.393310546875 1160.02685546875 341.903106689453 196.327117919922 1273.38464355469 199.595840454102 802.9794921875 348.520294189453 27.7109909057617 388.570648193359 45.0508689880371 719.178405761719 835.373840332031 217.888137817383 927.345947265625 571.974670410156 145.321517944336 442.802581787109 801.271850585938 710.36083984375 129.958190917969 447.559387207031 390.193206787109 51.344841003418 773.354125976562 117.887214660645 699.395568847656 681.207763671875 874.527526855469 276.483764648438 680.935424804688 210.420623779297 348.111053466797 20.3605556488037 66.0231170654297 138.242431640625 898.647644042969 20.4606761932373 479.627685546875 261.908111572266 288.219940185547 147.685043334961 90.7112274169922 254.946792602539 310.007720947266 260.647094726562 105.153190612793 665.756774902344 134.088165283203 86.8948593139648 145.783096313477 259.211090087891 780.802734375 707.196472167969 729.511108398438 1033.4013671875 350.104095458984 362.700317382812 223.50114440918 811.981506347656 783.712585449219 593.978332519531 1662.74975585938 708.130126953125 880.852416992188 116.180976867676 353.016265869141 261.486389160156 820.55908203125 1232.92590332031 205.693664550781 409.812957763672 429.535064697266 425.123016357422 1282.77746582031 1175.72521972656 724.381469726562 180.196136474609 50.1513786315918 845.212036132812 505.988098144531 1255.39697265625 303.682037353516 118.060348510742 127.267082214355 802.370666503906 341.69384765625 912.451477050781 68.9114151000977 182.283401489258 258.519256591797 1418.28076171875 317.212738037109 369.232208251953 245.253402709961 181.306030273438 147.177841186523 524.989624023438 419.327911376953 395.242950439453 510.624755859375 162.69157409668 623.305358886719 730.208190917969 20.5380268096924 612.315612792969 1223.24560546875 382.310852050781 826.06005859375 761.350402832031 845.671813964844 728.021911621094 1128.67163085938 573.338989257812 293.292175292969 79.0624465942383 1222.37805175781 373.187164306641 248.372863769531 2828.46948242188 612.803039550781 849.816162109375 206.974258422852 1094.51452636719 495.248504638672 1078.97912597656 41.0192680358887 469.117340087891 895.406982421875 417.061157226562 786.651672363281 330.986877441406 627.143981933594 902.248413085938 85.5847396850586 462.981506347656 464.4482421875 426.781799316406 446.458160400391 675.304382324219 783.706848144531 992.293518066406 274.711151123047 449.302520751953 616.449951171875 632.365356445312 435.922973632812 82.6504135131836 535.555786132812 158.467071533203 545.335571289062 679.120483398438 1426.26940917969 47.5097465515137 424.349975585938 226.183395385742 259.07763671875 515.482788085938 158.294021606445 268.387512207031 759.110656738281 571.265563964844 1232.50659179688 195.75048828125 682.412658691406 257.456176757812 223.151443481445 730.56494140625 70.338623046875 607.184326171875 194.077133178711 915.828796386719 788.046997070312

ENSG00000196735.10 446.636322021484 449.309631347656 107.595184326172 71.950065612793 211.165618896484 295.246429443359 31.3851413726807 228.739501953125 50.9826736450195 132.975189208984 137.526123046875 131.168991088867 145.472198486328 43.0772819519043 58.2558784484863 145.645172119141 4.02995681762695 378.55517578125 74.4815979003906 120.135223388672 176.285675048828 91.5402450561523 242.682678222656 162.079574584961 3.71078252792358 32.6873664855957 66.8109512329102 39.497673034668 131.893005371094 106.752288818359 192.855514526367 200.744598388672 128.454177856445 89.5232925415039 14.0229015350342 229.2431640625 87.5730361938477 314.274505615234 146.987869262695 102.939270019531 36.3855285644531 672.794738769531 364.247741699219 45.0362663269043 46.1544761657715 61.7724189758301 152.632537841797 96.6123046875 68.5811386108398 130.414932250977 19.6256847381592 41.8946533203125 14.936900138855 133.584213256836 194.55827331543 56.2789115905762 17.8750381469727 382.368865966797 90.3069458007812 357.840454101562 159.673690795898 75.9337463378906 77.1805267333984 41.5107307434082 207.322448730469 103.06128692627 69.9107818603516 7.70630550384521 192.483642578125 80.7614517211914 27.5133533477783 211.124664306641 52.9574775695801 113.335372924805 55.4863891601562 90.959846496582 23.6372108459473 339.112182617188 20.0719966888428 119.992416381836 54.9213790893555 175.636154174805 127.748397827148 88.6065139770508 65.1581802368164 328.073455810547 203.064346313477 66.3108367919922 412.475189208984 68.859130859375 124.564407348633 63.0750885009766 7.18838405609131 85.0789794921875 22.418851852417 99.5164947509766 211.560852050781 32.7266883850098 610.386657714844 93.0721664428711 17.795970916748 68.4916915893555 137.397430419922 148.501419067383 39.4439125061035 95.7195358276367 70.9269409179688 4.34791564941406 236.226760864258 32.0195960998535 489.781494140625 262.509796142578 175.700454711914 60.8818168640137 165.763336181641 89.0281372070312 54.1896743774414 3.17325949668884 12.01686668396 9.74148082733154 256.260101318359 4.91051864624023 152.045608520508 62.0468406677246 39.6427230834961 16.9310264587402 18.1144599914551 51.2028160095215 49.5060081481934 46.2803497314453 17.2488975524902 156.73161315918 25.1401424407959 6.45450019836426 61.5197448730469 73.5955200195312 51.2907829284668 112.741470336914 216.018341064453 164.326797485352 110.86994934082 78.6899261474609 33.9674491882324 114.130722045898 441.854644775391 129.299194335938 441.296630859375 263.3095703125 272.356964111328 34.6505813598633 63.8771781921387 89.1799011230469 333.493103027344 229.095626831055 83.5807037353516 128.79118347168 147.692977905273 114.137168884277 367.850006103516 471.382263183594 160.166381835938 31.2059841156006 6.32240009307861 102.899971008301 98.464469909668 510.545013427734 74.177978515625 26.1659145355225 35.6198654174805 89.6586303710938 75.3829193115234 167.447296142578 8.4510612487793 26.9345874786377 116.246002197266 329.028778076172 82.23388671875 130.865295410156 88.2401733398438 18.337230682373 97.6863479614258 81.8091430664062 176.364044189453 150.901458740234 147.370712280273 36.0785980224609 35.7346992492676 104.981758117676 2.85922193527222 160.593521118164 136.684921264648 78.788818359375 154.922729492188 190.91194152832 207.729858398438 143.146194458008 330.785491943359 192.045669555664 23.7819881439209 11.097731590271 200.481414794922 118.73876953125 33.2346267700195 1012.87268066406 131.55549621582 198.084564208984 57.9058532714844 239.00846862793 68.8984298706055 151.393417358398 6.85018491744995 105.785018920898 236.510681152344 26.600118637085 385.011016845703 91.0966873168945 75.259147644043 128.626968383789 26.0516700744629 83.3147125244141 208.225326538086 165.90625 154.225769042969 199.858383178711 200.039428710938 498.902130126953 74.7080001831055 110.760284423828 211.358856201172 278.325347900391 104.258781433105 15.0079917907715 180.520965576172 41.1060981750488 246.377258300781 145.674774169922 372.737030029297 7.57971477508545 97.3063125610352 36.113883972168 39.5801048278809 189.846313476562 44.4072151184082 44.4232940673828 108.054702758789 179.343978881836 222.604187011719 19.9595336914062 177.052520751953 70.4117126464844 19.4235801696777 345.956420898438 21.2984313964844 273.229095458984 84.6489028930664 404.784149169922 85.0753860473633

ENSG00000237541.3 621.546752929688 224.189392089844 75.902702331543 108.389343261719 353.810852050781 57.3925895690918 69.5432586669922 221.640518188477 9.89929962158203 2.71466517448425 218.179077148438 35.6494293212891 530.049926757812 52.0538482666016 89.2172012329102 180.963516235352 1.67128336429596 10.4255495071411 306.236663818359 24.8335800170898 449.251556396484 8.70711326599121 321.641326904297 29.6591396331787 4.20333099365234 49.0215759277344 39.845043182373 53.4646110534668 44.3747215270996 31.705228805542 7.10398817062378 124.941444396973 65.7317276000977 41.8364486694336 14.3448219299316 243.66455078125 450.067291259766 30.6800708770752 211.150177001953 218.48405456543 186.1533203125 95.8565902709961 340.431243896484 44.9300575256348 50.7730484008789 97.4711761474609 57.7745628356934 12.931300163269 146.341705322266 585.275756835938 94.9704132080078 13.9482717514038 9.68787670135498 32.8682861328125 97.5377578735352 0.753535568714142 99.5930633544922 71.1163864135742 154.50895690918 37.2332611083984 7.41232013702393 136.749893188477 123.225708007812 19.3076915740967 23.8850440979004 39.1921005249023 167.521224975586 13.0067453384399 162.19319152832 30.1225490570068 14.6107883453369 107.128494262695 7.38886070251465 202.214431762695 30.2942562103271 72.8896408081055 9.66719055175781 560.229858398438 47.7056770324707 110.020790100098 49.4114646911621 360.894989013672 2.16275095939636 27.0351696014404 219.003204345703 119.988754272461 2.72950863838196 15.2134246826172 123.814002990723 2.90696978569031 36.1523628234863 88.2746047973633 8.43914794921875 74.3591690063477 6.48996162414551 103.906669616699 178.305221557617 55.379467010498 133.343185424805 119.16202545166 103.652000427246 66.4961090087891 248.049438476562 152.173141479492 33.2649383544922 181.94921875 21.7095069885254 8.52655124664307 438.762329101562 8.85156154632568 14.0532474517822 63.2762145996094 130.568466186523 226.238235473633 63.8193740844727 173.238128662109 80.2604904174805 2.08974504470825 12.1179714202881 53.3214530944824 62.8815155029297 2.60667562484741 7.84913778305054 16.7761058807373 210.613891601562 100.22200012207 17.3267688751221 7.38574504852295 13.9852523803711 81.217041015625 17.7716541290283 233.651870727539 0.660513758659363 35.0095367431641 22.4168148040771 91.5758514404297 199.764938354492 473.516937255859 555.18701171875 291.626617431641 1.67358314990997 9.03149795532227 64.5543060302734 246.990356445312 128.487884521484 211.75422668457 382.612457275391 122.13306427002 150.071990966797 7.99837493896484 173.072616577148 22.0687408447266 221.834533691406 70.4636535644531 21.0065898895264 189.024444580078 128.280975341797 23.814811706543 7.99889516830444 183.487274169922 404.283752441406 49.3682670593262 33.7251396179199 86.5285263061523 106.654708862305 18.3155040740967 23.58571434021 27.1757640838623 0.654029846191406 211.818603515625 100.768997192383 168.973876953125 5.58986473083496 144.628662109375 35.869514465332 17.0640716552734 21.2986450195312 103.994361877441 24.2719249725342 115.9892578125 27.0440845489502 148.416030883789 1.34293234348297 94.9969635009766 193.070037841797 51.6421928405762 28.1350898742676 543.435485839844 12.7896661758423 251.870162963867 653.453491210938 358.823913574219 403.319854736328 87.7470092773438 192.822967529297 23.2671737670898 73.2091674804688 535.839965820312 65.8796920776367 50.5110321044922 443.772247314453 158.057968139648 140.534454345703 2080.82153320312 296.676544189453 137.533615112305 109.433151245117 28.3266944885254 149.619537353516 933.723449707031 6.5005841255188 28.5230865478516 440.510040283203 139.511154174805 4.83233833312988 111.204284667969 457.629119873047 647.012329101562 39.761402130127 201.416900634766 2.93955373764038 54.1554908752441 216.892013549805 362.901397705078 56.8793144226074 19.2942199707031 99.3536148071289 134.716827392578 58.038990020752 84.4587707519531 167.929656982422 20.444221496582 203.831161499023 90.0740280151367 100.101951599121 2.46532392501831 59.5289535522461 7.20522880554199 145.704391479492 21.0295791625977 202.010971069336 6.54235172271729 62.2833023071289 48.0664710998535 453.494445800781 31.842134475708 761.042907714844 85.4372863769531 736.503845214844 21.8125514984131 85.5359420776367 9.17822265625 29.0763759613037 139.11637878418 1.71976685523987 30.5130100250244 215.539199829102

ENSG00000237541.3.1 621.546752929688 224.189392089844 75.902702331543 108.389343261719 353.810852050781 57.3925895690918 69.5432586669922 221.640518188477 9.89929962158203 2.71466517448425 218.179077148438 35.6494293212891 530.049926757812 52.0538482666016 89.2172012329102 180.963516235352 1.67128336429596 10.4255495071411 306.236663818359 24.8335800170898 449.251556396484 8.70711326599121 321.641326904297 29.6591396331787 4.20333099365234 49.0215759277344 39.845043182373 53.4646110534668 44.3747215270996 31.705228805542 7.10398817062378 124.941444396973 65.7317276000977 41.8364486694336 14.3448219299316 243.66455078125 450.067291259766 30.6800708770752 211.150177001953 218.48405456543 186.1533203125 95.8565902709961 340.431243896484 44.9300575256348 50.7730484008789 97.4711761474609 57.7745628356934 12.931300163269 146.341705322266 585.275756835938 94.9704132080078 13.9482717514038 9.68787670135498 32.8682861328125 97.5377578735352 0.753535568714142 99.5930633544922 71.1163864135742 154.50895690918 37.2332611083984 7.41232013702393 136.749893188477 123.225708007812 19.3076915740967 23.8850440979004 39.1921005249023 167.521224975586 13.0067453384399 162.19319152832 30.1225490570068 14.6107883453369 107.128494262695 7.38886070251465 202.214431762695 30.2942562103271 72.8896408081055 9.66719055175781 560.229858398438 47.7056770324707 110.020790100098 49.4114646911621 360.894989013672 2.16275095939636 27.0351696014404 219.003204345703 119.988754272461 2.72950863838196 15.2134246826172 123.814002990723 2.90696978569031 36.1523628234863 88.2746047973633 8.43914794921875 74.3591690063477 6.48996162414551 103.906669616699 178.305221557617 55.379467010498 133.343185424805 119.16202545166 103.652000427246 66.4961090087891 248.049438476562 152.173141479492 33.2649383544922 181.94921875 21.7095069885254 8.52655124664307 438.762329101562 8.85156154632568 14.0532474517822 63.2762145996094 130.568466186523 226.238235473633 63.8193740844727 173.238128662109 80.2604904174805 2.08974504470825 12.1179714202881 53.3214530944824 62.8815155029297 2.60667562484741 7.84913778305054 16.7761058807373 210.613891601562 100.22200012207 17.3267688751221 7.38574504852295 13.9852523803711 81.217041015625 17.7716541290283 233.651870727539 0.660513758659363 35.0095367431641 22.4168148040771 91.5758514404297 199.764938354492 473.516937255859 555.18701171875 291.626617431641 1.67358314990997 9.03149795532227 64.5543060302734 246.990356445312 128.487884521484 211.75422668457 382.612457275391 122.13306427002 150.071990966797 7.99837493896484 173.072616577148 22.0687408447266 221.834533691406 70.4636535644531 21.0065898895264 189.024444580078 128.280975341797 23.814811706543 7.99889516830444 183.487274169922 404.283752441406 49.3682670593262 33.7251396179199 86.5285263061523 106.654708862305 18.3155040740967 23.58571434021 27.1757640838623 0.654029846191406 211.818603515625 100.768997192383 168.973876953125 5.58986473083496 144.628662109375 35.869514465332 17.0640716552734 21.2986450195312 103.994361877441 24.2719249725342 115.9892578125 27.0440845489502 148.416030883789 1.34293234348297 94.9969635009766 193.070037841797 51.6421928405762 28.1350898742676 543.435485839844 12.7896661758423 251.870162963867 653.453491210938 358.823913574219 403.319854736328 87.7470092773438 192.822967529297 23.2671737670898 73.2091674804688 535.839965820312 65.8796920776367 50.5110321044922 443.772247314453 158.057968139648 140.534454345703 2080.82153320312 296.676544189453 137.533615112305 109.433151245117 28.3266944885254 149.619537353516 933.723449707031 6.5005841255188 28.5230865478516 440.510040283203 139.511154174805 4.83233833312988 111.204284667969 457.629119873047 647.012329101562 39.761402130127 201.416900634766 2.93955373764038 54.1554908752441 216.892013549805 362.901397705078 56.8793144226074 19.2942199707031 99.3536148071289 134.716827392578 58.038990020752 84.4587707519531 167.929656982422 20.444221496582 203.831161499023 90.0740280151367 100.101951599121 2.46532392501831 59.5289535522461 7.20522880554199 145.704391479492 21.0295791625977 202.010971069336 6.54235172271729 62.2833023071289 48.0664710998535 453.494445800781 31.842134475708 761.042907714844 85.4372863769531 736.503845214844 21.8125514984131 85.5359420776367 9.17822265625 29.0763759613037 139.11637878418 1.71976685523987 30.5130100250244 215.539199829102

ENSG00000179344.15 572.640930175781 661.417236328125 209.137832641602 56.4129409790039 339.661163330078 560.692565917969 45.8468475341797 48.6053848266602 66.8942642211914 312.071899414062 164.698791503906 90.7761154174805 281.935333251953 99.7684936523438 158.538696289062 53.7980461120605 9.40076446533203 707.809631347656 118.286354064941 183.952651977539 315.970550537109 262.062469482422 463.94384765625 399.978149414062 5.47304439544678 31.6404151916504 88.0990829467773 114.866897583008 394.673370361328 136.565170288086 283.588745117188 392.273254394531 478.991943359375 209.325332641602 77.9131546020508 229.205429077148 251.901107788086 385.314666748047 84.4408111572266 115.169242858887 135.836883544922 688.582153320312 633.025268554688 45.9043502807617 64.6934356689453 82.7590255737305 182.047470092773 301.864074707031 206.47639465332 231.400360107422 43.3207244873047 87.0306549072266 60.7411575317383 121.295631408691 389.392974853516 130.434799194336 97.9728012084961 703.602600097656 206.316177368164 926.62744140625 410.987701416016 68.8939971923828 266.900360107422 175.383453369141 209.106246948242 297.821166992188 86.2000885009766 15.7013912200928 259.861907958984 253.323348999023 36.4952545166016 187.401290893555 212.473190307617 157.437545776367 55.0815391540527 85.6874923706055 46.3950424194336 696.632507324219 20.0087947845459 220.456939697266 149.442840576172 185.180191040039 341.646697998047 140.045623779297 220.755172729492 388.854095458984 229.979187011719 64.8776550292969 638.618225097656 350.190795898438 342.501586914062 37.8539810180664 11.039755821228 164.04460144043 40.5227394104004 315.609069824219 236.889465332031 101.837509155273 340.935791015625 159.149337768555 43.4939193725586 36.577808380127 101.794845581055 580.039489746094 68.8083877563477 308.787536621094 56.3597564697266 4.25944042205811 131.564743041992 24.6979064941406 674.693969726562 164.516983032227 127.975372314453 249.040618896484 308.031494140625 47.3750877380371 165.122619628906 5.6665358543396 18.7877769470215 19.2185688018799 825.548217773438 1.56950676441193 294.249847412109 132.925506591797 75.5739288330078 29.4684562683105 52.078369140625 254.603149414062 203.464569091797 42.9176025390625 35.6942291259766 177.903091430664 129.305969238281 16.1744575500488 83.3643341064453 152.700042724609 83.2293167114258 137.2265625 157.146133422852 330.346374511719 103.296440124512 124.429878234863 82.1962280273438 201.638427734375 365.933685302734 185.798934936523 827.56640625 463.984649658203 196.20198059082 76.8523941040039 97.0531463623047 96.2290725708008 323.023162841797 380.736602783203 129.996215820312 200.936492919922 108.849784851074 103.831207275391 377.905548095703 510.670257568359 350.436218261719 96.0683746337891 14.1896724700928 81.0345153808594 254.531219482422 1032.65087890625 127.054740905762 20.9276943206787 82.5290298461914 122.928901672363 253.482650756836 164.000869750977 27.6829471588135 68.5201568603516 81.4826354980469 527.252258300781 403.403289794922 50.1704750061035 72.2556762695312 46.6748504638672 78.450439453125 90.4033508300781 344.821899414062 101.105224609375 130.070571899414 79.0997924804688 149.228713989258 139.932739257812 22.1167392730713 350.7001953125 236.208419799805 76.9904479980469 214.73078918457 71.7349472045898 408.321441650391 268.267211914062 970.257568359375 386.642211914062 124.637825012207 13.8873090744019 186.920455932617 194.773498535156 55.9750747680664 877.832702636719 343.642639160156 102.58846282959 39.6626510620117 430.233551025391 264.639038085938 299.938659667969 10.4973068237305 155.62548828125 371.378295898438 56.2146987915039 428.791229248047 100.803611755371 322.731018066406 326.599395751953 49.2985305786133 258.810943603516 385.827697753906 127.94295501709 86.4163055419922 133.817031860352 158.709686279297 549.612915039062 39.4931297302246 202.289459228516 429.214813232422 200.286071777344 282.539825439453 25.2594604492188 276.097595214844 41.8147468566895 235.353790283203 424.561614990234 402.328033447266 14.3945055007935 216.684967041016 63.5557289123535 76.8394165039062 325.428741455078 79.5265350341797 194.258712768555 152.269821166992 452.393157958984 350.648010253906 108.761993408203 263.061950683594 156.923004150391 26.3402423858643 355.491668701172 24.8147506713867 146.965347290039 111.368766784668 617.884399414062 126.783226013184

ENSG00000204287.12 9241.73828125 9044.6044921875 2965.12841796875 2949.0576171875 5935.78369140625 4743.97998046875 1051.15234375 5660.486328125 2529.07592773438 2996.470703125 3842.96337890625 1893.73425292969 5220.4912109375 2220.681640625 1639.13146972656 3711.67065429688 101.523254394531 7271.5078125 5265.0439453125 2535.52685546875 6840.08642578125 1684.12878417969 5364.5625 3108.029296875 122.454109191895 1093.75476074219 4722.341796875 1310.42321777344 4958.48974609375 3388.74145507812 4023.2646484375 6986.06982421875 2679.00805664062 2449.16796875 804.259521484375 3283.63818359375 5508.59912109375 4984.2138671875 4864.21044921875 3712.3037109375 4760.33203125 8841.9921875 7821.33349609375 1121.28979492188 1497.27307128906 2266.546875 3110.46801757812 4943.77783203125 2792.40673828125 8354.37890625 1375.11755371094 897.675537109375 1254.88488769531 3049.46313476562 5419.3017578125 1279.0595703125 1553.30236816406 6721.0341796875 3131.95263671875 8160.966796875 4210.5888671875 2169.39892578125 3240.69775390625 1612.12683105469 5920.0810546875 3892.08447265625 1741.59423828125 424.386596679688 4250.9580078125 2155.00927734375 482.270538330078 4220.5615234375 1421.08410644531 3580.00390625 1143.20483398438 1535.82019042969 613.692138671875 9081.3154296875 759.994873046875 3261.765625 2127.71655273438 5042.17333984375 3092.34399414062 1814.99365234375 3319.2353515625 7723.94580078125 3164.43237304688 1787.35510253906 7007.966796875 2262.41284179688 4690.89599609375 2658.87426757812 190.909362792969 2910.65014648438 310.416687011719 5020.064453125 6215.31982421875 1626.59606933594 7210.302734375 3889.38305664062 925.896789550781 2911.12231445312 4222.3447265625 5230.2021484375 874.729370117188 3769.751953125 3466.43896484375 496.685333251953 8737.0576171875 842.20654296875 6948.689453125 7144.63720703125 4986.3359375 2688.8701171875 4294.66357421875 2799.96313476562 4213.87353515625 132.942733764648 457.981658935547 667.908569335938 7541.98828125 358.204071044922 2880.73657226562 2419.626953125 2105.65307617188 971.494812011719 1149.98669433594 1303.13562011719 2614.42797851562 2211.73168945312 647.02783203125 5382.52294921875 933.135925292969 760.415405273438 1055.63635253906 2997.31494140625 5547.68505859375 6871.2548828125 6317.06591796875 6422.576171875 2212.23999023438 2701.52026367188 1601.02185058594 4989.865234375 7332.4892578125 5310.15673828125 13083.4833984375 5459.18603515625 6187.37548828125 720.655944824219 3797.24096679688 1741.32873535156 5883.064453125 9012.078125 2190.88598632812 2859.6103515625 3031.263671875 3949.70849609375 8801.8896484375 8862.0517578125 6525.26220703125 1098.17590332031 260.255615234375 7305.6669921875 4877.68798828125 13766.9765625 2388.87109375 1290.33459472656 759.519836425781 6033.81689453125 3093.9892578125 5398.4111328125 302.736846923828 1374.5322265625 1755.03735351562 10170.818359375 3329.810546875 3235.01879882812 1664.81176757812 1020.99572753906 1581.53771972656 3917.45947265625 2897.70654296875 2739.27490234375 3285.39990234375 1143.72229003906 2546.8505859375 5562.18017578125 273.934783935547 4681.37890625 6793.443359375 3615.23291015625 6886.955078125 4566.12158203125 6302.96484375 5028.85205078125 7500.83544921875 6077.98095703125 2134.0224609375 1236.28979492188 8631.2890625 3910.5703125 1737.76818847656 23746.392578125 4492.29296875 5840.0205078125 1746.13488769531 6268.12939453125 3122.17578125 11526.8125 328.541900634766 2364.16479492188 5090.26171875 2603.80151367188 6518.77001953125 3012.18872070312 4099.8857421875 5476.0966796875 836.132263183594 4309.30712890625 5097.78271484375 3327.45483398438 4123.2333984375 4747.17529296875 4869.171875 6764.3056640625 1940.63366699219 3672.52490234375 4682.98046875 5976.306640625 3554.75244140625 545.789123535156 4246.40625 1326.07360839844 3853.63647460938 3295.8564453125 13662.6826171875 288.360534667969 2748.17651367188 899.885070800781 2154.25439453125 3553.83349609375 1113.90832519531 2186.69189453125 7304.48095703125 3662.64233398438 7882.14111328125 1593.72814941406 5669.24365234375 1779.13073730469 4794.72216796875 5576.0673828125 530.864379882812 4979.5791015625 2173.5517578125 7539.994140625 5907.15234375

ENSG00000196126.9 4071.86743164062 5771.91552734375 2470.1123046875 1588.95056152344 2500.9169921875 3291.20654296875 602.346862792969 2005.515625 866.693603515625 2242.87280273438 2519.70043945312 1058.4306640625 3834.740234375 1052.47119140625 813.204040527344 1563.62866210938 78.4815216064453 5055.56494140625 1455.91137695312 1849.10131835938 3225.63818359375 1456.87915039062 3926.5537109375 2509.22387695312 53.6020164489746 490.479064941406 2966.84912109375 851.051452636719 4507.28076171875 2064.02319335938 2219.32861328125 5741.93603515625 2801.54614257812 1730.26428222656 760.837341308594 2334.60668945312 1393.67626953125 2779.72875976562 1954.86450195312 1066.0048828125 1595.98474121094 5896.98828125 5557.77880859375 719.294311523438 674.070373535156 1072.4638671875 1546.36499023438 3278.91088867188 1996.4091796875 3822.3408203125 503.714233398438 702.471984863281 595.347290039062 1780.11010742188 3083.599609375 1105.33923339844 847.441467285156 5215.7421875 1781.61462402344 6790.13427734375 3198.86376953125 815.632446289062 1642.58679199219 1157.14624023438 2293.48046875 2537.93139648438 791.08203125 225.059967041016 2736.77514648438 2070.17846679688 338.670013427734 2388.38842773438 1286.7646484375 1508.78479003906 1020.05871582031 992.775634765625 392.765075683594 6013.47900390625 364.518371582031 1474.76525878906 1248.23999023438 2482.58178710938 2373.15356445312 1406.79833984375 1901.90478515625 5612.8642578125 2182.12548828125 1053.21520996094 5890.46484375 1333.7880859375 2513.28442382812 1198.58703613281 93.0980758666992 926.477111816406 168.088226318359 3459.02807617188 2253.0224609375 713.412292480469 5491.13525390625 1560.15759277344 447.448394775391 1983.04357910156 2792.11596679688 2934.35034179688 524.243347167969 1918.87670898438 1923.29016113281 243.166580200195 3407.6748046875 469.162200927734 5570.18115234375 3067.326171875 3372.30932617188 1772.42553710938 2932.41284179688 965.470153808594 1442.94860839844 115.027984619141 219.725677490234 258.433929443359 4497.5625 136.07487487793 1878.86645507812 1769.67932128906 561.208251953125 312.598907470703 526.5771484375 1234.34838867188 3199.13745117188 789.912170410156 303.067474365234 2159.234375 922.909240722656 144.819976806641 801.525695800781 1395.56958007812 1407.06652832031 1866.07336425781 2686.759765625 2732.03369140625 1239.19421386719 1473.95397949219 935.227722167969 3273.73681640625 4703.95263671875 3379.49633789062 6398.0458984375 4675.41162109375 4311.64892578125 434.102416992188 1558.10388183594 1199.01184082031 4930.42822265625 5683.708984375 1225.29565429688 1413.96374511719 1310.064453125 2302.51098632812 4931.0361328125 6629.0302734375 3529.40087890625 643.916625976562 92.5189666748047 5120.0859375 2644.42822265625 7457.521484375 1733.81262207031 290.382904052734 637.799194335938 3198.17578125 1180.30126953125 2324.94555664062 158.29817199707 529.751831054688 1212.61730957031 6434.37548828125 2160.74169921875 1948.74182128906 1088.40698242188 440.746032714844 360.993316650391 2030.87536621094 2277.59643554688 1354.38415527344 1550.87194824219 572.575866699219 2711.84155273438 1870.9169921875 69.8350448608398 2166.96948242188 2585.68994140625 1171.12463378906 2937.4580078125 3190.2529296875 3463.77221679688 3598.08520507812 6037.09326171875 2987.6064453125 1627.31176757812 169.675842285156 4742.5087890625 1520.28479003906 507.783050537109 8036.1650390625 2956.73583984375 3321.77075195312 648.148132324219 4957.54296875 1950.12243652344 3054.09497070312 150.206604003906 1827.26232910156 3181.58544921875 730.946716308594 3294.05737304688 1336.18493652344 1672.03503417969 2130.76977539062 581.140014648438 2419.26953125 2883.26342773438 1641.01818847656 1495.16345214844 2099.34326171875 3400.29809570312 4017.91357421875 973.166748046875 1675.546875 3651.54565429688 3330.68579101562 1818.65197753906 234.196228027344 1902.44494628906 484.939636230469 2188.27319335938 2851.08935546875 8687.3798828125 139.686782836914 1319.43322753906 740.500061035156 694.186950683594 2487.2080078125 480.836547851562 1096.42736816406 1770.12158203125 3376.134765625 5550.7861328125 531.776428222656 2657.47705078125 1112.52795410156 520.721801757812 3507.24658203125 275.795959472656 3603.97534179688 1060.38708496094 4863.36083984375 3531.54736328125

ENSG00000198502.5 1826.13977050781 3723.06201171875 1270.62622070312 379.544830322266 605.854614257812 747.758972167969 169.990951538086 262.195953369141 147.002349853516 1422.21362304688 1703.02661132812 169.085739135742 1027.87084960938 337.4794921875 220.601989746094 310.809112548828 9.78385162353516 3013.67578125 777.65283203125 1434.96142578125 918.792907714844 170.175048828125 767.041442871094 1118.19445800781 13.9006357192993 127.829116821289 584.6416015625 697.506530761719 2278.75341796875 415.698272705078 1264.31652832031 689.443786621094 245.854537963867 290.065551757812 788.047119140625 494.533813476562 928.952392578125 469.103637695312 470.557952880859 409.947235107422 1061.17504882812 4519.00732421875 1563.48852539062 99.2368316650391 186.182403564453 293.739501953125 199.576446533203 1764.23913574219 1668.72595214844 2358.88647460938 250.905364990234 384.045959472656 458.391510009766 309.757476806641 1945.60485839844 742.645812988281 323.974487304688 4521.26220703125 790.486267089844 5252.24560546875 1976.18823242188 270.520965576172 1067.27429199219 129.983505249023 469.654418945312 390.342895507812 284.547271728516 63.4538688659668 548.389526367188 280.902252197266 68.8356628417969 332.350128173828 957.261779785156 591.527587890625 129.855972290039 180.258636474609 62.1307373046875 5149.2548828125 96.7020797729492 471.584716796875 847.452880859375 671.945068359375 1636.6806640625 647.396362304688 808.644714355469 700.077758789062 302.078643798828 202.50732421875 2290.5283203125 534.980346679688 2316.2587890625 305.962432861328 34.2084197998047 336.911224365234 83.1894454956055 2343.27416992188 790.217041015625 278.013153076172 4045.22924804688 512.109741210938 211.118621826172 250.774429321289 579.19677734375 629.596374511719 102.592956542969 1025.00280761719 273.664459228516 47.3244476318359 957.713745117188 87.8549499511719 2842.39526367188 444.415313720703 463.609283447266 510.659393310547 1717.00061035156 158.89973449707 915.293151855469 20.1216506958008 72.5304718017578 141.506469726562 3178.06567382812 11.6917676925659 1002.80169677734 621.7578125 297.874053955078 193.770782470703 210.291290283203 78.0401153564453 1490.7265625 225.136260986328 86.1883010864258 719.755249023438 82.5537338256836 97.2801361083984 341.831329345703 352.858795166016 857.210632324219 1037.22705078125 981.229125976562 886.201904296875 236.61100769043 309.677947998047 335.653747558594 892.2470703125 2775.79638671875 1203.97912597656 2045.78112792969 573.416870117188 675.737670898438 49.4722518920898 564.325500488281 152.487060546875 831.353271484375 1051.49060058594 487.952850341797 404.449615478516 161.259704589844 443.1943359375 586.958129882812 3065.61328125 997.877563476562 396.031066894531 50.09765625 587.67529296875 597.446594238281 3694.13842773438 231.581115722656 44.338020324707 102.478828430176 1115.64233398438 453.562713623047 858.092041015625 37.9660339355469 251.803268432617 519.993713378906 889.554809570312 375.606109619141 247.540420532227 174.054672241211 276.679565429688 151.408950805664 585.585021972656 1115.77014160156 167.180267333984 365.730621337891 449.355712890625 654.974365234375 1343.494140625 51.3151741027832 1428.79711914062 1275.96801757812 711.960144042969 1215.46850585938 391.943969726562 2807.95385742188 1549.48449707031 5849.2314453125 1062.15686035156 455.724212646484 102.748237609863 1352.96472167969 647.280700683594 236.075485229492 3538.75317382812 1034.69409179688 297.089935302734 203.873352050781 1655.91149902344 1929.46911621094 1724.73913574219 45.1181640625 234.3310546875 1247.89208984375 425.156951904297 615.786804199219 504.541137695312 852.931884765625 1250.41271972656 294.821685791016 487.649566650391 703.981140136719 260.258575439453 1072.65466308594 601.777221679688 620.888061523438 883.488037109375 254.327392578125 367.32861328125 1766.15881347656 515.580200195312 537.364379882812 69.7033996582031 639.312683105469 186.872772216797 248.918685913086 2356.4677734375 1968.73449707031 41.8900909423828 303.510284423828 117.28719329834 379.417236328125 1330.72473144531 109.229789733887 675.478942871094 1045.67297363281 358.962127685547 1829.998046875 290.953979492188 1285.95397949219 543.455871582031 298.661071777344 765.641418457031 202.854675292969 394.205383300781 208.475204467773 4073.88256835938 673.042175292969

ENSG00000204592.8 1603.869140625 1504.736328125 700.740417480469 697.329833984375 1348.81884765625 1320.87829589844 411.564788818359 746.590942382812 583.472961425781 798.081665039062 680.263000488281 620.543884277344 1492.32604980469 1024.37048339844 947.337585449219 1732.29907226562 144.825759887695 1864.13684082031 1259.24792480469 597.760070800781 1335.16076660156 1489.62658691406 1145.66027832031 764.690185546875 223.364135742188 505.41259765625 1195.14575195312 710.838439941406 849.324401855469 1003.76037597656 1869.484375 2168.5859375 1485.10009765625 681.930847167969 772.862426757812 1209.76391601562 1063.9912109375 1741.4755859375 1249.21118164062 835.205200195312 866.257080078125 2382.60986328125 2234.63525390625 576.022644042969 837.550964355469 560.610717773438 882.302429199219 1220.63684082031 722.220275878906 1003.96954345703 758.556213378906 355.716003417969 256.760559082031 1866.681640625 1060.92993164062 812.080200195312 1328.83618164062 1016.04357910156 1097.572265625 1380.47192382812 856.687805175781 547.495910644531 1018.14770507812 350.986785888672 484.521697998047 817.327087402344 575.597717285156 309.553833007812 666.050903320312 1055.77551269531 408.476928710938 1083.73608398438 506.970672607422 717.100524902344 1400.11535644531 464.415069580078 459.654266357422 3272.76782226562 215.251815795898 1150.15234375 708.064514160156 2329.81298828125 1282.62255859375 616.626892089844 744.438415527344 2238.87915039062 1260.166015625 671.453369140625 1525.48815917969 724.490661621094 673.818481445312 764.705749511719 226.539916992188 835.169738769531 362.687744140625 1062.4814453125 1226.54370117188 785.13720703125 1674.83044433594 1082.81176757812 779.412414550781 910.854614257812 1332.73132324219 1222.140625 753.232360839844 986.490661621094 705.538635253906 275.217712402344 885.637878417969 282.454864501953 1247.23400878906 2109.1044921875 924.067077636719 1233.65026855469 1202.39184570312 399.302490234375 881.62060546875 732.964477539062 279.915252685547 281.584716796875 1515.37145996094 131.306350708008 1071.51489257812 517.171508789062 432.135101318359 408.561859130859 664.003479003906 381.657257080078 1070.18811035156 698.671264648438 450.509613037109 1274.75109863281 375.693084716797 317.223754882812 458.443328857422 557.608703613281 1471.34057617188 1656.53552246094 1462.92150878906 1350.63916015625 710.150573730469 586.122924804688 547.535522460938 1106.48278808594 2620.77172851562 823.672180175781 784.412780761719 1180.99780273438 1722.77758789062 313.461517333984 1378.53735351562 837.133483886719 1163.54187011719 1248.92395019531 991.340454101562 872.423828125 744.087524414062 853.803405761719 1463.74365234375 1075.7841796875 988.837890625 587.259521484375 288.856292724609 1017.03643798828 862.80322265625 1268.52258300781 938.130798339844 259.723999023438 395.513702392578 1256.98645019531 591.818237304688 1341.14270019531 424.938110351562 813.82861328125 1184.57250976562 1413.98608398438 763.70458984375 917.355590820312 1196.45703125 534.145812988281 384.756866455078 1061.27453613281 1306.89807128906 640.5029296875 802.794860839844 359.102600097656 653.6572265625 1493.54821777344 297.329620361328 1438.27502441406 1310.36694335938 1446.52197265625 1432.73828125 1563.45690917969 1238.50451660156 1047.40991210938 1207.62585449219 938.7578125 641.325012207031 101.938781738281 1062.90173339844 741.27587890625 876.717956542969 2527.70434570312 1006.98931884766 866.727661132812 602.256408691406 790.686279296875 1045.08129882812 1511.529296875 327.885162353516 1343.01220703125 793.16552734375 776.571228027344 1422.0048828125 1079.00354003906 961.036071777344 1235.93347167969 336.461822509766 657.826904296875 1453.57470703125 931.980041503906 610.501037597656 1651.45703125 1543.96704101562 971.521179199219 779.380126953125 1129.77661132812 1289.91943359375 1561.12854003906 669.340393066406 527.008483886719 1131.8681640625 626.504638671875 1123.52978515625 869.058715820312 1296.85009765625 273.5693359375 744.175659179688 547.692138671875 807.205322265625 1045.42272949219 491.415344238281 1685.38354492188 1046.50769042969 998.876037597656 1386.58825683594 545.612548828125 747.709533691406 955.462463378906 351.672149658203 1281.59619140625 565.881958007812 1249.04821777344 467.152893066406 1442.13415527344 1343.34875488281

ENSG00000204642.12 180.271957397461 168.377380371094 52.0591888427734 102.141426086426 157.903884887695 172.535385131836 39.7880973815918 37.9070053100586 53.3610992431641 33.3487396240234 78.2552185058594 27.7473297119141 156.483840942383 131.962478637695 65.4994354248047 185.94645690918 7.95435380935669 239.600494384766 89.2595291137695 59.6184463500977 96.3882446289062 117.05396270752 94.2264556884766 68.0931701660156 12.6056528091431 52.5359535217285 168.750625610352 59.6424789428711 82.6186676025391 55.5910186767578 132.073928833008 266.909851074219 176.047058105469 50.5150451660156 36.4133110046387 187.30290222168 172.685821533203 224.8603515625 93.5769882202148 45.0874938964844 57.5102310180664 219.70378112793 251.558166503906 37.2494125366211 132.793594360352 81.5982131958008 68.8072128295898 79.132926940918 116.252464294434 43.6673278808594 80.9359130859375 37.4742012023926 22.6103038787842 97.8618927001953 88.4136428833008 46.8447303771973 250.192367553711 113.045478820801 143.264297485352 76.5898590087891 97.9098739624023 29.3919658660889 81.1213226318359 11.9850330352783 41.2918167114258 54.8437843322754 46.2563591003418 21.827127456665 65.855354309082 138.692184448242 52.0682334899902 88.3656921386719 35.9587440490723 79.2898712158203 121.829727172852 26.8133277893066 32.2035102844238 131.4677734375 13.8109350204468 84.5233612060547 66.5724258422852 212.684661865234 49.3858108520508 53.4177131652832 87.3861923217773 173.5693359375 126.675682067871 27.5654850006104 182.699584960938 115.609901428223 53.650333404541 49.220085144043 10.977632522583 48.0697898864746 27.8586235046387 75.3578186035156 107.88395690918 81.1911392211914 176.229019165039 68.7413024902344 100.405334472656 51.8054580688477 157.809295654297 85.5754699707031 45.6907272338867 107.401626586914 52.9486961364746 14.1246004104614 114.135108947754 13.5739698410034 128.626724243164 254.19140625 106.768699645996 84.5480270385742 88.5514831542969 13.4831123352051 136.555358886719 9.29075050354004 11.2274370193481 27.5959148406982 147.967651367188 7.30028295516968 76.0866317749023 56.1174240112305 29.438892364502 30.3185291290283 76.3208541870117 112.614639282227 102.81079864502 33.4459457397461 46.2911987304688 93.1803436279297 31.8771667480469 11.5274648666382 31.1922721862793 20.4299087524414 68.0495834350586 225.379150390625 158.511688232422 229.515243530273 37.5290718078613 35.3979835510254 40.4663505554199 69.9081878662109 154.186614990234 30.0103855133057 49.6668472290039 119.746765136719 107.104476928711 18.4417915344238 202.704788208008 30.1555881500244 129.99626159668 45.0163650512695 85.4980545043945 93.5846099853516 59.4756088256836 51.4617042541504 107.29216003418 51.7454452514648 63.0010223388672 30.1445655822754 40.2393836975098 102.807312011719 66.4291000366211 139.913772583008 104.734146118164 20.4394931793213 54.4783210754395 137.332595825195 43.1586799621582 99.2038421630859 25.1788501739502 136.874908447266 99.8902816772461 116.12264251709 56.2444839477539 132.488418579102 95.0622634887695 13.101375579834 18.5399703979492 161.235305786133 182.471435546875 41.8311805725098 83.2853240966797 26.3422679901123 61.4773254394531 157.460357666016 26.6446781158447 132.015411376953 60.216911315918 103.02229309082 94.5406799316406 155.897094726562 142.807983398438 176.364059448242 133.419082641602 110.033645629883 34.7701301574707 10.2526540756226 89.4421997070312 61.2893371582031 44.432918548584 254.224517822266 81.0536956787109 35.0703582763672 26.9084243774414 60.3822708129883 37.8541564941406 344.32568359375 29.3201694488525 125.498756408691 68.9091567993164 57.8174438476562 167.952484130859 148.103927612305 112.801887512207 145.125335693359 30.8469676971436 51.3756523132324 69.9596405029297 56.5160789489746 16.159538269043 106.701179504395 134.197341918945 66.4243011474609 86.998664855957 194.512512207031 114.268051147461 117.96590423584 53.5373344421387 19.6539096832275 87.2088165283203 28.9044189453125 62.5638771057129 99.8750991821289 83.551383972168 11.7310009002686 51.5058975219727 89.1995010375977 51.4947166442871 116.757041931152 35.479061126709 78.7145004272461 70.2110595703125 80.0241088867188 114.615631103516 44.6942520141602 32.3879661560059 50.174877166748 20.6022968292236 166.998580932617 12.8126544952393 159.640502929688 52.0544166564941 111.217994689941 142.127471923828

ENSG00000204632.10 19.5296440124512 31.4785461425781 18.2694873809814 10.3800411224365 97.4557037353516 35.1325645446777 6.16793632507324 5.49475049972534 11.7428741455078 6.84440565109253 10.6171445846558 1.80280148983002 12.7396850585938 10.4495859146118 15.1417016983032 54.9337997436523 2.13818097114563 22.2933750152588 87.6638870239258 4.3604907989502 12.4308156967163 113.856872558594 5.82789707183838 11.0106077194214 4.73735904693604 6.38899660110474 28.6575527191162 3.28413343429565 8.61629676818848 6.48141622543335 24.6460075378418 12.3562440872192 216.152450561523 9.59387111663818 1.60318899154663 105.460914611816 39.5103912353516 7.8580584526062 3.29236912727356 9.23127174377441 7.26078367233276 16.2909049987793 33.0076179504395 0.227319419384003 10.8564434051514 9.04191875457764 14.8971004486084 19.1383056640625 20.0717449188232 8.13752746582031 16.424144744873 6.04335927963257 29.4944152832031 34.1228713989258 19.6088104248047 12.9510259628296 30.9124908447266 9.84879493713379 23.8786563873291 18.7901802062988 4.35857248306274 5.45814895629883 11.8481101989746 4.97991418838501 12.0346269607544 15.7731952667236 22.4486255645752 24.571647644043 11.7572813034058 5.71138525009155 6.38294696807861 13.4385442733765 5.67068147659302 13.1565217971802 111.003440856934 2.92056131362915 4.02639198303223 22.1595897674561 1.05353796482086 7.4074125289917 4.77581596374512 76.8741760253906 14.766806602478 10.1988220214844 6.86123847961426 32.1798477172852 22.3561153411865 5.09516191482544 57.7840614318848 10.4381504058838 2.36671423912048 5.67151880264282 2.26739621162415 10.1115798950195 4.38494825363159 3.99367165565491 23.5844268798828 17.3798789978027 89.9325332641602 9.78070449829102 30.9231567382812 8.75416946411133 32.2998924255371 10.2922239303589 7.98612117767334 41.4793395996094 4.8464035987854 4.75303936004639 63.7700042724609 2.49580240249634 3.09379887580872 36.5095138549805 3.77313685417175 23.7993259429932 9.49122524261475 2.09557771682739 14.4305591583252 9.24477386474609 6.43146848678589 5.42270135879517 3.12125897407532 0.938876330852509 8.38554000854492 4.99726438522339 4.10350561141968 2.4172842502594 10.0043058395386 9.67348670959473 15.1347599029541 8.25963592529297 31.8116340637207 14.5573015213013 7.16886568069458 1.17528116703033 1.22863173484802 6.46628093719482 12.1773471832275 34.8448219299316 35.8753662109375 73.7743225097656 9.1624641418457 2.84527206420898 4.31160259246826 25.4123268127441 15.2566270828247 36.123706817627 10.0823516845703 18.3599548339844 21.6341400146484 6.6330189704895 23.4381637573242 4.92858028411865 12.8247041702271 9.93939018249512 9.75089073181152 7.09445381164551 18.854944229126 5.35899209976196 7.49680614471436 211.257736206055 2.75376081466675 11.6669578552246 5.37676048278809 8.67486095428467 16.4472789764404 15.0067729949951 10.8819904327393 1.87159144878387 5.24249124526978 29.1658821105957 4.71862554550171 28.4165687561035 6.25692176818848 1580.24499511719 27.771821975708 21.6002063751221 8.70169544219971 16.8335189819336 22.2037410736084 11.3855962753296 6.25494766235352 8.19247817993164 37.7517700195312 5.71712875366211 17.9479160308838 1.20291066169739 13.4287605285645 30.2238540649414 5.23072099685669 41.2976951599121 7.4868483543396 7.25701570510864 15.8590211868286 25.2138385772705 10.5107231140137 15.9884805679321 22.5929145812988 6.82130479812622 5.82111597061157 0.654061138629913 24.293420791626 14.0750465393066 14.084282875061 39.6023712158203 3.70760536193848 5.04683256149292 5.84636211395264 4.27802562713623 8.88126468658447 72.1404037475586 3.99130463600159 5.54046249389648 14.4964332580566 7.14151191711426 14.7897043228149 17.9114265441895 9.51336479187012 17.7894554138184 1.99574542045593 5.95506477355957 34.0993194580078 2.87066555023193 3.50385284423828 13.8134250640869 18.7944164276123 6.7569751739502 11.9602174758911 2.69257855415344 7.46398735046387 23.1915340423584 33.9902458190918 6.57999801635742 16.0556373596191 27.0254192352295 28.7562789916992 7.25910615921021 12.4190053939819 4.5012378692627 7.98920822143555 7.01900482177734 3.86443185806274 20.562068939209 5.55122375488281 20.9947128295898 7.84560060501099 16.4835090637207 6.66117238998413 4.04242086410522 3.16207575798035 18.2696399688721 7.49220561981201 25.3604106903076 7.13908720016479 6.03607320785522 12.8319454193115 23.2099113464355 4.80876874923706

ENSG00000153029.13 21.2741966247559 23.1578311920166 23.7484397888184 28.4228458404541 15.525918006897 36.3666076660156 19.5704746246338 33.2634696960449 28.6492786407471 17.7489242553711 46.2655487060547 9.08442878723145 29.4774112701416 20.9945774078369 20.5786170959473 23.01953125 9.90695571899414 40.448070526123 29.0960845947266 21.7138175964355 19.9704284667969 16.2343120574951 33.747257232666 38.0228233337402 6.79194974899292 11.0248126983643 41.2365989685059 6.62732315063477 26.7266159057617 39.4651679992676 27.3460159301758 26.4656467437744 14.3749198913574 23.4968013763428 25.5768337249756 17.5652103424072 24.6970443725586 27.9579734802246 26.0790271759033 12.4298267364502 44.4872779846191 22.5604782104492 40.1242179870605 12.8859901428223 7.75889873504639 31.9670143127441 12.7344331741333 21.5854778289795 20.4528522491455 33.953067779541 11.6662902832031 7.26456356048584 25.3712387084961 25.2952747344971 21.9901485443115 25.147819519043 18.8477611541748 28.1912689208984 41.0999946594238 48.7553024291992 18.5690269470215 10.7713890075684 16.615873336792 14.7197351455688 21.5996875762939 16.685941696167 14.8263969421387 9.0942325592041 19.2468757629395 22.133638381958 22.1486644744873 27.3021774291992 15.0999984741211 22.6516284942627 16.0369548797607 10.7407474517822 7.2533540725708 45.144603729248 5.68263578414917 19.1839694976807 22.1484699249268 30.1364154815674 16.6689281463623 11.5601034164429 15.2375755310059 24.297700881958 20.580810546875 9.99886226654053 13.0082225799561 9.04097080230713 35.606876373291 37.8691749572754 4.87583112716675 15.5423889160156 22.7299499511719 28.349328994751 17.4464874267578 22.6237106323242 22.2282581329346 29.1623516082764 17.3995990753174 40.2891883850098 14.178092956543 17.2406921386719 31.6090316772461 31.6672039031982 16.7502269744873 20.2795734405518 49.5870170593262 8.66458797454834 36.5611572265625 16.0884704589844 25.0817184448242 19.5380439758301 19.3545017242432 40.6467132568359 19.6921329498291 6.01217889785767 19.2474479675293 7.67879152297974 24.1572132110596 2.58520436286926 34.7203636169434 26.8787746429443 9.41741943359375 7.0252857208252 16.0214328765869 4.89456605911255 45.2390365600586 21.0294666290283 6.51194715499878 23.8023471832275 4.98932361602783 6.65654420852661 4.9066801071167 30.9071578979492 22.8420524597168 29.4209461212158 21.9845199584961 15.1204843521118 12.9297485351562 12.809886932373 12.842212677002 23.0036659240723 52.8369140625 28.7701530456543 32.5467720031738 25.0688056945801 23.7958583831787 9.85929393768311 13.6898794174194 16.7392463684082 21.4739837646484 40.8766212463379 8.10841274261475 13.1229972839355 12.4681425094604 18.0157032012939 25.9507179260254 30.4031219482422 14.4284763336182 55.5397987365723 4.71132707595825 61.0055084228516 35.3961181640625 26.5622253417969 33.0653457641602 24.8543472290039 6.15523815155029 25.9961376190186 21.9024772644043 26.0228271484375 22.0584468841553 7.40571737289429 11.0197534561157 23.2784175872803 9.57419395446777 21.9210872650146 14.509147644043 7.84225225448608 17.3852577209473 48.9113922119141 31.6758975982666 22.9695739746094 14.9276494979858 19.2431697845459 28.9140453338623 17.8332099914551 7.18572378158569 16.6744899749756 29.1811981201172 27.8627872467041 31.8848686218262 21.6099319458008 36.185302734375 23.9124889373779 29.4633636474609 26.5521278381348 21.2337646484375 10.8439064025879 23.7481861114502 13.1721677780151 4.20800542831421 32.7297096252441 34.7829971313477 28.4320697784424 13.6367149353027 18.9257049560547 32.4286231994629 25.3373527526855 6.10615730285645 15.0001783370972 20.1969432830811 28.9868583679199 26.8211555480957 36.2186584472656 11.5102310180664 22.5931606292725 9.25084400177002 61.537223815918 30.26904296875 12.2855577468872 24.7119636535645 20.3316040039062 14.8109588623047 27.4279232025146 15.1893272399902 13.6368389129639 28.0801887512207 26.8414478302002 16.7349166870117 26.4406261444092 16.0439205169678 9.8638801574707 26.1747722625732 10.2615346908569 33.1151924133301 5.03427886962891 24.3742542266846 7.20925235748291 22.6287040710449 33.5451164245605 8.03823184967041 18.2996482849121 21.2811794281006 31.4843406677246 22.9527378082275 11.2604475021362 22.5226669311523 20.3621292114258 19.2265930175781 15.9117755889893 3.21862936019897 26.2314395904541 19.851131439209 20.5088748931885 26.8711948394775

ENSG00000204389.9 27.2379589080811 52.7629013061523 158.126419067383 148.435394287109 63.6591300964355 57.1777610778809 162.439071655273 22.4377899169922 173.674011230469 77.3042678833008 141.995483398438 250.767776489258 125.268135070801 6.91477298736572 134.074203491211 300.698516845703 92.9365463256836 69.688720703125 41.3593902587891 229.823745727539 72.5275726318359 81.1384887695312 79.5094299316406 98.4991607666016 74.4250335693359 90.1410140991211 54.7455368041992 133.2109375 121.650367736816 103.478988647461 85.939567565918 163.303146362305 36.9572830200195 132.126129150391 289.316436767578 85.2166290283203 27.3692607879639 75.2666854858398 22.3504524230957 65.3159942626953 66.2578201293945 66.7070770263672 124.525276184082 43.6710891723633 46.6559867858887 54.7772445678711 5.83578586578369 61.8542747497559 63.1114540100098 105.321365356445 30.0122680664062 44.6384353637695 60.3144721984863 114.960342407227 60.5356559753418 108.932426452637 61.1633453369141 145.49787902832 43.9034233093262 87.7960510253906 92.2858123779297 375.254577636719 178.197296142578 119.662727355957 72.1256408691406 25.5141563415527 110.350425720215 113.147880554199 79.5147094726562 59.4769248962402 115.182121276855 112.923027038574 73.128288269043 29.6442012786865 111.131195068359 7.33468532562256 1282.99084472656 85.4895858764648 102.588577270508 68.0444183349609 215.147750854492 65.858268737793 262.640686035156 78.7826232910156 79.5043716430664 45.533992767334 82.2153778076172 87.1672210693359 245.083984375 130.202407836914 48.8853492736816 112.632247924805 74.9835433959961 82.7982177734375 25.5484943389893 43.6370315551758 96.0960845947266 153.967102050781 106.13346862793 48.3364524841309 65.215690612793 77.5825271606445 101.29109954834 103.417259216309 93.6182174682617 97.4000091552734 91.2569198608398 39.4703941345215 62.5542144775391 328.466217041016 213.521759033203 54.9412956237793 46.7342529296875 168.117553710938 63.872200012207 44.523120880127 94.3417587280273 276.41943359375 112.248977661133 90.3209381103516 28.2667751312256 67.129508972168 67.414306640625 141.301788330078 820.635986328125 179.014266967773 115.723556518555 50.2966651916504 86.3783798217773 125.429153442383 124.404724121094 48.8040657043457 122.319976806641 61.0606689453125 14.5699968338013 157.465255737305 74.3456497192383 312.244293212891 106.955207824707 82.6164321899414 59.0640754699707 71.5454788208008 183.276901245117 18.1986351013184 96.4598999023438 163.342681884766 46.4372100830078 21.0076599121094 77.9288787841797 140.889801025391 174.395385742188 53.0646514892578 16.8878002166748 70.1660385131836 52.0706596374512 76.929069519043 46.1870193481445 75.2654647827148 41.3690910339355 92.3288345336914 36.1898612976074 209.667587280273 93.3713226318359 120.749534606934 127.906890869141 27.6389045715332 99.8761978149414 72.1262435913086 17.9911956787109 132.855575561523 60.9456405639648 196.981521606445 185.225250244141 113.924728393555 310.58837890625 94.5600662231445 75.8350982666016 68.3785705566406 52.7919998168945 112.154823303223 104.423469543457 92.8232040405273 16.4851303100586 78.8492660522461 110.342750549316 67.8535385131836 93.2026596069336 214.904449462891 124.41764831543 148.961669921875 47.0585556030273 303.167785644531 115.042259216309 70.4663696289062 45.4460983276367 733.86669921875 59.3474273681641 51.7938652038574 37.6797790527344 19.1530590057373 67.1855850219727 98.8643035888672 108.559280395508 47.5831909179688 32.7136840820312 40.7482986450195 56.3475952148438 50.0178108215332 45.3132400512695 147.498199462891 20.2850341796875 90.9454498291016 122.37858581543 97.5774230957031 104.049140930176 45.6858291625977 49.5162811279297 62.6498374938965 134.964767456055 82.9325866699219 43.650505065918 61.5657119750977 31.6846809387207 87.2525939941406 68.5669860839844 477.416107177734 320.669158935547 43.5732612609863 158.943161010742 58.248722076416 110.777030944824 149.986801147461 153.870056152344 60.3294143676758 160.281356811523 115.850608825684 50.2879028320312 143.820251464844 91.8371658325195 59.2306365966797 68.0975036621094 142.154479980469 121.807975769043 63.1981887817383 286.642730712891 88.1588973999023 32.215690612793 103.873733520508 78.5960845947266 103.094917297363 34.1193084716797 128.410232543945 66.1963272094727 116.302207946777 43.2080078125 85.3877944946289 70.4212036132812

ENSG00000204388.6 118.971717834473 57.6810836791992 118.925117492676 154.391860961914 60.9659461975098 114.351631164551 195.682388305664 18.6358051300049 141.702270507812 78.1220016479492 163.820877075195 361.203521728516 113.862854003906 121.645263671875 189.242004394531 275.309600830078 116.632217407227 61.7234153747559 57.6756706237793 187.538299560547 48.6317863464355 75.3619842529297 56.656063079834 100.294586181641 69.7329254150391 113.006690979004 41.7181777954102 143.145736694336 106.780540466309 114.696891784668 89.9164581298828 195.595916748047 31.9521179199219 120.395317077637 213.933944702148 79.6805648803711 90.8853073120117 116.968353271484 110.06778717041 86.1005630493164 116.861038208008 73.8434448242188 118.09944152832 64.8825912475586 63.759407043457 49.8720512390137 75.3203964233398 55.6904640197754 80.1031188964844 171.49626159668 37.8387413024902 67.9076385498047 80.1248550415039 103.049201965332 109.528991699219 144.992156982422 67.8816070556641 192.628234863281 73.4675521850586 112.541625976562 185.983856201172 353.633483886719 187.566757202148 114.988510131836 62.6223411560059 34.1404838562012 112.611793518066 132.808319091797 58.7062797546387 128.413055419922 120.165328979492 133.490676879883 67.0009307861328 284.998931884766 125.157730102539 174.059860229492 1346.42443847656 85.3437194824219 144.932434082031 68.8225784301758 345.235107421875 69.6421127319336 245.285339355469 209.202117919922 65.4127426147461 90.0971527099609 98.177864074707 184.851867675781 257.842468261719 138.828857421875 70.0409927368164 116.703575134277 90.6921920776367 89.1422958374023 35.7157821655273 47.4789085388184 90.2042236328125 106.489784240723 127.64665222168 76.2702865600586 109.985496520996 94.3307723999023 60.8978500366211 111.724800109863 100.940505981445 113.665756225586 124.206504821777 78.6661605834961 68.0371780395508 406.328765869141 250.716430664062 78.7520217895508 60.6889801025391 131.955978393555 49.7077178955078 45.7120552062988 82.5732803344727 239.902557373047 93.1318206787109 81.0906066894531 30.1470317840576 89.9186706542969 67.5489196777344 132.148208618164 891.660034179688 181.421112060547 132.849136352539 73.1577529907227 117.90975189209 110.665252685547 105.706329345703 60.739315032959 140.507507324219 75.384033203125 19.4302883148193 145.835693359375 46.7776489257812 364.563568115234 134.335876464844 120.199028015137 83.0170745849609 62.083309173584 157.2763671875 73.9678344726562 148.529769897461 153.383117675781 64.6887283325195 94.9913330078125 53.9792137145996 154.175842285156 156.131011962891 60.108959197998 59.400634765625 93.4000473022461 56.2809638977051 163.485870361328 46.9192771911621 58.6679611206055 27.2315483093262 75.8088607788086 178.396850585938 192.656356811523 61.8830528259277 129.535461425781 105.946243286133 22.3832168579102 90.243766784668 106.55859375 178.404205322266 151.87255859375 95.999755859375 151.030517578125 201.322113037109 92.4554290771484 330.932037353516 92.9221954345703 57.986743927002 167.695648193359 81.1046524047852 314.146545410156 146.099807739258 99.2768173217773 151.325500488281 63.4855346679688 59.270679473877 80.5707855224609 92.7629699707031 161.441192626953 98.1834411621094 188.361740112305 169.088836669922 321.221923828125 195.98371887207 57.4286766052246 78.87890625 635.32373046875 55.9300231933594 54.7774810791016 77.3135681152344 16.4803237915039 63.5958290100098 111.766532897949 108.919906616211 46.9610137939453 59.2871246337891 70.1842346191406 67.5791320800781 69.8071060180664 55.1514015197754 142.637252807617 51.6559677124023 104.852584838867 95.8060913085938 118.081268310547 98.6778793334961 94.5000991821289 31.2853164672852 48.9136085510254 170.892776489258 81.6825103759766 55.0304527282715 66.0940628051758 37.5872039794922 170.498138427734 62.2104988098145 337.452117919922 369.219696044922 43.7795028686523 145.608551025391 36.7373847961426 76.8494491577148 132.052474975586 170.32844543457 90.5531616210938 177.350387573242 110.471626281738 61.0094833374023 137.55290222168 117.597702026367 66.4952392578125 107.411949157715 170.113632202148 118.380096435547 87.7299728393555 278.271545410156 73.636100769043 47.117847442627 104.823997497559 62.4016571044922 84.8533554077148 36.2789611816406 113.123733520508 69.1571197509766 127.465232849121 31.7089290618896 71.9209594726562 69.4297790527344

ENSG00000204390.9 2.72439765930176 1.99829590320587 2.76813459396362 3.70569181442261 3.32853841781616 5.7563214302063 5.17744159698486 1.35868000984192 1.34187924861908 2.23607754707336 3.7606098651886 3.16240930557251 1.67537140846252 2.85722851753235 1.34738206863403 3.66050934791565 0.881050169467926 3.36295819282532 1.46549308300018 2.78938484191895 3.42779660224915 2.73221898078918 3.1373987197876 2.06598877906799 2.42241144180298 2.22885704040527 3.44403696060181 1.20002782344818 1.98080444335938 2.43831968307495 3.32475876808167 3.87320137023926 2.24067044258118 6.61794471740723 3.69582557678223 2.78008627891541 2.11477899551392 3.75750517845154 2.61551833152771 2.60355257987976 3.71803903579712 4.61043214797974 3.94772124290466 1.64284479618073 1.13843190670013 2.09711480140686 1.26574563980103 1.36425232887268 2.85905456542969 2.81966614723206 0.725407242774963 2.54130291938782 1.31794583797455 5.06726980209351 2.4217963218689 3.58473014831543 4.04723882675171 2.16499733924866 3.455073595047 5.08264541625977 3.27866721153259 2.70385074615479 5.50627946853638 4.45505142211914 0.988155663013458 1.92444980144501 1.40870189666748 1.2552102804184 3.78874254226685 3.9580078125 1.99994242191315 4.63835859298706 1.57475745677948 1.80144357681274 3.73192167282104 1.23686325550079 3.19831871986389 2.92770957946777 0.813545703887939 2.61251378059387 2.637770652771 3.07668828964233 2.45978188514709 5.36301565170288 3.04052257537842 3.30528783798218 3.63816475868225 3.81725740432739 2.11969304084778 1.25026416778564 4.11898899078369 3.85097908973694 5.94366073608398 2.04463958740234 1.11719954013824 2.53699135780334 2.46280694007874 3.72902178764343 2.22415018081665 5.86355924606323 3.22333192825317 1.52093911170959 3.12639379501343 2.60458064079285 3.12006878852844 4.35154676437378 2.21932148933411 7.78117370605469 3.31505703926086 1.47926223278046 7.43519926071167 1.70912861824036 2.67935872077942 3.41671013832092 2.95017910003662 2.40178489685059 1.5849198102951 5.52298069000244 2.00165939331055 1.81634211540222 3.59146666526794 0.975966453552246 4.38964700698853 4.44773244857788 5.92731809616089 1.6751856803894 1.30736863613129 1.05344748497009 3.46949791908264 1.95235645771027 1.50621855258942 2.06701254844666 2.54997062683105 4.19962882995605 2.83815217018127 1.78545939922333 2.49602437019348 2.77157711982727 2.65096354484558 2.55210781097412 1.61525332927704 1.38265240192413 3.22836422920227 2.85580921173096 2.12773942947388 2.89133524894714 1.99631476402283 2.30200862884521 3.3465461730957 2.6879026889801 2.37666320800781 2.56164145469666 2.21529674530029 2.93913292884827 2.5626859664917 1.92725968360901 1.76942670345306 4.18179750442505 2.54721546173096 3.40213108062744 4.51068067550659 4.91119241714478 1.76627373695374 5.46143531799316 1.96299171447754 2.46673202514648 3.17301774024963 1.69954168796539 3.93581819534302 3.78718614578247 2.64613628387451 7.83650541305542 2.49001836776733 1.41553914546967 2.03496694564819 2.592036485672 3.14320778846741 1.91919267177582 2.77629518508911 4.36614847183228 1.46296083927155 6.18158483505249 4.98785305023193 3.04152631759644 2.40204048156738 1.9821652173996 1.29252409934998 1.2966091632843 6.67312335968018 3.95296025276184 4.9048900604248 5.13501119613647 3.49692988395691 3.25196981430054 3.93670845031738 4.52281951904297 1.88561499118805 3.89108514785767 1.63630211353302 0.631825923919678 1.79125940799713 1.96270537376404 5.19886636734009 2.92030310630798 2.09937334060669 2.54815745353699 2.64004039764404 4.57408571243286 3.32961559295654 2.81627750396729 1.89251720905304 4.45825386047363 3.21293330192566 2.15177845954895 3.75547361373901 3.75059199333191 1.62095499038696 2.43662238121033 1.26011943817139 2.288170337677 1.47693347930908 1.42827308177948 1.97003364562988 3.32965135574341 2.47492218017578 3.77374505996704 2.10227274894714 2.1769597530365 3.23847651481628 4.1029577255249 2.9730052947998 1.9742351770401 2.61465692520142 1.83081090450287 2.73238468170166 2.39991140365601 2.04508757591248 1.99494695663452 2.56354570388794 1.73221254348755 4.57705402374268 3.31441831588745 1.14283716678619 2.43412041664124 4.2328519821167 2.11947131156921 0.756756246089935 1.596804022789 4.14251184463501 1.29193043708801 1.68296372890472 3.11732578277588 4.06917142868042 7.5437331199646 1.61313545703888 2.81791186332703 3.11032557487488

ENSG00000126803.9 2.38979411125183 3.90492701530457 5.8152060508728 2.27981019020081 9.39358997344971 50.782112121582 5.24924278259277 1.07854652404785 1.29621005058289 8.17594909667969 7.4162163734436 5.70802593231201 8.09898090362549 0.911131322383881 3.99157691001892 37.1620445251465 11.948187828064 5.23338222503662 3.05076789855957 1.53544127941132 3.50558805465698 7.74774360656738 4.93867206573486 2.17137336730957 93.2423782348633 3.23634696006775 57.8205146789551 4.50095844268799 1.63078594207764 6.0469388961792 5.08675003051758 8.47283267974854 7.89373779296875 3.45401382446289 2.25656056404114 8.40573215484619 6.25754022598267 2.92566800117493 3.40635108947754 67.2515029907227 4.2024073600769 5.12776374816895 4.1550087928772 15.8367185592651 3.24773216247559 4.10568141937256 3.03082203865051 8.78171443939209 6.42955732345581 4.12363004684448 1.9470956325531 2.2382116317749 8.51223754882812 7.45944595336914 11.2657432556152 1.62536990642548 12.5680780410767 6.4313645362854 4.1297869682312 6.15975475311279 3.35374736785889 7.56212663650513 13.8289785385132 17.122501373291 6.77095222473145 2.44069647789001 7.57900714874268 4.83919715881348 11.7149391174316 3.76707720756531 4.93196773529053 4.32452440261841 4.44821119308472 1.39231598377228 185.89143371582 1.68353700637817 3.13564920425415 1.7636194229126 5.49329948425293 8.09362030029297 2.76150894165039 3.42886805534363 18.1002998352051 3.62856483459473 1.88022172451019 1.71858906745911 9.8467845916748 26.5386943817139 4.7216649055481 1.02249658107758 2.23245143890381 6.20643949508667 7.63405990600586 6.69611692428589 3.6603147983551 3.70145440101624 2.59632015228271 15.6003103256226 16.9839458465576 5.04578971862793 33.0996780395508 0.985843181610107 4.62776613235474 9.82866191864014 5.93753576278687 7.62825775146484 1.58976709842682 1.97606003284454 4.03294372558594 9.6910982131958 3.39548349380493 3.45409774780273 1.49264585971832 7.70878505706787 2.60897183418274 2.06613969802856 1.69236552715302 46.3943901062012 38.4788703918457 4.13719177246094 5.96680736541748 1.33232843875885 8.10369968414307 4.29105424880981 7.84567260742188 7.83433675765991 8.16478443145752 1.03745687007904 13.2507753372192 2.13961315155029 18.7831344604492 3.49153351783752 2.21680474281311 5.31836271286011 2.82855916023254 5.45755100250244 8.57524490356445 1.79787850379944 5.41104173660278 2.00207471847534 30.2426948547363 9.60962677001953 3.88689279556274 5.13521671295166 1.36201083660126 43.3146286010742 1.46518516540527 1.76027286052704 6.84979677200317 3.46521806716919 1.99490821361542 6.13302803039551 2.26465487480164 6.19356822967529 5.27435636520386 4.72847986221313 2.721195936203 7.86470985412598 3.18743228912354 4.05606460571289 5.68403053283691 13.9585618972778 2.56783413887024 6.66988515853882 9.1741828918457 3.05892825126648 3.02642250061035 4.23449277877808 11.2697486877441 5.60147047042847 5.15371894836426 5.88268756866455 25.0120410919189 68.9776458740234 25.425853729248 8.29689407348633 6.90031814575195 6.42976236343384 9.3248291015625 1.42829692363739 2.91100859642029 32.0868072509766 4.26543378829956 10.0982780456543 89.9402160644531 7.07277584075928 3.92737579345703 3.18433284759521 18.4661617279053 3.95150947570801 8.58986568450928 15.3977479934692 6.04326438903809 14.6518974304199 5.88681125640869 4.8200626373291 6.62424039840698 6.4575080871582 2.82521843910217 2.54040908813477 5.30315828323364 3.80820536613464 1.04869699478149 2.33693385124207 1.50402867794037 2.28825354576111 4.18991661071777 8.3057804107666 4.99118375778198 4.72571229934692 5.56135606765747 3.53388261795044 3.51724672317505 15.6159896850586 9.07859802246094 1.41568875312805 1.47561764717102 12.9655122756958 3.37009334564209 1.83533465862274 6.80410861968994 8.760573387146 5.8508358001709 6.23164081573486 5.03103399276733 5.56844472885132 4.92480039596558 3.40667080879211 5.92235136032104 11.0205249786377 10.2610988616943 1.90803730487823 3.34496831893921 25.9409523010254 5.96812963485718 6.42234563827515 2.75805377960205 7.47531175613403 7.98070240020752 0.577885746955872 4.51719379425049 4.14429903030396 4.08993721008301 1.62004017829895 3.37449765205383 7.23645544052124 1.08306407928467 4.36711597442627 3.10647058486938 3.13720631599426 0.614973247051239 4.10749244689941 75.3770370483398 4.20368194580078 2.61464500427246 5.90807485580444 6.16969585418701

ENSG00000170606.12 127.246879577637 86.2724380493164 63.7006492614746 99.6617660522461 59.5826606750488 80.3923034667969 59.0078201293945 77.7906036376953 79.4295501708984 73.9206619262695 115.071640014648 75.4362335205078 74.8711013793945 65.3356857299805 33.9655456542969 84.6929626464844 42.8688468933105 79.9085388183594 139.637130737305 152.912506103516 50.0155830383301 117.81510925293 51.1185646057129 107.00675201416 131.601303100586 138.521499633789 49.5590019226074 33.9232788085938 94.3487548828125 111.591041564941 64.4276962280273 92.8568496704102 99.3916244506836 54.1803207397461 48.8552513122559 44.3472595214844 55.5862884521484 108.511245727539 107.057556152344 44.777759552002 90.9790496826172 53.8664245605469 63.7672004699707 26.0609951019287 41.1390686035156 59.3535232543945 87.1409225463867 72.8385162353516 77.5236663818359 51.1666946411133 60.0283126831055 37.3258972167969 94.587516784668 79.1260070800781 93.8208236694336 125.540260314941 71.4419250488281 86.3449325561523 65.0497970581055 76.1875610351562 115.0869140625 82.3236312866211 99.7739028930664 57.2034454345703 68.2976455688477 44.0394096374512 88.9829940795898 76.9252090454102 59.4384689331055 64.4248275756836 96.553825378418 58.8418617248535 111.852180480957 82.753059387207 123.236907958984 147.205825805664 71.8768157958984 106.637672424316 88.0730590820312 77.1156158447266 81.6133193969727 73.1002197265625 96.2598114013672 102.481079101562 73.7657089233398 61.9329261779785 93.7683181762695 160.093811035156 45.8962097167969 62.1514205932617 87.0960388183594 93.4768447875977 71.1838455200195 54.9767150878906 45.1349296569824 51.3603477478027 55.2346839904785 45.6913642883301 102.52596282959 80.6090469360352 101.017280578613 130.450088500977 54.0421676635742 61.5471229553223 117.356323242188 62.6575202941895 88.4780960083008 32.8811569213867 63.0144157409668 153.745651245117 84.1733016967773 88.6060943603516 88.4791641235352 55.7987060546875 75.8600769042969 93.2610244750977 66.8997955322266 61.8772621154785 58.2306785583496 69.8258438110352 66.2102355957031 17.2875518798828 95.236930847168 39.4346542358398 99.3847579956055 71.1656265258789 69.5299911499023 43.5006103515625 61.1303291320801 114.348541259766 61.0249557495117 89.1097564697266 37.9403381347656 59.3568534851074 98.9391632080078 134.302597045898 76.1705551147461 94.4320297241211 58.5772171020508 63.0575675964355 60.1578941345215 91.3684997558594 62.2838287353516 53.6301612854004 72.9042739868164 87.5554122924805 64.0063095092773 78.8649444580078 62.336296081543 76.7289428710938 97.5639572143555 87.5016250610352 66.7144927978516 87.5093231201172 80.7846527099609 79.1593246459961 73.0537414550781 66.6818237304688 49.2396011352539 88.1485977172852 88.1507034301758 123.57315826416 63.7023124694824 96.8532028198242 64.8773193359375 38.3727188110352 79.4797821044922 57.4636726379395 52.7579154968262 68.4483489990234 75.1314697265625 69.1977005004883 137.898559570312 46.433895111084 80.3762054443359 53.4351196289062 45.0788192749023 52.1281623840332 78.8006134033203 251.235504150391 32.7765731811523 47.7946128845215 82.1509552001953 55.7235984802246 65.347282409668 92.2438735961914 99.6530609130859 75.0973281860352 184.240921020508 70.858512878418 132.993637084961 139.611633300781 116.54460144043 64.5528182983398 108.441711425781 109.231521606445 57.6517791748047 81.6505966186523 33.0298500061035 32.3335380554199 32.3011474609375 72.4899063110352 66.8925018310547 76.3519058227539 75.245475769043 90.1047439575195 66.1365356445312 57.3136100769043 61.3331260681152 75.1274642944336 39.1468811035156 64.636100769043 58.186466217041 64.7424621582031 87.192626953125 117.011436462402 37.1532020568848 44.697696685791 135.587036132812 90.5616455078125 119.399192810059 147.764282226562 83.7524642944336 103.090034484863 44.9725189208984 122.686454772949 71.0022201538086 43.3677749633789 76.8093414306641 83.3704299926758 48.2195625305176 73.9539337158203 81.0871429443359 66.4803924560547 102.497192382812 67.313232421875 63.3814468383789 37.583065032959 89.8144302368164 25.3987808227539 82.9901657104492 47.4013595581055 77.1248321533203 86.1587448120117 119.531387329102 36.2484588623047 74.1174926757812 49.4562606811523 57.8449554443359 71.7763977050781 43.6569938659668 69.8808975219727 230.400451660156 87.6021499633789 82.7366180419922 58.223445892334 94.429084777832

ENSG00000044574.7 941.088928222656 690.120300292969 1859.99719238281 890.565490722656 665.762573242188 1797.66271972656 743.833190917969 242.726943969727 381.815521240234 1009.21105957031 903.065368652344 712.315307617188 592.427490234375 1543.876953125 482.383087158203 1127.71203613281 762.742126464844 765.378051757812 882.185668945312 744.2939453125 501.683502197266 1495.37060546875 373.627777099609 522.951599121094 709.449340820312 1092.80102539062 748.524353027344 498.005401611328 699.441955566406 1271.60522460938 468.854858398438 773.017822265625 1015.84136962891 997.723815917969 749.084838867188 531.080444335938 621.478698730469 504.946044921875 1033.06164550781 810.521179199219 711.838317871094 563.910522460938 527.075866699219 680.915222167969 408.903198242188 708.766784667969 645.705688476562 733.918090820312 593.032104492188 950.393798828125 933.545349121094 637.388854980469 482.195953369141 1085.53942871094 998.001220703125 1537.41857910156 880.219787597656 948.01708984375 680.07421875 799.110107421875 814.756225585938 918.002563476562 965.872436523438 690.499755859375 586.835144042969 607.264587402344 423.127746582031 770.146606445312 524.222229003906 792.941345214844 913.647888183594 461.349700927734 2047.65209960938 822.486511230469 443.988372802734 938.894165039062 723.325317382812 711.02880859375 463.762969970703 685.103210449219 727.643981933594 492.113006591797 916.782836914062 752.868408203125 573.616882324219 902.163940429688 1197.77038574219 748.678100585938 545.29833984375 980.240173339844 362.794891357422 796.438659667969 680.22705078125 817.443115234375 127.067924499512 571.189392089844 706.703918457031 525.467895507812 854.514770507812 513.826232910156 578.531311035156 972.946350097656 404.354095458984 626.686584472656 2149.37670898438 978.465270996094 715.03857421875 596.931945800781 1050.951171875 1712.37780761719 911.758666992188 969.547546386719 935.095153808594 734.28271484375 1245.62341308594 1494.84716796875 607.146484375 1150.51440429688 1045.25170898438 772.01806640625 637.408996582031 419.781829833984 586.171752929688 1071.23547363281 928.977844238281 1175.31860351562 585.400939941406 259.543426513672 702.60595703125 1047.44848632812 859.837463378906 1139.07360839844 346.788635253906 1007.25531005859 843.459167480469 875.884155273438 540.079711914062 824.355651855469 735.336486816406 830.630554199219 1619.21484375 706.013671875 878.492309570312 421.835968017578 979.46533203125 662.622497558594 883.522583007812 751.781188964844 504.90869140625 1162.46826171875 779.622802734375 579.581726074219 555.808227539062 587.510437011719 494.123229980469 944.708374023438 985.769165039062 829.721313476562 375.944152832031 947.420593261719 546.631408691406 1457.05932617188 370.44677734375 1233.83959960938 1196.29736328125 348.517242431641 1514.17553710938 127.492820739746 476.111724853516 777.189331054688 1007.44647216797 817.061584472656 684.480895996094 681.36865234375 1468.80847167969 664.655456542969 580.467590332031 633.979614257812 2072.13623046875 1122.44189453125 116.962326049805 505.973236083984 638.602661132812 425.428588867188 561.56884765625 1201.86840820312 701.826416015625 877.559326171875 1003.70959472656 801.058288574219 916.985107421875 1017.83306884766 732.887573242188 744.089477539062 1188.26525878906 768.991455078125 569.786682128906 882.945434570312 1146.91162109375 152.843109130859 563.490783691406 708.075317382812 575.639770507812 830.282897949219 650.150634765625 792.473999023438 1093.29772949219 354.279663085938 550.038391113281 1222.28442382812 1579.21606445312 478.685363769531 574.44921875 608.877258300781 785.940063476562 945.182800292969 157.738647460938 445.582672119141 1082.37963867188 832.278076171875 1525.92077636719 964.101257324219 527.320373535156 539.845703125 438.627655029297 1316.14807128906 947.656188964844 392.294891357422 899.012573242188 788.922485351562 833.934814453125 977.049560546875 727.044616699219 982.371337890625 899.764770507812 683.330505371094 495.685668945312 727.105346679688 1191.73010253906 283.762176513672 1295.02026367188 698.682678222656 625.708435058594 642.821166992188 1536.17431640625 539.469421386719 697.289428710938 397.772552490234 619.802062988281 799.056518554688 370.230285644531 841.421569824219 1452.15979003906 505.761871337891 624.063293457031 458.287261962891 906.611694335938

ENSG00000173110.7 9.57106685638428 20.7709846496582 3.01880192756653 116.621856689453 14.9768590927124 59.2256050109863 38.466625213623 7.34662628173828 8.59229183197021 4.90752124786377 64.0995178222656 13.9077453613281 10.401927947998 6.44855356216431 12.6607732772827 49.0949745178223 4.4688606262207 19.4524612426758 11.5072755813599 202.956878662109 10.6949625015259 6.48499917984009 8.44369602203369 15.9900398254395 3.07795572280884 3.29637217521667 4.14038896560669 8.08072376251221 6.2835955619812 8.30768299102783 4.83121061325073 8.0195894241333 5.71480846405029 18.1465473175049 3.77086591720581 28.6915321350098 10.3104000091553 6.28122711181641 9.73269367218018 26.4187240600586 18.1647281646729 12.0182657241821 15.8916339874268 5.28814506530762 5.89505338668823 3.68696141242981 3.64989614486694 15.745343208313 26.4543056488037 4.40737724304199 3.40985536575317 8.04930400848389 4.34156513214111 5.3852219581604 10.6621103286743 1.59549796581268 2.54563021659851 36.6162452697754 7.72095489501953 11.4929141998291 22.4092674255371 14.7062959671021 12.0874843597412 15.4848766326904 14.2074375152588 2.47784042358398 11.1390075683594 7.47090911865234 20.2385158538818 14.2016773223877 11.6634254455566 18.7796306610107 28.8307685852051 24.0230121612549 4.65006351470947 16.6492099761963 750.451354980469 16.8327865600586 1.8801052570343 7.94221830368042 14.2579793930054 2.74251794815063 34.4111137390137 59.2962608337402 18.2554130554199 12.9894361495972 6.88616752624512 13.4648885726929 44.975399017334 19.4995098114014 3.15966463088989 13.3756418228149 4.39816570281982 4.32375526428223 8.82254981994629 14.4903869628906 17.6567420959473 12.3482522964478 10.5612459182739 14.1412057876587 2.4323673248291 13.2570123672485 7.75202083587646 26.9573707580566 1.49451982975006 28.4632911682129 10.3663940429688 1.28051030635834 24.8985805511475 34.9183235168457 24.9461669921875 12.9678354263306 16.6256847381592 13.0347032546997 9.05523109436035 2.03765249252319 7.08818960189819 10.5087108612061 6.35722255706787 4.73777389526367 6.21817636489868 9.80856513977051 4.156813621521 46.1925735473633 77.6594619750977 6.56056642532349 22.784236907959 19.0097980499268 34.1900520324707 5.14180660247803 9.29268836975098 4.86599016189575 3.39645028114319 3.59118914604187 59.7187194824219 26.9705505371094 4.54859828948975 35.9705772399902 5.18909215927124 6.49416399002075 4.96028137207031 12.9003267288208 5.7117862701416 4.8051962852478 10.8736219406128 10.7598810195923 8.64723587036133 11.3542251586914 10.772180557251 3.09301328659058 5.83277177810669 2.30625581741333 12.4506177902222 7.77755689620972 2.83650398254395 30.2586040496826 3.68023324012756 11.646559715271 27.8448696136475 19.5873374938965 18.4284782409668 2.1400306224823 4.07587051391602 2.53881049156189 31.3894920349121 11.0103425979614 5.74038553237915 4.70283603668213 11.9428596496582 22.3493518829346 15.1886291503906 13.4184188842773 60.1133003234863 22.2586269378662 18.5037994384766 30.4537525177002 26.3308887481689 60.6058692932129 2.67593169212341 37.7789573669434 4.01146984100342 7.055983543396 14.7087955474854 3.3922712802887 11.1212472915649 15.3248424530029 7.01737546920776 2.99646592140198 36.5956954956055 18.4076232910156 11.772798538208 22.862361907959 9.5220947265625 16.3005237579346 17.8444976806641 256.640563964844 18.2315235137939 14.5607385635376 6.03194141387939 1.14952862262726 28.7644481658936 56.1704788208008 12.7758808135986 24.1910648345947 20.3402271270752 5.95065307617188 7.32007074356079 7.02850294113159 4.58298254013062 20.8166179656982 2.28443026542664 4.62735319137573 22.6020908355713 19.6246547698975 23.2535228729248 6.77096700668335 10.5084457397461 7.28300046920776 361.264923095703 20.5178451538086 7.55778980255127 10.6179456710815 4.42418479919434 11.73499584198 19.7687568664551 36.5266647338867 72.4401245117188 10.6362390518188 10.5690984725952 5.56979846954346 18.4852352142334 2.58693242073059 15.1329708099365 23.3072929382324 4.84716510772705 9.33445072174072 12.9372701644897 12.5317029953003 4.43151426315308 3.60456418991089 17.4672031402588 23.050760269165 2.24519944190979 11.3669443130493 58.2199211120605 11.9317378997803 4.62824630737305 30.5390853881836 12.2714309692383 4.39622163772583 6.18569278717041 13.4581365585327 6.62764453887939 18.6050605773926 10.3123035430908 8.80299472808838 8.68584251403809

ENSG00000109971.12 1127.08032226562 1059.79577636719 303.718231201172 870.33056640625 610.903991699219 1085.89367675781 312.471008300781 960.961303710938 349.733642578125 844.611083984375 744.639953613281 674.389221191406 411.912872314453 701.648681640625 417.561462402344 648.585388183594 463.393463134766 725.296569824219 1242.16735839844 795.803588867188 482.413757324219 984.992431640625 392.725433349609 879.801879882812 564.933654785156 812.892761230469 809.086303710938 163.953308105469 624.223449707031 932.753112792969 725.346252441406 662.031860351562 64.1159210205078 849.354309082031 151.098754882812 295.906494140625 969.923034667969 863.509887695312 1004.14147949219 684.706176757812 634.168579101562 668.048583984375 596.505615234375 465.064910888672 242.54426574707 605.635314941406 917.76025390625 682.524047851562 363.982696533203 721.543640136719 496.956909179688 510.891571044922 539.442321777344 847.534301757812 564.7431640625 986.25732421875 1090.47375488281 1042.79040527344 698.58544921875 646.568664550781 748.461364746094 761.547790527344 1028.53869628906 816.952697753906 918.098449707031 831.286499023438 449.915252685547 620.654235839844 482.123596191406 495.758636474609 652.502868652344 560.51025390625 497.536010742188 757.848876953125 1055.19860839844 756.868774414062 1402.45251464844 719.18408203125 480.853363037109 804.878479003906 1179.16662597656 673.889892578125 1136.50622558594 657.766540527344 477.9638671875 764.92626953125 979.849609375 1091.14721679688 254.824966430664 327.152038574219 666.684448242188 893.6806640625 933.377624511719 689.501586914062 253.373275756836 418.490905761719 635.016967773438 324.640350341797 872.881591796875 542.893310546875 302.874603271484 996.0771484375 557.021484375 591.180541992188 862.942626953125 706.307434082031 1279.29162597656 688.739685058594 997.927673339844 1199.50708007812 625.526672363281 1005.66375732422 583.776184082031 355.656646728516 789.337219238281 864.035705566406 492.207763671875 446.062957763672 477.640319824219 316.751800537109 467.377777099609 948.084350585938 577.963684082031 582.229309082031 1461.60168457031 573.088989257812 1922.2548828125 37.2610282897949 913.380737304688 1102.83728027344 913.21728515625 538.370971679688 82.7723159790039 877.425354003906 1105.29968261719 701.354431152344 919.109680175781 1696.93408203125 575.740112304688 902.422241210938 344.905395507812 1309.94104003906 679.515197753906 388.770568847656 1278.3203125 959.280700683594 1158.51049804688 980.681274414062 601.813598632812 1286.05493164062 1127.15185546875 690.96044921875 442.3994140625 602.524353027344 1046.07641601562 340.687469482422 474.314147949219 806.979797363281 602.549438476562 1013.84259033203 718.8525390625 809.297790527344 112.107467651367 1059.30603027344 545.3173828125 956.330078125 767.421813964844 313.533966064453 212.685165405273 1140.33325195312 965.48681640625 1040.66857910156 872.725280761719 309.904602050781 969.936279296875 469.827453613281 528.58056640625 659.890380859375 500.590057373047 1809.01123046875 164.536529541016 364.852447509766 673.222290039062 622.708435058594 215.600631713867 825.443298339844 400.4677734375 541.538146972656 1392.86389160156 677.10693359375 819.23876953125 552.656005859375 787.586608886719 817.311401367188 967.322814941406 422.000640869141 302.318603515625 800.512634277344 290.088562011719 775.492065429688 525.352661132812 1360.31286621094 624.32861328125 866.949340820312 555.150634765625 526.100036621094 1049.50830078125 535.088989257812 351.666625976562 392.089874267578 311.531372070312 732.440368652344 525.04345703125 858.880981445312 1015.10858154297 1066.42980957031 86.6432800292969 424.780731201172 1584.11071777344 748.389282226562 781.838439941406 982.458862304688 755.753662109375 1174.22033691406 479.444183349609 892.342468261719 950.557983398438 304.56103515625 756.689880371094 903.946350097656 426.470458984375 426.719360351562 857.062622070312 530.209533691406 1369.0400390625 385.320831298828 1049.27160644531 492.947235107422 859.628295898438 37.6369781494141 725.60888671875 648.847351074219 463.722564697266 777.779846191406 1117.05212402344 324.535919189453 772.139709472656 382.104034423828 564.628723144531 666.629699707031 1204.50390625 682.654846191406 1190.07348632812 490.970520019531 1390.87731933594 497.266174316406 1249.41625976562

ENSG00000080824.17 905.222290039062 782.43603515625 361.227081298828 666.289794921875 525.975830078125 1597.05969238281 496.005828857422 294.519561767578 1108.38293457031 864.156188964844 784.415710449219 833.8369140625 752.759399414062 328.402252197266 413.346008300781 1222.55786132812 860.818603515625 714.68359375 1077.17028808594 1470.76806640625 355.526489257812 828.208740234375 550.337646484375 909.095581054688 975.887329101562 1264.64001464844 669.123291015625 238.445327758789 401.394378662109 458.550079345703 608.245239257812 570.716735839844 371.152587890625 716.488037109375 559.3447265625 414.719207763672 1040.56530761719 976.270141601562 1627.53698730469 382.052032470703 728.542663574219 357.790191650391 470.643585205078 183.712646484375 230.294326782227 458.053039550781 1501.75122070312 433.084655761719 624.757141113281 669.031005859375 774.348693847656 207.335723876953 422.346038818359 1048.55090332031 2292.61596679688 825.082275390625 536.197143554688 559.594665527344 584.528930664062 551.860168457031 500.433715820312 1441.31176757812 853.673278808594 527.74658203125 470.277282714844 366.420013427734 344.481658935547 542.870788574219 397.81494140625 474.385620117188 705.488037109375 1503.701171875 365.449493408203 803.096984863281 719.310241699219 1002.76525878906 1179.83215332031 594.320556640625 1569.75927734375 538.477966308594 516.440124511719 757.091125488281 1255.29077148438 488.667816162109 360.481231689453 507.510528564453 601.85107421875 716.559326171875 370.254791259766 699.213623046875 215.815811157227 563.153381347656 505.729644775391 369.757232666016 196.676300048828 309.977264404297 489.627593994141 617.349060058594 682.25244140625 1090.1240234375 434.263122558594 625.879943847656 588.028869628906 416.284423828125 545.399291992188 607.274963378906 412.171844482422 563.828735351562 556.112487792969 1691.27502441406 692.265441894531 698.804260253906 602.597045898438 757.733764648438 562.317443847656 1390.31262207031 554.780212402344 534.210327148438 474.898803710938 704.310424804688 442.962097167969 632.889221191406 595.541748046875 396.551452636719 4348.927734375 613.484313964844 860.946411132812 174.099655151367 597.843200683594 847.946228027344 661.249145507812 531.551940917969 200.72998046875 336.850189208984 938.962524414062 1237.546875 865.536437988281 1281.02001953125 405.092498779297 783.224060058594 332.99267578125 1117.18298339844 661.033081054688 318.520660400391 897.73095703125 754.908386230469 396.713775634766 860.541748046875 583.247375488281 875.78955078125 951.954467773438 567.046997070312 475.510925292969 746.400756835938 621.557250976562 553.286254882812 513.271850585938 700.121154785156 349.112121582031 578.441467285156 412.220764160156 639.719970703125 238.226333618164 968.691711425781 540.861328125 369.012542724609 736.989685058594 327.615692138672 455.1103515625 505.410736083984 572.911315917969 1058.89904785156 803.126281738281 341.320983886719 1337.72692871094 386.022033691406 372.288024902344 650.143737792969 1163.38671875 1766.55090332031 231.717712402344 452.130065917969 1401.39477539062 524.633605957031 628.139953613281 1335.00305175781 266.400299072266 946.894287109375 1556.75524902344 796.285827636719 513.605346679688 684.973999023438 557.96337890625 416.608093261719 679.349853515625 1432.99963378906 369.949157714844 539.69482421875 282.358276367188 306.661010742188 424.534149169922 607.337463378906 653.47509765625 608.540283203125 442.069793701172 609.40673828125 934.47705078125 493.188537597656 450.092163085938 1134.96936035156 638.737426757812 611.248352050781 361.480072021484 715.599914550781 639.162170410156 759.239318847656 251.39289855957 361.493530273438 2303.33862304688 835.785583496094 810.182861328125 745.379455566406 991.586181640625 1022.86004638672 241.134338378906 1696.06225585938 740.919860839844 373.140808105469 733.849365234375 570.439331054688 249.324523925781 837.530212402344 550.572814941406 401.088562011719 928.520263671875 503.504974365234 659.425415039062 813.932739257812 779.669982910156 125.717590332031 424.112579345703 266.955871582031 651.251159667969 538.39453125 611.890991210938 327.709045410156 561.562072753906 372.947784423828 474.40087890625 921.047973632812 601.889343261719 636.274291992188 1203.45764160156 866.244995117188 572.584777832031 703.502075195312 721.444580078125

ENSG00000096384.18 2209.46704101562 1938.85815429688 679.3037109375 2248.09741210938 771.952758789062 2759.5537109375 1145.61169433594 802.295166015625 904.31982421875 1291.76013183594 1694.70715332031 1895.53771972656 1464.59191894531 1335.54113769531 1414.24768066406 1153.48913574219 1514.01623535156 1609.25244140625 1642.21130371094 1451.18786621094 1016.84381103516 1966.73278808594 927.870239257812 2133.86206054688 1229.77465820312 1753.56958007812 1160.65942382812 647.772338867188 1767.13916015625 2388.76879882812 1406.4755859375 1832.79406738281 761.216552734375 1942.81042480469 1819.30847167969 906.711975097656 2750.92236328125 1386.21264648438 2298.2626953125 1311.25646972656 3079.1318359375 879.872314453125 1145.77416992188 2662.87182617188 1020.42053222656 808.0673828125 1808.74938964844 894.066650390625 1120.24938964844 2525.93505859375 1434.23828125 2163.1357421875 1618.23681640625 1897.45629882812 1675.71411132812 2773.4501953125 1642.8369140625 1207.05688476562 977.759033203125 1448.97827148438 1986.32055664062 1572.48559570312 1965.29260253906 1405.751953125 1070.63781738281 1056.14562988281 1656.52429199219 1740.98498535156 1412.22265625 1276.83093261719 1324.79040527344 907.798217773438 786.87255859375 2058.21264648438 1845.28369140625 2044.66918945312 2788.06298828125 1765.98645019531 4997.796875 1170.42749023438 1754.32287597656 1108.3603515625 2618.60009765625 2208.19458007812 783.532653808594 969.983276367188 868.666381835938 2291.31518554688 810.39794921875 2134.90795898438 596.219360351562 1126.03576660156 1189.35473632812 3213.31323242188 457.606109619141 1315.95776367188 1018.07659912109 1202.67919921875 1550.70300292969 1603.65112304688 1264.40502929688 1591.50341796875 773.105590820312 1480.34301757812 2045.97473144531 1532.85131835938 1358.48974609375 1063.81335449219 1328.73217773438 2966.142578125 1095.70971679688 2534.24536132812 1469.55029296875 1600.89965820312 1082.45703125 2354.64990234375 925.498046875 1590.68835449219 1595.92724609375 932.068176269531 873.288513183594 764.353881835938 1826.666015625 966.667358398438 2381.37646484375 1095.66442871094 938.766357421875 204.357955932617 1751.876953125 3658.673828125 1123.47839355469 1839.67797851562 705.086486816406 1533.01489257812 2175.3486328125 5363.94873046875 1101.74279785156 2127.02709960938 1242.29150390625 1562.49401855469 776.478881835938 1343.95593261719 1176.22216796875 1284.44580078125 2575.08349609375 1895.87915039062 1360.39099121094 1169.63195800781 1018.20538330078 1032.65405273438 2033.85766601562 2071.19897460938 844.591796875 1363.07897949219 1248.39501953125 1259.88513183594 880.345153808594 1217.11291503906 649.712463378906 1551.6181640625 1569.52746582031 1868.2158203125 403.846282958984 1391.72607421875 1509.77770996094 654.811279296875 2009.74304199219 280.364227294922 625.320007324219 2576.15600585938 1725.404296875 1356.39733886719 3328.45361328125 1285.6064453125 2853.77416992188 1134.46057128906 1256.9111328125 1189.33264160156 3774.00048828125 2073.8134765625 245.435302734375 1498.80847167969 1599.30993652344 1276.56750488281 717.120422363281 1661.74572753906 1092.71325683594 844.1845703125 3440.47241210938 1107.2763671875 1508.11608886719 5182.18310546875 2849.83129882812 1186.11169433594 1596.09692382812 2532.4599609375 994.579895019531 999.884887695312 595.091857910156 420.391418457031 783.313415527344 1780.41320800781 1648.58654785156 1272.4140625 1258.21630859375 1501.88012695312 1396.33923339844 818.11572265625 1600.88146972656 1123.73461914062 1215.16784667969 1543.1123046875 974.620788574219 1124.14270019531 1535.00207519531 1906.82739257812 466.618072509766 814.343505859375 1969.15270996094 1217.97412109375 2310.45043945312 2619.63159179688 1118.90576171875 1848.03564453125 699.727416992188 1088.21374511719 1358.4345703125 925.110656738281 1549.20739746094 1191.79431152344 1048.76977539062 1807.92834472656 1012.20477294922 2165.80126953125 1879.99267578125 1305.27197265625 1168.78942871094 692.498779296875 1061.30493164062 481.453186035156 2030.3193359375 1329.34387207031 1634.79895019531 1687.11328125 2392.13818359375 932.297546386719 1344.62744140625 1256.41931152344 858.095886230469 2664.78735351562 717.846008300781 1293.19189453125 2381.41357421875 1264.68542480469 1217.42004394531 1019.21936035156 1670.45361328125

ENSG00000090339.7 403.731506347656 326.93115234375 121.643836975098 1008.94879150391 255.833923339844 505.666442871094 261.947937011719 243.010101318359 27.4261703491211 174.869735717773 774.193603515625 118.29793548584 319.421142578125 72.6716613769531 1029.12353515625 125.870582580566 72.9197311401367 452.347961425781 254.607986450195 310.873413085938 145.160034179688 239.415985107422 160.69319152832 551.815002441406 8.42377471923828 117.976081848145 268.333740234375 161.912307739258 323.379699707031 388.761016845703 132.93391418457 218.350189208984 126.226539611816 452.230560302734 84.6709518432617 259.916564941406 247.603912353516 98.9874496459961 213.045425415039 215.155746459961 357.555206298828 212.653656005859 166.189834594727 345.59765625 59.7153854370117 302.067657470703 37.6060676574707 546.523376464844 144.803161621094 458.993499755859 39.3387603759766 412.773895263672 865.854919433594 220.301803588867 174.227676391602 356.699920654297 102.89086151123 258.996917724609 125.747367858887 583.499877929688 612.904846191406 116.606620788574 513.573303222656 820.542419433594 264.627685546875 198.341995239258 130.966735839844 115.83430480957 224.200057983398 384.878021240234 136.996200561523 143.18147277832 96.5303039550781 1654.48181152344 125.218002319336 86.4230194091797 74.3940124511719 553.512512207031 47.5551910400391 151.288192749023 236.570861816406 126.736618041992 431.953826904297 1032.49267578125 84.3531646728516 247.778015136719 313.413940429688 107.730087280273 589.474182128906 255.75830078125 226.225433349609 195.81135559082 32.2300643920898 1159.37463378906 32.271427154541 371.526184082031 310.597381591797 156.398101806641 268.290557861328 464.91357421875 83.4006576538086 577.1162109375 82.7431030273438 511.539306640625 77.1552505493164 940.000732421875 241.072784423828 97.9806671142578 217.773406982422 41.4926872253418 387.398712158203 204.562576293945 509.230560302734 136.729049682617 236.59553527832 136.651702880859 276.4130859375 15.0808458328247 47.3771667480469 59.5471649169922 305.146575927734 12.0815477371216 72.5013275146484 799.160278320312 53.6356925964355 64.8220367431641 21.076452255249 118.66130065918 590.24365234375 476.335754394531 89.5541915893555 138.523727416992 19.2548236846924 91.5175018310547 37.8783149719238 1014.05798339844 78.5583419799805 700.643798828125 208.289413452148 57.6118545532227 41.5225639343262 190.931488037109 419.401641845703 437.937591552734 869.220336914062 835.349426269531 270.091552734375 529.754760742188 235.569366455078 30.0835227966309 210.036102294922 155.936111450195 392.847564697266 231.984649658203 87.0324783325195 329.123992919922 116.473739624023 158.87858581543 221.407608032227 435.487091064453 802.009155273438 206.179885864258 14.3752012252808 159.744384765625 410.510650634766 265.455108642578 67.6295394897461 185.652114868164 24.4380893707275 158.738983154297 473.266967773438 169.966857910156 27.8200702667236 131.045883178711 71.7160415649414 221.2578125 106.381065368652 700.4638671875 513.4677734375 42.1957550048828 70.5582733154297 457.050720214844 163.951202392578 265.324188232422 60.2635841369629 57.8982467651367 205.737564086914 213.170104980469 34.7518310546875 262.615142822266 1058.439453125 746.980712890625 387.002227783203 316.940734863281 810.060974121094 440.360870361328 642.889465332031 286.707702636719 249.060516357422 31.4258193969727 412.960510253906 334.001708984375 39.8782920837402 386.704406738281 280.761077880859 159.382431030273 139.167770385742 231.209136962891 177.124740600586 70.7593307495117 102.955787658691 91.7616806030273 343.208801269531 146.114517211914 186.409851074219 590.811767578125 57.1784210205078 347.269897460938 1014.83081054688 585.824157714844 596.5322265625 617.539855957031 62.0188598632812 320.7216796875 176.842239379883 204.053039550781 288.3271484375 125.207992553711 275.197082519531 140.995834350586 817.362060546875 150.921401977539 901.089721679688 426.791717529297 231.531158447266 152.308044433594 244.330001831055 57.2924003601074 75.9261932373047 19.2535667419434 1077.54016113281 275.863464355469 61.0809440612793 925.248840332031 252.667175292969 187.250152587891 157.843597412109 293.667510986328 208.977020263672 171.187637329102 186.638824462891 253.502548217773 142.518630981445 149.830841064453 242.350128173828 213.482330322266 421.014404296875

ENSG00000197919.4 0 0.275567263364792 0 0 0 0.0767422989010811 0 0 0 0 0.0450949817895889 0 0 0 0 0.0621277913451195 0 0 0 0 0.199030339717865 0.091339647769928 0 0 0 0 0 0 0 0 0.125756695866585 0 0 0 0 0 0 0 0 0 0 0 0 0 0 0 0 0 0 0 0 0 0 0 0 0 0 0 0.286591619253159 0 0 0.0317331105470657 0.0429298765957355 0 0 0 0 0 0 0 0.622970640659332 0 0 0 0 0 0 0 0 0.0505640134215355 0 0 0 0 0.0713687613606453 0 0 0 0.0389744117856026 0 0 0 0.0376844853162766 0.043799314647913 0.098600372672081 0 0.123499572277069 0 0.104412861168385 0 0 0 0 0 0 0 0 0 0.263107985258102 0 0.0433998890221119 0 0 0 0 0 0 0 0 0.0690967291593552 0 0 0 0 0 0 0.0874121785163879 0.253564953804016 0.035277247428894 0 0.377653151750565 0 0 0 0.0329586863517761 0 0.0437085181474686 0.256328165531158 0.0790423527359962 0 0 0.0265279058367014 0.0378889441490173 0 0 0 0 0 0 0 0 0 0.0581889450550079 0 0 0 0 0 0.111291520297527 0 0 0 0 0 0 0 0 0.197363123297691 0 0 0 0.358354568481445 0 0 0 0.0714889541268349 0 0 0 0.756921648979187 0.23910391330719 0 0.142003729939461 0 0 0 0 0 0 0 0 0 0 0 0 0 0 0.212860181927681 0 0 0 0 0 0 0 0 0 0.0622984282672405 0 0 0 0.762851774692535 0 0 0.143856659531593 0 0.150273308157921 0 0 0.0901546478271484 0 0 0.101086460053921 0.0510180741548538 0.0551587156951427 0 0 0.085772916674614 0 0.100031368434429 0.0560657419264317 0 0 0 0 0.0509154610335827 0 0 0.0785590782761574 0 0 0 0.0710303336381912 0 0 0 0 0 0 0 0 0 0 0.157768979668617 0 0 0

ENSG00000188379.6 0 0 0 0 0 0 0 0 0 0 0 0 0 0 0 0 0 0 0 0 0 0.0927882120013237 0 0 0 0 0 0 0 0 0 0 0 0 0 0 0 0 0 0 0 0 0 0 0 0 0 0 0 0 0 0 0 0 0 0 0.0782027617096901 0 0 0 0 0 0 0 0 0 0 0 0 0 0 0 0 0 0 0 0 0 0 0 0 0 0 0 0 0 0 0 0 0 0 0 0 0 0 0 0 0 0 0 0 0 0 0 0 0 0 0 0 0 0 0 0 0 0 0 0 0 0 0 0 0 0 0 0 0 0 0 0 0 0 0 0 0 0 0 0 0.0371990390121937 0 0 0 0 0 0 0 0 0 0 0 0 0 0 0 0 0 0 0 0 0 0 0 0 0 0 0 0 0 0 0.0399175621569157 0 0 0 0 0 0 0 0 0 0 0 0 0 0 0 0 0 0 0 0 0 0 0 0 0 0 0 0 0 0 0 0 0 0 0 0 0 0 0 0 0 0 0 0 0 0 0 0 0 0 0 0 0 0 0 0 0 0 0 0 0 0 0 0 0 0 0 0 0 0 0 0 0 0 0 0 0 0 0 0 0 0 0 0 0 0 0 0

ENSG00000236637.2 0 0 0 0 0 0 0 0 0 0 0 0 0 0 0 0 0 0 0 0 0 0.107683658599854 0 0 0 0 0 0 0 0 0 0 0 0 0 0 0 0 0 0 0 0 0 0 0 0 0 0 0 0 0 0 0 0 0 0 0 0 0 0 0 0 0 0 0 0 0 0 0 0 0 0 0 0 0 0 0 0 0 0 0 0 0 0 0 0 0 0 0 0 0 0 0 0 0 0 0 0 0 0 0 0 0 0 0 0 0 0 0 0 0 0 0 0 0 0 0 0 0 0 0 0 0 0 0 0 0 0 0 0.0577104762196541 0 0 0 0 0 0 0 0 0 0 0 0 0 0 0 0 0 0 0 0 0 0 0 0 0 0 0 0 0 0 0 0 0 0 0 0 0 0 0 0 0 0 0 0 0 0 0 0 0 0 0 0 0 0 0 0 0 0 0 0 0.147499784827232 0 0 0 0 0 0 0 0 0 0 0 0 0 0 0 0 0 0 0 0 0 0 0 0.0339195765554905 0 0 0 0 0 0 0 0 0 0 0 0 0 0 0 0 0 0 0 0 0 0 0 0 0 0 0 0 0 0 0 0 0 0 0 0 0 0 0 0 0 0

ENSG00000147873.5 0.0566302873194218 0 0 0.0702312439680099 0 0 0 0.0460790544748306 0 0 0 0 0 0.253949224948883 0 0 0 0.0651733130216599 0 0.299569219350815 0 0.106701739132404 0 0 0 0 0 0 0.221979930996895 0 0 0 0 0 0.0584418252110481 0.22161939740181 0 0 0.143436565995216 0.0958668142557144 0.0638884902000427 0 0 0.0580061003565788 0 0 0 0.0611542761325836 0.0414001978933811 0 0 0 0.0595967695116997 0 0 0 0 0 0.066958487033844 0 0 0 0 0 0 0 0 0 0 0 0 0 0.109701327979565 0 0 0 0 0 0 0 0 0 0.0483977608382702 0 0 0 0 0 0 0 0.0480394847691059 0 0 0.0511657670140266 0.230367213487625 0.132103323936462 0 0 0.12197370827198 0 0.0912984907627106 0.176190197467804 0 0 0 0 0.0762709006667137 0 0.122943669557571 0 0.0506991595029831 0 0 0 0.0623287670314312 0 0 0 0 0 0 0 0 0 0 0 0 0.0493685267865658 0.0412104018032551 0.114368483424187 0 0 0 0 0.0385018885135651 0 0 0 0 0 0 0 0 0 0 0 0.0952873453497887 0 0 0 0 0 0.0679755359888077 0.0929526016116142 0 0 0 0 0 0 0 0 0 0 0.244820609688759 0 0 0.461113840341568 0 0.159154906868935 0 0 0 0 0 0 0 0 0 0.0803841426968575 0 0 0 0.103152066469193 0 0 0 0 0 0 0 0.0682243853807449 0 0 0.217045903205872 0.0866736173629761 0.054608840495348 0.0828867852687836 0 0 0 0 0 0 0 0.25756448507309 0 0.0727761760354042 0 0 0 0.0469027943909168 0 0.110924504697323 0.0672205612063408 0 0 0 0 0 0 0 0 0 0 0 0.0444149486720562 0 0 0.116855300962925 0 0 0 0 0 0 0 0 0.0458858273923397 0.0450989007949829 0 0 0 0 0 0.0709474459290504 0 0.144413143396378 0 0 0 0.0632671713829041 0 0 0 0.0642760172486305 0

ENSG00000120235.4 0 0 0 0 0 0 0 0 0 0 0 0 0 0 0 0 0 0 0 0 0 0 0 0 0 0 0 0 0 0 0 0 0 0 0 0 0 0 0 0 0 0 0 0 0 0 0 0 0 0 0 0 0 0 0 0 0 0 0 0 0 0 0 0 0 0 0 0 0 0 0 0.0298534240573645 0 0 0 0 0 0 0 0 0 0 0 0 0 0 0 0 0 0 0 0 0 0 0 0 0 0 0 0 0 0 0 0 0 0 0 0 0 0 0 0 0 0 0 0 0 0 0 0 0 0 0 0 0 0 0 0 0 0 0 0 0 0 0 0 0 0 0 0 0 0 0 0 0 0 0 0 0 0 0 0 0 0 0 0 0 0 0 0 0 0 0 0 0 0 0 0 0 0 0 0 0 0 0 0 0 0 0 0 0 0 0 0 0 0 0 0 0 0 0 0 0 0 0 0 0 0.0529852733016014 0 0 0 0 0 0 0 0 0 0 0 0 0 0 0 0 0 0 0 0 0 0 0 0 0 0 0 0 0 0 0 0 0 0 0 0 0 0 0 0 0.0293324533849955 0 0 0 0 0 0 0 0 0 0 0 0 0 0 0 0 0 0

ENSG00000214042.1 0 0 0 0.0940545946359634 0 0 0 0 0 0 0 0 0 0 0 0 0 0 0 0 0 0 0 0 0 0 0 0 0 0 0 0 0 0 0 0 0 0 0 0 0 0 0 0 0 0 0 0 0 0 0 0 0 0 0 0 0 0 0 0 0 0 0 0 0 0 0 0 0 0 0 0 0 0 0 0 0 0 0 0 0 0 0 0 0 0 0 0 0 0 0 0 0 0 0 0 0 0 0 0 0 0 0 0 0 0 0 0 0 0 0 0 0 0 0 0 0 0 0 0 0 0 0 0 0 0 0 0 0 0 0 0 0 0 0 0 0 0 0 0 0 0 0 0 0 0 0 0 0 0 0 0 0 0 0 0 0 0 0 0 0 0 0 0 0 0 0 0 0 0 0 0 0 0 0 0 0 0 0 0 0 0 0 0 0 0 0 0 0 0 0 0 0 0 0 0 0 0 0 0 0.0417778752744198 0 0 0 0 0 0 0 0 0 0 0 0 0 0 0 0 0 0 0 0 0 0 0 0 0 0 0 0 0 0 0 0 0 0 0 0 0 0 0 0 0 0 0 0 0 0.130609765648842 0 0 0 0 0 0 0 0 0 0

ENSG00000120242.3 0 0 0 0 0 0.0425812639296055 0 0 0 0 0 0 0 0 0 0 0 0 0 0 0 0 0 0 0 0 0 0 0 0 0 0 0 0 0 0 0 0 0 0 0 0 0 0 0 0 0 0 0 0 0 0 0 0 0 0 0 0 0.0636073350906372 0 0 0 0 0 0 0 0 0 0 0 0 0 0.0347369946539402 0 0 0 0 0 0 0 0 0 0 0 0 0 0.200181484222412 0 0 0 0 0 0 0 0 0 0 0 0 0 0 0 0 0 0 0 0 0 0 0 0 0 0 0 0 0 0 0 0 0 0 0 0 0 0 0 0 0 0 0 0 0 0 0 0 0 0 0.0812721997499466 0 0 0 0 0 0 0 0 0 0 0 0 0 0 0 0 0 0 0 0 0 0 0 0 0 0 0 0 0 0 0 0 0 0 0 0 0 0 0 0 0 0.0763610750436783 0 0 0 0 0 0 0 0 0 0 0 0.0648098886013031 0 0 0 0 0 0 0 0 0 0 0 0 0 0 0 0 0 0 0 0.089110791683197 0 0 0 0 0 0 0 0 0 0 0 0 0 0 0 0 0 0.222013831138611 0 0 0 0 0 0 0 0 0 0 0 0 0 0 0 0 0 0 0 0 0 0 0.0716791376471519 0 0 0 0

ENSG00000186803.3 0 0 0 0 0 0 0 0 0 0 0 0 0 0 0 0 0 0 0 0 0 0 0 0 0 0 0 0 0 0 0 0 0 0 0 0 0 0 0 0 0 0 0 0 0 0 0 0 0 0 0 0 0 0 0 0 0 0 0 0 0 0 0 0 0 0 0 0 0 0 0 0 0 0 0 0 0 0 0 0 0 0 0 0 0 0 0 0 0 0 0 0 0 0 0 0 0 0 0 0 0 0 0 0 0 0 0 0 0 0 0 0 0 0 0 0 0 0 0 0 0 0 0 0 0 0 0 0 0 0 0 0 0 0 0 0 0 0 0 0 0 0 0 0.0571539290249348 0 0 0 0 0 0 0 0 0 0.0476345866918564 0 0 0 0 0 0 0 0 0 0 0 0 0 0.11815145611763 0 0 0 0 0 0 0 0 0 0 0 0 0 0 0 0 0 0 0 0 0 0 0 0 0 0 0 0 0 0 0 0 0 0 0 0 0 0 0 0 0 0 0 0 0 0 0 0 0 0 0 0 0 0 0 0 0 0 0 0 0 0 0 0 0 0 0 0 0 0 0 0 0 0 0 0 0 0 0 0 0 0 0 0 0 0 0 0 0

ENSG00000233816.3 0 0 0 0 0 0 0 0 0 0 0 0 0 0 0 0 0 0 0 0 0 0 0 0 0 0 0.0207776166498661 0 0 0 0 0 0 0 0 0 0 0 0 0 0 0 0 0 0 0 0 0 0 0 0 0 0 0 0 0 0 0 0 0 0 0 0 0 0 0 0 0 0 0 0.0388935022056103 0 0 0 0 0 0 0 0 0 0 0 0 0 0 0 0 0 0 0 0 0 0 0 0 0 0 0 0 0 0 0 0 0 0 0 0 0 0 0 0 0 0 0 0 0 0 0 0 0 0 0 0 0 0 0 0 0 0 0 0 0 0 0 0 0 0 0 0 0 0 0 0 0 0.0362597852945328 0 0 0 0 0 0 0 0 0 0 0 0 0 0 0 0 0 0 0 0 0 0 0 0 0 0 0 0 0 0 0 0 0 0 0 0 0 0 0 0 0 0 0 0.0680914521217346 0 0 0 0 0 0 0.0277930032461882 0 0 0 0 0 0 0 0 0 0 0 0 0 0 0 0.0300799608230591 0 0 0 0 0 0 0 0 0 0 0 0 0 0 0 0 0 0 0 0 0 0 0 0 0 0 0 0 0 0 0 0 0 0 0 0 0 0 0 0 0 0 0.0224079601466656 0 0

ENSG00000228083.2 0 0 0 0 0 0 0 0 0 0 0 0 0 0 0 0 0 0 0 0 0 0 0 0 0 0 0 0 0 0 0 0 0 0 0 0 0 0 0 0 0 0 0 0 0 0 0 0 0 0 0 0 0 0 0 0 0 0 0 0 0 0 0 0 0 0 0 0 0 0.109364323318005 0 0 0 0 0 0 0 0 0 0 0 0.0608165897428989 0 0 0 0 0 0 0 0 0 0 0 0 0 0 0 0 0 0 0 0 0 0 0 0 0 0 0.122571110725403 0 0 0 0 0 0 0 0 0 0 0 0 0 0 0 0 0 0 0 0 0 0 0 0 0 0.0383852161467075 0 0 0 0 0 0 0 0 0 0 0 0 0 0 0 0 0 0 0 0 0 0 0 0 0 0 0 0 0 0 0 0 0 0 0 0 0 0 0 0 0 0 0 0 0 0 0.0563290789723396 0 0 0 0 0 0 0 0 0.0728559643030167 0 0 0 0 0.043205488473177 0 0 0 0 0 0 0 0 0 0 0 0 0 0 0 0.0467606633901596 0 0 0 0 0 0 0 0 0 0 0 0 0 0 0 0 0 0 0 0 0 0 0 0 0 0 0 0 0 0 0 0 0 0 0 0 0 0 0 0 0 0 0 0 0

ENSG00000147885.4 0 0 0 0 0 0 0 0 0 0 0 0 0 0 0 0 0 0 0 0 0 0 0 0 0 0 0 0 0 0 0 0 0 0 0 0 0 0 0 0 0 0 0 0 0 0 0 0 0.0435165017843246 0 0 0 0 0 0 0 0 0 0 0 0 0 0 0 0 0 0 0 0 0 0 0 0 0 0 0 0 0 0 0 0 0 0 0 0 0 0 0 0 0 0.10099034756422 0 0 0 0.363214790821075 0 0 0 0 0 0 0 0 0 0 0 0 0 0 0 0 0 0 0 0 0 0 0 0 0 0.11057086288929 0 0 0 0 0 0 0 0 0 0 0 0 0 0 0 0 0 0 0 0 0 0 0 0 0 0 0.0483469143509865 0 0 0 0 0 0 0 0 0 0 0 0 0 0 0 0 0 0 0 0 0 0 0 0 0 0 0 0 0 0 0 0 0 0 0 0 0 0 0 0 0 0 0 0 0 0 0 0 0 0 0 0 0 0 0 0 0 0 0 0 0 0 0 0 0 0 0 0 0 0 0 0 0.153900176286697 0 0 0 0 0 0 0 0 0 0 0 0 0 0 0 0 0 0 0 0 0 0 0 0 0 0 0 0 0 0 0 0 0 0 0 0

ENSG00000234829.3 0 0 0 0 0 0 0 0 0 0 0 0 0 0 0 0 0 0 0 0 0 0 0 0 0 0 0 0 0 0 0 0 0 0 0 0 0 0 0 0 0 0 0 0 0 0 0 0 0 0 0 0 0 0 0 0 0 0 0 0 0 0 0 0 0 0 0 0 0 0 0 0 0 0 0 0 0 0 0 0 0 0 0 0 0 0 0 0 0 0 0 0 0 0 0.173965156078339 0 0 0 0 0 0 0 0 0 0 0 0 0 0 0 0 0 0 0 0 0 0 0 0 0 0 0 0 0 0 0 0 0 0 0 0 0 0 0 0 0 0 0 0 0 0 0 0 0 0 0 0 0 0 0 0 0 0 0 0 0 0 0 0 0 0 0 0 0 0 0 0 0 0 0 0 0 0 0 0 0 0 0 0 0 0 0 0 0 0 0 0 0 0 0 0 0 0 0 0.040976345539093 0 0 0 0 0 0 0 0 0 0 0 0 0 0 0 0 0 0 0 0 0 0 0 0 0 0 0 0 0 0 0 0 0 0 0 0 0 0 0 0 0 0 0 0 0 0 0 0 0 0 0 0 0 0 0 0 0 0 0 0 0 0

ENSG00000137080.4 0 0 0 0 0 0 0 0.0444140918552876 0 0 0 0 0 0 0 0 0 0.0628184154629707 0 0 0 0.102846302092075 0.043063398450613 0 0 0 0 0 0 0 0 0.0520134344696999 0 0 0 0 0 0 0 0 0.0615800134837627 0 0 0 0 0 0 0 0.0399042926728725 0 0 0.0505083911120892 0 0 0 0 0 0 0.0322695411741734 0.104357004165649 0.0641373097896576 0.0714614763855934 0 0.0519625209271908 0 0.0956210941076279 0 0.0588379837572575 0 0 0 0.0450133681297302 0.0352458357810974 0 0 0 0 0 0 0 0.030911372974515 0 0.0466490127146244 0 0 0 0.101556919515133 0 0.0438842736184597 0 0.092607356607914 0.0986317172646523 0 0.0493170060217381 0.111021712422371 0 0 0 0 0.133644759654999 0 0 0.0652700364589691 0 0 0 0 0.237194523215294 0 0 0.048867255449295 0 0 0 0 0 0 0 0 0 0 0 0.0388097167015076 0.168699786067009 0 0 0 0.0951694026589394 0 0 0 0 0 0.0316165275871754 0 0 0 0 0 0 0 0.0298697967082262 0.0426620617508888 0 0.0544959045946598 0.0454630441963673 0 0 0.133025214076042 0 0 0.0731503218412399 0 0.0447969809174538 0 0.0977150201797485 0 0 0 0 0 0.0532805398106575 0.0300553869456053 0 0.0786581784486771 0 0 0.444452524185181 0 0 0.0534471161663532 0.201749429106712 0.106657579541206 0 0 0.0804948806762695 0 0 0 0 0.269225418567657 0 0.07994644343853 0 0 0 0 0 0 0 0 0 0 0 0.104601718485355 0 0 0 0 0 0 0 0.0833286046981812 0 0 0 0 0.0701465681195259 0 0 0 0 0.11242438852787 0 0.0323958471417427 0 0.0338408425450325 0.0720652788877487 0 0.101512022316456 0 0 0 0 0 0 0 0 0 0 0 0 0 0 0 0.11465921998024 0 0 0 0 0.0806462243199348 0.060104064643383 0 0 0 0 0 0 0.0372882336378098 0 0 0 0 0 0 0 0

ENSG00000111537.4 16.5204200744629 5.21722602844238 0.379129141569138 5.00821399688721 11.2132539749146 1.52558410167694 1.94902896881104 5.67566823959351 3.79726457595825 1.62757313251495 0.640326499938965 1.46782886981964 0.117953859269619 1.23471868038177 0.255839645862579 8.88065338134766 0 9.29506301879883 3.54789781570435 4.4909553527832 2.35511040687561 3.54507327079773 0.434452444314957 0.115071170032024 0.466017454862595 0.503280758857727 0.210027903318405 0.950726926326752 1.0792818069458 0.357392638921738 0.476182192564011 2.71118950843811 0.843938171863556 0.575945913791656 0.568296432495117 1.6162930727005 4.10710525512695 4.49280309677124 3.13829278945923 2.4859254360199 0.310630202293396 7.46916770935059 1.63740146160126 2.2562370300293 19.2292346954346 0.105546534061432 1.98279368877411 0.421226441860199 0.503226518630981 0 0.360746741294861 0.806807398796082 0.0482939295470715 2.21756958961487 2.12771129608154 0.166384845972061 0.655863046646118 3.90881705284119 0.488335162401199 2.017911195755 6.7941312789917 1.68221962451935 0.487666428089142 0.218430310487747 1.73370206356049 0.0803907960653305 2.87834978103638 0.0989328250288963 0.340362668037415 0.800029575824738 1.08116269111633 13.5859050750732 0.148159846663475 2.15006923675537 4.46312427520752 1.40912687778473 0.879701316356659 0.28844428062439 1.26352882385254 0.717984080314636 3.22249817848206 12.2097539901733 0.941252946853638 5.1927809715271 0.810721099376678 4.49153470993042 0.256143540143967 0.896961510181427 6.12448644638062 4.01034784317017 1.12892782688141 0.414609491825104 0.463754266500473 0.0829238295555115 5.69364547729492 0.267623126506805 6.46895837783813 0.716201841831207 1.28492987155914 0.561790823936462 0.147966518998146 0.356937021017075 2.03033757209778 1.84126782417297 0 1.39829909801483 1.42153191566467 0 0.249066919088364 0.665471792221069 6.53232383728027 4.58975028991699 1.61820614337921 0.0881034508347511 2.47488164901733 1.86661791801453 1.17181396484375 0 0 0 1.53437495231628 0.203847154974937 0.65256404876709 1.91470086574554 3.47075247764587 0.384880065917969 0.165494650602341 1.96027100086212 0.534314155578613 0.185355812311172 0.0714998468756676 2.06199741363525 2.89259457588196 0.106322906911373 0.592796325683594 0 0.413759618997574 10.4685678482056 5.31251525878906 3.52454566955566 0.930684506893158 0.0502244271337986 0.573871254920959 1.03932869434357 12.4161176681519 0 0.308862417936325 2.04998064041138 2.79593229293823 1.22046566009521 2.33119082450867 0.184497356414795 1.43217432498932 0.338956445455551 1.91566908359528 3.94325804710388 8.24436187744141 0.28378900885582 2.00169396400452 0.462174236774445 1.01460409164429 0.403147339820862 0.783315479755402 0.229866713285446 2.51292872428894 3.37983584403992 0.208034589886665 14.1991271972656 0.743947982788086 0.386911064386368 0.539210319519043 0.508845806121826 0.269008249044418 0.662684857845306 0.936523199081421 0.406043171882629 0.729458212852478 0.510894954204559 0.491420716047287 0.130277767777443 16.8626174926758 1.14461803436279 3.96555352210999 2.08971858024597 2.40544438362122 0.27084693312645 0.0539931207895279 4.8955717086792 0 9.57745742797852 0.88826847076416 10.6147737503052 1.65719485282898 8.87052917480469 3.82543420791626 16.0136985778809 0.929291844367981 0.470168173313141 0.439901322126389 1.54918467998505 1.51676332950592 2.55904674530029 2.80224919319153 11.4776468276978 0.458123683929443 2.2958767414093 1.85625648498535 0.235895201563835 0.421482920646667 9.57311248779297 0.304064899682999 0.608119010925293 0.661624014377594 0.179774194955826 3.1048948764801 15.4188079833984 0.739719748497009 3.39286637306213 2.88875150680542 0.170686885714531 0 6.605637550354 1.24399411678314 14.4403352737427 1.09651720523834 1.85704112052917 2.30345106124878 14.3716106414795 1.59708118438721 3.0301787853241 0.743032217025757 0.474387913942337 3.02954626083374 0.310050785541534 5.09829807281494 1.301353931427 0.80090868473053 0.227279230952263 0.669300138950348 0.438547879457474 2.67814350128174 4.14352893829346 0.504297852516174 2.253169298172 2.21025776863098 0.574918925762177 0 0 0.313490569591522 0.385488003492355 0.673114836215973 8.30545234680176 6.48137092590332 1.12012100219727 0.707837760448456 1.3021434545517 0.522035658359528

ENSG00000125498.18 0.300505876541138 0.12653486430645 0.206890389323235 0.414087414741516 0.543916523456573 0.0528577491641045 0.182982012629509 0.0815054178237915 0.0657829567790031 0 0.0621200874447823 2.90730667114258 0.0286076832562685 0.074864961206913 0.248198106884956 0.599084198474884 0.0304305367171764 0.115279674530029 0.303699433803558 0.264941573143005 0.788244485855103 0.25164783000946 0.0790267437696457 0 0 0.0915465876460075 0.0573060065507889 0.0314430706202984 0.0436268821358681 0.065009593963623 0.115489825606346 0.0318170562386513 0 0.0465619340538979 0.0689152702689171 0.0435560196638107 0.253725618124008 0 0.422855079174042 0 0 0.359309494495392 0 0 0.150442615151405 0 0 0.883394360542297 0.146458759903908 0 5.11833667755127 0.154482066631317 0 0.586727440357208 0.087629422545433 0.0403537936508656 0.15906834602356 0 0.0394791066646576 0.127672120928764 0.156933352351189 0.152997568249702 0 0 0.300342679023743 0.233968928456306 0.0930792465806007 0 0.0619118474423885 0 0.143027722835541 0.302885621786118 0.237161949276924 0.167612925171852 0.0457375086843967 0.341759532690048 0 0 144.653671264648 0.417923331260681 0.397083759307861 0.215800806879997 0.114142380654812 0.269875705242157 0 0 0.124246455729008 0 0.107377529144287 0.203576028347015 0 0.301669269800186 0.0259559210389853 0.0301676280796528 0 0.0389444418251514 0.283542424440384 0.0651384294033051 0 0.081751637160778 0 0.103882752358913 0.359336107969284 0.121791251003742 0.0846716016530991 0.699462890625 0 0 0.0362441465258598 1.34883260726929 0.836990356445312 0.139145642518997 0.0981170386075974 0 0.0734987929463387 0 0 0.037303764373064 0.486711710691452 0.0475917123258114 0.0620227195322514 7.81146240234375 0.189921855926514 0.103195086121559 0.0260341726243496 0.458244323730469 0 1.45539832115173 0.0728936642408371 0.0674323067069054 0 0.046884573996067 0.0668142139911652 0.0193400979042053 0.204308405518532 0 0.0301050916314125 1.00886285305023 1.36104881763458 0.148356854915619 0.253936767578125 0.0913580432534218 0.0521934926509857 0.197273224592209 0.16667802631855 0 0.0561819635331631 0 0.773040115833282 1.18401193618774 0.0399099066853523 0.0894933342933655 0.200393825769424 0 0.0409952402114868 0 7.44651985168457 0 0.996504545211792 0.12610399723053 0.158190950751305 0.0651843175292015 0.404472947120667 0.0627190098166466 0.0962317511439323 0 0.129741996526718 0.0679688453674316 0.378906816244125 0 0 0.0617059133946896 0 0.111269675195217 0.0524163730442524 0.0492394007742405 0.0707670301198959 0.085783027112484 0.0715113952755928 0 0 0.0333128981292248 2.5919075012207 0.0608190521597862 0.250028282403946 5.54605197906494 0 0.530512273311615 0 0.360441952943802 0.0861737206578255 0.201127454638481 0.178632974624634 1.06955063343048 0.0639857575297356 0.051103264093399 0.0643953755497932 0.0977410525083542 0.0984835550189018 0 0.147145971655846 0.0903863459825516 0.203891262412071 0 0.238092944025993 0.151861518621445 0.119171433150768 0 0 0 0.138273254036903 0.0276541579514742 0.0343854762613773 0 0.0990840718150139 0 0.0621021948754787 0.13224883377552 0.28024685382843 0 0.0863275155425072 0.0775201991200447 0.278501033782959 0.983911275863647 0.341924726963043 0.210183620452881 0.707057297229767 0.354466557502747 0 0 0.0772327333688736 0.0265510734170675 0.435057938098907 0 0.775122344493866 0.0350690111517906 0.0647488832473755 0.110245354473591 0.0270545464009047 0.0531811378896236 0.073997974395752 0.036766167730093 0.0489235185086727 0 0.0554545000195503 0 0.0575026273727417 0.042573407292366 0.0228095278143883 0.112192213535309 0.0544174909591675 0.559540271759033 0 0.162999674677849 0.0412017367780209 0.0757950097322464 0.126610785722733

ENSG00000243772.5 0.41467696428299 0.0827096328139305 0 0.351869225502014 0.177765771746635 0.0172752570360899 0.0897047743201256 0.159828260540962 0.042999092489481 0.134620249271393 0.02030242793262 0.736875653266907 0 0.0978711023926735 0.162234932184219 0.139854252338409 0 0.0753526613116264 0.231598988175392 0 0.358425557613373 0.123367376625538 0.0172186344861984 0.0273636616766453 0 0.0299197565764189 0.0874022841453552 0.102763958275318 0.0855502560734749 0.127480730414391 0.0566175244748592 0.083188995718956 0 0.121740996837616 0.112616330385208 0.142352163791656 0.294840842485428 0.162285327911377 0.0552799180150032 0.0369467176496983 0 0.322936594486237 0.111248686909676 0 0.0491684749722481 0 0.117875955998898 0.259254962205887 0.0159554835408926 0 8.40690612792969 0.100977353751659 0.0229683741927147 0.527332961559296 0.0381860360503197 0.211018338799477 0.0346583910286427 0.0364512838423252 0.0516111105680466 0 0.205159246921539 0.0714335739612579 0.0386553257703781 0 0 0 0.030420670285821 0 0 0.0422767177224159 0.2570980489254 0.161984845995903 0.0140928300097585 0 0.0597927235066891 0.148927628993988 0.013074436224997 0.137182787060738 7.6462984085083 0.295940637588501 0.135956943035126 0.0705292001366615 0.0746092721819878 0.0705618038773537 0.096393808722496 0.0395583994686604 0.162427619099617 0 0.087734267115593 0.0887117758393288 0 0.0788745582103729 0.0169661119580269 0 0 0 0.111202664673328 0 0 0.0534370318055153 0.0351861044764519 0.0509272925555706 0.104391276836395 0 0 0 0 0.0948407575488091 0 0.135640874505043 0.293089032173157 0.18190535902977 0.0962014347314835 0 0.312276571989059 0.110969386994839 0.0417982041835785 0.0243836399167776 0 0 0.0405412092804909 2.45603609085083 0.0310356486588717 0 0 0.0332813151180744 0.0787084996700287 0.684952139854431 0.0317646749317646 0.0881543904542923 0.0340050049126148 0.0306461472064257 0 0.012641672976315 0.103869400918484 0 0.0787128806114197 3.72585964202881 0.498204469680786 0.138533726334572 0.110657326877117 0.0955460295081139 0.0170581620186567 0.0214913059026003 0.174318790435791 0.0181781183928251 0 0.0531797185540199 0.265946537256241 0.154786109924316 0.0521742962300777 0.0584974028170109 0.183382645249367 0.0537353865802288 0 0.195353716611862 0.432659983634949 0.0149965276941657 0.225472897291183 0.109903983771801 0.241270542144775 0 0.0360523834824562 0 0.0314509868621826 0.0588085800409317 0.0565373189747334 0.133283689618111 0.123836398124695 0 0 0 0 0.0484876818954945 0.513930201530457 0.0160926915705204 0.0462569333612919 0.130835026502609 0.0934869796037674 0 0 0 1.37454175949097 0.119263283908367 0.326862394809723 0.901695013046265 0 0.346769839525223 0 0.235603228211403 0.337965190410614 0.262934267520905 0.145954504609108 0.72322005033493 0.146385222673416 0.450949907302856 0 0.0319442637264729 0.0321869365870953 0 0.0360682904720306 0.0354486741125584 0.266547411680222 0.124770678579807 0.0311259329319 0 0.0778965055942535 0 0 0.0731394216418266 0.0180764868855476 0.0180761646479368 0.0224760994315147 0.042749896645546 0.090672954916954 0 0 0.115259423851967 0.183183610439301 0 0 0.304026901721954 0.364084959030151 1.07954728603363 0.223499402403831 0.294400125741959 0.308112442493439 0.347546070814133 0 0.0450356043875217 0.0252416264265776 0.0173551328480244 0.815211236476898 0 1.26664865016937 0.0458457805216312 0 0 0.106105364859104 0.0173809491097927 0.064491793513298 0.0720967203378677 0 0 0.0724957585334778 0 0 0 0 0.0366672687232494 0.0355700440704823 0.219446420669556 0.203562036156654 0.0710299238562584 0.148123532533646 0.074315220117569 0.0137932104989886

ENSG00000189013.13 1.32926785945892 2.27300381660461 0.200890094041824 6.67449378967285 1.63723909854889 0.66722172498703 1.28814458847046 1.02884042263031 0.383250594139099 0.149983674287796 0.150796160101891 1.38268792629242 0.250001966953278 0.218081116676331 0.301249712705612 1.70357728004456 0.118191927671432 1.67904448509216 1.57275450229645 0.171505108475685 0.798661112785339 5.1924262046814 0.0511565245687962 0.8942711353302 1.17585611343384 0.933361053466797 1.59512782096863 0.45796725153923 0.25416961312294 0.126248329877853 0.476596474647522 0.123577162623405 0.0542035847902298 0.0452115312218666 0.0334582887589931 0.634391963481903 1.01284217834473 1.44644808769226 0.903300821781158 1.53676044940948 0.0365765281021595 1.13388812541962 0.371834516525269 1.2287266254425 0.803436875343323 0.447409510612488 0.700418472290039 1.54049170017242 0.40293151140213 0 10.7808446884155 0.300003439188004 0 2.06520199775696 0.595615804195404 0.039183434098959 1.1326699256897 0.379038393497467 0.72834837436676 0.185954004526138 3.08573365211487 1.76150071620941 0.574224472045898 0.0925921574234962 0.408284842967987 0.283979117870331 0.497088581323624 0.419374167919159 0 0.314010053873062 1.24991619586945 2.24586415290833 0.104674406349659 1.13926231861115 0.843809187412262 1.32739078998566 0.815726518630981 0.570597648620605 21.6397647857666 0.202901303768158 2.27668380737305 2.54942870140076 0.277079969644547 4.1403694152832 0.0954619348049164 2.17426633834839 1.38739466667175 0.211233392357826 2.2416615486145 0.131781235337257 0 0.117168061435223 1.46178197860718 0.351512402296066 0 0.0378149598836899 2.47787141799927 0.221372425556183 0.0698306486010551 0.15876130759716 0.574958086013794 0.0252174772322178 0.426451057195663 0.82781320810318 0.0548106208443642 0.987893521785736 0.305658757686615 0.0704429224133492 0.14077191054821 0.0671647861599922 3.13476061820984 2.38694524765015 0.90507835149765 0.0622447803616524 0.356835782527924 0.164844885468483 0.331152975559235 0.21733121573925 0.315063953399658 0.369691520929337 0.60223925113678 2.92835211753845 0.368827372789383 1.3527295589447 1.1628395318985 0.271916449069977 0.11692138761282 2.96769547462463 0.0707795768976212 0.0654766261577606 0.151543185114861 0.956021070480347 1.13533782958984 0.413142174482346 0.242468103766441 0 0.0876959189772606 4.65311622619629 5.49775266647339 0.946641683578491 1.5616227388382 0.816117823123932 0.1773791462183 0.319253087043762 2.65424132347107 0.162021428346634 0 0.868982493877411 1.22469615936279 0 1.86011648178101 0.130346745252609 0.778327763080597 0.186255663633347 1.7514762878418 1.50902831554413 2.45035433769226 0.133663952350616 0.855956971645355 0.489786744117737 1.28002536296844 0.221528381109238 0.14281539618969 0.162400022149086 0.747526526451111 1.01920092105865 0.377937525510788 0.593978345394135 0.998633682727814 0.303723722696304 0.190475106239319 0.778911888599396 0.0633511319756508 0.180070981383324 1.1706120967865 0.19124536216259 0.446645051240921 0.111060164868832 1.18043577671051 0.184081554412842 0.799555778503418 0.161733716726303 5.08095788955688 0.590551555156708 1.35955035686493 1.36680293083191 0.190729767084122 9.41944980621338 0 3.14989471435547 0.794907569885254 9.1007137298584 0.346904397010803 13.1786022186279 0.838755309581757 11.9090766906738 0.125055521726608 0.569437980651855 0.0956273078918457 0.344562768936157 0.803691029548645 0.877649366855621 0.544439315795898 1.66812026500702 0.27742525935173 0.589828729629517 0.61714768409729 0.0416647829115391 0.198517501354218 0.543242633342743 0.510199368000031 0.61759877204895 0.233717516064644 0.0635049194097519 0.615746557712555 4.04107570648193 0.562810122966766 0.642066538333893 2.5851309299469 0 1.00588572025299 3.08614921569824 1.48733139038086 3.8556227684021 0.184448957443237 2.59483075141907 1.6528080701828 1.8930242061615 0.132744967937469 0.53520268201828 2.51225900650024 0.0257810298353434 4.22440242767334 0.255558103322983 3.01056814193726 0.238363489508629 0.188613057136536 0 1.07706594467163 0.929497718811035 0.59876549243927 0.642597556114197 0.760073959827423 0.653069198131561 0.43076953291893 0.121853463351727 0.223339661955833 0.206693410873413 0.0221480000764132 0.108938381075859 0.158517777919769 4.67248582839966 4.31987380981445 1.74099540710449 6.16104602813721 0.257588744163513 0.614693999290466

ENSG00000221957.7 0.486640095710754 0.26345694065094 0 0.775950372219086 0.339744865894318 0.137568205595016 0.476231187582016 0 0.13696600496769 0.214404508471489 0.0970046892762184 3.76785373687744 0 0.233813345432281 0.258385360240936 1.06915438175201 0.0633591040968895 0.480045229196548 0.526941239833832 0 0.677885115146637 1.04790663719177 0.0822703763842583 0 0.0504272617399693 0 0.0397720746695995 0.261869162321091 0.31792277097702 0.338389486074448 0.360690236091614 0.0662459582090378 0.406796544790268 0 0 0 0.41088405251503 0.258465617895126 0.528253257274628 0.235374495387077 0 0.233785793185234 0.265772253274918 0 0.430698096752167 0.0399737767875195 0 0.0750737190246582 0.0762350633740425 0 15.4660730361938 0.385974586009979 0 1.06891655921936 0.121634870767593 0.0420101061463356 0 0 0.164398074150085 0 0.163374647498131 0.659863173961639 0.123129725456238 0 0.375204175710678 0 0.242249190807343 0.0749379321932793 0 0.201997518539429 0.521143913269043 0 0.157115772366524 0 0 1.06736099720001 0.104115769267082 0.480668723583221 0.264020949602127 0.398820519447327 0.452751666307449 0.224658310413361 0.148534163832664 0.39333376288414 0.153522729873657 0.252012610435486 0.323365300893784 0.226471617817879 0.111784815788269 0.070643924176693 0 0.0628102421760559 0 0.188435107469559 0 0.0810858160257339 0.295180380344391 0 0 0.085107110440731 0.112079106271267 0.108146592974663 0.249389991164207 0.126790151000023 0 0 0 0 0.113195329904556 2.88040018081665 0.809105634689331 0.0965712368488312 0.919297993183136 0.0667350813746452 0.0765155330300331 0 0 0 0.0844481065869331 0 0 27.9475975036621 0.271861106157303 0.913160979747772 0.460746556520462 0.0795088112354279 0 5.09085273742676 0.101180769503117 0.105300083756447 0 0.146426826715469 0.139113172888756 0.120803453028202 0.070898063480854 0 0 1.52289354801178 1.07685375213623 0.154446139931679 0 0.11412937939167 0.0543357580900192 0.0684567540884018 0.798188745975494 0 0.0584879368543625 0.169394597411156 0.50827544927597 0.862826645374298 0.270062029361725 0.186333149671555 0.333790302276611 0 0.128033638000488 0.560038328170776 0.775216042995453 0 0.638403594493866 0 0.219578489661217 0.0339298956096172 0.15311798453331 0.0217644330114126 0.300544708967209 0.187324345111847 0.225112050771713 0.141517221927643 0.619864106178284 0.0651268288493156 0 0 0.0679212361574173 0 0.0545677952468395 0.0256302133202553 0.221014961600304 0.208375915884972 0.223339691758156 0.0986805334687233 0 0.0346802175045013 0.152733489871025 0 0.832929968833923 0.849933862686157 0.0408977754414082 1.10457408428192 0 1.68856298923492 0.358842790126801 1.21441948413849 0.60438597202301 3.76269364356995 0.432978272438049 0.053200788795948 0.134076923131943 0.0508764050900936 0 0 0.229778334498405 0.470481157302856 0.265324890613556 0.745190560817719 0.0495730824768543 0.237141937017441 0.330834090709686 0 0.0532096028327942 0 0.0287897270172834 0.259102940559387 0.0715936422348022 0 0.103150956332684 0.0470934323966503 0 0.413030833005905 0.145874753594398 0 0.0898708030581474 0 0 1.20720553398132 0.514162957668304 0.0312586538493633 1.74477827548981 0.492020756006241 0 0.215179443359375 0.0804027318954468 0.138204291462898 1.57010459899902 0.039141982793808 2.13261938095093 0.0730168223381042 0.134812980890274 0 0.0281649921089411 0.138409867882729 0.359497636556625 0.0765504539012909 0 0.175045281648636 0.577306151390076 0.174191847443581 0 0.0886416435241699 0.047491479665041 0.233594223856926 0 0 0 0.395943224430084 0 0.315623998641968 0.241647094488144

ENSG00000167633.15 0.216338858008385 0.298127204179764 0.243725880980492 0.390250444412231 0.192227199673653 0.0155671834945679 0.24250590801239 0.240042358636856 0.0387475974857807 0.12130980193615 0.0365900881588459 0.768862724304199 0.0674021989107132 0.220485478639603 0 0.0756157785654068 0.0179242491722107 0 0.119257062673569 0 0.242239907383919 0.259395629167557 0.0155161581933498 0 0 0 0.247532904148102 0.0926032811403275 0 0.191460356116295 0.153058558702469 0 0 0.0548519939184189 0.263851881027222 0.179588124155998 0.0830277353525162 0 0.348699182271957 0 0 0.0529102794826031 0.225560411810875 0 0.310148924589157 0 0.0354070290923119 0.552196025848389 0 0 10.9770078659058 0.236582607030869 0 0.647991061210632 0.0172052141278982 0.023769261315465 0.0624631457030773 0 0.104643255472183 0.112802498042583 0.161765024065971 0.0772447809576988 0.0870833024382591 0 0.0707633569836617 0.20671920478344 0 0 0 0.0380966477096081 0.0421232245862484 0.162187486886978 0.0380982495844364 0.164546117186546 0.107761554419994 0.201303824782372 0 0.0988951772451401 1.10169768333435 0.471818059682846 0.200477987527847 0.0423704609274864 0.18488897383213 0.476888060569763 0.0579086467623711 0.106941305100918 0.0365919359028339 0.0427124500274658 0.0948715582489967 0.0799404829740524 0 0.106613874435425 0.0305772069841623 0.0533081777393818 0 0.206452131271362 0.150311410427094 0.0191839784383774 0 0.0963069945573807 0 0.0611892081797123 0.0235174223780632 0.0358688570559025 0 0.0374544449150562 0.0132440868765116 0 0.0213485918939114 0 0.510612964630127 0.0819598287343979 0.144482687115669 0 0.0216461960226297 0.0499986931681633 0.0502206012606621 0.021972730755806 0.0238903183490038 0 0.146130949258804 2.21319794654846 0.181785687804222 0.12156830728054 0.015334696508944 0.0299906563013792 0 0.822970867156982 0.0143119879066944 0.0397191010415554 0.0306427925825119 0.0828481167554855 0.0590325407683849 0.0341752208769321 0.0401140376925468 0.0313381142914295 0.0354651100933552 0.207984775304794 0.448944985866547 0.124836325645447 0 0.0645742639899254 0.0153715526685119 0.0193663723766804 0.255260199308395 0.0327615477144718 0.0330924019217491 0.0479216277599335 0.551198065280914 0.383574873209 0.0705234184861183 0.158140584826469 0.259679913520813 0 0.0845148265361786 0.105622984468937 0.292410850524902 0 0.293481439352036 0.272352665662766 0.186355844140053 0.0383949838578701 0.314048111438751 0.0492571480572224 0.0283413045108318 0.0706585869193077 0.0509472414851189 0 0.382601827383041 0.0184243377298117 0.0770300403237343 0.181730642914772 0.0384297519922256 0.0873870179057121 0.030874390155077 0.0725077092647552 0.0416833236813545 0.0842134580016136 0.084243543446064 0.0558333247900009 0.0970045179128647 0.0392440445721149 1.32505178451538 0.0358237437903881 0.323998630046844 0.978365480899811 0.0462798215448856 0.624966621398926 0 0.601539850234985 0.0507582016289234 0.331711679697037 0.078914038836956 0.977568626403809 0.0753780156373978 0.0602018795907497 0.0379302799701691 0 0 0.0368853472173214 0.054170124232769 0.0958311557769775 0.390313327312469 0.056217048317194 0 0.536698460578918 0.187185525894165 0.202195897698402 0 0.0988617464900017 0.114024348556995 0.114022314548492 0.0405075885355473 0.0385230407118797 0.175088033080101 0.159872427582741 0.195090904831886 0.0778974518179893 0.110047675669193 0 0 0 0.533140361309052 0.641639292240143 0.246156916022301 0.123802751302719 0.802094578742981 0.13919235765934 0 0.040582749992609 0 0.125113293528557 1.19587361812592 0.0664394497871399 1.98931431770325 0.0206564124673605 0 0 0.0318714343011379 0.0939745455980301 0.029057614505291 0.0649682283401489 0 0.0742803066968918 0 0.0246393829584122 0.0338703021407127 0.0250766668468714 0 0.13216732442379 0.0320530906319618 0.0439441911876202 0.0786150097846985 0 0.0242687202990055 0.133934736251831 0.0994353666901588

ENSG00000240403.4 0.357341021299362 0.169274941086769 0 0.33237299323082 0.339563876390457 0.0471411161124706 0 0.218071520328522 0 0.137758046388626 0 1.16414904594421 0.0255137253552675 0.0667682215571404 0.11067757755518 0.381637424230576 0 0.411248117685318 0.0902846455574036 0.0787626057863235 0.275084942579269 0.112215898931026 0.0939731895923615 0 0.086400680243969 0.081645704805851 0.204433143138885 0 0.0389085747301579 0 0.180249035358429 0.198632001876831 0.0995705723762512 0.0415262021124363 0.0614619962871075 0.621526122093201 0.301713168621063 0.184519961476326 0.377122730016708 0.151231542229652 0 0.721011817455292 0.0379472374916077 0 0.100629039108753 0 0.268052160739899 0.353730410337448 0.108849212527275 0.0471808239817619 9.76161003112793 0.192884489893913 0 0.457863032817841 0.026050727814436 0 0 0.0497345477342606 0.0176046956330538 0.284660547971725 1.67952966690063 0.0779718235135078 0.0263708829879761 0 0.0535720400512218 0.104332447052002 0.0415063053369522 0.0641982927918434 0 0.0576828271150589 0.22322840988636 1.05595552921295 0.0961420834064484 0.199313834309578 0.0815818756818771 0.406397074460983 0.0178389083594084 0.112304255366325 9.66932773590088 0.279543310403824 0.118046216666698 4.04169511795044 0.0508988685905933 0.481376588344574 0.0438402704894543 0.215895712375641 0.110809035599232 0.129343405365944 0.119705639779568 0.0605196729302406 0.0252610389143229 0 0.0231487527489662 0.242144644260406 0.0605680495500565 0 0.278164625167847 0.0290468111634254 0.128277078270912 0.145820170640945 0 0.0231619235128164 0.106824479997158 0 0.176199942827225 0.453683614730835 0.0200531110167503 0.0647009015083313 0 0.0308449901640415 0.239936396479607 0.951409339904785 0.78754997253418 0.0571710169315338 0.229424297809601 0.0757039412856102 0 0.099807932972908 0.144691064953804 0.084889218211174 0.110629767179489 2.20463371276855 0.127036169171333 0.138051599264145 0.0928741693496704 0.0681141018867493 0.0536953881382942 0.415358394384384 0.0650101229548454 0.0300697106868029 0 0.16725580394268 0.0297940839082003 0 0.10122898966074 0 0.053698368370533 1.37212336063385 1.01963365077972 0.189016968011856 0.0754910558462143 0.0814775601029396 0.0930973961949348 0.234583824872971 0.386494129896164 0.0248024296015501 0.0501058101654053 0.14511801302433 0 0.89756566286087 0.088983990252018 0.0399072617292404 0.321697741746902 0.0488781109452248 0.109684646129608 0.479777097702026 0.442744612693787 0 0.102545917034149 0.0374885573983192 0.0940549224615097 0 0.0491902753710747 0.0372905768454075 0.128736257553101 0.0534927286207676 0.115710221230984 0.121235854923725 0.241376832127571 0.0557932518422604 0.0291581489145756 0.0550323389470577 0 0 0.0467474795877934 0.0439140982925892 0.189340457320213 0.0765054821968079 0.0637773498892784 0 0 0.0891301855444908 2.39881920814514 0.325448334217072 0.31218233704567 3.11336898803711 0.070073127746582 0.405545741319656 0 1.232262134552 0.115280874073505 0.430500596761703 0.079656794667244 1.24990832805634 0.142664030194283 0.0455763787031174 0.0574309267103672 0.174340456724167 0.0658743232488632 0.111697562038898 0.0984239652752876 0.370810359716415 0.272760212421417 0.383036643266678 0.0849371552467346 0.0677187442779541 0.106282882392406 0.0765371173620224 0.136751785874367 0.0249480623751879 0 0.123316615819931 0.0306666418910027 0 0.282777577638626 0.161377176642418 0.0923095941543579 0.0786306336522102 0.249937802553177 0 0.23097325861454 0.276545166969299 0.124190397560596 0.344732135534286 0.20329675078392 0.241009697318077 0.81742912530899 0.0526884235441685 0.243849039077759 0.184341251850128 0.0344399586319923 0.0473590791225433 0.672543466091156 0 0.888803780078888 0.0312762521207333 0.0577462129294872 0 0.0723856911063194 0.0237147640436888 0.21998330950737 0.131159439682961 0.065448559820652 0 0.0989140346646309 0.0746138840913773 0.102567292749882 0 0.22376911342144 0.100058473646641 0 0.299415171146393 0 0.145371049642563 0.0183728579431772 0 0.0188196115195751

ENSG00000134545.12 1.4708970785141 0.94211733341217 0.513468503952026 3.51986622810364 1.34991276264191 0.705116152763367 0.936646819114685 4.34909057617188 1.5101797580719 0.447245597839355 0.462516039609909 0.773085534572601 0.10649947822094 0.139352053403854 0.269494563341141 1.59303224086761 0.0566427558660507 0.834474503993988 1.09919393062592 2.02742171287537 1.44595742225647 3.12274622917175 0.931625127792358 0.441561102867126 0.661198973655701 0.738412439823151 0.829641401767731 0.214600697159767 0.297755897045135 0.282350778579712 0.841967046260834 0.177670791745186 0.0692712888121605 0.14444899559021 0.213795721530914 6.02652025222778 0.979542672634125 2.18230080604553 0.73462051153183 0.981977462768555 0.0701163113117218 1.44909060001373 0.475198447704315 0.594164729118347 0.583397269248962 0.214418292045593 0.261077642440796 0.88368833065033 0.302905797958374 0.0984711647033691 0.830573260784149 0.0575099289417267 0.0436041578650475 0.955606698989868 0.398717105388641 0.0751136913895607 0.592172920703888 0.41520419716835 0.808341383934021 0.15843078494072 3.26192164421082 1.55954480171204 0.440309673547745 0.0394437573850155 8.31123161315918 0.508088946342468 0.346511483192444 0.357302248477936 0.0384138002991676 0.401299774646759 1.50862967967987 0.905472755432129 0.334430485963821 0.381322532892227 0.510808944702148 1.41365325450897 0.595705807209015 0.234390377998352 2.51769137382507 0.432174295186996 1.80674564838409 0.379371106624603 0.478040009737015 3.04753518104553 0.457495480775833 1.53953731060028 0.501084387302399 0.584897816181183 2.36513113975525 0.631553530693054 0.527223527431488 0.299477756023407 0.853543758392334 0.50538045167923 3.2024290561676 0.28996142745018 1.81204426288605 0.141455188393593 0.66931825876236 0.963748157024384 0.367393523454666 0.128910109400749 0.767951130867004 0.377832770347595 0.087558850646019 0.591802418231964 0.348774015903473 0.225062265992165 0.314832389354706 0.107294373214245 1.37248528003693 2.33102488517761 0.456582397222519 0.0795478224754333 2.94139552116394 0.105334542691708 0.83319091796875 1.78220176696777 0.150992467999458 0.413402259349823 0.461791127920151 0.306752979755402 0.353516519069672 0.32014188170433 0.258450418710709 0.426483124494553 0.522982716560364 0.433447599411011 0.301517158746719 0.230114638805389 0.355061173439026 1.39631927013397 0.456010729074478 0.407991468906403 0.169020146131516 0 0.504332780838013 3.92789030075073 2.66854357719421 0.394497632980347 0.78778749704361 0.283419907093048 0.0485759228467941 1.02000057697296 1.15827023983002 1.12157917022705 0.453162312507629 0.740363657474518 0.959280431270599 0.257121086120605 1.54765522480011 0.360925495624542 2.08884930610657 0.272036224603653 1.34810209274292 0.407954752445221 1.9251104593277 0.427050918340683 0.309145390987396 0.391212165355682 0.261736243963242 0.242665410041809 0.148294016718864 0.363203018903732 0.507517337799072 0.707083523273468 0.429331541061401 2.15076804161072 0.18471859395504 0.329930871725082 1.54168570041656 0.229716166853905 7.8937668800354 1.08160054683685 0.552878201007843 0.977633953094482 0.153678297996521 0.674182116985321 0.488069236278534 0.676353096961975 1.43054687976837 0.206693068146706 1.75988852977753 0.981129825115204 0.558476746082306 0.157207682728767 0.536248981952667 10.2040042877197 0 1.41638469696045 1.09607994556427 2.39599585533142 0.249377712607384 4.3935751914978 0.416856080293655 4.64515018463135 0.49943408370018 0.727732479572296 2.16923022270203 0.64756715297699 1.92867314815521 0.224324315786362 0.284639149904251 1.83574295043945 0.886362314224243 0.895127356052399 0.764057636260986 0.346104949712753 0.190276578068733 1.16287755966187 0.188744515180588 0.549065172672272 0.661378800868988 0.324633002281189 0.786914110183716 2.30153870582581 0.680730283260345 0.574385404586792 0.289803087711334 0 0.535627365112305 1.44294452667236 1.33918941020966 5.4070839881897 0.0235722754150629 1.91889667510986 1.05613076686859 1.832768201828 0.0848229601979256 0.512985229492188 0.28751876950264 0.131790950894356 1.96153390407562 2.23953652381897 1.78631925582886 0.348142445087433 0.723133206367493 0.171006977558136 1.25896894931793 0.346465826034546 0.673387050628662 1.09497225284576 0.37943834066391 0.208652958273888 0.791367292404175 1.27176833152771 0.107034161686897 0.132075399160385 1.24541044235229 0.139221459627151 0.94538801908493 1.94416403770447 1.02133464813232 1.3484628200531 12.8586978912354 0.658388376235962 0.248763471841812

ENSG00000205809.8 0.298300683498383 1.05980336666107 0.154029130935669 1.10983169078827 0.36444941163063 3.24657297134399 0.306516230106354 6.73553419113159 1.02847862243652 0.0574987456202507 0.138744533061981 0.265038251876831 0.0638949498534203 0.0836050361394882 0.277173846960068 1.24247300624847 0.169915661215782 0.472039103507996 2.31755685806274 0.0986240208148956 0.574087619781494 2.10769724845886 0.500098526477814 0.0467500612139702 5.57169103622437 0.460053682327271 0.3626449406147 0.316024899482727 0.0487200804054737 0.217797324061394 0.128972619771957 0.319783717393875 0.187018632888794 0.051997784525156 0.461764544248581 0.68097323179245 0.629659295082092 1.34008991718292 0.283332645893097 0.0631224438548088 0 0.601885378360748 0.0950326398015022 0.611095547676086 0.420014888048172 0.128641426563263 0.469904899597168 0 0.136297509074211 0 0.4983069896698 0.20702038705349 0 1.1466429233551 0.0326198935508728 0.180259495973587 0.355277389287949 0 0.022044038400054 0.142577141523361 0.920085906982422 0.53698605298996 0.231145426630974 0.10649024695158 2.54908585548401 0.0653208866715431 0.155918627977371 0.924451053142548 0 0.288914382457733 1.03821516036987 2.02947521209717 0.120386056602001 0.0623936057090759 2.34954690933228 0.190829038619995 1.34023880958557 0.187498420476913 0.0708048194646835 0.661177635192871 0.675719738006592 4.33789825439453 0 1.44663465023041 0.219581559300423 0.540675401687622 3.12190985679626 10.7703189849854 2.27835178375244 0.45468482375145 0.0632621347904205 0.33688747882843 0.318847447633743 0.235826745629311 3.9437518119812 0.217454940080643 0.316644459962845 0.0363714918494225 0.803122341632843 0.456478416919708 0.0601144842803478 0 0.222937017679214 0.20401456952095 0.0630377009510994 0.994154334068298 0.100439473986626 0.081016406416893 0.769033551216125 0.231738686561584 0.300440698862076 1.29491782188416 0.383500784635544 0.0715877339243889 1.19015085697174 0.284382194280624 0.0238036923110485 0.999810457229614 0 0.0531477779150009 0.207790583372116 0.220845773816109 0.159070640802383 0.460970163345337 0.02907351590693 0.255870908498764 0.0672356560826302 0.520098507404327 0.135672673583031 0.0376523323357105 0.116193220019341 1.36131036281586 0.74614405632019 0.23757740855217 0.0253511425107718 0.237659558653831 0.134478762745857 2.92926931381226 1.51994383335114 0.449693918228149 1.22885739803314 0.0816188752651215 0 0.220303803682327 3.5738263130188 2.91934013366699 0.0627409070730209 0 0.863288402557373 0.330559730529785 0.779960513114929 0.19988240301609 0.671366035938263 0 0.869843006134033 0.333756417036057 1.61697292327881 0 0.513618886470795 0.046941976994276 0.706635475158691 0.0727942287921906 0.0615944936871529 0.0700411051511765 0.0537331365048885 0.736800789833069 0.120740547776222 3.1879608631134 0.302244365215302 0.06986253708601 0.109532713890076 0.137819468975067 1.38434267044067 0.538459122180939 1.40485692024231 0.082481749355793 0.118543036282063 0 0.159719929099083 0.105856105685234 0 0.0744039863348007 0.163839533925056 0.203758016228676 1.17271423339844 0.188635230064392 0.131614997982979 26.7447357177734 0 0.670869290828705 0.529287159442902 1.52733409404755 0 7.94903707504272 0.714557468891144 4.47993898391724 0.143826350569725 0.545758903026581 0.274952441453934 0.65269923210144 0.390270620584488 0.46431690454483 0.113847225904465 2.0783805847168 0.850844740867615 0.254385769367218 1.50828564167023 0.095837339758873 0.0570787452161312 0.406109184026718 0.0617663525044918 0.154413148760796 0.383997917175293 0.0730370134115219 0.641779243946075 2.37433862686157 0.277409136295319 0.787670195102692 0.99105304479599 0 0.0964057743549347 0.779132306575775 0.855289876461029 4.94449901580811 0.0848539024591446 1.20713865756989 1.16978132724762 0.19792428612709 0.152669996023178 0.192355200648308 0.474370777606964 0.0296507645398378 0.48584857583046 0.503858387470245 0.803784072399139 0.0391631238162518 0.0723079591989517 0.0615579523146152 0.392769187688828 0.534507691860199 0 0.615876257419586 0.546350717544556 0.234717175364494 0.123857006430626 0.467145621776581 0 0.142630845308304 1.24814832210541 0.0626450181007385 0.364622592926025 2.41613912582397 24.7420692443848 0.121352657675743 86.5022125244141 0.169287323951721 0.259218484163284

ENSG00000205810.7 0.146064698696136 0 0.201123401522636 0.422672182321548 0.158626511693001 0.462459295988083 0 2.49585509300232 0.0959239304065704 0.225236818194389 0.135874167084694 0.0865183621644974 0 0.109167195856571 0.0904798805713654 0.436788648366928 0.0887468978762627 0.112066306173801 2.14044618606567 0.128778219223022 0.249871477484703 0.733899772167206 0.0768239125609398 0.122087702155113 1.13013291358948 0.200238212943077 0.139271557331085 0.0458499118685722 0 0 0.0421014726161957 0.0463952571153641 0 0.0678960680961609 0 0.190538614988327 0.0411088541150093 0.181015983223915 0 0.0824220702052116 0 0.720418632030487 0.0620443932712078 0.249355480074883 0.329060435295105 0 0.350615948438644 0.184022173285484 0.0711881443858147 0 0.15309739112854 0.0450527854263783 0.0512386821210384 0.427779465913773 0.127780184149742 0 0.154634386301041 0 0 0.0930849835276604 0.171628758311272 0.159356608986855 0.172467425465584 0.0926996916532516 1.31386840343475 0.170585364103317 0 0.157447978854179 0.0902791246771812 0.471562147140503 0.364982306957245 0.562118113040924 0.031438797712326 0 0.733632802963257 0.0830582827329636 0.0875007808208466 0 0.138679996132851 0.152352720499039 0.248152643442154 2.04540801048279 0.0832205340266228 0.157411828637123 0 0.0882483124732971 0.181174695491791 1.69182980060577 0.0782883167266846 0.0989507213234901 0 0 0.0756972283124924 0.13197024166584 0.891268312931061 0 0.165383294224739 0.0949840918183327 0.314602881669998 0 0.0784944370388985 0 0.116439923644066 0.266391843557358 0 0.278167754411697 0.0327871851623058 0 0.634209394454956 0 0.697422623634338 0.879235684871674 0.071536548435688 0 0.4822878241539 0 0.155408248305321 0.0543959066271782 0 0.0693976655602455 0 0 0.0346177257597446 0 0.0379627235233784 0.037122581154108 0 0.0424448922276497 0.0354308970272541 0.147493496537209 0.0758595988154411 0.341832667589188 0 0.0846045091748238 0.0662044659256935 0 0.0438988842070103 0.551666915416718 0.238159835338593 0.216332226991653 0.617145955562592 0.0266434457153082 0 0.19177433848381 0.972191751003265 1.50043892860413 0 0.0395450815558434 0.29664158821106 0 0.32007884979248 0 0.175327122211456 0.0399582795798779 1.13579666614532 0.261481285095215 0.482596784830093 0 0.111776232719421 0.122588895261288 0.307562917470932 0 0 0.0304853729903698 0 0.218653872609138 0.0945941135287285 1.28844773769379 0.0394655354321003 0 0 0 2.18815279006958 0 0.152865886688232 0.0718003213405609 0.0515958219766617 0.083391860127449 0 0 0 0 0.784422218799591 0.443428099155426 0.510423541069031 0.164206862449646 0 4.97305727005005 0 0.350394815206528 0 0.234625339508057 0.260480493307114 0.69913375377655 0.233258172869682 0.260813593864441 0.187801137566566 0.142524838447571 0 0.213065356016159 0.0268208142369986 0.0527201257646084 0.0743279606103897 2.15715789794922 0.208310574293137 0.110721327364445 0.926796615123749 0 0.0745305195450783 0.0407905168831348 0 0 0.0501404963433743 0.0953680202364922 0.202276512980461 1.31927251815796 0.0603711232542992 0.0642812252044678 0.340543866157532 0 0.377645283937454 0.113039039075375 0.609160423278809 2.45953321456909 0.11079790443182 0.481622844934464 0.343674212694168 0.258439481258392 0 0 0.168929785490036 0 0.380637794733047 0.76756477355957 0.242201596498489 0.153411641716957 0 0 0 0.0775481089949608 0 0.536119937896729 0.142679393291473 0 0.323452353477478 0.0609975010156631 0 0 0.432387620210648 0.0817986726760864 0 0.380760371685028 3.89240169525146 0.0792280584573746 9.642822265625 0.110523387789726 0.03077039308846

ENSG00000134539.15 2.34765458106995 1.26575577259064 0.57709151506424 1.15105557441711 1.63227450847626 0.932934403419495 0.466402292251587 5.91104459762573 0.784587621688843 1.06475424766541 0.250950306653976 0.713363945484161 0.148587703704834 0.48245832324028 0.459552943706512 1.3006284236908 0.204886823892593 1.87389755249023 1.05160558223724 1.07030069828033 1.90861392021179 1.20418345928192 0.342052668333054 0.346284598112106 0.50318318605423 0.607571840286255 0.609990835189819 0.38711616396904 0.415427982807159 0.243864446878433 0.827573120594025 0.486590385437012 0.198663428425789 0.26423442363739 0.364573091268539 1.27358567714691 1.31242227554321 1.8666433095932 1.21203219890594 1.84848213195801 0.105072408914566 1.92672574520111 0.560681104660034 0.453963369131088 4.90181112289429 0.306542187929153 0.664909183979034 0.513282656669617 0.36626473069191 0.162828579545021 0.595816671848297 0.243684709072113 0.111533336341381 1.77062439918518 0.387716382741928 0.294987976551056 0.300899088382721 0.22528001666069 0.717687726020813 0.4482242166996 2.74344348907471 1.01330435276031 0.338443994522095 0.128407508134842 0.60665625333786 0.506345689296722 0.465546071529388 0.768531382083893 0.172694355249405 0.423029512166977 0.570919275283813 2.16113805770874 0.22811371088028 0.838334321975708 0.303548395633698 0.646483957767487 0.392476499080658 0.282610446214676 0.164656609296799 1.19923639297485 1.33676946163177 5.18572902679443 0.373277842998505 1.24598097801208 0.236405968666077 1.23987758159637 0.358518540859222 0.285965323448181 1.802250623703 0.659224212169647 0.316027134656906 0.760219037532806 0.189738899469376 0.220526680350304 2.47569966316223 0.35211107134819 1.22180664539337 0.20988841354847 0.581050038337708 1.1087201833725 0.217460811138153 0.177356943488121 0.614447414875031 0.638438403606415 0.214461833238602 0.807332158088684 0.333056330680847 0.251204878091812 0.373016476631165 0.292740285396576 1.93213796615601 2.01647663116455 0.452993512153625 0.135648131370544 0.827127516269684 0.326583087444305 0.524851977825165 0.139934122562408 0.128739207983017 0.196836709976196 1.08574688434601 0.347139894962311 1.05723690986633 0.570733308792114 0.385630041360855 0.320776283740997 0.13319256901741 0.643940985202789 0.247731253504753 0.334027081727982 0.185142040252686 1.01916575431824 0.285979717969894 0.327399283647537 0.294770389795303 0.28657341003418 0.448826014995575 4.00036096572876 3.9902036190033 1.24350011348724 0.781595230102539 0.434090316295624 0.165667250752449 0.56924045085907 2.46249842643738 0.403911650180817 0.324230998754501 0.503434062004089 1.94104909896851 0.398593813180923 0.884826242923737 0.628376483917236 1.07168090343475 0.218764945864677 0.798481523990631 1.10385847091675 3.35837864875793 0.36852565407753 1.15386939048767 0.456869721412659 0.689772188663483 0.210036277770996 0.166226804256439 0.255380660295486 0.684946238994598 0.752870798110962 0.528286397457123 4.26903200149536 0.458166629076004 0.111318819224834 0.525160014629364 0.635063171386719 1.53120160102844 0.217616468667984 0.423499286174774 0.402566939592361 0.248444974422455 0.789347767829895 0.639682650566101 0.227933675050735 4.60956478118896 0.253131300210953 2.05086231231689 0.754632890224457 1.26016306877136 0.58760279417038 0.12469544261694 5.43075323104858 0.108381368219852 2.71574091911316 0.729396820068359 3.94259262084961 0.635725617408752 3.90213632583618 0.775461494922638 3.19498181343079 0.445957571268082 0.338443279266357 0.208397656679153 0.610353231430054 0.46351745724678 0.431212067604065 0.730518579483032 7.67907571792603 0.343513786792755 0.61348420381546 0.657182037830353 0.507647693157196 0.324467271566391 0.809875965118408 0.210136547684669 0.444204807281494 0.294355005025864 0.144684970378876 0.77196079492569 2.01890230178833 0.551533401012421 0.678417325019836 0.822142243385315 0.0895900130271912 0.506506979465485 1.64783418178558 2.08942985534668 5.62077903747559 0.957404971122742 0.681583762168884 1.82110738754272 1.66493773460388 0.0986205786466599 0.924465477466583 0.757718622684479 0.286026656627655 1.12984085083008 0.379724889993668 2.13546752929688 0.371041595935822 0.604102194309235 0.281004041433334 0.408551007509232 0.179032564163208 0.721237897872925 0.937131762504578 1.22348153591156 0.347716957330704 0.837418913841248 0.647784769535065 0.304198265075684 0.405396491289139 0.331283360719681 0.474812090396881 0.32975047826767 2.88112497329712 1.00988328456879 0.517376959323883 1.50989544391632 0.6233229637146 0.237471625208855

ENSG00000226979.7 5.30945634841919 1.46334636211395 1.32924389839172 1.59627497196198 3.93141722679138 2.13950634002686 1.63119459152222 0.418929398059845 1.20454382896423 4.16811275482178 1.64634323120117 1.42952048778534 0.88224321603775 0.937945485115051 0.65778923034668 3.71157264709473 0.175961017608643 7.99909543991089 3.51221346855164 2.21288013458252 2.60925078392029 2.00079584121704 3.02103281021118 0.766544878482819 0.466822266578674 0.882262468338013 1.1229602098465 1.54543519020081 1.59769320487976 0.877124309539795 1.58604025840759 4.99808073043823 4.14243936538696 1.61543369293213 0.431702435016632 3.06426787376404 4.83612442016602 3.42954254150391 2.03759169578552 1.41631197929382 0.726055681705475 14.933219909668 7.62706661224365 1.48321282863617 2.57350397109985 1.55421352386475 0.811039090156555 0.469113528728485 3.08170461654663 0.713769495487213 0.252958834171295 0.833722829818726 0.440233796834946 2.75655484199524 1.32306814193726 0.350011646747589 1.3796911239624 5.37430906295776 1.29360556602478 5.2292652130127 2.83577752113342 1.36916410923004 1.36782443523407 0.398229748010635 1.56302678585052 1.69112265110016 1.16614317893982 0.659039556980133 1.43199217319489 1.74529588222504 0.379060536623001 6.20951223373413 0.706458926200867 0.807668507099152 2.46840333938599 2.08597278594971 0.597577750682831 1.77988564968109 0.916549444198608 1.98026371002197 2.38720512390137 7.69503116607666 1.15502631664276 2.54885721206665 0.852727353572845 3.14950442314148 3.17311263084412 1.25791490077972 3.49255132675171 1.89652693271637 0.95539665222168 1.22105717658997 0.550319314002991 1.22108554840088 0.392698556184769 2.62723636627197 6.20296859741211 2.63658785820007 3.11886119842529 8.03622817993164 0.311266362667084 0.700804889202118 5.73324203491211 2.58222699165344 0.190401434898376 1.96099984645844 1.47351694107056 0 1.71154999732971 0.666621148586273 6.59710693359375 1.92208445072174 3.45138096809387 0.926684021949768 8.92496204376221 1.7179172039032 0.677891373634338 0.287606209516525 0.0390882454812527 0.458655834197998 14.1662473678589 0.142939478158951 1.32699286937714 0.546987473964691 1.63084530830383 1.1285947561264 0.754301190376282 0.7293581366539 1.92016279697418 0.812332332134247 0.95259040594101 2.30438899993896 1.44879519939423 0.4100501537323 1.40016543865204 0.974205791950226 0.667303502559662 2.11469149589539 2.99064016342163 3.53355097770691 1.22363305091858 0.493049442768097 0.704206466674805 2.09130072593689 5.01174068450928 0.884448647499084 0.433154195547104 5.38395881652832 2.35263919830322 0.627587735652924 1.36541509628296 1.29371201992035 1.81538116931915 3.24828386306763 2.82484793663025 5.12685966491699 4.06664848327637 0.950755715370178 1.51441693305969 3.60539793968201 2.8457977771759 1.5705029964447 0.939070761203766 3.46546864509583 1.76208817958832 5.72261333465576 0.750217974185944 0.262014597654343 1.12157547473907 0.572756171226501 3.40289187431335 1.96243989467621 0.12575401365757 0.679148554801941 0.909274876117706 2.63367033004761 2.62571358680725 1.32274758815765 2.20536708831787 0.639463543891907 0.158714354038239 2.21522116661072 1.46103608608246 3.8098566532135 2.45779085159302 0.705418288707733 0.302883714437485 2.62939310073853 0.120659470558167 5.90527200698853 2.74059200286865 2.09339261054993 1.46330952644348 3.58988332748413 6.0123405456543 1.9453661441803 3.44431805610657 2.96717548370361 0.972844123840332 0.442567557096481 1.43581700325012 2.47386980056763 2.25970673561096 6.02467441558838 2.0651171207428 3.87836980819702 0.995359241962433 3.10147213935852 1.23144841194153 6.90146446228027 0.239864662289619 2.1054413318634 3.01558828353882 1.26059281826019 4.92730188369751 3.35689163208008 3.19198846817017 4.5033130645752 1.03531622886658 0.538592278957367 0.998357594013214 2.54009199142456 2.0800998210907 5.62162160873413 3.44168782234192 1.70729458332062 1.38806307315826 4.72560834884644 2.76678133010864 2.55638599395752 2.00965189933777 0.665290117263794 2.57157564163208 0.543525993824005 5.17570543289185 3.58249020576477 0.873606741428375 0.690604388713837 5.57968473434448 0.794410467147827 1.97302377223969 12.1888418197632 0.565789043903351 2.18761253356934 3.15314841270447 1.49161171913147 2.27209830284119 1.92837679386139 1.40686333179474 1.51372289657593 0.419550448656082 4.13422012329102 0.986134886741638 2.2515914440155 0.675025284290314 2.84879279136658 3.2538321018219

ENSG00000179583.16 41.7072944641113 17.6466255187988 8.28801250457764 14.7874326705933 18.5487442016602 12.8392896652222 6.42839431762695 15.3517580032349 1.400630235672 6.45576953887939 14.0971555709839 9.56595325469971 17.146333694458 11.653923034668 4.26434373855591 21.3351173400879 0.53273218870163 40.4719657897949 13.2319135665894 17.1634292602539 42.5657272338867 4.66593170166016 25.2173805236816 14.865403175354 0.590161442756653 2.77758359909058 11.2614555358887 8.424241065979 17.2463932037354 8.28191566467285 13.8488025665283 23.6802539825439 4.03449010848999 14.4631662368774 1.76893901824951 13.5706806182861 13.9091186523438 28.4916343688965 26.1996002197266 7.78252553939819 17.658182144165 55.7994194030762 34.8937644958496 7.60959911346436 11.4157686233521 7.44881677627563 3.44144725799561 20.7325038909912 13.0364542007446 14.8869724273682 6.3213472366333 4.3782696723938 2.44398951530457 8.89217281341553 10.9113492965698 2.07162761688232 4.06420040130615 14.426248550415 16.699462890625 43.3653182983398 31.9101428985596 4.51407098770142 10.7341585159302 9.04623794555664 4.35557126998901 5.53510475158691 7.11801719665527 3.03123688697815 13.9656791687012 9.92277145385742 3.57400035858154 34.6875152587891 4.80220079421997 16.7137050628662 4.4255108833313 6.66351413726807 1.35335206985474 45.1122131347656 1.36495411396027 15.6544075012207 8.53286743164062 22.5781593322754 12.5159635543823 6.08449220657349 7.44850015640259 30.0734443664551 7.80397987365723 4.21155261993408 20.1570949554443 3.0501880645752 26.6424427032471 9.68452930450439 1.58117997646332 10.0344619750977 14.2509441375732 14.0179681777954 20.4288692474365 11.5074777603149 16.7668743133545 20.6651515960693 1.88475394248962 16.4474849700928 13.3181867599487 14.9825954437256 2.88782095909119 3.62540602684021 8.30349922180176 1.31295013427734 13.2775115966797 2.25005841255188 26.2469921112061 9.19517612457275 20.821138381958 5.05764389038086 35.2366371154785 5.56256008148193 4.73251962661743 0.52068167924881 1.74154543876648 4.93143224716187 13.323070526123 0.321644097566605 15.1219673156738 11.6390447616577 7.85890531539917 3.65277600288391 0.868846833705902 11.9303369522095 7.67679738998413 12.2158222198486 4.28911161422729 14.4247407913208 1.97186434268951 0.976841747760773 4.91128444671631 5.10387420654297 4.45842981338501 23.7567195892334 27.6910705566406 22.2693176269531 3.25405812263489 11.9692621231079 9.22368717193604 27.7840785980225 46.2140045166016 12.8523950576782 9.94182586669922 17.152473449707 24.385799407959 2.55597496032715 5.59418296813965 4.77424716949463 29.6157989501953 28.683032989502 2.72040748596191 9.29049396514893 13.5794277191162 11.7020311355591 31.1095905303955 14.4292125701904 33.2386779785156 4.94241046905518 1.38095080852509 9.98330402374268 10.2446842193604 12.6961765289307 3.08726739883423 24.5617160797119 3.8641083240509 14.7443771362305 23.4434928894043 34.2980842590332 1.01870334148407 2.84733033180237 4.07972192764282 14.3745937347412 10.1998586654663 7.54939079284668 5.02457523345947 2.3882474899292 18.2336673736572 17.8779945373535 19.3498401641846 7.86316585540771 11.0433549880981 5.97417306900024 14.8981199264526 21.2821617126465 1.24400663375854 30.0865631103516 18.3274536132812 11.5290193557739 10.0842866897583 21.4812240600586 28.6967029571533 48.4072418212891 27.3760223388672 15.6369457244873 5.71691417694092 1.82713496685028 8.76365947723389 12.4214372634888 6.66251945495605 48.381103515625 16.6276359558105 10.9845390319824 4.29001951217651 30.6414833068848 17.2730274200439 9.42373943328857 2.06412076950073 12.6232986450195 22.3094730377197 3.29561471939087 17.6040000915527 16.9677734375 18.6340789794922 21.6920986175537 3.35916018486023 14.4992914199829 17.0326766967773 11.4803943634033 10.1464748382568 21.6806907653809 24.127872467041 25.0183029174805 5.76467561721802 11.1111450195312 10.4949951171875 31.0426445007324 12.017936706543 2.62872505187988 15.7438697814941 4.42521238327026 12.2074728012085 10.9595804214478 7.47514724731445 1.23885810375214 10.5222759246826 4.82178020477295 3.69377088546753 26.1590309143066 3.46932125091553 9.92474174499512 10.6002416610718 26.1307716369629 18.650598526001 5.18192863464355 26.4492244720459 8.65926170349121 1.53841698169708 25.4464778900146 1.910280585289 10.212381362915 4.9613618850708 16.6087341308594 7.1063175201416

ENSG00000204516.8 48.2231369018555 30.4708003997803 3.21826195716858 17.4658164978027 12.853964805603 32.2406387329102 20.417142868042 12.1095857620239 6.2971076965332 7.70109176635742 8.04632759094238 6.60260725021362 11.6385631561279 8.15187931060791 30.8865585327148 44.8284454345703 0.673626363277435 16.8286914825439 22.3792190551758 21.2403984069824 11.7898616790771 33.9129638671875 7.84855794906616 10.6444940567017 10.6647701263428 5.53186130523682 44.6165924072266 6.15149164199829 9.39646339416504 6.06750011444092 7.08231639862061 19.4925079345703 6.11179304122925 10.2793388366699 2.22647070884705 12.143443107605 12.818675994873 14.0617122650146 16.3935966491699 10.5171508789062 16.2264881134033 23.5928802490234 17.4885349273682 34.8054580688477 8.28068828582764 3.76753544807434 6.14981412887573 17.8079280853271 12.8077087402344 15.6037502288818 9.43792343139648 14.4181785583496 21.4223098754883 18.0780410766602 24.5185108184814 15.7895536422729 23.4113578796387 8.64120960235596 4.49955987930298 13.4818201065063 17.8157768249512 11.5727758407593 33.6651077270508 18.6556968688965 32.3083229064941 8.95871925354004 13.6156854629517 4.06978845596313 14.0385007858276 4.72087955474854 9.75512409210205 24.8260135650635 2.07675838470459 25.2371883392334 33.7945861816406 4.53240585327148 8.08968353271484 15.7707071304321 25.3392200469971 7.6678032875061 11.3693752288818 17.4944438934326 16.5773048400879 19.2786159515381 2.55863904953003 20.8194103240967 14.1235980987549 4.59871768951416 10.873010635376 13.3570041656494 6.32086610794067 4.29552030563354 0.760922193527222 4.85513353347778 4.06312465667725 5.89486312866211 11.993480682373 3.83867454528809 24.180871963501 14.7710418701172 3.28498339653015 14.0462245941162 9.29213905334473 10.7841415405273 3.51226758956909 43.5216865539551 3.52451705932617 1.77955234050751 22.2047348022461 31.8448925018311 27.8636283874512 40.5143623352051 11.0066061019897 9.85656642913818 13.30189037323 11.0203351974487 8.73552131652832 21.2469863891602 9.39094352722168 6.32109069824219 15.659252166748 8.34126853942871 9.30323505401611 12.2553930282593 4.87523794174194 6.04676103591919 8.24876689910889 10.1702690124512 16.237886428833 4.57901048660278 4.10845613479614 12.3140888214111 2.0586564540863 4.96390771865845 13.4322195053101 31.2897968292236 6.93439197540283 52.7685928344727 24.3962593078613 13.0096235275269 4.30458211898804 6.72294664382935 16.4563961029053 10.0911817550659 7.41923332214355 23.7928695678711 7.79816913604736 15.5598440170288 14.1670427322388 2.93976044654846 6.4827356338501 4.95267915725708 7.04967975616455 6.24634504318237 13.1463737487793 19.0250225067139 13.4644155502319 13.7537250518799 12.818154335022 20.5464782714844 10.757776260376 9.16471004486084 1.14395225048065 8.83060264587402 9.96029663085938 11.5908117294312 3.23427581787109 7.84828805923462 3.70806741714478 3.06910753250122 16.9197006225586 11.4444847106934 5.97221422195435 4.48244190216064 5.23710441589355 8.70518493652344 16.8931884765625 9.6657657623291 13.1775207519531 16.1060180664062 5.17282247543335 10.9418935775757 13.92702293396 13.3176755905151 6.01343822479248 3.50339269638062 7.59172630310059 24.5756168365479 4.68159151077271 14.6999263763428 12.5282049179077 44.9076919555664 13.893533706665 17.6522922515869 11.8290414810181 74.7694244384766 12.5597219467163 15.1455316543579 4.66950416564941 3.39686226844788 9.74989414215088 33.9493827819824 5.42833137512207 26.8089599609375 9.20209693908691 13.5830297470093 5.15725231170654 7.13679933547974 6.66629934310913 32.3173484802246 4.61615228652954 4.46716451644897 20.2637138366699 5.90845441818237 26.7117767333984 21.4620838165283 7.85204362869263 19.2794971466064 16.6270141601562 8.17320537567139 7.28242588043213 69.9861068725586 6.12338924407959 34.3103981018066 10.546630859375 9.34139919281006 40.9860725402832 19.8994083404541 20.6932239532471 13.0258159637451 11.6442012786865 2.60515570640564 13.4829397201538 3.55417013168335 14.5085515975952 7.36441707611084 14.9529676437378 1.31916177272797 6.03749561309814 2.13176918029785 19.7452411651611 24.0423164367676 3.46851658821106 3.8478684425354 71.8957824707031 16.5677738189697 9.15117931365967 5.93474531173706 10.7262172698975 11.5115699768066 28.0316963195801 17.4523773193359 28.1875 18.2687797546387 11.6226444244385 11.472785949707 13.3949975967407

ENSG00000001167.13 53.7381172180176 23.7608985900879 23.628173828125 66.4261779785156 29.8408489227295 67.239387512207 27.1067161560059 42.615894317627 33.0853233337402 33.607780456543 42.471508026123 37.7380828857422 29.8569622039795 43.4079246520996 43.4726066589355 32.5930786132812 28.3508968353271 38.7036628723145 46.7572059631348 40.1888084411621 21.6475372314453 51.5933837890625 17.5994281768799 41.484317779541 39.1711463928223 23.6448192596436 22.7050857543945 15.4412784576416 37.0618515014648 53.5135231018066 26.671012878418 38.5731925964355 25.1578598022461 30.8629035949707 35.6142616271973 35.1458320617676 55.5764999389648 35.079704284668 56.9482231140137 38.3100242614746 70.6195907592773 23.9110050201416 32.725902557373 53.6916046142578 17.9249153137207 34.3062515258789 37.8901672363281 19.7185230255127 34.1177787780762 55.5842781066895 24.7423248291016 65.9298477172852 27.8443737030029 36.3067016601562 52.0663604736328 63.457633972168 34.4233703613281 24.421854019165 30.5381832122803 40.7986297607422 62.1954536437988 26.5651435852051 70.5260925292969 37.3905487060547 25.4091300964355 23.1750793457031 37.0627136230469 37.0417137145996 22.9798393249512 44.3480796813965 26.716588973999 39.4052047729492 16.6868648529053 73.3793640136719 35.0979614257812 27.2967338562012 62.0207214355469 37.3173484802246 46.203182220459 35.4299430847168 53.082103729248 26.4784374237061 53.9605827331543 102.232513427734 12.9121837615967 35.7545738220215 26.6330146789551 51.0272483825684 19.7272186279297 63.4298286437988 24.7961254119873 33.7104606628418 28.6399707794189 79.4016418457031 36.6325607299805 29.5771942138672 34.3377113342285 40.9013824462891 31.7791576385498 44.3486022949219 34.592830657959 52.0877799987793 17.0116558074951 42.4501800537109 31.328405380249 38.9359931945801 57.0175704956055 30.273265838623 34.2925720214844 81.2458801269531 30.3838176727295 49.8946151733398 37.301643371582 38.1258850097656 23.3902626037598 35.3096809387207 34.4271125793457 31.4116878509521 44.5753059387207 13.1700782775879 28.6332092285156 16.4828968048096 22.806058883667 24.5909461975098 38.8697204589844 16.6955585479736 24.3040027618408 16.6854858398438 41.9234046936035 56.8259048461914 33.6830177307129 37.5433616638184 15.5400114059448 47.2844505310059 77.3289413452148 91.8906860351562 25.5351676940918 74.7922744750977 43.8819847106934 31.60573387146 26.2497692108154 25.1049919128418 28.0617179870605 34.2424583435059 104.549766540527 37.1724739074707 43.853157043457 42.5507164001465 20.8383598327637 27.9284553527832 21.3698234558105 49.5310745239258 33.9948463439941 37.1213798522949 26.4528045654297 40.2236137390137 26.3309020996094 54.9738464355469 20.8590126037598 28.4529113769531 48.4086723327637 35.0175743103027 18.5257892608643 31.1035251617432 66.1106872558594 23.8961200714111 52.3736190795898 51.9488143920898 18.6409034729004 58.1463737487793 32.872371673584 23.3370780944824 45.3376693725586 49.0060729980469 29.4248561859131 30.269287109375 33.5093688964844 54.913200378418 81.3563690185547 47.7367324829102 36.1337127685547 43.8400192260742 27.7752914428711 37.6682319641113 25.6334133148193 41.3400115966797 30.526330947876 29.4283676147461 136.772262573242 28.7361431121826 54.8667869567871 51.3468055725098 67.8725662231445 30.035135269165 45.6301727294922 44.7600212097168 21.7943439483643 27.369930267334 20.3535385131836 8.27952766418457 34.3371200561523 58.7201957702637 43.0784606933594 32.8257713317871 37.8173828125 33.3862991333008 44.0409545898438 18.4522857666016 23.6409797668457 31.81223487854 23.9668941497803 26.0679359436035 29.0902729034424 43.6665878295898 36.4462890625 64.9962005615234 13.9575939178467 29.6844673156738 49.9169578552246 24.6846561431885 55.1353530883789 47.6375122070312 19.8485164642334 62.9759292602539 17.7059764862061 22.447961807251 46.7132797241211 22.4728622436523 37.8618240356445 24.922420501709 30.5662860870361 40.6911163330078 33.1879234313965 100.050483703613 74.5399703979492 18.0383548736572 27.1898441314697 25.8351669311523 26.7742900848389 8.51474475860596 66.8931579589844 29.2309875488281 32.4389915466309 76.7938537597656 70.8349609375 54.9213066101074 20.3329334259033 40.0379257202148 24.8476028442383 24.7144031524658 31.6955718994141 32.6724548339844 42.5234260559082 39.8325729370117 19.11155128479 17.8951606750488 38.4110107421875

ENSG00000120837.6 21.5185508728027 15.3009147644043 9.73687171936035 19.9310913085938 16.9757270812988 26.8522491455078 15.2816009521484 17.5276126861572 17.9979305267334 24.9041481018066 17.4783821105957 40.1821594238281 21.5772247314453 24.8827209472656 15.048285484314 24.7305603027344 25.8917808532715 20.4036655426025 17.0113964080811 21.3880996704102 19.065616607666 19.1886806488037 24.246187210083 17.8165264129639 29.3359680175781 22.634838104248 24.9955463409424 13.3925790786743 16.8137264251709 23.6492462158203 24.4783191680908 19.2369937896729 10.1441640853882 16.0104293823242 17.6212024688721 13.4909238815308 11.0364503860474 21.677942276001 27.0803356170654 14.0327072143555 24.9381675720215 18.9025726318359 16.6138763427734 18.5757160186768 26.5002098083496 13.0734577178955 30.8418235778809 13.4517383575439 13.884503364563 19.0304069519043 14.7704401016235 11.0621128082275 15.6668787002563 32.5511169433594 19.5450744628906 15.811261177063 17.9812717437744 15.5586242675781 10.5612821578979 21.0230083465576 17.7443447113037 35.4292068481445 24.3995113372803 25.7244281768799 13.796854019165 29.6948642730713 9.80915546417236 19.6092395782471 17.733470916748 15.2924671173096 11.8723831176758 18.489559173584 12.5330677032471 24.0425128936768 33.1532707214355 30.7447052001953 13.2894067764282 8.64843940734863 21.3836212158203 21.1626815795898 25.8156642913818 17.9799156188965 34.8625869750977 17.8302230834961 12.5192279815674 12.2854614257812 14.3737058639526 23.3176193237305 14.7706174850464 17.3739566802979 15.8815279006958 22.6819496154785 17.2012329101562 19.3198471069336 17.0896377563477 13.9567728042603 22.1139430999756 15.0823459625244 12.4128913879395 23.6371097564697 16.0186309814453 11.3687467575073 18.8938579559326 20.8156871795654 13.6706714630127 20.3827285766602 10.1010465621948 22.1519393920898 20.2976875305176 41.0856323242188 18.8609466552734 16.1365165710449 16.5871829986572 13.2946615219116 16.136812210083 31.7394237518311 19.9503803253174 12.1465749740601 18.618049621582 24.0645236968994 21.9551429748535 29.507740020752 20.6804714202881 17.9336433410645 43.5548248291016 26.1668071746826 18.0585517883301 7.05928039550781 9.62699508666992 23.5056304931641 8.52241897583008 18.1009082794189 11.238862991333 24.5166645050049 31.00071144104 27.6389770507812 30.7703914642334 10.1037120819092 21.8093414306641 13.9737701416016 17.5262470245361 19.7430934906006 16.175085067749 13.1378955841064 15.3922128677368 17.6315898895264 15.352165222168 18.8974094390869 18.8687515258789 50.6706962585449 23.0920372009277 16.6860656738281 12.91477394104 21.2237167358398 13.6532011032104 22.6124706268311 34.5704231262207 14.3286714553833 16.4929943084717 18.3156108856201 20.5359039306641 22.7220230102539 9.68758583068848 21.855598449707 22.5093193054199 15.0629043579102 10.1523895263672 20.6851577758789 14.6541986465454 15.6473112106323 21.3243198394775 16.1529808044434 35.1937255859375 21.0218849182129 21.9185485839844 16.3656044006348 13.565037727356 11.5127878189087 13.8405714035034 14.503888130188 22.5845146179199 9.65436840057373 16.7992057800293 17.7080612182617 17.0763244628906 29.459358215332 12.0088939666748 16.869686126709 34.2542839050293 21.4072418212891 25.9780883789062 22.2827777862549 18.0106792449951 22.4357833862305 20.4239292144775 23.5271148681641 21.2507076263428 23.0274257659912 15.0856914520264 8.79913520812988 14.1526031494141 14.1415033340454 12.2930870056152 9.31662559509277 11.4037303924561 17.7222938537598 25.922815322876 12.6238918304443 19.2149791717529 27.9207668304443 24.1651058197021 13.712308883667 14.8316717147827 13.7847194671631 21.2119331359863 17.7244625091553 8.09689331054688 11.837550163269 31.3954524993896 23.3862724304199 18.0494518280029 18.747875213623 23.3999042510986 36.2723236083984 11.6134357452393 21.927074432373 21.2376861572266 14.4473009109497 12.8833341598511 41.9361953735352 17.1261730194092 17.8198547363281 14.9497699737549 15.1889848709106 21.3379173278809 13.7524442672729 13.7564744949341 11.5437307357788 25.0206069946289 9.89767932891846 22.0117988586426 23.6574268341064 42.8493614196777 18.3303775787354 25.6989307403564 13.1261787414551 10.527156829834 11.5759973526001 15.4165630340576 20.5393218994141 19.7225208282471 14.2503938674927 18.7689266204834 18.4990825653076 12.5529613494873 15.7449007034302 18.9950675964355

ENSG00000066136.18 22.8900489807129 21.7823486328125 26.7113647460938 29.1301097869873 22.3427600860596 39.9110679626465 25.441858291626 8.37198257446289 15.6433782577515 17.9256038665771 24.3263721466064 29.1356430053711 44.598258972168 39.0966110229492 30.7607116699219 34.1049346923828 13.2136936187744 27.8060760498047 24.0456104278564 20.5383853912354 25.0924968719482 20.6706829071045 32.9709510803223 24.2179126739502 39.2339363098145 18.1206569671631 45.6517105102539 19.9856052398682 22.2897415161133 43.042610168457 22.89182472229 37.0409736633301 47.8938865661621 37.0455627441406 34.1263465881348 22.1333789825439 32.8204231262207 17.2372875213623 21.3244380950928 31.4985961914062 30.0325393676758 36.205020904541 39.4988021850586 32.3528633117676 14.0488061904907 20.4337425231934 32.3703804016113 21.5156726837158 25.912483215332 30.9837646484375 19.1961364746094 60.8918800354004 22.3442211151123 36.7225532531738 29.4124660491943 37.1047515869141 25.2764511108398 29.6762561798096 24.1676540374756 41.120922088623 21.4628715515137 28.8159847259521 34.7090644836426 27.7447681427002 13.3896207809448 44.2138824462891 28.9263725280762 24.1874122619629 30.8290996551514 26.4431686401367 66.0617980957031 26.9057083129883 26.4502258300781 26.7112808227539 89.3085250854492 21.527811050415 17.1335964202881 35.1788291931152 21.7240333557129 30.3703460693359 24.8719005584717 22.7914867401123 34.7400741577148 15.143443107605 14.4153499603271 22.4723129272461 29.8032398223877 24.2257442474365 19.1213932037354 14.0964660644531 22.4806957244873 34.0546607971191 24.1883335113525 45.0077972412109 21.3770580291748 24.6676177978516 17.7172431945801 28.9496116638184 36.663990020752 24.6279907226562 38.9226341247559 25.1048393249512 19.9143733978271 43.107479095459 24.9548206329346 78.9044189453125 21.7042331695557 44.1103630065918 24.397066116333 20.7998313903809 29.7123241424561 21.1766014099121 20.3550472259521 41.0105323791504 27.9306926727295 14.5655717849731 13.6070423126221 21.076208114624 21.3154926300049 20.6392421722412 25.2892017364502 12.9162111282349 35.798469543457 24.7393703460693 28.4412593841553 18.7589416503906 19.8815307617188 26.9811840057373 29.0447158813477 23.1883697509766 18.9443969726562 32.7106132507324 23.0587635040283 18.0337181091309 49.4493675231934 34.3893241882324 18.1219348907471 21.3957290649414 27.7552719116211 22.0308055877686 21.2031326293945 19.6484622955322 23.5492343902588 26.1681251525879 31.0010929107666 30.3890933990479 19.7923641204834 23.5742130279541 24.0199527740479 20.3005180358887 27.965612411499 29.9352149963379 21.9474830627441 39.4615058898926 25.3389282226562 25.9384727478027 28.7715721130371 40.9504852294922 20.9565353393555 21.4182682037354 31.5522136688232 38.2760124206543 18.8423690795898 23.7967166900635 19.9241142272949 20.7993030548096 17.5561561584473 16.68821144104 22.2350940704346 36.9329643249512 32.6322631835938 26.6921005249023 48.9570198059082 23.544095993042 17.6270885467529 26.4681186676025 20.1084270477295 14.9170017242432 50.2080001831055 22.8028144836426 15.1518716812134 67.6945953369141 23.6502323150635 20.1172523498535 29.5631809234619 22.6394672393799 21.1878509521484 42.7772560119629 47.6471977233887 22.2903175354004 33.4248924255371 22.4939117431641 37.5144691467285 20.2269134521484 28.905101776123 23.4732246398926 30.4580459594727 32.1817245483398 25.436975479126 6.02917385101318 23.589994430542 21.0253524780273 23.1883125305176 21.2744274139404 23.5015640258789 22.5183238983154 26.4215297698975 31.3220863342285 23.4066123962402 37.9219512939453 51.7487525939941 22.902400970459 31.654914855957 30.4017753601074 30.1446666717529 19.9299297332764 21.1755771636963 26.9251461029053 32.7161445617676 44.3339195251465 35.7470169067383 31.9075603485107 15.5582857131958 22.296443939209 22.397647857666 21.2967224121094 21.116870880127 19.7531585693359 25.9099445343018 28.2448196411133 25.2481536865234 40.6156616210938 23.1240329742432 52.4329719543457 22.2389106750488 29.2560157775879 37.3683471679688 15.4045743942261 25.2569255828857 24.2515964508057 23.6531105041504 32.8728485107422 21.7566795349121 42.9886207580566 29.4035682678223 34.8625411987305 22.605411529541 32.8386611938477 31.2676448822021 25.0547122955322 11.6345643997192 23.7409229278564 41.741626739502 45.3270149230957 16.5836734771729 26.8273124694824 54.3313674926758

ENSG00000100600.13 307.396026611328 279.917755126953 142.033554077148 160.379531860352 325.067596435547 283.209655761719 80.9185104370117 178.059143066406 68.009895324707 332.250549316406 102.748840332031 153.761001586914 245.820922851562 135.898864746094 237.818817138672 107.257118225098 319.192779541016 277.283447265625 202.779083251953 146.624069213867 157.985885620117 155.446426391602 176.681747436523 227.597351074219 107.665451049805 102.742462158203 378.718231201172 159.135589599609 179.253784179688 197.248992919922 177.562103271484 313.505340576172 145.742782592773 295.426116943359 97.9367599487305 155.531066894531 653.269775390625 172.576812744141 664.941223144531 213.557510375977 218.6083984375 238.399185180664 450.452514648438 114.562774658203 103.297416687012 335.665802001953 144.062240600586 578.382385253906 281.359832763672 174.686508178711 423.770324707031 101.292236328125 140.801696777344 137.005401611328 486.990692138672 207.767776489258 358.250366210938 262.098388671875 291.005950927734 264.262451171875 128.119079589844 314.373931884766 242.196884155273 120.721305847168 205.261627197266 166.123199462891 94.3719711303711 146.677062988281 337.608917236328 209.293670654297 113.389213562012 200.357986450195 113.69962310791 176.549423217773 128.89714050293 81.2647018432617 108.531967163086 657.434814453125 80.0582809448242 253.013473510742 116.051078796387 253.928527832031 344.585601806641 49.4759216308594 169.092849731445 347.397338867188 130.612976074219 265.847351074219 150.878875732422 144.85676574707 82.2191848754883 195.521789550781 41.8789138793945 142.4482421875 81.7876052856445 119.902275085449 258.555847167969 253.489181518555 262.513244628906 168.160827636719 120.91919708252 271.754577636719 181.224822998047 226.912292480469 258.663543701172 277.593780517578 136.020324707031 74.6602478027344 124.410934448242 72.492561340332 205.755935668945 211.701889038086 285.485534667969 229.124359130859 184.429229736328 663.57763671875 213.358276367188 126.062171936035 115.40104675293 138.536193847656 101.589210510254 50.8798179626465 133.921249389648 252.366195678711 257.418609619141 128.768310546875 148.090362548828 131.795883178711 129.462066650391 154.007217407227 76.3210220336914 190.263687133789 33.6631050109863 229.068481445312 158.613540649414 255.26496887207 302.853759765625 392.9609375 110.442878723145 189.840393066406 135.300552368164 461.647735595703 104.626670837402 200.662384033203 236.869186401367 327.968597412109 134.210433959961 285.706604003906 132.60188293457 191.849319458008 317.742034912109 212.460800170898 170.181686401367 257.537933349609 124.693794250488 122.874618530273 262.890045166016 290.464080810547 1380.17932128906 198.95231628418 127.640251159668 234.231262207031 42.806396484375 133.050415039062 125.436058044434 205.781768798828 183.663848876953 43.1307029724121 71.7070999145508 69.2429580688477 166.351211547852 344.910675048828 71.2896041870117 151.248107910156 438.387512207031 184.450759887695 98.2592010498047 380.357330322266 333.984680175781 131.614685058594 49.2828216552734 205.470962524414 195.245971679688 339.64013671875 123.814422607422 267.57080078125 138.729385375977 139.167846679688 80.4154663085938 252.32389831543 142.053421020508 160.269729614258 222.797332763672 215.223114013672 177.920471191406 90.0008239746094 270.987609863281 198.113128662109 115.622901916504 42.7934074401855 119.749694824219 111.770629882812 163.369369506836 517.476440429688 205.26383972168 211.803344726562 432.737915039062 142.978530883789 209.919677734375 224.517486572266 226.731979370117 279.147491455078 253.925140380859 494.538482666016 242.993270874023 118.512825012207 173.922805786133 204.861602783203 164.218688964844 112.772850036621 248.328308105469 156.561294555664 193.37760925293 249.220565795898 173.667068481445 218.085327148438 167.794723510742 139.225723266602 220.752380371094 221.517044067383 121.080703735352 183.120941162109 250.221420288086 161.878799438477 205.522644042969 198.430374145508 267.318481445312 121.21940612793 224.193115234375 121.504928588867 165.41764831543 173.684753417969 86.6345672607422 601.242980957031 139.924774169922 156.321807861328 239.42643737793 128.826889038086 275.224212646484 369.116760253906 141.186553955078 163.887313842773 121.252853393555 138.401916503906 69.8650283813477 205.925109863281 217.563583374023

ENSG00000204264.7 389.809753417969 390.376983642578 67.3332290649414 247.866271972656 235.980743408203 307.135192871094 135.023086547852 281.699554443359 431.040618896484 141.746185302734 237.863220214844 162.402648925781 233.382583618164 174.428939819336 202.615585327148 265.571899414062 64.9075088500977 309.060089111328 229.317459106445 149.827239990234 142.940032958984 370.071166992188 116.727149963379 138.759307861328 44.626163482666 105.196342468262 473.561950683594 150.087844848633 205.332550048828 125.263900756836 214.363998413086 292.567932128906 254.719879150391 85.9101181030273 95.1783142089844 169.196899414062 270.100921630859 259.982391357422 170.719024658203 134.43098449707 240.475204467773 265.173217773438 286.896270751953 94.7540893554688 146.070251464844 113.463516235352 330.071899414062 148.084823608398 269.880645751953 114.95817565918 230.843780517578 103.905288696289 127.291297912598 218.310195922852 269.934112548828 209.896896362305 556.321655273438 276.332336425781 206.747467041016 162.796096801758 271.578979492188 70.148811340332 207.270401000977 69.4961242675781 264.670440673828 118.807357788086 187.97184753418 75.7781524658203 135.982849121094 220.643325805664 202.935272216797 196.507080078125 70.7441253662109 159.314666748047 377.822937011719 95.5885772705078 177.366439819336 319.555236816406 56.6396026611328 185.654220581055 201.580368041992 245.335815429688 98.9662322998047 170.84455871582 189.435134887695 204.537231445312 208.351455688477 105.53441619873 197.738418579102 354.546875 70.9806289672852 114.743980407715 90.5826416015625 112.959243774414 47.0386657714844 137.438262939453 175.734100341797 141.330261230469 199.846557617188 147.49479675293 293.379333496094 144.793640136719 213.936828613281 284.271362304688 89.2648086547852 388.955993652344 196.004089355469 71.1952514648438 228.384613037109 66.8142242431641 190.232086181641 607.831481933594 178.126281738281 183.004745483398 108.378036499023 166.534591674805 442.784362792969 82.3073501586914 86.8503799438477 93.8865280151367 327.212463378906 101.12141418457 134.180862426758 108.549140930176 120.858535766602 85.3821563720703 276.684844970703 225.197174072266 241.837890625 174.555847167969 122.555374145508 150.631317138672 105.044731140137 60.7803993225098 135.183303833008 138.478179931641 203.869827270508 419.262481689453 273.816375732422 250.188766479492 66.3982620239258 145.263824462891 117.75171661377 141.274673461914 355.742309570312 119.982551574707 222.650451660156 257.631103515625 189.217819213867 60.4689025878906 326.18603515625 103.060997009277 158.966873168945 98.5587158203125 336.099945068359 124.501594543457 151.140609741211 161.167816162109 104.619102478027 136.978729248047 140.282409667969 65.2481079101562 155.3564453125 240.269973754883 126.721389770508 272.687469482422 128.714080810547 29.4942779541016 81.8948745727539 336.411285400391 145.173248291016 164.727294921875 148.200210571289 133.060012817383 234.10334777832 255.066864013672 161.249237060547 228.024124145508 172.344329833984 118.059509277344 14.7738466262817 195.013458251953 228.944915771484 110.794410705566 109.081016540527 139.878555297852 151.721862792969 538.462585449219 139.532119750977 231.456024169922 102.006698608398 301.306640625 366.522399902344 287.273559570312 198.766220092773 469.654846191406 175.641723632812 161.538635253906 86.2317657470703 44.3490371704102 274.472625732422 238.771987915039 181.376220703125 511.164733886719 160.380737304688 185.466278076172 116.486930847168 96.8238143920898 110.384841918945 727.530639648438 111.326858520508 165.418060302734 180.28791809082 144.957870483398 391.862243652344 319.024169921875 164.213180541992 193.132080078125 140.163116455078 107.212882995605 246.088668823242 156.874053955078 108.94132232666 288.25439453125 152.368881225586 130.495742797852 202.579330444336 343.474395751953 227.483322143555 256.717864990234 99.7048492431641 58.6489906311035 186.803558349609 39.9416809082031 203.060577392578 177.96028137207 304.204223632812 45.7696876525879 124.173072814941 169.329879760742 217.096115112305 164.880981445312 150.853942871094 171.114700317383 271.550994873047 92.715217590332 173.228515625 71.0902481079102 93.0024261474609 139.780639648438 225.012542724609 237.513977050781 134.247375488281 355.789916992188 265.073455810547 199.429382324219 325.861541748047

ENSG00000100764.12 13.9712553024292 9.98758792877197 7.34529685974121 10.4410514831543 8.63076496124268 18.2971038818359 5.38196134567261 11.3607788085938 15.9255819320679 9.05134391784668 15.2582492828369 12.784158706665 8.6513147354126 9.39777088165283 9.62673091888428 13.2662954330444 8.4418888092041 11.4118461608887 11.0574007034302 12.1178197860718 5.64299917221069 16.1194095611572 8.10221004486084 12.4892101287842 17.9389114379883 9.89986896514893 22.8264484405518 2.83639860153198 5.09714365005493 6.18229532241821 8.8176794052124 11.402735710144 2.16895270347595 10.3867197036743 3.55773282051086 7.62146234512329 12.9299983978271 8.17373943328857 12.4085063934326 28.3661975860596 12.8858852386475 6.68749570846558 10.8788461685181 5.14812803268433 3.6788969039917 6.30154371261597 17.5088291168213 9.79209327697754 14.2794790267944 11.1973695755005 8.99318599700928 6.53118371963501 8.54499244689941 8.84776592254639 39.19873046875 12.6749544143677 16.4525356292725 11.6670742034912 10.1851291656494 11.0313186645508 6.57725429534912 9.19903373718262 17.3135051727295 7.27195310592651 7.76887655258179 8.13715267181396 7.41011190414429 8.82090282440186 8.47829532623291 8.27714443206787 7.53921604156494 26.499568939209 8.57625484466553 10.8541679382324 19.1256122589111 12.4276552200317 14.1467113494873 16.0009479522705 9.62997245788574 7.00247192382812 5.1530933380127 8.13072776794434 14.1964426040649 6.0568675994873 4.66135025024414 7.3009614944458 11.4273872375488 15.2893514633179 4.55866479873657 6.39792394638062 6.51850032806396 10.737585067749 7.52497482299805 9.93454647064209 5.03754949569702 6.87801456451416 9.45293998718262 8.33609104156494 11.9000682830811 8.44081401824951 10.5600690841675 7.62099170684814 6.12930250167847 8.22331237792969 8.22849655151367 19.9207420349121 10.1292991638184 7.0567774772644 10.103910446167 9.84824752807617 8.63375949859619 11.7832279205322 7.8378324508667 9.66678428649902 6.19079065322876 15.7064733505249 9.22014617919922 7.36870718002319 4.5954794883728 6.29744720458984 7.58346271514893 12.6540756225586 7.75987672805786 8.86033916473389 12.371940612793 5.36081171035767 11.8764505386353 1.68459582328796 9.01827621459961 11.0847959518433 5.76715183258057 6.22935485839844 2.10586762428284 6.10617208480835 18.0230464935303 13.3717250823975 13.4312772750854 16.1729183197021 7.23347234725952 8.91425037384033 4.09381818771362 11.9695615768433 5.091139793396 4.91341924667358 13.3146553039551 10.9414739608765 6.15185689926147 10.6181077957153 8.10320281982422 10.3750467300415 9.75949764251709 8.72950077056885 6.02204084396362 11.1757888793945 10.8213262557983 7.35712718963623 8.17183303833008 8.96412658691406 5.63387823104858 9.71944332122803 6.51892566680908 10.7772493362427 2.83739256858826 13.4910507202148 5.93537998199463 8.14850330352783 9.8294563293457 4.58000326156616 5.41235733032227 6.96063184738159 12.0368347167969 6.15314388275146 12.8167743682861 5.42178201675415 17.6600914001465 8.35505771636963 6.03760719299316 11.0946235656738 20.7515602111816 14.6933889389038 3.64688777923584 9.63976097106934 7.04244899749756 11.4023132324219 7.89404296875 14.8090553283691 6.54327249526978 19.5419483184814 29.714807510376 6.33315467834473 7.56278276443481 14.6019897460938 7.13482332229614 8.7683048248291 12.0307769775391 8.50469970703125 8.66967010498047 11.5523548126221 5.21798229217529 4.15161037445068 7.6013708114624 8.14369678497314 8.72536182403564 11.13791847229 8.38408756256104 8.6237907409668 15.4668302536011 5.00164031982422 8.34640216827393 15.5813026428223 10.076301574707 7.10813665390015 7.21267938613892 12.6879558563232 12.7072067260742 9.48882675170898 3.59410524368286 6.37215757369995 25.4072608947754 9.27959251403809 20.7116241455078 11.7320604324341 17.8773403167725 12.5362281799316 4.24261951446533 15.9986248016357 9.86184883117676 8.36307525634766 6.31470489501953 10.0809173583984 4.69011211395264 10.0709800720215 9.27548408508301 8.63245582580566 9.58265590667725 8.32797145843506 10.9251680374146 9.92997455596924 8.04930973052979 2.9260847568512 7.747483253479 7.95180511474609 9.15220832824707 10.5649394989014 10.939001083374 8.21752452850342 11.5305042266846 8.16671371459961 10.9758739471436 6.52350044250488 16.4565162658691 6.64887571334839 25.3849563598633 18.6897449493408 9.02307987213135 7.72282361984253 10.7504091262817

ENSG00000161057.9 159.246017456055 106.987342834473 63.4979209899902 79.0557098388672 98.2317886352539 137.465118408203 125.537658691406 101.693748474121 175.639038085938 56.518440246582 118.053115844727 70.997200012207 118.978698730469 99.8907699584961 99.5845413208008 95.1399383544922 43.5256614685059 89.052864074707 113.830383300781 122.933197021484 50.835636138916 131.747467041016 47.885913848877 90.83642578125 156.627716064453 51.9998321533203 162.839202880859 35.1549377441406 51.6256484985352 60.5648727416992 82.4439239501953 130.128494262695 56.7866020202637 81.2052307128906 51.1769561767578 68.3376998901367 74.8711471557617 75.3577041625977 61.8626670837402 93.9251861572266 70.6149063110352 67.1951904296875 62.0065116882324 117.190254211426 49.1509780883789 76.5047988891602 104.068229675293 73.5895156860352 93.4928894042969 99.3716354370117 51.0178642272949 105.063858032227 86.8031997680664 98.3777923583984 143.844619750977 88.421272277832 181.358673095703 91.2145233154297 67.7971954345703 61.9785003662109 81.2696914672852 53.7965545654297 134.196212768555 92.9739761352539 135.56982421875 67.5282745361328 113.844245910645 77.960693359375 76.1924133300781 67.6921157836914 123.67308807373 98.532096862793 68.2866363525391 110.102928161621 105.606323242188 134.968170166016 98.2079238891602 114.61262512207 78.0423736572266 44.7056922912598 55.5229110717773 68.0195236206055 153.945007324219 82.2587585449219 52.6337394714355 88.05126953125 74.7183380126953 96.7542037963867 63.4815139770508 93.7210388183594 66.9813537597656 79.4459762573242 83.7073516845703 101.929016113281 24.8066062927246 65.8194961547852 68.6619262695312 57.8032188415527 99.5634307861328 70.4761962890625 50.5572471618652 64.3030853271484 55.3129768371582 90.6158905029297 86.7481460571289 123.805358886719 99.4611358642578 94.3951034545898 90.1001586914062 87.7277297973633 76.9412002563477 138.695739746094 64.111457824707 103.609657287598 48.0776214599609 90.6074676513672 188.272064208984 108.513595581055 43.5434341430664 53.9755401611328 83.7533950805664 79.2297058105469 52.872127532959 78.8549880981445 90.9686813354492 44.7487640380859 86.2010040283203 61.0392570495605 63.9321174621582 101.012840270996 46.3494834899902 60.8268127441406 65.7393569946289 96.3115539550781 61.1802597045898 140.517013549805 95.8776016235352 239.220016479492 110.764282226562 62.152530670166 52.7057266235352 88.8379516601562 85.4950561523438 50.0922317504883 121.560180664062 97.2795028686523 87.0574264526367 91.8091888427734 72.4788131713867 89.2700576782227 179.570922851562 37.6858520507812 58.0653610229492 72.9441757202148 78.0625915527344 71.9483032226562 49.1501922607422 97.209358215332 49.8696403503418 92.3032073974609 83.1060485839844 104.384323120117 40.9805297851562 112.568733215332 84.347900390625 65.456787109375 66.8302612304688 34.6356391906738 66.8455581665039 70.6842193603516 132.756225585938 52.0857887268066 100.820770263672 49.7515068054199 108.329627990723 73.4339065551758 66.7490158081055 114.810279846191 68.8891220092773 75.9978485107422 33.7400779724121 74.3491287231445 135.338973999023 99.134880065918 73.2753524780273 61.8197021484375 62.808162689209 105.711563110352 97.4471817016602 67.9896621704102 85.5213241577148 62.9409294128418 73.0463714599609 75.6467132568359 105.825477600098 92.6314392089844 34.6427841186523 87.6825103759766 52.2472953796387 61.9349365234375 69.7263565063477 118.316909790039 73.3419876098633 100.408714294434 59.4359169006348 69.6256332397461 73.6774444580078 52.8878974914551 51.196361541748 150.290115356445 49.6304969787598 50.2576675415039 80.1107330322266 90.9465866088867 93.6598587036133 89.6814498901367 41.2349395751953 77.2572402954102 121.261260986328 101.891677856445 138.099487304688 129.145278930664 103.671936035156 197.753173828125 46.8806076049805 102.445755004883 87.712272644043 171.964157104492 70.6673736572266 75.8747787475586 64.2601013183594 73.6419906616211 93.6466979980469 87.2233810424805 82.1777725219727 72.6269302368164 84.677490234375 94.1792984008789 61.7767715454102 47.5572853088379 78.5203399658203 85.7442092895508 79.2475128173828 71.6808929443359 102.722633361816 53.241268157959 68.3447189331055 72.2347793579102 137.245651245117 80.8728485107422 109.658874511719 106.198348999023 86.073127746582 119.257987976074 420.692291259766 70.6487503051758 103.317420959473

ENSG00000165916.7 219.499740600586 157.561599731445 131.726821899414 148.686996459961 170.341751098633 290.488342285156 92.1223602294922 102.01033782959 362.827270507812 179.121978759766 202.815155029297 177.742935180664 212.324508666992 155.553680419922 266.385955810547 321.720611572266 109.826934814453 114.464218139648 192.918258666992 146.083633422852 103.372756958008 239.641647338867 114.117256164551 135.322631835938 116.678405761719 113.522750854492 284.868438720703 117.898429870605 130.30778503418 147.938415527344 94.3927612304688 161.610580444336 188.330978393555 165.033142089844 134.093109130859 136.695449829102 136.996444702148 100.494522094727 123.222274780273 189.90625 144.595504760742 125.099967956543 180.852645874023 134.85417175293 92.7529220581055 122.850875854492 499.199035644531 131.180648803711 193.519958496094 127.545890808105 165.253707885742 113.09903717041 119.789108276367 157.187896728516 264.616455078125 141.752868652344 156.110198974609 198.721084594727 120.285438537598 134.328186035156 141.885696411133 114.475021362305 174.200424194336 150.698257446289 120.656829833984 212.739273071289 137.809722900391 151.975311279297 163.38395690918 184.954467773438 128.945251464844 237.070587158203 149.171783447266 123.36164855957 221.182846069336 275.746795654297 144.74755859375 184.183288574219 194.039398193359 118.829139709473 128.337509155273 95.9472503662109 169.939468383789 166.814224243164 140.533477783203 78.7860488891602 135.24739074707 163.736190795898 105.966407775879 185.565811157227 128.495407104492 131.174285888672 249.953231811523 238.280349731445 26.0739059448242 138.721282958984 145.856872558594 86.2368698120117 150.379272460938 129.877349853516 175.868087768555 160.948944091797 118.667114257812 178.035430908203 160.028656005859 276.122283935547 154.114364624023 125.552185058594 139.072708129883 189.81559753418 101.223411560059 144.95002746582 146.892181396484 157.381729125977 94.250862121582 72.1719360351562 263.160614013672 259.855346679688 89.3450622558594 134.44938659668 187.443405151367 228.868057250977 76.9679183959961 171.382354736328 224.052581787109 153.09065246582 228.053207397461 165.81217956543 132.282028198242 142.837982177734 102.46858215332 110.520088195801 139.931594848633 161.833938598633 283.741302490234 165.408157348633 172.156539916992 212.328872680664 134.181091308594 113.902145385742 90.0234756469727 122.118240356445 131.211395263672 98.0114212036133 104.987655639648 155.719161987305 112.023628234863 156.957336425781 139.030746459961 130.016693115234 293.126739501953 100.0830078125 87.4906005859375 104.703689575195 160.197769165039 129.603424072266 132.450225830078 181.079086303711 92.5746994018555 120.883636474609 131.707733154297 135.918762207031 126.947189331055 185.565139770508 95.7633666992188 120.213516235352 122.398918151855 22.8459529876709 163.867553710938 219.409545898438 302.264556884766 97.9856872558594 228.032012939453 123.46768951416 155.905471801758 195.510055541992 198.593780517578 171.803573608398 187.507217407227 187.576446533203 11.0226278305054 168.350936889648 109.057624816895 145.559448242188 121.628814697266 162.481750488281 146.147796630859 282.666381835938 275.150024414062 137.76643371582 116.55738067627 187.160293579102 147.709274291992 164.49235534668 162.197738647461 111.27481842041 141.614929199219 205.206100463867 132.754791259766 41.6672019958496 125.203956604004 163.339492797852 203.798629760742 149.2392578125 148.814910888672 59.3655052185059 268.707794189453 89.5035629272461 110.560195922852 221.875183105469 77.3530883789062 124.722770690918 194.180816650391 176.44189453125 119.473602294922 158.838790893555 96.5138473510742 84.2674942016602 227.661544799805 184.421478271484 236.705505371094 253.664947509766 129.815933227539 183.113754272461 99.7840194702148 146.8828125 276.334564208984 143.999084472656 217.490142822266 118.832748413086 225.344421386719 141.385162353516 178.231903076172 205.136749267578 96.1845474243164 210.344253540039 219.065170288086 120.695861816406 142.573776245117 174.6474609375 185.355361938477 166.143280029297 145.048645019531 161.018280029297 149.157440185547 77.1937789916992 132.800750732422 191.070571899414 136.878875732422 144.240539550781 164.659210205078 135.589492797852 266.256591796875 292.003326416016 171.70198059082 151.075180053711 211.108367919922

ENSG00000013275.6 136.164291381836 154.316680908203 94.5032272338867 139.652862548828 223.185180664062 148.684036254883 162.716171264648 53.9587097167969 238.510192871094 101.392227172852 156.615234375 132.860748291016 142.744918823242 94.4523620605469 90.2506332397461 83.7873153686523 102.607025146484 95.262939453125 104.50415802002 116.003128051758 75.035888671875 235.645294189453 86.0139389038086 95.5613708496094 273.188903808594 188.591918945312 116.453247070312 104.455520629883 83.9233856201172 86.3040390014648 78.4415969848633 112.726600646973 245.029159545898 106.386161804199 89.7491760253906 82.3514251708984 186.834335327148 103.221229553223 87.6001968383789 170.213897705078 118.454864501953 99.9113693237305 90.0855560302734 83.2756652832031 61.1138916015625 68.5594329833984 137.386962890625 90.3113327026367 100.314735412598 117.014320373535 117.845489501953 128.46728515625 88.8121109008789 221.217178344727 135.547912597656 149.282821655273 186.14714050293 106.765922546387 129.767318725586 142.049331665039 119.632133483887 136.849655151367 191.978988647461 96.2989501953125 398.385437011719 115.349723815918 139.462936401367 92.8613662719727 87.8794403076172 125.597610473633 183.608581542969 187.811981201172 138.969085693359 88.1208724975586 353.441619873047 255.334320068359 102.975852966309 92.4013061523438 319.209655761719 104.290870666504 95.4503860473633 79.226921081543 147.628356933594 121.999244689941 70.1879959106445 121.04460144043 74.8479995727539 189.228179931641 107.364280700684 131.679336547852 39.6482086181641 97.9947204589844 102.991737365723 93.5541305541992 24.2422657012939 96.6833724975586 136.409698486328 93.3044662475586 123.010643005371 102.567512512207 150.248931884766 90.155387878418 84.7846527099609 130.082550048828 146.550048828125 195.142700195312 87.0900802612305 112.845367431641 98.3675537109375 144.776489257812 108.360763549805 270.856964111328 85.7708511352539 184.76921081543 88.0124206542969 80.3049468994141 160.156723022461 149.379776000977 131.696594238281 71.1576385498047 131.707260131836 106.477561950684 77.1514434814453 113.838912963867 103.521339416504 82.9303283691406 145.162582397461 140.015365600586 91.6756210327148 127.11604309082 86.7584609985352 82.5290374755859 114.447540283203 72.6579818725586 365.526336669922 189.879013061523 105.922218322754 125.212326049805 109.385902404785 90.6210021972656 114.271530151367 114.581497192383 163.030776977539 59.1665954589844 104.407127380371 289.876190185547 85.6563339233398 104.755256652832 82.9462051391602 145.476623535156 157.016983032227 132.489669799805 81.2859802246094 75.7360610961914 146.063903808594 183.101333618164 142.973190307617 93.7224884033203 56.5797538757324 101.525177001953 94.5201187133789 108.75284576416 87.0309295654297 88.6292877197266 74.6745986938477 85.473274230957 62.933292388916 20.6872444152832 180.333648681641 117.512817382812 180.950134277344 75.2539672851562 191.118927001953 117.179969787598 162.14631652832 130.201141357422 460.853302001953 142.858825683594 194.334548950195 118.260871887207 14.3791418075562 112.092269897461 170.177993774414 227.123611450195 174.432708740234 176.387054443359 93.8378295898438 125.858367919922 255.90153503418 124.282257080078 77.052734375 238.798355102539 100.157417297363 92.7764511108398 63.8544654846191 167.706985473633 145.506774902344 100.008140563965 93.0842361450195 38.9244613647461 66.3274841308594 156.834182739258 137.447860717773 151.536102294922 83.453742980957 67.0419311523438 165.032302856445 67.7676391601562 65.3088455200195 176.167190551758 93.7902069091797 141.652481079102 198.968658447266 146.725799560547 129.48307800293 156.782180786133 75.1855850219727 105.328468322754 207.411804199219 148.671783447266 251.973205566406 196.039520263672 86.2304306030273 122.379219055176 70.4453735351562 107.181945800781 133.110443115234 103.329307556152 113.659164428711 83.4943084716797 79.701171875 85.4482498168945 125.17463684082 159.420654296875 118.42546081543 87.4557342529297 87.9558944702148 207.16535949707 123.876800537109 61.239803314209 189.294509887695 128.249954223633 118.764068603516 104.50577545166 158.127410888672 56.3251953125 58.5541000366211 136.982421875 102.680198669434 121.9150390625 141.660751342773 105.975479125977 211.380645751953 145.632797241211 109.294296264648 119.944137573242 112.825019836426

ENSG00000087191.11 106.435302734375 74.6321258544922 82.7140350341797 121.299186706543 95.3101654052734 105.904747009277 122.268936157227 46.6651420593262 54.6434020996094 66.0346832275391 106.756660461426 91.7577133178711 81.9877014160156 54.0372314453125 139.103729248047 78.2702026367188 71.5892944335938 77.3202133178711 94.2621536254883 89.9536285400391 63.9252891540527 130.482238769531 66.4007339477539 89.6659393310547 107.778945922852 84.2016220092773 124.581130981445 60.4783058166504 93.8347778320312 64.5685119628906 63.7256889343262 76.9389190673828 101.147499084473 93.9155197143555 89.5292816162109 81.6980361938477 105.296012878418 64.656005859375 75.7677536010742 69.2689743041992 91.9044876098633 79.304801940918 112.313346862793 121.27481842041 56.3697395324707 90.6618118286133 83.6390075683594 70.8784484863281 113.922149658203 108.025466918945 71.9463653564453 202.954391479492 111.591506958008 89.4135284423828 99.5368804931641 107.752601623535 92.1009979248047 98.5910873413086 75.2432022094727 92.248420715332 100.972297668457 93.4671630859375 133.589889526367 73.5691223144531 65.9988327026367 74.9308166503906 114.796203613281 94.6959609985352 123.81453704834 103.110328674316 81.5069732666016 112.544563293457 101.780319213867 75.6347961425781 100.454780578613 139.012710571289 88.1462173461914 108.902969360352 194.22492980957 74.1413726806641 86.2110366821289 60.7850341796875 91.6858978271484 99.8929138183594 90.187126159668 61.6654052734375 81.9910354614258 96.2458953857422 81.7081832885742 64.5900039672852 28.4384231567383 88.4471130371094 120.567085266113 114.756736755371 24.0285587310791 67.2402572631836 98.30224609375 69.0678634643555 74.1096954345703 84.5801696777344 114.355445861816 95.3737335205078 90.2367401123047 141.784133911133 109.940780639648 57.3952369689941 73.4770355224609 66.5911178588867 62.6050338745117 76.4898376464844 58.2203369140625 69.79248046875 84.4468383789062 141.8212890625 67.5470275878906 74.6875839233398 92.2162170410156 169.384399414062 91.5616912841797 68.5078277587891 78.3666915893555 26.9483242034912 64.0549468994141 89.3560256958008 96.994873046875 81.541259765625 59.5963172912598 80.0464172363281 80.5430908203125 92.656005859375 62.3572654724121 60.1833152770996 65.45166015625 58.3837432861328 117.372589111328 119.114219665527 78.9161987304688 139.733337402344 71.4675369262695 56.3941955566406 69.6175842285156 74.7704544067383 115.052642822266 54.732780456543 120.009254455566 78.4784927368164 72.1807327270508 92.7367935180664 83.5661468505859 87.6245346069336 84.1364212036133 80.5280456542969 71.1562118530273 76.2355651855469 84.0401382446289 74.0534439086914 45.4383697509766 71.5859069824219 64.6739807128906 76.0548553466797 92.1434478759766 93.3583297729492 68.9494476318359 98.514404296875 47.4844551086426 47.3070335388184 73.4682693481445 13.2785387039185 83.1357955932617 122.741592407227 118.528511047363 63.1277084350586 144.512329101562 63.9414482116699 155.341903686523 86.2360229492188 127.131141662598 95.1901550292969 71.5594329833984 79.7455673217773 11.9730358123779 87.0890197753906 85.821662902832 138.249923706055 85.8161773681641 95.8291473388672 113.409294128418 127.85717010498 137.444198608398 81.6153030395508 56.6898880004883 129.215957641602 95.6534805297852 94.2996215820312 84.7951354980469 143.152862548828 84.7017440795898 86.1456985473633 76.4090728759766 12.9109077453613 59.228458404541 96.1219100952148 83.6941375732422 87.1789932250977 72.3457870483398 72.4148101806641 64.1576461791992 58.4399375915527 65.9334487915039 125.641662597656 79.2666778564453 75.9969100952148 81.8293304443359 78.5152816772461 82.4161911010742 189.889022827148 58.4316368103027 83.3766708374023 122.577156066895 151.593215942383 106.147308349609 169.884429931641 70.6065292358398 141.439224243164 43.158821105957 77.836296081543 137.234008789062 119.732643127441 111.317039489746 80.4912948608398 73.397346496582 100.771797180176 156.308670043945 61.9450416564941 64.6713256835938 90.1633148193359 54.6293411254883 167.80143737793 81.2181625366211 74.1348190307617 97.4059906005859 103.505149841309 84.114143371582 84.661262512207 126.758529663086 62.7418899536133 53.7466125488281 122.266479492188 64.2140274047852 105.784164428711 56.848072052002 98.4373245239258 131.898040771484 197.546020507812 121.606796264648 79.2793350219727 88.3031692504883

ENSG00000100519.10 36.0525131225586 36.1808586120605 20.644905090332 29.5728168487549 29.6568832397461 49.4750442504883 21.4524765014648 39.4682998657227 106.500015258789 18.3624496459961 47.9778289794922 29.6163864135742 27.8745994567871 20.8873252868652 29.237117767334 67.0282135009766 27.0226821899414 35.756763458252 31.5735378265381 47.9947929382324 20.4806976318359 54.9516525268555 26.9107933044434 47.1216430664062 34.8104209899902 23.8619441986084 50.8627815246582 16.3844623565674 24.011926651001 18.4867572784424 19.7606372833252 28.8874912261963 12.7643423080444 26.5336933135986 23.6988410949707 28.9624156951904 36.8801460266113 24.1357593536377 38.3731307983398 79.8115768432617 30.5260448455811 22.6702098846436 40.4685707092285 28.2663669586182 24.1507434844971 30.9877243041992 82.2686462402344 23.5483455657959 43.9585914611816 29.5313014984131 26.4150505065918 26.337272644043 13.2628602981567 23.7603416442871 51.4394569396973 43.3916206359863 50.1455612182617 35.3718528747559 24.9397964477539 25.1514587402344 26.9131870269775 25.6831912994385 36.4190444946289 44.1137809753418 34.9965934753418 27.7834358215332 24.094841003418 29.2553577423096 27.4961566925049 33.5722999572754 30.4405612945557 42.5513343811035 26.9554443359375 27.2133769989014 31.446533203125 25.9978446960449 40.0577354431152 36.4848442077637 34.0950164794922 24.1242656707764 23.9028148651123 20.882230758667 40.8542098999023 22.1357383728027 24.8118362426758 17.480619430542 26.3669013977051 33.5352325439453 18.0722942352295 59.7886695861816 18.9292335510254 33.0318450927734 22.8923721313477 21.9429950714111 24.6456890106201 21.9052639007568 25.1852550506592 34.1538772583008 26.6456298828125 26.4485893249512 32.2412147521973 26.9611988067627 23.32399559021 26.2818603515625 15.7741765975952 60.6207389831543 40.1147956848145 35.3358345031738 32.7853164672852 34.9991722106934 15.3758516311646 33.1971168518066 28.197868347168 30.6210746765137 20.6048278808594 65.1227188110352 32.9729347229004 26.1904716491699 17.5775337219238 24.6395301818848 36.8150863647461 66.789176940918 18.6329784393311 22.7768592834473 40.4474563598633 19.4799766540527 47.5912551879883 18.8342323303223 24.8168754577637 34.3910484313965 20.4738998413086 47.6657104492188 13.4987754821777 39.7962913513184 69.4558410644531 51.7331504821777 37.1132736206055 45.6687812805176 19.6108913421631 25.1649723052979 18.3323211669922 36.4162101745605 23.0224151611328 11.6596059799194 28.6924934387207 20.0540046691895 23.3047466278076 29.5006504058838 19.8537178039551 31.1663398742676 22.966215133667 30.6351413726807 21.1892585754395 30.2625942230225 35.9456596374512 18.5757274627686 61.459171295166 31.7484340667725 19.8687114715576 17.116569519043 19.6785984039307 29.5839328765869 19.941930770874 46.0595397949219 25.0939178466797 37.6421318054199 32.5356407165527 26.7645721435547 23.1679248809814 17.4539909362793 48.6658172607422 19.8942947387695 22.0857849121094 25.9656314849854 44.365062713623 24.1430416107178 24.3039627075195 39.1085968017578 67.5829162597656 43.6142616271973 35.1701507568359 30.2530517578125 26.7413063049316 37.4613647460938 32.1322631835938 42.3025131225586 21.3498859405518 42.4420166015625 55.0575065612793 26.8679580688477 37.6736030578613 52.7845840454102 17.7252750396729 27.2387161254883 38.7462005615234 27.5380401611328 32.7557334899902 25.5858955383301 20.4259014129639 29.6746921539307 23.4486923217773 46.1888008117676 21.9080505371094 24.2471961975098 27.6046142578125 29.7638816833496 223.96076965332 16.9830837249756 19.326135635376 58.7753524780273 51.194278717041 21.6554012298584 24.9262981414795 29.00217628479 35.9621772766113 40.4541282653809 14.1168689727783 22.6454124450684 28.5710220336914 30.9816513061523 36.3888130187988 27.2823238372803 40.3463554382324 40.1357536315918 15.3172397613525 41.1610527038574 32.4768180847168 25.933479309082 37.8055953979492 24.1324234008789 28.2611427307129 41.488151550293 30.8563175201416 25.7498378753662 29.8412342071533 26.4888858795166 50.4282722473145 26.8488998413086 26.7728710174561 16.3427085876465 43.3383026123047 49.8903961181641 31.3438720703125 37.9303665161133 29.8269596099854 34.1328010559082 24.6759586334229 25.5181331634521 31.6406745910645 21.951301574707 68.6438064575195 23.8945713043213 54.2285118103027 50.5339508056641 33.6930770874023 21.1892032623291 36.1223030090332

ENSG00000173692.11 109.245094299316 70.6472930908203 61.7842407226562 66.3781890869141 64.9140014648438 100.237373352051 80.2835998535156 57.6501159667969 52.6507148742676 81.5213394165039 73.9470825195312 78.4512557983398 58.2758903503418 91.6432876586914 59.8554267883301 87.3078918457031 50.1255874633789 67.9596710205078 99.61376953125 80.5168075561523 39.5631866455078 111.852363586426 52.8023872375488 70.2674865722656 162.455474853516 118.519058227539 106.219284057617 46.2731475830078 42.2109375 85.3278579711914 63.8376502990723 58.683422088623 52.4254302978516 94.8349456787109 59.7838592529297 41.3973655700684 84.1503295898438 79.7118530273438 83.2181701660156 87.305549621582 72.6497421264648 49.4569053649902 74.1655807495117 34.2275886535645 36.2078971862793 55.4794425964355 76.6874237060547 69.011116027832 68.8595886230469 79.4584121704102 73.0706329345703 42.5869789123535 65.0818405151367 67.5995407104492 86.1899490356445 95.9301834106445 65.8300094604492 67.7056579589844 53.3405685424805 88.8143768310547 63.2328834533691 56.0568809509277 103.624519348145 59.0978698730469 57.6924743652344 52.3464584350586 97.1325912475586 86.5245666503906 46.2555618286133 56.3792381286621 60.6187286376953 95.2354049682617 62.3794975280762 103.517318725586 166.635360717773 132.741271972656 84.9388122558594 75.3584671020508 94.718620300293 54.8834381103516 54.1162528991699 73.4758682250977 118.044677734375 58.7033500671387 37.0114402770996 65.3713989257812 94.9722213745117 97.705451965332 43.3520736694336 33.178539276123 49.5265693664551 71.1295318603516 76.7310409545898 101.78010559082 29.0407276153564 49.9222221374512 66.1051025390625 38.3653678894043 69.744758605957 58.3343544006348 103.158416748047 59.1064643859863 46.4518280029297 62.967700958252 90.6041030883789 70.2454299926758 49.9758148193359 53.7564086914062 56.5319328308105 103.380210876465 66.8358612060547 80.1131286621094 60.3857307434082 73.9812240600586 44.8075675964355 104.072456359863 48.5111999511719 77.0859680175781 60.4482421875 56.6142616271973 47.0389785766602 34.3525123596191 54.7580490112305 69.1726989746094 88.4142990112305 73.6159362792969 79.1988983154297 29.7038631439209 57.9027824401855 127.092552185059 62.8764457702637 51.6422882080078 43.3415184020996 36.8880195617676 103.241806030273 99.1934585571289 63.4248275756836 91.2466583251953 77.9407196044922 52.6876335144043 49.0078887939453 62.8952140808105 50.9121971130371 34.6342887878418 88.8535919189453 78.4249572753906 45.2564697265625 66.9632186889648 61.7405204772949 102.063209533691 67.4403076171875 55.9014511108398 70.2090911865234 49.7721672058105 60.8465614318848 50.1168937683105 69.9335479736328 65.9056854248047 39.9157333374023 62.8369827270508 58.3024711608887 83.8111190795898 31.6489315032959 82.9555892944336 67.6884384155273 25.9967346191406 56.2335891723633 39.5717849731445 46.8611907958984 41.4696311950684 221.937072753906 44.5085563659668 111.975692749023 52.3209457397461 109.161094665527 48.8331260681152 50.3020973205566 55.1203346252441 83.5685729980469 90.3979949951172 34.5206451416016 48.6378326416016 130.318222045898 85.8141632080078 40.878360748291 135.739898681641 44.222770690918 90.3021926879883 131.501052856445 63.8626441955566 66.2871246337891 126.405426025391 81.0994262695312 63.5004768371582 84.9236145019531 69.8377075195312 49.7736396789551 99.1268997192383 46.0703125 49.1771469116211 38.2162971496582 55.1611862182617 88.5527801513672 62.2658042907715 46.1806678771973 47.0988502502441 291.412811279297 31.6152095794678 36.1906547546387 46.8545989990234 63.5280227661133 47.4443588256836 53.7549438476562 94.3698043823242 80.0202255249023 77.6971435546875 34.0985221862793 55.1860847473145 188.078384399414 70.6415710449219 119.409980773926 148.18212890625 71.6797866821289 100.784164428711 56.3885116577148 80.3428039550781 77.7108764648438 68.9813613891602 98.145751953125 70.798225402832 45.4513092041016 81.1125030517578 95.7851181030273 63.0009765625 120.16382598877 62.3838119506836 43.4854125976562 77.6699905395508 70.7672729492188 31.7346515655518 59.3690605163574 58.2598114013672 145.993759155273 60.8582611083984 91.8286056518555 36.9363327026367 59.6077041625977 55.1511993408203 65.4641952514648 38.0724792480469 47.2948760986328 55.1010932922363 183.824661254883 86.8155670166016 76.0856246948242 62.9267387390137 53.0853691101074

ENSG00000175166.15 230.26969909668 138.364303588867 108.881858825684 157.412002563477 101.187026977539 228.974136352539 167.662643432617 88.1827087402344 122.176803588867 84.5942001342773 176.38850402832 174.329956054688 111.93775177002 208.199081420898 91.3942031860352 315.210388183594 97.3600158691406 215.851486206055 178.787475585938 149.057403564453 85.8084945678711 410.655548095703 102.580139160156 141.441818237305 163.675964355469 146.728958129883 576.912658691406 48.8711853027344 82.9555282592773 139.939163208008 118.867973327637 112.042098999023 55.8831100463867 105.932151794434 66.324348449707 91.7794418334961 178.819198608398 94.5475158691406 150.866333007812 253.790573120117 128.336151123047 87.7738800048828 80.2919769287109 151.429641723633 58.9999580383301 70.7610244750977 262.611358642578 123.325286865234 91.2482147216797 126.950485229492 101.310180664062 119.35668182373 184.405319213867 159.179000854492 183.734451293945 113.487281799316 347.373931884766 112.816436767578 109.445793151855 124.42032623291 191.357040405273 109.664192199707 318.420227050781 87.3426284790039 106.713768005371 90.1588516235352 160.472259521484 111.296371459961 118.44620513916 124.790237426758 113.391510009766 326.468078613281 86.1158294677734 222.577117919922 322.910736083984 572.140441894531 162.432159423828 169.367965698242 180.167221069336 79.8328247070312 101.977676391602 94.9003143310547 194.298049926758 146.144454956055 73.0528335571289 151.972869873047 229.348114013672 207.249145507812 65.7457046508789 128.573455810547 76.4656524658203 165.954498291016 201.498062133789 187.397750854492 38.1942367553711 75.8094177246094 162.667526245117 84.1459121704102 129.99169921875 103.774810791016 121.988609313965 111.226623535156 77.4631881713867 121.568153381348 179.542861938477 233.699966430664 113.574829101562 61.9355697631836 93.3734283447266 126.685424804688 119.965858459473 183.592956542969 125.904319763184 109.671501159668 93.4228134155273 83.3346633911133 152.385147094727 77.5628356933594 84.4065475463867 95.9148788452148 94.7057952880859 42.663459777832 86.8212585449219 195.906326293945 158.860000610352 62.6472854614258 130.432510375977 81.9231033325195 127.282691955566 123.22176361084 106.586730957031 66.4954452514648 59.4103813171387 165.029251098633 476.393402099609 226.373001098633 138.896057128906 239.992553710938 152.355438232422 55.2053833007812 117.660499572754 129.739547729492 87.1956558227539 80.7470855712891 237.552505493164 229.099746704102 96.842643737793 159.096115112305 96.8113327026367 124.708709716797 203.845809936523 108.024978637695 133.224014282227 79.5632476806641 119.563842773438 145.383605957031 99.9991302490234 143.792251586914 78.890495300293 142.383773803711 113.247894287109 161.546676635742 27.88134765625 160.917831420898 93.1308288574219 59.7500457763672 121.272979736328 48.5750007629395 91.1778411865234 129.046844482422 371.135192871094 100.018577575684 492.309295654297 103.955207824707 145.817962646484 73.1305084228516 122.037704467773 132.531616210938 165.021209716797 335.092590332031 15.6979207992554 64.2027587890625 147.186126708984 136.426483154297 87.6546936035156 140.094375610352 92.9349822998047 280.561859130859 254.439041137695 73.4169311523438 111.927757263184 369.078002929688 113.583198547363 106.426834106445 119.938896179199 140.227508544922 113.758071899414 164.038604736328 80.3446502685547 26.7084674835205 78.8757781982422 140.901458740234 163.443252563477 122.412292480469 126.389709472656 44.7081069946289 273.209869384766 69.6697845458984 88.510871887207 222.561676025391 62.9250411987305 97.1874084472656 105.622268676758 84.6667251586914 205.817108154297 127.761131286621 45.0559196472168 114.23112487793 189.385406494141 122.801208496094 211.24284362793 557.651306152344 195.444305419922 205.619140625 83.0517807006836 120.17066192627 173.203750610352 101.398956298828 113.586807250977 109.339889526367 130.567901611328 100.206352233887 115.117340087891 109.167327880859 123.429351806641 109.369041442871 92.3488464355469 157.949447631836 98.1282196044922 55.3938331604004 169.593185424805 126.388854980469 107.700408935547 126.515663146973 148.685958862305 80.2598648071289 117.636650085449 76.5218124389648 86.3724822998047 173.017105102539 90.7774810791016 141.608489990234 257.253631591797 115.205940246582 160.318420410156 87.9417190551758 127.318168640137

ENSG00000108344.13 104.52555847168 106.891120910645 72.1646499633789 130.607055664062 104.313163757324 149.240783691406 69.929084777832 44.234001159668 75.7800598144531 80.5128707885742 130.499526977539 112.82893371582 102.724601745605 71.8517303466797 128.938858032227 82.0666427612305 124.683670043945 90.5670471191406 106.307769775391 99.2928924560547 64.1083984375 98.6316757202148 56.9333534240723 131.855712890625 103.466018676758 263.678405761719 209.53662109375 38.2467727661133 60.1140785217285 75.7880096435547 57.8743629455566 79.6887741088867 104.74193572998 128.143951416016 75.9256134033203 67.0815200805664 130.740875244141 63.7150001525879 91.4627304077148 121.420799255371 107.645637512207 69.3390960693359 90.583366394043 119.982429504395 47.2962760925293 93.7881393432617 139.851776123047 81.6686248779297 118.091499328613 122.90064239502 84.4081039428711 191.701705932617 100.995162963867 106.05345916748 108.27522277832 95.6536483764648 1117.81628417969 112.608833312988 74.0694427490234 85.2266159057617 95.6438751220703 116.479560852051 170.381713867188 69.7063217163086 107.11792755127 113.45947265625 113.518081665039 146.984008789062 123.266143798828 82.8824615478516 69.5398712158203 132.904708862305 80.5229644775391 131.191696166992 151.478805541992 177.870559692383 113.887512207031 130.947174072266 181.772521972656 71.8183670043945 70.4291152954102 56.0724296569824 131.123046875 60.5396270751953 45.7359237670898 92.2475662231445 104.036178588867 132.368545532227 65.8401336669922 70.3678359985352 33.2305946350098 98.4555892944336 153.073593139648 155.143905639648 19.9935703277588 67.6488952636719 100.647994995117 61.7039184570312 90.824577331543 65.5865249633789 118.448486328125 94.8583755493164 45.6707229614258 82.4714202880859 95.397705078125 78.0757827758789 77.4631500244141 91.3762817382812 66.1972885131836 108.304328918457 79.1729888916016 135.813171386719 63.3631629943848 117.90266418457 57.8130073547363 54.2450637817383 97.0178070068359 133.183151245117 64.2990341186523 62.2871742248535 79.208137512207 60.4375648498535 79.8985977172852 110.142875671387 109.084701538086 78.1936187744141 67.4480056762695 57.9341659545898 92.534049987793 100.761238098145 49.1749267578125 60.9664192199707 61.2972869873047 85.9341735839844 88.2207260131836 163.216232299805 76.7613983154297 124.774528503418 95.9643402099609 95.6503982543945 72.1328735351562 87.8535079956055 101.681610107422 65.8829803466797 133.798721313477 101.511329650879 85.8259887695312 136.412750244141 74.7496643066406 114.753662109375 145.515899658203 54.5195693969727 78.4179916381836 73.0035552978516 95.8400344848633 86.3533096313477 62.9467964172363 130.529708862305 53.6903343200684 89.3325347900391 111.778541564941 97.5120620727539 47.4298667907715 115.256721496582 59.9227027893066 65.8787002563477 71.3022155761719 18.2451152801514 96.5636749267578 83.7538299560547 178.555557250977 53.6247024536133 224.111190795898 74.2960433959961 222.667984008789 114.806335449219 123.571044921875 253.229919433594 156.38996887207 92.2533416748047 11.940505027771 127.012084960938 95.7842102050781 156.818618774414 92.3266220092773 120.941886901855 86.4836654663086 80.1488189697266 200.866836547852 65.2932205200195 74.9898300170898 150.600448608398 90.375129699707 68.5669784545898 83.6678848266602 88.1491165161133 98.6663055419922 104.667724609375 65.4098892211914 20.9557781219482 82.3721084594727 122.807197570801 119.398010253906 89.8297424316406 69.1953125 41.865779876709 220.725616455078 64.2332305908203 58.3104286193848 267.777160644531 87.1227722167969 66.1102905273438 89.0437393188477 193.226440429688 114.56298828125 103.139625549316 45.5222358703613 95.2316284179688 221.556137084961 73.7940826416016 120.815040588379 167.177993774414 45.7617225646973 83.66162109375 54.1172294616699 72.57080078125 118.628082275391 103.518226623535 144.000854492188 65.4632720947266 66.3106842041016 90.6300277709961 178.23762512207 100.67406463623 86.3903884887695 78.7155456542969 109.808921813965 113.355194091797 65.269889831543 75.9512100219727 108.600639343262 114.748886108398 111.850791931152 80.447135925293 102.199935913086 67.9232482910156 50.9608535766602 122.780601501465 100.426918029785 172.070663452148 70.5049438476562 88.2704315185547 149.954437255859 219.634048461914 142.584136962891 68.1946182250977 129.918975830078

ENSG00000159352.14 104.974540710449 112.907356262207 156.024063110352 201.153411865234 134.998245239258 329.190979003906 542.598083496094 114.774337768555 190.883117675781 113.639495849609 266.5341796875 138.894821166992 197.980255126953 132.320877075195 175.177062988281 158.838119506836 187.725036621094 139.022491455078 105.502296447754 154.381896972656 114.740989685059 145.916580200195 109.958229064941 154.579818725586 550.759643554688 124.340759277344 222.294937133789 158.89826965332 200.661422729492 174.581893920898 117.166763305664 161.052139282227 177.941329956055 139.203475952148 283.513092041016 130.530014038086 251.742599487305 112.953674316406 122.630149841309 218.280532836914 277.875701904297 105.016296386719 242.788192749023 331.464019775391 171.887466430664 149.38703918457 207.76921081543 103.898910522461 293.579986572266 131.096862792969 158.54020690918 641.372985839844 157.927474975586 145.364181518555 157.126937866211 213.859649658203 262.028564453125 106.242736816406 124.18522644043 180.291381835938 200.479629516602 139.173248291016 207.90202331543 161.158187866211 204.830001831055 138.382766723633 248.44384765625 430.150238037109 128.018371582031 152.516067504883 331.082733154297 146.625732421875 212.866119384766 212.85578918457 286.655609130859 113.274482727051 202.981384277344 134.889358520508 211.519012451172 159.049865722656 240.91813659668 86.8019714355469 154.162979125977 236.646697998047 128.610961914062 131.296813964844 129.176910400391 210.495452880859 150.073715209961 149.846893310547 73.3232421875 212.541397094727 287.701538085938 175.208908081055 20.1683197021484 159.827285766602 167.719100952148 117.146919250488 139.637390136719 157.196975708008 274.6513671875 167.020156860352 116.406387329102 180.06916809082 235.172393798828 289.586669921875 260.083160400391 236.347625732422 184.904418945312 311.858276367188 146.334548950195 239.470626831055 105.022071838379 192.250473022461 114.745094299316 125.102958679199 192.754455566406 339.030670166016 157.078842163086 167.680267333984 143.957000732422 116.501045227051 105.346206665039 108.545684814453 209.49787902832 150.981735229492 142.738876342773 115.402038574219 123.488677978516 208.926223754883 134.269638061523 105.787216186523 125.148910522461 86.2593231201172 98.4244079589844 141.406143188477 126.064712524414 196.249816894531 187.660919189453 183.780197143555 146.799102783203 139.05973815918 216.568023681641 116.2060546875 101.689392089844 140.646774291992 113.684783935547 117.98616027832 119.814979553223 217.487060546875 261.354797363281 168.000106811523 109.980102539062 103.655502319336 190.983062744141 287.156524658203 97.1412887573242 146.56999206543 81.6036682128906 128.179138183594 134.403350830078 291.433410644531 225.431564331055 349.405517578125 137.622665405273 92.9591751098633 214.772506713867 31.085542678833 147.309509277344 157.708648681641 168.637344360352 106.435180664062 343.383392333984 138.883743286133 238.479766845703 166.596267700195 109.619323730469 113.555473327637 147.353729248047 172.019439697266 16.6213569641113 205.174621582031 156.509292602539 146.746536254883 188.463821411133 189.28791809082 176.151779174805 197.699829101562 267.399780273438 116.144477844238 103.199127197266 146.754302978516 202.540313720703 138.203735351562 186.499725341797 277.172393798828 149.962585449219 130.375900268555 147.174407958984 47.3290901184082 115.898468017578 235.012802124023 135.899795532227 113.532455444336 95.7773590087891 111.697219848633 138.047988891602 82.9118957519531 89.2921447753906 411.686096191406 177.20751953125 119.058952331543 139.025619506836 185.97917175293 206.875885009766 185.725677490234 88.721809387207 182.232070922852 630.082946777344 281.762756347656 391.429138183594 195.96076965332 98.3401184082031 131.134201049805 96.6891326904297 113.57950592041 227.67431640625 195.603363037109 131.626007080078 99.333137512207 207.171966552734 255.490264892578 132.76139831543 181.231491088867 184.078994750977 187.326278686523 127.546714782715 176.167602539062 168.589340209961 149.66943359375 212.86555480957 98.8974227905273 220.302032470703 156.288543701172 223.720138549805 152.658004760742 100.075424194336 192.331466674805 155.94921875 138.515121459961 217.730895996094 146.36442565918 403.053588867188 332.175231933594 273.756378173828 124.596412658691 177.59065246582

ENSG00000095261.12 48.6424903869629 36.7290573120117 31.4660091400146 43.3864593505859 35.972785949707 50.3711204528809 30.2026844024658 28.9278392791748 24.6231002807617 33.8189239501953 26.2670841217041 29.4697341918945 21.9996719360352 78.5254058837891 33.3033752441406 32.1315650939941 15.9304676055908 33.7160263061523 56.4944725036621 57.4070816040039 22.9637279510498 49.7583045959473 20.1182270050049 38.897274017334 40.7264785766602 30.4803638458252 24.3721714019775 17.7075424194336 30.4352436065674 51.6138076782227 29.1608963012695 27.6523704528809 20.072546005249 46.8160514831543 15.9562797546387 17.1094970703125 37.055850982666 20.911922454834 38.5553359985352 27.8396186828613 56.2000999450684 28.3472461700439 24.1708831787109 38.2627410888672 22.4452075958252 24.4957714080811 36.6452217102051 30.8265361785889 23.8515281677246 22.3194332122803 11.8769998550415 38.2769737243652 20.1205253601074 28.2277507781982 58.2861061096191 29.6150760650635 30.6815280914307 56.7019119262695 31.5677719116211 28.4755210876465 51.3320808410645 48.071647644043 43.1991920471191 34.5766525268555 31.0830268859863 31.8801403045654 32.4320945739746 37.452392578125 16.8836612701416 25.4797630310059 42.5293350219727 42.266414642334 43.5674209594727 89.1808242797852 39.5261192321777 67.2103424072266 29.8799095153809 27.490873336792 36.1251792907715 27.1375293731689 35.4384956359863 28.6234550476074 63.7956352233887 36.1662216186523 16.9515151977539 34.6992683410645 36.2525215148926 26.1566543579102 18.9301223754883 38.5998916625977 19.3425693511963 47.9471168518066 30.6465148925781 28.7826137542725 22.4961223602295 15.0479307174683 23.0256671905518 25.5628070831299 25.805196762085 34.2748146057129 28.0785503387451 36.6574516296387 17.2218647003174 25.0478057861328 53.8988227844238 50.700927734375 36.1110038757324 35.1287651062012 45.4908332824707 60.7176971435547 40.5785217285156 30.7279567718506 39.0422325134277 19.9269256591797 29.8847713470459 51.2983016967773 30.7787170410156 29.2224826812744 29.6586666107178 35.5200805664062 22.4425411224365 25.7137203216553 28.0035209655762 45.7401733398438 63.3264808654785 34.7991676330566 24.3096771240234 18.8772106170654 40.7664413452148 54.1995124816895 22.5969848632812 30.9370288848877 13.7663660049438 36.9627990722656 34.1343460083008 38.1612091064453 30.235279083252 5.43003606796265 25.9095554351807 32.0443115234375 22.8594665527344 36.1422424316406 29.4623889923096 23.5208282470703 35.4691619873047 36.0920791625977 35.9048347473145 40.1942176818848 23.4969577789307 32.4898300170898 36.939510345459 31.0981273651123 26.4461784362793 31.2369174957275 18.0158805847168 32.849178314209 46.7830352783203 33.1114044189453 23.8137378692627 24.5296745300293 29.4367942810059 65.232307434082 14.6592168807983 39.4903030395508 44.7800750732422 18.4636211395264 38.0438537597656 32.9632949829102 28.6334171295166 19.1040859222412 33.7369003295898 21.3490200042725 45.3675918579102 18.8142642974854 15.4424886703491 22.1410331726074 19.8063716888428 31.5584774017334 54.2908973693848 50.1061134338379 48.829906463623 37.5588607788086 26.9127998352051 22.9028987884521 22.0417289733887 30.7093505859375 26.6497001647949 40.0757026672363 30.2886962890625 26.5930042266846 44.8680648803711 46.2945098876953 40.1621284484863 29.8943252563477 45.2302360534668 25.8841018676758 15.5664758682251 44.6886787414551 26.9034461975098 12.9142990112305 20.5222339630127 38.1407699584961 44.4162521362305 36.0849723815918 40.7560539245605 41.9582862854004 52.198860168457 16.8068141937256 38.9001541137695 59.7494583129883 23.6483192443848 26.8127880096436 29.0144214630127 24.1098613739014 39.2810745239258 34.3604393005371 15.4343566894531 20.7464752197266 67.5155258178711 29.5520935058594 73.5929946899414 32.2958030700684 35.0815200805664 36.8976211547852 22.3712272644043 40.4311485290527 49.5744171142578 18.6992301940918 31.5239963531494 34.3250045776367 26.6188926696777 46.4923210144043 36.3267097473145 56.0810661315918 70.3901748657227 19.4581241607666 21.4480400085449 35.3527908325195 36.1231307983398 11.8814125061035 32.6637649536133 31.4954013824463 27.5888233184814 27.213695526123 48.0910606384277 26.1014232635498 25.9978561401367 29.3947849273682 28.3789024353027 23.8576030731201 25.1579742431641 37.4358215332031 49.7057609558105 23.4156837463379 32.1748657226562 23.8760452270508 30.3190574645996

ENSG00000103035.9 75.7018127441406 85.3848571777344 66.6546401977539 86.673095703125 52.2383766174316 124.135772705078 53.185359954834 44.8160858154297 65.027458190918 89.1789779663086 50.4764251708984 131.512466430664 63.8115730285645 141.012313842773 82.0731811523438 150.168594360352 76.1322021484375 87.2627563476562 81.2686386108398 78.9365463256836 55.674503326416 128.722137451172 74.3532028198242 85.0147476196289 85.2005767822266 188.540985107422 181.135452270508 54.0764579772949 43.6589164733887 84.3203430175781 73.6629486083984 75.9854736328125 67.3568496704102 112.536186218262 43.7036552429199 54.3652610778809 97.9684600830078 84.5382537841797 108.762336730957 107.743103027344 83.5862808227539 63.7430458068848 89.0597076416016 40.870418548584 34.6970634460449 72.8638534545898 139.335037231445 75.0746002197266 73.8796768188477 65.5784606933594 74.8405380249023 42.2070236206055 102.659423828125 104.804870605469 72.5427703857422 84.8101196289062 178.168151855469 70.5589218139648 65.7865295410156 71.2930450439453 54.1232757568359 75.8472366333008 66.4069976806641 80.086311340332 86.1320953369141 72.0790405273438 160.762084960938 200.892272949219 70.6948776245117 62.5915145874023 96.6451873779297 118.632369995117 79.9945755004883 70.6580200195312 194.345504760742 122.633804321289 99.241081237793 148.63362121582 98.0987243652344 73.6839294433594 52.0776786804199 61.9883079528809 88.5636520385742 55.6965522766113 50.724365234375 64.7046966552734 81.4874954223633 86.744255065918 43.3630447387695 54.6418914794922 67.3500213623047 80.5042114257812 95.2891616821289 119.358375549316 23.4840888977051 66.1685104370117 58.0147132873535 56.232105255127 118.674491882324 62.182861328125 104.64786529541 94.6769790649414 53.8107147216797 63.135383605957 93.9072341918945 116.818138122559 80.9528045654297 73.6639633178711 141.163681030273 81.5827102661133 58.3861656188965 75.1212844848633 64.1406097412109 76.1352767944336 49.9108047485352 88.2137985229492 60.7761535644531 97.7264938354492 69.4326248168945 85.1639099121094 64.7965469360352 118.43416595459 55.0139312744141 44.1013221740723 78.4143600463867 78.7631683349609 83.019905090332 40.9731369018555 60.0767860412598 103.303230285645 63.7259368896484 60.8686714172363 66.6387634277344 51.4224128723145 68.2498931884766 153.747299194336 96.0649261474609 127.92626953125 83.0866012573242 98.1744155883789 67.5903625488281 96.2252349853516 44.4990844726562 45.6785469055176 76.8829803466797 68.9987030029297 53.2713317871094 50.8487930297852 62.6900291442871 160.660675048828 97.6169967651367 57.3003005981445 52.8899230957031 62.8313941955566 84.6429443359375 40.3162155151367 86.1108551025391 88.6564407348633 45.6844177246094 57.1146125793457 46.7447357177734 86.0602874755859 40.6162796020508 111.569564819336 70.492431640625 51.0274276733398 76.0950927734375 18.3127422332764 94.4159622192383 93.7164535522461 98.4737777709961 47.651741027832 170.841461181641 70.4047012329102 154.418411254883 77.7789688110352 61.1127319335938 44.5826301574707 125.214996337891 86.2885665893555 24.7514457702637 101.640274047852 102.779609680176 94.0080490112305 66.9675674438477 79.2393341064453 103.517707824707 195.719711303711 106.399055480957 63.4209480285645 53.9112854003906 170.30973815918 69.6830978393555 68.7239456176758 95.814567565918 120.327598571777 100.539192199707 75.8974990844727 42.7045402526855 26.9977169036865 68.4016571044922 61.7044372558594 73.3389053344727 75.6569595336914 50.3009757995605 54.9719276428223 193.765441894531 50.0713386535645 38.2214241027832 101.755859375 74.4419097900391 50.5168762207031 75.6279678344727 96.3765487670898 127.027282714844 92.9719314575195 27.2165355682373 66.4961624145508 110.491722106934 70.7910690307617 153.742065429688 189.06462097168 73.9726715087891 93.753173828125 41.0601692199707 52.8688316345215 94.2347412109375 70.1657485961914 97.3457717895508 62.635124206543 62.6856117248535 63.4254760742188 81.6886367797852 66.1562423706055 97.1813049316406 79.4452819824219 52.0982284545898 82.2395782470703 78.3637313842773 61.4079055786133 63.5035514831543 62.8493766784668 213.93603515625 68.3393402099609 88.7313690185547 49.9120674133301 55.5775108337402 51.0059585571289 59.3093032836914 128.176727294922 63.8994026184082 62.9460754394531 152.020660400391 153.758422851562 140.510986328125 61.9586029052734 90.3222045898438

ENSG00000099341.10 154.289306640625 189.341278076172 126.524559020996 169.801773071289 153.717437744141 127.21159362793 190.224136352539 91.1211700439453 330.149200439453 139.20964050293 184.199569702148 145.89778137207 112.790397644043 101.833282470703 124.477821350098 311.172515869141 126.588920593262 90.7194290161133 134.125122070312 146.837539672852 87.3213882446289 207.440963745117 88.6341018676758 111.628814697266 276.218231201172 145.82096862793 191.963485717773 96.1343231201172 104.360801696777 148.781524658203 111.250343322754 121.704406738281 204.861038208008 147.580276489258 73.0453948974609 86.1098785400391 246.740921020508 99.6310424804688 92.147102355957 206.946228027344 141.103561401367 101.191703796387 91.0695877075195 77.2763900756836 62.7169075012207 69.3496780395508 224.587326049805 127.298774719238 89.4884262084961 135.447296142578 146.123550415039 105.123573303223 73.7690505981445 253.374389648438 154.234893798828 198.718902587891 170.002105712891 176.751983642578 127.323219299316 123.140319824219 139.81396484375 170.662796020508 159.605621337891 103.797340393066 639.275451660156 243.765106201172 95.822265625 121.916694641113 83.2127532958984 145.999786376953 154.799530029297 296.573150634766 170.601760864258 136.752716064453 371.529357910156 266.890411376953 117.273582458496 101.222373962402 216.579849243164 130.870574951172 119.201751708984 94.898567199707 167.228515625 164.733062744141 128.4013671875 142.778884887695 99.5062866210938 175.33935546875 108.993316650391 164.411544799805 62.7160301208496 148.554550170898 126.992164611816 153.876403808594 31.6344261169434 98.2619018554688 128.606460571289 79.4020538330078 152.879241943359 108.64436340332 84.829704284668 118.733467102051 93.2697677612305 138.447006225586 210.883331298828 148.440933227539 191.786636352539 195.404907226562 149.542098999023 162.575073242188 145.59162902832 323.826202392578 133.196853637695 129.255844116211 135.459548950195 89.971435546875 205.517593383789 126.582374572754 132.81867980957 88.8028182983398 180.366394042969 170.561584472656 146.063110351562 146.099105834961 111.626831054688 108.030990600586 226.274032592773 71.1206283569336 188.845733642578 158.761459350586 106.663818359375 81.9729919433594 110.640266418457 126.873733520508 533.867126464844 193.098739624023 148.542175292969 149.466110229492 136.54231262207 149.855178833008 147.264190673828 158.410415649414 215.264114379883 86.1838150024414 103.556053161621 327.58837890625 169.772125244141 155.668228149414 100.375648498535 162.137573242188 179.750061035156 175.142364501953 95.2250595092773 98.5253143310547 219.514892578125 83.6251678466797 103.756286621094 113.969047546387 88.1909866333008 124.505935668945 133.614028930664 154.523101806641 73.6361389160156 117.626678466797 103.088470458984 183.042617797852 111.543518066406 26.7868156433105 276.755920410156 183.764358520508 135.811096191406 96.1764450073242 215.620742797852 127.496337890625 146.778625488281 182.958206176758 449.075897216797 164.751647949219 156.220703125 139.412033081055 12.6078996658325 103.41365814209 148.701919555664 247.035339355469 151.108383178711 207.45149230957 124.643775939941 103.782699584961 158.623474121094 117.210510253906 65.948371887207 228.730514526367 130.230422973633 108.82349395752 88.8289260864258 181.976501464844 95.2648391723633 116.324600219727 111.155395507812 53.7251358032227 113.443145751953 175.584045410156 181.62190246582 205.023803710938 129.287353515625 82.6509552001953 240.440963745117 93.9921493530273 101.736343383789 136.591567993164 91.6484680175781 130.082733154297 172.294555664062 203.838394165039 168.932998657227 128.234634399414 65.8686447143555 96.3492202758789 253.503173828125 163.404739379883 166.61962890625 153.674682617188 108.519744873047 127.764236450195 89.016716003418 122.138885498047 120.641510009766 86.7292327880859 146.635009765625 99.4194107055664 106.115768432617 111.610092163086 195.022979736328 172.340911865234 130.859909057617 103.74144744873 199.709197998047 212.961471557617 131.63801574707 41.5020942687988 213.582168579102 104.183166503906 154.933563232422 110.708106994629 201.566970825195 66.1010131835938 80.045654296875 108.933135986328 98.7474136352539 124.208480834961 299.366241455078 159.480331420898 202.892379760742 157.887619018555 140.975509643555 144.309097290039 272.674896240234

ENSG00000101843.17 57.7352600097656 76.0189514160156 42.112247467041 60.776782989502 54.542106628418 111.369491577148 65.6471633911133 63.6239471435547 179.309936523438 63.757926940918 126.233131408691 76.6633605957031 109.859031677246 67.9368896484375 53.0125961303711 72.7694625854492 46.6525039672852 89.6222305297852 73.2231826782227 75.2714233398438 36.7927055358887 74.3926391601562 60.8820838928223 63.9511222839355 91.1775741577148 68.7096481323242 222.536849975586 33.5421485900879 39.619312286377 42.7515182495117 60.1453514099121 72.9939422607422 64.1094207763672 57.9434204101562 26.8354206085205 69.6214752197266 84.7131958007812 52.4234275817871 61.5454788208008 44.8668365478516 53.236385345459 51.3365058898926 93.9072113037109 44.1438903808594 27.3450698852539 93.2407684326172 158.981307983398 49.8534660339355 99.0060043334961 69.7951126098633 46.5299034118652 55.0228042602539 100.420822143555 86.3253402709961 77.9690933227539 69.8598403930664 107.096908569336 68.2959671020508 41.0933685302734 63.1000328063965 56.7559127807617 53.6399917602539 92.9776077270508 77.1204147338867 126.644149780273 105.441879272461 37.7338943481445 49.3222618103027 47.5001106262207 57.415599822998 49.7794914245605 90.5190811157227 32.7012557983398 98.0922088623047 101.224670410156 107.991989135742 96.8696823120117 110.32666015625 47.5650024414062 49.4094543457031 38.2053031921387 52.3242797851562 73.6829071044922 114.164726257324 37.6805610656738 39.0157318115234 48.6772766113281 81.9460678100586 41.8490676879883 62.5952835083008 51.232852935791 55.2023124694824 64.5126953125 73.8512420654297 23.8560752868652 44.6992225646973 45.7084693908691 83.2113876342773 101.392288208008 89.157096862793 58.2665557861328 48.4253082275391 44.494255065918 94.5187301635742 40.5603523254395 95.4070510864258 52.7169990539551 86.4279327392578 77.5495376586914 69.5976867675781 58.688720703125 87.4947204589844 44.0510368347168 53.720458984375 34.3742980957031 91.8222885131836 71.6101455688477 58.1801261901855 40.8371696472168 42.895938873291 65.6987838745117 116.082138061523 58.8117294311523 61.2944793701172 52.2300987243652 41.4728813171387 81.5634841918945 26.7313385009766 149.333419799805 65.0710906982422 38.1071243286133 72.0374755859375 36.9563598632812 74.5593566894531 100.960914611816 125.861595153809 90.5855865478516 75.5992965698242 64.4510803222656 58.7653007507324 44.8312454223633 52.802131652832 62.553165435791 41.8557777404785 76.8862533569336 77.3946304321289 90.9506225585938 65.0411834716797 50.2715187072754 57.5104827880859 102.129211425781 42.8294944763184 43.4910163879395 56.8597755432129 63.9793357849121 66.0828475952148 57.4805297851562 56.8360404968262 39.6594276428223 50.1633377075195 76.5681533813477 109.273857116699 24.7815780639648 94.4170227050781 66.7231216430664 66.3111419677734 48.1217231750488 27.8550090789795 53.1000938415527 64.7550811767578 136.435119628906 42.4272689819336 119.033157348633 36.2330093383789 102.268081665039 71.80078125 36.0835876464844 61.4931373596191 79.5957641601562 74.3381042480469 39.2957420349121 50.2549743652344 81.1993103027344 31.0939922332764 73.8032150268555 65.0277099609375 66.2182693481445 67.845344543457 32.7290840148926 40.7324333190918 65.2050476074219 46.0052680969238 48.4907875061035 49.1350173950195 73.3973159790039 41.7123565673828 65.6036987304688 63.7440185546875 36.1870613098145 25.7345542907715 67.2402954101562 85.0985107421875 63.2589836120605 70.073486328125 54.1040496826172 46.3151054382324 117.716262817383 49.0746383666992 43.0414810180664 113.231567382812 59.3781623840332 43.8833351135254 42.8842163085938 57.1677589416504 60.9369697570801 53.4918594360352 22.9000244140625 45.165210723877 125.916206359863 46.2039451599121 65.4095764160156 63.3803825378418 67.021354675293 78.0809326171875 44.4332466125488 73.1780471801758 46.1372909545898 36.920166015625 42.1594581604004 52.3679733276367 40.510814666748 29.4442253112793 53.468921661377 63.580207824707 66.298698425293 48.7262954711914 90.4055099487305 50.1341590881348 65.4383544921875 17.5045528411865 40.0314903259277 35.1158332824707 77.3351364135742 46.0441017150879 93.5103530883789 24.8604869842529 55.9784202575684 38.3148193359375 61.412914276123 66.1420288085938 79.7219161987305 56.9087295532227 68.8110427856445 98.3884353637695 41.7083854675293 46.9288864135742 90.7746887207031

ENSG00000108671.8 57.2293434143066 51.0278778076172 44.9914169311523 92.279899597168 65.0766220092773 90.2474060058594 54.5937156677246 20.8071117401123 53.4391136169434 56.6703300476074 78.4828338623047 63.3874435424805 51.6871490478516 40.9043464660645 73.2866973876953 93.272590637207 43.0590591430664 53.292350769043 66.9030914306641 67.8614807128906 33.3625335693359 86.986946105957 34.6712036132812 75.9084701538086 74.5775527954102 177.587905883789 110.901596069336 23.5764064788818 42.8856620788574 43.3943939208984 34.0542678833008 43.2455863952637 57.3530807495117 59.8225440979004 49.5425033569336 45.5704879760742 80.4246826171875 44.7264709472656 55.4096603393555 85.6578750610352 54.4662933349609 36.4962997436523 55.2305793762207 64.7901992797852 26.0935192108154 52.2078857421875 52.9408683776855 48.5640640258789 59.017204284668 62.9994621276855 44.640754699707 96.5738525390625 93.2655639648438 75.8803482055664 68.4968490600586 64.0245132446289 120.171356201172 62.5067749023438 41.2683029174805 53.6385650634766 55.9893035888672 58.0381507873535 108.689674377441 41.732608795166 53.3079681396484 36.7728958129883 70.452033996582 72.0239639282227 66.3010940551758 45.5187950134277 53.2312545776367 134.619171142578 50.1975898742676 81.0056610107422 97.8537979125977 123.823760986328 51.5833930969238 65.5221405029297 94.6825408935547 33.9599304199219 37.2585105895996 34.3271636962891 90.7404174804688 36.4052886962891 23.2806987762451 53.9752159118652 66.7441024780273 72.9007110595703 36.7239189147949 45.2330703735352 17.6357345581055 52.4212188720703 78.5664291381836 80.7238235473633 22.926721572876 35.077449798584 63.5767364501953 31.8038387298584 46.7172470092773 37.2732276916504 85.9564666748047 60.7864036560059 26.0848064422607 39.2041473388672 57.4310607910156 46.6586227416992 35.7263412475586 21.7755107879639 40.507453918457 67.3926086425781 45.654613494873 75.723762512207 38.8035697937012 65.9509429931641 32.3313827514648 71.008674621582 46.0591735839844 72.160400390625 29.4971618652344 42.3681640625 35.560417175293 27.8631896972656 35.2813110351562 43.8949317932129 46.9284820556641 55.0702743530273 33.0214920043945 49.7418746948242 38.2341842651367 65.9103698730469 29.028938293457 36.1029968261719 49.072868347168 15.6345825195312 80.2884368896484 101.506988525391 34.2482299804688 94.2992324829102 38.2643928527832 42.4756317138672 46.9370346069336 44.1085357666016 50.6939086914062 32.5037231445312 75.1026229858398 45.0217552185059 38.1288719177246 62.8758888244629 38.1315269470215 73.1977005004883 91.0831832885742 32.9794158935547 50.6916198730469 30.4157562255859 61.1581878662109 52.05908203125 71.7341918945312 48.087646484375 27.9806995391846 49.9598770141602 55.3162078857422 57.6964340209961 28.5368556976318 54.2505302429199 41.0896377563477 26.5238800048828 42.3466491699219 24.3362445831299 55.022144317627 41.9622993469238 121.158187866211 26.1682434082031 221.316833496094 45.4021987915039 137.637817382812 42.6386795043945 36.6251220703125 82.3178176879883 89.5996704101562 49.7302207946777 34.1162452697754 53.1091728210449 83.5024566650391 98.0355987548828 49.0862998962402 63.7397613525391 39.7230567932129 55.2757835388184 107.354354858398 41.7896423339844 48.7561187744141 125.649848937988 44.2940559387207 50.9002838134766 56.3752136230469 72.9584045410156 53.1727905273438 66.2882232666016 32.3655166625977 17.2141437530518 26.8431510925293 55.7145042419434 61.9935188293457 53.0232810974121 34.8414535522461 35.6785316467285 120.249923706055 25.1106014251709 27.4864826202393 82.7481231689453 54.2731437683105 32.6558952331543 43.9704284667969 92.7255783081055 66.8074340820312 50.8943481445312 18.9580898284912 57.6112289428711 153.4130859375 44.827709197998 90.6212310791016 106.608680725098 48.32080078125 61.0460739135742 23.5842571258545 51.2691917419434 68.5205307006836 72.8123016357422 72.755973815918 34.8010902404785 40.0342636108398 55.7741813659668 110.591728210449 40.4216690063477 66.5382995605469 37.8895225524902 45.2075119018555 93.1212310791016 36.8982048034668 41.6378402709961 57.8645629882812 63.2561950683594 86.3739242553711 46.5896186828613 64.9557876586914 37.5898780822754 26.2306861877441 45.9479866027832 25.800142288208 90.1486892700195 30.5420875549316 46.2350959777832 152.41438293457 119.503211975098 66.7732315063477 33.420768737793 61.0572662353516

ENSG00000185627.16 130.19677734375 84.4033813476562 68.6566162109375 57.9178810119629 84.6911926269531 134.75830078125 51.6302909851074 55.535816192627 112.107330322266 75.3422088623047 113.521896362305 80.3627319335938 85.4110565185547 103.818817138672 60.945442199707 136.895294189453 66.6920547485352 71.2619934082031 71.756591796875 88.7272491455078 67.5038909912109 124.998970031738 63.8558959960938 83.7647171020508 47.5091438293457 48.5111656188965 149.754760742188 87.8917922973633 86.8348999023438 65.7019958496094 55.9570007324219 60.412483215332 105.189102172852 88.4582977294922 66.5152969360352 80.2392349243164 87.9410781860352 62.3666114807129 65.4550857543945 89.4399337768555 80.6379699707031 64.1923751831055 95.2382354736328 61.2753944396973 58.5753364562988 73.972770690918 141.873840332031 68.785758972168 76.8980331420898 193.669250488281 96.6159210205078 69.3405380249023 70.8776245117188 109.302993774414 124.043228149414 83.5369186401367 103.80485534668 112.777992248535 59.9949531555176 70.2546997070312 81.0659027099609 133.750228881836 102.14249420166 85.2001342773438 114.877494812012 135.710296630859 70.9125137329102 106.091728210449 78.0292663574219 89.2148513793945 59.1539344787598 135.360916137695 76.3551864624023 90.2318725585938 97.7656021118164 149.757125854492 84.8926315307617 87.6875610351562 117.602821350098 65.2713928222656 73.7181396484375 54.5879592895508 99.8961715698242 93.5576324462891 69.9803466796875 49.0287437438965 56.2402420043945 92.9705200195312 64.7431716918945 118.551971435547 29.2228202819824 67.3537673950195 63.803539276123 130.208297729492 18.0295333862305 39.3865776062012 83.8080444335938 53.3085289001465 88.4411392211914 73.5505447387695 100.168121337891 82.636116027832 75.4311828613281 100.506523132324 76.8416366577148 140.074005126953 87.9443893432617 103.788803100586 83.9006576538086 102.128776550293 58.2068786621094 87.2529907226562 70.788200378418 109.561111450195 58.2922172546387 60.6003265380859 146.417114257812 123.347236633301 78.7351913452148 79.0000228881836 119.355285644531 233.73698425293 42.5756340026855 121.561805725098 97.2189025878906 81.925666809082 143.17692565918 70.4273529052734 73.9004211425781 87.7337265014648 56.5233116149902 69.7258377075195 89.5808334350586 124.604591369629 157.840789794922 88.203483581543 95.7490768432617 76.1871719360352 78.3649597167969 74.1126022338867 39.8605575561523 54.6415176391602 69.4924621582031 45.56005859375 55.6867446899414 72.6096420288086 78.8374710083008 88.9670181274414 66.4944076538086 77.9384689331055 118.356109619141 53.6456527709961 66.3740615844727 55.7643928527832 95.8129959106445 90.0898361206055 106.501174926758 78.3268661499023 59.435417175293 57.4353256225586 94.8996429443359 73.6155319213867 66.1050567626953 126.421630859375 65.7276840209961 91.380485534668 61.6133193969727 19.0707130432129 83.1615753173828 101.241928100586 80.8605651855469 46.8698539733887 120.46671295166 53.3359184265137 68.3995132446289 129.847640991211 101.751808166504 119.70246887207 43.0926132202148 129.152893066406 12.6363677978516 128.295227050781 60.5359077453613 95.6174240112305 73.6123504638672 89.8652725219727 89.630012512207 101.718132019043 106.947067260742 71.8520278930664 67.830322265625 73.0442733764648 82.4736328125 85.5822448730469 76.8457260131836 77.238037109375 92.9182662963867 88.948486328125 60.5045433044434 32.9122772216797 71.0689392089844 97.0496139526367 91.5576324462891 90.6261672973633 65.0650939941406 63.0874214172363 204.096328735352 55.5865707397461 47.4920310974121 123.262359619141 63.2789840698242 59.3351364135742 86.6136169433594 115.04044342041 74.8992538452148 98.7125778198242 51.169002532959 61.3796005249023 39.4798889160156 109.661437988281 122.442832946777 148.851669311523 60.3668098449707 91.0060348510742 51.911018371582 75.6858367919922 79.3986663818359 69.4990844726562 84.1573638916016 57.4987297058105 109.122863769531 95.8671798706055 62.7097854614258 36.4676170349121 57.0249099731445 92.2638473510742 90.9954452514648 59.6973419189453 74.6841354370117 93.5820007324219 90.8537750244141 119.633003234863 94.1903991699219 87.6687164306641 79.2966537475586 53.7931632995605 68.6557464599609 88.1467208862305 67.8758163452148 72.09814453125 118.57430267334 54.886360168457 159.697845458984 107.66780090332 143.115921020508 75.0452194213867 100.55638885498

ENSG00000092010.13 740.24365234375 545.426025390625 277.819519042969 405.673217773438 400.044311523438 382.552368164062 228.873977661133 311.465423583984 1166.62365722656 347.472564697266 396.717254638672 407.553680419922 447.298095703125 184.713851928711 381.073822021484 517.642456054688 220.151992797852 423.771850585938 468.762420654297 568.107360839844 253.609130859375 499.253997802734 284.933959960938 301.443145751953 161.764312744141 244.157211303711 493.224731445312 425.814239501953 301.146881103516 189.78874206543 286.385589599609 406.745147705078 389.895812988281 175.014465332031 264.378936767578 289.112884521484 486.927551269531 419.035308837891 472.727661132812 282.427185058594 305.553070068359 402.818695068359 467.834411621094 252.346054077148 327.916381835938 253.285705566406 468.329803466797 295.391021728516 392.444915771484 269.553680419922 649.413269042969 238.739486694336 245.790771484375 302.623352050781 1053.11596679688 437.667266845703 430.095458984375 454.231475830078 304.260955810547 337.144927978516 399.111694335938 157.343978881836 277.812164306641 246.807815551758 413.285034179688 318.763031005859 282.671112060547 414.105224609375 289.390167236328 414.966644287109 331.583465576172 327.547302246094 247.170654296875 323.540771484375 388.712524414062 374.283111572266 370.933227539062 434.333343505859 490.635131835938 270.846038818359 332.190246582031 421.864685058594 265.369110107422 257.314697265625 336.037017822266 359.160827636719 380.475433349609 280.741394042969 365.404144287109 831.587524414062 146.957122802734 318.024536132812 433.648223876953 351.20751953125 66.0031204223633 269.703521728516 302.939208984375 305.482177734375 358.325103759766 265.713104248047 266.084747314453 293.14453125 377.977172851562 430.995361328125 224.61442565918 634.668701171875 429.506744384766 247.455810546875 369.078063964844 240.133697509766 283.663421630859 500.605133056641 485.596527099609 258.785217285156 330.666198730469 345.227783203125 713.794982910156 181.554168701172 179.023544311523 367.969268798828 488.641143798828 702.152221679688 226.821319580078 321.345306396484 510.739379882812 256.370330810547 504.157562255859 276.628875732422 475.763427734375 254.728561401367 233.144271850586 379.247619628906 121.26700592041 201.923141479492 537.396057128906 502.072845458984 357.015899658203 541.025024414062 314.600280761719 294.843963623047 179.089996337891 404.599365234375 202.608642578125 402.642395019531 504.772216796875 331.943450927734 329.090911865234 441.538360595703 425.785858154297 250.898406982422 439.698944091797 329.611328125 298.579437255859 308.484100341797 500.361236572266 424.736053466797 253.330123901367 247.774383544922 227.026123046875 320.025177001953 205.68376159668 234.074890136719 223.07502746582 478.072418212891 221.874557495117 479.225128173828 411.368804931641 52.0026893615723 140.701782226562 303.587707519531 274.9091796875 255.36833190918 198.995132446289 207.252105712891 306.048309326172 312.328460693359 173.439010620117 332.542114257812 658.170166015625 364.398803710938 61.607723236084 482.134552001953 290.366180419922 344.191223144531 357.586517333984 577.938659667969 287.757385253906 849.468566894531 180.342376708984 411.1181640625 227.762222290039 367.123443603516 422.270874023438 418.410125732422 373.358947753906 325.7841796875 216.291763305664 226.640609741211 302.369476318359 139.417724609375 355.480102539062 388.724945068359 259.435028076172 625.350158691406 381.470153808594 414.368072509766 220.252471923828 206.657684326172 279.262390136719 617.802429199219 254.109497070312 340.166900634766 237.710983276367 290.826293945312 655.643920898438 481.824676513672 249.497894287109 336.158111572266 463.118713378906 434.119903564453 407.896209716797 259.974792480469 352.472045898438 649.242065429688 216.183197021484 436.457824707031 249.684509277344 335.502899169922 314.366790771484 398.172088623047 207.179565429688 305.036254882812 443.351348876953 154.96533203125 316.973052978516 360.869201660156 673.038208007812 186.105056762695 303.496856689453 235.109558105469 363.086059570312 291.179901123047 193.294845581055 358.597320556641 323.578735351562 217.93896484375 357.419097900391 217.525100708008 190.851440429688 374.82861328125 491.891876220703 547.591979980469 293.549591064453 690.299133300781 603.124877929688 366.964202880859 571.993713378906
[truncated: 5,003,221 more chars]
