# Supplementary material for: An Immune-Related Signature Predicts Survival in Patients With Lung Adenocarcinoma
Source: Front Oncol. 2019 Dec 10;9:1314. doi: 10.3389/fonc.2019.01314 (PMC6914845; doi:10.3389/fonc.2019.01314)
Supplement: Supplementary file 6 [file Table_6.doc]

**Table S6. Clinical follow-up information of test set samples.**

A1_OS A2_Event A3_T A4_N A5_M A6_Stage additional_studies additional_studies.additional_study.disease_code additional_studies.additional_study.project_code age_at_initial_pathologic_diagnosis anatomic_neoplasm_subdivision anatomic_neoplasm_subdivision_other bcr_patient_barcode bcr_patient_uuid day_of_form_completion days_to_birth days_to_death days_to_initial_pathologic_diagnosis days_to_last_followup days_to_last_known_alive diagnosis dlco_predictive_percent drugs drugs.drug.bcr_drug_barcode drugs.drug.bcr_drug_uuid drugs.drug.clinical_trail_drug_classification drugs.drug.day_of_form_completion drugs.drug.days_to_drug_therapy_end drugs.drug.days_to_drug_therapy_start drugs.drug.drug_name drugs.drug.measure_of_response drugs.drug.month_of_form_completion drugs.drug.number_cycles drugs.drug.prescribed_dose drugs.drug.prescribed_dose_units drugs.drug.regimen_indication drugs.drug.regimen_indication_notes drugs.drug.regimen_number drugs.drug.route_of_administrations.route_of_administration drugs.drug.therapy_ongoing drugs.drug.therapy_types.therapy_type drugs.drug.therapy_types.therapy_type_notes drugs.drug.total_dose drugs.drug.total_dose_units drugs.drug.tx_on_clinical_trial drugs.drug.year_of_form_completion eastern_cancer_oncology_group egfr_mutation_identified egfr_mutation_performed egfr_mutation_result eml4_alk_translocation_identified eml4_alk_translocation_method eml4_alk_translocation_performed eml4_alk_translocation_result ethnicity follow_ups follow_ups.follow_up.additional_pharmaceutical_therapy follow_ups.follow_up.additional_radiation_therapy follow_ups.follow_up.additional_surgery_locoregional_procedure follow_ups.follow_up.additional_surgery_metastatic_procedure follow_ups.follow_up.bcr_followup_barcode follow_ups.follow_up.bcr_followup_uuid follow_ups.follow_up.day_of_form_completion follow_ups.follow_up.days_to_additional_surgery_locoregional_procedure follow_ups.follow_up.days_to_additional_surgery_metastatic_procedure follow_ups.follow_up.days_to_death follow_ups.follow_up.days_to_last_followup follow_ups.follow_up.days_to_new_tumor_event_after_initial_treatment follow_ups.follow_up.eastern_cancer_oncology_group follow_ups.follow_up.followup_case_report_form_submission_reason follow_ups.follow_up.followup_treatment_success follow_ups.follow_up.karnofsky_performance_score follow_ups.follow_up.lost_follow_up follow_ups.follow_up.month_of_form_completion follow_ups.follow_up.new_neoplasm_event_types.new_neoplasm_event_type follow_ups.follow_up.new_tumor_event_after_initial_treatment follow_ups.follow_up.performance_status_scale_timing follow_ups.follow_up.person_neoplasm_cancer_status follow_ups.follow_up.postoperative_rx_tx follow_ups.follow_up.primary_therapy_outcome_success follow_ups.follow_up.progression_determined_by_list.progression_determined_by follow_ups.follow_up.radiation_therapy follow_ups.follow_up.vital_status follow_ups.follow_up.year_of_form_completion gender histological_type history_of_neoadjuvant_treatment icd_10 icd_o_3_histology icd_o_3_site informed_consent_verified karnofsky_performance_score kras_gene_analysis_performed kras_mutation_found kras_mutation_result location_in_lung_parenchyma month_of_form_completion new_tumor_events.new_tumor_event.additional_pharmaceutical_therapy new_tumor_events.new_tumor_event.additional_radiation_therapy new_tumor_events.new_tumor_event.days_to_new_tumor_event_after_initial_treatment new_tumor_events.new_tumor_event.locoregional_procedure.additional_surgery_locoregional_procedure new_tumor_events.new_tumor_event.locoregional_procedure.days_to_additional_surgery_locoregional_procedure new_tumor_events.new_tumor_event.metastatic_procedure.additional_surgery_metastatic_procedure new_tumor_events.new_tumor_event.metastatic_procedure.days_to_additional_surgery_metastatic_procedure new_tumor_events.new_tumor_event.new_neoplasm_event_types.new_neoplasm_event_type new_tumor_events.new_tumor_event.progression_determined_by_list.progression_determined_by new_tumor_events.new_tumor_event_after_initial_treatment number_pack_years_smoked other_dx patient_id performance_status_scale_timing person_neoplasm_cancer_status post_bronchodilator_fev1_fvc_percent post_bronchodilator_fev1_percent postoperative_rx_tx pre_bronchodilator_fev1_fvc_percent pre_bronchodilator_fev1_percent primary_therapy_outcome_success pulmonary_function_test_performed race_list.race radiation_therapy radiations radiations.radiation.anatomic_treatment_site radiations.radiation.bcr_radiation_barcode radiations.radiation.bcr_radiation_uuid radiations.radiation.course_number radiations.radiation.day_of_form_completion radiations.radiation.days_to_radiation_therapy_end radiations.radiation.days_to_radiation_therapy_start radiations.radiation.measure_of_response radiations.radiation.month_of_form_completion radiations.radiation.numfractions radiations.radiation.radiation_dosage radiations.radiation.radiation_treatment_ongoing radiations.radiation.radiation_type radiations.radiation.radiation_type_notes radiations.radiation.regimen_indication radiations.radiation.regimen_indication_notes radiations.radiation.units radiations.radiation.year_of_form_completion residual_tumor stage_event.ann_arbor.b_symptoms stage_event.ann_arbor.extranodal_involvement stage_event.clinical_stage stage_event.gleason_grading.gleason_score stage_event.gleason_grading.primary_pattern stage_event.gleason_grading.secondary_pattern stage_event.gleason_grading.tertiary_pattern stage_event.igcccg_stage stage_event.masaoka_stage stage_event.pathologic_stage stage_event.psa.days_to_psa stage_event.psa.psa_value stage_event.serum_markers stage_event.system_version stage_event.tnm_categories.clinical_categories.clinical_M stage_event.tnm_categories.clinical_categories.clinical_N stage_event.tnm_categories.clinical_categories.clinical_T stage_event.tnm_categories.pathologic_categories.pathologic_M stage_event.tnm_categories.pathologic_categories.pathologic_N stage_event.tnm_categories.pathologic_categories.pathologic_T stopped_smoking_year tissue_prospective_collection_indicator tissue_retrospective_collection_indicator tissue_source_site tobacco_smoking_history tumor_tissue_site vital_status year_of_form_completion year_of_initial_pathologic_diagnosis year_of_tobacco_smoking_onset

599 Alive T2b N0 M0 Stage IIA NA 76 R-Lower Not Applicable TCGA-55-8205 95fa64b0-91e1-4427-b81a-53c47a053563 17 -28063 Not Applicable 0 33 Not Available Lung Adenocarcinoma Not Available NA TCGA-55-8205-D59042;TCGA-55-8205-D59043;TCGA-55-8205-D59044 0EC2801F-BAED-4EC4-973E-BE336BDA8894;E6B41481-C1F8-465D-8757-E2F2F2B0A4DB;DA27B801-8C82-4033-87DD-101B032FF22E Not Available;Not Available;Not Available 30;30;30 154;154;Not Available 93;93;488 Carboplatin;Alimta;Tarceva Complete Response;Complete Response;Not Applicable 4;4;4 Not Available;Not Available;Not Available Not Available;Not Available;Not Available Not Available;Not Available;Not Available Not Available;Not Available;Not Available Not Applicable;Not Applicable;Not Applicable Not Available;Not Available;Not Available Not Available;Not Available;Not Available NO;NO;YES Chemotherapy;Chemotherapy;Targeted Molecular therapy Not Available;Not Available;Not Available Not Available;Not Available;Not Available Not Available;Not Available;Not Available NO;NO;NO 2014;2014;2014 0 Not Available YES Not Available Not Available Not Available NO Not Available NOT HISPANIC OR LATINO NA YES YES Not Available NO TCGA-55-8205-F59040 324D6FE4-9E52-45A9-82EB-03C088BC06E3 30 Not Available Not Available Not Applicable 599 495 Unknown Scheduled Follow-up Submission Progressive Disease Unknown NO 4 Distant Metastasis YES Not Available WITH TUMOR YES Progressive Disease Convincing Imaging NO Alive 2014 FEMALE Lung Adenocarcinoma- Not Otherwise Specified (NOS) No C34.3 8140/3 C34.3 YES 100 YES NO Not Available Central Lung 6 Not Available Not Available Not Available Not Available Not Available Not Available Not Available Not Available Not Available NO 30 Yes, History of Prior Malignancy 8205 Preoperative TUMOR FREE Not Available Not Available Unknown 73 42 Unknown YES WHITE Unknown NA Distant Recurrence TCGA-55-8205-R59041 78705F45-C5A7-452B-9548-2FA5A83DD7C9 Not Available 30 525 508 Radiographic Progressive Disease 4 14 3500 NO External Not Applicable Not Available Not Available cGy 2014 R0 Not Applicable Not Applicable Not Applicable Not Applicable Not Applicable Not Applicable Not Applicable Not Applicable Not Applicable Stage IIA Not Applicable Not Applicable Not Applicable 7th Not Applicable Not Applicable Not Applicable M0 N0 T2b 2012 YES NO 55 4 Lung Alive 2012 2012 1951

NA T3 N2 M0 Stage IIIA NA Not Available L-Upper Not Applicable TCGA-75-5126 93766482-2f20-4c8f-bbd8-bdd203c37d5a 31 Not Available Not Applicable Not Available Not Available Not Available Lung Adenocarcinoma Not Available NA 1 Not Available NO Not Available Not Available Not Available NO Not Available Not Available NA NO NO NO Not Available TCGA-75-5126-F11746 8ee06ba1-ac92-4ac5-9748-27ba9607d9e7 4 Not Available Not Available Not Applicable Not Available Not Available 1 Not Available Progressive Disease Not Available Not Available 4 Not Available YES Adjuvant therapy WITH TUMOR NO Partial Remission/Response Not Available NO Alive 2011 FEMALE Lung Papillary Adenocarcinoma No C34.1 8260/3 C34.1 YES Not Available NO Not Available Not Available Peripheral Lung 3 Not Available Not Available Not Available Not Available Not Available Not Available Not Available Not Available Not Available Not Available 40 No 5126 Other WITH TUMOR Not Available Not Available Not Available Not Available Not Available Not Available Not Available Not Available Not Available NA R2 Not Applicable Not Applicable Not Applicable Not Applicable Not Applicable Not Applicable Not Applicable Not Applicable Not Applicable Stage IIIA Not Applicable Not Applicable Not Applicable Not Available Not Applicable Not Applicable Not Applicable M0 N2 T3 2007 NO YES 75 4 Lung Alive 2011 2007 Not Available

35 Alive T2 N2 MX Stage IIIA NA 75 L-Upper Not Applicable TCGA-91-6849 953a908d-0993-42f6-853b-512611d19a2c 20 -27676 Not Applicable 0 35 Not Available Lung Adenocarcinoma 66 NA Not Available Not Available YES Not Available Not Available Not Available Not Available Not Available NOT HISPANIC OR LATINO NA Not Available Not Available Not Available Not Available TCGA-91-6849-F32642 7A852ADD-9BFF-47D8-8ACB-A7FAAB892A7C 5 Not Available Not Available Not Applicable 35 Not Available Unknown Scheduled Follow-up Submission Unknown Unknown YES 6 Not Available Unknown Not Available Unknown Unknown Unknown Not Available Unknown Alive 2012 FEMALE Lung Adenocarcinoma- Not Otherwise Specified (NOS) No C34.1 8140/3 C34.1 YES 90 NO Not Available Not Available Not Available 7 Not Available Not Available Not Available Not Available Not Available Not Available Not Available Not Available Not Available Not Available 30 No 6849 Preoperative Not Available 75 86 Not Available 76 88 Not Available YES BLACK OR AFRICAN AMERICAN Not Available NA Not Available Not Applicable Not Applicable Not Applicable Not Applicable Not Applicable Not Applicable Not Applicable Not Applicable Not Applicable Stage IIIA Not Applicable Not Applicable Not Applicable 6th Not Applicable Not Applicable Not Applicable MX N2 T2 1976 NO YES 91 3 Lung Alive 2011 2009 1946

567 Alive T1b N0 MX Stage IA NA 64 R-Lower Not Applicable TCGA-55-7903 77c4dbb2-eceb-4e0d-bcde-63dc817d5f35 10 -23509 Not Applicable 0 19 Not Available Lung Adenocarcinoma 83 NA Not Available Not Available NO Not Available Not Available Not Available NO Not Available NOT HISPANIC OR LATINO NA Not Available Not Available Not Available Not Available TCGA-55-7903-F47840 A3E1AF4A-8377-4442-A98B-0F3218B1E228 29 Not Applicable Not Applicable Not Applicable 567 Not Applicable Not Evaluated Scheduled Follow-up Submission Complete Remission/Response Not Evaluated NO 8 Not Available NO Not Evaluated TUMOR FREE NO Complete Remission/Response Not Available NO Alive 2013 MALE Lung Adenocarcinoma- Not Otherwise Specified (NOS) No C34.3 8140/3 C34.3 YES Not Available NO Not Available Not Available Not Available 3 Not Available Not Available Not Available Not Available Not Available Not Available Not Available Not Available Not Available Not Available 40 No 7903 Not Available TUMOR FREE Not Available Not Available Not Available 63 66 Not Available YES WHITE Not Available NA R0 Not Applicable Not Applicable Not Applicable Not Applicable Not Applicable Not Applicable Not Applicable Not Applicable Not Applicable Stage IA Not Applicable Not Applicable Not Applicable 7th Not Applicable Not Applicable Not Applicable MX N0 T1b Not Available YES NO 55 2 Lung Alive 2012 2011 Not Available

237 Dead T2 N1 M0 Stage IIB NA 59 L-Upper Not Applicable TCGA-55-6979 5af499be-d2b9-4eaf-9a9f-435dccb51917 26 -21780 237 0 Not Available Not Available Lung Adenocarcinoma Not Available NA TCGA-55-6979-D40547;TCGA-55-6979-D40548 F521F7CC-4443-4315-B2C2-B486B17D0DC5;7F10EFEE-013A-4927-8BAD-1683AED5FA99 Not Available;Not Available 22;22 149;149 57;57 Carboplatin;Taxol Clinical Progressive Disease;Clinical Progressive Disease 2;2 Not Available;Not Available Not Available;Not Available Not Available;Not Available Not Available;Not Available Not Applicable;Not Applicable Not Available;Not Available Not Available;Not Available NO;NO Chemotherapy;Chemotherapy Not Available;Not Available Not Available;Not Available Not Available;Not Available NO;NO 2013;2013 Not Available Not Available NO Not Available Not Available Not Available NO Not Available Not Available NA YES YES Not Available NO TCGA-55-6979-F37057 7DAF16E2-34A6-40ED-99FB-8C3F3205DA98 22 Not Available Not Available 237 Not Available 195 Unknown Scheduled Follow-up Submission Progressive Disease Unknown NO 2 Distant Metastasis YES Not Available WITH TUMOR YES Progressive Disease Convincing Imaging NO Dead 2013 FEMALE Lung Adenocarcinoma- Not Otherwise Specified (NOS) No C34.1 8140/3 C34.1 YES Not Available NO Not Available Not Available Not Available 7 Not Available Not Available Not Available Not Available Not Available Not Available Not Available Not Available Not Available Not Available Not Available Yes 6979 Not Available WITH TUMOR Not Available Not Available Not Available Not Available Not Available Not Available Not Available WHITE Not Available NA Not Available Not Applicable Not Applicable Not Applicable Not Applicable Not Applicable Not Applicable Not Applicable Not Applicable Not Applicable Stage IIB Not Applicable Not Applicable Not Applicable 6th Not Applicable Not Applicable Not Applicable M0 N1 T2 Not Available NO YES 55 3 Lung Dead 2011 2006 Not Available

24 Alive T1b N1 M0 Stage IIA NA 62 L-Lower Not Applicable TCGA-86-8074 482eb2a7-6fed-4e4c-b1b6-14d6d869f855 9 -22862 Not Applicable 0 24 Not Available Lung Adenocarcinoma Not Available NA 2 Not Available NO Not Available Not Available Not Available NO Not Available NOT HISPANIC OR LATINO NA Not Available Not Available Not Available Not Available TCGA-86-8074-F41667 69D69C1B-6691-4BAA-A3F5-3E34CBD24E19 26 Not Available Not Available Not Applicable Not Available Not Available Not Available Scheduled Follow-up Submission Not Available Not Available YES 3 Not Available Not Available Not Available Not Available Not Available Not Available Not Available Not Available Not Available 2013 FEMALE Lung Adenocarcinoma- Not Otherwise Specified (NOS) No C34.3 8260/3 C34.3 YES 80 NO Not Available Not Available Peripheral Lung 5 Not Available Not Available Not Available Not Available Not Available Not Available Not Available Not Available Not Available Unknown 40 No 8074 Not Available TUMOR FREE Not Available Not Available Unknown Not Available Not Available Unknown NO WHITE Unknown NA R0 Not Applicable Not Applicable Not Applicable Not Applicable Not Applicable Not Applicable Not Applicable Not Applicable Not Applicable Stage IIA Not Applicable Not Applicable Not Applicable 7th Not Applicable Not Applicable Not Applicable M0 N1 T1b Not Available YES NO 86 2 Lung Alive 2012 2011 1970

862 Alive T2 N0 M0 Stage IB NA 61 L-Upper Not Applicable TCGA-86-8673 bc4c4079-b449-485d-84e4-a40496e563e8 30 -22571 Not Applicable 0 0 Not Available Lung Adenocarcinoma Not Available NA Unknown Not Available Unknown Not Available Not Available Not Available Unknown Not Available NOT HISPANIC OR LATINO NA Not Available;NO Not Available;NO Not Available;YES Not Available;Not Available TCGA-86-8673-F41842;TCGA-86-8673-F59092 4D099A3E-8646-424C-AEF4-394D2DDC653A;7D5C34E4-E4BC-41BC-9704-35161E33F1E6 1;30 Not Applicable;639 Not Applicable;Not Available Not Applicable;Not Applicable 455;862 Not Applicable;636 Not Evaluated;Not Evaluated Scheduled Follow-up Submission;Scheduled Follow-up Submission Complete Remission/Response;Complete Remission/Response Not Evaluated;Not Evaluated NO;NO 4;4 Not Available;Locoregional Recurrence NO;YES Not Evaluated;Not Evaluated TUMOR FREE;TUMOR FREE NO;NO Complete Remission/Response;Complete Remission/Response Not Available;Not Available NO;NO Alive;Alive 2013;2014 MALE Lung Bronchioloalveolar Carcinoma Nonmucinous No C34.1 8252/3 C34.1 YES Unknown Unknown Not Available Not Available Unknown 8 Not Available Not Available Not Available Not Available Not Available Not Available Not Available Not Available Not Available Unknown 54 No 8673 Not Available TUMOR FREE Not Available Not Available Unknown Not Available Not Available Not Available NO WHITE Unknown NA R0 Not Applicable Not Applicable Not Applicable Not Applicable Not Applicable Not Applicable Not Applicable Not Applicable Not Applicable Stage IB Not Applicable Not Applicable Not Applicable 7th Not Applicable Not Applicable Not Applicable M0 N0 T2 Not Available YES NO 86 2 Lung Alive 2012 2011 1968

1429 Alive T2 N0 M0 Stage IB NA FPPP TCGA 59 R-Upper Not Applicable TCGA-44-2656 42ca54fc-c1ae-41cd-bca1-7fe9810db460 8 -21766 Not Applicable 0 582 Not Available Lung Adenocarcinoma 72 NA 0 Not Available Not Available Not Available Not Available Not Available Not Available Not Available NOT HISPANIC OR LATINO NA Not Available;Not Available;YES Not Available;Not Available;YES Not Available;Not Available;Not Available Not Available;Not Available;Not Available TCGA-44-2656-F5294;TCGA-44-2656-F9736;TCGA-44-2656-F39934 ca338d6c-2526-45f1-9b0d-c0a045d0ac8c;f823f92c-9e8e-4393-9381-62cfc40ff094;01F40F24-6888-4940-8620-3E5F531523DD 8;14;6 Not Applicable;Not Available;Not Available Not Applicable;Not Available;Not Available Not Applicable;Not Applicable;Not Applicable 582;749;1429 Not Applicable;Not Available;568 Not Available;Not Available;Not Available Not Available;Not Available;Scheduled Follow-up Submission Complete Remission/Response;Not Available;Complete Remission/Response Not Available;Not Available;Not Available Not Available;Not Available;NO 10;4;2 Not Available;Not Available;New Primary Tumor NO;Not Available;YES Not Available;Not Available;Not Available TUMOR FREE;TUMOR FREE;TUMOR FREE NO;NO;NO Complete Remission/Response;Complete Remission/Response;Complete Remission/Response Not Available;Not Available;Biopsy with Histologic Confirmation NO;NO;NO Alive;Alive;Alive 2010;2011;2013 MALE Lung Adenocarcinoma- Not Otherwise Specified (NOS) No C34.1 8140/3 C34.1 YES Not Available NO Not Available Not Available Not Available 10 Not Available Not Available Not Available Not Available Not Available Not Available Not Available Not Available Not Available Not Available 23 No 2656 Pre-Adjuvant Therapy TUMOR FREE 86 84 Not Available 86 79 Not Available YES WHITE Not Available NA Not Available Not Applicable Not Applicable Not Applicable Not Applicable Not Applicable Not Applicable Not Applicable Not Applicable Not Applicable Stage IB Not Applicable Not Applicable Not Applicable 6th Not Applicable Not Applicable Not Applicable M0 N0 T2 2009 YES NO 44 4 Lung Alive 2010 2009 1994

264 Alive T2 N2 M0 Stage IIIA NA 61 L-Upper Not Applicable TCGA-35-5375 5d2a9a4f-678d-4089-a8c5-81a5cb696629 20 -22628 Not Applicable 0 264 Not Available Lung Adenocarcinoma Not Available NA 1 Not Available Not Available Not Available Not Available Not Available Not Available Not Available NOT HISPANIC OR LATINO NA Not Available Not Available Not Available Not Available TCGA-35-5375-F68920 8B27426D-4A36-4ED5-82E4-1622898D6E17 24 Not Available Not Available Not Applicable Not Available Not Available Not Available Scheduled Follow-up Submission Not Applicable Not Available YES 8 Not Available Not Available Not Available Not Available Not Available Not Available Not Available Not Available Not Available 2015 MALE Lung Adenocarcinoma- Not Otherwise Specified (NOS) No C34.1 8140/3 C34.1 YES 90 Not Available Not Available Not Available Peripheral Lung 5 Not Available Not Available Not Available Not Available Not Available Not Available Not Available Not Available Not Available Not Available 35 No 5375 Post-Adjuvant Therapy TUMOR FREE Not Available Not Available Not Available Not Available Not Available Not Available Not Available WHITE Not Available NA R0 Not Applicable Not Applicable Not Applicable Not Applicable Not Applicable Not Applicable Not Applicable Not Applicable Not Applicable Stage IIIA Not Applicable Not Applicable Not Applicable Not Available Not Applicable Not Applicable Not Applicable M0 N2 T2 2006 NO YES 35 4 Lung Alive 2011 2010 1971

455 Alive T2 N0 M0 Stage IB NA 51 R-Upper Not Applicable TCGA-05-4417 a244a99a-d7cc-4fb1-bc76-b66886481621 22 -18780 Not Applicable 0 455 Not Available Lung Adenocarcinoma Not Available NA Not Available Not Available Not Available Not Available Not Available Not Available Not Available Not Available Not Available NA Not Available Not Available Not Available Not Available TCGA-05-4417-F36502 9B56A013-ECA5-4BE4-BC40-1E4BCAAE0501 31 Not Applicable Not Applicable Not Applicable 455 Not Applicable Not Available Scheduled Follow-up Submission Partial Remission/Response Not Available NO 10 Not Available NO Not Available TUMOR FREE NO Partial Remission/Response Not Available NO Alive 2012 FEMALE Lung Adenocarcinoma Mixed Subtype No C34.1 8255/3 C34.1 YES Not Available Not Available Not Available Not Available Not Available 7 Not Available Not Available Not Available Not Available Not Available Not Available Not Available Not Available Not Available Not Available 56 No 4417 Not Available TUMOR FREE Not Available Not Available Not Available Not Available Not Available Not Available Not Available Not Available Not Available NA R0 Not Applicable Not Applicable Not Applicable Not Applicable Not Applicable Not Applicable Not Applicable Not Applicable Not Applicable Stage IB Not Applicable Not Applicable Not Applicable 6th Not Applicable Not Applicable Not Applicable M0 N0 T2 2008 NO YES 05 4 Lung Alive 2010 2008 1971

0 Alive T2 N2 M1 Stage IV NA 70 R-Lower Not Applicable TCGA-05-4244 34040b83-7e8a-4264-a551-b16621843e28 22 -25752 Not Applicable 0 0 Not Available Lung Adenocarcinoma Not Available NA Not Available Not Available Not Available Not Available Not Available Not Available Not Available Not Available Not Available NA MALE Lung Adenocarcinoma- Not Otherwise Specified (NOS) No C34.3 8140/3 C34.3 YES Not Available Not Available Not Available Not Available Peripheral Lung 7 Not Available Not Available Not Available Not Available Not Available Not Available Not Available Not Available Not Available Not Available 38 No 4244 Not Available TUMOR FREE Not Available Not Available Not Available Not Available Not Available Not Available Not Available Not Available Not Available NA RX Not Applicable Not Applicable Not Applicable Not Applicable Not Applicable Not Applicable Not Applicable Not Applicable Not Applicable Stage IV Not Applicable Not Applicable Not Applicable 6th Not Applicable Not Applicable Not Applicable M1 N2 T2 Not Available NO YES 05 4 Lung Alive 2010 2009 Not Available

310 Alive T2 N0 MX Stage IB NA 66 R-Middle Not Applicable TCGA-91-6831 db0b9e63-4272-4f29-bf0e-1ec0fe79a9d7 19 -24436 Not Applicable 0 39 Not Available Lung Adenocarcinoma Not Available NA Not Available Not Available Not Available Not Available Not Available Not Available Not Available Not Available NOT HISPANIC OR LATINO NA Not Available Not Available Not Available Not Available TCGA-91-6831-F32636 05ae61a2-c054-4273-b50c-4760cbb08a73 5 Not Applicable Not Applicable Not Applicable 310 Not Applicable Unknown Scheduled Follow-up Submission Complete Remission/Response Unknown YES 6 Not Available NO Not Available TUMOR FREE NO Complete Remission/Response Not Available NO Alive 2012 MALE Lung Adenocarcinoma- Not Otherwise Specified (NOS) No C34.2 8140/3 C34.2 YES Not Available NO Not Available Not Available Not Available 7 Not Available Not Available Not Available Not Available Not Available Not Available Not Available Not Available Not Available Not Available Not Available No 6831 Not Available TUMOR FREE Not Available Not Available Not Available Not Available Not Available Not Available NO WHITE Not Available NA Not Available Not Applicable Not Applicable Not Applicable Not Applicable Not Applicable Not Applicable Not Applicable Not Applicable Not Applicable Stage IB Not Applicable Not Applicable Not Applicable 5th Not Applicable Not Applicable Not Applicable MX N0 T2 Not Available NO YES 91 2 Lung Alive 2011 2001 Not Available

1167 Dead T1 N0 M0 Stage IA NA 61 R-Upper Not Applicable TCGA-64-1681 c583fdd1-8cd2-4c15-a23e-0644261f65da 21 -22525 1167 0 Not Available Not Available Lung Adenocarcinoma Not Available NA TCGA-64-1681-D20366 27af4417-3bc7-4992-9965-9f4b6c1f6434 Not Available 21 Not Available 481 Tarceva Not Available 12 Not Available 150 mg RECURRENCE Not Applicable 1 PO NO Chemotherapy Not Available Not Available Not Available Not Available 2011 Not Available Not Available YES Exon 19 Deletion Not Available Not Available Not Available Not Available NOT HISPANIC OR LATINO NA YES YES NO Not Available TCGA-64-1681-F20365 2dd45a93-1ce5-4d03-902f-6cec7a998472 21 Not Available Not Available 1167 Not Available 439 Not Available Additional New Tumor Event Not Available Not Available Not Available 12 Not Available YES Not Available Not Available NO Not Available Not Available NO Dead 2011 FEMALE Lung Adenocarcinoma- Not Otherwise Specified (NOS) No C34.1 8140/3 C34.9 YES 0 YES NO Not Available Not Available 12 Not Available Not Available Not Available Not Available Not Available Not Available Not Available Not Available Not Available Not Available Not Available No 1681 Not Available Not Available Not Available Not Available Not Available Not Available Not Available Not Available Not Available WHITE Not Available NA Distant site TCGA-64-1681-R20367 99c88968-d1fb-474c-bf43-99a06c4386ac 1 21 Not Available 711 Not Available 12 Not Available 3000 NO EXTERNAL BEAM Not Applicable RECURRENCE Not Available cGy 2011 R0 Not Applicable Not Applicable Not Applicable Not Applicable Not Applicable Not Applicable Not Applicable Not Applicable Not Applicable Stage IA Not Applicable Not Applicable Not Applicable 6th Not Applicable Not Applicable Not Applicable M0 N0 T1 1988 NO YES 64 3 Lung Dead 2011 2008 1968

303 Dead T4 N1 M0 Stage IIIB NA 76 R-Lower Not Applicable TCGA-05-4396 9f81c602-8afa-4588-b0b6-6e5a1a128d5a 22 -28094 303 0 Not Available Not Available Lung Adenocarcinoma Not Available NA Not Available Not Available Not Available Not Available Not Available Not Available Not Available Not Available Not Available NA MALE Lung Adenocarcinoma Mixed Subtype No C34.3 8255/3 C34.3 YES Not Available Not Available Not Available Not Available Not Available 7 Not Available Not Available Not Available Not Available Not Available Not Available Not Available Not Available Not Available Not Available 19 Yes 4396 Not Available Not Available Not Available Not Available Not Available Not Available Not Available Not Available Not Available Not Available Not Available NA R0 Not Applicable Not Applicable Not Applicable Not Applicable Not Applicable Not Applicable Not Applicable Not Applicable Not Applicable Stage IIIB Not Applicable Not Applicable Not Applicable 5th Not Applicable Not Applicable Not Applicable M0 N1 T4 1984 NO YES 05 3 Lung Dead 2010 2006 1947

1272 Alive T3 N0 MX Stage IIB NA 54 R-Upper Not Applicable TCGA-95-7039 ae702159-5b6a-41dd-868c-7540f9e1131c Not Available -19981 Not Applicable 0 34 Not Available Lung Adenocarcinoma Not Available NA 1 Not Available Not Available Not Available Not Available Not Available Not Available Not Available NOT HISPANIC OR LATINO NA Not Available;Not Available;NO Not Available;Not Available;NO Not Available;Not Available;NO Not Available;Not Available;Not Available TCGA-95-7039-F15635;TCGA-95-7039-F40734;TCGA-95-7039-F67192 1a1c33a9-1df7-4697-9cac-7d942e1bf067;4C4658AB-3BB7-4D82-B7DF-5CA813F3F854;7EAB8F50-92B9-438E-B11F-A8EF2BCB7807 Not Available;15;31 Not Available;Not Available;Not Available Not Available;Not Available;Not Available Not Applicable;Not Applicable;Not Applicable 34;34;1272 Not Available;Not Available;1258 1;Not Available;Unknown Scheduled Follow-up Submission;Scheduled Follow-up Submission;Additional New Tumor Event Complete Remission/Response;Complete Remission/Response;Progressive Disease Not Available;Not Available;Not Evaluated Not Available;YES;NO 8;3;10 Not Available;Not Available;Locoregional Recurrence Not Available;Unknown;YES Preoperative;Not Available;Not Available Not Available;Not Available;WITH TUMOR NO;NO;NO Complete Remission/Response;Not Available;Complete Remission/Response Not Available;Not Available;Biopsy with Histologic Confirmation;Convincing Imaging;Positive Biomarker(s) NO;NO;NO Alive;Alive;Alive 2011;2013;2014 FEMALE Lung Adenocarcinoma- Not Otherwise Specified (NOS) No C34.1 8140/3 C34.1 YES Not Available Not Available Not Available Not Available Not Available 8 Not Available Not Available Not Available Not Available Not Available Not Available Not Available Not Available Not Available Not Available 34 No 7039 Preoperative TUMOR FREE Not Available Not Available Not Available Not Available Not Available Not Available Not Available WHITE Not Available NA Not Available Not Applicable Not Applicable Not Applicable Not Applicable Not Applicable Not Applicable Not Applicable Not Applicable Not Applicable Stage IIB Not Applicable Not Applicable Not Applicable 7th Not Applicable Not Applicable Not Applicable MX N0 T3 Not Available YES NO 95 2 Lung Alive 2011 2011 Not Available

440 Dead T1b N0 MX Stage IA NA 88 R-Lower Not Applicable TCGA-55-A4DF 362000E4-CF7C-48FC-93CC-8F5994B442EA 25 -32432 Not Applicable 0 47 Not Available Lung Adenocarcinoma 82 NA Not Evaluated Not Available Unknown Not Available Not Available Not Available Unknown Not Available NOT HISPANIC OR LATINO NA NO YES NO NO TCGA-55-A4DF-F59072 B4A1C81E-113A-41E4-8C10-FEFA43BCB273 30 Not Available Not Available 440 Not Available 440 Not Evaluated Scheduled Follow-up Submission Progressive Disease Not Evaluated NO 4 Distant Metastasis YES Not Available WITH TUMOR NO Complete Remission/Response Convincing Imaging NO Dead 2014 MALE Lung Adenocarcinoma- Not Otherwise Specified (NOS) No C34.3 8140/3 C34.3 YES Not Evaluated Unknown Not Available Not Available Unknown 1 Not Available Not Available Not Available Not Available Not Available Not Available Not Available Not Available Not Available NO 60 Yes, History of Synchronous/Bilateral Malignancy A4DF Not Available TUMOR FREE Not Available 58 NO Not Available 47 Complete Remission/Response YES WHITE NO NA Distant Recurrence TCGA-55-A4DF-R59073 64AB51F5-F53A-403A-8BA3-694141F508E7 Not Available 30 440 440 Radiographic Progressive Disease 4 Not Available 3500 NO External Not Applicable Not Available Not Available cGy 2014 R0 Not Applicable Not Applicable Not Applicable Not Applicable Not Applicable Not Applicable Not Applicable Not Applicable Not Applicable Stage IA Not Applicable Not Applicable Not Applicable 7th Not Applicable Not Applicable Not Applicable MX N0 T1b 1984 YES NO 55 3 Lung Alive 2013 2012 Not Available

896 Dead T2b N0 MX Stage IIA NA 56 L-Lower Not Applicable TCGA-MP-A4TE BEBD0025-74E2-451B-93B3-86F82DF43573 2 -20627 896 0 Not Available Not Available Lung Adenocarcinoma Not Available NA Not Evaluated Not Available NO Not Available Not Available Not Available NO Not Available NOT HISPANIC OR LATINO NA YES;Unknown NO;Unknown NO;Not Available Not Available;NO TCGA-MP-A4TE-F41404;TCGA-MP-A4TE-F41405 1239E6E6-49B6-494D-B3DC-33A65B8F4058;DCD8612E-0ACE-4A8A-AD8A-FDBC2CC58B58 18;18 Not Available;Not Available Not Available;Not Available 896;896 Not Available;Not Available 411;845 Not Evaluated;Not Evaluated Additional New Tumor Event;Additional New Tumor Event Progressive Disease;Unknown Not Evaluated;Not Evaluated NO;NO 3;3 Locoregional Recurrence;Distant Metastasis YES;YES Not Evaluated;Not Evaluated WITH TUMOR;WITH TUMOR NO;NO Complete Remission/Response;Complete Remission/Response Convincing Imaging;Convincing Imaging NO;NO Dead;Dead 2013;2013 MALE Lung Adenocarcinoma- Not Otherwise Specified (NOS) No C34.3 8140/3 C34.3 YES Not Evaluated YES YES G12C Peripheral Lung 4 NO NO 226 YES 226 Not Available Not Available Locoregional Recurrence Biopsy with Histologic Confirmation YES 40 No A4TE Not Evaluated WITH TUMOR Not Available Not Available NO Not Available Not Available Complete Remission/Response NO WHITE NO NA RX Not Applicable Not Applicable Not Applicable Not Applicable Not Applicable Not Applicable Not Applicable Not Applicable Not Applicable Stage IIA Not Applicable Not Applicable Not Applicable 7th Not Applicable Not Applicable Not Applicable MX N0 T2b 2010 NO YES MP 4 Lung Dead 2013 2010 1970

614 Alive T2a N0 M0 Stage IB NA 81 Other (please specify) Lingula TCGA-97-A4LX E2B0D873-4942-4483-9A88-D5C6338D0382 7 -29915 Not Applicable 0 272 Not Available Lung Adenocarcinoma 61 NA 1 Not Available NO Not Available Not Available Not Available NO Not Available NOT HISPANIC OR LATINO NA Not Available Not Available Not Available Not Available TCGA-97-A4LX-F57384 81B37883-9051-4643-B671-9899F7A40B07 10 Not Applicable Not Applicable Not Applicable 614 Not Applicable 0 Scheduled Follow-up Submission Complete Remission/Response Unknown NO 3 Not Available NO Other TUMOR FREE NO Complete Remission/Response Not Available NO Alive 2014 MALE Lung Adenocarcinoma Mixed Subtype No C34.1 8255/3 C34.1 YES Not Evaluated YES YES G12A Unknown 3 Not Available Not Available Not Available Not Available Not Available Not Available Not Available Not Available Not Available NO 120 Yes, History of Synchronous/Bilateral Malignancy A4LX Preoperative Unknown 96 90 NO 91 91 Complete Remission/Response YES WHITE NO NA Not Evaluated Not Applicable Not Applicable Not Applicable Not Applicable Not Applicable Not Applicable Not Applicable Not Applicable Not Applicable Stage IB Not Applicable Not Applicable Not Applicable 7th Not Applicable Not Applicable Not Applicable M0 N0 T2a 1985 YES NO 97 3 Lung Alive 2013 2012 1945

522 Alive T2a N2 M0 Stage IIIA NA 82 L-Upper Not Applicable TCGA-49-6745 9c7875ad-ab71-4d48-b2e3-e4c7a46393e9 11 -30133 Not Applicable 0 156 Not Available Lung Adenocarcinoma Not Available NA TCGA-49-6745-D16687 8a687c99-eae5-4635-80db-1d8acc5bcff1 Not Available 23 156 64 Not Available Not Available 9 Not Available Not Available Not Available ADJUVANT Not Applicable 1 IV NO Chemotherapy Not Available Not Available Not Available Not Available 2011 Not Available Not Available YES Not Available Not Available Not Available Not Available Not Available Not Available NA Not Available Not Available Not Available Not Available TCGA-49-6745-F15015 47cc0678-9e26-4402-8fcf-1e549c6cf16d 11 Not Applicable Not Applicable Not Applicable 522 Not Applicable Not Available Scheduled Follow-up Submission Not Available Not Available Not Available 8 Not Available NO Not Available TUMOR FREE YES Not Available Not Available NO Alive 2011 MALE Lung Adenocarcinoma- Not Otherwise Specified (NOS) No C34.1 8260/3 C34.1 YES Not Available YES NO Not Available Not Available 8 Not Available Not Available Not Available Not Available Not Available Not Available Not Available Not Available Not Available Not Available 20 Yes 6745 Not Available TUMOR FREE Not Available Not Available Not Available Not Available Not Available Not Available Not Available WHITE Not Available NA Not Available Not Applicable Not Applicable Not Applicable Not Applicable Not Applicable Not Applicable Not Applicable Not Applicable Not Applicable Stage IIIA Not Applicable Not Applicable Not Applicable 7th Not Applicable Not Applicable Not Applicable M0 N2 T2a Not Available NO YES 49 4 Lung Alive 2011 2011 Not Available

910 Alive T3 N0 M0 Stage IIB NA 70 R-Lower Not Applicable TCGA-62-A472 E499069B-A16A-49E9-941A-E3E9EA62AF25 30 -25893 Not Applicable 0 540 Not Available Lung Adenocarcinoma Not Available NA 1 Not Available NO Not Available Not Available Not Available NO Not Available NOT HISPANIC OR LATINO NA NO YES NO Not Available TCGA-62-A472-F51442 1082EB06-8D5B-4D85-8A83-B2125E571F3E 19 Not Available Not Available Not Applicable 910 290 1 Scheduled Follow-up Submission Partial Remission/Response 80 NO 11 Locoregional Recurrence YES Preoperative WITH TUMOR NO Complete Remission/Response Convincing Imaging NO Alive 2013 MALE Lung Adenocarcinoma- Not Otherwise Specified (NOS) No C34.3 8140/3 C34.3 YES 80 NO Not Available Not Available Central Lung 10 NO YES 290 NO Not Available Not Available Not Available Locoregional Recurrence Biopsy with Histologic Confirmation;Convincing Imaging YES 60 No A472 Preoperative WITH TUMOR Not Available Not Available NO 70 87 Complete Remission/Response YES WHITE NO NA R0 Not Applicable Not Applicable Not Applicable Not Applicable Not Applicable Not Applicable Not Applicable Not Applicable Not Applicable Stage IIB Not Applicable Not Applicable Not Applicable 7th Not Applicable Not Applicable Not Applicable M0 N0 T3 Not Available NO YES 62 2 Lung Alive 2012 2011 Not Available

624 Alive T1a N0 M0 Stage IA NA 66 R-Middle Not Applicable TCGA-97-A4M2 345AEF28-D49B-491B-A337-B1AC55C37D35 7 -24313 Not Applicable 0 197 Not Available Lung Adenocarcinoma 91 NA 1 Not Available NO Not Available Not Available Not Available NO Not Available NOT HISPANIC OR LATINO NA Not Available Not Available Not Available Not Available TCGA-97-A4M2-F57453 C19E7A8D-1092-4915-BC25-8935E50F3DA1 12 Not Applicable Not Applicable Not Applicable 624 Not Applicable Not Evaluated Scheduled Follow-up Submission Complete Remission/Response Not Evaluated NO 3 Not Available NO Not Available TUMOR FREE NO Complete Remission/Response Not Available NO Alive 2014 MALE Lung Adenocarcinoma Mixed Subtype No C34.2 8255/3 C34.2 YES Not Evaluated YES NO Not Available Unknown 3 Not Available Not Available Not Available Not Available Not Available Not Available Not Available Not Available Not Available NO 100 Yes, History of Prior Malignancy A4M2 Preoperative TUMOR FREE 89 103 NO 96 102 Complete Remission/Response YES WHITE NO NA R0 Not Applicable Not Applicable Not Applicable Not Applicable Not Applicable Not Applicable Not Applicable Not Applicable Not Applicable Stage IA Not Applicable Not Applicable Not Applicable 7th Not Applicable Not Applicable Not Applicable M0 N0 T1a Not Available YES NO 97 2 Lung Alive 2013 2012 1972

189 Dead T2 N2 M0 Stage IIIA NA 74 R-Upper Not Applicable TCGA-50-6595 82476d2d-e403-4f6b-8dd6-cc84e3329478 25 -27197 189 0 Not Available Not Available Lung Adenocarcinoma Not Available NA TCGA-50-6595-D44132 5CE76459-2545-4AE1-BFB7-D99A28C8576B Not Available 13 162 123 Carboplatin Clinical Progressive Disease 6 Not Available Not Available Not Available Not Available Not Applicable Not Available Not Available NO Chemotherapy Not Available Not Available Not Available NO 2013 Not Available Not Available YES Not Available Not Available Not Available YES Not Available NOT HISPANIC OR LATINO NA NO NO NO YES TCGA-50-6595-F44127 AEA27241-AF39-4C5C-ADA3-91E25F21073D 13 Not Available 187 189 Not Available 182 Not Available Scheduled Follow-up Submission Progressive Disease Not Available NO 6 Distant Metastasis YES Not Available WITH TUMOR YES Progressive Disease Biopsy with Histologic Confirmation YES Dead 2013 FEMALE Lung Adenocarcinoma- Not Otherwise Specified (NOS) No C34.1 8140/3 C34.1 YES Not Available YES Not Available Not Available Not Available 8 Not Available Not Available Not Available Not Available Not Available Not Available Not Available Not Available Not Available Not Available Not Available No 6595 Not Available WITH TUMOR Not Available Not Available Not Available Not Available Not Available Not Available NO WHITE Not Available NA Primary Tumor Field TCGA-50-6595-R44128 DF7FD391-A020-46D8-8EA6-A937605E3002 Not Available 13 161 123 Radiographic Progressive Disease 6 27 54 NO External Not Applicable Not Available Not Available Gy 2013 Not Available Not Applicable Not Applicable Not Applicable Not Applicable Not Applicable Not Applicable Not Applicable Not Applicable Not Applicable Stage IIIA Not Applicable Not Applicable Not Applicable 6th Not Applicable Not Applicable Not Applicable M0 N2 T2 1979 NO YES 50 3 Lung Dead 2011 2009 Not Available

3305 Alive T2 N0 M0 Stage IB NA Not Available L-Lower Not Applicable TCGA-75-7025 681b1176-9676-44d3-892c-1d5dba1ce25b 11 Not Available Not Applicable Not Available Not Available Not Available Lung Adenocarcinoma Not Available NA 0 Not Available Not Available Not Available Not Available Not Available Not Available Not Available Not Available NA Not Available;YES Not Available;NO Not Available;YES Not Available;YES TCGA-75-7025-F15874;TCGA-75-7025-F72041 68cda392-87e8-466a-9dc5-e7f00f2cb0ba;3D210D11-BA20-4227-95D6-9E77D7C10C7D 11;2 Not Applicable;1481 Not Applicable;2092 Not Applicable;Not Applicable Not Available;3305 Not Applicable;1481 Not Available;Unknown Not Available;Scheduled Follow-up Submission Not Available;Complete Remission/Response Not Available;Unknown Not Available;NO 8;4 Not Available;Locoregional Recurrence;Distant Metastasis NO;YES Not Available;Unknown TUMOR FREE;TUMOR FREE NO;NO Complete Remission/Response;Complete Remission/Response Not Available;Not Available NO;NO Alive;Alive 2011;2015 MALE Lung Adenocarcinoma- Not Otherwise Specified (NOS) No C34.1 8140/3 C34.1 YES Not Available Not Available Not Available Not Available Peripheral Lung 8 Not Available Not Available Not Available Not Available Not Available Not Available Not Available Not Available Not Available Not Available Not Available No 7025 Preoperative TUMOR FREE Not Available Not Available Not Available Not Available Not Available Not Available Not Available Not Available Not Available NA R0 Not Applicable Not Applicable Not Applicable Not Applicable Not Applicable Not Applicable Not Applicable Not Applicable Not Applicable Stage IB Not Applicable Not Applicable Not Applicable 6th Not Applicable Not Applicable Not Applicable M0 N0 T2 1986 NO YES 75 3 Lung Alive 2011 Not Available 1967

1501 Dead T2 N1 M0 Stage IIB NA 61 R-Lower Not Applicable TCGA-MP-A4SY 8CC6BCEC-65DF-46A9-A88C-09762A132857 2 -22448 1501 0 Not Available Not Available Lung Adenocarcinoma 73 NA 0 Not Available NO Not Available Not Available Not Available NO Not Available NOT HISPANIC OR LATINO NA MALE Lung Adenocarcinoma- Not Otherwise Specified (NOS) No C34.3 8140/3 C34.3 YES Not Evaluated NO Not Available Not Available Central Lung 4 YES NO 489 YES 489 Not Available Not Available Locoregional Recurrence Biopsy with Histologic Confirmation YES 40 No A4SY Pre-Adjuvant Therapy TUMOR FREE Not Available 74 NO Not Available 54 Complete Remission/Response YES WHITE NO NA R0 Not Applicable Not Applicable Not Applicable Not Applicable Not Applicable Not Applicable Not Applicable Not Applicable Not Applicable Stage IIB Not Applicable Not Applicable Not Applicable 5th Not Applicable Not Applicable Not Applicable M0 N1 T2 2002 NO YES MP 4 Lung Dead 2013 2002 Not Available

1454 Dead T2 N0 M0 Stage IB NA 65 R-Lower Not Applicable TCGA-62-A46O D7AE8EFB-AA4D-4807-9772-55B0A28CCD5C 29 -24050 1454 0 Not Available Not Available Lung Adenocarcinoma Not Available NA Unknown Not Available NO Not Available Not Available Not Available NO Not Available NOT HISPANIC OR LATINO NA FEMALE Lung Adenocarcinoma- Not Otherwise Specified (NOS) No C34.3 8140/3 C34.3 YES 100 NO Not Available Not Available Central Lung 10 NO NO 943 NO Not Available NO Not Available Locoregional Recurrence;Distant Metastasis Convincing Imaging YES 48 No A46O Preoperative WITH TUMOR Not Available Not Available NO 65 106 Complete Remission/Response YES WHITE NO NA R0 Not Applicable Not Applicable Not Applicable Not Applicable Not Applicable Not Applicable Not Applicable Not Applicable Not Applicable Stage IB Not Applicable Not Applicable Not Applicable 6th Not Applicable Not Applicable Not Applicable M0 N0 T2 Not Available NO YES 62 2 Lung Dead 2012 2006 Not Available

593 Dead T3 N2 M0 Stage IIIA NA 72 L-Upper Not Applicable TCGA-78-7154 d288b69f-1e79-4429-8646-9994b7c54f8f 27 -26481 593 0 Not Available Not Available Lung Adenocarcinoma Not Available NA 1 Not Available NO Not Available Not Available Not Available NO Not Available Not Available NA Not Available Not Available Not Available Not Available TCGA-78-7154-F16951 9a2d2bfa-8f97-4a0f-9f2c-95bdea60e9c7 28 Not Available Not Available 593 Not Available Not Available Not Available Scheduled Follow-up Submission Not Available Not Available Not Available 9 Not Available Not Available Not Available Not Available Not Available Not Available Not Available Not Available Dead 2011 MALE Lung Adenocarcinoma- Not Otherwise Specified (NOS) No C34.1 8140/3 C34.1 YES Not Available NO Not Available Not Available Peripheral Lung 9 Not Available Not Available Not Available Not Available Not Available Not Available Not Available Not Available Not Available Not Available 112.5 No 7154 Preoperative Not Available Not Available Not Available Not Available Not Available Not Available Not Available Not Available WHITE Not Available NA R0 Not Applicable Not Applicable Not Applicable Not Applicable Not Applicable Not Applicable Not Applicable Not Applicable Not Applicable Stage IIIA Not Applicable Not Applicable Not Applicable 6th Not Applicable Not Applicable Not Applicable M0 N2 T3 1993 NO YES 78 4 Lung Dead 2011 2003 1948

731 Dead T2 N1 M0 Stage IIB NA 65 R-Middle Not Applicable TCGA-05-4397 6dfd47d2-831a-4386-9051-f78199a16bb5 22 -23833 731 0 Not Available Not Available Lung Adenocarcinoma Not Available NA Not Available Not Available Not Available Not Available Not Available Not Available Not Available Not Available Not Available NA MALE Lung Adenocarcinoma Mixed Subtype No C34.2 8255/3 C34.2 YES Not Available Not Available Not Available Not Available Not Available 7 Not Available Not Available Not Available Not Available Not Available Not Available Not Available Not Available Not Available Not Available 45 No 4397 Not Available Not Available Not Available Not Available Not Available Not Available Not Available Not Available Not Available Not Available Not Available NA R0 Not Applicable Not Applicable Not Applicable Not Applicable Not Applicable Not Applicable Not Applicable Not Applicable Not Applicable Stage IIB Not Applicable Not Applicable Not Applicable 5th Not Applicable Not Applicable Not Applicable M0 N1 T2 Not Available NO YES 05 2 Lung Dead 2010 2006 Not Available

605 Alive T2b N2 M0 Stage IIIA NA 66 L-Lower Not Applicable TCGA-95-A4VP 9E22A3AF-7F28-4757-8684-ED64A2A27AD1 22 -24130 Not Applicable 0 168 Not Available Lung Adenocarcinoma Not Available NA TCGA-95-A4VP-D40540;TCGA-95-A4VP-D40543 EEC4E284-83E6-48D8-9E22-87A68963B598;B3A1D470-D0D9-4ABF-A3AF-1447CD0096B4 Not Available;Not Available 22;22 126;126 63;63 Carboplatin;Taxol Stable Disease;Stable Disease 2;2 Not Available;Not Available Not Available;Not Available Not Available;Not Available Not Available;Not Available Not Applicable;Not Applicable Not Available;Not Available Not Available;Not Available NO;NO Chemotherapy;Chemotherapy Not Available;Not Available Not Available;Not Available Not Available;Not Available NO;NO 2013;2013 Not Available Not Available NO Not Available Not Available Not Available NO Not Available Not Evaluated NA NO YES Not Available NO TCGA-95-A4VP-F58502 29D06D6D-2BAB-4F87-85B2-F4B369A1C6C4 11 Not Available Not Available Not Applicable 605 216 1 Scheduled Follow-up Submission Stable Disease Not Evaluated NO 4 Distant Metastasis YES Post-Adjuvant Therapy WITH TUMOR YES Stable Disease Biopsy with Histologic Confirmation;Convincing Imaging NO Alive 2014 FEMALE Lung Acinar Adenocarcinoma No C34.3 8550/3 C34.3 YES Not Available NO Not Available Not Available Not Available 2 Not Available Not Available Not Available Not Available Not Available Not Available Not Available Not Available Not Available NO 20 No A4VP Not Available TUMOR FREE Not Available Not Available YES Not Available Not Available Stable Disease NO WHITE NO NA Not Evaluated Not Applicable Not Applicable Not Applicable Not Applicable Not Applicable Not Applicable Not Applicable Not Applicable Not Applicable Stage IIIA Not Applicable Not Applicable Not Applicable 7th Not Applicable Not Applicable Not Applicable M0 N2 T2b 2012 YES NO 95 4 Lung Alive 2013 2012 1972

2261 Alive T2 N0 M0 Stage IB NA 65 L-Upper Not Applicable TCGA-NJ-A4YG 7FD03220-531E-4EF8-BECA-E9703E8C0AE1 26 -23812 Not Applicable 0 1726 Not Available Lung Adenocarcinoma Not Available NA Not Evaluated Not Available Not Available Not Available Not Available Not Available Not Available Not Available Not Evaluated NA Not Available;Not Available Not Available;Not Available Not Available;Not Available Not Available;Not Available TCGA-NJ-A4YG-F50865;TCGA-NJ-A4YG-F70643 C3CF3C86-3DA7-477A-A2DE-B89D114EE5BC;BF940C94-BC39-49DE-A9DF-843E7ED62019 1;27 Not Applicable;Not Applicable Not Applicable;Not Applicable Not Applicable;Not Applicable 1904;2261 Not Applicable;Not Applicable Not Available;Not Available Scheduled Follow-up Submission;Scheduled Follow-up Submission Stable Disease;Stable Disease Not Available;Not Available NO;NO 11;2 Not Available;Not Available NO;NO Not Available;Not Available TUMOR FREE;TUMOR FREE NO;NO Stable Disease;Stable Disease Not Available;Not Available NO;NO Alive;Alive 2013;2015 MALE Lung Adenocarcinoma- Not Otherwise Specified (NOS) No C34.1 8255/3 C34.1 YES Not Evaluated Not Available Not Available Not Available Unknown 4 Not Available Not Available Not Available Not Available Not Available Not Available Not Available Not Available Not Available NO 104 No A4YG Not Evaluated TUMOR FREE Not Available Not Available NO Not Available Not Available Not Applicable NO WHITE NO NA Not Evaluated Not Applicable Not Applicable Not Applicable Not Applicable Not Applicable Not Applicable Not Applicable Not Applicable Not Applicable Stage IB Not Applicable Not Applicable Not Applicable 6th Not Applicable Not Applicable Not Applicable M0 N0 T2 2007 NO YES NJ 4 Lung Alive 2013 2008 1955

2590 Alive T2 N0 M0 Stage IB NA Not Available R-Upper Not Applicable TCGA-75-6206 c4c1469d-752f-42b6-8686-714ad4a8ac97 21 Not Available Not Applicable Not Available Not Available Not Available Lung Adenocarcinoma Not Available NA Not Available Not Available Not Available Not Available Not Available Not Available Not Available Not Available Not Available NA Not Available;Not Available Not Available;Not Available Not Available;Not Available Not Available;Not Available TCGA-75-6206-F15085;TCGA-75-6206-F72036 9fa30583-7256-4ed8-93ac-9cf0f6a928c0;DBEE9981-7853-4690-9B0B-62CBF6625199 21;2 Not Applicable;Not Applicable Not Applicable;Not Applicable Not Applicable;Not Applicable Not Available;2590 Not Applicable;Not Applicable 0;Not Evaluated Not Available;Scheduled Follow-up Submission Complete Remission/Response;Complete Remission/Response Not Available;Not Evaluated Not Available;NO 7;4 Not Available;Not Available NO;NO Adjuvant therapy;Not Available TUMOR FREE;TUMOR FREE NO;NO Complete Remission/Response;Complete Remission/Response Not Available;Not Available NO;NO Alive;Alive 2011;2015 MALE Lung Adenocarcinoma- Not Otherwise Specified (NOS) No C34.1 8140/3 C34.1 YES Not Available Not Available Not Available Not Available Peripheral Lung 7 Not Available Not Available Not Available Not Available Not Available Not Available Not Available Not Available Not Available Not Available 20 No 6206 Not Available TUMOR FREE Not Available Not Available Not Available Not Available Not Available Not Available Not Available Not Available Not Available NA R0 Not Applicable Not Applicable Not Applicable Not Applicable Not Applicable Not Applicable Not Applicable Not Applicable Not Applicable Stage IB Not Applicable Not Applicable Not Applicable 6th Not Applicable Not Applicable Not Applicable M0 N0 T2 1962 NO YES 75 3 Lung Alive 2011 Not Available Not Available

243 Dead T3 N0 MX Stage IIB NA 74 R-Lower Not Applicable TCGA-55-7284 0b15fcb8-1fb7-497e-80b9-d3db66a3c0a2 13 -27087 Not Applicable 0 121 Not Available Lung Adenocarcinoma 89 NA Not Available Not Available Not Available Not Available Not Available Not Available Not Available Not Available NOT HISPANIC OR LATINO NA NO NO NO NO TCGA-55-7284-F61486 411282A6-43D3-468C-8DD9-47C991653F13 30 Not Available Not Available 243 Not Available 231 Not Evaluated Scheduled Follow-up Submission Progressive Disease Not Evaluated NO 6 Distant Metastasis YES Not Available WITH TUMOR NO Complete Remission/Response Convincing Imaging NO Dead 2014 MALE Lung Bronchioloalveolar Carcinoma Nonmucinous No C34.8 8252/3 C34.8 YES Not Available Not Available Not Available Not Available Not Available 6 Not Available Not Available Not Available Not Available Not Available Not Available Not Available Not Available Not Available NO Not Available Yes, History of Synchronous/Bilateral Malignancy 7284 Not Available TUMOR FREE 102 70 Unknown 108 74 Unknown YES WHITE Unknown NA R0 Not Applicable Not Applicable Not Applicable Not Applicable Not Applicable Not Applicable Not Applicable Not Applicable Not Applicable Stage IIB Not Applicable Not Applicable Not Applicable 7th Not Applicable Not Applicable Not Applicable MX N0 T3 Not Available YES NO 55 3 Lung Alive 2012 2011 Not Available

701 Alive T2b N0 M0 Stage IIA NA 54 L-Lower Not Applicable TCGA-86-8280 d8faa3a7-6b3f-4e69-8c88-184e41055bd7 30 -19977 Not Applicable 0 16 Not Available Lung Adenocarcinoma Not Available NA TCGA-86-8280-D66080;TCGA-86-8280-D66081 7B11F24A-04FA-472B-A420-D8E1764C3600;2895FB6B-3FC1-4DFD-B743-1CB915268336 Not Available;Not Available 2;2 129;129 16;16 Cisplatin;Etoposide Complete Response;Complete Response 10;10 Not Available;Not Available Not Available;Not Available Not Available;Not Available Not Available;Not Available Not Applicable;Not Applicable Not Available;Not Available Not Available;Not Available NO;NO Chemotherapy;Chemotherapy Not Available;Not Available Not Available;Not Available Not Available;Not Available NO;NO 2014;2014 Not Available Not Available Not Available Not Available Not Available Not Available Not Available Not Available NOT HISPANIC OR LATINO NA Not Available Not Available Not Available Not Available TCGA-86-8280-F66079 F949DB0E-42F2-40F9-B663-70562177DC57 2 Not Applicable Not Applicable Not Applicable 701 Not Applicable 0 Scheduled Follow-up Submission Complete Remission/Response 100 NO 10 Not Available NO Other TUMOR FREE YES Complete Remission/Response Not Available NO Alive 2014 FEMALE Lung Bronchioloalveolar Carcinoma Nonmucinous No C34.3 8252/3 C34.3 YES Not Available NO Not Available Not Available Peripheral Lung 5 Not Available Not Available Not Available Not Available Not Available Not Available Not Available Not Available Not Available NO Not Available No 8280 Not Available TUMOR FREE Not Available Not Available Unknown Not Available Not Available Complete Remission/Response NO WHITE Unknown NA R0 Not Applicable Not Applicable Not Applicable Not Applicable Not Applicable Not Applicable Not Applicable Not Applicable Not Applicable Stage IIA Not Applicable Not Applicable Not Applicable 7th Not Applicable Not Applicable Not Applicable M0 N0 T2b Not Available YES NO 86 1 Lung Alive 2012 2011 Not Available

1268 Dead T2 N0 M0 Stage IB NA 79 R-Lower Not Applicable TCGA-50-6597 0d66bf6c-eed0-4726-bd5b-3bf6d610b4e0 26 -29195 Not Applicable 0 1015 Not Available Lung Adenocarcinoma Not Available NA Not Available Not Available YES Not Available Not Available Not Available Not Available Not Available NOT HISPANIC OR LATINO NA Not Available Not Available Not Available Not Available TCGA-50-6597-F44134 FC850BDD-7D70-423E-97AE-7FE982CCC259 13 Not Applicable Not Applicable 1268 Not Available Not Applicable Not Available Scheduled Follow-up Submission Complete Remission/Response Not Available NO 6 Not Available NO Not Available TUMOR FREE NO Partial Remission/Response Not Available NO Dead 2013 FEMALE Lung Adenocarcinoma- Not Otherwise Specified (NOS) No C34.3 8140/3 C34.3 YES Not Available YES NO Not Available Not Available 8 Not Available Not Available Not Available Not Available Not Available Not Available Not Available Not Available Not Available Not Available Not Available No 6597 Not Available TUMOR FREE Not Available Not Available Not Available Not Available Not Available Not Available NO WHITE Not Available NA R0 Not Applicable Not Applicable Not Applicable Not Applicable Not Applicable Not Applicable Not Applicable Not Applicable Not Applicable Stage IB Not Applicable Not Applicable Not Applicable 6th Not Applicable Not Applicable Not Applicable M0 N0 T2 Not Available NO YES 50 1 Lung Alive 2011 2007 Not Available

1216 Alive T3 N0 M0 Stage IIB NA 80 R-Upper Not Applicable TCGA-62-8395 5c266025-e590-457c-af86-4c2dc9797267 4 -29311 Not Applicable 0 705 Not Available Lung Adenocarcinoma Not Available NA 0 Not Available YES Not Available Not Available Not Available NO Not Available NOT HISPANIC OR LATINO NA YES NO NO Not Available TCGA-62-8395-F42099 2705FAEA-A87F-4437-8A72-3CD9216FC451 15 Not Available Not Available Not Applicable 1216 395 0 Scheduled Follow-up Submission Progressive Disease 100 NO 5 Locoregional Recurrence YES Preoperative WITH TUMOR NO Complete Remission/Response Convincing Imaging NO Alive 2013 FEMALE Lung Adenocarcinoma Mixed Subtype No C34.1 8255/3 C34.1 YES 100 NO Not Available Not Available Central Lung 7 YES NO 500 NO Not Available NO Not Available Locoregional Recurrence;Distant Metastasis Convincing Imaging YES Not Available No 8395 Preoperative WITH TUMOR Not Available Not Available NO 86 130 Complete Remission/Response YES WHITE NO NA R0 Not Applicable Not Applicable Not Applicable Not Applicable Not Applicable Not Applicable Not Applicable Not Applicable Not Applicable Stage IIB Not Applicable Not Applicable Not Applicable 6th Not Applicable Not Applicable Not Applicable M0 N0 T3 Not Available NO YES 62 1 Lung Alive 2012 2009 Not Available

3940 Alive T4 N0 M0 Stage IIIB NA 71 R-Lower Not Applicable TCGA-78-7149 2de1ba51-eae1-4a54-b7cd-d6d739fdc39d 3 -26285 Not Applicable 0 1093 Not Available Lung Adenocarcinoma Not Available NA 1 Not Available NO Not Available Not Available Not Available NO Not Available Not Available NA Not Available;Not Available Not Available;Not Available Not Available;Not Available Not Available;Not Available TCGA-78-7149-F17123;TCGA-78-7149-F46148 95813dc5-e402-4f0b-a425-2578fff0d0c2;20D89F59-36DA-4A8A-A462-4A3DA7D3CF50 3;23 Not Available;Not Available Not Available;Not Available Not Applicable;Not Applicable 1093;3940 Not Available;Not Available Not Available;Not Available Scheduled Follow-up Submission;Scheduled Follow-up Submission Not Available;Not Available Not Available;Not Available Not Available;NO 10;8 Not Available;Not Available Not Available;Unknown Not Available;Not Available Not Available;Not Available NO;NO Complete Remission/Response;Complete Remission/Response Not Available;Not Available NO;NO Alive;Alive 2011;2013 MALE Lung Adenocarcinoma Mixed Subtype No C34.3 8255/3 C34.3 YES Not Available NO Not Available Not Available Central Lung 10 Not Available Not Available Not Available Not Available Not Available Not Available Not Available Not Available Not Available Not Available 115 No 7149 Preoperative Not Available Not Available Not Available Not Available Not Available Not Available Not Available NO WHITE Not Available NA R0 Not Applicable Not Applicable Not Applicable Not Applicable Not Applicable Not Applicable Not Applicable Not Applicable Not Applicable Stage IIIB Not Applicable Not Applicable Not Applicable 6th Not Applicable Not Applicable Not Applicable M0 N0 T4 Not Available NO YES 78 2 Lung Alive 2011 2002 1950

3759 Alive T1a N0 MX Stage IA NA 39 L-Upper Not Applicable TCGA-49-AARO 5202458B-C38A-47C0-A0BA-5F1A59A762A0 19 -14527 Not Applicable 0 3755 Not Available Lung Adenocarcinoma 103.3 NA 1 Not Available Not Available Not Available Not Available Not Available Not Available Not Available NOT HISPANIC OR LATINO NA YES NO NO Not Available TCGA-49-AARO-F70571 FC43C360-D46F-4775-B16A-C5739011E368 25 Not Available Not Available Not Applicable 3759 3521 3 Scheduled Follow-up Submission Stable Disease 40 NO 2 Locoregional Recurrence YES Post-Adjuvant Therapy WITH TUMOR YES Complete Remission/Response Convincing Imaging YES Alive 2015 FEMALE Lung Papillary Adenocarcinoma No C34.1 8260/3 C34.1 YES 90 Not Available Not Available Not Available Not Available 6 NO NO 1144 YES 3664 Not Available Not Available Locoregional Recurrence Convincing Imaging YES Not Available No AARO Preoperative WITH TUMOR 84.4 83.9 NO 83.6 83.0 Complete Remission/Response YES BLACK OR AFRICAN AMERICAN NO NA R0 Not Applicable Not Applicable Not Applicable Not Applicable Not Applicable Not Applicable Not Applicable Not Applicable Not Applicable Stage IA Not Applicable Not Applicable Not Applicable 6th Not Applicable Not Applicable Not Applicable MX N0 T1a Not Available NO YES 49 2 Lung Alive 2014 2003 Not Available

1369 Alive T1 N0 M0 Stage IA NA 70 R-Upper Not Applicable TCGA-05-4389 a3de401d-91fe-49a2-bb07-81c1a06506e6 22 -25660 Not Applicable 0 1369 Not Available Lung Adenocarcinoma Not Available NA Not Available Not Available Not Available Not Available Not Available Not Available Not Available Not Available Not Available NA Not Available Not Available Not Available Not Available TCGA-05-4389-F36455 A2734A4F-5C1D-4D7B-B01D-01305FF9E917 31 Not Applicable Not Applicable Not Applicable 1369 Not Applicable Not Available Scheduled Follow-up Submission Complete Remission/Response Not Available NO 10 Not Available NO Not Available TUMOR FREE NO Not Applicable Not Available NO Alive 2012 MALE Lung Adenocarcinoma Mixed Subtype No C34.1 8255/3 C34.1 YES Not Available Not Available Not Available Not Available Not Available 7 Not Available Not Available Not Available Not Available Not Available Not Available Not Available Not Available Not Available Not Available 43 No 4389 Not Available TUMOR FREE Not Available Not Available Not Available Not Available Not Available Not Available Not Available Not Available Not Available NA R0 Not Applicable Not Applicable Not Applicable Not Applicable Not Applicable Not Applicable Not Applicable Not Applicable Not Applicable Stage IA Not Applicable Not Applicable Not Applicable 5th Not Applicable Not Applicable Not Applicable M0 N0 T1 Not Available NO YES 05 3 Lung Alive 2010 2005 Not Available

186 Alive T2a N0 MX Stage IB NA 84 L-Lower Not Applicable TCGA-69-7761 77828a90-8e2a-4664-b674-d837e6e34fe4 13 -31041 Not Applicable 0 186 Not Available Lung Adenocarcinoma 80 NA TCGA-69-7761-D29256;TCGA-69-7761-D29253 15c9dc00-5f02-401f-96c4-e5098a9d7f91;827ec9b8-ee79-4f14-8cb1-001249e90c74 Not Available;Not Available 13;13 147;147 84;84 Alimta;Carboplatin Not Available;Not Available 3;3 4;4 1000/100;625/500 mg/mL;mg/mL ADJUVANT;ADJUVANT Not Applicable;Not Applicable 1;1 IV;IV NO;NO Chemotherapy;Chemotherapy Not Available;Not Available Not Available;Not Available Not Available;Not Available Not Available;Not Available 2012;2012 1 Not Available YES Other Not Available Not Available Not Available Not Available NOT HISPANIC OR LATINO NA Not Available Not Available Not Available Not Available TCGA-69-7761-F29252 86590873-3f3a-4004-8f1e-1c36818eb6fd 13 Not Applicable Not Applicable Not Applicable 186 Not Applicable 1 Scheduled Follow-up Submission Not Available Not Available Not Available 3 Not Available NO Adjuvant therapy TUMOR FREE YES Not Available Not Available NO Alive 2012 MALE Lung Acinar Adenocarcinoma No C34.3 8550/3 C34.3 YES Not Available Not Available Not Available Not Available Peripheral Lung 3 Not Available Not Available Not Available Not Available Not Available Not Available Not Available Not Available Not Available Not Available 20 No 7761 Other TUMOR FREE Not Available Not Available Not Available 91 98 Not Available YES WHITE Not Available NA Not Available Not Applicable Not Applicable Not Applicable Not Applicable Not Applicable Not Applicable Not Applicable Not Applicable Not Applicable Stage IB Not Applicable Not Applicable Not Applicable 7th Not Applicable Not Applicable Not Applicable MX N0 T2a 1975 NO YES 69 3 Lung Alive 2012 2011 1955

323 Alive T1a N0 M0 Stage IA NA 70 L-Lower Not Applicable TCGA-91-6828 9536e32d-2707-48d2-a36d-08c521665bb9 15 -25870 Not Applicable 0 38 Not Available Lung Adenocarcinoma 71 NA 2 Not Available Not Available Not Available Not Available Not Available Not Available Not Available NOT HISPANIC OR LATINO NA Not Available Not Available Not Available Not Available TCGA-91-6828-F21014 182ca854-9147-4d4e-b2aa-7b899e591214 8 Not Applicable Not Applicable Not Applicable 323 Not Applicable 2 Scheduled Follow-up Submission Not Available Not Available Not Available 5 Not Available NO Preoperative TUMOR FREE NO Complete Remission/Response Not Available NO Alive 2012 MALE Lung Adenocarcinoma- Not Otherwise Specified (NOS) No C34.3 8140/3 C34.3 YES Not Available NO Not Available Not Available Not Available 7 Not Available Not Available Not Available Not Available Not Available Not Available Not Available Not Available Not Available Not Available Not Available No 6828 Preoperative Not Available Not Available Not Available Not Available Not Available 81 Not Available YES WHITE Not Available NA R0 Not Applicable Not Applicable Not Applicable Not Applicable Not Applicable Not Applicable Not Applicable Not Applicable Not Applicable Stage IA Not Applicable Not Applicable Not Applicable 7th Not Applicable Not Applicable Not Applicable M0 N0 T1a 1993 YES NO 91 3 Lung Alive 2011 2011 Not Available

610 Alive T1 N0 M0 Stage IA NA 77 L-Upper Not Applicable TCGA-67-3771 0df573ee-28f0-4244-b434-09e6ca59fbf0 21 -28406 Not Applicable 0 610 Not Available Lung Adenocarcinoma Not Available NA Not Available Not Available NO Not Available Not Available Not Available NO Not Available NOT HISPANIC OR LATINO NA FEMALE Lung Adenocarcinoma- Not Otherwise Specified (NOS) No C34.1 8140/3 C34.1 YES Not Available NO Not Available Not Available Central Lung 1 Not Available Not Available Not Available Not Available Not Available Not Available Not Available Not Available Not Available Not Available Not Available No 3771 Not Available TUMOR FREE Not Available Not Available Not Available Not Available Not Available Not Available Not Available BLACK OR AFRICAN AMERICAN Not Available NA R0 Not Applicable Not Applicable Not Applicable Not Applicable Not Applicable Not Applicable Not Applicable Not Applicable Not Applicable Stage IA Not Applicable Not Applicable Not Applicable 6th Not Applicable Not Applicable Not Applicable M0 N0 T1 Not Available YES NO 67 4 Lung Alive 2011 2009 Not Available

179 Dead T4 N2 M0 Stage IIIB NA 59 L-Lower Not Applicable TCGA-78-7158 501c987e-d1eb-48a9-89eb-72a5062c90b4 15 -21742 179 0 Not Available Not Available Lung Adenocarcinoma Not Available NA TCGA-78-7158-D16167;TCGA-78-7158-D16978 b9fc15d3-ee12-436e-8bd1-d57382720f61;b2454d2f-cb59-470a-b300-029134f6bb34 Not Available;Not Available 15;29 103;103 54;54 Carboplatin;Paclitaxel Not Available;Not Available 9;9 3;3 Not Available;Not Available Not Available;Not Available ADJUVANT;ADJUVANT Not Applicable;Not Applicable 1;1 IV;IV NO;NO Chemotherapy;Chemotherapy Not Available;Not Available 1220;620 mg;mg Not Available;Not Available 2011;2011 1 Not Available NO Not Available Not Available Not Available NO Not Available Not Available NA Not Available Not Available NO NO TCGA-78-7158-F16166 cd749669-0955-4e60-80d4-d02a208e1f64 15 Not Available Not Available 179 Not Available 132 Not Available Scheduled Follow-up Submission Not Available Not Available Not Available 9 Not Available YES Not Available WITH TUMOR YES Progressive Disease Not Available NO Dead 2011 FEMALE Lung Adenocarcinoma Mixed Subtype No C34.3 8255/3 C34.3 YES Not Available NO Not Available Not Available Peripheral Lung 9 Not Available Not Available Not Available Not Available Not Available Not Available Not Available Not Available Not Available Not Available 14 No 7158 Preoperative WITH TUMOR Not Available Not Available Not Available Not Available Not Available Not Available Not Available WHITE Not Available NA R1 Not Applicable Not Applicable Not Applicable Not Applicable Not Applicable Not Applicable Not Applicable Not Applicable Not Applicable Stage IIIB Not Applicable Not Applicable Not Applicable 6th Not Applicable Not Applicable Not Applicable M0 N2 T4 2005 NO YES 78 4 Lung Dead 2011 2007 1963

839 Alive T2b N1 M0 Stage IIB NA 72 R-Upper Not Applicable TCGA-86-8671 c4cfa3cc-e305-42dd-be57-97efbb891659 30 -26448 Not Applicable 0 18 Not Available Lung Adenocarcinoma Not Available NA TCGA-86-8671-D66090;TCGA-86-8671-D66091 151A219B-0DB6-4158-A30B-35B97CC9A1A9;36EB3446-D9DC-4620-830A-8CE6A214F2DB Not Available;Not Available 2;2 104;104 18;18 Cisplatin;Etoposide Complete Response;Complete Response 10;10 Not Available;Not Available Not Available;Not Available Not Available;Not Available Not Available;Not Available Not Applicable;Not Applicable Not Available;Not Available Not Available;Not Available NO;NO Chemotherapy;Chemotherapy Not Available;Not Available Not Available;Not Available Not Available;Not Available NO;NO 2014;2014 1 Not Available Not Available Not Available Not Available Not Available Not Available Not Available NOT HISPANIC OR LATINO NA Not Available Not Available Not Available Not Available TCGA-86-8671-F66089 97BC43F2-A652-4731-B5BD-37770F5ADB10 2 Not Applicable Not Applicable Not Applicable 839 Not Applicable 0 Scheduled Follow-up Submission Complete Remission/Response 100 NO 10 Not Available NO Other TUMOR FREE YES Complete Remission/Response Not Available NO Alive 2014 FEMALE Lung Papillary Adenocarcinoma No C34.1 8260/3 C34.1 YES 90 Not Available Not Available Not Available Peripheral Lung 8 Not Available Not Available Not Available Not Available Not Available Not Available Not Available Not Available Not Available Unknown Not Available No 8671 Preoperative WITH TUMOR Not Available Not Available Unknown Not Available Not Available Complete Remission/Response NO WHITE Unknown NA R0 Not Applicable Not Applicable Not Applicable Not Applicable Not Applicable Not Applicable Not Applicable Not Applicable Not Applicable Stage IIB Not Applicable Not Applicable Not Applicable 7th Not Applicable Not Applicable Not Applicable M0 N1 T2b Not Available YES NO 86 1 Lung Alive 2012 2012 Not Available

568 Alive T1a N0 M0 Stage IA NA 45 R-Lower Not Applicable TCGA-97-A4M6 31FF69B5-9E58-44DA-8326-BDFC7EE495C4 7 -16764 Not Applicable 0 185 Not Available Lung Adenocarcinoma 92 NA 0 Not Available YES Exon 19 Deletion Not Available Not Available NO Not Available NOT HISPANIC OR LATINO NA Not Available Not Available Not Available Not Available TCGA-97-A4M6-F57434 5B7F6A58-58A0-4D88-A6E1-339DD38CBEC4 11 Not Applicable Not Applicable Not Applicable 568 Not Applicable 0 Scheduled Follow-up Submission Complete Remission/Response Not Evaluated NO 3 Not Available NO Other TUMOR FREE NO Complete Remission/Response Not Available NO Alive 2014 FEMALE Lung Adenocarcinoma Mixed Subtype No C34.3 8255/3 C34.3 YES Not Evaluated YES NO Not Available Unknown 3 Not Available Not Available Not Available Not Available Not Available Not Available Not Available Not Available Not Available NO Not Available No A4M6 Preoperative Unknown 95 116 NO 95 110 Complete Remission/Response YES WHITE NO NA Not Evaluated Not Applicable Not Applicable Not Applicable Not Applicable Not Applicable Not Applicable Not Applicable Not Applicable Not Applicable Stage IA Not Applicable Not Applicable Not Applicable 7th Not Applicable Not Applicable Not Applicable M0 N0 T1a Not Available YES NO 97 1 Lung Alive 2013 2012 Not Available

578 Alive T1 N0 M0 Stage I NA 48 L-Upper Not Applicable TCGA-L4-A4E5 32A7B827-C3F9-4607-AE63-DAE8CAA2BC97 5 -17680 Not Applicable 0 213 Not Available Lung Adenocarcinoma 93 NA 1 Not Available NO Not Available Not Available Not Available NO Not Available NOT HISPANIC OR LATINO NA Not Available Not Available Not Available Not Available TCGA-L4-A4E5-F49031 DC4F62D9-03A2-4A06-A9B0-89FCCD37B4FB 25 Not Applicable Not Applicable Not Applicable 578 Not Applicable 1 Scheduled Follow-up Submission Complete Remission/Response Not Evaluated NO 9 Not Available NO Other TUMOR FREE NO Complete Remission/Response Not Available NO Alive 2013 FEMALE Lung Adenocarcinoma- Not Otherwise Specified (NOS) No C34.1 8140/3 C34.1 YES Not Evaluated NO Not Available Not Available Central Lung 12 Not Available Not Available Not Available Not Available Not Available Not Available Not Available Not Available Not Available NO 33 No A4E5 Preoperative TUMOR FREE 94 82 NO 94 82 Complete Remission/Response YES WHITE NO NA R0 Not Applicable Not Applicable Not Applicable Not Applicable Not Applicable Not Applicable Not Applicable Not Applicable Not Applicable Stage I Not Applicable Not Applicable Not Applicable 7th Not Applicable Not Applicable Not Applicable M0 N0 T1 2012 NO YES L4 4 Lung Alive 2012 2012 1979

44 Alive T2b N0 MX Stage IIA NA 59 R-Upper Not Applicable TCGA-91-A4BC EA0B9461-0D4D-4ADE-A255-1B03DD85E30E 5 -21565 Not Applicable 0 44 Not Available Lung Adenocarcinoma 44 NA Not Evaluated Not Available NO Not Available Not Available Not Available Not Available Not Available NOT HISPANIC OR LATINO NA Not Available Not Available Not Available Not Available TCGA-91-A4BC-F45192 5A4ACA69-D8C5-4169-9815-150180962775 10 Not Available Not Available Not Applicable 44 Not Available Not Evaluated Scheduled Follow-up Submission Unknown Not Evaluated YES 7 Not Available Unknown Not Evaluated Unknown Unknown Unknown Not Available Unknown Alive 2013 MALE Lung Adenocarcinoma- Not Otherwise Specified (NOS) No C34.1 8140/3 C34.1 YES Not Evaluated NO Not Available Not Available Not Available 2 Not Available Not Available Not Available Not Available Not Available Not Available Not Available Not Available Not Available Unknown 30 Yes, History of Synchronous/Bilateral Malignancy A4BC Not Evaluated Unknown Not Available 69 Unknown Not Available Not Available Unknown YES WHITE Unknown NA Not Evaluated Not Applicable Not Applicable Not Applicable Not Applicable Not Applicable Not Applicable Not Applicable Not Applicable Not Applicable Stage IIA Not Applicable Not Applicable Not Applicable 7th Not Applicable Not Applicable Not Applicable MX N0 T2b 2001 YES NO 91 4 Lung Alive 2013 2012 1976

1725 Dead T2 N0 M0 Stage IB NA 54 L-Lower Not Applicable TCGA-62-A46R C497F8C7-2FB5-44D9-BC7C-69CFD0B51B54 29 -20063 1725 0 Not Available Not Available Lung Adenocarcinoma Not Available NA Unknown Not Available NO Not Available Not Available Not Available NO Not Available NOT HISPANIC OR LATINO NA FEMALE Lung Adenocarcinoma Mixed Subtype No C34.3 8255/3 C34.3 YES 100 NO Not Available Not Available Central Lung 10 Not Available Not Available Not Available Not Available Not Available Not Available Not Available Not Available Not Available NO 25 No A46R Preoperative TUMOR FREE Not Available Not Available NO 73 74 Complete Remission/Response YES WHITE NO NA R0 Not Applicable Not Applicable Not Applicable Not Applicable Not Applicable Not Applicable Not Applicable Not Applicable Not Applicable Stage IB Not Applicable Not Applicable Not Applicable 6th Not Applicable Not Applicable Not Applicable M0 N0 T2 Not Available NO YES 62 4 Lung Dead 2012 2006 Not Available

36 Alive T1b N0 MX Stage IA NA 76 L-Lower Not Applicable TCGA-91-8499 54480f58-7e63-4aed-a116-c2c2252e8364 29 -27853 Not Applicable 0 36 Not Available Lung Adenocarcinoma Not Available NA Unknown Not Available YES Not Available Not Available Not Available Not Available Not Available NOT HISPANIC OR LATINO NA Not Available Not Available Not Available Not Available TCGA-91-8499-F37976 A3296482-B65D-46DC-8306-36D9A0CE0CAB 7 Not Applicable Not Applicable Not Applicable 36 Not Applicable Not Available Scheduled Follow-up Submission Unknown Unknown YES 12 Not Available NO Not Available Unknown NO Unknown Not Available NO Alive 2012 FEMALE Lung Adenocarcinoma- Not Otherwise Specified (NOS) No C34.3 8140/3 C34.3 YES Unknown NO Not Available Not Available Unknown 8 Not Available Not Available Not Available Not Available Not Available Not Available Not Available Not Available Not Available NO 90 No 8499 Not Available Unknown 49 Not Available NO Not Available Not Available Unknown YES WHITE NO NA Not Evaluated Not Applicable Not Applicable Not Applicable Not Applicable Not Applicable Not Applicable Not Applicable Not Applicable Not Applicable Stage IA Not Applicable Not Applicable Not Applicable 7th Not Applicable Not Applicable Not Applicable MX N0 T1b Not Available YES NO 91 2 Lung Alive 2012 2011 1945

3674 Alive T2b N0 M0 NA 57 L-Upper Not Applicable TCGA-38-4626 2079155a-d91d-4246-a038-01934a580f32 7 -20903 Not Applicable 0 2595 Not Available Lung Adenocarcinoma 57 NA Not Available Not Available Not Available Not Available Not Available Not Available Not Available Not Available NOT HISPANIC OR LATINO NA Not Available;NO Not Available;NO Not Available;NO Not Available;Not Available TCGA-38-4626-F4507;TCGA-38-4626-F33409 f7d53f3d-25b9-43f5-9d12-f4c38dd72816;11F9E5D4-D903-4AC9-A6CC-3CB66E89CD21 8;26 Not Applicable;Not Available Not Applicable;Not Available Not Applicable;Not Applicable 2595;3674 Not Applicable;2518 Not Available;Not Available Not Available;Scheduled Follow-up Submission Not Available;Complete Remission/Response Not Available;Not Available Not Available;NO 12;7 Not Available;New Primary Tumor NO;YES Not Available;Not Available TUMOR FREE;TUMOR FREE NO;NO Complete Remission/Response;Complete Remission/Response Not Available;Not Available NO;NO Alive;Alive 2010;2012 FEMALE Lung Adenocarcinoma- Not Otherwise Specified (NOS) No C34.1 8140/3 C34.1 YES Not Available Not Available Not Available Not Available Central Lung 12 Not Available Not Available Not Available Not Available Not Available Not Available Not Available Not Available Not Available Not Available 40 No 4626 Not Available TUMOR FREE 68 85 Not Available 71 85 Not Available YES WHITE Not Available NA R0 Not Applicable Not Applicable Not Applicable Not Applicable Not Applicable Not Applicable Not Applicable Not Applicable Not Applicable Discrepancy Not Applicable Not Applicable Not Applicable 6th Not Applicable Not Applicable Not Applicable M0 N0 T2b Not Available NO YES 38 2 Lung Alive 2010 2003 1962

677 Dead T1 N1 MX Stage II NA 63 L-Lower Not Applicable TCGA-49-AAQV 96DFA373-4597-49A5-942E-D9B4DC5D28FC 13 -23370 677 0 Not Available Not Available Lung Adenocarcinoma 95.5 NA 1 Not Available YES Exon 19 Deletion Not Available FISH YES Not Available NOT HISPANIC OR LATINO NA FEMALE Lung Adenocarcinoma Mixed Subtype No C34.3 8255/3 C34.3 YES 90 Not Available Not Available Not Available Not Available 6 NO YES 509 Not Available Not Available YES 536 Distant Metastasis Biopsy with Histologic Confirmation;Convincing Imaging YES Not Available No AAQV Preoperative TUMOR FREE 77.29 81.7 NO 78.80 83.3 Complete Remission/Response YES BLACK OR AFRICAN AMERICAN NO NA R0 Not Applicable Not Applicable Not Applicable Not Applicable Not Applicable Not Applicable Not Applicable Not Applicable Not Applicable Stage II Not Applicable Not Applicable Not Applicable 7th Not Applicable Not Applicable Not Applicable MX N1 T1 Not Available NO YES 49 1 Lung Dead 2014 2012 Not Available

1750 Alive T1 N0 M0 Stage IA NA 69 R-Upper Not Applicable TCGA-50-5944 1c339545-c08a-4a77-b8e9-a7e49e53853f 2 -25440 Not Applicable 0 373 Not Available Lung Adenocarcinoma Not Available NA Not Available Not Available Not Available Not Available Not Available Not Available Not Available Not Available Not Available NA Not Available;Not Available Not Available;Not Available Not Available;Not Available Not Available;Not Available TCGA-50-5944-F32136;TCGA-50-5944-F70454 974a8262-421f-448c-805e-b4553844dc24;6CEEF46B-0ED2-4D96-BB16-9FB2A5D44BF9 17;16 Not Applicable;Not Applicable Not Applicable;Not Applicable Not Applicable;Not Applicable 764;1750 Not Applicable;Not Applicable Not Available;Not Available Scheduled Follow-up Submission;Scheduled Follow-up Submission Complete Remission/Response;Complete Remission/Response Not Available;Not Available NO;NO 5;2 Not Available;Not Available NO;NO Not Available;Not Available TUMOR FREE;TUMOR FREE NO;NO Complete Remission/Response;Complete Remission/Response Not Available;Not Available NO;NO Alive;Alive 2012;2015 FEMALE Lung Acinar Adenocarcinoma No C34.1 8550/3 C34.1 YES Not Available Not Available Not Available Not Available Not Available 7 Not Available Not Available Not Available Not Available Not Available Not Available Not Available Not Available Not Available Not Available Not Available No 5944 Not Available TUMOR FREE Not Available Not Available Not Available Not Available Not Available Not Available NO WHITE Not Available NA R0 Not Applicable Not Applicable Not Applicable Not Applicable Not Applicable Not Applicable Not Applicable Not Applicable Not Applicable Stage IA Not Applicable Not Applicable Not Applicable 7th Not Applicable Not Applicable Not Applicable M0 N0 T1 Not Available NO YES 50 Not Available Lung Alive 2011 2010 Not Available

2832 Alive T1 N0 M0 Stage IA NA Not Available R-Upper Not Applicable TCGA-80-5608 5c06d97f-2aaa-43de-b085-09d7829356ba 15 Not Available Not Applicable Not Available Not Available Not Available Lung Adenocarcinoma Not Available NA 1 Not Available Not Available Not Available Not Available Not Available Not Available Not Available Not Available NA Not Available;Not Available Not Available;Not Available Not Available;Not Available Not Available;Not Available TCGA-80-5608-F15870;TCGA-80-5608-F72033 242c20b9-5323-4ed7-91f3-3620555f7814;D9A05844-EA6C-4BE0-A6B9-C557069ACD65 15;2 Not Applicable;Not Applicable Not Applicable;Not Applicable Not Applicable;Not Applicable Not Available;2832 Not Applicable;Not Applicable Not Available;Unknown Not Available;Scheduled Follow-up Submission Not Available;Not Available Not Available;Unknown Not Available;NO 8;4 Not Available;Not Available NO;NO Not Available;Unknown TUMOR FREE;TUMOR FREE NO;NO Complete Remission/Response;Complete Remission/Response Not Available;Not Available NO;NO Alive;Alive 2011;2015 FEMALE Lung Adenocarcinoma- Not Otherwise Specified (NOS) No C34.1 8140/3 C34.1 YES Not Available Not Available Not Available Not Available Peripheral Lung 8 Not Available Not Available Not Available Not Available Not Available Not Available Not Available Not Available Not Available Not Available 26 No 5608 Preoperative TUMOR FREE Not Available Not Available Not Available Not Available Not Available Not Available Not Available Not Available Not Available NA R0 Not Applicable Not Applicable Not Applicable Not Applicable Not Applicable Not Applicable Not Applicable Not Applicable Not Applicable Stage IA Not Applicable Not Applicable Not Applicable 6th Not Applicable Not Applicable Not Applicable M0 N0 T1 Not Available NO YES 80 2 Lung Alive 2011 Not Available 1972

565 Alive T2a N1 M0 Stage IIA NA 69 L-Lower Not Applicable TCGA-L9-A7SV 54E90433-5218-4D83-848F-F995ECBA2A63 7 -25298 Not Applicable 0 111 Not Available Lung Adenocarcinoma 55 NA TCGA-L9-A7SV-D55465;TCGA-L9-A7SV-D55466 01FFB728-9015-4A08-9273-307A5FFCA9C7;AF22DDC1-B76C-49AC-AAC4-32359ACDB718 Not Available;Not Available 7;7 104;104 41;41 cisplatin;pemetrexed disodium Complete Response;Complete Response 1;1 Not Available;Not Available Not Available;Not Available Not Available;Not Available Not Available;Not Available Not Applicable;Not Applicable Not Available;Not Available Not Available;Not Available NO;NO Chemotherapy;Chemotherapy Not Available;Not Available Not Available;Not Available Not Available;Not Available NO;NO 2014;2014 0 Not Available NO Not Available Not Available Not Available NO Not Available NOT HISPANIC OR LATINO NA Not Available Not Available Not Available Not Available TCGA-L9-A7SV-F65116 4AC0A1A6-41A9-4E59-B676-A750306905C7 18 Not Applicable Not Applicable Not Applicable 565 Not Applicable 0 Scheduled Follow-up Submission Complete Remission/Response Not Available NO 9 Not Available NO Preoperative TUMOR FREE YES Complete Remission/Response Not Available NO Alive 2014 MALE Lung Adenocarcinoma- Not Otherwise Specified (NOS) No C34.3 8140/3 C34.3 YES Not Evaluated NO Not Available Not Available Unknown 1 Not Available Not Available Not Available Not Available Not Available Not Available Not Available Not Available Not Available NO 93 No A7SV Preoperative TUMOR FREE 40 45 YES 38 40 Complete Remission/Response YES BLACK OR AFRICAN AMERICAN NO NA R0 Not Applicable Not Applicable Not Applicable Not Applicable Not Applicable Not Applicable Not Applicable Not Applicable Not Applicable Stage IIA Not Applicable Not Applicable Not Applicable 7th Not Applicable Not Applicable Not Applicable M0 N1 T2a 1990 YES NO L9 3 Lung Alive 2014 2013 1959

202 Alive T3 N0 M0 Stage IIB NA 73 R-Lower Not Applicable TCGA-69-7760 8981f0c9-7155-4c96-bf77-7d40fe404323 24 -26777 Not Applicable 0 154 Not Available Lung Adenocarcinoma 125 NA TCGA-69-7760-D20981;TCGA-69-7760-D21152 94bb8b11-881e-449b-8206-95458a4e6762;5fa9b68b-32b6-4df9-a9a8-01cc626473d6 Not Available;Not Available 25;2 110;110 47;47 Carboplatin;pemetrexed disodium Not Available;Not Available 1;2 4;4 588.6;905 mg;mg ADJUVANT;ADJUVANT Not Applicable;Not Applicable 1;1 IV;IV NO;NO Chemotherapy;Chemotherapy Not Available;Not Available Not Available;Not Available Not Available;Not Available Not Available;Not Available 2012;2012 Not Available Not Available YES Not Available Not Available Not Available NO Not Available NOT HISPANIC OR LATINO NA Not Available Not Available Not Available Not Available TCGA-69-7760-F29247 58e0fa1d-b060-47b2-808b-631e2c8d3b82 13 Not Applicable Not Applicable Not Applicable 202 Not Applicable Not Available Scheduled Follow-up Submission Not Available Not Available Not Available 3 Not Available NO Post-Adjuvant Therapy TUMOR FREE YES Not Available Not Available NO Alive 2012 MALE Lung Adenocarcinoma Mixed Subtype No C34.3 8255/3 C34.3 YES Not Available NO Not Available Not Available Peripheral Lung 1 Not Available Not Available Not Available Not Available Not Available Not Available Not Available Not Available Not Available Not Available Not Available No 7760 Not Available Not Available Not Available Not Available Not Available 91 83 Not Available YES WHITE Not Available NA Not Available Not Applicable Not Applicable Not Applicable Not Applicable Not Applicable Not Applicable Not Applicable Not Applicable Not Applicable Stage IIB Not Applicable Not Applicable Not Applicable 7th Not Applicable Not Applicable Not Applicable M0 N0 T3 Not Available YES NO 69 1 Lung Alive 2012 2011 Not Available

60 Alive T1 N1 MX Stage IIA NA 65 L-Upper Not Applicable TCGA-91-6830 5a2f8140-8f90-4e94-b703-5fa5aa96be7b 19 -23922 Not Applicable 0 1 Not Available Lung Adenocarcinoma Not Available NA Not Available Not Available Not Available Not Available Not Available Not Available Not Available Not Available NOT HISPANIC OR LATINO NA NO YES Not Available YES TCGA-91-6830-F32595 557AFAED-4E1C-41AA-BFA7-0BEFFDF7227D 4 Not Available 18 Not Applicable 60 18 Unknown Scheduled Follow-up Submission Unknown Unknown YES 6 Distant Metastasis YES Not Available Unknown NO Unknown Biopsy with Histologic Confirmation NO Alive 2012 FEMALE Lung Adenocarcinoma- Not Otherwise Specified (NOS) No C34.1 8140/3 C34.1 YES Not Available NO Not Available Not Available Not Available 7 Not Available Not Available Not Available Not Available Not Available Not Available Not Available Not Available Not Available Not Available 12 No 6830 Not Available WITH TUMOR Not Available Not Available Not Available Not Available Not Available Not Available Not Available WHITE Not Available NA Distant site TCGA-91-6830-R32598 C7C5B07C-450D-4B29-8A87-B339547AAF1D Not Available 4 60 60 Unknown 6 1 2000 NO External Not Applicable Not Available Not Available cGy 2012 Not Available Not Applicable Not Applicable Not Applicable Not Applicable Not Applicable Not Applicable Not Applicable Not Applicable Not Applicable Stage IIA Not Applicable Not Applicable Not Applicable 5th Not Applicable Not Applicable Not Applicable MX N1 T1 2002 NO YES 91 4 Lung Alive 2011 2002 1990

87 Dead T2a N1 M0 Stage IIA NA 71 R-Lower Not Applicable TCGA-95-7562 193201a3-1447-47b1-bdf1-11ae0eb3b2f3 13 -26000 87 0 Not Available Not Available Lung Adenocarcinoma 57 NA TCGA-95-7562-D33261;TCGA-95-7562-D33262 1CB62B54-E04A-4A35-A0C0-5362A3F24D93;4F33410C-01F5-4F0E-A0A8-A416520BE0F7 Not Available;Not Available 26;26 81;81 60;60 CISPLATIN;ALIMTA Unknown;Unknown 6;6 Not Available;Not Available Not Available;Not Available Not Available;Not Available Not Available;Not Available Not Applicable;Not Applicable Not Available;Not Available Not Available;Not Available NO;NO Chemotherapy;Chemotherapy Not Available;Not Available Not Available;Not Available Not Available;Not Available YES;YES 2012;2012 1 Not Available Not Available Not Available Not Available Not Available Not Available Not Available NOT HISPANIC OR LATINO NA Not Available Not Available Not Available Not Available TCGA-95-7562-F32832 CCBA82F1-C522-4EF5-B5BB-A1F58E29ED89 13 Not Applicable Not Applicable 87 Not Available Not Applicable 1 Scheduled Follow-up Submission Not Evaluated Not Available NO 6 Not Available NO Post-Adjuvant Therapy TUMOR FREE YES Not Evaluated Not Available NO Dead 2012 MALE Lung Adenocarcinoma- Not Otherwise Specified (NOS) No C34.3 8140/3 C34.3 YES Not Available Not Available Not Available Not Available Not Available 6 Not Available Not Available Not Available Not Available Not Available Not Available Not Available Not Available Not Available NO 10 Yes, History of Prior Malignancy 7562 Post-Adjuvant Therapy TUMOR FREE 72 90 YES 76 80 Not Evaluated YES BLACK OR AFRICAN AMERICAN NO NA RX Not Applicable Not Applicable Not Applicable Not Applicable Not Applicable Not Applicable Not Applicable Not Applicable Not Applicable Stage IIA Not Applicable Not Applicable Not Applicable 7th Not Applicable Not Applicable Not Applicable M0 N1 T2a 1983 YES NO 95 3 Lung Dead 2012 2011 1973

1235 Dead T2 N1 M0 Stage IIB NA 75 R-Upper Not Applicable TCGA-50-5932 ebcba7f2-ce13-4bae-97cd-91a6b1dcd465 2 -27454 1235 0 1091 Not Available Lung Adenocarcinoma Not Available NA Not Available Not Available Not Available Not Available Not Available Not Available Not Available Not Available NOT HISPANIC OR LATINO NA YES NO NO NO TCGA-50-5932-F32080 aa578f39-0f94-41df-ad90-1a68f7a7fb43 16 Not Available Not Available 1235 Not Available 1090 Not Available Scheduled Follow-up Submission Progressive Disease Not Available NO 5 Locoregional Recurrence YES Not Available WITH TUMOR NO Progressive Disease Biopsy with Histologic Confirmation;Convincing Imaging NO Dead 2012 MALE Lung Adenocarcinoma- Not Otherwise Specified (NOS) No C34.1 8140/3 C34.1 YES Not Available Not Available Not Available Not Available Central Lung 7 Not Available Not Available Not Available Not Available Not Available Not Available Not Available Not Available Not Available Not Available Not Available Yes 5932 Not Available WITH TUMOR Not Available Not Available Not Available Not Available Not Available Not Available Not Available WHITE Not Available NA Not Available Not Applicable Not Applicable Not Applicable Not Applicable Not Applicable Not Applicable Not Applicable Not Applicable Not Applicable Stage IIB Not Applicable Not Applicable Not Applicable 5th Not Applicable Not Applicable Not Applicable M0 N1 T2 Not Available NO YES 50 Not Available Lung Dead 2011 2001 Not Available

2065 Alive T2 N1 M0 Stage IIB NA 55 R-Upper Not Applicable TCGA-55-1596 199386c2-bb53-4fad-a1b6-59ab216a4a50 13 -20381 Not Applicable 0 1375 Not Available Lung Adenocarcinoma Not Available NA TCGA-55-1596-D40545;TCGA-55-1596-D40546 D3AC7328-D4A7-4F72-86F7-07379246F800;5871BBDC-FC66-413D-8DE6-E44CB7DACA4D Not Available;Not Available 22;22 Not Available;Not Available 34;34 Carboplatin;Taxol Complete Response;Complete Response 2;2 Not Available;Not Available Not Available;Not Available Not Available;Not Available Not Available;Not Available Not Applicable;Not Applicable Not Available;Not Available Not Available;Not Available NO;NO Chemotherapy;Chemotherapy Not Available;Not Available Not Available;Not Available Not Available;Not Available NO;NO 2013;2013 Not Available Not Available Not Available Not Available Not Available Not Available Not Available Not Available NOT HISPANIC OR LATINO NA Not Available Not Available Not Available Not Available TCGA-55-1596-F40544 123C8E70-9662-45C2-80BF-E22343435AD4 22 Not Applicable Not Applicable Not Applicable 2065 Not Applicable Not Available Scheduled Follow-up Submission Complete Remission/Response Not Available NO 2 Not Available NO Not Available TUMOR FREE YES Complete Remission/Response Not Available NO Alive 2013 MALE Lung Adenocarcinoma- Not Otherwise Specified (NOS) No C34.1 8140/3 C34.1 YES Not Available Not Available Not Available Not Available Not Available 9 Not Available Not Available Not Available Not Available Not Available Not Available Not Available Not Available Not Available Not Available 50 No 1596 Not Available TUMOR FREE Not Available Not Available Not Available Not Available Not Available Not Available Not Available ASIAN Not Available NA R0 Not Applicable Not Applicable Not Applicable Not Applicable Not Applicable Not Applicable Not Applicable Not Applicable Not Applicable Stage IIB Not Applicable Not Applicable Not Applicable 6th Not Applicable Not Applicable Not Applicable M0 N1 T2 Not Available NO YES 55 2 Lung Alive 2010 2004 1979

669 Alive T2 N0 M1 Stage IV NA 70 R-Middle Not Applicable TCGA-05-4425 69b23036-add9-42c9-941f-02fb74d9b08c 22 -25902 Not Applicable 0 669 Not Available Lung Adenocarcinoma Not Available NA TCGA-05-4425-D36541;TCGA-05-4425-D36542 9F72525F-2709-42EF-831A-25435428F054;E3F50215-27BD-43FC-BD2F-C7C06D44035E Not Available;Not Available 5;5 153;153 31;31 Cisplatin;Vinorelbine Unknown;Unknown 12;12 Not Available;Not Available Not Available;Not Available Not Available;Not Available Not Available;Not Available Not Applicable;Not Applicable Not Available;Not Available Not Available;Not Available NO;NO Chemotherapy;Chemotherapy Not Available;Not Available Not Available;Not Available Not Available;Not Available NO;NO 2012;2012 Not Available Not Available Not Available Not Available Not Available Not Available Not Available Not Available Not Available NA FEMALE Lung Adenocarcinoma Mixed Subtype No C34.2 8255/3 C34.2 YES Not Available Not Available Not Available Not Available Not Available 7 Not Available Not Available Not Available Not Available Not Available Not Available Not Available Not Available Not Available Not Available 28 No 4425 Not Available Not Available Not Available Not Available Not Available Not Available Not Available Not Available Not Available Not Available Not Available NA R1 Not Applicable Not Applicable Not Applicable Not Applicable Not Applicable Not Applicable Not Applicable Not Applicable Not Applicable Stage IV Not Applicable Not Applicable Not Applicable 6th Not Applicable Not Applicable Not Applicable M1 N0 T2 Not Available NO YES 05 3 Lung Alive 2010 2008 Not Available

409 Alive T2b NA 85 R-Lower Not Applicable TCGA-69-8254 f0c94f68-bfd9-45cb-b4fd-8bb1ae4e9a2b 4 -31134 Not Applicable 0 77 Not Available Lung Adenocarcinoma Not Available NA Not Available Not Available YES Other Not Available Not Available NO Not Available NOT HISPANIC OR LATINO NA Not Available Not Available Not Available Not Available TCGA-69-8254-F41950 F1B78CC3-B120-4814-91CD-93C4CD359BEA 5 Not Applicable Not Applicable Not Applicable 409 Not Applicable 0 Scheduled Follow-up Submission Complete Remission/Response Not Available NO 4 Not Available NO Preoperative TUMOR FREE NO Complete Remission/Response Not Available NO Alive 2013 MALE Lung Adenocarcinoma Mixed Subtype No C34.3 8255/3 C34.3 YES 80 NO Not Available Not Available Not Available 6 Not Available Not Available Not Available Not Available Not Available Not Available Not Available Not Available Not Available NO 40 No 8254 Other TUMOR FREE Not Available Not Available NO Not Available 62 Complete Remission/Response YES WHITE NO NA Not Available Not Applicable Not Applicable Not Applicable Not Applicable Not Applicable Not Applicable Not Applicable Not Applicable Not Applicable Not Available Not Applicable Not Applicable Not Applicable 7th Not Applicable Not Applicable Not Applicable Not Available Not Available T2b 1989 YES NO 69 3 Lung Alive 2012 2012 Not Available

487 Alive T1b N0 MX Stage IA NA 72 R-Upper Not Applicable TCGA-55-7573 8e41153b-9faf-45c3-8397-8a428ef5c0d5 11 -26302 Not Applicable 0 4 Not Available Lung Adenocarcinoma 51 NA 1 Not Available NO Not Available Not Available Not Available NO Not Available NOT HISPANIC OR LATINO NA Not Available Not Available Not Available Not Available TCGA-55-7573-F46657 483D103F-ADB6-45F2-991D-ED029568C19C 13 Not Applicable Not Applicable Not Applicable 487 Not Applicable Unknown Scheduled Follow-up Submission Complete Remission/Response Unknown NO 8 Not Available NO Unknown TUMOR FREE NO Complete Remission/Response Not Available NO Alive 2013 FEMALE Lung Adenocarcinoma- Not Otherwise Specified (NOS) No C34.1 8140/3 C34.1 YES 90 NO Not Available Not Available Central Lung 1 Not Available Not Available Not Available Not Available Not Available Not Available Not Available Not Available Not Available Not Available Not Available Yes 7573 Preoperative TUMOR FREE 119 134 Not Available 115 130 Not Available YES WHITE Not Available NA Not Available Not Applicable Not Applicable Not Applicable Not Applicable Not Applicable Not Applicable Not Applicable Not Applicable Not Applicable Stage IA Not Applicable Not Applicable Not Applicable 7th Not Applicable Not Applicable Not Applicable MX N0 T1b 1983 YES NO 55 3 Lung Alive 2012 2011 Not Available

553 Alive T2a N1 M0 Stage IIA NA 62 R-Lower Not Applicable TCGA-95-A4VN 389F78A3-FA0B-406C-9434-AA0298341A8C 22 -22927 Not Applicable 0 142 Not Available Lung Adenocarcinoma 50 NA Not Available Not Available YES Not Available Not Available Not Available NO Not Available NOT HISPANIC OR LATINO NA Not Available Not Available Not Available Not Available TCGA-95-A4VN-F58501 878D540E-C5BA-45D0-BC56-66F85D659549 11 Not Applicable Not Applicable Not Applicable 553 Not Applicable 1 Scheduled Follow-up Submission Stable Disease Not Evaluated NO 4 Not Available NO Pre-Adjuvant Therapy TUMOR FREE NO Stable Disease Not Available NO Alive 2014 FEMALE Lung Solid Pattern Predominant Adenocarcinoma No C34.3 8140/3 C34.3 YES Not Available NO Not Available Not Available Not Available 2 Not Available Not Available Not Available Not Available Not Available Not Available Not Available Not Available Not Available NO 42 Yes, History of Prior Malignancy A4VN Not Available TUMOR FREE 65 67 NO 60 58 Stable Disease YES WHITE NO NA R0 Not Applicable Not Applicable Not Applicable Not Applicable Not Applicable Not Applicable Not Applicable Not Applicable Not Applicable Stage IIA Not Applicable Not Applicable Not Applicable 7th Not Applicable Not Applicable Not Applicable M0 N1 T2a 2007 YES NO 95 4 Lung Alive 2013 2012 1965

285 Alive T3 N0 M0 Stage IIB NA 73 R-Upper Not Applicable TCGA-44-8119 230dc42d-681c-4e31-9d2e-c724e61829fb 31 -27003 Not Applicable 0 99 Not Available Lung Adenocarcinoma 40 NA 0 Not Available Unknown Not Available Not Available Not Available Unknown Not Available NOT HISPANIC OR LATINO NA Not Available Not Available NO Not Available TCGA-44-8119-F38976 B83A8367-B557-44BB-9319-F428D59570DE 8 Not Applicable Not Applicable Not Applicable 285 Not Applicable 0 Scheduled Follow-up Submission Complete Remission/Response Not Available NO 1 Not Available NO Preoperative TUMOR FREE NO Complete Remission/Response Not Available NO Alive 2013 MALE Lung Adenocarcinoma- Not Otherwise Specified (NOS) No C34.1 8140/3 C34.1 YES Not Available Unknown Not Available Not Available Not Available 5 Not Available Not Available Not Available Not Available Not Available Not Available Not Available Not Available Not Available NO 50 Yes, History of Prior Malignancy 8119 Preoperative TUMOR FREE 74 59 NO 73 55 Complete Remission/Response YES WHITE NO NA Not Available Not Applicable Not Applicable Not Applicable Not Applicable Not Applicable Not Applicable Not Applicable Not Applicable Not Applicable Stage IIB Not Applicable Not Applicable Not Applicable 7th Not Applicable Not Applicable Not Applicable M0 N0 T3 Not Available YES NO 44 2 Lung Alive 2012 2012 1961

694 Dead T2 N0 M0 Stage IB NA 66 L-Upper Not Applicable TCGA-86-8075 8869000d-09d7-465a-ac32-54d1935c43e7 16 -24402 Not Applicable 0 43 Not Available Lung Adenocarcinoma Not Available NA TCGA-86-8075-D41680;TCGA-86-8075-D41681;TCGA-86-8075-D41682 3C971D88-EA87-4F2F-893C-B7BB558C402E;7E6A245F-8FC9-4F05-BA87-C6FC12A202EB;9C62E82D-EABF-452D-9CE1-6F3146D45E6C Not Available;Not Available;Not Available 25;25;25 364;364;Not Available 201;201;388 Etoposide;Cisplatin;Gefitinib Complete Response;Complete Response;Not Applicable 3;3;3 Not Available;Not Available;Not Available Not Available;Not Available;Not Available Not Available;Not Available;Not Available Not Available;Not Available;Not Available Not Applicable;Not Applicable;Not Applicable Not Available;Not Available;Not Available Not Available;Not Available;Not Available NO;NO;YES Chemotherapy;Chemotherapy;Targeted Molecular therapy Not Available;Not Available;Not Available Not Available;Not Available;Not Available Not Available;Not Available;Not Available NO;NO;NO 2013;2013;2013 2 Not Available NO Not Available Not Available Not Available NO Not Available NOT HISPANIC OR LATINO NA YES;Not Available NO;Not Available NO;Not Available NO;Not Available TCGA-86-8075-F41679;TCGA-86-8075-F63521 686D13D9-B93A-4157-9E8D-B441EA26F6D0;B67D4CCB-D243-4337-B31E-BE40BFD0ED38 25;12 Not Available;Not Applicable Not Available;Not Applicable Not Applicable;694 479;Not Available 199;Not Applicable 1;1 Scheduled Follow-up Submission;Scheduled Follow-up Submission Not Applicable;Complete Remission/Response 90;90 NO;NO 3;8 Locoregional Recurrence;Not Available YES;NO Pre-Adjuvant Therapy;Preoperative TUMOR FREE;TUMOR FREE NO;NO Complete Remission/Response;Complete Remission/Response Biopsy with Histologic Confirmation;Not Available NO;NO Alive;Dead 2013;2014 FEMALE Lung Adenocarcinoma- Not Otherwise Specified (NOS) No C34.1 8140/3 C34.1 YES 80 NO Not Available Not Available Peripheral Lung 5 Not Available Not Available Not Available Not Available Not Available Not Available Not Available Not Available Not Available Unknown Not Available No 8075 Preoperative TUMOR FREE Not Available Not Available Unknown Not Available Not Available Unknown NO WHITE Unknown NA R0 Not Applicable Not Applicable Not Applicable Not Applicable Not Applicable Not Applicable Not Applicable Not Applicable Not Applicable Stage IB Not Applicable Not Applicable Not Applicable 7th Not Applicable Not Applicable Not Applicable M0 N0 T2 Not Available YES NO 86 1 Lung Alive 2012 2011 Not Available

564 Alive T2a N0 MX Stage IB NA 65 L-Upper Not Applicable TCGA-97-7937 fcfe0b53-dfc1-42fa-9efc-2b2ff259297e 13 -23919 Not Applicable 0 181 Not Available Lung Adenocarcinoma 89 NA 0 Not Available YES Not Available Not Available FISH YES Not Available NOT HISPANIC OR LATINO NA Not Available Not Available NO Not Available TCGA-97-7937-F33767 483E201C-601E-4BEC-9BD0-EF8AFFBA9552 12 Not Applicable Not Applicable Not Applicable 564 Not Applicable Not Available Scheduled Follow-up Submission Not Available Not Available NO 9 Not Available NO Not Available TUMOR FREE NO Stable Disease Not Available NO Alive 2012 MALE Lung Micropapillary Adenocarcinoma No C34.1 8507/3 C34.1 YES Not Available YES NO Not Available Not Available 2 Not Available Not Available Not Available Not Available Not Available Not Available Not Available Not Available Not Available Not Available 35 No 7937 Preoperative Not Available Not Available Not Available Not Available 77 120 Not Available YES WHITE Not Available NA Not Available Not Applicable Not Applicable Not Applicable Not Applicable Not Applicable Not Applicable Not Applicable Not Applicable Not Applicable Stage IB Not Applicable Not Applicable Not Applicable 7th Not Applicable Not Applicable Not Applicable MX N0 T2a 1994 YES NO 97 3 Lung Alive 2012 2011 Not Available

1357 Dead T2 N1 M1 Stage IV NA 42 L-Upper Not Applicable TCGA-38-4632 875333ab-9048-462d-aaa2-693ad127e3cc 13 -15418 1357 0 Not Available Not Available Lung Adenocarcinoma Not Available NA TCGA-38-4632-D4840;TCGA-38-4632-D4844;TCGA-38-4632-D13822;TCGA-38-4632-D13824 77d7b5e2-ec9e-44d8-a8bb-fc29c602ae6a;54475225-34f4-4948-996f-e97089cd5a04;d1a79d21-2ed8-415a-aea3-af762a661683;e8d4b552-6c8f-4cfb-93cd-267605c6a506 Not Available;Not Available;Not Available;Not Available 13;13;14;14 802;1169;802;1169 679;1136;679;1127 gemcitabine;Carboplatin;Paclitaxel;CPT-11 Not Available;Not Available;Not Available;Not Available 12;12;7;7 6;2;6;2 Not Available;Not Available;Not Available;Not Available mg;mg;Not Available;Not Available RECURRENCE;PALLIATIVE;RECURRENCE;PALLIATIVE Not Applicable;Not Applicable;Not Applicable;Not Applicable LCCC 9831;3;LCCC 9831;3 IV;IV;IV;IV NO;NO;NO;NO Chemotherapy;Chemotherapy;Chemotherapy;Chemotherapy Not Available;Not Available;Not Available;Not Available Not Available;Not Available;Not Available;Not Available Not Available;Not Available;mg/m2;mg/m2 Not Available;Not Available;Not Available;Not Available 2010;2010;2011;2011 Not Available Not Available NO Not Available Not Available Not Available NO Not Available NOT HISPANIC OR LATINO NA YES;YES NO;YES NO;NO NO;NO TCGA-38-4632-F4839;TCGA-38-4632-F4842 ecb636f6-dc7d-48c5-9a00-02aec73f03b1;1fb434a3-8648-499c-8e36-97c1ea94dad2 13;13 Not Available;Not Available Not Available;Not Available 1357;1357 Not Available;Not Available 680;925 Not Available;Not Available Not Available;Not Available Stable Disease;Progressive Disease Not Available;Not Available Not Available;Not Available 12;12 Not Available;Not Available YES;YES Not Available;Not Available WITH TUMOR;WITH TUMOR NO;NO Complete Remission/Response;Complete Remission/Response Not Available;Not Available NO;NO Dead;Dead 2010;2010 MALE Lung Adenocarcinoma- Not Otherwise Specified (NOS) No C34.1 8140/3 C34.1 YES Not Available NO Not Available Not Available Peripheral Lung 12 Not Available Not Available Not Available Not Available Not Available Not Available Not Available Not Available Not Available Not Available 10 No 4632 Not Available WITH TUMOR Not Available Not Available Not Available Not Available Not Available Not Available NO BLACK OR AFRICAN AMERICAN Not Available NA Distant Recurrence;Distant site TCGA-38-4632-R4843;TCGA-38-4632-R40104 5dd66aad-2ab7-4f32-9a98-b640c47d392f;E3818E10-E37C-4DCF-881F-A1B668E6A6B7 3;Not Available 13;12 955;70 955;35 Not Available;Complete Response 12;2 10;22 2500;5800 NO;NO EXTERNAL BEAM;External Not Applicable;Not Applicable PALLIATIVE;Not Available Not Available;Not Available cGy;cGy 2010;2013 R0 Not Applicable Not Applicable Not Applicable Not Applicable Not Applicable Not Applicable Not Applicable Not Applicable Not Applicable Stage IV Not Applicable Not Applicable Not Applicable 5th Not Applicable Not Applicable Not Applicable M1 N1 T2 1991 NO YES 38 4 Lung Dead 2010 1998 Not Available

2368 Alive T2 N0 M0 Stage IB NA Not Available R-Lower Not Applicable TCGA-75-5146 1d276b62-5e64-48fb-b2ce-5192b511fe37 6 Not Available Not Applicable Not Available Not Available Not Available Lung Adenocarcinoma Not Available NA Not Available Not Available NO Not Available Not Available Not Available NO Not Available Not Available NA Not Available;YES Not Available;NO Not Available;Not Available Not Available;Not Available TCGA-75-5146-F11744;TCGA-75-5146-F72030 d54f5d79-f71c-4fc5-8a86-be0e098fae39;A034F00F-B0BD-4792-8085-A036CD719983 6;2 Not Applicable;Not Available Not Applicable;Not Available Not Applicable;Not Applicable Not Available;2368 Not Applicable;1773 Not Available;Not Evaluated Not Available;Scheduled Follow-up Submission Complete Remission/Response;Unknown Not Available;Not Evaluated Not Available;NO 4;4 Not Available;New Primary Tumor NO;YES Adjuvant therapy;Not Available TUMOR FREE;TUMOR FREE NO;NO Complete Remission/Response;Complete Remission/Response Not Available;Not Available NO;NO Alive;Alive 2011;2015 MALE Lung Adenocarcinoma- Not Otherwise Specified (NOS) No C34.3 8252/3 C34.3 YES Not Available NO Not Available Not Available Peripheral Lung 4 Not Available Not Available Not Available Not Available Not Available Not Available Not Available Not Available Not Available Not Available Not Available No 5146 Other TUMOR FREE Not Available Not Available Not Available Not Available Not Available Not Available Not Available Not Available Not Available NA R0 Not Applicable Not Applicable Not Applicable Not Applicable Not Applicable Not Applicable Not Applicable Not Applicable Not Applicable Stage IB Not Applicable Not Applicable Not Applicable 6th Not Applicable Not Applicable Not Applicable M0 N0 T2 1987 NO YES 75 3 Lung Alive 2011 Not Available Not Available

7062 Alive T1 N1 M0 Stage IIA NA 59 L-Upper Not Applicable TCGA-78-8640 18478121-f1d1-4bdd-9511-8ab22f4a0660 23 -21832 Not Applicable 0 6346 Not Available Lung Adenocarcinoma Not Available NA 0 Not Available NO Not Available Not Available Not Available NO Not Available Not Evaluated NA Not Available;Not Available Not Available;Not Available Not Available;Not Available Not Available;Not Available TCGA-78-8640-F46106;TCGA-78-8640-F70500 E3777749-8064-4049-A825-BB96779AEAD3;D76CC938-737A-46E4-BA56-1A16F671F442 4;25 Not Applicable;Not Available Not Applicable;Not Available Not Applicable;Not Applicable 6528;7062 Not Applicable;Not Available Unknown;Unknown Scheduled Follow-up Submission;Scheduled Follow-up Submission Complete Remission/Response;Unknown Not Available;Not Available NO;NO 8;3 Not Available;Not Available NO;Unknown Unknown;Not Available Unknown;Unknown NO;NO Complete Remission/Response;Complete Remission/Response Not Available;Not Available NO;NO Alive;Alive 2013;2015 MALE Lung Adenocarcinoma- Not Otherwise Specified (NOS) No C34.1 8140/3 C34.1 YES Not Available NO Not Available Not Available Peripheral Lung 10 Not Available Not Available Not Available Not Available Not Available Not Available Not Available Not Available Not Available NO 45 No 8640 Preoperative TUMOR FREE Not Available Not Available NO 73 66 Complete Remission/Response YES Not Evaluated NO NA R0 Not Applicable Not Applicable Not Applicable Not Applicable Not Applicable Not Applicable Not Applicable Not Applicable Not Applicable Stage IIA Not Applicable Not Applicable Not Applicable 6th Not Applicable Not Applicable Not Applicable M0 N1 T1 Not Available NO YES 78 2 Lung Alive 2012 1994 1949

161 Dead T2 N2 M0 Stage IIIA NA 68 L-Upper Not Applicable TCGA-MP-A4T8 7C4ED533-EE66-48BE-A84B-FF8607742818 2 -25069 161 0 Not Available Not Available Lung Adenocarcinoma 102 NA TCGA-MP-A4T8-D41347;TCGA-MP-A4T8-D41348 5847897E-0C0F-4333-98FC-46101B12E294;254AEB4C-E848-471D-90B6-75DC48A1F902 Not Available;Not Available 15;15 72;72 42;42 Navelbine;Cisplatin Unknown;Unknown 3;3 Not Available;Not Available Not Available;Not Available Not Available;Not Available Not Available;Not Available Not Applicable;Not Applicable Not Available;Not Available Not Available;Not Available NO;NO Chemotherapy;Chemotherapy Not Available;Not Available Not Available;Not Available Not Available;Not Available NO;NO 2013;2013 1 Not Available NO Not Available Not Available Not Available NO Not Available Unknown NA MALE Lung Adenocarcinoma- Not Otherwise Specified (NOS) No C34.1 8140/3 C34.1 YES Not Evaluated NO Not Available Not Available Unknown 4 Not Available Not Available Not Available Not Available Not Available Not Available Not Available Not Available Not Available Unknown 25 Yes, History of Prior Malignancy A4T8 Pre-Adjuvant Therapy Unknown Not Available 72 YES Not Available 60 Unknown YES Unknown NO NA R0 Not Applicable Not Applicable Not Applicable Not Applicable Not Applicable Not Applicable Not Applicable Not Applicable Not Applicable Stage IIIA Not Applicable Not Applicable Not Applicable 6th Not Applicable Not Applicable Not Applicable M0 N2 T2 1972 NO YES MP 3 Lung Dead 2013 2006 1957

657 Alive T2a N2 MX Stage IIIA NA 78 R-Lower Not Applicable TCGA-97-8547 ac0d7a82-82cb-4aec-b859-e37375f3de8b 27 -28801 Not Applicable 0 148 Not Available Lung Adenocarcinoma 87 NA TCGA-97-8547-D35445;TCGA-97-8547-D35446;TCGA-97-8547-D35447 46926057-7C1B-4651-913D-B4C08C699594;8EDFE56A-3EB6-47A8-A4BA-2255FF3A3418;393D56D3-0031-4F0A-9E38-F16B66B602D5 Not Available;Not Available;Not Available 27;27;27 111;111;Not Available 47;47;124 Taxol;Carboplatin;TARCEVA Complete Response;Complete Response;Not Applicable 9;9;9 Not Available;Not Available;Not Available Not Available;Not Available;Not Available Not Available;Not Available;Not Available Not Available;Not Available;Not Available Not Applicable;Not Applicable;Not Applicable Not Available;Not Available;Not Available Not Available;Not Available;Not Available NO;NO;YES Chemotherapy;Chemotherapy;Not Available Not Available;Not Available;Not Available Not Available;Not Available;Not Available Not Available;Not Available;Not Available NO;NO;NO 2012;2012;2012 Not Evaluated Not Available YES Exon 19 Deletion Not Available Not Available NO Not Available Not Evaluated NA Not Available Not Available Not Available Not Available TCGA-97-8547-F49559 C213E7BE-D60E-412A-BD5D-8BCFC8749CA7 3 Not Applicable Not Applicable Not Applicable 657 Not Applicable Not Evaluated Scheduled Follow-up Submission Complete Remission/Response Not Evaluated NO 10 Not Available NO Not Evaluated TUMOR FREE YES Complete Remission/Response Not Available NO Alive 2013 FEMALE Lung Adenocarcinoma Mixed Subtype No C34.3 8550/3 C34.3 YES Not Available YES NO Not Available Unknown 9 Not Available Not Available Not Available Not Available Not Available Not Available Not Available Not Available Not Available NO Not Available No 8547 Not Available TUMOR FREE Not Available Not Available YES Not Available 136 Complete Remission/Response YES Not Evaluated NO NA Not Evaluated Not Applicable Not Applicable Not Applicable Not Applicable Not Applicable Not Applicable Not Applicable Not Applicable Not Applicable Stage IIIA Not Applicable Not Applicable Not Applicable 7th Not Applicable Not Applicable Not Applicable MX N2 T2a Not Available YES NO 97 1 Lung Alive 2012 2012 Not Available

1135 Dead T1 N0 MX Stage IA NA 56 R-Lower Not Applicable TCGA-49-AARN 98EF41FA-3015-445A-BBDC-984361C911F7 19 -20605 1135 0 Not Available Not Available Lung Adenocarcinoma 110.6 NA 1 Not Available Not Available Not Available Not Available Not Available Not Available Not Available NOT HISPANIC OR LATINO NA FEMALE Lung Adenocarcinoma- Not Otherwise Specified (NOS) No C34.3 8140/3 C34.3 YES 90 Not Available Not Available Not Available Not Available 6 Not Available Not Available Not Available Not Available Not Available Not Available Not Available Not Available Not Available Unknown Not Available Yes, History of Synchronous/Bilateral Malignancy AARN Preoperative Unknown 85.9 101.1 NO 80.2 94.4 Complete Remission/Response YES BLACK OR AFRICAN AMERICAN NO NA R0 Not Applicable Not Applicable Not Applicable Not Applicable Not Applicable Not Applicable Not Applicable Not Applicable Not Applicable Stage IA Not Applicable Not Applicable Not Applicable 5th Not Applicable Not Applicable Not Applicable MX N0 T1 Not Available NO YES 49 5 Lung Dead 2014 2000 Not Available

58 Dead T3 N2 M1b Stage IV NA 40 R-Upper Not Applicable TCGA-L9-A5IP 2A43E61E-2151-4C5A-B062-74CE9DFD8D4A 14 -14681 58 0 Not Available Not Available Lung Adenocarcinoma Not Available NA Not Evaluated Not Available YES Not Available Not Available FISH YES Not Available NOT HISPANIC OR LATINO NA FEMALE Lung Adenocarcinoma- Not Otherwise Specified (NOS) No C34.1 8140/3 C34.1 YES Not Available NO Not Available Not Available Unknown 5 NO NO 49 NO Not Available NO Not Available Locoregional Recurrence;Distant Metastasis Convincing Imaging YES Not Available No A5IP Not Evaluated WITH TUMOR Not Available Not Available NO Not Available Not Available Progressive Disease NO BLACK OR AFRICAN AMERICAN NO NA R1 Not Applicable Not Applicable Not Applicable Not Applicable Not Applicable Not Applicable Not Applicable Not Applicable Not Applicable Stage IV Not Applicable Not Applicable Not Applicable 7th Not Applicable Not Applicable Not Applicable M1b N2 T3 2012 YES NO L9 4 Lung Dead 2013 2012 Not Available

889 Alive T1b N0 M0 Stage IA NA 73 L-Upper Not Applicable TCGA-55-7995 abd6647b-d2eb-404d-8283-443e92addf55 7 -26837 Not Applicable 0 5 Not Available Lung Adenocarcinoma 51 NA TCGA-55-7995-D65702;TCGA-55-7995-D65703 39A1C7F0-0574-41C9-98D5-D0D7AF66D866;386B9E74-6618-451B-8F90-9F3BFDE51CD1 Not Available;Not Available 26;26 525;525 483;483 Carboplatin;Paclitaxel Complete Response;Complete Response 9;9 Not Available;Not Available Not Available;Not Available Not Available;Not Available Not Available;Not Available Not Applicable;Not Applicable Not Available;Not Available Not Available;Not Available NO;NO Chemotherapy;Chemotherapy Not Available;Not Available Not Available;Not Available Not Available;Not Available NO;NO 2014;2014 Not Available Not Available NO Not Available Not Available Not Available NO Not Available NOT HISPANIC OR LATINO NA YES YES NO Not Available TCGA-55-7995-F65700 FD821C68-D4E0-4555-BAD0-65DC0C3F3FD0 26 Not Available Not Available Not Applicable 889 468 1 Scheduled Follow-up Submission Complete Remission/Response 80 NO 9 Locoregional Recurrence YES Post-Adjuvant Therapy TUMOR FREE NO Complete Remission/Response Convincing Imaging NO Alive 2014 FEMALE Lung Adenocarcinoma- Not Otherwise Specified (NOS) No C34.1 8140/3 C34.1 YES Not Available Unknown Not Available Not Available Peripheral Lung 5 Not Available Not Available Not Available Not Available Not Available Not Available Not Available Not Available Not Available NO 60 No 7995 Not Available TUMOR FREE Not Available Not Available Unknown 71 77 Unknown YES WHITE Unknown NA Regional site TCGA-55-7995-R65701 FB0F9AE8-CC60-4A32-80BE-77F43A903343 Not Available 26 525 483 Complete Response 9 Not Available 6500 NO External Not Applicable Not Available Not Available cGy 2014 R0 Not Applicable Not Applicable Not Applicable Not Applicable Not Applicable Not Applicable Not Applicable Not Applicable Not Applicable Stage IA Not Applicable Not Applicable Not Applicable 7th Not Applicable Not Applicable Not Applicable M0 N0 T1b 2002 YES NO 55 4 Lung Alive 2012 2012 Not Available

426 Alive T1a N1 MX Stage IIA NA 59 R-Lower Not Applicable TCGA-69-8253 44d8c859-4b7a-4e9d-bffc-0de0e6afdcc4 1 -21771 Not Applicable 0 100 Not Available Lung Adenocarcinoma Not Available NA TCGA-69-8253-D32564;TCGA-69-8253-D32565 E2D64977-0DCC-4BF6-A66E-7D40AF9263AF;3AF5EEC9-47EC-4D16-8427-FE85149B06A5 Not Available;Not Available 1;1 Not Available;Not Available 55;55 Cisplatin;Pemetrexed Not Applicable;Not Applicable 6;6 Not Available;Not Available Not Available;Not Available Not Available;Not Available Not Available;Not Available Not Applicable;Not Applicable Not Available;Not Available Not Available;Not Available YES;YES Chemotherapy;Chemotherapy Not Available;Not Available Not Available;Not Available Not Available;Not Available NO;NO 2012;2012 2 Not Available NO Not Available Not Available Not Available NO Not Available NOT HISPANIC OR LATINO NA Not Available Not Available Not Available Not Available TCGA-69-8253-F41949 909F53FB-3A49-456C-9593-B6B043711B7B 5 Not Applicable Not Applicable Not Applicable 426 Not Applicable 1 Scheduled Follow-up Submission Complete Remission/Response Not Evaluated NO 4 Not Available NO Preoperative TUMOR FREE YES Complete Remission/Response Not Available NO Alive 2013 FEMALE Lung Adenocarcinoma Mixed Subtype No C34.3 8255/3 C34.3 YES Not Available NO Not Available Not Available Not Available 6 Not Available Not Available Not Available Not Available Not Available Not Available Not Available Not Available Not Available NO 7.8 No 8253 Pre-Adjuvant Therapy Unknown Not Available Not Available YES Not Available 87 Unknown YES BLACK OR AFRICAN AMERICAN NO NA Not Available Not Applicable Not Applicable Not Applicable Not Applicable Not Applicable Not Applicable Not Applicable Not Applicable Not Applicable Stage IIA Not Applicable Not Applicable Not Applicable 7th Not Applicable Not Applicable Not Applicable MX N1 T1a 2011 YES NO 69 4 Lung Alive 2012 2012 1972

791 Alive T2 N1 M0 Stage IIB NA 65 L-Upper Not Applicable TCGA-05-4427 78a24c56-576c-4245-bb42-6603b3d19897 22 -23893 Not Applicable 0 791 Not Available Lung Adenocarcinoma Not Available NA TCGA-05-4427-D36565;TCGA-05-4427-D36566 8809622A-D398-4D9D-95B7-92480AD0CA12;2978B988-DC8D-449A-83CC-6C4831AA0FE2 Not Available;Not Available 5;5 122;122 61;61 Cisplatin;Vinorelbine Complete Response;Complete Response 12;12 Not Available;Not Available Not Available;Not Available Not Available;Not Available Not Available;Not Available Not Applicable;Not Applicable Not Available;Not Available Not Available;Not Available NO;NO Chemotherapy;Chemotherapy Not Available;Not Available Not Available;Not Available Not Available;Not Available NO;NO 2012;2012 Not Available Not Available Not Available Not Available Not Available Not Available Not Available Not Available Not Available NA Not Available Not Available Not Available Not Available TCGA-05-4427-F36564 C0280C41-DC59-4741-8314-055BD0B90DC2 2 Not Applicable Not Applicable Not Applicable 791 Not Applicable Not Available Scheduled Follow-up Submission Complete Remission/Response Not Available NO 11 Not Available NO Not Available TUMOR FREE YES Complete Remission/Response Not Available NO Alive 2012 FEMALE Lung Adenocarcinoma Mixed Subtype No C34.1 8255/3 C34.1 YES Not Available Not Available Not Available Not Available Not Available 7 Not Available Not Available Not Available Not Available Not Available Not Available Not Available Not Available Not Available Not Available 8 Yes 4427 Not Available TUMOR FREE Not Available Not Available Not Available Not Available Not Available Not Available Not Available Not Available Not Available NA R0 Not Applicable Not Applicable Not Applicable Not Applicable Not Applicable Not Applicable Not Applicable Not Applicable Not Applicable Stage IIB Not Applicable Not Applicable Not Applicable 6th Not Applicable Not Applicable Not Applicable M0 N1 T2 2008 NO YES 05 4 Lung Alive 2010 2008 1993

1043 Dead T2 N0 M1 Stage IV NA 40 R-Upper Not Applicable TCGA-53-7624 54a9cc9e-3eee-4caa-9f33-4ea64a2999ba 13 -14794 1043 0 Not Available Not Available Lung Adenocarcinoma Not Available NA TCGA-53-7624-D39727;TCGA-53-7624-D39729;TCGA-53-7624-D39730;TCGA-53-7624-D39731;TCGA-53-7624-D39732;TCGA-53-7624-D39735;TCGA-53-7624-D39736;TCGA-53-7624-D39737;TCGA-53-7624-D39738;TCGA-53-7624-D39739 CABE6493-EEA3-4417-991E-E2D8BAA5D0F4;28681B5B-2CCC-41DB-B342-A1BB56EE7432;03D35A9D-2F9E-4DD6-BEF9-4EAE9D35C1DB;F6AB7F08-1341-440F-90F1-54F3E9CF0C7F;588CEFB2-2FCB-4A28-893B-35DDF6E44FB9;9628B2FD-EDBE-415B-A139-7EC10C28CF7E;5CDA082A-CFF9-4E2A-9C47-299425450EF8;87E2D964-3168-438C-9CB9-1F81D70122AE;0F25327C-C54D-4542-A956-47AFFD9D63B5;EAC0A9FD-301B-48F7-AFD2-499DFE2F0418 Not Available;Not Available;Not Available;Not Available;Not Available;Not Available;Not Available;Not Available;Not Available;Not Available 31;31;31;31;31;31;31;31;31;31 966;922;980;861;782;186;186;490;490;490 924;880;980;803;726;66;66;339;339;339 Irinotecan;Erlotinib;Abraxane;Docetaxel;Gemcitabine;Cisplatin;Vinorelbine;Carboplatinum;Bevacizumab;Pemetrexed Clinical Progressive Disease;Clinical Progressive Disease;Clinical Progressive Disease;Clinical Progressive Disease;Clinical Progressive Disease;Clinical Progressive Disease;Clinical Progressive Disease;Clinical Progressive Disease;Clinical Progressive Disease;Clinical Progressive Disease 1;1;1;1;1;1;1;1;1;1 Not Available;Not Available;Not Available;Not Available;Not Available;Not Available;Not Available;Not Available;Not Available;Not Available Not Available;Not Available;Not Available;Not Available;Not Available;Not Available;Not Available;Not Available;Not Available;Not Available Not Available;Not Available;Not Available;Not Available;Not Available;Not Available;Not Available;Not Available;Not Available;Not Available Not Available;Not Available;Not Available;Not Available;Not Available;Not Available;Not Available;Not Available;Not Available;Not Available Not Applicable;Not Applicable;Not Applicable;Not Applicable;Not Applicable;Not Applicable;Not Applicable;Not Applicable;Not Applicable;Not Applicable Not Available;Not Available;Not Available;Not Available;Not Available;Not Available;Not Available;Not Available;Not Available;Not Available Not Available;Not Available;Not Available;Not Available;Not Available;Not Available;Not Available;Not Available;Not Available;Not Available NO;NO;NO;NO;NO;NO;NO;NO;NO;NO Chemotherapy;Chemotherapy;Chemotherapy;Chemotherapy;Chemotherapy;Chemotherapy;Chemotherapy;Chemotherapy;Chemotherapy;Chemotherapy Not Available;Not Available;Not Available;Not Available;Not Available;Not Available;Not Available;Not Available;Not Available;Not Available Not Available;Not Available;Not Available;Not Available;Not Available;Not Available;Not Available;Not Available;Not Available;Not Available Not Available;Not Available;Not Available;Not Available;Not Available;Not Available;Not Available;Not Available;Not Available;Not Available NO;NO;NO;NO;NO;NO;NO;NO;NO;NO 2013;2013;2013;2013;2013;2013;2013;2013;2013;2013 0 Not Available NO Not Available Not Available Not Available NO Not Available HISPANIC OR LATINO NA YES YES Not Available YES TCGA-53-7624-F39722 9661057B-3EF1-4814-AE67-408068F09148 31 Not Available 593 1043 Not Available 400 Not Available Scheduled Follow-up Submission Progressive Disease 0 NO 1 Distant Metastasis YES Not Available WITH TUMOR YES Progressive Disease Biopsy with Histologic Confirmation NO Dead 2013 FEMALE Lung Adenocarcinoma- Not Otherwise Specified (NOS) No C34.1 8140/3 C34.1 YES Not Available NO Not Available Not Available Peripheral Lung 12 Not Available Not Available Not Available Not Available Not Available Not Available Not Available Not Available Not Available Not Available 50 No 7624 Post-Adjuvant Therapy WITH TUMOR Not Available Not Available Not Available Not Available Not Available Not Available Not Available WHITE Not Available NA Distant site TCGA-53-7624-R39726 78FACC85-69B0-4EEE-9713-CBF425E45FE0 Not Available 31 624 613 Radiographic Progressive Disease 1 10 30 NO External Not Applicable Not Available Not Available Gy 2013 R0 Not Applicable Not Applicable Not Applicable Not Applicable Not Applicable Not Applicable Not Applicable Not Applicable Not Applicable Stage IV Not Applicable Not Applicable Not Applicable 6th Not Applicable Not Applicable Not Applicable M1 N0 T2 Not Available NO YES 53 2 Lung Dead 2011 2008 1986

1013 Alive T2b N2 M0 Stage IIIA NA 53 R-Lower Not Applicable TCGA-44-5643 27fceec1-3298-4cdd-a4e6-8f5cf34604f0 13 -19426 Not Applicable 0 106 Not Available Lung Adenocarcinoma 75 NA TCGA-44-5643-D12952;TCGA-44-5643-D12950;TCGA-44-5643-D12954 2b6a8199-4f87-4880-b81d-ac2b704b0451;9fedb69f-cc66-4f2f-b9f0-71e1dd2d695b;21d67e97-9cd3-4de6-8df9-83f9a1330403 Not Available;Not Available;Not Available 14;14;14 208;Not Available;208 131;223;131 Carboplatin;Tarceva;Taxol Not Available;Not Available;Not Available 6;6;6 8;132;8 205-300;150;185-190 mg;mg;mg ADJUVANT;ADJUVANT;ADJUVANT Not Applicable;Not Applicable;Not Applicable 1;1;1 IV;PO;IV NO;YES;NO Chemotherapy;Chemotherapy;Chemotherapy Not Available;Not Available;Not Available 2035;19800;1505 mg;mg;mg Not Available;Not Available;Not Available 2011;2011;2011 Not Available Not Available Not Available Not Available Not Available Not Available Not Available Not Available NOT HISPANIC OR LATINO NA Not Available;Not Available Not Available;Not Available Not Available;NO Not Available;Not Available TCGA-44-5643-F12927;TCGA-44-5643-F39742 0dd73d87-f8f5-42fe-85e8-a7552e3c8915;AC910C02-04DD-4779-8BE1-E41E05FE1AD3 14;25 Not Available;Not Applicable Not Available;Not Applicable Not Applicable;Not Applicable 417;1013 Not Available;Not Applicable Not Available;1 Not Available;Scheduled Follow-up Submission Not Available;Complete Remission/Response Not Available;Not Available Not Available;NO 6;2 Not Available;Not Available Not Available;NO Not Available;Other WITH TUMOR;TUMOR FREE YES;Not Available Not Available;Not Available Not Available;Not Available YES;Not Available Alive;Alive 2011;2013 MALE Lung Adenocarcinoma- Not Otherwise Specified (NOS) No C34.3 8140/3 C34.3 YES Not Available Not Available Not Available Not Available Not Available 4 Not Available Not Available Not Available Not Available Not Available Not Available Not Available Not Available Not Available Not Available 20 No 5643 Not Available TUMOR FREE Not Available Not Available Not Available 92 66 Not Available YES BLACK OR AFRICAN AMERICAN Not Available NA Primary Tumor Field TCGA-44-5643-R12947 973a5961-8dc0-4467-863b-01a26eba6b3a 1 14 168 129 Not Available 6 25 60 NO OTHER IMRT ADJUVANT Not Available cGy 2011 Not Available Not Applicable Not Applicable Not Applicable Not Applicable Not Applicable Not Applicable Not Applicable Not Applicable Not Applicable Stage IIIA Not Applicable Not Applicable Not Applicable 7th Not Applicable Not Applicable Not Applicable M0 N2 T2b Not Available YES NO 44 2 Lung Alive 2011 2010 Not Available

131 Alive T2 N0 M1 Stage IV NA 69 L-Upper Not Applicable TCGA-73-4670 1b354837-4925-4480-ac32-6b44d0957314 6 -25526 Not Applicable 0 14 Not Available Lung Adenocarcinoma Not Available NA TCGA-73-4670-D12984;TCGA-73-4670-D12987;TCGA-73-4670-D12986;TCGA-73-4670-D12988 fce5249c-69e4-43b1-ba19-7261731a8a90;06e09d14-17d7-4d81-a98c-780ac3ff2162;2aaeb964-d8d0-4811-85a8-5b26948bf157;634e1395-f22a-4b7b-80d6-9f2d2929ccd1 Not Available;Not Available;Not Available;Not Available 15;15;15;15 110;110;110;54 33;33;54;33 carboplatin;Avastin;alimta;taxol Not Available;Not Available;Not Available;Not Available 6;6;6;6 4;4;4;1 538;930;680;308 mg;mg;mg;mg ADJUVANT;ADJUVANT;ADJUVANT;ADJUVANT Not Applicable;Not Applicable;Not Applicable;Not Applicable 1;1;2;1 IV;IV;IV;IV NO;NO;NO;NO Chemotherapy;Chemotherapy;Chemotherapy;Chemotherapy Not Available;Not Available;Not Available;Not Available 2152;3720;2720;308 mg;mg;mg;mg Not Available;Not Available;Not Available;Not Available 2011;2011;2011;2011 Not Available Not Available NO Not Available Not Available Not Available NO Not Available NOT HISPANIC OR LATINO NA Not Available Not Available Not Available Not Available TCGA-73-4670-F12983 9f66a7c0-c40c-412d-ab8c-b6bac0bfe58d 15 Not Applicable Not Applicable Not Applicable 131 Not Applicable 2 Not Available Not Available 50 Not Available 6 Not Available NO Adjuvant therapy Not Available YES Stable Disease Not Available NO Alive 2011 FEMALE Lung Adenocarcinoma- Not Otherwise Specified (NOS) Discrepancy C34.1 8140/3 C34.1 YES Not Available NO Not Available Not Available Peripheral Lung 1 Not Available Not Available Not Available Not Available Not Available Not Available Not Available Not Available Not Available Not Available 30 No 4670 Not Available WITH TUMOR Not Available Not Available Not Available Not Available Not Available Not Available Not Available WHITE Not Available NA R0 Not Applicable Not Applicable Not Applicable Not Applicable Not Applicable Not Applicable Not Applicable Not Applicable Not Applicable Stage IV Not Applicable Not Applicable Not Applicable Not Available Not Applicable Not Applicable Not Applicable M1 N0 T2 2009 NO YES 73 4 Lung Alive 2011 2010 1979

1798 Dead T1 N0 MX Stage IA NA 74 L-Lower Not Applicable TCGA-O1-A52J 42432463-8E92-4F25-B72A-F03953527AA5 11 -27223 1798 0 Not Available Not Available Lung Adenocarcinoma Not Available NA 1 Not Available NO Not Available Not Available Not Available NO Not Available NOT HISPANIC OR LATINO NA FEMALE Lung Adenocarcinoma- Not Otherwise Specified (NOS) No C34.3 8140/3 C34.3 YES Not Evaluated NO Not Available Not Available Unknown 4 NO NO 898 NO Not Available Not Available Not Available Locoregional Recurrence Not Available YES 45 No A52J Unknown WITH TUMOR Not Available Not Available NO Not Available Not Available Unknown NO WHITE NO NA Not Evaluated Not Applicable Not Applicable Not Applicable Not Applicable Not Applicable Not Applicable Not Applicable Not Applicable Not Applicable Stage IA Not Applicable Not Applicable Not Applicable 6th Not Applicable Not Applicable Not Applicable MX N0 T1 1980 NO YES O1 3 Lung Dead 2013 2007 1950

1097 Alive T3 N0 MX Stage IIB NA 49 R-Lower Not Applicable TCGA-44-7667 7f6455e8-fa3d-4452-acb2-8c9995073072 21 -18062 Not Applicable 0 557 Not Available Lung Adenocarcinoma 50 NA Not Available Not Available Not Available Not Available Not Available Not Available Not Available Not Available NOT HISPANIC OR LATINO NA Not Available;Not Available Not Available;Not Available Not Available;Not Available Not Available;Not Available TCGA-44-7667-F19718;TCGA-44-7667-F39968 c40ab53b-c1c0-402e-aedc-d93158731d75;3218051E-D4B6-489A-B881-073C5B57FBEA 21;7 Not Applicable;Not Applicable Not Applicable;Not Applicable Not Applicable;Not Applicable 557;1097 Not Applicable;Not Applicable Not Available;Not Available Scheduled Follow-up Submission;Scheduled Follow-up Submission Complete Remission/Response;Complete Remission/Response Not Available;Not Available Not Available;NO 12;2 Not Available;Not Available NO;NO Not Available;Not Available TUMOR FREE;TUMOR FREE NO;NO Complete Remission/Response;Complete Remission/Response Not Available;Not Available NO;NO Alive;Alive 2011;2013 FEMALE Lung Adenocarcinoma- Not Otherwise Specified (NOS) No C34.3 8140/3 C34.3 YES Not Available NO Not Available Not Available Not Available 12 Not Available Not Available Not Available Not Available Not Available Not Available Not Available Not Available Not Available Not Available 50 No 7667 Not Available TUMOR FREE Not Available Not Available Not Available 104 74 Not Available YES WHITE Not Available NA Not Available Not Applicable Not Applicable Not Applicable Not Applicable Not Applicable Not Applicable Not Applicable Not Applicable Not Applicable Stage IIB Not Applicable Not Applicable Not Applicable 6th Not Applicable Not Applicable Not Applicable MX N0 T3 Not Available YES NO 44 2 Lung Alive 2011 2009 1974

634 Alive T1b N0 M0 Stage IA NA 83 R-Upper Not Applicable TCGA-97-A4M5 5FE77D4A-A8A5-4C90-8FF2-9C3BBBB309EF 7 -30665 Not Applicable 0 132 Not Available Lung Adenocarcinoma 51 NA Not Evaluated Not Available NO Not Available Not Available Not Available NO Not Available NOT HISPANIC OR LATINO NA Not Available Not Available Not Available Not Available TCGA-97-A4M5-F57443 45F6F53F-E17E-47FD-A616-202C86D48D1F 12 Not Applicable Not Applicable Not Applicable 634 Not Applicable Not Evaluated Scheduled Follow-up Submission Complete Remission/Response Not Evaluated NO 3 Not Available NO Not Evaluated TUMOR FREE NO Complete Remission/Response Not Available NO Alive 2014 MALE Lung Adenocarcinoma Mixed Subtype No C34.1 8255/3 C34.1 YES Not Evaluated YES YES G12C Unknown 3 Not Available Not Available Not Available Not Available Not Available Not Available Not Available Not Available Not Available NO 6 No A4M5 Not Evaluated Unknown 84 94 NO 85 98 Complete Remission/Response YES WHITE NO NA Not Evaluated Not Applicable Not Applicable Not Applicable Not Applicable Not Applicable Not Applicable Not Applicable Not Applicable Not Applicable Stage IA Not Applicable Not Applicable Not Applicable 7th Not Applicable Not Applicable Not Applicable M0 N0 T1b 1982 YES NO 97 3 Lung Alive 2013 2012 1952

50 Alive T2a N0 M0 Stage IB NA 52 R-Lower Not Applicable TCGA-NJ-A4YP 42259AA6-C9F5-4309-A956-DBF930312F44 27 -19106 Not Applicable 0 50 Not Available Lung Adenocarcinoma Not Available NA Unknown Not Available NO Not Available Not Available Not Available NO Not Available NOT HISPANIC OR LATINO NA Not Available Not Available Not Available Not Available TCGA-NJ-A4YP-F50888 5B07D5AE-C22E-4DED-8460-EA2FE846A2B4 1 Not Applicable Not Applicable Not Applicable 50 Not Applicable Not Available Scheduled Follow-up Submission Stable Disease Not Available YES 11 Not Available NO Not Available Not Available Not Available Not Available Not Available Not Available Alive 2013 MALE Lung Papillary Adenocarcinoma No C34.3 8255/3 C34.3 YES Unknown NO Not Available Not Available Unknown 9 Not Available Not Available Not Available Not Available Not Available Not Available Not Available Not Available Not Available NO 60 No A4YP Unknown TUMOR FREE Not Available Not Available NO Not Available Not Available Stable Disease NO WHITE NO NA Not Evaluated Not Applicable Not Applicable Not Applicable Not Applicable Not Applicable Not Applicable Not Applicable Not Applicable Not Applicable Stage IB Not Applicable Not Applicable Not Applicable 7th Not Applicable Not Applicable Not Applicable M0 N0 T2a Not Available Not Available Not Available NJ 5 Lung Alive 2013 2011 Not Available

1932 Alive T2 N0 MX Stage IB NA 70 L-Lower Not Applicable TCGA-97-7552 1b110eb4-a01d-4c50-ba18-77da4dedd549 17 -25578 Not Applicable 0 1476 Not Available Lung Adenocarcinoma Not Available NA Not Available Not Available Not Available Not Available Not Available Not Available Not Available Not Available NOT HISPANIC OR LATINO NA NO;NO NO;NO Not Available;Not Available Not Available;Not Available TCGA-97-7552-F18844;TCGA-97-7552-F37174 3e54d908-6857-428c-9f5c-f1832e592ace;85ED4CFE-DA99-429B-91F9-D17B739AD495 28;29 Not Available;Not Available Not Available;Not Available Not Applicable;Not Applicable 1932;1932 798;798 Not Available;Not Available Additional New Tumor Event;Scheduled Follow-up Submission Complete Remission/Response;Complete Remission/Response Not Available;Not Available Not Available;NO 11;11 Not Available;New Primary Tumor YES;YES Not Available;Not Available TUMOR FREE;TUMOR FREE NO;NO Not Available;Stable Disease Not Available;Not Available NO;NO Alive;Alive 2011;2012 MALE Lung Bronchioloalveolar Carcinoma Mucinous No C34.3 8253/3 C34.3 YES Not Available Not Available Not Available Not Available Not Available 11 Not Available Not Available Not Available Not Available Not Available Not Available Not Available Not Available Not Available Not Available 40 No 7552 Not Available TUMOR FREE Not Available Not Available Not Available 82 81 Not Available YES WHITE Not Available NA Not Available Not Applicable Not Applicable Not Applicable Not Applicable Not Applicable Not Applicable Not Applicable Not Applicable Not Applicable Stage IB Not Applicable Not Applicable Not Applicable 6th Not Applicable Not Applicable Not Applicable MX N0 T2 1985 NO YES 97 3 Lung Alive 2011 2007 Not Available

740 Alive T2a N0 M0 Stage IB NA 58 L-Upper Not Applicable TCGA-86-8073 cd902d08-215e-4bd0-88e4-4fd01ab43cbf 13 -21214 Not Applicable 0 13 Not Available Lung Adenocarcinoma Not Available NA Not Available Not Available NO Not Available Not Available Not Available NO Not Available NOT HISPANIC OR LATINO NA Not Available Not Available Not Available Not Available TCGA-86-8073-F66082 FD02835A-886E-4E52-B3D1-53DCF8D4BB81 2 Not Applicable Not Applicable Not Applicable 740 Not Applicable 0 Scheduled Follow-up Submission Complete Remission/Response 100 NO 10 Not Available NO Other TUMOR FREE NO Complete Remission/Response Not Available NO Alive 2014 MALE Lung Bronchioloalveolar Carcinoma Nonmucinous No C34.1 8250/3 C34.1 YES Not Available NO Not Available Not Available Peripheral Lung 6 Not Available Not Available Not Available Not Available Not Available Not Available Not Available Not Available Not Available Unknown 40 No 8073 Not Available TUMOR FREE Not Available Not Available Unknown Not Available Not Available Complete Remission/Response NO WHITE Unknown NA R0 Not Applicable Not Applicable Not Applicable Not Applicable Not Applicable Not Applicable Not Applicable Not Applicable Not Applicable Stage IB Not Applicable Not Applicable Not Applicable 7th Not Applicable Not Applicable Not Applicable M0 N0 T2a Not Available YES NO 86 2 Lung Alive 2012 2011 1970

354 Alive T1 N2 MX Stage IIIA NA 68 R-Upper Not Applicable TCGA-49-6761 4cd3d483-2283-4c6a-a57a-444216119d34 2 -24849 Not Applicable 0 1 Not Available Lung Adenocarcinoma 1 NA Not Available Not Available Not Available Not Available Not Available Not Available Not Available Not Available NOT HISPANIC OR LATINO NA NO NO Not Available Not Available TCGA-49-6761-F22056 8ad6b3d6-48a1-4f9e-8ea6-cf0d7394cdf6 23 Not Applicable Not Applicable Not Applicable 354 Not Applicable Not Available Scheduled Follow-up Submission Not Available Not Available Not Available 2 Not Available NO Not Available Not Available NO Complete Remission/Response Not Available NO Alive 2012 FEMALE Lung Adenocarcinoma- Not Otherwise Specified (NOS) No C34.1 8140/3 C34.1 YES Not Available Not Available Not Available Not Available Not Available 3 Not Available Not Available Not Available Not Available Not Available Not Available Not Available Not Available Not Available Not Available 50 Yes 6761 Not Available TUMOR FREE 64 56 Not Available 64 52 Not Available YES WHITE Not Available NA R0 Not Applicable Not Applicable Not Applicable Not Applicable Not Applicable Not Applicable Not Applicable Not Applicable Not Applicable Stage IIIA Not Applicable Not Applicable Not Applicable 6th Not Applicable Not Applicable Not Applicable MX N2 T1 Not Available NO YES 49 4 Lung Alive 2012 2009 Not Available

603 Alive T1b N1 MX Stage IIA NA 78 L-Lower Not Applicable TCGA-91-A4BD 0D980584-4484-4063-8243-C3CD67F6F588 5 -28544 Not Applicable 0 218 Not Available Lung Adenocarcinoma 94 NA 1 Not Available NO Not Available Not Available Not Available Not Available Not Available NOT HISPANIC OR LATINO NA Not Available Not Available Not Available Not Available TCGA-91-A4BD-F58335 13A3B033-52C9-4C1F-B2FB-196BBE0A75D9 7 Not Applicable Not Applicable Not Applicable 603 Not Applicable Not Evaluated Scheduled Follow-up Submission Complete Remission/Response Not Evaluated NO 4 Not Available NO Not Available TUMOR FREE NO Complete Remission/Response Not Available NO Alive 2014 MALE Lung Adenocarcinoma- Not Otherwise Specified (NOS) No C34.3 8140/3 C34.3 YES Unknown NO Not Available Not Available Not Available 2 Not Available Not Available Not Available Not Available Not Available Not Available Not Available Not Available Not Available NO Not Available No A4BD Other TUMOR FREE 70 92 NO Not Available Not Available Complete Remission/Response YES WHITE NO NA RX Not Applicable Not Applicable Not Applicable Not Applicable Not Applicable Not Applicable Not Applicable Not Applicable Not Applicable Stage IIA Not Applicable Not Applicable Not Applicable 7th Not Applicable Not Applicable Not Applicable MX N1 T1b 1981 YES NO 91 3 Lung Alive 2013 2012 Not Available

0 Alive T2 N0 M0 Stage IB NA 62 L-Upper Not Applicable TCGA-05-4410 d28e465c-e553-4c53-8bb7-3d8199b9a8a5 22 -22888 Not Applicable 0 0 Not Available Lung Adenocarcinoma Not Available NA Not Available Not Available Not Available Not Available Not Available Not Available Not Available Not Available Not Available NA MALE Lung Adenocarcinoma Mixed Subtype No C34.1 8255/3 C34.1 YES Not Available Not Available Not Available Not Available Not Available 7 Not Available Not Available Not Available Not Available Not Available Not Available Not Available Not Available Not Available Not Available 98 Yes 4410 Not Available Not Available Not Available Not Available Not Available Not Available Not Available Not Available Not Available Not Available Not Available NA RX Not Applicable Not Applicable Not Applicable Not Applicable Not Applicable Not Applicable Not Applicable Not Applicable Not Applicable Stage IB Not Applicable Not Applicable Not Applicable 6th Not Applicable Not Applicable Not Applicable M0 N0 T2 2007 NO YES 05 4 Lung Alive 2010 2007 1958

460 Dead T2 N0 M0 Stage IB NA 85 R-Upper Not Applicable TCGA-50-5939 12b79b4f-c28c-418e-a1e7-e622f33fdcc2 2 -31236 460 0 Not Available Not Available Lung Adenocarcinoma Not Available NA Not Available Not Available Not Available Not Available Not Available Not Available Not Available Not Available NOT HISPANIC OR LATINO NA Not Available Not Available Not Available Not Available TCGA-50-5939-F32124 2105f694-4ccf-4c6e-8eba-917a55c789e3 17 Not Applicable Not Applicable 460 Not Available Not Applicable Not Available Scheduled Follow-up Submission Complete Remission/Response Not Available Not Available 5 Not Available NO Not Available TUMOR FREE NO Complete Remission/Response Not Available NO Dead 2012 MALE Lung Adenocarcinoma Mixed Subtype No C34.1 8255/3 C34.1 YES Not Available Not Available Not Available Not Available Not Available 7 Not Available Not Available Not Available Not Available Not Available Not Available Not Available Not Available Not Available Not Available 15 No 5939 Not Available TUMOR FREE Not Available Not Available Not Available Not Available Not Available Not Available Not Available WHITE Not Available NA Not Available Not Applicable Not Applicable Not Applicable Not Applicable Not Applicable Not Applicable Not Applicable Not Applicable Not Applicable Stage IB Not Applicable Not Applicable Not Applicable 6th Not Applicable Not Applicable Not Applicable M0 N0 T2 1968 NO YES 50 3 Lung Dead 2011 2008 Not Available

257 Dead T2 N2 M0 Stage IIIA NA 58 R-Lower Not Applicable TCGA-50-5936 ef42ae4c-a108-468e-beed-437cf3cf2962 2 -21238 257 0 Not Available Not Available Lung Adenocarcinoma Not Available NA TCGA-50-5936-D41910 26C486FA-3DCE-4BA5-A30E-544DBBDDEE25 Not Available 3 257 188 TAXOL Clinical Progressive Disease 4 Not Available Not Available Not Available Not Available Not Applicable Not Available Not Available NO Chemotherapy Not Available Not Available Not Available NO 2013 Not Available Not Available YES Not Available Not Available Not Available Not Available Not Available NOT HISPANIC OR LATINO NA YES NO YES NO TCGA-50-5936-F32110 a515c2f5-9d6e-4d08-a136-8480b38b7e6b 17 177 Not Available 257 Not Available 150 Not Available Scheduled Follow-up Submission Progressive Disease Not Available NO 5 Not Available YES Not Available WITH TUMOR NO Progressive Disease Biopsy with Histologic Confirmation;Convincing Imaging NO Dead 2012 MALE Mucinous (Colloid) Carcinoma No C34.30 8480/3 C34.3 YES Not Available YES YES G12C Not Available 7 Not Available Not Available Not Available Not Available Not Available Not Available Not Available Not Available Not Available Not Available Not Available No 5936 Not Available Not Available Not Available Not Available Not Available Not Available Not Available Not Available Not Available WHITE Not Available NA Not Available Not Applicable Not Applicable Not Applicable Not Applicable Not Applicable Not Applicable Not Applicable Not Applicable Not Applicable Stage IIIA Not Applicable Not Applicable Not Applicable 6th Not Applicable Not Applicable Not Applicable M0 N2 T2 Not Available NO YES 50 Not Available Lung Dead 2011 2007 Not Available

1118 Alive T1a N0 M0 Stage IA NA 50 L-Upper Not Applicable TCGA-99-8028 9759bf3a-74ff-433f-99bb-f9406f222ed2 15 -18542 Not Applicable 0 459 Not Available Lung Adenocarcinoma 71 NA 0 Not Available YES Not Available Not Available RT-PCR YES Not Available NOT HISPANIC OR LATINO NA Not Available Not Available Not Available Not Available TCGA-99-8028-F57699 ADD85632-529E-47C6-BEDA-7857D1A5C780 17 Not Applicable Not Applicable Not Applicable 1118 Not Applicable Not Available Scheduled Follow-up Submission Complete Remission/Response Not Available NO 4 Not Available NO Not Available TUMOR FREE NO Complete Remission/Response Not Available NO Alive 2014 FEMALE Lung Adenocarcinoma- Not Otherwise Specified (NOS) No C34.1 8140/3 C34.1 YES Not Evaluated YES YES G12C Unknown 6 Not Available Not Available Not Available Not Available Not Available Not Available Not Available Not Available Not Available NO 30 No 8028 Preoperative TUMOR FREE 91 98 NO 91 94 Not Available YES BLACK OR AFRICAN AMERICAN NO NA R0 Not Applicable Not Applicable Not Applicable Not Applicable Not Applicable Not Applicable Not Applicable Not Applicable Not Applicable Stage IA Not Applicable Not Applicable Not Applicable 7th Not Applicable Not Applicable Not Applicable M0 N0 T1a 2011 NO YES 99 4 Lung Alive 2012 2011 1981

628 Dead T2 N2 M0 Stage IIIA NA 77 R-Lower Not Applicable TCGA-64-1677 8a16ddb3-4861-4128-bed9-0b1f94fe8cf7 18 -28482 628 0 Not Available Not Available Lung Adenocarcinoma Not Available NA TCGA-64-1677-D8693;TCGA-64-1677-D8696;TCGA-64-1677-D8692 654eaa9e-2e91-4ef6-9e36-02a237406432;8a35805b-e7b8-4f10-8aae-d78f856a372e;a4d38c0d-dd9a-4143-ae98-9be774d485c5 Not Available;Not Available;Not Available 18;18;18 121;Not Available;121 51;Not Available;51 Gemcitabine;Not Available;Carboplatin Not Available;Not Available;Not Available 2;2;2 4;2;4 1600;Not Available;425 mg;Not Available;mg ADJUVANT;RECURRENCE;ADJUVANT Not Applicable;Not Applicable;Not Applicable 1;2;1 IV;Not Available;IV NO;NO;NO Chemotherapy;Chemotherapy;Chemotherapy Not Available;Not Available;Not Available Not Available;Not Available;Not Available Not Available;Not Available;Not Available Not Available;Not Available;Not Available 2011;2011;2011 Not Available Not Available Not Available Not Available Not Available Not Available Not Available Not Available NOT HISPANIC OR LATINO NA YES YES NO NO TCGA-64-1677-F8691 52f57fe8-b728-4758-942d-efc01c5cbf0e 18 Not Available Not Available 628 Not Available 360 Not Available Not Available Not Available 0 Not Available 2 Not Available YES Not Available Not Available YES Not Available Not Available NO Dead 2011 FEMALE Lung Adenocarcinoma- Not Otherwise Specified (NOS) No C34.1 8140/3 C34.1 YES 0 Not Available Not Available Not Available Not Available 2 Not Available Not Available Not Available Not Available Not Available Not Available Not Available Not Available Not Available Not Available 50 Yes 1677 Not Available Not Available Not Available Not Available Not Available Not Available Not Available Not Available Not Available WHITE Not Available NA Regional site TCGA-64-1677-R8694 45477fba-0eb8-4966-8c18-d8e9b539d9fc 2 18 448 417 Not Available 2 31 Not Available NO EXTERNAL BEAM Not Applicable RECURRENCE Not Available cGy 2011 R0 Not Applicable Not Applicable Not Applicable Not Applicable Not Applicable Not Applicable Not Applicable Not Applicable Not Applicable Stage IIIA Not Applicable Not Applicable Not Applicable Not Available Not Applicable Not Applicable Not Applicable M0 N2 T2 Not Available NO YES 64 2 Lung Dead 2011 2005 1955

852 Alive T1 NX Stage IA NA FPPP TCGA 61 L-Upper Not Applicable TCGA-44-5645 ddeacccf-0953-4952-8a4e-c2617f2e7bcc 13 -22471 Not Applicable 0 208 Not Available Lung Adenocarcinoma 82 NA Not Available Not Available Not Available Not Available Not Available Not Available Not Available Not Available NOT HISPANIC OR LATINO NA Not Available;Not Available Not Available;Not Available Not Available;NO Not Available;Not Available TCGA-44-5645-F14601;TCGA-44-5645-F38800 9d996467-3fd5-408f-9225-258a86d0bd01;241EC925-44CC-4331-B9AF-BF5E912AF547 4;24 Not Applicable;Not Applicable Not Applicable;Not Applicable Not Applicable;Not Applicable 383;852 Not Applicable;Not Applicable Not Available;Not Available Scheduled Follow-up Submission;Scheduled Follow-up Submission Complete Remission/Response;Complete Remission/Response Not Available;Not Available Not Available;NO 8;12 Not Available;Not Available NO;NO Not Available;Not Available TUMOR FREE;TUMOR FREE NO;NO Complete Remission/Response;Complete Remission/Response Not Available;Not Available NO;NO Alive;Alive 2011;2012 FEMALE Lung Adenocarcinoma- Not Otherwise Specified (NOS) No C34.1 8140/3 C34.1 YES Not Available Not Available Not Available Not Available Not Available 4 Not Available Not Available Not Available Not Available Not Available Not Available Not Available Not Available Not Available Not Available 5 No 5645 Not Available TUMOR FREE Not Available Not Available Not Available 90 100 Not Available YES BLACK OR AFRICAN AMERICAN Not Available NA Not Available Not Applicable Not Applicable Not Applicable Not Applicable Not Applicable Not Applicable Not Applicable Not Applicable Not Applicable Stage IA Not Applicable Not Applicable Not Applicable 7th Not Applicable Not Applicable Not Applicable Not Available NX T1 1973 YES NO 44 3 Lung Alive 2011 2010 1963

426 Alive T2 N2 M0 Stage IIIA NA 66 R-Lower Not Applicable TCGA-05-4384 9a50e7e4-831d-489f-87d2-979e987561cc 22 -24411 Not Applicable 0 426 Not Available Lung Adenocarcinoma Not Available NA TCGA-05-4384-D36331;TCGA-05-4384-D36332;TCGA-05-4384-D36333;TCGA-05-4384-D36334 281E8624-371D-4473-BA5E-E41E0C21D551;19860FED-A03B-407A-9081-F0CD43C3BD54;8009725B-0627-43CB-B0EB-37B12CCB3848;5B01A1B0-5946-4ECC-A816-E6DC557CF563 Not Available;Not Available;Not Available;Not Available 5;5;5;5 153;153;426;426 61;61;395;245 Cisplatin;Vinorelbine;Cisplatin;Zoledronic acid Clinical Progressive Disease;Clinical Progressive Disease;Clinical Progressive Disease;Clinical Progressive Disease 12;12;12;12 Not Available;Not Available;Not Available;Not Available Not Available;Not Available;Not Available;Not Available Not Available;Not Available;Not Available;Not Available Not Available;Not Available;Not Available;Not Available Not Applicable;Not Applicable;Not Applicable;Not Applicable Not Available;Not Available;Not Available;Not Available Not Available;Not Available;Not Available;Not Available NO;NO;NO;NO Chemotherapy;Chemotherapy;Chemotherapy;Ancillary Not Available;Not Available;Not Available;Not Available Not Available;Not Available;Not Available;Not Available Not Available;Not Available;Not Available;Not Available NO;NO;NO;NO 2012;2012;2012;2012 Not Available Not Available Not Available Not Available Not Available Not Available Not Available Not Available Not Available NA Unknown YES Not Available Not Available TCGA-05-4384-F36330 F4555A19-ABBE-4673-9871-083B5C01710A 29 Not Available Not Available Not Applicable 426 183 Not Available Scheduled Follow-up Submission Progressive Disease Not Available NO 10 Distant Metastasis YES Not Available WITH TUMOR YES Progressive Disease Not Available YES Alive 2012 MALE Lung Adenocarcinoma Mixed Subtype No C34.3 8255/3 C34.3 YES Not Available Not Available Not Available Not Available Not Available 7 Not Available Not Available Not Available Not Available Not Available Not Available Not Available Not Available Not Available Not Available 20 Yes 4384 Not Available Not Available Not Available Not Available Not Available Not Available Not Available Not Available Not Available Not Available Not Available NA Distant Recurrence TCGA-05-4384-R36335 E131951D-3ADE-4C06-9406-088B151B75A3 Not Available 29 214 183 Radiographic Progressive Disease 10 Not Available Not Available NO External Not Applicable Not Available Not Available Gy 2012 RX Not Applicable Not Applicable Not Applicable Not Applicable Not Applicable Not Applicable Not Applicable Not Applicable Not Applicable Stage IIIA Not Applicable Not Applicable Not Applicable 6th Not Applicable Not Applicable Not Applicable M0 N2 T2 1987 NO YES 05 3 Lung Alive 2010 2009 1963

1072 Alive T2a N0 M0 Stage IB NA 62 R-Lower Not Applicable TCGA-86-7955 0d0d83d9-d558-4d38-977b-6f1b2471beda 14 -22772 Not Applicable 0 14 Not Available Lung Adenocarcinoma Not Available NA TCGA-86-7955-D41098;TCGA-86-7955-D41099;TCGA-86-7955-D63518;TCGA-86-7955-D63519 7C0A1FDD-293F-4DF9-AC3D-67AB0630FF20;6E123B02-3E4D-4660-9400-A5BDA061292D;B33DF174-A60F-4916-97D6-315FF1B3CBF3;4F1FAE32-57AF-472B-8BCD-8E1EAB496CDC Not Available;Not Available;Not Available;Not Available 12;12;12;12 274;274;922;922 27;27;823;823 Vepesid;Cisplatin;Gemcitabine;Cisplatin Complete Response;Complete Response;Complete Response;Complete Response 3;3;8;8 Not Available;Not Available;Not Available;Not Available Not Available;Not Available;Not Available;Not Available Not Available;Not Available;Not Available;Not Available Not Available;Not Available;Not Available;Not Available Not Applicable;Not Applicable;Not Applicable;Not Applicable Not Available;Not Available;Not Available;Not Available Not Available;Not Available;Not Available;Not Available NO;NO;NO;NO Chemotherapy;Chemotherapy;Chemotherapy;Chemotherapy Not Available;Not Available;Not Available;Not Available Not Available;Not Available;Not Available;Not Available Not Available;Not Available;Not Available;Not Available NO;NO;NO;NO 2013;2013;2014;2014 1 Not Available NO Not Available Not Available Not Available Not Available Not Available NOT HISPANIC OR LATINO NA Not Available;YES Not Available;YES Not Available;NO Not Available;Not Available TCGA-86-7955-F41096;TCGA-86-7955-F63517 BE3BF32A-D5A9-43DA-BE40-6F8B888E0949;F2DA8AAE-377B-4F5E-8640-51294564B4D4 12;12 Not Applicable;Not Available Not Applicable;Not Available Not Applicable;Not Applicable 508;1072 Not Applicable;820 1;0 Scheduled Follow-up Submission;Scheduled Follow-up Submission Complete Remission/Response;Complete Remission/Response 90;100 NO;NO 3;8 Not Available;Locoregional Recurrence NO;YES Preoperative;Post-Adjuvant Therapy TUMOR FREE;TUMOR FREE YES;YES Complete Remission/Response;Complete Remission/Response Not Available;Not Available YES;YES Alive;Alive 2013;2014 MALE Lung Adenocarcinoma- Not Otherwise Specified (NOS) No C34.3 8140/3 C34.3 YES 90 NO Not Available Not Available Peripheral Lung 3 Not Available Not Available Not Available Not Available Not Available Not Available Not Available Not Available Not Available Not Available Not Available No 7955 Not Available TUMOR FREE Not Available Not Available Not Available Not Available Not Available Not Available NO WHITE Not Available NA Primary Tumor Field;Regional site TCGA-86-7955-R41101;TCGA-86-7955-R63520 AA293A6B-ED04-439A-8F4C-E01F9D5B679E;29EF3999-EA2A-4E8E-A0AF-09FF97FDCE95 Not Available;Not Available 12;12 190;1057 114;1027 Complete Response;Complete Response 3;8 31;18 62;36 NO;NO External;External Not Applicable;Not Applicable Not Available;Not Available Not Available;Not Available Gy;Gy 2013;2014 RX Not Applicable Not Applicable Not Applicable Not Applicable Not Applicable Not Applicable Not Applicable Not Applicable Not Applicable Stage IB Not Applicable Not Applicable Not Applicable 7th Not Applicable Not Applicable Not Applicable M0 N0 T2a Not Available YES NO 86 1 Lung Alive 2012 2011 Not Available

260 Alive T2a N0 M0 Stage IB NA 58 R-Upper Not Applicable TCGA-44-8120 83e38dbd-edab-47f2-b19f-6ea38fc6bece 31 -21188 Not Applicable 0 169 Not Available Lung Adenocarcinoma Not Available NA 0 Not Available Unknown Not Available Not Available Not Available Unknown Not Available NOT HISPANIC OR LATINO NA Not Available Not Available Not Available Not Available TCGA-44-8120-F36757 9CE462FD-8E7F-483D-BB93-B5B2CDFCA189 13 Not Applicable Not Applicable Not Applicable 260 Not Applicable 0 Scheduled Follow-up Submission Complete Remission/Response Not Available NO 11 Not Available NO Preoperative TUMOR FREE NO Complete Remission/Response Not Available NO Alive 2012 MALE Lung Adenocarcinoma- Not Otherwise Specified (NOS) No C34.1 8140/3 C34.1 YES Not Available Unknown Not Available Not Available Not Available 5 Not Available Not Available Not Available Not Available Not Available Not Available Not Available Not Available Not Available NO 30 No 8120 Preoperative TUMOR FREE 100 101 NO 98 97 Complete Remission/Response YES BLACK OR AFRICAN AMERICAN NO NA Not Available Not Applicable Not Applicable Not Applicable Not Applicable Not Applicable Not Applicable Not Applicable Not Applicable Not Applicable Stage IB Not Applicable Not Applicable Not Applicable 7th Not Applicable Not Applicable Not Applicable M0 N0 T2a Not Available YES NO 44 5 Lung Alive 2012 2011 1981

97 Dead T2 N0 M0 Stage IB NA FPPP TCGA 43 R-Upper Not Applicable TCGA-44-2666 e16ca88f-488b-40f0-9169-e5a62482a2ff 8 -15970 97 0 97 97 Lung Adenocarcinoma 66 NA Not Available Not Available Not Available Not Available Not Available Not Available Not Available Not Available HISPANIC OR LATINO NA NO NO NO NO TCGA-44-2666-F5304 3f3411e8-5a55-4b73-b4e9-42c1e7bfdf7d 12 Not Applicable Not Applicable 97 97 Not Applicable Not Available Not Available Not Available Not Available Not Available 10 Not Available NO Not Available WITH TUMOR NO Not Available Not Available NO Dead 2010 MALE Lung Adenocarcinoma- Not Otherwise Specified (NOS) No C34.1 8140/3 C34.1 YES Not Available NO Not Available Not Available Not Available 10 Not Available Not Available Not Available Not Available Not Available Not Available Not Available Not Available Not Available Not Available 30 No 2666 Not Available WITH TUMOR Not Available Not Available Not Available 86 87 Not Available YES WHITE Not Available NA R0 Not Applicable Not Applicable Not Applicable Not Applicable Not Applicable Not Applicable Not Applicable Not Applicable Not Applicable Stage IB Not Applicable Not Applicable Not Applicable 6th Not Applicable Not Applicable Not Applicable M0 N0 T2 Not Available YES NO 44 2 Lung Dead 2010 2009 1979

829 Alive T1a N0 M0 Stage IA NA 74 R-Upper Not Applicable TCGA-50-8460 497cc506-03fe-4ffa-860b-5a12adb1bc7d 18 -27270 Not Applicable 0 105 Not Available Lung Adenocarcinoma Not Available NA Not Available Not Available YES Not Available Not Available Not Available Not Available Not Available NOT HISPANIC OR LATINO NA Not Available Not Available Not Available Not Available TCGA-50-8460-F69335 D2F418F0-13A0-45DB-B9B4-F1D07DFA9D55 5 Not Applicable Not Applicable Not Applicable 829 Not Applicable Not Available Scheduled Follow-up Submission Complete Remission/Response Not Available NO 1 Not Available NO Not Available TUMOR FREE NO Complete Remission/Response Not Available YES Alive 2015 MALE Lung Papillary Adenocarcinoma No C34.1 8260/3 C34.1 YES Not Available YES NO Not Available Not Available 10 Not Available Not Available Not Available Not Available Not Available Not Available Not Available Not Available Not Available NO Not Available No 8460 Not Available Unknown Not Available Not Available NO Not Available Not Available Unknown NO WHITE YES NA Primary Tumor Field TCGA-50-8460-R42675 1EB8F1A2-25A0-4301-B2A6-731A17BE04FB Not Available 26 49 49 Complete Response 4 Not Available Not Available NO Internal Not Applicable Not Available Not Available Not Available 2013 R0 Not Applicable Not Applicable Not Applicable Not Applicable Not Applicable Not Applicable Not Applicable Not Applicable Not Applicable Stage IA Not Applicable Not Applicable Not Applicable 7th Not Applicable Not Applicable Not Applicable M0 N0 T1a Not Available YES NO 50 Unknown Lung Alive 2012 2012 Not Available

674 Alive T1b N0 M0 Stage IA NA 73 R-Upper Not Applicable TCGA-55-8208 77f8c850-b4e6-46a1-ab12-a50e0044a0c8 13 -26716 Not Applicable 0 0 Not Available Lung Adenocarcinoma 50 NA 0 Not Available Not Available Not Available Not Available Not Available Not Available Not Available NOT HISPANIC OR LATINO NA Unknown YES Not Available YES TCGA-55-8208-F59045 6F292E9C-8A64-4D70-B169-8AE2D8E32EB1 30 Not Available 536 Not Applicable 674 511 Unknown Scheduled Follow-up Submission Stable Disease Unknown YES 4 Distant Metastasis YES Not Available WITH TUMOR NO Complete Remission/Response Biopsy with Histologic Confirmation;Convincing Imaging NO Alive 2014 FEMALE Lung Adenocarcinoma- Not Otherwise Specified (NOS) No C34.1 8140/3 C34.1 YES 100 Not Available Not Available Not Available Peripheral Lung 6 Not Available Not Available Not Available Not Available Not Available Not Available Not Available Not Available Not Available NO 50 No 8208 Preoperative TUMOR FREE 68 151 Unknown 72 156 Complete Remission/Response YES WHITE Unknown NA R0 Not Applicable Not Applicable Not Applicable Not Applicable Not Applicable Not Applicable Not Applicable Not Applicable Not Applicable Stage IA Not Applicable Not Applicable Not Applicable 7th Not Applicable Not Applicable Not Applicable M0 N0 T1b Not Available YES NO 55 2 Lung Alive 2012 2012 Not Available

409 Dead T1a N0 MX Stage IA NA 67 R-Lower Not Applicable TCGA-44-A4SU BF34664D-423D-4257-99D4-797F7D366455 29 -24820 Not Applicable 0 76 Not Available Lung Adenocarcinoma 74 NA 1 Not Available Not Available Not Available Not Available Not Available Not Available Not Available NOT HISPANIC OR LATINO NA NO NO NO Not Available TCGA-44-A4SU-F49438 D0EC82D0-E3BC-4820-AD56-81831A4CF3EC 3 Not Available Not Available 409 Not Available 260 3 Scheduled Follow-up Submission Progressive Disease Not Evaluated NO 10 Locoregional Recurrence YES Other WITH TUMOR NO Complete Remission/Response Biopsy with Histologic Confirmation;Convincing Imaging NO Dead 2013 FEMALE Lung Adenocarcinoma- Not Otherwise Specified (NOS) No C34.3 8140/3 C34.3 YES Not Available Not Available Not Available Not Available Not Available 1 Not Available Not Available Not Available Not Available Not Available Not Available Not Available Not Available Not Available NO 50 No A4SU Preoperative TUMOR FREE 100 95 NO 100 88 Complete Remission/Response YES WHITE NO NA R0 Not Applicable Not Applicable Not Applicable Not Applicable Not Applicable Not Applicable Not Applicable Not Applicable Not Applicable Stage IA Not Applicable Not Applicable Not Applicable 7th Not Applicable Not Applicable Not Applicable MX N0 T1a 2011 YES NO 44 4 Lung Alive 2013 2012 1961

2109 Alive T1 N0 M0 Stage IA NA 56 R-Lower Not Applicable TCGA-55-6980 2f0710f4-827b-45b2-9b7e-c27385b481a7 27 Not Available Not Applicable 0 67 Not Available Lung Adenocarcinoma Not Available NA Not Available Not Available NO Not Available Not Available Not Available NO Not Available Not Available NA Not Available Not Available Not Available Not Available TCGA-55-6980-F46642 280D1471-7D87-47DB-AAD2-6281B990E361 13 Not Applicable Not Applicable Not Applicable 2109 Not Applicable Not Evaluated Scheduled Follow-up Submission Complete Remission/Response Not Evaluated NO 8 Not Available NO Not Evaluated TUMOR FREE NO Complete Remission/Response Not Available NO Alive 2013 MALE Lung Bronchioloalveolar Carcinoma Nonmucinous No C34.3 8252/3 C34.3 YES Not Available NO Not Available Not Available Not Available 7 Not Available Not Available Not Available Not Available Not Available Not Available Not Available Not Available Not Available Not Available Not Available No 6980 Not Available TUMOR FREE Not Available Not Available Not Available Not Available Not Available Not Available Not Available WHITE Not Available NA R0 Not Applicable Not Applicable Not Applicable Not Applicable Not Applicable Not Applicable Not Applicable Not Applicable Not Applicable Stage IA Not Applicable Not Applicable Not Applicable 6th Not Applicable Not Applicable Not Applicable M0 N0 T1 Not Available NO YES 55 1 Lung Alive 2011 2006 Not Available

15 Alive T2 N0 M0 Stage IB NA 76 R-Lower Not Applicable TCGA-NJ-A55A B8B5ED35-B299-4C1E-9226-5CA055DC7A43 27 -28022 Not Applicable 0 3 Not Available Lung Adenocarcinoma Not Available NA Not Available Not Available NO Not Available Not Available Not Available NO Not Available NOT HISPANIC OR LATINO NA Not Available;Not Available Not Available;Not Available Not Available;Not Available Not Available;Not Available TCGA-NJ-A55A-F50896;TCGA-NJ-A55A-F70649 C6E65920-A007-4160-A4CD-196B5F996C14;24A27E33-8F11-4A44-BDB0-D081C9BBFBE0 1;27 Not Applicable;Not Applicable Not Applicable;Not Applicable Not Applicable;Not Applicable 8;15 Not Applicable;Not Applicable Not Available;Not Available Scheduled Follow-up Submission;Scheduled Follow-up Submission Stable Disease;Stable Disease Not Available;Not Available NO;YES 11;2 Not Available;Not Available NO;NO Not Available;Not Available TUMOR FREE;TUMOR FREE NO;NO Stable Disease;Stable Disease Not Available;Not Available NO;NO Alive;Alive 2013;2015 FEMALE Lung Adenocarcinoma- Not Otherwise Specified (NOS) No C34.3 8550/3 C34.3 YES Not Available NO Not Available Not Available Not Available 9 Not Available Not Available Not Available Not Available Not Available Not Available Not Available Not Available Not Available NO 25 No A55A Not Available TUMOR FREE Not Available Not Available NO Not Available Not Available Stable Disease NO WHITE NO NA Not Available Not Applicable Not Applicable Not Applicable Not Applicable Not Applicable Not Applicable Not Applicable Not Applicable Not Applicable Stage IB Not Applicable Not Applicable Not Applicable 6th Not Applicable Not Applicable Not Applicable M0 N0 T2 1983 Not Available Not Available NJ 3 Lung Alive 2013 2009 Not Available

488 Dead T2a N1 M0 Stage IIA NA 70 L-Upper Not Applicable TCGA-49-6742 21fb46f9-4bbb-441c-af19-a687e9138344 9 -25839 Not Applicable 0 Discrepancy Not Available Lung Adenocarcinoma Not Available NA TCGA-49-6742-D14744;TCGA-49-6742-D16680;TCGA-49-6742-D16679;TCGA-49-6742-D17830 3b0101a9-80a8-4824-a268-205c46c9ac31;f59fb2c2-a314-4463-af34-81a5299331fc;e4f03b6f-8150-4976-8b9a-1783d3aaaf2e;23b5df36-e5bf-4b57-a0db-04bc5b0c0c9c Not Available;Not Available;Not Available;Not Available 9;23;23;26 184;184;184;396 31;31;31;335 Alimta;Pemetrexed;Cisplatin;MDX-1106 clinical trial Not Available;Not Available;Not Available;Not Available 8;9;9;10 3;3;3;1 Not Available;Not Available;Not Available;Not Available Not Available;Not Available;Not Available;Not Available ADJUVANT;ADJUVANT;ADJUVANT;PROGRESSION Not Applicable;Not Applicable;Not Applicable;Not Applicable 1;1;1;2 IV;IV;IV;IV NO;NO;NO;NO Chemotherapy;Chemotherapy;Chemotherapy;Immunotherapy Not Available;Not Available;Not Available;Not Available Not Available;Not Available;Not Available;Not Available Not Available;Not Available;mg/m2;Not Available Not Available;Not Available;Not Available;Not Available 2011;2011;2011;2011 Not Available Not Available Not Available Not Available Not Available Not Available Not Available Not Available NOT HISPANIC OR LATINO NA YES;YES NO;NO NO;NO YES;YES TCGA-49-6742-F14741;TCGA-49-6742-F70579 e6565afe-c87f-4fa9-94e1-29b1e25d0ab8;E22C1C68-2566-426D-AEB5-065CFD61A1C2 9;25 Not Available;Not Available 214;214 Not Applicable;488 445;Not Available 214;214 Not Available;3 Scheduled Follow-up Submission;Scheduled Follow-up Submission Not Available;Progressive Disease Not Available;40 Not Available;NO 8;2 Not Available;Distant Metastasis YES;YES Not Available;Post-Adjuvant Therapy WITH TUMOR;WITH TUMOR YES;YES Progressive Disease;Progressive Disease Not Available;Not Available NO;NO Alive;Dead 2011;2015 MALE Mucinous (Colloid) Carcinoma No C34.1 8480/3 C34.1 YES Not Available YES NO Not Available Not Available 8 Not Available Not Available Not Available Not Available Not Available Not Available Not Available Not Available Not Available Not Available 10 Yes 6742 Not Available TUMOR FREE Not Available Not Available Not Available Not Available Not Available Not Available Not Available WHITE Not Available NA R0 Not Applicable Not Applicable Not Applicable Not Applicable Not Applicable Not Applicable Not Applicable Not Applicable Not Applicable Stage IIA Not Applicable Not Applicable Not Applicable 7th Not Applicable Not Applicable Not Applicable M0 N1 T2a Not Available NO YES 49 3 Lung Alive 2011 2010 Not Available

174 Alive T2a N0 M0 Stage IB NA 52 R-Lower Not Applicable TCGA-67-6215 dbd5b0de-94c9-45dd-afb3-6820a7ecaca2 2 -19310 Not Applicable 0 162 Not Available Lung Adenocarcinoma Not Available NA TCGA-67-6215-D13739;TCGA-67-6215-D13760 f689f65f-4ee1-458b-a0a3-0830819796fc;7199c0c9-6baf-4418-a719-d2c648677db4 Not Available;Not Available 11;12 148;148 85;85 CISPLATIN;ALIMTA Not Available;Not Available 7;7 4;4 135;900 mg;mg ADJUVANT;ADJUVANT Not Applicable;Not Applicable 1;1 IV;IV NO;NO Chemotherapy;Chemotherapy Not Available;Not Available Not Available;Not Available Not Available;Not Available Not Available;Not Available 2011;2011 2 Not Available NO Not Available Not Available Not Available NO Not Available NOT HISPANIC OR LATINO NA Not Available Not Available Not Available Not Available TCGA-67-6215-F13585 98f5011f-0421-4bb4-a740-a06c2e8d8794 6 Not Applicable Not Applicable Not Applicable 174 Not Applicable 2 Scheduled Follow-up Submission Not Available Not Available Not Available 7 Not Available NO Adjuvant therapy TUMOR FREE YES Complete Remission/Response Not Available NO Alive 2011 FEMALE Lung Adenocarcinoma- Not Otherwise Specified (NOS) No C34.3 8140/3 C34.3 YES Not Available NO Not Available Not Available Central Lung 6 Not Available Not Available Not Available Not Available Not Available Not Available Not Available Not Available Not Available Not Available Not Available No 6215 Post-Adjuvant Therapy TUMOR FREE Not Available Not Available Not Available Not Available Not Available Not Available Not Available WHITE Not Available NA R0 Not Applicable Not Applicable Not Applicable Not Applicable Not Applicable Not Applicable Not Applicable Not Applicable Not Applicable Stage IB Not Applicable Not Applicable Not Applicable 7th Not Applicable Not Applicable Not Applicable M0 N0 T2a Not Available YES NO 67 1 Lung Alive 2011 2010 Not Available

133 Alive T2a N0 M0 Stage IB NA 80 R-Upper Not Applicable TCGA-38-A44F 258A3A41-211B-4C66-BD3B-EF03014A6967 31 -29534 Not Applicable 0 133 Not Available Lung Adenocarcinoma 35 NA Not Evaluated Not Available NO Not Available Not Available FISH YES Not Available NOT HISPANIC OR LATINO NA Not Available Not Available Not Available Not Available TCGA-38-A44F-F47557 25815E78-A0C8-4308-9087-3C032BCFED78 26 Not Available Not Available Not Applicable Not Available Not Available Not Available Scheduled Follow-up Submission Unknown Not Available YES 8 Not Available Unknown Not Available Not Available Not Available Not Available Not Available Not Available Alive 2013 MALE Lung Adenocarcinoma Mixed Subtype No C34.1 8550/3 C34.1 YES Not Evaluated NO Not Available Not Available Peripheral Lung 10 Not Available Not Available Not Available Not Available Not Available Not Available Not Available Not Available Not Available NO 12 No A44F Not Available TUMOR FREE 71 Not Available NO 72 52 Complete Remission/Response YES WHITE NO NA R0 Not Applicable Not Applicable Not Applicable Not Applicable Not Applicable Not Applicable Not Applicable Not Applicable Not Applicable Stage IB Not Applicable Not Applicable Not Applicable 7th Not Applicable Not Applicable Not Applicable M0 N0 T2a 1977 YES NO 38 3 Lung Alive 2012 2012 1965

2360 Alive T1 N0 M0 Stage IA NA 77 L-Lower Not Applicable TCGA-78-8655 a905d275-9283-4fa6-bbbf-46019bd1bcb7 23 -28379 Not Applicable 0 2360 Not Available Lung Adenocarcinoma 93 NA 0 Not Available NO Not Available Not Available Not Available NO Not Available Not Evaluated NA Not Available Not Available Not Available Not Available TCGA-78-8655-F46154 36A22148-00C4-4964-A2D4-6D5680C6D49F 28 Not Available Not Available Not Applicable Not Available Not Available Not Available Scheduled Follow-up Submission Not Available Not Available YES 8 Not Available Not Available Not Available Not Available Not Available Not Available Not Available Not Available Not Available 2013 FEMALE Lung Adenocarcinoma Mixed Subtype No C34.3 8255/3 C34.3 YES Not Available NO Not Available Not Available Peripheral Lung 10 Not Available Not Available Not Available Not Available Not Available Not Available Not Available Not Available Not Available Unknown 10 No 8655 Preoperative Unknown Not Available Not Available NO 70 128 Complete Remission/Response YES Not Evaluated NO NA R0 Not Applicable Not Applicable Not Applicable Not Applicable Not Applicable Not Applicable Not Applicable Not Applicable Not Applicable Stage IA Not Applicable Not Applicable Not Applicable 6th Not Applicable Not Applicable Not Applicable M0 N0 T1 2001 NO YES 78 4 Lung Alive 2012 2003 1981

889 Alive T2a N0 M0 Stage IB NA 64 Not Available Not Applicable TCGA-44-7671 ebb33753-9d38-4368-9033-3e55f129d00d 17 -23538 Not Applicable 0 535 Not Available Lung Adenocarcinoma 65 NA Not Available Not Available Not Available Not Available Not Available Not Available Not Available Not Available NOT HISPANIC OR LATINO NA Not Available;NO Not Available;Unknown Not Available;NO Not Available;Not Available TCGA-44-7671-F20565;TCGA-44-7671-F38982 ca842d9f-9ec6-407c-85fa-3d6557381f6b;9F7BD92F-14C8-42FF-BA9E-7FBBA4D0A447 17;8 Not Applicable;Not Available Not Applicable;Not Available Not Applicable;Not Applicable 535;889 Not Applicable;883 Not Available;Not Available Scheduled Follow-up Submission;Scheduled Follow-up Submission Complete Remission/Response;Unknown Not Available;Not Available Not Available;NO 1;1 Not Available;Locoregional Recurrence NO;YES Not Available;Not Available TUMOR FREE;WITH TUMOR NO;NO Complete Remission/Response;Complete Remission/Response Not Available;Convincing Imaging NO;NO Alive;Alive 2012;2013 MALE Lung Adenocarcinoma Mixed Subtype No C34.2 8255/3 C34.2 YES Not Available Not Available Not Available Not Available Not Available 1 Not Available Not Available Not Available Not Available Not Available Not Available Not Available Not Available Not Available Not Available Not Available Yes 7671 Not Available TUMOR FREE 103 82 Not Available 97 76 Not Available YES BLACK OR AFRICAN AMERICAN Not Available NA Not Available Not Applicable Not Applicable Not Applicable Not Applicable Not Applicable Not Applicable Not Applicable Not Applicable Not Applicable Stage IB Not Applicable Not Applicable Not Applicable 7th Not Applicable Not Applicable Not Applicable M0 N0 T2a 1995 YES NO 44 3 Lung Alive 2012 2010 Not Available

869 Dead T1 N0 MX Stage IA NA 74 L-Lower Not Applicable TCGA-49-4488 d721bfe0-90e3-415e-b9f3-1a270efa5fbb 9 -27332 869 0 Not Available Not Available Lung Adenocarcinoma Not Available NA Not Available Not Available Not Available Not Available Not Available Not Available Not Available Not Available NOT HISPANIC OR LATINO NA NO YES Not Available Not Available TCGA-49-4488-F14699 0e28bc4e-7453-4d8a-a604-dafc492ada64 9 Not Available 690 869 Not Available 634 Not Available Scheduled Follow-up Submission Not Available Not Available Not Available 8 Not Available YES Not Available Not Available NO Not Available Not Available NO Dead 2011 FEMALE Lung Adenocarcinoma- Not Otherwise Specified (NOS) No C34.3 8140/3 C34.3 YES Not Available Not Available Not Available Not Available Not Available 8 Not Available Not Available Not Available Not Available Not Available Not Available Not Available Not Available Not Available Not Available Not Available No 4488 Not Available Not Available Not Available Not Available Not Available Not Available Not Available Not Available Not Available WHITE Not Available NA Distant site TCGA-49-4488-R14700 f8812bf8-fe15-4311-889d-637f25592e9f 2 9 717 703 Not Available 8 Not Available 3000 NO EXTERNAL BEAM Not Applicable RECURRENCE Not Available cGy 2011 R0 Not Applicable Not Applicable Not Applicable Not Applicable Not Applicable Not Applicable Not Applicable Not Applicable Not Applicable Stage IA Not Applicable Not Applicable Not Applicable 3rd Not Applicable Not Applicable Not Applicable MX N0 T1 1964 NO YES 49 3 Lung Dead 2011 1992 Not Available

14 Alive T2 N0 M0 Stage IB NA 57 R-Upper Not Applicable TCGA-35-3615 e8b7a103-644f-4865-92f8-80b053bd5e17 20 -21076 Not Applicable 0 14 Not Available Lung Adenocarcinoma Not Available NA Not Available Not Available NO Not Available Not Available Not Available NO Not Available NOT HISPANIC OR LATINO NA Not Available Not Available Not Available Not Available TCGA-35-3615-F68919 DEA1DE43-7C05-44A1-ABEF-9C495D3A8150 24 Not Available Not Available Not Applicable Not Available Not Available Not Available Scheduled Follow-up Submission Not Applicable Not Available YES 8 Not Available Not Available Not Available Not Available Not Available Not Available Not Available Not Available Not Available 2015 MALE Lung Adenocarcinoma- Not Otherwise Specified (NOS) No C34.1 8140/3 C34.1 YES Not Available NO Not Available Not Available Peripheral Lung 12 Not Available Not Available Not Available Not Available Not Available Not Available Not Available Not Available Not Available Not Available Not Available No 3615 Not Available TUMOR FREE Not Available Not Available Not Available Not Available Not Available Not Available Not Available WHITE Not Available NA R0 Not Applicable Not Applicable Not Applicable Not Applicable Not Applicable Not Applicable Not Applicable Not Applicable Not Applicable Stage IB Not Applicable Not Applicable Not Applicable Not Available Not Applicable Not Applicable Not Applicable M0 N0 T2 Not Available NO YES 35 1 Lung Alive 2010 2007 Not Available

1632 Dead T2 N0 M0 Stage IB NA 72 R-Upper Not Applicable TCGA-55-6972 c4d1e105-28b7-48df-abef-fbe09782fdb2 27 -26625 Not Applicable 0 1475 Not Available Lung Adenocarcinoma Not Available NA Not Available Not Available NO Not Available Not Available Not Available NO Not Available NOT HISPANIC OR LATINO NA Not Available Not Available Not Available Not Available TCGA-55-6972-F56187 6284B038-E7CE-476E-881E-8376A9D912BD 29 Not Applicable Not Applicable 1632 Not Available Not Applicable Unknown Scheduled Follow-up Submission Complete Remission/Response Unknown NO 1 Not Available NO Not Available TUMOR FREE NO Complete Remission/Response Not Available NO Dead 2014 MALE Lung Papillary Adenocarcinoma No C34.1 8260/3 C34.1 YES Not Available NO Not Available Not Available Not Available 7 Not Available Not Available Not Available Not Available Not Available Not Available Not Available Not Available Not Available Not Available Not Available No 6972 Not Available TUMOR FREE Not Available Not Available Not Available Not Available Not Available Not Available Not Available WHITE Not Available NA R0 Not Applicable Not Applicable Not Applicable Not Applicable Not Applicable Not Applicable Not Applicable Not Applicable Not Applicable Stage IB Not Applicable Not Applicable Not Applicable 6th Not Applicable Not Applicable Not Applicable M0 N0 T2 Not Available NO YES 55 2 Lung Alive 2011 2005 Not Available

1301 Alive T2 N1 M0 Stage IIB NA FPPP TCGA 55 R-Upper Not Applicable TCGA-44-2665 fb79c491-7b01-42ae-8369-8364e442e31b 13 -20349 Not Applicable 0 400 Not Available Lung Adenocarcinoma 71 NA TCGA-44-2665-D5300;TCGA-44-2665-D5303;TCGA-44-2665-D5301 166a00a1-1d11-4315-85ec-f0ff6b2c184b;180ce2c6-d1aa-416d-bcd6-2ecd63c5793d;b9858076-20a0-4a78-b334-9965e070182e Not Available;Not Available;Not Available 13;13;13 308;140;140 77;77;77 Bevacizumab;Pemetrexed disodium;Cisplatin Not Available;Not Available;Not Available 10;10;10 12;4;4 1139;910;137 mg;mg;mg ADJUVANT;ADJUVANT;ADJUVANT Not Applicable;Not Applicable;Not Applicable 1;1;1 IV;IV;IV NO;NO;NO Targeted Molecular therapy;Chemotherapy;Chemotherapy Not Available;Not Available;Not Available 12988;3625;545 mg;mg;mg Not Available;Not Available;Not Available 2010;2010;2010 1 Not Available Not Available Not Available Not Available Not Available Not Available Not Available NOT HISPANIC OR LATINO NA Not Available;Not Available;Not Available Not Available;Not Available;Not Available Not Available;Not Available;Not Available Not Available;Not Available;Not Available TCGA-44-2665-F5298;TCGA-44-2665-F15326;TCGA-44-2665-F40281 9ee191ec-e1cb-48f8-a661-441671526c98;628830b0-7076-423a-a197-1167e6b16fe7;3AD91BAD-E04E-4CDB-A793-F338823FC89E 13;18;15 Not Applicable;Not Applicable;Not Applicable Not Applicable;Not Applicable;Not Applicable Not Applicable;Not Applicable;Not Applicable 400;706;1301 Not Applicable;Not Applicable;Not Applicable 0;0;0 Not Available;Scheduled Follow-up Submission;Scheduled Follow-up Submission Stable Disease;Stable Disease;Complete Remission/Response Not Available;Not Available;Not Available Not Available;Not Available;NO 10;8;2 Not Available;Not Available;Not Available NO;NO;NO Post-Adjuvant Therapy;Post-Adjuvant Therapy;Other TUMOR FREE;TUMOR FREE;TUMOR FREE YES;Not Available;YES Stable Disease;Not Available;Stable Disease Not Available;Not Available;Not Available NO;Not Available;NO Alive;Alive;Alive 2010;2011;2013 FEMALE Lung Adenocarcinoma- Not Otherwise Specified (NOS) No C34.1 8140/3 C34.1 YES Not Available NO Not Available Not Available Not Available 10 Not Available Not Available Not Available Not Available Not Available Not Available Not Available Not Available Not Available Not Available Not Available No 2665 Pre-Adjuvant Therapy TUMOR FREE 79 105 Not Available 79 114 Not Available YES WHITE Not Available NA R0 Not Applicable Not Applicable Not Applicable Not Applicable Not Applicable Not Applicable Not Applicable Not Applicable Not Applicable Stage IIB Not Applicable Not Applicable Not Applicable 6th Not Applicable Not Applicable Not Applicable M0 N1 T2 Not Available YES NO 44 1 Lung Alive 2010 2009 Not Available

1148 Alive T2b N1 M0 Stage IIB NA 61 R-Upper Not Applicable TCGA-86-8054 0fe909ea-d52c-4b71-816f-57f62d4a5744 2 -22584 Not Applicable 0 19 Not Available Lung Adenocarcinoma Not Available NA TCGA-86-8054-D41093;TCGA-86-8054-D41094 AB80A551-9E5E-4095-8852-00599B1C8859;7BBDF359-8AE5-4BBC-9539-38B87210A586 Not Available;Not Available 12;12 136;136 65;65 Paclitaxel;Cisplatin Complete Response;Complete Response 3;3 Not Available;Not Available Not Available;Not Available Not Available;Not Available Not Available;Not Available Not Applicable;Not Applicable Not Available;Not Available Not Available;Not Available NO;NO Chemotherapy;Chemotherapy Not Available;Not Available Not Available;Not Available Not Available;Not Available NO;NO 2013;2013 Not Evaluated Not Available NO Not Available Not Available Not Available NO Not Available NOT HISPANIC OR LATINO NA Not Available;Not Available Not Available;Not Available Not Available;Not Available Not Available;Not Available TCGA-86-8054-F41092;TCGA-86-8054-F65792 8F4BDD45-6498-4773-B290-73CAA6C38692;8E595367-4502-4FF5-B1BC-6AE74B441506 12;30 Not Applicable;Not Applicable Not Applicable;Not Applicable Not Applicable;Not Applicable 745;1148 Not Applicable;Not Applicable 2;2 Scheduled Follow-up Submission;Scheduled Follow-up Submission Complete Remission/Response;Complete Remission/Response 80;80 NO;NO 3;9 Not Available;Not Available NO;NO Preoperative;Preoperative TUMOR FREE;TUMOR FREE YES;YES Complete Remission/Response;Complete Remission/Response Not Available;Not Available NO;NO Alive;Alive 2013;2014 MALE Lung Acinar Adenocarcinoma No C34.1 8550/3 C34.1 YES Not Evaluated NO Not Available Not Available Central Lung 5 Not Available Not Available Not Available Not Available Not Available Not Available Not Available Not Available Not Available Unknown 9 No 8054 Not Evaluated TUMOR FREE Not Available Not Available Unknown Not Available Not Available Unknown NO WHITE Unknown NA R0 Not Applicable Not Applicable Not Applicable Not Applicable Not Applicable Not Applicable Not Applicable Not Applicable Not Applicable Stage IIB Not Applicable Not Applicable Not Applicable 7th Not Applicable Not Applicable Not Applicable M0 N1 T2b Not Available YES NO 86 2 Lung Alive 2012 2011 1994

134 Alive T2b N1 MX Stage IIB NA 59 R-Upper Not Applicable TCGA-69-7978 f462cfef-f60a-4d3e-b92d-b8d8f50b6bb3 9 -21785 Not Applicable 0 70 Not Available Lung Adenocarcinoma Not Available NA Not Available Not Available YES Other Not Available Not Available Not Available Not Available NOT HISPANIC OR LATINO NA Not Available Not Available NO Not Available TCGA-69-7978-F39911 66B2ED66-C09F-466B-8570-8D90725A6E44 11 Not Applicable Not Applicable Not Applicable 134 Not Applicable Not Evaluated Scheduled Follow-up Submission Stable Disease Not Evaluated NO 2 Not Available NO Not Evaluated Discrepancy NO Unknown Not Available NO Alive 2013 MALE Lung Adenocarcinoma Mixed Subtype No C34.1 8255/3 C34.1 YES Not Available Not Available Not Available Not Available Peripheral Lung 4 Not Available Not Available Not Available Not Available Not Available Not Available Not Available Not Available Not Available Not Available 80 No 7978 Not Available WITH TUMOR 91 75 Not Available 88 67 Not Available YES WHITE Not Available NA Not Available Not Applicable Not Applicable Not Applicable Not Applicable Not Applicable Not Applicable Not Applicable Not Applicable Not Applicable Stage IIB Not Applicable Not Applicable Not Applicable 7th Not Applicable Not Applicable Not Applicable MX N1 T2b Not Available NO YES 69 2 Lung Alive 2012 2011 Not Available

882 Alive T2b N1 M0 Stage IIB NA 68 R-Upper Not Applicable TCGA-05-5425 9b4eeecb-6aff-435b-a244-ec362af92b7f 22 -25020 Not Applicable 0 90 Not Available Lung Adenocarcinoma Not Available NA TCGA-05-5425-D36415;TCGA-05-5425-D36417;TCGA-05-5425-D36418;TCGA-05-5425-D36419 B19108E0-0562-45A5-A84F-1EB6D1A472B0;5BEC112E-BA69-4195-810A-A41A10693781;188127D6-E1B8-4C4D-907E-3CEEF9D3C816;48588402-EA64-4934-989B-E4FAB2CEC262 Not Available;Not Available;Not Available;Not Available 5;5;5;5 761;761;669;Not Available 700;700;608;792 Paclitaxel;Carboplatin;Gefitinib;Erlotinib Clinical Progressive Disease;Clinical Progressive Disease;Clinical Progressive Disease;Not Applicable 12;12;12;12 Not Available;Not Available;Not Available;Not Available Not Available;Not Available;Not Available;Not Available Not Available;Not Available;Not Available;Not Available Not Available;Not Available;Not Available;Not Available Not Applicable;Not Applicable;Not Applicable;Not Applicable Not Available;Not Available;Not Available;Not Available Not Available;Not Available;Not Available;Not Available NO;NO;NO;YES Chemotherapy;Chemotherapy;Immunotherapy;Immunotherapy Not Available;Not Available;Not Available;Not Available Not Available;Not Available;Not Available;Not Available Not Available;Not Available;Not Available;Not Available NO;NO;NO;NO 2012;2012;2012;2012 Not Available Not Available Not Available Not Available Not Available Not Available Not Available Not Available Not Available NA Unknown Unknown Not Available Not Available TCGA-05-5425-F36403 714FDB72-0FF1-4CF6-A6F1-ED54B3FF03DD 30 Not Available Not Available Not Applicable 882 486 Not Available Scheduled Follow-up Submission Progressive Disease Not Available NO 10 Not Available YES Not Available WITH TUMOR YES Not Applicable Not Available YES Alive 2012 MALE Lung Adenocarcinoma Mixed Subtype No C34.9 8255/3 C34.9 YES Not Available Not Available Not Available Not Available Not Available 3 Not Available Not Available Not Available Not Available Not Available Not Available Not Available Not Available Not Available Not Available 40 No 5425 Not Available TUMOR FREE Not Available Not Available Not Available Not Available Not Available Not Available Not Available Not Available Not Available NA Distant Recurrence TCGA-05-5425-R36420 8C6DAC9A-FA50-4180-91E8-608C5A264A09 Not Available 30 547 516 Radiographic Progressive Disease 10 Not Available 40 NO External Not Applicable Not Available Not Available Gy 2012 R0 Not Applicable Not Applicable Not Applicable Not Applicable Not Applicable Not Applicable Not Applicable Not Applicable Not Applicable Stage IIB Not Applicable Not Applicable Not Applicable 7th Not Applicable Not Applicable Not Applicable M0 N1 T2b 1985 NO YES 05 3 Lung Alive 2011 2009 1965

952 Dead T3 N1 MX Stage IIIA NA 77 R-Lower Not Applicable TCGA-55-7227 4e7b2af4-b158-41a7-be22-bea227a759e6 6 -28458 Not Applicable 0 53 Not Available Lung Adenocarcinoma Not Available NA TCGA-55-7227-D65705;TCGA-55-7227-D65706;TCGA-55-7227-D65707 5BC0FDF2-9109-4D86-A20D-1322832BE50E;2D4D2D9E-CEB1-4321-A97C-750CB79A547B;DEF1B875-0578-458E-9F26-D60CAAD1BB9B Not Available;Not Available;Not Available 26;26;26 409;409;876 289;289;442 Carboplatin;Pemetrexed;Pemetrexed Stable Disease;Stable Disease;Clinical Progressive Disease 9;9;9 Not Available;Not Available;Not Available Not Available;Not Available;Not Available Not Available;Not Available;Not Available Not Available;Not Available;Not Available Not Applicable;Not Applicable;Not Applicable Not Available;Not Available;Not Available Not Available;Not Available;Not Available NO;NO;NO Chemotherapy;Chemotherapy;Chemotherapy Not Available;Not Available;Not Available Not Available;Not Available;Not Available Not Available;Not Available;Not Available NO;NO;NO 2014;2014;2014 Not Available Not Available Not Available Not Available Not Available Not Available Not Available Not Available NOT HISPANIC OR LATINO NA YES NO NO Not Available TCGA-55-7227-F65704 3B44DFC4-EC36-4F1D-837D-6297BF49D8AF 26 Not Available Not Available 952 Not Available 255 Not Available Scheduled Follow-up Submission Progressive Disease Not Available NO 9 Locoregional Recurrence YES Not Available WITH TUMOR NO Complete Remission/Response Convincing Imaging NO Dead 2014 MALE Lung Adenocarcinoma- Not Otherwise Specified (NOS) No C34.3 8140/3 C34.3 YES Not Available Not Available Not Available Not Available Not Available 10 Not Available Not Available Not Available Not Available Not Available Not Available Not Available Not Available Not Available Not Available Not Available No 7227 Not Available TUMOR FREE Not Available Not Available Not Available Not Available Not Available Not Available Not Available WHITE Not Available NA R0 Not Applicable Not Applicable Not Applicable Not Applicable Not Applicable Not Applicable Not Applicable Not Applicable Not Applicable Stage IIIA Not Applicable Not Applicable Not Applicable 7th Not Applicable Not Applicable Not Applicable MX N1 T3 1993 YES NO 55 3 Lung Alive 2011 2011 1953

385 Dead T3 N2 M0 Stage IIIA NA 45 L-Upper Not Applicable TCGA-49-4490 6c3655ca-8ae7-4ad7-b3e4-0c3ec293fba7 9 -16634 385 0 Not Available Not Available Lung Adenocarcinoma Not Available NA TCGA-49-4490-D14704 c7e179cb-df02-47c2-b3b6-a4877f84b06c Not Available 9 163 79 Cisplatin Not Available 8 2 Not Available Not Available ADJUVANT Not Applicable 1 IV NO Chemotherapy Not Available Not Available Not Available Not Available 2011 Not Available Not Available Not Available Not Available Not Available Not Available Not Available Not Available NOT HISPANIC OR LATINO NA Not Available Not Available Not Available Not Available TCGA-49-4490-F14703 b7af674f-dfd7-41ca-b313-fbe7cf636256 9 Not Applicable Not Applicable 385 Not Available Not Applicable Not Available Scheduled Follow-up Submission Not Available Not Available Not Available 8 Not Available NO Not Available WITH TUMOR YES Not Available Not Available YES Dead 2011 FEMALE Lung Adenocarcinoma- Not Otherwise Specified (NOS) No C34.1 8140/3 C34.1 YES Not Available Not Available Not Available Not Available Not Available 8 Not Available Not Available Not Available Not Available Not Available Not Available Not Available Not Available Not Available Not Available 5 No 4490 Not Available Not Available Not Available Not Available Not Available Not Available Not Available Not Available Not Available WHITE Not Available NA Regional site TCGA-49-4490-R14708 ba1585c0-b517-4b45-af9b-c6bbbf8cb070 1 9 253 240 Not Available 8 Not Available 3000 NO EXTERNAL BEAM Not Applicable ADJUVANT Not Available cGy 2011 Not Available Not Applicable Not Applicable Not Applicable Not Applicable Not Applicable Not Applicable Not Applicable Not Applicable Not Applicable Stage IIIA Not Applicable Not Applicable Not Applicable 4th Not Applicable Not Applicable Not Applicable M0 N2 T3 Not Available NO YES 49 3 Lung Dead 2011 1993 Not Available

741 Alive T1a N0 M0 Stage IA NA 70 L-Lower Not Applicable TCGA-MP-A4TH 7A111B4F-46E9-4E34-8024-0B9FDC5CBCD8 8 -25759 Not Applicable 0 142 Not Available Lung Adenocarcinoma 73 NA Not Evaluated Not Available NO Not Available Not Available Not Available NO Not Available NOT HISPANIC OR LATINO NA Not Available;Not Available Not Available;Not Available Not Available;Not Available Not Available;Not Available TCGA-MP-A4TH-F54545;TCGA-MP-A4TH-F69949 9659993E-D9ED-42F9-8410-F35E8CF2D676;0F71006D-E390-47A0-BCC2-C902FE43812E 19;22 Not Applicable;Not Applicable Not Applicable;Not Applicable Not Applicable;Not Applicable 446;741 Not Applicable;Not Applicable Not Evaluated;Not Evaluated Scheduled Follow-up Submission;Scheduled Follow-up Submission Complete Remission/Response;Complete Remission/Response Not Evaluated;Not Evaluated NO;NO 12;1 Not Available;Not Available NO;NO Not Available;Not Evaluated TUMOR FREE;TUMOR FREE NO;NO Complete Remission/Response;Complete Remission/Response Not Available;Not Available NO;NO Alive;Alive 2013;2015 FEMALE Lung Adenocarcinoma- Not Otherwise Specified (NOS) No C34.3 8140/3 C34.3 YES Not Evaluated NO Not Available Not Available Peripheral Lung 5 Not Available Not Available Not Available Not Available Not Available Not Available Not Available Not Available Not Available NO 20 No A4TH Not Available TUMOR FREE 117 115 NO 110 115 Complete Remission/Response YES WHITE NO NA R0 Not Applicable Not Applicable Not Applicable Not Applicable Not Applicable Not Applicable Not Applicable Not Applicable Not Applicable Stage IA Not Applicable Not Applicable Not Applicable 7th Not Applicable Not Applicable Not Applicable M0 N0 T1a 1982 YES NO MP 3 Lung Alive 2013 2012 1962

773 Alive T2a N0 MX Stage IB NA 76 R-Lower Not Applicable TCGA-55-7815 dd46c83e-6551-485b-b935-f3beed891244 9 -28119 Not Applicable 0 54 Not Available Lung Adenocarcinoma 59 NA TCGA-55-7815-D61496;TCGA-55-7815-D61499 ACA058EE-8C58-4DBD-99EB-8E97EE4F6CC6;A6E48EE4-3A3F-49A9-8FEF-18D08A8F7BE4 Not Available;Not Available 30;30 585;Not Available 493;706 Chemo, NOS;Chemo, NOS Clinical Progressive Disease;Not Applicable 6;6 Not Available;Not Available Not Available;Not Available Not Available;Not Available Not Available;Not Available Not Applicable;Not Applicable Not Available;Not Available Not Available;Not Available NO;YES Chemotherapy;Chemotherapy Not Available;Not Available Not Available;Not Available Not Available;Not Available NO;NO 2014;2014 Not Available Not Available Not Available Not Available Not Available Not Available Not Available Not Available Not Available NA YES;YES NO;YES NO;NO Not Available;NO TCGA-55-7815-F61495;TCGA-55-7815-F61497 65349788-E32F-44C4-B26D-8C918338BD6E;6453FCE4-3AA4-4688-B362-E357741C142B 30;30 Not Available;Not Available Not Available;Not Available Not Applicable;Not Applicable 773;773 466;592 Unknown;Not Available Scheduled Follow-up Submission;Additional New Tumor Event Progressive Disease;Progressive Disease Unknown;Not Available NO;NO 6;6 Locoregional Recurrence;Distant Metastasis YES;YES Not Available;Not Available WITH TUMOR;WITH TUMOR NO;NO Complete Remission/Response;Complete Remission/Response Convincing Imaging;Not Available NO;NO Alive;Alive 2014;2014 MALE Lung Adenocarcinoma- Not Otherwise Specified (NOS) No C34.3 8140/3 C34.3 YES Not Available Not Available Not Available Not Available Not Available 3 Not Available Not Available Not Available Not Available Not Available Not Available Not Available Not Available Not Available Not Available Not Available No 7815 Not Available TUMOR FREE Not Available 76 Not Available Not Available 72 Not Available YES Not Available Not Available NA Distant Recurrence TCGA-55-7815-R61498 3CE21127-ECB2-4DF3-97FC-5630023BAE41 Not Available 30 616 616 Radiographic Progressive Disease 6 Not Available Not Available NO External Not Applicable Not Available Not Available Not Available 2014 R0 Not Applicable Not Applicable Not Applicable Not Applicable Not Applicable Not Applicable Not Applicable Not Applicable Not Applicable Stage IB Not Applicable Not Applicable Not Applicable 7th Not Applicable Not Applicable Not Applicable MX N0 T2a Not Available YES NO 55 1 Lung Alive 2012 2011 Not Available

1081 Dead T3 N2 M0 Stage IIIA NA 77 L-Upper Not Applicable TCGA-49-4494 c6d78e27-e510-4f57-abae-19efa6105a8d 3 -28387 1081 0 Not Available Not Available Lung Adenocarcinoma Not Available NA 1 Not Available Not Available Not Available Not Available Not Available Not Available Not Available NOT HISPANIC OR LATINO NA Not Available Not Available Not Available Not Available TCGA-49-4494-F58840 7264A47D-2B6F-445A-94E5-514CA2BE320F 25 Not Available Not Available 1081 Not Available Not Available 1 Scheduled Follow-up Submission Unknown 90 NO 4 Not Available Unknown Preoperative Unknown Unknown Complete Remission/Response Not Available Unknown Dead 2014 MALE Lung Adenocarcinoma- Not Otherwise Specified (NOS) No C34.1 8140/3 C34.1 YES Not Available Not Available Not Available Not Available Not Available 2 Not Available Not Available Not Available Not Available Not Available Not Available Not Available Not Available Not Available Not Available 50 No 4494 Pre-Adjuvant Therapy WITH TUMOR Not Available Not Available Not Available Not Available Not Available Not Available NO WHITE Not Available NA R0 Not Applicable Not Applicable Not Applicable Not Applicable Not Applicable Not Applicable Not Applicable Not Applicable Not Applicable Stage IIIA Not Applicable Not Applicable Not Applicable 4th Not Applicable Not Applicable Not Applicable M0 N2 T3 1955 NO YES 49 3 Lung Dead 2011 1995 1930

541 Alive T2b N0 M1b Stage IV NA 51 L-Upper Not Applicable TCGA-55-8094 69584d55-18f7-4786-b3cc-cd528500be44 8 -18903 Not Applicable 0 4 Not Available Lung Adenocarcinoma Not Available NA Not Available Not Available YES Not Available Not Available FISH YES Not Available NOT HISPANIC OR LATINO NA Not Available Not Available Not Available Not Available TCGA-55-8094-F66029 AE9F74E2-023B-42DA-8C1F-9C7A9F3C253B 1 Not Applicable Not Applicable Not Applicable 541 Not Applicable Not Available Scheduled Follow-up Submission Partial Remission/Response Not Available NO 10 Not Available NO Not Available WITH TUMOR YES Progressive Disease Not Available YES Alive 2014 MALE Lung Adenocarcinoma- Not Otherwise Specified (NOS) No C34.1 8140/3 C34.1 YES Not Available NO Not Available Not Available Peripheral Lung 7 Not Available Not Available Not Available Not Available Not Available Not Available Not Available Not Available Not Available NO 70 No 8094 Not Available WITH TUMOR Not Available Not Available Unknown Not Available Not Available Unknown Not Available WHITE Unknown NA R0 Not Applicable Not Applicable Not Applicable Not Applicable Not Applicable Not Applicable Not Applicable Not Applicable Not Applicable Stage IV Not Applicable Not Applicable Not Applicable 7th Not Applicable Not Applicable Not Applicable M1b N0 T2b 2011 YES NO 55 4 Lung Alive 2012 2012 1976

446 Alive T3 N2 MX Stage IIIA NA 67 R-Middle Not Applicable TCGA-55-8615 06fe7f3e-4edd-4150-981c-93787ea19b39 11 -24786 Not Applicable 0 15 Not Available Lung Adenocarcinoma 83 NA TCGA-55-8615-D56256;TCGA-55-8615-D56257;TCGA-55-8615-D56258;TCGA-55-8615-D56259 C3E3F94E-7959-47E7-941B-5092013166FF;BCB6362E-FD58-49FE-82E2-ACD98A53F2D7;E08B7831-B7A9-4EC0-9D20-39B36957E6C1;2B2ABC4D-DB6C-4DBC-9DD8-519DEE12F63C Not Available;Not Available;Not Available;Not Available 30;30;30;30 Not Available;Not Available;324;416 89;89;234;234 Carboplatin;Taxol;Carboplatin;Alimta Clinical Progressive Disease;Clinical Progressive Disease;Clinical Progressive Disease;Clinical Progressive Disease 1;1;1;1 Not Available;Not Available;Not Available;Not Available Not Available;Not Available;Not Available;Not Available Not Available;Not Available;Not Available;Not Available Not Available;Not Available;Not Available;Not Available Not Applicable;Not Applicable;Not Applicable;Not Applicable Not Available;Not Available;Not Available;Not Available Not Available;Not Available;Not Available;Not Available NO;NO;NO;NO Chemotherapy;Chemotherapy;Chemotherapy;Chemotherapy Not Available;Not Available;Not Available;Not Available Not Available;Not Available;Not Available;Not Available Not Available;Not Available;Not Available;Not Available NO;NO;NO;NO 2014;2014;2014;2014 Not Evaluated Not Available Unknown Not Available Not Available Not Available Unknown Not Available NOT HISPANIC OR LATINO NA YES YES NO NO TCGA-55-8615-F56254 A2AF20F3-F8C6-4A5A-9E03-9E9583D59194 30 Not Available Not Available Not Applicable 446 224 Unknown Scheduled Follow-up Submission Progressive Disease Unknown NO 1 Distant Metastasis YES Not Available WITH TUMOR YES Complete Remission/Response Convincing Imaging NO Alive 2014 MALE Lung Adenocarcinoma- Not Otherwise Specified (NOS) No C34.2 8140/3 C34.2 YES Not Evaluated Unknown Not Available Not Available Unknown 1 Not Available Not Available Not Available Not Available Not Available Not Available Not Available Not Available Not Available NO 67 No 8615 Not Available TUMOR FREE Not Available Not Available YES Not Available 88 Complete Remission/Response YES WHITE NO NA Distant Recurrence TCGA-55-8615-R56255 D1E015AB-A6F7-4308-8187-F60F9195E63B Not Available 30 446 426 Radiographic Progressive Disease 1 Not Available Not Available NO External Not Applicable Not Available Not Available Not Available 2014 R0 Not Applicable Not Applicable Not Applicable Not Applicable Not Applicable Not Applicable Not Applicable Not Applicable Not Applicable Stage IIIA Not Applicable Not Applicable Not Applicable 7th Not Applicable Not Applicable Not Applicable MX N2 T3 Not Available YES NO 55 2 Lung Alive 2013 2012 1966

478 Dead T2 N2 M0 Stage IIIA NA 42 L-Upper Not Applicable TCGA-50-5051 e1c55bac-bb41-4964-add5-ecd6ba04b58d 25 -15585 Not Applicable 0 455 Not Available Lung Adenocarcinoma Not Available NA TCGA-50-5051-D32021;TCGA-50-5051-D32022 487b2e04-b00a-4221-b759-d6496965c6d9;f3f3dcc2-08bc-4d5d-9293-f608b65f06e9 Not Available;Not Available 15;15 123;123 62;62 Carboplatin;Taxol Partial Response;Partial Response 5;5 Not Available;Not Available Not Available;Not Available Not Available;Not Available Not Available;Not Available Not Applicable;Not Applicable Not Available;Not Available Not Available;Not Available NO;NO Chemotherapy;Chemotherapy Not Available;Not Available Not Available;Not Available Not Available;Not Available NO;NO 2012;2012 Not Available Not Available YES Not Available Not Available Not Available Not Available Not Available NOT HISPANIC OR LATINO NA NO NO NO NO TCGA-50-5051-F32019 c0485b95-039c-4102-9293-f2829790b4f3 15 Not Available Not Available 478 Not Available 184 Not Available Scheduled Follow-up Submission Progressive Disease Not Available NO 5 Distant Metastasis YES Not Available WITH TUMOR YES Progressive Disease Convincing Imaging YES Dead 2012 FEMALE Lung Adenocarcinoma- Not Otherwise Specified (NOS) No C34.1 8140/3 C34.1 YES Not Available YES YES Other Not Available 8 Not Available Not Available Not Available Not Available Not Available Not Available Not Available Not Available Not Available Not Available 30 No 5051 Not Available TUMOR FREE Not Available Not Available Not Available Not Available Not Available Not Available Not Available WHITE Not Available NA Primary Tumor Field TCGA-50-5051-R32020 47ffdbd3-4e1b-4e78-b229-1e3cf0b787d6 01 15 124 71 Not Available 5 33 5490 NO EXTERNAL BEAM Not Applicable ADJUVANT Not Available cGy 2012 R0 Not Applicable Not Applicable Not Applicable Not Applicable Not Applicable Not Applicable Not Applicable Not Applicable Not Applicable Stage IIIA Not Applicable Not Applicable Not Applicable 6th Not Applicable Not Applicable Not Applicable M0 N2 T2 2006 NO YES 50 4 Lung Alive 2011 2007 Not Available

1830 Dead T1 N1 M0 Stage IIA NA 79 L-Lower Not Applicable TCGA-50-5055 cc3d9750-7ce0-489f-86d4-8017b7ebed29 2 -28988 Not Applicable 0 785 Not Available Lung Adenocarcinoma Not Available NA TCGA-50-5055-D40457;TCGA-50-5055-D41755;TCGA-50-5055-D41761;TCGA-50-5055-D41762;TCGA-50-5055-D41763;TCGA-50-5055-D41764 851C0D83-B6C9-427B-A391-46ED77BABB58;7404E87B-2128-4132-AB47-14402C1130F0;9AAF3C36-BC75-459E-9B70-00E485E07138;53DEAEDA-8DF5-4023-9F27-12DD16A6A2C1;C6E4C339-ED92-49E9-BE3C-37BFC3B9D570;3B8F2796-A1BC-4B2A-AC33-8108523F87C6 Not Available;Not Available;Not Available;Not Available;Not Available;Not Available 27;27;27;27;27;27 153;1284;1284;1522;1649;Not Available 31;1135;1135;1284;1522;1649 Not specified;CARBOPLATIN;PACLITAXEL;ALIMTA;TARCEVA;TAXOTERE Clinical Progressive Disease;Clinical Progressive Disease;Clinical Progressive Disease;Clinical Progressive Disease;Clinical Progressive Disease;Clinical Progressive Disease 3;3;3;3;3;3 Not Available;Not Available;Not Available;Not Available;Not Available;Not Available Not Available;Not Available;Not Available;Not Available;Not Available;Not Available Not Available;Not Available;Not Available;Not Available;Not Available;Not Available Not Available;Not Available;Not Available;Not Available;Not Available;Not Available Not Applicable;Not Applicable;Not Applicable;Not Applicable;Not Applicable;Not Applicable Not Available;Not Available;Not Available;Not Available;Not Available;Not Available Not Available;Not Available;Not Available;Not Available;Not Available;Not Available NO;NO;NO;NO;NO;NO Chemotherapy;Chemotherapy;Chemotherapy;Not Available;Chemotherapy;Chemotherapy Not Available;Not Available;Not Available;Not Available;Not Available;Not Available Not Available;Not Available;Not Available;Not Available;Not Available;Not Available Not Available;Not Available;Not Available;Not Available;Not Available;Not Available NO;NO;NO;NO;NO;NO 2013;2013;2013;2013;2013;2013 Not Available Not Available YES Not Available Not Available Not Available Not Available Not Available NOT HISPANIC OR LATINO NA YES YES YES NO TCGA-50-5055-F32023 b971489c-681a-4176-861a-5190efe60761 15 792 Not Available 1830 Not Available 771 Not Available Scheduled Follow-up Submission Progressive Disease Not Available NO 5 Locoregional Recurrence;Distant Metastasis YES Not Available WITH TUMOR NO Stable Disease Biopsy with Histologic Confirmation;Convincing Imaging NO Dead 2012 FEMALE Lung Adenocarcinoma Mixed Subtype No C34.3 8255/3 C34.3 YES Not Available YES NO Not Available Not Available 7 Not Available Not Available Not Available Not Available Not Available Not Available Not Available Not Available Not Available Not Available Not Available No 5055 Not Available WITH TUMOR Not Available Not Available Not Available Not Available Not Available Not Available Not Available WHITE Not Available NA R0 Not Applicable Not Applicable Not Applicable Not Applicable Not Applicable Not Applicable Not Applicable Not Applicable Not Applicable Stage IIA Not Applicable Not Applicable Not Applicable 6th Not Applicable Not Applicable Not Applicable M0 N1 T1 Not Available NO YES 50 Not Available Lung Alive 2011 2008 Not Available

1189 Alive T2b N0 M0 NA 70 R-Middle Not Applicable TCGA-64-1678 f77cf4d3-9829-4fd3-95a1-b29aed9e94b8 15 -25931 Not Applicable 0 1189 Not Available Lung Adenocarcinoma Not Available NA TCGA-64-1678-D4374;TCGA-64-1678-D4372 8e66e3b4-e400-4d79-b659-1633ac4dc232;52f1e822-e632-41d3-8878-da3dcc2611d1 Not Available;Not Available 15;15 132;132 69;69 Carboplatin;Paclitaxel Not Available;Not Available 11;11 4;4 6;200 AUC;mg/m2 ADJUVANT;ADJUVANT Not Applicable;Not Applicable 1;1 IV;IV NO;NO Chemotherapy;Chemotherapy Not Available;Not Available Not Available;Not Available Not Available;Not Available Not Available;Not Available 2010;2010 Not Available Not Available Not Available Not Available Not Available Not Available Not Available Not Available NOT HISPANIC OR LATINO NA Not Available Not Available Not Available Not Available TCGA-64-1678-F4371 5b51f40d-c1d0-43fe-8136-48ede23d4525 15 Not Applicable Not Applicable Not Applicable 1189 Not Applicable Not Available Not Available Complete Remission/Response Not Available Not Available 11 Not Available NO Not Available TUMOR FREE YES Complete Remission/Response Not Available NO Alive 2010 FEMALE Lung Adenocarcinoma- Not Otherwise Specified (NOS) No C34.1 8140/3 C34.1 YES Not Available Not Available Not Available Not Available Not Available 11 Not Available Not Available Not Available Not Available Not Available Not Available Not Available Not Available Not Available Not Available 20 No 1678 Not Available TUMOR FREE Not Available Not Available Not Available Not Available Not Available Not Available Not Available WHITE Not Available NA R0 Not Applicable Not Applicable Not Applicable Not Applicable Not Applicable Not Applicable Not Applicable Not Applicable Not Applicable Discrepancy Not Applicable Not Applicable Not Applicable 6th Not Applicable Not Applicable Not Applicable M0 N0 T2b 1990 NO YES 64 3 Lung Alive 2010 2006 1970

164 Dead T2b N0 M0 Stage IIA NA 67 R-Upper Not Applicable TCGA-97-8174 8c695c24-e7f9-449a-9258-a1bc8a33bf9a 13 -24725 164 0 Not Available Not Available Lung Adenocarcinoma 90 NA TCGA-97-8174-D32911;TCGA-97-8174-D32912 78B19468-4913-4164-B4EF-AF0FD08CB063;093ACF14-8C3A-40FC-9089-FB1AE39C6278 Not Available;Not Available 15;15 102;102 53;53 Vinorelbine;CISplatinum Unknown;Unknown 6;6 Not Available;Not Available Not Available;Not Available Not Available;Not Available Not Available;Not Available Not Applicable;Not Applicable Not Available;Not Available Not Available;Not Available NO;NO Chemotherapy;Chemotherapy Not Available;Not Available Not Available;Not Available Not Available;Not Available NO;NO 2012;2012 0 Not Available YES Not Available Not Available FISH YES Not Available NOT HISPANIC OR LATINO NA MALE Lung Papillary Adenocarcinoma No C34.1 8260/3 C34.1 YES Not Available YES NO Not Available Not Available 6 Not Available Not Available Not Available Not Available Not Available Not Available Not Available Not Available Not Available NO 40 No 8174 Preoperative Not Available 86 99 YES 81 91 Not Available YES WHITE NO NA Not Available Not Applicable Not Applicable Not Applicable Not Applicable Not Applicable Not Applicable Not Applicable Not Applicable Not Applicable Stage IIA Not Applicable Not Applicable Not Applicable 7th Not Applicable Not Applicable Not Applicable M0 N0 T2b 1985 YES NO 97 3 Lung Dead 2012 2011 1965

11 Alive T3 N0 MX Stage IIB NA 62 R-Upper Not Applicable TCGA-55-8506 3ed05070-6633-4de5-9f72-090f93aa2e4d 28 -22850 Not Applicable 0 11 Not Available Lung Adenocarcinoma Not Available NA Not Evaluated Not Available Unknown Not Available Not Available Not Available Unknown Not Available NOT HISPANIC OR LATINO NA FEMALE Lung Adenocarcinoma- Not Otherwise Specified (NOS) No C34.1 8140/3 C34.1 YES Not Evaluated Unknown Not Available Not Available Peripheral Lung 12 Not Available Not Available Not Available Not Available Not Available Not Available Not Available Not Available Not Available NO Not Available No 8506 Not Evaluated Unknown Not Available Not Available Unknown Not Available Not Available Not Applicable Not Available WHITE Unknown NA R0 Not Applicable Not Applicable Not Applicable Not Applicable Not Applicable Not Applicable Not Applicable Not Applicable Not Applicable Stage IIB Not Applicable Not Applicable Not Applicable 7th Not Applicable Not Applicable Not Applicable MX N0 T3 Not Available YES NO 55 2 Lung Alive 2012 2012 1972

244 Dead T2 N2 M0 Stage IIIA NA 69 R-Upper Not Applicable TCGA-78-7536 daf0e08e-1738-43f8-874a-b37d79cbe3a7 19 -25558 244 0 Not Available Not Available Lung Adenocarcinoma Not Available NA 0 Not Available NO Not Available Not Available Not Available NO Not Available Not Available NA NO NO NO NO TCGA-78-7536-F20631 01b8e373-4376-4322-8924-06beb651d367 19 Not Available Not Available 244 Not Available 231 Not Available Scheduled Follow-up Submission Progressive Disease Not Available Not Available 1 Not Available YES Not Available WITH TUMOR NO Progressive Disease Not Available YES Dead 2012 MALE Lung Adenocarcinoma- Not Otherwise Specified (NOS) No C34.1 8140/3 C34.1 YES Not Available NO Not Available Not Available Not Available 1 Not Available Not Available Not Available Not Available Not Available Not Available Not Available Not Available Not Available Not Available 44 No 7536 Preoperative WITH TUMOR Not Available Not Available Not Available Not Available Not Available Not Available Not Available WHITE Not Available NA Regional site TCGA-78-7536-R20632 6428d89d-10a1-4a35-b613-ae1a428fa8ce 1 19 90 55 Not Available 1 25 5000 NO EXTERNAL BEAM Not Applicable ADJUVANT Not Available cGy 2012 R0 Not Applicable Not Applicable Not Applicable Not Applicable Not Applicable Not Applicable Not Applicable Not Applicable Not Applicable Stage IIIA Not Applicable Not Applicable Not Applicable 6th Not Applicable Not Applicable Not Applicable M0 N2 T2 1993 NO YES 78 4 Lung Dead 2012 2001 1949

44 Alive T1a N0 M0 Stage IA NA 61 R-Upper Not Applicable TCGA-99-8032 7e83f9dd-b038-497b-a113-a55605398b7b 15 -22635 Not Applicable 0 44 Not Available Lung Adenocarcinoma 54 NA 0 Not Available YES Not Available Not Available Not Available NO Not Available NOT HISPANIC OR LATINO NA MALE Lung Adenocarcinoma- Not Otherwise Specified (NOS) No C34.1 8140/3 C34.1 YES Not Evaluated YES YES G12C Unknown 6 Not Available Not Available Not Available Not Available Not Available Not Available Not Available Not Available Not Available Unknown 90 No 8032 Preoperative TUMOR FREE Not Available Not Available NO 55 59 Complete Remission/Response YES WHITE NO NA R0 Not Applicable Not Applicable Not Applicable Not Applicable Not Applicable Not Applicable Not Applicable Not Applicable Not Applicable Stage IA Not Applicable Not Applicable Not Applicable 7th Not Applicable Not Applicable Not Applicable M0 N0 T1a Not Available YES NO 99 2 Lung Alive 2012 2011 1966

275 Dead T3 N2 M0 Stage IIIA NA 60 L-Upper Not Applicable TCGA-05-5429 63bd2175-4b7c-44c9-aef3-9efc8f79837b 22 -22066 Not Applicable 0 30 Not Available Lung Adenocarcinoma Not Available NA Not Available Not Available Not Available Not Available Not Available Not Available Not Available Not Available Not Available NA Not Available Not Available Unknown Not Available TCGA-05-5429-F36396 6271312D-642D-4C7C-AF77-B70D79E7B2CC 30 Not Available Not Available 275 Not Available Not Available Not Available Scheduled Follow-up Submission Unknown Not Available NO 10 Not Available Unknown Not Available Unknown Unknown Not Applicable Not Available Unknown Dead 2012 MALE Lung Adenocarcinoma- Not Otherwise Specified (NOS) No C34.1 8140/3 C34.9 YES Not Available Not Available Not Available Not Available Not Available 3 Not Available Not Available Not Available Not Available Not Available Not Available Not Available Not Available Not Available Not Available Not Available No 5429 Not Available TUMOR FREE Not Available Not Available Not Available Not Available Not Available Not Available Not Available Not Available Not Available NA R0 Not Applicable Not Applicable Not Applicable Not Applicable Not Applicable Not Applicable Not Applicable Not Applicable Not Applicable Stage IIIA Not Applicable Not Applicable Not Applicable 7th Not Applicable Not Applicable Not Applicable M0 N2 T3 Not Available NO YES 05 1 Lung Alive 2011 2010 Not Available

6732 Alive T2 N0 MX Stage I NA 41 R-Upper Not Applicable TCGA-49-AARQ 9A2A226E-9605-4214-9320-469305E664E6 19 -15065 Not Applicable 0 6443 Not Available Lung Adenocarcinoma 99.9 NA 1 Not Available Not Available Not Available Not Available Not Available Not Available Not Available NOT HISPANIC OR LATINO NA Not Available Not Available Not Available Not Available TCGA-49-AARQ-F72093 60C40E3D-7A62-4E0D-9272-99ED242A7606 10 Not Applicable Not Applicable Not Applicable 6732 Not Applicable 1 Scheduled Follow-up Submission Complete Remission/Response Not Available NO 4 Not Available NO Preoperative TUMOR FREE NO Complete Remission/Response Not Available NO Alive 2015 FEMALE Lung Adenocarcinoma- Not Otherwise Specified (NOS) No C34.1 8140/3 C34.1 YES 90 Not Available Not Available Not Available Not Available 6 Not Available Not Available Not Available Not Available Not Available Not Available Not Available Not Available Not Available NO 60 No AARQ Preoperative TUMOR FREE 56.8 56.1 NO 83.2 82.2 Complete Remission/Response YES BLACK OR AFRICAN AMERICAN NO NA R0 Not Applicable Not Applicable Not Applicable Not Applicable Not Applicable Not Applicable Not Applicable Not Applicable Not Applicable Stage I Not Applicable Not Applicable Not Applicable 4th Not Applicable Not Applicable Not Applicable MX N0 T2 2007 NO YES 49 4 Lung Alive 2014 1996 Not Available

791 Alive T2 N0 M0 Stage IB NA 71 L-Lower Not Applicable TCGA-05-4426 36bf02f8-c1c8-49f9-a417-adf88a205c70 22 -26084 Not Applicable 0 791 Not Available Lung Adenocarcinoma Not Available NA Not Available Not Available Not Available Not Available Not Available Not Available Not Available Not Available Not Available NA Unknown Unknown Not Available Not Available TCGA-05-4426-F36562 76BC7F31-DA6E-448B-8B2D-68A57B4B9805 2 Not Available Not Available Not Applicable 791 457 Not Available Scheduled Follow-up Submission Complete Remission/Response Not Available NO 11 Not Available YES Not Available TUMOR FREE Unknown Complete Remission/Response Not Available Unknown Alive 2012 MALE Lung Adenocarcinoma Mixed Subtype No C34.3 8255/3 C34.3 YES Not Available Not Available Not Available Not Available Not Available 7 Not Available Not Available Not Available Not Available Not Available Not Available Not Available Not Available Not Available Not Available 20 No 4426 Not Available TUMOR FREE Not Available Not Available Not Available Not Available Not Available Not Available Not Available Not Available Not Available NA R0 Not Applicable Not Applicable Not Applicable Not Applicable Not Applicable Not Applicable Not Applicable Not Applicable Not Applicable Stage IB Not Applicable Not Applicable Not Applicable 6th Not Applicable Not Applicable Not Applicable M0 N0 T2 Not Available NO YES 05 3 Lung Alive 2010 2008 Not Available

724 Alive T3 N0 MX Stage IIB NA 69 R-Lower Not Applicable TCGA-J2-8194 2b68b51f-bd4c-4bbb-b4e6-2eead53da2a4 6 -25441 Not Applicable 0 114 Not Available Lung Adenocarcinoma 59 NA TCGA-J2-8194-D32562;TCGA-J2-8194-D32566;TCGA-J2-8194-D55424;TCGA-J2-8194-D55426;TCGA-J2-8194-D55427;TCGA-J2-8194-D55428 2FAE07DB-7F3D-45D6-BA62-5C795B46A511;04888E00-4D3A-44D4-9C38-BB9DA5B386B7;4C297630-A1FD-4811-899D-AA652ACB4F3D;726C1CA1-1718-4EA7-9CAC-8701FA927C14;7AE778AB-433A-4D39-AB87-A4A38B5EF832;F21DD793-5C47-438B-9CBC-8E3B6B4DE464 Not Available;Not Available;Not Available;Not Available;Not Available;Not Available 6;6;7;7;7;7 Not Available;Not Available;584;584;640;Not Available 72;72;546;546;597;663 carboplatin;alimta;Carboplatin;Gemcitabine;Doxetaxol;Erlotinib Not Available;Not Available;Clinical Progressive Disease;Clinical Progressive Disease;Stable Disease;Not Applicable 6;6;1;1;1;1 Not Available;Not Available;Not Available;Not Available;Not Available;Not Available Not Available;Not Available;Not Available;Not Available;Not Available;Not Available Not Available;Not Available;Not Available;Not Available;Not Available;Not Available Not Available;Not Available;Not Available;Not Available;Not Available;Not Available Not Applicable;Not Applicable;Not Applicable;Not Applicable;Not Applicable;Not Applicable Not Available;Not Available;Not Available;Not Available;Not Available;Not Available Not Available;Not Available;Not Available;Not Available;Not Available;Not Available YES;YES;NO;NO;NO;YES Chemotherapy;Chemotherapy;Chemotherapy;Chemotherapy;Chemotherapy;Targeted Molecular therapy Not Available;Not Available;Not Available;Not Available;Not Available;Not Available Not Available;Not Available;Not Available;Not Available;Not Available;Not Available Not Available;Not Available;Not Available;Not Available;Not Available;Not Available NO;NO;NO;NO;NO;NO 2012;2012;2014;2014;2014;2014 Not Available Not Available Unknown Not Available Not Available Not Available Unknown Not Available NOT HISPANIC OR LATINO NA YES NO NO YES TCGA-J2-8194-F55317 CCFB27C8-26F9-4711-BDF0-683C72901DE8 3 Not Available 518 Not Applicable 724 470 Not Available Scheduled Follow-up Submission Partial Remission/Response Not Available NO 1 Distant Metastasis YES Not Available WITH TUMOR YES Progressive Disease Biopsy with Histologic Confirmation;Convincing Imaging YES Alive 2014 FEMALE Lung Adenocarcinoma Mixed Subtype No C34.3 8255/3 C34.3 YES Not Available Unknown Not Available Not Available Not Available 6 Not Available Not Available Not Available Not Available Not Available Not Available Not Available Not Available Not Available NO 20 Yes, History of Prior Malignancy 8194 Not Available Unknown 2.04 83 YES 2.04 83 Unknown YES WHITE NO NA Unknown TCGA-J2-8194-R55430 39FF1C80-F11B-4726-A0BD-CF3836CB2627 Not Available 7 271 240 Unknown 1 Not Available Not Available NO Unknown Not Applicable Not Available Not Available Not Available 2014 Not Available Not Applicable Not Applicable Not Applicable Not Applicable Not Applicable Not Applicable Not Applicable Not Applicable Not Applicable Stage IIB Not Applicable Not Applicable Not Applicable 7th Not Applicable Not Applicable Not Applicable MX N0 T3 1982 YES NO J2 3 Lung Alive 2012 2011 1956

760 Dead T2 N1 M0 Stage IIB NA 71 L-Lower Not Applicable TCGA-55-6984 f063ddbb-1668-40df-9ec2-b0a23ca2c389 27 Not Available 760 0 Not Available Not Available Lung Adenocarcinoma Not Available NA TCGA-55-6984-D45633;TCGA-55-6984-D45634 2EEFA4BA-8311-4923-9CC0-43AEDFF385C4;0D8E1A7A-9075-4584-B85F-489D6DAB16F2 Not Available;Not Available 17;17 Not Available;747 65;724 Chemo, Multi-Agent, NOS;Chemo, NOS Complete Response;Clinical Progressive Disease 7;7 Not Available;Not Available Not Available;Not Available Not Available;Not Available Not Available;Not Available Not Applicable;Not Applicable Not Available;Not Available Not Available;Not Available NO;NO Chemotherapy;Chemotherapy Not Available;Not Available Not Available;Not Available Not Available;Not Available NO;NO 2013;2013 Not Available Not Available NO Not Available Not Available Not Available NO Not Available NOT HISPANIC OR LATINO NA YES NO NO NO TCGA-55-6984-F45632 DB66DE4C-0099-4F8A-8ABA-4D7D43922A68 17 Not Available Not Available 760 Not Available 724 Not Evaluated Scheduled Follow-up Submission Progressive Disease Not Evaluated NO 7 Not Available YES Not Evaluated WITH TUMOR YES Complete Remission/Response Not Available NO Dead 2013 FEMALE Lung Adenocarcinoma- Not Otherwise Specified (NOS) No C34.3 8140/3 C34.3 YES Not Available NO Not Available Not Available Not Available 7 Not Available Not Available Not Available Not Available Not Available Not Available Not Available Not Available Not Available Not Available Not Available No 6984 Not Available WITH TUMOR Not Available Not Available Not Available Not Available Not Available Not Available Not Available WHITE Not Available NA Not Available Not Applicable Not Applicable Not Applicable Not Applicable Not Applicable Not Applicable Not Applicable Not Applicable Not Applicable Stage IIB Not Applicable Not Applicable Not Applicable 6th Not Applicable Not Applicable Not Applicable M0 N1 T2 Not Available NO YES 55 Not Available Lung Dead 2011 2006 Not Available

492 Alive T3 N0 MX Stage IIB NA 62 R-Upper Not Applicable TCGA-91-7771 c0fdb152-25d2-404b-bec7-a43ece381f5b 24 -22926 Not Applicable 0 31 Not Available Lung Adenocarcinoma Not Available NA TCGA-91-7771-D37917;TCGA-91-7771-D37919 494CE129-C26D-4374-B915-382105753D85;7D947262-1775-4C0C-981A-AAF1E6E7D92E Not Available;Not Available 6;6 156;156 77;77 Cisplatin;Pemetrexed Complete Response;Complete Response 12;12 Not Available;Not Available Not Available;Not Available Not Available;Not Available Not Available;Not Available Not Applicable;Not Applicable Not Available;Not Available Not Available;Not Available NO;NO Chemotherapy;Chemotherapy Not Available;Not Available Not Available;Not Available Not Available;Not Available Not Available;Not Available 2012;2012 Not Available Not Available NO Not Available Not Available Not Available Not Available Not Available NOT HISPANIC OR LATINO NA Not Available Not Available Not Available Not Available TCGA-91-7771-F37916 5CE9E189-97C2-4431-8689-FBACCA998B25 6 Not Applicable Not Applicable Not Applicable 492 Not Applicable Unknown Scheduled Follow-up Submission Complete Remission/Response Unknown NO 12 Not Available NO Not Available TUMOR FREE YES Complete Remission/Response Not Available NO Alive 2012 MALE Lung Adenocarcinoma- Not Otherwise Specified (NOS) No C34.1 8140/3 C34.1 YES Not Available NO Not Available Not Available Not Available 1 Not Available Not Available Not Available Not Available Not Available Not Available Not Available Not Available Not Available Not Available 75 Yes 7771 Not Available Not Available Not Available Not Available Not Available Not Available Not Available Not Available NO WHITE Not Available NA Not Available Not Applicable Not Applicable Not Applicable Not Applicable Not Applicable Not Applicable Not Applicable Not Applicable Not Applicable Stage IIB Not Applicable Not Applicable Not Applicable 7th Not Applicable Not Applicable Not Applicable MX N0 T3 2011 YES NO 91 4 Lung Alive 2012 2011 Not Available

1700 Alive T1 N0 M0 Stage IA NA 79 L-Upper Not Applicable TCGA-49-4514 b87a5d92-4c09-48fe-bb08-b3c89a7c892e 9 -28908 Not Applicable 0 1314 Not Available Lung Adenocarcinoma Not Available NA Not Available Not Available Not Available Not Available Not Available Not Available Not Available Not Available Not Available NA Not Available Not Available Not Available Not Available TCGA-49-4514-F14817 8e78115f-ef3f-4e4f-b8e4-16fdf02e5002 10 Not Applicable Not Applicable Not Applicable 1700 Not Applicable Not Available Scheduled Follow-up Submission Not Available Not Available Not Available 8 Not Available NO Not Available Not Available NO Complete Remission/Response Not Available NO Alive 2011 FEMALE Lung Papillary Adenocarcinoma No C34.1 8260/3 C34.1 YES Not Available Not Available Not Available Not Available Not Available 8 Not Available Not Available Not Available Not Available Not Available Not Available Not Available Not Available Not Available Not Available 65 No 4514 Not Available TUMOR FREE Not Available Not Available Not Available Not Available Not Available Not Available Not Available WHITE Not Available NA R0 Not Applicable Not Applicable Not Applicable Not Applicable Not Applicable Not Applicable Not Applicable Not Applicable Not Applicable Stage IA Not Applicable Not Applicable Not Applicable 6th Not Applicable Not Applicable Not Applicable M0 N0 T1 Not Available NO YES 49 3 Lung Alive 2011 2007 Not Available

912 Alive T2 N0 M0 Stage IB NA 41 R-Lower Not Applicable TCGA-05-4420 3d2aa654-1b5f-4eb4-a1c2-af31f5760069 22 -15159 Not Applicable 0 912 Not Available Lung Adenocarcinoma Not Available NA Not Available Not Available Not Available Not Available Not Available Not Available Not Available Not Available Not Available NA Not Available Not Available Not Available Not Available TCGA-05-4420-F36531 F35DE973-C732-4FC5-9C26-51A06531E0A2 1 Not Applicable Not Applicable Not Applicable 912 Not Applicable Not Available Scheduled Follow-up Submission Complete Remission/Response Not Available NO 11 Not Available NO Not Available TUMOR FREE NO Complete Remission/Response Not Available NO Alive 2012 MALE Lung Adenocarcinoma Mixed Subtype No C34.3 8255/3 C34.3 YES Not Available Not Available Not Available Not Available Not Available 7 Not Available Not Available Not Available Not Available Not Available Not Available Not Available Not Available Not Available Not Available 38 No 4420 Not Available TUMOR FREE Not Available Not Available Not Available Not Available Not Available Not Available Not Available Not Available Not Available NA R0 Not Applicable Not Applicable Not Applicable Not Applicable Not Applicable Not Applicable Not Applicable Not Applicable Not Applicable Stage IB Not Applicable Not Applicable Not Applicable 6th Not Applicable Not Applicable Not Applicable M0 N0 T2 Not Available NO YES 05 2 Lung Alive 2010 2008 Not Available

1073 Dead T2 N0 M0 Stage IB NA 75 R-Upper Not Applicable TCGA-38-4630 f1985735-a188-4567-a88a-530f9b80291b 14 -27667 1073 0 Not Available Not Available Lung Adenocarcinoma Not Available NA Not Available Not Available NO Not Available Not Available Not Available NO Not Available NOT HISPANIC OR LATINO NA NO;NO;NO YES;YES;YES NO;NO;NO NO;Not Available;NO TCGA-38-4630-F4890;TCGA-38-4630-F4943;TCGA-38-4630-F4972 de9bcf35-aaab-4e5d-815f-d1568b93d2ce;46bd4471-369f-4e60-b754-b39b142348bf;9687a1c3-6c11-4d6b-9130-26b74c79e736 14;16;16 Not Available;Not Available;Not Available Not Available;Not Available;Not Available 1073;1073;1073 Not Available;Not Available;Not Available 524;746;836 Not Available;Not Available;Not Available Not Available;Not Available;Not Available Stable Disease;Stable Disease;Stable Disease Not Available;Not Available;Not Available Not Available;Not Available;Not Available 12;12;12 Not Available;Not Available;Not Available YES;YES;YES Not Available;Not Available;Not Available WITH TUMOR;WITH TUMOR;WITH TUMOR NO;NO;NO Not Available;Complete Remission/Response;Complete Remission/Response Not Available;Not Available;Not Available NO;YES;NO Dead;Dead;Dead 2010;2010;2010 FEMALE Lung Papillary Adenocarcinoma No C34.1 8260/3 C34.1 YES Not Available NO Not Available Not Available Peripheral Lung 12 Not Available Not Available Not Available Not Available Not Available Not Available Not Available Not Available Not Available Not Available Not Available Yes 4630 Not Available WITH TUMOR Not Available Not Available Not Available Not Available Not Available Not Available NO WHITE Not Available NA Distant site;Local Recurrence;Distant Recurrence TCGA-38-4630-R4944;TCGA-38-4630-R4942;TCGA-38-4630-R4973 3e17d3f4-0106-465e-8130-6031c5d2e239;17380ae4-f888-43ff-8c25-bd48f811558e;21b69307-a2ec-4d9e-bbe6-3ba5d6b5db41 2;1;3 16;16;16 772;573;869 754;554;848 Not Available;Not Available;Not Available 12;12;12 10;14;15 2250;3500;3000 NO;NO;NO EXTERNAL BEAM;EXTERNAL BEAM;EXTERNAL BEAM Not Applicable;Not Applicable;Not Applicable PALLIATIVE;PALLIATIVE;PALLIATIVE Not Available;Not Available;Not Available cGy;cGy;cGy 2010;2010;2010 R1 Not Applicable Not Applicable Not Applicable Not Applicable Not Applicable Not Applicable Not Applicable Not Applicable Not Applicable Stage IB Not Applicable Not Applicable Not Applicable 6th Not Applicable Not Applicable Not Applicable M0 N0 T2 Not Available NO YES 38 1 Lung Dead 2010 2000 Not Available

826 Dead T4 N1 M1 Stage IV NA 52 R-Middle Not Applicable TCGA-78-7145 4ef872e1-82c9-4939-9248-41ed9d3085b2 23 -19080 826 0 Not Available Not Available Lung Adenocarcinoma Not Available NA TCGA-78-7145-D16675;TCGA-78-7145-D16672;TCGA-78-7145-D16674;TCGA-78-7145-D16673;TCGA-78-7145-D17203 93f81bc9-f22b-4e9f-85b9-da28d41a7569;b3966e3e-c4e0-4a0f-875b-5a2b599ef98d;8798a8c5-378c-44b9-b21b-f09302daab77;12b1de25-cb72-4778-a205-ad6d7f68c4e4;703cc226-f1a6-4c2e-bdc6-dcf30f5b8c11 Not Available;Not Available;Not Available;Not Available;Not Available 23;23;23;23;5 Not Available;161;685;547;161 716;28;605;440;28 Not Available;Cisplatin;Gefitinib;Docetaxel;Gemcitabine Not Available;Not Available;Not Available;Not Available;Not Available 9;9;9;9;10 Not Available;6;Not Available;6;6 Not Available;Not Available;Not Available;Not Available;Not Available Not Available;Not Available;Not Available;Not Available;Not Available PROGRESSION;ADJUVANT;PROGRESSION;PROGRESSION;ADJUVANT Not Applicable;Not Applicable;Not Applicable;Not Applicable;Not Applicable 4;1;3;2;1 IM;IV;PO;IV;IV NO;NO;NO;NO;NO Other, specify in notes;Chemotherapy;Targeted Molecular therapy;Chemotherapy;Chemotherapy Phase II clinical trial KRW2170;Not Available;Not Available;Not Available;Not Available Not Available;Not Available;Not Available;Not Available;Not Available Not Available;Not Available;Not Available;Not Available;Not Available Not Available;Not Available;Not Available;Not Available;Not Available 2011;2011;2011;2011;2011 1 Not Available NO Not Available Not Available Not Available NO Not Available Not Available NA YES YES NO NO TCGA-78-7145-F16671 faa937a2-b75e-4764-89e0-8a9d3698ecda 23 Not Available Not Available 826 Not Available 422 Not Available Scheduled Follow-up Submission Progressive Disease Not Available Not Available 9 Not Available YES Not Available WITH TUMOR YES Not Available Not Available NO Dead 2011 FEMALE Lung Adenocarcinoma- Not Otherwise Specified (NOS) No C34.2 8140/3 C34.2 YES Not Available NO Not Available Not Available Peripheral Lung 9 Not Available Not Available Not Available Not Available Not Available Not Available Not Available Not Available Not Available Not Available 25 No 7145 Preoperative WITH TUMOR Not Available Not Available Not Available Not Available Not Available Not Available Not Available WHITE Not Available NA Regional site TCGA-78-7145-R17204 aade3c8c-dc1c-4127-b685-c52607c1406e 1 5 644 644 Not Available 10 1 800 NO EXTERNAL BEAM Not Applicable PALLIATIVE Not Available cGy 2011 R1 Not Applicable Not Applicable Not Applicable Not Applicable Not Applicable Not Applicable Not Applicable Not Applicable Not Applicable Stage IV Not Applicable Not Applicable Not Applicable 6th Not Applicable Not Applicable Not Applicable M1 N1 T4 1998 NO YES 78 4 Lung Dead 2011 2000 1973

1147 Dead T1b N1 M0 Stage IIA NA 64 R-Upper Not Applicable TCGA-38-4627 d717fa33-3c91-4ced-89c6-79ff5542f04c 7 -23510 1147 0 Not Available Not Available Lung Adenocarcinoma Not Available NA Not Available Not Available Not Available Not Available Not Available Not Available Not Available Not Available NOT HISPANIC OR LATINO NA Not Available Not Available Not Available Not Available TCGA-38-4627-F4508 f15fda3c-b93a-4ae2-95d3-c372aed5ea42 8 Not Available Not Available 1147 Not Available Not Available Not Available Not Available Not Available Not Available Not Available 12 Not Available Not Available Not Available Not Available NO Complete Remission/Response Not Available NO Dead 2010 FEMALE Lung Adenocarcinoma- Not Otherwise Specified (NOS) No C34.1 8140/3 C34.1 YES Not Available Not Available Not Available Not Available Central Lung 12 Not Available Not Available Not Available Not Available Not Available Not Available Not Available Not Available Not Available Not Available Not Available No 4627 Not Available Not Available Not Available Not Available Not Available Not Available Not Available Not Available Not Available WHITE Not Available NA R0 Not Applicable Not Applicable Not Applicable Not Applicable Not Applicable Not Applicable Not Applicable Not Applicable Not Applicable Stage IIA Not Applicable Not Applicable Not Applicable Not Available Not Applicable Not Applicable Not Applicable M0 N1 T1b Not Available NO YES 38 4 Lung Dead 2010 2003 Not Available

800 Alive T1 N0 M1 Stage IV NA 52 R-Upper Not Applicable TCGA-73-4666 4c31127e-d095-4978-9dac-35153c27f6ed 6 -19074 Not Applicable 0 618 Not Available Lung Adenocarcinoma Not Available NA TCGA-73-4666-D13246;TCGA-73-4666-D13245 6e0afbee-c9ca-4103-850d-1c7e07b03d56;d23a0b04-32a9-428b-b725-89722eeb2d40 Not Available;Not Available 6;6 119;119 56;56 carboplatin;taxol Not Available;Not Available 12;12 4;4 Not Available;Not Available mg;mg ADJUVANT;ADJUVANT Not Applicable;Not Applicable 1;1 IV;IV NO;NO Chemotherapy;Chemotherapy Not Available;Not Available Not Available;Not Available mg;mg Not Available;Not Available 2010;2010 0 Not Available NO Not Available Not Available Not Available NO Not Available NOT HISPANIC OR LATINO NA NO YES NO YES TCGA-73-4666-F12865 66debbbc-3fbd-4209-a23b-eb3076bc6ed8 10 Not Available -43 Not Applicable 800 Not Available 1 Not Available Complete Remission/Response 80 Not Available 6 Not Available YES Post-Adjuvant Therapy TUMOR FREE YES Complete Remission/Response Not Available YES Alive 2011 FEMALE Lung Adenocarcinoma- Not Otherwise Specified (NOS) No C34.1 8140/3 C34.1 YES 100 NO Not Available Not Available Central Lung 1 Not Available Not Available Not Available Not Available Not Available Not Available Not Available Not Available Not Available Not Available 10 No 4666 Preoperative TUMOR FREE Not Available Not Available Not Available Not Available Not Available Not Available Not Available WHITE Not Available NA Distant site TCGA-73-4666-R12866 daf9b0b5-1a62-4a76-afa0-77726b8a1718 1 10 70 70 Not Available 6 1 1800 NO OTHER Gamma Knife ADJUVANT Not Available cGy 2011 R0 Not Applicable Not Applicable Not Applicable Not Applicable Not Applicable Not Applicable Not Applicable Not Applicable Not Applicable Stage IV Not Applicable Not Applicable Not Applicable Not Available Not Applicable Not Applicable Not Applicable M1 N0 T1 2002 NO YES 73 4 Lung Alive 2011 2009 1982

595 Alive T1 N0 M0 Stage IA NA 62 R-Upper Not Applicable TCGA-44-6145 6144d666-8f77-4efe-95f1-f9e5c4e5e056 31 -22745 Not Applicable 0 103 Not Available Lung Adenocarcinoma 73 NA 0 Not Available Not Available Not Available Not Available Not Available Not Available Not Available NOT HISPANIC OR LATINO NA Not Available;Not Available Not Available;Not Available Not Available;Not Available Not Available;Not Available TCGA-44-6145-F28687;TCGA-44-6145-F38802 32fc1b78-c824-434f-b655-22498b49fb64;0C442DE0-4653-41AC-B01C-344325306F22 28;24 Not Applicable;Not Applicable Not Applicable;Not Applicable Not Applicable;Not Applicable 328;595 Not Applicable;Not Applicable Not Available;Not Available Scheduled Follow-up Submission;Scheduled Follow-up Submission Complete Remission/Response;Complete Remission/Response Not Available;Not Available Not Available;NO 2;12 Not Available;Not Available NO;NO Not Available;Not Available TUMOR FREE;TUMOR FREE NO;NO Complete Remission/Response;Complete Remission/Response Not Available;Not Available NO;NO Alive;Alive 2012;2012 FEMALE Lung Adenocarcinoma- Not Otherwise Specified (NOS) No C34.1 8140/3 C34.1 YES Not Available NO NO Not Available Not Available 5 Not Available Not Available Not Available Not Available Not Available Not Available Not Available Not Available Not Available Not Available 75 No 6145 Preoperative TUMOR FREE 91 90 Not Available 77 71 Not Available YES WHITE Not Available NA Not Available Not Applicable Not Applicable Not Applicable Not Applicable Not Applicable Not Applicable Not Applicable Not Applicable Not Applicable Stage IA Not Applicable Not Applicable Not Applicable 7th Not Applicable Not Applicable Not Applicable M0 N0 T1 1999 YES NO 44 4 Lung Alive 2011 2011 1963

365 Alive T2 N0 M0 Stage IB NA 68 L-Lower Not Applicable TCGA-05-4422 44dec838-b653-42a7-a58b-a1fd232cd68c 22 -24837 Not Applicable 0 365 Not Available Lung Adenocarcinoma Not Available NA Not Available Not Available Not Available Not Available Not Available Not Available Not Available Not Available Not Available NA MALE Lung Adenocarcinoma Mixed Subtype No C34.3 8255/3 C34.3 YES Not Available Not Available Not Available Not Available Not Available 7 Not Available Not Available Not Available Not Available Not Available Not Available Not Available Not Available Not Available Not Available 147 No 4422 Not Available Not Available Not Available Not Available Not Available Not Available Not Available Not Available Not Available Not Available Not Available NA R0 Not Applicable Not Applicable Not Applicable Not Applicable Not Applicable Not Applicable Not Applicable Not Applicable Not Applicable Stage IB Not Applicable Not Applicable Not Applicable 6th Not Applicable Not Applicable Not Applicable M0 N0 T2 Not Available NO YES 05 4 Lung Alive 2010 2008 Not Available

282 Dead T2 N2 M0 Stage IIIA NA 47 Discrepancy Not Available TCGA-50-5930 368e23f0-e573-4547-bf5a-14080baf737b 29 -17238 282 0 Not Available Not Available Lung Adenocarcinoma Not Available NA TCGA-50-5930-D32066 1a5b7d4d-a7ea-4064-b672-a951d639b77e Not Available 16 146 85 Taxotere Clinical Progressive Disease 5 Not Available Not Available Not Available Not Available Not Applicable Not Available Not Available NO Chemotherapy Not Available Not Available Not Available NO 2012 Not Available Not Available Not Available Not Available Not Available Not Available Not Available Not Available NOT HISPANIC OR LATINO NA NO YES Not Available NO TCGA-50-5930-F32064 51c0573d-cbd9-4d7a-a72a-1e71ab3ae05a 16 Not Available Not Available 282 Not Available 177 Not Available Scheduled Follow-up Submission Progressive Disease Not Available NO 5 Distant Metastasis YES Not Available WITH TUMOR YES Progressive Disease Convincing Imaging YES Dead 2012 MALE Lung Adenocarcinoma Mixed Subtype No C34.3 8255/3 C34.3 YES Not Available Not Available Not Available Not Available Not Available 6 Not Available Not Available Not Available Not Available Not Available Not Available Not Available Not Available Not Available Not Available Not Available No 5930 Not Available WITH TUMOR Not Available Not Available Not Available Not Available Not Available Not Available Not Available WHITE Not Available NA Primary Tumor Field TCGA-50-5930-R32065 873291ff-f54a-44c0-9f9b-ff040cd9518a Not Available 16 177 177 Not Available 5 Not Available 2520 NO EXTERNAL BEAM Not Applicable ADJUVANT Not Available cGy 2012 Not Available Not Applicable Not Applicable Not Applicable Not Applicable Not Applicable Not Applicable Not Applicable Not Applicable Not Applicable Stage IIIA Not Applicable Not Applicable Not Applicable 5th Not Applicable Not Applicable Not Applicable M0 N2 T2 Not Available NO YES 50 Not Available Lung Dead 2011 2001 Not Available

711 Dead T2 N2 M0 Stage IIIA NA 66 R-Upper Not Applicable TCGA-73-4659 cd0dc947-b708-464e-beb7-954c9d4583e7 3 -24253 711 0 711 Not Available Lung Adenocarcinoma Not Available NA TCGA-73-4659-D12858;TCGA-73-4659-D12862;TCGA-73-4659-D12421;TCGA-73-4659-D12823;TCGA-73-4659-D12430;TCGA-73-4659-D12827;TCGA-73-4659-D12854;TCGA-73-4659-D12830 e1e21cb8-57eb-41c0-bc61-810f106edb69;0d2c7085-2781-4e8e-8b42-63ec7e58b646;98e093a8-64cb-4848-a1c5-0cb8c8b550f9;9025783d-e657-4c73-a5f6-e9ee2e60a789;a14661c5-aff8-4e99-af96-b96b3dafcf52;cd7e24d6-b03d-4d4d-aa1d-1f3263695c72;4a12034c-0bf9-4eb9-a160-0cf1abb40e59;8cfd7bb0-b951-48a1-bb37-5452ea0cbf5a Not Available;Not Available;Not Available;Not Available;Not Available;Not Available;Not Available;Not Available 3;3;3;3;3;3;3;3 609;657;298;452;406;494;581;581 602;624;257;410;312;473;546;539 Taxol;CYC-116/Cyclocel;Alimta;Vinorelbine;Erlotonib;Docetoxel/Taxotere;Cisplatin;Gemcitabine Not Available;Not Available;Not Available;Not Available;Not Available;Not Available;Not Available;Not Available 12;12;12;12;12;12;12;12 2;3;3;2;5;4;2;2 50mg/m2 = 100mg;720 mg;500;25mg/m2 = 50;150;25mg/m2 = 50mg;75mg/m2 - 150mg;100mg/m2 mg/m2;mg;mg/m2;mg/m2;mg;g/m2;mg/m2;mg/m2 PROGRESSION;OTHER, SPECIFY IN NOTES;PROGRESSION;PROGRESSION;PROGRESSION;PROGRESSION;PROGRESSION;PROGRESSION Not Applicable;Protocol;Not Applicable;Not Applicable;Not Applicable;Not Applicable;Not Applicable;Not Applicable 7;8;3;3;4;4;6;5 IV;PO;IV;IV;PO;IV;IV;IV NO;NO;NO;NO;NO;NO;NO;NO Chemotherapy;Other, specify in notes;Chemotherapy;Chemotherapy;Targeted Molecular therapy;Chemotherapy;Chemotherapy;Chemotherapy Not Available;Aurora Kinase Inhibitor - Protocol therapy;Not Available;Not Available;Not Available;Not Available;Not Available;Not Available 200;15120;3000;300;9000;150;300;Not Available mg;mg;mg;mg;mg;mg;mg;Not Available Not Available;Not Available;Not Available;Not Available;Not Available;Not Available;Not Available;Not Available 2010;2010;2010;2010;2010;2010;2010;2010 0 Not Available NO Not Available Not Available Not Available NO Not Available NOT HISPANIC OR LATINO NA YES;YES;NO YES;NO;YES NO;NO;Not Available Not Available;Not Available;NO TCGA-73-4659-F12416;TCGA-73-4659-F42016;TCGA-73-4659-F42024 f2d5e4ac-03fa-459f-b244-372e45af7556;29AE1152-27C6-4EC5-BF73-5E60331EB46D;A3EC6F64-BB1F-4BDF-A2C0-1C928B6342DB 3;9;9 Not Available;Not Available;Not Available Not Available;Not Available;Not Available 711;711;711 711;Not Available;Not Available 35;47;316 Not Available;Not Available;Not Available Not Available;Scheduled Follow-up Submission;Scheduled Follow-up Submission Progressive Disease;Progressive Disease;Progressive Disease 0;0;0 Not Available;NO;NO 12;4;4 Not Available;Locoregional Recurrence;Distant Metastasis YES;YES;YES Other;Other;Other WITH TUMOR;WITH TUMOR;WITH TUMOR NO;NO;NO Not Available;Progressive Disease;Progressive Disease Not Available;Convincing Imaging;Convincing Imaging NO;NO;NO Dead;Dead;Dead 2010;2013;2013 MALE Lung Adenocarcinoma- Not Otherwise Specified (NOS) No C34.1 8140/3 C34.9 YES 90 NO Not Available Not Available Central Lung 1 Not Available Not Available Not Available Not Available Not Available Not Available Not Available Not Available Not Available Not Available 12 No 4659 Other WITH TUMOR Not Available Not Available Not Available Not Available Not Available Not Available Not Available WHITE Not Available NA Distant site;Regional site TCGA-73-4659-R12417;TCGA-73-4659-R12419 1982ff3d-adc9-4ee5-a1db-ac538d6ccbdb;a399e767-7839-4e48-bcc9-7df3fd55366c 1;2 3;3 337;529 337;516 Not Available;Not Available 12;12 1;10 2200;3000 NO;NO OTHER;EXTERNAL BEAM Gamma Knife;Not Applicable PROGRESSION;PALLIATIVE Not Available;Not Available cGy;cGy 2010;2010 R0 Not Applicable Not Applicable Not Applicable Not Applicable Not Applicable Not Applicable Not Applicable Not Applicable Not Applicable Stage IIIA Not Applicable Not Applicable Not Applicable 6th Not Applicable Not Applicable Not Applicable M0 N2 T2 1986 NO YES 73 3 Lung Dead 2011 2006 1974

1060 Alive T3 N2 M0 Stage IIIA NA 72 R-Upper Not Applicable TCGA-99-8025 84c3ba70-afa7-4b69-be69-7ec8d6022c56 25 -26634 Not Applicable 0 427 Not Available Lung Adenocarcinoma 87 NA TCGA-99-8025-D33590 E437156A-F538-4BCB-9CC6-2B6DC295AA6D Not Available 9 286 213 Gemzar Unknown 7 Not Available Not Available Not Available Not Available Not Applicable Not Available Not Available NO Chemotherapy Not Available Not Available Not Available NO 2012 1 Not Available YES Not Available Not Available Not Available YES Not Available NOT HISPANIC OR LATINO NA Not Available Not Available Not Available Not Available TCGA-99-8025-F57698 13030256-EACD-4E4B-B19C-592CBBE0A543 23 Not Applicable Not Applicable Not Applicable 1060 Not Applicable Not Available Scheduled Follow-up Submission Complete Remission/Response Not Available NO 5 Not Available NO Not Available TUMOR FREE YES Complete Remission/Response Not Available NO Alive 2014 FEMALE Lung Adenocarcinoma- Not Otherwise Specified (NOS) No C34.1 8140/3 C34.1 YES Unknown YES NO Not Available Central Lung 6 Not Available Not Available Not Available Not Available Not Available Not Available Not Available Not Available Not Available NO 20 No 8025 Preoperative TUMOR FREE Not Available Not Available YES Not Available 111 Unknown YES BLACK OR AFRICAN AMERICAN NO NA R0 Not Applicable Not Applicable Not Applicable Not Applicable Not Applicable Not Applicable Not Applicable Not Applicable Not Applicable Stage IIIA Not Applicable Not Applicable Not Applicable 7th Not Applicable Not Applicable Not Applicable M0 N2 T3 1998 NO YES 99 4 Lung Alive 2012 2010 1958

1125 Alive T1a N0 M0 Stage IA NA 63 R-Upper Not Applicable TCGA-50-8457 d45aee46-838e-44af-8422-46710b3240a8 16 -23055 Not Applicable 0 44 Not Available Lung Adenocarcinoma Not Available NA Not Available Not Available YES Not Available Not Available FISH YES Not Available NOT HISPANIC OR LATINO NA Not Available;Not Available Not Available;Not Available Not Available;Not Available Not Available;Not Available TCGA-50-8457-F69333;TCGA-50-8457-F70409 C2D58752-6A3C-46E7-9BF4-34591B2C0C16;367955C7-74FB-4A08-A87A-9719A41AAF35 5;11 Not Applicable;Not Applicable Not Applicable;Not Applicable Not Applicable;Not Applicable 779;1125 Not Applicable;Not Applicable Not Available;Not Available Scheduled Follow-up Submission;Scheduled Follow-up Submission Complete Remission/Response;Complete Remission/Response Not Available;Not Available NO;NO 1;2 Not Available;Not Available NO;NO Not Available;Not Available TUMOR FREE;TUMOR FREE NO;NO Complete Remission/Response;Complete Remission/Response Not Available;Not Available NO;NO Alive;Alive 2015;2015 FEMALE Lung Adenocarcinoma Mixed Subtype No C34.1 8255/3 C34.1 YES Not Available YES YES G12A Not Available 10 Not Available Not Available Not Available Not Available Not Available Not Available Not Available Not Available Not Available NO 30 No 8457 Not Available TUMOR FREE Not Available Not Available NO Not Available Not Available Complete Remission/Response NO BLACK OR AFRICAN AMERICAN NO NA Not Available Not Applicable Not Applicable Not Applicable Not Applicable Not Applicable Not Applicable Not Applicable Not Applicable Not Applicable Stage IA Not Applicable Not Applicable Not Applicable 7th Not Applicable Not Applicable Not Applicable M0 N0 T1a 1997 YES NO 50 4 Lung Alive 2012 2011 Not Available

603 Alive T3 N0 MX Stage IIB NA 81 R-Upper Not Applicable TCGA-55-7994 67dbe286-edb3-4d07-8f74-9e5254f01945 7 -29858 Not Applicable 0 31 Not Available Lung Adenocarcinoma Not Available NA TCGA-55-7994-D47824;TCGA-55-7994-D47825 711DE5E8-64BE-4AE2-9B48-BE206A7960AC;C4E8D500-182E-4A75-9E3A-5EC4E60DD487 Not Available;Not Available 28;28 121;121 56;56 Carboplatin;Taxol Complete Response;Complete Response 8;8 Not Available;Not Available Not Available;Not Available Not Available;Not Available Not Available;Not Available Not Applicable;Not Applicable Not Available;Not Available Not Available;Not Available NO;NO Chemotherapy;Chemotherapy Not Available;Not Available Not Available;Not Available Not Available;Not Available NO;NO 2013;2013 Not Available Not Available NO Not Available Not Available Not Available NO Not Available NOT HISPANIC OR LATINO NA Not Available Not Available Not Available Not Available TCGA-55-7994-F47823 F1F645DE-9236-459D-A930-61A18E1E6F22 28 Not Applicable Not Applicable Not Applicable 603 Not Applicable Not Evaluated Scheduled Follow-up Submission Complete Remission/Response Not Evaluated NO 8 Not Available NO Not Evaluated TUMOR FREE YES Complete Remission/Response Not Available NO Alive 2013 MALE Lung Adenocarcinoma- Not Otherwise Specified (NOS) No C34.1 8140/3 C34.1 YES Not Available NO Not Available Not Available Not Available 5 Not Available Not Available Not Available Not Available Not Available Not Available Not Available Not Available Not Available NO 28 No 7994 Not Available TUMOR FREE Not Available Not Available Unknown Not Available Not Available Complete Remission/Response Not Available WHITE Unknown NA R0 Not Applicable Not Applicable Not Applicable Not Applicable Not Applicable Not Applicable Not Applicable Not Applicable Not Applicable Stage IIB Not Applicable Not Applicable Not Applicable 7th Not Applicable Not Applicable Not Applicable MX N0 T3 Not Available YES NO 55 2 Lung Alive 2012 2011 Not Available

2488 Alive T1 N2 M0 Stage IIIA NA 58 R-Upper Not Applicable TCGA-64-1679 81a0b2ff-a3d3-41bb-9ce6-765e6ae894af 21 -21310 Not Applicable 0 1686 Not Available Lung Adenocarcinoma Not Available NA TCGA-64-1679-D20370;TCGA-64-1679-D20371 a1d5051d-3893-4e77-b34c-88e599bda776;42c98e6b-93c1-4647-8b65-82716be9d850 Not Available;Not Available 21;21 139;139 55;55 Cisplatin;Gemcitabine Not Available;Not Available 12;12 4;4 75;1000 mg/m2;mg/m2 ADJUVANT;ADJUVANT Not Applicable;Not Applicable 1;1 IV;IV NO;NO Chemotherapy;Chemotherapy Not Available;Not Available Not Available;Not Available Not Available;Not Available Not Available;Not Available 2011;2011 0 Not Available Not Available Not Available Not Available Not Available Not Available Not Available NOT HISPANIC OR LATINO NA Not Available;Not Available Not Available;Not Available Not Available;Not Available Not Available;Not Available TCGA-64-1679-F20369;TCGA-64-1679-F66261 8a815a0c-b766-4a8e-9b29-7eeb682a688e;B39531A0-0DD8-4B57-A36C-29DBE8F73F24 21;7 Not Applicable;Not Applicable Not Applicable;Not Applicable Not Applicable;Not Applicable 1686;2488 Not Applicable;Not Applicable 0;Unknown Scheduled Follow-up Submission;Scheduled Follow-up Submission Not Available;Complete Remission/Response Not Available;Unknown Not Available;NO 12;10 Not Available;Not Available NO;NO Post-Adjuvant Therapy;Unknown TUMOR FREE;TUMOR FREE YES;YES Complete Remission/Response;Complete Remission/Response Not Available;Not Available NO;NO Alive;Alive 2011;2014 FEMALE Lung Adenocarcinoma- Not Otherwise Specified (NOS) No C34.1 8140/3 C34.1 YES Not Available NO Not Available Not Available Not Available 12 Not Available Not Available Not Available Not Available Not Available Not Available Not Available Not Available Not Available Not Available 40 No 1679 Post-Adjuvant Therapy TUMOR FREE Not Available Not Available Not Available Not Available Not Available Not Available YES WHITE Not Available NA R0 Not Applicable Not Applicable Not Applicable Not Applicable Not Applicable Not Applicable Not Applicable Not Applicable Not Applicable Stage IIIA Not Applicable Not Applicable Not Applicable 6th Not Applicable Not Applicable Not Applicable M0 N2 T1 Not Available NO YES 64 2 Lung Alive 2011 2007 Not Available

1126 Alive T2a N2 M1 Stage IV NA 63 R-Middle Not Applicable TCGA-64-1680 1ab7ad70-0f80-41d8-8efa-3baebc8223cc 16 -23267 Not Applicable 0 1126 Not Available Lung Adenocarcinoma Not Available NA TCGA-64-1680-D4377 16e53760-5ca6-4a73-b332-a900418c84d6 Not Available 16 Not Available Not Available Not Available Not Available 11 Not Available Not Available Not Available ADJUVANT Not Applicable 1 Not Available Not Available Chemotherapy Not Available Not Available Not Available Not Available 2010 Not Available Not Available Not Available Not Available Not Available Not Available Not Available Not Available NOT HISPANIC OR LATINO NA Not Available Not Available Not Available Not Available TCGA-64-1680-F4387 509d68b7-f334-42a9-962b-b7ff425bab81 16 Not Applicable Not Applicable Not Applicable 1126 Not Applicable Not Available Not Available Not Available Not Available Not Available 11 Not Available NO Not Available TUMOR FREE YES Complete Remission/Response Not Available YES Alive 2010 MALE Lung Adenocarcinoma Mixed Subtype No C34.8 8255/3 C34.8 YES Not Available Not Available Not Available Not Available Not Available 11 Not Available Not Available Not Available Not Available Not Available Not Available Not Available Not Available Not Available Not Available 50 No 1680 Not Available TUMOR FREE Not Available Not Available Not Available Not Available Not Available Not Available Not Available WHITE Not Available NA Not Available TCGA-64-1680-R4378 d4ad9989-199c-4734-be42-ec485b3ea9eb 1 16 Not Available Not Available Not Available 11 Not Available Not Available Not Available EXTERNAL BEAM Not Applicable ADJUVANT Not Available Not Available 2010 R0 Not Applicable Not Applicable Not Applicable Not Applicable Not Applicable Not Applicable Not Applicable Not Applicable Not Applicable Stage IV Not Applicable Not Applicable Not Applicable 6th Not Applicable Not Applicable Not Applicable M1 N2 T2a 1982 NO YES 64 3 Lung Alive 2010 2007 1962

1026 Dead T1 N0 M0 Stage IA NA 71 R-Upper Not Applicable TCGA-44-3919 66ed89f6-8ba2-49d0-af0f-4b6ebf2381bf 7 -26081 Not Applicable 0 190 Not Available Lung Adenocarcinoma 103 NA Not Available Not Available Not Available Not Available Not Available Not Available Not Available Not Available NOT HISPANIC OR LATINO NA Not Available;NO;NO Not Available;YES;YES Not Available;NO;NO Not Available;NO;NO TCGA-44-3919-F5232;TCGA-44-3919-F38529;TCGA-44-3919-F38530 5d1a6aba-a69b-4767-b109-8ae19be0770f;E807118D-E91B-41E2-A96D-69FFF71A8E0F;9EC1AE84-0030-4A21-8ECF-F1E537C866C4 8;19;19 Not Applicable;Not Available;Not Available Not Applicable;Not Available;Not Available Not Applicable;1026;1026 190;Not Available;Not Available Not Applicable;921;984 Not Available;2;2 Not Available;Additional New Tumor Event;Additional New Tumor Event Complete Remission/Response;Progressive Disease;Not Applicable Not Available;Not Available;Not Available Not Available;NO;NO 10;12;12 Not Available;Locoregional Recurrence;Distant Metastasis NO;YES;YES Not Available;Other;Other TUMOR FREE;WITH TUMOR;WITH TUMOR NO;NO;NO Complete Remission/Response;Complete Remission/Response;Complete Remission/Response Not Available;Convincing Imaging;Convincing Imaging NO;NO;NO Alive;Dead;Dead 2010;2012;2012 FEMALE Lung Adenocarcinoma- Not Otherwise Specified (NOS) No C34.1 8140/3 C34.1 YES Not Available NO Not Available Not Available Not Available 10 Not Available Not Available Not Available Not Available Not Available Not Available Not Available Not Available Not Available Not Available Not Available No 3919 Not Available TUMOR FREE Not Available Not Available Not Available 99 100 Not Available YES WHITE Not Available NA R0 Not Applicable Not Applicable Not Applicable Not Applicable Not Applicable Not Applicable Not Applicable Not Applicable Not Applicable Stage IA Not Applicable Not Applicable Not Applicable 7th Not Applicable Not Applicable Not Applicable M0 N0 T1 Not Available YES NO 44 1 Lung Alive 2010 2010 Not Available

515 Alive T1a N0 MX Stage IA NA 75 L-Lower Not Applicable TCGA-55-8621 8d535aa6-6d59-4965-b5ed-17217d8f5930 11 -27485 Not Applicable 0 106 Not Available Lung Adenocarcinoma Not Available NA Not Evaluated Not Available Unknown Not Available Not Available Not Available Unknown Not Available NOT HISPANIC OR LATINO NA Not Available Not Available Not Available Not Available TCGA-55-8621-F48791 E4B4EB9A-0821-4025-BE16-EFAD0D846418 20 Not Applicable Not Applicable Not Applicable 515 Not Applicable 1 Scheduled Follow-up Submission Complete Remission/Response 90 NO 9 Not Available NO Other TUMOR FREE NO Complete Remission/Response Not Available NO Alive 2013 FEMALE Lung Adenocarcinoma- Not Otherwise Specified (NOS) No C34.3 8140/3 C34.3 YES Not Evaluated Unknown Not Available Not Available Unknown 1 Not Available Not Available Not Available Not Available Not Available Not Available Not Available Not Available Not Available NO 30 Yes, History of Prior Malignancy 8621 Not Available TUMOR FREE 112 105 NO 110 105 Complete Remission/Response YES WHITE NO NA R0 Not Applicable Not Applicable Not Applicable Not Applicable Not Applicable Not Applicable Not Applicable Not Applicable Not Applicable Stage IA Not Applicable Not Applicable Not Applicable 7th Not Applicable Not Applicable Not Applicable MX N0 T1a 2010 YES NO 55 4 Lung Alive 2013 2012 1980

NA T2 N0 M0 Stage IB NA Not Available Discrepancy Not Available TCGA-75-6211 2db08267-d346-4d42-aac0-b76831903253 21 Not Available Not Available Not Available Not Available Not Available Lung Adenocarcinoma Not Available NA Not Available Not Available Not Available Not Available Not Available Not Available Not Available Not Available Not Available NA NO YES Not Available NO TCGA-75-6211-F15095 3b3000b8-4b0f-4a15-bdbc-0565868daa27 21 Not Available Not Available Not Available Not Available Not Available Not Available Not Available Progressive Disease Not Available Not Available 7 Not Available YES Adjuvant therapy WITH TUMOR NO Complete Remission/Response Not Available NO Dead 2011 FEMALE Lung Adenocarcinoma- Not Otherwise Specified (NOS) No C34.1 8140/3 C34.9 YES Not Available Not Available Not Available Not Available Not Available 7 Not Available Not Available Not Available Not Available Not Available Not Available Not Available Not Available Not Available Not Available 34 No 6211 Preoperative WITH TUMOR Not Available Not Available Not Available Not Available Not Available Not Available Not Available Not Available Not Available NA Distant site TCGA-75-6211-R15097 faa5218b-def1-499d-aa96-930ae142d62c 1 21 Not Available Not Available Not Available 7 5 2000 NO EXTERNAL BEAM Not Applicable PALLIATIVE Not Available cGy 2011 R0 Not Applicable Not Applicable Not Applicable Not Applicable Not Applicable Not Applicable Not Applicable Not Applicable Not Applicable Stage IB Not Applicable Not Applicable Not Applicable 6th Not Applicable Not Applicable Not Applicable M0 N0 T2 Not Available NO YES 75 2 Lung Dead 2011 2009 Not Available

2973 Alive T2a N0 M0 Stage IB NA 66 R-Upper Not Applicable TCGA-38-4625 0ea4920f-f4c8-4590-84b3-eb419824e144 7 -24241 Not Applicable 0 2973 Not Available Lung Adenocarcinoma 68 NA Not Available Not Available NO Not Available Not Available Not Available NO Not Available NOT HISPANIC OR LATINO NA Not Available;Not Available Not Available;Not Available Not Available;Not Available Not Available;Not Available TCGA-38-4625-F4505;TCGA-38-4625-F33407 3ba4cc67-5bc8-4ad5-a486-601d63f9f7c7;F9656835-E4F0-4DCD-8208-0E49AF248244 8;26 Not Available;Not Available Not Available;Not Available Not Applicable;Not Applicable 2973;2973 Not Available;Not Available Not Available;Not Available Not Available;Scheduled Follow-up Submission Not Available;Complete Remission/Response Not Available;Not Available Not Available;YES 12;7 Not Available;Not Available Not Available;Not Available Not Available;Not Available Not Available;Not Available NO;Not Available Complete Remission/Response;Not Available Not Available;Not Available NO;Not Available Alive;Alive 2010;2012 FEMALE Lung Adenocarcinoma- Not Otherwise Specified (NOS) No C34.1 8140/3 C34.1 YES Not Available NO Not Available Not Available Peripheral Lung 12 Not Available Not Available Not Available Not Available Not Available Not Available Not Available Not Available Not Available Not Available 50 No 4625 Not Available Not Available 66 66 Not Available 68 64 Not Available YES WHITE Not Available NA R0 Not Applicable Not Applicable Not Applicable Not Applicable Not Applicable Not Applicable Not Applicable Not Applicable Not Applicable Stage IB Not Applicable Not Applicable Not Applicable 5th Not Applicable Not Applicable Not Applicable M0 N0 T2a Not Available NO YES 38 2 Lung Alive 2010 2002 1952

690 Alive T1b N0 M0 Stage IA NA 69 R-Middle Not Applicable TCGA-69-7763 1b31cdb3-1837-49d6-a857-1830f8800299 25 -25316 Not Applicable 0 690 Not Available Lung Adenocarcinoma 72 NA 1 Not Available NO Not Available Not Available Not Available NO Not Available NOT HISPANIC OR LATINO NA Not Available Not Available Not Available Not Available TCGA-69-7763-F20990 742bb503-45e8-406d-8578-66299cd22dfb 26 Not Applicable Not Applicable Not Applicable 690 Not Applicable Not Available Scheduled Follow-up Submission Not Available Not Available Not Available 1 Not Available NO Not Available Not Available NO Not Available Not Available NO Alive 2012 MALE Lung Adenocarcinoma Mixed Subtype No C34.2 8550/3 C34.2 YES Not Available NO Not Available Not Available Peripheral Lung 1 Not Available Not Available Not Available Not Available Not Available Not Available Not Available Not Available Not Available Not Available 7.5 No 7763 Other Not Available Not Available Not Available Not Available 93 89 Not Available YES WHITE Not Available NA Not Available Not Applicable Not Applicable Not Applicable Not Applicable Not Applicable Not Applicable Not Applicable Not Applicable Not Applicable Stage IA Not Applicable Not Applicable Not Applicable 7th Not Applicable Not Applicable Not Applicable M0 N0 T1b 2004 NO YES 69 4 Lung Alive 2012 2010 1959

351 Alive T1 N0 M0 Stage IA NA 73 R-Upper Not Applicable TCGA-44-A47G DA345947-498B-4272-A231-A987ED4FE151 27 -26800 Not Applicable 0 135 Not Available Lung Adenocarcinoma 85 NA 0 Not Available NO Not Available Not Available Not Available NO Not Available NOT HISPANIC OR LATINO NA Not Available Not Available Not Available Not Available TCGA-44-A47G-F49023 B45EC546-C7BD-426B-B7AE-16F5FF6A1559 25 Not Applicable Not Applicable Not Applicable 351 Not Applicable Not Evaluated Scheduled Follow-up Submission Complete Remission/Response Not Evaluated NO 9 Not Available NO Not Available TUMOR FREE NO Complete Remission/Response Not Available NO Alive 2013 FEMALE Lung Adenocarcinoma- Not Otherwise Specified (NOS) No C34.1 8140/3 C34.1 YES Not Evaluated NO Not Available Not Available Not Available 11 Not Available Not Available Not Available Not Available Not Available Not Available Not Available Not Available Not Available NO 28 No A47G Preoperative TUMOR FREE Not Available Not Available NO 113 100 Complete Remission/Response YES WHITE NO NA R0 Not Applicable Not Applicable Not Applicable Not Applicable Not Applicable Not Applicable Not Applicable Not Applicable Not Applicable Stage IA Not Applicable Not Applicable Not Applicable 7th Not Applicable Not Applicable Not Applicable M0 N0 T1 2012 YES NO 44 4 Lung Alive 2012 2012 1956

888 Alive T1b N0 M0 Stage IA NA 56 R-Upper Not Applicable TCGA-55-8206 a214a959-3b21-4f25-a615-383cda7afdff 15 -20690 Not Applicable 0 46 Not Available Lung Adenocarcinoma 98 NA Not Available Not Available Not Available Not Available Not Available Not Available Not Available Not Available NOT HISPANIC OR LATINO NA Not Available Not Available Not Available Not Available TCGA-55-8206-F65698 E1AC477E-3512-4DF7-979A-AB575A80690D 26 Not Applicable Not Applicable Not Applicable 888 Not Applicable Not Available Scheduled Follow-up Submission Complete Remission/Response Not Available NO 9 Not Available NO Not Available TUMOR FREE NO Complete Remission/Response Not Available NO Alive 2014 MALE Lung Adenocarcinoma- Not Otherwise Specified (NOS) No C34.1 8140/3 C34.1 YES Not Available Not Available Not Available Not Available Not Available 6 Not Available Not Available Not Available Not Available Not Available Not Available Not Available Not Available Not Available NO Not Available No 8206 Not Available TUMOR FREE Not Available Not Available NO 104 84 Complete Remission/Response YES WHITE NO NA R0 Not Applicable Not Applicable Not Applicable Not Applicable Not Applicable Not Applicable Not Applicable Not Applicable Not Applicable Stage IA Not Applicable Not Applicable Not Applicable 7th Not Applicable Not Applicable Not Applicable M0 N0 T1b Not Available YES NO 55 1 Lung Alive 2012 2012 Not Available

274 Dead T3 N2 M0 Stage IIIA NA 69 L-Lower Not Applicable TCGA-05-4418 d2c1e896-6886-4122-bb48-5fbcd3f641f4 22 -25417 274 0 Not Available Not Available Lung Adenocarcinoma Not Available NA Not Available Not Available Not Available Not Available Not Available Not Available Not Available Not Available Not Available NA MALE Lung Adenocarcinoma Mixed Subtype No C34.3 8255/3 C34.3 YES Not Available Not Available Not Available Not Available Not Available 7 Not Available Not Available Not Available Not Available Not Available Not Available Not Available Not Available Not Available Not Available 25 Yes 4418 Not Available Not Available Not Available Not Available Not Available Not Available Not Available Not Available Not Available Not Available Not Available NA R0 Not Applicable Not Applicable Not Applicable Not Applicable Not Applicable Not Applicable Not Applicable Not Applicable Not Applicable Stage IIIA Not Applicable Not Applicable Not Applicable 6th Not Applicable Not Applicable Not Applicable M0 N2 T3 Not Available NO YES 05 2 Lung Dead 2010 2008 Not Available

855 Dead T1 N0 M0 Stage IA NA 72 L-Upper Not Applicable TCGA-49-4487 bd4e9a9f-17f3-4d33-92fc-fcc45dd0e7b4 9 -26583 855 0 Not Available Not Available Lung Adenocarcinoma Not Available NA Not Available Not Available Not Available Not Available Not Available Not Available Not Available Not Available NOT HISPANIC OR LATINO NA NO YES YES YES TCGA-49-4487-F14693 c1cdbc66-08a8-47a7-906e-949c08e2bbfc 9 697 712 855 Not Available 697 Not Available Scheduled Follow-up Submission Progressive Disease Not Available Not Available 8 Not Available YES Not Available WITH TUMOR NO Complete Remission/Response Not Available YES Dead 2011 FEMALE Lung Adenocarcinoma- Not Otherwise Specified (NOS) No C34.1 8140/3 C34.1 YES Not Available Not Available Not Available Not Available Not Available 8 Not Available Not Available Not Available Not Available Not Available Not Available Not Available Not Available Not Available Not Available 40 No 4487 Not Available WITH TUMOR Not Available Not Available Not Available Not Available Not Available Not Available Not Available WHITE Not Available NA Distant site TCGA-49-4487-R14697 3b6918b1-000b-4ce2-bf5e-797ed368d94b 2 9 741 725 Not Available 8 Not Available 3600 NO OTHER Whole Brain RECURRENCE Not Available cGy 2011 R0 Not Applicable Not Applicable Not Applicable Not Applicable Not Applicable Not Applicable Not Applicable Not Applicable Not Applicable Stage IA Not Applicable Not Applicable Not Applicable 3rd Not Applicable Not Applicable Not Applicable M0 N0 T1 Not Available NO YES 49 4 Lung Dead 2011 1992 Not Available

486 Alive T2 N0 MX Stage IB NA 73 R-Upper Not Applicable TCGA-44-A479 851867CB-9AD0-4FDF-AC75-9BB0427AD440 28 -27024 Not Applicable 0 256 Not Available Lung Adenocarcinoma 60 NA 0 Not Available YES Not Available Not Available Not Available NO Not Available NOT HISPANIC OR LATINO NA Not Available;NO Not Available;NO Not Available;YES Not Available;NO TCGA-44-A479-F41312;TCGA-44-A479-F45874 7FF14E7B-72D0-4EEB-9483-855DE51FB0D5;DD21BCBF-913D-49C3-A321-E156FEF9D73C 14;12 Not Applicable;437 Not Applicable;Not Available Not Applicable;Not Applicable 392;486 Not Applicable;437 Not Available;Not Evaluated Scheduled Follow-up Submission;Additional New Tumor Event Complete Remission/Response;Complete Remission/Response Not Available;Not Evaluated NO;NO 3;8 Not Available;New Primary Tumor NO;YES Not Available;Not Available TUMOR FREE;TUMOR FREE NO;NO Complete Remission/Response;Complete Remission/Response Not Available;Not Available NO;NO Alive;Alive 2013;2013 FEMALE Lung Adenocarcinoma- Not Otherwise Specified (NOS) No C34.1 8140/3 C34.1 YES Not Evaluated NO Not Available Not Available Not Available 11 Not Available Not Available Not Available Not Available Not Available Not Available Not Available Not Available Not Available NO 40 No A479 Preoperative TUMOR FREE Not Available Not Available NO 115 102 Complete Remission/Response YES BLACK OR AFRICAN AMERICAN NO NA R0 Not Applicable Not Applicable Not Applicable Not Applicable Not Applicable Not Applicable Not Applicable Not Applicable Not Applicable Stage IB Not Applicable Not Applicable Not Applicable 7th Not Applicable Not Applicable Not Applicable MX N0 T2 2002 YES NO 44 4 Lung Alive 2012 2012 1962

701 Dead T2 N0 M0 Stage IA NA 65 L-Upper Not Applicable TCGA-55-1592 a41c46da-7ed4-4192-bd16-b3cbb94a5133 15 Not Available 701 0 Not Available Not Available Lung Adenocarcinoma Not Available NA Not Available Not Available Not Available Not Available Not Available Not Available Not Available Not Available NOT HISPANIC OR LATINO NA NO NO NO NO TCGA-55-1592-F9167 d1fe03fa-cf81-4ff3-beb4-aaeb15b9e6ae 15 Not Available Not Available 701 Not Available 452 Not Available Not Available Progressive Disease Not Available Not Available 3 Not Available YES Not Available WITH TUMOR NO Complete Remission/Response Not Available NO Dead 2011 MALE Lung Adenocarcinoma- Not Otherwise Specified (NOS) No C34.1 8140/3 C34.1 YES Not Available Not Available Not Available Not Available Not Available 3 Not Available Not Available Not Available Not Available Not Available Not Available Not Available Not Available Not Available Not Available Not Available Yes 1592 Not Available WITH TUMOR Not Available Not Available Not Available Not Available Not Available Not Available Not Available WHITE Not Available NA R0 Not Applicable Not Applicable Not Applicable Not Applicable Not Applicable Not Applicable Not Applicable Not Applicable Not Applicable Stage IA Not Applicable Not Applicable Not Applicable Not Available Not Applicable Not Applicable Not Applicable M0 N0 T2 1997 NO YES 55 4 Lung Dead 2011 2005 1957

435 Alive T1 N0 M0 Stage IA NA 67 R-Upper Not Applicable TCGA-L4-A4E6 26A92E1B-08D8-45CD-AF0C-EAF2514808A2 5 -24631 Not Applicable 0 76 Not Available Lung Adenocarcinoma 98 NA Not Evaluated Not Available NO Not Available Not Available Not Available NO Not Available NOT HISPANIC OR LATINO NA Not Available Not Available Not Available Not Available TCGA-L4-A4E6-F51277 4A1DF958-C4FC-4CEB-B14F-05210285715F 14 Not Applicable Not Applicable Not Applicable 435 Not Applicable Not Evaluated Scheduled Follow-up Submission Complete Remission/Response Not Evaluated NO 11 Not Available NO Not Available TUMOR FREE NO Complete Remission/Response Not Available NO Alive 2013 MALE Lung Adenocarcinoma- Not Otherwise Specified (NOS) No C34.1 8140/3 C34.1 YES Not Evaluated NO Not Available Not Available Peripheral Lung 12 NO NO Not Available Not Available Not Available Not Available Not Available Not Available Not Available NO 30 No A4E6 Not Evaluated TUMOR FREE 82 85 NO 84 69 Complete Remission/Response YES WHITE NO NA R0 Not Applicable Not Applicable Not Applicable Not Applicable Not Applicable Not Applicable Not Applicable Not Applicable Not Applicable Stage IA Not Applicable Not Applicable Not Applicable 7th Not Applicable Not Applicable Not Applicable M0 N0 T1 1984 NO YES L4 3 Lung Alive 2012 2012 1954

987 Dead T2 NX MX Stage IB NA 85 L-Upper Not Applicable TCGA-44-6777 349e6f38-2c67-4a69-a777-b9173a2a27cf 30 -31234 987 0 Not Available Not Available Lung Adenocarcinoma 178 NA 1 Not Available Not Available Not Available Not Available Not Available Not Available Not Available Not Available NA Not Available Not Available Not Available Not Available TCGA-44-6777-F15694 d62760e3-307f-4aad-a7b8-1ab9f020e3f7 30 Not Applicable Not Applicable 987 Not Available Not Applicable 3 Scheduled Follow-up Submission Not Available Not Available Not Available 8 Not Available NO Post-Adjuvant Therapy TUMOR FREE NO Complete Remission/Response Not Available YES Dead 2011 FEMALE Lung Adenocarcinoma- Not Otherwise Specified (NOS) No C34.1 8140/3 C34.1 YES Not Available Not Available Not Available Not Available Not Available 8 Not Available Not Available Not Available Not Available Not Available Not Available Not Available Not Available Not Available Not Available 64 No 6777 Preoperative TUMOR FREE 77 71 Not Available 68 62 Not Available YES WHITE Not Available NA Primary Tumor Field TCGA-44-6777-R15693 b19b3be9-6f5a-4ada-b550-8e5fde588838 1 30 356 306 Not Available 8 37 7400 NO EXTERNAL BEAM Not Applicable ADJUVANT Not Available cGy 2011 Not Available Not Applicable Not Applicable Not Applicable Not Applicable Not Applicable Not Applicable Not Applicable Not Applicable Not Applicable Stage IB Not Applicable Not Applicable Not Applicable 6th Not Applicable Not Applicable Not Applicable MX NX T2 2006 NO YES 44 4 Lung Dead 2011 2006 1942

988 Alive T1b N0 MX Stage IA NA 66 L-Upper Not Applicable TCGA-J2-A4AG E21E2AF3-DC71-41FA-94BA-7A7929F47D90 27 -24224 Not Applicable 0 238 Not Available Lung Adenocarcinoma Not Available NA Not Available Not Available NO Not Available Not Available Not Available NO Not Available NOT HISPANIC OR LATINO NA Not Available;Not Available Not Available;Not Available Not Available;Not Available Not Available;Not Available TCGA-J2-A4AG-F55526;TCGA-J2-A4AG-F70848 E18C8E1A-90F4-4EBC-AD9D-7B4C8E64F099;BDE8905E-3F01-45AE-BDB2-150D1F22DE83 8;10 Not Applicable;Not Applicable Not Applicable;Not Applicable Not Applicable;Not Applicable 500;988 Not Applicable;Not Applicable Not Available;Not Available Scheduled Follow-up Submission;Scheduled Follow-up Submission Complete Remission/Response;Complete Remission/Response Not Available;Not Available NO;NO 1;3 Not Available;Not Available NO;NO Not Available;Not Available TUMOR FREE;TUMOR FREE NO;NO Complete Remission/Response;Complete Remission/Response Not Available;Not Available NO;NO Alive;Alive 2014;2015 FEMALE Lung Adenocarcinoma- Not Otherwise Specified (NOS) No C34.1 8140/3 C34.1 YES Not Available NO Not Available Not Available Not Available 12 Not Available Not Available Not Available Not Available Not Available Not Available Not Available Not Available Not Available NO 25 No A4AG Not Available TUMOR FREE Not Available Not Available NO Not Available Not Available Complete Remission/Response NO WHITE NO NA R0 Not Applicable Not Applicable Not Applicable Not Applicable Not Applicable Not Applicable Not Applicable Not Applicable Not Applicable Stage IA Not Applicable Not Applicable Not Applicable 7th Not Applicable Not Applicable Not Applicable MX N0 T1b 1981 YES NO J2 3 Lung Alive 2012 2012 1956

0 Alive T1 NX M0 Stage IA NA 75 R-Lower Not Applicable TCGA-86-8281 5d7ec32a-049f-4997-9882-1b3ac157e02d 30 -27409 Not Applicable 0 0 Not Available Lung Adenocarcinoma Not Available NA 0 Not Available Not Available Not Available Not Available Not Available Not Available Not Available NOT HISPANIC OR LATINO NA Not Available Not Available Not Available Not Available TCGA-86-8281-F57953 017E9B68-F25D-49A5-B8E0-DB66626379F3 21 Not Available Not Available Not Applicable Not Available Not Available Not Available Scheduled Follow-up Submission Not Available Not Available YES 4 Not Available Not Available Not Available Not Available Not Available Not Available Not Available Not Available Not Available 2014 MALE Lung Adenocarcinoma- Not Otherwise Specified (NOS) No C34.3 8140/3 C34.3 YES 100 NO Not Available Not Available Peripheral Lung 5 Not Available Not Available Not Available Not Available Not Available Not Available Not Available Not Available Not Available NO 30 No 8281 Preoperative TUMOR FREE Not Available Not Available Unknown Not Available Not Available Complete Remission/Response NO WHITE Unknown NA Not Evaluated Not Applicable Not Applicable Not Applicable Not Applicable Not Applicable Not Applicable Not Applicable Not Applicable Not Applicable Stage IA Not Applicable Not Applicable Not Applicable 7th Not Applicable Not Applicable Not Applicable M0 NX T1 1985 YES NO 86 3 Lung Alive 2012 2011 1955

258 Dead T2 N1 M0 Stage IIB NA 84 L-Lower Not Applicable TCGA-78-7166 b40d0849-f4ef-4a14-9732-9beb708cb46b 28 -30869 258 0 Not Available Not Available Lung Adenocarcinoma Not Available NA 2 Not Available NO Not Available Not Available Not Available NO Not Available Not Available NA NO NO Not Available Not Available TCGA-78-7166-F16954 91193834-639f-45c9-9c1b-79a5c5c33f76 28 Not Applicable Not Applicable 258 Not Available Not Applicable Not Available Scheduled Follow-up Submission Not Available Not Available Not Available 9 Not Available NO Not Available Not Available NO Not Available Not Available YES Dead 2011 MALE Lung Adenocarcinoma- Not Otherwise Specified (NOS) No C34.3 8140/3 C34.3 YES Not Available NO Not Available Not Available Central Lung 9 Not Available Not Available Not Available Not Available Not Available Not Available Not Available Not Available Not Available Not Available 38 Yes 7166 Preoperative Not Available Not Available Not Available Not Available Not Available Not Available Not Available Not Available WHITE Not Available NA Regional site TCGA-78-7166-R16955 cd485d3f-a20d-4299-8f2f-2dd37177e390 1 28 Not Available Not Available Not Available 9 Not Available Not Available NO EXTERNAL BEAM Not Applicable ADJUVANT Not Available Not Available 2011 R0 Not Applicable Not Applicable Not Applicable Not Applicable Not Applicable Not Applicable Not Applicable Not Applicable Not Applicable Stage IIB Not Applicable Not Applicable Not Applicable 6th Not Applicable Not Applicable Not Applicable M0 N1 T2 1972 NO YES 78 3 Lung Dead 2011 2002 1934

1324 Alive T1 N0 M0 Stage IA NA 65 R-Upper Not Applicable TCGA-44-2655 07b5663f-9a54-4462-b6c1-6fc8116b8714 9 -23854 Not Applicable 0 575 575 Lung Adenocarcinoma 73 NA 0 Not Available Not Available Not Available Not Available Not Available Not Available Not Available NOT HISPANIC OR LATINO NA NO;Not Available;NO NO;Not Available;NO NO;Not Available;NO NO;Not Available;NO TCGA-44-2655-F5293;TCGA-44-2655-F5096;TCGA-44-2655-F39289 42f22d9e-2d8f-4b1f-81e7-29eca0f4fed2;9cfda329-c1e1-43cb-9157-daa5aee723ef;08096B08-FEB1-4550-830D-4BEE7FD6E37F 8;20;16 Not Applicable;Not Applicable;Not Available Not Applicable;Not Applicable;Not Available Not Applicable;Not Applicable;Not Applicable 575;667;1324 Not Applicable;Not Applicable;1009 Not Available;Not Available;Not Available Not Available;Not Available;Scheduled Follow-up Submission Complete Remission/Response;Not Available;Complete Remission/Response Not Available;Not Available;Not Available Not Available;Not Available;NO 10;12;1 Not Available;Not Available;New Primary Tumor NO;NO;YES Not Available;Not Available;Not Available TUMOR FREE;TUMOR FREE;TUMOR FREE NO;NO;NO Complete Remission/Response;Complete Remission/Response;Complete Remission/Response Not Available;Not Available;Not Available NO;NO;NO Alive;Alive;Alive 2010;2010;2013 FEMALE Lung Adenocarcinoma- Not Otherwise Specified (NOS) No C34.1 8140/3 C34.1 YES Not Available NO Not Available Not Available Not Available 10 Not Available Not Available Not Available Not Available Not Available Not Available Not Available Not Available Not Available Not Available 30 Yes 2655 Pre-Adjuvant Therapy TUMOR FREE Not Available Not Available Not Available 100 91 Not Available YES WHITE Not Available NA R0 Not Applicable Not Applicable Not Applicable Not Applicable Not Applicable Not Applicable Not Applicable Not Applicable Not Applicable Stage IA Not Applicable Not Applicable Not Applicable 6th Not Applicable Not Applicable Not Applicable M0 N0 T1 1988 YES NO 44 3 Lung Alive 2010 2009 1958

165 Alive T4 N0 MX NA 56 R-Upper Not Applicable TCGA-69-7765 bcf2e591-9dae-440f-bd03-5f27c57db741 27 -20625 Not Applicable 0 129 Not Available Lung Adenocarcinoma 45 NA TCGA-69-7765-D21012;TCGA-69-7765-D29489 dd4077d4-121a-4548-b9cb-131aee81484b;5e1b818b-56f7-4340-91eb-d490e7bc91a2 Not Available;Not Available 27;20 Not Available;Not Available 93;93 Carboplatin;Pemetrexed Not Available;Not Available 1;3 4;4 6;500 AUC;mg/m2 ADJUVANT;ADJUVANT Not Applicable;Not Applicable 1;1 IV;IV YES;YES Chemotherapy;Chemotherapy Not Available;Not Available Not Available;Not Available Not Available;Not Available Not Available;Not Available 2012;2012 Not Available Not Available NO Other Not Available Not Available NO Not Available NOT HISPANIC OR LATINO NA Not Available Not Available Not Available Not Available TCGA-69-7765-F29244 79cba3a7-8b66-4cac-a18e-ebc14740d0d0 13 Not Applicable Not Applicable Not Applicable 165 Not Applicable Not Available Scheduled Follow-up Submission Not Available Not Available Not Available 3 Not Available NO Not Available Not Available YES Not Available Not Available NO Alive 2012 MALE Lung Adenocarcinoma Mixed Subtype No C34.1 8255/3 C34.1 YES Not Available NO Not Available Not Available Peripheral Lung 1 Not Available Not Available Not Available Not Available Not Available Not Available Not Available Not Available Not Available Not Available Not Available No 7765 Not Available Not Available Not Available Not Available Not Available 86 84 Not Available YES BLACK OR AFRICAN AMERICAN Not Available NA Not Available Not Applicable Not Applicable Not Applicable Not Applicable Not Applicable Not Applicable Not Applicable Not Applicable Not Applicable Discrepancy Not Applicable Not Applicable Not Applicable 7th Not Applicable Not Applicable Not Applicable MX N0 T4 2011 YES NO 69 4 Lung Alive 2012 2011 1981

1159 Alive T1 N0 M0 Stage IA NA 69 L-Upper Not Applicable TCGA-44-2661 f3501466-cf32-4866-b5fb-e94dd32341bc 8 -25313 Not Applicable 0 103 Not Available Lung Adenocarcinoma Not Available NA 1 Not Available Not Available Not Available Not Available Not Available Not Available Not Available NOT HISPANIC OR LATINO NA Not Available;Not Available;Not Available Not Available;Not Available;Not Available Not Available;Not Available;Not Available Not Available;Not Available;Not Available TCGA-44-2661-F5051;TCGA-44-2661-F12810;TCGA-44-2661-F41293 7ac97c53-3160-4567-ba1e-bac4b06376cd;2463a46b-1698-45e4-b11c-5f691e0f69f3;B711FC80-F8AA-4F4C-AF2A-C9AA98016533 8;8;14 Not Applicable;Not Available;Not Applicable Not Applicable;Not Available;Not Applicable Not Applicable;Not Applicable;Not Applicable 446;741;1159 Not Applicable;Not Available;Not Applicable 1;Not Available;Not Available Not Available;Not Available;Scheduled Follow-up Submission Complete Remission/Response;Complete Remission/Response;Complete Remission/Response Not Available;Not Available;Not Available Not Available;Not Available;NO 10;6;3 Not Available;Not Available;Not Available NO;Not Available;NO Pre-Adjuvant Therapy;Not Available;Not Available TUMOR FREE;TUMOR FREE;TUMOR FREE NO;NO;NO Complete Remission/Response;Complete Remission/Response;Complete Remission/Response Not Available;Not Available;Not Available NO;NO;NO Alive;Alive;Alive 2010;2011;2013 FEMALE Lung Adenocarcinoma- Not Otherwise Specified (NOS) No C34.1 8140/3 C34.1 YES Not Available NO Not Available Not Available Not Available 10 Not Available Not Available Not Available Not Available Not Available Not Available Not Available Not Available Not Available Not Available Not Available Yes 2661 Pre-Adjuvant Therapy TUMOR FREE Not Available Not Available Not Available Not Available Not Available Not Available YES WHITE Not Available NA R0 Not Applicable Not Applicable Not Applicable Not Applicable Not Applicable Not Applicable Not Applicable Not Applicable Not Applicable Stage IA Not Applicable Not Applicable Not Applicable 6th Not Applicable Not Applicable Not Applicable M0 N0 T1 Not Available YES NO 44 1 Lung Alive 2010 2009 Not Available

38 Dead T2a N0 M0 NA 74 R-Upper Not Applicable TCGA-73-4677 03c3409a-9d16-41d0-9e26-f9fc2547a4c8 7 -27381 38 0 Not Available Not Available Lung Adenocarcinoma Not Available NA Not Available Not Available NO Not Available Not Available Not Available NO Not Available NOT HISPANIC OR LATINO NA Not Available Not Available Not Available Not Available TCGA-73-4677-F12744 579419ee-f9cb-451a-9a14-ad2ecfa0376b 1 Not Applicable Not Applicable 38 Not Available Not Applicable 1 Not Available Not Available 80 Not Available 6 Not Available NO Adjuvant therapy Not Available NO Stable Disease Not Available NO Dead 2011 MALE Lung Adenocarcinoma- Not Otherwise Specified (NOS) No C34.1 8140/3 C34.1 YES Not Available NO Not Available Not Available Peripheral Lung 1 Not Available Not Available Not Available Not Available Not Available Not Available Not Available Not Available Not Available Not Available Not Available No 4677 Other WITH TUMOR Not Available Not Available Not Available Not Available Not Available Not Available Not Available WHITE Not Available NA R0 Not Applicable Not Applicable Not Applicable Not Applicable Not Applicable Not Applicable Not Applicable Not Applicable Not Applicable Discrepancy Not Applicable Not Applicable Not Applicable Not Available Not Applicable Not Applicable Not Applicable M0 N0 T2a 1985 NO YES 73 3 Lung Dead 2011 2010 Not Available

808 Dead T2a N0 M0 Stage IB NA FPPP TCGA 60 R-Upper Not Applicable TCGA-44-4112 68b86559-38b2-41f2-b66e-c3c2b628b14d 12 -22106 Not Applicable 0 141 141 Lung Adenocarcinoma Not Available NA TCGA-44-4112-D13710;TCGA-44-4112-D13711 75c42d72-040a-42d2-953d-8a4a9c467011;1c60b058-9bc5-4159-996a-57177380956d Not Available;Not Available 11;11 142;142 78;78 Cisplatin;Alimta Not Available;Not Available 7;7 4;4 165;1100 mg;mg ADJUVANT;ADJUVANT Not Applicable;Not Applicable 1;1 IV;IV NO;NO Chemotherapy;Chemotherapy Not Available;Not Available 660;4400 mg;mg Not Available;Not Available 2011;2011 Not Available Not Available Not Available Not Available Not Available Not Available Not Available Not Available NOT HISPANIC OR LATINO NA Not Available;NO;Not Available Not Available;YES;Not Available Not Available;NO;NO Not Available;YES;Not Available TCGA-44-4112-F13709;TCGA-44-4112-F30573;TCGA-44-4112-F32266 84d22f7a-401b-4420-92c0-d5bd3374c131;646d47cb-6adc-4cae-966a-f63c8ac3c781;dfbce6d3-9f66-439a-8c79-9b18418af662 11;12;22 Not Available;Not Available;Not Applicable Not Available;619;Not Applicable Not Applicable;Not Applicable;808 370;722;Not Available Not Available;619;Not Applicable 0;Not Available;Not Available Scheduled Follow-up Submission;Additional New Tumor Event;Not Available Complete Remission/Response;Stable Disease;Not Evaluated Not Available;70;Not Available Not Available;Not Available;Not Available 7;4;5 Not Available;Not Available;Not Available Not Available;YES;NO Adjuvant therapy;Other;Not Available TUMOR FREE;WITH TUMOR;WITH TUMOR YES;Not Available;Not Available Complete Remission/Response;Not Available;Not Available Not Available;Not Available;Not Available NO;Not Available;Not Available Alive;Alive;Dead 2011;2012;2012 FEMALE Lung Adenocarcinoma- Not Otherwise Specified (NOS) No C34.1 8140/3 C34.1 YES Not Available NO Not Available Not Available Not Available 10 Not Available Not Available Not Available Not Available Not Available Not Available Not Available Not Available Not Available Not Available 30 No 4112 Not Available Not Available Not Available Not Available Not Available Not Available Not Available Not Available YES WHITE Not Available NA Distant Recurrence TCGA-44-4112-R30706 a33297b6-1b28-4a0e-af21-09030850c6b7 1 16 675 654 Not Available 4 14 3500 NO EXTERNAL BEAM Not Applicable PROGRESSION Not Available cGy 2012 R0 Not Applicable Not Applicable Not Applicable Not Applicable Not Applicable Not Applicable Not Applicable Not Applicable Not Applicable Stage IB Not Applicable Not Applicable Not Applicable 6th Not Applicable Not Applicable Not Applicable M0 N0 T2a 1992 YES NO 44 3 Lung Alive 2010 2010 1972

1229 Dead T1 N0 MX Stage IA NA 51 R-Upper Not Applicable TCGA-49-AARE CD9E70E4-8622-4A07-8646-63F8275C1737 19 -18893 1229 0 Not Available Not Available Lung Adenocarcinoma 107.2 NA 1 Not Available Not Available Not Available Not Available Not Available Not Available Not Available NOT HISPANIC OR LATINO NA FEMALE Lung Adenocarcinoma- Not Otherwise Specified (NOS) No C34.1 8140/3 C34.1 YES 90 Not Available Not Available Not Available Not Available 6 YES YES 388 YES 392 Not Available Not Available Locoregional Recurrence Biopsy with Histologic Confirmation;Convincing Imaging YES 15 No AARE Other WITH TUMOR 55.2 41.7 NO 81 61.2 Complete Remission/Response YES BLACK OR AFRICAN AMERICAN NO NA R0 Not Applicable Not Applicable Not Applicable Not Applicable Not Applicable Not Applicable Not Applicable Not Applicable Not Applicable Stage IA Not Applicable Not Applicable Not Applicable 5th Not Applicable Not Applicable Not Applicable MX N0 T1 1995 NO YES 49 4 Lung Dead 2014 1999 1980

476 Alive T2a N0 M0 Stage IB NA 42 R-Lower Not Applicable TCGA-95-7948 a52e99d6-a61a-439d-b0b1-ca7a0eabcb04 22 -15363 Not Applicable 0 133 Not Available Lung Adenocarcinoma 87 NA Not Available Not Available Not Available Not Available Not Available Not Available Not Available Not Available NOT HISPANIC OR LATINO NA Not Available;Not Available Not Available;Not Available Not Available;Not Available Not Available;Not Available TCGA-95-7948-F29540;TCGA-95-7948-F40945 DE1F1595-55A3-445C-A427-26050FE637FA;BDEC4035-069A-4823-947C-D9277CAF627E 22;7 Not Applicable;Not Applicable Not Applicable;Not Applicable Not Applicable;Not Applicable 133;476 Not Applicable;Not Applicable Not Available;Not Available Scheduled Follow-up Submission;Scheduled Follow-up Submission Not Available;Complete Remission/Response Not Available;Not Available Not Available;NO 3;3 Not Available;Not Available NO;NO Not Available;Not Available TUMOR FREE;TUMOR FREE NO;NO Not Available;Complete Remission/Response Not Available;Not Available NO;NO Alive;Alive 2012;2013 FEMALE Lung Adenocarcinoma Mixed Subtype No C34.3 8255/3 C34.3 YES Not Available Not Available Not Available Not Available Not Available 3 Not Available Not Available Not Available Not Available Not Available Not Available Not Available Not Available Not Available Not Available .15 Yes 7948 Not Available TUMOR FREE Not Available 79 Not Available Not Available 72 Not Available YES WHITE Not Available NA R0 Not Applicable Not Applicable Not Applicable Not Applicable Not Applicable Not Applicable Not Applicable Not Applicable Not Applicable Stage IB Not Applicable Not Applicable Not Applicable 7th Not Applicable Not Applicable Not Applicable M0 N0 T2a 1995 YES NO 95 3 Lung Alive 2012 2011 1993

997 Alive T1b N0 M0 Stage IA NA 69 L-Upper Not Applicable TCGA-86-7953 54775a66-08cc-4f38-98f2-e7b2b5cec994 14 -25315 Not Applicable 0 34 Not Available Lung Adenocarcinoma Not Available NA Not Available Not Available NO Not Available Not Available Not Available Not Available Not Available NOT HISPANIC OR LATINO NA Not Available;Not Available Not Available;Not Available Not Available;Not Available Not Available;Not Available TCGA-86-7953-F41095;TCGA-86-7953-F66262 F62E2EA8-719A-48F5-8571-DB4C59F3A166;5ACE5873-EB42-4FFE-84C9-1DA5D2DD3D9B 12;7 Not Applicable;Not Applicable Not Applicable;Not Applicable Not Applicable;Not Applicable 591;997 Not Applicable;Not Applicable 1;1 Scheduled Follow-up Submission;Scheduled Follow-up Submission Complete Remission/Response;Complete Remission/Response 90;90 NO;NO 3;10 Not Available;Not Available NO;NO Preoperative;Preoperative TUMOR FREE;TUMOR FREE NO;NO Complete Remission/Response;Complete Remission/Response Not Available;Not Available NO;NO Alive;Alive 2013;2014 FEMALE Lung Adenocarcinoma- Not Otherwise Specified (NOS) No C34.1 8250/3 C34.1 YES Not Available Not Available Not Available Not Available Peripheral Lung 3 Not Available Not Available Not Available Not Available Not Available Not Available Not Available Not Available Not Available Not Available Not Available No 7953 Not Available TUMOR FREE Not Available Not Available Not Available Not Available Not Available Not Available NO WHITE Not Available NA RX Not Applicable Not Applicable Not Applicable Not Applicable Not Applicable Not Applicable Not Applicable Not Applicable Not Applicable Stage IA Not Applicable Not Applicable Not Applicable 7th Not Applicable Not Applicable Not Applicable M0 N0 T1b Not Available YES NO 86 1 Lung Alive 2012 2011 Not Available

791 Alive T3 N0 MX Stage IIB NA 77 L-Lower Not Applicable TCGA-55-8513 a3fd20b2-e001-44ab-9716-754e5ae70808 11 -28284 Not Applicable 0 146 Not Available Lung Adenocarcinoma 65 NA TCGA-55-8513-D64284;TCGA-55-8513-D64285 2FEFA276-5D66-46D2-B73C-6138E1E304B9;3D2150F8-FBEF-4037-83F8-E9BB0CD7746E Not Available;Not Available 28;28 460;Not Available 352;561 Tarceva;Alimta Clinical Progressive Disease;Not Applicable 8;8 Not Available;Not Available Not Available;Not Available Not Available;Not Available Not Available;Not Available Not Applicable;Not Applicable Not Available;Not Available Not Available;Not Available NO;YES Chemotherapy;Chemotherapy Not Available;Not Available Not Available;Not Available Not Available;Not Available NO;NO 2014;2014 Not Evaluated Not Available YES Not Available Not Available Not Available YES Not Available NOT HISPANIC OR LATINO NA YES NO NO NO TCGA-55-8513-F64283 BED3B17C-69EA-4302-B3E2-D8C6371B867F 28 Not Available Not Available Not Applicable 791 317 2 Scheduled Follow-up Submission Stable Disease 60 NO 8 Distant Metastasis YES Other WITH TUMOR NO Complete Remission/Response Convincing Imaging NO Alive 2014 FEMALE Mucinous (Colloid) Carcinoma No C34.30 8480/3 C34.3 YES Not Evaluated Unknown Not Available Not Available Unknown 1 Not Available Not Available Not Available Not Available Not Available Not Available Not Available Not Available Not Available NO Not Available No 8513 Not Evaluated TUMOR FREE Not Available Not Available NO 108 87 Complete Remission/Response YES WHITE NO NA R0 Not Applicable Not Applicable Not Applicable Not Applicable Not Applicable Not Applicable Not Applicable Not Applicable Not Applicable Stage IIB Not Applicable Not Applicable Not Applicable 7th Not Applicable Not Applicable Not Applicable MX N0 T3 Not Available YES NO 55 1 Lung Alive 2013 2012 Not Available

434 Dead T2 N0 M0 Stage IB NA 75 L-Upper Not Applicable TCGA-50-5931 12ccd581-a921-41bc-bcee-4e9be54532cc 2 -27733 434 0 Not Available Not Available Lung Adenocarcinoma Not Available NA Not Available Not Available Not Available Not Available Not Available Not Available Not Available Not Available NOT HISPANIC OR LATINO NA NO NO Not Available NO TCGA-50-5931-F32067 6abc6f4d-700c-429a-8a96-cab76c1bd8ae 16 Not Available Not Available 434 Not Available 425 Not Available Scheduled Follow-up Submission Progressive Disease Not Available NO 5 Distant Metastasis YES Not Available WITH TUMOR NO Progressive Disease Convincing Imaging NO Dead 2012 FEMALE Lung Adenocarcinoma- Not Otherwise Specified (NOS) No C34.1 8140/3 C34.1 YES Not Available Not Available Not Available Not Available Central Lung 7 Not Available Not Available Not Available Not Available Not Available Not Available Not Available Not Available Not Available Not Available 40 No 5931 Not Available TUMOR FREE Not Available Not Available Not Available Not Available Not Available Not Available Not Available WHITE Not Available NA R0 Not Applicable Not Applicable Not Applicable Not Applicable Not Applicable Not Applicable Not Applicable Not Applicable Not Applicable Stage IB Not Applicable Not Applicable Not Applicable 5th Not Applicable Not Applicable Not Applicable M0 N0 T2 Not Available NO YES 50 4 Lung Dead 2011 2001 Not Available

1175 Alive T1b N0 M0 Stage IA NA 57 L-Upper Not Applicable TCGA-MN-A4N4 CEEAD734-1CE0-4385-B65A-A9C853B7308E 4 -20888 Not Applicable 0 622 Not Available Lung Adenocarcinoma Not Available NA 1 Not Available NO Not Available Not Available Not Available NO Not Available NOT HISPANIC OR LATINO NA Not Available Not Available Not Available Not Available TCGA-MN-A4N4-F56667 6E245328-C4AD-4FC6-BC4E-0967868C7228 17 Not Applicable Not Applicable Not Applicable 1175 Not Applicable Not Available Scheduled Follow-up Submission Complete Remission/Response Not Available NO 2 Not Available NO Not Available TUMOR FREE NO Complete Remission/Response Not Available NO Alive 2014 MALE Lung Adenocarcinoma- Not Otherwise Specified (NOS) No C34.1 8140/3 C34.1 YES 100 NO Not Available Not Available Unknown 12 Not Available Not Available Not Available Not Available Not Available Not Available Not Available Not Available Not Available NO Not Available No A4N4 Preoperative Unknown 75 82 NO 70 77 Complete Remission/Response YES WHITE NO NA R0 Not Applicable Not Applicable Not Applicable Not Applicable Not Applicable Not Applicable Not Applicable Not Applicable Not Applicable Stage IA Not Applicable Not Applicable Not Applicable 7th Not Applicable Not Applicable Not Applicable M0 N0 T1b Not Available NO YES MN 2 Lung Alive 2012 2010 1980

930 Alive T1a N1 M0 NA 58 R-Upper Not Applicable TCGA-55-5899 996a0070-e9bf-4601-8ef7-60212108258d 20 Not Available Not Applicable 0 87 Not Available Lung Adenocarcinoma Not Available NA TCGA-55-5899-D43195;TCGA-55-5899-D43196 C4183530-28BE-42D7-AD22-5C1C476E2388;314FCD94-F0EA-4781-95F8-659D257724EB Not Available;Not Available 15;15 208;208 146;146 Carboplatin;Taxol Complete Response;Complete Response 5;5 Not Available;Not Available Not Available;Not Available Not Available;Not Available Not Available;Not Available Not Applicable;Not Applicable Not Available;Not Available Not Available;Not Available NO;NO Chemotherapy;Chemotherapy Not Available;Not Available Not Available;Not Available Not Available;Not Available NO;NO 2013;2013 Not Available Not Available Not Available Not Available Not Available Not Available Not Available Not Available NOT HISPANIC OR LATINO NA Not Available Not Available Not Available Not Available TCGA-55-5899-F43194 2C5BA987-9730-46DB-B69A-829E5803E4EF 15 Not Applicable Not Applicable Not Applicable 930 Not Applicable 0 Scheduled Follow-up Submission Complete Remission/Response 100 NO 5 Not Available NO Post-Adjuvant Therapy TUMOR FREE YES Complete Remission/Response Not Available NO Alive 2013 MALE Lung Adenocarcinoma- Not Otherwise Specified (NOS) No C34.1 8140/3 C34.1 YES Not Available Not Available Not Available Not Available Not Available 9 Not Available Not Available Not Available Not Available Not Available Not Available Not Available Not Available Not Available Not Available Not Available No 5899 Not Available Not Available Not Available Not Available Not Available Not Available Not Available Not Available Not Available WHITE Not Available NA R0 Not Applicable Not Applicable Not Applicable Not Applicable Not Applicable Not Applicable Not Applicable Not Applicable Not Applicable Discrepancy Not Applicable Not Applicable Not Applicable 7th Not Applicable Not Applicable Not Applicable M0 N1 T1a Not Available NO YES 55 2 Lung Alive 2010 2010 1985

499 Alive T2a N0 M0 Stage IB NA 59 L-Lower Not Applicable TCGA-97-8177 09078761-1c14-4d01-8e15-535c19a9b0f9 13 -21648 Not Applicable 0 147 Not Available Lung Adenocarcinoma 97 NA TCGA-97-8177-D32866;TCGA-97-8177-D32868;TCGA-97-8177-D44245 E1EB8A22-561A-4E54-9A4A-AE4C3AB3056A;AA1A57A5-C0DC-4888-A6D2-E6C632A31929;46424E43-2394-4493-8F96-9A71AF33D4F8 Not Available;Not Available;Not Available 14;14;19 Not Available;Not Available;Not Available 93;93;205 Alimta;CARBOplatin;Tarceva Not Available;Not Available;Not Available 6;6;6 Not Available;Not Available;Not Available Not Available;Not Available;Not Available Not Available;Not Available;Not Available Not Available;Not Available;Not Available Not Applicable;Not Applicable;Not Applicable Not Available;Not Available;Not Available Not Available;Not Available;Not Available YES;YES;YES Chemotherapy;Chemotherapy;Targeted Molecular therapy Not Available;Not Available;Not Available Not Available;Not Available;Not Available Not Available;Not Available;Not Available NO;NO;NO 2012;2012;2013 0 Not Available YES L858R Not Available Not Available NO Not Available NOT HISPANIC OR LATINO NA Not Available Not Available Not Available Not Available TCGA-97-8177-F44243 76A1E7C1-9C2C-4E07-A5AA-389AA2168572 19 Not Applicable Not Applicable Not Applicable 499 Not Applicable Not Evaluated Scheduled Follow-up Submission Complete Remission/Response Not Evaluated NO 6 Not Available NO Not Evaluated TUMOR FREE YES Complete Remission/Response Not Available NO Alive 2013 FEMALE Lung Adenocarcinoma Mixed Subtype No C34.3 8255/3 C34.3 YES Not Available YES NO Not Available Not Available 6 Not Available Not Available Not Available Not Available Not Available Not Available Not Available Not Available Not Available NO Not Available No 8177 Preoperative TUMOR FREE 82 109 YES 80 106 Not Evaluated YES WHITE NO NA Not Available Not Applicable Not Applicable Not Applicable Not Applicable Not Applicable Not Applicable Not Applicable Not Applicable Not Applicable Stage IB Not Applicable Not Applicable Not Applicable 7th Not Applicable Not Applicable Not Applicable M0 N0 T2a Not Available YES NO 97 1 Lung Alive 2012 2012 Not Available

626 Alive T1b N0 MX Stage IA NA 81 L-Lower Not Applicable TCGA-55-A491 A250A369-E536-4A18-B7AF-2911133D7BDC 21 -29593 Not Applicable 0 0 Not Available Lung Adenocarcinoma 68 NA Not Evaluated Not Available NO Not Available Not Available Not Available NO Not Available NOT HISPANIC OR LATINO NA Not Available Not Available Not Available Not Available TCGA-55-A491-F60134 83201851-01DF-44E5-AB4C-DCB1F355360C 29 Not Applicable Not Applicable Not Applicable 626 Not Applicable 2 Scheduled Follow-up Submission Complete Remission/Response 80 NO 5 Not Available NO Other TUMOR FREE NO Complete Remission/Response Not Available NO Alive 2014 FEMALE Lung Adenocarcinoma- Not Otherwise Specified (NOS) No C34.3 8140/3 C34.3 YES Not Evaluated NO Not Available Not Available Unknown 2 Not Available Not Available Not Available Not Available Not Available Not Available Not Available Not Available Not Available NO 45 Yes, History of Prior Malignancy A491 Not Available TUMOR FREE 73 68 Unknown 72 64 Unknown YES WHITE Unknown NA R0 Not Applicable Not Applicable Not Applicable Not Applicable Not Applicable Not Applicable Not Applicable Not Applicable Not Applicable Stage IA Not Applicable Not Applicable Not Applicable 7th Not Applicable Not Applicable Not Applicable MX N0 T1b 1982 YES NO 55 3 Lung Alive 2013 2012 1952

551 Alive T2a N0 M0 Stage IB NA 55 L-Upper Not Applicable TCGA-97-8175 f4350d9c-3f40-4829-bbc4-8acb2f3ff512 13 -20206 Not Applicable 0 87 Not Available Lung Adenocarcinoma Not Available NA TCGA-97-8175-D44236;TCGA-97-8175-D44238 9EF4D129-7435-454B-B14A-430A7F80A624;8165798D-0A35-4166-92DD-22748F0BB7A0 Not Available;Not Available 19;19 Not Available;Not Available 472;472 Alimta;Cisplatin Not Available;Not Available 6;6 Not Available;Not Available Not Available;Not Available Not Available;Not Available Not Available;Not Available Not Applicable;Not Applicable Not Available;Not Available Not Available;Not Available YES;YES Chemotherapy;Chemotherapy Not Available;Not Available Not Available;Not Available Not Available;Not Available NO;NO 2013;2013 Not Available Not Available YES Not Available Not Available FISH YES Not Available NOT HISPANIC OR LATINO NA YES YES YES Not Available TCGA-97-8175-F44234 891298DD-4123-4570-8D90-B7AF4B6AF406 19 294 Not Available Not Applicable 551 294 Not Evaluated Scheduled Follow-up Submission Complete Remission/Response Not Evaluated NO 6 Locoregional Recurrence YES Not Available TUMOR FREE NO Complete Remission/Response Biopsy with Histologic Confirmation NO Alive 2013 FEMALE Lung Solid Pattern Predominant Adenocarcinoma No C34.1 8230/3 C34.1 YES Not Available YES NO Not Available Peripheral Lung 6 Not Available Not Available Not Available Not Available Not Available Not Available Not Available Not Available Not Available NO Not Available No 8175 Not Available TUMOR FREE Not Available Not Available NO Not Available Not Available Complete Remission/Response NO WHITE NO NA Local Recurrence TCGA-97-8175-R44237 54EAF59D-B6E1-49F1-BE58-190269254416 Not Available 19 441 427 Complete Response 6 5 5000 NO External Not Applicable Not Available Not Available cGy 2013 Not Available Not Applicable Not Applicable Not Applicable Not Applicable Not Applicable Not Applicable Not Applicable Not Applicable Not Applicable Stage IB Not Applicable Not Applicable Not Applicable 7th Not Applicable Not Applicable Not Applicable M0 N0 T2a 1981 YES NO 97 3 Lung Alive 2012 2011 Not Available

372 Alive T1b N0 M0 Stage IA NA 59 R-Upper Not Applicable TCGA-91-6840 d5326429-9805-47f9-97b0-fbda658e3f01 15 -21854 Not Applicable 0 0 Not Available Lung Adenocarcinoma Not Available NA Not Available Not Available Not Available Not Available Not Available Not Available Not Available Not Available NOT HISPANIC OR LATINO NA Not Available Not Available Not Available Not Available TCGA-91-6840-F21664 733715F8-948C-4764-9C90-5A5A19666BC2 8 Not Applicable Not Applicable Not Applicable 372 Not Applicable Not Available Scheduled Follow-up Submission Not Available Not Available Not Available 5 Not Available NO Not Available TUMOR FREE NO Complete Remission/Response Not Available NO Alive 2012 FEMALE Lung Adenocarcinoma- Not Otherwise Specified (NOS) No C34.1 8140/3 C34.1 YES Not Available NO Not Available Not Available Not Available 7 Not Available Not Available Not Available Not Available Not Available Not Available Not Available Not Available Not Available Not Available 50 No 6840 Not Available TUMOR FREE Not Available Not Available Not Available Not Available Not Available Not Available NO WHITE Not Available NA R0 Not Applicable Not Applicable Not Applicable Not Applicable Not Applicable Not Applicable Not Applicable Not Applicable Not Applicable Stage IA Not Applicable Not Applicable Not Applicable 7th Not Applicable Not Applicable Not Applicable M0 N0 T1b 1996 YES NO 91 3 Lung Alive 2011 2011 1971

62 Dead T4 N0 M0 Stage IIIA NA 71 L-Upper Not Applicable TCGA-64-5775 c8544fb3-b26e-4672-bb81-a271db724b04 2 -26058 62 0 Not Available Not Available Lung Adenocarcinoma Not Available NA TCGA-64-5775-D11419;TCGA-64-5775-D11422 4d9280cc-1045-45ad-9f20-9ea5dc1ea652;7c68848c-8750-490a-811a-2d79abb12d7a Not Available;Not Available 3;3 28;34 0;0 Carboplatin;Taxol Not Available;Not Available 5;5 02;05 5;80 AUC;mg/m2 OTHER, SPECIFY IN NOTES;OTHER, SPECIFY IN NOTES Neo-Adjuvant;Neo-Adjuvant 1;1 IV;IV NO;NO Chemotherapy;Chemotherapy Not Available;Not Available Not Available;Not Available Not Available;Not Available Not Available;Not Available 2011;2011 Not Available Not Available Not Available Not Available Not Available Not Available Not Available Not Available NOT HISPANIC OR LATINO NA Not Available Not Available Not Available Not Available TCGA-64-5775-F11418 ef8d69d0-68e9-401f-a2c7-795dffabb80f 2 Not Applicable Not Applicable 62 Not Available Not Applicable Not Available Not Available Progressive Disease 0 Not Available 5 Not Available NO Not Available WITH TUMOR NO Progressive Disease Not Available NO Dead 2011 MALE Lung Adenocarcinoma- Not Otherwise Specified (NOS) Yes C34.1 8140/3 C34.1 YES Not Available Not Available Not Available Not Available Not Available 5 Not Available Not Available Not Available Not Available Not Available Not Available Not Available Not Available Not Available Not Available 110 No 5775 Not Available WITH TUMOR Not Available Not Available Not Available Not Available Not Available Not Available Not Available WHITE Not Available NA R0 Not Applicable Not Applicable Not Applicable Not Applicable Not Applicable Not Applicable Not Applicable Not Applicable Not Applicable Stage IIIA Not Applicable Not Applicable Not Applicable Not Available Not Applicable Not Applicable Not Applicable M0 N0 T4 Not Available NO YES 64 2 Lung Dead 2011 2005 1950

845 Alive T1b NX M0 Stage IA NA FPPP TCGA 67 R-Upper Not Applicable TCGA-44-6147 889aec8e-14ba-48d9-8fe1-f2416e82b333 5 -24631 Not Applicable 0 236 Not Available Lung Adenocarcinoma 68 NA Not Available Not Available Not Available Not Available Not Available Not Available Not Available Not Available NOT HISPANIC OR LATINO NA Not Available;NO Not Available;NO Not Available;NO NO;Not Available TCGA-44-6147-F20460;TCGA-44-6147-F38947 7717ec1a-6c2f-486f-a3f4-3a5d0ddb4e2d;286EBBD3-255A-40BB-AC90-973B1E9D0203 13;4 Not Applicable;Not Applicable Not Applicable;Not Applicable Not Applicable;Not Applicable 441;845 Not Applicable;Not Applicable 2;Not Available Scheduled Follow-up Submission;Scheduled Follow-up Submission Complete Remission/Response;Complete Remission/Response Not Available;80 Not Available;NO 1;1 Not Available;Not Available NO;NO Other;Other TUMOR FREE;TUMOR FREE NO;NO Complete Remission/Response;Complete Remission/Response Not Available;Not Available NO;NO Alive;Alive 2012;2013 FEMALE Lung Adenocarcinoma- Not Otherwise Specified (NOS) No C34.1 8140/3 C34.1 YES Not Available Not Available Not Available Not Available Not Available 7 Not Available Not Available Not Available Not Available Not Available Not Available Not Available Not Available Not Available Not Available Not Available Yes 6147 Not Available TUMOR FREE Not Available Not Available Not Available 105 97 Not Available YES WHITE Not Available NA Not Available Not Applicable Not Applicable Not Applicable Not Applicable Not Applicable Not Applicable Not Applicable Not Applicable Not Applicable Stage IA Not Applicable Not Applicable Not Applicable 7th Not Applicable Not Applicable Not Applicable M0 NX T1b 1980 YES NO 44 3 Lung Alive 2011 2010 1976

1790 Dead T1 N2 MX Stage IIIA NA 76 L-Lower Not Applicable TCGA-MP-A4T6 5F4934BE-EEE3-40E1-8838-38103BADC898 2 -27993 1790 0 Not Available Not Available Lung Adenocarcinoma 63 NA Not Evaluated Not Available NO Not Available Not Available Not Available NO Not Available NOT HISPANIC OR LATINO NA FEMALE Lung Adenocarcinoma- Not Otherwise Specified (NOS) No C34.3 8140/3 C34.3 YES Not Evaluated NO Not Available Not Available Peripheral Lung 4 Not Available Not Available Not Available Not Available Not Available Not Available Not Available Not Available Not Available NO 50 No A4T6 Not Evaluated TUMOR FREE Not Available 79 NO Not Available 77 Complete Remission/Response YES WHITE NO NA R0 Not Applicable Not Applicable Not Applicable Not Applicable Not Applicable Not Applicable Not Applicable Not Applicable Not Applicable Stage IIIA Not Applicable Not Applicable Not Applicable 6th Not Applicable Not Applicable Not Applicable MX N2 T1 1989 NO YES MP 3 Lung Dead 2013 2005 Not Available

415 Alive T1b N0 M0 Stage IA NA 73 L-Upper Not Applicable TCGA-44-A4SS F934ED41-090E-4313-BBC5-D1A4A9ED9AD7 28 -26991 Not Applicable 0 114 Not Available Lung Adenocarcinoma 54 NA 0 Not Available Not Available Not Available Not Available Not Available Not Available Not Available NOT HISPANIC OR LATINO NA Not Available Not Available Not Available Not Available TCGA-44-A4SS-F49165 7E3B2EA0-2129-451F-A502-B57F589DC4F8 26 Not Applicable Not Applicable Not Applicable 415 Not Applicable Not Evaluated Scheduled Follow-up Submission Complete Remission/Response Not Evaluated NO 9 Not Available NO Not Evaluated TUMOR FREE NO Complete Remission/Response Not Available NO Alive 2013 MALE Lung Adenocarcinoma- Not Otherwise Specified (NOS) No C34.1 8140/3 C34.1 YES Not Available Not Available Not Available Not Available Not Available 1 Not Available Not Available Not Available Not Available Not Available Not Available Not Available Not Available Not Available NO 90 No A4SS Preoperative TUMOR FREE 88 71 NO 86 69 Complete Remission/Response YES WHITE NO NA Not Available Not Applicable Not Applicable Not Applicable Not Applicable Not Applicable Not Applicable Not Applicable Not Applicable Not Applicable Stage IA Not Applicable Not Applicable Not Applicable 7th Not Applicable Not Applicable Not Applicable M0 N0 T1b 2012 YES NO 44 4 Lung Alive 2013 2012 1952

343 Dead T2a N1 MX Stage IIA NA 77 L-Upper Not Applicable TCGA-55-7907 bf755bc2-b7aa-4c3c-8b3c-58b089b5cab6 10 -28201 Not Applicable 0 16 Not Available Lung Adenocarcinoma 75 NA Not Available Not Available Not Available Not Available Not Available Not Available Not Available Not Available NOT HISPANIC OR LATINO NA NO NO Not Available NO TCGA-55-7907-F60132 6ECFF27E-F203-441F-A6A5-2608A1B7989E 29 Not Available Not Available 343 Not Available 294 Not Evaluated Scheduled Follow-up Submission Progressive Disease Not Evaluated NO 5 Distant Metastasis YES Not Evaluated WITH TUMOR NO Complete Remission/Response Convincing Imaging NO Dead 2014 MALE Lung Adenocarcinoma- Not Otherwise Specified (NOS) No C34.1 8140/3 C34.1 YES Not Available Not Available Not Available Not Available Not Available 3 Not Available Not Available Not Available Not Available Not Available Not Available Not Available Not Available Not Available Not Available 80 No 7907 Not Available TUMOR FREE 80 87 Not Available 75 76 Not Available YES WHITE Not Available NA R0 Not Applicable Not Applicable Not Applicable Not Applicable Not Applicable Not Applicable Not Applicable Not Applicable Not Applicable Stage IIA Not Applicable Not Applicable Not Applicable 7th Not Applicable Not Applicable Not Applicable MX N1 T2a 1994 YES NO 55 3 Lung Alive 2012 2011 1954

353 Alive T2a N0 M0 Stage IB NA 57 L-Upper Not Applicable TCGA-86-8585 2f1a4b16-104c-40eb-8114-8245847bb716 30 -20918 Not Applicable 0 35 Not Available Lung Adenocarcinoma Not Available NA Unknown Not Available NO Not Available Not Available Not Available Unknown Not Available NOT HISPANIC OR LATINO NA Not Available Not Available Not Available Not Available TCGA-86-8585-F46400 FCD3444C-82E2-4973-B06F-3F0471930298 19 Not Applicable Not Applicable Not Applicable 353 Not Applicable Not Evaluated Scheduled Follow-up Submission Complete Remission/Response Not Evaluated YES 9 Not Available NO Not Evaluated TUMOR FREE NO Complete Remission/Response Not Available NO Alive 2013 MALE Lung Solid Pattern Predominant Adenocarcinoma No C34.1 8230/3 C34.1 YES Unknown NO Not Available Not Available Peripheral Lung 8 Not Available Not Available Not Available Not Available Not Available Not Available Not Available Not Available Not Available Unknown Not Available No 8585 Unknown TUMOR FREE Not Available Not Available Unknown Not Available Not Available Complete Remission/Response NO WHITE Unknown NA R0 Not Applicable Not Applicable Not Applicable Not Applicable Not Applicable Not Applicable Not Applicable Not Applicable Not Applicable Stage IB Not Applicable Not Applicable Not Applicable 7th Not Applicable Not Applicable Not Applicable M0 N0 T2a Not Available YES NO 86 1 Lung Alive 2012 2012 Not Available

230 Alive T2a N0 M0 Stage IB NA 42 R-Middle Not Applicable TCGA-69-7973 dc9eaaa0-0bfd-46d0-994f-310826879da4 2 -15355 Not Applicable 0 230 Not Available Lung Adenocarcinoma Not Available NA TCGA-69-7973-D30332;TCGA-69-7973-D30341;TCGA-69-7973-D30344;TCGA-69-7973-D30346 2B5DC03D-AFEF-4C32-BB62-02A37D57897F;8C513402-CD6E-424D-9844-E3390F02D0C3;D947DBDE-9875-4B03-AE2A-F2B3AE12CCD8;8C538EDD-BE6D-4A7E-8936-5487EB1589D3 Not Available;Not Available;Not Available;Not Available 9;9;9;9 63;63;132;132 63;63;91;91 Cisplatin;Taxotere;Cisplatin;Pemetrexed Unknown;Unknown;Complete Response;Complete Response 4;4;4;4 Not Available;Not Available;Not Available;Not Available Not Available;Not Available;Not Available;Not Available Not Available;Not Available;Not Available;Not Available Not Available;Not Available;Not Available;Not Available Not Applicable;Not Applicable;Not Applicable;Not Applicable Not Available;Not Available;Not Available;Not Available Not Available;Not Available;Not Available;Not Available NO;NO;NO;NO Chemotherapy;Chemotherapy;Chemotherapy;Chemotherapy Not Available;Not Available;Not Available;Not Available Not Available;Not Available;Not Available;Not Available Not Available;Not Available;Not Available;Not Available NO;NO;NO;NO 2012;2012;2012;2012 1 Not Available YES Not Available Not Available Not Available Not Available Not Available NOT HISPANIC OR LATINO NA NO NO NO NO TCGA-69-7973-F30347 3A3CBBC0-50FA-42BB-88C1-99E0CF4F765C 9 Not Applicable Not Applicable Not Applicable 230 Not Applicable 1 Scheduled Follow-up Submission Complete Remission/Response Not Available Not Available 4 Not Available NO Post-Adjuvant Therapy TUMOR FREE YES Complete Remission/Response Not Available NO Alive 2012 FEMALE Lung Adenocarcinoma Mixed Subtype No C34.2 8255/3 C34.2 YES Not Available Not Available Not Available Not Available Central Lung 4 Not Available Not Available Not Available Not Available Not Available Not Available Not Available Not Available Not Available Not Available 25 No 7973 Pre-Adjuvant Therapy TUMOR FREE Not Available Not Available Not Available Not Available Not Available Not Available NO WHITE Not Available NA Not Available Not Applicable Not Applicable Not Applicable Not Applicable Not Applicable Not Applicable Not Applicable Not Applicable Not Applicable Stage IB Not Applicable Not Applicable Not Applicable 7th Not Applicable Not Applicable Not Applicable M0 N0 T2a 2011 NO YES 69 4 Lung Alive 2012 2011 Not Available

617 Alive T4 N1 M0 Stage IIIA NA 49 L-Upper Not Applicable TCGA-NJ-A7XG 01E9888D-B5B9-48F1-8BA6-8A89AF108A04 22 -18127 Not Applicable 0 295 Not Available Lung Adenocarcinoma Not Available NA TCGA-NJ-A7XG-D71392;TCGA-NJ-A7XG-D71393;TCGA-NJ-A7XG-D71463 722B8970-EEDA-4CD9-B2C5-9FE8E763C119;4006A0DF-514F-45D2-8CFA-20E163DF66DE;3340775B-1C82-4EB8-9F90-84B465A9C799 Not Available;Not Available;Not Available 25;25;25 100;128;128 30;100;30 Cisplatin;carboplatin;Pemetrexed Stable Disease;Stable Disease;Stable Disease 3;3;3 Not Available;Not Available;Not Available Not Available;Not Available;Not Available Not Available;Not Available;Not Available Not Available;Not Available;Not Available Not Applicable;Not Applicable;Not Applicable Not Available;Not Available;Not Available Not Available;Not Available;Not Available NO;NO;NO Chemotherapy;Chemotherapy;Chemotherapy Not Available;Not Available;Not Available Not Available;Not Available;Not Available Not Available;Not Available;Not Available NO;NO;NO 2015;2015;2015 Not Available Not Available YES Other Not Available Not Available Not Available Not Available NOT HISPANIC OR LATINO NA Not Available Not Available Not Available Not Available TCGA-NJ-A7XG-F71390 79C34A2C-8467-47E1-BC78-F4652344C551 25 Not Applicable Not Applicable Not Applicable 617 Not Applicable Not Available Scheduled Follow-up Submission Stable Disease Not Available NO 3 Not Available NO Not Available WITH TUMOR YES Stable Disease Not Available NO Alive 2015 MALE Lung Adenocarcinoma- Not Otherwise Specified (NOS) No C34.1 8140/3 C34.1 YES Not Available YES NO Not Available Not Available 5 Not Available Not Available Not Available Not Available Not Available Not Available Not Available Not Available Not Available NO Not Available No A7XG Not Available WITH TUMOR Not Available Not Available YES Not Available Not Available Stable Disease NO BLACK OR AFRICAN AMERICAN NO NA Not Available Not Applicable Not Applicable Not Applicable Not Applicable Not Applicable Not Applicable Not Applicable Not Applicable Not Applicable Stage IIIA Not Applicable Not Applicable Not Applicable 7th Not Applicable Not Applicable Not Applicable M0 N1 T4 Not Available Not Available Not Available NJ 3 Lung Alive 2014 2013 Not Available

500 Dead T2 N1 MX Stage IIB NA 50 Discrepancy Not Available TCGA-44-6779 cbbea9f1-396a-4bf3-b67c-2cac3394dceb 31 -18469 500 0 Not Available Not Available Lung Adenocarcinoma 44 NA TCGA-44-6779-D16019;TCGA-44-6779-D16027;TCGA-44-6779-D16021;TCGA-44-6779-D16023 cf85260a-95a7-40dd-85dc-fe3146f7e584;53ef5ace-fd50-4f47-a1ff-3a77d45be688;4d765560-c992-4deb-be5c-3f95e52efc0a;ea0269ad-e8e3-4fa6-a6fd-adc142fc13a0 Not Available;Not Available;Not Available;Not Available 12;12;12;12 141;435;141;303 77;391;77;257 Taxol;Gemcitabine;Carboplatin;Taxotere Not Available;Not Available;Not Available;Not Available 9;9;9;9 4;5;4;3 340;1600;650;130 mg;mg;mg;mg ADJUVANT;PROGRESSION;ADJUVANT;RECURRENCE Not Applicable;Not Applicable;Not Applicable;Not Applicable 1;3;1;2 IV;IV;IV;IV NO;NO;NO;NO Chemotherapy;Chemotherapy;Chemotherapy;Chemotherapy Not Available;Not Available;Not Available;Not Available 1360;8000;2600;390 mg;mg;mg;mg Not Available;Not Available;Not Available;Not Available 2011;2011;2011;2011 Not Available Not Available Not Available Not Available Not Available Not Available Not Available Not Available Not Available NA YES NO NO Not Available TCGA-44-6779-F15758 8f506af0-e7ee-46c3-b57c-ab3eace80f10 12 Not Available Not Available 500 Not Available 232 Not Available Scheduled Follow-up Submission Not Available Not Available Not Available 9 Not Available YES Not Available WITH TUMOR YES Progressive Disease Not Available NO Dead 2011 FEMALE Lung Adenocarcinoma- Not Otherwise Specified (NOS) No C34.1 8140/3 C34.9 YES Not Available Not Available Not Available Not Available Not Available 8 Not Available Not Available Not Available Not Available Not Available Not Available Not Available Not Available Not Available Not Available 15 No 6779 Not Available WITH TUMOR 83 48 Not Available 83 49 Not Available YES WHITE Not Available NA Not Available Not Applicable Not Applicable Not Applicable Not Applicable Not Applicable Not Applicable Not Applicable Not Applicable Not Applicable Stage IIB Not Applicable Not Applicable Not Applicable 6th Not Applicable Not Applicable Not Applicable MX N1 T2 2008 NO YES 44 4 Lung Dead 2011 2008 1978

119 Dead T2 N0 M1 Stage IV NA 63 L-Lower Not Applicable TCGA-50-6591 9b132e4f-7e35-4cc5-8711-43ad62b906d0 25 -23067 119 0 Not Available Not Available Lung Adenocarcinoma Not Available NA Not Available Not Available Not Available Not Available Not Available Not Available Not Available Not Available NOT HISPANIC OR LATINO NA Not Available Not Available Not Available Not Available TCGA-50-6591-F43833 911B7995-6444-4317-A9F1-A366B72E0186 4 Not Applicable Not Applicable 119 Not Available Not Applicable Not Available Scheduled Follow-up Submission Progressive Disease Not Available NO 6 Not Available NO Not Available WITH TUMOR NO Progressive Disease Not Available NO Dead 2013 FEMALE Lung Adenocarcinoma- Not Otherwise Specified (NOS) No C34.3 8140/3 C34.3 YES Not Available Not Available Not Available Not Available Not Available 8 Not Available Not Available Not Available Not Available Not Available Not Available Not Available Not Available Not Available Not Available Not Available No 6591 Not Available WITH TUMOR Not Available Not Available Not Available Not Available Not Available Not Available Not Available WHITE Not Available NA Not Available Not Applicable Not Applicable Not Applicable Not Applicable Not Applicable Not Applicable Not Applicable Not Applicable Not Applicable Stage IV Not Applicable Not Applicable Not Applicable 6th Not Applicable Not Applicable Not Applicable M1 N0 T2 Not Available NO YES 50 1 Lung Dead 2011 2007 Not Available

339 Dead T1 N0 M0 Stage IA NA 62 R-Upper Not Applicable TCGA-MP-A4TJ 8F52CCCD-5709-4753-9670-31A573EE27AB 2 -22875 339 0 Not Available Not Available Lung Adenocarcinoma 29 NA Not Evaluated Not Available NO Not Available Not Available Not Available NO Not Available Unknown NA FEMALE Lung Signet Ring Adenocarcinoma No C34.1 8490/3 C34.1 YES Not Evaluated NO Not Available Not Available Central Lung 4 Not Available Not Available Not Available Not Available Not Available Not Available Not Available Not Available Not Available Unknown Not Available No A4TJ Not Evaluated Unknown 30.1 36 NO 29.7 27 Complete Remission/Response YES Unknown NO NA R0 Not Applicable Not Applicable Not Applicable Not Applicable Not Applicable Not Applicable Not Applicable Not Applicable Not Applicable Stage IA Not Applicable Not Applicable Not Applicable 6th Not Applicable Not Applicable Not Applicable M0 N0 T1 Not Available NO YES MP 2 Lung Dead 2013 2006 Not Available

1285 Alive T1 N0 MX Stage IA NA 76 L-Upper Not Applicable TCGA-97-7546 1d90c9f3-5025-4867-a2f0-e455b6a254fa 17 -27913 Not Applicable 0 964 Not Available Lung Adenocarcinoma 60 NA TCGA-97-7546-D32027 BFAB42A6-E8C6-4F2C-AAE1-8C7D88AB5CFD Not Available 15 Not Available 1255 Tarceva Not Available 5 Not Available Not Available Not Available Not Available Not Applicable Not Available Not Available YES Chemotherapy Not Available Not Available Not Available NO 2012 Not Available Not Available Not Available Not Available Not Available Not Available Not Available Not Available NOT HISPANIC OR LATINO NA YES YES Not Available Not Available TCGA-97-7546-F31995 14A442B8-34A0-48DF-A9B0-6768DA7DE62F 15 Not Available Not Available Not Applicable 1285 1255 Not Available Additional New Tumor Event Not Evaluated Not Available NO 5 New Primary Tumor YES Not Available WITH TUMOR YES Progressive Disease Convincing Imaging YES Alive 2012 FEMALE Lung Acinar Adenocarcinoma No C34.1 8550/3 C34.1 YES Not Available Not Available Not Available Not Available Not Available 11 Not Available Not Available Not Available Not Available Not Available Not Available Not Available Not Available Not Available Not Available 25 No 7546 Not Available TUMOR FREE 96 84 Not Available 100 85 Not Available YES WHITE Not Available NA Primary Tumor Field TCGA-97-7546-R32026 85B50FCD-5DA2-4D3E-9A31-E2F14A14CEFB 1 15 1255 1255 Not Available 5 Not Available Not Available NO OTHER Direct to the Spot ADJUVANT Not Available Not Available 2012 R0 Not Applicable Not Applicable Not Applicable Not Applicable Not Applicable Not Applicable Not Applicable Not Applicable Not Applicable Stage IA Not Applicable Not Applicable Not Applicable 6th Not Applicable Not Applicable Not Applicable MX N0 T1 1983 NO YES 97 3 Lung Alive 2011 2008 1963

777 Dead T2 N0 M0 Stage IB NA 71 L-Lower Not Applicable TCGA-50-6592 09918b81-4696-4cba-a45a-5fe7407c2337 25 -26191 777 0 Not Available Not Available Lung Adenocarcinoma Not Available NA Not Available Not Available Not Available Not Available Not Available Not Available Not Available Not Available NOT HISPANIC OR LATINO NA Not Available Not Available Not Available Not Available TCGA-50-6592-F43835 BCFF54CF-3536-4F06-99D9-8C54307CBEFF 4 Not Applicable Not Applicable 777 Not Available Not Applicable Not Available Scheduled Follow-up Submission Progressive Disease Not Available NO 6 Not Available NO Not Available WITH TUMOR NO Progressive Disease Not Available YES Dead 2013 FEMALE Lung Adenocarcinoma- Not Otherwise Specified (NOS) No C34.3 8140/3 C34.3 YES Not Available Not Available Not Available Not Available Not Available 8 Not Available Not Available Not Available Not Available Not Available Not Available Not Available Not Available Not Available Not Available Not Available No 6592 Not Available WITH TUMOR Not Available Not Available Not Available Not Available Not Available Not Available Not Available WHITE Not Available NA Primary Tumor Field TCGA-50-6592-R43837 D910E461-431F-4861-97AF-9701837BFDE4 Not Available 4 39 39 Radiographic Progressive Disease 6 Not Available Not Available NO Internal Not Applicable Not Available Not Available Not Available 2013 Not Available Not Applicable Not Applicable Not Applicable Not Applicable Not Applicable Not Applicable Not Applicable Not Applicable Not Applicable Stage IB Not Applicable Not Applicable Not Applicable 6th Not Applicable Not Applicable Not Applicable M0 N0 T2 Not Available NO YES 50 4 Lung Dead 2011 2007 Not Available

609 Alive T3 N2 MX Stage IIIA NA 76 R-Upper Not Applicable TCGA-55-7283 ccfdad76-cc45-447f-bed8-ede8f6a8844d 6 -27946 Not Applicable 0 35 Not Available Lung Adenocarcinoma 64 NA TCGA-55-7283-D46989;TCGA-55-7283-D46990 E0804552-E06E-4FA2-A637-AFD1546794D4;1B14B0D0-2927-4B35-9310-FE9EC2E5726C Not Available;Not Available 21;21 386;386 83;83 Carboplatin;Alimta Partial Response;Partial Response 8;8 Not Available;Not Available Not Available;Not Available Not Available;Not Available Not Available;Not Available Not Applicable;Not Applicable Not Available;Not Available Not Available;Not Available NO;NO Chemotherapy;Chemotherapy Not Available;Not Available Not Available;Not Available Not Available;Not Available NO;NO 2013;2013 0 Not Available NO Not Available Not Available Not Available NO Not Available NOT HISPANIC OR LATINO NA Not Available Not Available Not Available Not Available TCGA-55-7283-F46988 CD19EF4F-EC9B-43BD-8940-11F6F54CB88D 21 Not Applicable Not Applicable Not Applicable 609 Not Applicable Unknown Scheduled Follow-up Submission Partial Remission/Response Unknown NO 8 Not Available NO Unknown WITH TUMOR YES Partial Remission/Response Not Available NO Alive 2013 FEMALE Lung Bronchioloalveolar Carcinoma Nonmucinous No C34.1 8252/3 C34.1 YES 100 NO Not Available Not Available Central Lung 10 Not Available Not Available Not Available Not Available Not Available Not Available Not Available Not Available Not Available Not Available 5 No 7283 Preoperative WITH TUMOR Not Available Not Available Not Available 105 82 Not Available YES WHITE Not Available NA Not Available Not Applicable Not Applicable Not Applicable Not Applicable Not Applicable Not Applicable Not Applicable Not Applicable Not Applicable Stage IIIA Not Applicable Not Applicable Not Applicable 7th Not Applicable Not Applicable Not Applicable MX N2 T3 1961 YES NO 55 3 Lung Alive 2011 2011 1950

469 Dead T1b N0 MX Stage IA NA 61 R-Upper Not Applicable TCGA-55-8299 2cd8ea73-cb3f-4b17-9b97-fa12eb03b85b 8 -22626 Not Applicable 0 216 Not Available Lung Adenocarcinoma Not Available NA Not Available Not Available Not Available Not Available Not Available Not Available Not Available Not Available NOT HISPANIC OR LATINO NA NO YES NO NO TCGA-55-8299-F57915 A9C89030-2543-4E48-87D9-5FA314877305 28 Not Available Not Available 469 Not Available 274 Not Evaluated Scheduled Follow-up Submission Progressive Disease Not Evaluated NO 3 Distant Metastasis YES Not Available WITH TUMOR NO Complete Remission/Response Convincing Imaging NO Dead 2014 FEMALE Lung Adenocarcinoma- Not Otherwise Specified (NOS) No C34.1 8140/3 C34.1 YES Not Available Not Available Not Available Not Available Unknown 10 Not Available Not Available Not Available Not Available Not Available Not Available Not Available Not Available Not Available NO 20 No 8299 Not Available TUMOR FREE Not Available Not Available NO 75 74 Complete Remission/Response YES WHITE NO NA Distant Recurrence TCGA-55-8299-R57916 A2DBBFEE-391A-4404-BA88-F6BBCBEF60A9 Not Available 28 320 304 Radiographic Progressive Disease 3 13 3250 NO External Not Applicable Not Available Not Available Not Available 2014 R0 Not Applicable Not Applicable Not Applicable Not Applicable Not Applicable Not Applicable Not Applicable Not Applicable Not Applicable Stage IA Not Applicable Not Applicable Not Applicable 7th Not Applicable Not Applicable Not Applicable MX N0 T1b 2012 YES NO 55 4 Lung Alive 2012 2012 1971

747 Alive T4 N0 M0 Stage IIIA NA 74 R-Lower Not Applicable TCGA-99-7458 96d93e23-bb0d-46a8-9685-5ed0d6b83059 6 -27359 Not Applicable 0 251 Not Available Lung Adenocarcinoma Not Available NA TCGA-99-7458-D20337 c261c20d-0ded-4038-a815-f22775bf86a6 Not Available 9 323 53 Tarceva (Erlotinib) Not Available 1 Not Available 150 mg/day ADJUVANT Not Applicable 1 PO NO Chemotherapy Not Available Not Available Not Available Not Available 2012 1 Not Available YES Not Available Not Available RT-PCR YES Not Available NOT HISPANIC OR LATINO NA Not Available;Not Available Not Available;Not Available Not Available;Not Available Not Available;Not Available TCGA-99-7458-F20338;TCGA-99-7458-F40777 6b1fe365-99fc-4a3b-8dcd-7bb0f50b1369;F3957B1D-CF6F-4A0B-8F7B-E040D49F7CC4 9;17 Not Applicable;Not Applicable Not Applicable;Not Applicable Not Applicable;Not Applicable 392;747 Not Applicable;Not Applicable 1;Unknown Scheduled Follow-up Submission;Scheduled Follow-up Submission Not Available;Stable Disease 90;Unknown Not Available;NO 1;4 Not Available;Not Available NO;NO Post-Adjuvant Therapy;Not Evaluated TUMOR FREE;WITH TUMOR YES;YES Not Available;Stable Disease Not Available;Not Available NO;NO Alive;Alive 2012;2013 FEMALE Lung Bronchioloalveolar Carcinoma Nonmucinous No C34.3 8252/3 C34.3 YES 90 YES YES G12A Not Available 1 Not Available Not Available Not Available Not Available Not Available Not Available Not Available Not Available Not Available Not Available 52 No 7458 Preoperative TUMOR FREE Not Available Not Available Not Available Not Available Not Available Not Available Not Available WHITE Not Available NA R0 Not Applicable Not Applicable Not Applicable Not Applicable Not Applicable Not Applicable Not Applicable Not Applicable Not Applicable Stage IIIA Not Applicable Not Applicable Not Applicable 7th Not Applicable Not Applicable Not Applicable M0 N0 T4 2010 NO YES 99 4 Lung Alive 2012 2010 1958

656 Dead TX NX M1 Stage IV NA 74 R-Upper Not Applicable TCGA-99-8033 9582b436-145b-4f84-8f7f-8b39b8254cc1 18 -27342 Not Applicable 0 170 Not Available Lung Adenocarcinoma Not Available NA TCGA-99-8033-D32956;TCGA-99-8033-D32958;TCGA-99-8033-D32959 AAAFB521-2D55-4F3A-A369-95F7C05964EB;05A9596F-9551-49BF-8D1F-F899C9375EFC;987496D9-FF47-4ADB-B105-C8AF22F185E4 Not Available;Not Available;Not Available 18;18;18 Not Available;78;Not Available 78;78;107 Carboplatin;Alimta;Taxol Not Applicable;Unknown;Not Applicable 6;6;6 Not Available;Not Available;Not Available Not Available;Not Available;Not Available Not Available;Not Available;Not Available Not Available;Not Available;Not Available Not Applicable;Not Applicable;Not Applicable Not Available;Not Available;Not Available Not Available;Not Available;Not Available YES;NO;YES Chemotherapy;Chemotherapy;Chemotherapy Not Available;Not Available;Not Available Not Available;Not Available;Not Available Not Available;Not Available;Not Available NO;NO;NO 2012;2012;2012 1 Not Available YES Not Available Not Available Not Available NO Not Available NOT HISPANIC OR LATINO NA Not Available Not Available Not Available Not Available TCGA-99-8033-F58623 D7BC0D18-7B7D-4404-86A3-C62CB2474F57 30 Not Applicable Not Applicable 656 Not Available Not Applicable 2 Scheduled Follow-up Submission Not Applicable Unknown NO 3 Not Available NO Post-Adjuvant Therapy WITH TUMOR YES Progressive Disease Not Available YES Dead 2015 FEMALE Lung Adenocarcinoma- Not Otherwise Specified (NOS) No C34.1 8140/3 C34.1 YES Not Evaluated YES NO Not Available Peripheral Lung 6 Not Available Not Available Not Available Not Available Not Available Not Available Not Available Not Available Not Available NO Not Available No 8033 Preoperative WITH TUMOR Not Available Not Available YES Not Available Not Available Unknown NO WHITE NO NA R0 Not Applicable Not Applicable Not Applicable Not Applicable Not Applicable Not Applicable Not Applicable Not Applicable Not Applicable Stage IV Not Applicable Not Applicable Not Applicable 7th Not Applicable Not Applicable Not Applicable M1 NX TX Not Available YES NO 99 1 Lung Alive 2012 2011 Not Available

629 Alive T1b N0 M0 Stage IA NA 74 R-Middle Not Applicable TCGA-97-A4M7 C6B680E3-D96E-4FC7-8053-77454B7A14FA 7 -27062 Not Applicable 0 271 Not Available Lung Adenocarcinoma 99 NA 0 Not Available YES L858R Not Available Not Available NO Not Available NOT HISPANIC OR LATINO NA Not Available Not Available Not Available Not Available TCGA-97-A4M7-F57383 FF856855-D4FF-4537-86B6-C69C98F1AA5C 10 Not Applicable Not Applicable Not Applicable 629 Not Applicable 0 Scheduled Follow-up Submission Complete Remission/Response Unknown NO 3 Not Available NO Other TUMOR FREE NO Complete Remission/Response Not Available NO Alive 2014 MALE Lung Adenocarcinoma Mixed Subtype No C34.2 8255/3 C34.2 YES Not Evaluated YES NO Not Available Unknown 3 Not Available Not Available Not Available Not Available Not Available Not Available Not Available Not Available Not Available NO 40 Yes, History of Synchronous/Bilateral Malignancy A4M7 Preoperative TUMOR FREE 82 101 NO 74 91 Complete Remission/Response YES WHITE NO NA R0 Not Applicable Not Applicable Not Applicable Not Applicable Not Applicable Not Applicable Not Applicable Not Applicable Not Applicable Stage IA Not Applicable Not Applicable Not Applicable 7th Not Applicable Not Applicable Not Applicable M0 N0 T1b 1978 YES NO 97 3 Lung Alive 2013 2012 1958

705 Alive T2a N0 MX Stage IB NA FPPP TCGA 72 R-Upper Not Applicable TCGA-44-6775 3c4ff061-d214-4d1c-8d2e-3034f207c252 24 -26415 Not Applicable 0 83 Not Available Lung Adenocarcinoma 66 NA Not Available Not Available Not Available Not Available Not Available Not Available Not Available Not Available HISPANIC OR LATINO NA Not Available;NO Not Available;NO Not Available;NO Not Available;Not Available TCGA-44-6775-F30930;TCGA-44-6775-F40408 b3652245-e7e8-4b5a-b946-07b14256210b;5A214384-F238-4FFD-A355-8617658950AD 19;19 Not Applicable;Not Available Not Applicable;Not Available Not Applicable;Not Applicable 370;705 Not Applicable;684 Not Available;Not Available Scheduled Follow-up Submission;Scheduled Follow-up Submission Complete Remission/Response;Progressive Disease Not Available;100 Not Available;NO 4;2 Not Available;Locoregional Recurrence NO;YES Not Available;Other TUMOR FREE;WITH TUMOR Not Available;NO Not Available;Complete Remission/Response Not Available;Convincing Imaging Not Available;NO Alive;Alive 2012;2013 FEMALE Lung Adenocarcinoma- Not Otherwise Specified (NOS) No C34.1 8140/3 C34.1 YES Not Available Not Available Not Available Not Available Not Available 8 Not Available Not Available Not Available Not Available Not Available Not Available Not Available Not Available Not Available Not Available 25 No 6775 Not Available Not Available 89 72 Not Available 84 68 Not Available YES WHITE Not Available NA RX Not Applicable Not Applicable Not Applicable Not Applicable Not Applicable Not Applicable Not Applicable Not Applicable Not Applicable Stage IB Not Applicable Not Applicable Not Applicable 7th Not Applicable Not Applicable Not Applicable MX N0 T2a 2005 YES NO 44 4 Lung Alive 2011 2011 1955

1622 Dead T2 N0 M0 Stage IB NA 72 R-Upper Not Applicable TCGA-78-7537 67a2bc21-7c87-4678-997d-a434de672436 13 -26610 1622 0 Not Available Not Available Lung Adenocarcinoma Not Available NA 1 Not Available NO Not Available Not Available Not Available NO Not Available Not Available NA Not Available Not Available Not Available Not Available TCGA-78-7537-F20455 6c5bda4d-e611-4338-b11b-ff753eac94d6 13 Not Available Not Available 1622 Not Available Not Available Not Available Scheduled Follow-up Submission Not Available Not Available Not Available 1 Not Available Not Available Not Available Not Available NO Not Available Not Available NO Dead 2012 MALE Lung Adenocarcinoma Mixed Subtype No C34.1 8255/3 C34.1 YES Not Available NO Not Available Not Available Not Available 1 Not Available Not Available Not Available Not Available Not Available Not Available Not Available Not Available Not Available Not Available 20 No 7537 Preoperative Not Available Not Available Not Available Not Available Not Available Not Available Not Available Not Available Not Available Not Available NA R0 Not Applicable Not Applicable Not Applicable Not Applicable Not Applicable Not Applicable Not Applicable Not Applicable Not Applicable Stage IB Not Applicable Not Applicable Not Applicable 6th Not Applicable Not Applicable Not Applicable M0 N0 T2 1984 YES NO 78 3 Lung Dead 2012 2002 1944

1183 Alive T2 N0 M0 Stage IB NA FPPP TCGA 33 R-Upper Not Applicable TCGA-44-3917 7099597a-b208-45b5-b9e0-11ab40d5e497 8 -12179 Not Applicable 0 392 Not Available Lung Adenocarcinoma 83 NA Not Available Not Available Not Available Not Available Not Available Not Available Not Available Not Available NOT HISPANIC OR LATINO NA Not Available;Not Available Not Available;Not Available Not Available;Not Available Not Available;Not Available TCGA-44-3917-F5229;TCGA-44-3917-F38799 e67f8c0a-a881-475e-80c7-42f9756190a8;0886A555-3179-409D-BF92-241BDEE939BF 18;24 Not Applicable;Not Applicable Not Applicable;Not Applicable Not Applicable;Not Applicable 392;1183 Not Applicable;Not Applicable Not Available;Not Available Not Available;Scheduled Follow-up Submission Not Available;Complete Remission/Response Not Available;100 Not Available;NO 10;12 Not Available;Not Available NO;NO Not Available;Post-Adjuvant Therapy TUMOR FREE;TUMOR FREE NO;NO Not Available;Complete Remission/Response Not Available;Not Available NO;NO Alive;Alive 2010;2012 FEMALE Lung Adenocarcinoma- Not Otherwise Specified (NOS) No C34.1 8140/3 C34.1 YES Not Available NO Not Available Not Available Not Available 10 Not Available Not Available Not Available Not Available Not Available Not Available Not Available Not Available Not Available Not Available 16 No 3917 Not Available TUMOR FREE Not Available Not Available Not Available 87 106 Not Available YES WHITE Not Available NA R0 Not Applicable Not Applicable Not Applicable Not Applicable Not Applicable Not Applicable Not Applicable Not Applicable Not Applicable Stage IB Not Applicable Not Applicable Not Applicable 6th Not Applicable Not Applicable Not Applicable M0 N0 T2 Not Available YES NO 44 2 Lung Alive 2010 2009 1993

428 Dead T2 N1 M0 Stage IIB NA 61 R-Lower Not Applicable TCGA-49-4505 088edf89-d2b5-40c7-b066-df3bd383f7ea 3 -22628 428 0 Not Available Not Available Lung Adenocarcinoma Not Available NA 3 Not Available Not Available Not Available Not Available Not Available Not Available Not Available NOT HISPANIC OR LATINO NA Unknown Unknown Unknown Not Available TCGA-49-4505-F58844 7535318E-69AD-45DE-A08C-DB0184BC487D 25 Not Available Not Available 428 Not Available 417 1 Scheduled Follow-up Submission Unknown 80 NO 4 Distant Metastasis;New Primary Tumor YES Preoperative WITH TUMOR NO Complete Remission/Response Convincing Imaging NO Dead 2014 FEMALE Lung Adenocarcinoma- Not Otherwise Specified (NOS) No C34.3 8140/3 C34.3 YES Not Available Not Available Not Available Not Available Not Available 2 Not Available Not Available Not Available Not Available Not Available Not Available Not Available Not Available Not Available Not Available 74 No 4505 Pre-Adjuvant Therapy WITH TUMOR Not Available Not Available Not Available Not Available Not Available Not Available NO WHITE Not Available NA R0 Not Applicable Not Applicable Not Applicable Not Applicable Not Applicable Not Applicable Not Applicable Not Applicable Not Applicable Stage IIB Not Applicable Not Applicable Not Applicable 4th Not Applicable Not Applicable Not Applicable M0 N1 T2 1986 NO YES 49 4 Lung Dead 2011 1996 1949

1431 Alive T4 N3 M0 Stage IIIB NA 47 R-Upper Not Applicable TCGA-05-4398 31c96e35-5e2f-429c-b12a-7bc5a497a300 22 -17471 Not Applicable 0 1431 Not Available Lung Adenocarcinoma Not Available NA TCGA-05-4398-D36463;TCGA-05-4398-D36465 9DC1C815-EC00-4CDE-BB84-3F8EB3DD9B4F;48CF8515-16A9-4E33-B2F5-AC6157DC8C51 Not Available;Not Available 5;5 92;92 0;0 Carboplatin;Etoposide Complete Response;Complete Response 12;12 Not Available;Not Available Not Available;Not Available Not Available;Not Available Not Available;Not Available Not Applicable;Not Applicable Not Available;Not Available Not Available;Not Available NO;NO Chemotherapy;Chemotherapy Not Available;Not Available Not Available;Not Available Not Available;Not Available NO;NO 2012;2012 Not Available Not Available Not Available Not Available Not Available Not Available Not Available Not Available Not Available NA Not Available Not Available Not Available Not Available TCGA-05-4398-F36462 F7EFBD0A-009C-48EE-B6F6-B67D4F976432 31 Not Applicable Not Applicable Not Applicable 1431 Not Applicable Not Available Scheduled Follow-up Submission Complete Remission/Response Not Available NO 10 Not Available NO Not Available TUMOR FREE YES Complete Remission/Response Not Available NO Alive 2012 FEMALE Lung Adenocarcinoma Mixed Subtype No C34.1 8255/3 C34.1 YES Not Available Not Available Not Available Not Available Not Available 7 Not Available Not Available Not Available Not Available Not Available Not Available Not Available Not Available Not Available Not Available 72 No 4398 Not Available TUMOR FREE Not Available Not Available Not Available Not Available Not Available Not Available Not Available Not Available Not Available NA R0 Not Applicable Not Applicable Not Applicable Not Applicable Not Applicable Not Applicable Not Applicable Not Applicable Not Applicable Stage IIIB Not Applicable Not Applicable Not Applicable 5th Not Applicable Not Applicable Not Applicable M0 N3 T4 Not Available NO YES 05 2 Lung Alive 2010 2006 Not Available

375 Dead T1a N1 M1b Stage IV NA 60 L-Upper Not Applicable TCGA-55-8620 5510ad32-62fd-4adc-964a-ae4e997c2f4b 11 -22091 Not Applicable 0 66 Not Available Lung Adenocarcinoma 64 NA 2 Not Available NO Not Available Not Available Not Available NO Not Available NOT HISPANIC OR LATINO NA Not Available Not Available Not Available Not Available TCGA-55-8620-F59081 B7AB11B2-6770-4031-B6CC-3416F978BF1A 30 Not Applicable Not Applicable 375 Not Available Not Applicable Not Evaluated Scheduled Follow-up Submission Progressive Disease Not Evaluated NO 4 Not Available NO Not Available WITH TUMOR NO Progressive Disease Not Available YES Dead 2014 MALE Lung Adenocarcinoma- Not Otherwise Specified (NOS) No C34.1 8140/3 C34.1 YES 70 NO Not Available Not Available Central Lung 1 Not Available Not Available Not Available Not Available Not Available Not Available Not Available Not Available Not Available NO 154 No 8620 Preoperative WITH TUMOR Not Available Not Available NO 73 76 Progressive Disease YES WHITE YES NA Distant site TCGA-55-8620-R59083 265BD0A0-A402-44D1-A79C-9846E6AFF684 Not Available 30 161 132 Radiographic Progressive Disease 4 18 3600 NO External Not Applicable Not Available Not Available cGy 2014 R0 Not Applicable Not Applicable Not Applicable Not Applicable Not Applicable Not Applicable Not Applicable Not Applicable Not Applicable Stage IV Not Applicable Not Applicable Not Applicable 7th Not Applicable Not Applicable Not Applicable M1b N1 T1a 2012 YES NO 55 4 Lung Alive 2013 2012 1965

NA T2 N0 M0 Stage IB NA Not Available L-Upper Not Applicable TCGA-75-5122 78ae716b-0ca5-4c01-a053-d41288385fcd 28 Not Available Not Available Not Available Not Available Not Available Lung Adenocarcinoma Not Available NA Not Available Not Available NO Not Available Not Available Not Available NO Not Available Not Available NA NO NO Not Available NO TCGA-75-5122-F11750 ded07f2c-1693-4ffa-b33d-e4c0e47bc2f2 31 Not Available Not Available Not Available Not Available Not Available Not Available Not Available Progressive Disease 0 Not Available 3 Not Available YES Adjuvant therapy WITH TUMOR NO Complete Remission/Response Not Available NO Dead 2011 MALE Lung Adenocarcinoma- Not Otherwise Specified (NOS) No C34.1 8140/3 C34.1 YES 0 NO Not Available Not Available Peripheral Lung 3 Not Available Not Available Not Available Not Available Not Available Not Available Not Available Not Available Not Available Not Available 20 No 5122 Other WITH TUMOR Not Available Not Available Not Available Not Available Not Available Not Available Not Available Not Available Not Available NA R0 Not Applicable Not Applicable Not Applicable Not Applicable Not Applicable Not Applicable Not Applicable Not Applicable Not Applicable Stage IB Not Applicable Not Applicable Not Applicable Not Available Not Applicable Not Applicable Not Applicable M0 N0 T2 1985 NO YES 75 3 Lung Dead 2011 2005 Not Available

141 Alive T1a N0 M0 Stage IA NA 57 R-Middle Not Applicable TCGA-67-6216 ccecce70-dd54-4c5b-bf9f-ca87d290cb00 3 -20967 Not Applicable 0 141 Not Available Lung Adenocarcinoma Not Available NA Not Available Not Available NO Not Available Not Available Not Available NO Not Available NOT HISPANIC OR LATINO NA Not Available Not Available Not Available Not Available TCGA-67-6216-F13587 106303e5-cdb6-4df7-9e15-6f4d77d4dd50 6 Not Applicable Not Applicable Not Applicable 141 Not Applicable Not Available Scheduled Follow-up Submission Not Available Not Available Not Available 7 Not Available NO Not Available TUMOR FREE NO Complete Remission/Response Not Available NO Alive 2011 FEMALE Lung Adenocarcinoma- Not Otherwise Specified (NOS) No C34.2 8140/3 C34.2 YES Not Available NO Not Available Not Available Central Lung 6 Not Available Not Available Not Available Not Available Not Available Not Available Not Available Not Available Not Available Not Available Not Available No 6216 Not Available TUMOR FREE Not Available Not Available Not Available Not Available Not Available Not Available Not Available WHITE Not Available NA R0 Not Applicable Not Applicable Not Applicable Not Applicable Not Applicable Not Applicable Not Applicable Not Applicable Not Applicable Stage IA Not Applicable Not Applicable Not Applicable 7th Not Applicable Not Applicable Not Applicable M0 N0 T1a Not Available YES NO 67 1 Lung Alive 2011 2010 Not Available

442 Alive T1a N0 MX Stage IA NA 68 R-Lower Not Applicable TCGA-55-7725 9fcdccae-676e-4071-93c3-23d2d3ab0c00 8 -24848 Not Applicable 0 39 Not Available Lung Adenocarcinoma Not Available NA 1 Not Available NO Not Available Not Available Not Available NO Not Available NOT HISPANIC OR LATINO NA Not Available Not Available Not Available Not Available TCGA-55-7725-F46647 6AC6EF5E-2614-45DD-8C0B-9F1053CF037A 13 Not Applicable Not Applicable Not Applicable 442 Not Applicable 0 Scheduled Follow-up Submission Complete Remission/Response 100 NO 8 Not Available NO Post-Adjuvant Therapy TUMOR FREE NO Complete Remission/Response Not Available NO Alive 2013 FEMALE Lung Adenocarcinoma- Not Otherwise Specified (NOS) No C34.3 8140/3 C34.3 YES 90 NO Not Available Not Available Central Lung 3 Not Available Not Available Not Available Not Available Not Available Not Available Not Available Not Available Not Available Not Available 16 Yes 7725 Preoperative TUMOR FREE 88 64 Not Available 89 61 Not Available YES WHITE Not Available NA R0 Not Applicable Not Applicable Not Applicable Not Applicable Not Applicable Not Applicable Not Applicable Not Applicable Not Applicable Stage IA Not Applicable Not Applicable Not Applicable 7th Not Applicable Not Applicable Not Applicable MX N0 T1a 1994 YES NO 55 3 Lung Alive 2012 2011 1963

1178 Alive T2 N2 M0 Stage IIIA NA 68 R-Lower Not Applicable TCGA-55-1594 6d72de06-232a-4983-a06c-eba6d82cb3f1 14 -25008 Not Applicable 0 1178 1178 Lung Adenocarcinoma Not Available NA Not Available Not Available Not Available Not Available Not Available Not Available Not Available Not Available NOT HISPANIC OR LATINO NA MALE Lung Adenocarcinoma- Not Otherwise Specified (NOS) No C34.3 8140/3 C34.3 YES Not Available Not Available Not Available Not Available Not Available 9 Not Available Not Available Not Available Not Available Not Available Not Available Not Available Not Available Not Available Not Available Not Available No 1594 Not Available TUMOR FREE Not Available Not Available Not Available Not Available Not Available Not Available Not Available WHITE Not Available NA R0 Not Applicable Not Applicable Not Applicable Not Applicable Not Applicable Not Applicable Not Applicable Not Applicable Not Applicable Stage IIIA Not Applicable Not Applicable Not Applicable Not Available Not Applicable Not Applicable Not Applicable M0 N2 T2 Not Available NO YES 55 2 Lung Alive 2010 2005 1965

626 Alive T1a N0 MX Stage I NA 55 L-Lower Not Applicable TCGA-97-8552 9ca1a26c-82e0-48b3-b3bb-2ed57086eeb1 26 -20331 Not Applicable 0 115 Not Available Lung Adenocarcinoma 75 NA 0 Not Available YES Exon 19 Deletion Not Available Not Available NO Not Available NOT HISPANIC OR LATINO NA Not Available Not Available Not Available Not Available TCGA-97-8552-F49557 D1BF3872-D42B-4E2D-8D18-D1AE4F0E4BDF 3 Not Applicable Not Applicable Not Applicable 626 Not Applicable 0 Scheduled Follow-up Submission Complete Remission/Response Not Available NO 10 Not Available NO Other TUMOR FREE NO Complete Remission/Response Not Available NO Alive 2013 FEMALE Lung Bronchioloalveolar Carcinoma Nonmucinous No C34.3 8252/3 C34.3 YES Not Available YES NO Not Available Unknown 9 Not Available Not Available Not Available Not Available Not Available Not Available Not Available Not Available Not Available NO Not Available No 8552 Preoperative TUMOR FREE Not Available Not Available NO 77 95 Complete Remission/Response YES WHITE NO NA Not Evaluated Not Applicable Not Applicable Not Applicable Not Applicable Not Applicable Not Applicable Not Applicable Not Applicable Not Applicable Stage I Not Applicable Not Applicable Not Applicable 7th Not Applicable Not Applicable Not Applicable MX N0 T1a Not Available YES NO 97 1 Lung Alive 2012 2012 Not Available

1847 Alive T1 N0 M0 Stage IA NA 67 R-Upper Not Applicable TCGA-50-5942 75dd635e-8e0c-4c0b-b21c-20f77770218d 26 -24781 Not Applicable 0 136 Not Available Lung Adenocarcinoma Not Available NA Not Available Not Available Not Available Not Available Not Available Not Available Not Available Not Available NOT HISPANIC OR LATINO NA Not Available;NO Not Available;YES Not Available;Not Available Not Available;Not Available TCGA-50-5942-F32133;TCGA-50-5942-F70452 266b3dcd-9cf6-4c25-adb5-da7f91e8ce40;709DA356-FD1C-424F-9DD2-8B1AFBB6A742 17;16 Not Applicable;Not Available Not Applicable;Not Available Not Applicable;Not Applicable 883;1847 Not Applicable;1378 Not Available;Not Available Scheduled Follow-up Submission;Scheduled Follow-up Submission Stable Disease;Unknown Not Available;Not Available NO;NO 5;2 Not Available;Locoregional Recurrence NO;YES Not Available;Not Available TUMOR FREE;WITH TUMOR NO;NO Stable Disease;Stable Disease Not Available;Convincing Imaging YES;YES Alive;Alive 2012;2015 FEMALE Lung Adenocarcinoma- Not Otherwise Specified (NOS) No C34.1 8140/3 C34.1 YES Not Available Not Available Not Available Not Available Not Available 8 Not Available Not Available Not Available Not Available Not Available Not Available Not Available Not Available Not Available Not Available 30 No 5942 Not Available Not Available Not Available Not Available Not Available Not Available Not Available Not Available NO WHITE Not Available NA Primary Tumor Field;Local Recurrence TCGA-50-5942-R32134;TCGA-50-5942-R70453 b1859915-5ae6-41cf-a335-c12501ca2e70;D7ED9FE9-30E7-4159-B185-18F1303BF56F 1;Not Available 17;16 53;1641 53;1641 Not Available;Radiographic Progressive Disease 5;2 Not Available;Not Available Not Available;Not Available NO;NO IMPLANTS;External Not Applicable;Not Applicable ADJUVANT;Not Available Not Available;Not Available Not Available;Not Available 2012;2015 R0 Not Applicable Not Applicable Not Applicable Not Applicable Not Applicable Not Applicable Not Applicable Not Applicable Not Applicable Stage IA Not Applicable Not Applicable Not Applicable 7th Not Applicable Not Applicable Not Applicable M0 N0 T1 2009 NO YES 50 4 Lung Alive 2011 2009 Not Available

1531 Dead T2a N0 M0 Stage IB NA 81 R-Upper Not Applicable TCGA-73-7499 7782ceaa-da49-4232-8b3f-534971adaad8 31 -29683 Not Applicable 0 519 Not Available Lung Adenocarcinoma 54 NA 0 Not Available NO Not Available Not Available Not Available NO Not Available NOT HISPANIC OR LATINO NA Not Available;NO Not Available;NO Not Available;Not Available Not Available;YES TCGA-73-7499-F48754;TCGA-73-7499-F69827 B21C6B2B-AE23-49B9-B9FE-0F1511785AA0;4A31205D-4A32-435E-8909-40A109975FC6 20;21 Not Applicable;Not Available Not Applicable;1451 Not Applicable;1531 715;Not Available Not Applicable;1447 1;3 Scheduled Follow-up Submission;Scheduled Follow-up Submission Complete Remission/Response;Complete Remission/Response 80;40 NO;NO 9;1 Not Available;Distant Metastasis NO;YES Preoperative;Other TUMOR FREE;TUMOR FREE NO;NO Complete Remission/Response;Complete Remission/Response Not Available;Not Available NO;NO Alive;Dead 2013;2015 FEMALE Lung Adenocarcinoma Mixed Subtype No C34.1 8255/3 C34.1 YES 90 NO Not Available Not Available Peripheral Lung 5 Not Available Not Available Not Available Not Available Not Available Not Available Not Available Not Available Not Available NO Not Available No 7499 Other TUMOR FREE Not Available Not Available NO 103.7 91.5 Complete Remission/Response YES WHITE NO NA R0 Not Applicable Not Applicable Not Applicable Not Applicable Not Applicable Not Applicable Not Applicable Not Applicable Not Applicable Stage IB Not Applicable Not Applicable Not Applicable 7th Not Applicable Not Applicable Not Applicable M0 N0 T2a Not Available NO YES 73 1 Lung Alive 2012 2010 Not Available

291 Dead T3 N0 M0 Stage IIB NA 69 L-Upper Not Applicable TCGA-78-7161 86faf16c-56fd-4b7b-a6b2-b4c83bec93e1 27 -25294 Not Applicable 0 215 Not Available Lung Adenocarcinoma Not Available NA TCGA-78-7161-D18780;TCGA-78-7161-D18781 0303cacf-83d5-48d3-970a-5ba7b72c1bc6;d8438615-27ba-430d-b895-ba73933869c4 Not Available;Not Available 25;25 132;132 40;40 Cisplatin;Vinorelbine Not Available;Not Available 11;11 4;4 80;40 mg;mg ADJUVANT;ADJUVANT Not Applicable;Not Applicable 1;1 IV;IV NO;NO Chemotherapy;Chemotherapy Not Available;Not Available 600;450 mg;mg Not Available;Not Available 2011;2011 1 Not Available YES Not Available Not Available Not Available NO Not Available Not Available NA NO;Unknown NO;Unknown NO;NO NO;Not Available TCGA-78-7161-F18779;TCGA-78-7161-F46153 943f77cc-64c8-471a-9f85-66e8393a2463;864BF99A-DB82-4628-9D33-4CC4606FDFB6 25;23 Not Available;Not Available Not Available;Not Available Not Applicable;291 215;Not Available 162;162 Not Available;Unknown Scheduled Follow-up Submission;Scheduled Follow-up Submission Not Available;Progressive Disease Not Available;Not Available Not Available;NO 11;8 Not Available;Locoregional Recurrence YES;YES Not Available;Unknown WITH TUMOR;WITH TUMOR YES;YES Progressive Disease;Progressive Disease Not Available;Biopsy with Histologic Confirmation NO;NO Alive;Dead 2011;2013 FEMALE Lung Adenocarcinoma Mixed Subtype No C34.1 8255/3 C34.1 YES Not Available NO Not Available Not Available Peripheral Lung 10 Not Available Not Available Not Available Not Available Not Available Not Available Not Available Not Available Not Available Not Available 27.8 No 7161 Preoperative WITH TUMOR Not Available Not Available Not Available Not Available Not Available Not Available NO WHITE Not Available NA R1 Not Applicable Not Applicable Not Applicable Not Applicable Not Applicable Not Applicable Not Applicable Not Applicable Not Applicable Stage IIB Not Applicable Not Applicable Not Applicable 7th Not Applicable Not Applicable Not Applicable M0 N0 T3 1998 NO YES 78 4 Lung Alive 2011 2011 1961

484 Alive T1b N0 MX Stage IA NA 72 L-Upper Not Applicable TCGA-97-7941 b266cbf5-80de-4473-b16f-08c502d365ca 13 -26298 Not Applicable 0 22 Not Available Lung Adenocarcinoma 57 NA 0 Not Available YES Not Available Not Available Not Available NO Not Available NOT HISPANIC OR LATINO NA Not Available Not Available Not Available Not Available TCGA-97-7941-F33765 BB66B454-B72A-45B2-9CF6-FDF7E2D2CE4D 13 Not Applicable Not Applicable Not Applicable 484 Not Applicable Not Evaluated Scheduled Follow-up Submission Not Available Not Evaluated NO 8 Not Available NO Not Evaluated TUMOR FREE NO Complete Remission/Response Not Available NO Alive 2012 FEMALE Lung Acinar Adenocarcinoma No C34.1 8550/3 C34.1 YES Not Available YES YES G12A Not Available 2 Not Available Not Available Not Available Not Available Not Available Not Available Not Available Not Available Not Available Not Available 22.5 No 7941 Preoperative TUMOR FREE 81 98 Not Available 94 97 Not Available YES WHITE Not Available NA R0 Not Applicable Not Applicable Not Applicable Not Applicable Not Applicable Not Applicable Not Applicable Not Applicable Not Applicable Stage IA Not Applicable Not Applicable Not Applicable 7th Not Applicable Not Applicable Not Applicable MX N0 T1b 1985 YES NO 97 3 Lung Alive 2012 2011 1960

1778 Dead T2 N1 M0 Stage IIB NA 53 L-Upper Not Applicable TCGA-MP-A4SW 15F12D2C-B834-4BFC-B526-F03BF85BFE96 2 -19640 1778 0 Not Available Not Available Lung Adenocarcinoma 102 NA Not Evaluated Not Available NO Not Available Not Available Not Available NO Not Available Unknown NA MALE Lung Adenocarcinoma- Not Otherwise Specified (NOS) No C34.1 8140/3 C34.1 YES Not Evaluated NO Not Available Not Available Unknown 4 Not Available Not Available Not Available Not Available Not Available Not Available Not Available Not Available Not Available Unknown 15 No A4SW Not Evaluated Unknown Not Available 101 NO Not Available 90 Complete Remission/Response YES WHITE NO NA R0 Not Applicable Not Applicable Not Applicable Not Applicable Not Applicable Not Applicable Not Applicable Not Applicable Not Applicable Stage IIB Not Applicable Not Applicable Not Applicable 5th Not Applicable Not Applicable Not Applicable M0 N1 T2 1982 NO YES MP 3 Lung Dead 2013 2002 1967

62 Alive T2a N0 M0 Stage IB NA 69 L-Upper Not Applicable TCGA-05-5715 e0a19099-5d25-40df-ad73-6260df3a1b32 22 -25383 Not Applicable 0 62 Not Available Lung Adenocarcinoma Not Available NA Not Available Not Available Not Available Not Available Not Available Not Available Not Available Not Available Not Available NA FEMALE Lung Adenocarcinoma Mixed Subtype No C34.1 8255/3 C34.1 YES Not Available Not Available Not Available Not Available Not Available 3 Not Available Not Available Not Available Not Available Not Available Not Available Not Available Not Available Not Available Not Available Not Available No 5715 Not Available TUMOR FREE Not Available Not Available Not Available Not Available Not Available Not Available Not Available Not Available Not Available NA R0 Not Applicable Not Applicable Not Applicable Not Applicable Not Applicable Not Applicable Not Applicable Not Applicable Not Applicable Stage IB Not Applicable Not Applicable Not Applicable 7th Not Applicable Not Applicable Not Applicable M0 N0 T2a Not Available NO YES 05 1 Lung Alive 2011 2010 Not Available

457 Dead T4 N1 M1 Stage IV NA 67 R-Lower Not Applicable TCGA-05-4434 d94fe32f-bedd-4f4c-9feb-fcb60d40f4bb 22 -24472 457 0 Not Available Not Available Lung Adenocarcinoma Not Available NA Not Available Not Available Not Available Not Available Not Available Not Available Not Available Not Available Not Available NA FEMALE Lung Adenocarcinoma Mixed Subtype No C34.3 8255/3 C34.3 YES Not Available Not Available Not Available Not Available Not Available 7 Not Available Not Available Not Available Not Available Not Available Not Available Not Available Not Available Not Available Not Available 15 Yes 4434 Not Available Not Available Not Available Not Available Not Available Not Available Not Available Not Available Not Available Not Available Not Available NA R2 Not Applicable Not Applicable Not Applicable Not Applicable Not Applicable Not Applicable Not Applicable Not Applicable Not Applicable Stage IV Not Applicable Not Applicable Not Applicable 6th Not Applicable Not Applicable Not Applicable M1 N1 T4 Not Available NO YES 05 2 Lung Dead 2010 2008 Not Available

537 Alive T1a N0 MX Stage IA NA 70 R-Middle Not Applicable TCGA-55-7911 1f0a5312-0590-4230-9da9-fd0cfe693586 10 -25746 Not Applicable 0 21 Not Available Lung Adenocarcinoma Not Available NA Not Available Not Available NO Not Available Not Available Not Available NO Not Available NOT HISPANIC OR LATINO NA NO YES NO NO TCGA-55-7911-F48785 6C67B112-4633-4A5E-8731-C18F764ADB3E 20 Not Available Not Available Not Applicable 537 515 Unknown Scheduled Follow-up Submission Complete Remission/Response Unknown NO 9 New Primary Tumor YES Not Available TUMOR FREE NO Complete Remission/Response Not Available NO Alive 2013 FEMALE Lung Adenocarcinoma- Not Otherwise Specified (NOS) No C34.2 8140/3 C34.2 YES Not Available NO Not Available Not Available Not Available 3 Not Available Not Available Not Available Not Available Not Available Not Available Not Available Not Available Not Available Not Available 96 No 7911 Not Available TUMOR FREE Not Available 86 Not Available Not Available 64 Not Available YES WHITE Not Available NA Primary Tumor Field TCGA-55-7911-R48786 D8885BBE-DB76-4E6B-BE99-41D87580699B Not Available 20 536 524 Complete Response 9 5 6000 NO External Not Applicable Not Available Not Available cGy 2013 R0 Not Applicable Not Applicable Not Applicable Not Applicable Not Applicable Not Applicable Not Applicable Not Applicable Not Applicable Stage IA Not Applicable Not Applicable Not Applicable 7th Not Applicable Not Applicable Not Applicable MX N0 T1a 1981 YES NO 55 3 Lung Alive 2012 2011 1959

704 Alive T2a N0 MX Stage IB NA 64 L-Upper Not Applicable TCGA-55-7728 db769d5c-a794-448d-9130-38a82d051c95 8 -23505 Not Applicable 0 24 Not Available Lung Adenocarcinoma 64 NA Not Available Not Available NO Not Available Not Available Not Available NO Not Available NOT HISPANIC OR LATINO NA Not Available Not Available Not Available Not Available TCGA-55-7728-F47841 CCDF4AD4-BA17-413E-8B00-5016A4E94A75 29 Not Applicable Not Applicable Not Applicable 704 Not Applicable Not Evaluated Scheduled Follow-up Submission Complete Remission/Response Not Evaluated NO 8 Not Available NO Not Evaluated TUMOR FREE NO Complete Remission/Response Not Available NO Alive 2013 FEMALE Mucinous (Colloid) Carcinoma No C34.1 8480/3 C34.1 YES Not Available NO Not Available Not Available Not Available 3 Not Available Not Available Not Available Not Available Not Available Not Available Not Available Not Available Not Available Not Available 30 No 7728 Not Available TUMOR FREE Not Available 74 Not Available Not Available 67 Not Available YES WHITE Not Available NA R0 Not Applicable Not Applicable Not Applicable Not Applicable Not Applicable Not Applicable Not Applicable Not Applicable Not Applicable Stage IB Not Applicable Not Applicable Not Applicable 7th Not Applicable Not Applicable Not Applicable MX N0 T2a 1996 YES NO 55 4 Lung Alive 2012 2011 1980

539 Alive T2a N0 MX Stage IB NA 55 R-Lower Not Applicable TCGA-55-8510 962fd4e7-9199-43d9-abce-e3dfa46da62c 11 -20410 Not Applicable 0 228 Not Available Lung Adenocarcinoma 54 NA Not Evaluated Not Available Unknown Not Available Not Available Not Available Unknown Not Available NOT HISPANIC OR LATINO NA Not Available Not Available Not Available Not Available TCGA-55-8510-F57923 DA25CA2A-4E7D-4A2E-83C3-00BD003968B2 28 Not Applicable Not Applicable Not Applicable 539 Not Applicable Not Evaluated Scheduled Follow-up Submission Complete Remission/Response Not Evaluated NO 3 Not Available NO Not Available TUMOR FREE NO Complete Remission/Response Not Available NO Alive 2014 FEMALE Lung Adenocarcinoma- Not Otherwise Specified (NOS) No C34.3 8140/3 C34.3 YES Not Evaluated Unknown Not Available Not Available Unknown 1 Not Available Not Available Not Available Not Available Not Available Not Available Not Available Not Available Not Available NO Not Available No 8510 Not Available TUMOR FREE 90 70 NO 98 70 Complete Remission/Response YES WHITE NO NA R0 Not Applicable Not Applicable Not Applicable Not Applicable Not Applicable Not Applicable Not Applicable Not Applicable Not Applicable Stage IB Not Applicable Not Applicable Not Applicable 7th Not Applicable Not Applicable Not Applicable MX N0 T2a Not Available YES NO 55 2 Lung Alive 2013 2012 Not Available

417 Alive T2 N0 MX Stage IB NA 52 R-Upper Not Applicable TCGA-91-6836 0b31dbdf-0623-48e4-a0ec-017650dceda7 20 -19216 Not Applicable 0 11 Not Available Lung Adenocarcinoma 97 NA Not Available Not Available NO Not Available Not Available Not Available Not Available Not Available NOT HISPANIC OR LATINO NA Not Available Not Available Not Available Not Available TCGA-91-6836-F32646 5fc630d3-b549-4447-ba28-c379c0b08fa4 5 Not Applicable Not Applicable Not Applicable 417 Not Applicable Unknown Scheduled Follow-up Submission Complete Remission/Response Unknown NO 6 Not Available NO Not Available TUMOR FREE NO Complete Remission/Response Not Available NO Alive 2012 FEMALE Lung Adenocarcinoma- Not Otherwise Specified (NOS) No C34.1 8140/3 C34.1 YES Not Available NO Not Available Not Available Not Available 7 Not Available Not Available Not Available Not Available Not Available Not Available Not Available Not Available Not Available Not Available 50 Yes 6836 Not Available TUMOR FREE 101 109 Not Available Not Available 108 Not Available YES WHITE Not Available NA Not Available Not Applicable Not Applicable Not Applicable Not Applicable Not Applicable Not Applicable Not Applicable Not Applicable Not Applicable Stage IB Not Applicable Not Applicable Not Applicable 7th Not Applicable Not Applicable Not Applicable MX N0 T2 Not Available NO YES 91 2 Lung Alive 2011 2010 1973

4 Dead T2 N2 M0 Stage IIIA NA 87 R-Upper Not Applicable TCGA-NJ-A4YI 2C03141E-A0BD-4E8D-91E4-9744D139CCCE 24 -31867 4 0 Not Available Not Available Lung Adenocarcinoma 52 NA Not Evaluated Not Available Unknown Not Available Not Available Not Available Unknown Not Available Not Evaluated NA Not Available Not Available Not Available Not Available TCGA-NJ-A4YI-F50887 5768A19F-C788-44AE-9925-B7C2D4F5236D 1 Not Applicable Not Applicable 4 Not Available Not Applicable Not Available Scheduled Follow-up Submission Stable Disease Not Available NO 11 Not Available NO Not Available Unknown NO Unknown Not Available NO Dead 2013 FEMALE Lung Papillary Adenocarcinoma No C34.1 8550/3 C34.1 YES Not Evaluated Unknown Not Available Not Available Unknown 4 Not Available Not Available Not Available Not Available Not Available Not Available Not Available Not Available Not Available NO 3 No A4YI Not Evaluated Unknown Not Available 94 NO Not Available 89 Not Applicable YES WHITE NO NA Not Evaluated Not Applicable Not Applicable Not Applicable Not Applicable Not Applicable Not Applicable Not Applicable Not Applicable Not Applicable Stage IIIA Not Applicable Not Applicable Not Applicable 6th Not Applicable Not Applicable Not Applicable M0 N2 T2 1976 NO YES NJ 3 Lung Dead 2013 2009 1936

448 Alive T2b N2 Stage IIIA NA 70 L-Upper Not Applicable TCGA-38-6178 edf80622-aea2-4b50-bb69-691d6708aa83 14 -25593 Not Applicable 0 158 Not Available Lung Adenocarcinoma Not Available NA TCGA-38-6178-D22083;TCGA-38-6178-D22081 5ad35ad6-ca82-4ca6-9db3-cd262b61c75f;7068c8ff-4abb-437c-961f-cffe77e553b7 Not Available;Not Available 23;23 98;98 57;57 Carboplatin;Taxol Not Available;Not Available 2;2 3;3 6;175 AUC;mg/m2 ADJUVANT;ADJUVANT Not Applicable;Not Applicable 1;1 IV;IV NO;NO Chemotherapy;Chemotherapy Not Available;Not Available Not Available;Not Available Not Available;Not Available Not Available;Not Available 2012;2012 Not Available Not Available YES Exon 19 Deletion Not Available Not Available NO Not Available NOT HISPANIC OR LATINO NA Not Available Not Available Not Available Not Available TCGA-38-6178-F22078 e24c953c-286d-4f97-a3ac-9c2723bd1f76 23 Not Applicable Not Applicable Not Applicable 448 Not Applicable Not Available Scheduled Follow-up Submission Not Available Not Available Not Available 2 Not Available NO Not Available WITH TUMOR YES Stable Disease Not Available NO Alive 2012 FEMALE Lung Adenocarcinoma- Not Otherwise Specified (NOS) No C34.1 8140/3 C34.1 YES Not Available YES NO Not Available Central Lung 4 Not Available Not Available Not Available Not Available Not Available Not Available Not Available Not Available Not Available Not Available Not Available Yes 6178 Not Available WITH TUMOR Not Available Not Available Not Available Not Available Not Available Not Available Not Available WHITE Not Available NA R1 Not Applicable Not Applicable Not Applicable Not Applicable Not Applicable Not Applicable Not Applicable Not Applicable Not Applicable Stage IIIA Not Applicable Not Applicable Not Applicable Not Available Not Applicable Not Applicable Not Applicable Not Available N2 T2b Not Available YES NO 38 1 Lung Alive 2011 2010 Not Available

224 Alive T2 N2 MX Stage IIIA NA 59 L-Upper Not Applicable TCGA-91-6848 0232d299-4cdf-4fd7-9a5e-8d13c208b40c 19 -21829 Not Applicable 0 127 Not Available Lung Adenocarcinoma Not Available NA TCGA-91-6848-D32644;TCGA-91-6848-D33330 84DFEC23-536C-4115-8F5A-A423FC91FAE2;C0CD8924-B559-41B3-B3A3-53EFB06231E5 Not Available;Not Available 5;28 154;154 91;91 Cisplatin;Docetaxel Complete Response;Complete Response 6;6 Not Available;Not Available Not Available;Not Available Not Available;Not Available Not Available;Not Available Not Applicable;Not Applicable Not Available;Not Available Not Available;Not Available NO;NO Chemotherapy;Chemotherapy Not Available;Not Available Not Available;Not Available Not Available;Not Available Not Available;Not Available 2012;2012 Not Available Not Available Not Available Not Available Not Available Not Available Not Available Not Available NOT HISPANIC OR LATINO NA Not Available Not Available Not Available Not Available TCGA-91-6848-F32641 DA1E6F28-C0EE-4F97-B849-62A0E36951AB 5 Not Applicable Not Applicable Not Applicable 224 Not Applicable Unknown Scheduled Follow-up Submission Complete Remission/Response Unknown YES 6 Not Available NO Not Available TUMOR FREE YES Complete Remission/Response Not Available NO Alive 2012 MALE Lung Adenocarcinoma- Not Otherwise Specified (NOS) No C34.1 8140/3 C34.1 YES Not Available YES NO Not Available Not Available 7 Not Available Not Available Not Available Not Available Not Available Not Available Not Available Not Available Not Available Not Available Not Available No 6848 Not Available Not Available Not Available Not Available Not Available Not Available Not Available Not Available Not Available WHITE Not Available NA Not Available Not Applicable Not Applicable Not Applicable Not Applicable Not Applicable Not Applicable Not Applicable Not Applicable Not Applicable Stage IIIA Not Applicable Not Applicable Not Applicable 6th Not Applicable Not Applicable Not Applicable MX N2 T2 2007 NO YES 91 4 Lung Alive 2011 2009 Not Available

705 Alive T2a N0 MX Stage IB NA 76 L-Upper Not Applicable TCGA-55-7724 294cb595-0907-44c7-bbef-985a27c1e6e2 8 -27891 Not Applicable 0 0 Not Available Lung Adenocarcinoma 49 NA Not Available Not Available Not Available Not Available Not Available Not Available Not Available Not Available NOT HISPANIC OR LATINO NA Not Available Not Available Not Available Not Available TCGA-55-7724-F56186 519BD0D9-C612-41B2-A0ED-177FDA150DFD 29 Not Applicable Not Applicable Not Applicable 705 Not Applicable Unknown Scheduled Follow-up Submission Complete Remission/Response Unknown NO 1 Not Available NO Not Available TUMOR FREE NO Complete Remission/Response Not Available NO Alive 2014 FEMALE Lung Adenocarcinoma- Not Otherwise Specified (NOS) No C34.1 8140/3 C34.1 YES Not Available Not Available Not Available Not Available Not Available 3 Not Available Not Available Not Available Not Available Not Available Not Available Not Available Not Available Not Available Not Available 50 Yes 7724 Not Available TUMOR FREE Not Available Not Available Not Available 36 33 Not Available YES WHITE Not Available NA R0 Not Applicable Not Applicable Not Applicable Not Applicable Not Applicable Not Applicable Not Applicable Not Applicable Not Applicable Stage IB Not Applicable Not Applicable Not Applicable 7th Not Applicable Not Applicable Not Applicable MX N0 T2a 2008 YES NO 55 4 Lung Alive 2012 2011 1958

176 Dead T2b N0 MX Stage IIA NA 81 R-Middle Not Applicable TCGA-55-6978 5134c56f-8286-4ec8-8348-237cee7dad5e 27 Not Available 176 0 Not Available Not Available Lung Adenocarcinoma Not Available NA Not Available Not Available NO Not Available Not Available Not Available NO Not Available NOT HISPANIC OR LATINO NA NO YES NO NO TCGA-55-6978-F45607 503F8882-F797-4D4A-86CC-4FD93814BA3C 17 Not Available Not Available 176 Not Available 42 Not Evaluated Scheduled Follow-up Submission Progressive Disease Not Evaluated NO 7 Not Available YES Not Evaluated WITH TUMOR NO Progressive Disease Not Available NO Dead 2013 MALE Lung Adenocarcinoma- Not Otherwise Specified (NOS) No C34.2 8140/3 C34.2 YES Not Available NO Not Available Not Available Not Available 7 Not Available Not Available Not Available Not Available Not Available Not Available Not Available Not Available Not Available Not Available Not Available Yes 6978 Not Available WITH TUMOR Not Available Not Available Not Available Not Available Not Available Not Available Not Available WHITE Not Available NA Distant site TCGA-55-6978-R45613 4BF13E15-8053-43E3-8D6E-D647C58C72E3 Not Available 17 95 60 Radiographic Progressive Disease 7 11 3200 NO External Not Applicable Not Available Not Available cGy 2013 Not Available Not Applicable Not Applicable Not Applicable Not Applicable Not Applicable Not Applicable Not Applicable Not Applicable Not Applicable Stage IIA Not Applicable Not Applicable Not Applicable 7th Not Applicable Not Applicable Not Applicable MX N0 T2b Not Available NO YES 55 1 Lung Dead 2011 2010 Not Available

416 Alive T3 N0 MX Stage IIB NA 72 R-Lower Not Applicable TCGA-55-8619 772324a5-5513-454d-ad6b-605798f69b73 11 -26616 Not Applicable 0 49 Not Available Lung Adenocarcinoma Not Available NA Not Evaluated Not Available Unknown Not Available Not Available Not Available Unknown Not Available NOT HISPANIC OR LATINO NA Not Available Not Available Not Available Not Available TCGA-55-8619-F57919 127448B8-A332-41F4-BDDF-9AAC9647E785 28 Not Applicable Not Applicable Not Applicable 416 Not Applicable Not Evaluated Scheduled Follow-up Submission Complete Remission/Response Not Evaluated NO 3 Not Available NO Not Available TUMOR FREE NO Complete Remission/Response Not Available NO Alive 2014 FEMALE Mucinous (Colloid) Carcinoma No C34.30 8480/3 C34.3 YES 90 Unknown Not Available Not Available Unknown 1 Not Available Not Available Not Available Not Available Not Available Not Available Not Available Not Available Not Available NO Not Available Yes, History of Prior Malignancy 8619 Preoperative TUMOR FREE Not Available Not Available NO Not Available Not Available Complete Remission/Response Not Available WHITE NO NA R0 Not Applicable Not Applicable Not Applicable Not Applicable Not Applicable Not Applicable Not Applicable Not Applicable Not Applicable Stage IIB Not Applicable Not Applicable Not Applicable 7th Not Applicable Not Applicable Not Applicable MX N0 T3 Not Available YES NO 55 1 Lung Alive 2013 2012 Not Available

600 Alive T2 N0 MX Stage IB NA 74 R-Lower Not Applicable TCGA-55-8091 d12b6930-9ae4-4fe7-ad69-d183135c91ab 15 -27385 Not Applicable 0 43 Not Available Lung Adenocarcinoma 65 NA Not Available Not Available NO Not Available Not Available Not Available NO Not Available NOT HISPANIC OR LATINO NA Not Available Not Available Not Available Not Available TCGA-55-8091-F47820 B40ED298-8EB6-4147-BE8D-F6338BF3163B 28 Not Applicable Not Applicable Not Applicable 600 Not Applicable Not Evaluated Scheduled Follow-up Submission Complete Remission/Response Not Evaluated NO 8 Not Available NO Not Evaluated TUMOR FREE NO Complete Remission/Response Not Available NO Alive 2013 MALE Lung Adenocarcinoma- Not Otherwise Specified (NOS) No C34.3 8140/3 C34.3 YES Not Available NO Not Available Not Available Unknown 6 Not Available Not Available Not Available Not Available Not Available Not Available Not Available Not Available Not Available NO Not Available Yes, History of Prior Malignancy 8091 Not Available TUMOR FREE Not Available Not Available Unknown 79 82 Complete Remission/Response YES WHITE Unknown NA R0 Not Applicable Not Applicable Not Applicable Not Applicable Not Applicable Not Applicable Not Applicable Not Applicable Not Applicable Stage IB Not Applicable Not Applicable Not Applicable 7th Not Applicable Not Applicable Not Applicable MX N0 T2 1985 YES NO 55 3 Lung Alive 2012 2011 Not Available

218 Alive T2a N0 MX Stage IB NA 61 L-Upper Not Applicable TCGA-44-7662 ab9e6815-4d13-44db-93e5-553eb4f5bc95 21 -22543 Not Applicable 0 50 Not Available Lung Adenocarcinoma 57 NA Not Available Not Available Not Available Not Available Not Available Not Available Not Available Not Available NOT HISPANIC OR LATINO NA Not Available Not Available Not Available Not Available TCGA-44-7662-F34016 58426E9F-D0BD-4163-9767-80A6768F6B3F 25 Not Applicable Not Applicable Not Applicable 218 Not Applicable Not Available Scheduled Follow-up Submission Complete Remission/Response Not Available NO 7 Not Available NO Not Available TUMOR FREE NO Complete Remission/Response Not Available NO Alive 2012 MALE Lung Adenocarcinoma- Not Otherwise Specified (NOS) No C34.1 8140/3 C34.1 YES Not Available NO Not Available Not Available Not Available 12 Not Available Not Available Not Available Not Available Not Available Not Available Not Available Not Available Not Available Not Available 50 No 7662 Not Available TUMOR FREE 66 68 Not Available 68 60 Not Available YES WHITE Not Available NA Not Available Not Applicable Not Applicable Not Applicable Not Applicable Not Applicable Not Applicable Not Applicable Not Applicable Not Applicable Stage IB Not Applicable Not Applicable Not Applicable 7th Not Applicable Not Applicable Not Applicable MX N0 T2a Not Available YES NO 44 2 Lung Alive 2011 2011 1961

1893 Alive T2 N1 MX Stage IIB NA 69 R-Upper Not Applicable TCGA-49-AAR3 5813997A-D7DC-4F7C-8D4B-E979832168A9 18 -25469 Not Applicable 0 1893 Not Available Lung Adenocarcinoma Not Available NA 1 Not Available Not Available Not Available Not Available Not Available Not Available Not Available NOT HISPANIC OR LATINO NA Not Available Not Available Not Available Not Available TCGA-49-AAR3-F70567 9B56C5FC-1A78-41F4-8707-440A0C3F8FFF 25 Not Applicable Not Applicable Not Applicable 1893 Not Applicable 0 Scheduled Follow-up Submission Complete Remission/Response 100 NO 2 Not Available NO Post-Adjuvant Therapy Discrepancy YES Complete Remission/Response Not Available NO Alive 2015 MALE Lung Adenocarcinoma- Not Otherwise Specified (NOS) No C34.1 8140/3 C34.1 YES 90 Not Available Not Available Not Available Not Available 6 NO NO 1893 NO Not Available Not Available Not Available Locoregional Recurrence Convincing Imaging YES Not Available No AAR3 Preoperative Discrepancy Not Available Not Available NO Not Available Not Available Complete Remission/Response NO BLACK OR AFRICAN AMERICAN NO NA R0 Not Applicable Not Applicable Not Applicable Not Applicable Not Applicable Not Applicable Not Applicable Not Applicable Not Applicable Stage IIB Not Applicable Not Applicable Not Applicable 6th Not Applicable Not Applicable Not Applicable MX N1 T2 Not Available NO YES 49 Unknown Lung Alive 2014 2008 Not Available

995 Dead T2 N1 M0 Stage IIB NA 79 R-Lower Not Applicable TCGA-55-6982 35cb7841-9b09-465a-90c5-e3b8a9faad49 26 Not Available 995 0 Not Available Not Available Lung Adenocarcinoma Not Available NA TCGA-55-6982-D46628;TCGA-55-6982-D46629;TCGA-55-6982-D46637 51A05329-99B3-4DE3-BEEC-F7B7584AEE8C;F2442589-3EA6-4F5B-AA58-D61A68517EBF;8D09A550-760E-4CF9-8FC7-95C7C2613527 Not Available;Not Available;Not Available 13;13;13 Not Available;Not Available;939 397;397;698 Gemzar;Navelbine;Tarceva Clinical Progressive Disease;Clinical Progressive Disease;Clinical Progressive Disease 8;8;8 Not Available;Not Available;Not Available Not Available;Not Available;Not Available Not Available;Not Available;Not Available Not Available;Not Available;Not Available Not Applicable;Not Applicable;Not Applicable Not Available;Not Available;Not Available Not Available;Not Available;Not Available NO;NO;NO Chemotherapy;Chemotherapy;Chemotherapy Not Available;Not Available;Not Available Not Available;Not Available;Not Available Not Available;Not Available;Not Available NO;NO;NO 2013;2013;2013 Not Available Not Available NO Not Available Not Available Not Available NO Not Available Not Available NA YES YES NO YES TCGA-55-6982-F46626 482B5DF1-D91C-483A-ACE3-C2C65F5FA3DD 13 Not Available 183 995 Not Available 183 Not Evaluated Scheduled Follow-up Submission Progressive Disease Not Evaluated NO 8 Distant Metastasis YES Not Evaluated WITH TUMOR NO Progressive Disease Convincing Imaging NO Dead 2013 FEMALE Lung Adenocarcinoma- Not Otherwise Specified (NOS) No C34.3 8140/3 C34.3 YES Not Available NO Not Available Not Available Not Available 7 Not Available Not Available Not Available Not Available Not Available Not Available Not Available Not Available Not Available Not Available Not Available No 6982 Not Available WITH TUMOR Not Available Not Available Not Available Not Available Not Available Not Available Not Available WHITE Not Available NA Distant Recurrence TCGA-55-6982-R46627 BC037851-C729-42AC-B820-0FCC9D22FA88 Not Available 13 687 642 Radiographic Progressive Disease 8 25 03500 NO External Not Applicable Not Available Not Available cGy 2013 Not Available Not Applicable Not Applicable Not Applicable Not Applicable Not Applicable Not Applicable Not Applicable Not Applicable Not Applicable Stage IIB Not Applicable Not Applicable Not Applicable 6th Not Applicable Not Applicable Not Applicable M0 N1 T2 Not Available NO YES 55 1 Lung Dead 2011 2004 Not Available

468 Dead TX NX MX Stage IV NA 49 R-Upper Not Applicable TCGA-55-7816 548b0b68-ff45-4d45-adbe-984259e4a68b 9 -18170 Not Applicable 0 44 Not Available Lung Adenocarcinoma Not Available NA Not Available Not Available Not Available Not Available Not Available Not Available Not Available Not Available NOT HISPANIC OR LATINO NA Not Available Not Available Not Available Not Available TCGA-55-7816-F60131 6AFEC225-5C90-4B90-8F48-FB7786DF92B3 29 Not Applicable Not Applicable 468 Not Available Not Applicable Not Evaluated Scheduled Follow-up Submission Progressive Disease Not Evaluated NO 5 Not Available NO Not Evaluated WITH TUMOR NO Progressive Disease Not Available NO Dead 2014 FEMALE Lung Adenocarcinoma- Not Otherwise Specified (NOS) No C34.1 8140/3 C34.1 YES Not Available Not Available Not Available Not Available Not Available 3 Not Available Not Available Not Available Not Available Not Available Not Available Not Available Not Available Not Available Not Available Not Available No 7816 Not Available WITH TUMOR Not Available Not Available Not Available Not Available Not Available Not Available NO WHITE Not Available NA Not Available Not Applicable Not Applicable Not Applicable Not Applicable Not Applicable Not Applicable Not Applicable Not Applicable Not Applicable Stage IV Not Applicable Not Applicable Not Applicable 7th Not Applicable Not Applicable Not Applicable MX NX TX Not Available YES NO 55 1 Lung Alive 2012 2011 Not Available

582 Dead T2 N1 MX Stage IIB NA 56 R-Lower Not Applicable TCGA-MP-A4TK F7F9F522-DE88-4F9C-8BA4-7BE3726FAAF8 2 -20581 582 0 Not Available Not Available Lung Adenocarcinoma 88 NA Not Evaluated Not Available NO Not Available Not Available Not Available NO Not Available Unknown NA YES YES Not Available NO TCGA-MP-A4TK-F41426 445D9B7D-F0FC-4879-AC28-B16DC3E929A5 18 Not Available Not Available 582 Not Available 545 Not Evaluated Additional New Tumor Event Progressive Disease Not Evaluated NO 3 Distant Metastasis YES Not Evaluated WITH TUMOR NO Complete Remission/Response Convincing Imaging NO Dead 2013 FEMALE Lung Adenocarcinoma- Not Otherwise Specified (NOS) No C34.3 8140/3 C34.3 YES Not Evaluated NO Not Available Not Available Unknown 4 NO YES 397 Not Available Not Available NO Not Available Distant Metastasis Biopsy with Histologic Confirmation YES 28 No A4TK Not Evaluated WITH TUMOR Not Available 64 NO Not Available 57 Complete Remission/Response YES Unknown NO NA R0 Not Applicable Not Applicable Not Applicable Not Applicable Not Applicable Not Applicable Not Applicable Not Applicable Not Applicable Stage IIB Not Applicable Not Applicable Not Applicable 5th Not Applicable Not Applicable Not Applicable MX N1 T2 1999 NO YES MP 4 Lung Dead 2013 2002 1971

2681 Dead T2 N0 M1 Stage IV NA 77 Not Available Not Applicable TCGA-78-7167 fe3eeeb6-0db4-46d7-a020-55604d474c12 4 -28295 2681 0 Not Available Not Available Lung Adenocarcinoma Not Available NA 1 Not Available NO Not Available Not Available Not Available NO Not Available Not Available NA NO NO NO NO TCGA-78-7167-F17172 c8c4cb37-14b3-47ab-83a8-795fe5195bcb 4 Not Available Not Available 2681 Not Available 726 Not Available Scheduled Follow-up Submission Progressive Disease Not Available Not Available 10 Not Available YES Not Available WITH TUMOR NO Not Available Not Available NO Dead 2011 MALE Lung Papillary Adenocarcinoma No C34.9 8260/3 C34.9 YES Not Available NO Not Available Not Available Peripheral Lung 10 Not Available Not Available Not Available Not Available Not Available Not Available Not Available Not Available Not Available Not Available 64 No 7167 Preoperative WITH TUMOR Not Available Not Available Not Available Not Available Not Available Not Available Not Available WHITE Not Available NA R0 Not Applicable Not Applicable Not Applicable Not Applicable Not Applicable Not Applicable Not Applicable Not Applicable Not Applicable Stage IV Not Applicable Not Applicable Not Applicable 6th Not Applicable Not Applicable Not Applicable M1 N0 T2 Not Available NO YES 78 2 Lung Dead 2011 2002 1937

184 Alive T2a N2 MX Stage IIIA NA 54 L-Upper Not Applicable TCGA-69-7974 3e1df30f-744a-41e9-a169-7dbbff66622e 9 -20021 Not Applicable 0 184 Not Available Lung Adenocarcinoma Not Available NA TCGA-69-7974-D30366;TCGA-69-7974-D30368;TCGA-69-7974-D30370 FF1F92D3-3742-477D-8C65-C4AC2811A0C2;E368B4BF-E5C5-4E85-BDA5-8A7A50246BA7;D76675FA-0F02-4B08-91C1-D014BBC62EF2 Not Available;Not Available;Not Available 9;9;9 54;96;96 33;33;75 Cisplatin;Pemetrexed;Carboplatin Unknown;Clinical Progressive Disease;Clinical Progressive Disease 4;4;4 Not Available;Not Available;Not Available Not Available;Not Available;Not Available Not Available;Not Available;Not Available Not Available;Not Available;Not Available Not Applicable;Not Applicable;Not Applicable Not Available;Not Available;Not Available Not Available;Not Available;Not Available NO;NO;NO Chemotherapy;Chemotherapy;Chemotherapy Not Available;Not Available;Not Available Not Available;Not Available;Not Available Not Available;Not Available;Not Available NO;NO;NO 2012;2012;2012 Not Available Not Available YES Other Not Available Not Available Not Available Not Available NOT HISPANIC OR LATINO NA Not Available Not Available Not Available Not Available TCGA-69-7974-F30365 21EB1C00-AEF8-466D-B783-A523818D00B2 9 Not Applicable Not Applicable Not Applicable 184 Not Applicable Not Available Scheduled Follow-up Submission Progressive Disease 80 Not Available 4 Not Available NO Post-Adjuvant Therapy WITH TUMOR YES Progressive Disease Not Available YES Alive 2012 FEMALE Lung Adenocarcinoma Mixed Subtype No C34.1 8255/3 C34.1 YES 80 Not Available Not Available Not Available Peripheral Lung 4 Not Available Not Available Not Available Not Available Not Available Not Available Not Available Not Available Not Available Not Available 30 No 7974 Post-Adjuvant Therapy WITH TUMOR Not Available Not Available Not Available 58 47 Not Available YES WHITE Not Available NA Regional site TCGA-69-7974-R30373 5051D4B2-CA36-4831-B8B9-975261929A95 1 9 Not Available 141 Not Available 4 30 6000 YES EXTERNAL BEAM Not Applicable ADJUVANT Not Available cGy 2012 Not Available Not Applicable Not Applicable Not Applicable Not Applicable Not Applicable Not Applicable Not Applicable Not Applicable Not Applicable Stage IIIA Not Applicable Not Applicable Not Applicable 7th Not Applicable Not Applicable Not Applicable MX N2 T2a 2011 NO YES 69 4 Lung Alive 2012 2011 1981

2696 Alive T2 N2 M0 Stage IIIA NA 62 L-Upper Not Applicable TCGA-62-8399 62380447-3b94-4e7c-823a-72dd7b4ccf3a 4 -22952 Not Applicable 0 2093 Not Available Lung Adenocarcinoma Not Available NA Unknown Not Available NO Not Available Not Available Not Available NO Not Available NOT HISPANIC OR LATINO NA Not Available Not Available Not Available Not Available TCGA-62-8399-F52545 147B5527-FA29-41B2-92BD-C0882730DDC1 4 Not Applicable Not Applicable Not Applicable 2696 Not Applicable Unknown Scheduled Follow-up Submission Complete Remission/Response 100 NO 12 Not Available NO Preoperative TUMOR FREE NO Complete Remission/Response Not Available NO Alive 2013 MALE Lung Papillary Adenocarcinoma No C34.1 8255/3 C34.1 YES 100 NO Not Available Not Available Peripheral Lung 7 Not Available Not Available Not Available Not Available Not Available Not Available Not Available Not Available Not Available NO 70 No 8399 Preoperative TUMOR FREE Not Available Not Available NO 48 32 Complete Remission/Response YES WHITE NO NA R0 Not Applicable Not Applicable Not Applicable Not Applicable Not Applicable Not Applicable Not Applicable Not Applicable Not Applicable Stage IIIA Not Applicable Not Applicable Not Applicable 6th Not Applicable Not Applicable Not Applicable M0 N2 T2 Not Available NO YES 62 4 Lung Alive 2012 2006 Not Available
[truncated: 40,853 more chars]
