# Supplementary material for: An Immune-Related Signature Predicts Survival in Patients With Lung Adenocarcinoma
Source: Front Oncol. 2019 Dec 10;9:1314. doi: 10.3389/fonc.2019.01314 (PMC6914845; doi:10.3389/fonc.2019.01314)
Supplement: Supplementary file 7 [file Table_7.doc]

**Table S7. Univariate Cox proportional hazards regression analysis of each immune-related gene.**

p.value HR Low 95%CI High 95%CI

ENSG00000160862.11 0.158104836375161 0.999451774010464 0.998691009331886 1.00021311821053

ENSG00000166710.16 0.530797677085043 0.999958167718548 0.999827369775071 1.00008898277308

ENSG00000179218.12 0.858545775843345 0.99996591833297 0.999591195673365 1.00034078146707

ENSG00000127022.13 0.692733655583128 0.999779224877543 0.998684883853233 1.00087476506116

ENSG00000158477.6 0.509370958540002 0.998120699148773 0.992559105321494 1.0037134561841

ENSG00000158485.9 0.338534655019285 0.971147911275489 0.914633754869655 1.0311540117051

ENSG00000158481.11 0.324693859559309 0.994104273206273 0.982474416801438 1.00587179585227

ENSG00000158473.6 0.0960819477081079 0.951648077626606 0.897690888789796 1.00884845213404

ENSG00000158488.14 0.329245998343371 0.988494037905127 0.965778121725153 1.01174425159743

ENSG00000010610.8 0.263138608244006 0.997633907299157 0.99350311656148 1.00178187305303

ENSG00000153563.14 0.700926944199444 0.997596580076381 0.985421228229802 1.00992236423388

ENSG00000172116.20 0.145666575609415 0.97894615943959 0.951284676705845 1.0074119835506

ENSG00000019582.13 0.0502692377249596 0.999920378345482 0.999840669571664 1.0000000934738

ENSG00000118260.13 0.937651796797888 0.998416202197464 0.959540223388684 1.03886724966048

ENSG00000164733.19 0.209075140578214 1.00030948214031 0.999826626337751 1.00079257113302

ENSG00000196188.9 0.374412059404222 1.00012152430407 0.999853391822758 1.00038972869096

ENSG00000135047.13 0.0024537407248344 1.00090199002553 1.00031824754762 1.00148607315031

ENSG00000163131.9 0.595689229343237 0.999534979266302 0.997819161574857 1.00125374742258

ENSG00000158869.9 0.666373288552357 0.999626746357236 0.997931653183358 1.00132471882736

ENSG00000104870.11 0.0742867874464721 0.996934593931875 0.993579247427282 1.00030127154085

ENSG00000167004.11 0.90068692038607 1.00004060347456 0.999403090277759 1.00067852333718

ENSG00000010704.17 0.556015464540851 1.00785659890119 0.981939262465807 1.03445799834698

ENSG00000206503.10 0.482436768088913 1.00005654494133 0.999898768437695 1.00021434634091

ENSG00000234745.8 0.952739249845994 0.999996331595565 0.99987502527755 1.00011765263064

ENSG00000204525.13 0.851265467736656 1.00001782448038 0.999831522365004 1.00020416131008

ENSG00000204257.13 0.075621219792148 0.99852377660026 0.996897790773316 1.00015241448435

ENSG00000242574.7 0.0287816957778789 0.994893941035995 0.990338992375784 0.999469839651177

ENSG00000204252.11 0.0372795488720987 0.994454947419655 0.989264731056545 0.999672394457276

ENSG00000241106.5 0.0716295599284067 0.982734988959733 0.964289349089257 1.00153347067229

ENSG00000231389.6 0.0409834899009704 0.998910643859931 0.997867034884346 0.999955344283231

ENSG00000223865.9 0.0661245992034677 0.999395580539866 0.998751308319048 1.00004026836634

ENSG00000196735.10 0.0446604127728323 0.99772819069758 0.995515528512752 0.999945770810662

ENSG00000237541.3 0.104579268032908 0.998684181297581 0.997097528976515 1.00027335841238

ENSG00000237541.3.1 0.104579268032908 0.998684181297581 0.997097528976515 1.00027335841238

ENSG00000179344.15 0.0509498139744875 0.998824280239337 0.997645095404021 1.0000048588337

ENSG00000204287.12 0.0217081819145832 0.999906389784432 0.99982646737519 0.999986318582374

ENSG00000196126.9 0.128855571581363 0.999885699702743 0.999738201569229 1.00003321959765

ENSG00000198502.5 0.0209349724200496 0.999640290042612 0.999335077062537 0.999945596239633

ENSG00000204592.8 0.628063810821897 0.999892160233917 0.999455989563019 1.00032852125322

ENSG00000204642.12 0.62136361214842 1.00075367279841 0.997766295698971 1.00374999429893

ENSG00000204632.10 0.381165838021471 0.996175452476082 0.987668879183532 1.00475529100024

ENSG00000153029.13 0.347919489802815 0.990355364358028 0.970515201287755 1.01060111826308

ENSG00000204389.9 0.0214930707162317 1.00155988858892 1.00023000649901 1.00289153885851

ENSG00000204388.6 0.12644324566365 1.00099687037303 0.999718599212123 1.00227677597103

ENSG00000204390.9 0.700395078156329 0.971917445890132 0.840655376392172 1.12367510891279

ENSG00000126803.9 0.0134600872511205 1.02346319140721 1.00481065229793 1.04246198203605

ENSG00000170606.12 0.0119047198358712 1.00725859490739 1.00159729141765 1.01295189764222

ENSG00000044574.7 0.800748174997406 0.999924697655043 0.999340095868849 1.00050964142616

ENSG00000173110.7 0.464011719020113 1.0012090153414 0.99797623175727 1.00445227100829

ENSG00000109971.12 0.410364989367025 1.00025930741531 0.999642069257897 1.00087692669209

ENSG00000080824.17 0.742450802074457 1.00011040524725 0.99945208293502 1.00076916118533

ENSG00000096384.18 0.613272024826686 1.00006137888142 0.999823373786423 1.00029944063285

ENSG00000090339.7 0.961599218934255 0.999980058939456 0.999168634676419 1.00079214215966

ENSG00000226979.7 0.185546181013638 0.925534028494126 0.825347763457583 1.03788157650298

ENSG00000179583.16 0.114859756435671 0.9790966620381 0.953720619458457 1.00514789557395

ENSG00000204516.8 0.225475148134346 1.01333468606621 0.991860151415947 1.03527416089759

ENSG00000001167.13 0.914695080392351 0.999655930053702 0.99338127855885 1.00597021512353

ENSG00000120837.6 0.377503518568093 1.01415593286899 0.982984513808696 1.04631582870851

ENSG00000066136.18 0.390173704541215 0.991426980706784 0.972146961157709 1.01108936955666

ENSG00000100600.13 0.909004676222544 0.999865550260948 0.997562759209868 1.00217365711454

ENSG00000204264.7 0.167258758893672 1.00101711353313 0.99957396620415 1.00246234442399

ENSG00000100764.12 0.474912101686281 1.00565002169727 0.99022728971099 1.02131296183009

ENSG00000161057.9 0.299601551758261 1.00283474622465 0.997485206300995 1.00821297587443

ENSG00000165916.7 0.0449460934246644 1.00361118641121 1.00008124986005 1.0071535824021

ENSG00000013275.6 0.0363379039372715 1.00317533013249 1.00020171082951 1.00615779006398

ENSG00000087191.11 1.12502943133697e-05 1.01369117127746 1.00755785923769 1.01986181865855

ENSG00000100519.10 0.130270116958412 1.01087086225276 0.99681134872094 1.02512867802201

ENSG00000173692.11 0.11110836858782 1.00227934478335 0.999476070442072 1.00509048159103

ENSG00000175166.15 0.0011976026933086 1.00428801857871 1.00169144771414 1.00689132023875

ENSG00000108344.13 0.0351981539607465 1.00488602433132 1.00033819663713 1.00945452776977

ENSG00000159352.14 0.100568669981292 1.00237725562841 0.999540487545246 1.00522207466424

ENSG00000095261.12 0.601939024201889 0.995174227839147 0.977248629998528 1.01342863356763

ENSG00000103035.9 0.0842034524791123 1.00535120006835 0.999279634130589 1.0114596564937

ENSG00000099341.10 0.255365746642932 1.00162058083825 0.998829666685413 1.00441929331954

ENSG00000101843.17 0.704177192411198 0.998517018261095 0.990896710873126 1.0061959282098

ENSG00000108671.8 4.46482815337212e-05 1.01101662572215 1.00571196047548 1.01634927062352

ENSG00000185627.16 0.276271068559061 1.0030492690836 0.997566584573846 1.00856208674927

ENSG00000092010.13 0.445111751175208 0.999430557237114 0.99797040904241 1.00089284180052

ENSG00000100911.12 0.206838913317193 1.00191777457953 0.998941648253834 1.00490276761723

ENSG00000104856.12 0.0118890843631901 1.01395753723563 1.00306548413779 1.02496786458631

ENSG00000143390.16 0.194367416169923 0.992344518518113 0.980893708178342 1.00392900395065

ENSG00000133111.3 0.00933407131340958 0.897222573160575 0.826778087157965 0.973669184383061

ENSG00000168394.10 0.174873428679563 1.00098490648086 0.999562377623769 1.00240945981227

ENSG00000204267.12 0.00526032070039584 1.01562676204568 1.0046262569617 1.02674772099124

ENSG00000231925.10 0.189893812763407 1.00131488898738 0.999349590177093 1.00328405271086

ENSG00000137801.10 0.223045186672119 1.00070253755771 0.999572716174175 1.00183363598326

ENSG00000127922.8 0.036829613250203 1.01748808235699 1.00106091625505 1.03418481425834

ENSG00000132842.12 0.368845278015694 1.00893790120026 0.98954592762681 1.02870989618412

ENSG00000064490.12 0.344116620407668 0.995514757416471 0.98628655990951 1.00482929862179

ENSG00000163636.9 0.79104866378061 0.995033355244991 0.959050667817541 1.03236607957659

ENSG00000131467.9 0.00612503923280527 1.01030402541075 1.00292536442767 1.01773697222588

ENSG00000115233.10 0.00763090596117577 1.01758241585962 1.00463610249734 1.03069556279403

ENSG00000216490.3 0.438802541063683 1.03923081715046 0.942767958897769 1.14556363644124

ENSG00000101000.4 0.000312434020541041 1.00518411345562 1.00236211051391 1.00801406133113

ENSG00000130706.11 0.0201717990815237 1.00359846701413 1.00056169155125 1.00664445930521

ENSG00000136813.13 0.627983745259032 1.00288911168551 0.991254450209989 1.01466033279778

ENSG00000100991.10 0.16102258318042 1.00340314860809 0.998647621540614 1.00818132133876

ENSG00000090659.16 0.878162630934834 1.00268942985923 0.968842651911723 1.03771865407411

ENSG00000162191.12 0.171355555596868 1.00367745583058 0.99841232970336 1.00897034759362

ENSG00000164307.11 0.366892768807921 0.994584654440322 0.982922451122371 1.00638522776506

ENSG00000139192.10 0.522498031655418 1.00305260561865 0.993726220197613 1.0124665216525

ENSG00000164308.15 0.0270355985469685 1.00847788058406 1.00095898882831 1.01605325190977

ENSG00000131019.9 0.550096378152165 1.02080032826644 0.954153391755488 1.09210250594163

ENSG00000131015.4 0.155984519273873 1.02120594191727 0.992026174955268 1.05124401163524

ENSG00000159459.10 0.644840542561579 1.00716405315088 0.977052323961003 1.03820379429321

ENSG00000105697.6 0.66611808512205 1.02214471850154 0.925344163098279 1.12907161165033

ENSG00000124102.4 0.486667400081752 1.0000319936812 0.999941851476171 1.00012214401233

ENSG00000163736.3 0.994128921322694 0.999969422403083 0.991858025723462 1.00814715393546

ENSG00000145824.11 0.91328629273911 0.999979872993515 0.999617680937047 1.00034219628324

ENSG00000161921.13 0.893301822292339 0.999838934769588 0.9974882935821 1.00219511538448

ENSG00000124107.5 0.995322469592574 0.999999950069089 0.999983257049496 1.00001664336734

ENSG00000169429.9 0.701597730496829 1.00031567682412 0.998701974512664 1.00193198655522

ENSG00000169245.5 0.617673377951857 0.999619406165669 0.998126367177102 1.00111467850414

ENSG00000138755.5 0.734616173621985 0.999803303619507 0.99866681248955 1.00094108808581

ENSG00000163735.6 0.0548653081980868 1.00351383555626 0.999926882493727 1.00711365579188

ENSG00000169248.11 0.753467733085478 0.998908639895711 0.992124833695631 1.00573883141445

ENSG00000124875.8 0.407756677128412 0.995109392776325 0.983625812332378 1.00672704109259

ENSG00000163739.4 0.786636495666501 0.999772717855258 0.998128538691672 1.00141960541262

ENSG00000107562.15 0.608626746340807 0.996794060805876 0.984617062906715 1.0091216545899

ENSG00000156234.7 0.565181041717331 0.999419038195253 0.997441847471241 1.00140014822861

ENSG00000081041.8 0.919034053020099 0.999890967984572 0.997791001982798 1.00199535360649

ENSG00000143184.4 0.103633829122374 0.945134737711633 0.883041448685303 1.01159427313313

ENSG00000163734.4 0.465603693413538 1.00531504393663 0.991101507522505 1.01973241882326

ENSG00000181374.6 0.713261730458133 0.999199901837977 0.994943346382902 1.00347466764082

ENSG00000164825.3 0.535297476348856 1.00030448573689 0.999342158663865 1.00126773949292

ENSG00000108700.4 0.913248255967059 1.00080076591661 0.986491820813631 1.01531726054573

ENSG00000034510.5 0.0090819895010259 1.00003719533974 1.0000092523747 1.00006513908557

ENSG00000148346.10 0.256021077853977 1.00022264317508 0.999838491258639 1.00060694268805

ENSG00000163220.10 0.746332948067513 1.00000392419928 0.999980147611917 1.00002770135198

ENSG00000143546.8 0.535385213518272 1.00002785151004 0.999939781578063 1.0001159291988

ENSG00000163221.8 0.374599286845582 1.00229096705452 0.997240778746833 1.00736673033115

ENSG00000262406.2 0.754734991597148 1.00031861683562 0.998321340382107 1.00231988910998

ENSG00000164406.7 0.396411040646435 0.895365771681011 0.693532127479946 1.1559376030797

ENSG00000133661.14 0.443114822324464 0.999880391846336 0.999574792175145 1.00018608494841

ENSG00000107317.10 0.324313044277019 0.998754889628905 0.996283551925366 1.0012323576254

ENSG00000205542.9 0.429356027500804 0.999961243439854 0.999865131955749 1.00005736416262

ENSG00000105939.11 0.221513545410477 1.01377961370373 0.99177852443317 1.03626876348091

ENSG00000158164.6 0.387184630051007 0.991011234107335 0.970935713857663 1.01150184518902

ENSG00000160307.8 0.371967107849495 0.997337904799738 0.991518601694192 1.00319136186727

ENSG00000189171.12 0.425674623576185 0.998546699219331 0.994980033092808 1.00212615063484

ENSG00000197956.8 0.0336967494173844 1.00004367939901 1.00000336724498 1.0000839931781

ENSG00000099937.9 0.405690946433824 0.998538780063385 0.995102920987476 1.00198650236202

ENSG00000163993.6 0.0179184453005019 1.00017324689063 1.00002980801266 1.00031670634269

ENSG00000197747.7 4.95999822214266e-07 1.00068255979112 1.00041642812531 1.00094876225351

ENSG00000196754.9 0.051668648300916 1.00030500167076 0.999997793450043 1.00061230426858

ENSG00000184925.10 0.516941517880794 1.01980878858055 0.96107062468944 1.08213687792424

ENSG00000163191.5 7.34202854952848e-05 1.00026718333986 1.0001350987142 1.00039928540951

ENSG00000196420.6 0.562113282059387 0.99146135350887 0.963128793262087 1.02062737857962

ENSG00000188015.8 0.666324207843384 1.00515460453917 0.98193698759844 1.02892119533791

ENSG00000160678.10 0.283835942681961 1.01576892382703 0.98711694671746 1.04525255091993

ENSG00000139636.14 0.821260310472532 1.00362678543451 0.972596132008623 1.03564747102313

ENSG00000158270.11 0.105748299834984 0.987973770480878 0.973595840091343 1.00256403218262

ENSG00000158164.6.1 0.387184630051007 0.991011234107335 0.970935713857663 1.01150184518902

ENSG00000088888.16 0.502246664869555 1.00552513522535 0.989470494222552 1.0218402706029

ENSG00000189334.7 0.007036025790362 1.00065363835216 1.00017825507413 1.00112924757917

ENSG00000188643.9 7.46695144293597e-06 1.00117847630544 1.00066272289277 1.00169449554352

ENSG00000146858.7 0.438604359155367 1.01069014204703 0.983857826799704 1.03825424304828

ENSG00000142166.11 0.105535382486958 0.990335805009815 0.978758291715156 1.0020502661242

ENSG00000142910.14 0.481457511187805 1.00235930213468 0.995804325013654 1.00895742801895

ENSG00000027697.11 0.178435945966762 0.9981672003491 0.995503575965741 1.00083795167306

ENSG00000092096.13 0.0450390713727069 0.982176471903097 0.96505437393646 0.999602351963984

ENSG00000101443.16 0.322629718567392 0.999872079673518 0.999618637704504 1.00012558589987

ENSG00000136244.10 0.378500175554389 0.995757117818437 0.986378854634316 1.00522454737097

ENSG00000105329.8 0.117362953587666 1.00378930878708 0.999048779801955 1.00855233178402

ENSG00000100985.7 0.154291274257347 1.00090605063503 0.999659663016776 1.00215399226427

ENSG00000011201.9 0.0355956270052948 0.982452165239934 0.966363930892488 0.998808239969399

ENSG00000136869.13 0.0863712543970049 0.977316726229661 0.952020731146233 1.00328485727224

ENSG00000175899.13 0.203595948880489 0.999684045068285 0.999197160723453 1.00017116665995

ENSG00000109320.10 0.749368071039506 1.00211412662152 0.989214017381796 1.01518246317654

ENSG00000239713.6 0.775243727869787 1.00339199959918 0.980337240648499 1.02698894126845

ENSG00000170231.14 0.568520770490307 1.00312126207778 0.992433670375547 1.01392394924665

ENSG00000167207.10 0.764617233099583 1.01382342646998 0.926689301230703 1.10915054128099

ENSG00000114115.8 0.262702420597532 1.00321832137984 0.997593272513222 1.00887508775662

ENSG00000137462.6 0.0741367535344356 0.993544707325787 0.986507679509867 1.00063193217667

ENSG00000138449.9 0.435084880088479 0.999339502878302 0.997682850840566 1.00099890578619

ENSG00000122861.14 0.0193092753049492 1.00062809412929 1.00010186991394 1.00115459522836

ENSG00000125538.10 0.974356315838224 0.999712342077911 0.982328600986251 1.01740371388911

ENSG00000122133.15 0.813474424732548 1.00005242270688 0.999617039783225 1.00048799526145

ENSG00000100979.13 0.144559057292837 1.00092833774026 0.999681462233336 1.0021767684411

ENSG00000157601.12 0.220190361139451 1.00232952025825 0.9986080549822 1.00606485414244

ENSG00000107201.8 0.0723531162070132 1.0056155133951 0.999491926739268 1.01177661742604

ENSG00000126456.14 0.06989818538936 1.00887743805548 0.999281550413258 1.01856547296051

ENSG00000185303.14 0.12694823481185 0.999970659696005 0.999932982968162 1.00000833784348

ENSG00000138207.11 0.158958099854624 1.00172295454419 0.999326369933064 1.00412528664479

ENSG00000122852.13 0.108232850508616 0.999961441469651 0.999914392090891 1.00000849306224

ENSG00000086991.11 0.657135524417584 1.01717613446182 0.943490610812051 1.09661641214234

ENSG00000012223.11 0.0802931078663054 0.998662411442803 0.997165598677315 1.00016147102513

ENSG00000139194.6 0.373371194376972 0.987380638330318 0.960154180805009 1.01537913851731

ENSG00000164687.9 0.791889009694922 0.998684841994957 0.988969720257281 1.00849540001187

ENSG00000121769.6 0.328782818551088 0.998559315421096 0.995674134763526 1.0014528565122

ENSG00000170323.7 0.519641637361952 0.998061494518042 0.992183965600343 1.00397384091655

ENSG00000125999.9 0.416091219883826 0.999897736992244 0.999651313443446 1.00014422128679

ENSG00000135114.11 0.031861917915104 1.01474662815499 1.00127071644669 1.02840390959018

ENSG00000143320.7 0.584345176948163 1.00005292068696 0.999863329658846 1.00024254766474

ENSG00000162444.11 0.998894033729147 0.999989485644675 0.985232348551317 1.01496766003498

ENSG00000137857.16 0.328222994404104 0.995365584502646 0.986139698411906 1.00467778389595

ENSG00000087237.9 0.503828351376275 1.03410215650741 0.937267888153033 1.14094090239297

ENSG00000198183.10 0.202064025838564 0.999965879114858 0.999913458347076 1.00001830263081

ENSG00000176919.10 0.604913688611429 1.00597100703328 0.983536464385851 1.02891728332966

ENSG00000137558.6 0.597733430620744 0.996240698979939 0.982400373603358 1.01027601064895

ENSG00000007952.16 0.024880526880551 1.07571580340273 1.0092596671265 1.14654783836252

ENSG00000189058.7 0.998149876404013 0.999998288376481 0.998552579746346 1.00144609010966

ENSG00000228278.3 0.707639438255249 0.999417682423583 0.996379900328582 1.00246472616673

ENSG00000229314.5 0.38748014361372 0.999518489516865 0.998427301095623 1.00061087050583

ENSG00000232810.3 0.383274163851152 0.979439117707329 0.934748891832188 1.02626597760924

ENSG00000100448.3 0.0652776170202958 0.951007444913515 0.901545669058134 1.00318285730971

ENSG00000100030.13 0.140356031930309 1.00450642260132 0.998520599286233 1.01052812908274

ENSG00000140464.18 0.0426824491361154 1.01094476316412 1.00035904095953 1.0216425026645

ENSG00000181026.14 0.00480788413991196 1.04515482197617 1.01355638489112 1.0777383658013

ENSG00000165168.7 0.21261744353303 0.997867900957349 0.994524367058997 1.00122267562496

ENSG00000131050.9 0.0976621331155597 1.00225566062283 0.999586992637277 1.00493145333977

ENSG00000172183.13 0.137305641589366 1.00742206174975 0.997643576520647 1.01729639160275

ENSG00000069399.11 0.920979989599412 1.00019191703259 0.996406869018205 1.00399134329839

ENSG00000143319.15 0.343078450860588 1.00834563070307 0.991169274930922 1.0258196421886

ENSG00000140279.11 0.888101015012921 0.998127098918269 0.972400608057176 1.02453422729289

ENSG00000164342.11 0.386422551355482 1.01570707705983 0.980509898718393 1.05216772185359

ENSG00000072274.11 0.391087812258273 1.00137326008369 0.998237738345417 1.00451863067479

ENSG00000115267.5 0.0620494082172998 1.01086965267436 0.999455401487106 1.02241425998354

ENSG00000123384.12 0.866448238568155 0.99959835895259 0.994929294338501 1.00428933483665

ENSG00000132256.17 0.86230311830992 0.998045559180145 0.97622344218773 1.02035547924044

ENSG00000131203.11 0.616424277209542 0.999418749862467 0.997147741408066 1.00169493054881

ENSG00000130513.6 0.180880440197717 0.999231721523308 0.998107418522167 1.00035729097856

ENSG00000069869.14 0.0429813585182844 1.06070988093573 1.00186337582332 1.12301285650858

ENSG00000168610.13 0.588734714927569 0.998779447012983 0.994367342328978 1.00321112863492

ENSG00000115415.17 0.0720470552765955 1.00098555658659 0.999911758485545 1.00206050783175

ENSG00000184557.4 0.446621938981154 1.00046019971147 0.999275471178337 1.00164633284397

ENSG00000121858.9 0.23800769612161 0.998686677825529 0.996509043463711 1.00086907089119

ENSG00000115009.10 0.305194600363716 1.0004679092398 0.999573694386719 1.00136292405411

ENSG00000185338.4 0.951735182763376 0.999603189520946 0.98683875482381 1.01253272798234

ENSG00000135828.10 0.4725669025471 0.983673378232436 0.94046809373211 1.02886352178454

ENSG00000125347.12 0.16596726485463 1.00412983833817 0.998291718186028 1.01000210046133

ENSG00000164136.15 0.960476332248626 0.997133478322561 0.890113654415809 1.117020471104

ENSG00000128394.15 0.106551755720867 1.03888328581767 0.99185339784437 1.08814315088999

ENSG00000133321.9 0.904987385510148 0.999957222370164 0.999255069797905 1.0006598683282

ENSG00000133063.14 0.678500975560018 0.999345437217103 0.996256205069202 1.0024442485829

ENSG00000101017.12 0.963242872311667 1.00016585306761 0.993136396691214 1.00724506419784

ENSG00000196664.4 0.035182801287828 0.928488274328667 0.866544469134575 0.994860051933403

ENSG00000196262.12 0.0022917446111147 1.00285876159958 1.00102048948499 1.00470040951357

ENSG00000159840.14 0.0501421903036552 1.00233478058909 0.999998553443233 1.00467646570011

ENSG00000160703.14 0.0567933910198065 1.03045499210453 0.999133128360477 1.06275876618721

ENSG00000096088.15 0.802250657155823 0.999997333755179 0.999976467366656 1.00001820057912

ENSG00000112715.19 0.363646011876311 1.00159074294805 0.998161859736574 1.00503140505086

ENSG00000263528.6 0.238254924336026 1.0141220040307 0.990761143099421 1.03803368372112

ENSG00000187608.7 0.131039632959748 1.00033838043917 0.999899192676705 1.00077776110698

ENSG00000108771.11 0.418139390478797 1.00591473580948 0.991656428976712 1.02037805247005

ENSG00000118503.13 0.316552250479059 1.00317500205687 0.996971263779491 1.00941734362203

ENSG00000106327.11 0.616423918536717 1.01289229063932 0.963376246580635 1.06495338252115

ENSG00000145113.20 0.802548139708414 1.0008504295561 0.994203870399225 1.00754142300854

ENSG00000181104.6 0.783796655984972 1.00107611983592 0.993414318923045 1.00879701310544

ENSG00000049540.15 0.348845001607501 0.997837904748188 0.993329652570001 1.00236661774485

ENSG00000090382.5 0.0858646890504167 0.999771532360282 0.999510894370516 1.00003223831545

ENSG00000271503.4 0.876877080174518 1.00013432980666 0.998436292433008 1.00183525502698

ENSG00000083799.16 0.0888584150351848 0.966885769832589 0.930097084190269 1.00512958033692

ENSG00000159176.12 0.340850443622129 0.996618897826346 0.989697408306339 1.00358879306791

ENSG00000112062.9 0.83599592268577 0.998392501147952 0.983300795458153 1.01371583441466

ENSG00000177606.6 0.968347736911676 1.00003036424928 0.998531670353421 1.00153130753137

ENSG00000138448.10 0.0196829626471375 1.00456255676136 1.00072704998372 1.0084127639633

ENSG00000128604.17 0.850801150525367 1.00210944322515 0.9803456840581 1.0243563597323

ENSG00000101916.11 0.114544549984346 0.965374574903152 0.924034872388248 1.00856374333659

ENSG00000115523.15 0.748317581281041 0.99981613771479 0.998693748961164 1.00093978787264

ENSG00000110651.10 0.139124327410556 0.998274959456011 0.995993619655533 1.0005615247029

ENSG00000055332.15 0.265825602870584 1.00815198063532 0.993837266928544 1.02267287601321

ENSG00000204444.9 0.393681148902077 0.976981214838314 0.926075480119839 1.03068520292043

ENSG00000116161.16 0.0626920007683526 1.00707367160101 0.999626705258628 1.01457611596075

ENSG00000106100.9 0.281797508185549 0.986612414869535 0.962691806635066 1.01112739348751

ENSG00000107643.14 0.534585858587427 1.01356489676088 0.971368078737334 1.05759477013212

ENSG00000102882.10 0.799957709540331 1.00104179078 0.993012128584568 1.00913638216725

ENSG00000130303.11 0.916906135122901 0.999962911087871 0.99926641829807 1.000659889336

ENSG00000137274.11 0.120136965296086 0.97187967017141 0.937542378971866 1.00747455739367

ENSG00000188257.9 0.171996609174317 0.995274066928996 0.988531286987439 1.00206283942733

ENSG00000030582.15 0.871521041018855 0.999928915441075 0.999067844332449 1.00079072868494

ENSG00000134853.10 0.971909406580647 0.99973668245559 0.985189427632967 1.01449874127117

ENSG00000127955.14 0.481901861450195 1.00391211167299 0.993046899274028 1.01489620349302

ENSG00000114251.12 0.288113543101101 0.989540787688049 0.970529059031915 1.00892493778085

ENSG00000140564.9 0.00501989539855408 1.00026506068431 1.0000798947389 1.00045026091341

ENSG00000160710.14 0.470812141652348 1.00089546302507 0.998463643284421 1.00333320561257

ENSG00000105397.12 0.725555449947455 1.00231988865824 0.989435891428933 1.01537165560971

ENSG00000131323.13 0.928174712602404 1.00162459533518 0.966888668850508 1.03760842618321

ENSG00000133112.15 0.417605027627884 0.999894948122118 0.999640981698975 1.00014897906737

ENSG00000198467.12 0.131637245624454 1.00191581960339 0.999425989039041 1.00441185298447

ENSG00000067141.15 0.733773767104688 0.998103360537866 0.987243410604691 1.00908277291696

ENSG00000124942.12 0.0252237996989348 1.0023168440231 1.00028750142838 1.00435030366552

ENSG00000174125.6 0.342679918595321 0.980434868899087 0.941225670520784 1.02127742820838

ENSG00000166548.14 0.154843462492662 0.985500730147153 0.96586871867523 1.00553177708526

ENSG00000167815.10 0.829118199892379 0.999770996264531 0.997693825309061 1.00185249183249

ENSG00000183486.11 0.565419585506646 1.00452716883374 0.989174264210963 1.02011836481618

ENSG00000138685.11 0.684676054938502 1.01741944307228 0.936055755885138 1.10585541153226

ENSG00000171560.13 0.201944779961271 1.00012394386064 0.999933574227962 1.00031434973632

ENSG00000148737.14 0.996688397545313 1.00003473660194 0.983764774384032 1.01657377906897

ENSG00000164251.4 0.137858313425563 1.00422954723491 0.998646533563596 1.00984377319265

ENSG00000149476.13 0.115987422866783 1.01676342355803 0.995903458210056 1.03806031695433

ENSG00000038945.13 0.17591532477767 0.993337474312296 0.983767838788748 1.00300019879489

ENSG00000144802.10 0.487030951485288 0.996873337381688 0.988108907536532 1.0057155068666

ENSG00000105983.18 0.251275723308995 1.0169339191949 0.988175045946325 1.04652976236487

ENSG00000197122.10 0.233297946712924 1.00511280690311 0.996719124621228 1.01357717499859

ENSG00000066044.12 0.11867085861822 1.01620261141532 0.99589248877485 1.03692693647857

ENSG00000154134.13 0.566599698583534 1.02647188360448 0.938725468538141 1.12242030619597

ENSG00000185591.8 0.0172082010393682 1.01360917265998 1.00239891572631 1.02494479870424

ENSG00000142168.13 0.26129453236736 1.00102041400129 0.999240702292927 1.00280329549023

ENSG00000258429.1 0.0813226496582086 1.06343762279391 0.992375516027048 1.13958835068916

ENSG00000128917.6 0.243132994076596 1.00911010939857 0.993859237618518 1.02459500736789

ENSG00000122882.9 0.51110809957672 1.00795276667895 0.984417199433057 1.03205102515565

ENSG00000018280.15 0.958165572367321 1.0005118998423 0.981562054151499 1.0198275875603

ENSG00000187908.14 0.230529455913559 0.999141091425873 0.997738646961163 1.00054550719888

ENSG00000184584.11 0.0685782642442672 0.996579359993787 0.99291113576472 1.00026113615969

ENSG00000204351.10 0.827470222317896 1.00149962392341 0.988094170792167 1.01508694855938

ENSG00000175084.10 0.468424130833001 0.997404027866311 0.990420202272304 1.00443709904297

ENSG00000156136.8 0.694183412809791 1.00251348027566 0.990046593563063 1.01513735279612

ENSG00000204209.9 0.842273844149046 1.00105372671032 0.990722972824879 1.01149220442852

ENSG00000104689.8 0.607827961910753 1.00389669630433 0.989095363901792 1.01891952346752

ENSG00000120889.11 0.0751806808557338 1.00522570970816 0.999471116095631 1.01101343619178

ENSG00000074266.16 0.556646483133871 1.01636122603237 0.962826629080883 1.07287242643894

ENSG00000275302.1 0.813252257032282 0.99867957551461 0.987791358773792 1.00968781078236

ENSG00000169756.15 0.271013696830072 1.00900729781236 0.9930248029502 1.02524702707718

ENSG00000100298.14 0.197791296374724 0.95792445206121 0.897263405277739 1.02268659399157

ENSG00000187045.15 0.637729996472794 1.00145679417661 0.995406985843074 1.00754337157185

ENSG00000133710.14 0.570800503984205 0.99892683906348 0.995225250579072 1.00264219504153

ENSG00000019169.10 0.450518757634845 0.999291930367322 0.997455043162503 1.00113220033573

ENSG00000126581.11 0.759112454902245 1.00268438462432 0.985650197676181 1.0200129595061

ENSG00000120659.13 0.664844623879656 0.993860070071809 0.966550529114991 1.02194123238189

ENSG00000103653.15 0.111062130711358 1.00845097881019 0.998066123544458 1.01894388825826

ENSG00000055118.13 0.946179104027468 0.999467972441012 0.984143863888558 1.01503069275699

ENSG00000130522.5 0.548425294394733 1.00046246449882 0.998952871497153 1.00197433876029

ENSG00000162434.10 0.145221569787261 1.00451813447462 0.998443455978069 1.01062977221871

ENSG00000189143.9 0.576236501316426 1.00029396266253 0.999263449343609 1.00132553872191

ENSG00000151882.10 0.151033299202238 0.983635720559188 0.961730163035234 1.00604022619653

ENSG00000185507.18 0.236204660891381 1.0028616468478 0.998131078247017 1.00761463562944

ENSG00000136381.11 0.300828372859295 1.01309183801705 0.988435210927787 1.03836352743178

ENSG00000166333.12 0.659696639502282 0.989083407881893 0.94192023995348 1.03860809679127

ENSG00000150782.10 0.221283138617106 1.00548360662744 0.996710980464246 1.01433344571526

ENSG00000213903.7 0.86550291603838 0.997252784832158 0.966006124393914 1.02951015707014

ENSG00000128383.11 0.375713826699917 1.02818283053625 0.966859913453138 1.09339514266744

ENSG00000204713.9 0.179276343986208 0.988730518513778 0.972511284942226 1.00522025129878

ENSG00000173039.17 0.0583274469726266 1.01001506249842 0.999648792867113 1.02048882942961

ENSG00000168685.13 0.278769674135273 0.995387869171378 0.987095726611693 1.00374967025189

ENSG00000163661.3 0.109127125030396 1.01609590552641 0.996439960438646 1.03613958714888

ENSG00000159110.18 0.0282066040606911 0.975255458867322 0.953672604195675 0.997326761685354

ENSG00000142765.16 0.666526037133543 1.00231828180674 0.991817184240963 1.01293056221128

ENSG00000244509.3 0.247330956367679 1.00390693988792 0.997296688165829 1.0105610054804

ENSG00000100201.17 0.914017212524693 0.999819155529459 0.9965420781759 1.00310700938329

ENSG00000073756.10 0.780806669106235 0.999779547875562 0.998228182369668 1.00133332438825

ENSG00000122545.16 0.245006340892556 1.00625782974881 0.995730286723155 1.01689667717449

ENSG00000102245.6 0.0265445897031312 0.913243895928821 0.842871057021524 0.989492291262712

ENSG00000170458.12 0.796647299733069 0.999780151324688 0.998109576167895 1.00145352258866

ENSG00000115718.16 0.283236262962574 1.01214050225145 0.990076006561415 1.0346967197556

ENSG00000126934.12 0.0253938712323254 1.01159470582771 1.00142124701369 1.02187151701671

ENSG00000169032.8 0.0096814061170386 1.01078447573728 1.00260334237381 1.01903236625214

ENSG00000104419.13 0.0824537806486199 1.00122496500963 0.999842570160362 1.00260927117531

ENSG00000213928.7 0.347190543352414 0.978836780589815 0.936141862778044 1.02347889901234

ENSG00000132274.14 0.097376247407877 0.993213637183939 0.985249287701064 1.00124236718805

ENSG00000115365.10 0.0306533006246198 1.0074739739499 1.00069465456968 1.01429922059779

ENSG00000149923.12 0.0348509465169582 1.00377110677357 1.00026782658578 1.00728665664727

ENSG00000100292.15 0.166563993155292 1.00249232066433 0.99896291720403 1.00603419374544

ENSG00000189403.13 0.508927312257131 1.00274788070667 0.99461590525741 1.01094634315294

ENSG00000125257.12 0.0149620030452178 0.978408618740899 0.96135672691185 0.995762965430678

ENSG00000019991.14 0.0341865443115156 0.926486823370442 0.863278337566634 0.994323379292251

ENSG00000116478.10 0.515060810868268 1.00147492013505 0.997040802678574 1.00592875734379

ENSG00000185436.10 0.134906252406223 0.96045819381393 0.910978221257988 1.01262568142457

ENSG00000188313.11 0.0605930364735409 1.00498277724689 0.999778819430233 1.01021382223165

ENSG00000112182.13 0.227293587418502 1.08510605516823 0.950360810538962 1.23895591853688

ENSG00000136560.12 0.409162420184297 1.00864559158214 0.988236131857461 1.02947655587726

ENSG00000105851.9 0.0056986920861446 0.911542459804813 0.853613085293529 0.973403138192623

ENSG00000137486.15 0.0773066874767474 0.989827613927228 0.978662417143189 1.00112019030309

ENSG00000134321.10 0.582213945568325 1.00406148263277 0.989666980238367 1.0186653500998

ENSG00000183735.8 0.305531117869059 1.0091272633613 0.991740505739651 1.0268188379576

ENSG00000113721.12 0.745169509896048 1.00081256808386 0.99592247343286 1.00572667366576

ENSG00000188389.9 0.439640315821846 1.0136466563579 0.979393276070798 1.04909801715984

ENSG00000081181.6 0.109016735093437 0.978860624694897 0.953614315321243 1.00477531344033

ENSG00000103569.8 0.67391126365183 0.996453965786334 0.980102000206783 1.01307874662211

ENSG00000117560.7 0.345853215957062 0.965323460329456 0.89702597642879 1.03882095674897

ENSG00000091583.9 0.883415678564887 1.00019131020359 0.997637325124745 1.00275183357006

ENSG00000089685.13 0.00367280968602279 1.00795105923978 1.00257963672075 1.01335125970204

ENSG00000197043.12 0.583802445938802 0.998384843174711 0.992627745570632 1.00417533111366

ENSG00000026025.12 0.153568202572996 1.00043595729337 0.999837241963876 1.00103503114126

ENSG00000162692.9 0.641764544125566 0.997509152007724 0.987083492237111 1.00804492848326

ENSG00000117450.12 0.200711146971686 1.00021114798684 0.99988773249021 1.00053466809281

ENSG00000162645.11 0.200921989861828 1.00330956904306 0.998242098399327 1.00840276416663

ENSG00000198246.7 0.305777303074994 0.987969676983998 0.965348124586031 1.01112133310296

ENSG00000089127.11 0.00128209138652569 1.00351150158088 1.00137261185487 1.00565495988527

ENSG00000204305.12 0.207831748737598 0.999345005157212 0.998326632090425 1.00036441704603

ENSG00000110057.6 0.593874643093741 1.00135788594002 0.996375823939318 1.00636485916516

ENSG00000117586.9 0.718405009468402 0.987687594908743 0.923368121602979 1.05648739902683

ENSG00000184009.8 0.00057992805143614 1.00018865236812 1.00008118800069 1.0002961282832

ENSG00000122729.17 0.506457563892445 1.00235677728265 0.995419889443176 1.00934200694593

ENSG00000172724.10 0.298605963309401 0.99897740503423 0.997052024840082 1.00090650327799

ENSG00000275385.1 0.84448922087431 0.999938616317185 0.999325481201439 1.00055212762135

ENSG00000102970.9 0.3179396320709 0.995848180879483 0.98775022212843 1.00401252983169

ENSG00000006606.7 0.961109099375228 1.00108100757287 0.958537291381644 1.04551298393262

ENSG00000102962.4 0.941387225517296 0.999687185327866 0.991384584518899 1.00805931836607

ENSG00000276070.3 0.880490655774276 1.00138107525777 0.983525714594629 1.01956059003267

ENSG00000126353.3 0.266582883328014 0.987069964183698 0.964665130542625 1.00999516137331

ENSG00000179934.6 0.321086607576267 0.935822322417689 0.820901831816981 1.06683087452348

ENSG00000108691.8 0.286825865413118 0.998026887246261 0.994406230457401 1.0016607269328

ENSG00000137077.6 0.0470066071897488 1.00034945761118 1.00000462230196 1.00069441183125

ENSG00000108688.10 0.430043080387344 1.01391339691781 0.979707156039331 1.04931394050994

ENSG00000277632.1 0.807356228175821 0.998459671934768 0.986164426731532 1.01090821110249

ENSG00000172156.3 0.726082471441156 0.995855980976269 0.972987287034534 1.01926217131654

ENSG00000160791.13 0.267339669547132 0.986541574865534 0.963201924667036 1.01044677550308

ENSG00000274736.3 0.510911755030918 0.989069922986728 0.957187953797981 1.02201381523387

ENSG00000276085.1 0.349057286441891 0.980000133781306 0.93942492334042 1.02232785010256

ENSG00000276070.3.1 0.880490655774276 1.00138107525777 0.983525714594629 1.01956059003267

ENSG00000276085.1.1 0.349057286441891 0.980000133781306 0.93942492334042 1.02232785010256

ENSG00000163823.3 0.347008751714758 0.993933213024885 0.981407059227288 1.00661924393717

ENSG00000106178.5 0.75187311842392 0.996999919537758 0.978601994386133 1.01574372958623

ENSG00000143185.3 0.170161410487349 0.963558285825771 0.913780083665725 1.01604815729726

ENSG00000121966.6 0.378218094527504 0.999077667152145 0.9970292281357 1.0011303147738

ENSG00000172215.5 0.091504908595601 0.968633043536987 0.93343472283416 1.0051586362493

ENSG00000183813.6 0.111370460962815 0.9629757878184 0.919273004976117 1.00875622682792

ENSG00000219438.7 0.693662878752196 0.99481307944285 0.96940060605647 1.02089173129

ENSG00000120899.16 0.489323147398403 0.993664314147704 0.975921039437196 1.01173018032282

ENSG00000039068.17 0.109642806599411 1.00118747435423 0.999732858063052 1.00264420711937

ENSG00000049323.14 0.785198763824047 1.00093347698349 0.99424000989313 1.00767200613256

ENSG00000136634.5 0.257400684608068 0.922050877686301 0.801236756784314 1.06108190100287

ENSG00000132170.18 0.0224724985343761 1.01349742635255 1.00189525116127 1.02523395737496

ENSG00000000938.11 0.472003590258244 0.994394532094137 0.979277966979832 1.00974444315164

ENSG00000240972.1 0.00261810525958461 1.00286667238316 1.0009986991003 1.00473813150889

ENSG00000096968.11 0.423671556284225 0.982937849624259 0.942359881279561 1.02526310321283

ENSG00000169398.18 0.85869324476478 0.998764350209007 0.985261970500908 1.01245177132055

ENSG00000114013.14 0.312276114623292 0.991256807514219 0.974515159744552 1.0082860678133

ENSG00000101336.11 0.257079410532878 0.995136674834506 0.986781802367146 1.00356228623704

ENSG00000111424.9 0.940941074220388 1.00037066010554 0.990611001862152 1.01022647206503

ENSG00000173391.7 0.154620304024944 0.995309814120212 0.988887595540302 1.00177374107191

ENSG00000173020.9 0.463324739415403 1.00296732348317 0.995056768944562 1.01094076576352

ENSG00000074966.9 0.428248505614471 0.928015671366263 0.771407524238167 1.11641779376199

ENSG00000169385.2 0.102354113373475 1.02014550035737 0.996022441341609 1.04485280522153

ENSG00000105369.8 0.0153998492760073 0.996061952813118 0.99288760275745 0.999246451548504

ENSG00000007312.11 0.0559720582292598 0.9831909429956 0.966246880266683 1.00043213606214

ENSG00000254087.6 0.141946473120871 1.00464312812853 0.99845121884287 1.01087343662676

ENSG00000165025.13 0.378603159429523 0.995366632707201 0.985129530944655 1.00571011464535

ENSG00000010671.14 0.0174637615480098 0.960145726130665 0.928478680755602 0.992892819743313

ENSG00000095585.15 0.046959763584665 0.955807159262389 0.914122426723222 0.999392749800512

ENSG00000134215.14 0.657799628501865 1.00475322662971 0.983891037009673 1.02605777311588

ENSG00000141968.6 0.90960931283565 1.00112892231034 0.981817309605608 1.02082038000416

ENSG00000160293.15 0.0598442658863924 1.01054296119502 0.999565274044454 1.02164121037221

ENSG00000136238.16 0.0168646093839792 1.0012838430074 1.00023072633573 1.00233806847797

ENSG00000128340.13 0.103517758661626 1.00289319823007 0.999411147636768 1.00638738064356

ENSG00000169750.7 0.138288872103569 1.00413720485673 0.998670048204785 1.00963429111548

ENSG00000138814.15 0.148943537069181 1.00710884260604 0.997467255162537 1.01684362630029

ENSG00000107758.14 0.462239707285715 0.991033142528332 0.967518262787807 1.01511953558336

ENSG00000120910.13 0.119648351220675 0.965259907556325 0.923219673693153 1.00921450840455

ENSG00000187446.10 0.455860360132226 1.00064013284303 0.998958469347862 1.00232462727882

ENSG00000221823.9 0.389054880526945 1.00410502324088 0.994788380496299 1.01350892055512

ENSG00000102908.19 0.861007411070275 0.995384948998207 0.945155516746261 1.04828377884626

ENSG00000131196.16 0.152782448974647 0.955500061920536 0.897698586541909 1.01702328823654

ENSG00000101096.18 0.102085295784724 0.964101445554311 0.922757903262074 1.00729735723101

ENSG00000072736.17 0.0879054783817088 0.962756202223635 0.921690100934316 1.00565201251536

ENSG00000100968.12 0.271308496368417 0.987079586582615 0.964471605287216 1.01021751693556

ENSG00000174775.15 0.093005267839295 1.00892994671097 0.998518049868048 1.01945041204284

ENSG00000133703.10 0.0015595931436434 1.00368829620261 1.00140149073488 1.00598032383078

ENSG00000213281.4 0.000665932565129923 1.01116384602417 1.0047193194003 1.01764970948969

ENSG00000170345.8 0.46084223383446 0.999789754936239 0.999231197272715 1.00034862482647

ENSG00000198286.8 0.0117594112749133 1.01141328108251 1.00252274697282 1.02038265788878

ENSG00000142867.11 0.340008490860835 1.00522921330147 0.994517074441941 1.0160567347139

ENSG00000172175.11 0.37912916879172 0.976959514002931 0.92750565434805 1.02905021390056

ENSG00000213341.9 0.224579195067925 1.02011392129819 0.987849406209747 1.05343223965599

ENSG00000104365.12 0.0359068512890421 0.981860103316076 0.965210964834171 0.998796426488467

ENSG00000269335.4 0.644410377181933 1.01997454129002 0.937808551209867 1.10933949529211

ENSG00000100906.9 0.94222744261857 1.00003122061382 0.999187203167491 1.00087595100508

ENSG00000104825.15 0.00429383303798025 1.01638354868816 1.00511044121527 1.02778309296529

ENSG00000146232.13 0.645772949287728 1.00256023332998 0.991688538009379 1.01355111300597

ENSG00000177455.10 0.0817334649782882 0.969143215240063 0.935538017660602 1.00395553565476

ENSG00000117322.15 0.733636432710883 0.997643714778571 0.984180016542389 1.01129159798818

ENSG00000141506.12 0.251174690197695 0.970561362641992 0.922269637591428 1.02138173074151

ENSG00000145675.13 0.0183286728336099 0.962678994799986 0.932731522572882 0.993587998905331

ENSG00000117461.13 0.05512150229596 0.981686703291515 0.963318361723452 1.00040528833605

ENSG00000121879.3 0.182602472170803 1.03161045269143 0.985461793981941 1.07992022887264

ENSG00000051382.7 0.419171248112411 1.00709487025897 0.989968254254609 1.0245177795783

ENSG00000171608.14 0.366440996884886 0.989684239974093 0.967662390639973 1.01220725774546

ENSG00000117020.15 0.316117705874614 1.01317812554308 0.987573131280198 1.03944698530658

ENSG00000142208.14 0.218463812725571 0.991750385122142 0.97875183660019 1.00492156398547

ENSG00000105221.15 0.0986834766105988 1.020606481625 0.996194167520753 1.0456170336023

ENSG00000082701.13 0.341365606467078 1.00807506848605 0.99151158465867 1.02491525003512

ENSG00000168918.12 0.641024242543184 0.995545850197262 0.977038684167897 1.01440358084601

ENSG00000012124.13 0.128770957445771 0.967070485325842 0.926177772254631 1.00976869841273

ENSG00000137101.11 0.768031044602446 0.992773099365364 0.946058789988724 1.0417940589456

ENSG00000111679.15 0.75306938634523 1.00202223291955 0.989489774241986 1.01471342241435

ENSG00000204577.10 0.401814082642521 0.967091235687632 0.894321132892811 1.04578257601783

ENSG00000072694.17 0.240269603542298 0.978769622641053 0.944334652583695 1.01446025684206

ENSG00000152689.16 0.772632370681487 1.01329587434834 0.926455501385636 1.10827614217381

ENSG00000197943.8 0.0444926457414797 0.944516824051582 0.893366233224114 0.99859609389634

ENSG00000166501.11 0.0723960810690334 0.94966449120893 0.897637796227023 1.00470663072996

ENSG00000185885.14 0.798803940427352 0.999929694226524 0.999389257891373 1.0004704228116

ENSG00000125730.15 0.258064272086092 1.00026141897366 0.999808428189893 1.0007146149974

ENSG00000106804.7 0.759747729018163 1.00093196398204 0.994973983556212 1.00692562125103

ENSG00000217555.11 0.968999892463279 1.0001476462308 0.992728494470785 1.0076222449868

ENSG00000006210.6 0.166767299374326 0.99736851490401 0.993650179137392 1.00110076504529

ENSG00000189377.7 0.0931636204208074 0.999684226069251 0.999315723607581 1.00005286441797

ENSG00000142871.14 0.681599572201388 1.00016818157477 0.999365012805906 1.00097199583357

ENSG00000078401.6 0.971944708903098 0.999896311393627 0.994134777421366 1.00569123648595

ENSG00000127129.8 0.638388127268821 1.00132876168549 0.995798744714041 1.00688948876565

ENSG00000173432.9 0.356651820325846 0.999504260514458 0.998450976804619 1.00055865535203

ENSG00000134339.7 0.288620452011481 0.995974343991665 0.988581462282754 1.00342251168564

ENSG00000126524.8 0.239013556638698 1.00224535131145 0.998510578334041 1.00599409362428

ENSG00000075213.9 0.150709463350448 1.00778372957359 0.997182451209544 1.01849771259146

ENSG00000012171.16 0.0761168525878576 1.00551170162372 0.999422863882715 1.01163763471883

ENSG00000075223.12 0.0171076011682625 1.00660546884274 1.00117260003628 1.01206781914266

ENSG00000170381.11 0.715738334255632 0.994950370388754 0.968207243294771 1.02243217698727

ENSG00000001617.10 0.746138860069488 1.00120343539392 0.993939658517557 1.00852029643296

ENSG00000010319.5 0.224870122820872 0.974667469692601 0.935106018386328 1.01590264397647

ENSG00000196189.11 0.0267628846679564 0.983104166923079 0.968391669204497 0.99804018741246

ENSG00000185033.13 4.28044029284758e-05 1.0026018541961 1.00135472659211 1.00385053502316

ENSG00000168758.9 0.367498786295963 1.00511836095937 0.994019656442571 1.01634098781629

ENSG00000187764.10 0.33144464490451 0.981627121948151 0.945570987746213 1.01905813421893

ENSG00000135622.11 0.627779657911666 1.00883695356387 0.97358782280542 1.04536229299103

ENSG00000095539.14 0.000882748051891813 1.01999694369661 1.00816321463593 1.03196957600376

ENSG00000112902.10 0.401738956391839 1.01302212813831 0.982845343709135 1.04412544523542

ENSG00000092421.15 0.969334968515452 0.998857838628024 0.942321686269006 1.05878597121008

ENSG00000167680.14 0.199893320967828 1.02106812943165 0.989031228062523 1.05414277664765

ENSG00000143434.14 0.129241462851482 0.969583334601852 0.931653215575201 1.0090576912325

ENSG00000137872.14 0.102554515481382 0.903707543532818 0.800273535259655 1.02051022338648

ENSG00000138623.8 0.00319204459385292 1.01153500997043 1.00385287131094 1.01927593737882

ENSG00000145147.18 0.254112156543377 0.981284043046691 0.949932776960221 1.01367001591354

ENSG00000041982.13 0.388533494891172 1.00057697532513 0.999265990596285 1.0018896799974

ENSG00000025708.11 0.00699152138831116 1.00295592789645 1.00080712691409 1.0051093425007

ENSG00000197405.6 0.989553586034617 0.999945803201021 0.991865832143502 1.00809159559261

ENSG00000121797.9 0.17997821421779 0.974068343218087 0.937367475592332 1.01220616456749

ENSG00000174600.12 0.507638466223978 0.992853985541005 0.972011174705678 1.01414372823763

ENSG00000168329.12 0.106346316776176 0.959244543290171 0.912003589736012 1.00893253512118

ENSG00000186810.7 0.566520107656114 0.993392459338293 0.97112655772176 1.01616887152717

ENSG00000144476.5 0.372126399675594 1.00180654598077 0.997843568354422 1.00578526273915

ENSG00000173198.5 0.320145218523916 0.972375206127665 0.920128805896115 1.02758824137777

ENSG00000152207.6 0.113670693625857 0.823310708397642 0.647065585510106 1.04756076932736

ENSG00000213088.8 0.466944259834815 0.996696703958925 0.987850978121376 1.00562163897612

ENSG00000151617.14 0.734861111711677 1.00238804771184 0.988646271022941 1.01632082944684

ENSG00000136160.13 0.410006530277792 0.993391577315289 0.977845541877717 1.00918476755132

ENSG00000171051.7 0.387344275237687 0.993808593213255 0.979912353542827 1.00790189691322

ENSG00000171049.8 0.973366862750505 0.999141360044542 0.950004755664291 1.05081943158654

ENSG00000134830.5 0.157359310058505 1.01568511001703 0.994008931435869 1.03783397722606

ENSG00000213906.8 0.294661979088226 0.9326581444463 0.818643661569 1.0625517001315

ENSG00000011422.10 0.000281254769541217 1.00722653498233 1.00332034035394 1.01114793747192

ENSG00000114554.10 0.0211878144579403 1.00982116153261 1.00146266143722 1.01824942411294

ENSG00000076356.6 0.178046850085259 0.988916471416265 0.973005910949568 1.00508720084136

ENSG00000130827.6 0.67409313479166 1.00344680800849 0.9874832323066 1.01966844961049

ENSG00000164050.11 0.175952603178072 0.992372026800276 0.981427770332571 1.00343832663505

ENSG00000196576.13 0.074547535873398 1.00144482973808 0.999856925381306 1.00303525589592

ENSG00000198753.10 0.703685773897543 1.00365400264644 0.984966526778563 1.02269603041513

ENSG00000136040.7 0.132697463015262 0.978669468862887 0.951545529354987 1.00656658009167

ENSG00000004399.11 0.38727143397857 0.998060225656942 0.993676747306935 1.00246304116312

ENSG00000169403.10 0.525131573375139 1.00345041585325 0.992846227985275 1.01416786275084

ENSG00000169855.18 0.61086616338147 1.00442030635464 0.987500518014911 1.02162999756757

ENSG00000148926.8 0.0499025049120981 1.00516528634846 1.0000021933239 1.01035503684414

ENSG00000128165.8 0.842160845780927 1.0018352079889 0.983917547957527 1.02007915810592

ENSG00000135744.7 0.634279364327686 1.00083760527245 0.997391122799669 1.00429599705659

ENSG00000171388.11 0.466389780894865 1.00263050300259 0.995567756417688 1.00974335405201

ENSG00000109321.9 0.331731204023163 1.00046516706042 0.999526094402471 1.00140512199394

ENSG00000145050.14 0.343095547474957 0.99846053588591 0.995285469692662 1.00164573087701

ENSG00000185267.8 0.454849294429887 1.03495762511635 0.945774551329572 1.13255033589207

ENSG00000117407.15 0.611519261516861 0.994567066709741 0.97387715378698 1.01569653455489

ENSG00000168487.16 0.00981923609762236 1.01697608266121 1.00406430838355 1.03005389601984

ENSG00000125845.6 0.0819087488340384 1.00878997821492 0.998892669755679 1.01878535197958

ENSG00000152785.6 0.0794206126282408 0.990112456747587 0.979179836192783 1.00116714087823

ENSG00000125378.14 0.840075017362881 0.99928199987393 0.992335167156594 1.00627746382636

ENSG00000112175.7 0.00609880238390692 0.971592926478345 0.951786650223858 0.991811362935938

ENSG00000153162.8 0.73449367002453 1.00064089933616 0.996942819785159 1.00435269661711

ENSG00000101144.11 0.0170231326026964 1.00850426565573 1.00151382242366 1.0155435013213

ENSG00000183682.7 0.214941697170351 0.957180779750593 0.893212800302814 1.02572986505942

ENSG00000116985.9 0.272605091383541 0.970560899073749 0.920107794483293 1.02378054447396

ENSG00000174808.10 0.480798628038606 0.995594170134343 0.983447613059795 1.00789074928104

ENSG00000074842.6 0.0865270901594815 1.00206399404493 0.999704172115724 1.00442938638155

ENSG00000121691.4 0.0501772798302819 0.995886833658324 0.991787424524623 1.00000318710371

ENSG00000167775.9 0.863729006862042 0.999265002439538 0.990909722925137 1.00769073306987

ENSG00000125726.9 0.00282941474588583 1.06523404096059 1.02194766710132 1.11035388459743

ENSG00000093072.14 0.127262855150103 0.995233460838796 0.989140687729807 1.00136376337572

ENSG00000175505.10 0.00669376046763093 1.00522254047117 1.00144507611242 1.00901425347652

ENSG00000105472.11 0.148497157541117 1.00830469567622 0.997055880666155 1.01968042016205

ENSG00000089505.16 0.0933715951235496 1.08902410762962 0.985764427563281 1.20310032887888

ENSG00000140931.18 0.0552416617960113 1.00536553806175 0.999879991921997 1.01088117902958

ENSG00000183723.11 0.605440948500094 0.996624639151881 0.983921717960675 1.00949156140521

ENSG00000091317.7 0.432275122564524 1.00209939265946 0.996867931936153 1.00735830755238

ENSG00000153551.12 0.435384396612119 0.993762559932707 0.978260664402219 1.00951010447453

ENSG00000170293.7 0.677158373083678 1.00182790536293 0.993251987941557 1.01047786880737

ENSG00000184371.12 0.5070756743211 1.00154973392341 0.996978071029622 1.00614236026888

ENSG00000164400.5 0.0793363312134798 1.00503419041253 0.999412272982414 1.01068773238483

ENSG00000114646.8 0.421086922909575 0.984571538910341 0.947974610818753 1.02258130562699

ENSG00000150281.6 0.514957319924295 0.994982925425777 0.98003264032224 1.01016127540744

ENSG00000118523.5 0.927526435705346 0.999958796991704 0.999071360630995 1.00084702162773

ENSG00000107984.8 1.87724153966062e-06 1.00542742947485 1.00319200114621 1.00766783904319

ENSG00000105246.5 0.51106136878216 1.01149732106799 0.977593065294663 1.04657742249771

ENSG00000138798.10 0.189011319337104 1.01474900921915 0.992820063736154 1.03716231099948

ENSG00000124882.3 0.276191763301786 1.00133680964286 0.998931937140077 1.00374747174104

ENSG00000164283.11 0.650821306351493 1.00141071496211 0.995316281136641 1.00754246569313

ENSG00000183844.15 0.86667870011585 1.00063276381519 0.99327020478581 1.00804989739559

ENSG00000196937.9 0.348480396948903 1.00121582605789 0.99867577956948 1.00376233293744

ENSG00000198643.5 0.739631869887168 0.999250044590141 0.994838644708168 1.00368100588443

ENSG00000113578.16 0.0881599220368809 0.93227160897908 0.860070647197022 1.01053367620549

ENSG00000156427.7 0.369588784098959 0.982878131983632 0.946489397330464 1.02066586805551

ENSG00000140285.8 0.979139380353923 0.999623599325646 0.971809384428781 1.02823388654155

ENSG00000165197.4 0.0668446773804708 0.979760727737718 0.958569289485477 1.00142065278598

ENSG00000090554.11 0.358191434852601 1.08845933906475 0.908419906924356 1.30418072497824

ENSG00000266524.2 0.0650553990818468 0.958230946503205 0.915765888401808 1.00266515543496

ENSG00000135414.8 0.464668464137915 0.977344127525996 0.919114055053645 1.03926334099395

ENSG00000157017.14 0.561020535591722 0.95678439471329 0.824381108274412 1.11045288250608

ENSG00000197045.11 0.0970384385223283 1.01137846842718 0.997952646454238 1.02498491289388

ENSG00000130755.11 0.138447271095164 0.995079731422645 0.988608023765729 1.00159380470779

ENSG00000147437.8 0.698993854628244 1.02725510781693 0.896363863340316 1.17725970411563

ENSG00000105220.13 0.0404600348940227 1.00227809867934 1.0000989060348 1.00446203973481

ENSG00000166923.9 0.408686955073165 1.00267721819347 0.996337832803729 1.00905693910575

ENSG00000134443.8 0.60350107203011 0.997399883379795 0.987648530258354 1.00724751456452

ENSG00000113070.7 0.979268453511861 1.00006899348442 0.994878589186642 1.00528647676154

ENSG00000143321.17 0.014848630092434 1.00146476861434 1.00028609648751 1.0026448296118

ENSG00000166503.7 0.29856675648282 1.02258329959984 0.980419943288939 1.06655990810697

ENSG00000167244.16 0.48740385595712 0.999665180335491 0.998720806538432 1.00061044711672

ENSG00000168811.5 0.392869874809272 0.926898385226948 0.778766255693179 1.10320729776819

ENSG00000172349.15 0.0130039554802283 0.935939940056075 0.888297668000919 0.9861374209881

ENSG00000172458.4 0.104010156148981 0.827921214305197 0.659350747653887 1.03958862492472

ENSG00000125571.8 0.0343401725605746 1.00133347135018 1.00009830266767 1.00257016552441

ENSG00000136689.17 0.30379954601112 1.00407174117887 0.996325312285473 1.01187839855372

ENSG00000110944.7 0.372556569933508 1.00735230757433 0.991262398631535 1.02370338366132

ENSG00000162892.14 0.149150081546878 0.947498315479412 0.880553663592713 1.01953247707066

ENSG00000008517.15 0.0236775100042689 1.00367723157283 1.00049067894095 1.00687393334242

ENSG00000137033.10 0.0211528259317975 0.989404694150165 0.98048470672324 0.998405831415687

ENSG00000157368.9 0.384309596473175 0.977250847523801 0.927878135985688 1.02925069785319

ENSG00000134352.18 0.0819845256931934 0.995623947239292 0.990715610869502 1.0005566011485

ENSG00000104432.11 0.883029042760382 1.00506904892709 0.939601593036559 1.0750979996177

ENSG00000123999.4 0.0849039293665494 1.00228333479366 0.999686252175322 1.00488716436696

ENSG00000122641.9 0.529635738452007 1.00276857760026 0.994160897800866 1.01145078472385

ENSG00000163083.5 0.268767671856355 1.00211306569434 0.99837118884909 1.00586896702516

ENSG00000248099.3 0.0769527823860763 1.13761627014267 0.986149733812351 1.31234713524709

ENSG00000101384.10 0.00517817229535245 1.00894196283892 1.00266482024965 1.01525840322572

ENSG00000184916.7 0.344845091927973 0.993578686603954 0.980386737222152 1.00694814504609

ENSG00000049130.12 0.86396840698351 0.999566464043642 0.994620144939093 1.00453738155675

ENSG00000133116.7 0.15089526157421 0.926219617453139 0.834246775725652 1.02833214909201

ENSG00000128342.4 0.0201611317048747 1.00456966107574 1.00071332504257 1.00844085783597

ENSG00000148356.12 0.831265026120788 0.996988023807075 0.969705047870363 1.02503861539929

ENSG00000227507.2 0.246103785692048 0.996240293783395 0.989919161049163 1.0026017901358

ENSG00000119681.10 0.35056968338526 0.998313370104232 0.994781338373804 1.00185794252844

ENSG00000168056.13 0.747749265299834 1.00103361792067 0.994751032070898 1.00735588293003

ENSG00000090006.16 0.992581043664021 0.999969449792036 0.993550721058711 1.00642964603948

ENSG00000110492.14 0.208252151101648 1.00027013498174 0.999849425496303 1.0006910214903

ENSG00000105835.10 0.0300885033136453 1.0021624864979 1.00020813594388 1.0041206557432

ENSG00000117691.8 0.837734522038011 0.999759554821041 0.997461295739694 1.00206310934075

ENSG00000134259.3 0.330529194481578 0.988278859578108 0.965084951499915 1.01203018736438

ENSG00000197696.8 0.00518942442918635 1.00656055111877 1.00195559864797 1.01118666778814

ENSG00000136999.4 0.191703595680984 1.00747551672291 0.996273804238197 1.01880317687588

ENSG00000171119.2 0.887362466611187 1.00278505085602 0.964926696167523 1.04212875673795

ENSG00000225950.6 0.987001208227252 1.00056211159544 0.935156743632779 1.07054196633528

ENSG00000106809.9 0.127604144289815 0.974836575837668 0.943395577626903 1.00732542331965

ENSG00000140961.11 0.899667695916155 0.999680753431438 0.994731069297263 1.00465506670789

ENSG00000099985.3 0.197863847856968 1.01221510803018 0.993681040854555 1.03109487129132

ENSG00000197461.12 0.792396858649377 1.00122800479747 0.992119508954299 1.01042012433293

ENSG00000100311.15 0.0109685694154699 1.01258878482573 1.00287499935162 1.02239665742765

ENSG00000145431.9 0.0449019710379719 1.01411392282064 1.00031876418212 1.02809932721749

ENSG00000170962.11 0.835271022078426 1.00229334580579 0.980884948831435 1.0241689937677

ENSG00000104213.11 0.863436540989381 1.00085368837726 0.991168872586874 1.01063313552613

ENSG00000119630.12 0.313548748296002 0.989825537923639 0.970333848144032 1.00970876920331

ENSG00000168081.7 0.0356391362854673 0.911247968135333 0.835570381900944 0.993779671248822

ENSG00000115138.9 0.899704898503282 1.0027396596512 0.96097159139251 1.04632315257143

ENSG00000125650.4 0.268023274027068 1.13405339283526 0.907733918975541 1.41679964901238

ENSG00000087494.14 0.977385630926916 0.999765996057141 0.983718442376666 1.01607533600494

ENSG00000105894.10 0.167130130292862 0.990344296103362 0.976805133700734 1.00407112021275

ENSG00000029725.15 0.666771363257358 0.994159995718867 0.968004974360808 1.02102171297246

ENSG00000177548.11 0.341039798164054 1.01155305381596 0.987914338185139 1.03575739427384

ENSG00000104918.6 0.752120693155792 0.998844331670774 0.991702659529189 1.00603743402734

ENSG00000171951.4 0.809851190993091 0.99976021205008 0.997809149801741 1.00171508930041

ENSG00000161055.3 0.0979908005032202 0.999862187695486 0.999698973296904 1.00002542874103

ENSG00000070031.3 0.117380732695736 1.04173136380344 0.989762923318276 1.09642845651716

ENSG00000164022.15 0.0158064183587089 1.01614129891399 1.00301274122913 1.02944169791231

ENSG00000141574.6 0.0119946524387559 1.0064778973195 1.00142060171857 1.01156073287712

ENSG00000118785.12 0.978358748153042 0.999997684601758 0.999830405617431 1.00016499157309

ENSG00000159167.10 0.560253318646066 1.00225950734972 0.994676635555557 1.00990018681985

ENSG00000113739.9 0.00258393324088579 1.01746876132587 1.00607219044339 1.02899443012907

ENSG00000163235.14 0.466032716544568 1.00241685580766 0.995931751083614 1.00894418891054

ENSG00000092969.10 0.327207433944657 1.00791209457581 0.992146380381999 1.02392833404387

ENSG00000119699.6 0.730430628586592 1.00317584896356 0.985243806224607 1.02143426590021

ENSG00000164761.7 0.697752339067885 0.995938063039275 0.975688586388051 1.01660779807045

ENSG00000239697.9 0.587792716213454 0.996964393442606 0.986064581881209 1.00798468990353

ENSG00000161955.15 0.133822731386616 0.988994738383251 0.974789550473681 1.0034069323727

ENSG00000102524.10 0.821872839409282 0.998543084532305 0.985949016726176 1.01129802327721

ENSG00000125735.9 0.0141596340270689 1.07095687701695 1.01387819963988 1.13124893388307

ENSG00000181634.7 0.44397021580274 0.992949274723833 0.975122701138403 1.01110174239975

ENSG00000106952.6 0.347936765403527 0.965020147440888 0.895871142840163 1.03950650984746

ENSG00000125657.4 0.00413424695604692 1.02215068547208 1.00695928112216 1.03757127363354

ENSG00000160404.16 0.631295102510007 0.989752982855106 0.948982148421159 1.03227544237832

ENSG00000084652.14 0.966382635081706 0.999802909478434 0.99068007275356 1.009009755312

ENSG00000163794.6 0.155525397581052 1.02082782571283 0.992197697550265 1.05028408382976

ENSG00000178473.6 0.181633325058306 1.00032992770055 0.999845855071163 1.00081423469237

ENSG00000173511.8 0.336413644956356 1.00152839265044 0.99841459250347 1.00465190394489

ENSG00000150630.3 0.000147885847419982 1.0105022718755 1.00506399197755 1.01596997764929

ENSG00000135503.11 0.50792350653805 1.00261167999031 0.994899948826012 1.01038318681106

ENSG00000121989.13 0.226481160052544 0.953832864667137 0.883500696422039 1.02976391235861

ENSG00000114739.12 0.835472404022301 0.994904270457963 0.948076251519677 1.04404525035711

ENSG00000139567.11 0.418850117056101 0.994281624721279 0.980553116437163 1.00820234282712

ENSG00000159346.11 0.754340930053315 0.999507244193403 0.996426409705278 1.0025976042632

ENSG00000006831.9 6.08416643159693e-05 1.01592559701013 1.0081092913855 1.02380250581948

ENSG00000043591.5 0.38387821462303 0.989083097286552 0.964945011827066 1.01382499660331

ENSG00000169252.5 0.00999182321641146 0.928861186100681 0.878146705303815 0.982504515285819

ENSG00000144891.16 0.181768509152696 0.935867883188457 0.849110001376131 1.03149025846378

ENSG00000154188.8 0.0826341346638083 0.955488042424838 0.907599463781316 1.00590341406022

ENSG00000116194.11 0.504470119264129 0.961244415353731 0.855908175851703 1.07954433912176

ENSG00000136859.8 0.533593887378279 1.00143958700912 0.996914202952111 1.00598551355695

ENSG00000167772.10 0.000838423874357619 1.00275129274038 1.00113577250333 1.00436941992217

ENSG00000134817.10 0.694981275652186 1.00365224486382 0.985528542039901 1.02210923951039

ENSG00000169083.14 0.144960354009463 0.947403080930804 0.88101138290308 1.01879795786466

ENSG00000107779.10 0.987569989822067 0.99941346950629 0.928303062988303 1.07597111638873

ENSG00000138696.9 0.0961334852178077 1.01891010647603 0.996672150625356 1.04164424021238

ENSG00000204217.11 0.903737505298645 0.998953578491953 0.982147225056144 1.01604751968304

ENSG00000112983.16 0.539941580413091 0.993086587784974 0.971297743199557 1.01536421529169

ENSG00000171860.4 0.615953460734721 0.997845056601293 0.989469101523926 1.00629191497756

ENSG00000064989.11 0.268320602778077 0.991204514751319 0.975820402453671 1.00683116236652

ENSG00000150938.8 0.940639090515144 0.999666681137739 0.990933551191899 1.00847677644474

ENSG00000006016.9 0.533719881878983 0.999815375106169 0.999234147910941 1.00039694038537

ENSG00000176390.11 0.64415120314194 1.00763439885111 0.975635562608347 1.04068273099185

ENSG00000182578.12 0.511595891837702 0.998195842866175 0.992827735052206 1.00359297543488

ENSG00000198223.13 0.730491833406289 0.996942716843595 0.979724508916659 1.01446352686073

ENSG00000100368.12 0.110080122183703 0.985054711023341 0.967026927110506 1.0034185776074

ENSG00000119535.16 0.626035954089347 0.998032676049434 0.99015915831805 1.00596880218164

ENSG00000146648.14 0.257597735976483 1.00141494298851 0.998966541359989 1.00386934548922

ENSG00000106991.12 0.428884604215473 0.999041875906746 0.996672064845084 1.00141732172498

ENSG00000187266.12 0.786897684296493 0.996958867090513 0.975185611547023 1.01921826050494

ENSG00000091831.20 0.824113374992808 0.994764019948997 0.949761679243361 1.04189869628498

ENSG00000173153.12 0.193195231553605 1.00547929419544 0.997237273701542 1.01378943378558

ENSG00000077782.18 0.495930653855337 0.993891749709798 0.976516852076163 1.01157579415143

ENSG00000066468.19 0.158082766224074 0.981713227069539 0.956874892607598 1.00719630920289

ENSG00000068078.16 0.44746750965564 0.99771429119524 0.991841013405755 1.00362234813937

ENSG00000160867.13 0.527792197216305 1.00529325813788 0.98895248779304 1.02190403212674

ENSG00000127418.13 0.0839120759749044 1.00554370700187 0.999259692329674 1.01186723977003

ENSG00000102755.9 0.773968607539748 1.00300463322224 0.982677012477953 1.02375275038588

ENSG00000037280.14 0.26202720830669 1.01809391781761 0.98668599099647 1.05050161343672

ENSG00000010310.7 0.711829988175893 0.991369454190847 0.946808140674676 1.03802803596759

ENSG00000164850.13 0.683501125340741 1.00998154938036 0.962889936911595 1.05937624954368

ENSG00000164749.10 0.704371001735009 1.00522805162809 0.978513002990618 1.03267246596792

ENSG00000166736.10 0.138873939374901 1.0065365203797 0.997889319994925 1.01525865299693

ENSG00000159128.13 0.544103507387115 0.999064198544 0.996046641536716 1.00209089734235

ENSG00000140443.12 0.191025340635825 1.00887506221507 0.995600965508898 1.02232613910654

ENSG00000197081.11 0.544198235768502 0.997745739099118 0.990495284952808 1.00504926677957

ENSG00000110324.8 0.138459249314122 0.988126842723931 0.972636029884966 1.00386437198614

ENSG00000243646.7 0.744051196306186 1.00121653505791 0.993935743364037 1.00855066010663

ENSG00000137070.16 0.044410766400547 0.945982858371087 0.896126132760077 0.998613404539026

ENSG00000096996.14 0.751000839621277 0.990141725314962 0.931368649994808 1.05262361602482

ENSG00000131724.9 0.455877201786108 1.00138843640182 0.997743021144149 1.00504717077284

ENSG00000123496.6 0.847607800997467 1.00397940104804 0.964125322570608 1.04548092880837

ENSG00000134470.18 0.00874719346765052 1.02402080094621 1.00600987702831 1.04235418032681

ENSG00000177663.12 0.896251128100912 1.00239298816828 0.967021186125313 1.0390586236843

ENSG00000056736.8 0.437196252442062 0.991590627956744 0.970689332719165 1.01294197876605

ENSG00000163702.17 0.47261408851596 1.00669640415251 0.988527679124768 1.02519906274236

ENSG00000144730.15 0.540729281798744 0.980159819632325 0.919203205428783 1.04515874873775

ENSG00000163701.17 0.836568350289019 0.998538695018302 0.984760566374682 1.01250959826664

ENSG00000115604.9 0.101464900365658 1.04718394395945 0.990971741925484 1.10658474514699

ENSG00000115607.8 0.844601926348699 0.986871785081551 0.864713412025093 1.12628751520022

ENSG00000115594.10 0.661563645365992 0.998962866892012 0.994332479610401 1.003614816867

ENSG00000115590.12 0.0224195359861816 1.00814766997997 1.00114941509296 1.01519484420982

ENSG00000196083.8 0.574680614713892 1.00889933743966 0.978157456658099 1.04060738499483

ENSG00000115602.15 0.0602837602670734 1.01095052061395 0.999529091661164 1.02250246006454

ENSG00000115598.8 0.431787399775112 1.02158476749766 0.968616429462802 1.07744965441279

ENSG00000016402.11 0.516782827521441 1.00825022787936 0.983514204782101 1.03360837807523

ENSG00000174564.11 7.37509310982931e-05 1.00929268936702 1.00468708471019 1.01391940666138

ENSG00000103522.14 0.334207135062488 0.97095726505658 0.914578644593904 1.03081130981881

ENSG00000142677.3 0.250124718447205 1.00485021405811 0.996598304221829 1.01317045033611

ENSG00000104998.3 0.505980227674642 1.00240226035916 0.995339715068535 1.00951491873704

ENSG00000134460.14 0.605520006440162 1.00495918466939 0.98626917637756 1.02400337254861

ENSG00000100385.12 0.23709635240849 0.991347164244877 0.977167215953492 1.00573288175392

ENSG00000147168.11 0.529724141460906 0.998370085863965 0.993303687829608 1.00346232532966

ENSG00000185291.9 0.652054901237385 0.996113208372588 0.979393310950987 1.01311854267298

ENSG00000077238.12 0.0794549039900696 1.00768110446843 0.999101200466697 1.01633468944727

ENSG00000160712.11 0.621935852411243 0.997486869005035 0.987560186765494 1.00751333151276

ENSG00000171105.12 0.532196168909654 0.995752034661242 0.982540129127941 1.00914159649849

ENSG00000128052.8 0.181605832657166 0.995261820416432 0.988350431275276 1.00222153988492

ENSG00000116678.17 0.210405304785561 0.978781360067069 0.946476225826503 1.01218913341237

ENSG00000205213.12 0.118351403871693 1.0049562722104 0.998740092747859 1.0112111413054

ENSG00000133067.16 0.155246192884008 0.973814171020842 0.938823710224719 1.0101087449677

ENSG00000113594.8 0.019130027648351 0.979715332282145 0.963063150931104 0.996655444018103

ENSG00000111321.9 3.92489976075439e-08 1.01059384846573 1.00680185315626 1.01440012585899

ENSG00000258839.2 0.00290124041659334 1.1052294123735 1.0347952670152 1.18045771266321

ENSG00000105976.13 0.0122255539103427 1.00087503450089 1.00019047886047 1.00156005866849

ENSG00000064300.7 0.241440482192685 0.952918287588505 0.879046429929499 1.03299806688645

ENSG00000169418.9 0.98887405534501 1.00015860649995 0.978111011202876 1.02270317652976

ENSG00000113389.14 0.20080889079353 1.01571911983412 0.991734960804211 1.04028331275122

ENSG00000131910.4 0.467916348487933 0.996307878438018 0.986406567466384 1.00630857637867

ENSG00000126368.5 0.328235196362441 1.00513709601079 0.994865098843845 1.01551515170357

ENSG00000174738.11 0.542160776759403 0.995144691465937 0.97969195986871 1.01084115979226

ENSG00000131408.12 0.340182164767187 1.00391130417212 0.995890524207758 1.01199668251319

ENSG00000025434.17 0.844631280395638 0.998118172279246 0.979491369203384 1.01709919776455

ENSG00000120798.15 0.584287567344021 1.01189311969243 0.969931554311264 1.0556700430351

ENSG00000177463.14 0.364460261227057 0.986492668592311 0.957920980725187 1.01591655759502

ENSG00000175745.10 0.551700812668809 0.996467757331824 0.984924467683213 1.0081463340408

ENSG00000185551.11 0.173608899505401 1.00383703025208 0.998314789255584 1.0093898178717

ENSG00000160113.5 0.0346625560089562 1.00545019017205 1.00039201841077 1.010533937009

ENSG00000113580.13 0.955467260853034 1.000412392113 0.986039493356939 1.01499479588385

ENSG00000151623.13 0.107284572128224 0.971421924067662 0.93774252670794 1.00631093043434

ENSG00000123358.18 0.708305498618407 1.00047203277404 0.998001673192849 1.00294850725125

ENSG00000153234.12 0.0812842865740285 1.00339326701214 0.999579036039099 1.00722205247

ENSG00000119508.16 0.994574581265094 1.00006454157217 0.981632568605565 1.0188426090331

ENSG00000116833.12 0.177808395340327 1.05808696040502 0.974665232656337 1.148648765

ENSG00000148200.15 0.907630440766084 1.00181661521662 0.971568225629742 1.03300674522734

ENSG00000099250.16 0.811845478725614 1.00076866402516 0.994457380279259 1.00712000207938

ENSG00000118257.15 0.0465585232529303 1.01279406264833 1.0001937523447 1.02555311001603

ENSG00000060491.15 0.120453863789002 1.01029024004687 0.997319748251353 1.02342941762015

ENSG00000125510.14 0.952153801397002 0.997901639052633 0.93172725835788 1.06877594520417

ENSG00000145623.11 0.927871230756416 1.00011267930618 0.997675846024554 1.00255546457759

ENSG00000164040.15 0.939651357918637 0.999545490157681 0.987850567464804 1.01137886619694

ENSG00000186951.15 0.594454676361054 0.983800276884942 0.926390997748194 1.04476726042406

ENSG00000112033.12 0.0101856898742685 1.01000495027218 1.00236425191738 1.01770389119822

ENSG00000160951.3 0.194999281605641 1.03831937498886 0.980916504296939 1.09908144042288

ENSG00000125384.6 0.987143608628762 1.00015081108221 0.981973091007741 1.0186650266372

ENSG00000171522.5 0.197424246123099 0.98478974738481 0.962102944235718 1.00801151515511

ENSG00000160801.12 0.417355651238695 0.980242841467025 0.934092782171767 1.02867300399572

ENSG00000131759.16 0.523277756963794 1.00358933795852 0.992608691513503 1.01469145684015

ENSG00000077092.17 0.0417288670444839 1.03481812407328 1.00128247004555 1.0694769777222

ENSG00000172819.15 1.52377012674343e-07 1.02077046100903 1.01296580900118 1.02863524593788

ENSG00000069667.14 0.201128176000163 0.933791092108869 0.840691088656241 1.03720119728594

ENSG00000143365.15 0.864229091009259 1.00069800717893 0.992726416161335 1.0087336100555

ENSG00000186350.9 0.876661547127835 0.998633989951204 0.981543171086603 1.01602239744773

ENSG00000204231.9 0.151749904254167 0.989808829642779 0.976041576621419 1.0037702723998

ENSG00000170989.8 0.363359616418555 0.995032831070794 0.984406323329275 1.0057740502522

ENSG00000267534.2 0.949473668678174 0.999168671825261 0.973794150583964 1.02520438653115

ENSG00000080293.8 0.750537601875058 1.00113981039485 0.994133785135036 1.00819520968327

ENSG00000115884.9 0.419511287775124 0.999799897455756 0.999314236433074 1.00028579450693

ENSG00000169439.10 0.360431862610717 0.996612389760501 0.989390973800829 1.0038865137495

ENSG00000162512.14 0.451195746686297 1.00222221709191 0.996451572930845 1.0080262801716

ENSG00000124145.6 0.552700954910778 1.00013765341548 0.99968330781871 1.00059220550757

ENSG00000134243.10 0.231783016474616 0.994922944515959 0.986656987237941 1.00325815184805

ENSG00000139874.5 0.633947386135559 1.00542479632518 0.983283160238999 1.02806501925633

ENSG00000120156.19 0.190313140083663 0.981549392990187 0.95457110896737 1.00929014279683

ENSG00000106799.11 0.944542899493894 0.999599856326529 0.988390943377368 1.0109358846953

ENSG00000163513.16 0.212032902965628 0.997884209417956 0.994570412985746 1.00120904704609

ENSG00000069702.9 0.552163856974071 0.987009170874021 0.945365562539411 1.03048719140201

ENSG00000126351.11 0.27822026621895 0.986870902130626 0.963575345224667 1.01072965627305

ENSG00000151090.16 0.012300078632808 1.0877654874714 1.01843011583244 1.16182125541992

ENSG00000066056.12 0.702907093748611 1.0039931691703 0.983640953970685 1.02476648585198

ENSG00000173535.12 0.382881167391992 0.988525945498484 0.963231515018112 1.014484606959

ENSG00000173530.5 0.767368012363 1.00398435890952 0.977878699354764 1.03078694075253

ENSG00000141655.14 0.130813756674146 1.05670216513719 0.98374132060755 1.1350742745217

ENSG00000006327.12 0.0638172507417908 1.00143578618451 0.999917554511757 1.00295632307473

ENSG00000159958.4 0.163984898913898 0.967332976825932 0.923131754953471 1.01365063332924

ENSG00000157873.16 0.842077205319985 0.998745856943915 0.986491959125176 1.01115196888905

ENSG00000048462.9 0.00677640424303572 0.97239665446044 0.952892170647521 0.992300370107272

ENSG00000186891.12 0.884136695328924 1.00073559322019 0.990887347166417 1.01068171916981

ENSG00000127863.14 0.0759402005267499 0.991535297267471 0.982270413944556 1.0008875679959

ENSG00000067182.6 1.3461759640343e-07 1.0075691717615 1.00474876218671 1.01039749844996

ENSG00000028137.15 0.731161199460074 1.00118777946688 0.99443095675557 1.00799051250795

ENSG00000146072.6 0.448866511181515 1.00054399629148 0.999136701516942 1.00195327325582

ENSG00000215788.8 0.645919898158329 0.995657050399773 0.977340857979074 1.0143165037228

ENSG00000186827.9 0.478716352032744 1.01118389025991 0.980541020682034 1.04278437959684

ENSG00000120949.13 0.517042826413284 0.939306709147564 0.7772279828511 1.13518441604882

ENSG00000049249.7 0.762661118378582 1.01099165400561 0.941748372468241 1.08532613843564

ENSG00000258947.5 0.00458185857696169 1.04491424326129 1.01365358793276 1.07713896420671

ENSG00000114812.11 0.0881907150095845 0.970253601855276 0.937150926031704 1.00452555267633

ENSG00000179295.14 0.00800587169907274 1.00907093847189 1.00235870823748 1.01582811672177

ENSG00000108622.9 0.514214056099347 0.988537605135295 0.954880519657437 1.02338101641994

ENSG00000005844.16 0.131709358444862 0.988616412276805 0.974008668688296 1.00344323622836

ENSG00000160255.15 0.615133740352521 0.999156397180592 0.995874471333959 1.00244913868479

ENSG00000149269.8 0.0305313089637371 1.0081682923305 1.00076435433617 1.01562700675405

ENSG00000011600.10 0.369891684077518 0.999585820235244 0.998681099141226 1.00049136093049

ENSG00000182866.15 0.218198857524531 0.988397407176485 0.970206100798696 1.00692979946113

ENSG00000203747.8 0.896325447215722 0.999862223660363 0.997792162701981 1.00193657925297

ENSG00000162747.8 0.624826325054686 0.985592794444438 0.929902960836263 1.04461776913499

ENSG00000204475.8 0.209084866746961 0.94920283560422 0.875045497529899 1.02964477351454

ENSG00000198821.9 0.0919822882074806 0.964733687832627 0.925274924668493 1.00587518760718

ENSG00000115085.12 0.599439777363299 0.990279950390766 0.954833498078148 1.02704228760277

ENSG00000043462.10 0.269357724384974 0.989284924077122 0.97055331933295 1.00837804735851

ENSG00000124181.13 0.200337233872359 0.988855178314787 0.972037915948977 1.00596339673163

ENSG00000087266.14 0.04141794111908 1.03258865213289 1.00124869974657 1.0649095722007

ENSG00000010810.16 0.155659272438531 0.98710950368686 0.96958765690225 1.00494799550357

ENSG00000129946.9 0.572669437147654 1.00366157543939 0.990997308232893 1.0164876833114

ENSG00000148082.8 0.0757457667534638 0.931802513471868 0.861923525185104 1.00734682224392

ENSG00000160691.17 0.000378614383058529 1.00276102119121 1.00123766778368 1.00428669233575

ENSG00000177885.12 0.291965013188937 1.00283130608719 0.997571852385446 1.00811848897274

ENSG00000115904.11 0.722332671393974 1.0043710266635 0.9804974663681 1.02882587033875

ENSG00000100485.10 0.205494890139305 0.982564877749029 0.956171338564021 1.00968696722896

ENSG00000078061.11 0.706839891941696 0.998501542259312 0.990728990518438 1.00633507188732

ENSG00000157764.11 0.415146405364482 0.981554333282256 0.938569325263429 1.02650798747846

ENSG00000132155.10 0.740934807526166 1.00207666396096 0.989828429179679 1.01447645960959

ENSG00000126264.8 0.715633306050247 0.998218588398071 0.988688794633699 1.00784023813339

ENSG00000117091.8 0.154285624242779 0.993390475404067 0.984368153551524 1.00249549222324

ENSG00000122223.11 0.427791237798334 0.956704940330081 0.857566766112629 1.06730388702094

ENSG00000154229.10 0.00540953515792919 1.02390046687437 1.00700099547886 1.04108354487477

ENSG00000183918.13 0.090903843632287 0.965849982978201 0.927716997088002 1.00555039149563

ENSG00000026103.18 0.0990234966706333 1.01055442913004 0.998026639993352 1.02323947408973

ENSG00000100453.11 0.60753990652281 1.00168754118063 0.995262780086356 1.0081537763016

ENSG00000180644.6 0.457139082473803 1.00350936987295 0.99428546995568 1.01281883910834

ENSG00000164305.16 0.269317473824228 1.00623657651079 0.995197533701229 1.0173980678414

ENSG00000015475.17 0.174133098936687 1.00679413054414 0.997010508185967 1.01667375917875

ENSG00000167286.8 0.355091757390658 0.99676540988561 0.989944453303779 1.00363336450711

ENSG00000198851.8 0.295409454223151 0.994978627444445 0.985640432350307 1.00440529485034

ENSG00000160654.8 0.328864516820559 0.977176890567187 0.932923576278218 1.02352936482526

ENSG00000081237.17 0.0548515866197923 0.990998407742579 0.98189325794062 1.00018799009588

ENSG00000113263.11 0.186676320726535 0.950829515326149 0.882268388819639 1.02471853085986

ENSG00000135605.11 0.46628117081279 1.02448535116298 0.959936796687567 1.09337431211019

ENSG00000158092.5 0.409320490504378 1.00523742422648 0.992840709842398 1.01778892530091

ENSG00000071051.12 0.372742475703798 1.00305357136185 0.996351396480797 1.00980082988336

ENSG00000100351.15 0.845086045702581 0.989207066341404 0.887183675463646 1.10296283302145

ENSG00000180370.9 0.14193902170739 1.0058064944932 0.998064801753009 1.01360823724856

ENSG00000130669.16 0.0346700813577839 1.01068644418776 1.00076638592749 1.02070483464351

ENSG00000067560.9 0.859576075507448 0.999901080017191 0.998805835981768 1.0009975250463

ENSG00000070831.14 0.477284035718773 0.999160828361585 0.996850140540953 1.00147687233154

ENSG00000178562.16 0.0384334817694808 0.912542804175603 0.836802540643379 0.995138433509561

ENSG00000163600.11 0.179621887645339 0.949308029327784 0.87985031581426 1.02424891864953

ENSG00000107968.8 0.288070802784342 0.989974273907387 0.971740754430395 1.00854992294003

ENSG00000006062.12 0.590656136456623 1.00846893168449 0.977950572508618 1.03993966030823

ENSG00000163599.13 0.182192716584091 0.972704735262821 0.933948111542839 1.01306966662175

ENSG00000142273.9 0.00186167039276908 1.00560903016374 1.00207214209002 1.0091584019467

ENSG00000110395.4 0.88162381185517 1.00220570524405 0.973559637532949 1.0316946562914

ENSG00000114423.17 0.197490090051522 0.973316787958738 0.934094296436796 1.0141862265256

ENSG00000135446.15 0.910381289311229 0.999811335806766 0.996531829491788 1.00310163471396

ENSG00000172575.10 0.541877694678721 0.987951267842087 0.950210444134867 1.02719109609392

ENSG00000152256.12 0.263020130411332 0.972245961449101 0.925488581711111 1.02136560972629

ENSG00000065675.13 0.979558695642192 1.00060749645004 0.955186269929084 1.04818860307356
