# Supplementary material for: An Immune-Related Signature Predicts Survival in Patients With Lung Adenocarcinoma
Source: Front Oncol. 2019 Dec 10;9:1314. doi: 10.3389/fonc.2019.01314 (PMC6914845; doi:10.3389/fonc.2019.01314)
Supplement: Supplementary file 8 [file Table_8.doc]

**Table S8. Multivariate Cox proportional hazards regression analysis of each immune-related gene regarding prognosis.**

Genes p.value HR Low 95%CI High 95%CI

ENSG00000135047.13 0.0272564247671578 1.00075248747517 1.00008444986385 1.00142097132306

ENSG00000242574.7 0.00492424611688613 0.991798181126306 0.986121225493437 0.997507818162256

ENSG00000204252.11 0.0337319541128992 0.993475764112918 0.987491086498687 0.999496711792404

ENSG00000231389.6 0.0613701851036808 0.998836850776431 0.99761972827624 1.00005545819832

ENSG00000196735.10 0.0160992181085791 0.996711788205554 0.994041883948943 0.99938886357724

ENSG00000204287.12 0.0157911428719067 0.999880048454326 0.999782656711452 0.999977449684414

ENSG00000198502.5 0.0329738909881342 0.999634158881642 0.999298019627646 0.999970411204608

ENSG00000204389.9 0.0232754658526041 1.00174385510232 1.00023725510871 1.00325272440107

ENSG00000126803.9 0.013584120163129 1.0307957691808 1.00626391961806 1.05592568415287

ENSG00000170606.12 0.0338024950807349 1.00752917674294 1.0005743101403 1.01453238575151

ENSG00000165916.7 0.257539737397831 1.00246088965161 0.99820473483183 1.00673519190469

ENSG00000013275.6 0.220276956733864 1.00233360375264 0.998604921074372 1.0060762089284

ENSG00000087191.11 0.000790433627765208 1.0128862397724 1.00534068658055 1.02048842587857

ENSG00000175166.15 0.0160092241838095 1.00387330285175 1.00072046092823 1.00703607803095

ENSG00000108344.13 0.509795226012819 1.00177738889172 0.996501672700398 1.00708103597578

ENSG00000108671.8 0.00232979527388633 1.00984352658783 1.0034957242923 1.01623148310923

ENSG00000104856.12 0.145605783216015 1.0094901501787 0.996729298447784 1.02241437559308

ENSG00000133111.3 0.167012107791537 0.939024222712248 0.858861445283829 1.02666908112163

ENSG00000204267.12 0.150339913971462 1.00967519389589 0.996514104833591 1.02301010314244

ENSG00000127922.8 0.125550143022428 1.01539158732651 0.995740779819149 1.03543020082064

ENSG00000131467.9 0.125794447875201 1.00625949585073 0.998252645754553 1.01433056781371

ENSG00000115233.10 0.0948585508191553 1.01241688277378 0.997862272026715 1.02718378403422

ENSG00000101000.4 0.00916695494714681 1.00535854967151 1.00132536947164 1.00940797488326

ENSG00000130706.11 0.421790940549602 1.00147674056768 0.997877560238359 1.00508890254882

ENSG00000164308.15 0.0109711761922113 1.01054757988116 1.00241044668457 1.01875076679545

ENSG00000034510.5 0.0651738743136351 1.0000331024238 0.999997919531227 1.00006828655421

ENSG00000197956.8 0.184315386050888 1.00003274683929 0.999984401802225 1.00008109421363

ENSG00000163993.6 0.0657950470325367 1.00014854261438 0.999990300410974 1.00030680985863

ENSG00000197747.7 4.70009548222405e-05 1.0006503384412 1.00033711096643 1.00096366399436

ENSG00000163191.5 0.0290460747021584 1.00017691533942 1.00001806377573 1.00033579213647

ENSG00000189334.7 0.00786593811781222 1.00074521384408 1.00019560469289 1.00129512500642

ENSG00000188643.9 0.00294487511234187 1.0009446455089 1.00032187434929 1.00156780438763

ENSG00000092096.13 0.170152635293048 0.986453595417583 0.967415706663295 1.005866133049

ENSG00000011201.9 0.0936456051009089 0.984542890486914 0.966774663857197 1.00263767705802

ENSG00000122861.14 0.287959736549074 1.00036120270626 0.999695071518311 1.00102777776032

ENSG00000135114.11 0.214297467923048 1.01090702149432 0.993746465367382 1.02836391546682

ENSG00000007952.16 0.0229568178238369 1.08273199770786 1.0110422168706 1.15950507238858

ENSG00000140464.18 0.458760448219889 1.00503243359152 0.991774315808833 1.01846778694518

ENSG00000181026.14 0.00973303302562822 1.04633092614788 1.01101324263584 1.08288236082762

ENSG00000069869.14 0.116042711556055 1.05252521570506 0.987429480625774 1.12191235063481

ENSG00000196664.4 0.0426898092498778 0.921033594841761 0.850604828225434 0.997293754606238

ENSG00000196262.12 0.0276293251943311 1.00239507893999 1.00026347125357 1.0045312291809

ENSG00000138448.10 0.070178560879457 1.00405862997885 0.999666298716736 1.00847026025498

ENSG00000140564.9 0.153990001055047 1.00016325011973 0.999938811738121 1.00038773887701

ENSG00000124942.12 0.0110693687337077 1.00304281418152 1.0006945076044 1.00539663147519

ENSG00000185591.8 0.0933660791461652 1.01054127904935 0.998238802225433 1.02299537383849

ENSG00000159110.18 0.0479591591239096 0.972912309152781 0.94679189452926 0.999753342598714

ENSG00000102245.6 0.0386852304907056 0.907332139217881 0.827426927881916 0.994953854070342

ENSG00000126934.12 0.00518622784473988 1.01719076637388 1.00510626902369 1.02942055689425

ENSG00000169032.8 0.166416878328784 1.00668689778508 0.997228995724025 1.01623450031792

ENSG00000115365.10 0.106347469132169 1.00587140830818 0.998749570075715 1.01304403062238

ENSG00000149923.12 0.104198586523044 1.00335662265692 0.999309070631825 1.00742056868652

ENSG00000125257.12 0.186664121289512 0.987249184278021 0.968622967057498 1.00623357591702

ENSG00000019991.14 0.136901068350063 0.941418371796239 0.869433776874784 1.01936291679536

ENSG00000105851.9 0.0081890166409132 0.905616442974705 0.841452816589918 0.974672763126364

ENSG00000089685.13 0.0225742898441835 1.00773440936447 1.00108369964919 1.01442930313723

ENSG00000089127.11 0.154644720952113 1.00216231557794 0.999185929767832 1.00514756747815

ENSG00000184009.8 0.011057731030217 1.00016409880074 1.00003751464412 1.00029069898031

ENSG00000137077.6 0.0776445144757558 1.00119117890447 0.99986814816252 1.00251596028758

ENSG00000132170.18 0.0376301976270134 1.01431782281018 1.00081340299709 1.02800446375854

ENSG00000240972.1 0.027075740739952 1.00270613631818 1.00030626136272 1.0051117689101

ENSG00000105369.8 0.0853983346015955 0.996861215272786 0.993296922327212 1.00043829813436

ENSG00000010671.14 0.0320787465433418 0.956892764980041 0.919103724560508 0.996235505529026

ENSG00000095585.15 0.0712286642438003 0.951723069812105 0.901910733197094 1.0042865311091

ENSG00000136238.16 0.0288027086933824 1.0013969865545 1.00014446129383 1.00265108040808

ENSG00000133703.10 0.101893516621049 1.00244133990022 0.999516806855997 1.00537442997266

ENSG00000213281.4 0.001663390525244 1.01182743240163 1.00443921734871 1.01926999192928

ENSG00000198286.8 0.21633694958901 1.00648473138822 0.996223812668167 1.01685133564966

ENSG00000104365.12 0.210125293929161 0.988228159787306 0.970094912981575 1.00670035759187

ENSG00000104825.15 0.0780401769681047 1.01225907252761 0.998633304996605 1.0260707556894

ENSG00000145675.13 0.0941366176311033 0.971016995187224 0.938148070896167 1.00503751400538

ENSG00000197943.8 0.145621087197915 0.952390788232914 0.891828824300935 1.01706537038866

ENSG00000075223.12 0.0742268675970815 1.00562505066942 0.999451066462972 1.01183717389264

ENSG00000196189.11 0.190876227814023 0.988861199624165 0.972402050302884 1.00559894111449

ENSG00000185033.13 0.0300135224211356 1.00189731495393 1.00018341591032 1.0036141509088

ENSG00000095539.14 0.0230161499322725 1.01616335234875 1.0022116622184 1.03030926258732

ENSG00000138623.8 0.0251100022364533 1.01412970816059 1.00175400345891 1.02665830275972

ENSG00000025708.11 0.164271993917359 1.00178825116636 0.999269189918761 1.00431366272439

ENSG00000011422.10 0.0131092585864603 1.00586430084608 1.00122832525321 1.01052174234153

ENSG00000114554.10 0.1524224694031 1.00780873634796 0.997129070546697 1.01860278579824

ENSG00000148926.8 0.466671350634096 1.00243498660656 0.995891994848514 1.00902096569795

ENSG00000168487.16 0.134291048181166 1.01216888998391 0.996270476145764 1.02832100958633

ENSG00000112175.7 0.042902423086568 0.976541411117141 0.954356745577944 0.999241774153492

ENSG00000101144.11 0.0897816866872496 1.01161819777493 0.998208115676205 1.0252084329891

ENSG00000125726.9 0.127213715032416 1.04465353176878 0.987623904660776 1.10497629339158

ENSG00000175505.10 0.0722890266428153 1.00426398341966 0.999614967694581 1.00893462081699

ENSG00000107984.8 0.000948090158350623 1.00439588092627 1.00178705516741 1.00701150051601

ENSG00000105220.13 0.110929238700929 1.00215739855165 0.999505392545609 1.00481644117391

ENSG00000143321.17 0.0109767195874392 1.00168898657073 1.00038720837973 1.00299245873228

ENSG00000172349.15 0.0314616394315013 0.932298615102471 0.874613859872455 0.993787941856693

ENSG00000125571.8 0.0492051851409764 1.0013505230288 1.00000469731067 1.00269815998529

ENSG00000008517.15 0.229313412213547 1.00224156338299 0.998589472705479 1.00590701066667

ENSG00000137033.10 0.0669319420539548 0.99088648118266 0.981229016457421 1.00063899673024

ENSG00000101384.10 0.00334201875341411 1.0101930124167 1.00337335167712 1.01705902456927

ENSG00000128342.4 0.318420058304021 1.00236166734732 0.997727531009016 1.00701732781815

ENSG00000105835.10 0.319393532204032 1.00119139893086 0.998847578572097 1.00354071912163

ENSG00000197696.8 0.0183238038351933 1.00622670445924 1.00105058234442 1.01142959069628

ENSG00000100311.15 0.0301543398394128 1.01115488032089 1.00106530049837 1.02134615143262

ENSG00000145431.9 0.970353950424267 0.999679492105431 0.9829213969147 1.0167233006353

ENSG00000168081.7 0.135448691857406 0.929337537745769 0.844095107165214 1.02318832526336

ENSG00000164022.15 0.07608820819534 1.01354003515145 0.998589881583748 1.02871401142737

ENSG00000141574.6 0.0754116009316502 1.00502250096733 0.999487258977475 1.01058839757895

ENSG00000113739.9 0.00542071756266427 1.01850849031165 1.00542857031836 1.0317585708833

ENSG00000125735.9 0.0539013146002183 1.0654848284684 0.998936554501985 1.13646648986862

ENSG00000125657.4 0.0172602506195172 1.02650352770572 1.00463740390181 1.04884557184501

ENSG00000150630.3 0.00166129427633677 1.01209268962448 1.00453928808939 1.01970288722064

ENSG00000006831.9 0.00802369642672207 1.01200953284115 1.00311695945925 1.02098093836781

ENSG00000169252.5 0.0505097317810699 0.940886394904659 0.885147443746126 1.00013530443025

ENSG00000167772.10 0.037929711316353 1.00230251974664 1.00012815307583 1.00448161368206

ENSG00000137070.16 0.13344777837044 0.954127785188398 0.897368891788579 1.01447669826625

ENSG00000134470.18 0.164661683882148 1.01471448483989 0.994021804196335 1.03583792769651

ENSG00000115590.12 0.0154397143969762 1.01024412998469 1.00194583390264 1.01861115405135

ENSG00000174564.11 0.0342603307809939 1.00584678873372 1.00043252240732 1.01129035666636

ENSG00000113594.8 0.0667692901057599 0.983686936433254 0.966540305182387 1.0011377525812

ENSG00000111321.9 0.00262716742713354 1.00718352963165 1.00249748455292 1.01189147902318

ENSG00000258839.2 0.00228189467260009 1.11848051328264 1.0408497229976 1.20190132249848

ENSG00000105976.13 0.0424350155719248 1.00099539115691 1.00003395981573 1.00195774681693

ENSG00000160113.5 0.101713031304911 1.00493611176081 0.999027599560383 1.0108795684577

ENSG00000118257.15 0.0704620293347772 1.01273831913191 0.998943700525434 1.02672343045824

ENSG00000112033.12 0.139754300303515 1.0065915370349 0.997852284436275 1.01540732855335

ENSG00000077092.17 0.365357995161982 1.0161335929752 0.981523655675871 1.05196392649517

ENSG00000172819.15 0.00231117941753556 1.01709048915349 1.00606402603506 1.02823780232296

ENSG00000151090.16 0.00774173688641333 1.10730507324296 1.02727857032395 1.19356575777002

ENSG00000048462.9 0.0291144873565986 0.974417057774001 0.951995167608057 0.997367040072888

ENSG00000067182.6 0.000369023601054841 1.00656874383114 1.00294830986428 1.01020224680861

ENSG00000258947.5 0.0429437437713687 1.03744091833401 1.00116812746819 1.075027889427

ENSG00000179295.14 0.0550470770626329 1.00727601976208 0.999843344318798 1.01476394852534

ENSG00000149269.8 0.210839444858385 1.00552915275655 0.996881831804925 1.01425148376177

ENSG00000087266.14 0.113735659786969 1.02938896394198 0.993095121385968 1.06700920814785

ENSG00000160691.17 0.0739619532280651 1.00177030599291 0.999828653689944 1.00371572895563

ENSG00000154229.10 0.000807579376601586 1.03468362765284 1.01424920059252 1.0555297541348

ENSG00000130669.16 0.2162635583134 1.00794159828195 0.995382746168645 1.02065890679507

ENSG00000178562.16 0.0475113009371134 0.902069869588506 0.814651010785834 0.998869502211111

ENSG00000142273.9 0.0100796869559486 1.00502872085792 1.00119596562975 1.00887614855096
